# Biodiversity inventories in high gear: DNA barcoding facilitates a rapid biotic survey of a temperate nature reserve

**DOI:** 10.3897/BDJ.3.e6313

**Published:** 2015-08-30

**Authors:** Angela C Telfer, Monica R Young, Jenna Quinn, Kate Perez, Crystal N Sobel, Jayme E Sones, Valerie Levesque-Beaudin, Rachael Derbyshire, Jose Fernandez-Triana, Rodolphe Rougerie, Abinah Thevanayagam, Adrian Boskovic, Alex V Borisenko, Alex Cadel, Allison Brown, Anais Pages, Anibal H Castillo, Annegret Nicolai, Barb Mockford Glenn Mockford, Belén Bukowski, Bill Wilson, Brock Trojahn, Carole Ann Lacroix, Chris Brimblecombe, Christoper Hay, Christmas Ho, Claudia Steinke, Connor P Warne, Cristina Garrido Cortes, Daniel Engelking, Danielle Wright, Dario A Lijtmaer, David Gascoigne, David Hernandez Martich, Derek Morningstar, Dirk Neumann, Dirk Steinke, Donna DeBruin Marco DeBruin, Dylan Dobias, Elizabeth Sears, Ellen Richard, Emily Damstra, Evgeny V Zakharov, Frederic Laberge, Gemma E Collins, Gergin A Blagoev, Gerrie Grainge, Graham Ansell, Greg Meredith, Ian Hogg, Jaclyn McKeown, Janet Topan, Jason Bracey, Jerry Guenther, Jesse Sills-Gilligan, Joseph Addesi, Joshua Persi, Kara K S Layton, Kareina D'Souza, Kencho Dorji, Kevin Grundy, Kirsti Nghidinwa, Kylee Ronnenberg, Kyung Min Lee, Linxi Xie, Liuqiong Lu, Lyubomir Penev, Mailyn Gonzalez, Margaret E Rosati, Mari Kekkonen, Maria Kuzmina, Marianne Iskandar, Marko Mutanen, Maryam Fatahi, Mikko Pentinsaari, Miriam Bauman, Nadya Nikolova, Natalia V Ivanova, Nathaniel Jones, Nimalka Weerasuriya, Norman Monkhouse, Pablo D Lavinia, Paul Jannetta, Priscila E Hanisch, R. Troy McMullin, Rafael Ojeda Flores, Raphaëlle Mouttet, Reid Vender, Renee N Labbee, Robert Forsyth, Rob Lauder, Ross Dickson, Ruth Kroft, Scott E Miller, Shannon MacDonald, Sishir Panthi, Stephanie Pedersen, Stephanie Sobek-Swant, Suresh Naik, Tatsiana Lipinskaya, Thanushi Eagalle, Thibaud Decaëns, Thibault Kosuth, Thomas Braukmann, Tom Woodcock, Tomas Roslin, Tony Zammit, Victoria Campbell, Vlad Dinca, Vlada Peneva, Paul D N Hebert, Jeremy R deWaard

**Affiliations:** ‡ Biodiversity Institute of Ontario, Guelph, Canada; § rare Charitable Research Reserve, Cambridge, Canada; | CNC, Ottawa, Canada; ¶ Muséum national d'Histoire Naturelle, Paris, France; # University of Waterloo, Waterloo, Canada; ¤ Université de Montpellier, Montpellier, France; « EcoBio, Université of Rennes, Rennes, France; » rare Charitable Research Reserve (Affiliate of), Cambridge, Canada; ˄ Museo Argentino de Ciencias Naturales "Bernardino Rivadavia" (MACN-CONICET), Buenos Aires, Argentina; ˅ Biodiversity Institute of Ontario Herbarium, Guelph, Canada; ¦ University of Waikato, Hamilton, New Zealand; ˀ University of Western Ontario, London, Canada; ˁ University of Guelph, Guelph, Canada; ₵ Universidad Autonoma de Santo Domingo DR, Santo Domingo, Dominican Republic; ℓ Myotistar, Cambridge, Canada; ₰ SNSB, Zoologische Staatssammlung Muenchen, Munich, Germany; ₱ Grand River Conservation Authority, Guelph, Canada; ₳ The University of Western Australia, Perth, Australia; ₴ National Biodiversity Centre, Thimphu, Bhutan; ₣ Ministry of Environment and Tourism in Namibia, Windhoek, Namibia; ₮ University of Oulu, Oulu, Finland; ₦ The University of Western Ontario, London, Canada; ₭ Pensoft, Sofia, Bulgaria; ₲ Instituto de Investigación de Recursos Biológicos Alexander von Humboldt, Bogotá, Colombia; ‽ Smithsonian National Museum of Natural History, Washington, United States of America; ₩ Universidad Nacional Autónoma de México, Mexico City, Mexico; ₸ ANSES, Laboratoire de la Santé des Végétaux, Montferrier sur Lez, France; ‡‡ New Brunswick Museum, Saint John, Canada; §§ London Homeopathy, London, Canada; || Ministry of Forests and Soil Conservation, Kathmandu, Nepal; ¶¶ Scientific and Practical Center for Bioresources, National Academy of Sciences of Belarus, Minsk, Belarus; ## Université de Montpellier Centre d'Ecologie Fonctionnelle et Evolutive, Montpellier, France; ¤¤ University of Helsinki, Helsinki, Finland; «« Swedish University of Agricultural Sciences, Uppsala, Sweden; »» Grand River Conservation Authority, Cambridge, Canada; ˄˄ Bulgarian Academy of Sciences, Sofia, Bulgaria

**Keywords:** DNA barcoding, species identification, biodiversity assessment, biotic inventory, Barcode Index Numbers, Operational Taxonomic Units, *rare* Charitable Research Reserve

## Abstract

**Background:**

Comprehensive biotic surveys, or ‘all taxon biodiversity inventories’ (ATBI), have traditionally been limited in scale or scope due to the complications surrounding specimen sorting and species identification. To circumvent these issues, several ATBI projects have successfully integrated DNA barcoding into their identification procedures and witnessed acceleration in their surveys and subsequent increase in project scope and scale. The Biodiversity Institute of Ontario partnered with the ***rare* Charitable Research Reserve** and delegates of the 6th International Barcode of Life Conference to complete its own rapid, barcode-assisted ATBI of an established land trust in Cambridge, Ontario, Canada.

**New information:**

The existing species inventory for the ***rare* Charitable Research Reserve** was rapidly expanded by integrating a DNA barcoding workflow with two surveying strategies – a comprehensive sampling scheme over four months, followed by a one-day bioblitz involving international taxonomic experts. The two surveys resulted in 25,287 and 3,502 specimens barcoded, respectively, as well as 127 human observations. This barcoded material, all vouchered at the Biodiversity Institute of Ontario collection, covers 14 phyla, 29 classes, 117 orders, and 531 families of animals, plants, fungi, and lichens. Overall, the ATBI documented 1,102 new species records for the nature reserve, expanding the existing long-term inventory by 49%. In addition, 2,793 distinct Barcode Index Numbers (BINs) were assigned to genus or higher level taxonomy, and represent additional species that will be added once their taxonomy is resolved. For the 3,502 specimens, the collection, sequence analysis, taxonomic assignment, data release and manuscript submission by 100+ co-authors all occurred in less than one week. This demonstrates the speed at which barcode-assisted inventories can be completed and the utility that barcoding provides in minimizing and guiding valuable taxonomic specialist time. The final product is more than a comprehensive biotic inventory – it is also a rich dataset of fine-scale occurrence and sequence data, all archived and cross-linked in the major biodiversity data repositories. This model of rapid generation and dissemination of essential biodiversity data could be followed to conduct regional assessments of biodiversity status and change, and potentially be employed for evaluating progress towards the Aichi Targets of the Strategic Plan for Biodiversity 2011–2020.

## Introduction

It is now universally accepted that we have entered a period of unprecedented global biodiversity loss (Simberloff 1996, Pimm et al. 1995, Pimm et al. 2014), and quantifying this diversity rapidly and on a massive scale is required to begin the challenging process of halting this trend. The completion of biodiversity inventories at various geographic and time scales can contribute to national and international assessments of biodiversity knowledge, deemed necessary by the newly established Intergovernmental Science-Policy Platform on Biodiversity and Ecosystem Services (Díaz et al. 2015). These assessments are fundamental for evaluating progress towards – and potentially reaching – the Convention on Biological Diversity’s Aichi Targets of the Strategic Plan for Biodiversity 2011–2020 (https://www.cbd.int/sp/targets/). Specifically, biodiversity inventories address a component of Aichi target 19, to improve and disseminate biodiversity knowledge, particularly its status and trends.

Even prior to the concept’s introduction (Janzen and Hallwachs 1994), several ‘all taxon biodiversity inventories’ (ATBI) and similar initiatives emerged to document large blocks of life in a circumscribed region or protected area. These comprehensive biotic surveys, particularly those in tropical locales, have traditionally been mired at the stage of specimen sorting and species identification. The taxonomic impediment – the shortage of taxonomic information and the gaps in our taxonomic knowledge – have severely limited and slowed the sorting and naming of collected material (Janzen 1993, Lawton et al. 1998, Janzen 2004). The integration of DNA barcoding however, has simplified, accelerated, and democratized this task (Hebert et al. 2003, Packer et al. 2009, Cristescu 2014, Joly et al. 2014). Several ATBI projects have now successfully integrated DNA barcoding into their identification procedures, observed this acceleration, and in many cases, even increased the scope or scale of their project as a result. A few notable ATBIs that have incorporated DNA barcoding for species identification include projects in the Área de Conservación Guanacaste, Costa Rica (Janzen et al. 2009), Churchill, Canada (Zhou et al. 2009), Great Smoky Mountains, United States (Zhou et al. 2011), Madang, Papua New Guinea (Novotny et al. 2007), Moorea, French Polynesia (Check 2006), Zackenberg, Greenland (Wirta et al. 2015), and Mount Kinabalu, Malaysia (Merckx et al. 2015).​

Following this model, the present study introduces DNA barcoding to a long-term biotic inventorying effort being conducted in a temperate nature reserve. The objective is to gauge the effect of adding this tool, in terms of both acceleration and increase of taxonomic scope, while concurrently constructing a reference DNA barcode library to facilitate future research and monitoring at this site. The existing inventory is expanded by employing two surveying strategies – a longer and comprehensive invertebrate trapping scheme, followed by a concentrated effort involving taxonomic experts in the form of a bioblitz (Lundmark 2003). In both cases, DNA barcoding is employed to sort the material rapidly into operational taxonomic units (OTUs), provide taxonomic assignment at varying levels of resolution depending on the taxon group, and organize the OTUs and linked specimen vouchers for examination by experts. The results demonstrate the speed at which barcode-assisted surveys can be completed, the role that barcoding plays in limiting and optimizing valuable taxonomic specialist time, and ultimately, a scalable model for rapid biotic surveys and dissemination of the rich biodiversity data captured. The product is not merely a comprehensive biotic inventory, but also a rich dataset of fine-scale occurrence and sequence data, all stored and cross-linked in several public biodiversity data repositories.

## Materials and methods

### Study Site and Existing Species Inventory

**Figure 1.**
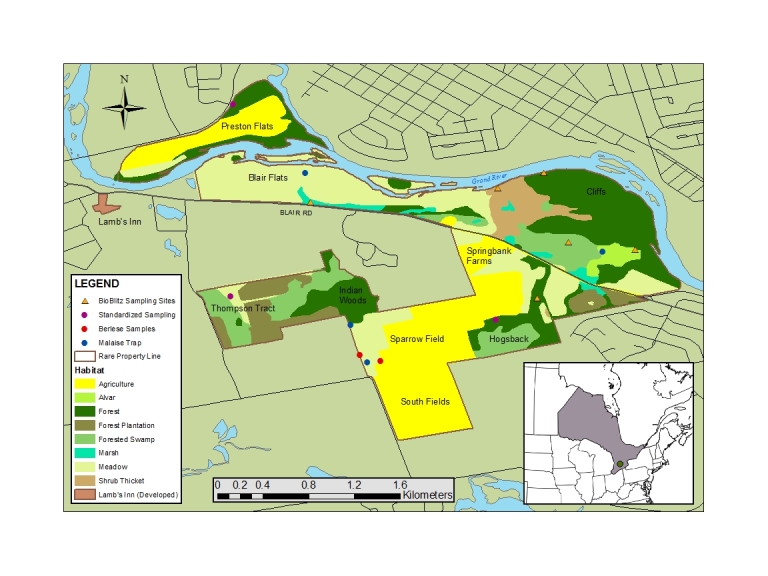
Map indicating habitat types and sampling sites for the 2015 biotic survey conducted at ***rare* Charitable Research Reserve** in Cambridge, Ontario, Canada.

The ***rare* Charitable Research Reserve** is a 365+ hectare land reserve which was set aside in 2001 to preserve the cultural history and ecological integrity of the area, while providing opportunities for scientific research and public education within the context of an urbanized region. It is located at the confluence of the Speed and Grand Rivers in Cambridge, Ontario, Canada (43.381128, -80.357807), where the Carolinian and Northern Hardwood forests also meet. The reserve contains a diversity of habitats including existing and reclaimed agricultural lands, wetlands, floodplains, shrub thickets, limestone cliffs and alvars, cold-water creeks, and old growth forest. Due to these diverse habitats, as well as the organization’s mandate to facilitate scientific research, ***rare*** has been the site of a variety of innovative research studies, including studies on fern genetics (Henry et al. 2014), prairie community establishment (Harvey and MacDougall 2014), and pollination services (Woodcock et al. 2014). For the present study, we chose sampling sites to encompass a wide range of habitats within the area. In advance of the bioblitz, sampling was conducted within an alvar, reclaimed agricultural fields, forest edges, and a wetland (Fig. 1). Six additional sites were sampled for the bioblitz: terrestrial and aquatic sampling was performed at the Grand River, Blair Flats Wetland, and Cruickston Creek; terrestrial sampling was conducted at an alvar, a cedar stand, and a silver maple wetland (Fig. 1​​). The wetlands on the reserve are part of the Barrie's Lake Bauman Creek Wetland Complex, which has been classified as a Provincially Significant Wetland by the Ontario Ministry of Natural Resources. Much of the property is also considered locally significant by the Regional Municipality of Waterloo.

Prior to this study, 2,246 species had been recorded at ***rare***, including birds (231), mammals (37), insects (832), plants (836), mosses (63) and lichens (21) (Suppl. material 1​​​; note that 218 species still require confirmation, denoted by parentheses). These observations have come from a variety of sources including ***rare*** staff and advisors, citizen scientists, and academic researchers, some of whom have made notable contributions to this inventory, such as Woodcock et al. 2014 who added 150 species of pollinators. Others have provided observations of rarely encountered species, such as the rove beetles *Xantholinus elegans* (Olivier, 1795) (first record for North America) and *Xantholinus linearis* (Olivier, 1794) (previously known from the east coast of Canada, now present in Ontario) (Brunke and Majka 2010). The ***rare*** reserve also hosts several species that fall on the provincial or national lists of conservation concern: 1% of the total have a status ranging from special concern, threatened or endangered (15, 6 and 7 species, respectively), while 1.5% have an undefined status of rare or uncommon (15 and 19 species, respectively). Several of these species, such as the barn swallow [*Hirundo rustica* (Linnaeus, 1758)], which has been nationally listed as threatened since 2011, are the subjects of active recovery projects at ***rare***. Similar to many species inventories, ***rare's*** list is evidently more complete for charismatic and well-studied taxa, such as birds, butterflies and vascular plants. The observations for these groups would be enabled by excellent field guides and the disproportionate interest of citizen scientists; the opposite would be the case for most invertebrate taxa, and ***rare's*** inventory reflects this. If it is assumed that the proportions of major taxonomic groups inhabiting this reserve resemble those for similar temperate sites (e.g., Great Smoky Mountains: Sharkey 2001) or the Canadian terrestrial and freshwater biota in general (Mosquin et al. 1995), this inventory is deficient in several major groups such as insects, arachnids, nematodes, fungi and lichens. The supporting data for most species entries are also lacking, such as specific locality, date or identifier of each observation. In addition, the bioblitz has highlighted errors in the inventory which may have been present due to the compilation of observations from a variety of sources including non-experts. Furthermore, nearly all observations are not supported by voucher specimens or images to permit verification (Bortolus 2008).

### Survey Strategies and Specimen Collection

Two strategies were employed in an effort to maximize the diversity of organisms inventoried. The first was a comprehensive collecting scheme executed over a period of approximately four months (May to August 2015). It involved a variety of targeted taxa and techniques, but heavily favoured the collection of terrestrial arthropods by passive trapping. Four Malaise traps were set up in various habitats around the ***rare*** property (Fig. 1) and were serviced weekly. Three pitfall traps were set up in close proximity to each Malaise trap and serviced in parallel. From May 25-31 and July 6-12, 2015, 'standardized sampling' procedures developed by the Biodiversity Institute of Ontario were employed at three different sites at ***rare*** (Fig. 1). Standardized sampling includes 20 pitfall traps, 10 pan traps, three litter and/or soil samples for Berlese funnels, one flight intercept trap and one Malaise trap – all deployed for a total of seven days. Standardized sampling also includes 60 total minutes of sweep netting, which is performed in three sessions with four collectors sweep netting for five consecutive minutes simultaneously. Each session is pooled into one sample and preserved in 95% ethanol. Passive traps (pitfall, pan, intercept) were deployed with soapy water and serviced every two days, while the Malaise trap used 95% ethanol and ran for the duration of the collecting period. All specimens collected through this standardized sampling routine were consolidated into individual jars for each technique. From July 23 to Aug 13, 2015, at least two ultraviolet light traps were deployed for one night per week; each trap was left overnight using ethyl acetate as a killing agent. On August 5, 2015, several aquatic samples were collected using stable dip nets for surber sampling, deploying hand nets along the stream banks and selective turning of stones to recover macroinvertebrates hiding below. Then the sample was passed through a 50 µm mesh net to capture the smaller invertebrates. All specimens collected from these samples were preserved in 95% ethanol. This first strategy of comprehensive sampling over several months was conducted by technical staff, including undergraduate students, and completed on Aug 15, 2015.

The second strategy for surveying the reserve was a more concentrated effort and involved taxonomic experts – the execution of a bioblitz (Lundmark 2003). Termed the '***rare*** BioBlitz', the event involved 113 participants from 31 institutions and coincided with the 6th International Barcode of Life Conference (Adamowicz 2015​). Collection efforts at the bioblitz were focused on six sites selected based on habitat diversity, proximity, and potential species diversity (Fig. 1​​). Sampling efforts were concentrated between 1-3 pm and 9-11 pm and targeted taxa that fell within the expertise of the participants and in groups underrepresented in the ***rare*** inventory (e.g., spiders, parasitic wasps, mites, nematodes, fungi, and lichens). Various methods for collecting both terrestrial and aquatic arthropods were employed, including dip nets, seine nets, sweep netting, and plankton netting. Small teams surveyed the property and adjacent rivers for taxonomic groups not collected in the four previous months, including fungi, lichens, and vascular plants. Since the inventory is fairly complete for vascular plants (836 species), an effort was made to barcode all species of Blair Flats, a tall grass prairie site that is an active research site (e.g., Harvey and MacDougall 2014​). For these taxa, specimens were collected in the field into bags and processed afterward (e.g., pressed and dried onto herbarium sheets). Fungal specimens were collected and processed in a similar manner. Several taxa were targeted for sightings only, where no voucher specimens were collected; these groups included fish, birds, bats, herptiles and odonates (for bat sightings protocol, see Suppl. material 2​). All collected specimens were sorted and identified to the lowest taxonomic level possible by the appropriate expert, both on site and following the event from August 16-20, 2015. Vertebrates, plants, fungi and lichens were almost all identified to species, while most invertebrate specimens were assigned to order or family prior to analysis. Invertebrate specimens were either stored in 95% ethanol or pinned after collection. For all invertebrate, plant, and fungal taxa, voucher specimens were collected where possible and deposited in the Biodiversity Institute of Ontario's natural history collection (BIOUG) or herbarium (BIO-OAC) for permanent storage (Schilthuizen et al. 2015).

### DNA Barcode Analysis

Both surveying strategies provided a large number of specimens that were sorted and prepared for subsequent DNA barcode analyses at the Canadian Centre for DNA barcoding (CCDB; www.ccdb.ca). A total of 25,287 specimens were sequenced from collection efforts from May to August 2015, followed by 3,502 specimens directly following the ***rare*** BioBlitz on Aug 16, 2015. Tissue samples were prepared in 96-well plate format and when necessary, the whole specimen proceeded through lysis and was recovered as voucher from the filter plate (Porco et al. 2010​). Tissue lysis and DNA extraction varied slightly for different taxa (Suppl. material 3), but followed standard CCDB procedures (Ivanova et al. 2006, deWaard et al. 2008, Ivanova et al. 2008, Ivanova et al. 2011, Fazekas et al. 2012​).

One or more standard DNA barcode markers were targeted for each major group of organisms: for animals, the mitochondrial gene *cytochrome oxidase subunit 1* (COI) (Hebert et al. 2003); for plants, the plastid marker *rbcL* (Hollingsworth et al. 2009) and the nuclear ribosomal internal transcribed spacer 2 (ITS2) marker (Hollingsworth 2011, Schoch et al. 2012); and for fungi and lichens (where only the fungal component was targeted), the internal transcribed spacer (ITS) marker (Schoch et al. 2012). PCR amplification, cycle sequencing and sequence analysis followed typical CCDB protocols (Ivanova and Grainger 2006, Kuzmina and Ivanova 2011). The primer cocktails used for PCR and sequencing are detailed in Suppl. material 3​. The sequences were manually assembled and edited before upload to in the Barcode of Life Data Systems (BOLD, www.boldsystems.org) (Ratnasingham and Hebert 2007​). The DNA extracts for all specimens are stored in the DNA Archive of the CCDB where they are available for additional study.

### Barcode Index Numbers and Taxonomic Assignment

For the sequences derived from animal specimens, the records were assigned operational taxonomic units (OTUs) called Barcode Index Numbers (BINs) by the Refined Single Linkage (RESL) algorithm implemented on BOLD (Ratnasingham and Hebert 2013). For the sequences that have at least 500 bp coverage of the barcode region, < 1% ambiguous bases, and no stop codon or contamination flags, the RESL algorithm calculates the number of clusters and their membership (see Ratnasingham and Hebert 2013​). The RESL algorithm runs weekly on all qualifying barcode sequences in BOLD, which as of August 2015, includes 5M specimens and 420K BINs. The BIN system is accessible through public, individual ‘BIN pages’ and permits rapid diversity assessments, even in the absence of taxonomic information. BINs show a high concordance with traditional taxonomic species names and can be used as a reliable proxy for species.

For each specimen that was assigned an existing BIN, the record received the existing identification of the BIN to the lowest level that did not have taxonomic conflict. For each specimen assigned a new BIN for BOLD, the sequence was queried through the BOLD Identification Engine (BOLD-ID Engine; http://www.boldsystems.org/index.php/IDS_OpenIdEngine). Identifications were applied based on sequence similarity (<15% for family, <5% for genus) if the query sequence fell within a monophyletic cluster of BINs assigned to this family or genus. For animal records that did not receive BINs (<500bp), the sequence was similarly queried through the BOLD-ID Engine, but used a <2% similarity cutoff for assignment to species, in addition to the genus and family thresholds. Following this, a neighbour-joining tree was constructed and examined for unexpected placements which might indicate overlooked contamination events or analytical error. Finally, specimens and images were inspected morphologically to check for errors and refine the assigned taxonomy where possible.

## Data resources

Collection data, taxonomic assignment, sequence, electropherograms and primer details for each specimen record, and often a high resolution image, are available on BOLD in the public dataset, "***rare*** BioBlitz 2015 [DS-RBB15]" (http://dx.doi.org/10.5883/DS-RBB15 or http://boldsystems.org/index.php/MAS_Management_OpenDataSet?datasetcode=DS-RBB15). The sequence data for each successfully barcoded specimen were deposited to GenBank by using the 'Submit to GenBank' function in the BOLD workbench (see ​Suppl. material 4​ for accession numbers).

With the 'Data Spreadsheets' function in the BOLD workbench, the complete dataset was downloaded and reformatted into a Darwin Core Archive (Suppl. material 5) for upload to the Canadensys repository (http://www.canadensys.net), Canada's national node for the Global Biodiversity Information Facility (http://www.gbif.org). The online resource (http://doi.org/10.5886/hh6td9jn) contains all records of the 2015 inventory, including human observations. The citation for the resource is as follows:

Telfer A, Young MR, Quinn J, Perez K, Sobel CN, Sones JE, Levesque-Beaudin V, Derbyshire R, Fernandez-Triana J, Rougerie R, Hebert PDN, deWaard JR and contributors* (2015+). Inventory and BioBlitz Records from ***rare* Charitable Research Reserve**. 28,916 records. Online at http://data.canadensys.net/ipt/resource.do?r=rare_inventory, http://doi.org/10.5886/hh6td9jn, and http://www.gbif.org/dataset/09e90dfb-5b1b-4dd9-a796-e2fba53d26f0, released on 2015-08-20, version 1. GBIF key: 09e90dfb-5b1b-4dd9-a796-e2fba53d26f0.

* See Suppl. material 6​ for complete list of contributors, institutions and email addresses

## Checklists

### Checklist of species observed or collected at the *rare* Charitable Research Reserve in Cambridge, Ontario, Canada. The first of five checklists for Kingdom Animalia, this checklist contains members of Phylum Annelida and Phylum Arthopoda (Class Arachnida and Class Insecta up to Order Dermaptera).

#### Animalia

#### Annelida

#### Clitellata

#### Arhynchobdellida

#### Erpobdellidae

#### Erpobdella punctata

Leidy, 1870

#### Haplotaxida

#### Lumbricidae

#### Lumbricus terrestris

Linnaeus, 1758

#### Arthropoda

#### Arachnida

#### Araneae

#### Agelenidae

#### Agelenopsis potteri

Blackwall, 1846

#### Amaurobiidae

#### Callobius bennetti

Blackwall, 1846

##### Notes

BOLD:AAB8212

#### Anyphaenidae

#### Anyphaena celer

Hentz, 1847

#### Anyphaena pectorosa

L. Koch, 1866

##### Notes

BOLD:AAD6926

#### Hibana gracilis

Hentz, 1847

##### Notes

BOLD:AAN6394

#### Wulfila saltabundus

Hentz, 1847

##### Notes

BOLD:AAC6924

#### Araneidae

#### Acanthepeira stellata

Walckenaer, 1805

##### Notes

BOLD:AAD7855

#### Araneus diadematus

Clerck, 1757

##### Notes

BOLD:AAA4125

#### Araneus trifolium

Hentz, 1847

##### Notes

BOLD:AAB8544

#### Araniella displicata

Hentz, 1847

##### Notes

BOLD:AAA8399

#### Argiope aurantia

Lucas, 1833

##### Notes

BOLD:AAB7933

#### Argiope trifasciata

Forsskål, 1775

#### Eustala anastera

Walckenaer, 1841

##### Notes

BOLD:AAL4913

#### Eustala cepina

Walckenaer, 1841

##### Notes

BOLD:AAB7935

#### Eustala emertoni

Banks, 1904

#### Hypsosinga pygmaea

Sundevall, 1831

##### Notes

BOLD:ABX6180

#### Hypsosinga rubens

Hentz, 1847

##### Notes

BOLD:AAN6264

#### Larinioides cornutus

Clerck, 1757

##### Notes

BOLD:AAA8999

#### Larinioides patagiatus

Clerck, 1757

#### Mangora gibberosa

Hentz, 1847

##### Notes

BOLD:AAB7330

#### Mangora maculata

Keyserling, 1865

#### Mangora placida

Hentz, 1847

##### Notes

BOLD:AAI4456|BOLD:ACE4103

#### Neoscona arabesca

Walckenaer, 1841

##### Notes

BOLD:AAA4123

#### Clubionidae

#### Clubiona abboti

L. Koch, 1866

##### Notes

BOLD:AAD1564

#### Clubiona bryantae

Gertsch, 1941

#### Clubiona johnsoni

Gertsch, 1941

##### Notes

BOLD:AAN4847

#### Clubiona maritima

L. Koch, 1867

##### Notes

BOLD:AAI4085

#### Clubiona obesa

Hentz, 1847

##### Notes

BOLD:AAD5417

#### Clubiona pallidula

Clerck, 1757

##### Notes

BOLD:AAI4087

#### Dictynidae

#### Cicurina brevis

Emerton, 1890

##### Notes

BOLD:AAC8284

#### Cicurina itasca

Chamberlin & Ivie, 1940

##### Notes

BOLD:AAI4031

#### Cicurina pallida

Keyserling, 1887

##### Notes

BOLD:AAF3046

#### Dictyna bellans

Chamberlin, 1919

##### Notes

BOLD:AAI6249

#### Dictyna bostoniensis

Emerton, 1888

##### Notes

BOLD:AAL1061

#### Dictyna brevitarsa

Emerton, 1915

##### Notes

BOLD:AAB2306

#### Dictyna foliacea

Hentz, 1850

##### Notes

BOLD:AAI6247

#### Dictyna volucripes

Keyserling, 1881

##### Notes

BOLD:AAB1638|BOLD:ACE2869

#### Emblyna annulipes

Blackwall, 1846

#### Emblyna hentzi

Kaston, 1945

##### Notes

BOLD:AAI6251

#### Emblyna manitoba

Ivie, 1947

##### Notes

BOLD:AAI9209

#### Emblyna sublata

Hentz, 1850

##### Notes

BOLD:AAA7272

#### Gnaphosidae

#### Drassyllus depressus

Emerton, 1890

##### Notes

BOLD:AAD8676

#### Drassyllus niger

Banks, 1896

##### Notes

BOLD:AAI9037

#### Gnaphosa parvula

Banks, 1896

##### Notes

BOLD:AAC3779

#### Haplodrassus signifer

C. L. Koch, 1839

##### Notes

BOLD:AAD0462

#### Herpyllus ecclesiasticus

Hentz, 1832

##### Notes

BOLD:AAF2106

#### Micaria pulicaria

Sundevall, 1831

##### Notes

BOLD:AAC6612

#### Sergiolus ocellatus

Walckenaer, 1837

##### Notes

BOLD:ACV6055

#### Zelotes hentzi

Barrows, 1945

##### Notes

BOLD:AAA8914

#### Zelotes pseustes

Chamberlin, 1922

#### Hahniidae

#### Neoantistea agilis

Keyserling, 1887

##### Notes

BOLD:ACV5090

#### Neoantistea gosiuta

Gertsch, 1934

##### Notes

BOLD:AAG9583

#### Linyphiidae

#### Agyneta fabra

Keyserling, 1886

##### Notes

BOLD:AAE3860

#### Agyneta micaria

Emerton, 1882

##### Notes

BOLD:AAN6265

#### Agyneta unimaculata

Banks, 1892

##### Notes

BOLD:AAH0003

#### Bathyphantes brevis

Emerton, 1911

##### Notes

BOLD:AAC5851

#### Bathyphantes pallidus

Banks, 1892

##### Notes

BOLD:AAC9112

#### Centromerus sylvaticus

Blackwall, 1841

##### Notes

BOLD:AAA4132

#### Ceraticelus atriceps

O. P.-Cambridge, 1874

##### Notes

BOLD:AAI3701

#### Ceraticelus similis

Banks, 1892

##### Notes

BOLD:AAF1318

#### Ceratinella brunnea

Emerton, 1882

##### Notes

BOLD:AAD2101

#### Ceratinops crenatus

Emerton, 1882

##### Notes

BOLD:ACF8798

#### Ceratinops latus

Emerton, 1882

##### Notes

BOLD:AAI5447

#### Ceratinopsis auriculata

Emerton, 1909

##### Notes

BOLD:ACR6338

#### Ceratinopsis labradorensis

Emerton, 1925

##### Notes

BOLD:ACV5182

#### Collinsia plumosa

Emerton, 1882

##### Notes

BOLD:AAM9146

#### Eridantes erigonoides

Emerton, 1882

##### Notes

BOLD:AAH0004

#### Erigone atra

Blackwall, 1833

##### Notes

BOLD:ACE5877

#### Erigone autumnalis

Emerton, 1882

##### Notes

BOLD:AAH0001

#### Erigone blaesa

Crosby & Bishop, 1928

##### Notes

BOLD:ACE9601

#### Frontinella communis

Hentz, 1850

##### Notes

BOLD:AAE0825

#### Grammonota angusta

Dondale, 1959

##### Notes

BOLD:AAD1498

#### Grammonota inornata

Emerton, 1882

##### Notes

BOLD:ACV5737

#### Hypomma marxi

Keyserling, 1886

##### Notes

BOLD:AAB9520

#### Hypselistes florens

O. P.-Cambridge, 1875

##### Notes

BOLD:AAB4233

#### Mermessus index

Emerton, 1914

##### Notes

BOLD:ACL4554

#### Mermessus trilobatus

Emerton, 1882

##### Notes

BOLD:AAC8898

#### Microlinyphia mandibulata

Emerton, 1882

##### Notes

BOLD:AAF4994

#### Neriene clathrata

Sundevall, 1830

##### Notes

BOLD:AAA8358|BOLD:AAB7327

#### Neriene montana

Clerck, 1757

#### Neriene variabilis

Banks, 1892

#### Pityohyphantes costatus

Hentz, 1850

#### Pityohyphantes subarcticus

Chamberlin & Ivie, 1943

#### Pocadicnemis americana

Millidge, 1976

##### Notes

BOLD:AAC9060

#### Tennesseellum formica

Emerton, 1882

##### Notes

BOLD:AAG5631

#### Tenuiphantes zebra

Emerton, 1882

##### Notes

BOLD:AAI8098

#### Walckenaeria directa

O. P.-Cambridge, 1874

##### Notes

BOLD:AAH8313

#### Walckenaeria fallax

Millidge, 1983

##### Notes

BOLD:AAH8314

#### Walckenaeria pinocchio

Kaston, 1945

##### Notes

BOLD:ACT1115

#### Walckenaeria spiralis

Emerton, 1882

##### Notes

BOLD:AAH8314

#### Lycosidae

#### Arctosa emertoni

Gertsch, 1934

##### Notes

BOLD:ACW1682

#### Pardosa distincta

Blackwall, 1846

##### Notes

BOLD:AAC7802

#### Pardosa milvina

Hentz, 1844

##### Notes

BOLD:AAB7668

#### Pardosa modica

Blackwall, 1846

##### Notes

BOLD:AAA5090

#### Pardosa moesta

Banks, 1892

##### Notes

BOLD:AAB0863

#### Pardosa saxatilis

Hentz, 1844

##### Notes

BOLD:AAB7667

#### Pirata piraticus

Clerck, 1757

##### Notes

BOLD:AAB6784

#### Pirata praedo

Kulczynski, 1885

##### Notes

BOLD:AAC5349

#### Piratula cantralli

Wallace & Exline, 1978

##### Notes

BOLD:ABZ5613

#### Piratula minuta

Emerton, 1885

##### Notes

BOLD:AAE4247

#### Schizocosa avida

Walckenaer, 1837

##### Notes

BOLD:AAD3880

#### Schizocosa crassipalpata

Roewer, 1951

##### Notes

BOLD:AAC4687

#### Schizocosa mccooki

Montgomery, 1904

##### Notes

BOLD:AAH0055

#### Schizocosa ocreata

Hentz, 1844

##### Notes

BOLD:AAA7232

#### Trochosa ruricola

De Geer, 1778

##### Notes

BOLD:AAB0726

#### Trochosa terricola

Thorell, 1856

##### Notes

BOLD:AAB0727

#### Mimetidae

#### Mimetus epeiroides

Emerton, 1882

##### Notes

BOLD:AAG5658

#### Mimetus haynesi

Gertsch & Mulaik, 1940

##### Notes

BOLD:AAK6284

#### Mimetus notius

Chamberlin, 1923

#### Philodromidae

#### Philodromus cespitum

Walckenaer, 1802

##### Notes

BOLD:AAB3836

#### Philodromus imbecillus

Keyserling, 1880

##### Notes

BOLD:AAI2838

#### Philodromus praelustris

Keyserling, 1880

##### Notes

BOLD:AAD2665

#### Philodromus rufus vibrans

Dondale

#### Thanatus coloradensis

Keyserling, 1880

##### Notes

BOLD:AAM7986

#### Tibellus maritimus

Menge, 1875

##### Notes

BOLD:AAA7188

#### Tibellus oblongus

Walckenaer, 1802

##### Notes

BOLD:AAA7188

#### Phrurolithidae

#### Phrurotimpus borealis

Emerton, 1911

##### Notes

BOLD:AAC7234

#### Scotinella pugnata

Emerton, 1890

##### Notes

BOLD:AAK7452

#### Pisauridae

#### Dolomedes striatus

Giebel, 1869

##### Notes

BOLD:ACI5773

#### Dolomedes tenebrosus

Hentz, 1844

#### Pisaurina mira

Walckenaer, 1837

##### Notes

BOLD:AAI2721

#### Salticidae

#### Eris militaris

Hentz, 1845

##### Notes

BOLD:AAA5654

#### Evarcha hoyi

Peckham & Peckham, 1883

##### Notes

BOLD:AAC0342|BOLD:ACL8050

#### Habronattus decorus

Blackwall, 1846

#### Marpissa formosa

Banks, 1892

##### Notes

BOLD:AAG0312

#### Naphrys pulex

Hentz, 1846

##### Notes

BOLD:AAC2433

#### Neon nelli

Peckham & Peckham, 1888

##### Notes

BOLD:AAD9221

#### Pelegrina galathea

Walckenaer, 1837

##### Notes

BOLD:AAB2930

#### Pelegrina insignis

Banks, 1892

##### Notes

BOLD:AAB2928

#### Pelegrina proterva

Walckenaer, 1837

#### Phidippus audax

Hentz, 1845

#### Phidippus clarus

Keyserling, 1885

#### Salticus scenicus

Clerck, 1757

#### Sitticus floricola palustris

Peckham and Peckham, 1883

#### Synageles noxiosus

Hentz, 1850

##### Notes

BOLD:ACL8115

#### Tutelina harti

Peckham, 1891

##### Notes

BOLD:AAW8769

#### Tutelina similis

Banks, 1895

##### Notes

BOLD:AAF6387

#### Zygoballus nervosus

Peckham & Peckham, 1888

##### Notes

BOLD:ACA1490

#### Tetragnathidae

#### Leucauge venusta

Walckenaer, 1841

##### Notes

BOLD:AAB8714

#### Pachygnatha dorothea

McCook, 1894

##### Notes

BOLD:AAE5304

#### Pachygnatha tristriata

C. L. Koch, 1845

##### Notes

BOLD:AAF1571

#### Pachygnatha xanthostoma

C. L. Koch, 1845

##### Notes

BOLD:ACO7247|BOLD:ACP5884|BOLD:ACU5364

#### Tetragnatha caudata

Emerton, 1884

##### Notes

BOLD:AAE3958|BOLD:ACN4034|BOLD:AAP3715

#### Tetragnatha extensa

Linnaeus, 1758

#### Tetragnatha guatemalensis

O. P.-Cambridge, 1889

#### Tetragnatha laboriosa

Hentz, 1850

##### Notes

BOLD:AAA6381|BOLD:ACR6860

#### Tetragnatha shoshone

Levi, 1981

##### Notes

BOLD:AAB7995

#### Tetragnatha straminea

Emerton, 1884

##### Notes

BOLD:AAD7095

#### Tetragnatha viridis

Walckenaer, 1841

##### Notes

BOLD:AAG5659|BOLD:AAN6335

#### Theridiidae

#### Dipoena nigra

Emerton, 1882

##### Notes

BOLD:AAF4974

#### Enoplognatha caricis

Fickert, 1876

##### Notes

BOLD:AAO3896

#### Enoplognatha ovata

Clerck, 1757

##### Notes

BOLD:AAA6910

#### Euryopis funebris

Hentz, 1850

##### Notes

BOLD:AAJ0542

#### Hentziectypus globosus

Hentz, 1850

##### Notes

BOLD:AAN6263

#### Neospintharus trigonum

Hentz, 1850

##### Notes

BOLD:AAB0273

#### Neottiura bimaculata

Linnaeus, 1767

##### Notes

BOLD:AAK8332|BOLD:ACN7831

#### Parasteatoda tabulata

Levi, 1980

#### Parasteatoda tepidariorum

C. L. Koch, 1841

##### Notes

BOLD:AAC0175

#### Theridion albidum

Banks, 1895

##### Notes

BOLD:AAV3042

#### Theridion differens

Emerton, 1882

##### Notes

BOLD:AAC3269

#### Theridion glaucescens

Becker, 1879

##### Notes

BOLD:AAG1794

#### Theridion murarium

Emerton, 1882

##### Notes

BOLD:AAC6350

#### Theridula emertoni

Levi, 1954

##### Notes

BOLD:AAD2291

#### Thymoites unimaculatus

Emerton, 1882

##### Notes

BOLD:AAE7853

#### Yunohamella lyrica

Walckenaer, 1841

##### Notes

BOLD:AAG4815

#### Thomisidae

#### Mecaphesa asperata

Hentz, 1847

##### Notes

BOLD:ACE7683

#### Misumena vatia

Clerck, 1757

##### Notes

BOLD:AAA6275

#### Misumenoides formosipes

Walckenaer, 1837

#### Misumessus oblongus

Keyserling, 1880

#### Ozyptila americana

Banks, 1895

#### Ozyptila praticola

C. L. Koch, 1837

##### Notes

BOLD:AAC7413

#### Tmarus angulatus

Walckenaer, 1837

##### Notes

BOLD:ABY7475

#### Xysticus bicuspis

Keyserling, 1887

##### Notes

BOLD:AAJ9685

#### Xysticus discursans

Keyserling, 1880

##### Notes

BOLD:AAJ9718|BOLD:ACV2014|BOLD:ACV5078

#### Xysticus elegans

Keyserling, 1880

##### Notes

BOLD:AAC1568

#### Xysticus emertoni

Keyserling, 1880

##### Notes

BOLD:AAB4300

#### Xysticus funestus

Keyserling, 1880

#### Xysticus luctans

C. L. Koch, 1845

##### Notes

BOLD:AAF8190

#### Xysticus punctatus

Keyserling, 1880

##### Notes

BOLD:AAD2346

#### Xysticus winnipegensis

Turnbull, Dondale & Redner, 1965

##### Notes

BOLD:AAM6956

#### Uloboridae

#### Uloborus glomosus

Walckenaer, 1841

##### Notes

BOLD:AAJ7823

#### Mesostigmata

#### Ascidae

#### Arctoseius cetratus

Sellnick, 1940

##### Notes

BOLD:ACF8021

#### Rhodacaridae

#### Rhodacarellus silesiacus

Willmann, 1935

#### Opiliones

#### Phalangiidae

#### Oligolophus tridens

C. L. Koch, 1836

##### Notes

BOLD:AAM8194

#### Phalangium opilio

Linnaeus, 1758

##### Notes

BOLD:AAI4346

#### Platybunus triangularis

Herbst, 1799

##### Notes

BOLD:ABW0506

#### Sclerosomatidae

#### Leiobunum aldrichi

Weed, 1893

##### Notes

BOLD:AAH7061

#### Leiobunum ventricosum

Wood, 1868

#### Sarcoptiformes

#### Euzetidae

#### Euzetes globulus

Nicolet, 1855

##### Notes

BOLD:AAF9090

#### Gustaviidae

#### Gustavia microcephala

Nicolet, 1855

##### Notes

BOLD:ACE3149

#### Liacaridae

#### Dorycranosus acutidens

Aoki, 1965

##### Notes

BOLD:AAF9274

#### Mochlozetidae

#### Podoribates pratensis

Banks, 1895

##### Notes

BOLD:AAF9173

#### Mycobatidae

#### Punctoribates punctum

Koch, 1839

##### Notes

BOLD:AAH6516|BOLD:ACB6310

#### Oppiidae

#### Oppia nitens

Koch, 1836

##### Notes

BOLD:AAF0868

#### Oribatulidae

#### Oribatula tibialis

Nicolet, 1855

##### Notes

BOLD:ACI4357

#### Scheloribatidae

#### Scheloribates clavilanceolatus

Ewing, 1907

##### Notes

BOLD:AAF9097

#### Tectocepheidae

#### Tectocepheus sarekensis

Trägårdh, 1910

##### Notes

BOLD:AAM3402|BOLD:AAM4355

#### Trombidiformes

#### Arrenuridae

#### Arrenurus reflexus

Marshall, 1908

##### Notes

BOLD:AAE6722

#### Branchiopoda

#### Diplostraca

#### Bosminidae

#### Bosmina liederi

De Melo and Hebert, 1994

#### Eurycercidae

#### Eurycercus longirostris

Hann, 1982

#### Chilopoda

#### Geophilomorpha

#### Schendylidae

#### Schendyla nemorensis

C.L.Koch, 1837

##### Notes

BOLD:AAG8560

#### Lithobiomorpha

#### Lithobiidae

#### Lithobius microps

Meinert, 1868

##### Notes

BOLD:AAH6432

#### Collembola

#### Entomobrya atrocincta

Schott, 1896

##### Notes

BOLD:ACE5102

#### Entomobryidae

#### Entomobrya nivalis

Linnaeus, 1758

##### Notes

BOLD:ACL6239

#### Lepidocyrtus paradoxus

Uzel, 1891

#### Orchesella villosa

Linnaeus, 1767

##### Notes

BOLD:AAA8726

#### Pseudosinella octopunctata

Boerner, 1901

##### Notes

BOLD:AAA9292

#### Isotomidae

#### Parisotoma notabilis

Schaffer, 1896

#### Poduromorpha

#### Hypogastruridae

#### Ceratophysella bengtssoni

Ågren, 1904

##### Notes

BOLD:AAI3738

#### Symphypleona

#### Dicyrtomidae

#### Dicyrtomina minuta

Fabricius, 1783

#### Katiannidae

#### Sminthurinus elegans

Fitch, 1862

##### Notes

BOLD:AAB3495|BOLD:AAB3496

#### Brachyiulus pusillus

Leach, 1815

##### Notes

BOLD:AAM7944

#### Julidae

#### Cylindroiulus caeruleocinctus

Wood, 1864

##### Notes

BOLD:AAH7472

#### Julus scandinavius

Latzel, 1884

##### Notes

BOLD:AAH7469

#### Ophyiulus pilosus

Newport, 1843

#### Insecta

#### Coleoptera

#### Anobiidae

#### Caenocara sp.

#### Ptilinus ruficornis

Say, 1823

#### Anthicidae

#### Malporus formicarius

LaFerté-Sénectère, 1849

##### Notes

BOLD:ACF8552

#### Notoxus desertus

Casey, 1895

##### Notes

BOLD:ABX0657

#### Stricticomus tobias

Marseul, 1879

##### Notes

BOLD:AAQ1028

#### Anthribidae

#### Anthribus nebulosus

Forster, 1770

##### Notes

BOLD:AAO1339

#### Ormiscus walshii

LeConte, 1876

##### Notes

BOLD:AAU7341

#### Buprestidae

#### Agrilus sulciollis

Lacordaire, 1835

#### Byrrhidae

#### Simplocaria semistriata

Fabricius, 1801

##### Notes

BOLD:ABW1696

#### Byturidae

#### Byturus unicolor

Say, 1823

#### Cantharidae

#### Cantharis rufa

Linnaeus, 1758

#### Chauliognathus pensylvanicus

DeGeer, 1774

#### Rhagonycha fulva

Scopoli, 1763

#### Rhaxonycha carolina

Fabricius, 1801

#### Carabidae

#### Agonoleptus conjunctus

Say, 1823

##### Notes

BOLD:AAE9008

#### Agonum fidele

Casey, 1920

#### Agonum gratiosum

Mannerheim, 1853

#### Amara angustata

Say, 1823

#### Amara rubrica

Haldeman, 1843

##### Notes

BOLD:AAM7658

#### Bembidion frontale

LeConte, 1847

##### Notes

BOLD:AAU7150

#### Bembidion obtusum

Audinet-Serville, 1821

##### Notes

BOLD:AAP9490

#### Calleida punctata

LeConte, 1846

#### Carabus granulatus

Linnaeus, 1758

#### Carabus nemoralis

Müller, 1764

#### Chlaenius tricolor

Dejean, 1826

#### Clivina fossor

Linnaeus, 1758

##### Notes

BOLD:AAH0274

#### Colliuris pensylvanica

Linnaeus, 1758

#### Dyschirius setosus

LeConte, 1857

#### Elaphrus clairvillei

Kirby, 1837

#### Lebia fuscata

Dejean, 1825

##### Notes

BOLD:AAH0212

#### Lebia solea

Hentz, 1830

#### Lebia viridis

Say, 1823

##### Notes

BOLD:AAH0141

#### Platynus hypolithos

Say, 1823

#### Pterostichus melanarius

Illiger, 1798

#### Cerambycidae

#### Acalymma vittata

Barber, 1947

#### Astylopsis macula

Say, 1826

#### Astylopsis sexguttata

Say, 1826

#### Bellamira scalaris

Say, 1826

#### Calligrapha californica coreopsivora

Brown

#### Chaetocnema sp.

#### Charidotella sexpunctata bicolor

Fabricius, 1798

#### Chrysochus auratus

Fabricius, 1775

#### Chrysolina hudsonica

Brown, 1938

#### Cicindela punctulata punctulata

Olivier

#### Cicindela sexguttata

Fabricius, 1775

#### Clytus ruricola

Olivier, 1795

#### Crepidodera sp.

#### Cyrtophorus verrucosus

Olivier, 1795

##### Notes

BOLD:AAD4513

#### Deloyala guttata

Olivier, 1790

#### Dibolia sp.

#### Epitrix sp.

#### Euderces picipes

Fabricius, 1787

#### Gaurotes cyanipennis

Say, 1824

##### Notes

BOLD:AAI7042

#### Longitarsus sp.

#### Mantura floridana

Crotch, 1873

#### Megacyllene robiniae

Forster, 1771

#### Molorchus bimaculatus

Say, 1824

##### Notes

BOLD:AAH0019

#### Neoclytus acuminatus acuminatus

Fabricius

#### Oberea tripunctata

Swederus, 1787

#### Obrium rufulum

Gahan, 1908

#### Oulema palustris

Blatchley, 1913

#### Paria sp.

#### Phyllotreta sp.

#### Phymatodes amoenus

Say, 1824

#### Plagiodera versicolor

Laicharting, 1781

#### Psylliodes punctulata

Melsheimer, 1847

#### Pyrrhalta sp.

#### Systena blanda

F. E. Melsheimer, 1847

#### Tetraopes tetrophthalmus

Forster, 1771

#### Tetrops praeusta

Linnaeus, 1758

##### Notes

BOLD:AAE9431

#### Trigonarthris proxima

Say, 1824

#### Trirhabda sp.

#### Typocerus velutinus velutinus

Olivier

#### Urgleptes querci

Fitch, 1858

#### Xylotrechus convergens

LeConte, 1873

#### Xylotrechus gemellus

Casey, 1893

#### Zeugophora varians

Crotch, 1873

#### Cerylonidae

#### Philothermus glabriculus

LeConte, 1863

##### Notes

BOLD:ABX9329

#### Chrysomelidae

#### Altica chalybea

Illiger, 1807

#### Altica sp.

#### Cassida rubiginosa

Müller, 1776

##### Notes

BOLD:AAO0522

#### Crepidodera heikertingeri

Lazorko, 1974

##### Notes

BOLD:AAG4462

#### Dibolia borealis

Chevrolat in Guérin-Méneville, 1834

##### Notes

BOLD:AAL0908

#### Epitrix cucumeris

Harris, 1851

##### Notes

BOLD:ABA9101

#### Exema canadensis

Pierce, 1940

#### Helocassis clavata

Fabricius, 1798

#### Microrhopala vittata

Fabricius, 1798

#### Neogalerucella calmariensis

Linnaeus, 1767

##### Notes

BOLD:AAL2945

#### Ophraella conferta

J. L. LeConte, 1865

##### Notes

BOLD:ACF8270

#### Paria fragariae

Wilcox, 1954

##### Notes

BOLD:ABA3960|BOLD:ACF6671

#### Phyllotreta striolata

Fabricius, 1801

##### Notes

BOLD:AAL5267

#### Plagiodera versicolora

Laicharting, 1781

#### Psylliodes affinis

Paykull, 1799

##### Notes

BOLD:AAU6967

#### Psylliodes napi

Fabricius, 1792

#### Psylliodes picinus

Marsham, 1802

#### Pyrrhalta viburni

Paykull, 1799

#### Trirhabda borealis

Blake, 1931

##### Notes

BOLD:AAG4458

#### Xanthonia decemnotata

Say, 1824

##### Notes

BOLD:ABA6335|BOLD:ACJ0239

#### Ciidae

#### Ceracis thoracicornis

Ziegler, 1845

#### Cleridae

#### Cymatodera bicolor

Say, 1825

##### Notes

BOLD:AAU6910

#### Enoclerus nigripes

Say, 1823

##### Notes

BOLD:AAU6970

#### Enoclerus rosmarus

Say, 1823

#### Isohydnocera curtipennis

Newman

#### Phyllobaenus verticalis

Say, 1835

#### Placopterus thoracicus

Olivier, 1795

##### Notes

BOLD:AAP8584

#### Zenodosus sanguineus

Say, 1835

##### Notes

BOLD:ABA6311

#### Coccinellidae

#### Coccinella septempunctata

Linnaeus, 1758

#### Coleomegilla maculata

De Geer, 1775

##### Notes

BOLD:AAD7604

#### Coleomegilla maculata lengi

Timberlake

#### Didion punctatum

Melsheimer, 1847

#### Harmonia axyridis

Pallas, 1773

##### Notes

BOLD:AAB5640

#### Hippodamia glacialis

Fabricius, 1775

##### Notes

BOLD:AAH3305

#### Hippodamia variegata

Goeze, 1777

#### Hyperaspis binotata

Say, 1826

#### Hyperaspis cf. binotata

#### Propylaea quatuordecimpunctata

Linnaeus, 1758

##### Notes

BOLD:AAF6935

#### Propylea quatuordecimpunctata

Linnaeus, 1758

#### Psyllobora vigintimaculata maculata

Say

#### Scymnus sp.

#### Stethorus punctillum

Weise, 1891

##### Notes

BOLD:AAN6149

#### Corylophidae

#### Orthoperus scutellaris

LeConte, 1878

##### Notes

BOLD:AAU7040

#### Sericoderus lateralis

Gyllenhal, 1827

##### Notes

BOLD:ABA2914

#### Cryptophagidae

#### Atomaria ephippiata

Zimmermann, 1869

#### Curculionidae

#### Acoptus suturalis

LeConte, 1876

##### Notes

BOLD:AAU6930

#### Barypeithes pellucidus

Boheman, 1834

##### Notes

BOLD:AAG5192

#### Hylesinus aculeatus

Say, 1824

##### Notes

BOLD:AAU7331

#### Hypera zoilus

Scopoli, 1763

#### Isochnus sequensi

Stierlin 1894

##### Notes

BOLD:ACA3052

#### Madarellus undulatus

Say, 1824

##### Notes

BOLD:AAY6533

#### Monarthrum mali

Fitch, 1855

##### Notes

BOLD:ACD0202

#### Orchestes alni

Linnaeus 1758

##### Notes

BOLD:AAM7726

#### Phyllobius oblongus

Linnaeus, 1758

##### Notes

BOLD:AAF9187

#### Pityogenes hopkinsi

Swaine, 1915

##### Notes

BOLD:ABW5076

#### Polydrusus impressifrons

Gyllenhal, 1834

##### Notes

BOLD:AAO4332

#### Sitona lineellus

Bonsdorff, 1785

#### Tychius meliloti

Stephens, 1831

##### Notes

BOLD:AAM7740

#### Xyleborinus alni

Niijima 1909

##### Notes

BOLD:AAB2754

#### Xyleborinus saxeseni

Ratzeburg, 1837

##### Notes

BOLD:AAB9578

#### Xyleborus dispar

Fabricius, 1792

##### Notes

BOLD:AAD0158

#### Xylosandrus germanus

Blandford, 1894

##### Notes

BOLD:AAF7523

#### Dytiscidae

#### Desmopachria convexa

Aubé, 1838

#### Elateridae

#### Aeolus mellillus

Say, 1836

#### Ampedus areolatus

Say, 1823

##### Notes

BOLD:ACM2015

#### Ampedus linteus

Say, 1839

##### Notes

BOLD:AAU7141

#### Ampedus nigricollis

Herbst, 1801

##### Notes

BOLD:AAH2376

#### Ampedus oblessus

Say, 1833

##### Notes

BOLD:ACA3849

#### Ampedus protervus

LeConte, 1853

##### Notes

BOLD:ACR3975

#### Athous brightwelli

Kirby, 1837

#### Corymbitodes tarsalis

Melsheimer, 1844

##### Notes

BOLD:ACV5201

#### Ctenicera cylindriformis

Herbst, 1806

##### Notes

BOLD:AAH2370

#### Dalopius vagus

Brown, 1934

#### Hemicrepidius memnonius

Herbst, 1806

#### Melanotus castanipes

Paykull, 1800

##### Notes

BOLD:AAH2378

#### Endomychidae

#### Mycetina perpulchra

Newman, 1838

#### Phymaphora pulchella

Newman, 1838

##### Notes

BOLD:ACI7114

#### Erotylidae

#### Triplax flavicollis

Lacordaire, 1842

#### Triplax thoracica

Say, 1825

#### Tritoma pulchra

Say, 1826

#### Tritoma sanguinipennis

Say, 1825

#### Eucnemidae

#### Dirrhagofarsus lewisi

Fleutiaux, 1900

#### Hylis terminalis

LeConte, 1866

#### Isorhipis obliqua

Say, 1836

#### Microrhagus sp.

#### Microrhagus subsinuata

LeConte

#### Microrhagus triangularis

Say, 1823

#### Gyrinidae

#### Dineutus assimilis

Kirby, 1937

#### Haliplidae

#### Haliplus immaculicollis

Harris, 1828

#### Hydrophilidae

#### Anacaena lutescens

Stephens, 1829

#### Cercyon haemorrhoidalis

Fabricius, 1775

##### Notes

BOLD:ABV1545

#### Enochrus ochraceus

Melsheimer, 1844

#### Tropisternus natator

Orchymont, 1938

#### Kateretidae

#### Brachypterolus pulicarius

Linnaeus, 1758

#### Lampyridae

#### Ellychnia corrusca

Linnaeus, 1767

##### Notes

BOLD:ACV4844

#### Lucidota atra

G. Olivier, 1790

#### Photinus sp.

#### Pyractomena sp.

#### Pyropyga nigricans

Say, 1823

#### Languriidae

#### Acropteroxys gracilis

Newman 1838

#### Latridiidae

#### Cortinicara gibbosa

Herbst, 1793

##### Notes

BOLD:AAI8935

#### Leiodidae

#### Anisotoma obsoleta

Horn, 1880

##### Notes

BOLD:AAR3435

#### Anisotoma sp.

#### Catops paramericanus

Peck and Cook, 2002

##### Notes

BOLD:AAH3504

#### Catops sp.

#### Lionothus sp.

#### Nemadus sp.

#### Prionochaeta opaca

Say, 1825

##### Notes

BOLD:AAP6949

#### Ptomophagus sp.

#### Sciodrepoides fumatus terminans

LeConte

#### Melandryidae

#### Dircaea liturata

LeConte, 1866

#### Epicauta pensylvanica

De Geer, 1775

#### Melandrya striata

Say, 1824

##### Notes

BOLD:AAK7242

#### Meloidae

#### Epicauta murina

LeConte, 1853

#### Meloe impressus

Kirby, 1837

#### Melyridae

#### Collops quadrimaculata

Fabricius, 1798

#### Hypebaeus apicalis

Say, 1825

##### Notes

BOLD:AAN5932

#### Mordellidae

#### Mordellina infima

LeConte, 1862

#### Mordellistena andreae

LeConte, 1862

#### Mordellistena bifasciata

Ray, 1936

#### Mordellistena cervicalis

LeConte, 1862

#### Mordellistena cf. lutea

#### Mordellistena ornata

Melsheimer, 1845

#### Mordellistena picilabris

Helmuth, 1864

#### Mordellistena sp.

#### Mordellochroa scapularis

Say, 1824

##### Notes

BOLD:AAU6912

#### Paramordellaria triloba

Say, 1824

#### Tomoxia lineela

LeConte

#### Mycetophagidae

#### Mycetophagus pluripunctatus

LeConte, 1856

#### Nitidulidae

#### Carpophilus sayi

Parsons, 1943

#### Conotelus obscurus

Erichson, 1843

#### Glischrochilus fasciatus

Olivier, 1790

#### Glischrochilus quadrisiginatus

Say 1835

#### Glischrochilus sanguinolentus sanguinolentus

Olivier, 1790

#### Omosita colon

Linnaeus, 1758

#### Stelidota octomaculata

Say, 1825

##### Notes

BOLD:AAH0115

#### Pedilidae

#### Pedilus lugubris

Say

#### Phalacridae

#### Acylomus pugetanus

Casey, 1916

##### Notes

BOLD:AAH0135

#### Olibrus semistriatus

LeConte, 1856

#### Stilbus apicialis

Melsheimer, 1844

##### Notes

BOLD:AAH0134

#### Psephenidae

#### Psephenus herricki

DeKay, 1844

#### Ptilodactylidae

#### Ptilodactyla sp.

#### Ptinidae

#### Hadrobregmus notatus

Say, 1825

##### Notes

BOLD:AAP8586

#### Pyrochroidae

#### Dendroides canadensis

Leconte

#### Schizotus cervicalis

Newman, 1838

#### Rhipiphoridae

#### Pelecotoma flavipes

Melsheimer, 1846

#### Ripiphorus fasciatus

Say, 1824

#### Scarabaeidae

#### Amphimallon majale

Razumowski, 1789

#### Aphodius granarius

Linnaeus, 1767

#### Ataenius strigatus

Say, 1823

#### Calamosternus granarius

Linnaeus, 1767

##### Notes

BOLD:AAM7733

#### Onthophagus orpheus canadensis

Fabricius, 1801

#### Osmoderma scabra

Palisot de Beauvois, 1805

#### Phyllophaga futilis

LeConte, 1850

##### Notes

BOLD:AAD1098

#### Phyllophaga rugosa

Melsheimer, 1845

##### Notes

BOLD:AAJ2312

#### Popillia japonica

Newman, 1841

#### Rhyssemus germanus

Linnaeus, 1767

#### Trox scabrosus

Beauvois, 1805

#### Scirtidae

#### Cyphon laevipennis

Tournier, 1868

##### Notes

BOLD:AAG3633

#### Cyphon obscurus

Guerin and Memeville, 1834

##### Notes

BOLD:AAG7259

#### Cyphon pusillus

LeConte

##### Notes

BOLD:AAP7021

#### Scirtes tibialis

Guerin

#### Scolytidae

#### Hylurgopinus rufipes

Eichhoff, 1868

#### Scolytus mali

Bechstein, 1805

#### Xyleborus sayi

Hopkins, 1915

#### Scraptiidae

#### Anaspis rufa

Say, 1826

##### Notes

BOLD:AAH0469

#### Canifa pallipes

Melsheimer, 1846

#### Silphidae

#### Necrophila americana

Linnaeus, 1758

#### Nicrophorus orbicollis

Say, 1825

##### Notes

BOLD:AAE1939

#### Oiceoptoma inaequale

Fabricius, 1781

#### Silvanidae

#### Ahasverus advena

Waltl, 1834

##### Notes

BOLD:AAJ2005

#### Silvanus bidentatus

Fabricius, 1792

##### Notes

BOLD:AAO0157

#### Telephanus velox

Haldeman, 1846

##### Notes

BOLD:AAW6380

#### Staphylinidae

#### Amischa analis

Gravenhorst, 1802

##### Notes

BOLD:ABA5313

#### Anotylus insecatus

Gravenhorst, 1806

##### Notes

BOLD:AAR3352

#### Anotylus tetracarinatus

Block, 1799

#### Atheta brunneipennis

Thomson, 1852

##### Notes

BOLD:ABA9094

#### Bisnius blandus

Gravenhorst, 1806

#### Carpelimus fuliginosus

Gravenhorst, 1802

##### Notes

BOLD:AAO0558

#### Coproporus ventriculus

Say, 1834

##### Notes

BOLD:ACV1788

#### Lordithon appalachianus

Campbell, 1982

##### Notes

BOLD:ABA6331

#### Lordithon cinctus

Gravenhorst, 1802

##### Notes

BOLD:ABA6370

#### Meronera venustula

Erichson, 1839

##### Notes

BOLD:ABW2870

#### Myllaena arcana

Casey, 1911

##### Notes

BOLD:ACJ6804

#### Philonthus caeruleipennis

Mannerheim, 1830

#### Philonthus flavibasis

Casey, 1915

##### Notes

BOLD:AAH0113

#### Platydracus cinnamopterus

Gravenhorst, 1802

##### Notes

BOLD:ACJ0017

#### Scaphidium quadriguttatum

Melsheimer

##### Notes

BOLD:ACP0011

#### Sepedophilus cinctulus

Erichson, 1839

##### Notes

BOLD:ACC1294

#### Sepedophilus testaceus

Fabricius, 1793

##### Notes

BOLD:AAH0108

#### Stenichnus scutellaris

Muller & Kunze, 1822

##### Notes

BOLD:AAN9916

#### Tachinus corticinus

Gravenhorst, 1802

##### Notes

BOLD:AAH0107

#### Tachyporus atriceps

Stephens, 1832

##### Notes

BOLD:ABX2484

#### Tachyporus chrysomelinus

Linnaeus, 1758

##### Notes

BOLD:AAN9511

#### Tachyporus elegans

Horn, 1877

##### Notes

BOLD:AAU6934

#### Tachyporus nitidulus

Fabricius, 1781

##### Notes

BOLD:ABA9096

#### Trichophya pilicornis

Gyllenhal, 1810

##### Notes

BOLD:ABW9580

#### Xantholinus linearis

Olivier, 1795

##### Notes

BOLD:AAG4333

#### Stenotrachelidae

#### Cephaloon lepturoides

Newman, 1838

#### Synchroidae

#### Mallodrya subaenea

Horn, 1888

##### Notes

BOLD:AAK7440

#### Synchroa punctata

Newman, 1838

#### Tetratomidae

#### Eustrophus tomentosus

Say, 1827

##### Notes

BOLD:ACI7017

#### Throscidae

#### Aulonothroscus constrictor

Say, 1839

##### Notes

BOLD:AAU7339

#### Aulonothroscus distans

Blanchard, 1917

##### Notes

BOLD:ABA9083

#### Aulonothroscus sp.

#### Trixagus carinicollis

Schaeffer, 1916

##### Notes

BOLD:AAN6148

#### Trixagus chevrolati

Bonvouloir, 1859

#### Trogossitidae

#### Tenebroides corticalis

Melsheimer, 1844

#### Dermaptera

#### Forficulidae

#### Forficula auricularia

Linnaeus, 1758

#### Forficula auricularia-A

Linnaeus, 1758

##### Notes

BOLD:AAG9897

### Checklist of species observed or collected at the *rare* Charitable Research Reserve in Cambridge, Ontario, Canada. The second of five checklists for Kingdom Animalia, this checklist contains members of Phylum Arthopoda, Class Insecta (Orders Diptera, Ephemeroptera, and Hemiptera).

#### Arthropoda

#### Animalia

#### Insecta

#### Diptera

#### Agromyzidae

#### Agromyza frontella

Rondani, 1874

##### Notes

BOLD:AAJ7105

#### Aulagromyza luteoscutellata

de Meijere, 1924

##### Notes

BOLD:AAJ9681

#### Calycomyza majuscula

Frick, 1956

##### Notes

BOLD:AAV4861

#### Cerodontha biseta

Hendel, 1920

#### Cerodontha dorsalis

Loew, 1863

#### Cerodontha fasciata

Strobl, 1880

#### Cerodontha muscina

Meigen, 1830

##### Notes

BOLD:AAF1051

#### Chromatomyia lactuca

Frost, 1924

#### Japanagromyza viridula

Coquillett, 1902

##### Notes

BOLD:AAI7960

#### Liriomyza brassicae

Riley, 1885

##### Notes

BOLD:AAF6806

#### Liriomyza fricki

Spencer, 1965

##### Notes

BOLD:ACE7414

#### Nemorimyza posticata

Meigen, 1830

##### Notes

BOLD:AAG9234|BOLD:ACJ0616

#### Ophiomyia labiatarum

Hering, 1937

#### Ophiomyia nasuta

Melander, 1913

##### Notes

BOLD:AAK5607

#### Ophiomyia quinta

Spencer, 1969

##### Notes

BOLD:AAI3360|BOLD:ABZ1036

#### Ophiomyia similata

Malloch, 1918

##### Notes

BOLD:AAP8823|BOLD:ACV3095|BOLD:ACV5944

#### Phytoliriomyza dorsata

Siebke, 1864

##### Notes

BOLD:AAG4751

#### Phytoliriomyza robiniae

Valley, 1982

##### Notes

BOLD:AAY1337

#### Phytomyza flavicornis

Fallen, 1823

##### Notes

BOLD:AAH9376

#### Phytomyza solidaginophaga

Sehgal, 1971

##### Notes

BOLD:AAL4176

#### Pseudonapomyza europaea

Spencer, 1973

#### Anisopodidae

#### Sylvicola alternatus

Say, 1823

##### Notes

BOLD:AAG2000

#### Sylvicola fuscatus

Fabricius, 1775

##### Notes

BOLD:AAG1998

#### Anthomyiidae

#### Anthomyia pluvialis

Linnaeus, 1758

##### Notes

BOLD:AAP2970

#### Delia antiqua

Meigen, 1826

#### Delia platura

Meigen, 1826

##### Notes

BOLD:AAA3453

#### Eustalomyia festiva

Zetterstedt, 1845

##### Notes

BOLD:AAP8833

#### Eutrichota pilimana

Ringdahl, 1918

##### Notes

BOLD:AAP2968

#### Hylemyza partita

Meigen, 1826

##### Notes

BOLD:AAG2463

#### Pegomya flavifrons

Walker, 1849

##### Notes

BOLD:AAG2479

#### Anthomyzidae

#### Mumetopia occipitalis

Melander, 1913

##### Notes

BOLD:AAG4827

#### Stiphrosoma balteatum

Rohacek & Barber, 2005

#### Asilidae

#### Dioctria baumhaueri

Meigen, 1820

#### Efferia aestuans

Linnaeus, 1763

#### Efferia albibarbis

Macquart, 1838

#### Laphria canis

Williston, 1883

#### Laphria canis complex

Williston, 1883

#### Laphria cinerea

Back, 1904

#### Laphria flavicollis

Say, 1824

#### Laphria janus

McAtee, 1919

#### Laphria sicula

McAtee, 1919

#### Laphria thoracica

Fabricius, 1805

#### Machimus sadyates

Walker, 1849

#### Aulacigastridae

#### Aulacigaster neoleucopeza

Mathis & Freidberg, 1994

##### Notes

BOLD:AAV0437|BOLD:ABV3853

#### Bombyliidae

#### Anthrax irroratus

Say, 1823

#### Bombylius major

Linnaeus, 1758

##### Notes

BOLD:ABV0388

#### Hemipenthes morio

Linnaeus, 1758

#### Hemipenthes sinuosa

Wiedemann, 1821

#### Hemipenthes webberi

Johnson, 1919

#### Xenox tigrinus

De Geer, 1776

#### Calliphoridae

#### Calliphora livida

Hall, 1948

##### Notes

BOLD:ABY7153

#### Cynomya cadaverina

Robineau-Desvoidy, 1830

##### Notes

BOLD:AAB0868

#### Phormia regina

Meigen, 1826

##### Notes

BOLD:AAB9140

#### Pollenia angustigena

Wainwright, 1940

##### Notes

BOLD:AAP2825

#### Pollenia griseotomentosa

Jacentkovsky, 1944

##### Notes

BOLD:AAI2766

#### Pollenia labialis

Robineau-Desvoidy, 1863

##### Notes

BOLD:AAI2765

#### Pollenia pediculata

Macquart, 1834

##### Notes

BOLD:AAG6745

#### Pollenia rudis

Fabricius, 1794

##### Notes

BOLD:AAH3035

#### Cecidomyiidae

#### Asteromyia carbonifera

Osten Sacken, 1862

##### Notes

BOLD:AAA2254|BOLD:ABX5689

#### Asteromyia laeviana

Felt, 1907

##### Notes

BOLD:ABV1420

#### Asteromyia modesta

Felt, 1907

##### Notes

BOLD:AAM1947|BOLD:AAM1948|BOLD:AAM1954|BOLD:ACG8775|BOLD:ACN2213

#### Asteromyia tumifica

Beutenmuller, 1907

##### Notes

BOLD:ACL0470

#### Janetiella glechomae

Tavares, 1930

##### Notes

BOLD:AAQ0642

#### Mayetiola destructor

Say, 1817

##### Notes

BOLD:ABV9277

#### Ceratopogonidae

#### Stilobezzia antennalis

Coquillett, 1901

#### Chironomidae

#### Bryophaenocladius ictericus

Meigen, 1830

##### Notes

BOLD:AAM6273

#### Bryophaenocladius sp. 8ES

##### Notes

BOLD:AAG1021

#### Camptocladius stercorarius

De Geer, 1776

##### Notes

BOLD:AAN5341

#### Chironomus acidophilus

Keyl, 1960

##### Notes

BOLD:AAJ4295

#### Chironomus dilutus

Kiknadze & Butler, 1999

##### Notes

BOLD:AAB4658

#### Chironomus maturus

Johannsen, 1908

##### Notes

BOLD:AAB4657

#### Chironomus melanescens

Keyl, 1961

##### Notes

BOLD:AAI4303

#### Chironomus ochreatus

Townes, 1945

##### Notes

BOLD:ACV5571

#### Cladotanytarsus atridorsum

Kieffer, 1924

##### Notes

BOLD:AAJ3263

#### Conchapelopia telema

Roback, 1971

##### Notes

BOLD:AAC4802|BOLD:AAN5351

#### Corynoneura scutellata

Winnertz, 1846

##### Notes

BOLD:AAN5330

#### Cricotopus annulator cmplx

Goetghebuer, 1927

#### Cricotopus bicinctus

Meigen, 1818

##### Notes

BOLD:ACC7282

#### Cricotopus sp. 18ES

#### Cricotopus sp. 19ES

##### Notes

BOLD:AAP5141

#### Cricotopus tremulus

Linnaeus, 1758

##### Notes

BOLD:AAE4298

#### Cricotopus triannulatus

Macquart, 1826

##### Notes

BOLD:AAP5920

#### Cricotopus trifascia

Edwards, 1929

##### Notes

BOLD:ACS9429|BOLD:ACT0257

#### Cricotopus vierriensis

Goetghebuer, 1935

##### Notes

BOLD:AAG1005|BOLD:ACV5403|BOLD:ACV5404

#### Dicrotendipes modestus

Say, 1823

##### Notes

BOLD:AAL7329

#### Dicrotendipes tritomus

Kieffer, 1916

##### Notes

BOLD:AAC0706

#### Gymnometriocnemus brumalis

Edwards, 1929

##### Notes

BOLD:AAP6873

#### Lauterborniella agrayloides

Kieffer, 1911

##### Notes

BOLD:AAN5343

#### Limnophyes natalensis

Kieffer, 1914

##### Notes

BOLD:AAB7361

#### Limnophyes sp. 14ES

##### Notes

BOLD:ABU5525

#### Metriocnemus sp. 4ES

##### Notes

BOLD:ABX5809

#### Micropsectra nigripila

Johannsen, 1905

#### Micropsectra subletteorum

Anderson, Stur & Ekrem, 2013

##### Notes

BOLD:AAF7088

#### Microtendipes pedellus

De Geer, 1776

##### Notes

BOLD:AAE0707

#### Monopelopia tenuicalcar

Kieffer, 1918

##### Notes

BOLD:AAM6277

#### Nanocladius anderseni

Saether, 1977

##### Notes

BOLD:AAC3041

#### Nilotanypus fimbriatus

Walker, 1828

##### Notes

BOLD:AAE5762

#### Orthocladius carlatus

Roback, 1957

##### Notes

BOLD:AAG1000

#### Orthocladius dorenus

Roback, 1957

##### Notes

BOLD:AAB2641|BOLD:AAB2645|BOLD:ACV3368

#### Orthocladius oliveri

Soponis, 1977

##### Notes

BOLD:AAB7872

#### Orthocladius rivulorum

Kieffer, 1909

##### Notes

BOLD:AAB3988

#### Pagastia orthogonia

Oliver, 1959

##### Notes

BOLD:AAI2601

#### Paraphaenocladius impensus

Walker, 1856

##### Notes

BOLD:AAC4197

#### Paratanytarsus dissimilis

Johannsen, 1905

##### Notes

BOLD:AAE3698

#### Paratanytarsus grimmii

Schneider, 1885

##### Notes

BOLD:AAD1485

#### Paratanytarsus laccophilus

Edwards, 1929

##### Notes

BOLD:AAC8842|BOLD:ACF2457

#### Paratanytarsus sp. 7TE

##### Notes

BOLD:AAP2907

#### Paratanytarsus sp. TE03

##### Notes

BOLD:AAE3675

#### Polypedilum convictum

Walker, 1856

##### Notes

BOLD:AAD1397

#### Psectrocladius obvius

Walker, 1856

##### Notes

BOLD:AAF6432

#### Rheocricotopus robacki

Beck and Beck, 1964

##### Notes

BOLD:AAM6249

#### Rheotanytarsus pellucidus

Walker, 1848

##### Notes

BOLD:AAI0332

#### Smittia edwardsi

Goetghebuer, 1932

##### Notes

BOLD:AAF4817

#### Smittia sp. 14ES

##### Notes

BOLD:AAM7064

#### Smittia sp. 22ES

##### Notes

BOLD:AAN5358

#### Smittia sp. 23ES

##### Notes

BOLD:AAH9641

#### Smittia sp. 8ES

##### Notes

BOLD:ACP4736

#### Stempellinella fimbriata

Ekrem, 2007

##### Notes

BOLD:AAD0300

#### Tanytarsus glabrescens

Edwards, 1929

##### Notes

BOLD:ACV5604|BOLD:ACV5898

#### Tanytarsus guerlus

Roback, 1957

##### Notes

BOLD:AAC4523|BOLD:AAC4525

#### Tanytarsus mendax

Kieffer, 1925

##### Notes

BOLD:ACJ3722|BOLD:ACV3832

#### Tanytarsus recurvatus

Brundin, 1947

##### Notes

BOLD:AAC3354

#### Tanytarsus wirthi

Ekrem, Sublette & Sublette, 2003

##### Notes

BOLD:AAD2144

#### Thienemanniella xena

Roback, 1957

##### Notes

BOLD:AAD5253|BOLD:AAD5254

#### Chloropidae

#### Chaetochlorops inquilinus

Coquillett, 1898

#### Elachiptera costata

Loew, 1863

#### Elachiptera nigriceps

Loew, 1863

##### Notes

BOLD:AAP5169

#### Elachiptera sibirica

Loew, 1858

##### Notes

BOLD:AAH4208

#### Eribolus longulus

Loew, 1863

#### Gaurax dubius

Macquart, 1835

##### Notes

BOLD:ACC7744

#### Gaurax pallidipes

Malloch, 1915

##### Notes

BOLD:AAH4210

#### Gaurax varihalteratus

Malloch, 1913

##### Notes

BOLD:ACM2340

#### Hapleginella conicola

Greene, 1918

#### Malloewia abdominalis

Becker, 1912

##### Notes

BOLD:ABW1379

#### Malloewia nigripalpis

Malloch, 1913

##### Notes

BOLD:ABZ4644

#### Olcella provocans

Becker, 1912

#### Oscinella frit

Linnaeus, 1758

#### Oscinisoma alienum

Becker, 1912

##### Notes

BOLD:ACE3223

#### Psilacrum arpidia

Malloch, 1916

##### Notes

BOLD:ACE0829

#### Rhopalopterum carbonarium

Loew, 1869

#### Thaumatomyia glabra

Meigen, 1830

##### Notes

BOLD:AAH4135

#### Chyromyidae

#### Gymnochiromyia concolor

Malloch, 1914

##### Notes

BOLD:ACV5890

#### Clusiidae

#### Clusia czernyi

Johnson, 1913

##### Notes

BOLD:AAF4394

#### Clusia lateralis

Walker, 1849

#### Clusiodes johnsoni

Malloch, 1922

##### Notes

BOLD:AAJ4032

#### Clusiodes melanostomus

Loew, 1864

##### Notes

BOLD:AAJ4031

#### Sobarocephala flaviseta

Johnson, 1913

##### Notes

BOLD:AAN5648

#### Sobarocephala setipes

Melander and Argo, 1924

#### Conopidae

#### Physocephala marginata

Say, 1823

#### Culicidae

#### Aedes canadensis

Theobald

#### Aedes cinereus

Meigen, 1818

##### Notes

BOLD:AAC1222

#### Aedes sp.

#### Aedes stimulans

(Walker, 1848)

#### Aedes vexans

Meigen, 1830

##### Notes

BOLD:AAA7067

#### Anopheles quadrimaculatus

Say, 1824

#### Coquillettidia perturbans

Walker, 1856

##### Notes

BOLD:AAB2539

#### Culex restuans

Theobald, 1901

##### Notes

BOLD:AAA7661

#### Culex territans

Walker, 1856

##### Notes

BOLD:AAB6943|BOLD:ABY7666

#### Mansonia perturbans

Walker

#### Dolichopodidae

#### Dolichopus orichalceus

Gosseries, 1989

#### Dolichopus terminalis

Loew, 1866

#### Gymnopternus celer

Meigen, 1824

#### Medetera signaticornis

Loew, 1857

##### Notes

BOLD:AAZ3931

#### Neurigona disjuncta

Van Duzee, 1913

##### Notes

BOLD:ABW1193

#### Xanthochlorus helvinus

Loew, 1861

#### Drosophilidae

#### Chymomyza amoena

Loew, 1862

##### Notes

BOLD:AAE2703

#### Drosophila affinis

Sturtevant, 1916

##### Notes

BOLD:AAB8851

#### Drosophila falleni

Wheeler, 1960

##### Notes

BOLD:AAB7507

#### Leucophenga varia

Walker, 1849

##### Notes

BOLD:AAG8500

#### Scaptomyza adusta

Loew, 1862

##### Notes

BOLD:AAG8491

#### Empididae

#### Rhamphomyia versicolor

Chillcott, 1959

##### Notes

BOLD:AAM7337

#### Ephydridae

#### Athyroglossa dinorata

Mathis & Zatwarnicki, 1990

#### Athyroglossa granulosa

Cresson, 1922

##### Notes

BOLD:ABY0801

#### Axysta extera

Cresson, 1942

#### Coenia curvicauda

Meigen, 1830

#### Discocerina obscurella

Fallen, 1813

#### Discomyza incurva

Fallen 1823

##### Notes

BOLD:ABA8754

#### Hyadina albovenosa

Coquillett, 1900

#### Hydrellia albilabris

Meigen, 1830

#### Hydrellia griseola

Fallen, 1813

#### Hydrellia ischiaca

Loew, 1862

#### Hydrellia notata

Deonier, 1971

#### Nostima scutellaris

Cresson, 1933

#### Ochthera anatolikos

Clausen, 1977

#### Parydra aquila

Fallen, 1813

#### Philygria debilis

Loew, 1861

#### Philygria obtecta

Becker, 1896

##### Notes

BOLD:AAG2740

#### Pseudohyadina longicornis

Sturtevant and Wheeler, 1954

#### Scatella favillacea

Loew, 1862

#### Scatella stagnalis

Fallen, 1813

#### Scatella tenuicosta

Collin, 1930

#### Scatophila despecta

Haliday, 1839

#### Scatophila virildella

Sturtevant & Wheeler, 1954

#### Fanniidae

#### Fannia armata

Meigen, 1826

##### Notes

BOLD:AAU6630

#### Heleomyzidae

#### Suillia quinquepunctata

Say, 1823

##### Notes

BOLD:AAC8595

#### Hybotidae

#### Leptopeza flavipes

Meigen, 1820

##### Notes

BOLD:ACE5974

#### Platypalpus annulatus

Fallen, 1815

#### Platypalpus holosericus

Melander, 1924

##### Notes

BOLD:AAP6357

#### Platypalpus melleus

Melander, 1927

##### Notes

BOLD:AAV3697

#### Platypalpus niger

Meigen, 1804

#### Platypalpus pulicarius

Meigen, 1830

##### Notes

BOLD:AAQ0265

#### Platypalpus stabilis

Collin, 1961

#### Platypalpus unguiculatus

Zetterstedt, 1838

##### Notes

BOLD:ABA0579

#### Tachydromia aemula

Loew, 1864

##### Notes

BOLD:AAN5500

#### Keroplatidae

#### Orfelia nemoralis

Meigen, 1818

##### Notes

BOLD:AAP2528

#### Lauxaniidae

#### Lauxania shewelli

Perusse & Wheeler 2000

#### Poecilominettia puncticeps

Coquillett, 1902

##### Notes

BOLD:AAN8633

#### Limoniidae

#### Epiphragma fasciapenne

Say, 1823

##### Notes

BOLD:ACL8650

#### Erioptera caliptera

Say, 1823

##### Notes

BOLD:AAN5882

#### Erioptera ebenina

Alexander, 1926

##### Notes

BOLD:ACB0353

#### Helius flavipes

Macq.

##### Notes

BOLD:AAF9008

#### Ilisia venusta

Osten Sacken, 1860

#### Ormosia affinis

Lundbeck, 1898

##### Notes

BOLD:AAU6544

#### Ormosia meigenii

Osten Sacken, 1859

##### Notes

BOLD:ACA9818

#### Pseudolimnophila inornata

Osten Sacken, 1869

##### Notes

BOLD:AAI1351

#### Lonchopteridae

#### Lonchoptera bifurcata

Fallen, 1810

#### Micropezidae

#### Compsobata univitta

Walker, 1849

##### Notes

BOLD:AAP8989

#### Rainieria antennaepes

Say, 1823

#### Milichiidae

#### Leptometopa latipes

Meigen, 1830

##### Notes

BOLD:AAP8985

#### Paramyia nitens

Loew, 1869

##### Notes

BOLD:AAG0166|BOLD:AAG0169

#### Pholeomyia indecora

Loew, 1869

#### Muscidae

#### Coenosia tigrina

Fabricius, 1775

#### Helina depuncta

Fallen, 1825

#### Helina evecta

Harris, 1780

##### Notes

BOLD:AAC2498

#### Helina rufitibia

Stein, 1898

##### Notes

BOLD:AAG1742

#### Lispe albitarsis

Stein, 1898

##### Notes

BOLD:AAP1125

#### Lispocephala erythrocera

(Robineau-Desvoidy, 1830)

#### Macrorchis ausoba

Walker, 1849

#### Muscina levida

Harris, 1788

##### Notes

BOLD:AAB8817

#### Myospila meditabunda

Fabricius, 1781

##### Notes

BOLD:AAD7145

#### Mycetophilidae

#### Aglaomyia gatineau

Vockeroth, 1980

##### Notes

BOLD:ABV3010

#### Exechia attrita

Johannsen, 1912

##### Notes

BOLD:ACM3454

#### Mycetophila caudata

Staeger, 1840

##### Notes

BOLD:AAI3260

#### Mycetophila fungorum

De Geer, 1776

##### Notes

BOLD:ACF2821

#### Mycetophila ocellus

Walker, 1848

##### Notes

BOLD:AAP4734

#### Paratinia recurva

Johannsen, 1910

#### Symmerus lautus

Loew, 1869

#### Trichonta submaculata

(Staeger, 1840)

##### Notes

BOLD:AAU4912

#### Zygomyia zaitzevi

Chandler, 1991

#### Odiniidae

#### Odinia betulae

Sabrosky, 1959

##### Notes

BOLD:AAP8071

#### Odinia meijerei

Collin, 1952

##### Notes

BOLD:ACV3828

#### Opomyzidae

#### Geomyza apicalis

(Meigen, 1830)

##### Notes

BOLD:ACM2703

#### Geomyza tripunctata

Fallen, 1823

#### Pediciidae

#### Pedicia inconstans

Osten Sacken, 1859

#### Phoridae

#### Conicera dauci

Meigen, 1830

##### Notes

BOLD:AAN8685

#### Megaselia arcticae

Disney, 2004

##### Notes

BOLD:AAG3248

#### Megaselia citrinella

Buck & Disney, 2001

#### Megaselia fungivora

Wood, 1909

#### Megaselia lucifrons

(Schmitz, 1918)

##### Notes

BOLD:AAL9075

#### Megaselia nigriceps

Loew, 1866

##### Notes

BOLD:AAY6384

#### Megaselia rufipes

Meigen, 1804

##### Notes

BOLD:AAG3274

#### Megaselia variana

Schmitz, 1926

##### Notes

BOLD:AAZ6701

#### Pipunculidae

#### Pipunculus hertzogi

Rapp, 1943

#### Psilidae

#### Loxocera cylindrica

Say, 1823

#### Psila lateralis

Loew, 1860

##### Notes

BOLD:AAF9707

#### Psila persimilis

Wakerly, 1959

#### Psila rosae

Fabricius, 1794

##### Notes

BOLD:AAP6388

#### Psychodidae

#### Psychoda trinodulosa

Tonnoir, 1922

##### Notes

BOLD:AAN8770

#### Rhagionidae

#### Bolbomyia nana

Loew, 1862

##### Notes

BOLD:ACV5660

#### Chrysopilus thoracicus

Fabricius, 1805

#### Rhagio tringarius

Linnaeus, 1758

#### Sarcophagidae

#### Boettcheria bisetosa

Parker, 1914

##### Notes

BOLD:AAH7139

#### Boettcheria cimbicis

Townsend, 1892

#### Sarcophaga subvicina

Baranov, 1937

##### Notes

BOLD:AAG6743

#### Senotainia trilineata

Wulp, 1890

##### Notes

BOLD:AAG6744

#### Scathophagidae

#### Americina adusta

Loew, 1863

##### Notes

BOLD:AAH4235

#### Megaphthalma pallida

Fallen, 1819

##### Notes

BOLD:AAH4234

#### Parallelomma vittatum

Meigen, 1826

#### Scathophaga furcata

Say, 1823

##### Notes

BOLD:AAH0022

#### Sciaridae

#### Bradysia difformis

Frey, 1948

##### Notes

BOLD:AAV1295

#### Bradysia fenestralis

Zetterstedt, 1838

##### Notes

BOLD:AAV1366

#### Bradysia nitidicollis

Meigen, 1818

#### Bradysia pallipes

Fabricius, 1787

##### Notes

BOLD:AAM9254

#### Bradysia scabricornis

Tuomikoski, 1960

##### Notes

BOLD:ABA0929

#### Bradysia vagans

Winnertz, 1868

##### Notes

BOLD:AAM9252

#### Camptochaeta uniformis

Mohrig & Menzel, 1990

##### Notes

BOLD:ACA4924

#### Corynoptera bicuspidata

Lengersdorf, 1926

##### Notes

BOLD:AAU6513

#### Corynoptera cuniculata

Lengersdorf, 1942

##### Notes

BOLD:AAU6537

#### Corynoptera melanochaeta

Mohrig & Menzel, 1992

##### Notes

BOLD:AAM9242

#### Corynoptera saccata

Tuomikoski, 1960

##### Notes

BOLD:AAN6437

#### Corynoptera subcavipes

Menzel & Smith, 2007

##### Notes

BOLD:AAU6542

#### Cratyna ambigua

Lengersdorf, 1934

##### Notes

BOLD:AAH3968|BOLD:AAN6439

#### Ctenosciara hyalipennis

Meigen, 1804

##### Notes

BOLD:AAH3983

#### Leptosciarella scutellata

Staeger, 1840

##### Notes

BOLD:ACD1218

#### Lycoriella castanescens

Lengersdorf, 1940

##### Notes

BOLD:ABA1215

#### Lycoriella perochaeta

Mohrig & Menzel, 1990

##### Notes

BOLD:ACC1855

#### Lycoriella stylata

Mohrig & Mamaev, 1985

##### Notes

BOLD:AAN6430

#### Scatopsciara atomaria

Zetterstedt, 1851

##### Notes

BOLD:AAN6431

#### Sciara humeralis

Zetterstedt, 1851

#### Sciomyzidae

#### Poecilographa decorum

Loew, 1864

#### Pteromicra similis

Steyskal, 1954

##### Notes

BOLD:AAG6869

#### Tetanocera plumosa

Loew, 1847

#### Trypetoptera canadensis

Macquart, 1843

#### Sepsidae

#### Nemopoda nitidula

Fallen, 1820

##### Notes

BOLD:AAG5640

#### Saltella sphondylii

Schrank, 1803

#### Sepsis punctum

Fabricius, 1794

##### Notes

BOLD:AAG5639

#### Simuliidae

#### Prosimulium arvum

Adler and Kim, 1985

##### Notes

BOLD:AAD4764

#### Prosimulium mixtum

Syme and Davies, 1958

#### Simulium vittatum

Zetterstedt, 1838

##### Notes

BOLD:AAA4121

#### Sphaeroceridae

#### Apteromyia claviventris

Strobl, 1909

##### Notes

BOLD:AAG7283

#### Coproica acutangula

Zetterstedt, 1847

#### Coproica ferruginata

Stenhammar, 1854

##### Notes

BOLD:AAN6407

#### Coproica hirtula

Rondani, 1880

##### Notes

BOLD:ACF7714

#### Copromyza equina

Fallen, 1820

##### Notes

BOLD:AAJ7412

#### Gonioneura spinipennis

Haliday, 1836

#### Ischiolepta pusilla

Fallen, 1820

#### Leptocera caenosa

Rondani, 1880

#### Leptocera erythrocera

Becker, 1919

##### Notes

BOLD:AAG7276

#### Lotophila atra

Meigen, 1830

#### Minilimosina fungicola

Spuler, 1925

#### Minilimosina intercepta

Marshall, 1985

##### Notes

BOLD:AAG7309

#### Opalimosina mirabilis

Collin, 1902

#### Pullimosina heteroneura

Haliday, 1836

#### Pullimosina longicosta

Spuler, 1925

#### Pullimosina pullula

Zetterstedt, 1847

#### Rachispoda fumipennis-group

Spuler 1924

#### Rachispoda limosa

Fallén, 1820

#### Rachispoda lutosa-group

Stenhammar, 1855

#### Sclerocoelus sordipes

Adams 1904

#### Spelobia bifrons

Stenhammar, 1854

#### Spelobia clunipes

Meigen, 1830

#### Spelobia luteilabris

Rondani, 1880

#### Spelobia ochripes

Meigen, 1830

##### Notes

BOLD:AAG7279

#### Spelobia semioculata

Richards, 1965

#### Sphaerocera curvipes

Latreille, 1805

#### Telomerina flavipes

Meigen, 1830

##### Notes

BOLD:ACJ1971

#### Trachyopella nuda

Rohacek and Marshall, 1985

#### Stratiomyidae

#### Actina viridis

Say, 1824

##### Notes

BOLD:AAP7640

#### Allognosta fuscitarsis

Say, 1823

#### Allognosta obscuriventris

Loew, 1863

#### Caloparyphus tetraspilus

Loew, 1866

#### Euparyphus stigmaticalis

Loew, 1866

#### Microchrysa polita

Linnaeus, 1758

#### Nemotelus bruesii

Melander, 1903

#### Nemotelus centralis

Hanson, 1958

#### Psellidotus meganticus

Curran, 1925

#### Ptecticus gigliotosi

McFadden, 1971

#### Sargus decorus

Say, 1824

#### Stratiomys norma

Wiedemann, 1830

#### Stratiomys obesa

Loew, 1866

#### Syrphidae

#### Allograpta micrura

Osten Sacken, 1877

#### Allograpta obliqua

Say, 1823

##### Notes

BOLD:AAD8276

#### Brachyopa sedmani

#### Brachypalpus oarus

Walker, 1849

##### Notes

BOLD:AAP8757

#### Chalcosyrphus libo

Walker, 1849

##### Notes

BOLD:AAG4679

#### Chalcosyrphus nemorum

Fabricius, 1805

##### Notes

BOLD:AAG6762

#### Chrysotoxum pubescens

Loew, 1864

#### Dasysyrphus venustus

Meigen

##### Notes

BOLD:ACV5348

#### Epistrophe nitidicollis

Meigen, 1822

#### Eristalis arbustorum

Linnaeus, 1758

#### Eristalis dimidiata

Wiedemann, 1830

#### Eristalis flavipes

Walker, 1849

#### Eristalis stipator

Osten Sacken, 1877

#### Eristalis tenax

Linnaeus, 1758

#### Eristalis transversa

Wiedemann, 1830

#### Eumerus sp.

#### Eupeodes americanus

Wiedemann, 1830

#### Eupeodes volucris

Osten Sacken, 1877

#### Ferdinandea buccata

Loew, 1863

##### Notes

BOLD:AAE0948

#### Helophilus fasciatus

Walker, 1849

#### Heringia salax

Loew, 1866

#### Lejota aerea

Loew, 1872

##### Notes

BOLD:AAY9807

#### Mallota posticata

Fabricius, 1805

#### Melanotstoma mellinum

Linnaeus 1758

#### Merodon equestris

Fabricius, 1794

#### Microdon tristis

Loew, 1864

#### Myolepta nigra

Loew, 1972

##### Notes

BOLD:AAV0836

#### Neoascia distincta

Williston, 1887

##### Notes

BOLD:AAG6766

#### Ocyptamus fuscipennis

Macquart, 1834

#### Orthonevra nitida

Wiedemann, 1830

#### Paragus haemorrhous

Meigen, 1822

##### Notes

BOLD:AAC2439

#### Parhelophilus laetus

Loew, 1963

#### Platycheirus hyperboreus

Staeger, 1845

##### Notes

BOLD:ACF4734

#### Platycheirus obscurus

Say, 1824

##### Notes

BOLD:AAF1237

#### Platycheirus quadratus

Say, 1823

#### Platycheirus scambus

Staeger, 1843

#### Sericomyia chrysotoxoides

Macquart, 1842

##### Notes

BOLD:ABX5395

#### Sphaerophoria asymmetrica

Knutson, 1973

#### Sphaerophoria bifurcata

Knutson, 1973

#### Sphaerophoria brevipilosa

Knutson, 1973

#### Sphaerophoria contigua

Macquart, 1847

#### Sphaerophoria philanthus

Meigen

#### Sphegina keeniana

Williston, 1887

##### Notes

BOLD:ACR0385

#### Sphegina petiolata

Coquillett, 1910

#### Spilomyia longicornis

Loew, 1872

#### Syritta pipiens

Linnaeus, 1758

#### Syrphus rectus

Osten Sacken, 1875

#### Syrphus ribesii

Linnaeus

##### Notes

BOLD:AAA4570

#### Syrphus torvus

Osten Sacken, 1875

##### Notes

BOLD:AAC6088

#### Toxomerus geminatus

Say, 1923

##### Notes

BOLD:AAC1312

#### Toxomerus marginatus

Say, 1823

##### Notes

BOLD:AAA4277

#### Xanthogramma flavipes

Loew, 1863

##### Notes

BOLD:AAK0114

#### Xylota quadrimaculata

Loew, 1866

#### Tabanidae

#### Chrysops aestuans

Wulp, 1867

#### Chrysops ater

Macquart, 1850

##### Notes

BOLD:ACE5640

#### Chrysops calvus

Pechuman and Teskey, 1967

#### Chrysops lateralis

Wiedemann, 1828

#### Chrysops striatus

Osten Sacken, 1875

#### Chrysops vittatus

Wiedemann, 1821

#### Hybomitra epistates

Osten Sacken, 1878

#### Hybomitra lasiophthalma

Macquart, 1838

##### Notes

BOLD:AAF0889

#### Tabanus lineola

Fabricius

#### Tachinidae

#### Actia interrupta

Curran, 1933

#### Blepharomyia pagana

Meigen, 1824

##### Notes

BOLD:AAV0903

#### Campylocheta teliosis

Reinhard, 1952

#### Ceracia dentata

Coquillett, 1895

##### Notes

BOLD:ABX6290

#### Homalactia harringtoni

Coquillett, 1902

##### Notes

BOLD:AAP2717

#### Leschenaultia exul

Townsend, 1892

##### Notes

BOLD:ACE2864

#### Lydina americana

Townsend, 1892

##### Notes

BOLD:AAG2432

#### Oswaldia minor

Curran, 1925

##### Notes

BOLD:ACF1129

#### Phorocera obscura

Fallen, 1810

##### Notes

BOLD:ABY8575

#### Siphona hokkaidensis

Mesnil, 1957

##### Notes

BOLD:AAG2172

#### Siphona intrudens

Curran, 1932

##### Notes

BOLD:AAP2721

#### Siphona pisinnia

O'Hara, 1983

##### Notes

BOLD:AAZ4865

#### Tephritidae

#### Euaresta bella

Loew, 1862

#### Eurosta solidaginis

Fitch, 1855

#### Eutreta novaeboracensis

Fitch, 1855

#### Rhagoletis suavis

Loew, 1862

#### Urophora cardui

Linnaeus, 1758

#### Xanthomyia platyptera

Loew, 1873

#### Tipulidae

#### Nephrotoma cornicina

Linnaeus, 1758

#### Tipula (Beringotipula) coloradensis

Doane, 1911

#### Tipula dorsimacula

Walker, 1848

##### Notes

BOLD:AAF8990

#### Tipula mallochi

Alexander

#### Xylomyidae

#### Macroceromys terminalis

Vasey 1977

#### Solva pallipes

Loew, 1863

#### Xylomyia americana

Weid

#### Xylomyia simillima

Steyskal 1947

#### Xylomyia tenthredinoides

Van der Wulp 1867

#### Xylophagidae

#### Xylophagus lugens

Loew, 1863

##### Notes

BOLD:AAJ9649

#### Xylophagus reflectens

Walker, 1848

##### Notes

BOLD:AAM7333|BOLD:AAP7637

#### Ephemeroptera

#### Baetidae

#### Baetis intercalaris

McDunnough, 1921

#### Callibaetis ferrugineus

Walsh, 1862

#### Cloeon dipterum

Linnaeus, 1761

#### Iswaeon anoka

Daggy, 1945

#### Caenidae

#### Caenis latipennis

Banks, 1907

#### Ephemeridae

#### Hexagenia limbata

Serville, 1829

#### Heptageniidae

#### Stenacron interpunctatum

Say, 1839

#### Hemiptera

#### Anthocoridae

#### Cardiastethus borealis

Kelton, 1977

#### Orius insidiosus

Say, 1832

#### Orius tristicolor

White, 1879

#### Aphididae

#### Acyrthosiphon malvae

Mosley, 1841

##### Notes

BOLD:AAF3206

#### Acyrthosiphon pisum

Harris, 1776

#### Aphis glycines

Matsumura, 1917

##### Notes

BOLD:AAB7938

#### Aphis middletonii

Thomas, 1879

##### Notes

BOLD:AAB6817

#### Aphis rubicola

Oestlund, 1887

##### Notes

BOLD:AAF7621

#### Eriosoma americanum

Riley, C.V., 1879

##### Notes

BOLD:AAD7955

#### Eucallipterus tiliae

Linnaeus, 1758

##### Notes

BOLD:AAD0131

#### Lipaphis pseudobrassicae

Davis, 1914

##### Notes

BOLD:AAD9153

#### Melaphis rhois

Fitch, 1866

##### Notes

BOLD:AAA2079

#### Rhopalosiphum nymphaeae

Linnaeus, 1761

#### Schizaphis scirpicola

Hille Ris Lambers, 1960

##### Notes

BOLD:AAD1238

#### Belostomatidae

#### Belostoma flumineum

Say, 1832

#### Berytidae

#### Neoneides muticus

Say, 1832

#### Cercopidae

#### Aphrophora alni

Fallén, 1805

#### Aphrophora cribrata

Walker, 1851

#### Clastoptera obtusa

Say, 1825

#### Lepyronia quadrangularis

Say, 1825

#### Neophilaenus lineatus

Linnaeus, 1758

#### Philaenus spumarius quadrimaculatus

Schrank, 1776

#### Philaenus spumarius

Linnaeus, 1758

#### Cicadellidae

#### Agallia quadripunctata

Oman 1933

##### Notes

BOLD:AAG2899

#### Agallia sp.

#### Agalliopsis ancistra

Oman 1970

#### Agalliopsis sp.

#### Anoscopus flavostriatus

Donovan, 1799

#### Anoscopus flavostrigata

Donovan, 1799

#### Aphrodes sp.

#### Arboridia sp.

#### Athysansus argentarius

Metcalf, 1955

#### Balclutha sp.

#### Ceratagallia sp.

#### Chlorotettix unicolor

Fitch 1851

#### Cicadula melanogaster

Provancher 1872

#### Colladonus clitellarius

Say, 1830

##### Notes

BOLD:ACV9851

#### Cosmotettix cf. bilineatus

#### Cuerna striata

Walker, 1851

#### Deltocephalus pulicaris

Fallén 1806

##### Notes

BOLD:AAY8918

#### Dicraneura sp.

#### Dikraneura mali

Provancher, 1890

##### Notes

BOLD:ABA5842

#### Dikrella cruentata

Gillette, 1898

##### Notes

BOLD:AAV0158

#### Diplocolenus abdominalis

Fabricius 1803

##### Notes

BOLD:AAG2897

#### Doratura stylata

Boheman, 1847

##### Notes

BOLD:AAG8821

#### Draeculacephala constricta

Davidson et DeLong, 1943

#### Draeculacephala sp.

#### Elymana caduca

DeLong 1936

#### Empoasca coccinea

Fitch, 1851

##### Notes

BOLD:ABA5764

#### Empoasca decipiens

Zachvatkin, 1935

##### Notes

BOLD:AAY6741

#### Empoasca fabae

Cardoso, 1974

##### Notes

BOLD:AAG2868|BOLD:AAG2873

#### Empoasca sp.

#### Erasmoneura nigra

Gillette, 1898

#### Eratoneura certa

Beamer, 1932

##### Notes

BOLD:ABA5787

#### Eratoneura flexibilis

Knull, 1949

##### Notes

BOLD:AAZ8495|BOLD:AAZ8496

#### Errastunus ocellaris

Fallén, 1806

##### Notes

BOLD:AAG8839

#### Erythridula dunni

Hepner, 1976

##### Notes

BOLD:ABA5786

#### Erythridula scytha

Auten & Johnson, 1936

##### Notes

BOLD:AAN8412

#### Erythridula tenuispica

Beamer, 1930

##### Notes

BOLD:ABA5830

#### Erythridula wysongi

Ross & DeLong, 1953

##### Notes

BOLD:AAN8287|BOLD:ABZ1306

#### Erythroneura aza

Robinson, 1924

##### Notes

BOLD:AAY6747

#### Erythroneura bakeri

Dmitriev & Dietrich, 2007

##### Notes

BOLD:AAV0161

#### Erythroneura elegans

McAtee, 1920

##### Notes

BOLD:ABA5798

#### Erythroneura ontari

Robinson, 1924

##### Notes

BOLD:ABA5810

#### Erythroneura ontari

Robinson, 1924

##### Notes

BOLD:ABA5810

### Checklist of species observed or collected at the *rare* Charitable Research Reserve in Cambridge, Ontario, Canada. The third of five checklists for Kingdom Animalia, this checklist contains members of Phylum Arthopoda, Class Insecta (Orders Hemiptera, Hymenoptera and Lepidoptera).

#### Insecta

#### Hemiptera

#### Cicadellidae

#### Erythroneura rubrella

McAtee, 1920

##### Notes

BOLD:AAV0164|BOLD:ACC8414

#### Erythroneura sp.

#### Erythroneura tricincta

Fitch, 1851

##### Notes

BOLD:AAY6738|BOLD:AAY6751

#### Erythroneura vitifex

Fitch, 1856

##### Notes

BOLD:AAY6742|BOLD:ACQ8506

#### Erythroneura vitis

Harris, 1831

#### Erythroneura vulnerata

Fitch, 1851

##### Notes

BOLD:AAO8361|BOLD:AAY6752|BOLD:ABA5772|BOLD:ABY9043|BOLD:ABY9046|BOLD:ACQ3943| BOLD:ACV2800|BOLD:ACV2885|BOLD:ACV2886

#### Eupteryx atropunctata

Goeze, 1778

##### Notes

BOLD:AAG2869

#### Eupteryx flavoscuta

Gillette, 1898

##### Notes

BOLD:ABA5805

#### Fieberiella florii

Stål, 1864

#### Forcipata acclina

DeLong & Caldwell, 1936

#### Forcipata loca

DeLong & Caldwell, 1936

##### Notes

BOLD:ACC8165

#### Graphocephala coccinea

Forster, 1771

#### Graphocephala sp.

#### Gyponana sp.

#### Hymetta balteata

McAtee, 1919

##### Notes

BOLD:AAV0157

#### Jikradia olitoria

Say, 1830

#### Latalus ocellaris

De Long & Sleesman, 1929

#### Macropsis basalis

Van Duzee, 1889

##### Notes

BOLD:ACC9200

#### Macrosteles quadrilineatus

Forbes, 1885

##### Notes

BOLD:AAA9422

#### Macrosteles sp.

#### Macrosteles variatus

Fallén, 1806

##### Notes

BOLD:AAV0236

#### Neokolla hieroglyphica

Say, 1830

##### Notes

BOLD:AAN8418

#### Oncopsis sobria

Walker, 1851

##### Notes

BOLD:ACI7197

#### Osbornellus limosus

DeLong, 1941

#### Penthimia americana

Fitch, 1851

#### Scaphoideus major

Osborn, 1900

##### Notes

BOLD:AAY6734

#### Scaphoideus sp.

#### Scaphytopius sp.

#### Sorhoanus pascuellus

Fallén, 1826

#### Typhlocyba arsinoe

McAtee, 1926

#### Typhlocyba niobe

McAtee, 1926

##### Notes

BOLD:ABA5877

#### Typhlocyba pomaria

McAtee, 1926

##### Notes

BOLD:AAF5980

#### Zonocyba hockingensis

Knull, 1945

##### Notes

BOLD:ACV8488

#### Cicadidae

#### Tibicen canicularis

Harris, 1841

#### Clastopteridae

#### Clastoptera proteus

Fitch, 1851

#### Corixidae

#### Palmacorixa buenoi

Abbott, 1913

#### Sehirus cinctus

Palisot, 1811

#### Trichocorixa borealis

Sailer, 1948

#### Trichocorixa sexcincta

Champion, 1901

#### Cydnidae

#### Sehirus cinctus cinctus

Palisot, 1811

#### Cymidae

#### Cymus angustatus

Stål, 1874

#### Delphacidae

#### Delphacodes sp.

#### Javasella pellucida

Fabricius, 1794

#### Kosswigianella lutulenta

Van Duzee, 1894

#### Liburniella ornata

Stål, 1862

#### Megamelus lunatus

Beamer, 1955

#### Pissonotus basalis

Van Duzee, 1897

#### Derbidae

#### Cedusa incisa

Metcalf, 1923

#### Dictyopharidae

#### Scolops sulcipes

Say, 1825

#### Flatidae

#### Metcalfa pruinosa

Say, 1830

#### Gelastocoridae

#### Gelastocoris oculatus

Fabricius, 1798

#### Gerridae

#### Aquarius remigis

Say, 1832

#### Issidae

#### Acanalonia bivittata

Say, 1825

#### Lygaeidae

#### Kleidocerys resedae geminatus

Say, 1832

#### Lygaeus kalmii

Stål, 1874

#### Nysius sp.

#### Membracidae

#### Atymna helena

Woodruff, 1915

##### Notes

BOLD:AAY9905

#### Campylenchia latipes

Say, 1824

#### Ceresa sp.

#### Cyrtolobus sp.

#### Enchenopa binotata

Say, 1824

#### Entylia carinata

Forster, 1771

#### Micrutalis calva

Say, 1831

#### Publilia concava

Say, 1824

#### Telamona decorata

Ball, 1903

#### Miridae

#### Adelphocoris lineolatus

Goeze, 1778

#### Amblytylus nasutus

Kirschbaum, 1856

#### Capsus ater

Linnaeus, 1758

#### Ceratocapsus sp.

#### Chlamydatus associatus

Uhler, 1872

##### Notes

BOLD:AAF3365

#### Chlamydatus sp.

#### Collaria meilleurii

Provancher, 1872

#### Criocoris saliens

Reuter, 1876

#### Deraeocoris sp.

#### Fulvius slateri

Wheeler, 1977

#### Halticus sp.

#### Heterocordylus malinus

Slingerland, 1909

#### Leptopterna dolabrata

Linnaeus, 1758

#### Litomiris sp.

#### Lopidea sp.

#### Lygidea sp.

#### Lygocoris pabulinus

Linnaeus, 1761

##### Notes

BOLD:AAB2218

#### Lygus sp.

#### Nabicula subcoleoptrata

Kirby, 1837

#### Nabis roseipennis

Reuter, 1872

#### Neolygus sp.

#### Orthocephalus sp.

#### Orthops scutellatus

Uhler, 1877

#### Pagasa fusca

Stein, 1857

#### Paraproba capitata

Van Duzee, 1912

#### Phoenicocoris strobicola

Knight, 1923

##### Notes

BOLD:AAH8507

#### Phytocoris sp.

#### Pithanus maerkelii

Herrich-Schaeffer, 1838

#### Plagiognathus sp.

#### Poecilocapsus lineatus

Fabricius, 1798

#### Prepops sp.

#### Slaterocoris sp.

#### Stenodema trispinosa

Reuter, 1904

#### Stenodema vicinum

Provancher, 1872

#### Stenotus binotatus

Fabricius, 1794

#### Taedia sp.

#### Nabidae

#### Hoplistoscelis sordidus

Reuter, 1872

#### Nabis rufusculus

Reuter, 1872

#### Notonectidae

#### Notonecta undulata

Say, 1832

#### Oxycarenidae

#### Crophius disconotus

Say, 1832

#### Pachygronthidae

#### Phlegyas abbreviatus

Uhler, 1876

#### Pentatomidae

#### Acrosternum hilare

Say, 1832

#### Banasa calva

Say, 1832

#### Banasa dimidiata

Say, 1832

#### Brochymena quadripustulata

Fabricius, 1775

#### Cosmopepla lintneriana

Kirkaldy, 1909

#### Euschistus tristigmus luridus

Dallas, 1851

#### Neottiglossa undata

Say, 1832

#### Parabrochymena arborea

Say,1825

#### Picromerus bidens

Linnaeus, 1758

#### Podisus maculiventris

Say, 1832

#### Phymatidae

#### Phymata americana

Melin, 1930

#### Piesma cinerum

Say, 1832

#### Psyllidae

#### Pachypsylla celtidismamma

Fletcher, 1882

#### Psyllopsis fraxinicola

Foerster, 1848

#### Reduviidae

#### Empicornis errabundus

Say, 1832

#### Sinea diadema

Fabricius, 1776

#### Zelus luridus

Stål, 1862

#### Zelus tetracanthus

Stål, 1862

#### Rhopalidae

#### Arhyssus sp.

#### Harmostes reflexulus

Say, 1832

#### Stictopleurus punctiventris

Dallas, 1852

#### Rhyparochromidae

#### Ligyrocoris diffusus

Uhler, 1871

#### Megalonotus sabulicola

Thomson, 1870

#### Sphragisticus nebulosus

Fallén, 1807

#### Saldidae

#### Saldula confluenta

Say, 1832

#### Saldula pallipes

Fabricius, 1794

#### Saldula saltatoria

Linnaeus, 1758

#### Thyreocoridae

#### Corimelaena pulicaria

Germar, 1839

#### Galgupha atra

Amyot and Serville, 1843

#### Tingidae

#### Corythucha marmorata

Uhler, 1878

#### Corythucha sp.

#### Dictyla echii

Schrank, 1782

#### Hymenoptera

#### Andrenidae

#### Andrena andrenoides

Cresson, 1878

#### Andrena barbilabris

Kirby, 1802

##### Notes

BOLD:AAB4998

#### Andrena canadensis

Dalla Torre, 1896

#### Andrena carlini

Cockerell, 1901

#### Andrena erythrogaster

Ashmead, 1890

#### Andrena hirticincta

Provancher, 1888

#### Andrena nasonii

Robertson, 1895

#### Andrena obscuripennis

Smith, 1853

#### Andrena rudbeckiae

Robertson, 1891

#### Andrena solidaginis

Robertson, 1893

#### Andrena sp.

#### Andrena thaspii

Graenicher, 1903

#### Andrena wilkella

Kirby, 1802

#### Perdita octomaculata

Say, 1824

#### Pseudopanurgus nebrascensis

Crawford, 1903

#### Anthophoridae

#### Anthophora furcata

Panzer, 1798

#### Ceratina calcarata

Robertson, 1900

#### Ceratina dupla

Say, 1837

#### Ceratina sp.

#### Melissodes desponsa

Smith, 1854

#### Apidae

#### Apis mellifera

Linnaeus, 1758

#### Bombus bimaculatus

Cresson, 1863

#### Bombus borealis

Kirby, 1837

#### Bombus griseocollis

DeGeer, 1773

#### Bombus impatiens

Cresson, 1863

##### Notes

BOLD:ABZ2516

#### Bombus perplexus

Cresson, 1863

#### Bombus rufocinctus

Cresson, 1863

#### Bombus vagans

Smith, 1854

#### Ceratina mikmaqi

Rehan and Sheffield, 2011

##### Notes

BOLD:AAA2368

#### Melissodes boltoniae

Robertson, 1905

#### Melissodes communis

Cresson, 1878

#### Melissodes druriella

Kirby, 1802

#### Melissodes illata

Lovell & Cockerell, 1906

#### Melissodes subillata

LaBerge, 1961

#### Melissodes trinodis

Robertson, 1901

#### Melissodes wheeleri

Cockerell, 1906

#### Nomada articulata

Smith, 1854

#### Nomada bella

Cresson, 1863

##### Notes

BOLD:ABZ2527

#### Nomada bethunei

Cockerell, 1903

#### Nomada cressonii

Robertson, 1893

#### Nomada luteoloides

Robertson, 1895

#### Nomada pygmaea

Cresson, 1863

##### Notes

BOLD:ABZ6834

#### Nomada subrutila

Lovell & Cockerell, 1905

##### Notes

BOLD:AAC5044

#### Triepeolus helianthi

Robertson, 1897

#### Triepeolus nigrihirtus

Mitchell, 1962

#### Triepeolus obliteratus

Graenicher, 1911

#### Triepeolus simplex

Robertson, 1903

#### Xylocopa virginiana

Linnaeus, 1771

#### Bethylidae

#### Parasierola sp.

#### Braconidae

#### Aphidius ervi

Haliday, 1834

##### Notes

BOLD:AAA4188

#### Ascogaster quadridentata

Wesmael, 1835

#### Asobara rufescens

Foerster, 1862

##### Notes

BOLD:AAU8583

#### Asobara sp.

#### Cotesia xylina

Say, 1836

##### Notes

BOLD:AAA9386

#### Diaeretiella rapae

McIntosh, 1855

##### Notes

BOLD:AAG1421

#### Diolcogaster facetosa

Weed, 1888

##### Notes

BOLD:ABA5941

#### Ephedrus lacertosus

Haliday, 1833

##### Notes

BOLD:ACW2698

#### Peristenus sp.

#### Pholetesor ornigis

Weed, 1887

##### Notes

BOLD:AAB0520

#### Pygostolus falcatus

Nees,1834

#### Spathius elegans

Matthews, 1970

#### Chalcididae

#### Conura albifrons

Walsh, 1861

##### Notes

BOLD:AAG8371

#### Chrysididae

#### Cleptes semiauratus

Linnaeus, 1761

#### Colletidae

#### Colletes eulophi

Robertson, 1891

#### Colletes hyalinus

Provancher, 1888

#### Colletes mandibularis

Smith, 1853

#### Colletes nudus

Robertson, 1898

#### Colletes simulans

Cresson, 1868

#### Hylaeus affinis

Smith, 1853

#### Hylaeus cf. affinis

#### Hylaeus ellipticus

Kirby, 1827

#### Hylaeus mesillae cressoni

Cockerell, 1907

#### Hylaeus modestus

Say, 1837

#### Hylaeus sp.

#### Crabronidae

#### Astata unicolor

Say, 1824

#### Cerceris arelate

Banks, 1912

#### Cerceris atramontensis

Banks, 1913

#### Cerceris clypeata

Dahlbom, 1844

#### Crossocerus annulipes

Lepeletier et Brullé, 1834

#### Crossocerus barbipes

Dahlbom, 1845

##### Notes

BOLD:AAG3190

#### Crossocerus elongatulus

van der Linden, 1829

#### Crossocerus tarsatus planipes

Fox, 1895

#### Diodontus minutus

Fabricius, 1793

#### Ectemnius cephalotes

Olivier, 1792

#### Ectemnius continuus

Fabricius, 1804

#### Ectemnius lapidarius

Panzer, 1804

#### Ectemnius maculosus

Gmelin, 1781

#### Ectemnius stirpicola

Packard, 1866

#### Gorytes atricornis

Packard,1867

#### Gorytes simillimus

Smith,1856

#### Hoplisoides nebulosus

Packard, 1867

#### Lestica confluenta

Say,1837

#### Lestica producticollis

Packard,1866

#### Lyroda subita

Say, 1837

#### Mimesa pauper

Packard, 1867

#### Oxybelus uniglumis

Linnaeus, 1758

#### Passaloecus cuspidatus

Smith, 1856

##### Notes

BOLD:AAG7762

#### Passaloecus singularis

Dahlbom, 1844

#### Pemphredon inornata

Say, 1824

#### Pemphredon lethifer

Shuckard, 1837

#### Philanthus bilunatus

Cresson, 1865

#### Pison koreense

Radoszkowski, 1887

#### Psen monticola

Packard, 1867

#### Rhopalum coarctatum

Scopoli, 1763

#### Saygorytes phaleratus

Say, 1837

#### Solierella peckhami

Ashmead, 1897

#### Stigmus americanus

Packard, 1867

##### Notes

or fraternus

#### Stigmus fraternus

Say, 1824

#### Tachysphex antennatus

Fox, 1894[", 1893"]

#### Tachysphex pompiliformis

Panzer, 1803

#### Trypoxylon attenuatum

Smith, 1851

#### Trypoxylon carinatum

Say, 1837

#### Trypoxylon clavicerum

Lepeletier & Serville, 1828

#### Trypoxylon frigidum

Smith, 1856

##### Notes

BOLD:AAG3193

#### Trypoxylon johnsoni

Fox, 1891

#### Trypoxylon lactitarse

Saussure, 1867

#### Trypoxylon politum

Drury, 1773

#### Diapriidae

#### Belyta validicornis

Thomson, 1858

##### Notes

BOLD:AAU8736

#### Encyrtidae

#### Copidosoma floridanum

Ashmead, 1900

##### Notes

BOLD:AAA7203

#### Eupelmidae

#### Eupelmus vesicularis

Retzius, 1783

#### Formicidae

#### Camponotus herculeanus

Linnaeus, 1758

##### Notes

BOLD:AAA2372

#### Camponotus nearcticus

Emery, 1893

##### Notes

BOLD:AAD4432

#### Camponotus pennsylvanicus

De Geer, 1773

##### Notes

BOLD:AAA9461

#### Camponotus sp.

#### Crematogaster cerasi

Fitch, 1855

#### Crematogaster sp.

#### Formica lasioides

Emery, 1893

##### Notes

BOLD:AAE0406

#### Formica sp.

#### Lasius alienus

Foerster, 1850

##### Notes

BOLD:AAA9049

#### Lasius claviger

Roger, 1862

#### Lasius nearcticus

Wheeler, 1906

##### Notes

BOLD:AAD1528

#### Lasius neoniger

Emery, 1893

##### Notes

BOLD:AAB9126

#### Lasius sp.

#### Leptothorax ambiguus

Emery, 1895

#### Myrmica sp.

#### Ponera pennsylvanica

Buckley, 1866

##### Notes

BOLD:AAF0443

#### Prenolepis imparis

Say, 1836

##### Notes

BOLD:AAC1302

#### Stenamma brevicorne

Mayr, 1886

##### Notes

BOLD:AAH7068

#### Stigmatomma pallipes

Haldeman, 1844

#### Tapinoma sessile

Say, 1836

##### Notes

BOLD:AAA3893|BOLD:AAA3900

#### Temnothorax ambiguus

Emery, 1895

##### Notes

BOLD:AAG0685

#### Halictidae

#### Agapostemon radiatus

Forster, 1771

#### Agapostemon sericeus

Förster, 1771

#### Agapostemon virescens

Fabricius, 1775

#### Augochlora aurata

Smith, 1853

#### Augochlora pura

Say, 1837

#### Augochlorella aurata

Smith, 1853

##### Notes

BOLD:AAD6445

#### Dialictus sp.

#### Halictus confusus

Smith, 1853

#### Halictus ligatus

Say, 1837

#### Halictus rubicundus

Christ, 1791

#### Lasioglossum anomalum

Robertson, 1892

#### Lasioglossum atwoodi

Gibbs, 2010

#### Lasioglossum birkmanni

Crawford, 1906

#### Lasioglossum bruneri

Crawford, 1902

#### Lasioglossum comagenense

(Knerer & Atwood, 1964)

#### Lasioglossum coriaceum

Smith, 1853

##### Notes

BOLD:AAB7007

#### Lasioglossum cressonii

Robertson, 1890

#### Lasioglossum dreisbachi

Mitchell, 1960

#### Lasioglossum ephialtum

Gibbs, 2010

#### Lasioglossum fattigi

Mitchell, 1960

#### Lasioglossum foxii

Robertson, 1895

#### Lasioglossum heterognathum

Mitchell, 1960

#### Lasioglossum hitchensi

Gibbs, 2012

#### Lasioglossum imitatum

Smith, 1853

#### Lasioglossum leucozonium

Schrank, 1781

#### Lasioglossum lineatulum

Crawford, 1906

#### Lasioglossum macoupinense

Robertson, 1895

#### Lasioglossum michiganense

Mitchell, 1960

#### Lasioglossum nigroviride

Graenicher, 1911

#### Lasioglossum paradmirandum

Knerer & Atwood, 1966

#### Lasioglossum pectinatum

Robertson, 1890

#### Lasioglossum pectorale

Smith, 1853

#### Lasioglossum perpunctatum

Ellis, 1913

#### Lasioglossum pilosum

Smith, 1853

#### Lasioglossum platyparium

Robertson, 1895

#### Lasioglossum pruinosum

Robertson, 1892

#### Lasioglossum sp. 1

#### Lasioglossum sp. 2

#### Lasioglossum subversans

Mitchell, 1960

#### Lasioglossum tegulare

Robertson, 1890

#### Lasioglossum tenax

Sandhouse, 1924

#### Lasioglossum timothyi

Gibbs, 2010

#### Lasioglossum versans

Lovell, 1905

##### Notes

BOLD:ABZ6180

#### Lasioglossum versatum

Robertson, 1902

#### Lasioglossum vierecki

Crawford, 1904

#### Lasioglossum weemsi

Mitchell, 1960

#### Lasioglossum zonulum

Smith, 1848

#### Lasioglossum zophops

Ellis, 1914

#### Sphecodes atlantis

Mitchell, 1956

#### Sphecodes clematidis

Robertson, 1897

#### Sphecodes confertus

Say, 1837

#### Sphecodes cressonii

Robertson, 1903

#### Sphecodes davisii

Robertson, 1897

#### Sphecodes dichrous

Smith, 1853

#### Sphecodes heraclei

Robertson, 1897

#### Sphecodes levis

Lovell & Cockerell, 1907

#### Sphecodes minor

Robertson, 1898

#### Sphecodes persimilis

Lovell & Cockerell, 1907

#### Sphecodes pycnanthemi

Robertson, 1897

#### Sphecodes ranunculi

Robertson, 1897

##### Notes

BOLD:AAC7655

#### Sphecodes sp.

#### Sphecodes stygius

Robertson, 1893

#### Sphecodes wheeleri

Mitchell, 1956

#### Ichneumonidae

#### Agrothereutes abbreviatus

Fabricius, 1794

##### Notes

BOLD:AAG7687

#### Agrypon flexorium

Thunberg, 1822

##### Notes

BOLD:AAH7052

#### Aritranis director

Thunberg, 1822

##### Notes

BOLD:AAG7768

#### Bathyplectes anurus

Thomson, 1887

##### Notes

BOLD:ABA6269

#### Bathythrix decipiens

Gravenhorst, 1829

##### Notes

BOLD:AAU8495

#### Campoletis flavicincta

Ashmead, 1890

##### Notes

BOLD:AAZ8146

#### Cryptus albitarsis

Cresson, 1864

##### Notes

BOLD:AAH1693

#### Cylloceria melancholica

Gravenhorst, 1820

#### Cymodusa distincta

Cresson, 1864

##### Notes

BOLD:ABZ4364

#### Diadegma pendulum

Walley, 1967

##### Notes

BOLD:AAZ9563

#### Dialipsis dissimilis

Dasch, 1992

##### Notes

BOLD:ABA6048

#### Diplazon laetatorius

Fabricius, 1781

##### Notes

BOLD:AAD4214

#### Dolichomitus irritator

Fabricius, 1775

##### Notes

BOLD:AAU8680

#### Dusona minor

Provancher, 1879

##### Notes

BOLD:AAH1652

#### Enytus apostata

Gravenhorst, 1829

##### Notes

BOLD:AAG5797

#### Exochus nigripalpis

Thomson, 1887

##### Notes

BOLD:AAG7713

#### Hyposoter inquinatus

Holmgren, 1860

##### Notes

BOLD:AAU8361

#### Ichneumon discoensis

Fox, 1892

##### Notes

BOLD:ACE9045

#### Ischnus inquisitorius

Muller, 1776

##### Notes

BOLD:AAG7737

#### Iseropus stercorator

Fabricius, 1793

##### Notes

BOLD:AAO2094

#### Lissonota coracina

Gmelin, 1790

#### Megacara hortulana

Gravenhorst, 1829

##### Notes

BOLD:AAU8687

#### Mesochorus suomiensis

Schwenke, 1999

##### Notes

BOLD:AAZ1979

#### Ophion bilineatus

Say, 1829

##### Notes

BOLD:AAG8323

#### Ophion clave

Viereck, 1905

##### Notes

BOLD:AAG7774

#### Ophion idoneus

Viereck, 1905

##### Notes

BOLD:AAN8172

#### Ophion sp. 5 MDS2014

##### Notes

BOLD:AAI3361

#### Oresbius taeniatus

Townes, 1962

#### Orthocentrus fulvipes

Gravenhorst, 1829

##### Notes

BOLD:AAM9125

#### Phobocampe bicingulata

Gravenhorst, 1829

##### Notes

BOLD:AAM7401

#### Pimpla aequalis

Provancher, 1880

##### Notes

BOLD:AAG7634

#### Pleolophus basizonus

Gravenhorst

#### Podoschistus vittifrons

Cresson, 1868

##### Notes

BOLD:AAL0380

#### Stenomacrus nemoralis

Holmgren, 1858

##### Notes

BOLD:AAM7494

#### Tranosema rostrale

Brischke, 1880

##### Notes

BOLD:AAD1926

#### Zaglyptus varipes

Gravenhorst, 1829

#### Leucospidae

#### Leucospis affinis

Say, 1824

#### Megachilidae

#### Anthidiellum notatum

Latreille, 1809

#### Anthidium manicatum

Linnaeus, 1758

#### Coelioxys rufitarsus

Smith, 1854

#### Heriades variolosa variolosa

Cresson, 1872

#### Hoplitis pilosifrons

Cresson, 1864

#### Hoplitis producta

Cresson, 1864

#### Hoplitis truncata

Cresson, 1878

#### Megachile brevis

Say, 1837

#### Megachile campanulae

Robertson, 1903

#### Megachile frigide

Smith, 1853

#### Megachile gemula

Cresson, 1878

#### Megachile inermis

Provancher, 1888

#### Megachile latimanus

Say, 1823

#### Megachile lippiae

Cockerell, 1900

#### Megachile mendica

Cresson, 1878

#### Megachile montivaga

Cresson, 1878

#### Megachile perihirta

Cockerell, 1898

#### Megachile pugnata

Say, 1837

#### Megachile relativa

Cresson, 1878

#### Megachile rotundata

Fabricius, 1793

#### Megachile texana

Cresson, 1878

#### Osmia coerulescens

Mitchell, T.B. 1962

#### Osmia conjuncta

Cresson, 1864

#### Osmia lignaria

Say, 1837

##### Notes

BOLD:AAE5495

#### Osmia proxima

Cresson, 1864

#### Osmia pumila

Cresson, 1864

#### Stelis lateralis

Cresson, 1864

#### Mutillidae

#### Myrmosa unicolor

Say, 1824

#### Pseudomethoca frigida

Smith 1855

#### Mymaridae

#### Anaphes listronoti

Huber, 1997

#### Gonatocerus morrilli

Howard, 1908

#### Ooctonus silvensis

Girault, 1916

##### Notes

BOLD:AAN7553

#### Platygastridae

#### Platygaster variabilis

Fouts, 1924

##### Notes

BOLD:ABW3242

#### Synopeas pennsylvanicum

Fouts, 1924

##### Notes

BOLD:ABA6127

#### Telenomus podisi

Ashmead, 1893

##### Notes

BOLD:AAG7891|BOLD:AAY9192|BOLD:ACU5364

#### Pompilidae

#### Agenioideus cinctellus

Spinola, 1808

#### Agenioideus humilis

Cresson, 1867

#### Anoplius imbellis

Banks, 1944

#### Anoplius nigerrimus

van der Vecht, 1973

#### Anoplius virginiensis

Cresson

#### Aporinellus wheeleri

Baquaert, 1919

#### Arachnospila michiganensis michiganensis

(Dreisbach)

#### Auplopus carbonarius

Scopoli, 1763

#### Auplopus mellipes variitarsatus

Dalla Torre, 1897

#### Auplopus nigrellus

Banks, 1912

#### Caliadurgus fasciatellus alienatus

Smith, 1855

#### Dipogon sayi sayi

Banks, 1941

#### Episyron biguttatus biguttatus

Fabricius, 1798

#### Priocnemis cornica

Say, 1836

#### Priocnemis germana

Cresson, 1867

#### Priocnemis minorata

Banks, 1912

#### Priocnemis notha notha

Cresson, 1867

#### Priocnemis scitula relicta

Banks, 1912

#### Pteromalidae

#### Mesopolobus bruchophagi

Gahan, 1917

##### Notes

BOLD:ACL4975

#### Sapygidae

#### Sapyga centrata

Say, 1836

##### Notes

BOLD:ACL7820

#### Sierolomorphidae

#### Sierolomorpha canadensis

Provancher, 1888

#### Siricidae

#### Tremex columba

Linnaeus

#### Sphecidae

#### Chalybion californicum

de Saussure, 1867

#### Isodontia auripes

Fernald, 1906

#### Isodontia mexicana

de Saussure, 1867

#### Sceliphron caementarium

Drury, 1773

#### Sphex ichneumoneus

Linnaeus, 1758

#### Tenthredinidae

#### Ametastegia aperta

Norton, 1861

##### Notes

BOLD:AAI4543

#### Ametastegia pallipes

Spinola

##### Notes

BOLD:AAE5602

#### Caulocampus acericaulis

MacGillivray, 1906

##### Notes

BOLD:ACJ9109

#### Dolerus asper

Zaddach, 1859

##### Notes

BOLD:AAG7773

#### Dolerus nitens

Zaddach 1859

##### Notes

BOLD:ACV5952

#### Empria maculata

Norton, 1861

##### Notes

BOLD:ACC8799

#### Empria nordica

Ross 1936

##### Notes

BOLD:ACI4328

#### Fenusa ulmi

Sundevall

##### Notes

BOLD:AAN7643

#### Halidamia affinis

Fallén 1807

##### Notes

BOLD:AAN7641

#### Macrophya flavolineata

Norton 1860

##### Notes

BOLD:ABU8852

#### Metallus lanceolatus

Thomson, 1870

##### Notes

BOLD:AAP1085

#### Monophadnus pallescens

Gmelin 1790

##### Notes

BOLD:ACK2140

#### Pachynematus extensicornis

Norton

##### Notes

BOLD:AAN8130

#### Periclista sp. tM8

##### Notes

BOLD:AAG3550

#### Priophorus compressicornis

Fabricius, 1804

##### Notes

BOLD:ACI7354

#### Pristiphora chlorea

Norton 1867

##### Notes

BOLD:ACG2990|BOLD:ACM9731

#### Taxonus epicera

Say 1836

##### Notes

BOLD:ACC7921

#### Taxonus pallicoxus

Provancher 1885

##### Notes

BOLD:AAG7788

#### Taxonus terminalis

Say 1824

##### Notes

BOLD:AAU8702

#### Tomostethus multicinctus

Rohwer

##### Notes

BOLD:ACV5036

#### Trichogrammatidae

#### Trichogramma platneri

Nagarkatti, 1975

##### Notes

BOLD:AAE0242

#### Vespidae

#### Ancistrocerus adiabatus adiabatus

Saussure, 1852

#### Ancistrocerus albophaleratus

de Saussure, 1855

#### Ancistrocerus antilope antilope

Panzer, 1798

#### Ancistrocerus campestris

Saussure, 1852

#### Ancistrocerus catskill

Saussure, 1853

#### Ancistrocerus unifasciatus unifasciatus

Saussure, 1852

#### Dolichovespula arenaria

Fabricius

##### Notes

BOLD:ACE9710

#### Eumenes crucifer

Provancher, 1888

#### Eumenes fraternus

Say

#### Euodynerus foraminatus foraminatus

de Saussure, 1853

#### Euodynerus leucomelas leucomelas

de Saussure, 1856

#### Monobia quadridens

Linnaeus, 1763

#### Parancistrocerus leionotus

Viereck, 1906

#### Parancistrocerus pedestris

de Saussure, 1855

##### Notes

subsp pedestris? No authorship found

#### Parancistrocerus pensylvanicus

de Saussure, 1856

#### Parancistrocerus pensylvanicus pensylvanicus

de Saussure, 1856

#### Polistes dominula

Christ, 1791

##### Notes

BOLD:AAB7105

#### Polistes fuscatus

Fabricius, 1793

#### Symmorphus canadensis

Saussure, 1855

#### Symmorphus cristatus

Saussure, 1856

#### Vespula flavopilosa

Jakobson, 1978

#### Vespula germanica

Fabricius, 1793

##### Notes

BOLD:AAG9055

#### Vespula maculifrons

Buysson

##### Notes

BOLD:AAD5593

#### Vespula vidua

de Saussure, 1854

##### Notes

BOLD:AAN8137

#### Vespula vulgaris

Linnaeus, 1758

#### Lepidoptera

#### Blastobasidae

#### Asaphocrita busckiella

Dietz, 1910

##### Notes

BOLD:AAA8938

#### Blastobasis glandulella

Riley, 1871

##### Notes

BOLD:AAB1096

#### Bucculatricidae

#### Bucculatrix ainsliella

Murtfeldt 1905

##### Notes

BOLD:AAB4931

#### Bucculatrix pomifoliella

Clemens, 1860

##### Notes

BOLD:AAD2085

#### Cosmopterigidae

#### Cosmopterix montisella

Chambers, 1875

##### Notes

BOLD:AAH4285

#### Teladoma helianthi

Busck, 1932

##### Notes

BOLD:AAE1519

#### Crambidae

#### Acentria ephemerella

Denis & Schiffermüller, 1775

#### Agriphila vulgivagella

Clemens, 1860

#### Anania funebris

Ström, 1768

##### Notes

BOLD:AAB4181

#### Desmia maculalis

Westwood, 1832

##### Notes

BOLD:ACE8375

#### Elophila gyralis

Hulst, 1886

#### Elophila icciusalis

Walker, 1859

#### Elophila tinealis

Munroe, 1972

### Checklist of species observed or collected at the *rare* Charitable Research Reserve in Cambridge, Ontario, Canada. The fourth of five checklists for Kingdom Animalia, this checklist contains members of Phylum Arthopoda, Class Insecta (Orders Lepidoptera to Trichoptera), and Class Malacostraca. From Phylum Chordata, it contains Classes Actinopterygii, Amphibia, and Aves (Orders Anseriformes to Passeriformes).

#### Animalia

#### Arthropoda

#### Insecta

#### Lepidoptera

#### Crambidae

#### Loxostege sticticalis

Linnaeus, 1761

#### Microcrambus elegans

Clemens, 1860

#### Nomophila nearctica

Munroe, 1973

#### Perispasta caeculalis

Zell., 1875

#### Petrophila bifascialis

Robinson, 1869

#### Sitochroa palealis

Denis & Schiffermüller, 1775

#### Udea rubigalis

Guenée, 1854

#### Urola nivalis

Drury, 1773

#### Depressariidae

#### Agonopterix arenella

Denis & Schiffermüller, 1775

##### Notes

BOLD:AAC6982

#### Agonopterix pulvipennella

Clemens, 1864

##### Notes

BOLD:AAA7550

#### Depressaria depressana

Fabricius, 1775

#### Machimia tentoriferella

Clemens, 1860

#### Drepanidae

#### Drepana arcuata

Walker

#### Elachistidae

#### Perittia herrichiella

Herrich-Schaffer, 1855

#### Epermeniidae

#### Epermenia albapunctella

Busck, 1908

##### Notes

BOLD:AAF0142

#### Erebidae

#### Apantesis phalerata

Harris, 1841

#### Cisseps fulvicollis

Hübner, 1818

#### Ctenucha virginica

Esper, 1794

#### Hypena madefactalis

Guenée, 1854

#### Hypena scabra

Fabricius, 1798

#### Hyphantria cunea

Drury, 1773

#### Lymantria dispar dispar

Linnaeus, 1758

#### Rivula propinqualis

Guenée, 1854

##### Notes

BOLD:AAA4282

#### Gelechiidae

#### Bryotropha hodgesi

Rutten & Karsholt, 2004

##### Notes

BOLD:AAH4276

#### Chionodes fondella

Busck, 1906

##### Notes

BOLD:ABA4737

#### Chrysoesthia sexguttella

Thunberg, 1794

##### Notes

BOLD:AAD8505

#### Dichomeris furia

Hodges, 1986

##### Notes

BOLD:AAI9560

#### Dichomeris inserrata

Walsingham, 1882

##### Notes

BOLD:AAH4488

#### Dichomeris leuconotella

Busck, 1904

#### Dichomeris ligulella

Hübner, 1818

##### Notes

BOLD:AAA8109

#### Dichomeris mercatrix

Hodges, 1986

#### Helcystogramma hystricella

Braun, 1921

##### Notes

BOLD:AAE7016

#### Metzneria lappella

Linnaeus, 1758

#### Monochroa fragariae

Busck, 1919

#### Scrobipalpa acuminatella

Sircom, 1850

##### Notes

BOLD:AAC1644

#### Scrobipalpula physaliella

Chambers, 1872

##### Notes

BOLD:ACB8750

#### Scrobipalpula sacculicola

Braun, 1925

##### Notes

BOLD:ABY8834

#### Sinoe chambersi

Lee, 2012

##### Notes

BOLD:ACF2217

#### Xenolechia ontariensis

Keifer, 1933

##### Notes

BOLD:AAC6357

#### Geometridae

#### Alsophila pometaria

Harris, 1841

##### Notes

BOLD:AAB0196

#### Besma quercivoraria

Guenée in Boisduval and Guenée, 1858

#### Biston betularia

Linnaeus, 1758

#### Campaea perlata

Guenée in Boisduval and Guenée, 1858

#### Coryphista meadii

Packard, 1874

#### Costaconvexa centrostrigaria

Wollaston, 1858

#### Ecliptopera silaceata

[Denis and Schiffermüller], 1775

#### Ennomos magnaria

Guenée in Boisduval and Guenée, 1858

#### Epirrhoe alternata

Müller, 1764

#### Euchlaena serrata

Drury, 1773

#### Idaea dimidiata

Hufnagel, 1767

#### Operophtera bruceata

Hulst, 1886

##### Notes

BOLD:AAA2999

#### Orthonama obstipata

Fabricius, 1794

#### Phigalia titea

Cramer, 1780

##### Notes

BOLD:AAA5234

#### Plagodis phlogosaria

Guenée in Boisduval and Guenée, 1858

##### Notes

BOLD:AAA3984

#### Pleuroprucha insulsaria

Guenée in Boisduval and Guenée, 1858

#### Scopula inductata

Guenée in Boisduval and Guenée, 1858

#### Speranza pustularia

Guenée in Boisduval and Guenée, 1858

##### Notes

BOLD:AAA4456

#### Synchlora frondaria

Guenée in Boisduval and Guenée, 1858

#### Trichodezia albovittata

Guenée in Boisduval and Guenée, 1858

##### Notes

BOLD:AAA6926

#### Xanthorhoe ferrugata

Clerck, 1759

##### Notes

BOLD:AAA3817

#### Xanthorhoe lacustrata

Guenée in Boisduval and Guenée, 1858

##### Notes

BOLD:AAA8660

#### Gracillariidae

#### Acrocercops astericola

Frey & Boll, 1873

##### Notes

BOLD:AAD3996

#### Caloptilia packardella

Chambers, 1872

##### Notes

BOLD:AAD2590

#### Cameraria saccharella

Braun, 1908

##### Notes

BOLD:AAH4493

#### Cremastobombycia solidaginis

Frey & Boll, 1876

#### Parornix betulae

Stainton, 1854

##### Notes

BOLD:AAE3418

#### Parornix crataegifoliella

Clemens, 1860

##### Notes

BOLD:AAF8198

#### Phyllocnistis ampelopsiella

Chambers, 1871

##### Notes

BOLD:AAI3015

#### Phyllocnistis vitegenella

Clemens, 1859

##### Notes

BOLD:AAI3014

#### Phyllonorycter clemensella

Chambers, 1871

##### Notes

BOLD:AAN8981

#### Phyllonorycter maestingella

Müller, 1764

##### Notes

BOLD:AAL6962

#### Phyllonorycter ostryaefoliella

Clemens, 1859

##### Notes

BOLD:AAD7999

#### Phyllonorycter propinquinella

Braun, 1908

##### Notes

BOLD:AAH4497

#### Phyllonorycter salicifoliella

Chambers, 1871

##### Notes

BOLD:AAD4915

#### Phyllonorycter trinotella

Braun, 1908

##### Notes

BOLD:AAG1128

#### Phyllonorycter tritaenianella

Chambers, 1871

##### Notes

BOLD:AAF6577

#### Hesperiidae

#### Anatrytone logan

W. H. Edwards, 1863

#### Ancyloxypha numitor

Fabricius, 1793

#### Atalopedes campestris

Boisduval, 1852

#### Carterocephalus palaemon

Pallas, 1771

#### Epargyreus clarus

Cramer, 1775

#### Erynnis baptisiae

W. Forbes, 1936

#### Erynnis juvenalis

Fabricius, 1793

##### Notes

BOLD:AAC6872

#### Erynnis lucilius

Scudder and Burgess, 1870

#### Euphyes conspicua

W. H. Edwards, 1863

#### Euphyes dion

W. H. Edwards, 1879

#### Euphyes vestris

Boisduval, 1852

#### Pholisora catullus

Fabricius, 1793

#### Poanes hobomok

T. Harris, 1862

#### Poanes massasoit

Scudder, 1863

#### Poanes viator

W. H. Edwards, 1865

#### Polites mystic

W. H. Edwards, 1863

#### Polites origenes

Fabricius, 1793

#### Polites peckius

W. Kirby, 1837

#### Polites themistocles

Latreille, 1824

#### Pompeius verna

W. H. Edwards, 1862

#### Thymelicus lineola

Ochsenheimer, 1808

#### Wallengrenia egeremet

Scudder, 1863

#### Lasiocampidae

#### Malacosoma disstria

Hubner

##### Notes

BOLD:AAA4130

#### Tolype velleda

Stoll, 1791

#### Lycaenidae

#### Celastrina ladon

Cramer, 1780

#### Celastrina neglecta

W. H. Edwards, 1862

#### Cupido comyntas

Godart, 1824

#### Feniseca tarquinius

Fabricius, 1793

#### Lycaena hyllus

Cramer, 1775

#### Satyirum titus

Fabricius, 1793

#### Satyrium acadicum

W. H. Edwards, 1862

#### Satyrium calanus

Hübner, 1809

#### Satyrium caryaevorus

McDunnough, 1942

#### Satyrium liparops

LeConte, 1833

#### Lymantriidae

#### Dasychira basiflava

Packard, 1864

#### Orgyia definita

Packard, 1864

#### Orgyia leucostigma

J. E. Smith, 1797

#### Momphidae

#### Mompha terminella

Humphreys & Westwood, 1845

##### Notes

BOLD:AAX4784

#### Nepticulidae

#### Ectoedemia argyropeza

Zeller, 1839

##### Notes

BOLD:AAC1036

#### Stigmella microtheriella

Stainton, 1854

##### Notes

BOLD:AAI0007

#### Stigmella rhamnicola

Braun, 1916

##### Notes

BOLD:AAU7678

#### Noctuidae

#### Achatia distincta

Hübner, 1813

##### Notes

BOLD:AAB7392

#### Agrotis ipsilon

Hufnagel, 1766

#### Agrotis venerabilis

Walker, 1857

#### Allagrapha aerea

Hübner, 1803

#### Amphipoea americana

Speyer, 1875

#### Amphipoea interoceanica

Smith, 1899

#### Amphipyra pyramidoides

Guenée, 1852

##### Notes

BOLD:AAA8525

#### Anathix ralla

Grote and Robinson, 1868

##### Notes

BOLD:AAC9569

#### Apamea devastator

Brace, 1819

#### Autographa precationis

Guenée, 1852

#### Caenurgina crassiuscula

Haworth, 1809

#### Catocala cerogama

Guenée, 1852

##### Notes

BOLD:AAB3383

#### Catocala grynea

Cramer, 1779

#### Cerastis tenebrifera

Walker, 1865

##### Notes

BOLD:AAC1487

#### Chrysodeixis includens

Walker, 1858

#### Crocigrapha normani

Grote, 1874

##### Notes

BOLD:AAA6924

#### Cucullia asteroides

Guenée, 1852

#### Cucullia convexipennis

Grote and Robinson, 1868

#### Euplexia benesimilis

McDunnough, 1922

##### Notes

BOLD:AAA4097

#### Eupsilia devia

Grote, 1875

##### Notes

BOLD:AAD9847

#### Feltia jaculifera

Guenée, 1852

#### Hyppa xylinoides

Guenée, 1852

##### Notes

BOLD:ABY9574

#### Idia aemula

Hübner, 1814

#### Lacinipolia meditata

Grote, 1873

#### Lacinipolia renigera

Stephens, 1829

#### Leucania commoides

Guenée, 1852

#### Leucania multilinea

Walker, 1856

#### Leucania phragmitidicola

Guenée, 1852

#### Leucania pseudargyria

Guenée, 1852

#### Loscopia velata

Walker, 1865

#### Macrochilo absorptalis

Walker, 1859

#### Melanchra adjuncta

Guenée, 1852

##### Notes

BOLD:ACF4823

#### Meropleon diversicolor

Morrison, 1874

#### Morrisonia confusa

Hübner, 1831

##### Notes

BOLD:AAA6652

#### Mythimna unipuncta

Haworth, 1809

#### Noctua pronuba

Linnaeus, 1758

#### Ochropleura implecta

Lafontaine, 1998

#### Oligia modica

Guenée, 1852

#### Orthosia hibisci

Guenée, 1852

##### Notes

BOLD:AAA4128

#### Orthosia rubescens

Walker, 1865

##### Notes

BOLD:AAC0946

#### Palthis angulalis

Hübner, 1796

##### Notes

BOLD:AAA3933

#### Peridroma saucia

Hübner, 1808

#### Phalaenostola metonalis

Walker, 1859

#### Protodeltote albidula

Guenée, 1852

#### Pseudohermonassa bicarnea

Guenée, 1852

#### Renia adspergillus

Bosc, 1800

#### Striacosta albicosta

Smith, 1888

#### Sunira bicolorago

Guenée, 1852

##### Notes

BOLD:AAA4426

#### Xestia smithii

Snellen, 1896

##### Notes

BOLD:AAA2590

#### Nolidae

#### Nola ovilla

Grote, 1875

##### Notes

BOLD:AAD1810

#### Notodontidae

#### Schizura unicornis

J. E. Smith, 1797

#### Symmerista leucitys

Franclemont, 1946

#### Nymphalidae

#### Aglais milberti

Godart, 1819

#### Asterocampa clyton

Boisduval and Le Conte, 1835

#### Boloria Bellona

Fabricius, 1775

#### Boloria selene

[Schiffermüller], 1775

#### Cercyonis pegala

Fabricius, 1775

#### Chlosyne nycteis

E. Doubleday, 1847

#### Coenonympha tullia

Müller, 1764

#### Danaus plexippus

Linnaeus, 1758

#### Euphydryas phaeton

Drury, 1773

#### Euptoieta claudia

Cramer, 1775

#### Junonia coenia

Hübner, 1822

#### Lethe anthedon

A. Clark, 1936

#### Lethe appalachia

R. Chermock, 1947

#### Lethe eurydice

Linnaeus, 1763

#### Libytheana carinenta

Cramer, 1777

#### Limenitis archippus

Cramer, 1775

#### Limenitis arthemis

Drury, 1773

#### Megisto cymela

Cramer, 1777

#### Nymphalis antiopa

Linnaeus, 1758

#### Nymphalis l-album

[Schiffermüller], 1775

#### Phyciodes cocyta

Cramer, 1777

#### Phyciodes tharos

Drury, 1773

#### Polygonia comma

T. Harris, 1842

#### Polygonia interrogationis

Fabricius, 1798

#### Polygonia progne

Cramer, 1775

#### Speyeria Cybele

Fabricius, 1775

#### Vanessa atalanta

Linnaeus, 1758

#### Vanessa cardui

Linnaeus, 1758

#### Vanessa virginiensis

Drury, 1773

#### Papilionidae

#### Papilio canadensis

Rothschild and Jordan, 1906

#### Papilio cresphontes

Cramer, 1777

#### Papilio glaucus

Linnaeus, 1758

#### Papilio polyxenes

Fabricius, 1775

#### Pieridae

#### Colias eurytheme

Boisduval, 1852

#### Colias philodice

Godart, 1819

#### Pieris oleracea

T. Harris, 1829

#### Pieris rapae

Linnaeus, 1758

#### Pyrisitia lisa

Boisduval and Le Conte, 1830

#### Plutellidae

#### Plutella porrectella

Linnaeus, 1758

##### Notes

BOLD:ACG9804

#### Plutella xylostella

Linnaeus, 1758

##### Notes

BOLD:AAA1513

#### Psychidae

#### Psyche casta

Pallas, 1767

##### Notes

BOLD:ACL8669

#### Pterophoridae

#### Gillmeria pallidactyla

Haworth, 1811

#### Hellinsia homodactylus

Walker, 1864

#### Hellinsia pectodactylus

Staudinger, 1859

#### Sphingidae

#### Deidamia inscriptum

Harris, 1839

##### Notes

BOLD:AAB0001

#### Tischeriidae

#### Coptotriche badiiella

Chambers, 1875

##### Notes

BOLD:ACU4456

#### Tortricidae

#### Acleris chalybeana

Fernald, 1882

##### Notes

BOLD:AAA7667

#### Acleris cornana

McDunnough, 1933

#### Ancylis muricana

Walsingham, 1879

##### Notes

BOLD:AAU7760

#### Argyrotaenia mariana

Fernald, 1882

##### Notes

BOLD:AAA4119

#### Choristoneura rosaceana

Harris, 1841

##### Notes

BOLD:AAA1517

#### Cochylis hoffmanana

Kearfott, 1907

##### Notes

BOLD:AAB3571

#### Cochylis temerana

Busck, 1907

##### Notes

BOLD:AAB7534

#### Endothenia hebesana

Walker, 1863

#### Epinotia medioviridana

Kearfott, 1908

#### Eucosma similana

Clemens, 1860

#### Grapholita prunivora

Walsh, 1868

##### Notes

BOLD:AAG0330

#### Olethreutes atrodentana

Fernald, 1882

#### Olethreutes fasciatana

Clemens, 1860

#### Olethreutes permundana

Clemens, 1860

#### Pandemis lamprosana

Robinson, 1869

#### Phaneta ochrocephala

Walsingham, 1895

#### Phaneta parmatana

Clemens, 1860

#### Phaneta tomonana

Kearfott, 1907

#### Platynota idaeusalis

Walker, 1859

##### Notes

BOLD:ABY7901

#### Pristerognatha fuligana

Denis & Schiffermüller, 1775

##### Notes

BOLD:AAC7661

#### Proteoteras aesculana

Riley, 1881

##### Notes

BOLD:AAA6740

#### Mantodea

#### Mantidae

#### Mantis religiosa religiosa

Linnaeus, 1758

#### Mecoptera

#### Bittacidae

#### Bittacus strigosus

Hagen, 1861

#### Panorpidae

#### Panorpa galerita

Byers, 1962

#### Panorpa latipennis

Hine, 1901

#### Panorpa subfurcata

Westwood, 1846

#### Neuroptera

#### Chrysopidae

#### Chrysopa oculata

Say, 1839

#### Hemerobiidae

#### Hemerobius humulinus

Linnaeus, 1758

##### Notes

BOLD:AAG0892|BOLD:AAN7492

#### Hemerobius stigma

Stephens, 1836

##### Notes

BOLD:AAG0891

#### Micromus posticus

Walker, 1853

##### Notes

BOLD:AAG0906

#### Odonata

#### Aeshnidae

#### Aeshna umbrosa

Walker, 1908

#### Anax junius

Drury, 1773

#### Calopterygidae

#### Hetaerina americana

Fabricius, 1798

#### Coenagrionidae

#### Argia moesta

Hagen, 1861

#### Enallagma antennatum

Say, 1839

#### Enallagma civile

Hagen, 1861

#### Enallagma ebrium

Hagen, 1861

#### Enallagma exsulans

Hagen, 1861

#### Enallagma geminatum

Kellicott, 1895

#### Enallagma signatum

Hagen, 1861

#### Ischnura kellicotti

Williamson, 1898

#### Ischnura posita

Hagen, 1861

#### Ishnura verticalis

Say, 1839

#### Nehalennia irene

Hagen, 1861

#### Lestidae

#### Lestes disjunctus

Selys, 1862

#### Lestes rectangularis

Say, 1839

#### Libellulidae

#### Erythemis simplicicollis

Say, 1839

#### Libellula luctuosa

Burmeister, 1839

#### Libellula pulchella

Drury, 1773

#### Libellula quadrimaculata

Linnaeus, 1758

#### Pachydiplax longipennis

Burmeister, 1839

#### Plathemis lydia

Drury, 1773

#### Sympetrum internum

Montgomery, 1943

#### Sympetrum obtrusum

Hagen, 1867

#### Sympetrum semicinctum

Say, 1839

#### Tramea lacerat

Hagen, 1861

#### Orthoptera

#### Acrididae

#### Cholealtis conspera

Harris, 1841

#### Chorthippus curtipennis

Harris, 1835

#### Chortophaga viridifasciata

De Geer, 1773

#### Dissosteira carolina

Linnaeus, 1758

#### Dissosteria carolina

Linnaeus, 1758

#### Melanoplus bivatattus

Say, 1825

#### Melanoplus sanguinipes sanguinipes

Fabricius, 1798

#### Melanoplus sp.

#### Gryllidae

#### Allonemobius fasciatus

De Geer, 1773

#### Gryllus veletis

Alexander and Bigelow, 1960

#### Oecanthus nigricornis

F. Walker, 1869

#### Oecanthus quadripunctatus

Beutenmuller, 1894

#### Tetrigidae

#### Tetrix arenonsum angusta

Hancock, 1896

#### Tetrix subulata

Linnaeus, 1761

#### Tetrigoniidae

#### Conocephalus brevipennis

Scudder, 1863

#### Scudderia sp.

#### Phasmatodea

#### Heteronemiidae

#### Diapheromera femorata

Say, 1824

#### Plecoptera

#### Chloroperlidae

#### Sweltsa onkos

Ricker, 1935

#### Psocoptera

#### Amphipsocidae

#### Polypsocus corruptus

Hagen, 1861

#### Caeciliidae

#### Caecillius sp.

#### Caeciliusidae

#### Valenzuela flavidus

Stephens, 1836

##### Notes

BOLD:AAH3228|BOLD:AAN8447

#### Ectopsocidae

#### Ectopsocus meridionalis

Ribaga, 1904

#### Mesopsocidae

#### Mesopsocus unipunctatus

Müller, 1764

#### Peripsocidae

#### Peripsocus subfasciatus

Rambur, 1842

#### Psocidae

#### Blaste opposita

Banks, 1907

#### Metylophorus novaescotiae

Walker, 1853

#### Stenopsocidae

#### Graphopsocus cruciatus

Linnaeus, 1768

##### Notes

BOLD:ACA2933|BOLD:ACB0984

#### Thysanoptera

#### Aeolothripidae

#### Aeolothrips ericae

Bagnall, 1920

##### Notes

BOLD:ABA2981

#### Phlaeothripidae

#### Haplothrips verbasci

Osborn, 1897

##### Notes

BOLD:AAI6861

#### Thripidae

#### Chirothrips manicatus

Haliday, 1836

#### Odontothrips biuncus

John, 1921

#### Taeniothrips inconsequens

Uzel, 1895

##### Notes

BOLD:ACC0651

#### Trichoptera

#### Helicopsychidae

#### Helicopsyche borealis

Hagen, 1861

#### Hydropsychidae

#### Ceratopsyche morosa

Hagen, 1861

##### Notes

BOLD:AAA3679

#### Cheumatopsyche campyla

Ross, 1938

##### Notes

BOLD:AAA3892|BOLD:ACE5263

#### Hydropsyche phalerata

Hagen, 1861

##### Notes

BOLD:AAC3243

#### Hydroptilidae

#### Agraylea multipunctata

Curtis, 1834

#### Hydroptila armata

Ross, 1938

#### Hydroptila perdita

Morton, 1905

##### Notes

BOLD:AAE5187

#### Hydroptila spatulata

Morton, 1905

##### Notes

BOLD:AAD0137

#### Orthotrichia cristata

Morton, 1905

#### Leptoceridae

#### Oecetis avara

Banks, 1895

#### Oecetis cinerascens

Hagen, 1861

#### Oecetis inconspicua

Walker, 1852

##### Notes

BOLD:AAA1532

#### Oecetis nocturna

Ross, 1966

#### Limnephilidae

#### Ironoquia punctatissima

Walker, 1852

#### Pycnopsyche antica

Walker, 1852

#### Phryganeidae

#### Banksiola crotchi

Banks, 1944

#### Polycentropodidae

#### Plectrocnemia cinerea

Hagen, 1861

##### Notes

BOLD:AAA3441|BOLD:ACL7631

#### Malacostraca

#### Amphipoda

#### Hyalellidae

#### Hyalella azteca

Saussure, 1858

#### Decapoda

#### Cambaridae

#### Orconectes propinquus

Girard, 1852

#### Isopoda

#### Trachelipodidae

#### Trachelipus rathkii

Brandt, 1833

##### Notes

BOLD:AAH4102

#### Trichoniscidae

#### Hyloniscus riparius

Koch, 1838

##### Notes

BOLD:AAV6495

#### Trichoniscus pusillus

Brandt, 1833

##### Notes

BOLD:AAN7523

#### Chordata

#### Actinopterygii

#### Cypriniformes

#### Catostomidae

#### Catostomus catostomus

Forster, 1773

#### Catostomus commersonii

Lacepède, 1803

#### Hypentelium nigricans

Lesueur, 1817

#### Moxostoma erythrurum

Rafinesque, 1818

#### Moxostoma valenciennesi

Jordan, 1885

#### Cyprinidae

#### Cyprinella spiloptera

Cope, 1867

#### Cyprinus Carpio

Linnaeus, 1758

#### Luxilus chrysocephalus

Rafinesque, 1820

#### Luxilus cornutus

Mitchill, 1817

#### Nocomis biguttatus

Kirtland, 1840

#### Nocomis micropogon

Cope, 1865

#### Notropis heterodon

Cope, 1865

#### Notropis photogenis

Cope, 1865

#### Pimephales notatus

Rafinesque, 1820

#### Rhinichthys atratulus

Hermann, 1804

#### Semotilus atromaculatus

Mitchill, 1818

#### Esociformes

#### Esocidae

#### Esox lucius

Linnaeus, 1758

#### Gasterosteiformes

#### Gasterosteidae

#### Culaea inconstans

Kirtland, 1840

#### Perciformes

#### Centrarchidae

#### Ambloplites rupestris

Rafinesque, 1817

#### Lepomis gibbosus

Linnaeus, 1758

#### Micropterus dolomieu

Lacepède, 1802

#### Micropterus salmoides

Lacepède, 1802

#### Pomoxis nigromaculatus

Lesueur in Cuvier and Valenciennes, 1829

#### Percidae

#### Etheostoma blennioides

Rafinesque, 1819

#### Etheostoma exile

Girard, 1859

#### Etheostoma nigrum

Rafinesque, 1820

#### Perca flavescens

Mitchill, 1814

#### Percina maculata

Girard, 1859

#### Salmoniformes

#### Salmonidae

#### Salvelinus fontinalis

Mitchill, 1814

#### Siluriformes

#### Ictaluridae

#### Ameiurus nebulosus

Lesueur, 1819

#### Noturus flavus

Rafinesque, 1818

#### Amphibia

#### Anura

#### Bufonidae

#### Anaxyrus americanus

Holbrook, 1836

#### Hylidae

#### Hyla versicolor

LeConte, 1825

#### Pseudacris crucifer

Wied-Neuwied, 1838

#### Pseudacris triseriata

Wied-Neuwied, 1838

#### Ranidae

#### Lithobates catesbeiana

Shaw, 1802

#### Lithobates clamitans

Latreille in Sonnini de Manoncourt and Latreille, 1801

#### Lithobates pipiens

Schreber, 1782

#### Lithobates sylvatica

LeConte, 1825

#### Caudata

#### Ambystomidae

#### Ambystoma jeffersonianum x lateralevar.hybrid species: jefferson and blue-spotted

Green, 1827 and Hallowell, 1856

#### Ambystoma laterale

Hallowell, 1856

#### Ambystoma maculatum

Shaw, 1802

#### Plethodontidae

#### Hemidactylium scutatum

Temminck and Schlegel, 1838

#### Plethodon cinereus

Green, 1818

#### Aves

#### Anseriformes

#### Anatidae

#### Aix sponsa

Linnaeus, 1758

#### Anas acuta

Linnaeus, 1758

#### Anas americana

Gmelin, 1789

#### Anas clypeata

Linnaeus, 1758

#### Anas crecca

Linnaeus, 1758

#### Anas discors

Linnaeus, 1766

#### Anas platyrhynchos

Linnaeus, 1758

#### Anas rubripes

Brewster, 1902

#### Anas strepera

Linnaeus, 1758

#### Aythya affinis

Eyton, 1838

#### Aythya americana

Eyton, 1838

#### Aythya collaris

Donovan, 1809

#### Aythya marila

Linnaeus, 1761

#### Aythya valisineria

A. Wilson, 1814

#### Branta bernicla

Linnaeus, 1758

#### Branta canadensis

Linnaeus, 1758

#### Branta hutchinsii

Richardson, 1832

#### Bucephala albeola

Linnaeus, 1758

#### Bucephala clangula

Linnaeus, 1758

#### Chen caerulescens

Linnaeus, 1758

#### Clangula hyemalis

Linnaeus, 1758

#### Cygnus buccinator

Richardson, 1831

#### Cygnus columbianus

Ord, 1815

#### Cygnus olor

Gmelin, 1789

#### Lophodytes cucullatus

Linnaeus, 1758

#### Melanerpes erythrocephalus

Linnaeus, 1758

#### Mergus merganser

Linnaeus, 1758

#### Mergus serrator

Linnaeus, 1758

#### Oxyura jamaicensis

Gmelin, 1789

#### Apodiformes

#### Apodidae

#### Chaetura pelagica

Linnaeus, 1758

#### Trochilidae

#### Archilochus colubris

Linnaeus, 1758

#### Caprimulgiformes

#### Caprimulgidae

#### Caprimulgus vociferus

A. Wilson, 1812

#### Chordeiles minor

J. R. Forster, 1771

#### Charadriiformes

#### Charadriidae

#### Charadrius semipalmatus

Bonaparte, 1825

#### Charadrius vociferus

Linnaeus, 1758

#### Laridae

#### Chroicocephalus philadelphia

Ord, 1815

#### Larus delawarensis

Ord, 1815

#### Larus fuscus

Linnaeus, 1758

#### Larus glaucoides

B. Meyer, 1822

#### Larus hyperboreus

Gunnerus, 1767

#### Larus marinus

Linnaeus, 1758

#### Larus smithsonianus

Coues, 1862

#### Larus thayeri

W. S. Brooks, 1915

#### Mniotilta varia

Linnaeus, 1766

#### Sterna hirundo

Linnaeus, 1758

#### Scolopacidae

#### Actitis macularius

Linnaeus, 1766

#### Arenaria interpres

Linnaeus, 1758

#### Bartramia longicauda

Bechstein, 1812

#### Calidris alpina

Linnaeus, 1758

#### Calidris bairdii

Coues, 1861

#### Calidris melanotos

Vieillot, 1819

#### Calidris minutilla

Vieillot, 1819

#### Calidris pusilla

Linnaeus, 1766

#### Gallinago delicata

Ord, 1825

#### Scolopax minor

Gmelin, 1789

#### Tringa flavipes

Gmelin, 1789

#### Tringa melanoleuca

Gmelin, 1789

#### Tringa solitaria

A. Wilson, 1813

#### Sternidae

#### Hydroprogne caspia

Pallas, 1770

#### Ciconiiformes

#### Ardeidae

#### Ardea alba

Linnaeus, 1758

#### Ardea herodias

Linnaeus, 1758

#### Botaurus lentiginosus

Rackett, 1813

#### Butorides virescens

Linnaeus, 1758

#### Chlidonias niger

Linnaeus, 1758

#### Egretta caerulea

Linnaeus, 1758

#### Columbiformes

#### Columbidae

#### Columba livia

Gmelin, 1789

#### Zenaida macroura

(Linnaeus, 1758)

#### Coraciiformes

#### Alcedinidae

#### Megaceryle alcyon

Linnaeus, 1758

#### Cuculiformes

#### Cuculidae

#### Coccyzus americanus

Linnaeus, 1758

#### Coccyzus erythropthalmus

A. Wilson, 1811

#### Falconiformes

#### Accipitridae

#### Accipiter cooperii

Bonaparte, 1828

#### Accipiter gentilis

Linnaeus, 1758

#### Accipiter striatus

Vieillot, 1808

#### Agelaius phoeniceus

Linnaeus, 1766

#### Aquila chrysaetos

Linnaeus, 1758

#### Buteo jamaicensis

Gmelin, 1788

#### Buteo lagopus

Pontoppidan, 1763

#### Buteo lineatus

Gmelin, 1788

#### Buteo platypterus

Vieillot, 1823

#### Circus cyaneus

Linnaeus, 1766

#### Haliaeetus leucocephalus

Linnaeus, 1766

#### Cathartidae

#### Cathartes aura

Linnaeus, 1758

#### Falconidae

#### Falco columbarius

Linnaeus, 1758

#### Falco peregrinus

Tunstall, 1771

#### Falco sparverius

Linnaeus, 1758

#### Pandionidae

#### Pandion haliaetus

Linnaeus, 1758

#### Galliformes

#### Odontophoridae

#### Colinus virginianus

Linnaeus, 1758

#### Phasianidae

#### Bonasa umbellus

Linnaeus, 1766

#### Meleagris gallopavo

Linnaeus, 1758

#### Phasianus colchicus

Linnaeus, 1758

#### Gaviiformes

#### Gaviidae

#### Gavia immer

Brunnich, 1764

#### Gruiformes

#### Gruidae

#### Grus canadensis

Linnaeus, 1758

#### Rallidae

#### Fulica americana

Gmelin, 1789

#### Porzana carolina

Linnaeus, 1758

#### Rallus limicola

Vieillot, 1819

#### Passeriformes

#### Alaudidae

#### Eremophilia alpestris

(Linnaeus, 1758)

#### Bombycillidae

#### Bombycilla cedrorum

Vieillot, 1808

#### Calcariidae

#### Calcarius lapponicus

Linnaeus, 1758

#### Cardinalidae

#### Cardinalis cardinalis

Linnaeus, 1758

#### Passerina cyanea

Linnaeus, 1766

#### Pheucticus ludovicianus

Linnaeus, 1766

#### Plectrophenax nivalis

Linnaeus, 1758

#### Certhiidae

#### Certhia americana

Bonaparte, 1838

#### Corvidae

#### Corvus brachyrhynchos

C. L. Brehm, 1822

#### Corvus corax

Linnaeus, 1758

#### Cyanocitta cristata

Linnaeus, 1758

#### Emberizidae

#### Ammodramus leconteii

Audubon, 1844

#### Ammodramus nelsoni

Allen, 1875

#### Ammodramus savannarum

Gmelin, 1789

#### Junco hyemalis

Linnaeus, 1758

#### Melospiza georgiana

Latham, 1790

#### Melospiza lincolnii

Audubon, 1834

#### Melospiza melodia

A. Wilson, 1810

#### Passerculus sandwichensis

Gmelin, 1789

#### Passerella iliaca

Merrem, 1786

#### Pipilo erythrophthalmus

Linnaeus, 1758

#### Pipilo maculatus

Swainson, 1827

#### Pooecetes gramineus

Gmelin, 1789

#### Spizella arborea

A. Wilson, 1810

#### Spizella pallida

Swainson, 1832

#### Spizella passerina

Bechstein, 1798

#### Spizella pusilla

A. Wilson, 1810

#### Zonotrichia albicollis

Gmelin, 1789

#### Zonotrichia leucophrys

J. R. Forster, 1772

#### Fringillidae

#### Carduelis flammea

Linnaeus, 1758

#### Carduelis pinus

A. Wilson, 1810

#### Carduelis tristis

Linnaeus, 1758

#### Carpodacus mexicanus

Statius Muller, 1776

#### Carpodacus purpureus

Gmelin, 1789

#### Coccothraustes vespertinus

W. Cooper, 1825

#### Loxia curvirostra

Linnaeus, 1758

### Checklist of species observed or collected at the *rare* Charitable Research Reserve in Cambridge, Ontario, Canada. The fifth of five checklists for Kingdom Animalia, this checklist contains records from Phylum Chordata, Class Aves (Orders Passeriformes to Strigiformes), Class Mammalia, and Class Reptilia, as well as Phylum Gastropoda.

#### Chordata

#### Aves

#### Passeriformes

#### Fringillidae

#### Loxia leucoptera

Gmelin 1789

#### Hirundinidae

#### Hirundo rustica

Linnaeus, 1758

#### Petrochelidon pyrrhonota

(Vieillot, 1817)

#### Riparia riparia

Linnaeus, 1758

#### Stelgidopteryx serripennis

Audubon, 1838

#### Tachycineta bicolor

Vieillot, 1808

#### Icteridae

#### Dolichonyx oryzivorus

Linnaeus, 1758

#### Euphagus carolinus

Statius Muller, 1776

#### Euphagus cyanocephalus

Wagler, 1829

#### Icterus galbula

Linnaeus, 1758

#### Icterus spurius

Linnaeus, 1766

#### Molothrus ater

Boddaert, 1783

#### Quiscalus quiscula

Linnaeus, 1758

#### Sturnella magna

Linnaeus, 1758

#### Xanthocephalus xanthocephalus

Bonaparte, 1826

#### Laniidae

#### Lanius excubitor

Linnaeus, 1758

#### Mimidae

#### Dumetella carolinensis

Linnaeus, 1766

#### Mimus polyglottos

Linnaeus, 1758

#### Toxostoma rufum

Linnaeus, 1758

#### Motacillidae

#### Anthus rubescens

Tunstall, 1771

#### Paridae

#### Poecile atricapillus

Linnaeus, 1766

#### Parulidae

#### Cardellina canadensis

Linnaeus, 1766

#### Cardellina pusilla

A. Wilson, 1811

#### Geothlypis philadelphia

A. Wilson, 1810

#### Geothlypis trichas

Linnaeus, 1766

#### Leiothlypis celata

Say, 1822

#### Leiothlypis peregrina

A. Wilson, 1811

#### Leiothlypis ruficapilla

A. Wilson, 1811

#### Oporornis agilis

A. Wilson, 1812

#### Setophaga americana

Linnaeus, 1758

#### Setophaga caerulescens

J. F. Gmelin, 1789

#### Setophaga castanea

A. Wilson, 1810

#### Setophaga citrina

Boddaert, 1783

#### Setophaga coronata

Linnaeus, 1766

#### Setophaga discolor

Vieillot, 1809

#### Setophaga fusca

Statius Müller, 1776

#### Setophaga magnolia

A. Wilson, 1811

#### Setophaga palmarum

J. F. Gmelin, 1789

#### Setophaga pensylvanica

Linnaeus, 1766

#### Setophaga petechia

Linnaeus, 1766

#### Setophaga pinus

Linnaeus, 1766

#### Setophaga ruticilla

Linnaeus, 1758

#### Setophaga striata

J. R. Forster, 1772

#### Setophaga tigrina

J. F. Gmelin, 1789

#### Setophaga virens

J. F. Gmelin, 1789

#### Vermivora chrysoptera

Linnaeus, 1766

#### Vermivora cyanoptera

Olson & Reveal, 2009

#### Passeridae

#### Passer domesticus

Linnaeus, 1758

#### Peucedramidae

#### Nycticorax nycticorax

Linnaeus, 1758

#### Parkesia noveboracensis

J. F. Gmelin, 1789

#### Picoides arcticus

Swainson, 1832

#### Seiurus aurocapilla

Linnaeus, 1766

#### Regulidae

#### Regulus calendula

(Linnaeus, 1766)

#### Regulus satrapa

Lichtenstein 1823

#### Sittidae

#### Sitta canadensis

Linnaeus, 1766

#### Sitta carolinensis

Latham, 1790

#### Sturnidae

#### Sturnus vulgaris

Linnaeus, 1758

#### Sylviidae

#### Polioptila caerulea

Linnaeus, 1766

#### Thraupidae

#### Piranga olivacea

Gmelin, 1789

#### Troglodytidae

#### Cistothorus palustris

A. Wilson, 1810

#### Thryothorus ludovicianus

(Latham, 1790)

#### Troglodytes aedon

Vieillot, 1809

#### Troglodytes hiemalis

Vieillot, 1819

#### Turdidae

#### Catharus fuscescens

(Stephens, 1817)

#### Catharus guttatus

Pallas, 1811

#### Catharus minimus

Lafresnaye, 1848

#### Catharus ustulatus

Nuttall, 1840

#### Hylocichla mustelina

Gmelin, 1789

#### Sialia sialis

Linnaeus, 1758

#### Turdus migratorius

Linnaeus, 1766

#### Tyrannidae

#### Contopus cooperi

Nuttall, 1831

#### Contopus virens

Linnaeus, 1766

#### Empidonax alnorum

Brewster, 1895

#### Empidonax flaviventris

W. M. Baird & S. F. Baird, 1843

#### Empidonax Minimus

W. M. Baird & S. F. Baird, 1843

#### Empidonax traillii

Audubon, 1828

#### Myiarchus crinitus

Linnaeus, 1758

#### Sayornis phoebe

Latham, 1790

#### Tyrannus tyrannus

Linnaeus, 1758

#### Vireonidae

#### Vireo flavifrons

Vieillot, 1808

#### Vireo gilvus

Vieillot, 1808

#### Vireo griseus

Boddaert, 1783

#### Vireo olivaceus

Linnaeus, 1766

#### Vireo philadelphicus

Cassin, 1851

#### Vireo solitarius

A. Wilson, 1810

#### Pelecaniformes

#### Phalacrocoracidae

#### Phalacrocorax auritus

Lesson, 1831

#### Piciformes

#### Picidae

#### Colaptes auratus

Linnaeus, 1758

#### Dryocopus Pileatus

Linnaeus, 1758

#### Melanerpes carolinus

Linnaeus, 1758

#### Picoides pubescens

Linnaeus, 1766

#### Picoides villosus

Linnaeus, 1766

#### Sphyrapicus varius

Linnaeus, 1766

#### Podicipediformes

#### Podicipedidae

#### Podiceps auritus

Linnaeus, 1758

#### Podiceps grisegena

Boddaert, 1783

#### Podilymbus podiceps

Linnaeus, 1758

#### Strigiformes

#### Strigidae

#### Aegolius acadicus

Gmelin, 1788

#### Asio flammeus

Pontoppidan, 1763

#### Bubo virginianus

Gmelin, 1788

#### Megascops asio

Linnaeus, 1758

#### Strix varia

Barton, 1799

#### Mammalia

#### Artiodactyla

#### Cervidae

#### Odocoileus virginianus

Zimmermann, 1780

#### Carnivora

#### Canidae

#### Canis latrans

Say, 1823

#### Vulpes vulpes

Linnaeus, 1758

#### Felidae

#### Lynx rufus

Schreber, 1777

#### Mephitidae

#### Mephitis mephitis

Schreber, 1776

#### Mustelidae

#### Lontra canadensis

Schreber, 1777

#### Mustela erminea

Linnaeus, 1758

#### Neovison vison

Schreber, 1777

#### Procyonidae

#### Procyon lotor

Linnaeus, 1758

#### Chiroptera

#### Vespertilionidae

#### Eptesicus fuscus

Palisot de Beauvois, 1796

#### Lasionycteris noctivagans

LeConte, 1831

#### Lasiurus borealis

Müller, 1776

#### Lasiurus cinereus

Palisot de Beauvois, 1796

#### Myotis lucifugus

LeConte, 1831

#### Myotis septentrionalis

Trouessart, 1897

#### Didelphimorphia

#### Didelphidae

#### Didelphis virginiana

Kerr, 1792

#### Lagomorpha

#### Leporidae

#### Lepus americanus

Erxleben, 1777

#### Lepus europaeus

Pallas, 1778

#### Sylvilagus floridanus

J. A. Allen, 1890

#### Rodentia

#### Castoridae

#### Castor canadensis

Kuhl, 1820

#### Cricetidae

#### Microtus pennsylvanicus

Ord, 1815

#### Myodes gapperi

Vigors, 1830

#### Ondatra zibethicus

Linnaeus, 1766

#### Peromyscus leucopus

Rafinesque, 1818

#### Peromyscus maniculatus

Wagner, 1845

#### Dipodidae

#### Zapus hudsonius

Zimmermann, 1780

#### Erethizontidae

#### Erethizon dorsatus

Linnaeus, 1758

#### Muridae

#### Mus musculus

Linnaeus, 1758

#### Rattus norvegicus

Berkenhout, 1769

#### Sciuridae

#### Glaucomys sabrinus

Shaw, 1801

#### Marmota monax

Linnaeus, 1758

#### Sciurus carolinensis

Gmelin, 1788

#### Tamias striatus

Linnaeus, 1758

#### Tamiasciurus hudsonicus

Erxleben, 1777

#### Soricomorpha

#### Soricidae

#### Blarina brevicauda

Say, 1823

#### Sorex cinereus

Kerr, 1792

#### Sorex fumeus

G. M. Miller, 1895

#### Talpidae

#### Condylura cristata

Linnaeus, 1758

#### Reptilia

#### Squamata

#### Colubridae

#### Lampropeltis triangulum triangulum

(Lacépède, 1789)

#### Nerodia sipedon

Linnaeus, 1758

#### Opheodrys vernalis

Harlan, 1827

#### Regina septemvittata

Say, 1825

#### Storeria dekayi

Holbrook, 1839

#### Storeria occipitomaculata

Storer, 1839

#### Thamnophis sauritus

Linnaeus, 1766

#### Thamnophis sirtalis sirtalis

(Linnaeus, 1758)

#### Testudines

#### Chelydridae

#### Chelydra serpentina

Linnaeus, 1758

#### Emydidae

#### Chrysemys picta

Schneider, 1783

#### Mollusca

#### Gastropoda

#### Discidae

#### Anguispira alternata

Say, 1816

#### Pristilomatidae

#### Hawaiia minuscula

A. Binney, 1841

#### Succineidae

#### Novisuccinea ovalis

Say, 1817

#### Vertiginidae

#### Vertigo bollesiana

E. S. Morse, 1865

#### Basommatophora

#### Planorbidae

#### Gyraulus circumstriatus

Tryon, 1866

#### Hygrophila

#### Physidae

#### Physa gyrina

Say, 1821

#### Pulmonata

#### Zonitidae

#### Paravitrea multidentata

A. Binney, 1840

#### Stylommatophora

#### Agriolimacidae

#### Deroceras reticulatum

Muller, 1774

#### Arionidae

#### Arion fuscus

(O. F. Müller, 1774)

#### Arion subfuscus

Draparnaud, 1805

#### Gastrodontidae

#### Oxychilus allarius

(J. S. Miller, 1822)

#### Zonitoides arboreus

Say, 1816

##### Notes

BOLD:AAN3419

#### Helicidae

#### Cepaea nemoralis

Linnaeus, 1758

#### Cochlicopa lubrica

(Muller, 1774)

#### Helicodiscidae

#### Helicodiscus parallelus

Say, 1817

#### Hygromiidae

#### Trochulus hispidus

(Linnaeus, 1758)

##### Notes

BOLD:ACI9420

#### Succineidae

#### Succinea putris

Linnaeus, 1758

#### Vitrinidae

#### Vitrina angelicae

Beck, 1837

### Checklist of species observed or collected at the *rare* Charitable Research Reserve in Cambridge, Ontario, Canada. This checklist contains members of Kingdom Amoebozoa, Phylum Mycetozoa.

#### Myxogastria

#### Liceales

#### Tubiferaceae

#### Lycogala epidendrum

Linnaeus, 1829

##### Notes

/ flavofuscum

#### Liceida

#### Reticulariaceae

#### Tubifera ferruginosa

(Batsch) J.F. Gmel.

#### Stemonitida

#### Stemonitidae

#### Stemonitis axifera

Bull, 1791

### Checklist of species observed or collected at the *rare* Charitable Research Reserve in Cambridge, Ontario, Canada. This checklist contains records from Kingdom Fungi, including Phyla Ascomycota, Basidiomycota, and Zygomycota.

#### Ascomycota

#### Dictyocatenulata alba

Finley & E. F. Morris 1967

#### Ascomycota

#### Arthoniomycetes

#### Arthoniales

#### Arthoniaceae

#### Arthonia caesia

Koerber, 1861

#### Arthonia caudata

Willey, 1892

#### Arthonia radiata

Vetensk-Akad, 1808

#### Dothideomycetes

#### Pleosporales

#### Venturiaceae

#### Apiosporina morbosa

Arx, 1954

#### Polyporales

#### Fomitopsidaceae

#### Ischnoderma resinosum

Schrader, 1794

#### Piptoporus betulinus

Bulliard, 1780

#### Postia caesia

Schrad, 1881

##### Notes

(*Oligoporus caesius*)

#### Postia fragilis

Jülich, 1982

#### Ganodermataceae

#### Ganoderma applanatum

Persoon, 1799

#### Ganoderma lucidum

Curtis 1791

##### Notes

or Ganoderma tsugae

#### Meripilaceae

#### Gloeoporus dichrous

Fries, 1815

#### Grifola frondosa

Dickson, 1785

#### Meruliaceae

#### Bjerkandera adusta

Willdenow, 1787

#### Climacodon septentrionale

Fries, 1821

#### Phlebia radiata

Fries, 1821

#### Porotheleum fimbriatum

Persoon, 1818

#### Phanerochaetaceae

#### Steccherinum ochraceum

Persoon, 1792

#### Polyporaceae

#### Cerrena unicolor

Bulliard, 1785

#### Favolus alveolaris

De Candolle 1815

#### Laetiporus sulphureus

Bulliard, 1780

#### Lenzites betulina

Linnaeus, 1753

#### Polyporus badius

Persoon, 1801

#### Polyporus brumalis

Persoon, 1794

#### Trametes elegans

Sprengel, 1820

#### Trichaptum abietinum

Dickson, 1972

##### Notes

(?)

#### Tyromyces chioneus

Fries, 1815

##### Notes

(Tyromyces albellus, Polyorus albellus

#### Steccherinaceae

#### Irpex lacteus

Fries, 1818

#### Eurotiomycetes

#### Mycocaliciales

#### Mycocaliciaceae

#### Mycocalicium subtile

Persoon, 1925

#### Phaeocalicium curtisii

Tuckerman, 1975

#### Verrucariales

#### Verrucariaceae

#### Verrucaria calkinsiana

Servít, 1950

#### Lecanoromycetes

#### Baeomycetales

#### Trapeliaceae

#### Trapelia involuta

(Taylor) Hertel

#### Trapelia placodioides

Coppins & P. James

#### Candelariales

#### Candelariaceae

#### Candelaria concolor

(Dicks.) Stein

#### Candelariella aurella

(Hoffm.) Zahlbr.

#### Candelariella efflorescens

R.C. Harris & W.R. Buck

#### Lecanorales

#### Acarosporaceae

#### Acarospora glaucocarpa

(Ach.) Körb.

#### Cladoniaceae

#### Cladonia ochrochlora

Flörke

#### Lecanoraceae

#### Lecanora allophana

(Ach.) Nyl. 1872

##### Notes

f. sorediata

#### Lecanora allophana f. sorediata

#### Lecanora pulicaris

(Pers.) Ach.

#### Lecanora sambuci

(Pers.) Nyl.

#### Lecanora symmicta

(Ach.) Ach.

#### Lecanora thysanophora

R. C. Harris

#### Lecidella carpathica

Körb.

#### Parmeliaceae

#### Flavoparmelia caperata

(Linnaeus) Hale (1986)

#### Flavopunctelia flaventior

(Stirt.) Hale

#### Melanelixia subaurifera

(Nyl.) O. Blanco et al.

#### Parmelia squarrosa

Hale

#### Parmelia sulcata

Taylor

#### Punctelia rudecta

(Ach.) Krog

#### Punctelia subrudecta

(Nyl.) Krog

#### Xanthoparmelia cumberlandia

(Gyeln.) Hale, 1974

#### Peltigeraceae

#### Peltigera evansiana

Gyelnik

#### Peltigera rufescens

(Weiss) Humb.

#### Porpidiaceae

#### Bilimbia sabuletorum

(Schreb.) Arnold

#### Psoraceae

#### Protoblastenia rupestris

(Scop.) J. Steiner

#### Psora decipiens

(Hedwig) Hoffm.

#### Ramalinaceae

#### Bacidia schweinitzii

(Fr. ex E. Michener) A. Schneider

#### Lecania croatica

(Zahlbr.) Kotlov

#### Lecania naegelii

(Hepp) Diederich & v. d. Boom

#### Stereocaulaceae

#### Lepraria incana

(Linnaeus) Ach.

#### Lepraria lobificans

Nyl.

#### Lecanoromycetes

#### Thelenellaceae

#### Julella fallaciosa

(Arnold) R. C. Harris

#### Ostropales

#### Coenogoniaceae

#### Coenogonium pineti

(Ach.) Lücking & Lumbsch, 2004

#### Graphidaceae

#### Graphis scripta

(Linnaeus) Ach. (1809)

#### Phlyctidaceae

#### Phlyctis spp.

#### Stictidaceae

#### Conotrema urceolatum

(Ach.) Gilenstam 2005

#### Teloschistales

#### Physciaceae

#### Buellia punctata

(Hoffm.) A. Massal.

#### Buellia stillingiana

J. Steiner 1919

#### Hyperphyscia adglutinata

(Flörke) H. Mayrhofer & Poelt

#### Phaeophyscia adiastola

(Essl.) Essl. 1978

#### Phaeophyscia orbicularis

(Neck.) Moberg

#### Phaeophyscia pusilloides

(Zahlbr.) Essl. 1978

#### Phaeophyscia rubropulchra

(Degel.) Moberg 1978

#### Physcia adscendens

(Th. Fr.) H. Olivier

#### Physcia aipolia

(Ehrh. ex Humb.) Fürnr.

#### Physcia millegrana

Degel.

#### Physcia stellaris

(Linnaeus) Nyl.

#### Physciella chloantha

(Ach.) Essl.

#### Physconia detersa

(Nyl.) Poelt

#### Teloschistaceae

#### Caloplaca flavovirescens

(Wulfen) Søchting, Frödén & Arup

#### Caloplaca pyracea

(Ach.) Zwackh

#### Xanthomendoza fallax

(Hepp ex Arn.) Soechting, KSrnefelt & S. Kondratyuk

#### Xanthomendoza ulophyllodes

(RSsSnen) Soechting, KSrnefelt & S. Kondratyuk

#### Xanthoria parietina

(Linnaeus) Th. Fr.

#### Leotiomycetes

#### Helotiales

#### Bulgariaceae

#### Bulgaria inquinans

(Pers.) Fr. (1822)

#### Helotiaceae

#### Ascocoryne cylichnium

(Tul.) Korf 1971

#### Bisporella citrina

(Batsch) Korf & S.E.Carp. (1974)

#### Chlorociboria aeruginascens

(Nyl.) Kanouse 1947

#### Hemiphacidiaceae

#### Chlorencoelia versiformis

(Pers.) J.R.Dixon (1975)

#### Pezizomycetes

#### Pezizales

#### Pezizaceae

#### Peziza repanda

Pers. (1808)

#### Pyronemataceae

#### Aleuria aurantia

(Pers.) Fuckel, 1870

#### Jafnea semitosta

(Berk. & M.A. Curtis) Korf

#### Scutellinia scutellata

(Linnaeus) Lambotte 1887

#### Scutellinia setosa

Nees) Kuntze (1891)

##### Notes

/erinaceous

#### Tarzetta cupularis

(Linnaeus) Svrček (1981)

#### Sordariomycetes

#### Hypocreales

#### Bionectriaceae

#### Ovicuculispora parmeliae

(Berk. & Curt.) Etayo

#### Hypocreaceae

#### Hypomyces lactifluorum

(Schwein.) Tul. & C. Tul. 1860

#### Xylariales

#### Xylariaceae

#### Daldinia concentrica

(Bolton) Ces. & De Not. 1863

#### Hypoxylon fragiforme

(Pers.) J. Kickx f. 1835

#### Ustulina deusta

(Hoffm.) P.M.D. Martin, (1970)

##### Notes

(?)

#### Xylaria longipes

Nitschke 1867

#### Xylaria polymorpha

(Pers.) Grev. 1824

#### Basidiomycota

#### Agaricomycetes

#### Agaricales

#### Agaricaceae

#### Agaricus arvensis

Schaeff. 1774

#### Agaricus placomyces

Peck 1878

#### Calvatia gigantea

(Batsch) Lloyd 1904

#### Coprinus comatus

(O.F. Müll.) Pers. 1797

#### Lepiota clypeolaria

(Bull.) P. Kumm. 1871

#### Lepiota cristata

Barla

#### Leucoagaricus naucinus

Wasser, 1977

##### Notes

(*Lepiota naucina*)

#### Lycoperdon perlatum

Persoon,1796

##### Notes

(*Lycoperdon gemmatum*)

#### Parasola plicatilis

(Curtis) Redhead, Vilgalys & Hopple 2001

#### Amanitaceae

#### Amanita bisporigera

G.F.Atk., 1906

#### Bolbitiaceae

#### Conocybe rugosa

(Peck) Watling, 1981

#### Panaeolus foenisecii

R.Maire, 1933

##### Notes

(*Panaeolina foenisecii*)

#### Cortinariaceae

#### Gymnopilus spectabilis

(Fr.) A. H. Smith

##### Notes

(*Gymnopilus junonius*)

#### Phaeomarasmius proximans

Singer, 1989

#### Entolomataceae

#### Clitopilus prunulus

P. Kumm., 1871

#### Clitopilus scyphoides

Singer, 1946

#### Entoloma abortivum

Donk, 1949

#### Entoloma spp.

#### Entoloma strictus

Hesler (1967)

##### Notes

(*Nolanea strictia*)

#### Leptonia incana

Gillet, ?

##### Notes

Or euchlora

#### Hygrophoraceae

#### Arrhenia epichysium

(Pers.) Redhead, Lutzoni, Moncalvo & Vilgalys 2002

#### Hygrocybe coccinea

P. Kumm, 1871

#### Hygrocybe flavescens

Singer, 1951

#### Hygrocybe miniata

P. Kumm, 1871

#### Hygrocybe pratensis

Murrill, 1914

##### Notes

(*Camarophyllus pratensis*)

#### Hygrocybe punicea

P. Kumm, 1871

#### Hygrocybe virginea

P.D. Orton & Watling, 1969

#### Hygrophorus eburneus

Fries, 1838

#### Hymenogastraceae

#### Galerina autumnalis

(Peck) A.H. Sm. & Singer

##### Notes

(*Galerina marginata*)

#### Hebeloma crustuliniforme

Bulliard, 1787

#### Inocybaceae

#### Crepidotus applanatus

Persoon, 1796

#### Crepidotus mollis

Staude, 1857

#### Inocybe rimosa

Bulliard, 1789

#### Inocybe spp.

#### Crepidotus crocophyllus

Berkeley, 1847

#### Lycoperdaceae

#### Lycoperdon pyriforme

Schaeffer, 1774

#### Lyophyllaceae

#### Lyophyllum decastes

Fries, 1818

#### Marasmiaceae

#### Baeospora myosura

Fries, 1818

#### Clitocybula oculus

Peck, 1878

#### Marasmius rotula

Scopoli, 1772

#### Micromphale foetidum

(Sowerby) Singer, 1949

#### Pleurocybella porrigens

(Pers.) Singer, 1947

##### Notes

/ *Phylotus porrigens*

#### Rhodocollybia butryacea

(Bulliard, 1792) Lennox, 1979

##### Notes

(*Collybia butryacea*)

#### Gymnopus acervatus

Fries, 1821

##### Notes

/ *Connopus acervatus*

#### Xerula furfuracea

(Peck, 1893) R. H. Petersen, 2010

#### Mycenaceae

#### Mycena galericulata

(Scop.) Gray (1821)

#### Mycena haematopus

(Pers.) P.Kummmer, 1871

#### Mycena inclinata

(Fr.) Quél.,1872

#### Mycena leaiana

(Berk.) Saccardo,1891

#### Mycena olida

Bresadola,1887

#### Mycena osmundicola

J. E. Lange, 1914

#### Mycena spp.

#### Panellus stipticus

(Bull.) P.Karst. (1879)

#### Xeromphalina kauffmanii

A. H. Smith, 1953

#### Physalacriaceae

#### Armillaria gallica

Marxmüller & Romagnesi, 1987

#### Armillaria mellea complex

(Vahl, 1790) Kummer, 1871

#### Armillaria ostoyae

(Romagnesi, 1970) Herink, 1973

#### Flammulina velutipes

(Curtis, 1782) Singer, 1951

#### Hymenopellis limonispora

R.H. Petersen, 2010

#### Xerula megalospora

(Clements, 1896) R. H. Petersen, 2010

#### Pleurotaceae

#### Pleurotus ostreatus

(Jacquin) Kummer, 1871

#### Pluteaceae

#### Pluteus atricapillus

(Schaeffer) Kummer, 1871

#### Pluteus atromarginatus

(Konrad, 1927) Kühner, 1935

#### Pluteus cervinus

(Schaeffer, 1774) Kummer, 1871

##### Notes

(*Pluteus atricapillus*)

#### Pluteus hongoi

Singer, 1989

#### Pluteus longistriatus

Peck, 1885

#### Pluteus umbrosus

(Pers.) P. Kummer, 1871

##### Notes

(*Pluteus granularis*)

#### Psathyrellaceae

#### Coprinellus micaceus

(Bull.:Fr.) Vilgalys, Hopple & Jacq. Johnson

#### Coprinopsis atramentaria

(Bull.) Redhead, Vilgalys & Moncalvo (2001)

#### Coprinus disseminatus

(Pers.) J.E.Lange (1938)

#### Psathyrella candolleana

(Fr.) Maire, 1937

#### Schizophyllaceae

#### Schizophyllum commune

Fries, 1815

#### Strophariaceae

#### Agrocybe praecox

(Pers.) Fayod

#### Gymnopilus penetrans

(Fr.) Maire

#### Gymnopolis sapineus

(Fr.) Maire

#### Hypholoma capnoides

(Fr.) P. Kummer, 1871

#### Hypholoma fasciculare

(Huds.:Fr.) P. Kummer

#### Hypholoma sublateritium

(Fr.) Quélet

#### Pholiota aurivella

(Batsch) P.Kumm. (1871)

#### Pholiota squarrosa

(Batsch) Kummer, 1871

#### Pholiota squarrosoides

(Peck, 1879) Saccardo, 1887

#### Tricholomataceae

#### Clitocybe ectypoides

(Pk) Sacc.

#### Clitocybe gibba

(Persoon, 1801) Harmaja, 2003

#### Collybia cookei

(Bres.) J.D.Arnold (1935)

#### Hygrophorus langei

(Kühner) A. Pearson, 1952

##### Notes

(?)

#### Hypsizygus tessulatus

(Bull:Fr) Singer

#### Lepista irina

(Fr.) H.E. Bigelow 1959

#### Lepista nuda

(Bull.) Cooke 1871

##### Notes

(*Clitocybe nuda*)

#### Omphalina spp.

#### Phyllotopsis nidulans

(Pers.) Singer 1936

#### Rhodotus palmatus

(Bull.) Maire 1926

#### Tricholoma aurantium

(Schaeff.) Ricken 1914

#### Tricholoma myomyces

(Schaeff.) P. Kumm. 1871

#### Tricholoma terreum

(P.Kumm., 1871)

#### Tricholoma virgatum

(P.Kumm., 1871)

#### Xeromphalina cauticinalis

(Kühner & Maire, 1934)

#### Panellus serotinus

(Kühner,1950)

#### Clavariaceae

#### Multiclavula mucida

(R.H. Peterson, 1967)

#### Boletales

#### Boletaceae

#### Boletinellus merulioides

(Schwein.) Murrill 1909

#### Leccinum scabrum

Gray 1821

#### Sclerodermataceae

#### Scleroderma areolatum

Ehrenberg, 1818

#### Scleroderma citrinum

Persoon, 1801

#### Scleroderma michiganense

(Guzmán) Guzmán 1970

#### Suillaceae

#### Suillus americanus

(Peck) Snell (1959)

#### Suillus brevipes

(Peck) Kuntze 1898

#### Suillus granulatus

(Linnaeus) Roussel 1796

#### Suillus luteus

(Linnaeus) Roussel 1796

#### Cantharellales

#### Cantharellaceae

#### Craterellus fallax

Smith 1968

#### Corticiales

#### Corticiaceae

#### Phlebia tremellosa

(Schrader) Nakasone & Burdsall, 1984

#### Geastrales

#### Geastraceae

#### Geastrum fimbriatum

Fries 1829

#### Geastrum quadrifidum

Persoon, 1794

#### Gomphales

#### Gomphaceae

#### Ramaria spp.

#### Ramaria strictavar.concolor

(Persoon) Quélet 1888

#### Hymenochaetales

#### Hymenochaetaceae

#### Phellinus igniarius

(Linnaeus) Quélet 1886

#### Phallales

#### Phallaceae

#### Phallus duplicatus

Bosc (1811)

#### Phallus impudicus

Linnaeus 1753

#### Phallus ravenelii

Berkeley & M.A.Curtis (1873)

#### Polyporales

#### Meruliaceae

#### Loweomyces fractipes

(Berkeley & M.A. Curtis) Jülich 1982

#### Polyporaceae

#### Daedaleopsis confragosa

(Bolton) J. Schröter 1888

#### Fomes fomentarius

(Linnaeus) J. Kickx, 1867

#### Polyporus radicatus

Schweinitz, 1832.

#### Polyporus squamosus

(Hudson) Fries 1821

#### Polyporus varius

(Persoon) Fries 1821

#### Trametes gibbosa

(Persoon) Fries 1836

#### Trametes pubescens

(Schumacher) Pilát, 1939.

#### Trametes versicolor

(Linnaeus) Lloyd 1921

#### Trichaptum biforme

(Fries) Ryvarden, 1972

#### Truncospora ohiensis

(Berkeley) Pilát 1953

#### Russulales

#### Hericiaceae

#### Hericium americanum

Ginns 1984

#### Hericium coralloides

(Scopoli) Persoon, 1794

#### Hericium erinaceus

(Bulliard) Persoon, 1797.

#### Russulaceae

#### Lactarius torminosus

(Schaeffer) Gray 1821

#### Russula rosacea

(Persoon) Gray 1821

#### Russula spp.

#### Stereaceae

#### Stereum ostrea

(Blume & Nees) Fries, 1838.

#### Stereum rugosum

Persoon 1794

#### Stereum striatum

(Fries) Fries 1838

#### Auriscalpiaceae

#### Lentinellus ursinus

(Fries) Kühner 1926

#### Thelephorales

#### Bankeraceae

#### Boletopsis subsquamosa

(Linnaeus) Kotl.& Pouz.

#### Basidiomycetes

#### Cortinariales

#### Cortinariaceae

#### Inocybe geophyllavar.geophylla

(Persoon) Kummer, 1871

#### Dacrymycetes

#### Dacrymycetales

#### Dacrymycetaceae

#### Calocera cornea

(Batsch) Fries, 1827.

#### Tremellomycetes

#### Tremellales

#### Exidiaceae

#### Exidia alba

Lloyd) Burt 1921

##### Notes

/ *Ductifera pululahuana*

#### Exidia glandulosa

(Bulliard) Fries (1822)

#### Tremellaceae

#### Tremella foliacea

Persoon (1800)

#### Tremella reticulate

(Berkeley) Farl. (1908)

#### Ustilaginomycetes

#### Ustilaginales

#### Ustilaginaceae

#### Ustilago maydis

(DC.) Corda

#### Zygomycota

#### Zygomycetes

#### Mortierellales

#### Mortierellaceae

#### Mortierella hyalina

(Harz) W. Gams

### Checklist of species observed or collected at the *rare* Charitable Research Reserve in Cambridge, Ontario, Canada. The first of two checklists for Kingdom Plantae, this checklist contains records from Phyla Bryophyta, Equisetophyta, Lycopodiophyta and Magnoliophyta (Class Liliopsida and Magnoliopsida).

#### Plantae

#### Bryophyta

#### Bryopsida

#### Bryales

#### Bryaceae

#### Bryum caespiticium

Hedw.

#### Bryum argenteum

Hedw.

#### Mniaceae

#### Mnium marginatum

(With.) Brid. ex P. Beauv.

#### Plagiomniaceae

#### Plagiomnium cuspidatum

(Hedw.) T. Kop.

#### Dicranales

#### Dicranaceae

#### Dicranum flagellare

Hedw.

#### Dicranum montanum

Hedw.

#### Ditrichaceae

#### Ceratodon purpureus

(Hedw.) Brid.

#### Ditrichum spp.

#### Fissidentaceae

#### Fissidens adianthoides

Hedw.

#### Fissidens taxifolius

Hedw.

#### Fissidens spp.

#### Leucobryaceae

#### Leucobryum glaucum

(Hedw.) Ångstr. in Fries

#### Encalyptales

#### Encalyptaceae

#### Encalypta procera

Bruch

#### Grimmiales

#### Grimmiaceae

#### Schistidium apocarpa

(Hedw.) Bsg

#### Schistidium rivulare

(Brid.) Podp.

#### Hedwigiales

#### Hedwigiaceae

#### Hedwigia ciliata

(Hedw.) P. Beauv.

#### Hypnales

#### Amblystegiaceae

#### Amblystegium serpens

(Hedw.) Schimp. in B.S.G.

#### Amblystegium varium

(Hedw.) Lindb.

#### Campylium chrysophyllum

(Brid.) J. Lange

#### Campylium hispidulum

(Brid.) Mitt.

#### Campylium spp.

#### Hygroamblystegium fluviatile

(Hedw.) Loeske

#### Hygroamblystegium tenax

(Hedw.) Jenn.

#### Leptodictyum riparium

(Hedw.) Warnst.

#### Drepanocladus aduncus

(Hedw.) Warnst.

#### Anomodontaceae

#### Anomodon attenuatus

(Hedw.) Hüb.

#### Anomodon rostratus

(Hedw.) Schimp.

#### Brachytheciaceae

#### Brachythecium spp.

#### Cirriphyllum piliferum

(Hedw.) Grout

#### Eurhynchium hians

(Hedw.) Sande Lac.

#### Eurhynchium pulchellum

(Hedw.) Jenn.

#### Climaciaceae

#### Climacium americanum

Brid.

#### Climacium dendroides

(Hedw.) Web. & Mohr

#### Entodontaceae

#### Entodon seductrix

(Hedw.) C. Müll.

#### Fontinalaceae

#### Fontinalis hypnoides

Hartm.

#### Hylocomiaceae

#### Rhytidiadelphus triquetrus

(Hedw.) Warnst.

#### Hypnaceae

#### Callicladium haldanianum

(Grev.) Crum

#### Homomallium adnatum

(Hedw.) Broth.

#### Hypnum curvifolium

Hedw.

#### Hypnum imponens

Hedw.

#### Hypnum lindbergii

Mitt.

#### Hypnum spp.

#### Platydictya subtilis

(Hedw.) Crum

#### Taxiphyllum deplanatum

(Bruch & Schimp. ex Sull.) Fleisch.

#### Leskeaceae

#### Leskea polycarpa

Hedw.

#### Leskeella nervosa

(Brid.) Loeske

#### Plagiotheciaceae

#### Isopterygiopsis muelleriana

(Schimp.) Iwats.

#### Thuidiaceae

#### Thuidium recognitum

(Hedw.) Lindb.

#### Thuidium delicatulum

(Hedw.) Schimp. in B.S.G.

#### Orthotrichales

#### Orthotrichaceae

#### Orthotrichum anomalum

Hedw.

#### Pottiales

#### Pottiaceae

#### Barbula convoluta

Hedw.

#### Desmatodon obtusifolius

(Schwaegr.) Schimp.

#### Hymenostylium recurvirostre

(Hedw.) Dix.

#### Tortella tortuosa

(Hedw.) Limpr.

#### Barbula unguiculata

Hedw.

#### Tortula ruralis

(Hedw.) Gaertn. et al.

#### Weissia spp.

#### Splachnales

#### Meesiaceae

#### Leptobryum pyriforme

(Hedw.) Wils.

#### Polytrichopsida

#### Polytrichales

#### Polytrichaceae

#### Atrichum angustatum

(Brid.) Bruch & Schimp. in B.S.G.

#### Atrichum undulatum

(Hedw.) P. Beauv.

#### Polytrichum spp.

#### Sphagnopsida

#### Sphagnales

#### Sphagnaceae

#### Sphagnum spp.

#### Tetraphidopsida

#### Tetraphidales

#### Tetraphidaceae

#### Tetraphis pellucida

Hedw.

#### Equisetophyta

#### Equisetopsida

#### Equisetales

#### Equisetaceae

#### Equisetum arvense

Linnaeus

#### Equisetum hyemale affine

(Engelm.) Calder & Roy L. Taylor

#### Equisetum palustre

Linnaeus

#### Equisetum pratense

Ehrh.

#### Equisetum scirpoides

Michx.

#### Equisetum sylvaticum

Linnaeus

#### Equisetum variegatum variegatum

Schleich. ex F. Weber & D. Mohr

#### Lycopodiophyta

#### Lycopodiopsida

#### Huperziaceae

#### Huperziaceae

#### Huperzia lucidula

(Michx.) Trevis.

#### Lycopodiales

#### Lycopodiaceae

#### Lycopodiella inundata

(Linnaeus) Holub

#### Lycopodium annotinum

Linnaeus

#### Lycopodium obscurum

Linnaeus

#### Lycopodium ssp.

#### Selaginellales

#### Selaginellales

#### Selaginellaceae

#### Selaginella eclipes

W.R. Buck

#### Magnoliophyta

#### Liliopsida

#### Alismatales

#### Alismataceae

#### Sagittaria latifolia

Willd.

#### Sagittaria rigida

Pursh

#### Araceae

#### Arisaema triphyllum

(Linnaeus) Schott

#### Symplocarpus foetidus

(Linnaeus) Salisb. ex Nutt.

#### Butomaceae

#### Butomus umbellatus

Linnaeus

#### Potamogetonaceae

#### Potamogeton crispus

Linnaeus

#### Stuckenia pectinata

(Linnaeus) Börner

#### Tofieldiaceae

#### Triantha glutinosa

(Michx.) Baker

#### Arales

#### Araceae

#### Calla palustris

Linnaeus

#### Lemna minor

Linnaeus

#### Wolffia borealis

(Engelm. ex Hegelm.) Landolt

#### Wolffia columbiana

H. Karst.

#### Asparagales

#### Amarylilidaceae

#### Allium tricoccum

Aiton

#### Asparagaceae

#### Asparagus officinalis

Linnaeus

#### Convallaria majalis

Linnaeus

#### Maianthemum canadense

Desf.

#### Maianthemum racemosum racemosum

(Linnaeus) Link

#### Maianthemum stellatum

(Linnaeus) Link

#### Iridaceae

#### Iris pseudacorus

Linnaeus

#### Orchidaceae

#### Cypripedium acaule

Aiton

#### Cypripedium arietinum

R. Br.

#### Cypripedium parviflorumvar.pubescens

(Willd.) O.W. Knight

#### Cypripedium reginae

Walter

#### Cyperales

#### Cyperaceae

#### Carex albursina

E. Sheld.

#### Carex aquatilis

Wahlenb.

#### Carex arctata

Boott ex Hook.

#### Carex atherodes

Spreng.

#### Carex backii

Boott

#### Carex bebbii

Olney ex Fernald

#### Carex blanda

Dewey

#### Carex bromoides

Schkuhr ex Willd.

#### Carex chordorrhiza

Ehrh. ex L. f.

#### Carex comosa

Boott

#### Carex crinita

Lam.

#### Carex cristatella

Britton

#### Carex deweyana

Schwein.

#### Carex diandra

Schrank

#### Carex disperma

Dewey

#### Carex eburnea

Boott

#### Carex foenea

Willd.

#### Carex formosa

Dewey

#### Carex glaucodea

Tuck. ex Olney

#### Carex gracillima

Schwein.

#### Carex granularis

Muhl. ex Willd.

#### Carex grisea

Wahlenb.

#### Carex hirtifolia

Mack.

#### Carex hitchcockiana

Dewey

#### Carex hystericina

Muhl. ex Willd.

#### Carex intumescens

Rudge

#### Carex jamesii

Schwein.

#### Carex lacustris

Willd.

#### Carex laevivaginata

(Kük.) Mack.

#### Carex lasiocarpa

Ehrh.

#### Carex laxiculmis

Schwein.

#### Carex laxiflora

Lam.

#### Carex leptalea

Wahlenb.

#### Carex livida

(Wahlenb.) Willd.

#### Carex lupuliformis

Sartwell ex Dewey

#### Carex lupulina

Muhl. ex Willd.

#### Carex normalis

Mack.

#### Carex peckii

Howe

#### Carex pedunculata

Muhl. ex Willd.

#### Carex pensylvanica

Lam.

#### Carex plantaginea

Lam.

#### Carex platyphylla

J. Carey

#### Carex projecta

Mack.

#### Carex pseudocyperus

Linnaeus

#### Carex retrorsa

Schwein.

#### Carex rosea

Schkuhr ex Willd.

#### Carex scabrata

Schwein.

#### Carex schweinitzii

Dewey ex Schwein.

#### Carex scoparia

Schkuhr ex Willd.

#### Carex sparganioides

Muhl. ex Willd.

#### Carex spp.

#### Carex stipata

Muhl. ex Willd.

#### Carex stricta

Lam.

#### Carex tetanica

Schkuhr

#### Carex tonsavar.rugosperma

(Mack.) Crins

#### Carex trichocarpa

Muhl. ex Willd.

#### Carex trisperma

Dewey

#### Carex utriculata

Boott

#### Carex vulpinoidea

Michx.

#### Carex woodii

Dewey

#### Cyperus bipartitus

Torr.

#### Cyperus esculentus

Linnaeus

#### Dulichium arundinaceum

(Linnaeus) Britton

#### Eleocharis acicularis

(Linnaeus) Roem. & Schult.

#### Eleocharis intermedia

Schult.

#### Eriophorum virginicum

Linnaeus

#### Eriophorum viridicarinatum

(Engelm.) Fernald

#### Schoenoplectiella smithiivar.smithii

(A. Gray) Hayasaka

#### Schoenoplectus tabernaemontani

(C.C. Gmel.) Palla

#### Scirpus atrovirens

Willd.

#### Scirpus cyperinus

(Linnaeus) Kunth

#### Juncales

#### Juncaceae

#### Juniperus virginiana

Linnaeus

#### Luzula acuminata

Raf.

#### Luzula multiflora multiflora

(Ehrh.) Lej.

#### Liliales

#### Iridaceae

#### Iris spp.

#### Iris versicolor

Linnaeus

#### Sisyrinchium angustifolium

Mill.

#### Sisyrinchium montanum

Greene

#### Sisyrinchium mucronatum

Michx.

#### Liliaceae

#### Clintonia borealis

(Aiton) Raf.

#### Erythronium albidum

Nutt.

#### Erythronium americanum americanum

Ker Gawl.

#### Hemerocallis fulva

(Linnaeus) L.

#### Hemerocallis lilioasphodelus

Linnaeus

#### Lilium canadense

Linnaeus

#### Lilium michiganense

Farw.

#### Lilium philadelphicum

Linnaeus

#### Lilium superbum

Linnaeus

#### Medeola virginiana

Linnaeus

#### Polygonatum biflorum

(Walter) Elliott

#### Polygonatum pubescens

(Willd.) Pursh

#### Streptopus lanceolatusvar.lanceolatus

(Aiton) Reveal

#### Trillium erectum

Linnaeus

#### Trillium grandiflorum

(Michx.) Salisb.

#### Trillium spp.

#### Uvularia grandiflora

Sm.

#### Melanthiaceae

#### Anticlea elegansvar.glaucus

(Nutt.) Zomlefer & Judd

#### Smilacaceae

#### Smilax herbacea

Linnaeus

#### Smilax tamnoides

Linnaeus

#### Najadales

#### Najadaceae

#### Najas flexilis

(Willd.) Rostk. & W.L.E. Schmidt

#### Potamogetonaceae

#### Potamogeton amplifolius

Tuck.

#### Potamogeton pusillus pusillus

Linnaeus

#### Orchidales

#### Orchidaceae

#### Aplectrum hyemale

(Muhl. ex Willd.) Torr.

#### Epipactis helleborine

(Linnaeus) Crantz

#### Galearis spectabilis

(Linnaeus) Raf.

#### Liparis loeselii

(Linnaeus) Rich.

#### Platanthera dilatata

(Pursh) Lindl. ex L.C. Beck

#### Platanthera hyperborea

(Linnaeus) Lindl.

#### Platanthera nivea

(Nutt.) Luer

#### Spiranthes lucida

(H.H. Eaton) Ames

#### Poales

#### Juncaceae

#### Juncus acuminatus

Michx.

#### Juncus articulatus

Linnaeus

#### Juncus brachycarpus

Engelm.

#### Juncus bufonius

Linnaeus

#### Juncus dudleyi

Wiegand

#### Juncus effusus

Linnaeus

#### Juncus nodosus

Linnaeus

#### Juncus tenuis

Willd.

#### Poaceae

#### Agrostis capillaris

Linnaeus

#### Agrostis gigantea

Roth

#### Agrostis stolonifera

Linnaeus

#### Alopecurus aequalis

Sobol.

#### Andropogon gerardii

Vitman

#### Avena fatua

Linnaeus

#### Avena sativa

Linnaeus

#### Brachyelytrum erectum

(Schreb.) P. Beauv.

#### Briza maxima

Linnaeus

#### Bromus ciliatus

Linnaeus

#### Bromus latiglumis

(Scribn. ex Shear) Hitchc.

#### Bromus pubescens

Muhl. ex Willd.

#### Calamagrostis canadensis

(Michx.) P. Beauv.

#### Cinna arundinacea

Linnaeus

#### Dactylis glomerata

Linnaeus

#### Danthonia spicata

(Linnaeus) P. Beauv. ex Roem. & Schult.

#### Dichanthelium dichotomumvar.dichotomum

(Linnaeus) Gould

#### Dichanthelium latifolium

(Linnaeus) Harvill

#### Dichanthelium linearifolium

(Scribn.) Gould

#### Digitaria sanguinalis

(Linnaeus) Scop.

#### Eleusine indica

(Linnaeus) Gaertn.

#### Elymus canadensis

Linnaeus

#### Elymus hystrix

Linnaeus

#### Elymus lanceolatus psammophilus

(J.M. Gillett & H. Senn) Á. Löve

#### Elymus repens

(Linnaeus) Gould

#### Elymus riparius

Wiegand

#### Elymus virginicusvar.virginicus

Linnaeus

#### Festuca rubra rubra

Linnaeus

#### Glyceria borealis

(Nash) Batch.

#### Glyceria canadensis

(Michx.) Trin.

#### Glyceria maxima

(Hartm.) Holmb.

#### Glyceria septentrionalis

Hitchc.

#### Glyceria striata

(Lam.) Hitchc.

#### Leersia oryzoides

(Linnaeus) Sw.

#### Lolium perenne

Linnaeus

#### Milium effusum

Linnaeus

#### Muhlenbergia glomerata

(Willd.) Trin.

#### Muhlenbergia mexicanavar.mexicana

(Linnaeus) Trin.

#### Muhlenbergia tenuiflora

(Willd.) Britton, Sterns & Poggenb.

#### Oryzopsis asperifolia

Michx.

#### Panicum capillare

Linnaeus

#### Panicum virgatum

Linnaeus

#### Patis racemosa

(Smith) Romaschenko, P.M. Peterson & Soreng

#### Phalaris arundinacea

Linnaeus

#### Poa compressa

Linnaeus

#### Poa nemoralis

Linnaeus

#### Poa pratensis pratensis

Linnaeus

#### Poa ssp.

#### Schizachne purpurascens

(Torr.) Swallen

#### Schizachyrium scoparium

(Michx.) Nash

#### Setaria viridis

(Linnaeus) P. Beauv.

#### Sorghastrum nutans

(Linnaeus) Nash

#### Sporobolus vaginiflorus

(Torr. ex A. Gray) Alph. Wood

#### Bromus inermis

Leysser

#### Bromus kalmii

A. Gray

#### Echinochloa crus-galli

(Linnaeus) P. Beauv.

#### Phleum pratense

Linnaeus

#### Phragmites australis

(Cav.) Trin. ex Steud.

#### Setaria pumila

(Poir.) Roem. & Schult.

#### Spartina pectinata

Link

#### Typhaceae

#### Typha angustifolia

Linnaeus

#### Typha latifolia

Linnaeus

#### Typhales

#### Sparganiaceae

#### Sparganium americanum

Nutt.

#### Sparganium eurycarpum

Engelm.

#### Magnoliopsida

#### Apiales

#### Apiaceae

#### Aegopodium podagraria

Linnaeus

#### Angelica atropurpurea

Linnaeus

#### Cicuta bulbifera

Linnaeus

#### Cicuta maculata

Linnaeus

#### Conioselinum chinense

(Linnaeus) Britton, Sterns & Poggenb.

#### Cryptotaenia canadensis

(Linnaeus) DC.

#### Daucus carota

Linnaeus

#### Erigenia bulbosa

(Michx.) Nutt.

#### Heracleum mantegazzianum

Sommier & Levier

#### Osmorhiza claytonii

(Michx.) C.B. Clarke

#### Sanicula canadensisvar.grandis

Fernald

#### Sanicula marilandica

Linnaeus

#### Sium suave

Walter

#### Thaspium trifoliatumvar.aureum

(Linnaeus) Britton

#### Zizia aurea

(Linnaeus) W.D.J. Koch

#### Araliaceae

#### Aralia elata

(Miq.) Seem.

#### Aralia nudicaulis

Linnaeus

#### Aralia racemosa racemosa

Linnaeus

#### Hydrocotyle americana

Linnaeus

#### Panax quinquefolius

Linnaeus

#### Panax trifolius

Linnaeus

#### Aristolochiales

#### Aristolochiaceae

#### Asarum canadense

Linnaeus

#### Asterales

#### Asteraceae

#### Achillea millefolium

Linnaeus

#### Ageratina altissima

(Linnaeus) King & H. Rob.

#### Ambrosia artemisiifolia

Linnaeus

#### Ambrosia trifida

Linnaeus

#### Antennaria neglecta

Greene

#### Arctium lappa

Linnaeus

#### Arctium minus

Bernh.

#### Artemisia campestris caudata

(Michx.) H.M. Hall & Clem.

#### Artemisia vulgaris

Linnaeus

#### Aster spp.

#### Bidens cernua

Linnaeus

#### Bidens connata

Muhl. ex Willd.

#### Bidens frondosa

Linnaeus

#### Bidens ssp.

#### Bidens vulgata

Greene

#### Carduus nutans

Linnaeus

#### Centaurea jacea

Linnaeus

#### Centaurea stoebe micranthos

(Gugler) Hayek

#### Cichorium intybus

Linnaeus

#### Cirsium arvense

(Linnaeus) Scop.

#### Cirsium discolor

(Muhl. ex Willd.) Spreng.

#### Cirsium muticum

Michx.

#### Cirsium oleraceum

(Linnaeus) Scop.

#### Cirsium pumilumvar.hilii

(Canby) B. Boivin

#### Cirsium vulgare

(Savi) Ten.

#### Echinacea purpurea

(Linnaeus) Moench

#### Erechtites hieraciifolius

(Linnaeus) Rafinesque ex de Candolle

#### Erigeron annuus

(Linnaeus) Pers.

#### Erigeron canadensis

Linnaeus

#### Erigeron philadelphicusvar.philadelphicus

Linnaeus

#### Erigeron pulchellus

Michx.

#### Erigeron strigosus

Muhl. ex Willd.

#### Eupatorium hyssopifolium

Linnaeus

#### Eupatorium perfoliatum

Linnaeus

#### Eurybia macrophylla

(Linnaeus) Cass.

#### Euthamia graminifolia

(Linnaeus) Nutt.

#### Eutrochium maculatumvar.maculatum

(Linnaeus) E.E. Lamont

#### Eutrochium purpureumvar.purpureum

(Linnaeus) E.E. Lamont

#### Helenium flexuosum

Raf.

#### Helianthus annuus

Linnaeus

#### Helianthus decapetalus

Linnaeus

#### Helianthus giganteus

Linnaeus

#### Helianthus tuberosus

Linnaeus

#### Heliopsis helianthoides

(Linnaeus) Sweet

#### Hieracium umbellatum

Linnaeus

#### Hieracium vulgatum

Fr.

#### Inula helenium

Linnaeus

#### Lactuca canadensis

Linnaeus

#### Lactuca serriola

Linnaeus

#### Lapsana communis

Linnaeus

#### Leucanthemum vulgare

Lam.

#### Liatris cylindracea

Michx.

#### Matricaria discoidea

DC.

#### Nabalus albus

(Linnaeus) Hooker

#### Nabalus altissimus

(Linnaeus) Hooker

#### Onopordum acanthium

Linnaeus

#### Packera aurea

(Linnaeus) Á. Löve & D. Löve

#### Pilosella ×floribunda

(Wimmer & Grabowski) Fries

##### Notes

X floribundum

#### Pilosella aurantiaca

(Linnaeus) F.W. Schultz & Schultz Bipontinus

#### Pilosella caespitosa

(Dumortier) P.D. Sell & C. West

#### Pilosella officinarum

F.W. Schultz & Schultz Bipontinus

#### Pilosella piloselloides piloselloides

(Villars) Soják

#### Pilosella piloselloides praealta

(Gochnat) S. Bräutigam & Greuter

#### Prenanthes spp.

#### Ratibida pinnata

(Vent.) Barnhart

#### Rudbeckia hirta

Linnaeus

#### Rudbeckia laciniata

Linnaeus

#### Solidago altissimavar.altissima

Linnaeus

#### Solidago caesia

Linnaeus

#### Solidago canadensis

Linnaeus

#### Solidago flexicaulis

Linnaeus

#### Solidago gigantea

Aiton

#### Solidago hispida

Muhl. ex Willd.

#### Solidago juncea

Aiton

#### Solidago nemoralis nemoralis

Aiton

#### Solidago patula

Muhl. ex Willd.

#### Solidago ptarmicoides

(Torr. & A. Gray) B. Boivin

#### Solidago rigida

Linnaeus

#### Solidago rugosa rugosa

Mill.

#### Solidago spp.

#### Solidago ulmifolia

Muhl. ex Willd.

#### Sonchus arvensis arvensis

Linnaeus

#### Sonchus asper

(Linnaeus) Hill

#### Sonchus oleraceus

Linnaeus

#### Symphyotrichum cordifolium

(Linnaeus) G.L. Nesom

#### Symphyotrichum ericoidesvar.ericoides

(Linnaeus) G.L. Nesom

#### Symphyotrichum laeve

(Linnaeus) Á. Löve & D. Löve

#### Symphyotrichum lanceolatum

(Willd.) G.L. Nesom

#### Symphyotrichum lateriflorumvar.lateriflorum

(Linnaeus) Á. Löve & D. Löve

#### Symphyotrichum lateriflorumvar.hirsuticaule

(Lindl. ex DC.) G.L. Nesom

#### Symphyotrichum novae-angliae

(Linnaeus) G.L. Nesom

#### Symphyotrichum novi-belgii

(Linnaeus) G.L. Nesom

#### Symphyotrichum oolentangiensevar.oolentangiense

(Riddell) G.L. Nesom

#### Symphyotrichum oolentangiense

(Riddell) G.L. Nesom

#### Symphyotrichum pilosumvar.pilosum

(Willd.) G.L. Nesom

#### Symphyotrichum puniceum

(Linnaeus) Á. Löve & D. Löve

#### Symphyotrichum racemosum

(Elliot) G.L. Nesom

#### Symphyotrichum spp.

#### Symphyotrichum urophyllum

(Lindl.) G.L. Nesom

#### Tanacetum vulgare

Linnaeus

#### Taraxacum officinale

F.H. Wigg.

#### Tragopogon dubius

Scop.

#### Tragopogon pratensis

Linnaeus

#### Tussilago farfara

Linnaeus

#### Xanthium strumarium

Linnaeus

#### Campanulaceae

#### Lobelia siphilitica

Linnaeus

#### Boraginales

#### Boraginaceae

#### Hackelia virginiana

(Linnaeus) I.M. Johnst.

#### Myosotis scorpioides

Linnaeus

#### Brassicales

#### Brassicaceae

#### Hesperis matronalis

Linnaeus

#### Rorippa sylvestris

(Linnaeus) Besser

#### Campanulales

#### Campanulaceae

#### Campanula rapunculoides

Linnaeus

#### Campanula rotundifolia

Linnaeus

#### Lobelia cardinalis

Linnaeus

#### Lobelia inflata

Linnaeus

#### Capparales

#### Brassicaceae

#### Alliaria petiolata

(M. Bieb.) Cavara & Grande

##### Notes

or petiolata

#### Barbarea vulgaris

W.T. Aiton

#### Berteroa incana

(Linnaeus) DC.

#### Brassica juncea

(Linnaeus) Czern.

#### Brassica rapa

Linnaeus

#### Capsella bursa-pastoris

(Linnaeus) Medik.

#### Cardamine bulbosa

(Schreb. ex Muhl.) Britton, Sterns & Poggenb.

#### Cardamine concatenata

(Michx.) Sw.

#### Cardamine diphylla

(Michx.) Alph. Wood

#### Cardamine maxima

(Nutt.) Alph. Wood

#### Cardamine pensylvanica

Muhl. ex Willd.

#### Erysimum cheiranthoides

Linnaeus

#### Nasturtium officinale

W.T. Aiton

#### Rorippa palustris palustris

Linnaeus

#### Sinapis alba

Linnaeus

#### Sinapis arvensis

Linnaeus

#### Sisymbrium altissimum

Linnaeus

#### Thlaspi arvense

Linnaeus

#### Turritis glabra

Linnaeus

#### Caryophyllales

#### Amaranthaceae

#### Amaranthus albus

Linnaeus

#### Amaranthus retroflexus

Linnaeus

#### Amaranthus tuberculatus

(Moq.) J.D. Sauer

#### Caryophyllaceae

#### Arenaria serpyllifolia

Linnaeus

#### Cerastium arvense arvense

Linnaeus

#### Cerastium fontanum vulgare

(Hartm.) Greuter & Burdet

#### Dianthus armeria

Linnaeus

#### Dianthus barbatus

Linnaeus

#### Saponaria officinalis

Linnaeus

#### Silene latifolia

(Mill.) Britten & Rendle

#### Silene vulgaris

(Moench) Garcke

#### Stellaria graminea

Linnaeus

#### Chenopodiaceae

#### Chenopodium album

Linnaeus

#### Phytolaccaceae

#### Phytolacca americanavar.americana

Linnaeus

#### Polygonaceae

#### Persicaria hydropiper

(Linnaeus) Opiz

#### Persicaria pensylvanica

(Linnaeus) M. Gómez

#### Rumex britannica

Linnaeus

#### Rumex crispus

Linnaeus

#### Rumex obtusifolius

Linnaeus

#### Rumex verticillatus

Linnaeus

#### Portulacaceae

#### Claytonia virginica

Linnaeus

#### Celastrales

#### Aquifoliaceae

#### Ilex verticillata

(Linnaeus) A. Gray

#### Celastraceae

#### Celastrus scandens

Linnaeus

#### Euonymus alatus

(Thunb.) Siebold

#### Euonymus atropurpureus

Jacq.

#### Euonymus obovatus

Nutt.

#### Cornales

#### Cornaceae

#### Cornus alternifolia

L. f.

#### Cornus canadensis

Linnaeus

#### Cornus obliqua

Raf.

#### Cornus racemosa

Lam.

#### Cornus rugosa

Lam.

#### Cornus stolonifera

Michx.

#### Cucurbitales

#### Cucurbitaceae

#### Echinocystis lobata

(Michx.) Torr. & A. Gray

#### Dipsacales

#### Adoxaceae

#### Viburnum acerifolium

Linnaeus

#### Viburnum lantana

Linnaeus

#### Viburnum lentago

Linnaeus

#### Viburnum opulus

Linnaeus

#### Viburnum opulus trilobumvar.americanum

Aiton

#### Viburnum rafinesquianum

Schult.

#### Caprifoliaceae

#### Caprifoliaceae spp.

#### Diervilla lonicera

Mill.

#### Dipsacus sativus

(Linnaeus) Honck.

#### Linnaea borealis longiflora

(Torr.) Hultén

#### Lonicera canadensis

Bartram & W. Bartram ex Marshall

#### Lonicera dioica

Linnaeus

#### Lonicera morrowii

A. Gray

#### Lonicera tatarica

Linnaeus

#### Sambucus canadensis

Linnaeus

#### Sambucus nigra

Linnaeus

#### Sambucus racemosa pubens

(Michx.) House

#### Symphoricarpos albus

(Linnaeus) S.F. Blake

#### Symphoricarpos occidentalis

Hook.

#### Triosteum aurantiacum

E.P. Bicknell

#### Dipsacaceae

#### Dipsacus fullonum

Linnaeus

#### Valerianaceae

#### Valeriana edulis cilata

(Torrey & A. Gray) F.G. Meyer

#### Ericales

#### Balsaminaceae

#### Impatiens capensis

Meerb.

#### Ericaceae

#### Epigaea repens

Linnaeus

#### Gaultheria hispidula

(Linnaeus) Muhl. ex Bigelow

#### Gaultheria procumbens

Linnaeus

#### Vaccinium angustifolium

Aiton

#### Vaccinium myrtilloides

Michx.

#### Primulaceae

#### Lysimachia arvensis

(Linnaeus) U. Manns & Anderberg

#### Pyrolaceae

#### Pyrola elliptica

Nutt.

#### Pyrola spp.

#### Euphorbiales

#### Euphorbiaceae

#### Euphorbia cyparissias

Linnaeus

#### Euphorbia esula

Linnaeus

### Checklist of species observed or collected at the *rare* Charitable Research Reserve in Cambridge, Ontario, Canada. The second of two checklists for Kingdom Plantae, this checklist contains records from Phyla Magnoliophyta (Class Magnoliopsida), Marchantiophyta, Pinophyta, and Pteridophyta.

#### Magnoliophyta

#### Plantae

#### Magnoliopsida

#### Fabales

#### Fabaceae

#### Amorpha canescens

Pursh

#### Amphicarpaea bracteata

(Linnaeus) Fernald

#### Apios americana

Medik.

#### Desmodium canadense

(Linnaeus) DC.

#### Glycyrrhiza lepidota

Pursh

#### Lespedeza capitata

Michx.

#### Lotus corniculatus

Linnaeus

#### Medicago lupulina

Linnaeus

#### Medicago sativa sativa

Linnaeus

#### Melilotus albus

Medik.

#### Robinia pseudoacacia

Linnaeus

#### Trifolium aureum

Pollich

#### Trifolium campestre

Schreb.

#### Trifolium hybridum

Linnaeus

#### Trifolium pratense

Linnaeus

#### Trifolium repens

Linnaeus

#### Vicia americana

Muhl. ex Willd.

#### Vicia cracca

Linnaeus

#### Fagales

#### Betulaceae

#### Alnus glutinosa

(Linnaeus) Gaertn.

#### Alnus incana rugosa

(Du Roi) R.T. Clausen

#### Betula alleghaniensis

Britton

#### Betula papyrifera

Marshall

#### Betula pendula

Roth

#### Betula populifolia

Marshall

#### Betula pumila

Linnaeus

#### Carpinus caroliniana virginiana

(Marshall) Furlow

#### Corylus americana

Walter

#### Corylus cornuta cornuta

Marshall

#### Ostrya virginiana

(Mill.) K. Koch

#### Fagaceae

#### Fagus grandifolia

Ehrh.

#### Quercus alba

Linnaeus

#### Quercus bicolor

Willd.

#### Quercus ellipsoidalis

E.J. Hill

#### Quercus macrocarpa

Michx.

#### Quercus palustris

Münchh.

#### Quercus rubra

Linnaeus

#### Quercus velutina

Lam.

#### Juglandaceae

#### Juglans nigra

Linnaeus

#### Gentianales

#### Apocynaceae

#### Apocynum androsaemifolium

Linnaeus

#### Apocynum cannabinum

Linnaeus

#### Apocynum sp.

##### Notes

X floribundum

#### Vinca minor

Linnaeus

#### Asclepiadaceae

#### Asclepias exaltata

Linnaeus

#### Asclepias incarnata incarnata

Linnaeus

#### Asclepias syriaca

Linnaeus

#### Asclepias tuberosa

Linnaeus

#### Gentianaceae

#### Gentiana andrewsii

Griseb.

#### Gentiana rubricaulis

Schwein.

#### Gentianella quinquefolia quinquefolia

(Linnaeus) Small

#### Gentianopsis crinita

(Froel.) Ma

#### Gentianopsis virgata virgata

(Raf.) Holub

##### Notes

or virgata

#### Rubiaceae

#### Galium aparine

Linnaeus

#### Galium asprellum

Michx.

#### Geraniales

#### Balsaminaceae

#### Impatiens glandulifera

Royle

#### Impatiens pallida

Nutt.

#### Geraniaceae

#### Geranium maculatum

Linnaeus

#### Geranium robertianum

Linnaeus

#### Oxalidaceae

#### Oxalis corniculata

Linnaeus

#### Oxalis montana

Raf.

#### Oxalis stricta

Linnaeus

#### Haloragales

#### Haloragaceae

#### Myriophyllum heterophyllum

Michx.

#### Myriophyllum verticillatum

Linnaeus

#### Hamamelidales

#### Platanaceae

#### Platanus occidentalis

Linnaeus

#### Juglandales

#### Juglandaceae

#### Carya cordiformis

(Wangenh.) K. Koch

#### Carya glabra

(Mill.) Sweet

#### Carya ovatavar.ovata

(Mill.) K. Koch

#### Juglans cinerea

Linnaeus

#### Lamiales

#### Boraginaceae

#### Anchusa arvensis

(Linnaeus) M. Bieb.

#### Cynoglossum officinale

Linnaeus

#### Echium vulgare

Linnaeus

#### Lithospermum parviflorum

Weakley, Witsell & D. Estes

#### Myosotis laxa

Lehm.

#### Myosotis verna

Nutt.

#### Symphytum officinale

Linnaeus

#### Lamiaceae

#### Agastache nepetoides

(Linnaeus) Kuntze

#### Ajuga reptans

Linnaeus

#### Blephilia hirsuta

(Pursh) Benth.

#### Clinopodium vulgare

Linnaeus

#### Collinsonia canadensis

Linnaeus

#### Galeopsis tetrahit

Linnaeus

#### Glechoma hederacea

Linnaeus

#### Lamium amplexicaule

Linnaeus

#### Leonurus cardiaca

Linnaeus

#### Lycopus americanus

Muhl. ex W.P.C. Bartram

#### Lycopus asper

Greene

#### Lycopus uniflorus

Michx.

#### Melissa officinalis

Linnaeus

#### Mentha arvensis

auct. non L.

#### Mentha canadensis

Linnaeus

#### Mentha sp.

##### Notes

X piperita

#### Mentha spicata

Linnaeus

#### Monarda didyma

Linnaeus

#### Monarda fistulosa

Sims

#### Nepeta cataria

Linnaeus

#### Physostegia virginiana virginiana

(Linnaeus) Benth.

#### Prunella vulgaris vulgaris

Linnaeus

#### Prunella vulgaris lanceolata

(W.P.C. Barton) Piper & Beattie

#### Prunella vulgaris

Linnaeus

#### Pycnanthemum virginianum

(Linnaeus) Rob. & Fernald

#### Scutellaria galericulata

Linnaeus

#### Scutellaria lateriflora

Linnaeus

#### Stachys hispida

Pursh

#### Stachys palustris

Linnaeus

#### Teucrium canadense canadense

Linnaeus

#### Oleaceae

#### Ligustrum vulgare

Linnaeus

#### Syringa vulgaris

Linnaeus

#### Plantaginaceae

#### Linaria vulgaris

Mill.

#### Plantago lanceolata

Linnaeus

#### Plantago major

Linnaeus

#### Plantago rugelii

Decne.

#### Veronica anagallis-aquatica

Linnaeus

#### Scrophulariaceae

#### Verbascum phoeniceum

Linnaeus

#### Verbascum thapsus

Linnaeus

#### Veronica chamaedrys

Linnaeus

#### Veronica officinalis

Linnaeus

#### Veronica serpyllifolia

Linnaeus

#### Verbenaceae

#### Verbena hastata

Linnaeus

#### Verbena simplex

Lehm.

#### Verbena stricta

Vent.

#### Verbena urticifolia

Linnaeus

#### Laurales

#### Lauraceae

#### Lindera benzoin

(Linnaeus) Blume

#### Linales

#### Linaceae

#### Linum virginianum

Linnaeus

#### Magnoliales

#### Annonaceae

#### Asimina triloba

(Linnaeus) Dunal

#### Malpighiales

#### Hypericaceae

#### Hypericum ascyron

Linnaeus

#### Hypericum mutilum mutilum

Linnaeus

#### Hypericum perforatum

Linnaeus

#### Hypericum punctatum

Lam.

#### Salicaceae

#### Populus alba

Linnaeus

#### Populus balsamifera

Linnaeus

#### Populus deltoides

W. Bartram ex Marshall

#### Populus grandidentata

Michx.

#### Populus nigra

Linnaeus

#### Populus tremuloides

Michx.

#### Malvales

#### Malvaceae

#### Abutilon theophrasti

Medik.

#### Malva moschata

Linnaeus

#### Tiliaceae

#### Tilia americana

Linnaeus

#### Myrtales

#### Lythraceae

#### Lythrum salicaria

Linnaeus

#### Onagraceae

#### Chamerion angustifolium angustifolium

(Linnaeus) Holub

#### Circaea alpina

Linnaeus

#### Circaea canadensis canadensis

(Linnaeus) Hill

#### Epilobium ciliatum glandulosum

(Lehm.) Hoch & P.H. Raven

#### Epilobium coloratum

Biehler

#### Epilobium hirsutum

Linnaeus

#### Epilobium leptophyllum

Raf.

#### Epilobium strictum

Muhl.

#### Ludwigia polycarpa

Short & Peter

#### Oenothera biennis

Linnaeus

#### Oenothera serrulata

Nuttall

#### Thymelaeaceae

#### Dirca palustris

Linnaeus

#### Nymphaeales

#### Cabombaceae

#### Brasenia schreberi

J.F. Gmel.

#### Papaverales

#### Papaveraceae

#### Chelidonium majus

Linnaeus

#### Dicentra canadensis

(Goldie) Walp.

#### Dicentra cucullaria

(Linnaeus) Bernh.

#### Sanguinaria canadensis

Linnaeus

#### Piperales

#### Saururaceae

#### Saururus cernuus

Linnaeus

#### Plantaginales

#### Plantaginaceae

#### Callitriche palustris

Linnaeus

#### Chelone glabra

Linnaeus

#### Digitalis lanata

Ehrh.

#### Digitalis lutea

Linnaeus

##### Notes

or grandiflora

#### Digitalis purpurea

Linnaeus

#### Polygalales

#### Polygalaceae

#### Polygala spp

#### Polygala verticillatavar.verticillata

Linnaeus

#### Polygaloides paucifolia

(Willdenow) J.R. Abbott

#### Polygonales

#### Polygonaceae

#### Fallopia convolvulus

(Linnaeus) Á. Löve

#### Fallopia scandens

(Linnaeus) Holub

#### Persicaria hydropiperoides

(Michx.) Small

#### Persicaria maculosa

Gray

#### Persicaria punctata

(Elliott) Small

#### Persicaria sagittata

(Linnaeus) H. Gross

#### Polygonum aviculare

Linnaeus

#### Primulales

#### Primulaceae

#### Lysimachia borealis

(Rafinesque) U. Manns & Anderberg

#### Lysimachia ciliata

Linnaeus

#### Lysimachia nummularia

Linnaeus

#### Lysimachia thyrsiflora

Linnaeus

#### Primula mistassinica

Michx.

#### Ranunculales

#### Berberidaceae

#### Berberis canadensis

Mill.

#### Berberis thunbergii

DC.

#### Berberis vulgaris

Linnaeus

#### Caulophyllum thalictroides

(Linnaeus) Michx.

#### Podophyllum peltatum

Linnaeus

#### Menispermaceae

#### Menispermum canadense

Linnaeus

#### Ranunculaceae

#### Actaea pachypoda

Elliott

#### Actaea rubra

(Aiton) Willd.

#### Actaea spp.

#### Anemone acutiloba

(DC.) G. Lawson

#### Anemone americana

(DC.) H. Hara

#### Anemone canadensis

Linnaeus

#### Anemone cylindrica

A. Gray

#### Anemone quinquefolia

Linnaeus

#### Anemone virginianavar.virginiana

Linnaeus

#### Anemone virginianavar.alba

(Oakes) Alph. Wood

#### Aquilegia canadensis

Linnaeus

#### Caltha palustris

Linnaeus

#### Clematis virginiana

Linnaeus

#### Coptis trifolia

(Linnaeus) Salisb.

#### Ranunculus abortivus

Linnaeus

#### Ranunculus acris

Linnaeus

#### Ranunculus hispidusvar.caricetorum

(Greene) T. Duncan

#### Ranunculus recurvatusvar.recurvatus

Poir.

#### Ranunculus repens

Linnaeus

#### Ranunculus sceleratusvar.sceleratus

Linnaeus

#### Thalictrum dasycarpum

Fisch. & Avé-Lall.

#### Thalictrum dioicum

Linnaeus

#### Thalictrum pubescens

Pursh

#### Thalictrum thalictroides

(Linnaeus) Eames & B. Boivin

#### Rhamnales

#### Elaeagnaceae

#### Elaeagnus angustifolia

Linnaeus

#### Elaeagnus umbellata

Thunb.

#### Shepherdia canadensis

(Linnaeus) Nutt.

#### Rhamnaceae

#### Frangula alnus

Mill.

#### Rhamnus alnifolia

L'Hér.

#### Rosales

#### Cannabaceae

#### Humulus lupulus

Linnaeus

#### Crassulaceae

#### Sedum acre

Linnaeus

#### Grossulariaceae

#### Ribes americanum

Mill.

#### Ribes cynosbati

Linnaeus

#### Ribes glandulosum

Grauer

#### Ribes hirtellum

Michx.

#### Ribes lacustre

(Pers.) Poir.

#### Ribes oxyacanthoidesvar.oxycanthoides

Linnaeus

#### Ribes rubrum

Linnaeus

#### Ribes ssp.

#### Ribes triste

Pall.

#### Hydrangeaceae

#### Philadelphus coronarius

Linnaeus

#### Rhamnaceae

#### Rhamnus cathartica

Linnaeus

#### Rosaceae

#### Agrimonia gryposepala

Wallr.

#### Amelanchier arborea

(F. Michx.) Fernald

#### Amelanchier laevis

Wiegand

#### Amelanchier spicata

(Lam.) K. Koch

#### Aruncus dioicus

(Walter) Fernald

#### Crataegus chrysocarpa

Ashe

#### Crataegus crus-galli

Linnaeus

#### Crataegus dissona

Sarg.

#### Crataegus punctata

Jacq.

#### Crataegus spp.

#### Dasiphora fruticosa

auct. non (Linnaeus) Rydb.

#### Fragaria vesca

Linnaeus

#### Fragaria virginiana

Duchesne

#### Geum aleppicum

Jacq.

#### Geum canadense

Jacq.

#### Geum fragarioides

(Michaux) Smedmark

#### Geum laciniatum

Murray

#### Geum rivale

Linnaeus

#### Geum urbanum

Linnaeus

#### Malus coronaria

(Linnaeus) Mill.

#### Malus pumila

Mill.

#### Physocarpus opulifolius

(Linnaeus) Maxim.

#### Potentilla anserina anserina

Linnaeus

#### Potentilla argentea

Linnaeus

#### Potentilla norvegica

Linnaeus

#### Potentilla recta

Linnaeus

#### Potentilla simplex

Michx.

#### Prunus americana

Marshall

#### Prunus avium

(Linnaeus) L.

#### Prunus mahaleb

Linnaeus

#### Prunus pensylvanica

L. f.

#### Prunus serotina

Ehrh.

#### Prunus virginiana virginiana

Linnaeus

#### Pyrus communis

Linnaeus

#### Rosa blanda

Aiton

#### Rosa cinnamomea

L. sensu 1759, non 1753

#### Rosa multiflora

Thunb.

#### Rosa palustris

Marshall

#### Rosa rugosa

Thunb.

#### Rosa virginiana

Mill.

#### Rubus allegheniensis

Porter

#### Rubus canadensis

Linnaeus

#### Rubus flagellaris

Willd.

#### Rubus hispidus

Linnaeus

#### Rubus idaeus

Linnaeus

#### Rubus occidentalis

Linnaeus

#### Rubus pubescens

Raf.

#### Rubus repens

(Linnaeus) Kuntze

#### Sorbus americana

Marshall

#### Sorbus aucuparia

Linnaeus

#### Spiraea alba

Du Roi

#### Saxifragaceae

#### Micranthes virginiensis

(Michx.) Small

#### Mitella diphylla

Linnaeus

#### Mitella nuda

Linnaeus

#### Parnassia glauca

Raf.

#### Tiarella cordifolia

Linnaeus

#### Rubiales

#### Rubiaceae

#### Galium circaezans

Michx.

#### Galium mollugo

Linnaeus

#### Galium palustre

Linnaeus

#### Galium tinctorium

Linnaeus

#### Galium trifidum trifidum

Linnaeus

#### Galium triflorum

Michx.

#### Mitchella repens

Linnaeus

#### Salicales

#### Salicaceae

#### Salix ×fragilis

Linnaeus

#### Salix alba

Linnaeus

#### Salix amygdaloides

Andersson

#### Salix bebbiana

Sarg.

#### Salix candida

Flüggé ex Willd.

#### Salix cordata

Muhl.

#### Salix discolor

Muhl.

#### Salix eriocephala

Michx.

#### Salix exigua

Nutt.

#### Salix humilisvar.humilis

Marshall

#### Salix lucida

Muhl.

#### Salix nigra

Marshall

#### Salix pedicellaris

Pursh

#### Salix petiolaris

Sm.

#### Salix purpurea

Linnaeus

#### Salix serissima

(L.H. Bailey) Fernald

#### Salix viminalis

Linnaeus

#### Sapindales

#### Aceraceae

#### Acer nigrum

F. Michaux

#### Acer platanoides

Linnaeus

#### Acer rubrum

Linnaeus

#### Acer saccharinum

Linnaeus

#### Acer saccharum

Marshall

#### Acer spicatum

Lam.

#### Anacardiaceae

#### Rhus aromatica

Aiton

#### Rhus typhina

Linnaeus

#### Toxicodendron radicans negundo

(Greene) Gillis

#### Toxicodendron radicansvar.rydbergii

(Small ex Rydb.) Erskine

#### Toxicodendron vernix

(Linnaeus) Kuntze

#### Rutaceae

#### Zanthoxylum americanum

Mill.

#### Sapindaceae

#### Acer negundo

Linnaeus

#### Aesculus hippocastanum

Linnaeus

#### Staphyleaceae

#### Staphylea trifolia

Linnaeus

#### Saxifragales

#### Haloragaceae

#### Myriophyllum sibiricum

Kom.

#### Scrophulariales

#### Oleaceae

#### Fraxinus americana

Linnaeus

#### Fraxinus nigra

Marsh.

#### Fraxinus pennsylvanica

Marshall

#### Orobanchaceae

#### Conopholis americana

(Linnaeus) Wallr.

#### Epifagus virginiana

(Linnaeus) W.P.C. Barton

#### Scrophulariaceae

#### Aureolaria flava

(Linnaeus) Farw.

#### Aureolaria virginica

(Linnaeus) Pennell

#### Pedicularis canadensis

Linnaeus

#### Penstemon digitalis

Nutt. ex Sims

#### Penstemon hirsutus

(Linnaeus) Willd.

#### Scrophularia lanceolata

Pursh

#### Scrophularia marilandica

Linnaeus

#### Verbascum blattaria

Linnaeus

#### Veronica americana

Schwein. ex Benth.

#### Solanales

#### Convolvulaceae

#### Calystegia spithamaea

(Linnaeus) Pursh

#### Convolvulus arvensis

Linnaeus

#### Cuscuta gronovii

Willd. ex Schult.

#### Hydrophyllaceae

#### Hydrophyllum canadense

Linnaeus

#### Hydrophyllum virginianum

Linnaeus

#### Polemoniaceae

#### Phlox subulata subulata

Linnaeus

#### Solanaceae

#### Physalis heterophylla

Nees

#### Solanum dulcamara

Linnaeus

#### Solanum lycopersicum

Linnaeus

#### Urticales

#### Moraceae

#### Morus alba

Linnaeus

#### Ulmaceae

#### Celtis occidentalis

Linnaeus

#### Ulmus americana

Linnaeus

#### Ulmus pumila

Linnaeus

#### Ulmus rubra

Muhl.

#### Ulmus thomasii

Sarg.

#### Urticaceae

#### Boehmeria cylindrica

(Linnaeus) Sw.

#### Laportea canadensis

(Linnaeus) Wedd.

#### Pilea pumila

(Linnaeus) A. Gray

#### Urtica dioica gracilis

(Aiton) Selander

#### Urtica dioica dioica

Linnaeus

#### Violales

#### Cucurbitaceae

#### Sicyos angulatus

Linnaeus

#### Violaceae

#### Viola aduncavar.adunca

Sm.

#### Viola canadensis

Linnaeus

#### Viola cucullata

Aiton

#### Viola labradorica

Schrank

#### Viola macloskeyi

F.E. Lloyd

#### Viola nephrophylla

Greene

#### Viola pubescens

Aiton

#### Viola renifolia

A. Gray

#### Viola rostrata

Pursh

#### Viola sororia

Willd.

#### Viola sororiavar.affinis

(Leconte) L.E. McKinney

#### Viola spp.

#### Vitales

#### Vitaceae

#### Parthenocissus quinquefolia

(Linnaeus) Planch.

#### Vitis riparia

Michx.

#### Lamiales

#### Phrymaceae

#### Phryma leptostachya

Linnaeus

#### Saxifragales

#### Hamamelidaceae

#### Hamamelis virginiana

Linnaeus

#### Marchantiophyta

#### Jungermanniopsida

#### Metzgeriales

#### Aneuraceae

#### Aneura pinguis

(Linnaeus) Dumort.

#### Jungermanniales

#### Geocalycaceae

#### Lophocolea heterophylla

(Schrad.) Dum.

#### Porellaceae

#### Porella platyphylla

(Linnaeus) Pfeiff.

#### Radulaceae

#### Radula complanata

(Linnaeus) Dum.

#### Porellales

#### Frullaniaceae

#### Frullania eboracensis

Gottsche

#### Marchantiopsida

#### Marchantiales

#### Aytoniaceae

#### Reboulia hemisphaerica

(Linnaeus) Raddi

#### Conocephalaceae

#### Conocephalum conicum

(Linnaeus) Lindb.

#### Marchantiaceae

#### Marchantia polymorpha

Linnaeus

#### Pinophyta

#### Pinopsida

#### Cupressales

#### Cupressaceae

#### Juniperus communis

Linnaeus

#### Juniperus communisvar.depressa

Pursh

#### Pinales

#### Cupressaceae

#### Thuja occidentalis

Linnaeus

#### Pinaceae

#### Larix decidua

Mill.

#### Larix laricina

(Du Roi) K. Koch

#### Picea abies

(Linnaeus) Karst.

#### Picea glauca

(Moench) Voss

#### Picea pungens

Engelm.

#### Pinus banksiana

Lamb.

#### Pinus resinosa

Aiton

#### Pinus strobus

Linnaeus

#### Pinus sylvestris

Linnaeus

#### Tsuga canadensis

(Linnaeus) Carrière

#### Pteridophyta

#### Filicopsida

#### Ophioglossales

#### Ophioglossaceae

#### Botrypus virginianus

(Linnaeus) Michaux

#### Polypodiales

#### Aspleniaceae

#### Asplenium platyneuron

(Linnaeus) Britton, Sterns & Poggenb.

#### Asplenium trichomanes trichomanes

Linnaeus

#### Asplenium viride

Huds.

#### Dennstaedtiaceae

#### Pteridium aquilinumvar.latiusculum

(Desv.) Underw. ex A. Heller

#### Dryopteridaceae

#### Athyrium filix-feminavar.angustum

(Willd.) G. Lawson

#### Cystopteris bulbifera

(Linnaeus) Bernh.

#### Cystopteris fragilis

(Linnaeus) Bernh.

##### Notes

or tenuis

#### Deparia acrostichoides

(Sw.) M. Kato

#### Dryopteris ×triploidea

Wherry

#### Dryopteris carthusiana

(Vill.) H.P. Fuchs

##### Notes

or spinulosa

#### Dryopteris clintoniana

(D.C. Eaton) Dowell

#### Dryopteris cristata

(Linnaeus) A. Gray

#### Dryopteris goldiana

(Hook. ex Goldie) A. Gray

#### Dryopteris intermedia

(Muhl. ex Willd.) A. Gray

#### Dryopteris marginalis

(Linnaeus) A. Gray

#### Gymnocarpium dryopteris

(Linnaeus) Newman

#### Matteuccia struthiopterisvar.pensylvanica

(Willd.) C.V. Morton

#### Onoclea sensibilis

Linnaeus

#### Polystichum acrostichoides

(Michx.) Schott

#### Osmundaceae

#### Osmunda claytoniana

Linnaeus

#### Osmunda regalisvar.spectabilis

(Willd.) A. Gray

#### Polypodiaceae

#### Polypodium virginianum

Linnaeus

##### Notes

or vulgare

#### Pteridaceae

#### Adiantum pedatum

Linnaeus

#### Pellaea glabella

Mett. ex Kuhn

#### Thelypteridaceae

#### Thelypteris noveboracensis

(Linnaeus) Nieuwl.

#### Thelypteris palustrisvar.pubescens

(G. Lawson) Fernald

#### Polypodiopsida

#### Osmundales

#### Osmundaceae

#### Osmundastrum cinnamomea

(Linnaeus) C. Presl

#### Osmundastrum cinnamomea

(Linnaeus) C. Presl

## Analysis

The two surveying strategies – a four-month long terrestrial arthropod survey, followed by a concentrated bioblitz targeting a variety of taxa – resulted in 25,287 and 3,502 specimens barcoded, respectively, out of a total of 32,645 specimens collected. Observations of an additional 125 species and two higher taxa (for which no voucher specimen was kept) were recorded at the bioblitz (Suppl. material 7​). Altogether the surveys covered 14 phyla, 29 classes, 117 orders, and 531 families of animals, plants, fungi and lichens. This comprised 3,986 BINs of animals, 1,193 of which are identified to species (Suppl. materials 4, 8, 9).

The most diverse groups were Ichneumonidae, Chironomidae, and Cecidomyiidae with 188, 365, and 584 BINs respectively. In terms of abundance, the three groups Sciaridae, Cecidomyiidae, and Chironomidae had the largest number of specimens with 1,528, 2,477, and 9,636 specimens. The most abundant BINs were BOLD:ACC0651 (Thripidae: *Taeniothrips inconsequens*), BOLD:AAD5253 (Chironomidae: *Thienemanniella xena*), and BOLD:AAP5920 (Chironomidae: *Cricotopus triannulatus*) with 349, 636, and 1619 specimens collected. For these three BINs, and many similarly abundant BINs, the presence of closely allied and morphologically similar taxa makes oversampling of these exceptionally common species unavoidable.

**Figure 2.**
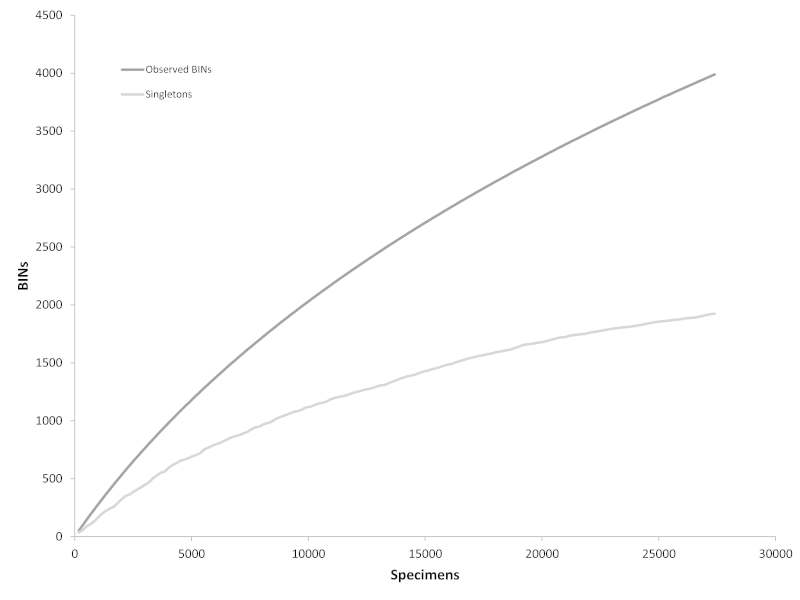
Accumulation curves for singleton and total observed BINs for the 2015 survey of ***rare* Charitable Research Reserve**.

​Combining all existing data results in a final tally of 3,348 species (Table 1), 1,102 of them new records for the reserve (Suppl. material 10). An incidence-based rarefaction analysis (Fig. 2 Suppl. material 11) computed in EstimateS (Colwell and Elsensohn 2014) approximates that 6,744 BINs of invertebrate animals are present at the reserve, indicating that our inventory is at most 30% complete. 1,918 BINs were singletons (i.e. represented by one specimen); the high proportion of singletons (48%) is another indication that the species inventory is far from complete (Magurran 2004​). For comparison, the Great Smoky Mountains National Park (North Carolina and Tennessee, United States) established an ATBI effort in 1998 and have tallied 18,545 of the estimated 100,000 species present in the park. In only a few months, our survey found 20% of the Great Smoky Mountains total from 17 years of effort. The Great Smoky Mountains National Park has recognized the contribution DNA barcoding can play in their ATBI effort and have included this method in their survey (e.g., Scholtens and Wagner 2007, Zhou et al. 2011).

## Discussion

The present study has conducted an expansive biotic survey, released the data in several public biodiversity data repositories, and published the unique process and findings – all within a relatively modest timeframe. This model for rapid generation and dissemination of critical biodiversity data could be followed to conduct regional assessments of biodiversity status and change, and potentially aid in evaluating progress towards the Aichi Targets of the Strategic Plan for Biodiversity 2011–2020. To fully appreciate this approach, a closer look at four elements is presented: highlights of the biotic inventory, the multiple levels of acceleration, future improvements to the resource, and the utility of the resource going forward.

### Resource Highlights

The biotic inventory of ***rare* Charitable Research Reserve** performed between May and August 2015 was noteworthy for a number of reasons. The taxonomic scope of the surveys, which covered fourteen phyla over several kingdoms, was only made possible by the integration of DNA barcoding and by assembling a diverse group of experts. Taxa that appeared under-represented on the prior species inventory were targeted where possible, and in many cases, increased significantly. Spiders (Araneae) for instance were completely absent from the prior inventory. Using the expertise of single specialist, focused collecting efforts, and a comprehensive barcode reference library for Canadian spiders (Blagoev et al. 2015) our survey resulted in the addition of 181 species (12.3% of Canadian fauna), two of which were newly recorded for BOLD (*Grammonota inornata*, *Philodromus praelustris*) and three representing new provincial records (*Cicurina pallida*, *Neoantistea gosiuta*, *Xysticus winnipegensis*) (Suppl. material 12​). Likewise, only 21 lichen species were reported from ***rare*** previously, but 53 were collected in a single day at the reserve, increasing the species list to 66. Not all the significant increases were in understudied taxa however: 18 snail and slug species (Mollusca) were added, all new to the reserve; 14 species of fishes were observed, adding three to the previous list of 28; and one mammal (hoary bat, *Lasiurus cinereus*) of the six observed were added to the list for a total of 37. In terms of BINs, many under-represented taxa did not witness a dramatic increase in named species, but did see large numbers of individual (and unnamed) BINs inventoried; the mites (Acari; 268 BINs) and gall midges (Cecidomyiidae; 584 BINs) are remarkable examples.

### Rapid Creation of the Resource

The rapidness of conducting this inventory was due to the acceleration of several steps that comprise the procedure. Firstly, several types of passive traps were employed to acquire large sample sizes for minimal collector effort. For instance, malaise traps often collect 2,000 specimens in a single week with only minutes of servicing time. Secondly, as discussed above, the addition of DNA barcoding streamlines sorting of the material, dividing the specimens into distinct units, minimizing the total number of specimens that require examination. Thirdly, in addition to the initial sorting, barcoding also provides nearly instant taxonomic assignment by querying against reference libraries using BOLD; the resolution of the assignment for queried records depends on the completeness of the reference library. Fourthly, the processing of the bioblitz material and analyses of the DNA barcode data were accelerated at all possible stages to test how quickly a large volume of specimens could proceed through all steps. The generation of data following the bioblitz was impressive: tissue sampling and lysis within 12 hours; DNA extraction and PCR within 24 h; cycle sequencing, cleanup and sequencer loading by 48 h; edited and validated sequences on BOLD by 72 h; taxonomic assignment, data release and manuscript presubmission by 96 h; final manuscript submission to BDJ by 108 h. The laboratory steps are mostly accelerated by automation, while data sharing is greatly facilitated by data release platforms such as BOLD and the Integrated Publishing Toolkit of Canadensys (Robertson et al. 2014), and finally, the manuscript stage is sped up considerably by the online writing tools introduced by Pensoft and other publishers.

### ​Refinement of the Resource

Many BINs are presently identified only to family, subfamily, or at most, genus level. It's important to note that the inventoried taxa that lack species-level determination, including intractable groups such as mites and midges, will be refined over time. Not only does DNA barcoding, empowered by the BIN system, roughly sort the material to direct and minimize the efforts of taxonomic experts (deWaard et al. 2009), but it also facilitates crowd-sourcing of taxonomic refinement. For example, if two specimens are collected in unrelated projects and locations, but assigned to the same BIN, any taxonomic determination on one specimen will be applicable to the other, and the pertinent data (such as identifier and locality) is shared on the public BIN page. For the specimens of this study, their taxonomy could be honed by the efforts of completely unrelated (but now linked) projects.

In addition to this passive approach to refining the taxonomy of these specimens, we will be actively pursuing expert determinations for the unnamed BINs. For many groups, the experts among our coauthors will be able to provide species level resolution in a short period of time (e.g., J.F.-T. specializes in Braconidae, particularly the subfamily Microgastrinae). For other BINs, and over a longer period of time, it will be necessary to solicit determinations from our network of collaborators that specialize on Nearctic taxa. The public release of these data to multiple data repositories should also aid in recruiting active specialists presently outside our network. The vouchered material, all deposited in a single repository (BIOUG), can be quickly assembled and loaned out, along with the associated data and files that may assist in determinations (e.g., tree files and image libraries: Suppl. materials 8, 9). There will however be taxa for which species names will be problematic, due to a lack of active specialists, keys, and/or revisionary work, and where a large proportion of that taxon awaits description. For example, the number of gall midges (Diptera: Cecidomyiidae) inventoried for rare (584) is nearly six times the number of Canadian species described to date (ca. 100, following Gagne and Jaschhof 2014).

When a significant number of species and other taxon categories become available, the refined dataset can be updated in the various data repositories and potentially in a second, updated version of the paper in BDJ. Any subsequent versions – each with a separate Digital Object Identifier (DOI) – would be linked to the original paper, allowing for continuing improvement and additions to the species list for the reserve. It is important to emphasize that the taxa awaiting species-level determination still have persistent identifiers as part of the BIN system; each BIN is assigned a BOLD-generated uniform resource identifier (URI) upon its establishment (e.g. BOLD:AAA0001). Until the species binomial is determined or described, this URI can be used in its place. For example, this URI permits assessing and comparing local diversity (Young et al. 2012) despite uncertainty in taxonomic placement.

### Utility of the Resource

While the overarching objective of the study was to develop, assess and demonstrate a model for conducting and disseminating DNA barcode-assisted biotic surveys, a valuable resource was created in the process. The ***rare* Charitable Research Reserve** provides a unique urban reserve with the infrastructure necessary to conduct research in its diversity of habitats; a barcode reference library and updated species inventory can now both be added to the infrastructure shared with their researchers and educators. Ecological studies in particular, such as the ongoing prairie community field experiments (e.g., Harvey and MacDougall 2014), could benefit greatly from this resource that allows the investigation of ecological questions previously impossible to address (Joly et al. 2014, Kress et al. 2015). Moreover, these studies and others are increasingly using next generation sequencing (NGS) technologies, where validated reference libraries linked to voucher specimens are of critical importance, since this link is generally lost in NGS studies (Cristescu 2014). This reference library created can also be amalgamated with external barcode records, local inventories, and taxon-specific efforts to create regional and national libraries; this study for instance has enhanced the reference library for animals by providing 468 unique BINs to BOLD. Finally, as mentioned above, it is crucial that we begin to assess biodiversity change and disseminate these data widely -- the resource created here can form the baseline for accessible, repeated assessments, to gauge trends in an important temperate reserve.​

## Supplementary Material

Supplementary material 1 Species inventory of rare prior to May 2015 Data type: checklist
Brief description: Species inventory for *rare* Charitable Research Reserve in Cambridge, Canada as of May 2015, prior to the present study.
File: oo_55088.xlsx Telfer et al.

Supplementary material 2 Bat protocols Data type: protocols
Brief description: Protocols for the bat component of *rare* BioBlitz
File: oo_54234.pdf Telfer et al.

Supplementary material 3 Lysis, primer and marker details Data type: laboratory
Brief description: Details for laboratory work.
File: oo_54237.xlsx Telfer et al.

Supplementary material 4 Summary data for 2015 inventory Data type: summary
Brief description: Summary data for the 28,789 specimens collected for the 2015 inventory, including BIN assignments and locality
File: oo_55064.xlsx Telfer et al.

Supplementary material 5 Darwin core archive Data type: occurrences
Brief description: Darwin core archive of 2015 *rare* species inventory
File: oo_55065.zip Telfer et al.

Supplementary material 6 Contributors list Data type: authors
Brief description: A list of all contributors who took part in the collection and identification of specimens collected as part of the 2015 inventory.
File: oo_54646.xls Telfer et al.

Supplementary material 7 Human observations during rare BioBlitz Data type: occurrences
Brief description: Human observations during *rare* BioBlitz, of mostly plants, birds, etc.
File: oo_55066.xlsx Telfer et al.

Supplementary material 8 BIN representative tree Data type: tree
Brief description: A neighbour-joining tree constructed from a single representative of each distinct BIN collected in *rare*, along with a single representative of clusters without BINs.
File: oo_55067.pdf Telfer et al.

Supplementary material 9 BIN image library Data type: images
Brief description: A collection of representative images of each BIN collected from the *rare* Charitable Research Reserve. BINs are listed in the same order as the tree. Specimens without BINs are not included.
File: oo_55068.pdf Telfer et al.

Supplementary material 10 Final combined inventory for rare Charitable Research Reserve Data type: checklist
Brief description: The final combined inventory for *rare* Charitable Research Reserve as of August 2015; including previous checklist and all 2015 species added.
File: oo_55089.xlsx Telfer et al.

Supplementary material 11 Raw data for accumulation curve Data type: occurences
Brief description: Lot and BIN data was downloaded for each specimen that received a BIN from BOLD Systems. It was formatted for input into EstimateS (Version 9.1.0) for creation of an accumulation curve.
File: oo_54647.xlsx Telfer et al.

Supplementary material 12 Sampling and new record images Data type: images
Brief description: Images of sampling sites, sampling techniques, specimen processing, and three new provincial spider reccords.
File: oo_54975.pdf Telfer et al.

## Figures and Tables

**Table 1.** Summary of species inventory for ***rare* Charitable Research Reserve** in Cambridge, Ontario, Canada, following the present study.

**Major Group**	**Common Name**	**No. on previous inventory**	**No. on 2015 surveys**	**New Species**	**New Total**
Annelida	Earthworms	0	2	2	2
Arthropoda:Arachnida	Spiders and others	0	198	198	198
Arthropoda:Crustacea	Crustaceans	0	7	7	7
Arthropoda:Entognatha	Collembola	0	9	9	9
Arthropoda:Insecta	Insects	832	895	778	1,610
Arthropoda:Myriapoda	Millipedes, Centipedes	0	6	6	6
Chordata:Actinopterygii	Fishes	28	14	3	31
Chordata:Amphibia	Amphibians	13	2	0	13
Chordata:Aves	Birds	231	87	0	231
Chordata:Mammalia	Mammals	37	6	1	38
Chordata:Reptilia	Reptiles	10	0	0	10
Fungi	Fungus, Lichens	191	84	60	251
Mollusca	Snails, Clams	0	18	18	18
Plantae	Plants, Mosses, Liverworts	901	103	20	921
Protozoa	Protozoans	3	3	0	3
	**Total Species**	2,246	1,433	1,102	3,348
