# Supplementary material for: Biodiversity inventories in high gear: DNA barcoding facilitates a rapid biotic survey of a temperate nature reserve
Source: Biodivers Data J. 2015 Aug 30;(3):e6313. doi: 10.3897/BDJ.3.e6313 (PMC4568406; doi:10.3897/BDJ.3.e6313)
Supplement: Supplementary material 2 — Bat protocols [file biodiversity_data_journal-3-e6313-s002.pdf]

rare BioBlitz – Bat Methods

Acoustic monitoring of bats was conducted to determine species presence and a measure of relative abundance at the rare Site. SM3BAT+ Bat Detectors were installed at two locations to monitor for bats along the Grand River from August 6 to 16, 2015. The locations of these acoustic bat monitors are described in Table 1. They were set to record nightly from 30 minutes before sunset to 30 minutes after sunrise. During this time, they were triggered to record when they detected a sound in the ultrasound frequency range of 15 – 120 kHz and would record a file of 5 – 15 seconds in duration. The data was filtered through Kaleidoscope®, then automated species classification was conducted through Sonobat® 3.2.1 NNE and the results were tallied into a nightly table.

Table 1: Locations of the acoustic bat monitoring stations

Station	Easting	Northing	Orientation	Description
Cliffs	553473	4802907	1m above cliff edge facing SW	Within a clearing at the top of the cliff. Cliff is very high at this location, so the detector is not near the water.
West End	553472	4802906	2m facing NE (downstream)	Located approximately 10m from the river at the edge of the woodland and near some short herbaceous vegetation.

On night of netting for bats was conducted as part of the BioBlitz on August 16, 2015 for 3 hours from approximately 21:00 to midnight. Bat nets (Avinet) were installed on poles in areas where bats were expected to be commuting to the water. The location of the netting stations is described in Table 2. These specialized mist nets are designed for the safe capture of bats which can be extracted easily. The netting arrangement included a triple-high 12 m net, a 9 m net and a 6 m net. To quantify netting effort, we used the calculation of **one, 6 m net for 1 hour = 1 net-hour.** Nets were checked on a frequency of 10 minutes or less.

The capture of and handling of specially protected wildlife (including bats) in Ontario was conducted under an approved Wildlife Scientific Collector Permit (WSCP) #1079564, with an Animal Care Protocol (ACP) #15-335, approved by the Ontario Wildlife Animal Care Committee (WACC). The project was also registered for activities concerning endangered species under ESA Registration #M-102-1771477948.

Table 2: Net locations established for bat capture

Net	Easting	Northing	Size	Description
1	553472	4802906	12 m wide X 8 m high	Clearing at the top of the cliff where trails intersect. Nets were approximately 5m from edge of cliff and parallel to it
2	553424	4802936	6 m raised	Within small clearing near edge of cliffs
3	553246	4803053	9 m	On a trail crossing a dry stream within the understory of mature deciduous forest

Results

The acoustic monitoring resulted in the identification of five species of bats (Table 3). Due to vandalism of the detector at the cliffs, it ceased to operate after August 3, 2015. The total bat passes at the site was considered to be very high, and is likely due to the use of the river by bats for feeding, commuting and possibly as a migratory pathway. The most frequently recorded bat was the Big Brown Bat (*Eptesicus fuscus*), followed by the Silver-haired Bat (*Lasionycteris noctivagans*) although there can be some discrepancy in the classification of call signatures between these two species. The endangered Little Brown Myotis (*Myotis lucifugus*) was recorded, but very infrequently. Only four passes of this species were recorded in total across 3 nights at the two stations.

The netting was conducted for a total of approximately 3 hours, which was calculated to equal 25.5 net-hours. During this time the weather was mild with nearly no wind and a clear sky. Only one bat was captured in the net, which was in Net 1. This bat was viewed as the net was lowered to extract the bat, but it escaped before it could be retrieved. From the view of the bat it was determined to be an Eastern Red Bat (*Lasiurus borealis*), although this species was rarely recorded on the acoustic detectors.

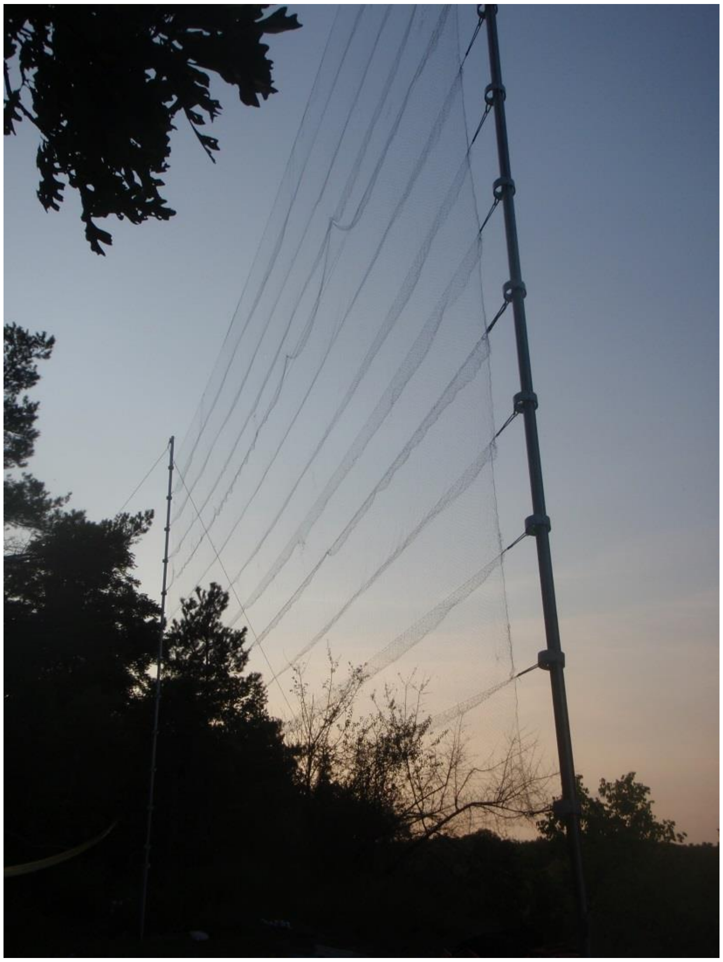

Figure 1: Triple-high 12 m net at the top of the cliffs at the rare Site

Table 3: Results of the acoustic bat monitoring at the rare Site

Date	Station	LowF Bat Unknown[1]	HiF Bat Unknown[2]	Hoary Bat	Silver-haired Bat	Big Brown Bat	Red Bat	Little Brown Myotis	Northern Myotis	Small-footed Myotis	Tricolored Bat	Total Bat Passes
6-Aug-15	Cliffs	65	0	17	57	165	0	0	0	0	0	304
7-Aug-15	Cliffs	90	0	10	45	213	3	1	0	0	0	362
8-Aug-15	Cliffs	100	0	7	51	240	0	0	0	0	0	398
9-Aug-15	Cliffs	116	0	17	35	341	1	1	0	0	0	511
10-Aug-15	Cliffs	7	0	0	2	77	0	0	0	0	0	86
11-Aug-15	Cliffs	199	0	17	101	704	1	0	0	0	0	1022
12-Aug-15	Cliffs	41	0	8	34	152	1	0	0	0	0	236
13-Aug-15	Cliffs	42	0	24	32	263	4	0	0	0	0	365
6-Aug-15	West End	135	0	41	88	248	2	0	0	0	0	514
7-Aug-15	West End	174	0	31	102	354	0	1	0	0	0	662
8-Aug-15	West End	127	0	22	41	182	1	0	0	0	0	373
9-Aug-15	West End	34	0	22	58	24	0	0	0	0	0	138
10-Aug-15	West End	30	0	11	31	14	1	0	0	0	0	87
11-Aug-15	West End	153	0	11	71	86	0	0	0	0	0	321
12-Aug-15	West End	5	0	6	12	4	0	0	0	0	0	27
13-Aug-15	West End	99	0	22	76	57	0	1	0	0	0	255
14-Aug-15	West End	24	0	4	20	24	0	0	0	0	0	72
15-Aug-15	West End	33	0	15	70	18	0	0	0	0	0	136

[1] - Recordings classified as bats with low frequency calls but could not be classified to the species level, typically including Hoary Bat, Big Brown Bat and Silver-haired Bat

[2] - Recordings classified as bats with high frequency calls but could not be classified to the species level, typically including Red Bats, Tricolored Bats and all bats in the Myotis genera
