# Supplementary material for: Biodiversity inventories in high gear: DNA barcoding facilitates a rapid biotic survey of a temperate nature reserve
Source: Biodivers Data J. 2015 Aug 30;(3):e6313. doi: 10.3897/BDJ.3.e6313 (PMC4568406; doi:10.3897/BDJ.3.e6313)
Supplement: Supplementary material 8 — BIN representative tree [file biodiversity_data_journal-3-e6313-s008.pdf]

BOLD TaxonID Tree

Title : Neighbour-Joining Tree for Representative Records from the rare BioBlitz

Data Type : Nucleotide

Distance Model : Kimura 2 Parameter

Marker : COI-5P

Sequence Count : 4312

Species count : 1118

Genus count : 1128

Family count : 379

Unidentified : 3074

BIN Count : 3332

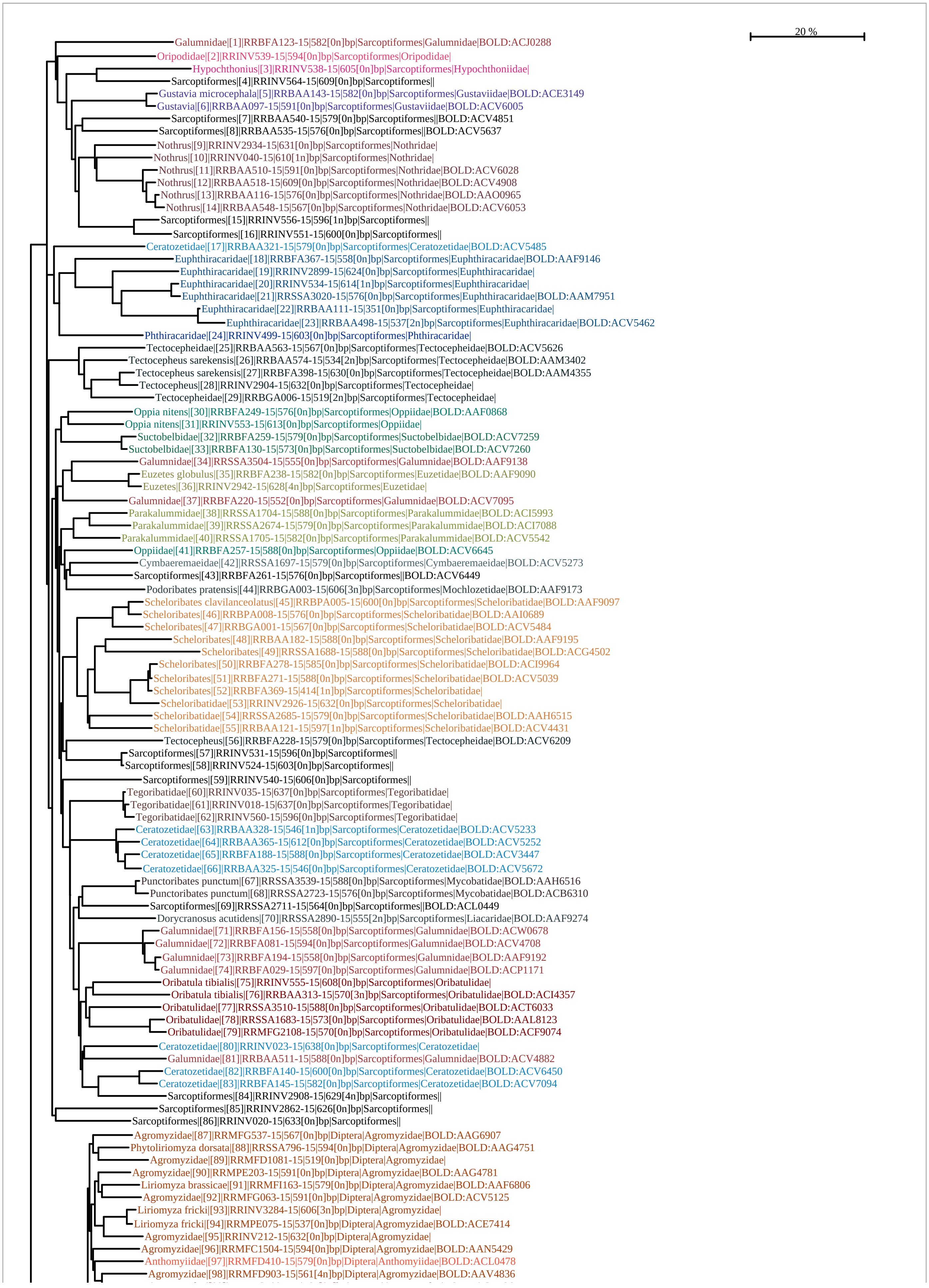
20 %
Galumnidae|[1]|RRBFA123-15|582[0n]bp|Sarcoptiformes|Galumnidae|BOLD:ACJ0288
Oripodidae|[2]|RRINV539-15|594[0n]bp|Sarcoptiformes|Oripodidae|
Hypochthonius|[3]|RRINV538-15|605[0n]bp|Sarcoptiformes|Hypochthoniidae|
Sarcoptiformes|[4]|RRINV564-15|609[0n]bp|Sarcoptiformes||
Gustavia microcephala|[5]|RRBAA143-15|582[0n]bp|Sarcoptiformes|Gustaviidae|BOLD:ACE3149
Gustavia|[6]|RRBAA097-15|591[0n]bp|Sarcoptiformes|Gustaviidae|BOLD:ACV6005
Sarcoptiformes|[7]|RRBAA540-15|579[0n]bp|Sarcoptiformes||BOLD:ACV4851
Sarcoptiformes|[8]|RRBAA535-15|576[0n]bp|Sarcoptiformes||BOLD:ACV5637
Nothrus|[9]|RRINV2934-15|631[0n]bp|Sarcoptiformes|Nothridae|
Nothrus|[10]|RRINV040-15|610[1n]bp|Sarcoptiformes|Nothridae|
Nothrus|[11]|RRBAA510-15|591[0n]bp|Sarcoptiformes|Nothridae|BOLD:ACV6028
Nothrus|[12]|RRBAA518-15|609[0n]bp|Sarcoptiformes|Nothridae|BOLD:ACV4908
Nothrus|[13]|RRBAA116-15|576[0n]bp|Sarcoptiformes|Nothridae|BOLD:AAO0965
Nothrus|[14]|RRBAA548-15|567[0n]bp|Sarcoptiformes|Nothridae|BOLD:ACV6053
Sarcoptiformes|[15]|RRINV556-15|596[1n]bp|Sarcoptiformes||
Sarcoptiformes|[16]|RRINV551-15|600[0n]bp|Sarcoptiformes||
Ceratozetidae|[17]|RRBAA321-15|579[0n]bp|Sarcoptiformes|Ceratozetidae|BOLD:ACV5485
Euphthiracaridae|[18]|RRBFA367-15|558[0n]bp|Sarcoptiformes|Euphthiracaridae|BOLD:AAF9146
Euphthiracaridae|[19]|RRINV2899-15|624[0n]bp|Sarcoptiformes|Euphthiracaridae|
Euphthiracaridae|[20]|RRINV534-15|614[1n]bp|Sarcoptiformes|Euphthiracaridae|
Euphthiracaridae|[21]|RRSSA3020-15|576[0n]bp|Sarcoptiformes|Euphthiracaridae|BOLD:AAM7951
Euphthiracaridae|[22]|RRBAA111-15|351[0n]bp|Sarcoptiformes|Euphthiracaridae|
Euphthiracaridae|[23]|RRBAA498-15|537[2n]bp|Sarcoptiformes|Euphthiracaridae|BOLD:ACV5462
Phthiracaridae|[24]|RRINV499-15|603[0n]bp|Sarcoptiformes|Phthiracaridae|
Tectocepheidae|[25]|RRBAA563-15|567[0n]bp|Sarcoptiformes|Tectocepheidae|BOLD:ACV5626
Tectocepheus sarekensis|[26]|RRBAA574-15|534[2n]bp|Sarcoptiformes|Tectocepheidae|BOLD:AAM3402
Tectocepheus sarekensis|[27]|RRBFA398-15|630[0n]bp|Sarcoptiformes|Tectocepheidae|BOLD:AAM4355
Tectocepheus|[28]|RRINV2904-15|632[0n]bp|Sarcoptiformes|Tectocepheidae|
Tectocepheidae|[29]|RRBGA006-15|519[2n]bp|Sarcoptiformes|Tectocepheidae|
Oppia nitens|[30]|RRBFA249-15|576[0n]bp|Sarcoptiformes|Oppiidae|BOLD:AAF0868
Oppia nitens|[31]|RRINV553-15|613[0n]bp|Sarcoptiformes|Oppiidae|
Suctobelbidae|[32]|RRBFA259-15|579[0n]bp|Sarcoptiformes|Suctobelbidae|BOLD:ACV7259
Suctobelbidae|[33]|RRBFA130-15|573[0n]bp|Sarcoptiformes|Suctobelbidae|BOLD:ACV7260
Galumnidae|[34]|RRSSA3504-15|555[0n]bp|Sarcoptiformes|Galumnidae|BOLD:AAF9138
Euzetes globulus|[35]|RRBFA238-15|582[0n]bp|Sarcoptiformes|Euzetidae|BOLD:AAF9090
Euzetes|[36]|RRINV2942-15|628[4n]bp|Sarcoptiformes|Euzetidae|
Galumnidae|[37]|RRBFA220-15|552[0n]bp|Sarcoptiformes|Galumnidae|BOLD:ACV7095
Parakalummidae|[38]|RRSSA1704-15|588[0n]bp|Sarcoptiformes|Parakalummidae|BOLD:ACI5993
Parakalummidae|[39]|RRSSA2674-15|579[0n]bp|Sarcoptiformes|Parakalummidae|BOLD:ACI7088
Parakalummidae|[40]|RRSSA1705-15|582[0n]bp|Sarcoptiformes|Parakalummidae|BOLD:ACV5542
Oppiidae|[41]|RRBFA257-15|588[0n]bp|Sarcoptiformes|Oppiidae|BOLD:ACV6645
Cymbaeremaeidae|[42]|RRSSA1697-15|579[0n]bp|Sarcoptiformes|Cymbaeremaeidae|BOLD:ACV5273
Sarcoptiformes|[43]|RRBFA261-15|576[0n]bp|Sarcoptiformes||BOLD:ACV6449
Podoribates pratensis|[44]|RRBGA003-15|606[3n]bp|Sarcoptiformes|Mochlozetidae|BOLD:AAF9173
Scheloribates clavilanceolatus|[45]|RRBPA005-15|600[0n]bp|Sarcoptiformes|Scheloribatidae|BOLD:AAF9097
Scheloribates|[46]|RRBPA008-15|576[0n]bp|Sarcoptiformes|Scheloribatidae|BOLD:AAI0689
Scheloribates|[47]|RRBGA001-15|567[0n]bp|Sarcoptiformes|Scheloribatidae|BOLD:ACV5484
Scheloribates|[48]|RRBAA182-15|588[0n]bp|Sarcoptiformes|Scheloribatidae|BOLD:AAF9195
Scheloribates|[49]|RRSSA1688-15|588[0n]bp|Sarcoptiformes|Scheloribatidae|BOLD:ACG4502
Scheloribates|[50]|RRBFA278-15|585[0n]bp|Sarcoptiformes|Scheloribatidae|BOLD:ACI9964
Scheloribates|[51]|RRBFA271-15|588[0n]bp|Sarcoptiformes|Scheloribatidae|BOLD:ACV5039
Scheloribates|[52]|RRBFA369-15|414[1n]bp|Sarcoptiformes|Scheloribatidae|
Scheloribatidae|[53]|RRINV2926-15|632[0n]bp|Sarcoptiformes|Scheloribatidae|
Scheloribatidae|[54]|RRSSA2685-15|579[0n]bp|Sarcoptiformes|Scheloribatidae|BOLD:AAH6515
Scheloribatidae|[55]|RRBAA121-15|597[1n]bp|Sarcoptiformes|Scheloribatidae|BOLD:ACV4431
Tectocepheus|[56]|RRBFA228-15|579[0n]bp|Sarcoptiformes|Tectocepheidae|BOLD:ACV6209
Sarcoptiformes|[57]|RRINV531-15|596[0n]bp|Sarcoptiformes||
Sarcoptiformes|[58]|RRINV524-15|603[0n]bp|Sarcoptiformes||
Sarcoptiformes|[59]|RRINV540-15|606[0n]bp|Sarcoptiformes||
Tegoribatidae|[60]|RRINV035-15|637[0n]bp|Sarcoptiformes|Tegoribatidae|
Tegoribatidae|[61]|RRINV018-15|637[0n]bp|Sarcoptiformes|Tegoribatidae|
Tegoribatidae|[62]|RRINV560-15|596[0n]bp|Sarcoptiformes|Tegoribatidae|
Ceratozetidae|[63]|RRBAA328-15|546[1n]bp|Sarcoptiformes|Ceratozetidae|BOLD:ACV5233
Ceratozetidae|[64]|RRBAA365-15|612[0n]bp|Sarcoptiformes|Ceratozetidae|BOLD:ACV5252
Ceratozetidae|[65]|RRBFA188-15|588[0n]bp|Sarcoptiformes|Ceratozetidae|BOLD:ACV3447
Ceratozetidae|[66]|RRBAA325-15|546[0n]bp|Sarcoptiformes|Ceratozetidae|BOLD:ACV5672
Punctoribates punctum|[67]|RRSSA3539-15|588[0n]bp|Sarcoptiformes|Mycobatidae|BOLD:AAH6516
Punctoribates punctum|[68]|RRSSA2723-15|576[0n]bp|Sarcoptiformes|Mycobatidae|BOLD:ACB6310
Sarcoptiformes|[69]|RRSSA2711-15|564[0n]bp|Sarcoptiformes||BOLD:ACL0449
Dorycranosus acutidens|[70]|RRSSA2890-15|555[2n]bp|Sarcoptiformes|Liacaridae|BOLD:AAF9274
Galumnidae|[71]|RRBFA156-15|558[0n]bp|Sarcoptiformes|Galumnidae|BOLD:ACW0678
Galumnidae|[72]|RRBFA081-15|594[0n]bp|Sarcoptiformes|Galumnidae|BOLD:ACV4708
Galumnidae|[73]|RRBFA194-15|558[0n]bp|Sarcoptiformes|Galumnidae|BOLD:AAF9192
Galumnidae|[74]|RRBFA029-15|597[0n]bp|Sarcoptiformes|Galumnidae|BOLD:ACP1171
Oribatula tibialis|[75]|RRINV555-15|608[0n]bp|Sarcoptiformes|Oribatulidae|
Oribatula tibialis|[76]|RRBAA313-15|570[3n]bp|Sarcoptiformes|Oribatulidae|BOLD:ACI4357
Oribatulidae|[77]|RRSSA3510-15|588[0n]bp|Sarcoptiformes|Oribatulidae|BOLD:ACT6033
Oribatulidae|[78]|RRSSA1683-15|573[0n]bp|Sarcoptiformes|Oribatulidae|BOLD:AAL8123
Oribatulidae|[79]|RRMFG2108-15|570[0n]bp|Sarcoptiformes|Oribatulidae|BOLD:ACF9074
Ceratozetidae|[80]|RRINV023-15|638[0n]bp|Sarcoptiformes|Ceratozetidae|
Galumnidae|[81]|RRBAA511-15|588[0n]bp|Sarcoptiformes|Galumnidae|BOLD:ACV4882
Ceratozetidae|[82]|RRBFA140-15|600[0n]bp|Sarcoptiformes|Ceratozetidae|BOLD:ACV6450
Ceratozetidae|[83]|RRBFA145-15|582[0n]bp|Sarcoptiformes|Ceratozetidae|BOLD:ACV7094
Sarcoptiformes|[84]|RRINV2908-15|629[4n]bp|Sarcoptiformes||
Sarcoptiformes|[85]|RRINV2862-15|626[0n]bp|Sarcoptiformes||
Sarcoptiformes|[86]|RRINV020-15|633[0n]bp|Sarcoptiformes||
Agromyzidae|[87]|RRMFG537-15|567[0n]bp|Diptera|Agromyzidae|BOLD:AAG6907
Phytoliriomyza dorsata|[88]|RRSSA796-15|594[0n]bp|Diptera|Agromyzidae|BOLD:AAG4751
Agromyzidae|[89]|RRMFD1081-15|519[0n]bp|Diptera|Agromyzidae|
Agromyzidae|[90]|RRMPE203-15|591[0n]bp|Diptera|Agromyzidae|BOLD:AAG4781
Liriomyza brassicae|[91]|RRMFI163-15|579[0n]bp|Diptera|Agromyzidae|BOLD:AAF6806
Agromyzidae|[92]|RRMFG063-15|591[0n]bp|Diptera|Agromyzidae|BOLD:ACV5125
Liriomyza fricki|[93]|RRINV3284-15|606[3n]bp|Diptera|Agromyzidae|
Liriomyza fricki|[94]|RRMPE075-15|537[0n]bp|Diptera|Agromyzidae|BOLD:ACE7414
Agromyzidae|[95]|RRINV212-15|632[0n]bp|Diptera|Agromyzidae|
Agromyzidae|[96]|RRMFC1504-15|594[0n]bp|Diptera|Agromyzidae|BOLD:AAN5429
Anthomyiidae|[97]|RRMFD410-15|579[0n]bp|Diptera|Anthomyiidae|BOLD:ACL0478
Agromyzidae|[98]|RRMFD903-15|561[4n]bp|Diptera|Agromyzidae|BOLD:AAV4836

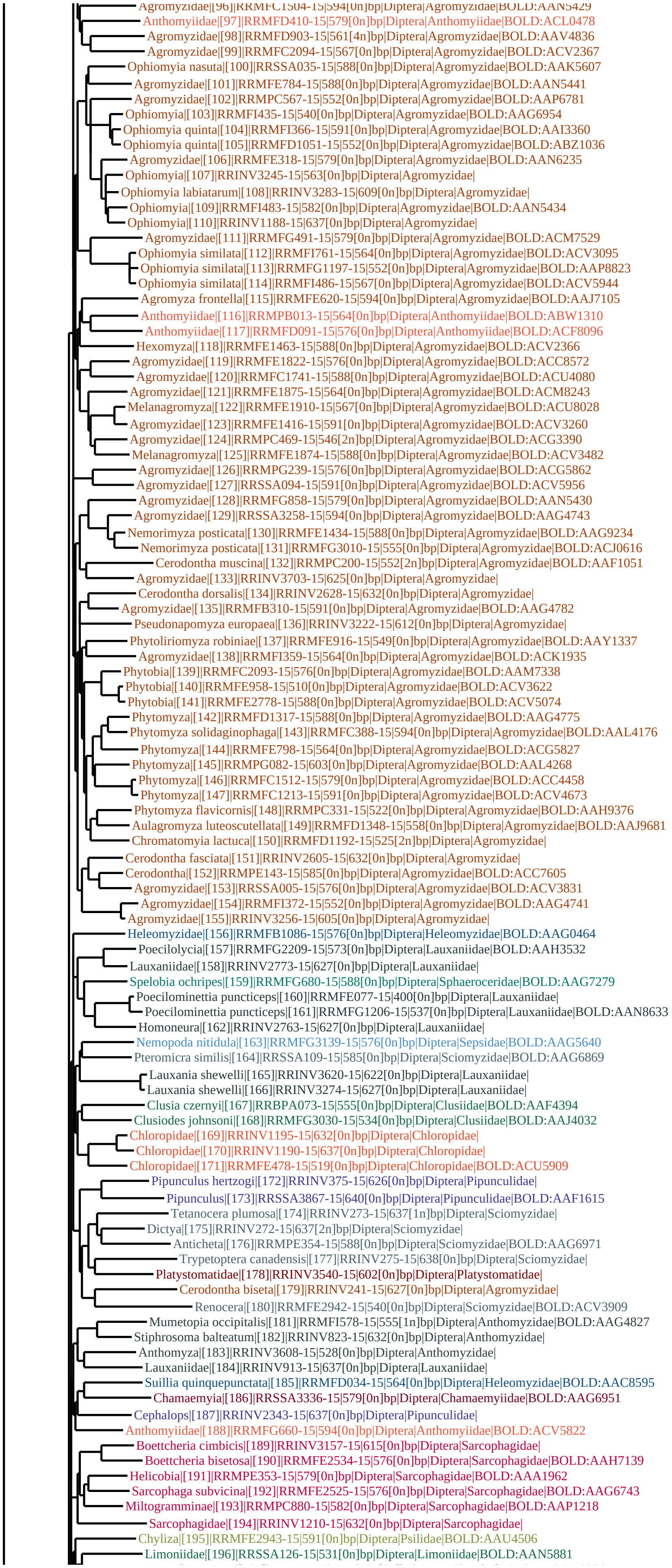
Agromyzidae|[96]|RRMFC1504-15|594[0n]bp|Diptera|Agromyzidae|BOLD:AAN5429
Anthomyiidae|[97]|RRMFD410-15|579[0n]bp|Diptera|Anthomyiidae|BOLD:ACL0478
Agromyzidae|[98]|RRMFD903-15|561[4n]bp|Diptera|Agromyzidae|BOLD:AAV4836
Agromyzidae|[99]|RRMFC2094-15|567[0n]bp|Diptera|Agromyzidae|BOLD:ACV2367
Ophiomyia nasuta|[100]|RRSSA035-15|588[0n]bp|Diptera|Agromyzidae|BOLD:AAK5607
Agromyzidae|[101]|RRMFE784-15|588[0n]bp|Diptera|Agromyzidae|BOLD:AAN5441
Agromyzidae|[102]|RRMPC567-15|552[0n]bp|Diptera|Agromyzidae|BOLD:AAP6781
Ophiomyia|[103]|RRMFI435-15|540[0n]bp|Diptera|Agromyzidae|BOLD:AAG6954
Ophiomyia quinta|[104]|RRMFI366-15|591[0n]bp|Diptera|Agromyzidae|BOLD:AAI3360
Ophiomyia quinta|[105]|RRMFD1051-15|552[0n]bp|Diptera|Agromyzidae|BOLD:ABZ1036
Agromyzidae|[106]|RRMFE318-15|579[0n]bp|Diptera|Agromyzidae|BOLD:AAN6235
Ophiomyia|[107]|RRINV3245-15|563[0n]bp|Diptera|Agromyzidae|
Ophiomyia labiatarum|[108]|RRINV3283-15|609[0n]bp|Diptera|Agromyzidae|
Ophiomyia|[109]|RRMFI483-15|582[0n]bp|Diptera|Agromyzidae|BOLD:AAN5434
Ophiomyia|[110]|RRINV1188-15|637[0n]bp|Diptera|Agromyzidae|
Agromyzidae|[111]|RRMFG491-15|579[0n]bp|Diptera|Agromyzidae|BOLD:ACM7529
Ophiomyia similata|[112]|RRMFI761-15|564[0n]bp|Diptera|Agromyzidae|BOLD:ACV3095
Ophiomyia similata|[113]|RRMFG1197-15|552[0n]bp|Diptera|Agromyzidae|BOLD:AAP8823
Ophiomyia similata|[114]|RRMFI486-15|567[0n]bp|Diptera|Agromyzidae|BOLD:ACV5944
Agromyza frontella|[115]|RRMFE620-15|594[0n]bp|Diptera|Agromyzidae|BOLD:AAJ7105
Anthomyiidae|[116]|RRMPB013-15|564[0n]bp|Diptera|Anthomyiidae|BOLD:ABW1310
Anthomyiidae|[117]|RRMFD091-15|576[0n]bp|Diptera|Anthomyiidae|BOLD:ACF8096
Hexomyza|[118]|RRMFE1463-15|588[0n]bp|Diptera|Agromyzidae|BOLD:ACV2366
Agromyzidae|[119]|RRMFE1822-15|576[0n]bp|Diptera|Agromyzidae|BOLD:ACC8572
Agromyzidae|[120]|RRMFC1741-15|588[0n]bp|Diptera|Agromyzidae|BOLD:ACU4080
Agromyzidae|[121]|RRMFE1875-15|564[0n]bp|Diptera|Agromyzidae|BOLD:ACM8243
Melanagromyza|[122]|RRMFE1910-15|567[0n]bp|Diptera|Agromyzidae|BOLD:ACU8028
Agromyzidae|[123]|RRMFE1416-15|591[0n]bp|Diptera|Agromyzidae|BOLD:ACV3260
Agromyzidae|[124]|RRMPC469-15|546[2n]bp|Diptera|Agromyzidae|BOLD:ACG3390
Melanagromyza|[125]|RRMFE1874-15|588[0n]bp|Diptera|Agromyzidae|BOLD:ACV3482
Agromyzidae|[126]|RRMPG239-15|576[0n]bp|Diptera|Agromyzidae|BOLD:ACG5862
Agromyzidae|[127]|RRSSA094-15|591[0n]bp|Diptera|Agromyzidae|BOLD:ACV5956
Agromyzidae|[128]|RRMFG858-15|579[0n]bp|Diptera|Agromyzidae|BOLD:AAN5430
Agromyzidae|[129]|RRSSA3258-15|594[0n]bp|Diptera|Agromyzidae|BOLD:AAG4743
Nemorimyza posticata|[130]|RRMFE1434-15|588[0n]bp|Diptera|Agromyzidae|BOLD:AAG9234
Nemorimyza posticata|[131]|RRMFG3010-15|555[0n]bp|Diptera|Agromyzidae|BOLD:ACJ0616
Cerodontha muscina|[132]|RRMPC200-15|552[2n]bp|Diptera|Agromyzidae|BOLD:AAF1051
Agromyzidae|[133]|RRINV3703-15|625[0n]bp|Diptera|Agromyzidae|
Cerodontha dorsalis|[134]|RRINV2628-15|632[0n]bp|Diptera|Agromyzidae|
Agromyzidae|[135]|RRMFB310-15|591[0n]bp|Diptera|Agromyzidae|BOLD:AAG4782
Pseudonapomyza europaea|[136]|RRINV3222-15|612[0n]bp|Diptera|Agromyzidae|
Phytoliriomyza robiniae|[137]|RRMFE916-15|549[0n]bp|Diptera|Agromyzidae|BOLD:AAY1337
Agromyzidae|[138]|RRMFI359-15|564[0n]bp|Diptera|Agromyzidae|BOLD:ACK1935
Phytobia|[139]|RRMFC2093-15|576[0n]bp|Diptera|Agromyzidae|BOLD:AAM7338
Phytobia|[140]|RRMFE958-15|510[0n]bp|Diptera|Agromyzidae|BOLD:ACV3622
Phytobia|[141]|RRMFE2778-15|588[0n]bp|Diptera|Agromyzidae|BOLD:ACV5074
Phytomyza|[142]|RRMFD1317-15|588[0n]bp|Diptera|Agromyzidae|BOLD:AAG4775
Phytomyza solidaginophaga|[143]|RRMFC388-15|594[0n]bp|Diptera|Agromyzidae|BOLD:AAL4176
Phytomyza|[144]|RRMFE798-15|564[0n]bp|Diptera|Agromyzidae|BOLD:ACG5827
Phytomyza|[145]|RRMPG082-15|603[0n]bp|Diptera|Agromyzidae|BOLD:AAL4268
Phytomyza|[146]|RRMFC1512-15|579[0n]bp|Diptera|Agromyzidae|BOLD:ACC4458
Phytomyza|[147]|RRMFC1213-15|591[0n]bp|Diptera|Agromyzidae|BOLD:ACV4673
Phytomyza flavicornis|[148]|RRMPC331-15|522[0n]bp|Diptera|Agromyzidae|BOLD:AAH9376
Aulagromyza luteoscutellata|[149]|RRMFD1348-15|558[0n]bp|Diptera|Agromyzidae|BOLD:AAJ9681
Chromatomyia lactuca|[150]|RRMFD1192-15|525[2n]bp|Diptera|Agromyzidae|
Cerodontha fasciata|[151]|RRINV2605-15|632[0n]bp|Diptera|Agromyzidae|
Cerodontha|[152]|RRMPE143-15|585[0n]bp|Diptera|Agromyzidae|BOLD:ACC7605
Agromyzidae|[153]|RRSSA005-15|576[0n]bp|Diptera|Agromyzidae|BOLD:ACV3831
Agromyzidae|[154]|RRMFI372-15|552[0n]bp|Diptera|Agromyzidae|BOLD:AAG4741
Agromyzidae|[155]|RRINV3256-15|605[0n]bp|Diptera|Agromyzidae|
Heleomyzidae|[156]|RRMFB1086-15|576[0n]bp|Diptera|Heleomyzidae|BOLD:AAG0464
Poecilolycia|[157]|RRMFG2209-15|573[0n]bp|Diptera|Lauxaniidae|BOLD:AAH3532
Lauxaniidae|[158]|RRINV2773-15|627[0n]bp|Diptera|Lauxaniidae|
Spelobia ochripes|[159]|RRMFG680-15|588[0n]bp|Diptera|Sphaeroceridae|BOLD:AAG7279
Poecilominettia puncticeps|[160]|RRMFE077-15|400[0n]bp|Diptera|Lauxaniidae|
Poecilominettia puncticeps|[161]|RRMFG1206-15|537[0n]bp|Diptera|Lauxaniidae|BOLD:AAN8633
Homoneura|[162]|RRINV2763-15|627[0n]bp|Diptera|Lauxaniidae|
Nemopoda nitidula|[163]|RRMFG3139-15|576[0n]bp|Diptera|Sepsidae|BOLD:AAG5640
Pteromicra similis|[164]|RRSSA109-15|585[0n]bp|Diptera|Sciomyzidae|BOLD:AAG6869
Lauxania shewelli|[165]|RRINV3620-15|622[0n]bp|Diptera|Lauxaniidae|
Lauxania shewelli|[166]|RRINV3274-15|627[0n]bp|Diptera|Lauxaniidae|
Clusia czernyi|[167]|RRBPA073-15|555[0n]bp|Diptera|Clusiidae|BOLD:AAF4394
Clusiodes johnsoni|[168]|RRMFG3030-15|534[0n]bp|Diptera|Clusiidae|BOLD:AAJ4032
Chloropidae|[169]|RRINV1195-15|632[0n]bp|Diptera|Chloropidae|
Chloropidae|[170]|RRINV1190-15|637[0n]bp|Diptera|Chloropidae|
Chloropidae|[171]|RRMFE478-15|519[0n]bp|Diptera|Chloropidae|BOLD:ACU5909
Pipunculus hertzogi|[172]|RRINV375-15|626[0n]bp|Diptera|Pipunculidae|
Pipunculus|[173]|RRSSA3867-15|640[0n]bp|Diptera|Pipunculidae|BOLD:AAF1615
Tetanocera plumosa|[174]|RRINV273-15|637[1n]bp|Diptera|Sciomyzidae|
Dictya|[175]|RRINV272-15|637[2n]bp|Diptera|Sciomyzidae|
Anticheta|[176]|RRMPE354-15|588[0n]bp|Diptera|Sciomyzidae|BOLD:AAG6971
Trypetoptera canadensis|[177]|RRINV275-15|638[0n]bp|Diptera|Sciomyzidae|
Platystomatidae|[178]|RRINV3540-15|602[0n]bp|Diptera|Platystomatidae|
Cerodontha biseta|[179]|RRINV241-15|627[0n]bp|Diptera|Agromyzidae|
Renocera|[180]|RRMFE2942-15|540[0n]bp|Diptera|Sciomyzidae|BOLD:ACV3909
Mumetopia occipitalis|[181]|RRMFI578-15|555[1n]bp|Diptera|Anthomyzidae|BOLD:AAG4827
Stiphrosoma balteatum|[182]|RRINV823-15|632[0n]bp|Diptera|Anthomyzidae|
Anthomyza|[183]|RRINV3608-15|528[0n]bp|Diptera|Anthomyzidae|
Lauxaniidae|[184]|RRINV913-15|637[0n]bp|Diptera|Lauxaniidae|
Suillia quinquepunctata|[185]|RRMFD034-15|564[0n]bp|Diptera|Heleomyzidae|BOLD:AAC8595
Chamaemyia|[186]|RRSSA3336-15|579[0n]bp|Diptera|Chamaemyiidae|BOLD:AAG6951
Cephalops|[187]|RRINV2343-15|637[0n]bp|Diptera|Pipunculidae|
Anthomyiidae|[188]|RRMFG660-15|594[0n]bp|Diptera|Anthomyiidae|BOLD:ACV5822
Boettcheria cimbicis|[189]|RRINV3157-15|615[0n]bp|Diptera|Sarcophagidae|
Boettcheria bisetosa|[190]|RRMFE2534-15|576[0n]bp|Diptera|Sarcophagidae|BOLD:AAH7139
Helicobia|[191]|RRMPE353-15|579[0n]bp|Diptera|Sarcophagidae|BOLD:AAA1962
Sarcophaga subvicina|[192]|RRMFE2525-15|576[0n]bp|Diptera|Sarcophagidae|BOLD:AAG6743
Miltogramminae|[193]|RRMPC880-15|582[0n]bp|Diptera|Sarcophagidae|BOLD:AAP1218
Sarcophagidae|[194]|RRINV1210-15|632[0n]bp|Diptera|Sarcophagidae|
Chyliza|[195]|RRMFE2943-15|591[0n]bp|Diptera|Psilidae|BOLD:AAU4506
Limoniidae|[196]|RRSSA126-15|531[0n]bp|Diptera|Limoniidae|BOLD:AAN5881

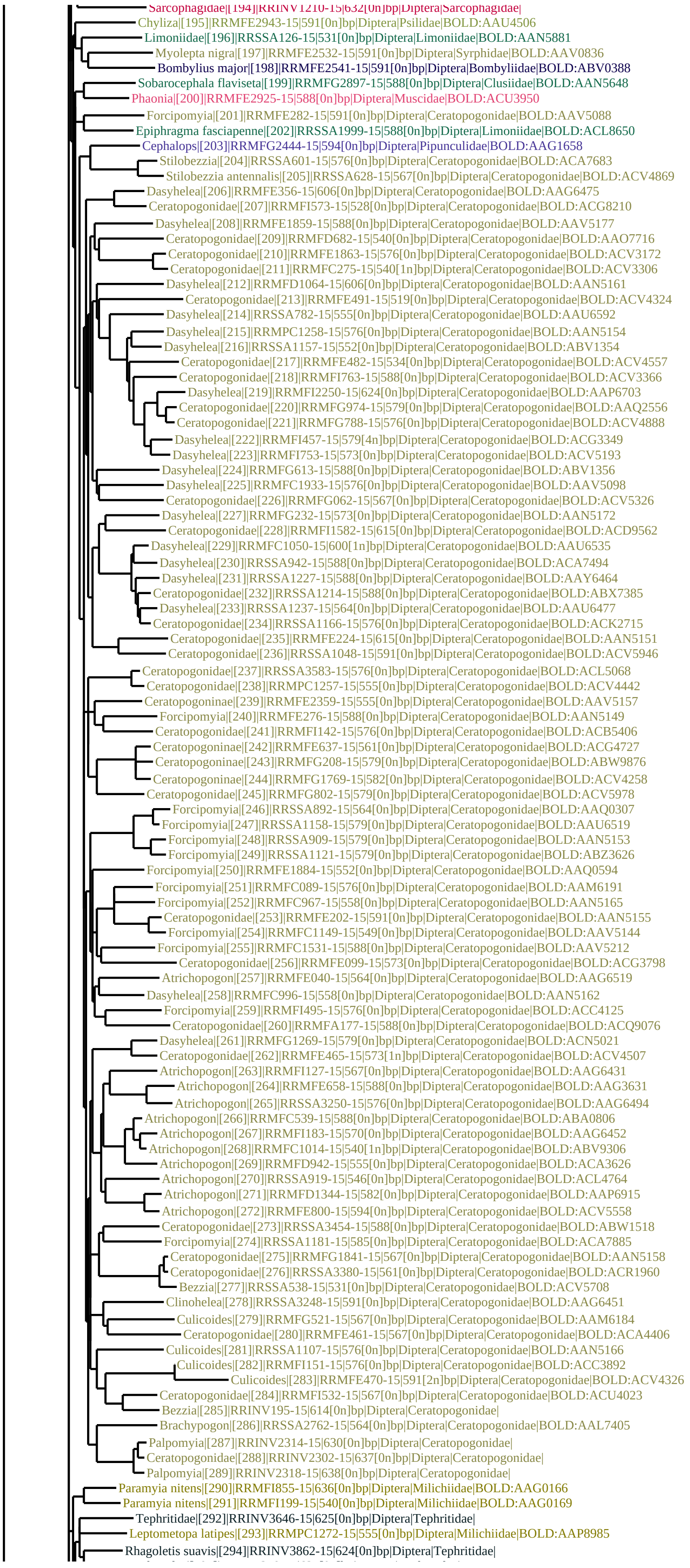
Sarcophagidae|[194]|RRINV1210-15|632[0n]bp|Diptera|Sarcophagidae|
Chyliza|[195]|RRMFE2943-15|591[0n]bp|Diptera|Psilidae|BOLD:AAU4506
Limoniidae|[196]|RRSSA126-15|531[0n]bp|Diptera|Limoniidae|BOLD:AAN5881
Myolepta nigra|[197]|RRMFE2532-15|591[0n]bp|Diptera|Syrphidae|BOLD:AAV0836
Bombylius major|[198]|RRMFE2541-15|591[0n]bp|Diptera|Bombyliidae|BOLD:ABV0388
Sobarocephala flaviseta|[199]|RRMFG2897-15|588[0n]bp|Diptera|Clusiidae|BOLD:AAN5648
Phaonia|[200]|RRMFE2925-15|588[0n]bp|Diptera|Muscidae|BOLD:ACU3950
Forcipomyia|[201]|RRMFE282-15|591[0n]bp|Diptera|Ceratopogonidae|BOLD:AAV5088
Epiphragma fasciapenne|[202]|RRSSA1999-15|588[0n]bp|Diptera|Limoniidae|BOLD:ACL8650
Cephalops|[203]|RRMFG2444-15|594[0n]bp|Diptera|Pipunculidae|BOLD:AAG1658
Stilobezzia|[204]|RRSSA601-15|576[0n]bp|Diptera|Ceratopogonidae|BOLD:ACA7683
Stilobezzia antennalis|[205]|RRSSA628-15|567[0n]bp|Diptera|Ceratopogonidae|BOLD:ACV4869
Dasyhelea|[206]|RRMFE356-15|606[0n]bp|Diptera|Ceratopogonidae|BOLD:AAG6475
Ceratopogonidae|[207]|RRMFI573-15|528[0n]bp|Diptera|Ceratopogonidae|BOLD:ACG8210
Dasyhelea|[208]|RRMFE1859-15|588[0n]bp|Diptera|Ceratopogonidae|BOLD:AAV5177
Ceratopogonidae|[209]|RRMFD682-15|540[0n]bp|Diptera|Ceratopogonidae|BOLD:AAO7716
Ceratopogonidae|[210]|RRMFE1863-15|576[0n]bp|Diptera|Ceratopogonidae|BOLD:ACV3172
Ceratopogonidae|[211]|RRMFC275-15|540[1n]bp|Diptera|Ceratopogonidae|BOLD:ACV3306
Dasyhelea|[212]|RRMFD1064-15|606[0n]bp|Diptera|Ceratopogonidae|BOLD:AAN5161
Ceratopogonidae|[213]|RRMFE491-15|519[0n]bp|Diptera|Ceratopogonidae|BOLD:ACV4324
Dasyhelea|[214]|RRSSA782-15|555[0n]bp|Diptera|Ceratopogonidae|BOLD:AAU6592
Dasyhelea|[215]|RRMPC1258-15|576[0n]bp|Diptera|Ceratopogonidae|BOLD:AAN5154
Dasyhelea|[216]|RRSSA1157-15|552[0n]bp|Diptera|Ceratopogonidae|BOLD:ABV1354
Ceratopogonidae|[217]|RRMFE482-15|534[0n]bp|Diptera|Ceratopogonidae|BOLD:ACV4557
Ceratopogonidae|[218]|RRMFI763-15|588[0n]bp|Diptera|Ceratopogonidae|BOLD:ACV3366
Dasyhelea|[219]|RRMFI2250-15|624[0n]bp|Diptera|Ceratopogonidae|BOLD:AAP6703
Ceratopogonidae|[220]|RRMFG974-15|579[0n]bp|Diptera|Ceratopogonidae|BOLD:AAQ2556
Ceratopogonidae|[221]|RRMFG788-15|576[0n]bp|Diptera|Ceratopogonidae|BOLD:ACV4888
Dasyhelea|[222]|RRMFI457-15|579[4n]bp|Diptera|Ceratopogonidae|BOLD:ACG3349
Dasyhelea|[223]|RRMFI753-15|573[0n]bp|Diptera|Ceratopogonidae|BOLD:ACV5193
Dasyhelea|[224]|RRMFG613-15|588[0n]bp|Diptera|Ceratopogonidae|BOLD:ABV1356
Dasyhelea|[225]|RRMFC1933-15|576[0n]bp|Diptera|Ceratopogonidae|BOLD:AAV5098
Ceratopogonidae|[226]|RRMFG062-15|567[0n]bp|Diptera|Ceratopogonidae|BOLD:ACV5326
Dasyhelea|[227]|RRMFG232-15|573[0n]bp|Diptera|Ceratopogonidae|BOLD:AAN5172
Ceratopogonidae|[228]|RRMFI1582-15|615[0n]bp|Diptera|Ceratopogonidae|BOLD:ACD9562
Dasyhelea|[229]|RRMFC1050-15|600[1n]bp|Diptera|Ceratopogonidae|BOLD:AAU6535
Dasyhelea|[230]|RRSSA942-15|588[0n]bp|Diptera|Ceratopogonidae|BOLD:ACA7494
Dasyhelea|[231]|RRSSA1227-15|588[0n]bp|Diptera|Ceratopogonidae|BOLD:AAY6464
Ceratopogonidae|[232]|RRSSA1214-15|588[0n]bp|Diptera|Ceratopogonidae|BOLD:ABX7385
Dasyhelea|[233]|RRSSA1237-15|564[0n]bp|Diptera|Ceratopogonidae|BOLD:AAU6477
Ceratopogonidae|[234]|RRSSA1166-15|576[0n]bp|Diptera|Ceratopogonidae|BOLD:ACK2715
Ceratopogonidae|[235]|RRMFE224-15|615[0n]bp|Diptera|Ceratopogonidae|BOLD:AAN5151
Ceratopogonidae|[236]|RRSSA1048-15|591[0n]bp|Diptera|Ceratopogonidae|BOLD:ACV5946
Ceratopogonidae|[237]|RRSSA3583-15|576[0n]bp|Diptera|Ceratopogonidae|BOLD:ACL5068
Ceratopogonidae|[238]|RRMPC1257-15|555[0n]bp|Diptera|Ceratopogonidae|BOLD:ACV4442
Ceratopogoninae|[239]|RRMFE2359-15|555[0n]bp|Diptera|Ceratopogonidae|BOLD:AAV5157
Forcipomyia|[240]|RRMFE276-15|588[0n]bp|Diptera|Ceratopogonidae|BOLD:AAN5149
Ceratopogonidae|[241]|RRMFI142-15|576[0n]bp|Diptera|Ceratopogonidae|BOLD:ACB5406
Ceratopogoninae|[242]|RRMFE637-15|561[0n]bp|Diptera|Ceratopogonidae|BOLD:ACG4727
Ceratopogoninae|[243]|RRMFG208-15|579[0n]bp|Diptera|Ceratopogonidae|BOLD:ABW9876
Ceratopogoninae|[244]|RRMFG1769-15|582[0n]bp|Diptera|Ceratopogonidae|BOLD:ACV4258
Ceratopogonidae|[245]|RRMFG802-15|579[0n]bp|Diptera|Ceratopogonidae|BOLD:ACV5978
Forcipomyia|[246]|RRSSA892-15|564[0n]bp|Diptera|Ceratopogonidae|BOLD:AAQ0307
Forcipomyia|[247]|RRSSA1158-15|579[0n]bp|Diptera|Ceratopogonidae|BOLD:AAU6519
Forcipomyia|[248]|RRSSA909-15|579[0n]bp|Diptera|Ceratopogonidae|BOLD:AAN5153
Forcipomyia|[249]|RRSSA1121-15|579[0n]bp|Diptera|Ceratopogonidae|BOLD:ABZ3626
Forcipomyia|[250]|RRMFE1884-15|552[0n]bp|Diptera|Ceratopogonidae|BOLD:AAQ0594
Forcipomyia|[251]|RRMFC089-15|576[0n]bp|Diptera|Ceratopogonidae|BOLD:AAM6191
Forcipomyia|[252]|RRMFC967-15|558[0n]bp|Diptera|Ceratopogonidae|BOLD:AAN5165
Ceratopogonidae|[253]|RRMFE202-15|591[0n]bp|Diptera|Ceratopogonidae|BOLD:AAN5155
Forcipomyia|[254]|RRMFC1149-15|549[0n]bp|Diptera|Ceratopogonidae|BOLD:AAV5144
Forcipomyia|[255]|RRMFC1531-15|588[0n]bp|Diptera|Ceratopogonidae|BOLD:AAV5212
Ceratopogonidae|[256]|RRMFE099-15|573[0n]bp|Diptera|Ceratopogonidae|BOLD:ACG3798
Atrichopogon|[257]|RRMFE040-15|564[0n]bp|Diptera|Ceratopogonidae|BOLD:AAG6519
Dasyhelea|[258]|RRMFC996-15|558[0n]bp|Diptera|Ceratopogonidae|BOLD:AAN5162
Forcipomyia|[259]|RRMFI495-15|576[0n]bp|Diptera|Ceratopogonidae|BOLD:ACC4125
Ceratopogonidae|[260]|RRMFA177-15|588[0n]bp|Diptera|Ceratopogonidae|BOLD:ACQ9076
Dasyhelea|[261]|RRMFG1269-15|579[0n]bp|Diptera|Ceratopogonidae|BOLD:ACN5021
Ceratopogonidae|[262]|RRMFE465-15|573[1n]bp|Diptera|Ceratopogonidae|BOLD:ACV4507
Atrichopogon|[263]|RRMFI127-15|567[0n]bp|Diptera|Ceratopogonidae|BOLD:AAG6431
Atrichopogon|[264]|RRMFE658-15|588[0n]bp|Diptera|Ceratopogonidae|BOLD:AAG3631
Atrichopogon|[265]|RRSSA3250-15|576[0n]bp|Diptera|Ceratopogonidae|BOLD:AAG6494
Atrichopogon|[266]|RRMFC539-15|588[0n]bp|Diptera|Ceratopogonidae|BOLD:ABA0806
Atrichopogon|[267]|RRMFI183-15|570[0n]bp|Diptera|Ceratopogonidae|BOLD:AAG6452
Atrichopogon|[268]|RRMFC1014-15|540[1n]bp|Diptera|Ceratopogonidae|BOLD:ABV9306
Atrichopogon|[269]|RRMFD942-15|555[0n]bp|Diptera|Ceratopogonidae|BOLD:ACA3626
Atrichopogon|[270]|RRSSA919-15|546[0n]bp|Diptera|Ceratopogonidae|BOLD:ACL4764
Atrichopogon|[271]|RRMFD1344-15|582[0n]bp|Diptera|Ceratopogonidae|BOLD:AAP6915
Atrichopogon|[272]|RRMFE800-15|594[0n]bp|Diptera|Ceratopogonidae|BOLD:ACV5558
Ceratopogonidae|[273]|RRSSA3454-15|588[0n]bp|Diptera|Ceratopogonidae|BOLD:ABW1518
Forcipomyia|[274]|RRSSA1181-15|585[0n]bp|Diptera|Ceratopogonidae|BOLD:ACA7885
Ceratopogonidae|[275]|RRMFG1841-15|567[0n]bp|Diptera|Ceratopogonidae|BOLD:AAN5158
Ceratopogonidae|[276]|RRSSA3380-15|561[0n]bp|Diptera|Ceratopogonidae|BOLD:ACR1960
Bezzia|[277]|RRSSA538-15|531[0n]bp|Diptera|Ceratopogonidae|BOLD:ACV5708
Clinohelea|[278]|RRSSA3248-15|591[0n]bp|Diptera|Ceratopogonidae|BOLD:AAG6451
Culicoides|[279]|RRMFG521-15|567[0n]bp|Diptera|Ceratopogonidae|BOLD:AAM6184
Ceratopogonidae|[280]|RRMFE461-15|567[0n]bp|Diptera|Ceratopogonidae|BOLD:ACA4406
Culicoides|[281]|RRSSA1107-15|576[0n]bp|Diptera|Ceratopogonidae|BOLD:AAN5166
Culicoides|[282]|RRMFI151-15|576[0n]bp|Diptera|Ceratopogonidae|BOLD:ACC3892
Culicoides|[283]|RRMFE470-15|591[2n]bp|Diptera|Ceratopogonidae|BOLD:ACV4326
Ceratopogonidae|[284]|RRMFI532-15|567[0n]bp|Diptera|Ceratopogonidae|BOLD:ACU4023
Bezzia|[285]|RRINV195-15|614[0n]bp|Diptera|Ceratopogonidae|
Brachypogon|[286]|RRSSA2762-15|564[0n]bp|Diptera|Ceratopogonidae|BOLD:AAL7405
Palpomyia|[287]|RRINV2314-15|630[0n]bp|Diptera|Ceratopogonidae|
Ceratopogonidae|[288]|RRINV2302-15|637[0n]bp|Diptera|Ceratopogonidae|
Palpomyia|[289]|RRINV2318-15|638[0n]bp|Diptera|Ceratopogonidae|
Paramyia nitens|[290]|RRMFI855-15|636[0n]bp|Diptera|Milichiidae|BOLD:AAG0166
Paramyia nitens|[291]|RRMFI199-15|540[0n]bp|Diptera|Milichiidae|BOLD:AAG0169
Tephritidae|[292]|RRINV3646-15|625[0n]bp|Diptera|Tephritidae|
Leptometopa latipes|[293]|RRMPC1272-15|555[0n]bp|Diptera|Milichiidae|BOLD:AAP8985
Rhagoletis suavis|[294]|RRINV3862-15|624[0n]bp|Diptera|Tephritidae|

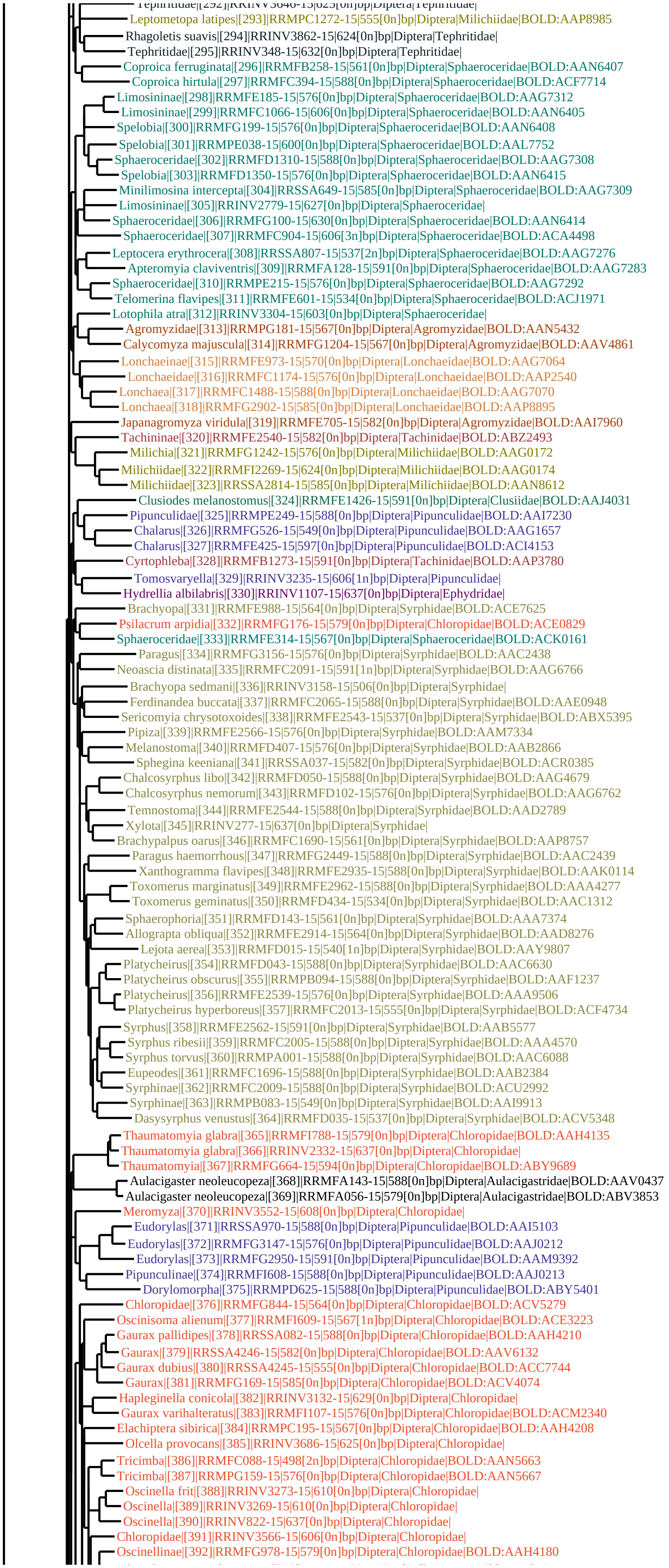
Tephritidae|[292]|RRINV3646-15|625[0n]bp|Diptera|Tephritidae|
Leptometopa latipes|[293]|RRMPC1272-15|555[0n]bp|Diptera|Milichiidae|BOLD:AAP8985
Rhagoletis suavis|[294]|RRINV3862-15|624[0n]bp|Diptera|Tephritidae|
Tephritidae|[295]|RRINV348-15|632[0n]bp|Diptera|Tephritidae|
Coproica ferruginata|[296]|RRMFB258-15|561[0n]bp|Diptera|Sphaeroceridae|BOLD:AAN6407
Coproica hirtula|[297]|RRMFC394-15|588[0n]bp|Diptera|Sphaeroceridae|BOLD:ACF7714
Limosininae|[298]|RRMFE185-15|576[0n]bp|Diptera|Sphaeroceridae|BOLD:AAG7312
Limosininae|[299]|RRMFC1066-15|606[0n]bp|Diptera|Sphaeroceridae|BOLD:AAN6405
Spelobia|[300]|RRMFG199-15|576[0n]bp|Diptera|Sphaeroceridae|BOLD:AAN6408
Spelobia|[301]|RRMPE038-15|600[0n]bp|Diptera|Sphaeroceridae|BOLD:AAL7752
Sphaeroceridae|[302]|RRMFD1310-15|588[0n]bp|Diptera|Sphaeroceridae|BOLD:AAG7308
Spelobia|[303]|RRMFD1350-15|576[0n]bp|Diptera|Sphaeroceridae|BOLD:AAN6415
Minilimosina intercepta|[304]|RRSSA649-15|585[0n]bp|Diptera|Sphaeroceridae|BOLD:AAG7309
Limosininae|[305]|RRINV2779-15|627[0n]bp|Diptera|Sphaeroceridae|
Sphaeroceridae|[306]|RRMFG100-15|630[0n]bp|Diptera|Sphaeroceridae|BOLD:AAN6414
Sphaeroceridae|[307]|RRMFC904-15|606[3n]bp|Diptera|Sphaeroceridae|BOLD:ACA4498
Leptocera erythrocera|[308]|RRSSA807-15|537[2n]bp|Diptera|Sphaeroceridae|BOLD:AAG7276
Apteromyia claviventris|[309]|RRMFA128-15|591[0n]bp|Diptera|Sphaeroceridae|BOLD:AAG7283
Sphaeroceridae|[310]|RRMPE215-15|576[0n]bp|Diptera|Sphaeroceridae|BOLD:AAG7292
Telomerina flavipes|[311]|RRMFE601-15|534[0n]bp|Diptera|Sphaeroceridae|BOLD:ACJ1971
Lotophila atra|[312]|RRINV3304-15|603[0n]bp|Diptera|Sphaeroceridae|
Agromyzidae|[313]|RRMPG181-15|567[0n]bp|Diptera|Agromyzidae|BOLD:AAN5432
Calycomyza majuscula|[314]|RRMFG1204-15|567[0n]bp|Diptera|Agromyzidae|BOLD:AAV4861
Lonchaeinae|[315]|RRMFE973-15|570[0n]bp|Diptera|Lonchaeidae|BOLD:AAG7064
Lonchaeidae|[316]|RRMFC1174-15|576[0n]bp|Diptera|Lonchaeidae|BOLD:AAP2540
Lonchaea|[317]|RRMFC1488-15|588[0n]bp|Diptera|Lonchaeidae|BOLD:AAG7070
Lonchaea|[318]|RRMFG2902-15|585[0n]bp|Diptera|Lonchaeidae|BOLD:AAP8895
Japanagromyza viridula|[319]|RRMFE705-15|582[0n]bp|Diptera|Agromyzidae|BOLD:AAI7960
Tachininae|[320]|RRMFE2540-15|582[0n]bp|Diptera|Tachinidae|BOLD:ABZ2493
Milichia|[321]|RRMFG1242-15|576[0n]bp|Diptera|Milichiidae|BOLD:AAG0172
Milichiidae|[322]|RRMFI2269-15|624[0n]bp|Diptera|Milichiidae|BOLD:AAG0174
Milichiidae|[323]|RRSSA2814-15|585[0n]bp|Diptera|Milichiidae|BOLD:AAN8612
Clusiodes melanostomus|[324]|RRMFE1426-15|591[0n]bp|Diptera|Clusiidae|BOLD:AAJ4031
Pipunculidae|[325]|RRMPE249-15|588[0n]bp|Diptera|Pipunculidae|BOLD:AAI7230
Chalarus|[326]|RRMFG526-15|549[0n]bp|Diptera|Pipunculidae|BOLD:AAG1657
Chalarus|[327]|RRMFE425-15|597[0n]bp|Diptera|Pipunculidae|BOLD:ACI4153
Cyrtophleba|[328]|RRMFB1273-15|591[0n]bp|Diptera|Tachinidae|BOLD:AAP3780
Tomosvaryella|[329]|RRINV3235-15|606[1n]bp|Diptera|Pipunculidae|
Hydrellia albilabris|[330]|RRINV1107-15|637[0n]bp|Diptera|Ephydridae|
Brachyopa|[331]|RRMFE988-15|564[0n]bp|Diptera|Syrphidae|BOLD:ACE7625
Psilacrum arpidia|[332]|RRMFG176-15|579[0n]bp|Diptera|Chloropidae|BOLD:ACE0829
Sphaeroceridae|[333]|RRMFE314-15|567[0n]bp|Diptera|Sphaeroceridae|BOLD:ACK0161
Paragus|[334]|RRMFG3156-15|576[0n]bp|Diptera|Syrphidae|BOLD:AAC2438
Neoascia distinata|[335]|RRMFC2091-15|591[1n]bp|Diptera|Syrphidae|BOLD:AAG6766
Brachyopa sedmani|[336]|RRINV3158-15|506[0n]bp|Diptera|Syrphidae|
Ferdinandea buccata|[337]|RRMFC2065-15|588[0n]bp|Diptera|Syrphidae|BOLD:AAE0948
Sericomyia chrysotoxoides|[338]|RRMFE2543-15|537[0n]bp|Diptera|Syrphidae|BOLD:ABX5395
Pipiza|[339]|RRMFE2566-15|576[0n]bp|Diptera|Syrphidae|BOLD:AAM7334
Melanostoma|[340]|RRMFD407-15|576[0n]bp|Diptera|Syrphidae|BOLD:AAB2866
Sphegina keeniana|[341]|RRSSA037-15|582[0n]bp|Diptera|Syrphidae|BOLD:ACR0385
Chalcosyrphus libo|[342]|RRMFD050-15|588[0n]bp|Diptera|Syrphidae|BOLD:AAG4679
Chalcosyrphus nemorum|[343]|RRMFD102-15|576[0n]bp|Diptera|Syrphidae|BOLD:AAG6762
Temnostoma|[344]|RRMFE2544-15|588[0n]bp|Diptera|Syrphidae|BOLD:AAD2789
Xylota|[345]|RRINV277-15|637[0n]bp|Diptera|Syrphidae|
Brachypalpus oarus|[346]|RRMFC1690-15|561[0n]bp|Diptera|Syrphidae|BOLD:AAP8757
Paragus haemorrhous|[347]|RRMFG2449-15|588[0n]bp|Diptera|Syrphidae|BOLD:AAC2439
Xanthogramma flavipes|[348]|RRMFE2935-15|588[0n]bp|Diptera|Syrphidae|BOLD:AAK0114
Toxomerus marginatus|[349]|RRMFE2962-15|588[0n]bp|Diptera|Syrphidae|BOLD:AAA4277
Toxomerus geminatus|[350]|RRMFD434-15|534[0n]bp|Diptera|Syrphidae|BOLD:AAC1312
Sphaerophoria|[351]|RRMFD143-15|561[0n]bp|Diptera|Syrphidae|BOLD:AAA7374
Allograpta obliqua|[352]|RRMFE2914-15|564[0n]bp|Diptera|Syrphidae|BOLD:AAD8276
Lejota aerea|[353]|RRMFD015-15|540[1n]bp|Diptera|Syrphidae|BOLD:AAY9807
Platycheirus|[354]|RRMFD043-15|588[0n]bp|Diptera|Syrphidae|BOLD:AAC6630
Platycheirus obscurus|[355]|RRMPB094-15|588[0n]bp|Diptera|Syrphidae|BOLD:AAF1237
Platycheirus|[356]|RRMFE2539-15|576[0n]bp|Diptera|Syrphidae|BOLD:AAA9506
Platycheirus hyperboreus|[357]|RRMFC2013-15|555[0n]bp|Diptera|Syrphidae|BOLD:ACF4734
Syrphus|[358]|RRMFE2562-15|591[0n]bp|Diptera|Syrphidae|BOLD:AAB5577
Syrphus ribesii|[359]|RRMFC2005-15|588[0n]bp|Diptera|Syrphidae|BOLD:AAA4570
Syrphus torvus|[360]|RRMPA001-15|588[0n]bp|Diptera|Syrphidae|BOLD:AAC6088
Eupeodes|[361]|RRMFC1696-15|588[0n]bp|Diptera|Syrphidae|BOLD:AAB2384
Syrphinae|[362]|RRMFC2009-15|588[0n]bp|Diptera|Syrphidae|BOLD:ACU2992
Syrphinae|[363]|RRMPB083-15|549[0n]bp|Diptera|Syrphidae|BOLD:AAI9913
Dasysyrphus venustus|[364]|RRMFD035-15|537[0n]bp|Diptera|Syrphidae|BOLD:ACV5348
Thaumatomyia glabra|[365]|RRMFI788-15|579[0n]bp|Diptera|Chloropidae|BOLD:AAH4135
Thaumatomyia glabra|[366]|RRINV2332-15|637[0n]bp|Diptera|Chloropidae|
Thaumatomyia|[367]|RRMFG664-15|594[0n]bp|Diptera|Chloropidae|BOLD:ABY9689
Aulacigaster neoleucopeza|[368]|RRMFA143-15|588[0n]bp|Diptera|Aulacigastridae|BOLD:AAV0437
Aulacigaster neoleucopeza|[369]|RRMFA056-15|579[0n]bp|Diptera|Aulacigastridae|BOLD:ABV3853
Meromyza|[370]|RRINV3552-15|608[0n]bp|Diptera|Chloropidae|
Eudorylas|[371]|RRSSA970-15|588[0n]bp|Diptera|Pipunculidae|BOLD:AAI5103
Eudorylas|[372]|RRMFG3147-15|576[0n]bp|Diptera|Pipunculidae|BOLD:AAJ0212
Eudorylas|[373]|RRMFG2950-15|591[0n]bp|Diptera|Pipunculidae|BOLD:AAM9392
Pipunculinae|[374]|RRMFI608-15|588[0n]bp|Diptera|Pipunculidae|BOLD:AAJ0213
Dorylomorpha|[375]|RRMPD625-15|588[0n]bp|Diptera|Pipunculidae|BOLD:ABY5401
Chloropidae|[376]|RRMFG844-15|564[0n]bp|Diptera|Chloropidae|BOLD:ACV5279
Oscinisoma alienum|[377]|RRMFI609-15|567[1n]bp|Diptera|Chloropidae|BOLD:ACE3223
Gaurax pallidipes|[378]|RRSSA082-15|588[0n]bp|Diptera|Chloropidae|BOLD:AAH4210
Gaurax|[379]|RRSSA4246-15|582[0n]bp|Diptera|Chloropidae|BOLD:AAV6132
Gaurax dubius|[380]|RRSSA4245-15|555[0n]bp|Diptera|Chloropidae|BOLD:ACC7744
Gaurax|[381]|RRMFG169-15|585[0n]bp|Diptera|Chloropidae|BOLD:ACV4074
Hapleginella conicola|[382]|RRINV3132-15|629[0n]bp|Diptera|Chloropidae|
Gaurax varihalteratus|[383]|RRMFI107-15|576[0n]bp|Diptera|Chloropidae|BOLD:ACM2340
Elachiptera sibirica|[384]|RRMPC195-15|567[0n]bp|Diptera|Chloropidae|BOLD:AAH4208
Olcella provocans|[385]|RRINV3686-15|625[0n]bp|Diptera|Chloropidae|
Tricimba|[386]|RRMFC088-15|498[2n]bp|Diptera|Chloropidae|BOLD:AAN5663
Tricimba|[387]|RRMPG159-15|576[0n]bp|Diptera|Chloropidae|BOLD:AAN5667
Oscinella frit|[388]|RRINV3273-15|610[0n]bp|Diptera|Chloropidae|
Oscinella|[389]|RRINV3269-15|610[0n]bp|Diptera|Chloropidae|
Oscinella|[390]|RRINV822-15|637[0n]bp|Diptera|Chloropidae|
Chloropidae|[391]|RRINV3566-15|606[0n]bp|Diptera|Chloropidae|
Oscinellinae|[392]|RRMFG978-15|579[0n]bp|Diptera|Chloropidae|BOLD:AAH4180

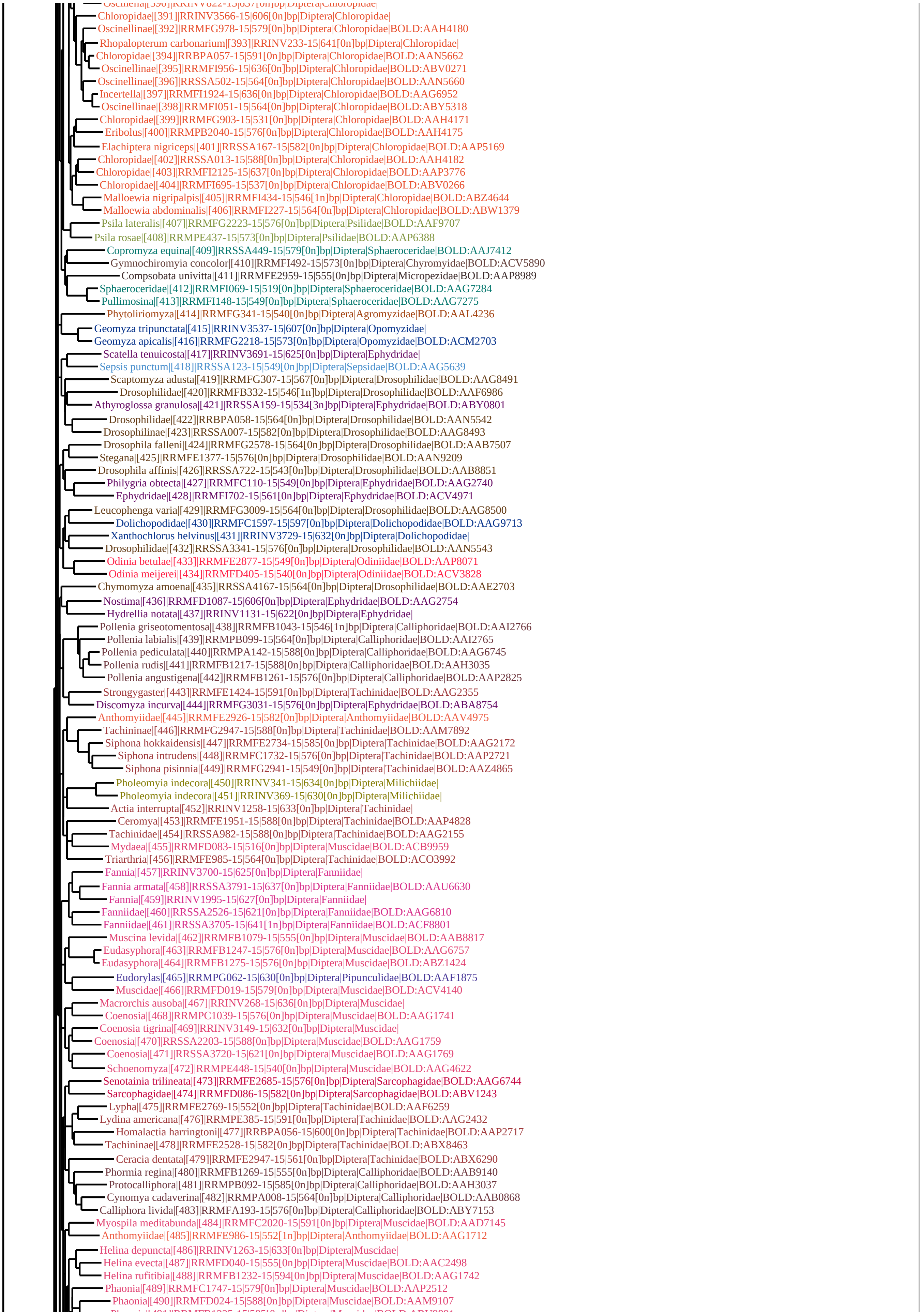
Chloropidae|[391]|RRINV3566-15|606[0n]bp|Diptera|Chloropidae|
Oscinellinae|[392]|RRMFG978-15|579[0n]bp|Diptera|Chloropidae|BOLD:AAH4180
Rhopalopterum carbonarium|[393]|RRINV233-15|641[0n]bp|Diptera|Chloropidae|
Chloropidae|[394]|RRBPA057-15|591[0n]bp|Diptera|Chloropidae|BOLD:AAN5662
Oscinellinae|[395]|RRMFI956-15|636[0n]bp|Diptera|Chloropidae|BOLD:ABV0271
Oscinellinae|[396]|RRSSA502-15|564[0n]bp|Diptera|Chloropidae|BOLD:AAN5660
Incertella|[397]|RRMFI1924-15|636[0n]bp|Diptera|Chloropidae|BOLD:AAG6952
Oscinellinae|[398]|RRMFI051-15|564[0n]bp|Diptera|Chloropidae|BOLD:ABY5318
Chloropidae|[399]|RRMFG903-15|531[0n]bp|Diptera|Chloropidae|BOLD:AAH4171
Eribolus|[400]|RRMPB2040-15|576[0n]bp|Diptera|Chloropidae|BOLD:AAH4175
Elachiptera nigriceps|[401]|RRSSA167-15|582[0n]bp|Diptera|Chloropidae|BOLD:AAP5169
Chloropidae|[402]|RRSSA013-15|588[0n]bp|Diptera|Chloropidae|BOLD:AAH4182
Chloropidae|[403]|RRMFI2125-15|637[0n]bp|Diptera|Chloropidae|BOLD:AAP3776
Chloropidae|[404]|RRMFI695-15|537[0n]bp|Diptera|Chloropidae|BOLD:ABV0266
Malloewia nigripalpis|[405]|RRMFI434-15|546[1n]bp|Diptera|Chloropidae|BOLD:ABZ4644
Malloewia abdominalis|[406]|RRMFI227-15|564[0n]bp|Diptera|Chloropidae|BOLD:ABW1379
Psila lateralis|[407]|RRMFG2223-15|576[0n]bp|Diptera|Psilidae|BOLD:AAF9707
Psila rosae|[408]|RRMPE437-15|573[0n]bp|Diptera|Psilidae|BOLD:AAP6388
Copromyza equina|[409]|RRSSA449-15|579[0n]bp|Diptera|Sphaeroceridae|BOLD:AAJ7412
Gymnochiromyia concolor|[410]|RRMFI492-15|573[0n]bp|Diptera|Chyromyidae|BOLD:ACV5890
Compsobata univitta|[411]|RRMFE2959-15|555[0n]bp|Diptera|Micropezidae|BOLD:AAP8989
Sphaeroceridae|[412]|RRMFI069-15|519[0n]bp|Diptera|Sphaeroceridae|BOLD:AAG7284
Pullimosina|[413]|RRMFI148-15|549[0n]bp|Diptera|Sphaeroceridae|BOLD:AAG7275
Phytoliriomyza|[414]|RRMFG341-15|540[0n]bp|Diptera|Agromyzidae|BOLD:AAL4236
Geomyza tripunctata|[415]|RRINV3537-15|607[0n]bp|Diptera|Opomyzidae|
Geomyza apicalis|[416]|RRMFG2218-15|573[0n]bp|Diptera|Opomyzidae|BOLD:ACM2703
Scatella tenuicosta|[417]|RRINV3691-15|625[0n]bp|Diptera|Ephydridae|
Sepsis punctum|[418]|RRSSA123-15|549[0n]bp|Diptera|Sepsidae|BOLD:AAG5639
Scaptomyza adusta|[419]|RRMFG307-15|567[0n]bp|Diptera|Drosophilidae|BOLD:AAG8491
Drosophilidae|[420]|RRMFB332-15|546[1n]bp|Diptera|Drosophilidae|BOLD:AAF6986
Athyroglossa granulosa|[421]|RRSSA159-15|534[3n]bp|Diptera|Ephydridae|BOLD:ABY0801
Drosophilidae|[422]|RRBPA058-15|564[0n]bp|Diptera|Drosophilidae|BOLD:AAN5542
Drosophilinae|[423]|RRSSA007-15|582[0n]bp|Diptera|Drosophilidae|BOLD:AAG8493
Drosophila falleni|[424]|RRMFG2578-15|564[0n]bp|Diptera|Drosophilidae|BOLD:AAB7507
Stegana|[425]|RRMFE1377-15|576[0n]bp|Diptera|Drosophilidae|BOLD:AAN9209
Drosophila affinis|[426]|RRSSA722-15|543[0n]bp|Diptera|Drosophilidae|BOLD:AAB8851
Philygria obtecta|[427]|RRMFC110-15|549[0n]bp|Diptera|Ephydridae|BOLD:AAG2740
Ephydridae|[428]|RRMFI702-15|561[0n]bp|Diptera|Ephydridae|BOLD:ACV4971
Leucophenga varia|[429]|RRMFG3009-15|564[0n]bp|Diptera|Drosophilidae|BOLD:AAG8500
Dolichopodidae|[430]|RRMFC1597-15|597[0n]bp|Diptera|Dolichopodidae|BOLD:AAG9713
Xanthochlorus helvinus|[431]|RRINV3729-15|632[0n]bp|Diptera|Dolichopodidae|
Drosophilidae|[432]|RRSSA3341-15|576[0n]bp|Diptera|Drosophilidae|BOLD:AAN5543
Odinia betulae|[433]|RRMFE2877-15|549[0n]bp|Diptera|Odiniidae|BOLD:AAP8071
Odinia meijerei|[434]|RRMFD405-15|540[0n]bp|Diptera|Odiniidae|BOLD:ACV3828
Chymomyza amoena|[435]|RRSSA4167-15|564[0n]bp|Diptera|Drosophilidae|BOLD:AAE2703
Nostima|[436]|RRMFD1087-15|606[0n]bp|Diptera|Ephydridae|BOLD:AAG2754
Hydrellia notata|[437]|RRINV1131-15|622[0n]bp|Diptera|Ephydridae|
Pollenia griseotomentosa|[438]|RRMFB1043-15|546[1n]bp|Diptera|Calliphoridae|BOLD:AAI2766
Pollenia labialis|[439]|RRMPB099-15|564[0n]bp|Diptera|Calliphoridae|BOLD:AAI2765
Pollenia pediculata|[440]|RRMPA142-15|588[0n]bp|Diptera|Calliphoridae|BOLD:AAG6745
Pollenia rudis|[441]|RRMFB1217-15|588[0n]bp|Diptera|Calliphoridae|BOLD:AAH3035
Pollenia angustigena|[442]|RRMFB1261-15|576[0n]bp|Diptera|Calliphoridae|BOLD:AAP2825
Strongygaster|[443]|RRMFE1424-15|591[0n]bp|Diptera|Tachinidae|BOLD:AAG2355
Discomyza incurva|[444]|RRMFG3031-15|576[0n]bp|Diptera|Ephydridae|BOLD:ABA8754
Anthomyiidae|[445]|RRMFE2926-15|582[0n]bp|Diptera|Anthomyiidae|BOLD:AAV4975
Tachininae|[446]|RRMFG2947-15|588[0n]bp|Diptera|Tachinidae|BOLD:AAM7892
Siphona hokkaidensis|[447]|RRMFE2734-15|585[0n]bp|Diptera|Tachinidae|BOLD:AAG2172
Siphona intrudens|[448]|RRMFC1732-15|576[0n]bp|Diptera|Tachinidae|BOLD:AAP2721
Siphona pisinnia|[449]|RRMFG2941-15|549[0n]bp|Diptera|Tachinidae|BOLD:AAZ4865
Pholeomyia indecora|[450]|RRINV341-15|634[0n]bp|Diptera|Milichiidae|
Pholeomyia indecora|[451]|RRINV369-15|630[0n]bp|Diptera|Milichiidae|
Actia interrupta|[452]|RRINV1258-15|633[0n]bp|Diptera|Tachinidae|
Ceromya|[453]|RRMFE1951-15|588[0n]bp|Diptera|Tachinidae|BOLD:AAP4828
Tachinidae|[454]|RRSSA982-15|588[0n]bp|Diptera|Tachinidae|BOLD:AAG2155
Mydaea|[455]|RRMFD083-15|516[0n]bp|Diptera|Muscidae|BOLD:ACB9959
Triarthria|[456]|RRMFE985-15|564[0n]bp|Diptera|Tachinidae|BOLD:ACO3992
Fannia|[457]|RRINV3700-15|625[0n]bp|Diptera|Fanniidae|
Fannia armata|[458]|RRSSA3791-15|637[0n]bp|Diptera|Fanniidae|BOLD:AAU6630
Fannia|[459]|RRINV1995-15|627[0n]bp|Diptera|Fanniidae|
Fanniidae|[460]|RRSSA2526-15|621[0n]bp|Diptera|Fanniidae|BOLD:AAG6810
Fanniidae|[461]|RRSSA3705-15|641[1n]bp|Diptera|Fanniidae|BOLD:ACF8801
Muscina levida|[462]|RRMFB1079-15|555[0n]bp|Diptera|Muscidae|BOLD:AAB8817
Eudasyphora|[463]|RRMFB1247-15|576[0n]bp|Diptera|Muscidae|BOLD:AAG6757
Eudasyphora|[464]|RRMFB1275-15|576[0n]bp|Diptera|Muscidae|BOLD:ABZ1424
Eudorylas|[465]|RRMPG062-15|630[0n]bp|Diptera|Pipunculidae|BOLD:AAF1875
Muscidae|[466]|RRMFD019-15|579[0n]bp|Diptera|Muscidae|BOLD:ACV4140
Macrorchis ausoba|[467]|RRINV268-15|636[0n]bp|Diptera|Muscidae|
Coenosia|[468]|RRMPC1039-15|576[0n]bp|Diptera|Muscidae|BOLD:AAG1741
Coenosia tigrina|[469]|RRINV3149-15|632[0n]bp|Diptera|Muscidae|
Coenosia|[470]|RRSSA2203-15|588[0n]bp|Diptera|Muscidae|BOLD:AAG1759
Coenosia|[471]|RRSSA3720-15|621[0n]bp|Diptera|Muscidae|BOLD:AAG1769
Schoenomyza|[472]|RRMPE448-15|540[0n]bp|Diptera|Muscidae|BOLD:AAG4622
Senotainia trilineata|[473]|RRMFE2685-15|576[0n]bp|Diptera|Sarcophagidae|BOLD:AAG6744
Sarcophagidae|[474]|RRMFD086-15|582[0n]bp|Diptera|Sarcophagidae|BOLD:ABV1243
Lypha|[475]|RRMFE2769-15|552[0n]bp|Diptera|Tachinidae|BOLD:AAF6259
Lydina americana|[476]|RRMPE385-15|591[0n]bp|Diptera|Tachinidae|BOLD:AAG2432
Homalactia harringtoni|[477]|RRBPA056-15|600[0n]bp|Diptera|Tachinidae|BOLD:AAP2717
Tachininae|[478]|RRMFE2528-15|582[0n]bp|Diptera|Tachinidae|BOLD:ABX8463
Ceracia dentata|[479]|RRMFE2947-15|561[0n]bp|Diptera|Tachinidae|BOLD:ABX6290
Phormia regina|[480]|RRMFB1269-15|555[0n]bp|Diptera|Calliphoridae|BOLD:AAB9140
Protocalliphora|[481]|RRMPB092-15|585[0n]bp|Diptera|Calliphoridae|BOLD:AAH3037
Cynomya cadaverina|[482]|RRMPA008-15|564[0n]bp|Diptera|Calliphoridae|BOLD:AAB0868
Calliphora livida|[483]|RRMFA193-15|576[0n]bp|Diptera|Calliphoridae|BOLD:ABY7153
Myospila meditabunda|[484]|RRMFC2020-15|591[0n]bp|Diptera|Muscidae|BOLD:AAD7145
Anthomyiidae|[485]|RRMFE986-15|552[1n]bp|Diptera|Anthomyiidae|BOLD:AAG1712
Helina depuncta|[486]|RRINV1263-15|633[0n]bp|Diptera|Muscidae|
Helina evecta|[487]|RRMFD040-15|555[0n]bp|Diptera|Muscidae|BOLD:AAC2498
Helina rufitibia|[488]|RRMFB1232-15|594[0n]bp|Diptera|Muscidae|BOLD:AAG1742
Phaonia|[489]|RRMFC1747-15|579[0n]bp|Diptera|Muscidae|BOLD:AAP2512
Phaonia|[490]|RRMFD024-15|588[0n]bp|Diptera|Muscidae|BOLD:AAM9107

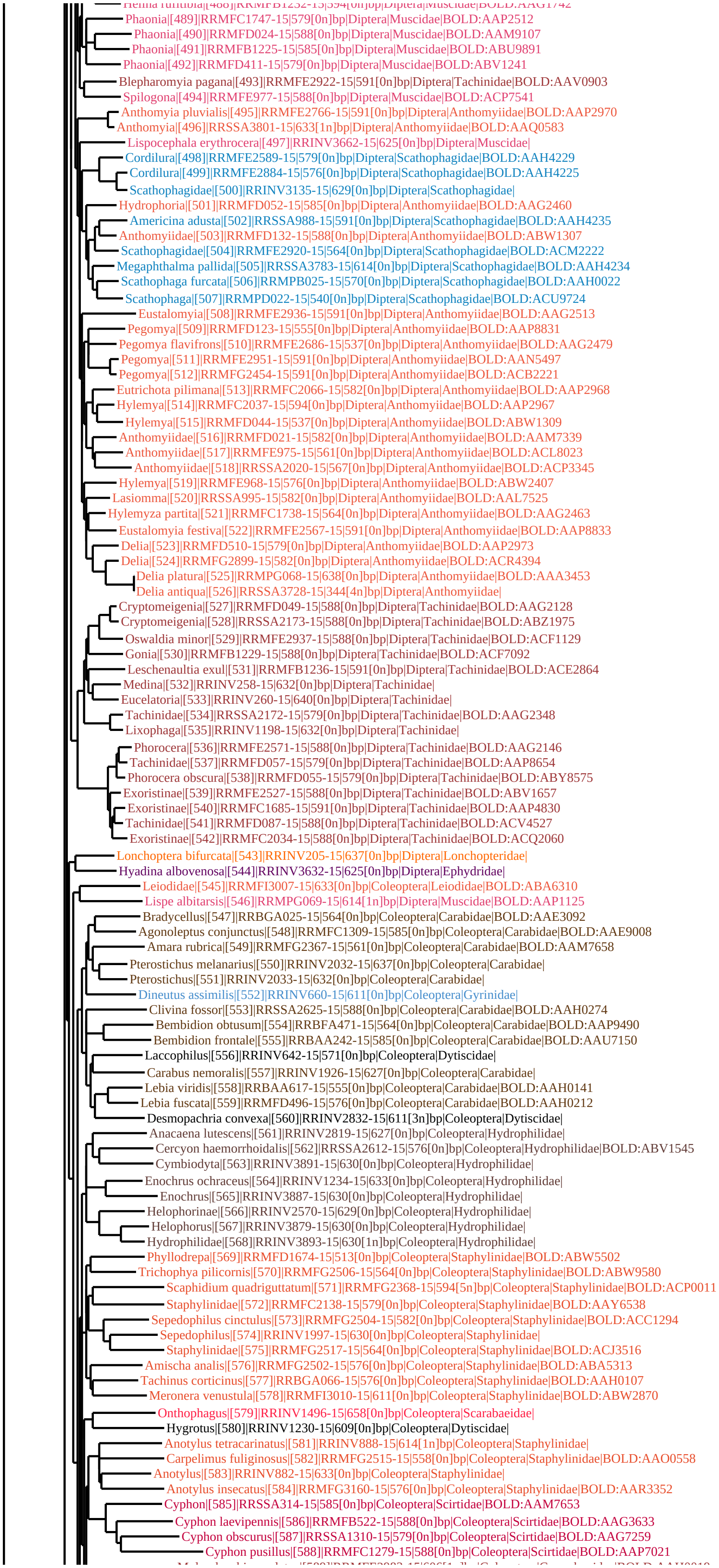
Phaonia|[489]|RRMFC1747-15|579[0n]bp|Diptera|Muscidae|BOLD:AAP2512
Phaonia|[490]|RRMFD024-15|588[0n]bp|Diptera|Muscidae|BOLD:AAM9107
Phaonia|[491]|RRMFB1225-15|585[0n]bp|Diptera|Muscidae|BOLD:ABU9891
Phaonia|[492]|RRMFD411-15|579[0n]bp|Diptera|Muscidae|BOLD:ABV1241
Blepharomyia pagana|[493]|RRMFE2922-15|591[0n]bp|Diptera|Tachinidae|BOLD:AAV0903
Spilogona|[494]|RRMFE977-15|588[0n]bp|Diptera|Muscidae|BOLD:ACP7541
Anthomyia pluvialis|[495]|RRMFE2766-15|591[0n]bp|Diptera|Anthomyiidae|BOLD:AAP2970
Anthomyia|[496]|RRSSA3801-15|633[1n]bp|Diptera|Anthomyiidae|BOLD:AAQ0583
Lispocephala erythrocera|[497]|RRINV3662-15|625[0n]bp|Diptera|Muscidae|
Cordilura|[498]|RRMFE2589-15|579[0n]bp|Diptera|Scathophagidae|BOLD:AAH4229
Cordilura|[499]|RRMFE2884-15|576[0n]bp|Diptera|Scathophagidae|BOLD:AAH4225
Scathophagidae|[500]|RRINV3135-15|629[0n]bp|Diptera|Scathophagidae|
Hydrophoria|[501]|RRMFD052-15|585[0n]bp|Diptera|Anthomyiidae|BOLD:AAG2460
Americina adusta|[502]|RRSSA988-15|591[0n]bp|Diptera|Scathophagidae|BOLD:AAH4235
Anthomyiidae|[503]|RRMFD132-15|588[0n]bp|Diptera|Anthomyiidae|BOLD:ABW1307
Scathophagidae|[504]|RRMFE2920-15|564[0n]bp|Diptera|Scathophagidae|BOLD:ACM2222
Megaphthalma pallida|[505]|RRSSA3783-15|614[0n]bp|Diptera|Scathophagidae|BOLD:AAH4234
Scathophaga furcata|[506]|RRMPB025-15|570[0n]bp|Diptera|Scathophagidae|BOLD:AAH0022
Scathophaga|[507]|RRMPD022-15|540[0n]bp|Diptera|Scathophagidae|BOLD:ACU9724
Eustalomyia|[508]|RRMFE2936-15|591[0n]bp|Diptera|Anthomyiidae|BOLD:AAG2513
Pegomya|[509]|RRMFD123-15|555[0n]bp|Diptera|Anthomyiidae|BOLD:AAP8831
Pegomya flavifrons|[510]|RRMFE2686-15|537[0n]bp|Diptera|Anthomyiidae|BOLD:AAG2479
Pegomya|[511]|RRMFE2951-15|591[0n]bp|Diptera|Anthomyiidae|BOLD:AAN5497
Pegomya|[512]|RRMFG2454-15|591[0n]bp|Diptera|Anthomyiidae|BOLD:ACB2221
Eutrichota pilimana|[513]|RRMFC2066-15|582[0n]bp|Diptera|Anthomyiidae|BOLD:AAP2968
Hylemya|[514]|RRMFC2037-15|594[0n]bp|Diptera|Anthomyiidae|BOLD:AAP2967
Hylemya|[515]|RRMFD044-15|537[0n]bp|Diptera|Anthomyiidae|BOLD:ABW1309
Anthomyiidae|[516]|RRMFD021-15|582[0n]bp|Diptera|Anthomyiidae|BOLD:AAM7339
Anthomyiidae|[517]|RRMFE975-15|561[0n]bp|Diptera|Anthomyiidae|BOLD:ACL8023
Anthomyiidae|[518]|RRSSA2020-15|567[0n]bp|Diptera|Anthomyiidae|BOLD:ACP3345
Hylemya|[519]|RRMFE968-15|576[0n]bp|Diptera|Anthomyiidae|BOLD:ABW2407
Lasiomma|[520]|RRSSA995-15|582[0n]bp|Diptera|Anthomyiidae|BOLD:AAL7525
Hylemyza partita|[521]|RRMFC1738-15|564[0n]bp|Diptera|Anthomyiidae|BOLD:AAG2463
Eustalomyia festiva|[522]|RRMFE2567-15|591[0n]bp|Diptera|Anthomyiidae|BOLD:AAP8833
Delia|[523]|RRMFD510-15|579[0n]bp|Diptera|Anthomyiidae|BOLD:AAP2973
Delia|[524]|RRMFG2899-15|582[0n]bp|Diptera|Anthomyiidae|BOLD:ACR4394
Delia platura|[525]|RRMPG068-15|638[0n]bp|Diptera|Anthomyiidae|BOLD:AAA3453
Delia antiqua|[526]|RRSSA3728-15|344[4n]bp|Diptera|Anthomyiidae|
Cryptomeigenia|[527]|RRMFD049-15|588[0n]bp|Diptera|Tachinidae|BOLD:AAG2128
Cryptomeigenia|[528]|RRSSA2173-15|588[0n]bp|Diptera|Tachinidae|BOLD:ABZ1975
Oswaldia minor|[529]|RRMFE2937-15|588[0n]bp|Diptera|Tachinidae|BOLD:ACF1129
Gonia|[530]|RRMFB1229-15|588[0n]bp|Diptera|Tachinidae|BOLD:ACF7092
Leschenaultia exul|[531]|RRMFB1236-15|591[0n]bp|Diptera|Tachinidae|BOLD:ACE2864
Medina|[532]|RRINV258-15|632[0n]bp|Diptera|Tachinidae|
Eucelatoria|[533]|RRINV260-15|640[0n]bp|Diptera|Tachinidae|
Tachinidae|[534]|RRSSA2172-15|579[0n]bp|Diptera|Tachinidae|BOLD:AAG2348
Lixophaga|[535]|RRINV1198-15|632[0n]bp|Diptera|Tachinidae|
Phorocera|[536]|RRMFE2571-15|588[0n]bp|Diptera|Tachinidae|BOLD:AAG2146
Tachinidae|[537]|RRMFD057-15|579[0n]bp|Diptera|Tachinidae|BOLD:AAP8654
Phorocera obscura|[538]|RRMFD055-15|579[0n]bp|Diptera|Tachinidae|BOLD:ABY8575
Exoristinae|[539]|RRMFE2527-15|588[0n]bp|Diptera|Tachinidae|BOLD:ABV1657
Exoristinae|[540]|RRMFC1685-15|591[0n]bp|Diptera|Tachinidae|BOLD:AAP4830
Tachinidae|[541]|RRMFD087-15|588[0n]bp|Diptera|Tachinidae|BOLD:ACV4527
Exoristinae|[542]|RRMFC2034-15|588[0n]bp|Diptera|Tachinidae|BOLD:ACQ2060
Lonchoptera bifurcata|[543]|RRINV205-15|637[0n]bp|Diptera|Lonchopteridae|
Hyadina albovenosa|[544]|RRINV3632-15|625[0n]bp|Diptera|Ephydridae|
Leiodidae|[545]|RRMFI3007-15|633[0n]bp|Coleoptera|Leiodidae|BOLD:ABA6310
Lispe albitarsis|[546]|RRMPG069-15|614[1n]bp|Diptera|Muscidae|BOLD:AAP1125
Bradycellus|[547]|RRBGA025-15|564[0n]bp|Coleoptera|Carabidae|BOLD:AAE3092
Agonoleptus conjunctus|[548]|RRMFC1309-15|585[0n]bp|Coleoptera|Carabidae|BOLD:AAE9008
Amara rubrica|[549]|RRMFG2367-15|561[0n]bp|Coleoptera|Carabidae|BOLD:AAM7658
Pterostichus melanarius|[550]|RRINV2032-15|637[0n]bp|Coleoptera|Carabidae|
Pterostichus|[551]|RRINV2033-15|632[0n]bp|Coleoptera|Carabidae|
Dineutus assimilis|[552]|RRINV660-15|611[0n]bp|Coleoptera|Gyrinidae|
Clivina fossor|[553]|RRSSA2625-15|588[0n]bp|Coleoptera|Carabidae|BOLD:AAH0274
Bembidion obtusum|[554]|RRBFA471-15|564[0n]bp|Coleoptera|Carabidae|BOLD:AAP9490
Bembidion frontale|[555]|RRBAA242-15|585[0n]bp|Coleoptera|Carabidae|BOLD:AAU7150
Laccophilus|[556]|RRINV642-15|571[0n]bp|Coleoptera|Dytiscidae|
Carabus nemoralis|[557]|RRINV1926-15|627[0n]bp|Coleoptera|Carabidae|
Lebia viridis|[558]|RRBAA617-15|555[0n]bp|Coleoptera|Carabidae|BOLD:AAH0141
Lebia fuscata|[559]|RRMFD496-15|576[0n]bp|Coleoptera|Carabidae|BOLD:AAH0212
Desmopachria convexa|[560]|RRINV2832-15|611[3n]bp|Coleoptera|Dytiscidae|
Anacaena lutescens|[561]|RRINV2819-15|627[0n]bp|Coleoptera|Hydrophilidae|
Cercyon haemorrhoidalis|[562]|RRSSA2612-15|576[0n]bp|Coleoptera|Hydrophilidae|BOLD:ABV1545
Cymbiodyta|[563]|RRINV3891-15|630[0n]bp|Coleoptera|Hydrophilidae|
Enochrus ochraceus|[564]|RRINV1234-15|633[0n]bp|Coleoptera|Hydrophilidae|
Enochrus|[565]|RRINV3887-15|630[0n]bp|Coleoptera|Hydrophilidae|
Helophorinae|[566]|RRINV2570-15|629[0n]bp|Coleoptera|Hydrophilidae|
Helophorus|[567]|RRINV3879-15|630[0n]bp|Coleoptera|Hydrophilidae|
Hydrophilidae|[568]|RRINV3893-15|630[1n]bp|Coleoptera|Hydrophilidae|
Phyllodrepa|[569]|RRMFD1674-15|513[0n]bp|Coleoptera|Staphylinidae|BOLD:ABW5502
Trichophya pilicornis|[570]|RRMFG2506-15|564[0n]bp|Coleoptera|Staphylinidae|BOLD:ABW9580
Scaphidium quadriguttatum|[571]|RRMFG2368-15|594[5n]bp|Coleoptera|Staphylinidae|BOLD:ACP0011
Staphylinidae|[572]|RRMFC2138-15|579[0n]bp|Coleoptera|Staphylinidae|BOLD:AAY6538
Sepedophilus cinctulus|[573]|RRMFG2504-15|582[0n]bp|Coleoptera|Staphylinidae|BOLD:ACC1294
Sepedophilus|[574]|RRINV1997-15|630[0n]bp|Coleoptera|Staphylinidae|
Staphylinidae|[575]|RRMFG2517-15|564[0n]bp|Coleoptera|Staphylinidae|BOLD:ACJ3516
Amischa analis|[576]|RRMFG2502-15|576[0n]bp|Coleoptera|Staphylinidae|BOLD:ABA5313
Tachinus corticinus|[577]|RRBGA066-15|576[0n]bp|Coleoptera|Staphylinidae|BOLD:AAH0107
Meronera venustula|[578]|RRMFI3010-15|611[0n]bp|Coleoptera|Staphylinidae|BOLD:ABW2870
Onthophagus|[579]|RRINV1496-15|658[0n]bp|Coleoptera|Scarabaeidae|
Hygrotus|[580]|RRINV1230-15|609[0n]bp|Coleoptera|Dytiscidae|
Anotylus tetracarinatus|[581]|RRINV888-15|614[1n]bp|Coleoptera|Staphylinidae|
Carpelimus fuliginosus|[582]|RRMFG2515-15|558[0n]bp|Coleoptera|Staphylinidae|BOLD:AAO0558
Anotylus|[583]|RRINV882-15|633[0n]bp|Coleoptera|Staphylinidae|
Anotylus insecatus|[584]|RRMFG3160-15|576[0n]bp|Coleoptera|Staphylinidae|BOLD:AAR3352
Cyphon|[585]|RRSSA314-15|585[0n]bp|Coleoptera|Scirtidae|BOLD:AAM7653
Cyphon laevipennis|[586]|RRMFB522-15|588[0n]bp|Coleoptera|Scirtidae|BOLD:AAG3633
Cyphon obscurus|[587]|RRSSA1310-15|579[0n]bp|Coleoptera|Scirtidae|BOLD:AAG7259
Cyphon pusillus|[588]|RRMFC1279-15|588[0n]bp|Coleoptera|Scirtidae|BOLD:AAP7021

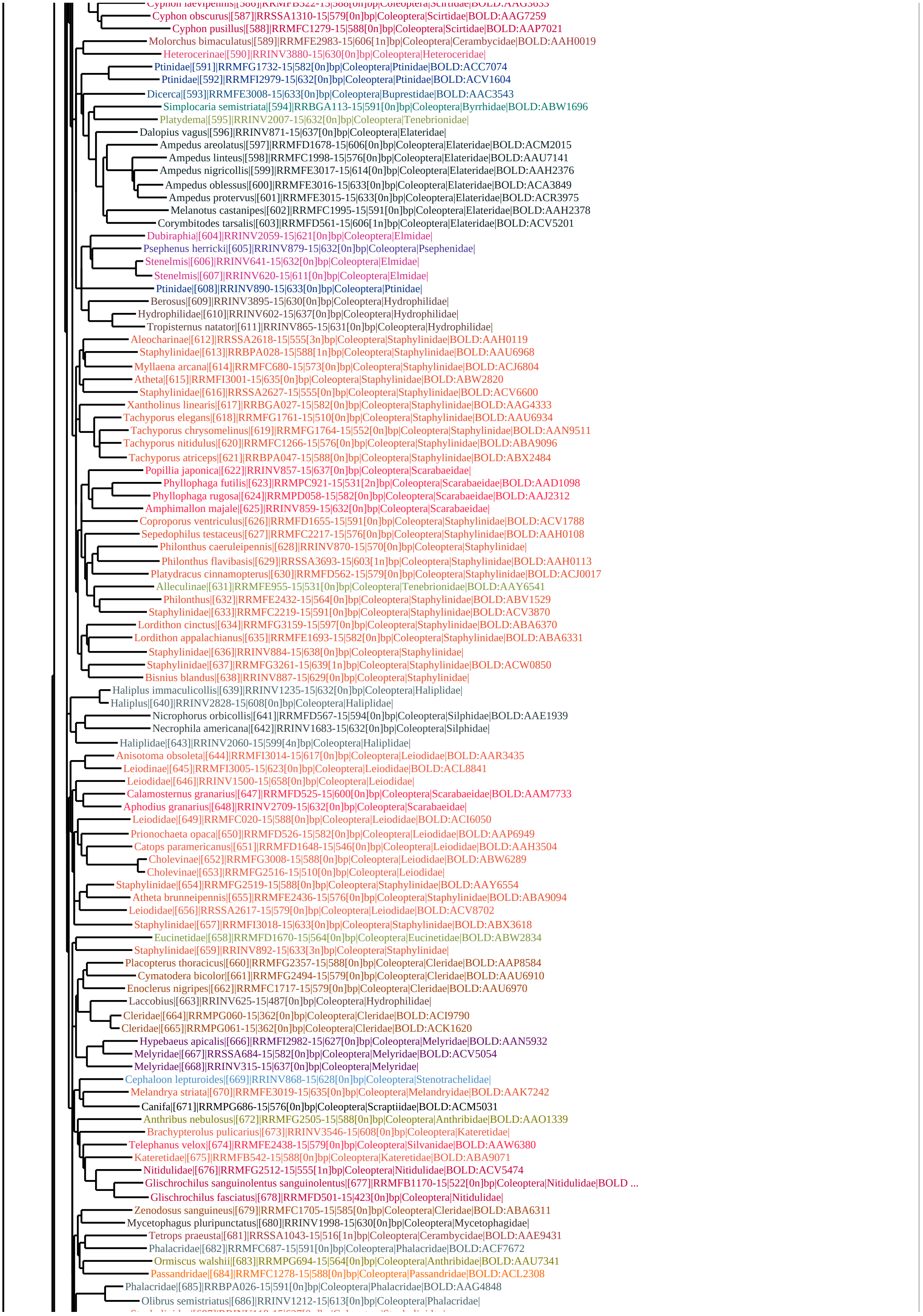

Cyphon obscurus|[587]|RRSSA1310-15|579[0n]bp|Coleoptera|Scirtidae|BOLD:AAG7259
Cyphon pusillus|[588]|RRMFC1279-15|588[0n]bp|Coleoptera|Scirtidae|BOLD:AAP7021
Molorchus bimaculatus|[589]|RRMFE2983-15|606[1n]bp|Coleoptera|Cerambycidae|BOLD:AAH0019
Heterocerinae|[590]|RRINV3880-15|630[0n]bp|Coleoptera|Heteroceridae|
Ptinidae|[591]|RRMFG1732-15|582[0n]bp|Coleoptera|Ptinidae|BOLD:ACC7074
Ptinidae|[592]|RRMFI2979-15|632[0n]bp|Coleoptera|Ptinidae|BOLD:ACV1604
Dicerca|[593]|RRMFE3008-15|633[0n]bp|Coleoptera|Buprestidae|BOLD:AAC3543
Simplocaria semistriata|[594]|RRBGA113-15|591[0n]bp|Coleoptera|Byrrhidae|BOLD:ABW1696
Platydema|[595]|RRINV2007-15|632[0n]bp|Coleoptera|Tenebrionidae|
Dalopius vagus|[596]|RRINV871-15|637[0n]bp|Coleoptera|Elateridae|
Ampedus areolatus|[597]|RRMFD1678-15|606[0n]bp|Coleoptera|Elateridae|BOLD:ACM2015
Ampedus linteus|[598]|RRMFC1998-15|576[0n]bp|Coleoptera|Elateridae|BOLD:AAU7141
Ampedus nigricollis|[599]|RRMFE3017-15|614[0n]bp|Coleoptera|Elateridae|BOLD:AAH2376
Ampedus oblessus|[600]|RRMFE3016-15|633[0n]bp|Coleoptera|Elateridae|BOLD:ACA3849
Ampedus protervus|[601]|RRMFE3015-15|633[0n]bp|Coleoptera|Elateridae|BOLD:ACR3975
Melanotus castanipes|[602]|RRMFC1995-15|591[0n]bp|Coleoptera|Elateridae|BOLD:AAH2378
Corymbitodes tarsalis|[603]|RRMFD561-15|606[1n]bp|Coleoptera|Elateridae|BOLD:ACV5201
Dubiraphia|[604]|RRINV2059-15|621[0n]bp|Coleoptera|Elmidae|
Psephenus herricki|[605]|RRINV879-15|632[0n]bp|Coleoptera|Psephenidae|
Stenelmis|[606]|RRINV641-15|632[0n]bp|Coleoptera|Elmidae|
Stenelmis|[607]|RRINV620-15|611[0n]bp|Coleoptera|Elmidae|
Ptinidae|[608]|RRINV890-15|633[0n]bp|Coleoptera|Ptinidae|
Berosus|[609]|RRINV3895-15|630[0n]bp|Coleoptera|Hydrophilidae|
Hydrophilidae|[610]|RRINV602-15|637[0n]bp|Coleoptera|Hydrophilidae|
Tropisternus natator|[611]|RRINV865-15|631[0n]bp|Coleoptera|Hydrophilidae|
Aleocharinae|[612]|RRSSA2618-15|555[3n]bp|Coleoptera|Staphylinidae|BOLD:AAH0119
Staphylinidae|[613]|RRBPA028-15|588[1n]bp|Coleoptera|Staphylinidae|BOLD:AAU6968
Myllaena arcana|[614]|RRMFC680-15|573[0n]bp|Coleoptera|Staphylinidae|BOLD:ACJ6804
Atheta|[615]|RRMFI3001-15|635[0n]bp|Coleoptera|Staphylinidae|BOLD:ABW2820
Staphylinidae|[616]|RRSSA2627-15|555[0n]bp|Coleoptera|Staphylinidae|BOLD:ACV6600
Xantholinus linearis|[617]|RRBGA027-15|582[0n]bp|Coleoptera|Staphylinidae|BOLD:AAG4333
Tachyporus elegans|[618]|RRMFG1761-15|510[0n]bp|Coleoptera|Staphylinidae|BOLD:AAU6934
Tachyporus chrysomelinus|[619]|RRMFG1764-15|552[0n]bp|Coleoptera|Staphylinidae|BOLD:AAN9511
Tachyporus nitidulus|[620]|RRMFC1266-15|576[0n]bp|Coleoptera|Staphylinidae|BOLD:ABA9096
Tachyporus atriceps|[621]|RRBPA047-15|588[0n]bp|Coleoptera|Staphylinidae|BOLD:ABX2484
Popillia japonica|[622]|RRINV857-15|637[0n]bp|Coleoptera|Scarabaeidae|
Phyllophaga futilis|[623]|RRMPC921-15|531[2n]bp|Coleoptera|Scarabaeidae|BOLD:AAD1098
Phyllophaga rugosa|[624]|RRMPD058-15|582[0n]bp|Coleoptera|Scarabaeidae|BOLD:AAJ2312
Amphimallon majale|[625]|RRINV859-15|632[0n]bp|Coleoptera|Scarabaeidae|
Coproporus ventriculus|[626]|RRMFD1655-15|591[0n]bp|Coleoptera|Staphylinidae|BOLD:ACV1788
Sepedophilus testaceus|[627]|RRMFC2217-15|576[0n]bp|Coleoptera|Staphylinidae|BOLD:AAH0108
Philonthus caeruleipennis|[628]|RRINV870-15|570[0n]bp|Coleoptera|Staphylinidae|
Philonthus flavibasis|[629]|RRSSA3693-15|603[1n]bp|Coleoptera|Staphylinidae|BOLD:AAH0113
Platydracus cinnamopterus|[630]|RRMFD562-15|579[0n]bp|Coleoptera|Staphylinidae|BOLD:ACJ0017
Alleculinae|[631]|RRMFE955-15|531[0n]bp|Coleoptera|Tenebrionidae|BOLD:AAY6541
Philonthus|[632]|RRMFE2432-15|564[0n]bp|Coleoptera|Staphylinidae|BOLD:ABV1529
Staphylinidae|[633]|RRMFC2219-15|591[0n]bp|Coleoptera|Staphylinidae|BOLD:ACV3870
Lordithon cinctus|[634]|RRMFG3159-15|597[0n]bp|Coleoptera|Staphylinidae|BOLD:ABA6370
Lordithon appalachianus|[635]|RRMFE1693-15|582[0n]bp|Coleoptera|Staphylinidae|BOLD:ABA6331
Staphylinidae|[636]|RRINV884-15|638[0n]bp|Coleoptera|Staphylinidae|
Staphylinidae|[637]|RRMFG3261-15|639[1n]bp|Coleoptera|Staphylinidae|BOLD:ACW0850
Bisnius blandus|[638]|RRINV887-15|629[0n]bp|Coleoptera|Staphylinidae|
Haliplus immaculicollis|[639]|RRINV1235-15|632[0n]bp|Coleoptera|Haliplidae|
Haliplus|[640]|RRINV2828-15|608[0n]bp|Coleoptera|Haliplidae|
Nicrophorus orbicollis|[641]|RRMFD567-15|594[0n]bp|Coleoptera|Silphidae|BOLD:AAE1939
Necrophila americana|[642]|RRINV1683-15|632[0n]bp|Coleoptera|Silphidae|
Haliplidae|[643]|RRINV2060-15|599[4n]bp|Coleoptera|Haliplidae|
Anisotoma obsoleta|[644]|RRMFI3014-15|617[0n]bp|Coleoptera|Leiodidae|BOLD:AAR3435
Leiodinae|[645]|RRMFI3005-15|623[0n]bp|Coleoptera|Leiodidae|BOLD:ACL8841
Leiodidae|[646]|RRINV1500-15|658[0n]bp|Coleoptera|Leiodidae|
Calamosternus granarius|[647]|RRMFD525-15|600[0n]bp|Coleoptera|Scarabaeidae|BOLD:AAM7733
Aphodius granarius|[648]|RRINV2709-15|632[0n]bp|Coleoptera|Scarabaeidae|
Leiodidae|[649]|RRMFC020-15|588[0n]bp|Coleoptera|Leiodidae|BOLD:ACI6050
Prionochaeta opaca|[650]|RRMFD526-15|582[0n]bp|Coleoptera|Leiodidae|BOLD:AAP6949
Catops paramericanus|[651]|RRMFD1648-15|546[0n]bp|Coleoptera|Leiodidae|BOLD:AAH3504
Cholevinae|[652]|RRMFG3008-15|588[0n]bp|Coleoptera|Leiodidae|BOLD:ABW6289
Cholevinae|[653]|RRMFG2516-15|510[0n]bp|Coleoptera|Leiodidae|
Staphylinidae|[654]|RRMFG2519-15|588[0n]bp|Coleoptera|Staphylinidae|BOLD:AAY6554
Atheta brunneipennis|[655]|RRMFE2436-15|576[0n]bp|Coleoptera|Staphylinidae|BOLD:ABA9094
Leiodidae|[656]|RRSSA2617-15|579[0n]bp|Coleoptera|Leiodidae|BOLD:ACV8702
Staphylinidae|[657]|RRMFI3018-15|633[0n]bp|Coleoptera|Staphylinidae|BOLD:ABX3618
Eucinetidae|[658]|RRMFD1670-15|564[0n]bp|Coleoptera|Eucinetidae|BOLD:ABW2834
Staphylinidae|[659]|RRINV892-15|633[3n]bp|Coleoptera|Staphylinidae|
Placopterus thoracicus|[660]|RRMFG2357-15|588[0n]bp|Coleoptera|Cleridae|BOLD:AAP8584
Cymatodera bicolor|[661]|RRMFG2494-15|579[0n]bp|Coleoptera|Cleridae|BOLD:AAU6910
Enoclerus nigripes|[662]|RRMFC1717-15|579[0n]bp|Coleoptera|Cleridae|BOLD:AAU6970
Laccobius|[663]|RRINV625-15|487[0n]bp|Coleoptera|Hydrophilidae|
Cleridae|[664]|RRMPG060-15|362[0n]bp|Coleoptera|Cleridae|BOLD:ACI9790
Cleridae|[665]|RRMPG061-15|362[0n]bp|Coleoptera|Cleridae|BOLD:ACK1620
Hypebaeus apicalis|[666]|RRMFI2982-15|627[0n]bp|Coleoptera|Melyridae|BOLD:AAN5932
Melyridae|[667]|RRSSA684-15|582[0n]bp|Coleoptera|Melyridae|BOLD:ACV5054
Melyridae|[668]|RRINV315-15|637[0n]bp|Coleoptera|Melyridae|
Cephaloon lepturoides|[669]|RRINV868-15|628[0n]bp|Coleoptera|Stenotrachelidae|
Melandrya striata|[670]|RRMFE3019-15|635[0n]bp|Coleoptera|Melandryidae|BOLD:AAK7242
Canifa|[671]|RRMPG686-15|576[0n]bp|Coleoptera|Scraptiidae|BOLD:ACM5031
Anthribus nebulosus|[672]|RRMFG2505-15|588[0n]bp|Coleoptera|Anthribidae|BOLD:AAO1339
Brachypterolus pulicarius|[673]|RRINV3546-15|608[0n]bp|Coleoptera|Kateretidae|
Telephanus velox|[674]|RRMFE2438-15|579[0n]bp|Coleoptera|Silvanidae|BOLD:AAW6380
Kateretidae|[675]|RRMFB542-15|588[0n]bp|Coleoptera|Kateretidae|BOLD:ABA9071
Nitidulidae|[676]|RRMFG2512-15|555[1n]bp|Coleoptera|Nitidulidae|BOLD:ACV5474
Glischrochilus sanguinolentus sanguinolentus|[677]|RRMFB1170-15|522[0n]bp|Coleoptera|Nitidulidae|BOLD ...
Glischrochilus fasciatus|[678]|RRMFD501-15|423[0n]bp|Coleoptera|Nitidulidae|
Zenodosus sanguineus|[679]|RRMFC1705-15|585[0n]bp|Coleoptera|Cleridae|BOLD:ABA6311
Mycetophagus pluripunctatus|[680]|RRINV1998-15|630[0n]bp|Coleoptera|Mycetophagidae|
Tetrops praeusta|[681]|RRSSA1043-15|516[1n]bp|Coleoptera|Cerambycidae|BOLD:AAE9431
Phalacridae|[682]|RRMFC687-15|591[0n]bp|Coleoptera|Phalacridae|BOLD:ACF7672
Ormiscus walshii|[683]|RRMPG694-15|564[0n]bp|Coleoptera|Anthribidae|BOLD:AAU7341
Passandridae|[684]|RRMFC1278-15|588[0n]bp|Coleoptera|Passandridae|BOLD:ACL2308
Phalacridae|[685]|RRBPA026-15|591[0n]bp|Coleoptera|Phalacridae|BOLD:AAG4848
Olibrus semistriatus|[686]|RRINV1212-15|613[0n]bp|Coleoptera|Phalacridae|

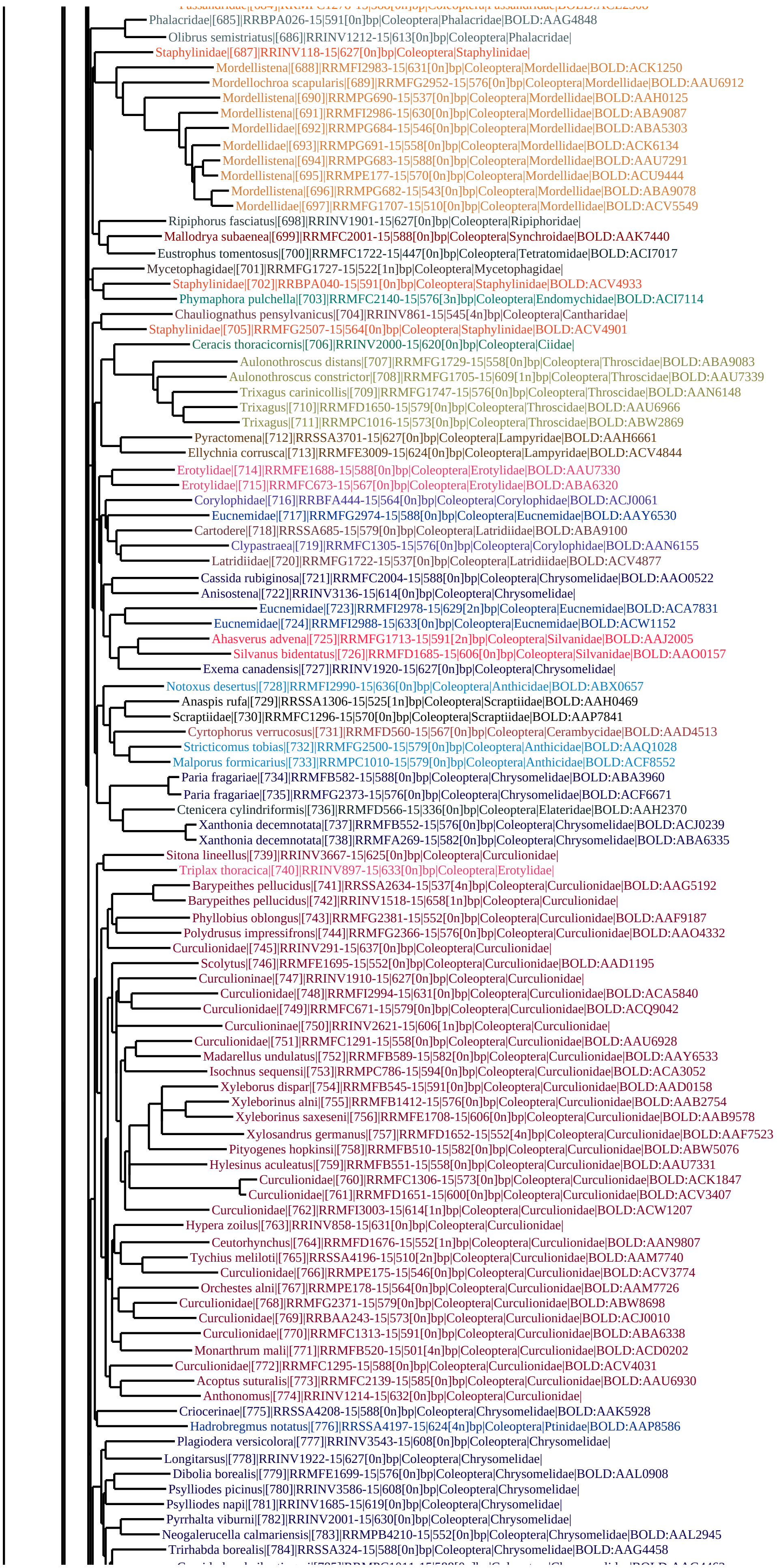

Phalacridae|[685]|RRBPA026-15|591[0n]bp|Coleoptera|Phalacridae|BOLD:AAG4848
Olibrus semistriatus|[686]|RRINV1212-15|613[0n]bp|Coleoptera|Phalacridae|
Staphylinidae|[687]|RRINV118-15|627[0n]bp|Coleoptera|Staphylinidae|
Mordellistena|[688]|RRMFI2983-15|631[0n]bp|Coleoptera|Mordellidae|BOLD:ACK1250
Mordellochroa scapularis|[689]|RRMFG2952-15|576[0n]bp|Coleoptera|Mordellidae|BOLD:AAU6912
Mordellistena|[690]|RRMPG690-15|537[0n]bp|Coleoptera|Mordellidae|BOLD:AAH0125
Mordellistena|[691]|RRMFI2986-15|630[0n]bp|Coleoptera|Mordellidae|BOLD:ABA9087
Mordellidae|[692]|RRMPG684-15|546[0n]bp|Coleoptera|Mordellidae|BOLD:ABA5303
Mordellidae|[693]|RRMPG691-15|558[0n]bp|Coleoptera|Mordellidae|BOLD:ACK6134
Mordellistena|[694]|RRMPG683-15|588[0n]bp|Coleoptera|Mordellidae|BOLD:AAU7291
Mordellistena|[695]|RRMPE177-15|570[0n]bp|Coleoptera|Mordellidae|BOLD:ACU9444
Mordellistena|[696]|RRMPG682-15|543[0n]bp|Coleoptera|Mordellidae|BOLD:ABA9078
Mordellidae|[697]|RRMFG1707-15|510[0n]bp|Coleoptera|Mordellidae|BOLD:ACV5549
Ripiphorus fasciatus|[698]|RRINV1901-15|627[0n]bp|Coleoptera|Ripiphoridae|
Mallodrya subaenea|[699]|RRMFC2001-15|588[0n]bp|Coleoptera|Synchroidae|BOLD:AAK7440
Eustrophus tomentosus|[700]|RRMFC1722-15|447[0n]bp|Coleoptera|Tetratomidae|BOLD:ACI7017
Mycetophagidae|[701]|RRMFG1727-15|522[1n]bp|Coleoptera|Mycetophagidae|
Staphylinidae|[702]|RRBPA040-15|591[0n]bp|Coleoptera|Staphylinidae|BOLD:ACV4933
Phymaphora pulchella|[703]|RRMFC2140-15|576[3n]bp|Coleoptera|Endomychidae|BOLD:ACI7114
Chauliognathus pensylvanicus|[704]|RRINV861-15|545[4n]bp|Coleoptera|Cantharidae|
Staphylinidae|[705]|RRMFG2507-15|564[0n]bp|Coleoptera|Staphylinidae|BOLD:ACV4901
Ceracis thoracicornis|[706]|RRINV2000-15|620[0n]bp|Coleoptera|Ciidae|
Aulonothroscus distans|[707]|RRMFG1729-15|558[0n]bp|Coleoptera|Throscidae|BOLD:ABA9083
Aulonothroscus constrictor|[708]|RRMFG1705-15|609[1n]bp|Coleoptera|Throscidae|BOLD:AAU7339
Trixagus carinicollis|[709]|RRMFG1747-15|576[0n]bp|Coleoptera|Throscidae|BOLD:AAN6148
Trixagus|[710]|RRMFD1650-15|579[0n]bp|Coleoptera|Throscidae|BOLD:AAU6966
Trixagus|[711]|RRMPC1016-15|573[0n]bp|Coleoptera|Throscidae|BOLD:ABW2869
Pyractomena|[712]|RRSSA3701-15|627[0n]bp|Coleoptera|Lampyridae|BOLD:AAH6661
Ellychnia corrusca|[713]|RRMFE3009-15|624[0n]bp|Coleoptera|Lampyridae|BOLD:ACV4844
Erotylidae|[714]|RRMFE1688-15|588[0n]bp|Coleoptera|Erotylidae|BOLD:AAU7330
Erotylidae|[715]|RRMFC673-15|567[0n]bp|Coleoptera|Erotylidae|BOLD:ABA6320
Corylophidae|[716]|RRBFA444-15|564[0n]bp|Coleoptera|Corylophidae|BOLD:ACJ0061
Eucnemidae|[717]|RRMFG2974-15|588[0n]bp|Coleoptera|Eucnemidae|BOLD:AAY6530
Cartodere|[718]|RRSSA685-15|579[0n]bp|Coleoptera|Latridiidae|BOLD:ABA9100
Clypastraea|[719]|RRMFC1305-15|576[0n]bp|Coleoptera|Corylophidae|BOLD:AAN6155
Latridiidae|[720]|RRMFG1722-15|537[0n]bp|Coleoptera|Latridiidae|BOLD:ACV4877
Cassida rubiginosa|[721]|RRMFC2004-15|588[0n]bp|Coleoptera|Chrysomelidae|BOLD:AAO0522
Anisostena|[722]|RRINV3136-15|614[0n]bp|Coleoptera|Chrysomelidae|
Eucnemidae|[723]|RRMFI2978-15|629[2n]bp|Coleoptera|Eucnemidae|BOLD:ACA7831
Eucnemidae|[724]|RRMFI2988-15|633[0n]bp|Coleoptera|Eucnemidae|BOLD:ACW1152
Ahasverus advena|[725]|RRMFG1713-15|591[2n]bp|Coleoptera|Silvanidae|BOLD:AAJ2005
Silvanus bidentatus|[726]|RRMFD1685-15|606[0n]bp|Coleoptera|Silvanidae|BOLD:AAO0157
Exema canadensis|[727]|RRINV1920-15|627[0n]bp|Coleoptera|Chrysomelidae|
Notoxus desertus|[728]|RRMFI2990-15|636[0n]bp|Coleoptera|Anthicidae|BOLD:ABX0657
Anaspis rufa|[729]|RRSSA1306-15|525[1n]bp|Coleoptera|Scraptiidae|BOLD:AAH0469
Scraptiidae|[730]|RRMFC1296-15|570[0n]bp|Coleoptera|Scraptiidae|BOLD:AAP7841
Cyrtophorus verrucosus|[731]|RRMFD560-15|567[0n]bp|Coleoptera|Cerambycidae|BOLD:AAD4513
Stricticomus tobias|[732]|RRMFG2500-15|579[0n]bp|Coleoptera|Anthicidae|BOLD:AAQ1028
Malporus formicarius|[733]|RRMPC1010-15|579[0n]bp|Coleoptera|Anthicidae|BOLD:ACF8552
Paria fragariae|[734]|RRMFB582-15|588[0n]bp|Coleoptera|Chrysomelidae|BOLD:ABA3960
Paria fragariae|[735]|RRMFG2373-15|576[0n]bp|Coleoptera|Chrysomelidae|BOLD:ACF6671
Ctenicera cylindriformis|[736]|RRMFD566-15|336[0n]bp|Coleoptera|Elateridae|BOLD:AAH2370
Xanthonia decemnotata|[737]|RRMFB552-15|576[0n]bp|Coleoptera|Chrysomelidae|BOLD:ACJ0239
Xanthonia decemnotata|[738]|RRMFA269-15|582[0n]bp|Coleoptera|Chrysomelidae|BOLD:ABA6335
Sitona lineellus|[739]|RRINV3667-15|625[0n]bp|Coleoptera|Curculionidae|
Triplax thoracica|[740]|RRINV897-15|633[0n]bp|Coleoptera|Erotylidae|
Barypeithes pellucidus|[741]|RRSSA2634-15|537[4n]bp|Coleoptera|Curculionidae|BOLD:AAG5192
Barypeithes pellucidus|[742]|RRINV1518-15|658[1n]bp|Coleoptera|Curculionidae|
Phyllobius oblongus|[743]|RRMFG2381-15|552[0n]bp|Coleoptera|Curculionidae|BOLD:AAF9187
Polydrusus impressifrons|[744]|RRMFG2366-15|576[0n]bp|Coleoptera|Curculionidae|BOLD:AAO4332
Curculionidae|[745]|RRINV291-15|637[0n]bp|Coleoptera|Curculionidae|
Scolytus|[746]|RRMFE1695-15|552[0n]bp|Coleoptera|Curculionidae|BOLD:AAD1195
Curculioninae|[747]|RRINV1910-15|627[0n]bp|Coleoptera|Curculionidae|
Curculionidae|[748]|RRMFI2994-15|631[0n]bp|Coleoptera|Curculionidae|BOLD:ACA5840
Curculionidae|[749]|RRMFC671-15|579[0n]bp|Coleoptera|Curculionidae|BOLD:ACQ9042
Curculioninae|[750]|RRINV2621-15|606[1n]bp|Coleoptera|Curculionidae|
Curculionidae|[751]|RRMFC1291-15|558[0n]bp|Coleoptera|Curculionidae|BOLD:AAU6928
Madarellus undulatus|[752]|RRMFB589-15|582[0n]bp|Coleoptera|Curculionidae|BOLD:AAY6533
Isochnus sequensi|[753]|RRMPC786-15|594[0n]bp|Coleoptera|Curculionidae|BOLD:ACA3052
Xyleborus dispar|[754]|RRMFB545-15|591[0n]bp|Coleoptera|Curculionidae|BOLD:AAD0158
Xyleborinus alni|[755]|RRMFB1412-15|576[0n]bp|Coleoptera|Curculionidae|BOLD:AAB2754
Xyleborinus saxeseni|[756]|RRMFE1708-15|606[0n]bp|Coleoptera|Curculionidae|BOLD:AAB9578
Xylosandrus germanus|[757]|RRMFD1652-15|552[4n]bp|Coleoptera|Curculionidae|BOLD:AAF7523
Pityogenes hopkinsi|[758]|RRMFB510-15|582[0n]bp|Coleoptera|Curculionidae|BOLD:ABW5076
Hylesinus aculeatus|[759]|RRMFB551-15|558[0n]bp|Coleoptera|Curculionidae|BOLD:AAU7331
Curculionidae|[760]|RRMFC1306-15|573[0n]bp|Coleoptera|Curculionidae|BOLD:ACK1847
Curculionidae|[761]|RRMFD1651-15|600[0n]bp|Coleoptera|Curculionidae|BOLD:ACV3407
Curculionidae|[762]|RRMFI3003-15|614[1n]bp|Coleoptera|Curculionidae|BOLD:ACW1207
Hypera zoilus|[763]|RRINV858-15|631[0n]bp|Coleoptera|Curculionidae|
Ceutorhynchus|[764]|RRMFD1676-15|552[1n]bp|Coleoptera|Curculionidae|BOLD:AAN9807
Tychius meliloti|[765]|RRSSA4196-15|510[2n]bp|Coleoptera|Curculionidae|BOLD:AAM7740
Curculionidae|[766]|RRMPE175-15|546[0n]bp|Coleoptera|Curculionidae|BOLD:ACV3774
Orchestes alni|[767]|RRMPE178-15|564[0n]bp|Coleoptera|Curculionidae|BOLD:AAM7726
Curculionidae|[768]|RRMFG2371-15|579[0n]bp|Coleoptera|Curculionidae|BOLD:ABW8698
Curculionidae|[769]|RRBAA243-15|573[0n]bp|Coleoptera|Curculionidae|BOLD:ACJ0010
Curculionidae|[770]|RRMFC1313-15|591[0n]bp|Coleoptera|Curculionidae|BOLD:ABA6338
Monarthrum mali|[771]|RRMFB520-15|501[4n]bp|Coleoptera|Curculionidae|BOLD:ACD0202
Curculionidae|[772]|RRMFC1295-15|588[0n]bp|Coleoptera|Curculionidae|BOLD:ACV4031
Acoptus suturalis|[773]|RRMFC2139-15|585[0n]bp|Coleoptera|Curculionidae|BOLD:AAU6930
Anthonomus|[774]|RRINV1214-15|632[0n]bp|Coleoptera|Curculionidae|
Criocerinae|[775]|RRSSA4208-15|588[0n]bp|Coleoptera|Chrysomelidae|BOLD:AAK5928
Hadrobregmus notatus|[776]|RRSSA4197-15|624[4n]bp|Coleoptera|Ptinidae|BOLD:AAP8586
Plagiodera versicolora|[777]|RRINV3543-15|608[0n]bp|Coleoptera|Chrysomelidae|
Longitarsus|[778]|RRINV1922-15|627[0n]bp|Coleoptera|Chrysomelidae|
Dibolia borealis|[779]|RRMFE1699-15|576[0n]bp|Coleoptera|Chrysomelidae|BOLD:AAL0908
Psylliodes picinus|[780]|RRINV3586-15|608[0n]bp|Coleoptera|Chrysomelidae|
Psylliodes napi|[781]|RRINV1685-15|619[0n]bp|Coleoptera|Chrysomelidae|
Pyrrhalta viburni|[782]|RRINV2001-15|630[0n]bp|Coleoptera|Chrysomelidae|
Neogalerucella calmariensis|[783]|RRMPB4210-15|552[0n]bp|Coleoptera|Chrysomelidae|BOLD:AAL2945
Trirhabda borealis|[784]|RRSSA324-15|588[0n]bp|Coleoptera|Chrysomelidae|BOLD:AAG4458

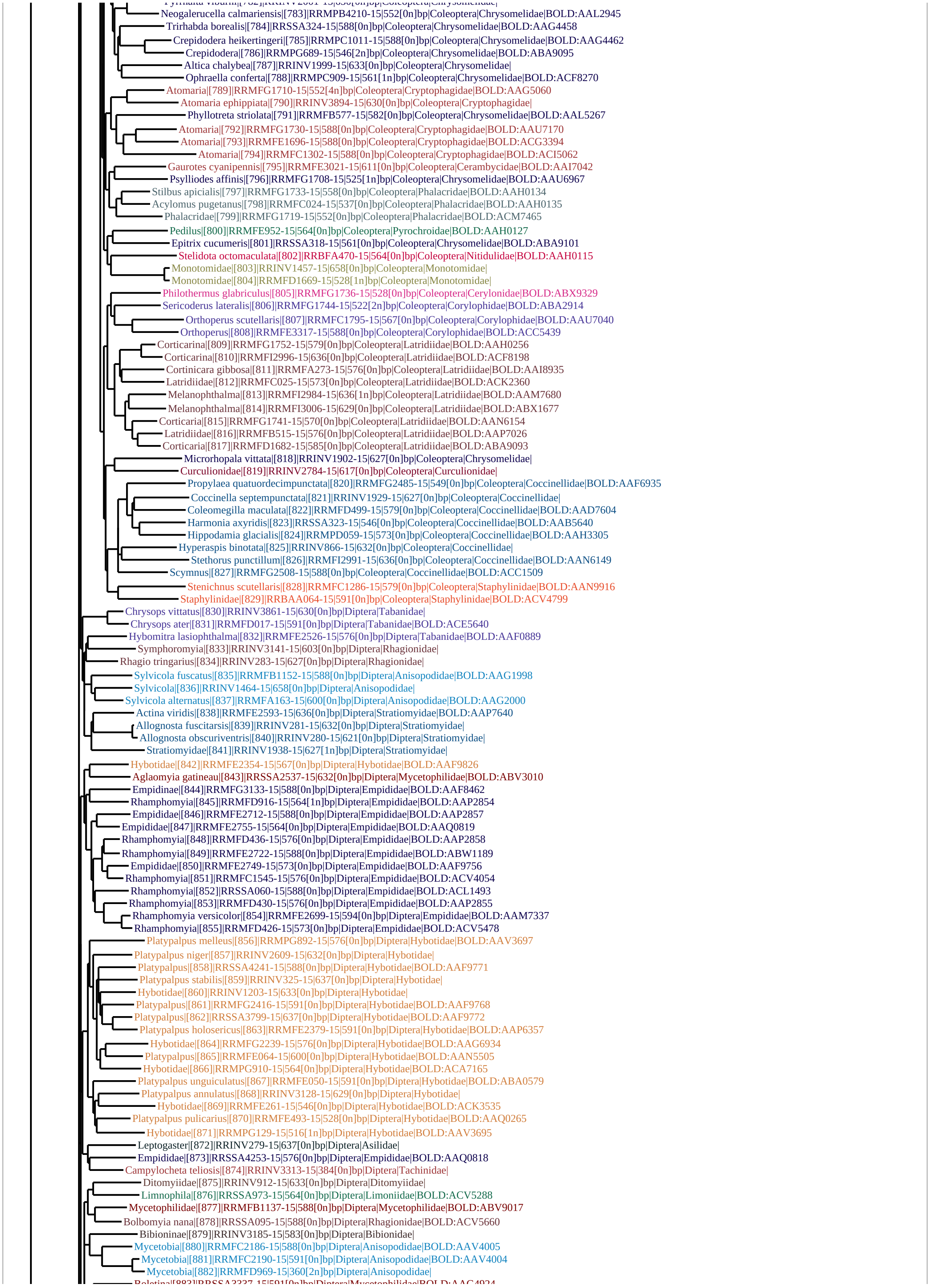

Neogalerucella calmariensis|[783]|RRMPB4210-15|552[0n]bp|Coleoptera|Chrysomelidae|BOLD:AAL2945
Trirhabda borealis|[784]|RRSSA324-15|588[0n]bp|Coleoptera|Chrysomelidae|BOLD:AAG4458
Crepidodera heikertingeri|[785]|RRMPC1011-15|588[0n]bp|Coleoptera|Chrysomelidae|BOLD:AAG4462
Crepidodera|[786]|RRMPG689-15|546[2n]bp|Coleoptera|Chrysomelidae|BOLD:ABA9095
Altica chalybea|[787]|RRINV1999-15|633[0n]bp|Coleoptera|Chrysomelidae|
Ophraella conferta|[788]|RRMPC909-15|561[1n]bp|Coleoptera|Chrysomelidae|BOLD:ACF8270
Atomaria|[789]|RRMFG1710-15|552[4n]bp|Coleoptera|Cryptophagidae|BOLD:AAG5060
Atomaria ephippiata|[790]|RRINV3894-15|630[0n]bp|Coleoptera|Cryptophagidae|
Phyllotreta striolata|[791]|RRMFB577-15|582[0n]bp|Coleoptera|Chrysomelidae|BOLD:AAL5267
Atomaria|[792]|RRMFG1730-15|588[0n]bp|Coleoptera|Cryptophagidae|BOLD:AAU7170
Atomaria|[793]|RRMFE1696-15|588[0n]bp|Coleoptera|Cryptophagidae|BOLD:ACG3394
Atomaria|[794]|RRMFC1302-15|588[0n]bp|Coleoptera|Cryptophagidae|BOLD:ACI5062
Gaurotes cyanipennis|[795]|RRMFE3021-15|611[0n]bp|Coleoptera|Cerambycidae|BOLD:AAI7042
Psylliodes affinis|[796]|RRMFG1708-15|525[1n]bp|Coleoptera|Chrysomelidae|BOLD:AAU6967
Stilbus apicialis|[797]|RRMFG1733-15|558[0n]bp|Coleoptera|Phalacridae|BOLD:AAH0134
Acylomus pugetanus|[798]|RRMFC024-15|537[0n]bp|Coleoptera|Phalacridae|BOLD:AAH0135
Phalacridae|[799]|RRMFG1719-15|552[0n]bp|Coleoptera|Phalacridae|BOLD:ACM7465
Pedilus|[800]|RRMFE952-15|564[0n]bp|Coleoptera|Pyrochroidae|BOLD:AAH0127
Epitrix cucumeris|[801]|RRSSA318-15|561[0n]bp|Coleoptera|Chrysomelidae|BOLD:ABA9101
Stelidota octomaculata|[802]|RRBFA470-15|564[0n]bp|Coleoptera|Nitidulidae|BOLD:AAH0115
Monotomidae|[803]|RRINV1457-15|658[0n]bp|Coleoptera|Monotomidae|
Monotomidae|[804]|RRMFD1669-15|528[1n]bp|Coleoptera|Monotomidae|
Philothermus glabriculus|[805]|RRMFG1736-15|528[0n]bp|Coleoptera|Cerylonidae|BOLD:ABX9329
Sericoderus lateralis|[806]|RRMFG1744-15|522[2n]bp|Coleoptera|Corylophidae|BOLD:ABA2914
Orthoperus scutellaris|[807]|RRMFC1795-15|567[0n]bp|Coleoptera|Corylophidae|BOLD:AAU7040
Orthoperus|[808]|RRMFE3317-15|588[0n]bp|Coleoptera|Corylophidae|BOLD:ACC5439
Corticarina|[809]|RRMFG1752-15|579[0n]bp|Coleoptera|Latridiidae|BOLD:AAH0256
Corticarina|[810]|RRMFI2996-15|636[0n]bp|Coleoptera|Latridiidae|BOLD:ACF8198
Cortinicara gibbosa|[811]|RRMFA273-15|576[0n]bp|Coleoptera|Latridiidae|BOLD:AAI8935
Latridiidae|[812]|RRMFC025-15|573[0n]bp|Coleoptera|Latridiidae|BOLD:ACK2360
Melanophthalma|[813]|RRMFI2984-15|636[1n]bp|Coleoptera|Latridiidae|BOLD:AAM7680
Melanophthalma|[814]|RRMFI3006-15|629[0n]bp|Coleoptera|Latridiidae|BOLD:ABX1677
Corticaria|[815]|RRMFG1741-15|570[0n]bp|Coleoptera|Latridiidae|BOLD:AAN6154
Latridiidae|[816]|RRMFB515-15|576[0n]bp|Coleoptera|Latridiidae|BOLD:AAP7026
Corticaria|[817]|RRMFD1682-15|585[0n]bp|Coleoptera|Latridiidae|BOLD:ABA9093
Microrhopala vittata|[818]|RRINV1902-15|627[0n]bp|Coleoptera|Chrysomelidae|
Curculionidae|[819]|RRINV2784-15|617[0n]bp|Coleoptera|Curculionidae|
Propylaea quatuordecimpunctata|[820]|RRMFG2485-15|549[0n]bp|Coleoptera|Coccinellidae|BOLD:AAF6935
Coccinella septempunctata|[821]|RRINV1929-15|627[0n]bp|Coleoptera|Coccinellidae|
Coleomegilla maculata|[822]|RRMFD499-15|579[0n]bp|Coleoptera|Coccinellidae|BOLD:AAD7604
Harmonia axyridis|[823]|RRSSA323-15|546[0n]bp|Coleoptera|Coccinellidae|BOLD:AAB5640
Hippodamia glacialis|[824]|RRMPD059-15|573[0n]bp|Coleoptera|Coccinellidae|BOLD:AAH3305
Hyperaspis binotata|[825]|RRINV866-15|632[0n]bp|Coleoptera|Coccinellidae|
Stethorus punctillum|[826]|RRMFI2991-15|636[0n]bp|Coleoptera|Coccinellidae|BOLD:AAN6149
Scymnus|[827]|RRMFG2508-15|588[0n]bp|Coleoptera|Coccinellidae|BOLD:ACC1509
Stenichnus scutellaris|[828]|RRMFC1286-15|579[0n]bp|Coleoptera|Staphylinidae|BOLD:AAN9916
Staphylinidae|[829]|RRBAA064-15|591[0n]bp|Coleoptera|Staphylinidae|BOLD:ACV4799
Chrysops vittatus|[830]|RRINV3861-15|630[0n]bp|Diptera|Tabanidae|
Chrysops ater|[831]|RRMFD017-15|591[0n]bp|Diptera|Tabanidae|BOLD:ACE5640
Hybomitra lasiophthalma|[832]|RRMFE2526-15|576[0n]bp|Diptera|Tabanidae|BOLD:AAF0889
Symphoromyia|[833]|RRINV3141-15|603[0n]bp|Diptera|Rhagionidae|
Rhagio tringarius|[834]|RRINV283-15|627[0n]bp|Diptera|Rhagionidae|
Sylvicola fuscatus|[835]|RRMFB1152-15|588[0n]bp|Diptera|Anisopodidae|BOLD:AAG1998
Sylvicola|[836]|RRINV1464-15|658[0n]bp|Diptera|Anisopodidae|
Sylvicola alternatus|[837]|RRMFA163-15|600[0n]bp|Diptera|Anisopodidae|BOLD:AAG2000
Actina viridis|[838]|RRMFE2593-15|636[0n]bp|Diptera|Stratiomyidae|BOLD:AAP7640
Allognosta fuscitarsis|[839]|RRINV281-15|632[0n]bp|Diptera|Stratiomyidae|
Allognosta obscuriventris|[840]|RRINV280-15|621[0n]bp|Diptera|Stratiomyidae|
Stratiomyidae|[841]|RRINV1938-15|627[1n]bp|Diptera|Stratiomyidae|
Hybotidae|[842]|RRMFE2354-15|567[0n]bp|Diptera|Hybotidae|BOLD:AAF9826
Aglaomyia gatineau|[843]|RRSSA2537-15|632[0n]bp|Diptera|Mycetophilidae|BOLD:ABV3010
Empidinae|[844]|RRMFG3133-15|588[0n]bp|Diptera|Empididae|BOLD:AAF8462
Rhamphomyia|[845]|RRMFD916-15|564[1n]bp|Diptera|Empididae|BOLD:AAP2854
Empididae|[846]|RRMFE2712-15|588[0n]bp|Diptera|Empididae|BOLD:AAP2857
Empididae|[847]|RRMFE2755-15|564[0n]bp|Diptera|Empididae|BOLD:AAQ0819
Rhamphomyia|[848]|RRMFD436-15|576[0n]bp|Diptera|Empididae|BOLD:AAP2858
Rhamphomyia|[849]|RRMFE2722-15|588[0n]bp|Diptera|Empididae|BOLD:ABW1189
Empididae|[850]|RRMFE2749-15|573[0n]bp|Diptera|Empididae|BOLD:AAF9756
Rhamphomyia|[851]|RRMFC1545-15|576[0n]bp|Diptera|Empididae|BOLD:ACV4054
Rhamphomyia|[852]|RRSSA060-15|588[0n]bp|Diptera|Empididae|BOLD:ACL1493
Rhamphomyia|[853]|RRMFD430-15|576[0n]bp|Diptera|Empididae|BOLD:AAP2855
Rhamphomyia versicolor|[854]|RRMFE2699-15|594[0n]bp|Diptera|Empididae|BOLD:AAM7337
Rhamphomyia|[855]|RRMFD426-15|573[0n]bp|Diptera|Empididae|BOLD:ACV5478
Platypalpus melleus|[856]|RRMPG892-15|576[0n]bp|Diptera|Hybotidae|BOLD:AAV3697
Platypalpus niger|[857]|RRINV2609-15|632[0n]bp|Diptera|Hybotidae|
Platypalpus|[858]|RRSSA4241-15|588[0n]bp|Diptera|Hybotidae|BOLD:AAF9771
Platypalpus stabilis|[859]|RRINV325-15|637[0n]bp|Diptera|Hybotidae|
Hybotidae|[860]|RRINV1203-15|633[0n]bp|Diptera|Hybotidae|
Platypalpus|[861]|RRMFG2416-15|591[0n]bp|Diptera|Hybotidae|BOLD:AAF9768
Platypalpus|[862]|RRSSA3799-15|637[0n]bp|Diptera|Hybotidae|BOLD:AAF9772
Platypalpus holosericus|[863]|RRMFE2379-15|591[0n]bp|Diptera|Hybotidae|BOLD:AAP6357
Hybotidae|[864]|RRMFG2239-15|576[0n]bp|Diptera|Hybotidae|BOLD:AAG6934
Platypalpus|[865]|RRMFE064-15|600[0n]bp|Diptera|Hybotidae|BOLD:AAN5505
Hybotidae|[866]|RRMPG910-15|564[0n]bp|Diptera|Hybotidae|BOLD:ACA7165
Platypalpus unguiculatus|[867]|RRMFE050-15|591[0n]bp|Diptera|Hybotidae|BOLD:ABA0579
Platypalpus annulatus|[868]|RRINV3128-15|629[0n]bp|Diptera|Hybotidae|
Hybotidae|[869]|RRMFE261-15|546[0n]bp|Diptera|Hybotidae|BOLD:ACK3535
Platypalpus pulicarius|[870]|RRMFE493-15|528[0n]bp|Diptera|Hybotidae|BOLD:AAQ0265
Hybotidae|[871]|RRMPG129-15|516[1n]bp|Diptera|Hybotidae|BOLD:AAV3695
Leptogaster|[872]|RRINV279-15|637[0n]bp|Diptera|Asilidae|
Empididae|[873]|RRSSA4253-15|576[0n]bp|Diptera|Empididae|BOLD:AAQ0818
Campylocheta teliosis|[874]|RRINV3313-15|384[0n]bp|Diptera|Tachinidae|
Ditomyiidae|[875]|RRINV912-15|633[0n]bp|Diptera|Ditomyiidae|
Limnophila|[876]|RRSSA973-15|564[0n]bp|Diptera|Limoniidae|BOLD:ACV5288
Mycetophilidae|[877]|RRMFB1137-15|588[0n]bp|Diptera|Mycetophilidae|BOLD:ABV9017
Bolbomyia nana|[878]|RRSSA095-15|588[0n]bp|Diptera|Rhagionidae|BOLD:ACV5660
Bibioninae|[879]|RRINV3185-15|583[0n]bp|Diptera|Bibionidae|
Mycetobia|[880]|RRMFC2186-15|588[0n]bp|Diptera|Anisopodidae|BOLD:AAV4005
Mycetobia|[881]|RRMFC2190-15|591[0n]bp|Diptera|Anisopodidae|BOLD:AAV4004
Mycetobia|[882]|RRMFD969-15|360[2n]bp|Diptera|Anisopodidae|

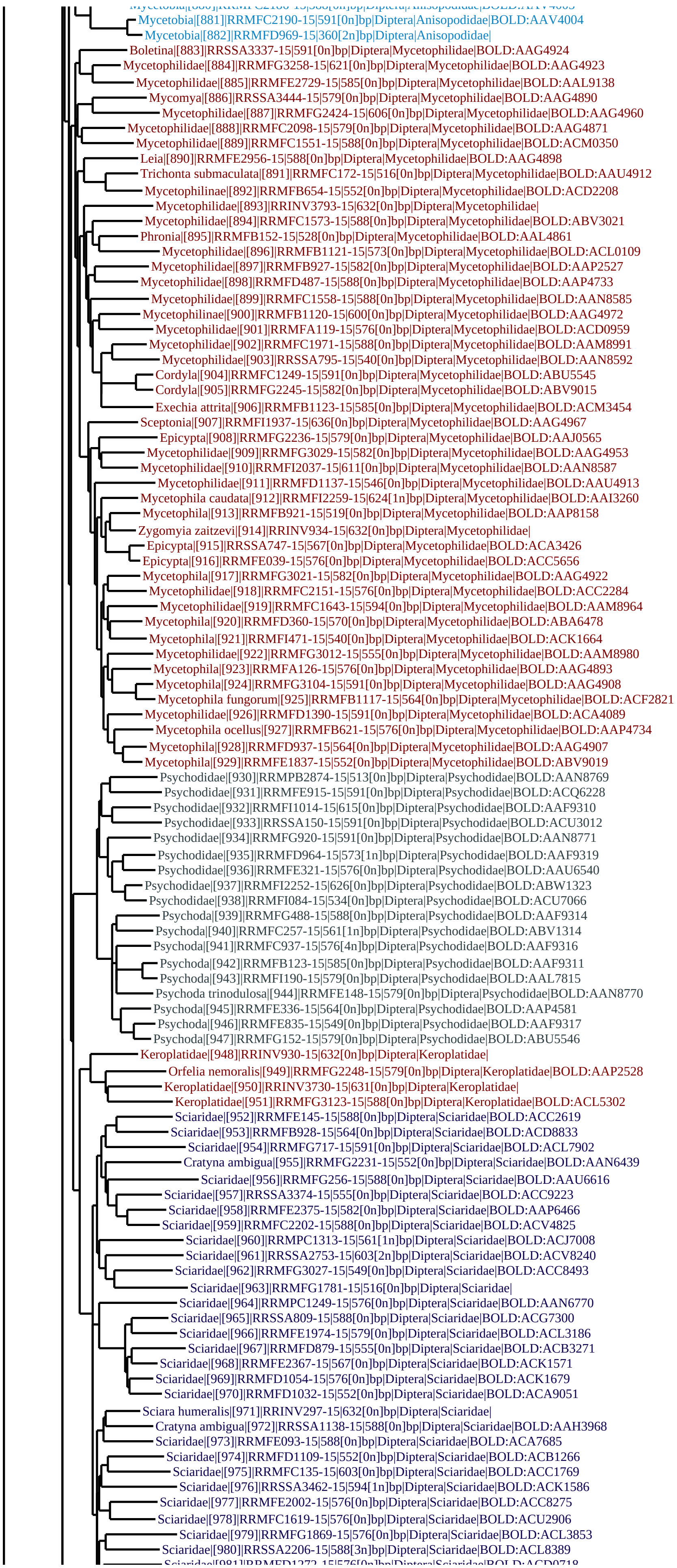
Mycetobia|[881]|RRMFC2190-15|591[0n]bp|Diptera|Anisopodidae|BOLD:AAV4004
Mycetobia|[882]|RRMFD969-15|360[2n]bp|Diptera|Anisopodidae|
Boletina|[883]|RRSSA3337-15|591[0n]bp|Diptera|Mycetophilidae|BOLD:AAG4924
Mycetophilidae|[884]|RRMFG3258-15|621[0n]bp|Diptera|Mycetophilidae|BOLD:AAG4923
Mycetophilidae|[885]|RRMFE2729-15|585[0n]bp|Diptera|Mycetophilidae|BOLD:AAL9138
Mycomya|[886]|RRSSA3444-15|579[0n]bp|Diptera|Mycetophilidae|BOLD:AAG4890
Mycetophilidae|[887]|RRMFG2424-15|606[0n]bp|Diptera|Mycetophilidae|BOLD:AAG4960
Mycetophilidae|[888]|RRMFC2098-15|579[0n]bp|Diptera|Mycetophilidae|BOLD:AAG4871
Mycetophilidae|[889]|RRMFC1551-15|588[0n]bp|Diptera|Mycetophilidae|BOLD:ACM0350
Leia|[890]|RRMFE2956-15|588[0n]bp|Diptera|Mycetophilidae|BOLD:AAG4898
Trichonta submaculata|[891]|RRMFC172-15|516[0n]bp|Diptera|Mycetophilidae|BOLD:AAU4912
Mycetophilinae|[892]|RRMFB654-15|552[0n]bp|Diptera|Mycetophilidae|BOLD:ACD2208
Mycetophilidae|[893]|RRINV3793-15|632[0n]bp|Diptera|Mycetophilidae|
Mycetophilidae|[894]|RRMFC1573-15|588[0n]bp|Diptera|Mycetophilidae|BOLD:ABV3021
Phronia|[895]|RRMFB152-15|528[0n]bp|Diptera|Mycetophilidae|BOLD:AAL4861
Mycetophilidae|[896]|RRMFB1121-15|573[0n]bp|Diptera|Mycetophilidae|BOLD:ACL0109
Mycetophilidae|[897]|RRMFB927-15|582[0n]bp|Diptera|Mycetophilidae|BOLD:AAP2527
Mycetophilidae|[898]|RRMFD487-15|588[0n]bp|Diptera|Mycetophilidae|BOLD:AAP4733
Mycetophilidae|[899]|RRMFC1558-15|588[0n]bp|Diptera|Mycetophilidae|BOLD:AAN8585
Mycetophilinae|[900]|RRMFB1120-15|600[0n]bp|Diptera|Mycetophilidae|BOLD:AAG4972
Mycetophilidae|[901]|RRMFA119-15|576[0n]bp|Diptera|Mycetophilidae|BOLD:ACD0959
Mycetophilidae|[902]|RRMFC1971-15|588[0n]bp|Diptera|Mycetophilidae|BOLD:AAM8991
Mycetophilidae|[903]|RRSSA795-15|540[0n]bp|Diptera|Mycetophilidae|BOLD:AAN8592
Cordyla|[904]|RRMFC1249-15|591[0n]bp|Diptera|Mycetophilidae|BOLD:ABU5545
Cordyla|[905]|RRMFG2245-15|582[0n]bp|Diptera|Mycetophilidae|BOLD:ABV9015
Exechia attrita|[906]|RRMFB1123-15|585[0n]bp|Diptera|Mycetophilidae|BOLD:ACM3454
Sceptonia|[907]|RRMFI1937-15|636[0n]bp|Diptera|Mycetophilidae|BOLD:AAG4967
Epicypta|[908]|RRMFG2236-15|579[0n]bp|Diptera|Mycetophilidae|BOLD:AAJ0565
Mycetophilidae|[909]|RRMFG3029-15|582[0n]bp|Diptera|Mycetophilidae|BOLD:AAG4953
Mycetophilidae|[910]|RRMFI2037-15|611[0n]bp|Diptera|Mycetophilidae|BOLD:AAN8587
Mycetophilidae|[911]|RRMFD1137-15|546[0n]bp|Diptera|Mycetophilidae|BOLD:AAU4913
Mycetophila caudata|[912]|RRMFI2259-15|624[1n]bp|Diptera|Mycetophilidae|BOLD:AAI3260
Mycetophila|[913]|RRMFB921-15|519[0n]bp|Diptera|Mycetophilidae|BOLD:AAP8158
Zygomyia zaitzevi|[914]|RRINV934-15|632[0n]bp|Diptera|Mycetophilidae|
Epicypta|[915]|RRSSA747-15|567[0n]bp|Diptera|Mycetophilidae|BOLD:ACA3426
Epicypta|[916]|RRMFE039-15|576[0n]bp|Diptera|Mycetophilidae|BOLD:ACC5656
Mycetophila|[917]|RRMFG3021-15|582[0n]bp|Diptera|Mycetophilidae|BOLD:AAG4922
Mycetophilidae|[918]|RRMFC2151-15|576[0n]bp|Diptera|Mycetophilidae|BOLD:ACC2284
Mycetophilidae|[919]|RRMFC1643-15|594[0n]bp|Diptera|Mycetophilidae|BOLD:AAM8964
Mycetophila|[920]|RRMFD360-15|570[0n]bp|Diptera|Mycetophilidae|BOLD:ABA6478
Mycetophila|[921]|RRMFI471-15|540[0n]bp|Diptera|Mycetophilidae|BOLD:ACK1664
Mycetophilidae|[922]|RRMFG3012-15|555[0n]bp|Diptera|Mycetophilidae|BOLD:AAM8980
Mycetophila|[923]|RRMFA126-15|576[0n]bp|Diptera|Mycetophilidae|BOLD:AAG4893
Mycetophila|[924]|RRMFG3104-15|591[0n]bp|Diptera|Mycetophilidae|BOLD:AAG4908
Mycetophila fungorum|[925]|RRMFB1117-15|564[0n]bp|Diptera|Mycetophilidae|BOLD:ACF2821
Mycetophilidae|[926]|RRMFD1390-15|591[0n]bp|Diptera|Mycetophilidae|BOLD:ACA4089
Mycetophila ocellus|[927]|RRMFB621-15|576[0n]bp|Diptera|Mycetophilidae|BOLD:AAP4734
Mycetophila|[928]|RRMFD937-15|564[0n]bp|Diptera|Mycetophilidae|BOLD:AAG4907
Mycetophila|[929]|RRMFE1837-15|552[0n]bp|Diptera|Mycetophilidae|BOLD:ABV9019
Psychodidae|[930]|RRMPB2874-15|513[0n]bp|Diptera|Psychodidae|BOLD:AAN8769
Psychodidae|[931]|RRMFE915-15|591[0n]bp|Diptera|Psychodidae|BOLD:ACQ6228
Psychodidae|[932]|RRMFI1014-15|615[0n]bp|Diptera|Psychodidae|BOLD:AAF9310
Psychodidae|[933]|RRSSA150-15|591[0n]bp|Diptera|Psychodidae|BOLD:ACU3012
Psychodidae|[934]|RRMFG920-15|591[0n]bp|Diptera|Psychodidae|BOLD:AAN8771
Psychodidae|[935]|RRMFD964-15|573[1n]bp|Diptera|Psychodidae|BOLD:AAF9319
Psychodidae|[936]|RRMFE321-15|576[0n]bp|Diptera|Psychodidae|BOLD:AAU6540
Psychodidae|[937]|RRMFI2252-15|626[0n]bp|Diptera|Psychodidae|BOLD:ABW1323
Psychodidae|[938]|RRMFI084-15|534[0n]bp|Diptera|Psychodidae|BOLD:ACU7066
Psychoda|[939]|RRMFG488-15|588[0n]bp|Diptera|Psychodidae|BOLD:AAF9314
Psychoda|[940]|RRMFC257-15|561[1n]bp|Diptera|Psychodidae|BOLD:ABV1314
Psychoda|[941]|RRMFC937-15|576[4n]bp|Diptera|Psychodidae|BOLD:AAF9316
Psychoda|[942]|RRMFB123-15|585[0n]bp|Diptera|Psychodidae|BOLD:AAF9311
Psychoda|[943]|RRMFI190-15|579[0n]bp|Diptera|Psychodidae|BOLD:AAL7815
Psychoda trinodulosa|[944]|RRMFE148-15|579[0n]bp|Diptera|Psychodidae|BOLD:AAN8770
Psychoda|[945]|RRMFE336-15|564[0n]bp|Diptera|Psychodidae|BOLD:AAP4581
Psychoda|[946]|RRMFE835-15|549[0n]bp|Diptera|Psychodidae|BOLD:AAF9317
Psychoda|[947]|RRMFG152-15|579[0n]bp|Diptera|Psychodidae|BOLD:ABU5546
Keroplatidae|[948]|RRINV930-15|632[0n]bp|Diptera|Keroplatidae|
Orfelia nemoralis|[949]|RRMFG2248-15|579[0n]bp|Diptera|Keroplatidae|BOLD:AAP2528
Keroplatidae|[950]|RRINV3730-15|631[0n]bp|Diptera|Keroplatidae|
Keroplatidae|[951]|RRMFG3123-15|588[0n]bp|Diptera|Keroplatidae|BOLD:ACL5302
Sciaridae|[952]|RRMFE145-15|588[0n]bp|Diptera|Sciaridae|BOLD:ACC2619
Sciaridae|[953]|RRMFB928-15|564[0n]bp|Diptera|Sciaridae|BOLD:ACD8833
Sciaridae|[954]|RRMFG717-15|591[0n]bp|Diptera|Sciaridae|BOLD:ACL7902
Cratyna ambigua|[955]|RRMFG2231-15|552[0n]bp|Diptera|Sciaridae|BOLD:AAN6439
Sciaridae|[956]|RRMFG256-15|588[0n]bp|Diptera|Sciaridae|BOLD:AAU6616
Sciaridae|[957]|RRSSA3374-15|555[0n]bp|Diptera|Sciaridae|BOLD:ACC9223
Sciaridae|[958]|RRMFE2375-15|582[0n]bp|Diptera|Sciaridae|BOLD:AAP6466
Sciaridae|[959]|RRMFC2202-15|588[0n]bp|Diptera|Sciaridae|BOLD:ACV4825
Sciaridae|[960]|RRMPC1313-15|561[1n]bp|Diptera|Sciaridae|BOLD:ACJ7008
Sciaridae|[961]|RRSSA2753-15|603[2n]bp|Diptera|Sciaridae|BOLD:ACV8240
Sciaridae|[962]|RRMFG3027-15|549[0n]bp|Diptera|Sciaridae|BOLD:ACC8493
Sciaridae|[963]|RRMFG1781-15|516[0n]bp|Diptera|Sciaridae|
Sciaridae|[964]|RRMPC1249-15|576[0n]bp|Diptera|Sciaridae|BOLD:AAN6770
Sciaridae|[965]|RRSSA809-15|588[0n]bp|Diptera|Sciaridae|BOLD:ACG7300
Sciaridae|[966]|RRMFE1974-15|579[0n]bp|Diptera|Sciaridae|BOLD:ACL3186
Sciaridae|[967]|RRMFD879-15|555[0n]bp|Diptera|Sciaridae|BOLD:ACB3271
Sciaridae|[968]|RRMFE2367-15|567[0n]bp|Diptera|Sciaridae|BOLD:ACK1571
Sciaridae|[969]|RRMFD1054-15|576[0n]bp|Diptera|Sciaridae|BOLD:ACK1679
Sciaridae|[970]|RRMFD1032-15|552[0n]bp|Diptera|Sciaridae|BOLD:ACA9051
Sciara humeralis|[971]|RRINV297-15|632[0n]bp|Diptera|Sciaridae|
Cratyna ambigua|[972]|RRSSA1138-15|588[0n]bp|Diptera|Sciaridae|BOLD:AAH3968
Sciaridae|[973]|RRMFE093-15|588[0n]bp|Diptera|Sciaridae|BOLD:ACA7685
Sciaridae|[974]|RRMFD1109-15|552[0n]bp|Diptera|Sciaridae|BOLD:ACB1266
Sciaridae|[975]|RRMFC135-15|603[0n]bp|Diptera|Sciaridae|BOLD:ACC1769
Sciaridae|[976]|RRSSA3462-15|594[1n]bp|Diptera|Sciaridae|BOLD:ACK1586
Sciaridae|[977]|RRMFE2002-15|576[0n]bp|Diptera|Sciaridae|BOLD:ACC8275
Sciaridae|[978]|RRMFC1619-15|576[0n]bp|Diptera|Sciaridae|BOLD:ACU2906
Sciaridae|[979]|RRMFG1869-15|576[0n]bp|Diptera|Sciaridae|BOLD:ACL3853
Sciaridae|[980]|RRSSA2206-15|588[3n]bp|Diptera|Sciaridae|BOLD:ACL8389

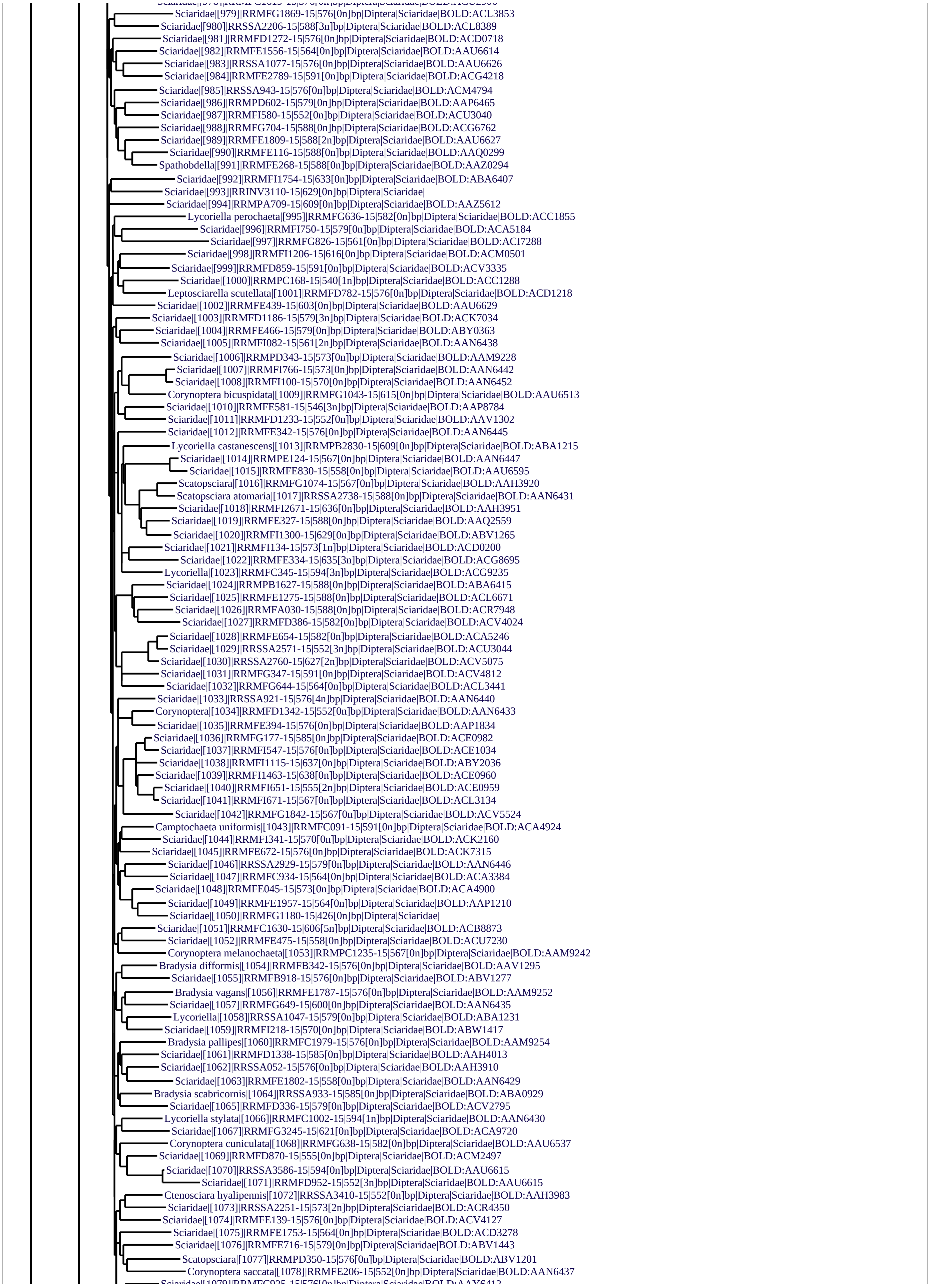

Sciaridae|[979]|RRMFG1869-15|576[0n]bp|Diptera|Sciaridae|BOLD:ACL3853
Sciaridae|[980]|RRSSA2206-15|588[3n]bp|Diptera|Sciaridae|BOLD:ACL8389
Sciaridae|[981]|RRMFD1272-15|576[0n]bp|Diptera|Sciaridae|BOLD:ACD0718
Sciaridae|[982]|RRMFE1556-15|564[0n]bp|Diptera|Sciaridae|BOLD:AAU6614
Sciaridae|[983]|RRSSA1077-15|576[0n]bp|Diptera|Sciaridae|BOLD:AAU6626
Sciaridae|[984]|RRMFE2789-15|591[0n]bp|Diptera|Sciaridae|BOLD:ACG4218
Sciaridae|[985]|RRSSA943-15|576[0n]bp|Diptera|Sciaridae|BOLD:ACM4794
Sciaridae|[986]|RRMPD602-15|579[0n]bp|Diptera|Sciaridae|BOLD:AAP6465
Sciaridae|[987]|RRMFI580-15|552[0n]bp|Diptera|Sciaridae|BOLD:ACU3040
Sciaridae|[988]|RRMFG704-15|588[0n]bp|Diptera|Sciaridae|BOLD:ACG6762
Sciaridae|[989]|RRMFE1809-15|588[2n]bp|Diptera|Sciaridae|BOLD:AAU6627
Sciaridae|[990]|RRMFE116-15|588[0n]bp|Diptera|Sciaridae|BOLD:AAQ0299
Spathobdella|[991]|RRMFE268-15|588[0n]bp|Diptera|Sciaridae|BOLD:AAZ0294
Sciaridae|[992]|RRMFI1754-15|633[0n]bp|Diptera|Sciaridae|BOLD:ABA6407
Sciaridae|[993]|RRINV3110-15|629[0n]bp|Diptera|Sciaridae|
Sciaridae|[994]|RRMPA709-15|609[0n]bp|Diptera|Sciaridae|BOLD:AAZ5612
Lycoriella perochaeta|[995]|RRMFG636-15|582[0n]bp|Diptera|Sciaridae|BOLD:ACC1855
Sciaridae|[996]|RRMFI750-15|579[0n]bp|Diptera|Sciaridae|BOLD:ACA5184
Sciaridae|[997]|RRMFG826-15|561[0n]bp|Diptera|Sciaridae|BOLD:ACI7288
Sciaridae|[998]|RRMFI1206-15|616[0n]bp|Diptera|Sciaridae|BOLD:ACM0501
Sciaridae|[999]|RRMFD859-15|591[0n]bp|Diptera|Sciaridae|BOLD:ACV3335
Sciaridae|[1000]|RRMPC168-15|540[1n]bp|Diptera|Sciaridae|BOLD:ACC1288
Leptosciarella scutellata|[1001]|RRMFD782-15|576[0n]bp|Diptera|Sciaridae|BOLD:ACD1218
Sciaridae|[1002]|RRMFE439-15|603[0n]bp|Diptera|Sciaridae|BOLD:AAU6629
Sciaridae|[1003]|RRMFD1186-15|579[3n]bp|Diptera|Sciaridae|BOLD:ACK7034
Sciaridae|[1004]|RRMFE466-15|579[0n]bp|Diptera|Sciaridae|BOLD:ABY0363
Sciaridae|[1005]|RRMFI082-15|561[2n]bp|Diptera|Sciaridae|BOLD:AAN6438
Sciaridae|[1006]|RRMPD343-15|573[0n]bp|Diptera|Sciaridae|BOLD:AAM9228
Sciaridae|[1007]|RRMFI766-15|573[0n]bp|Diptera|Sciaridae|BOLD:AAN6442
Sciaridae|[1008]|RRMFI100-15|570[0n]bp|Diptera|Sciaridae|BOLD:AAN6452
Corynoptera bicuspidata|[1009]|RRMFG1043-15|615[0n]bp|Diptera|Sciaridae|BOLD:AAU6513
Sciaridae|[1010]|RRMFE581-15|546[3n]bp|Diptera|Sciaridae|BOLD:AAP8784
Sciaridae|[1011]|RRMFD1233-15|552[0n]bp|Diptera|Sciaridae|BOLD:AAV1302
Sciaridae|[1012]|RRMFE342-15|576[0n]bp|Diptera|Sciaridae|BOLD:AAN6445
Lycoriella castanescens|[1013]|RRMPB2830-15|609[0n]bp|Diptera|Sciaridae|BOLD:ABA1215
Sciaridae|[1014]|RRMPE124-15|567[0n]bp|Diptera|Sciaridae|BOLD:AAN6447
Sciaridae|[1015]|RRMFE830-15|558[0n]bp|Diptera|Sciaridae|BOLD:AAU6595
Scatopsciara|[1016]|RRMFG1074-15|567[0n]bp|Diptera|Sciaridae|BOLD:AAH3920
Scatopsciara atomaria|[1017]|RRSSA2738-15|588[0n]bp|Diptera|Sciaridae|BOLD:AAN6431
Sciaridae|[1018]|RRMFI2671-15|636[0n]bp|Diptera|Sciaridae|BOLD:AAH3951
Sciaridae|[1019]|RRMFE327-15|588[0n]bp|Diptera|Sciaridae|BOLD:AAQ2559
Sciaridae|[1020]|RRMFI1300-15|629[0n]bp|Diptera|Sciaridae|BOLD:ABV1265
Sciaridae|[1021]|RRMFI134-15|573[1n]bp|Diptera|Sciaridae|BOLD:ACD0200
Sciaridae|[1022]|RRMFE334-15|635[3n]bp|Diptera|Sciaridae|BOLD:ACG8695
Lycoriella|[1023]|RRMFC345-15|594[3n]bp|Diptera|Sciaridae|BOLD:ACG9235
Sciaridae|[1024]|RRMPB1627-15|588[0n]bp|Diptera|Sciaridae|BOLD:ABA6415
Sciaridae|[1025]|RRMFE1275-15|588[0n]bp|Diptera|Sciaridae|BOLD:ACL6671
Sciaridae|[1026]|RRMFA030-15|588[0n]bp|Diptera|Sciaridae|BOLD:ACR7948
Sciaridae|[1027]|RRMFD386-15|582[0n]bp|Diptera|Sciaridae|BOLD:ACV4024
Sciaridae|[1028]|RRMFE654-15|582[0n]bp|Diptera|Sciaridae|BOLD:ACA5246
Sciaridae|[1029]|RRSSA2571-15|552[3n]bp|Diptera|Sciaridae|BOLD:ACU3044
Sciaridae|[1030]|RRSSA2760-15|627[2n]bp|Diptera|Sciaridae|BOLD:ACV5075
Sciaridae|[1031]|RRMFG347-15|591[0n]bp|Diptera|Sciaridae|BOLD:ACV4812
Sciaridae|[1032]|RRMFG644-15|564[0n]bp|Diptera|Sciaridae|BOLD:ACL3441
Sciaridae|[1033]|RRSSA921-15|576[4n]bp|Diptera|Sciaridae|BOLD:AAN6440
Corynoptera|[1034]|RRMFD1342-15|552[0n]bp|Diptera|Sciaridae|BOLD:AAN6433
Sciaridae|[1035]|RRMFE394-15|576[0n]bp|Diptera|Sciaridae|BOLD:AAP1834
Sciaridae|[1036]|RRMFG177-15|585[0n]bp|Diptera|Sciaridae|BOLD:ACE0982
Sciaridae|[1037]|RRMFI547-15|576[0n]bp|Diptera|Sciaridae|BOLD:ACE1034
Sciaridae|[1038]|RRMFI1115-15|637[0n]bp|Diptera|Sciaridae|BOLD:ABY2036
Sciaridae|[1039]|RRMFI1463-15|638[0n]bp|Diptera|Sciaridae|BOLD:ACE0960
Sciaridae|[1040]|RRMFI651-15|555[2n]bp|Diptera|Sciaridae|BOLD:ACE0959
Sciaridae|[1041]|RRMFI671-15|567[0n]bp|Diptera|Sciaridae|BOLD:ACL3134
Sciaridae|[1042]|RRMFG1842-15|567[0n]bp|Diptera|Sciaridae|BOLD:ACV5524
Camptochaeta uniformis|[1043]|RRMFC091-15|591[0n]bp|Diptera|Sciaridae|BOLD:ACA4924
Sciaridae|[1044]|RRMFI341-15|570[0n]bp|Diptera|Sciaridae|BOLD:ACK2160
Sciaridae|[1045]|RRMFE672-15|576[0n]bp|Diptera|Sciaridae|BOLD:ACK7315
Sciaridae|[1046]|RRSSA2929-15|579[0n]bp|Diptera|Sciaridae|BOLD:AAN6446
Sciaridae|[1047]|RRMFC934-15|564[0n]bp|Diptera|Sciaridae|BOLD:ACA3384
Sciaridae|[1048]|RRMFE045-15|573[0n]bp|Diptera|Sciaridae|BOLD:ACA4900
Sciaridae|[1049]|RRMFE1957-15|564[0n]bp|Diptera|Sciaridae|BOLD:AAP1210
Sciaridae|[1050]|RRMFG1180-15|426[0n]bp|Diptera|Sciaridae|
Sciaridae|[1051]|RRMFC1630-15|606[5n]bp|Diptera|Sciaridae|BOLD:ACB8873
Sciaridae|[1052]|RRMFE475-15|558[0n]bp|Diptera|Sciaridae|BOLD:ACU7230
Corynoptera melanochaeta|[1053]|RRMPC1235-15|567[0n]bp|Diptera|Sciaridae|BOLD:AAM9242
Bradysia difformis|[1054]|RRMFB342-15|576[0n]bp|Diptera|Sciaridae|BOLD:AAV1295
Sciaridae|[1055]|RRMFB918-15|576[0n]bp|Diptera|Sciaridae|BOLD:ABV1277
Bradysia vagans|[1056]|RRMFE1787-15|576[0n]bp|Diptera|Sciaridae|BOLD:AAM9252
Sciaridae|[1057]|RRMFG649-15|600[0n]bp|Diptera|Sciaridae|BOLD:AAN6435
Lycoriella|[1058]|RRSSA1047-15|579[0n]bp|Diptera|Sciaridae|BOLD:ABA1231
Sciaridae|[1059]|RRMFI218-15|570[0n]bp|Diptera|Sciaridae|BOLD:ABW1417
Bradysia pallipes|[1060]|RRMFC1979-15|576[0n]bp|Diptera|Sciaridae|BOLD:AAM9254
Sciaridae|[1061]|RRMFD1338-15|585[0n]bp|Diptera|Sciaridae|BOLD:AAH4013
Sciaridae|[1062]|RRSSA052-15|576[0n]bp|Diptera|Sciaridae|BOLD:AAH3910
Sciaridae|[1063]|RRMFE1802-15|558[0n]bp|Diptera|Sciaridae|BOLD:AAN6429
Bradysia scabricornis|[1064]|RRSSA933-15|585[0n]bp|Diptera|Sciaridae|BOLD:ABA0929
Sciaridae|[1065]|RRMFD336-15|579[0n]bp|Diptera|Sciaridae|BOLD:ACV2795
Lycoriella stylata|[1066]|RRMFC1002-15|594[1n]bp|Diptera|Sciaridae|BOLD:AAN6430
Sciaridae|[1067]|RRMFG3245-15|621[0n]bp|Diptera|Sciaridae|BOLD:ACA9720
Corynoptera cuniculata|[1068]|RRMFG638-15|582[0n]bp|Diptera|Sciaridae|BOLD:AAU6537
Sciaridae|[1069]|RRMFD870-15|555[0n]bp|Diptera|Sciaridae|BOLD:ACM2497
Sciaridae|[1070]|RRSSA3586-15|594[0n]bp|Diptera|Sciaridae|BOLD:AAU6615
Sciaridae|[1071]|RRMFD952-15|552[3n]bp|Diptera|Sciaridae|BOLD:AAU6615
Ctenosciara hyalipennis|[1072]|RRSSA3410-15|552[0n]bp|Diptera|Sciaridae|BOLD:AAH3983
Sciaridae|[1073]|RRSSA2251-15|573[2n]bp|Diptera|Sciaridae|BOLD:ACR4350
Sciaridae|[1074]|RRMFE139-15|576[0n]bp|Diptera|Sciaridae|BOLD:ACV4127
Sciaridae|[1075]|RRMFE1753-15|564[0n]bp|Diptera|Sciaridae|BOLD:ACD3278
Sciaridae|[1076]|RRMFE716-15|579[0n]bp|Diptera|Sciaridae|BOLD:ABV1443
Scatopsciara|[1077]|RRMPD350-15|576[0n]bp|Diptera|Sciaridae|BOLD:ABV1201
Corynoptera saccata|[1078]|RRMFE206-15|552[0n]bp|Diptera|Sciaridae|BOLD:AAN6437

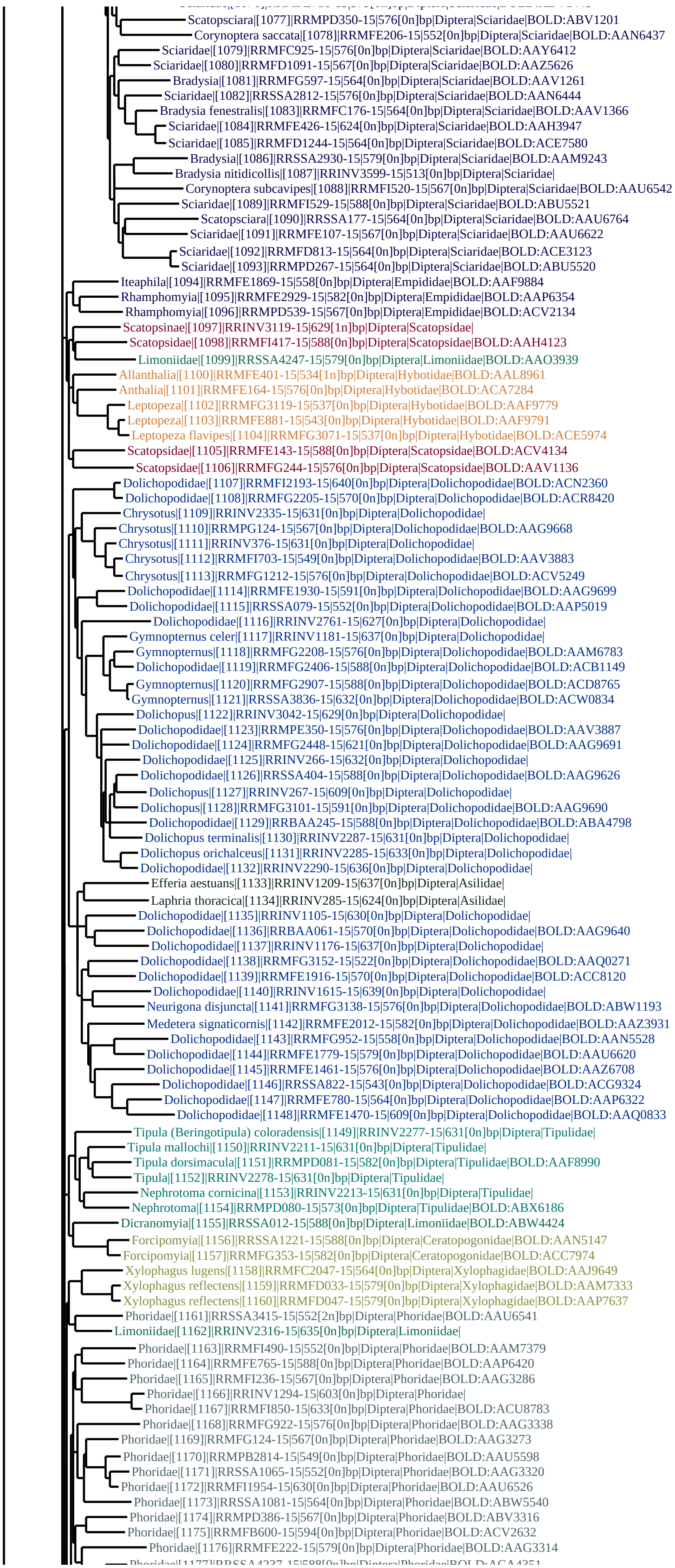

Scatopsciara|[1077]|RRMPD350-15|576[0n]bp|Diptera|Sciaridae|BOLD:ABV1201
Corynoptera saccata|[1078]|RRMFE206-15|552[0n]bp|Diptera|Sciaridae|BOLD:AAN6437
Sciaridae|[1079]|RRMFC925-15|576[0n]bp|Diptera|Sciaridae|BOLD:AAY6412
Sciaridae|[1080]|RRMFD1091-15|567[0n]bp|Diptera|Sciaridae|BOLD:AAZ5626
Bradysia|[1081]|RRMFG597-15|564[0n]bp|Diptera|Sciaridae|BOLD:AAV1261
Sciaridae|[1082]|RRSSA2812-15|576[0n]bp|Diptera|Sciaridae|BOLD:AAN6444
Bradysia fenestralis|[1083]|RRMFC176-15|564[0n]bp|Diptera|Sciaridae|BOLD:AAV1366
Sciaridae|[1084]|RRMFE426-15|624[0n]bp|Diptera|Sciaridae|BOLD:AAH3947
Sciaridae|[1085]|RRMFD1244-15|564[0n]bp|Diptera|Sciaridae|BOLD:ACE7580
Bradysia|[1086]|RRSSA2930-15|579[0n]bp|Diptera|Sciaridae|BOLD:AAM9243
Bradysia nitidicollis|[1087]|RRINV3599-15|513[0n]bp|Diptera|Sciaridae|
Corynoptera subcavipes|[1088]|RRMFI520-15|567[0n]bp|Diptera|Sciaridae|BOLD:AAU6542
Sciaridae|[1089]|RRMFI529-15|588[0n]bp|Diptera|Sciaridae|BOLD:ABU5521
Scatopsciara|[1090]|RRSSA177-15|564[0n]bp|Diptera|Sciaridae|BOLD:AAU6764
Sciaridae|[1091]|RRMFE107-15|567[0n]bp|Diptera|Sciaridae|BOLD:AAU6622
Sciaridae|[1092]|RRMFD813-15|564[0n]bp|Diptera|Sciaridae|BOLD:ACE3123
Sciaridae|[1093]|RRMPD267-15|564[0n]bp|Diptera|Sciaridae|BOLD:ABU5520
Iteaphila|[1094]|RRMFE1869-15|558[0n]bp|Diptera|Empididae|BOLD:AAF9884
Rhamphomyia|[1095]|RRMFE2929-15|582[0n]bp|Diptera|Empididae|BOLD:AAP6354
Rhamphomyia|[1096]|RRMPD539-15|567[0n]bp|Diptera|Empididae|BOLD:ACV2134
Scatopsinae|[1097]|RRINV3119-15|629[1n]bp|Diptera|Scatopsidae|
Scatopsidae|[1098]|RRMFI417-15|588[0n]bp|Diptera|Scatopsidae|BOLD:AAH4123
Limoniidae|[1099]|RRSSA4247-15|579[0n]bp|Diptera|Limoniidae|BOLD:AAO3939
Allanthalia|[1100]|RRMFE401-15|534[1n]bp|Diptera|Hybotidae|BOLD:AAL8961
Anthalia|[1101]|RRMFE164-15|576[0n]bp|Diptera|Hybotidae|BOLD:ACA7284
Leptopeza|[1102]|RRMFG3119-15|537[0n]bp|Diptera|Hybotidae|BOLD:AAF9779
Leptopeza|[1103]|RRMFE881-15|543[0n]bp|Diptera|Hybotidae|BOLD:AAF9791
Leptopeza flavipes|[1104]|RRMFG3071-15|537[0n]bp|Diptera|Hybotidae|BOLD:ACE5974
Scatopsidae|[1105]|RRMFE143-15|588[0n]bp|Diptera|Scatopsidae|BOLD:ACV4134
Scatopsidae|[1106]|RRMFG244-15|576[0n]bp|Diptera|Scatopsidae|BOLD:AAV1136
Dolichopodidae|[1107]|RRMFI2193-15|640[0n]bp|Diptera|Dolichopodidae|BOLD:ACN2360
Dolichopodidae|[1108]|RRMFG2205-15|570[0n]bp|Diptera|Dolichopodidae|BOLD:ACR8420
Chrysotus|[1109]|RRINV2335-15|631[0n]bp|Diptera|Dolichopodidae|
Chrysotus|[1110]|RRMPG124-15|567[0n]bp|Diptera|Dolichopodidae|BOLD:AAG9668
Chrysotus|[1111]|RRINV376-15|631[0n]bp|Diptera|Dolichopodidae|
Chrysotus|[1112]|RRMFI703-15|549[0n]bp|Diptera|Dolichopodidae|BOLD:AAV3883
Chrysotus|[1113]|RRMFG1212-15|576[0n]bp|Diptera|Dolichopodidae|BOLD:ACV5249
Dolichopodidae|[1114]|RRMFE1930-15|591[0n]bp|Diptera|Dolichopodidae|BOLD:AAG9699
Dolichopodidae|[1115]|RRSSA079-15|552[0n]bp|Diptera|Dolichopodidae|BOLD:AAP5019
Dolichopodidae|[1116]|RRINV2761-15|627[0n]bp|Diptera|Dolichopodidae|
Gymnopternus celer|[1117]|RRINV1181-15|637[0n]bp|Diptera|Dolichopodidae|
Gymnopternus|[1118]|RRMFG2208-15|576[0n]bp|Diptera|Dolichopodidae|BOLD:AAM6783
Dolichopodidae|[1119]|RRMFG2406-15|588[0n]bp|Diptera|Dolichopodidae|BOLD:ACB1149
Gymnopternus|[1120]|RRMFG2907-15|588[0n]bp|Diptera|Dolichopodidae|BOLD:ACD8765
Gymnopternus|[1121]|RRSSA3836-15|632[0n]bp|Diptera|Dolichopodidae|BOLD:ACW0834
Dolichopus|[1122]|RRINV3042-15|629[0n]bp|Diptera|Dolichopodidae|
Dolichopodidae|[1123]|RRMPE350-15|576[0n]bp|Diptera|Dolichopodidae|BOLD:AAV3887
Dolichopodidae|[1124]|RRMFG2448-15|621[0n]bp|Diptera|Dolichopodidae|BOLD:AAG9691
Dolichopodidae|[1125]|RRINV266-15|632[0n]bp|Diptera|Dolichopodidae|
Dolichopodidae|[1126]|RRSSA404-15|588[0n]bp|Diptera|Dolichopodidae|BOLD:AAG9626
Dolichopus|[1127]|RRINV267-15|609[0n]bp|Diptera|Dolichopodidae|
Dolichopus|[1128]|RRMFG3101-15|591[0n]bp|Diptera|Dolichopodidae|BOLD:AAG9690
Dolichopodidae|[1129]|RRBAA245-15|588[0n]bp|Diptera|Dolichopodidae|BOLD:ABA4798
Dolichopus terminalis|[1130]|RRINV2287-15|631[0n]bp|Diptera|Dolichopodidae|
Dolichopus orichalceus|[1131]|RRINV2285-15|633[0n]bp|Diptera|Dolichopodidae|
Dolichopodidae|[1132]|RRINV2290-15|636[0n]bp|Diptera|Dolichopodidae|
Efferia aestuans|[1133]|RRINV1209-15|637[0n]bp|Diptera|Asilidae|
Laphria thoracica|[1134]|RRINV285-15|624[0n]bp|Diptera|Asilidae|
Dolichopodidae|[1135]|RRINV1105-15|630[0n]bp|Diptera|Dolichopodidae|
Dolichopodidae|[1136]|RRBAA061-15|570[0n]bp|Diptera|Dolichopodidae|BOLD:AAG9640
Dolichopodidae|[1137]|RRINV1176-15|637[0n]bp|Diptera|Dolichopodidae|
Dolichopodidae|[1138]|RRMFG3152-15|522[0n]bp|Diptera|Dolichopodidae|BOLD:AAQ0271
Dolichopodidae|[1139]|RRMFE1916-15|570[0n]bp|Diptera|Dolichopodidae|BOLD:ACC8120
Dolichopodidae|[1140]|RRINV1615-15|639[0n]bp|Diptera|Dolichopodidae|
Neurigona disjuncta|[1141]|RRMFG3138-15|576[0n]bp|Diptera|Dolichopodidae|BOLD:ABW1193
Medetera signaticornis|[1142]|RRMFE2012-15|582[0n]bp|Diptera|Dolichopodidae|BOLD:AAZ3931
Dolichopodidae|[1143]|RRMFG952-15|558[0n]bp|Diptera|Dolichopodidae|BOLD:AAN5528
Dolichopodidae|[1144]|RRMFE1779-15|579[0n]bp|Diptera|Dolichopodidae|BOLD:AAU6620
Dolichopodidae|[1145]|RRMFE1461-15|576[0n]bp|Diptera|Dolichopodidae|BOLD:AAZ6708
Dolichopodidae|[1146]|RRSSA822-15|543[0n]bp|Diptera|Dolichopodidae|BOLD:ACG9324
Dolichopodidae|[1147]|RRMFE780-15|564[0n]bp|Diptera|Dolichopodidae|BOLD:AAP6322
Dolichopodidae|[1148]|RRMFE1470-15|609[0n]bp|Diptera|Dolichopodidae|BOLD:AAQ0833
Tipula (Beringotipula) coloradensis|[1149]|RRINV2277-15|631[0n]bp|Diptera|Tipulidae|
Tipula mallochi|[1150]|RRINV2211-15|631[0n]bp|Diptera|Tipulidae|
Tipula dorsimacula|[1151]|RRMPD081-15|582[0n]bp|Diptera|Tipulidae|BOLD:AAF8990
Tipula|[1152]|RRINV2278-15|631[0n]bp|Diptera|Tipulidae|
Nephrotoma cornicina|[1153]|RRINV2213-15|631[0n]bp|Diptera|Tipulidae|
Nephrotoma|[1154]|RRMPD080-15|573[0n]bp|Diptera|Tipulidae|BOLD:ABX6186
Dicranomyia|[1155]|RRSSA012-15|588[0n]bp|Diptera|Limoniidae|BOLD:ABW4424
Forcipomyia|[1156]|RRSSA1221-15|588[0n]bp|Diptera|Ceratopogonidae|BOLD:AAN5147
Forcipomyia|[1157]|RRMFG353-15|582[0n]bp|Diptera|Ceratopogonidae|BOLD:ACC7974
Xylophagus lugens|[1158]|RRMFC2047-15|564[0n]bp|Diptera|Xylophagidae|BOLD:AAJ9649
Xylophagus reflectens|[1159]|RRMFD033-15|579[0n]bp|Diptera|Xylophagidae|BOLD:AAM7333
Xylophagus reflectens|[1160]|RRMFD047-15|579[0n]bp|Diptera|Xylophagidae|BOLD:AAP7637
Phoridae|[1161]|RRSSA3415-15|552[2n]bp|Diptera|Phoridae|BOLD:AAU6541
Limoniidae|[1162]|RRINV2316-15|635[0n]bp|Diptera|Limoniidae|
Phoridae|[1163]|RRMFI490-15|552[0n]bp|Diptera|Phoridae|BOLD:AAM7379
Phoridae|[1164]|RRMFE765-15|588[0n]bp|Diptera|Phoridae|BOLD:AAP6420
Phoridae|[1165]|RRMFI236-15|567[0n]bp|Diptera|Phoridae|BOLD:AAG3286
Phoridae|[1166]|RRINV1294-15|603[0n]bp|Diptera|Phoridae|
Phoridae|[1167]|RRMFI850-15|633[0n]bp|Diptera|Phoridae|BOLD:ACU8783
Phoridae|[1168]|RRMFG922-15|576[0n]bp|Diptera|Phoridae|BOLD:AAG3338
Phoridae|[1169]|RRMFG124-15|567[0n]bp|Diptera|Phoridae|BOLD:AAG3273
Phoridae|[1170]|RRMPB2814-15|549[0n]bp|Diptera|Phoridae|BOLD:AAU5598
Phoridae|[1171]|RRSSA1065-15|552[0n]bp|Diptera|Phoridae|BOLD:AAG3320
Phoridae|[1172]|RRMFI1954-15|630[0n]bp|Diptera|Phoridae|BOLD:AAU6526
Phoridae|[1173]|RRSSA1081-15|564[0n]bp|Diptera|Phoridae|BOLD:ABW5540
Phoridae|[1174]|RRMPD386-15|567[0n]bp|Diptera|Phoridae|BOLD:ABV3316
Phoridae|[1175]|RRMFB600-15|594[0n]bp|Diptera|Phoridae|BOLD:ACV2632
Phoridae|[1176]|RRMFE222-15|579[0n]bp|Diptera|Phoridae|BOLD:AAG3314

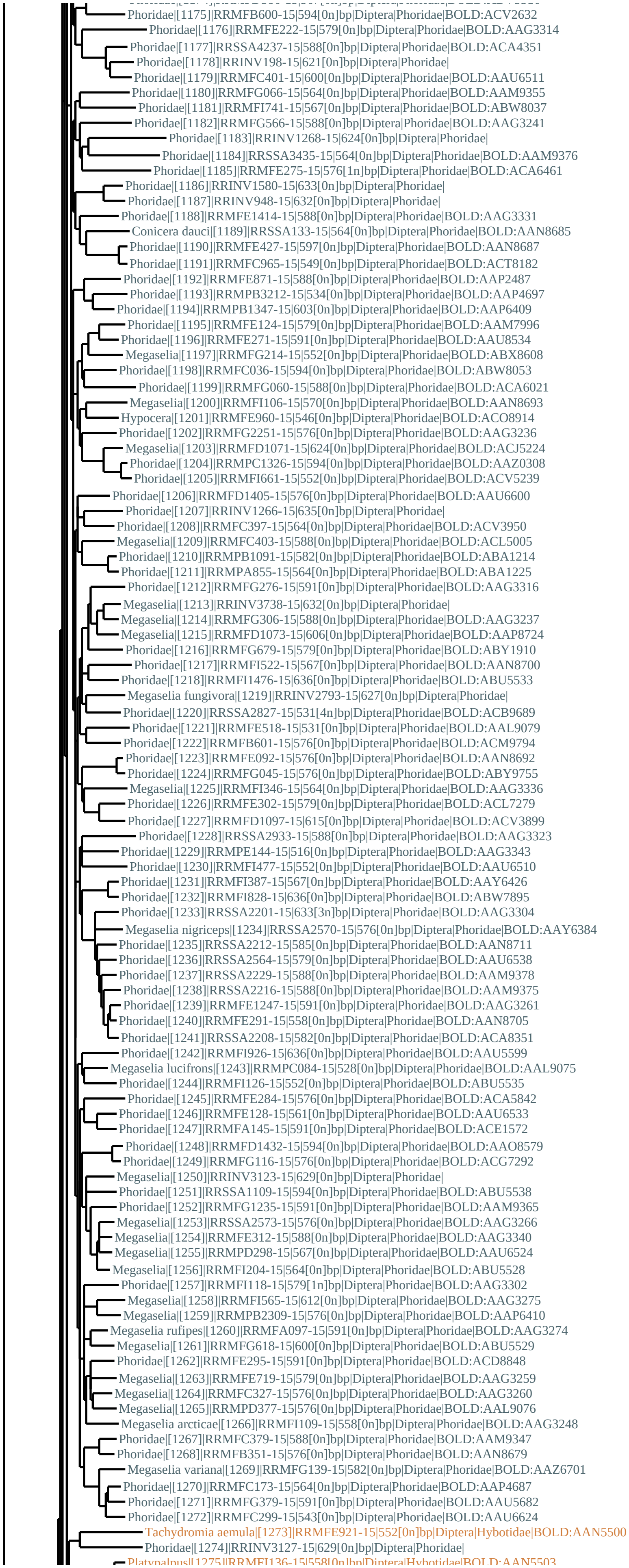
Phoridae|[1175]|RRMFB600-15|594[0n]bp|Diptera|Phoridae|BOLD:ACV2632
Phoridae|[1176]|RRMFE222-15|579[0n]bp|Diptera|Phoridae|BOLD:AAG3314
Phoridae|[1177]|RRSSA4237-15|588[0n]bp|Diptera|Phoridae|BOLD:ACA4351
Phoridae|[1178]|RRINV198-15|621[0n]bp|Diptera|Phoridae|
Phoridae|[1179]|RRMFC401-15|600[0n]bp|Diptera|Phoridae|BOLD:AAU6511
Phoridae|[1180]|RRMFG066-15|564[0n]bp|Diptera|Phoridae|BOLD:AAM9355
Phoridae|[1181]|RRMFI741-15|567[0n]bp|Diptera|Phoridae|BOLD:ABW8037
Phoridae|[1182]|RRMFG566-15|588[0n]bp|Diptera|Phoridae|BOLD:AAG3241
Phoridae|[1183]|RRINV1268-15|624[0n]bp|Diptera|Phoridae|
Phoridae|[1184]|RRSSA3435-15|564[0n]bp|Diptera|Phoridae|BOLD:AAM9376
Phoridae|[1185]|RRMFE275-15|576[1n]bp|Diptera|Phoridae|BOLD:ACA6461
Phoridae|[1186]|RRINV1580-15|633[0n]bp|Diptera|Phoridae|
Phoridae|[1187]|RRINV948-15|632[0n]bp|Diptera|Phoridae|
Phoridae|[1188]|RRMFE1414-15|588[0n]bp|Diptera|Phoridae|BOLD:AAG3331
Conicera dauci|[1189]|RRSSA133-15|564[0n]bp|Diptera|Phoridae|BOLD:AAN8685
Phoridae|[1190]|RRMFE427-15|597[0n]bp|Diptera|Phoridae|BOLD:AAN8687
Phoridae|[1191]|RRMFC965-15|549[0n]bp|Diptera|Phoridae|BOLD:ACT8182
Phoridae|[1192]|RRMFE871-15|588[0n]bp|Diptera|Phoridae|BOLD:AAP2487
Phoridae|[1193]|RRMPB3212-15|534[0n]bp|Diptera|Phoridae|BOLD:AAP4697
Phoridae|[1194]|RRMPB1347-15|603[0n]bp|Diptera|Phoridae|BOLD:AAP6409
Phoridae|[1195]|RRMFE124-15|579[0n]bp|Diptera|Phoridae|BOLD:AAM7996
Phoridae|[1196]|RRMFE271-15|591[0n]bp|Diptera|Phoridae|BOLD:AAU8534
Megaselia|[1197]|RRMFG214-15|552[0n]bp|Diptera|Phoridae|BOLD:ABX8608
Phoridae|[1198]|RRMFC036-15|594[0n]bp|Diptera|Phoridae|BOLD:ABW8053
Phoridae|[1199]|RRMFG060-15|588[0n]bp|Diptera|Phoridae|BOLD:ACA6021
Megaselia|[1200]|RRMFI106-15|570[0n]bp|Diptera|Phoridae|BOLD:AAN8693
Hypocera|[1201]|RRMFE960-15|546[0n]bp|Diptera|Phoridae|BOLD:ACO8914
Phoridae|[1202]|RRMFG2251-15|576[0n]bp|Diptera|Phoridae|BOLD:AAG3236
Megaselia|[1203]|RRMFD1071-15|624[0n]bp|Diptera|Phoridae|BOLD:ACJ5224
Phoridae|[1204]|RRMPC1326-15|594[0n]bp|Diptera|Phoridae|BOLD:AAZ0308
Phoridae|[1205]|RRMFI661-15|552[0n]bp|Diptera|Phoridae|BOLD:ACV5239
Phoridae|[1206]|RRMFD1405-15|576[0n]bp|Diptera|Phoridae|BOLD:AAU6600
Phoridae|[1207]|RRINV1266-15|635[0n]bp|Diptera|Phoridae|
Phoridae|[1208]|RRMFC397-15|564[0n]bp|Diptera|Phoridae|BOLD:ACV3950
Megaselia|[1209]|RRMFC403-15|588[0n]bp|Diptera|Phoridae|BOLD:ACL5005
Phoridae|[1210]|RRMPB1091-15|582[0n]bp|Diptera|Phoridae|BOLD:ABA1214
Phoridae|[1211]|RRMPA855-15|564[0n]bp|Diptera|Phoridae|BOLD:ABA1225
Phoridae|[1212]|RRMFG276-15|591[0n]bp|Diptera|Phoridae|BOLD:AAG3316
Megaselia|[1213]|RRINV3738-15|632[0n]bp|Diptera|Phoridae|
Megaselia|[1214]|RRMFG306-15|588[0n]bp|Diptera|Phoridae|BOLD:AAG3237
Megaselia|[1215]|RRMFD1073-15|606[0n]bp|Diptera|Phoridae|BOLD:AAP8724
Phoridae|[1216]|RRMFG679-15|579[0n]bp|Diptera|Phoridae|BOLD:ABY1910
Phoridae|[1217]|RRMFI522-15|567[0n]bp|Diptera|Phoridae|BOLD:AAN8700
Phoridae|[1218]|RRMFI1476-15|636[0n]bp|Diptera|Phoridae|BOLD:ABU5533
Megaselia fungivora|[1219]|RRINV2793-15|627[0n]bp|Diptera|Phoridae|
Phoridae|[1220]|RRSSA2827-15|531[4n]bp|Diptera|Phoridae|BOLD:ACB9689
Phoridae|[1221]|RRMFE518-15|531[0n]bp|Diptera|Phoridae|BOLD:AAL9079
Phoridae|[1222]|RRMFB601-15|576[0n]bp|Diptera|Phoridae|BOLD:ACM9794
Phoridae|[1223]|RRMFE092-15|576[0n]bp|Diptera|Phoridae|BOLD:AAN8692
Phoridae|[1224]|RRMFG045-15|576[0n]bp|Diptera|Phoridae|BOLD:ABY9755
Megaselia|[1225]|RRMFI346-15|564[0n]bp|Diptera|Phoridae|BOLD:AAG3336
Phoridae|[1226]|RRMFE302-15|579[0n]bp|Diptera|Phoridae|BOLD:ACL7279
Phoridae|[1227]|RRMFD1097-15|615[0n]bp|Diptera|Phoridae|BOLD:ACV3899
Phoridae|[1228]|RRSSA2933-15|588[0n]bp|Diptera|Phoridae|BOLD:AAG3323
Phoridae|[1229]|RRMPE144-15|516[0n]bp|Diptera|Phoridae|BOLD:AAG3343
Phoridae|[1230]|RRMFI477-15|552[0n]bp|Diptera|Phoridae|BOLD:AAU6510
Phoridae|[1231]|RRMFI387-15|567[0n]bp|Diptera|Phoridae|BOLD:AAY6426
Phoridae|[1232]|RRMFI828-15|636[0n]bp|Diptera|Phoridae|BOLD:ABW7895
Phoridae|[1233]|RRSSA2201-15|633[3n]bp|Diptera|Phoridae|BOLD:AAG3304
Megaselia nigriceps|[1234]|RRSSA2570-15|576[0n]bp|Diptera|Phoridae|BOLD:AAY6384
Phoridae|[1235]|RRSSA2212-15|585[0n]bp|Diptera|Phoridae|BOLD:AAN8711
Phoridae|[1236]|RRSSA2564-15|579[0n]bp|Diptera|Phoridae|BOLD:AAU6538
Phoridae|[1237]|RRSSA2229-15|588[0n]bp|Diptera|Phoridae|BOLD:AAM9378
Phoridae|[1238]|RRSSA2216-15|588[0n]bp|Diptera|Phoridae|BOLD:AAM9375
Phoridae|[1239]|RRMFE1247-15|591[0n]bp|Diptera|Phoridae|BOLD:AAG3261
Phoridae|[1240]|RRMFE291-15|558[0n]bp|Diptera|Phoridae|BOLD:AAN8705
Phoridae|[1241]|RRSSA2208-15|582[0n]bp|Diptera|Phoridae|BOLD:ACA8351
Phoridae|[1242]|RRMFI926-15|636[0n]bp|Diptera|Phoridae|BOLD:AAU5599
Megaselia lucifrons|[1243]|RRMPC084-15|528[0n]bp|Diptera|Phoridae|BOLD:AAL9075
Phoridae|[1244]|RRMFI126-15|552[0n]bp|Diptera|Phoridae|BOLD:ABU5535
Phoridae|[1245]|RRMFE284-15|576[0n]bp|Diptera|Phoridae|BOLD:ACA5842
Phoridae|[1246]|RRMFE128-15|561[0n]bp|Diptera|Phoridae|BOLD:AAU6533
Phoridae|[1247]|RRMFA145-15|591[0n]bp|Diptera|Phoridae|BOLD:ACE1572
Phoridae|[1248]|RRMFD1432-15|594[0n]bp|Diptera|Phoridae|BOLD:AAO8579
Phoridae|[1249]|RRMFG116-15|576[0n]bp|Diptera|Phoridae|BOLD:ACG7292
Megaselia|[1250]|RRINV3123-15|629[0n]bp|Diptera|Phoridae|
Phoridae|[1251]|RRSSA1109-15|594[0n]bp|Diptera|Phoridae|BOLD:ABU5538
Phoridae|[1252]|RRMFG1235-15|591[0n]bp|Diptera|Phoridae|BOLD:AAM9365
Megaselia|[1253]|RRSSA2573-15|576[0n]bp|Diptera|Phoridae|BOLD:AAG3266
Megaselia|[1254]|RRMFE312-15|588[0n]bp|Diptera|Phoridae|BOLD:AAG3340
Megaselia|[1255]|RRMPD298-15|567[0n]bp|Diptera|Phoridae|BOLD:AAU6524
Megaselia|[1256]|RRMFI204-15|564[0n]bp|Diptera|Phoridae|BOLD:ABU5528
Phoridae|[1257]|RRMFI118-15|579[1n]bp|Diptera|Phoridae|BOLD:AAG3302
Megaselia|[1258]|RRMFI565-15|612[0n]bp|Diptera|Phoridae|BOLD:AAG3275
Megaselia|[1259]|RRMPB2309-15|576[0n]bp|Diptera|Phoridae|BOLD:AAP6410
Megaselia rufipes|[1260]|RRMFA097-15|591[0n]bp|Diptera|Phoridae|BOLD:AAG3274
Megaselia|[1261]|RRMFG618-15|600[0n]bp|Diptera|Phoridae|BOLD:ABU5529
Phoridae|[1262]|RRMFE295-15|591[0n]bp|Diptera|Phoridae|BOLD:ACD8848
Megaselia|[1263]|RRMFE719-15|579[0n]bp|Diptera|Phoridae|BOLD:AAG3259
Megaselia|[1264]|RRMFC327-15|576[0n]bp|Diptera|Phoridae|BOLD:AAG3260
Megaselia|[1265]|RRMPD377-15|576[0n]bp|Diptera|Phoridae|BOLD:AAL9076
Megaselia arcticae|[1266]|RRMFI109-15|558[0n]bp|Diptera|Phoridae|BOLD:AAG3248
Phoridae|[1267]|RRMFC379-15|588[0n]bp|Diptera|Phoridae|BOLD:AAM9347
Phoridae|[1268]|RRMFB351-15|576[0n]bp|Diptera|Phoridae|BOLD:AAN8679
Megaselia variana|[1269]|RRMFG139-15|582[0n]bp|Diptera|Phoridae|BOLD:AAZ6701
Phoridae|[1270]|RRMFC173-15|564[0n]bp|Diptera|Phoridae|BOLD:AAP4687
Phoridae|[1271]|RRMFG379-15|591[0n]bp|Diptera|Phoridae|BOLD:AAU5682
Phoridae|[1272]|RRMFC299-15|543[0n]bp|Diptera|Phoridae|BOLD:AAU6624
Tachydromia aemula|[1273]|RRMFE921-15|552[0n]bp|Diptera|Hybotidae|BOLD:AAN5500
Phoridae|[1274]|RRINV3127-15|629[0n]bp|Diptera|Phoridae|
Platypalpus|[1275]|RRMFI136-15|558[0n]bp|Diptera|Hybotidae|BOLD:AAN5503

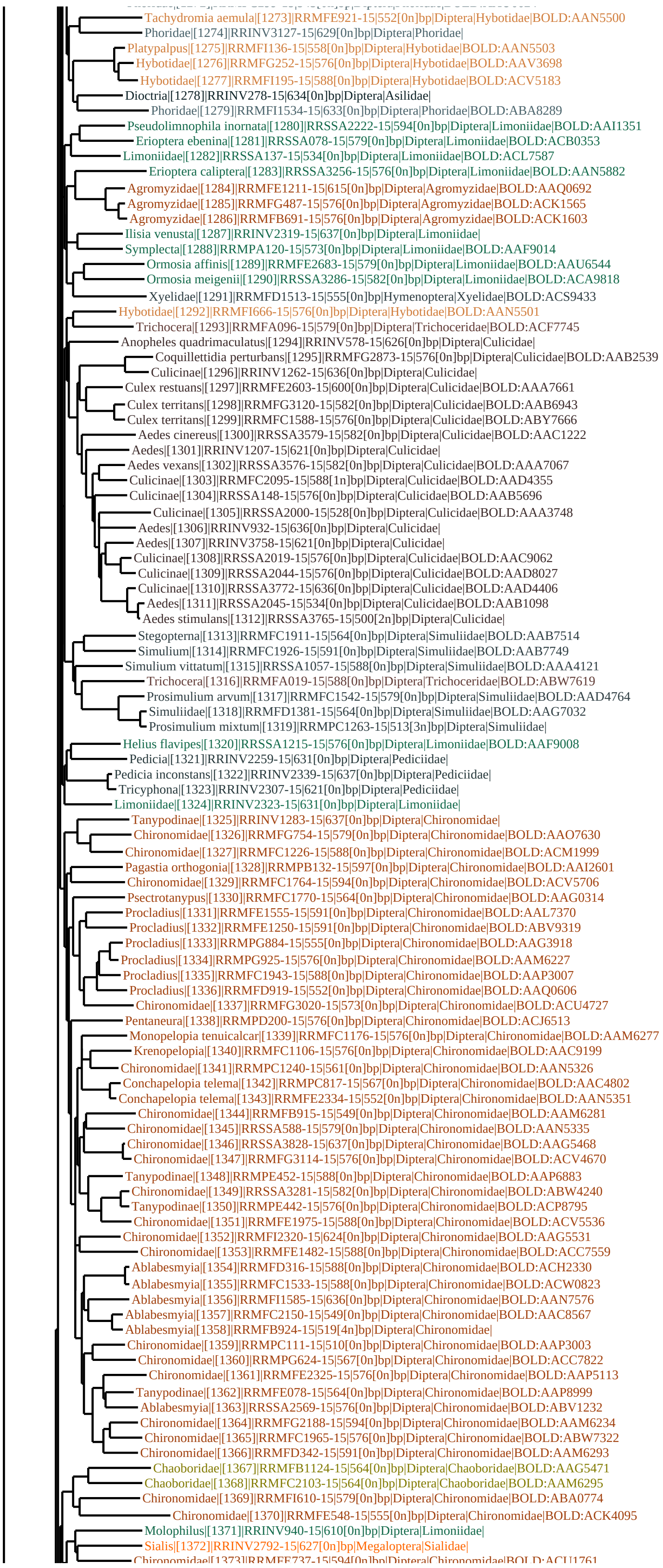
Tachydromia aemula|[1273]|RRMFE921-15|552[0n]bp|Diptera|Hybotidae|BOLD:AAN5500
Phoridae|[1274]|RRINV3127-15|629[0n]bp|Diptera|Phoridae|
Platypalpus|[1275]|RRMFI136-15|558[0n]bp|Diptera|Hybotidae|BOLD:AAN5503
Hybotidae|[1276]|RRMFG252-15|576[0n]bp|Diptera|Hybotidae|BOLD:AAV3698
Hybotidae|[1277]|RRMFI195-15|588[0n]bp|Diptera|Hybotidae|BOLD:ACV5183
Dioctria|[1278]|RRINV278-15|634[0n]bp|Diptera|Asilidae|
Phoridae|[1279]|RRMFI1534-15|633[0n]bp|Diptera|Phoridae|BOLD:ABA8289
Pseudolimnophila inornata|[1280]|RRSSA2222-15|594[0n]bp|Diptera|Limoniidae|BOLD:AAI1351
Erioptera ebenina|[1281]|RRSSA078-15|579[0n]bp|Diptera|Limoniidae|BOLD:ACB0353
Limoniidae|[1282]|RRSSA137-15|534[0n]bp|Diptera|Limoniidae|BOLD:ACL7587
Erioptera caliptera|[1283]|RRSSA3256-15|576[0n]bp|Diptera|Limoniidae|BOLD:AAN5882
Agromyzidae|[1284]|RRMFE1211-15|615[0n]bp|Diptera|Agromyzidae|BOLD:AAQ0692
Agromyzidae|[1285]|RRMFG487-15|576[0n]bp|Diptera|Agromyzidae|BOLD:ACK1565
Agromyzidae|[1286]|RRMFB691-15|576[0n]bp|Diptera|Agromyzidae|BOLD:ACK1603
Ilisia venusta|[1287]|RRINV2319-15|637[0n]bp|Diptera|Limoniidae|
Symplecta|[1288]|RRMPA120-15|573[0n]bp|Diptera|Limoniidae|BOLD:AAF9014
Ormosia affinis|[1289]|RRMFE2683-15|579[0n]bp|Diptera|Limoniidae|BOLD:AAU6544
Ormosia meigenii|[1290]|RRSSA3286-15|582[0n]bp|Diptera|Limoniidae|BOLD:ACA9818
Xyelidae|[1291]|RRMFD1513-15|555[0n]bp|Hymenoptera|Xyelidae|BOLD:ACS9433
Hybotidae|[1292]|RRMFI666-15|576[0n]bp|Diptera|Hybotidae|BOLD:AAN5501
Trichocera|[1293]|RRMFA096-15|579[0n]bp|Diptera|Trichoceridae|BOLD:ACF7745
Anopheles quadrimaculatus|[1294]|RRINV578-15|626[0n]bp|Diptera|Culicidae|
Coquillettidia perturbans|[1295]|RRMFG2873-15|576[0n]bp|Diptera|Culicidae|BOLD:AAB2539
Culicinae|[1296]|RRINV1262-15|636[0n]bp|Diptera|Culicidae|
Culex restuans|[1297]|RRMFE2603-15|600[0n]bp|Diptera|Culicidae|BOLD:AAA7661
Culex territans|[1298]|RRMFG3120-15|582[0n]bp|Diptera|Culicidae|BOLD:AAB6943
Culex territans|[1299]|RRMFC1588-15|576[0n]bp|Diptera|Culicidae|BOLD:ABY7666
Aedes cinereus|[1300]|RRSSA3579-15|582[0n]bp|Diptera|Culicidae|BOLD:AAC1222
Aedes|[1301]|RRINV1207-15|621[0n]bp|Diptera|Culicidae|
Aedes vexans|[1302]|RRSSA3576-15|582[0n]bp|Diptera|Culicidae|BOLD:AAA7067
Culicinae|[1303]|RRMFC2095-15|588[1n]bp|Diptera|Culicidae|BOLD:AAD4355
Culicinae|[1304]|RRSSA148-15|576[0n]bp|Diptera|Culicidae|BOLD:AAB5696
Culicinae|[1305]|RRSSA2000-15|528[0n]bp|Diptera|Culicidae|BOLD:AAA3748
Aedes|[1306]|RRINV932-15|636[0n]bp|Diptera|Culicidae|
Aedes|[1307]|RRINV3758-15|621[0n]bp|Diptera|Culicidae|
Culicinae|[1308]|RRSSA2019-15|576[0n]bp|Diptera|Culicidae|BOLD:AAC9062
Culicinae|[1309]|RRSSA2044-15|576[0n]bp|Diptera|Culicidae|BOLD:AAD8027
Culicinae|[1310]|RRSSA3772-15|636[0n]bp|Diptera|Culicidae|BOLD:AAD4406
Aedes|[1311]|RRSSA2045-15|534[0n]bp|Diptera|Culicidae|BOLD:AAB1098
Aedes stimulans|[1312]|RRSSA3765-15|500[2n]bp|Diptera|Culicidae|
Stegopterna|[1313]|RRMFC1911-15|564[0n]bp|Diptera|Simuliidae|BOLD:AAB7514
Simulium|[1314]|RRMFC1926-15|591[0n]bp|Diptera|Simuliidae|BOLD:AAB7749
Simulium vittatum|[1315]|RRSSA1057-15|588[0n]bp|Diptera|Simuliidae|BOLD:AAA4121
Trichocera|[1316]|RRMFA019-15|588[0n]bp|Diptera|Trichoceridae|BOLD:ABW7619
Prosimulium arvum|[1317]|RRMFC1542-15|579[0n]bp|Diptera|Simuliidae|BOLD:AAD4764
Simuliidae|[1318]|RRMFD1381-15|564[0n]bp|Diptera|Simuliidae|BOLD:AAG7032
Prosimulium mixtum|[1319]|RRMPC1263-15|513[3n]bp|Diptera|Simuliidae|
Helius flavipes|[1320]|RRSSA1215-15|576[0n]bp|Diptera|Limoniidae|BOLD:AAF9008
Pedicia|[1321]|RRINV2259-15|631[0n]bp|Diptera|Pediciidae|
Pedicia inconstans|[1322]|RRINV2339-15|637[0n]bp|Diptera|Pediciidae|
Tricyphona|[1323]|RRINV2307-15|621[0n]bp|Diptera|Pediciidae|
Limoniidae|[1324]|RRINV2323-15|631[0n]bp|Diptera|Limoniidae|
Tanypodinae|[1325]|RRINV1283-15|637[0n]bp|Diptera|Chironomidae|
Chironomidae|[1326]|RRMFG754-15|579[0n]bp|Diptera|Chironomidae|BOLD:AAO7630
Chironomidae|[1327]|RRMFC1226-15|588[0n]bp|Diptera|Chironomidae|BOLD:ACM1999
Pagastia orthogonia|[1328]|RRMPB132-15|597[0n]bp|Diptera|Chironomidae|BOLD:AAI2601
Chironomidae|[1329]|RRMFC1764-15|594[0n]bp|Diptera|Chironomidae|BOLD:ACV5706
Psectrotanypus|[1330]|RRMFC1770-15|564[0n]bp|Diptera|Chironomidae|BOLD:AAG0314
Procladius|[1331]|RRMFE1555-15|591[0n]bp|Diptera|Chironomidae|BOLD:AAL7370
Procladius|[1332]|RRMFE1250-15|591[0n]bp|Diptera|Chironomidae|BOLD:ABV9319
Procladius|[1333]|RRMPG884-15|555[0n]bp|Diptera|Chironomidae|BOLD:AAG3918
Procladius|[1334]|RRMPG925-15|576[0n]bp|Diptera|Chironomidae|BOLD:AAM6227
Procladius|[1335]|RRMFC1943-15|588[0n]bp|Diptera|Chironomidae|BOLD:AAP3007
Procladius|[1336]|RRMFD919-15|552[0n]bp|Diptera|Chironomidae|BOLD:AAQ0606
Chironomidae|[1337]|RRMFG3020-15|573[0n]bp|Diptera|Chironomidae|BOLD:ACU4727
Pentaneura|[1338]|RRMPD200-15|576[0n]bp|Diptera|Chironomidae|BOLD:ACJ6513
Monopelopia tenuicalcar|[1339]|RRMFC1176-15|576[0n]bp|Diptera|Chironomidae|BOLD:AAM6277
Krenopelopia|[1340]|RRMFC1106-15|576[0n]bp|Diptera|Chironomidae|BOLD:AAC9199
Chironomidae|[1341]|RRMPC1240-15|561[0n]bp|Diptera|Chironomidae|BOLD:AAN5326
Conchapelopia telema|[1342]|RRMPC817-15|567[0n]bp|Diptera|Chironomidae|BOLD:AAC4802
Conchapelopia telema|[1343]|RRMFE2334-15|552[0n]bp|Diptera|Chironomidae|BOLD:AAN5351
Chironomidae|[1344]|RRMFB915-15|549[0n]bp|Diptera|Chironomidae|BOLD:AAM6281
Chironomidae|[1345]|RRSSA588-15|579[0n]bp|Diptera|Chironomidae|BOLD:AAN5335
Chironomidae|[1346]|RRSSA3828-15|637[0n]bp|Diptera|Chironomidae|BOLD:AAG5468
Chironomidae|[1347]|RRMFG3114-15|576[0n]bp|Diptera|Chironomidae|BOLD:ACV4670
Tanypodinae|[1348]|RRMPE452-15|588[0n]bp|Diptera|Chironomidae|BOLD:AAP6883
Chironomidae|[1349]|RRSSA3281-15|582[0n]bp|Diptera|Chironomidae|BOLD:ABW4240
Tanypodinae|[1350]|RRMPE442-15|576[0n]bp|Diptera|Chironomidae|BOLD:ACP8795
Chironomidae|[1351]|RRMFE1975-15|588[0n]bp|Diptera|Chironomidae|BOLD:ACV5536
Chironomidae|[1352]|RRMFI2320-15|624[0n]bp|Diptera|Chironomidae|BOLD:AAG5531
Chironomidae|[1353]|RRMFE1482-15|588[0n]bp|Diptera|Chironomidae|BOLD:ACC7559
Ablabesmyia|[1354]|RRMFD316-15|588[0n]bp|Diptera|Chironomidae|BOLD:ACH2330
Ablabesmyia|[1355]|RRMFC1533-15|588[0n]bp|Diptera|Chironomidae|BOLD:ACW0823
Ablabesmyia|[1356]|RRMFI1585-15|636[0n]bp|Diptera|Chironomidae|BOLD:AAN7576
Ablabesmyia|[1357]|RRMFC2150-15|549[0n]bp|Diptera|Chironomidae|BOLD:AAC8567
Ablabesmyia|[1358]|RRMFB924-15|519[4n]bp|Diptera|Chironomidae|
Chironomidae|[1359]|RRMPC111-15|510[0n]bp|Diptera|Chironomidae|BOLD:AAP3003
Chironomidae|[1360]|RRMPG624-15|567[0n]bp|Diptera|Chironomidae|BOLD:ACC7822
Chironomidae|[1361]|RRMFE2325-15|576[0n]bp|Diptera|Chironomidae|BOLD:AAP5113
Tanypodinae|[1362]|RRMFE078-15|564[0n]bp|Diptera|Chironomidae|BOLD:AAP8999
Ablabesmyia|[1363]|RRSSA2569-15|576[0n]bp|Diptera|Chironomidae|BOLD:ABV1232
Chironomidae|[1364]|RRMFG2188-15|594[0n]bp|Diptera|Chironomidae|BOLD:AAM6234
Chironomidae|[1365]|RRMFC1965-15|576[0n]bp|Diptera|Chironomidae|BOLD:ABW7322
Chironomidae|[1366]|RRMFD342-15|591[0n]bp|Diptera|Chironomidae|BOLD:AAM6293
Chaoboridae|[1367]|RRMFB1124-15|564[0n]bp|Diptera|Chaoboridae|BOLD:AAG5471
Chaoboridae|[1368]|RRMFC2103-15|564[0n]bp|Diptera|Chaoboridae|BOLD:AAM6295
Chironomidae|[1369]|RRMFI610-15|579[0n]bp|Diptera|Chironomidae|BOLD:ABA0774
Chironomidae|[1370]|RRMFE548-15|555[0n]bp|Diptera|Chironomidae|BOLD:ACK4095
Molophilus|[1371]|RRINV940-15|610[0n]bp|Diptera|Limoniidae|
Sialis|[1372]|RRINV2792-15|627[0n]bp|Megaloptera|Sialidae|

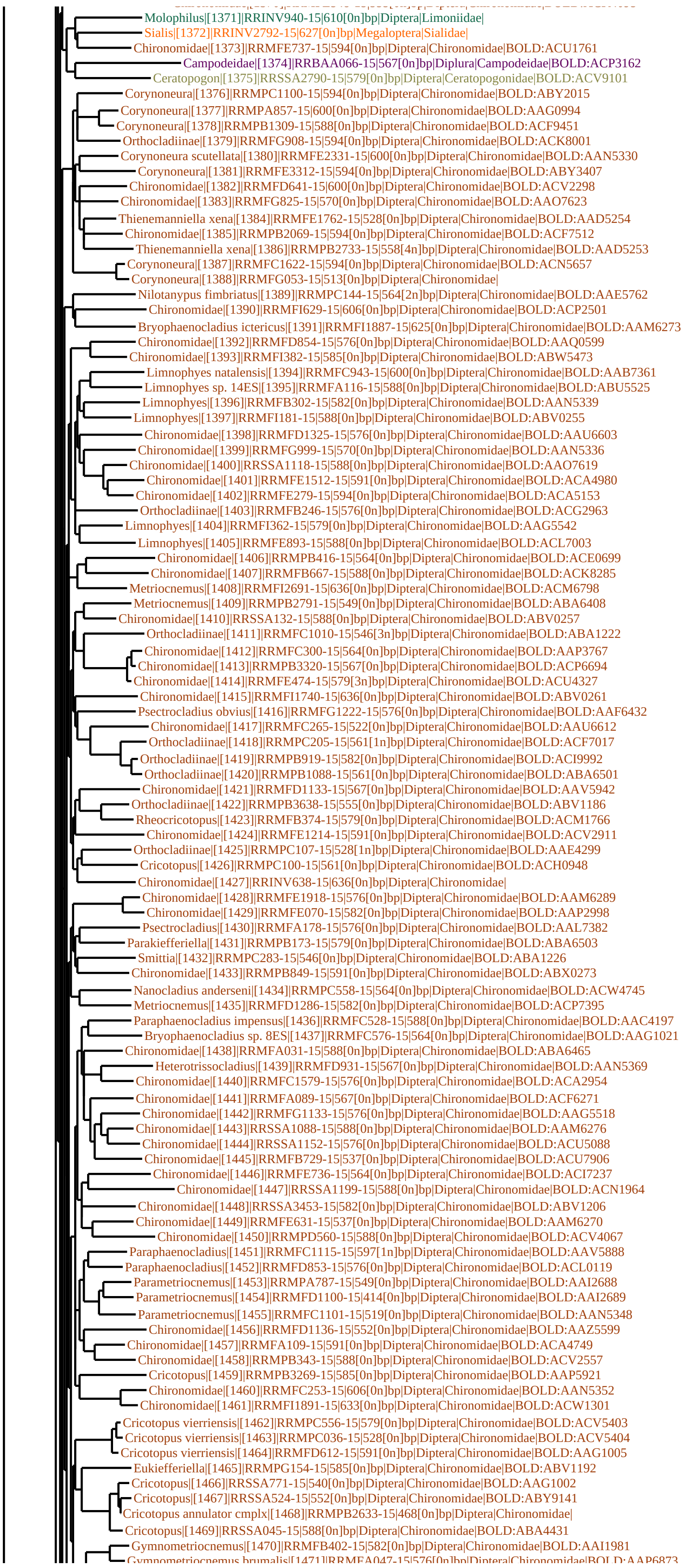
Molophilus|[1371]|RRINV940-15|610[0n]bp|Diptera|Limoniidae|
Sialis|[1372]|RRINV2792-15|627[0n]bp|Megaloptera|Sialidae|
Chironomidae|[1373]|RRMFE737-15|594[0n]bp|Diptera|Chironomidae|BOLD:ACU1761
Campodeidae|[1374]|RRBAA066-15|567[0n]bp|Diplura|Campodeidae|BOLD:ACP3162
Ceratopogon|[1375]|RRSSA2790-15|579[0n]bp|Diptera|Ceratopogonidae|BOLD:ACV9101
Corynoneura|[1376]|RRMPC1100-15|594[0n]bp|Diptera|Chironomidae|BOLD:ABY2015
Corynoneura|[1377]|RRMPA857-15|600[0n]bp|Diptera|Chironomidae|BOLD:AAG0994
Corynoneura|[1378]|RRMPB1309-15|588[0n]bp|Diptera|Chironomidae|BOLD:ACF9451
Orthocladiinae|[1379]|RRMFG908-15|594[0n]bp|Diptera|Chironomidae|BOLD:ACK8001
Corynoneura scutellata|[1380]|RRMFE2331-15|600[0n]bp|Diptera|Chironomidae|BOLD:AAN5330
Corynoneura|[1381]|RRMFE3312-15|594[0n]bp|Diptera|Chironomidae|BOLD:ABY3407
Chironomidae|[1382]|RRMFD641-15|600[0n]bp|Diptera|Chironomidae|BOLD:ACV2298
Chironomidae|[1383]|RRMFG825-15|570[0n]bp|Diptera|Chironomidae|BOLD:AAO7623
Thienemanniella xena|[1384]|RRMFE1762-15|528[0n]bp|Diptera|Chironomidae|BOLD:AAD5254
Chironomidae|[1385]|RRMPB2069-15|594[0n]bp|Diptera|Chironomidae|BOLD:ACF7512
Thienemanniella xena|[1386]|RRMPB2733-15|558[4n]bp|Diptera|Chironomidae|BOLD:AAD5253
Corynoneura|[1387]|RRMFC1622-15|594[0n]bp|Diptera|Chironomidae|BOLD:ACN5657
Corynoneura|[1388]|RRMFG053-15|513[0n]bp|Diptera|Chironomidae|
Nilotanypus fimbriatus|[1389]|RRMPC144-15|564[2n]bp|Diptera|Chironomidae|BOLD:AAE5762
Chironomidae|[1390]|RRMFI629-15|606[0n]bp|Diptera|Chironomidae|BOLD:ACP2501
Bryophaenocladius ictericus|[1391]|RRMFI1887-15|625[0n]bp|Diptera|Chironomidae|BOLD:AAM6273
Chironomidae|[1392]|RRMFD854-15|576[0n]bp|Diptera|Chironomidae|BOLD:AAQ0599
Chironomidae|[1393]|RRMFI382-15|585[0n]bp|Diptera|Chironomidae|BOLD:ABW5473
Limnophyes natalensis|[1394]|RRMFC943-15|600[0n]bp|Diptera|Chironomidae|BOLD:AAB7361
Limnophyes sp. 14ES|[1395]|RRMFA116-15|588[0n]bp|Diptera|Chironomidae|BOLD:ABU5525
Limnophyes|[1396]|RRMFB302-15|582[0n]bp|Diptera|Chironomidae|BOLD:AAN5339
Limnophyes|[1397]|RRMFI181-15|588[0n]bp|Diptera|Chironomidae|BOLD:ABV0255
Chironomidae|[1398]|RRMFD1325-15|576[0n]bp|Diptera|Chironomidae|BOLD:AAU6603
Chironomidae|[1399]|RRMFG999-15|570[0n]bp|Diptera|Chironomidae|BOLD:AAN5336
Chironomidae|[1400]|RRSSA1118-15|588[0n]bp|Diptera|Chironomidae|BOLD:AAO7619
Chironomidae|[1401]|RRMFE1512-15|591[0n]bp|Diptera|Chironomidae|BOLD:ACA4980
Chironomidae|[1402]|RRMFE279-15|594[0n]bp|Diptera|Chironomidae|BOLD:ACA5153
Orthocladiinae|[1403]|RRMFB246-15|576[0n]bp|Diptera|Chironomidae|BOLD:ACG2963
Limnophyes|[1404]|RRMFI362-15|579[0n]bp|Diptera|Chironomidae|BOLD:AAG5542
Limnophyes|[1405]|RRMFE893-15|588[0n]bp|Diptera|Chironomidae|BOLD:ACL7003
Chironomidae|[1406]|RRMPB416-15|564[0n]bp|Diptera|Chironomidae|BOLD:ACE0699
Chironomidae|[1407]|RRMFB667-15|588[0n]bp|Diptera|Chironomidae|BOLD:ACK8285
Metriocnemus|[1408]|RRMFI2691-15|636[0n]bp|Diptera|Chironomidae|BOLD:ACM6798
Metriocnemus|[1409]|RRMPB2791-15|549[0n]bp|Diptera|Chironomidae|BOLD:ABA6408
Chironomidae|[1410]|RRSSA132-15|588[0n]bp|Diptera|Chironomidae|BOLD:ABV0257
Orthocladiinae|[1411]|RRMFC1010-15|546[3n]bp|Diptera|Chironomidae|BOLD:ABA1222
Chironomidae|[1412]|RRMFC300-15|564[0n]bp|Diptera|Chironomidae|BOLD:AAP3767
Chironomidae|[1413]|RRMPB3320-15|567[0n]bp|Diptera|Chironomidae|BOLD:ACP6694
Chironomidae|[1414]|RRMFE474-15|579[3n]bp|Diptera|Chironomidae|BOLD:ACU4327
Chironomidae|[1415]|RRMFI1740-15|636[0n]bp|Diptera|Chironomidae|BOLD:ABV0261
Psectrocladius obvius|[1416]|RRMFG1222-15|576[0n]bp|Diptera|Chironomidae|BOLD:AAF6432
Chironomidae|[1417]|RRMFC265-15|522[0n]bp|Diptera|Chironomidae|BOLD:AAU6612
Orthocladiinae|[1418]|RRMPC205-15|561[1n]bp|Diptera|Chironomidae|BOLD:ACF7017
Orthocladiinae|[1419]|RRMPB919-15|582[0n]bp|Diptera|Chironomidae|BOLD:ACI9992
Orthocladiinae|[1420]|RRMPB1088-15|561[0n]bp|Diptera|Chironomidae|BOLD:ABA6501
Chironomidae|[1421]|RRMFD1133-15|567[0n]bp|Diptera|Chironomidae|BOLD:AAV5942
Orthocladiinae|[1422]|RRMPB3638-15|555[0n]bp|Diptera|Chironomidae|BOLD:ABV1186
Rheocricotopus|[1423]|RRMFB374-15|579[0n]bp|Diptera|Chironomidae|BOLD:ACM1766
Chironomidae|[1424]|RRMFE1214-15|591[0n]bp|Diptera|Chironomidae|BOLD:ACV2911
Orthocladiinae|[1425]|RRMPC107-15|528[1n]bp|Diptera|Chironomidae|BOLD:AAE4299
Cricotopus|[1426]|RRMPC100-15|561[0n]bp|Diptera|Chironomidae|BOLD:ACH0948
Chironomidae|[1427]|RRINV638-15|636[0n]bp|Diptera|Chironomidae|
Chironomidae|[1428]|RRMFE1918-15|576[0n]bp|Diptera|Chironomidae|BOLD:AAM6289
Chironomidae|[1429]|RRMFE070-15|582[0n]bp|Diptera|Chironomidae|BOLD:AAP2998
Psectrocladius|[1430]|RRMFA178-15|576[0n]bp|Diptera|Chironomidae|BOLD:AAL7382
Parakiefferiella|[1431]|RRMPB173-15|579[0n]bp|Diptera|Chironomidae|BOLD:ABA6503
Smittia|[1432]|RRMPC283-15|546[0n]bp|Diptera|Chironomidae|BOLD:ABA1226
Chironomidae|[1433]|RRMPB849-15|591[0n]bp|Diptera|Chironomidae|BOLD:ABX0273
Nanocladius anderseni|[1434]|RRMPC558-15|564[0n]bp|Diptera|Chironomidae|BOLD:ACW4745
Metriocnemus|[1435]|RRMFD1286-15|582[0n]bp|Diptera|Chironomidae|BOLD:ACP7395
Paraphaenocladius impensus|[1436]|RRMFC528-15|588[0n]bp|Diptera|Chironomidae|BOLD:AAC4197
Bryophaenocladius sp. 8ES|[1437]|RRMFC576-15|564[0n]bp|Diptera|Chironomidae|BOLD:AAG1021
Chironomidae|[1438]|RRMFA031-15|588[0n]bp|Diptera|Chironomidae|BOLD:ABA6465
Heterotrissocladius|[1439]|RRMFD931-15|567[0n]bp|Diptera|Chironomidae|BOLD:AAN5369
Chironomidae|[1440]|RRMFC1579-15|576[0n]bp|Diptera|Chironomidae|BOLD:ACA2954
Chironomidae|[1441]|RRMFA089-15|567[0n]bp|Diptera|Chironomidae|BOLD:ACF6271
Chironomidae|[1442]|RRMFG1133-15|576[0n]bp|Diptera|Chironomidae|BOLD:AAG5518
Chironomidae|[1443]|RRSSA1088-15|588[0n]bp|Diptera|Chironomidae|BOLD:AAM6276
Chironomidae|[1444]|RRSSA1152-15|576[0n]bp|Diptera|Chironomidae|BOLD:ACU5088
Chironomidae|[1445]|RRMFB729-15|537[0n]bp|Diptera|Chironomidae|BOLD:ACU7906
Chironomidae|[1446]|RRMFE736-15|564[0n]bp|Diptera|Chironomidae|BOLD:ACI7237
Chironomidae|[1447]|RRSSA1199-15|588[0n]bp|Diptera|Chironomidae|BOLD:ACN1964
Chironomidae|[1448]|RRSSA3453-15|582[0n]bp|Diptera|Chironomidae|BOLD:ABV1206
Chironomidae|[1449]|RRMFE631-15|537[0n]bp|Diptera|Chironomidae|BOLD:AAM6270
Chironomidae|[1450]|RRMPD560-15|588[0n]bp|Diptera|Chironomidae|BOLD:ACV4067
Paraphaenocladius|[1451]|RRMFC1115-15|597[1n]bp|Diptera|Chironomidae|BOLD:AAV5888
Paraphaenocladius|[1452]|RRMFD853-15|576[0n]bp|Diptera|Chironomidae|BOLD:ACL0119
Parametriocnemus|[1453]|RRMPA787-15|549[0n]bp|Diptera|Chironomidae|BOLD:AAI2688
Parametriocnemus|[1454]|RRMFD1100-15|414[0n]bp|Diptera|Chironomidae|BOLD:AAI2689
Parametriocnemus|[1455]|RRMFC1101-15|519[0n]bp|Diptera|Chironomidae|BOLD:AAN5348
Chironomidae|[1456]|RRMFD1136-15|552[0n]bp|Diptera|Chironomidae|BOLD:AAZ5599
Chironomidae|[1457]|RRMFA109-15|591[0n]bp|Diptera|Chironomidae|BOLD:ACA4749
Chironomidae|[1458]|RRMPB343-15|588[0n]bp|Diptera|Chironomidae|BOLD:ACV2557
Cricotopus|[1459]|RRMPB3269-15|585[0n]bp|Diptera|Chironomidae|BOLD:AAP5921
Chironomidae|[1460]|RRMFC253-15|606[0n]bp|Diptera|Chironomidae|BOLD:AAN5352
Chironomidae|[1461]|RRMFI1891-15|633[0n]bp|Diptera|Chironomidae|BOLD:ACW1301
Cricotopus vierriensis|[1462]|RRMPC556-15|579[0n]bp|Diptera|Chironomidae|BOLD:ACV5403
Cricotopus vierriensis|[1463]|RRMPC036-15|528[0n]bp|Diptera|Chironomidae|BOLD:ACV5404
Cricotopus vierriensis|[1464]|RRMFD612-15|591[0n]bp|Diptera|Chironomidae|BOLD:AAG1005
Eukiefferiella|[1465]|RRMPG154-15|585[0n]bp|Diptera|Chironomidae|BOLD:ABV1192
Cricotopus|[1466]|RRSSA771-15|540[0n]bp|Diptera|Chironomidae|BOLD:AAG1002
Cricotopus|[1467]|RRSSA524-15|552[0n]bp|Diptera|Chironomidae|BOLD:ABY9141
Cricotopus annulator cmplx|[1468]|RRMPB2633-15|468[0n]bp|Diptera|Chironomidae|
Cricotopus|[1469]|RRSSA045-15|588[0n]bp|Diptera|Chironomidae|BOLD:ABA4431
Gymnometriocnemus|[1470]|RRMFB402-15|582[0n]bp|Diptera|Chironomidae|BOLD:AAI1981
Gymnometriocnemus brumalis|[1471]|RRMFA047-15|576[0n]bp|Diptera|Chironomidae|BOLD:AAP6873

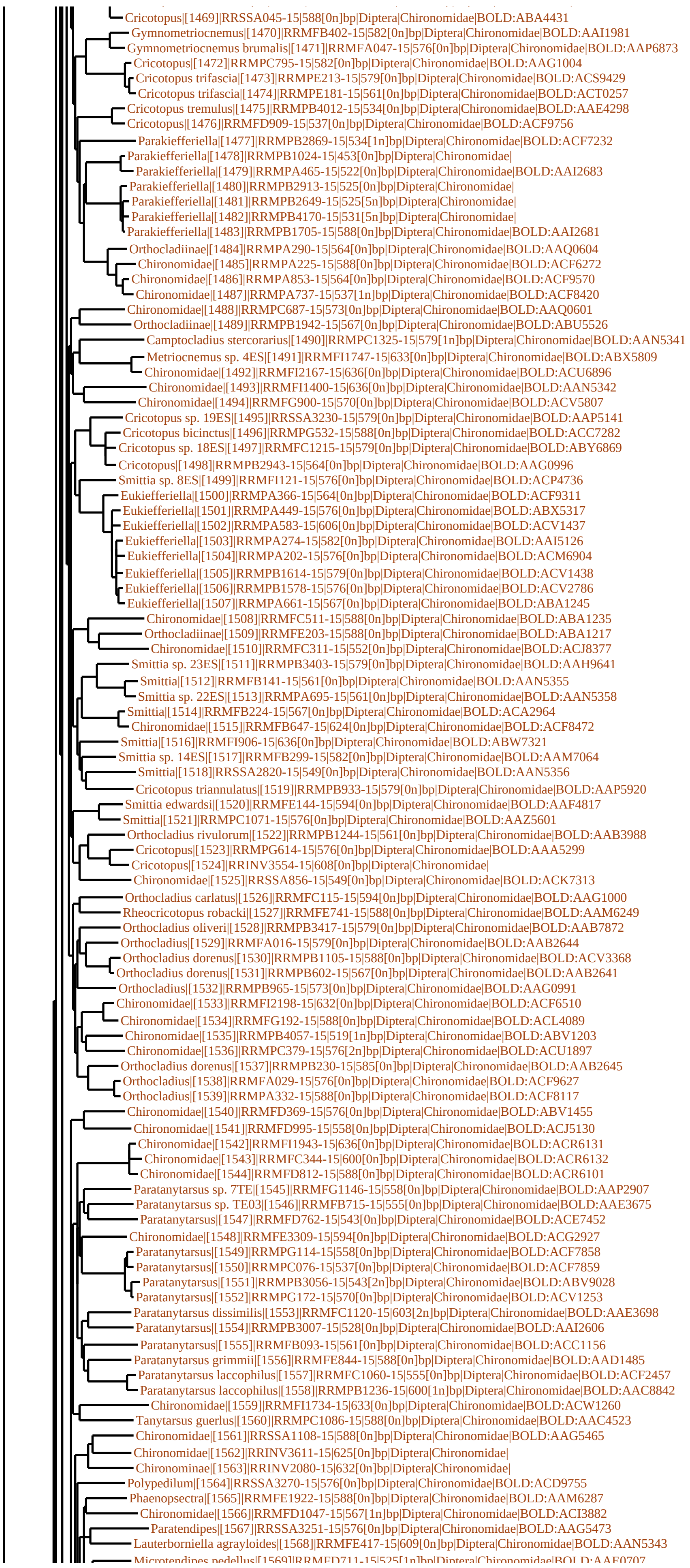
Cricotopus|[1469]|RRSSA045-15|588[0n]bp|Diptera|Chironomidae|BOLD:ABA4431
Gymnometriocnemus|[1470]|RRMFB402-15|582[0n]bp|Diptera|Chironomidae|BOLD:AAI1981
Gymnometriocnemus brumalis|[1471]|RRMFA047-15|576[0n]bp|Diptera|Chironomidae|BOLD:AAP6873
Cricotopus|[1472]|RRMPC795-15|582[0n]bp|Diptera|Chironomidae|BOLD:AAG1004
Cricotopus trifascia|[1473]|RRMPE213-15|579[0n]bp|Diptera|Chironomidae|BOLD:ACS9429
Cricotopus trifascia|[1474]|RRMPE181-15|561[0n]bp|Diptera|Chironomidae|BOLD:ACT0257
Cricotopus tremulus|[1475]|RRMPB4012-15|534[0n]bp|Diptera|Chironomidae|BOLD:AAE4298
Cricotopus|[1476]|RRMFD909-15|537[0n]bp|Diptera|Chironomidae|BOLD:ACF9756
Parakiefferiella|[1477]|RRMPB2869-15|534[1n]bp|Diptera|Chironomidae|BOLD:ACF7232
Parakiefferiella|[1478]|RRMPB1024-15|453[0n]bp|Diptera|Chironomidae|
Parakiefferiella|[1479]|RRMPA465-15|522[0n]bp|Diptera|Chironomidae|BOLD:AAI2683
Parakiefferiella|[1480]|RRMPB2913-15|525[0n]bp|Diptera|Chironomidae|
Parakiefferiella|[1481]|RRMPB2649-15|525[5n]bp|Diptera|Chironomidae|
Parakiefferiella|[1482]|RRMPB4170-15|531[5n]bp|Diptera|Chironomidae|
Parakiefferiella|[1483]|RRMPB1705-15|588[0n]bp|Diptera|Chironomidae|BOLD:AAI2681
Orthocladiinae|[1484]|RRMPA290-15|564[0n]bp|Diptera|Chironomidae|BOLD:AAQ0604
Chironomidae|[1485]|RRMPA225-15|588[0n]bp|Diptera|Chironomidae|BOLD:ACF6272
Chironomidae|[1486]|RRMPA853-15|564[0n]bp|Diptera|Chironomidae|BOLD:ACF9570
Chironomidae|[1487]|RRMPA737-15|537[1n]bp|Diptera|Chironomidae|BOLD:ACF8420
Chironomidae|[1488]|RRMPC687-15|573[0n]bp|Diptera|Chironomidae|BOLD:AAQ0601
Orthocladiinae|[1489]|RRMPB1942-15|567[0n]bp|Diptera|Chironomidae|BOLD:ABU5526
Camptocladius stercorarius|[1490]|RRMPC1325-15|579[1n]bp|Diptera|Chironomidae|BOLD:AAN5341
Metriocnemus sp. 4ES|[1491]|RRMFI1747-15|633[0n]bp|Diptera|Chironomidae|BOLD:ABX5809
Chironomidae|[1492]|RRMFI2167-15|636[0n]bp|Diptera|Chironomidae|BOLD:ACU6896
Chironomidae|[1493]|RRMFI1400-15|636[0n]bp|Diptera|Chironomidae|BOLD:AAN5342
Chironomidae|[1494]|RRMFG900-15|570[0n]bp|Diptera|Chironomidae|BOLD:ACV5807
Cricotopus sp. 19ES|[1495]|RRSSA3230-15|579[0n]bp|Diptera|Chironomidae|BOLD:AAP5141
Cricotopus bicinctus|[1496]|RRMPG532-15|588[0n]bp|Diptera|Chironomidae|BOLD:ACC7282
Cricotopus sp. 18ES|[1497]|RRMFC1215-15|579[0n]bp|Diptera|Chironomidae|BOLD:ABY6869
Cricotopus|[1498]|RRMPB2943-15|564[0n]bp|Diptera|Chironomidae|BOLD:AAG0996
Smittia sp. 8ES|[1499]|RRMFI121-15|576[0n]bp|Diptera|Chironomidae|BOLD:ACP4736
Eukiefferiella|[1500]|RRMPA366-15|564[0n]bp|Diptera|Chironomidae|BOLD:ACF9311
Eukiefferiella|[1501]|RRMPA449-15|576[0n]bp|Diptera|Chironomidae|BOLD:ABX5317
Eukiefferiella|[1502]|RRMPA583-15|606[0n]bp|Diptera|Chironomidae|BOLD:ACV1437
Eukiefferiella|[1503]|RRMPA274-15|582[0n]bp|Diptera|Chironomidae|BOLD:AAI5126
Eukiefferiella|[1504]|RRMPA202-15|576[0n]bp|Diptera|Chironomidae|BOLD:ACM6904
Eukiefferiella|[1505]|RRMPB1614-15|579[0n]bp|Diptera|Chironomidae|BOLD:ACV1438
Eukiefferiella|[1506]|RRMPB1578-15|576[0n]bp|Diptera|Chironomidae|BOLD:ACV2786
Eukiefferiella|[1507]|RRMPA661-15|567[0n]bp|Diptera|Chironomidae|BOLD:ABA1245
Chironomidae|[1508]|RRMFC511-15|588[0n]bp|Diptera|Chironomidae|BOLD:ABA1235
Orthocladiinae|[1509]|RRMFE203-15|588[0n]bp|Diptera|Chironomidae|BOLD:ABA1217
Chironomidae|[1510]|RRMFC311-15|552[0n]bp|Diptera|Chironomidae|BOLD:ACJ8377
Smittia sp. 23ES|[1511]|RRMPB3403-15|579[0n]bp|Diptera|Chironomidae|BOLD:AAH9641
Smittia|[1512]|RRMFB141-15|561[0n]bp|Diptera|Chironomidae|BOLD:AAN5355
Smittia sp. 22ES|[1513]|RRMPA695-15|561[0n]bp|Diptera|Chironomidae|BOLD:AAN5358
Smittia|[1514]|RRMFB224-15|567[0n]bp|Diptera|Chironomidae|BOLD:ACA2964
Chironomidae|[1515]|RRMFB647-15|624[0n]bp|Diptera|Chironomidae|BOLD:ACF8472
Smittia|[1516]|RRMFI906-15|636[0n]bp|Diptera|Chironomidae|BOLD:ABW7321
Smittia sp. 14ES|[1517]|RRMFB299-15|582[0n]bp|Diptera|Chironomidae|BOLD:AAM7064
Smittia|[1518]|RRSSA2820-15|549[0n]bp|Diptera|Chironomidae|BOLD:AAN5356
Cricotopus triannulatus|[1519]|RRMPB933-15|579[0n]bp|Diptera|Chironomidae|BOLD:AAP5920
Smittia edwardsi|[1520]|RRMFE144-15|594[0n]bp|Diptera|Chironomidae|BOLD:AAF4817
Smittia|[1521]|RRMPC1071-15|576[0n]bp|Diptera|Chironomidae|BOLD:AAZ5601
Orthocladius rivulorum|[1522]|RRMPB1244-15|561[0n]bp|Diptera|Chironomidae|BOLD:AAB3988
Cricotopus|[1523]|RRMPG614-15|576[0n]bp|Diptera|Chironomidae|BOLD:AAA5299
Cricotopus|[1524]|RRINV3554-15|608[0n]bp|Diptera|Chironomidae|
Chironomidae|[1525]|RRSSA856-15|549[0n]bp|Diptera|Chironomidae|BOLD:ACK7313
Orthocladius carlatus|[1526]|RRMFC115-15|594[0n]bp|Diptera|Chironomidae|BOLD:AAG1000
Rheocricotopus robacki|[1527]|RRMFE741-15|588[0n]bp|Diptera|Chironomidae|BOLD:AAM6249
Orthocladius oliveri|[1528]|RRMPB3417-15|579[0n]bp|Diptera|Chironomidae|BOLD:AAB7872
Orthocladius|[1529]|RRMFA016-15|579[0n]bp|Diptera|Chironomidae|BOLD:AAB2644
Orthocladius dorenus|[1530]|RRMPB1105-15|588[0n]bp|Diptera|Chironomidae|BOLD:ACV3368
Orthocladius dorenus|[1531]|RRMPB602-15|567[0n]bp|Diptera|Chironomidae|BOLD:AAB2641
Orthocladius|[1532]|RRMPB965-15|573[0n]bp|Diptera|Chironomidae|BOLD:AAG0991
Chironomidae|[1533]|RRMFI2198-15|632[0n]bp|Diptera|Chironomidae|BOLD:ACF6510
Chironomidae|[1534]|RRMFG192-15|588[0n]bp|Diptera|Chironomidae|BOLD:ACL4089
Chironomidae|[1535]|RRMPB4057-15|519[1n]bp|Diptera|Chironomidae|BOLD:ABV1203
Chironomidae|[1536]|RRMPC379-15|576[2n]bp|Diptera|Chironomidae|BOLD:ACU1897
Orthocladius dorenus|[1537]|RRMPB230-15|585[0n]bp|Diptera|Chironomidae|BOLD:AAB2645
Orthocladius|[1538]|RRMFA029-15|576[0n]bp|Diptera|Chironomidae|BOLD:ACF9627
Orthocladius|[1539]|RRMPA332-15|588[0n]bp|Diptera|Chironomidae|BOLD:ACF8117
Chironomidae|[1540]|RRMFD369-15|576[0n]bp|Diptera|Chironomidae|BOLD:ABV1455
Chironomidae|[1541]|RRMFD995-15|558[0n]bp|Diptera|Chironomidae|BOLD:ACJ5130
Chironomidae|[1542]|RRMFI1943-15|636[0n]bp|Diptera|Chironomidae|BOLD:ACR6131
Chironomidae|[1543]|RRMFC344-15|600[0n]bp|Diptera|Chironomidae|BOLD:ACR6132
Chironomidae|[1544]|RRMFD812-15|588[0n]bp|Diptera|Chironomidae|BOLD:ACR6101
Paratanytarsus sp. 7TE|[1545]|RRMFG1146-15|558[0n]bp|Diptera|Chironomidae|BOLD:AAP2907
Paratanytarsus sp. TE03|[1546]|RRMFB715-15|555[0n]bp|Diptera|Chironomidae|BOLD:AAE3675
Paratanytarsus|[1547]|RRMFD762-15|543[0n]bp|Diptera|Chironomidae|BOLD:ACE7452
Chironomidae|[1548]|RRMFE3309-15|594[0n]bp|Diptera|Chironomidae|BOLD:ACG2927
Paratanytarsus|[1549]|RRMPG114-15|558[0n]bp|Diptera|Chironomidae|BOLD:ACF7858
Paratanytarsus|[1550]|RRMPC076-15|537[0n]bp|Diptera|Chironomidae|BOLD:ACF7859
Paratanytarsus|[1551]|RRMPB3056-15|543[2n]bp|Diptera|Chironomidae|BOLD:ABV9028
Paratanytarsus|[1552]|RRMPG172-15|570[0n]bp|Diptera|Chironomidae|BOLD:ACV1253
Paratanytarsus dissimilis|[1553]|RRMFC1120-15|603[2n]bp|Diptera|Chironomidae|BOLD:AAE3698
Paratanytarsus|[1554]|RRMPB3007-15|528[0n]bp|Diptera|Chironomidae|BOLD:AAI2606
Paratanytarsus|[1555]|RRMFB093-15|561[0n]bp|Diptera|Chironomidae|BOLD:ACC1156
Paratanytarsus grimmii|[1556]|RRMFE844-15|588[0n]bp|Diptera|Chironomidae|BOLD:AAD1485
Paratanytarsus laccophilus|[1557]|RRMFC1060-15|555[0n]bp|Diptera|Chironomidae|BOLD:ACF2457
Paratanytarsus laccophilus|[1558]|RRMPB1236-15|600[1n]bp|Diptera|Chironomidae|BOLD:AAC8842
Chironomidae|[1559]|RRMFI1734-15|633[0n]bp|Diptera|Chironomidae|BOLD:ACW1260
Tanytarsus guerlus|[1560]|RRMPC1086-15|588[0n]bp|Diptera|Chironomidae|BOLD:AAC4523
Chironomidae|[1561]|RRSSA1108-15|588[0n]bp|Diptera|Chironomidae|BOLD:AAG5465
Chironomidae|[1562]|RRINV3611-15|625[0n]bp|Diptera|Chironomidae|
Chironominae|[1563]|RRINV2080-15|632[0n]bp|Diptera|Chironomidae|
Polypedilum|[1564]|RRSSA3270-15|576[0n]bp|Diptera|Chironomidae|BOLD:ACD9755
Phaenopsectra|[1565]|RRMFE1922-15|588[0n]bp|Diptera|Chironomidae|BOLD:AAM6287
Chironomidae|[1566]|RRMFD1047-15|567[1n]bp|Diptera|Chironomidae|BOLD:ACI3882
Paratendipes|[1567]|RRSSA3251-15|576[0n]bp|Diptera|Chironomidae|BOLD:AAG5473
Lauterborniella agrayloides|[1568]|RRMFE417-15|609[0n]bp|Diptera|Chironomidae|BOLD:AAN5343
Microtendipes pedellus|[1569]|RRMFD711-15|525[1n]bp|Diptera|Chironomidae|BOLD:AAF0707

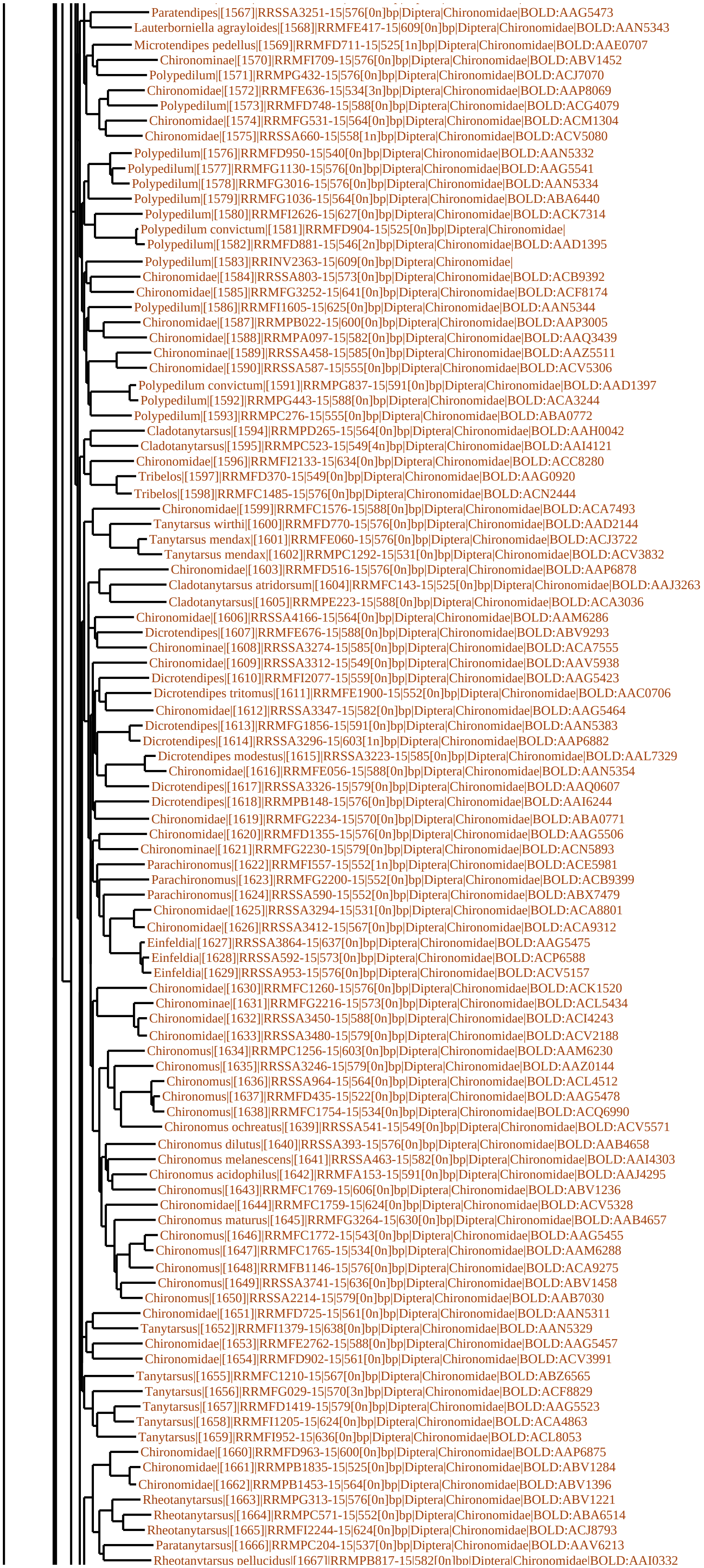

Paratendipes|[1567]|RRSSA3251-15|576[0n]bp|Diptera|Chironomidae|BOLD:AAG5473
Lauterborniella agrayloides|[1568]|RRMFE417-15|609[0n]bp|Diptera|Chironomidae|BOLD:AAN5343
Microtendipes pedellus|[1569]|RRMFD711-15|525[1n]bp|Diptera|Chironomidae|BOLD:AAE0707
Chironominae|[1570]|RRMFI709-15|576[0n]bp|Diptera|Chironomidae|BOLD:ABV1452
Polypedilum|[1571]|RRMPG432-15|576[0n]bp|Diptera|Chironomidae|BOLD:ACJ7070
Chironomidae|[1572]|RRMFE636-15|534[3n]bp|Diptera|Chironomidae|BOLD:AAP8069
Polypedilum|[1573]|RRMFD748-15|588[0n]bp|Diptera|Chironomidae|BOLD:ACG4079
Chironomidae|[1574]|RRMFG531-15|564[0n]bp|Diptera|Chironomidae|BOLD:ACM1304
Chironomidae|[1575]|RRSSA660-15|558[1n]bp|Diptera|Chironomidae|BOLD:ACV5080
Polypedilum|[1576]|RRMFD950-15|540[0n]bp|Diptera|Chironomidae|BOLD:AAN5332
Polypedilum|[1577]|RRMFG1130-15|576[0n]bp|Diptera|Chironomidae|BOLD:AAG5541
Polypedilum|[1578]|RRMFG3016-15|576[0n]bp|Diptera|Chironomidae|BOLD:AAN5334
Polypedilum|[1579]|RRMFG1036-15|564[0n]bp|Diptera|Chironomidae|BOLD:ABA6440
Polypedilum|[1580]|RRMFI2626-15|627[0n]bp|Diptera|Chironomidae|BOLD:ACK7314
Polypedilum convictum|[1581]|RRMFD904-15|525[0n]bp|Diptera|Chironomidae|
Polypedilum|[1582]|RRMFD881-15|546[2n]bp|Diptera|Chironomidae|BOLD:AAD1395
Polypedilum|[1583]|RRINV2363-15|609[0n]bp|Diptera|Chironomidae|
Chironomidae|[1584]|RRSSA803-15|573[0n]bp|Diptera|Chironomidae|BOLD:ACB9392
Chironomidae|[1585]|RRMFG3252-15|641[0n]bp|Diptera|Chironomidae|BOLD:ACF8174
Polypedilum|[1586]|RRMFI1605-15|625[0n]bp|Diptera|Chironomidae|BOLD:AAN5344
Chironomidae|[1587]|RRMPB022-15|600[0n]bp|Diptera|Chironomidae|BOLD:AAP3005
Chironomidae|[1588]|RRMPA097-15|582[0n]bp|Diptera|Chironomidae|BOLD:AAQ3439
Chironominae|[1589]|RRSSA458-15|585[0n]bp|Diptera|Chironomidae|BOLD:AAZ5511
Chironomidae|[1590]|RRSSA587-15|555[0n]bp|Diptera|Chironomidae|BOLD:ACV5306
Polypedilum convictum|[1591]|RRMPG837-15|591[0n]bp|Diptera|Chironomidae|BOLD:AAD1397
Polypedilum|[1592]|RRMPG443-15|588[0n]bp|Diptera|Chironomidae|BOLD:ACA3244
Polypedilum|[1593]|RRMPC276-15|555[0n]bp|Diptera|Chironomidae|BOLD:ABA0772
Cladotanytarsus|[1594]|RRMPD265-15|564[0n]bp|Diptera|Chironomidae|BOLD:AAH0042
Cladotanytarsus|[1595]|RRMPC523-15|549[4n]bp|Diptera|Chironomidae|BOLD:AAI4121
Chironomidae|[1596]|RRMFI2133-15|634[0n]bp|Diptera|Chironomidae|BOLD:ACC8280
Tribelos|[1597]|RRMFD370-15|549[0n]bp|Diptera|Chironomidae|BOLD:AAG0920
Tribelos|[1598]|RRMFC1485-15|576[0n]bp|Diptera|Chironomidae|BOLD:ACN2444
Chironomidae|[1599]|RRMFC1576-15|588[0n]bp|Diptera|Chironomidae|BOLD:ACA7493
Tanytarsus wirthi|[1600]|RRMFD770-15|576[0n]bp|Diptera|Chironomidae|BOLD:AAD2144
Tanytarsus mendax|[1601]|RRMFE060-15|576[0n]bp|Diptera|Chironomidae|BOLD:ACJ3722
Tanytarsus mendax|[1602]|RRMPC1292-15|531[0n]bp|Diptera|Chironomidae|BOLD:ACV3832
Chironomidae|[1603]|RRMFD516-15|576[0n]bp|Diptera|Chironomidae|BOLD:AAP6878
Cladotanytarsus atridorsum|[1604]|RRMFC143-15|525[0n]bp|Diptera|Chironomidae|BOLD:AAJ3263
Cladotanytarsus|[1605]|RRMPE223-15|588[0n]bp|Diptera|Chironomidae|BOLD:ACA3036
Chironomidae|[1606]|RRSSA4166-15|564[0n]bp|Diptera|Chironomidae|BOLD:AAM6286
Dicrotendipes|[1607]|RRMFE676-15|588[0n]bp|Diptera|Chironomidae|BOLD:ABV9293
Chironominae|[1608]|RRSSA3274-15|585[0n]bp|Diptera|Chironomidae|BOLD:ACA7555
Chironomidae|[1609]|RRSSA3312-15|549[0n]bp|Diptera|Chironomidae|BOLD:AAV5938
Dicrotendipes|[1610]|RRMFI2077-15|559[0n]bp|Diptera|Chironomidae|BOLD:AAG5423
Dicrotendipes tritomus|[1611]|RRMFE1900-15|552[0n]bp|Diptera|Chironomidae|BOLD:AAC0706
Chironomidae|[1612]|RRSSA3347-15|582[0n]bp|Diptera|Chironomidae|BOLD:AAG5464
Dicrotendipes|[1613]|RRMFG1856-15|591[0n]bp|Diptera|Chironomidae|BOLD:AAN5383
Dicrotendipes|[1614]|RRSSA3296-15|603[1n]bp|Diptera|Chironomidae|BOLD:AAP6882
Dicrotendipes modestus|[1615]|RRSSA3223-15|585[0n]bp|Diptera|Chironomidae|BOLD:AAL7329
Chironomidae|[1616]|RRMFE056-15|588[0n]bp|Diptera|Chironomidae|BOLD:AAN5354
Dicrotendipes|[1617]|RRSSA3326-15|579[0n]bp|Diptera|Chironomidae|BOLD:AAQ0607
Dicrotendipes|[1618]|RRMPB148-15|576[0n]bp|Diptera|Chironomidae|BOLD:AAI6244
Chironomidae|[1619]|RRMFG2234-15|570[0n]bp|Diptera|Chironomidae|BOLD:ABA0771
Chironomidae|[1620]|RRMFD1355-15|576[0n]bp|Diptera|Chironomidae|BOLD:AAG5506
Chironominae|[1621]|RRMFG2230-15|579[0n]bp|Diptera|Chironomidae|BOLD:ACN5893
Parachironomus|[1622]|RRMFI557-15|552[1n]bp|Diptera|Chironomidae|BOLD:ACE5981
Parachironomus|[1623]|RRMFG2200-15|552[0n]bp|Diptera|Chironomidae|BOLD:ACB9399
Parachironomus|[1624]|RRSSA590-15|552[0n]bp|Diptera|Chironomidae|BOLD:ABX7479
Chironomidae|[1625]|RRSSA3294-15|531[0n]bp|Diptera|Chironomidae|BOLD:ACA8801
Chironomidae|[1626]|RRSSA3412-15|567[0n]bp|Diptera|Chironomidae|BOLD:ACA9312
Einfeldia|[1627]|RRSSA3864-15|637[0n]bp|Diptera|Chironomidae|BOLD:AAG5475
Einfeldia|[1628]|RRSSA592-15|573[0n]bp|Diptera|Chironomidae|BOLD:ACP6588
Einfeldia|[1629]|RRSSA953-15|576[0n]bp|Diptera|Chironomidae|BOLD:ACV5157
Chironomidae|[1630]|RRMFC1260-15|576[0n]bp|Diptera|Chironomidae|BOLD:ACK1520
Chironominae|[1631]|RRMFG2216-15|573[0n]bp|Diptera|Chironomidae|BOLD:ACL5434
Chironomidae|[1632]|RRSSA3450-15|588[0n]bp|Diptera|Chironomidae|BOLD:ACI4243
Chironomidae|[1633]|RRSSA3480-15|579[0n]bp|Diptera|Chironomidae|BOLD:ACV2188
Chironomus|[1634]|RRMPC1256-15|603[0n]bp|Diptera|Chironomidae|BOLD:AAM6230
Chironomus|[1635]|RRSSA3246-15|579[0n]bp|Diptera|Chironomidae|BOLD:AAZ0144
Chironomus|[1636]|RRSSA964-15|564[0n]bp|Diptera|Chironomidae|BOLD:ACL4512
Chironomus|[1637]|RRMFD435-15|522[0n]bp|Diptera|Chironomidae|BOLD:AAG5478
Chironomus|[1638]|RRMFC1754-15|534[0n]bp|Diptera|Chironomidae|BOLD:ACQ6990
Chironomus ochreatus|[1639]|RRSSA541-15|549[0n]bp|Diptera|Chironomidae|BOLD:ACV5571
Chironomus dilutus|[1640]|RRSSA393-15|576[0n]bp|Diptera|Chironomidae|BOLD:AAB4658
Chironomus melanescens|[1641]|RRSSA463-15|582[0n]bp|Diptera|Chironomidae|BOLD:AAI4303
Chironomus acidophilus|[1642]|RRMFA153-15|591[0n]bp|Diptera|Chironomidae|BOLD:AAJ4295
Chironomus|[1643]|RRMFC1769-15|606[0n]bp|Diptera|Chironomidae|BOLD:ABV1236
Chironomidae|[1644]|RRMFC1759-15|624[0n]bp|Diptera|Chironomidae|BOLD:ACV5328
Chironomus maturus|[1645]|RRMFG3264-15|630[0n]bp|Diptera|Chironomidae|BOLD:AAB4657
Chironomus|[1646]|RRMFC1772-15|543[0n]bp|Diptera|Chironomidae|BOLD:AAG5455
Chironomus|[1647]|RRMFC1765-15|534[0n]bp|Diptera|Chironomidae|BOLD:AAM6288
Chironomus|[1648]|RRMFB1146-15|576[0n]bp|Diptera|Chironomidae|BOLD:ACA9275
Chironomus|[1649]|RRSSA3741-15|636[0n]bp|Diptera|Chironomidae|BOLD:ABV1458
Chironomus|[1650]|RRSSA2214-15|579[0n]bp|Diptera|Chironomidae|BOLD:AAB7030
Chironomidae|[1651]|RRMFD725-15|561[0n]bp|Diptera|Chironomidae|BOLD:AAN5311
Tanytarsus|[1652]|RRMFI1379-15|638[0n]bp|Diptera|Chironomidae|BOLD:AAN5329
Chironomidae|[1653]|RRMFE2762-15|588[0n]bp|Diptera|Chironomidae|BOLD:AAG5457
Chironomidae|[1654]|RRMFD902-15|561[0n]bp|Diptera|Chironomidae|BOLD:ACV3991
Tanytarsus|[1655]|RRMFC1210-15|567[0n]bp|Diptera|Chironomidae|BOLD:ABZ6565
Tanytarsus|[1656]|RRMFG029-15|570[3n]bp|Diptera|Chironomidae|BOLD:ACF8829
Tanytarsus|[1657]|RRMFD1419-15|579[0n]bp|Diptera|Chironomidae|BOLD:AAG5523
Tanytarsus|[1658]|RRMFI1205-15|624[0n]bp|Diptera|Chironomidae|BOLD:ACA4863
Tanytarsus|[1659]|RRMFI952-15|636[0n]bp|Diptera|Chironomidae|BOLD:ACL8053
Chironomidae|[1660]|RRMFD963-15|600[0n]bp|Diptera|Chironomidae|BOLD:AAP6875
Chironomidae|[1661]|RRMPB1835-15|525[0n]bp|Diptera|Chironomidae|BOLD:ABV1284
Chironomidae|[1662]|RRMPB1453-15|564[0n]bp|Diptera|Chironomidae|BOLD:ABV1396
Rheotanytarsus|[1663]|RRMPG313-15|576[0n]bp|Diptera|Chironomidae|BOLD:ABV1221
Rheotanytarsus|[1664]|RRMPC571-15|552[0n]bp|Diptera|Chironomidae|BOLD:ABA6514
Rheotanytarsus|[1665]|RRMFI2244-15|624[0n]bp|Diptera|Chironomidae|BOLD:ACJ8793
Paratanytarsus|[1666]|RRMPC204-15|537[0n]bp|Diptera|Chironomidae|BOLD:AAV6213
Rheotanytarsus pellucidus|[1667]|RRMPB817-15|582[0n]bp|Diptera|Chironomidae|BOLD:AAI0332

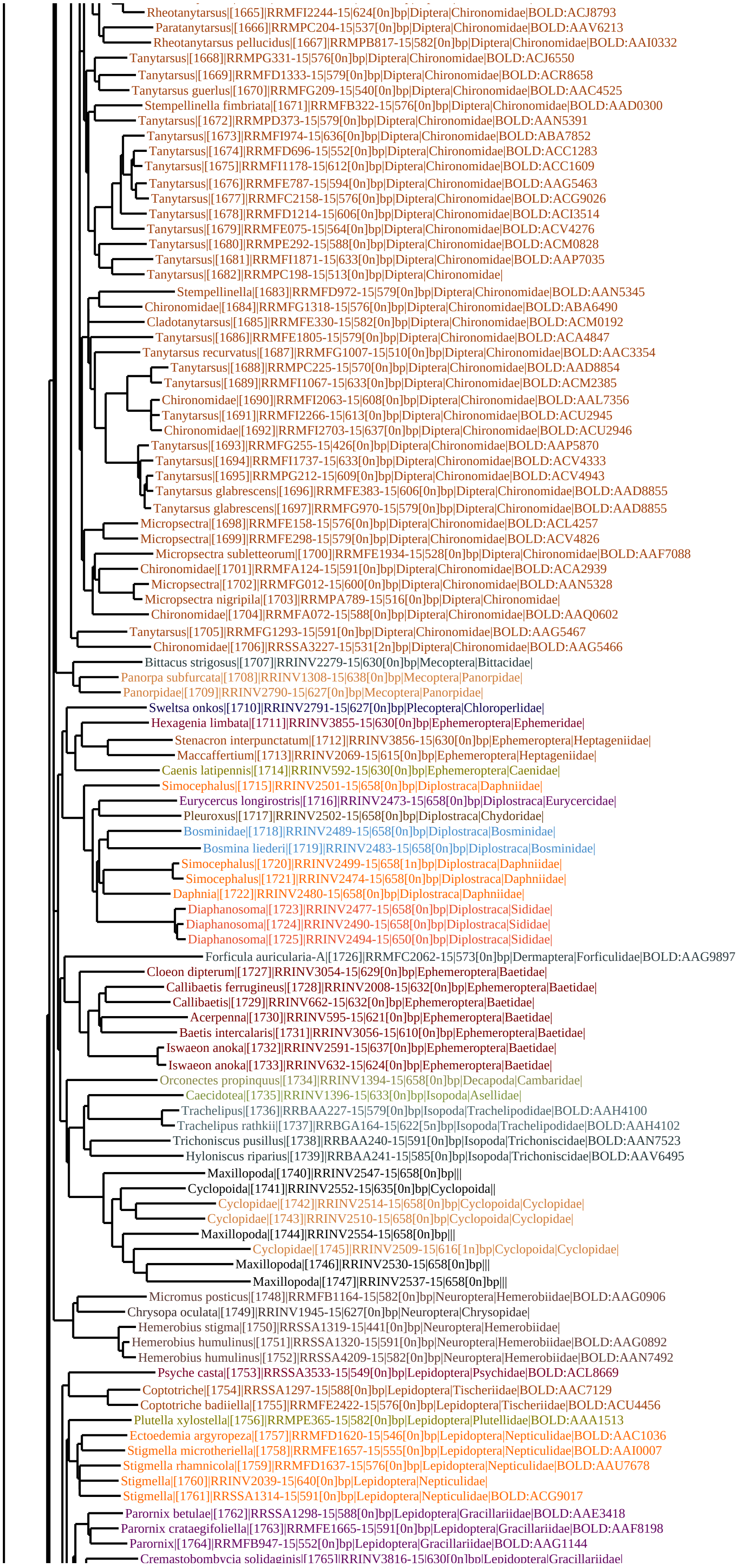
Rheotanytarsus|[1665]|RRMFI2244-15|624[0n]bp|Diptera|Chironomidae|BOLD:ACJ8793
Paratanytarsus|[1666]|RRMPC204-15|537[0n]bp|Diptera|Chironomidae|BOLD:AAV6213
Rheotanytarsus pellucidus|[1667]|RRMPB817-15|582[0n]bp|Diptera|Chironomidae|BOLD:AAI0332
Tanytarsus|[1668]|RRMPG331-15|576[0n]bp|Diptera|Chironomidae|BOLD:ACJ6550
Tanytarsus|[1669]|RRMFD1333-15|579[0n]bp|Diptera|Chironomidae|BOLD:ACR8658
Tanytarsus guerlus|[1670]|RRMFG209-15|540[0n]bp|Diptera|Chironomidae|BOLD:AAC4525
Stempellinella fimbriata|[1671]|RRMFB322-15|576[0n]bp|Diptera|Chironomidae|BOLD:AAD0300
Tanytarsus|[1672]|RRMPD373-15|579[0n]bp|Diptera|Chironomidae|BOLD:AAN5391
Tanytarsus|[1673]|RRMFI974-15|636[0n]bp|Diptera|Chironomidae|BOLD:ABA7852
Tanytarsus|[1674]|RRMFD696-15|552[0n]bp|Diptera|Chironomidae|BOLD:ACC1283
Tanytarsus|[1675]|RRMFI1178-15|612[0n]bp|Diptera|Chironomidae|BOLD:ACC1609
Tanytarsus|[1676]|RRMFE787-15|594[0n]bp|Diptera|Chironomidae|BOLD:AAG5463
Tanytarsus|[1677]|RRMFC2158-15|576[0n]bp|Diptera|Chironomidae|BOLD:ACG9026
Tanytarsus|[1678]|RRMFD1214-15|606[0n]bp|Diptera|Chironomidae|BOLD:ACI3514
Tanytarsus|[1679]|RRMFE075-15|564[0n]bp|Diptera|Chironomidae|BOLD:ACV4276
Tanytarsus|[1680]|RRMPE292-15|588[0n]bp|Diptera|Chironomidae|BOLD:ACM0828
Tanytarsus|[1681]|RRMFI1871-15|633[0n]bp|Diptera|Chironomidae|BOLD:AAP7035
Tanytarsus|[1682]|RRMPC198-15|513[0n]bp|Diptera|Chironomidae|
Stempellinella|[1683]|RRMFD972-15|579[0n]bp|Diptera|Chironomidae|BOLD:AAN5345
Chironomidae|[1684]|RRMFG1318-15|576[0n]bp|Diptera|Chironomidae|BOLD:ABA6490
Cladotanytarsus|[1685]|RRMFE330-15|582[0n]bp|Diptera|Chironomidae|BOLD:ACM0192
Tanytarsus|[1686]|RRMFE1805-15|579[0n]bp|Diptera|Chironomidae|BOLD:ACA4847
Tanytarsus recurvatus|[1687]|RRMFG1007-15|510[0n]bp|Diptera|Chironomidae|BOLD:AAC3354
Tanytarsus|[1688]|RRMPC225-15|570[0n]bp|Diptera|Chironomidae|BOLD:AAD8854
Tanytarsus|[1689]|RRMFI1067-15|633[0n]bp|Diptera|Chironomidae|BOLD:ACM2385
Chironomidae|[1690]|RRMFI2063-15|608[0n]bp|Diptera|Chironomidae|BOLD:AAL7356
Tanytarsus|[1691]|RRMFI2266-15|613[0n]bp|Diptera|Chironomidae|BOLD:ACU2945
Chironomidae|[1692]|RRMFI2703-15|637[0n]bp|Diptera|Chironomidae|BOLD:ACU2946
Tanytarsus|[1693]|RRMFG255-15|426[0n]bp|Diptera|Chironomidae|BOLD:AAP5870
Tanytarsus|[1694]|RRMFI1737-15|633[0n]bp|Diptera|Chironomidae|BOLD:ACV4333
Tanytarsus|[1695]|RRMPG212-15|609[0n]bp|Diptera|Chironomidae|BOLD:ACV4943
Tanytarsus glabrescens|[1696]|RRMFE383-15|606[0n]bp|Diptera|Chironomidae|BOLD:AAD8855
Tanytarsus glabrescens|[1697]|RRMFG970-15|579[0n]bp|Diptera|Chironomidae|BOLD:AAD8855
Micropsectra|[1698]|RRMFE158-15|576[0n]bp|Diptera|Chironomidae|BOLD:ACL4257
Micropsectra|[1699]|RRMFE298-15|579[0n]bp|Diptera|Chironomidae|BOLD:ACV4826
Micropsectra subletteorum|[1700]|RRMFE1934-15|528[0n]bp|Diptera|Chironomidae|BOLD:AAF7088
Chironomidae|[1701]|RRMFA124-15|591[0n]bp|Diptera|Chironomidae|BOLD:ACA2939
Micropsectra|[1702]|RRMFG012-15|600[0n]bp|Diptera|Chironomidae|BOLD:AAN5328
Micropsectra nigripila|[1703]|RRMPA789-15|516[0n]bp|Diptera|Chironomidae|
Chironomidae|[1704]|RRMFA072-15|588[0n]bp|Diptera|Chironomidae|BOLD:AAQ0602
Tanytarsus|[1705]|RRMFG1293-15|591[0n]bp|Diptera|Chironomidae|BOLD:AAG5467
Chironomidae|[1706]|RRSSA3227-15|531[2n]bp|Diptera|Chironomidae|BOLD:AAG5466
Bittacus strigosus|[1707]|RRINV2279-15|630[0n]bp|Mecoptera|Bittacidae|
Panorpa subfurcata|[1708]|RRINV1308-15|638[0n]bp|Mecoptera|Panorpidae|
Panorpidae|[1709]|RRINV2790-15|627[0n]bp|Mecoptera|Panorpidae|
Sweltsa onkos|[1710]|RRINV2791-15|627[0n]bp|Plecoptera|Chloroperlidae|
Hexagenia limbata|[1711]|RRINV3855-15|630[0n]bp|Ephemeroptera|Ephemeridae|
Stenacron interpunctatum|[1712]|RRINV3856-15|630[0n]bp|Ephemeroptera|Heptageniidae|
Maccaffertium|[1713]|RRINV2069-15|615[0n]bp|Ephemeroptera|Heptageniidae|
Caenis latipennis|[1714]|RRINV592-15|630[0n]bp|Ephemeroptera|Caenidae|
Simocephalus|[1715]|RRINV2501-15|658[0n]bp|Diplostraca|Daphniidae|
Eurycercus longirostris|[1716]|RRINV2473-15|658[0n]bp|Diplostraca|Eurycercidae|
Pleuroxus|[1717]|RRINV2502-15|658[0n]bp|Diplostraca|Chydoridae|
Bosminidae|[1718]|RRINV2489-15|658[0n]bp|Diplostraca|Bosminidae|
Bosmina liederi|[1719]|RRINV2483-15|658[0n]bp|Diplostraca|Bosminidae|
Simocephalus|[1720]|RRINV2499-15|658[1n]bp|Diplostraca|Daphniidae|
Simocephalus|[1721]|RRINV2474-15|658[0n]bp|Diplostraca|Daphniidae|
Daphnia|[1722]|RRINV2480-15|658[0n]bp|Diplostraca|Daphniidae|
Diaphanosoma|[1723]|RRINV2477-15|658[0n]bp|Diplostraca|Sididae|
Diaphanosoma|[1724]|RRINV2490-15|658[0n]bp|Diplostraca|Sididae|
Diaphanosoma|[1725]|RRINV2494-15|650[0n]bp|Diplostraca|Sididae|
Forficula auricularia-A|[1726]|RRMFC2062-15|573[0n]bp|Dermaptera|Forficulidae|BOLD:AAG9897
Cloeon dipterum|[1727]|RRINV3054-15|629[0n]bp|Ephemeroptera|Baetidae|
Callibaetis ferrugineus|[1728]|RRINV2008-15|632[0n]bp|Ephemeroptera|Baetidae|
Callibaetis|[1729]|RRINV662-15|632[0n]bp|Ephemeroptera|Baetidae|
Acerpenna|[1730]|RRINV595-15|621[0n]bp|Ephemeroptera|Baetidae|
Baetis intercalaris|[1731]|RRINV3056-15|610[0n]bp|Ephemeroptera|Baetidae|
Iswaeon anoka|[1732]|RRINV2591-15|637[0n]bp|Ephemeroptera|Baetidae|
Iswaeon anoka|[1733]|RRINV632-15|624[0n]bp|Ephemeroptera|Baetidae|
Orconectes propinquus|[1734]|RRINV1394-15|658[0n]bp|Decapoda|Cambaridae|
Caecidotea|[1735]|RRINV1396-15|633[0n]bp|Isopoda|Asellidae|
Trachelipus|[1736]|RRBAA227-15|579[0n]bp|Isopoda|Trachelipodidae|BOLD:AAH4100
Trachelipus rathkii|[1737]|RRBGA164-15|622[5n]bp|Isopoda|Trachelipodidae|BOLD:AAH4102
Trichoniscus pusillus|[1738]|RRBAA240-15|591[0n]bp|Isopoda|Trichoniscidae|BOLD:AAN7523
Hyloniscus riparius|[1739]|RRBAA241-15|585[0n]bp|Isopoda|Trichoniscidae|BOLD:AAV6495
Maxillopoda|[1740]|RRINV2547-15|658[0n]bp|||
Cyclopoida|[1741]|RRINV2552-15|635[0n]bp|Cyclopoida||
Cyclopidae|[1742]|RRINV2514-15|658[0n]bp|Cyclopoida|Cyclopidae|
Cyclopidae|[1743]|RRINV2510-15|658[0n]bp|Cyclopoida|Cyclopidae|
Maxillopoda|[1744]|RRINV2554-15|658[0n]bp|||
Cyclopidae|[1745]|RRINV2509-15|616[1n]bp|Cyclopoida|Cyclopidae|
Maxillopoda|[1746]|RRINV2530-15|658[0n]bp|||
Maxillopoda|[1747]|RRINV2537-15|658[0n]bp|||
Micromus posticus|[1748]|RRMFB1164-15|582[0n]bp|Neuroptera|Hemerobiidae|BOLD:AAG0906
Chrysopa oculata|[1749]|RRINV1945-15|627[0n]bp|Neuroptera|Chrysopidae|
Hemerobius stigma|[1750]|RRSSA1319-15|441[0n]bp|Neuroptera|Hemerobiidae|
Hemerobius humulinus|[1751]|RRSSA1320-15|591[0n]bp|Neuroptera|Hemerobiidae|BOLD:AAG0892
Hemerobius humulinus|[1752]|RRSSA4209-15|582[0n]bp|Neuroptera|Hemerobiidae|BOLD:AAN7492
Psyche casta|[1753]|RRSSA3533-15|549[0n]bp|Lepidoptera|Psychidae|BOLD:ACL8669
Coptotriche|[1754]|RRSSA1297-15|588[0n]bp|Lepidoptera|Tischeriidae|BOLD:AAC7129
Coptotriche badiiella|[1755]|RRMFE2422-15|576[0n]bp|Lepidoptera|Tischeriidae|BOLD:ACU4456
Plutella xylostella|[1756]|RRMPE365-15|582[0n]bp|Lepidoptera|Plutellidae|BOLD:AAA1513
Ectoedemia argyropeza|[1757]|RRMFD1620-15|546[0n]bp|Lepidoptera|Nepticulidae|BOLD:AAC1036
Stigmella microtheriella|[1758]|RRMFE1657-15|555[0n]bp|Lepidoptera|Nepticulidae|BOLD:AAI0007
Stigmella rhamnicola|[1759]|RRMFD1637-15|576[0n]bp|Lepidoptera|Nepticulidae|BOLD:AAU7678
Stigmella|[1760]|RRINV2039-15|640[0n]bp|Lepidoptera|Nepticulidae|
Stigmella|[1761]|RRSSA1314-15|591[0n]bp|Lepidoptera|Nepticulidae|BOLD:ACG9017
Parornix betulae|[1762]|RRSSA1298-15|588[0n]bp|Lepidoptera|Gracillariidae|BOLD:AAE3418
Parornix crataegifoliella|[1763]|RRMFE1665-15|591[0n]bp|Lepidoptera|Gracillariidae|BOLD:AAF8198
Parornix|[1764]|RRMFB947-15|552[0n]bp|Lepidoptera|Gracillariidae|BOLD:AAG1144
Cremastobombycia solidaginis|[1765]|RRINV3816-15|630[0n]bp|Lepidoptera|Gracillariidae|

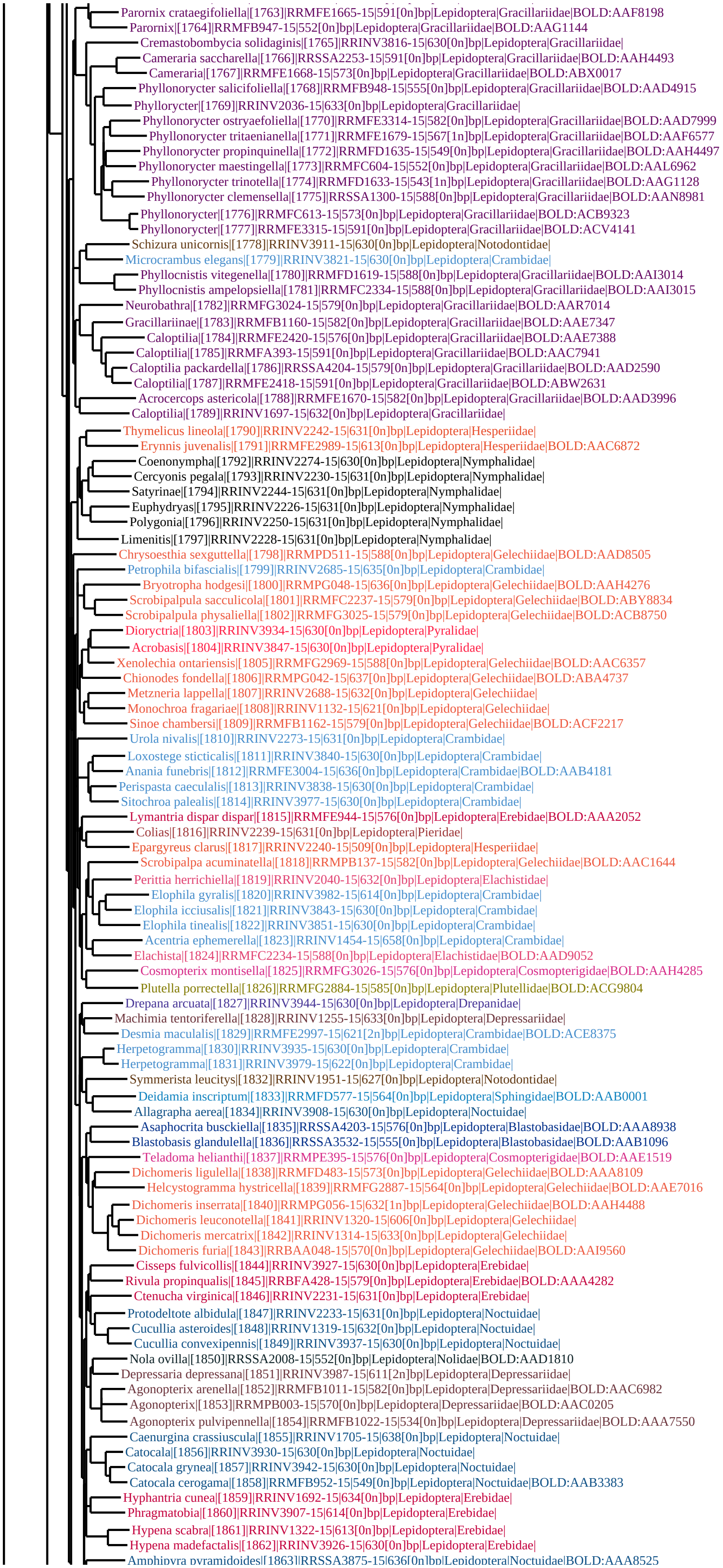
Parornix crataegifoliella|[1763]|RRMFE1665-15|591[0n]bp|Lepidoptera|Gracillariidae|BOLD:AAF8198
Parornix|[1764]|RRMFB947-15|552[0n]bp|Lepidoptera|Gracillariidae|BOLD:AAG1144
Cremastobombycia solidaginis|[1765]|RRINV3816-15|630[0n]bp|Lepidoptera|Gracillariidae|
Cameraria saccharella|[1766]|RRSSA2253-15|591[0n]bp|Lepidoptera|Gracillariidae|BOLD:AAH4493
Cameraria|[1767]|RRMFE1668-15|573[0n]bp|Lepidoptera|Gracillariidae|BOLD:ABX0017
Phyllonorycter salicifoliella|[1768]|RRMFB948-15|555[0n]bp|Lepidoptera|Gracillariidae|BOLD:AAD4915
Phyllorycter|[1769]|RRINV2036-15|633[0n]bp|Lepidoptera|Gracillariidae|
Phyllonorycter ostryaefoliella|[1770]|RRMFE3314-15|582[0n]bp|Lepidoptera|Gracillariidae|BOLD:AAD7999
Phyllonorycter tritaenianella|[1771]|RRMFE1679-15|567[1n]bp|Lepidoptera|Gracillariidae|BOLD:AAF6577
Phyllonorycter propinquinella|[1772]|RRMFD1635-15|549[0n]bp|Lepidoptera|Gracillariidae|BOLD:AAH4497
Phyllonorycter maestingella|[1773]|RRMFC604-15|552[0n]bp|Lepidoptera|Gracillariidae|BOLD:AAL6962
Phyllonorycter trinotella|[1774]|RRMFD1633-15|543[1n]bp|Lepidoptera|Gracillariidae|BOLD:AAG1128
Phyllonorycter clemensella|[1775]|RRSSA1300-15|588[0n]bp|Lepidoptera|Gracillariidae|BOLD:AAN8981
Phyllonorycter|[1776]|RRMFC613-15|573[0n]bp|Lepidoptera|Gracillariidae|BOLD:ACB9323
Phyllonorycter|[1777]|RRMFE3315-15|591[0n]bp|Lepidoptera|Gracillariidae|BOLD:ACV4141
Schizura unicornis|[1778]|RRINV3911-15|630[0n]bp|Lepidoptera|Notodontidae|
Microcrambus elegans|[1779]|RRINV3821-15|630[0n]bp|Lepidoptera|Crambidae|
Phyllocnistis vitegenella|[1780]|RRMFD1619-15|588[0n]bp|Lepidoptera|Gracillariidae|BOLD:AAI3014
Phyllocnistis ampelopsiella|[1781]|RRMFC2334-15|588[0n]bp|Lepidoptera|Gracillariidae|BOLD:AAI3015
Neurobathra|[1782]|RRMFG3024-15|579[0n]bp|Lepidoptera|Gracillariidae|BOLD:AAR7014
Gracillariinae|[1783]|RRMFB1160-15|582[0n]bp|Lepidoptera|Gracillariidae|BOLD:AAE7347
Caloptilia|[1784]|RRMFE2420-15|576[0n]bp|Lepidoptera|Gracillariidae|BOLD:AAE7388
Caloptilia|[1785]|RRMFA393-15|591[0n]bp|Lepidoptera|Gracillariidae|BOLD:AAC7941
Caloptilia packardella|[1786]|RRSSA4204-15|579[0n]bp|Lepidoptera|Gracillariidae|BOLD:AAD2590
Caloptilia|[1787]|RRMFE2418-15|591[0n]bp|Lepidoptera|Gracillariidae|BOLD:ABW2631
Acrocercops astericola|[1788]|RRMFE1670-15|582[0n]bp|Lepidoptera|Gracillariidae|BOLD:AAD3996
Caloptilia|[1789]|RRINV1697-15|632[0n]bp|Lepidoptera|Gracillariidae|
Thymelicus lineola|[1790]|RRINV2242-15|631[0n]bp|Lepidoptera|Hesperiidae|
Erynnis juvenalis|[1791]|RRMFE2989-15|613[0n]bp|Lepidoptera|Hesperiidae|BOLD:AAC6872
Coenonympha|[1792]|RRINV2274-15|630[0n]bp|Lepidoptera|Nymphalidae|
Cercyonis pegala|[1793]|RRINV2230-15|631[0n]bp|Lepidoptera|Nymphalidae|
Satyrinae|[1794]|RRINV2244-15|631[0n]bp|Lepidoptera|Nymphalidae|
Euphydryas|[1795]|RRINV2226-15|631[0n]bp|Lepidoptera|Nymphalidae|
Polygonia|[1796]|RRINV2250-15|631[0n]bp|Lepidoptera|Nymphalidae|
Limenitis|[1797]|RRINV2228-15|631[0n]bp|Lepidoptera|Nymphalidae|
Chrysoesthia sexguttella|[1798]|RRMPD511-15|588[0n]bp|Lepidoptera|Gelechiidae|BOLD:AAD8505
Petrophila bifascialis|[1799]|RRINV2685-15|635[0n]bp|Lepidoptera|Crambidae|
Bryotropha hodgesi|[1800]|RRMPG048-15|636[0n]bp|Lepidoptera|Gelechiidae|BOLD:AAH4276
Scrobipalpula sacculicola|[1801]|RRMFC2237-15|579[0n]bp|Lepidoptera|Gelechiidae|BOLD:ABY8834
Scrobipalpula physaliella|[1802]|RRMFG3025-15|579[0n]bp|Lepidoptera|Gelechiidae|BOLD:ACB8750
Dioryctria|[1803]|RRINV3934-15|630[0n]bp|Lepidoptera|Pyralidae|
Acrobasis|[1804]|RRINV3847-15|630[0n]bp|Lepidoptera|Pyralidae|
Xenolechia ontariensis|[1805]|RRMFG2969-15|588[0n]bp|Lepidoptera|Gelechiidae|BOLD:AAC6357
Chionodes fondella|[1806]|RRMPG042-15|637[0n]bp|Lepidoptera|Gelechiidae|BOLD:ABA4737
Metzneria lappella|[1807]|RRINV2688-15|632[0n]bp|Lepidoptera|Gelechiidae|
Monochroa fragariae|[1808]|RRINV1132-15|621[0n]bp|Lepidoptera|Gelechiidae|
Sinoe chambersi|[1809]|RRMFB1162-15|579[0n]bp|Lepidoptera|Gelechiidae|BOLD:ACF2217
Urola nivalis|[1810]|RRINV2273-15|631[0n]bp|Lepidoptera|Crambidae|
Loxostege sticticalis|[1811]|RRINV3840-15|630[0n]bp|Lepidoptera|Crambidae|
Anania funebris|[1812]|RRMFE3004-15|636[0n]bp|Lepidoptera|Crambidae|BOLD:AAB4181
Perispasta caeculalis|[1813]|RRINV3838-15|630[0n]bp|Lepidoptera|Crambidae|
Sitochroa palealis|[1814]|RRINV3977-15|630[0n]bp|Lepidoptera|Crambidae|
Lymantria dispar dispar|[1815]|RRMFE944-15|576[0n]bp|Lepidoptera|Erebidae|BOLD:AAA2052
Colias|[1816]|RRINV2239-15|631[0n]bp|Lepidoptera|Pieridae|
Epargyreus clarus|[1817]|RRINV2240-15|509[0n]bp|Lepidoptera|Hesperiidae|
Scrobipalpa acuminatella|[1818]|RRMPB137-15|582[0n]bp|Lepidoptera|Gelechiidae|BOLD:AAC1644
Perittia herrichiella|[1819]|RRINV2040-15|632[0n]bp|Lepidoptera|Elachistidae|
Elophila gyralis|[1820]|RRINV3982-15|614[0n]bp|Lepidoptera|Crambidae|
Elophila icciusalis|[1821]|RRINV3843-15|630[0n]bp|Lepidoptera|Crambidae|
Elophila tinealis|[1822]|RRINV3851-15|630[0n]bp|Lepidoptera|Crambidae|
Acentria ephemerella|[1823]|RRINV1454-15|658[0n]bp|Lepidoptera|Crambidae|
Elachista|[1824]|RRMFC2234-15|588[0n]bp|Lepidoptera|Elachistidae|BOLD:AAD9052
Cosmopterix montisella|[1825]|RRMFG3026-15|576[0n]bp|Lepidoptera|Cosmopterigidae|BOLD:AAH4285
Plutella porrectella|[1826]|RRMFG2884-15|585[0n]bp|Lepidoptera|Plutellidae|BOLD:ACG9804
Drepana arcuata|[1827]|RRINV3944-15|630[0n]bp|Lepidoptera|Drepanidae|
Machimia tentoriferella|[1828]|RRINV1255-15|633[0n]bp|Lepidoptera|Depressariidae|
Desmia maculalis|[1829]|RRMFE2997-15|621[2n]bp|Lepidoptera|Crambidae|BOLD:ACE8375
Herpetogramma|[1830]|RRINV3935-15|630[0n]bp|Lepidoptera|Crambidae|
Herpetogramma|[1831]|RRINV3979-15|622[0n]bp|Lepidoptera|Crambidae|
Symmerista leucitys|[1832]|RRINV1951-15|627[0n]bp|Lepidoptera|Notodontidae|
Deidamia inscriptum|[1833]|RRMFD577-15|564[0n]bp|Lepidoptera|Sphingidae|BOLD:AAB0001
Allagrapha aerea|[1834]|RRINV3908-15|630[0n]bp|Lepidoptera|Noctuidae|
Asaphocrita busckiella|[1835]|RRSSA4203-15|576[0n]bp|Lepidoptera|Blastobasidae|BOLD:AAA8938
Blastobasis glandulella|[1836]|RRSSA3532-15|555[0n]bp|Lepidoptera|Blastobasidae|BOLD:AAB1096
Teladoma helianthi|[1837]|RRMPE395-15|576[0n]bp|Lepidoptera|Cosmopterigidae|BOLD:AAE1519
Dichomeris ligulella|[1838]|RRMFD483-15|573[0n]bp|Lepidoptera|Gelechiidae|BOLD:AAA8109
Helcystogramma hystricella|[1839]|RRMFG2887-15|564[0n]bp|Lepidoptera|Gelechiidae|BOLD:AAE7016
Dichomeris inserrata|[1840]|RRMPG056-15|632[1n]bp|Lepidoptera|Gelechiidae|BOLD:AAH4488
Dichomeris leuconotella|[1841]|RRINV1320-15|606[0n]bp|Lepidoptera|Gelechiidae|
Dichomeris mercatrix|[1842]|RRINV1314-15|633[0n]bp|Lepidoptera|Gelechiidae|
Dichomeris furia|[1843]|RRBAA048-15|570[0n]bp|Lepidoptera|Gelechiidae|BOLD:AAI9560
Cisseps fulvicollis|[1844]|RRINV3927-15|630[0n]bp|Lepidoptera|Erebidae|
Rivula propinqualis|[1845]|RRBFA428-15|579[0n]bp|Lepidoptera|Erebidae|BOLD:AAA4282
Ctenucha virginica|[1846]|RRINV2231-15|631[0n]bp|Lepidoptera|Erebidae|
Protodeltote albidula|[1847]|RRINV2233-15|631[0n]bp|Lepidoptera|Noctuidae|
Cucullia asteroides|[1848]|RRINV1319-15|632[0n]bp|Lepidoptera|Noctuidae|
Cucullia convexipennis|[1849]|RRINV3937-15|630[0n]bp|Lepidoptera|Noctuidae|
Nola ovilla|[1850]|RRSSA2008-15|552[0n]bp|Lepidoptera|Nolidae|BOLD:AAD1810
Depressaria depressana|[1851]|RRINV3987-15|611[2n]bp|Lepidoptera|Depressariidae|
Agonopterix arenella|[1852]|RRMFB1011-15|582[0n]bp|Lepidoptera|Depressariidae|BOLD:AAC6982
Agonopterix|[1853]|RRMPB003-15|570[0n]bp|Lepidoptera|Depressariidae|BOLD:AAC0205
Agonopterix pulvipennella|[1854]|RRMFB1022-15|534[0n]bp|Lepidoptera|Depressariidae|BOLD:AAA7550
Caenurgina crassiuscula|[1855]|RRINV1705-15|638[0n]bp|Lepidoptera|Noctuidae|
Catocala|[1856]|RRINV3930-15|630[0n]bp|Lepidoptera|Noctuidae|
Catocala grynea|[1857]|RRINV3942-15|630[0n]bp|Lepidoptera|Noctuidae|
Catocala cerogama|[1858]|RRMFB952-15|549[0n]bp|Lepidoptera|Noctuidae|BOLD:AAB3383
Hyphantria cunea|[1859]|RRINV1692-15|634[0n]bp|Lepidoptera|Erebidae|
Phragmatobia|[1860]|RRINV3907-15|614[0n]bp|Lepidoptera|Erebidae|
Hypena scabra|[1861]|RRINV1322-15|613[0n]bp|Lepidoptera|Erebidae|
Hypena madefactalis|[1862]|RRINV3926-15|630[0n]bp|Lepidoptera|Erebidae|
Amphipyra pyramidoides|[1863]|RRSSA3875-15|636[0n]bp|Lepidoptera|Noctuidae|BOLD:AAA8525

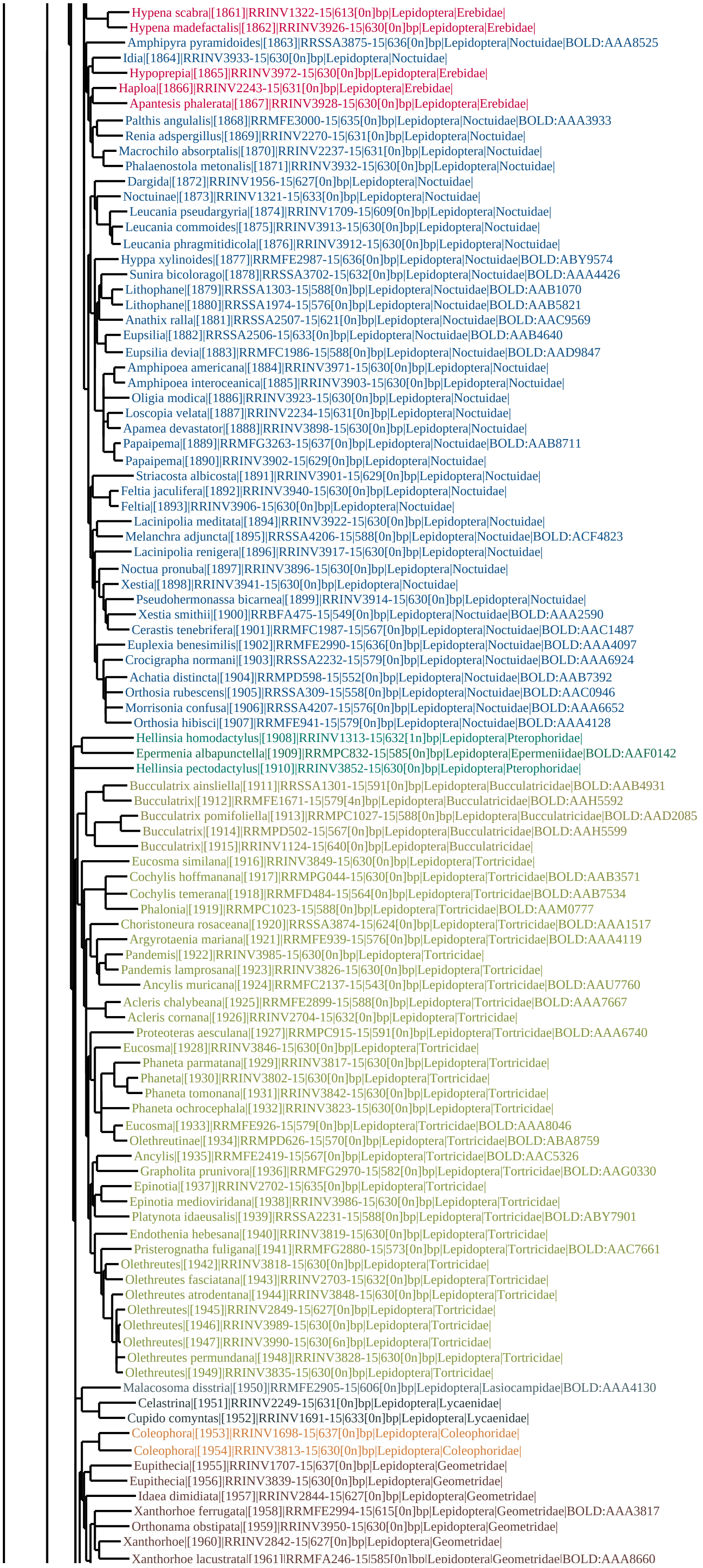
Hypena scabra|[1861]|RRINV1322-15|613[0n]bp|Lepidoptera|Erebidae|
Hypena madefactalis|[1862]|RRINV3926-15|630[0n]bp|Lepidoptera|Erebidae|
Amphipyra pyramidoides|[1863]|RRSSA3875-15|636[0n]bp|Lepidoptera|Noctuidae|BOLD:AAA8525
Idia|[1864]|RRINV3933-15|630[0n]bp|Lepidoptera|Noctuidae|
Hypoprepia|[1865]|RRINV3972-15|630[0n]bp|Lepidoptera|Erebidae|
Haploa|[1866]|RRINV2243-15|631[0n]bp|Lepidoptera|Erebidae|
Apantesis phalerata|[1867]|RRINV3928-15|630[0n]bp|Lepidoptera|Erebidae|
Palthis angulalis|[1868]|RRMFE3000-15|635[0n]bp|Lepidoptera|Noctuidae|BOLD:AAA3933
Renia adspergillus|[1869]|RRINV2270-15|631[0n]bp|Lepidoptera|Noctuidae|
Macrochilo absorptalis|[1870]|RRINV2237-15|631[0n]bp|Lepidoptera|Noctuidae|
Phalaenostola metonalis|[1871]|RRINV3932-15|630[0n]bp|Lepidoptera|Noctuidae|
Dargida|[1872]|RRINV1956-15|627[0n]bp|Lepidoptera|Noctuidae|
Noctuinae|[1873]|RRINV1321-15|633[0n]bp|Lepidoptera|Noctuidae|
Leucania pseudargyria|[1874]|RRINV1709-15|609[0n]bp|Lepidoptera|Noctuidae|
Leucania commoides|[1875]|RRINV3913-15|630[0n]bp|Lepidoptera|Noctuidae|
Leucania phragmitidicola|[1876]|RRINV3912-15|630[0n]bp|Lepidoptera|Noctuidae|
Hyppa xylinoides|[1877]|RRMFE2987-15|636[0n]bp|Lepidoptera|Noctuidae|BOLD:ABY9574
Sunira bicolorago|[1878]|RRSSA3702-15|632[0n]bp|Lepidoptera|Noctuidae|BOLD:AAA4426
Lithophane|[1879]|RRSSA1303-15|588[0n]bp|Lepidoptera|Noctuidae|BOLD:AAB1070
Lithophane|[1880]|RRSSA1974-15|576[0n]bp|Lepidoptera|Noctuidae|BOLD:AAB5821
Anathix ralla|[1881]|RRSSA2507-15|621[0n]bp|Lepidoptera|Noctuidae|BOLD:AAC9569
Eupsilia|[1882]|RRSSA2506-15|633[0n]bp|Lepidoptera|Noctuidae|BOLD:AAB4640
Eupsilia devia|[1883]|RRMFC1986-15|588[0n]bp|Lepidoptera|Noctuidae|BOLD:AAD9847
Amphipoea americana|[1884]|RRINV3971-15|630[0n]bp|Lepidoptera|Noctuidae|
Amphipoea interoceanica|[1885]|RRINV3903-15|630[0n]bp|Lepidoptera|Noctuidae|
Oligia modica|[1886]|RRINV3923-15|630[0n]bp|Lepidoptera|Noctuidae|
Loscopia velata|[1887]|RRINV2234-15|631[0n]bp|Lepidoptera|Noctuidae|
Apamea devastator|[1888]|RRINV3898-15|630[0n]bp|Lepidoptera|Noctuidae|
Papaipema|[1889]|RRMFG3263-15|637[0n]bp|Lepidoptera|Noctuidae|BOLD:AAB8711
Papaipema|[1890]|RRINV3902-15|629[0n]bp|Lepidoptera|Noctuidae|
Striacosta albicosta|[1891]|RRINV3901-15|629[0n]bp|Lepidoptera|Noctuidae|
Feltia jaculifera|[1892]|RRINV3940-15|630[0n]bp|Lepidoptera|Noctuidae|
Feltia|[1893]|RRINV3906-15|630[0n]bp|Lepidoptera|Noctuidae|
Lacinipolia meditata|[1894]|RRINV3922-15|630[0n]bp|Lepidoptera|Noctuidae|
Melanchra adjuncta|[1895]|RRSSA4206-15|588[0n]bp|Lepidoptera|Noctuidae|BOLD:ACF4823
Lacinipolia renigera|[1896]|RRINV3917-15|630[0n]bp|Lepidoptera|Noctuidae|
Noctua pronuba|[1897]|RRINV3896-15|630[0n]bp|Lepidoptera|Noctuidae|
Xestia|[1898]|RRINV3941-15|630[0n]bp|Lepidoptera|Noctuidae|
Pseudohermonassa bicarnea|[1899]|RRINV3914-15|630[0n]bp|Lepidoptera|Noctuidae|
Xestia smithii|[1900]|RRBFA475-15|549[0n]bp|Lepidoptera|Noctuidae|BOLD:AAA2590
Cerastis tenebrifera|[1901]|RRMFC1987-15|567[0n]bp|Lepidoptera|Noctuidae|BOLD:AAC1487
Euplexia benesimilis|[1902]|RRMFE2990-15|636[0n]bp|Lepidoptera|Noctuidae|BOLD:AAA4097
Crocigrapha normani|[1903]|RRSSA2232-15|579[0n]bp|Lepidoptera|Noctuidae|BOLD:AAA6924
Achatia distincta|[1904]|RRMPD598-15|552[0n]bp|Lepidoptera|Noctuidae|BOLD:AAB7392
Orthosia rubescens|[1905]|RRSSA309-15|558[0n]bp|Lepidoptera|Noctuidae|BOLD:AAC0946
Morrisonia confusa|[1906]|RRSSA4207-15|576[0n]bp|Lepidoptera|Noctuidae|BOLD:AAA6652
Orthosia hibisci|[1907]|RRMFE941-15|579[0n]bp|Lepidoptera|Noctuidae|BOLD:AAA4128
Hellinsia homodactylus|[1908]|RRINV1313-15|632[1n]bp|Lepidoptera|Pterophoridae|
Epermenia albapunctella|[1909]|RRMPC832-15|585[0n]bp|Lepidoptera|Epermeniidae|BOLD:AAF0142
Hellinsia pectodactylus|[1910]|RRINV3852-15|630[0n]bp|Lepidoptera|Pterophoridae|
Bucculatrix ainsliella|[1911]|RRSSA1301-15|591[0n]bp|Lepidoptera|Bucculatricidae|BOLD:AAB4931
Bucculatrix|[1912]|RRMFE1671-15|579[4n]bp|Lepidoptera|Bucculatricidae|BOLD:AAH5592
Bucculatrix pomifoliella|[1913]|RRMPC1027-15|588[0n]bp|Lepidoptera|Bucculatricidae|BOLD:AAD2085
Bucculatrix|[1914]|RRMPD502-15|567[0n]bp|Lepidoptera|Bucculatricidae|BOLD:AAH5599
Bucculatrix|[1915]|RRINV1124-15|640[0n]bp|Lepidoptera|Bucculatricidae|
Eucosma similana|[1916]|RRINV3849-15|630[0n]bp|Lepidoptera|Tortricidae|
Cochylis hoffmanana|[1917]|RRMPG044-15|630[0n]bp|Lepidoptera|Tortricidae|BOLD:AAB3571
Cochylis temerana|[1918]|RRMFD484-15|564[0n]bp|Lepidoptera|Tortricidae|BOLD:AAB7534
Phalonia|[1919]|RRMPC1023-15|588[0n]bp|Lepidoptera|Tortricidae|BOLD:AAM0777
Choristoneura rosaceana|[1920]|RRSSA3874-15|624[0n]bp|Lepidoptera|Tortricidae|BOLD:AAA1517
Argyrotaenia mariana|[1921]|RRMFE939-15|576[0n]bp|Lepidoptera|Tortricidae|BOLD:AAA4119
Pandemis|[1922]|RRINV3985-15|630[0n]bp|Lepidoptera|Tortricidae|
Pandemis lamprosana|[1923]|RRINV3826-15|630[0n]bp|Lepidoptera|Tortricidae|
Ancylis muricana|[1924]|RRMFC2137-15|543[0n]bp|Lepidoptera|Tortricidae|BOLD:AAU7760
Acleris chalybeana|[1925]|RRMFE2899-15|588[0n]bp|Lepidoptera|Tortricidae|BOLD:AAA7667
Acleris cornana|[1926]|RRINV2704-15|632[0n]bp|Lepidoptera|Tortricidae|
Proteoteras aesculana|[1927]|RRMPC915-15|591[0n]bp|Lepidoptera|Tortricidae|BOLD:AAA6740
Eucosma|[1928]|RRINV3846-15|630[0n]bp|Lepidoptera|Tortricidae|
Phaneta parmatana|[1929]|RRINV3817-15|630[0n]bp|Lepidoptera|Tortricidae|
Phaneta|[1930]|RRINV3802-15|630[0n]bp|Lepidoptera|Tortricidae|
Phaneta tomonana|[1931]|RRINV3842-15|630[0n]bp|Lepidoptera|Tortricidae|
Phaneta ochrocephala|[1932]|RRINV3823-15|630[0n]bp|Lepidoptera|Tortricidae|
Eucosma|[1933]|RRMFE926-15|579[0n]bp|Lepidoptera|Tortricidae|BOLD:AAA8046
Olethreutinae|[1934]|RRMPD626-15|570[0n]bp|Lepidoptera|Tortricidae|BOLD:ABA8759
Ancylis|[1935]|RRMFE2419-15|567[0n]bp|Lepidoptera|Tortricidae|BOLD:AAC5326
Grapholita prunivora|[1936]|RRMFG2970-15|582[0n]bp|Lepidoptera|Tortricidae|BOLD:AAG0330
Epinotia|[1937]|RRINV2702-15|635[0n]bp|Lepidoptera|Tortricidae|
Epinotia medioviridana|[1938]|RRINV3986-15|630[0n]bp|Lepidoptera|Tortricidae|
Platynota idaeusalis|[1939]|RRSSA2231-15|588[0n]bp|Lepidoptera|Tortricidae|BOLD:ABY7901
Endothenia hebesana|[1940]|RRINV3819-15|630[0n]bp|Lepidoptera|Tortricidae|
Pristerognatha fuligana|[1941]|RRMFG2880-15|573[0n]bp|Lepidoptera|Tortricidae|BOLD:AAC7661
Olethreutes|[1942]|RRINV3818-15|630[0n]bp|Lepidoptera|Tortricidae|
Olethreutes fasciatana|[1943]|RRINV2703-15|632[0n]bp|Lepidoptera|Tortricidae|
Olethreutes atrodentana|[1944]|RRINV3848-15|630[0n]bp|Lepidoptera|Tortricidae|
Olethreutes|[1945]|RRINV2849-15|627[0n]bp|Lepidoptera|Tortricidae|
Olethreutes|[1946]|RRINV3989-15|630[0n]bp|Lepidoptera|Tortricidae|
Olethreutes|[1947]|RRINV3990-15|630[6n]bp|Lepidoptera|Tortricidae|
Olethreutes permundana|[1948]|RRINV3828-15|630[0n]bp|Lepidoptera|Tortricidae|
Olethreutes|[1949]|RRINV3835-15|630[0n]bp|Lepidoptera|Tortricidae|
Malacosoma disstria|[1950]|RRMFE2905-15|606[0n]bp|Lepidoptera|Lasiocampidae|BOLD:AAA4130
Celastrina|[1951]|RRINV2249-15|631[0n]bp|Lepidoptera|Lycaenidae|
Cupido comyntas|[1952]|RRINV1691-15|633[0n]bp|Lepidoptera|Lycaenidae|
Coleophora|[1953]|RRINV1698-15|637[0n]bp|Lepidoptera|Coleophoridae|
Coleophora|[1954]|RRINV3813-15|630[0n]bp|Lepidoptera|Coleophoridae|
Eupithecia|[1955]|RRINV1707-15|637[0n]bp|Lepidoptera|Geometridae|
Eupithecia|[1956]|RRINV3839-15|630[0n]bp|Lepidoptera|Geometridae|
Idaea dimidiata|[1957]|RRINV2844-15|627[0n]bp|Lepidoptera|Geometridae|
Xanthorhoe ferrugata|[1958]|RRMFE2994-15|615[0n]bp|Lepidoptera|Geometridae|BOLD:AAA3817
Orthonama obstipata|[1959]|RRINV3950-15|630[0n]bp|Lepidoptera|Geometridae|
Xanthorhoe|[1960]|RRINV2842-15|627[0n]bp|Lepidoptera|Geometridae|
Xanthorhoe lacustrata|[1961]|RRMFA246-15|585[0n]bp|Lepidoptera|Geometridae|BOLD:AAA8660

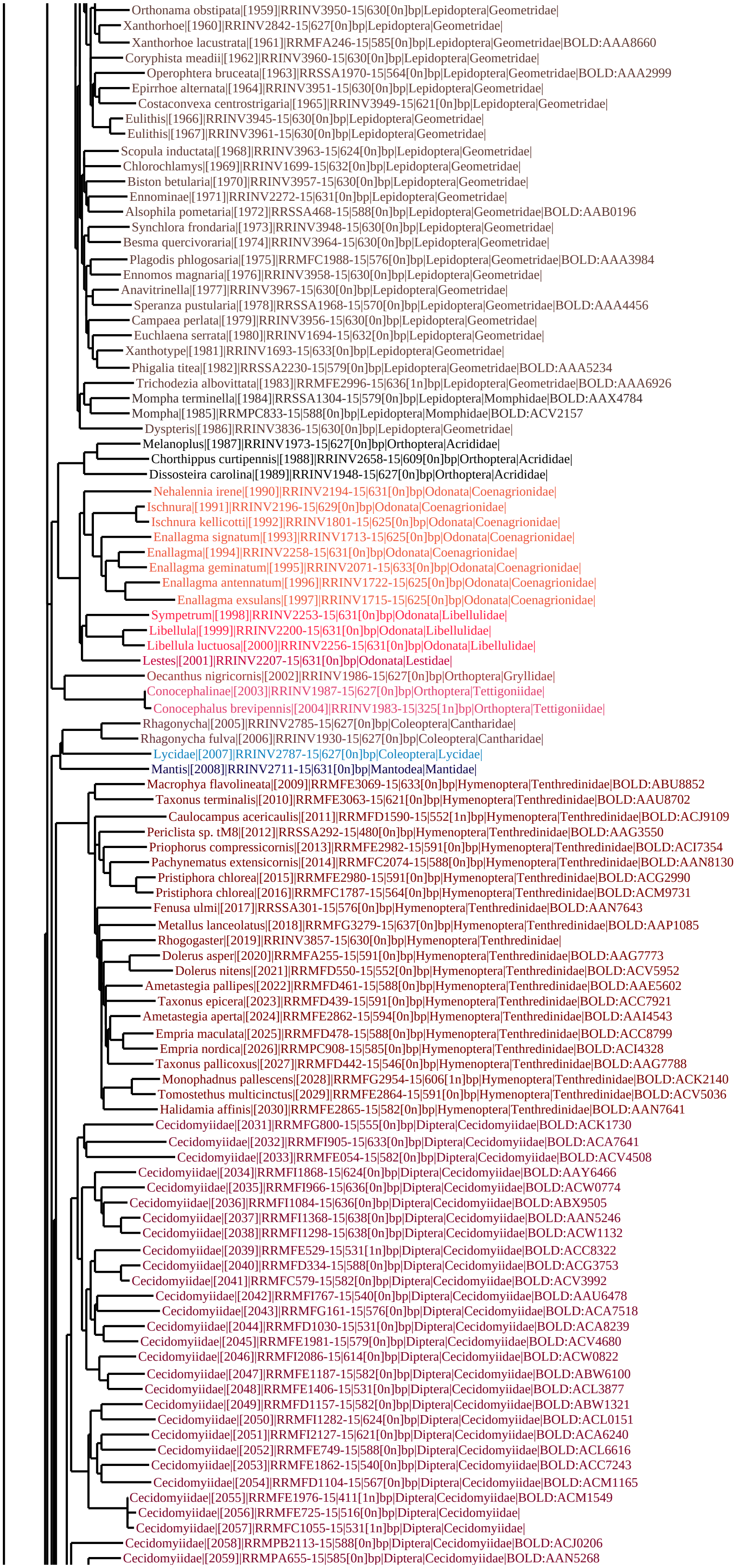
Orthonama obstipata|[1959]|RRINV3950-15|630[0n]bp|Lepidoptera|Geometridae|
Xanthorhoe|[1960]|RRINV2842-15|627[0n]bp|Lepidoptera|Geometridae|
Xanthorhoe lacustrata|[1961]|RRMFA246-15|585[0n]bp|Lepidoptera|Geometridae|BOLD:AAA8660
Coryphista meadii|[1962]|RRINV3960-15|630[0n]bp|Lepidoptera|Geometridae|
Operophtera bruceata|[1963]|RRSSA1970-15|564[0n]bp|Lepidoptera|Geometridae|BOLD:AAA2999
Epirrhoe alternata|[1964]|RRINV3951-15|630[0n]bp|Lepidoptera|Geometridae|
Costaconvexa centrostrigaria|[1965]|RRINV3949-15|621[0n]bp|Lepidoptera|Geometridae|
Eulithis|[1966]|RRINV3945-15|630[0n]bp|Lepidoptera|Geometridae|
Eulithis|[1967]|RRINV3961-15|630[0n]bp|Lepidoptera|Geometridae|
Scopula inductata|[1968]|RRINV3963-15|624[0n]bp|Lepidoptera|Geometridae|
Chlorochlamys|[1969]|RRINV1699-15|632[0n]bp|Lepidoptera|Geometridae|
Biston betularia|[1970]|RRINV3957-15|630[0n]bp|Lepidoptera|Geometridae|
Ennominae|[1971]|RRINV2272-15|631[0n]bp|Lepidoptera|Geometridae|
Alsophila pometaria|[1972]|RRSSA468-15|588[0n]bp|Lepidoptera|Geometridae|BOLD:AAB0196
Synchlora frondaria|[1973]|RRINV3948-15|630[0n]bp|Lepidoptera|Geometridae|
Besma quercivoraria|[1974]|RRINV3964-15|630[0n]bp|Lepidoptera|Geometridae|
Plagodis phlogosaria|[1975]|RRMFC1988-15|576[0n]bp|Lepidoptera|Geometridae|BOLD:AAA3984
Ennomos magnaria|[1976]|RRINV3958-15|630[0n]bp|Lepidoptera|Geometridae|
Anavitrinella|[1977]|RRINV3967-15|630[0n]bp|Lepidoptera|Geometridae|
Speranza pustularia|[1978]|RRSSA1968-15|570[0n]bp|Lepidoptera|Geometridae|BOLD:AAA4456
Campaea perlata|[1979]|RRINV3956-15|630[0n]bp|Lepidoptera|Geometridae|
Euchlaena serrata|[1980]|RRINV1694-15|632[0n]bp|Lepidoptera|Geometridae|
Xanthotype|[1981]|RRINV1693-15|633[0n]bp|Lepidoptera|Geometridae|
Phigalia titea|[1982]|RRSSA2230-15|579[0n]bp|Lepidoptera|Geometridae|BOLD:AAA5234
Trichodezia albovittata|[1983]|RRMFE2996-15|636[1n]bp|Lepidoptera|Geometridae|BOLD:AAA6926
Mompha terminella|[1984]|RRSSA1304-15|579[0n]bp|Lepidoptera|Momphidae|BOLD:AAX4784
Mompha|[1985]|RRMPC833-15|588[0n]bp|Lepidoptera|Momphidae|BOLD:ACV2157
Dyspteris|[1986]|RRINV3836-15|630[0n]bp|Lepidoptera|Geometridae|
Melanoplus|[1987]|RRINV1973-15|627[0n]bp|Orthoptera|Acrididae|
Chorthippus curtipennis|[1988]|RRINV2658-15|609[0n]bp|Orthoptera|Acrididae|
Dissosteira carolina|[1989]|RRINV1948-15|627[0n]bp|Orthoptera|Acrididae|
Nehalennia irene|[1990]|RRINV2194-15|631[0n]bp|Odonata|Coenagrionidae|
Ischnura|[1991]|RRINV2196-15|629[0n]bp|Odonata|Coenagrionidae|
Ischnura kellicotti|[1992]|RRINV1801-15|625[0n]bp|Odonata|Coenagrionidae|
Enallagma signatum|[1993]|RRINV1713-15|625[0n]bp|Odonata|Coenagrionidae|
Enallagma|[1994]|RRINV2258-15|631[0n]bp|Odonata|Coenagrionidae|
Enallagma geminatum|[1995]|RRINV2071-15|633[0n]bp|Odonata|Coenagrionidae|
Enallagma antennatum|[1996]|RRINV1722-15|625[0n]bp|Odonata|Coenagrionidae|
Enallagma exsulans|[1997]|RRINV1715-15|625[0n]bp|Odonata|Coenagrionidae|
Sympetrum|[1998]|RRINV2253-15|631[0n]bp|Odonata|Libellulidae|
Libellula|[1999]|RRINV2200-15|631[0n]bp|Odonata|Libellulidae|
Libellula luctuosa|[2000]|RRINV2256-15|631[0n]bp|Odonata|Libellulidae|
Lestes|[2001]|RRINV2207-15|631[0n]bp|Odonata|Lestidae|
Oecanthus nigricornis|[2002]|RRINV1986-15|627[0n]bp|Orthoptera|Gryllidae|
Conocephalinae|[2003]|RRINV1987-15|627[0n]bp|Orthoptera|Tettigoniidae|
Conocephalus brevipennis|[2004]|RRINV1983-15|325[1n]bp|Orthoptera|Tettigoniidae|
Rhagonycha|[2005]|RRINV2785-15|627[0n]bp|Coleoptera|Cantharidae|
Rhagonycha fulva|[2006]|RRINV1930-15|627[0n]bp|Coleoptera|Cantharidae|
Lycidae|[2007]|RRINV2787-15|627[0n]bp|Coleoptera|Lycidae|
Mantis|[2008]|RRINV2711-15|631[0n]bp|Mantodea|Mantidae|
Macrophya flavolineata|[2009]|RRMFE3069-15|633[0n]bp|Hymenoptera|Tenthredinidae|BOLD:ABU8852
Taxonus terminalis|[2010]|RRMFE3063-15|621[0n]bp|Hymenoptera|Tenthredinidae|BOLD:AAU8702
Caulocampus acericaulis|[2011]|RRMFD1590-15|552[1n]bp|Hymenoptera|Tenthredinidae|BOLD:ACJ9109
Periclista sp. tM8|[2012]|RRSSA292-15|480[0n]bp|Hymenoptera|Tenthredinidae|BOLD:AAG3550
Priophorus compressicornis|[2013]|RRMFE2982-15|591[0n]bp|Hymenoptera|Tenthredinidae|BOLD:ACI7354
Pachynematus extensicornis|[2014]|RRMFC2074-15|588[0n]bp|Hymenoptera|Tenthredinidae|BOLD:AAN8130
Pristiphora chlorea|[2015]|RRMFE2980-15|591[0n]bp|Hymenoptera|Tenthredinidae|BOLD:ACG2990
Pristiphora chlorea|[2016]|RRMFC1787-15|564[0n]bp|Hymenoptera|Tenthredinidae|BOLD:ACM9731
Fenusa ulmi|[2017]|RRSSA301-15|576[0n]bp|Hymenoptera|Tenthredinidae|BOLD:AAN7643
Metallus lanceolatus|[2018]|RRMFG3279-15|637[0n]bp|Hymenoptera|Tenthredinidae|BOLD:AAP1085
Rhogogaster|[2019]|RRINV3857-15|630[0n]bp|Hymenoptera|Tenthredinidae|
Dolerus asper|[2020]|RRMFA255-15|591[0n]bp|Hymenoptera|Tenthredinidae|BOLD:AAG7773
Dolerus nitens|[2021]|RRMFD550-15|552[0n]bp|Hymenoptera|Tenthredinidae|BOLD:ACV5952
Ametastegia pallipes|[2022]|RRMFD461-15|588[0n]bp|Hymenoptera|Tenthredinidae|BOLD:AAE5602
Taxonus epicera|[2023]|RRMFD439-15|591[0n]bp|Hymenoptera|Tenthredinidae|BOLD:ACC7921
Ametastegia aperta|[2024]|RRMFE2862-15|594[0n]bp|Hymenoptera|Tenthredinidae|BOLD:AAI4543
Empria maculata|[2025]|RRMFD478-15|588[0n]bp|Hymenoptera|Tenthredinidae|BOLD:ACC8799
Empria nordica|[2026]|RRMPC908-15|585[0n]bp|Hymenoptera|Tenthredinidae|BOLD:ACI4328
Taxonus pallicoxus|[2027]|RRMFD442-15|546[0n]bp|Hymenoptera|Tenthredinidae|BOLD:AAG7788
Monophadnus pallescens|[2028]|RRMFG2954-15|606[1n]bp|Hymenoptera|Tenthredinidae|BOLD:ACK2140
Tomostethus multicinctus|[2029]|RRMFE2864-15|591[0n]bp|Hymenoptera|Tenthredinidae|BOLD:ACV5036
Halidamia affinis|[2030]|RRMFE2865-15|582[0n]bp|Hymenoptera|Tenthredinidae|BOLD:AAN7641
Cecidomyiidae|[2031]|RRMFG800-15|555[0n]bp|Diptera|Cecidomyiidae|BOLD:ACK1730
Cecidomyiidae|[2032]|RRMFI905-15|633[0n]bp|Diptera|Cecidomyiidae|BOLD:ACA7641
Cecidomyiidae|[2033]|RRMFE054-15|582[0n]bp|Diptera|Cecidomyiidae|BOLD:ACV4508
Cecidomyiidae|[2034]|RRMFI1868-15|624[0n]bp|Diptera|Cecidomyiidae|BOLD:AAY6466
Cecidomyiidae|[2035]|RRMFI966-15|636[0n]bp|Diptera|Cecidomyiidae|BOLD:ACW0774
Cecidomyiidae|[2036]|RRMFI1084-15|636[0n]bp|Diptera|Cecidomyiidae|BOLD:ABX9505
Cecidomyiidae|[2037]|RRMFI1368-15|638[0n]bp|Diptera|Cecidomyiidae|BOLD:AAN5246
Cecidomyiidae|[2038]|RRMFI1298-15|638[0n]bp|Diptera|Cecidomyiidae|BOLD:ACW1132
Cecidomyiidae|[2039]|RRMFE529-15|531[1n]bp|Diptera|Cecidomyiidae|BOLD:ACC8322
Cecidomyiidae|[2040]|RRMFD334-15|588[0n]bp|Diptera|Cecidomyiidae|BOLD:ACG3753
Cecidomyiidae|[2041]|RRMFC579-15|582[0n]bp|Diptera|Cecidomyiidae|BOLD:ACV3992
Cecidomyiidae|[2042]|RRMFI767-15|540[0n]bp|Diptera|Cecidomyiidae|BOLD:AAU6478
Cecidomyiidae|[2043]|RRMFG161-15|576[0n]bp|Diptera|Cecidomyiidae|BOLD:ACA7518
Cecidomyiidae|[2044]|RRMFD1030-15|531[0n]bp|Diptera|Cecidomyiidae|BOLD:ACA8239
Cecidomyiidae|[2045]|RRMFE1981-15|579[0n]bp|Diptera|Cecidomyiidae|BOLD:ACV4680
Cecidomyiidae|[2046]|RRMFI2086-15|614[0n]bp|Diptera|Cecidomyiidae|BOLD:ACW0822
Cecidomyiidae|[2047]|RRMFE1187-15|582[0n]bp|Diptera|Cecidomyiidae|BOLD:ABW6100
Cecidomyiidae|[2048]|RRMFE1406-15|531[0n]bp|Diptera|Cecidomyiidae|BOLD:ACL3877
Cecidomyiidae|[2049]|RRMFD1157-15|582[0n]bp|Diptera|Cecidomyiidae|BOLD:ABW1321
Cecidomyiidae|[2050]|RRMFI1282-15|624[0n]bp|Diptera|Cecidomyiidae|BOLD:ACL0151
Cecidomyiidae|[2051]|RRMFI2127-15|621[0n]bp|Diptera|Cecidomyiidae|BOLD:ACA6240
Cecidomyiidae|[2052]|RRMFE749-15|588[0n]bp|Diptera|Cecidomyiidae|BOLD:ACL6616
Cecidomyiidae|[2053]|RRMFE1862-15|540[0n]bp|Diptera|Cecidomyiidae|BOLD:ACC7243
Cecidomyiidae|[2054]|RRMFD1104-15|567[0n]bp|Diptera|Cecidomyiidae|BOLD:ACM1165
Cecidomyiidae|[2055]|RRMFE1976-15|411[1n]bp|Diptera|Cecidomyiidae|BOLD:ACM1549
Cecidomyiidae|[2056]|RRMFE725-15|516[0n]bp|Diptera|Cecidomyiidae|
Cecidomyiidae|[2057]|RRMFC1055-15|531[1n]bp|Diptera|Cecidomyiidae|
Cecidomyiidae|[2058]|RRMPB2113-15|588[0n]bp|Diptera|Cecidomyiidae|BOLD:ACJ0206
Cecidomyiidae|[2059]|RRMPA655-15|585[0n]bp|Diptera|Cecidomyiidae|BOLD:AAN5268

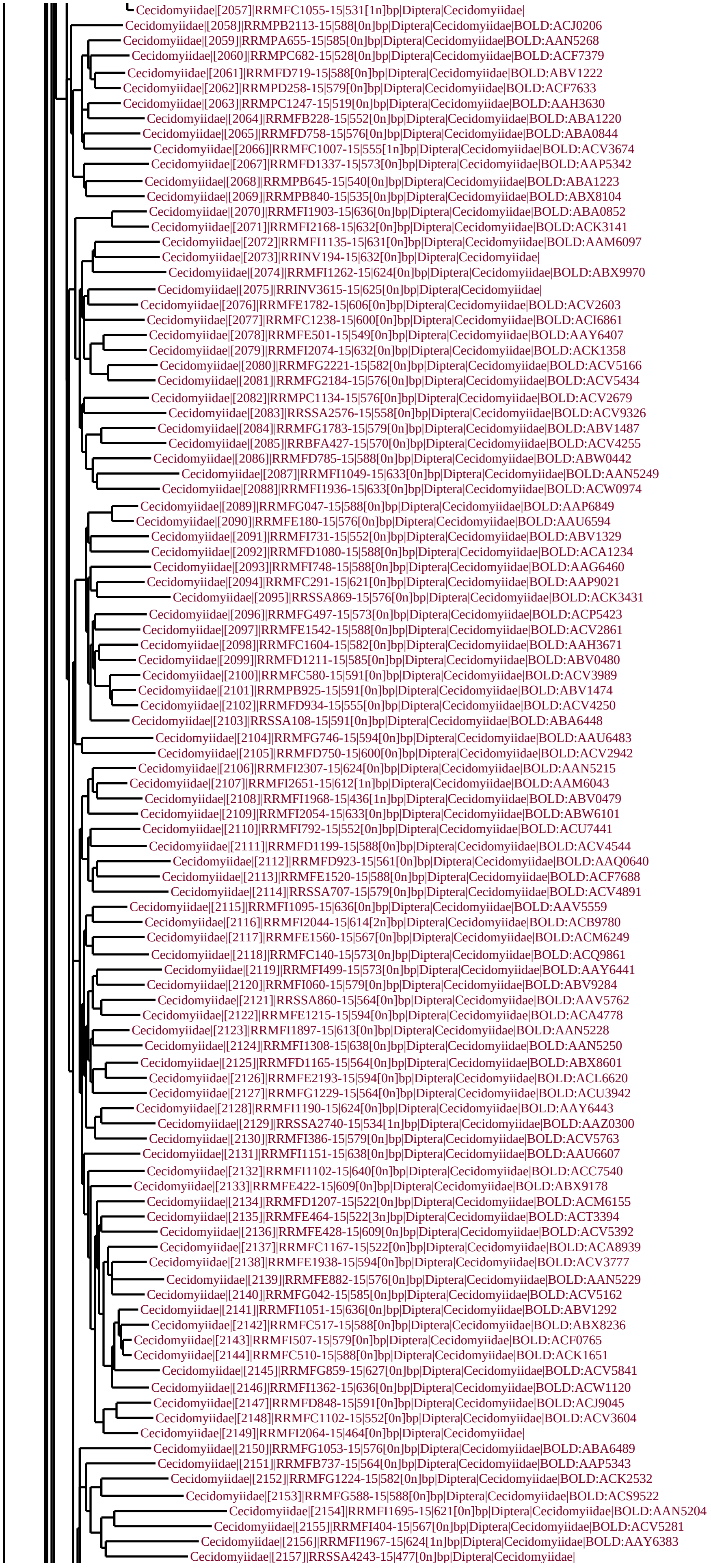
Cecidomyiidae|[2057]|RRMFC1055-15|531[1n]bp|Diptera|Cecidomyiidae|
Cecidomyiidae|[2058]|RRMPB2113-15|588[0n]bp|Diptera|Cecidomyiidae|BOLD:ACJ0206
Cecidomyiidae|[2059]|RRMPA655-15|585[0n]bp|Diptera|Cecidomyiidae|BOLD:AAN5268
Cecidomyiidae|[2060]|RRMPC682-15|528[0n]bp|Diptera|Cecidomyiidae|BOLD:ACF7379
Cecidomyiidae|[2061]|RRMFD719-15|588[0n]bp|Diptera|Cecidomyiidae|BOLD:ABV1222
Cecidomyiidae|[2062]|RRMPD258-15|579[0n]bp|Diptera|Cecidomyiidae|BOLD:ACF7633
Cecidomyiidae|[2063]|RRMPC1247-15|519[0n]bp|Diptera|Cecidomyiidae|BOLD:AAH3630
Cecidomyiidae|[2064]|RRMFB228-15|552[0n]bp|Diptera|Cecidomyiidae|BOLD:ABA1220
Cecidomyiidae|[2065]|RRMFD758-15|576[0n]bp|Diptera|Cecidomyiidae|BOLD:ABA0844
Cecidomyiidae|[2066]|RRMFC1007-15|555[1n]bp|Diptera|Cecidomyiidae|BOLD:ACV3674
Cecidomyiidae|[2067]|RRMFD1337-15|573[0n]bp|Diptera|Cecidomyiidae|BOLD:AAP5342
Cecidomyiidae|[2068]|RRMPB645-15|540[0n]bp|Diptera|Cecidomyiidae|BOLD:ABA1223
Cecidomyiidae|[2069]|RRMPB840-15|535[0n]bp|Diptera|Cecidomyiidae|BOLD:ABX8104
Cecidomyiidae|[2070]|RRMFI1903-15|636[0n]bp|Diptera|Cecidomyiidae|BOLD:ABA0852
Cecidomyiidae|[2071]|RRMFI2168-15|632[0n]bp|Diptera|Cecidomyiidae|BOLD:ACK3141
Cecidomyiidae|[2072]|RRMFI1135-15|631[0n]bp|Diptera|Cecidomyiidae|BOLD:AAM6097
Cecidomyiidae|[2073]|RRINV194-15|632[0n]bp|Diptera|Cecidomyiidae|
Cecidomyiidae|[2074]|RRMFI1262-15|624[0n]bp|Diptera|Cecidomyiidae|BOLD:ABX9970
Cecidomyiidae|[2075]|RRINV3615-15|625[0n]bp|Diptera|Cecidomyiidae|
Cecidomyiidae|[2076]|RRMFE1782-15|606[0n]bp|Diptera|Cecidomyiidae|BOLD:ACV2603
Cecidomyiidae|[2077]|RRMFC1238-15|600[0n]bp|Diptera|Cecidomyiidae|BOLD:ACI6861
Cecidomyiidae|[2078]|RRMFE501-15|549[0n]bp|Diptera|Cecidomyiidae|BOLD:AAY6407
Cecidomyiidae|[2079]|RRMFI2074-15|632[0n]bp|Diptera|Cecidomyiidae|BOLD:ACK1358
Cecidomyiidae|[2080]|RRMFG2221-15|582[0n]bp|Diptera|Cecidomyiidae|BOLD:ACV5166
Cecidomyiidae|[2081]|RRMFG2184-15|576[0n]bp|Diptera|Cecidomyiidae|BOLD:ACV5434
Cecidomyiidae|[2082]|RRMPC1134-15|576[0n]bp|Diptera|Cecidomyiidae|BOLD:ACV2679
Cecidomyiidae|[2083]|RRSSA2576-15|558[0n]bp|Diptera|Cecidomyiidae|BOLD:ACV9326
Cecidomyiidae|[2084]|RRMFG1783-15|579[0n]bp|Diptera|Cecidomyiidae|BOLD:ABV1487
Cecidomyiidae|[2085]|RRBFA427-15|570[0n]bp|Diptera|Cecidomyiidae|BOLD:ACV4255
Cecidomyiidae|[2086]|RRMFD785-15|588[0n]bp|Diptera|Cecidomyiidae|BOLD:ABW0442
Cecidomyiidae|[2087]|RRMFI1049-15|633[0n]bp|Diptera|Cecidomyiidae|BOLD:AAN5249
Cecidomyiidae|[2088]|RRMFI1936-15|633[0n]bp|Diptera|Cecidomyiidae|BOLD:ACW0974
Cecidomyiidae|[2089]|RRMFG047-15|588[0n]bp|Diptera|Cecidomyiidae|BOLD:AAP6849
Cecidomyiidae|[2090]|RRMFE180-15|576[0n]bp|Diptera|Cecidomyiidae|BOLD:AAU6594
Cecidomyiidae|[2091]|RRMFI731-15|552[0n]bp|Diptera|Cecidomyiidae|BOLD:ABV1329
Cecidomyiidae|[2092]|RRMFD1080-15|588[0n]bp|Diptera|Cecidomyiidae|BOLD:ACA1234
Cecidomyiidae|[2093]|RRMFI748-15|588[0n]bp|Diptera|Cecidomyiidae|BOLD:AAG6460
Cecidomyiidae|[2094]|RRMFC291-15|621[0n]bp|Diptera|Cecidomyiidae|BOLD:AAP9021
Cecidomyiidae|[2095]|RRSSA869-15|576[0n]bp|Diptera|Cecidomyiidae|BOLD:ACK3431
Cecidomyiidae|[2096]|RRMFG497-15|573[0n]bp|Diptera|Cecidomyiidae|BOLD:ACP5423
Cecidomyiidae|[2097]|RRMFE1542-15|588[0n]bp|Diptera|Cecidomyiidae|BOLD:ACV2861
Cecidomyiidae|[2098]|RRMFC1604-15|582[0n]bp|Diptera|Cecidomyiidae|BOLD:AAH3671
Cecidomyiidae|[2099]|RRMFD1211-15|585[0n]bp|Diptera|Cecidomyiidae|BOLD:ABV0480
Cecidomyiidae|[2100]|RRMFC580-15|591[0n]bp|Diptera|Cecidomyiidae|BOLD:ACV3989
Cecidomyiidae|[2101]|RRMPB925-15|591[0n]bp|Diptera|Cecidomyiidae|BOLD:ABV1474
Cecidomyiidae|[2102]|RRMFD934-15|555[0n]bp|Diptera|Cecidomyiidae|BOLD:ACV4250
Cecidomyiidae|[2103]|RRSSA108-15|591[0n]bp|Diptera|Cecidomyiidae|BOLD:ABA6448
Cecidomyiidae|[2104]|RRMFG746-15|594[0n]bp|Diptera|Cecidomyiidae|BOLD:AAU6483
Cecidomyiidae|[2105]|RRMFD750-15|600[0n]bp|Diptera|Cecidomyiidae|BOLD:ACV2942
Cecidomyiidae|[2106]|RRMFI2307-15|624[0n]bp|Diptera|Cecidomyiidae|BOLD:AAN5215
Cecidomyiidae|[2107]|RRMFI2651-15|612[1n]bp|Diptera|Cecidomyiidae|BOLD:AAM6043
Cecidomyiidae|[2108]|RRMFI1968-15|436[1n]bp|Diptera|Cecidomyiidae|BOLD:ABV0479
Cecidomyiidae|[2109]|RRMFI2054-15|633[0n]bp|Diptera|Cecidomyiidae|BOLD:ABW6101
Cecidomyiidae|[2110]|RRMFI792-15|552[0n]bp|Diptera|Cecidomyiidae|BOLD:ACU7441
Cecidomyiidae|[2111]|RRMFD1199-15|588[0n]bp|Diptera|Cecidomyiidae|BOLD:ACV4544
Cecidomyiidae|[2112]|RRMFD923-15|561[0n]bp|Diptera|Cecidomyiidae|BOLD:AAQ0640
Cecidomyiidae|[2113]|RRMFE1520-15|588[0n]bp|Diptera|Cecidomyiidae|BOLD:ACF7688
Cecidomyiidae|[2114]|RRSSA707-15|579[0n]bp|Diptera|Cecidomyiidae|BOLD:ACV4891
Cecidomyiidae|[2115]|RRMFI1095-15|636[0n]bp|Diptera|Cecidomyiidae|BOLD:AAV5559
Cecidomyiidae|[2116]|RRMFI2044-15|614[2n]bp|Diptera|Cecidomyiidae|BOLD:ACB9780
Cecidomyiidae|[2117]|RRMFE1560-15|567[0n]bp|Diptera|Cecidomyiidae|BOLD:ACM6249
Cecidomyiidae|[2118]|RRMFC140-15|573[0n]bp|Diptera|Cecidomyiidae|BOLD:ACQ9861
Cecidomyiidae|[2119]|RRMFI499-15|573[0n]bp|Diptera|Cecidomyiidae|BOLD:AAY6441
Cecidomyiidae|[2120]|RRMFI060-15|579[0n]bp|Diptera|Cecidomyiidae|BOLD:ABV9284
Cecidomyiidae|[2121]|RRSSA860-15|564[0n]bp|Diptera|Cecidomyiidae|BOLD:AAV5762
Cecidomyiidae|[2122]|RRMFE1215-15|594[0n]bp|Diptera|Cecidomyiidae|BOLD:ACA4778
Cecidomyiidae|[2123]|RRMFI1897-15|613[0n]bp|Diptera|Cecidomyiidae|BOLD:AAN5228
Cecidomyiidae|[2124]|RRMFI1308-15|638[0n]bp|Diptera|Cecidomyiidae|BOLD:AAN5250
Cecidomyiidae|[2125]|RRMFD1165-15|564[0n]bp|Diptera|Cecidomyiidae|BOLD:ABX8601
Cecidomyiidae|[2126]|RRMFE2193-15|594[0n]bp|Diptera|Cecidomyiidae|BOLD:ACL6620
Cecidomyiidae|[2127]|RRMFG1229-15|564[0n]bp|Diptera|Cecidomyiidae|BOLD:ACU3942
Cecidomyiidae|[2128]|RRMFI1190-15|624[0n]bp|Diptera|Cecidomyiidae|BOLD:AAY6443
Cecidomyiidae|[2129]|RRSSA2740-15|534[1n]bp|Diptera|Cecidomyiidae|BOLD:AAZ0300
Cecidomyiidae|[2130]|RRMFI386-15|579[0n]bp|Diptera|Cecidomyiidae|BOLD:ACV5763
Cecidomyiidae|[2131]|RRMFI1151-15|638[0n]bp|Diptera|Cecidomyiidae|BOLD:AAU6607
Cecidomyiidae|[2132]|RRMFI1102-15|640[0n]bp|Diptera|Cecidomyiidae|BOLD:ACC7540
Cecidomyiidae|[2133]|RRMFE422-15|609[0n]bp|Diptera|Cecidomyiidae|BOLD:ABX9178
Cecidomyiidae|[2134]|RRMFD1207-15|522[0n]bp|Diptera|Cecidomyiidae|BOLD:ACM6155
Cecidomyiidae|[2135]|RRMFE464-15|522[3n]bp|Diptera|Cecidomyiidae|BOLD:ACT3394
Cecidomyiidae|[2136]|RRMFE428-15|609[0n]bp|Diptera|Cecidomyiidae|BOLD:ACV5392
Cecidomyiidae|[2137]|RRMFC1167-15|522[0n]bp|Diptera|Cecidomyiidae|BOLD:ACA8939
Cecidomyiidae|[2138]|RRMFE1938-15|594[0n]bp|Diptera|Cecidomyiidae|BOLD:ACV3777
Cecidomyiidae|[2139]|RRMFE882-15|576[0n]bp|Diptera|Cecidomyiidae|BOLD:AAN5229
Cecidomyiidae|[2140]|RRMFG042-15|585[0n]bp|Diptera|Cecidomyiidae|BOLD:ACV5162
Cecidomyiidae|[2141]|RRMFI1051-15|636[0n]bp|Diptera|Cecidomyiidae|BOLD:ABV1292
Cecidomyiidae|[2142]|RRMFC517-15|588[0n]bp|Diptera|Cecidomyiidae|BOLD:ABX8236
Cecidomyiidae|[2143]|RRMFI507-15|579[0n]bp|Diptera|Cecidomyiidae|BOLD:ACF0765
Cecidomyiidae|[2144]|RRMFC510-15|588[0n]bp|Diptera|Cecidomyiidae|BOLD:ACK1651
Cecidomyiidae|[2145]|RRMFG859-15|627[0n]bp|Diptera|Cecidomyiidae|BOLD:ACV5841
Cecidomyiidae|[2146]|RRMFI1362-15|636[0n]bp|Diptera|Cecidomyiidae|BOLD:ACW1120
Cecidomyiidae|[2147]|RRMFD848-15|591[0n]bp|Diptera|Cecidomyiidae|BOLD:ACJ9045
Cecidomyiidae|[2148]|RRMFC1102-15|552[0n]bp|Diptera|Cecidomyiidae|BOLD:ACV3604
Cecidomyiidae|[2149]|RRMFI2064-15|464[0n]bp|Diptera|Cecidomyiidae|
Cecidomyiidae|[2150]|RRMFG1053-15|576[0n]bp|Diptera|Cecidomyiidae|BOLD:ABA6489
Cecidomyiidae|[2151]|RRMFB737-15|564[0n]bp|Diptera|Cecidomyiidae|BOLD:AAP5343
Cecidomyiidae|[2152]|RRMFG1224-15|582[0n]bp|Diptera|Cecidomyiidae|BOLD:ACK2532
Cecidomyiidae|[2153]|RRMFG588-15|588[0n]bp|Diptera|Cecidomyiidae|BOLD:ACS9522
Cecidomyiidae|[2154]|RRMFI1695-15|621[0n]bp|Diptera|Cecidomyiidae|BOLD:AAN5204
Cecidomyiidae|[2155]|RRMFI404-15|567[0n]bp|Diptera|Cecidomyiidae|BOLD:ACV5281
Cecidomyiidae|[2156]|RRMFI1967-15|624[1n]bp|Diptera|Cecidomyiidae|BOLD:AAY6383
Cecidomyiidae|[2157]|RRSSA4243-15|477[0n]bp|Diptera|Cecidomyiidae|

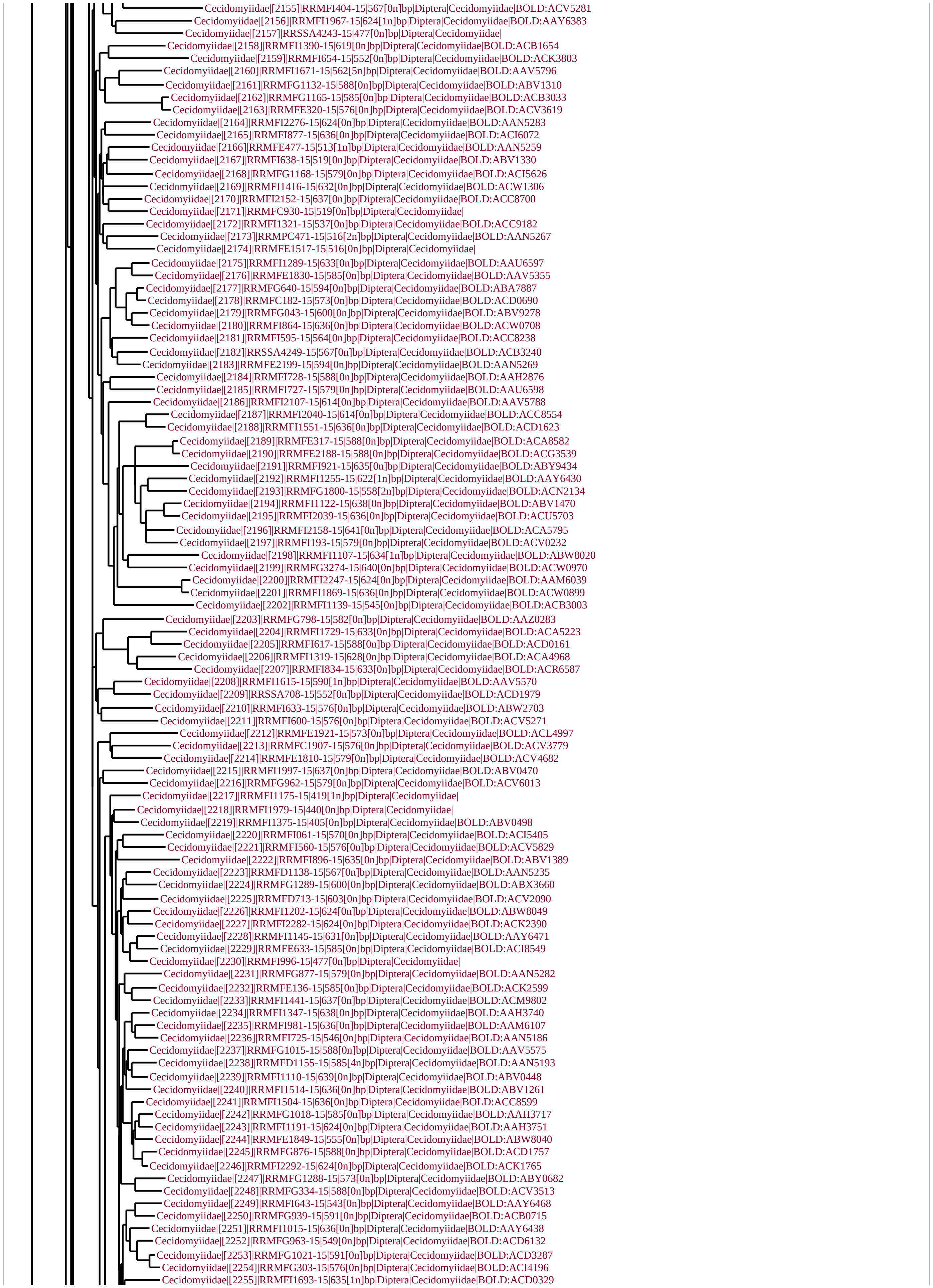
Cecidomyiidae|[2155]|RRMFI404-15|567[0n]bp|Diptera|Cecidomyiidae|BOLD:ACV5281
Cecidomyiidae|[2156]|RRMFI1967-15|624[1n]bp|Diptera|Cecidomyiidae|BOLD:AAY6383
Cecidomyiidae|[2157]|RRSSA4243-15|477[0n]bp|Diptera|Cecidomyiidae|
Cecidomyiidae|[2158]|RRMFI1390-15|619[0n]bp|Diptera|Cecidomyiidae|BOLD:ACB1654
Cecidomyiidae|[2159]|RRMFI654-15|552[0n]bp|Diptera|Cecidomyiidae|BOLD:ACK3803
Cecidomyiidae|[2160]|RRMFI1671-15|562[5n]bp|Diptera|Cecidomyiidae|BOLD:AAV5796
Cecidomyiidae|[2161]|RRMFG1132-15|588[0n]bp|Diptera|Cecidomyiidae|BOLD:ABV1310
Cecidomyiidae|[2162]|RRMFG1165-15|585[0n]bp|Diptera|Cecidomyiidae|BOLD:ACB3033
Cecidomyiidae|[2163]|RRMFE320-15|576[0n]bp|Diptera|Cecidomyiidae|BOLD:ACV3619
Cecidomyiidae|[2164]|RRMFI2276-15|624[0n]bp|Diptera|Cecidomyiidae|BOLD:AAN5283
Cecidomyiidae|[2165]|RRMFI877-15|636[0n]bp|Diptera|Cecidomyiidae|BOLD:ACI6072
Cecidomyiidae|[2166]|RRMFE477-15|513[1n]bp|Diptera|Cecidomyiidae|BOLD:AAN5259
Cecidomyiidae|[2167]|RRMFI638-15|519[0n]bp|Diptera|Cecidomyiidae|BOLD:ABV1330
Cecidomyiidae|[2168]|RRMFG1168-15|579[0n]bp|Diptera|Cecidomyiidae|BOLD:ACI5626
Cecidomyiidae|[2169]|RRMFI1416-15|632[0n]bp|Diptera|Cecidomyiidae|BOLD:ACW1306
Cecidomyiidae|[2170]|RRMFI2152-15|637[0n]bp|Diptera|Cecidomyiidae|BOLD:ACC8700
Cecidomyiidae|[2171]|RRMFC930-15|519[0n]bp|Diptera|Cecidomyiidae|
Cecidomyiidae|[2172]|RRMFI1321-15|537[0n]bp|Diptera|Cecidomyiidae|BOLD:ACC9182
Cecidomyiidae|[2173]|RRMPC471-15|516[2n]bp|Diptera|Cecidomyiidae|BOLD:AAN5267
Cecidomyiidae|[2174]|RRMFE1517-15|516[0n]bp|Diptera|Cecidomyiidae|
Cecidomyiidae|[2175]|RRMFI1289-15|633[0n]bp|Diptera|Cecidomyiidae|BOLD:AAU6597
Cecidomyiidae|[2176]|RRMFE1830-15|585[0n]bp|Diptera|Cecidomyiidae|BOLD:AAV5355
Cecidomyiidae|[2177]|RRMFG640-15|594[0n]bp|Diptera|Cecidomyiidae|BOLD:ABA7887
Cecidomyiidae|[2178]|RRMFC182-15|573[0n]bp|Diptera|Cecidomyiidae|BOLD:ACD0690
Cecidomyiidae|[2179]|RRMFG043-15|600[0n]bp|Diptera|Cecidomyiidae|BOLD:ABV9278
Cecidomyiidae|[2180]|RRMFI864-15|636[0n]bp|Diptera|Cecidomyiidae|BOLD:ACW0708
Cecidomyiidae|[2181]|RRMFI595-15|564[0n]bp|Diptera|Cecidomyiidae|BOLD:ACC8238
Cecidomyiidae|[2182]|RRSSA4249-15|567[0n]bp|Diptera|Cecidomyiidae|BOLD:ACB3240
Cecidomyiidae|[2183]|RRMFE2199-15|594[0n]bp|Diptera|Cecidomyiidae|BOLD:AAN5269
Cecidomyiidae|[2184]|RRMFI728-15|588[0n]bp|Diptera|Cecidomyiidae|BOLD:AAH2876
Cecidomyiidae|[2185]|RRMFI727-15|579[0n]bp|Diptera|Cecidomyiidae|BOLD:AAU6598
Cecidomyiidae|[2186]|RRMFI2107-15|614[0n]bp|Diptera|Cecidomyiidae|BOLD:AAV5788
Cecidomyiidae|[2187]|RRMFI2040-15|614[0n]bp|Diptera|Cecidomyiidae|BOLD:ACC8554
Cecidomyiidae|[2188]|RRMFI1551-15|636[0n]bp|Diptera|Cecidomyiidae|BOLD:ACD1623
Cecidomyiidae|[2189]|RRMFE317-15|588[0n]bp|Diptera|Cecidomyiidae|BOLD:ACA8582
Cecidomyiidae|[2190]|RRMFE2188-15|588[0n]bp|Diptera|Cecidomyiidae|BOLD:ACG3539
Cecidomyiidae|[2191]|RRMFI921-15|635[0n]bp|Diptera|Cecidomyiidae|BOLD:ABY9434
Cecidomyiidae|[2192]|RRMFI1255-15|622[1n]bp|Diptera|Cecidomyiidae|BOLD:AAY6430
Cecidomyiidae|[2193]|RRMFG1800-15|558[2n]bp|Diptera|Cecidomyiidae|BOLD:ACN2134
Cecidomyiidae|[2194]|RRMFI1122-15|638[0n]bp|Diptera|Cecidomyiidae|BOLD:ABV1470
Cecidomyiidae|[2195]|RRMFI2039-15|636[0n]bp|Diptera|Cecidomyiidae|BOLD:ACU5703
Cecidomyiidae|[2196]|RRMFI2158-15|641[0n]bp|Diptera|Cecidomyiidae|BOLD:ACA5795
Cecidomyiidae|[2197]|RRMFI193-15|579[0n]bp|Diptera|Cecidomyiidae|BOLD:ACV0232
Cecidomyiidae|[2198]|RRMFI1107-15|634[1n]bp|Diptera|Cecidomyiidae|BOLD:ABW8020
Cecidomyiidae|[2199]|RRMFG3274-15|640[0n]bp|Diptera|Cecidomyiidae|BOLD:ACW0970
Cecidomyiidae|[2200]|RRMFI2247-15|624[0n]bp|Diptera|Cecidomyiidae|BOLD:AAM6039
Cecidomyiidae|[2201]|RRMFI1869-15|636[0n]bp|Diptera|Cecidomyiidae|BOLD:ACW0899
Cecidomyiidae|[2202]|RRMFI1139-15|545[0n]bp|Diptera|Cecidomyiidae|BOLD:ACB3003
Cecidomyiidae|[2203]|RRMFG798-15|582[0n]bp|Diptera|Cecidomyiidae|BOLD:AAZ0283
Cecidomyiidae|[2204]|RRMFI1729-15|633[0n]bp|Diptera|Cecidomyiidae|BOLD:ACA5223
Cecidomyiidae|[2205]|RRMFI617-15|588[0n]bp|Diptera|Cecidomyiidae|BOLD:ACD0161
Cecidomyiidae|[2206]|RRMFI1319-15|628[0n]bp|Diptera|Cecidomyiidae|BOLD:ACA4968
Cecidomyiidae|[2207]|RRMFI834-15|633[0n]bp|Diptera|Cecidomyiidae|BOLD:ACR6587
Cecidomyiidae|[2208]|RRMFI1615-15|590[1n]bp|Diptera|Cecidomyiidae|BOLD:AAV5570
Cecidomyiidae|[2209]|RRSSA708-15|552[0n]bp|Diptera|Cecidomyiidae|BOLD:ACD1979
Cecidomyiidae|[2210]|RRMFI633-15|576[0n]bp|Diptera|Cecidomyiidae|BOLD:ABW2703
Cecidomyiidae|[2211]|RRMFI600-15|576[0n]bp|Diptera|Cecidomyiidae|BOLD:ACV5271
Cecidomyiidae|[2212]|RRMFE1921-15|573[0n]bp|Diptera|Cecidomyiidae|BOLD:ACL4997
Cecidomyiidae|[2213]|RRMFC1907-15|576[0n]bp|Diptera|Cecidomyiidae|BOLD:ACV3779
Cecidomyiidae|[2214]|RRMFE1810-15|579[0n]bp|Diptera|Cecidomyiidae|BOLD:ACV4682
Cecidomyiidae|[2215]|RRMFI1997-15|637[0n]bp|Diptera|Cecidomyiidae|BOLD:ABV0470
Cecidomyiidae|[2216]|RRMFG962-15|579[0n]bp|Diptera|Cecidomyiidae|BOLD:ACV6013
Cecidomyiidae|[2217]|RRMFI1175-15|419[1n]bp|Diptera|Cecidomyiidae|
Cecidomyiidae|[2218]|RRMFI1979-15|440[0n]bp|Diptera|Cecidomyiidae|
Cecidomyiidae|[2219]|RRMFI1375-15|405[0n]bp|Diptera|Cecidomyiidae|BOLD:ABV0498
Cecidomyiidae|[2220]|RRMFI061-15|570[0n]bp|Diptera|Cecidomyiidae|BOLD:ACI5405
Cecidomyiidae|[2221]|RRMFI560-15|576[0n]bp|Diptera|Cecidomyiidae|BOLD:ACV5829
Cecidomyiidae|[2222]|RRMFI896-15|635[0n]bp|Diptera|Cecidomyiidae|BOLD:ABV1389
Cecidomyiidae|[2223]|RRMFD1138-15|567[0n]bp|Diptera|Cecidomyiidae|BOLD:AAN5235
Cecidomyiidae|[2224]|RRMFG1289-15|600[0n]bp|Diptera|Cecidomyiidae|BOLD:ABX3660
Cecidomyiidae|[2225]|RRMFD713-15|603[0n]bp|Diptera|Cecidomyiidae|BOLD:ACV2090
Cecidomyiidae|[2226]|RRMFI1202-15|624[0n]bp|Diptera|Cecidomyiidae|BOLD:ABW8049
Cecidomyiidae|[2227]|RRMFI2282-15|624[0n]bp|Diptera|Cecidomyiidae|BOLD:ACK2390
Cecidomyiidae|[2228]|RRMFI1145-15|631[0n]bp|Diptera|Cecidomyiidae|BOLD:AAY6471
Cecidomyiidae|[2229]|RRMFE633-15|585[0n]bp|Diptera|Cecidomyiidae|BOLD:ACI8549
Cecidomyiidae|[2230]|RRMFI996-15|477[0n]bp|Diptera|Cecidomyiidae|
Cecidomyiidae|[2231]|RRMFG877-15|579[0n]bp|Diptera|Cecidomyiidae|BOLD:AAN5282
Cecidomyiidae|[2232]|RRMFE136-15|585[0n]bp|Diptera|Cecidomyiidae|BOLD:ACK2599
Cecidomyiidae|[2233]|RRMFI1441-15|637[0n]bp|Diptera|Cecidomyiidae|BOLD:ACM9802
Cecidomyiidae|[2234]|RRMFI1347-15|638[0n]bp|Diptera|Cecidomyiidae|BOLD:AAH3740
Cecidomyiidae|[2235]|RRMFI981-15|636[0n]bp|Diptera|Cecidomyiidae|BOLD:AAM6107
Cecidomyiidae|[2236]|RRMFI725-15|546[0n]bp|Diptera|Cecidomyiidae|BOLD:AAN5186
Cecidomyiidae|[2237]|RRMFG1015-15|588[0n]bp|Diptera|Cecidomyiidae|BOLD:AAV5575
Cecidomyiidae|[2238]|RRMFD1155-15|585[4n]bp|Diptera|Cecidomyiidae|BOLD:AAN5193
Cecidomyiidae|[2239]|RRMFI1110-15|639[0n]bp|Diptera|Cecidomyiidae|BOLD:ABV0448
Cecidomyiidae|[2240]|RRMFI1514-15|636[0n]bp|Diptera|Cecidomyiidae|BOLD:ABV1261
Cecidomyiidae|[2241]|RRMFI1504-15|636[0n]bp|Diptera|Cecidomyiidae|BOLD:ACC8599
Cecidomyiidae|[2242]|RRMFG1018-15|585[0n]bp|Diptera|Cecidomyiidae|BOLD:AAH3717
Cecidomyiidae|[2243]|RRMFI1191-15|624[0n]bp|Diptera|Cecidomyiidae|BOLD:AAH3751
Cecidomyiidae|[2244]|RRMFE1849-15|555[0n]bp|Diptera|Cecidomyiidae|BOLD:ABW8040
Cecidomyiidae|[2245]|RRMFG876-15|588[0n]bp|Diptera|Cecidomyiidae|BOLD:ACD1757
Cecidomyiidae|[2246]|RRMFI2292-15|624[0n]bp|Diptera|Cecidomyiidae|BOLD:ACK1765
Cecidomyiidae|[2247]|RRMFG1288-15|573[0n]bp|Diptera|Cecidomyiidae|BOLD:ABY0682
Cecidomyiidae|[2248]|RRMFG334-15|588[0n]bp|Diptera|Cecidomyiidae|BOLD:ACV3513
Cecidomyiidae|[2249]|RRMFI643-15|543[0n]bp|Diptera|Cecidomyiidae|BOLD:AAY6468
Cecidomyiidae|[2250]|RRMFG939-15|591[0n]bp|Diptera|Cecidomyiidae|BOLD:ACB0715
Cecidomyiidae|[2251]|RRMFI1015-15|636[0n]bp|Diptera|Cecidomyiidae|BOLD:AAY6438
Cecidomyiidae|[2252]|RRMFG963-15|549[0n]bp|Diptera|Cecidomyiidae|BOLD:ACD6132
Cecidomyiidae|[2253]|RRMFG1021-15|591[0n]bp|Diptera|Cecidomyiidae|BOLD:ACD3287
Cecidomyiidae|[2254]|RRMFG303-15|576[0n]bp|Diptera|Cecidomyiidae|BOLD:ACI4196
Cecidomyiidae|[2255]|RRMFI1693-15|635[1n]bp|Diptera|Cecidomyiidae|BOLD:ACD0329

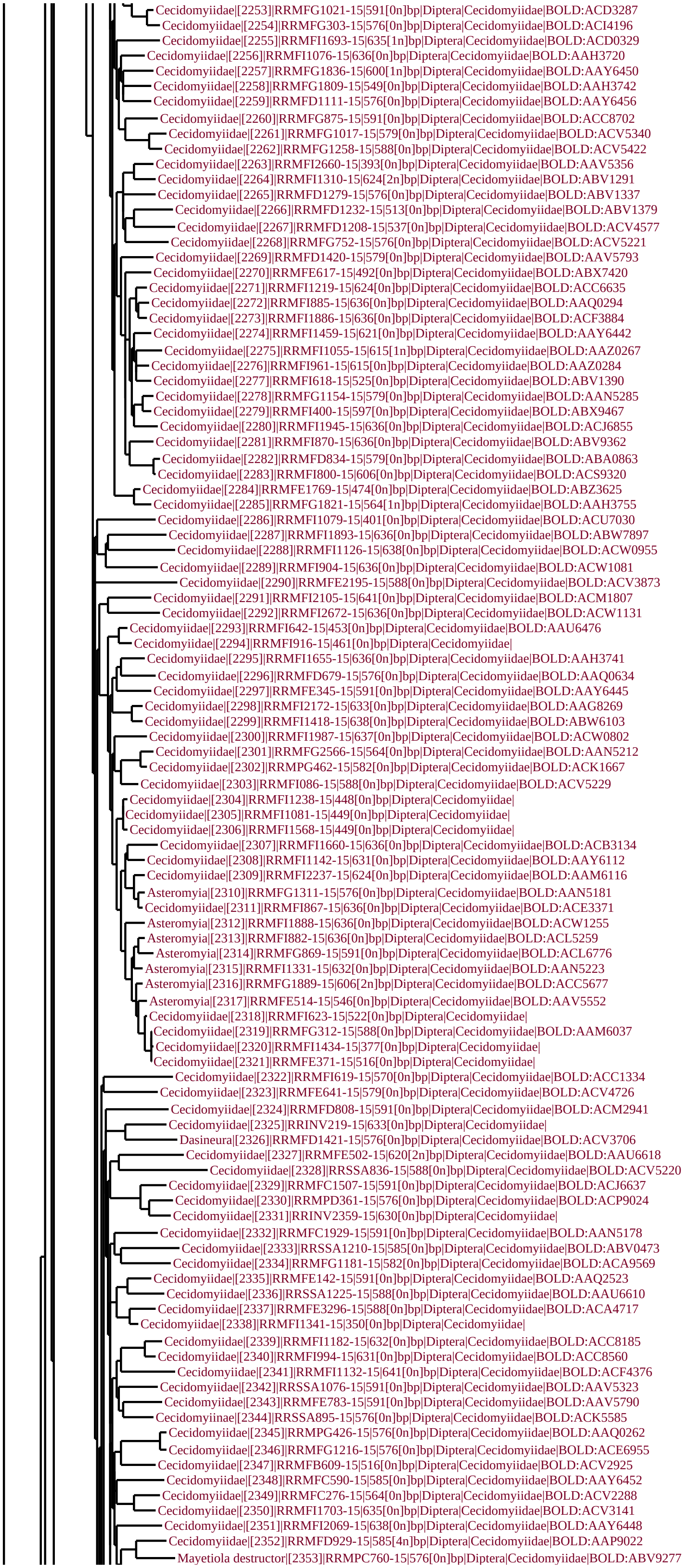

Cecidomyiidae|[2253]|RRMFG1021-15|591[0n]bp|Diptera|Cecidomyiidae|BOLD:ACD3287
Cecidomyiidae|[2254]|RRMFG303-15|576[0n]bp|Diptera|Cecidomyiidae|BOLD:ACI4196
Cecidomyiidae|[2255]|RRMFI1693-15|635[1n]bp|Diptera|Cecidomyiidae|BOLD:ACD0329
Cecidomyiidae|[2256]|RRMFI1076-15|636[0n]bp|Diptera|Cecidomyiidae|BOLD:AAH3720
Cecidomyiidae|[2257]|RRMFG1836-15|600[1n]bp|Diptera|Cecidomyiidae|BOLD:AAY6450
Cecidomyiidae|[2258]|RRMFG1809-15|549[0n]bp|Diptera|Cecidomyiidae|BOLD:AAH3742
Cecidomyiidae|[2259]|RRMFD1111-15|576[0n]bp|Diptera|Cecidomyiidae|BOLD:AAY6456
Cecidomyiidae|[2260]|RRMFG875-15|591[0n]bp|Diptera|Cecidomyiidae|BOLD:ACC8702
Cecidomyiidae|[2261]|RRMFG1017-15|579[0n]bp|Diptera|Cecidomyiidae|BOLD:ACV5340
Cecidomyiidae|[2262]|RRMFG1258-15|588[0n]bp|Diptera|Cecidomyiidae|BOLD:ACV5422
Cecidomyiidae|[2263]|RRMFI2660-15|393[0n]bp|Diptera|Cecidomyiidae|BOLD:AAV5356
Cecidomyiidae|[2264]|RRMFI1310-15|624[2n]bp|Diptera|Cecidomyiidae|BOLD:ABV1291
Cecidomyiidae|[2265]|RRMFD1279-15|576[0n]bp|Diptera|Cecidomyiidae|BOLD:ABV1337
Cecidomyiidae|[2266]|RRMFD1232-15|513[0n]bp|Diptera|Cecidomyiidae|BOLD:ABV1379
Cecidomyiidae|[2267]|RRMFD1208-15|537[0n]bp|Diptera|Cecidomyiidae|BOLD:ACV4577
Cecidomyiidae|[2268]|RRMFG752-15|576[0n]bp|Diptera|Cecidomyiidae|BOLD:ACV5221
Cecidomyiidae|[2269]|RRMFD1420-15|579[0n]bp|Diptera|Cecidomyiidae|BOLD:AAV5793
Cecidomyiidae|[2270]|RRMFE617-15|492[0n]bp|Diptera|Cecidomyiidae|BOLD:ABX7420
Cecidomyiidae|[2271]|RRMFI1219-15|624[0n]bp|Diptera|Cecidomyiidae|BOLD:ACC6635
Cecidomyiidae|[2272]|RRMFI885-15|636[0n]bp|Diptera|Cecidomyiidae|BOLD:AAQ0294
Cecidomyiidae|[2273]|RRMFI1886-15|636[0n]bp|Diptera|Cecidomyiidae|BOLD:ACF3884
Cecidomyiidae|[2274]|RRMFI1459-15|621[0n]bp|Diptera|Cecidomyiidae|BOLD:AAY6442
Cecidomyiidae|[2275]|RRMFI1055-15|615[1n]bp|Diptera|Cecidomyiidae|BOLD:AAZ0267
Cecidomyiidae|[2276]|RRMFI961-15|615[0n]bp|Diptera|Cecidomyiidae|BOLD:AAZ0284
Cecidomyiidae|[2277]|RRMFI618-15|525[0n]bp|Diptera|Cecidomyiidae|BOLD:ABV1390
Cecidomyiidae|[2278]|RRMFG1154-15|579[0n]bp|Diptera|Cecidomyiidae|BOLD:AAN5285
Cecidomyiidae|[2279]|RRMFI400-15|597[0n]bp|Diptera|Cecidomyiidae|BOLD:ABX9467
Cecidomyiidae|[2280]|RRMFI1945-15|636[0n]bp|Diptera|Cecidomyiidae|BOLD:ACJ6855
Cecidomyiidae|[2281]|RRMFI870-15|636[0n]bp|Diptera|Cecidomyiidae|BOLD:ABV9362
Cecidomyiidae|[2282]|RRMFD834-15|579[0n]bp|Diptera|Cecidomyiidae|BOLD:ABA0863
Cecidomyiidae|[2283]|RRMFI800-15|606[0n]bp|Diptera|Cecidomyiidae|BOLD:ACS9320
Cecidomyiidae|[2284]|RRMFE1769-15|474[0n]bp|Diptera|Cecidomyiidae|BOLD:ABZ3625
Cecidomyiidae|[2285]|RRMFG1821-15|564[1n]bp|Diptera|Cecidomyiidae|BOLD:AAH3755
Cecidomyiidae|[2286]|RRMFI1079-15|401[0n]bp|Diptera|Cecidomyiidae|BOLD:ACU7030
Cecidomyiidae|[2287]|RRMFI1893-15|636[0n]bp|Diptera|Cecidomyiidae|BOLD:ABW7897
Cecidomyiidae|[2288]|RRMFI1126-15|638[0n]bp|Diptera|Cecidomyiidae|BOLD:ACW0955
Cecidomyiidae|[2289]|RRMFI904-15|636[0n]bp|Diptera|Cecidomyiidae|BOLD:ACW1081
Cecidomyiidae|[2290]|RRMFE2195-15|588[0n]bp|Diptera|Cecidomyiidae|BOLD:ACV3873
Cecidomyiidae|[2291]|RRMFI2105-15|641[0n]bp|Diptera|Cecidomyiidae|BOLD:ACM1807
Cecidomyiidae|[2292]|RRMFI2672-15|636[0n]bp|Diptera|Cecidomyiidae|BOLD:ACW1131
Cecidomyiidae|[2293]|RRMFI642-15|453[0n]bp|Diptera|Cecidomyiidae|BOLD:AAU6476
Cecidomyiidae|[2294]|RRMFI916-15|461[0n]bp|Diptera|Cecidomyiidae|
Cecidomyiidae|[2295]|RRMFI1655-15|636[0n]bp|Diptera|Cecidomyiidae|BOLD:AAH3741
Cecidomyiidae|[2296]|RRMFD679-15|576[0n]bp|Diptera|Cecidomyiidae|BOLD:AAQ0634
Cecidomyiidae|[2297]|RRMFE345-15|591[0n]bp|Diptera|Cecidomyiidae|BOLD:AAY6445
Cecidomyiidae|[2298]|RRMFI2172-15|633[0n]bp|Diptera|Cecidomyiidae|BOLD:AAG8269
Cecidomyiidae|[2299]|RRMFI1418-15|638[0n]bp|Diptera|Cecidomyiidae|BOLD:ABW6103
Cecidomyiidae|[2300]|RRMFI1987-15|637[0n]bp|Diptera|Cecidomyiidae|BOLD:ACW0802
Cecidomyiidae|[2301]|RRMFG2566-15|564[0n]bp|Diptera|Cecidomyiidae|BOLD:AAN5212
Cecidomyiidae|[2302]|RRMPG462-15|582[0n]bp|Diptera|Cecidomyiidae|BOLD:ACK1667
Cecidomyiidae|[2303]|RRMFI086-15|588[0n]bp|Diptera|Cecidomyiidae|BOLD:ACV5229
Cecidomyiidae|[2304]|RRMFI1238-15|448[0n]bp|Diptera|Cecidomyiidae|
Cecidomyiidae|[2305]|RRMFI1081-15|449[0n]bp|Diptera|Cecidomyiidae|
Cecidomyiidae|[2306]|RRMFI1568-15|449[0n]bp|Diptera|Cecidomyiidae|
Cecidomyiidae|[2307]|RRMFI1660-15|636[0n]bp|Diptera|Cecidomyiidae|BOLD:ACB3134
Cecidomyiidae|[2308]|RRMFI1142-15|631[0n]bp|Diptera|Cecidomyiidae|BOLD:AAY6112
Cecidomyiidae|[2309]|RRMFI2237-15|624[0n]bp|Diptera|Cecidomyiidae|BOLD:AAM6116
Asteromyia|[2310]|RRMFG1311-15|576[0n]bp|Diptera|Cecidomyiidae|BOLD:AAN5181
Cecidomyiidae|[2311]|RRMFI867-15|636[0n]bp|Diptera|Cecidomyiidae|BOLD:ACE3371
Asteromyia|[2312]|RRMFI1888-15|636[0n]bp|Diptera|Cecidomyiidae|BOLD:ACW1255
Asteromyia|[2313]|RRMFI882-15|636[0n]bp|Diptera|Cecidomyiidae|BOLD:ACL5259
Asteromyia|[2314]|RRMFG869-15|591[0n]bp|Diptera|Cecidomyiidae|BOLD:ACL6776
Asteromyia|[2315]|RRMFI1331-15|632[0n]bp|Diptera|Cecidomyiidae|BOLD:AAN5223
Asteromyia|[2316]|RRMFG1889-15|606[2n]bp|Diptera|Cecidomyiidae|BOLD:ACC5677
Asteromyia|[2317]|RRMFE514-15|546[0n]bp|Diptera|Cecidomyiidae|BOLD:AAV5552
Cecidomyiidae|[2318]|RRMFI623-15|522[0n]bp|Diptera|Cecidomyiidae|
Cecidomyiidae|[2319]|RRMFG312-15|588[0n]bp|Diptera|Cecidomyiidae|BOLD:AAM6037
Cecidomyiidae|[2320]|RRMFI1434-15|377[0n]bp|Diptera|Cecidomyiidae|
Cecidomyiidae|[2321]|RRMFE371-15|516[0n]bp|Diptera|Cecidomyiidae|
Cecidomyiidae|[2322]|RRMFI619-15|570[0n]bp|Diptera|Cecidomyiidae|BOLD:ACC1334
Cecidomyiidae|[2323]|RRMFE641-15|579[0n]bp|Diptera|Cecidomyiidae|BOLD:ACV4726
Cecidomyiidae|[2324]|RRMFD808-15|591[0n]bp|Diptera|Cecidomyiidae|BOLD:ACM2941
Cecidomyiidae|[2325]|RRINV219-15|633[0n]bp|Diptera|Cecidomyiidae|
Dasineura|[2326]|RRMFD1421-15|576[0n]bp|Diptera|Cecidomyiidae|BOLD:ACV3706
Cecidomyiidae|[2327]|RRMFE502-15|620[2n]bp|Diptera|Cecidomyiidae|BOLD:AAU6618
Cecidomyiidae|[2328]|RRSSA836-15|588[0n]bp|Diptera|Cecidomyiidae|BOLD:ACV5220
Cecidomyiidae|[2329]|RRMFC1507-15|591[0n]bp|Diptera|Cecidomyiidae|BOLD:ACJ6637
Cecidomyiidae|[2330]|RRMPD361-15|576[0n]bp|Diptera|Cecidomyiidae|BOLD:ACP9024
Cecidomyiidae|[2331]|RRINV2359-15|630[0n]bp|Diptera|Cecidomyiidae|
Cecidomyiidae|[2332]|RRMFC1929-15|591[0n]bp|Diptera|Cecidomyiidae|BOLD:AAN5178
Cecidomyiidae|[2333]|RRSSA1210-15|585[0n]bp|Diptera|Cecidomyiidae|BOLD:ABV0473
Cecidomyiidae|[2334]|RRMFG1181-15|582[0n]bp|Diptera|Cecidomyiidae|BOLD:ACA9569
Cecidomyiidae|[2335]|RRMFE142-15|591[0n]bp|Diptera|Cecidomyiidae|BOLD:AAQ2523
Cecidomyiidae|[2336]|RRSSA1225-15|588[0n]bp|Diptera|Cecidomyiidae|BOLD:AAU6610
Cecidomyiidae|[2337]|RRMFE3296-15|588[0n]bp|Diptera|Cecidomyiidae|BOLD:ACA4717
Cecidomyiidae|[2338]|RRMFI1341-15|350[0n]bp|Diptera|Cecidomyiidae|
Cecidomyiidae|[2339]|RRMFI1182-15|632[0n]bp|Diptera|Cecidomyiidae|BOLD:ACC8185
Cecidomyiidae|[2340]|RRMFI994-15|631[0n]bp|Diptera|Cecidomyiidae|BOLD:ACC8560
Cecidomyiidae|[2341]|RRMFI1132-15|641[0n]bp|Diptera|Cecidomyiidae|BOLD:ACF4376
Cecidomyiidae|[2342]|RRSSA1076-15|591[0n]bp|Diptera|Cecidomyiidae|BOLD:AAV5323
Cecidomyiidae|[2343]|RRMFE783-15|591[0n]bp|Diptera|Cecidomyiidae|BOLD:AAV5790
Cecidomyiinae|[2344]|RRSSA895-15|576[0n]bp|Diptera|Cecidomyiidae|BOLD:ACK5585
Cecidomyiidae|[2345]|RRMPG426-15|576[0n]bp|Diptera|Cecidomyiidae|BOLD:AAQ0262
Cecidomyiidae|[2346]|RRMFG1216-15|576[0n]bp|Diptera|Cecidomyiidae|BOLD:ACE6955
Cecidomyiidae|[2347]|RRMFB609-15|516[0n]bp|Diptera|Cecidomyiidae|BOLD:ACV2925
Cecidomyiidae|[2348]|RRMFC590-15|585[0n]bp|Diptera|Cecidomyiidae|BOLD:AAY6452
Cecidomyiidae|[2349]|RRMFC276-15|564[0n]bp|Diptera|Cecidomyiidae|BOLD:ACV2288
Cecidomyiidae|[2350]|RRMFI1703-15|635[0n]bp|Diptera|Cecidomyiidae|BOLD:ACV3141
Cecidomyiidae|[2351]|RRMFI2069-15|638[0n]bp|Diptera|Cecidomyiidae|BOLD:AAY6448
Cecidomyiidae|[2352]|RRMFD929-15|585[4n]bp|Diptera|Cecidomyiidae|BOLD:AAP9022
Mayetiola destructor|[2353]|RRMPC760-15|576[0n]bp|Diptera|Cecidomyiidae|BOLD:ABV9277

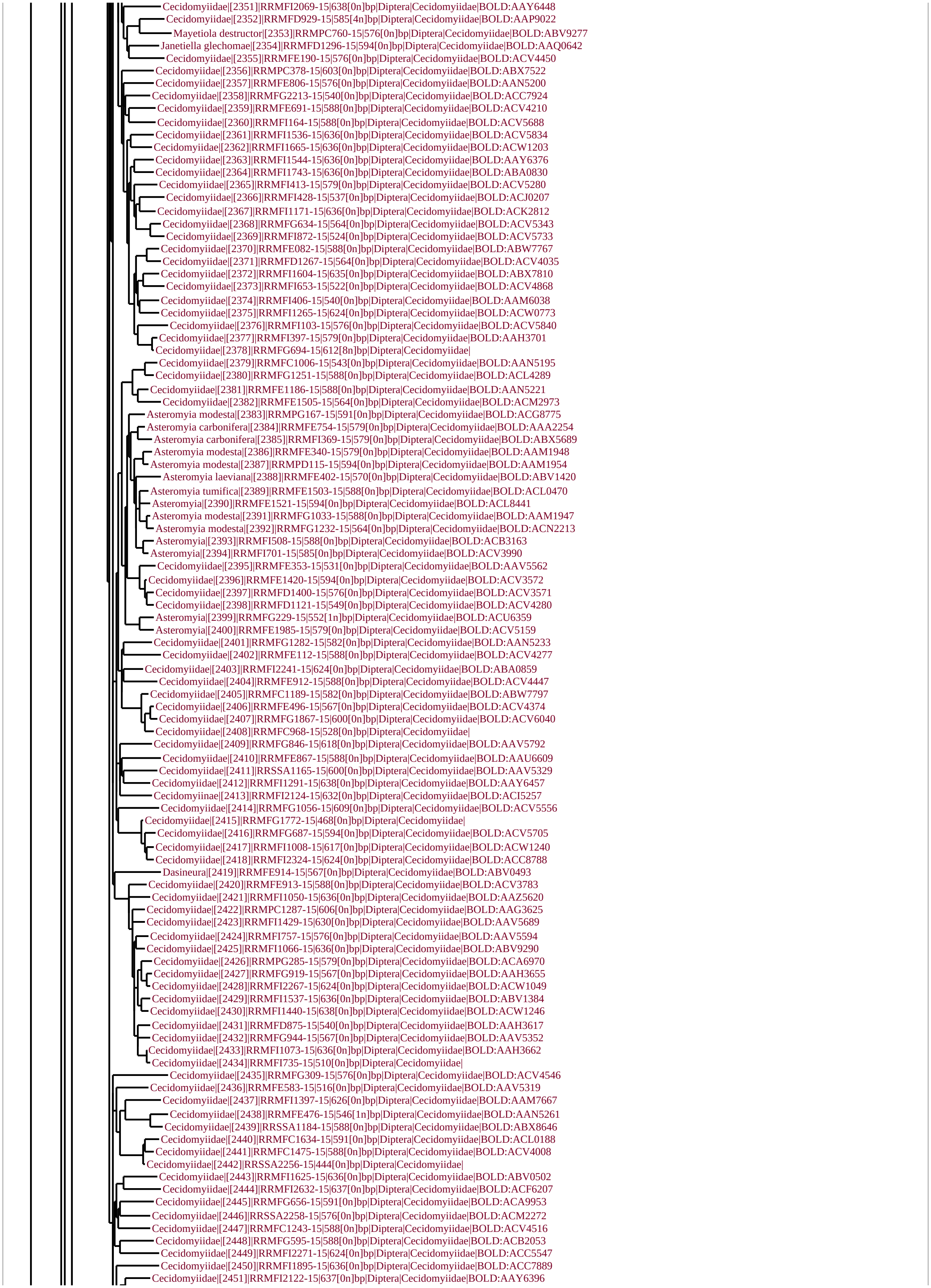
Cecidomyiidae|[2351]|RRMFI2069-15|638[0n]bp|Diptera|Cecidomyiidae|BOLD:AAY6448
Cecidomyiidae|[2352]|RRMFD929-15|585[4n]bp|Diptera|Cecidomyiidae|BOLD:AAP9022
Mayetiola destructor|[2353]|RRMPC760-15|576[0n]bp|Diptera|Cecidomyiidae|BOLD:ABV9277
Janetiella glechomae|[2354]|RRMFD1296-15|594[0n]bp|Diptera|Cecidomyiidae|BOLD:AAQ0642
Cecidomyiidae|[2355]|RRMFE190-15|576[0n]bp|Diptera|Cecidomyiidae|BOLD:ACV4450
Cecidomyiidae|[2356]|RRMPC378-15|603[0n]bp|Diptera|Cecidomyiidae|BOLD:ABX7522
Cecidomyiidae|[2357]|RRMFE806-15|576[0n]bp|Diptera|Cecidomyiidae|BOLD:AAN5200
Cecidomyiidae|[2358]|RRMFG2213-15|540[0n]bp|Diptera|Cecidomyiidae|BOLD:ACC7924
Cecidomyiidae|[2359]|RRMFE691-15|588[0n]bp|Diptera|Cecidomyiidae|BOLD:ACV4210
Cecidomyiidae|[2360]|RRMFI164-15|588[0n]bp|Diptera|Cecidomyiidae|BOLD:ACV5688
Cecidomyiidae|[2361]|RRMFI1536-15|636[0n]bp|Diptera|Cecidomyiidae|BOLD:ACV5834
Cecidomyiidae|[2362]|RRMFI1665-15|636[0n]bp|Diptera|Cecidomyiidae|BOLD:ACW1203
Cecidomyiidae|[2363]|RRMFI1544-15|636[0n]bp|Diptera|Cecidomyiidae|BOLD:AAY6376
Cecidomyiidae|[2364]|RRMFI1743-15|636[0n]bp|Diptera|Cecidomyiidae|BOLD:ABA0830
Cecidomyiidae|[2365]|RRMFI413-15|579[0n]bp|Diptera|Cecidomyiidae|BOLD:ACV5280
Cecidomyiidae|[2366]|RRMFI428-15|537[0n]bp|Diptera|Cecidomyiidae|BOLD:ACJ0207
Cecidomyiidae|[2367]|RRMFI1171-15|636[0n]bp|Diptera|Cecidomyiidae|BOLD:ACK2812
Cecidomyiidae|[2368]|RRMFG634-15|564[0n]bp|Diptera|Cecidomyiidae|BOLD:ACV5343
Cecidomyiidae|[2369]|RRMFI872-15|524[0n]bp|Diptera|Cecidomyiidae|BOLD:ACV5733
Cecidomyiidae|[2370]|RRMFE082-15|588[0n]bp|Diptera|Cecidomyiidae|BOLD:ABW7767
Cecidomyiidae|[2371]|RRMFD1267-15|564[0n]bp|Diptera|Cecidomyiidae|BOLD:ACV4035
Cecidomyiidae|[2372]|RRMFI1604-15|635[0n]bp|Diptera|Cecidomyiidae|BOLD:ABX7810
Cecidomyiidae|[2373]|RRMFI653-15|522[0n]bp|Diptera|Cecidomyiidae|BOLD:ACV4868
Cecidomyiidae|[2374]|RRMFI406-15|540[0n]bp|Diptera|Cecidomyiidae|BOLD:AAM6038
Cecidomyiidae|[2375]|RRMFI1265-15|624[0n]bp|Diptera|Cecidomyiidae|BOLD:ACW0773
Cecidomyiidae|[2376]|RRMFI103-15|576[0n]bp|Diptera|Cecidomyiidae|BOLD:ACV5840
Cecidomyiidae|[2377]|RRMFI397-15|579[0n]bp|Diptera|Cecidomyiidae|BOLD:AAH3701
Cecidomyiidae|[2378]|RRMFG694-15|612[8n]bp|Diptera|Cecidomyiidae|
Cecidomyiidae|[2379]|RRMFC1006-15|543[0n]bp|Diptera|Cecidomyiidae|BOLD:AAN5195
Cecidomyiidae|[2380]|RRMFG1251-15|588[0n]bp|Diptera|Cecidomyiidae|BOLD:ACL4289
Cecidomyiidae|[2381]|RRMFE1186-15|588[0n]bp|Diptera|Cecidomyiidae|BOLD:AAN5221
Cecidomyiidae|[2382]|RRMFE1505-15|564[0n]bp|Diptera|Cecidomyiidae|BOLD:ACM2973
Asteromyia modesta|[2383]|RRMPG167-15|591[0n]bp|Diptera|Cecidomyiidae|BOLD:ACG8775
Asteromyia carbonifera|[2384]|RRMFE754-15|579[0n]bp|Diptera|Cecidomyiidae|BOLD:AAA2254
Asteromyia carbonifera|[2385]|RRMFI369-15|579[0n]bp|Diptera|Cecidomyiidae|BOLD:ABX5689
Asteromyia modesta|[2386]|RRMFE340-15|579[0n]bp|Diptera|Cecidomyiidae|BOLD:AAM1948
Asteromyia modesta|[2387]|RRMPD115-15|594[0n]bp|Diptera|Cecidomyiidae|BOLD:AAM1954
Asteromyia laeviana|[2388]|RRMFE402-15|570[0n]bp|Diptera|Cecidomyiidae|BOLD:ABV1420
Asteromyia tumifica|[2389]|RRMFE1503-15|588[0n]bp|Diptera|Cecidomyiidae|BOLD:ACL0470
Asteromyia|[2390]|RRMFE1521-15|594[0n]bp|Diptera|Cecidomyiidae|BOLD:ACL8441
Asteromyia modesta|[2391]|RRMFG1033-15|588[0n]bp|Diptera|Cecidomyiidae|BOLD:AAM1947
Asteromyia modesta|[2392]|RRMFG1232-15|564[0n]bp|Diptera|Cecidomyiidae|BOLD:ACN2213
Asteromyia|[2393]|RRMFI508-15|588[0n]bp|Diptera|Cecidomyiidae|BOLD:ACB3163
Asteromyia|[2394]|RRMFI701-15|585[0n]bp|Diptera|Cecidomyiidae|BOLD:ACV3990
Cecidomyiidae|[2395]|RRMFE353-15|531[0n]bp|Diptera|Cecidomyiidae|BOLD:AAV5562
Cecidomyiidae|[2396]|RRMFE1420-15|594[0n]bp|Diptera|Cecidomyiidae|BOLD:ACV3572
Cecidomyiidae|[2397]|RRMFD1400-15|576[0n]bp|Diptera|Cecidomyiidae|BOLD:ACV3571
Cecidomyiidae|[2398]|RRMFD1121-15|549[0n]bp|Diptera|Cecidomyiidae|BOLD:ACV4280
Asteromyia|[2399]|RRMFG229-15|552[1n]bp|Diptera|Cecidomyiidae|BOLD:ACU6359
Asteromyia|[2400]|RRMFE1985-15|579[0n]bp|Diptera|Cecidomyiidae|BOLD:ACV5159
Cecidomyiidae|[2401]|RRMFG1282-15|582[0n]bp|Diptera|Cecidomyiidae|BOLD:AAN5233
Cecidomyiidae|[2402]|RRMFE112-15|588[0n]bp|Diptera|Cecidomyiidae|BOLD:ACV4277
Cecidomyiidae|[2403]|RRMFI2241-15|624[0n]bp|Diptera|Cecidomyiidae|BOLD:ABA0859
Cecidomyiidae|[2404]|RRMFE912-15|588[0n]bp|Diptera|Cecidomyiidae|BOLD:ACV4447
Cecidomyiidae|[2405]|RRMFC1189-15|582[0n]bp|Diptera|Cecidomyiidae|BOLD:ABW7797
Cecidomyiidae|[2406]|RRMFE496-15|567[0n]bp|Diptera|Cecidomyiidae|BOLD:ACV4374
Cecidomyiidae|[2407]|RRMFG1867-15|600[0n]bp|Diptera|Cecidomyiidae|BOLD:ACV6040
Cecidomyiidae|[2408]|RRMFC968-15|528[0n]bp|Diptera|Cecidomyiidae|
Cecidomyiidae|[2409]|RRMFG846-15|618[0n]bp|Diptera|Cecidomyiidae|BOLD:AAV5792
Cecidomyiidae|[2410]|RRMFE867-15|588[0n]bp|Diptera|Cecidomyiidae|BOLD:AAU6609
Cecidomyiidae|[2411]|RRSSA1165-15|600[0n]bp|Diptera|Cecidomyiidae|BOLD:AAV5329
Cecidomyiidae|[2412]|RRMFI1291-15|638[0n]bp|Diptera|Cecidomyiidae|BOLD:AAY6457
Cecidomyiinae|[2413]|RRMFI2124-15|632[0n]bp|Diptera|Cecidomyiidae|BOLD:ACI5257
Cecidomyiidae|[2414]|RRMFG1056-15|609[0n]bp|Diptera|Cecidomyiidae|BOLD:ACV5556
Cecidomyiidae|[2415]|RRMFG1772-15|468[0n]bp|Diptera|Cecidomyiidae|
Cecidomyiidae|[2416]|RRMFG687-15|594[0n]bp|Diptera|Cecidomyiidae|BOLD:ACV5705
Cecidomyiidae|[2417]|RRMFI1008-15|617[0n]bp|Diptera|Cecidomyiidae|BOLD:ACW1240
Cecidomyiidae|[2418]|RRMFI2324-15|624[0n]bp|Diptera|Cecidomyiidae|BOLD:ACC8788
Dasineura|[2419]|RRMFE914-15|567[0n]bp|Diptera|Cecidomyiidae|BOLD:ABV0493
Cecidomyiidae|[2420]|RRMFE913-15|588[0n]bp|Diptera|Cecidomyiidae|BOLD:ACV3783
Cecidomyiidae|[2421]|RRMFI1050-15|636[0n]bp|Diptera|Cecidomyiidae|BOLD:AAZ5620
Cecidomyiidae|[2422]|RRMPC1287-15|606[0n]bp|Diptera|Cecidomyiidae|BOLD:AAG3625
Cecidomyiidae|[2423]|RRMFI1429-15|630[0n]bp|Diptera|Cecidomyiidae|BOLD:AAV5689
Cecidomyiidae|[2424]|RRMFI757-15|576[0n]bp|Diptera|Cecidomyiidae|BOLD:AAV5594
Cecidomyiidae|[2425]|RRMFI1066-15|636[0n]bp|Diptera|Cecidomyiidae|BOLD:ABV9290
Cecidomyiidae|[2426]|RRMPG285-15|579[0n]bp|Diptera|Cecidomyiidae|BOLD:ACA6970
Cecidomyiidae|[2427]|RRMFG919-15|567[0n]bp|Diptera|Cecidomyiidae|BOLD:AAH3655
Cecidomyiidae|[2428]|RRMFI2267-15|624[0n]bp|Diptera|Cecidomyiidae|BOLD:ACW1049
Cecidomyiidae|[2429]|RRMFI1537-15|636[0n]bp|Diptera|Cecidomyiidae|BOLD:ABV1384
Cecidomyiidae|[2430]|RRMFI1440-15|638[0n]bp|Diptera|Cecidomyiidae|BOLD:ACW1246
Cecidomyiidae|[2431]|RRMFD875-15|540[0n]bp|Diptera|Cecidomyiidae|BOLD:AAH3617
Cecidomyiidae|[2432]|RRMFG944-15|567[0n]bp|Diptera|Cecidomyiidae|BOLD:AAV5352
Cecidomyiidae|[2433]|RRMFI1073-15|636[0n]bp|Diptera|Cecidomyiidae|BOLD:AAH3662
Cecidomyiidae|[2434]|RRMFI735-15|510[0n]bp|Diptera|Cecidomyiidae|
Cecidomyiidae|[2435]|RRMFG309-15|576[0n]bp|Diptera|Cecidomyiidae|BOLD:ACV4546
Cecidomyiidae|[2436]|RRMFE583-15|516[0n]bp|Diptera|Cecidomyiidae|BOLD:AAV5319
Cecidomyiidae|[2437]|RRMFI1397-15|626[0n]bp|Diptera|Cecidomyiidae|BOLD:AAM7667
Cecidomyiidae|[2438]|RRMFE476-15|546[1n]bp|Diptera|Cecidomyiidae|BOLD:AAN5261
Cecidomyiidae|[2439]|RRSSA1184-15|588[0n]bp|Diptera|Cecidomyiidae|BOLD:ABX8646
Cecidomyiidae|[2440]|RRMFC1634-15|591[0n]bp|Diptera|Cecidomyiidae|BOLD:ACL0188
Cecidomyiidae|[2441]|RRMFC1475-15|588[0n]bp|Diptera|Cecidomyiidae|BOLD:ACV4008
Cecidomyiidae|[2442]|RRSSA2256-15|444[0n]bp|Diptera|Cecidomyiidae|
Cecidomyiidae|[2443]|RRMFI1625-15|636[0n]bp|Diptera|Cecidomyiidae|BOLD:ABV0502
Cecidomyiidae|[2444]|RRMFI2632-15|637[0n]bp|Diptera|Cecidomyiidae|BOLD:ACF6207
Cecidomyiidae|[2445]|RRMFG656-15|591[0n]bp|Diptera|Cecidomyiidae|BOLD:ACA9953
Cecidomyiidae|[2446]|RRSSA2258-15|576[0n]bp|Diptera|Cecidomyiidae|BOLD:ACM2272
Cecidomyiidae|[2447]|RRMFC1243-15|588[0n]bp|Diptera|Cecidomyiidae|BOLD:ACV4516
Cecidomyiidae|[2448]|RRMFG595-15|588[0n]bp|Diptera|Cecidomyiidae|BOLD:ACB2053
Cecidomyiidae|[2449]|RRMFI2271-15|624[0n]bp|Diptera|Cecidomyiidae|BOLD:ACC5547
Cecidomyiidae|[2450]|RRMFI1895-15|636[0n]bp|Diptera|Cecidomyiidae|BOLD:ACC7889
Cecidomyiidae|[2451]|RRMFI2122-15|637[0n]bp|Diptera|Cecidomyiidae|BOLD:AAY6396

Cecidomyiidae|[2449]|RRMFI2271-15|624[0n]bp|Diptera|Cecidomyiidae|BOLD:ACC5547
Cecidomyiidae|[2450]|RRMFI1895-15|636[0n]bp|Diptera|Cecidomyiidae|BOLD:ACC7889
Cecidomyiidae|[2451]|RRMFI2122-15|637[0n]bp|Diptera|Cecidomyiidae|BOLD:AAY6396
Cecidomyiidae|[2452]|RRMFG1270-15|588[0n]bp|Diptera|Cecidomyiidae|BOLD:ACL8670
Cecidomyiidae|[2453]|RRMFI1306-15|636[0n]bp|Diptera|Cecidomyiidae|BOLD:ACW0868
Cecidomyiidae|[2454]|RRMFG698-15|558[0n]bp|Diptera|Cecidomyiidae|BOLD:ABV1369
Cecidomyiidae|[2455]|RRMPG519-15|591[0n]bp|Diptera|Cecidomyiidae|BOLD:AAN5225
Cecidomyiidae|[2456]|RRMFI2200-15|637[0n]bp|Diptera|Cecidomyiidae|BOLD:ACA6930
Cecidomyiidae|[2457]|RRMFI1675-15|636[0n]bp|Diptera|Cecidomyiidae|BOLD:ACF3692
Cecidomyiidae|[2458]|RRMFI2052-15|632[0n]bp|Diptera|Cecidomyiidae|BOLD:AAH3691
Cecidomyiidae|[2459]|RRMFE1273-15|591[0n]bp|Diptera|Cecidomyiidae|BOLD:ACF3694
Cecidomyiidae|[2460]|RRMFE1763-15|540[0n]bp|Diptera|Cecidomyiidae|BOLD:ACK2185
Cecidomyiidae|[2461]|RRMFI2286-15|624[0n]bp|Diptera|Cecidomyiidae|BOLD:ACK3192
Cecidomyiidae|[2462]|RRMFG1171-15|588[0n]bp|Diptera|Cecidomyiidae|BOLD:ACV4879
Cecidomyiidae|[2463]|RRMFE3305-15|561[0n]bp|Diptera|Cecidomyiidae|BOLD:ACV4196
Cecidomyiidae|[2464]|RRMFG774-15|576[0n]bp|Diptera|Cecidomyiidae|BOLD:ACV5488
Cecidomyiidae|[2465]|RRMFG1019-15|579[0n]bp|Diptera|Cecidomyiidae|BOLD:AAN5271
Cecidomyiidae|[2466]|RRMFI1919-15|636[0n]bp|Diptera|Cecidomyiidae|BOLD:AAY6381
Cecidomyiidae|[2467]|RRMFI2104-15|636[0n]bp|Diptera|Cecidomyiidae|BOLD:ABW8023
Cecidomyiidae|[2468]|RRMFI1243-15|624[0n]bp|Diptera|Cecidomyiidae|BOLD:AAM6040
Cecidomyiidae|[2469]|RRMFI2663-15|636[0n]bp|Diptera|Cecidomyiidae|BOLD:AAN5220
Cecidomyiidae|[2470]|RRMFI1894-15|634[0n]bp|Diptera|Cecidomyiidae|BOLD:ABW7834
Cecidomyiidae|[2471]|RRMFG1849-15|600[0n]bp|Diptera|Cecidomyiidae|BOLD:ABA7871
Cecidomyiidae|[2472]|RRMFI456-15|540[0n]bp|Diptera|Cecidomyiidae|BOLD:ACV5891
Cecidomyiidae|[2473]|RRMFI1990-15|636[0n]bp|Diptera|Cecidomyiidae|BOLD:ACV5892
Cecidomyiidae|[2474]|RRMFI2233-15|624[0n]bp|Diptera|Cecidomyiidae|BOLD:AAN5198
Cecidomyiidae|[2475]|RRMFI2692-15|638[0n]bp|Diptera|Cecidomyiidae|BOLD:AAN5276
Cecidomyiidae|[2476]|RRMFG548-15|582[0n]bp|Diptera|Cecidomyiidae|BOLD:AAN5244
Cecidomyiidae|[2477]|RRMFI1026-15|636[0n]bp|Diptera|Cecidomyiidae|BOLD:ACU9494
Cecidomyiidae|[2478]|RRMFI1112-15|637[0n]bp|Diptera|Cecidomyiidae|BOLD:ABV9080
Cecidomyiidae|[2479]|RRMFI535-15|564[0n]bp|Diptera|Cecidomyiidae|BOLD:ABV0477
Cecidomyiidae|[2480]|RRMFI1217-15|624[0n]bp|Diptera|Cecidomyiidae|BOLD:ACB3295
Cecidomyiidae|[2481]|RRMFI762-15|576[0n]bp|Diptera|Cecidomyiidae|BOLD:ACV5110
Cecidomyiidae|[2482]|RRMFB401-15|564[0n]bp|Diptera|Cecidomyiidae|BOLD:ABA1219
Cecidomyiidae|[2483]|RRMFI2262-15|624[0n]bp|Diptera|Cecidomyiidae|BOLD:ACA6105
Cecidomyiidae|[2484]|RRMPG859-15|576[0n]bp|Diptera|Cecidomyiidae|BOLD:ACB5167
Cecidomyiidae|[2485]|RRMFG789-15|576[0n]bp|Diptera|Cecidomyiidae|BOLD:ACC8041
Cecidomyiidae|[2486]|RRMFG254-15|588[0n]bp|Diptera|Cecidomyiidae|BOLD:ABW2751
Cecidomyiidae|[2487]|RRMFC938-15|582[1n]bp|Diptera|Cecidomyiidae|BOLD:ACA3288
Cecidomyiidae|[2488]|RRMPB3873-15|576[0n]bp|Diptera|Cecidomyiidae|BOLD:ACV3885
Cecidomyiidae|[2489]|RRMFG1883-15|579[0n]bp|Diptera|Cecidomyiidae|BOLD:AAV5325
Cecidomyiidae|[2490]|RRMFE1253-15|579[0n]bp|Diptera|Cecidomyiidae|BOLD:ACV3079
Cecidomyiidae|[2491]|RRMFE1791-15|570[0n]bp|Diptera|Cecidomyiidae|BOLD:ABX7483
Cecidomyiidae|[2492]|RRMFE1452-15|591[0n]bp|Diptera|Cecidomyiidae|BOLD:ACV3083
Cecidomyiidae|[2493]|RRMFG1850-15|579[0n]bp|Diptera|Cecidomyiidae|BOLD:ACV4945
Cecidomyiinae|[2494]|RRMFE1236-15|588[0n]bp|Diptera|Cecidomyiidae|BOLD:ACK8692
Cecidomyiidae|[2495]|RRMFI859-15|636[0n]bp|Diptera|Cecidomyiidae|BOLD:ACW1275
Cecidomyiidae|[2496]|RRMFG628-15|576[0n]bp|Diptera|Cecidomyiidae|BOLD:ACU6072
Cecidomyiidae|[2497]|RRMFI095-15|579[0n]bp|Diptera|Cecidomyiidae|BOLD:ACV5761
Cecidomyiidae|[2498]|RRMFI2615-15|636[0n]bp|Diptera|Cecidomyiidae|BOLD:ACW1108
Cecidomyiidae|[2499]|RRMPC170-15|555[0n]bp|Diptera|Cecidomyiidae|BOLD:ACC5578
Cecidomyiidae|[2500]|RRMFI2203-15|640[0n]bp|Diptera|Cecidomyiidae|BOLD:AAY6432
Cecidomyiidae|[2501]|RRMFG126-15|552[0n]bp|Diptera|Cecidomyiidae|BOLD:ACV4441
Cecidomyiidae|[2502]|RRMFI1157-15|638[0n]bp|Diptera|Cecidomyiidae|BOLD:ACW1309
Cecidomyiidae|[2503]|RRMPC306-15|567[0n]bp|Diptera|Cecidomyiidae|BOLD:AAV5358
Cecidomyiidae|[2504]|RRSSA1172-15|588[0n]bp|Diptera|Cecidomyiidae|BOLD:ACK1253
Cecidomyiinae|[2505]|RRSSA2259-15|591[0n]bp|Diptera|Cecidomyiidae|BOLD:ACK5235
Cecidomyiidae|[2506]|RRMFC1466-15|576[0n]bp|Diptera|Cecidomyiidae|BOLD:ACJ8925
Cecidomyiidae|[2507]|RRMFC1184-15|552[0n]bp|Diptera|Cecidomyiidae|BOLD:ACV4007
Cecidomyiidae|[2508]|RRMFI902-15|636[0n]bp|Diptera|Cecidomyiidae|BOLD:ACV3021
Cecidomyiidae|[2509]|RRSSA1112-15|594[0n]bp|Diptera|Cecidomyiidae|BOLD:ACU5220
Cecidomyiidae|[2510]|RRMFE730-15|582[0n]bp|Diptera|Cecidomyiidae|BOLD:ABW5461
Cecidomyiidae|[2511]|RRMFE824-15|564[0n]bp|Diptera|Cecidomyiidae|BOLD:ACK7254
Cecidomyiidae|[2512]|RRMFE415-15|606[0n]bp|Diptera|Cecidomyiidae|BOLD:ACV5276
Cecidomyiidae|[2513]|RRMFE600-15|606[0n]bp|Diptera|Cecidomyiidae|BOLD:ACJ8520
Cecidomyiidae|[2514]|RRMFC1023-15|594[0n]bp|Diptera|Cecidomyiidae|BOLD:ACV3127
Cecidomyiidae|[2515]|RRSSA2255-15|588[0n]bp|Diptera|Cecidomyiidae|BOLD:ACW1780
Cecidomyiidae|[2516]|RRMFC193-15|561[0n]bp|Diptera|Cecidomyiidae|BOLD:ACV1921
Cecidomyiidae|[2517]|RRSSA2254-15|576[0n]bp|Diptera|Cecidomyiidae|BOLD:ACW2016
Cecidomyiidae|[2518]|RRMFI1031-15|636[0n]bp|Diptera|Cecidomyiidae|BOLD:AAN5238
Cecidomyiidae|[2519]|RRMFI827-15|636[0n]bp|Diptera|Cecidomyiidae|BOLD:ACR5313
Cecidomyiidae|[2520]|RRMFI2701-15|637[0n]bp|Diptera|Cecidomyiidae|BOLD:ACW1135
Cecidomyiidae|[2521]|RRMFI1962-15|632[0n]bp|Diptera|Cecidomyiidae|BOLD:AAY6397
Cecidomyiidae|[2522]|RRMFG1854-15|579[0n]bp|Diptera|Cecidomyiidae|BOLD:ABA7903
Cecidomyiidae|[2523]|RRMFI1668-15|636[0n]bp|Diptera|Cecidomyiidae|BOLD:ACI6169
Cecidomyiidae|[2524]|RRMFG1203-15|588[0n]bp|Diptera|Cecidomyiidae|BOLD:AAN5241
Cecidomyiidae|[2525]|RRMFI1684-15|636[0n]bp|Diptera|Cecidomyiidae|BOLD:ABA0856
Cecidomyiidae|[2526]|RRMFI2156-15|637[0n]bp|Diptera|Cecidomyiidae|BOLD:AAN5255
Cecidomyiidae|[2527]|RRMFI909-15|636[0n]bp|Diptera|Cecidomyiidae|BOLD:ACR0558
Cecidomyiidae|[2528]|RRMFG1812-15|582[0n]bp|Diptera|Cecidomyiidae|BOLD:ABV0455
Cecidomyiidae|[2529]|RRMFI814-15|636[0n]bp|Diptera|Cecidomyiidae|BOLD:ACE7472
Cecidomyiidae|[2530]|RRMFI2275-15|624[0n]bp|Diptera|Cecidomyiidae|BOLD:AAM6057
Cecidomyiidae|[2531]|RRMFG2178-15|576[0n]bp|Diptera|Cecidomyiidae|BOLD:ACS1971
Cecidomyiidae|[2532]|RRMFI1414-15|638[0n]bp|Diptera|Cecidomyiidae|BOLD:AAQ0296
Cecidomyiidae|[2533]|RRMFI1685-15|636[0n]bp|Diptera|Cecidomyiidae|BOLD:ACU6062
Cecidomyiidae|[2534]|RRMFI1688-15|636[0n]bp|Diptera|Cecidomyiidae|BOLD:ACU6282
Cecidomyiidae|[2535]|RRMFI2025-15|640[2n]bp|Diptera|Cecidomyiidae|BOLD:AAN5201
Cecidomyiidae|[2536]|RRMFI836-15|634[0n]bp|Diptera|Cecidomyiidae|BOLD:AAZ0297
Cecidomyiidae|[2537]|RRMFI1285-15|620[0n]bp|Diptera|Cecidomyiidae|BOLD:AAH3734
Cecidomyiidae|[2538]|RRMFI1857-15|636[0n]bp|Diptera|Cecidomyiidae|BOLD:AAN5227
Cecidomyiidae|[2539]|RRMFE2330-15|591[0n]bp|Diptera|Cecidomyiidae|BOLD:AAH3664
Cecidomyiidae|[2540]|RRMFI551-15|579[0n]bp|Diptera|Cecidomyiidae|BOLD:ACF4901
Cecidomyiidae|[2541]|RRMFI953-15|636[0n]bp|Diptera|Cecidomyiidae|BOLD:AAN5183
Cecidomyiidae|[2542]|RRMFI1635-15|614[0n]bp|Diptera|Cecidomyiidae|BOLD:AAV5569
Cecidomyiidae|[2543]|RRMFI1462-15|607[0n]bp|Diptera|Cecidomyiidae|BOLD:AAQ2553
Cecidomyiidae|[2544]|RRMFG1177-15|588[0n]bp|Diptera|Cecidomyiidae|BOLD:ACV4936
Cecidomyiidae|[2545]|RRMFI821-15|636[0n]bp|Diptera|Cecidomyiidae|BOLD:ACB4105
Cecidomyiidae|[2546]|RRMFI2033-15|632[0n]bp|Diptera|Cecidomyiidae|BOLD:AAH3661
Cecidomyiidae|[2547]|RRMFG114-15|588[0n]bp|Diptera|Cecidomyiidae|BOLD:AAV5568
Cecidomyiidae|[2548]|RRMFI1124-15|637[0n]bp|Diptera|Cecidomyiidae|BOLD:ACW0871
Cecidomyiidae|[2549]|RRMFI2315-15|624[0n]bp|Diptera|Cecidomyiidae|BOLD:AAN5242

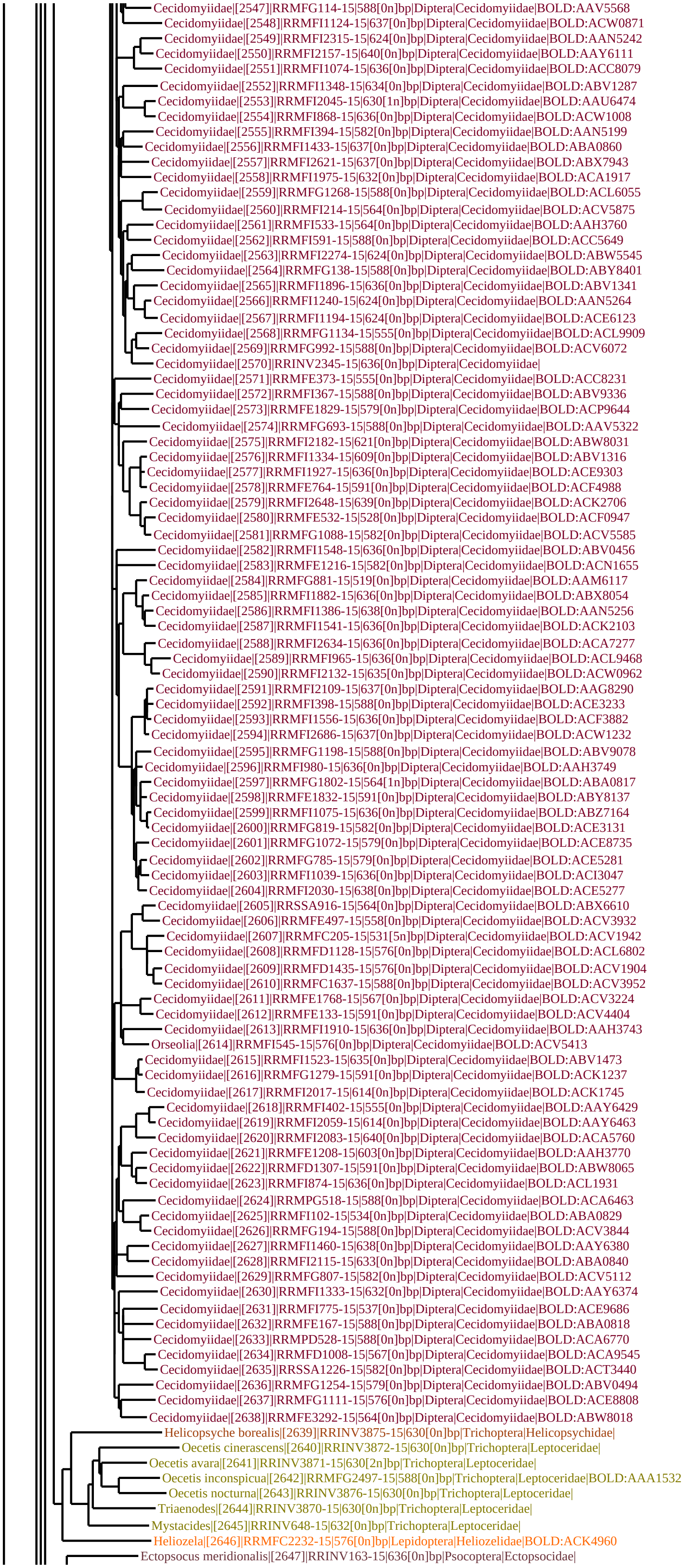

Cecidomyiidae|[2547]|RRMFG114-15|588[0n]bp|Diptera|Cecidomyiidae|BOLD:AAV5568
Cecidomyiidae|[2548]|RRMFI1124-15|637[0n]bp|Diptera|Cecidomyiidae|BOLD:ACW0871
Cecidomyiidae|[2549]|RRMFI2315-15|624[0n]bp|Diptera|Cecidomyiidae|BOLD:AAN5242
Cecidomyiidae|[2550]|RRMFI2157-15|640[0n]bp|Diptera|Cecidomyiidae|BOLD:AAY6111
Cecidomyiidae|[2551]|RRMFI1074-15|636[0n]bp|Diptera|Cecidomyiidae|BOLD:ACC8079
Cecidomyiidae|[2552]|RRMFI1348-15|634[0n]bp|Diptera|Cecidomyiidae|BOLD:ABV1287
Cecidomyiidae|[2553]|RRMFI2045-15|630[1n]bp|Diptera|Cecidomyiidae|BOLD:AAU6474
Cecidomyiidae|[2554]|RRMFI868-15|636[0n]bp|Diptera|Cecidomyiidae|BOLD:ACW1008
Cecidomyiidae|[2555]|RRMFI394-15|582[0n]bp|Diptera|Cecidomyiidae|BOLD:AAN5199
Cecidomyiidae|[2556]|RRMFI1433-15|637[0n]bp|Diptera|Cecidomyiidae|BOLD:ABA0860
Cecidomyiidae|[2557]|RRMFI2621-15|637[0n]bp|Diptera|Cecidomyiidae|BOLD:ABX7943
Cecidomyiidae|[2558]|RRMFI1975-15|632[0n]bp|Diptera|Cecidomyiidae|BOLD:ACA1917
Cecidomyiidae|[2559]|RRMFG1268-15|588[0n]bp|Diptera|Cecidomyiidae|BOLD:ACL6055
Cecidomyiidae|[2560]|RRMFI214-15|564[0n]bp|Diptera|Cecidomyiidae|BOLD:ACV5875
Cecidomyiidae|[2561]|RRMFI533-15|564[0n]bp|Diptera|Cecidomyiidae|BOLD:AAH3760
Cecidomyiidae|[2562]|RRMFI591-15|588[0n]bp|Diptera|Cecidomyiidae|BOLD:ACC5649
Cecidomyiidae|[2563]|RRMFI2274-15|624[0n]bp|Diptera|Cecidomyiidae|BOLD:ABW5545
Cecidomyiidae|[2564]|RRMFG138-15|588[0n]bp|Diptera|Cecidomyiidae|BOLD:ABY8401
Cecidomyiidae|[2565]|RRMFI1896-15|636[0n]bp|Diptera|Cecidomyiidae|BOLD:ABV1341
Cecidomyiidae|[2566]|RRMFI1240-15|624[0n]bp|Diptera|Cecidomyiidae|BOLD:AAN5264
Cecidomyiidae|[2567]|RRMFI1194-15|624[0n]bp|Diptera|Cecidomyiidae|BOLD:ACE6123
Cecidomyiidae|[2568]|RRMFG1134-15|555[0n]bp|Diptera|Cecidomyiidae|BOLD:ACL9909
Cecidomyiidae|[2569]|RRMFG992-15|588[0n]bp|Diptera|Cecidomyiidae|BOLD:ACV6072
Cecidomyiidae|[2570]|RRINV2345-15|636[0n]bp|Diptera|Cecidomyiidae|
Cecidomyiidae|[2571]|RRMFE373-15|555[0n]bp|Diptera|Cecidomyiidae|BOLD:ACC8231
Cecidomyiidae|[2572]|RRMFI367-15|588[0n]bp|Diptera|Cecidomyiidae|BOLD:ABV9336
Cecidomyiidae|[2573]|RRMFE1829-15|579[0n]bp|Diptera|Cecidomyiidae|BOLD:ACP9644
Cecidomyiidae|[2574]|RRMFG693-15|588[0n]bp|Diptera|Cecidomyiidae|BOLD:AAV5322
Cecidomyiidae|[2575]|RRMFI2182-15|621[0n]bp|Diptera|Cecidomyiidae|BOLD:ABW8031
Cecidomyiidae|[2576]|RRMFI1334-15|609[0n]bp|Diptera|Cecidomyiidae|BOLD:ABV1316
Cecidomyiidae|[2577]|RRMFI1927-15|636[0n]bp|Diptera|Cecidomyiidae|BOLD:ACE9303
Cecidomyiidae|[2578]|RRMFE764-15|591[0n]bp|Diptera|Cecidomyiidae|BOLD:ACF4988
Cecidomyiidae|[2579]|RRMFI2648-15|639[0n]bp|Diptera|Cecidomyiidae|BOLD:ACK2706
Cecidomyiidae|[2580]|RRMFE532-15|528[0n]bp|Diptera|Cecidomyiidae|BOLD:ACF0947
Cecidomyiidae|[2581]|RRMFG1088-15|582[0n]bp|Diptera|Cecidomyiidae|BOLD:ACV5585
Cecidomyiidae|[2582]|RRMFI1548-15|636[0n]bp|Diptera|Cecidomyiidae|BOLD:ABV0456
Cecidomyiidae|[2583]|RRMFE1216-15|582[0n]bp|Diptera|Cecidomyiidae|BOLD:ACN1655
Cecidomyiidae|[2584]|RRMFG881-15|519[0n]bp|Diptera|Cecidomyiidae|BOLD:AAM6117
Cecidomyiidae|[2585]|RRMFI1882-15|636[0n]bp|Diptera|Cecidomyiidae|BOLD:ABX8054
Cecidomyiidae|[2586]|RRMFI1386-15|638[0n]bp|Diptera|Cecidomyiidae|BOLD:AAN5256
Cecidomyiidae|[2587]|RRMFI1541-15|636[0n]bp|Diptera|Cecidomyiidae|BOLD:ACK2103
Cecidomyiidae|[2588]|RRMFI2634-15|636[0n]bp|Diptera|Cecidomyiidae|BOLD:ACA7277
Cecidomyiidae|[2589]|RRMFI965-15|636[0n]bp|Diptera|Cecidomyiidae|BOLD:ACL9468
Cecidomyiidae|[2590]|RRMFI2132-15|635[0n]bp|Diptera|Cecidomyiidae|BOLD:ACW0962
Cecidomyiidae|[2591]|RRMFI2109-15|637[0n]bp|Diptera|Cecidomyiidae|BOLD:AAG8290
Cecidomyiidae|[2592]|RRMFI398-15|588[0n]bp|Diptera|Cecidomyiidae|BOLD:ACE3233
Cecidomyiidae|[2593]|RRMFI1556-15|636[0n]bp|Diptera|Cecidomyiidae|BOLD:ACF3882
Cecidomyiidae|[2594]|RRMFI2686-15|637[0n]bp|Diptera|Cecidomyiidae|BOLD:ACW1232
Cecidomyiidae|[2595]|RRMFG1198-15|588[0n]bp|Diptera|Cecidomyiidae|BOLD:ABV9078
Cecidomyiidae|[2596]|RRMFI980-15|636[0n]bp|Diptera|Cecidomyiidae|BOLD:AAH3749
Cecidomyiidae|[2597]|RRMFG1802-15|564[1n]bp|Diptera|Cecidomyiidae|BOLD:ABA0817
Cecidomyiidae|[2598]|RRMFE1832-15|591[0n]bp|Diptera|Cecidomyiidae|BOLD:ABY8137
Cecidomyiidae|[2599]|RRMFI1075-15|636[0n]bp|Diptera|Cecidomyiidae|BOLD:ABZ7164
Cecidomyiidae|[2600]|RRMFG819-15|582[0n]bp|Diptera|Cecidomyiidae|BOLD:ACE3131
Cecidomyiidae|[2601]|RRMFG1072-15|579[0n]bp|Diptera|Cecidomyiidae|BOLD:ACE8735
Cecidomyiidae|[2602]|RRMFG785-15|579[0n]bp|Diptera|Cecidomyiidae|BOLD:ACE5281
Cecidomyiidae|[2603]|RRMFI1039-15|636[0n]bp|Diptera|Cecidomyiidae|BOLD:ACI3047
Cecidomyiidae|[2604]|RRMFI2030-15|638[0n]bp|Diptera|Cecidomyiidae|BOLD:ACE5277
Cecidomyiidae|[2605]|RRSSA916-15|564[0n]bp|Diptera|Cecidomyiidae|BOLD:ABX6610
Cecidomyiidae|[2606]|RRMFE497-15|558[0n]bp|Diptera|Cecidomyiidae|BOLD:ACV3932
Cecidomyiidae|[2607]|RRMFC205-15|531[5n]bp|Diptera|Cecidomyiidae|BOLD:ACV1942
Cecidomyiidae|[2608]|RRMFD1128-15|576[0n]bp|Diptera|Cecidomyiidae|BOLD:ACL6802
Cecidomyiidae|[2609]|RRMFD1435-15|576[0n]bp|Diptera|Cecidomyiidae|BOLD:ACV1904
Cecidomyiidae|[2610]|RRMFC1637-15|588[0n]bp|Diptera|Cecidomyiidae|BOLD:ACV3952
Cecidomyiidae|[2611]|RRMFE1768-15|567[0n]bp|Diptera|Cecidomyiidae|BOLD:ACV3224
Cecidomyiidae|[2612]|RRMFE133-15|591[0n]bp|Diptera|Cecidomyiidae|BOLD:ACV4404
Cecidomyiidae|[2613]|RRMFI1910-15|636[0n]bp|Diptera|Cecidomyiidae|BOLD:AAH3743
Orseolia|[2614]|RRMFI545-15|576[0n]bp|Diptera|Cecidomyiidae|BOLD:ACV5413
Cecidomyiidae|[2615]|RRMFI1523-15|635[0n]bp|Diptera|Cecidomyiidae|BOLD:ABV1473
Cecidomyiidae|[2616]|RRMFG1279-15|591[0n]bp|Diptera|Cecidomyiidae|BOLD:ACK1237
Cecidomyiidae|[2617]|RRMFI2017-15|614[0n]bp|Diptera|Cecidomyiidae|BOLD:ACK1745
Cecidomyiidae|[2618]|RRMFI402-15|555[0n]bp|Diptera|Cecidomyiidae|BOLD:AAY6429
Cecidomyiidae|[2619]|RRMFI2059-15|614[0n]bp|Diptera|Cecidomyiidae|BOLD:AAY6463
Cecidomyiidae|[2620]|RRMFI2083-15|640[0n]bp|Diptera|Cecidomyiidae|BOLD:ACA5760
Cecidomyiidae|[2621]|RRMFE1208-15|603[0n]bp|Diptera|Cecidomyiidae|BOLD:AAH3770
Cecidomyiidae|[2622]|RRMFD1307-15|591[0n]bp|Diptera|Cecidomyiidae|BOLD:ABW8065
Cecidomyiidae|[2623]|RRMFI874-15|636[0n]bp|Diptera|Cecidomyiidae|BOLD:ACL1931
Cecidomyiidae|[2624]|RRMPG518-15|588[0n]bp|Diptera|Cecidomyiidae|BOLD:ACA6463
Cecidomyiidae|[2625]|RRMFI102-15|534[0n]bp|Diptera|Cecidomyiidae|BOLD:ABA0829
Cecidomyiidae|[2626]|RRMFG194-15|588[0n]bp|Diptera|Cecidomyiidae|BOLD:ACV3844
Cecidomyiidae|[2627]|RRMFI1460-15|638[0n]bp|Diptera|Cecidomyiidae|BOLD:AAY6380
Cecidomyiidae|[2628]|RRMFI2115-15|633[0n]bp|Diptera|Cecidomyiidae|BOLD:ABA0840
Cecidomyiidae|[2629]|RRMFG807-15|582[0n]bp|Diptera|Cecidomyiidae|BOLD:ACV5112
Cecidomyiidae|[2630]|RRMFI1333-15|632[0n]bp|Diptera|Cecidomyiidae|BOLD:AAY6374
Cecidomyiidae|[2631]|RRMFI775-15|537[0n]bp|Diptera|Cecidomyiidae|BOLD:ACE9686
Cecidomyiidae|[2632]|RRMFE167-15|588[0n]bp|Diptera|Cecidomyiidae|BOLD:ABA0818
Cecidomyiidae|[2633]|RRMPD528-15|588[0n]bp|Diptera|Cecidomyiidae|BOLD:ACA6770
Cecidomyiidae|[2634]|RRMFD1008-15|567[0n]bp|Diptera|Cecidomyiidae|BOLD:ACA9545
Cecidomyiidae|[2635]|RRSSA1226-15|582[0n]bp|Diptera|Cecidomyiidae|BOLD:ACT3440
Cecidomyiidae|[2636]|RRMFG1254-15|579[0n]bp|Diptera|Cecidomyiidae|BOLD:ABV0494
Cecidomyiidae|[2637]|RRMFG1111-15|576[0n]bp|Diptera|Cecidomyiidae|BOLD:ACE8808
Cecidomyiidae|[2638]|RRMFE3292-15|564[0n]bp|Diptera|Cecidomyiidae|BOLD:ABW8018
Helicopsyche borealis|[2639]|RRINV3875-15|630[0n]bp|Trichoptera|Helicopsychidae|
Oecetis cinerascens|[2640]|RRINV3872-15|630[0n]bp|Trichoptera|Leptoceridae|
Oecetis avara|[2641]|RRINV3871-15|630[2n]bp|Trichoptera|Leptoceridae|
Oecetis inconspicua|[2642]|RRMFG2497-15|588[0n]bp|Trichoptera|Leptoceridae|BOLD:AAA1532
Oecetis nocturna|[2643]|RRINV3876-15|630[0n]bp|Trichoptera|Leptoceridae|
Triaenodes|[2644]|RRINV3870-15|630[0n]bp|Trichoptera|Leptoceridae|
Mystacides|[2645]|RRINV648-15|632[0n]bp|Trichoptera|Leptoceridae|
Heliozela|[2646]|RRMFC2232-15|576[0n]bp|Lepidoptera|Heliozelidae|BOLD:ACK4960
Ectopsocus meridionalis|[2647]|RRINV163-15|636[0n]bp|Psocoptera|Ectopsocidae|

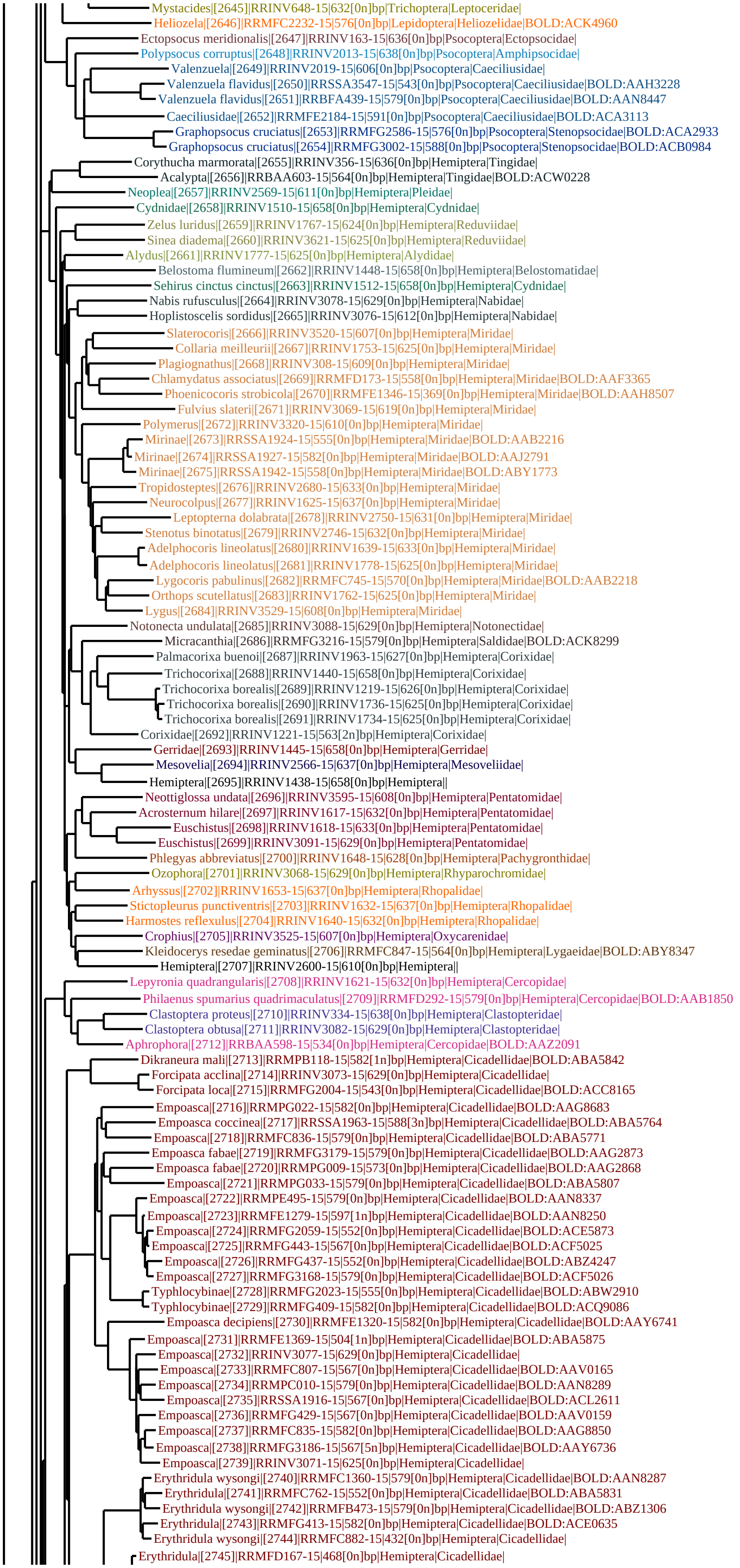
Mystacides|[2645]|RRINV648-15|632[0n]bp|Trichoptera|Leptoceridae|
Heliozela|[2646]|RRMFC2232-15|576[0n]bp|Lepidoptera|Heliozelidae|BOLD:ACK4960
Ectopsocus meridionalis|[2647]|RRINV163-15|636[0n]bp|Psocoptera|Ectopsocidae|
Polypsocus corruptus|[2648]|RRINV2013-15|638[0n]bp|Psocoptera|Amphipsocidae|
Valenzuela|[2649]|RRINV2019-15|606[0n]bp|Psocoptera|Caeciliusidae|
Valenzuela flavidus|[2650]|RRSSA3547-15|543[0n]bp|Psocoptera|Caeciliusidae|BOLD:AAH3228
Valenzuela flavidus|[2651]|RRBFA439-15|579[0n]bp|Psocoptera|Caeciliusidae|BOLD:AAN8447
Caeciliusidae|[2652]|RRMFE2184-15|591[0n]bp|Psocoptera|Caeciliusidae|BOLD:ACA3113
Graphopsocus cruciatus|[2653]|RRMFG2586-15|576[0n]bp|Psocoptera|Stenopsocidae|BOLD:ACA2933
Graphopsocus cruciatus|[2654]|RRMFG3002-15|588[0n]bp|Psocoptera|Stenopsocidae|BOLD:ACB0984
Corythucha marmorata|[2655]|RRINV356-15|636[0n]bp|Hemiptera|Tingidae|
Acalypta|[2656]|RRBAA603-15|564[0n]bp|Hemiptera|Tingidae|BOLD:ACW0228
Neoplea|[2657]|RRINV2569-15|611[0n]bp|Hemiptera|Pleidae|
Cydnidae|[2658]|RRINV1510-15|658[0n]bp|Hemiptera|Cydnidae|
Zelus luridus|[2659]|RRINV1767-15|624[0n]bp|Hemiptera|Reduviidae|
Sinea diadema|[2660]|RRINV3621-15|625[0n]bp|Hemiptera|Reduviidae|
Alydus|[2661]|RRINV1777-15|625[0n]bp|Hemiptera|Alydidae|
Belostoma flumineum|[2662]|RRINV1448-15|658[0n]bp|Hemiptera|Belostomatidae|
Sehirus cinctus cinctus|[2663]|RRINV1512-15|658[0n]bp|Hemiptera|Cydnidae|
Nabis rufusculus|[2664]|RRINV3078-15|629[0n]bp|Hemiptera|Nabidae|
Hoplistoscelis sordidus|[2665]|RRINV3076-15|612[0n]bp|Hemiptera|Nabidae|
Slaterocoris|[2666]|RRINV3520-15|607[0n]bp|Hemiptera|Miridae|
Collaria meilleurii|[2667]|RRINV1753-15|625[0n]bp|Hemiptera|Miridae|
Plagiognathus|[2668]|RRINV308-15|609[0n]bp|Hemiptera|Miridae|
Chlamydatus associatus|[2669]|RRMFD173-15|558[0n]bp|Hemiptera|Miridae|BOLD:AAF3365
Phoenicocoris strobicola|[2670]|RRMFE1346-15|369[0n]bp|Hemiptera|Miridae|BOLD:AAH8507
Fulvius slateri|[2671]|RRINV3069-15|619[0n]bp|Hemiptera|Miridae|
Polymerus|[2672]|RRINV3320-15|610[0n]bp|Hemiptera|Miridae|
Mirinae|[2673]|RRSSA1924-15|555[0n]bp|Hemiptera|Miridae|BOLD:AAB2216
Mirinae|[2674]|RRSSA1927-15|582[0n]bp|Hemiptera|Miridae|BOLD:AAJ2791
Mirinae|[2675]|RRSSA1942-15|558[0n]bp|Hemiptera|Miridae|BOLD:ABY1773
Tropidosteptes|[2676]|RRINV2680-15|633[0n]bp|Hemiptera|Miridae|
Neurocolpus|[2677]|RRINV1625-15|637[0n]bp|Hemiptera|Miridae|
Leptopterna dolabrata|[2678]|RRINV2750-15|631[0n]bp|Hemiptera|Miridae|
Stenotus binotatus|[2679]|RRINV2746-15|632[0n]bp|Hemiptera|Miridae|
Adelphocoris lineolatus|[2680]|RRINV1639-15|633[0n]bp|Hemiptera|Miridae|
Adelphocoris lineolatus|[2681]|RRINV1778-15|625[0n]bp|Hemiptera|Miridae|
Lygocoris pabulinus|[2682]|RRMFC745-15|570[0n]bp|Hemiptera|Miridae|BOLD:AAB2218
Orthops scutellatus|[2683]|RRINV1762-15|625[0n]bp|Hemiptera|Miridae|
Lygus|[2684]|RRINV3529-15|608[0n]bp|Hemiptera|Miridae|
Notonecta undulata|[2685]|RRINV3088-15|629[0n]bp|Hemiptera|Notonectidae|
Micracanthia|[2686]|RRMFG3216-15|579[0n]bp|Hemiptera|Saldidae|BOLD:ACK8299
Palmacorixa buenoi|[2687]|RRINV1963-15|627[0n]bp|Hemiptera|Corixidae|
Trichocorixa|[2688]|RRINV1440-15|658[0n]bp|Hemiptera|Corixidae|
Trichocorixa borealis|[2689]|RRINV1219-15|626[0n]bp|Hemiptera|Corixidae|
Trichocorixa borealis|[2690]|RRINV1736-15|625[0n]bp|Hemiptera|Corixidae|
Trichocorixa borealis|[2691]|RRINV1734-15|625[0n]bp|Hemiptera|Corixidae|
Corixidae|[2692]|RRINV1221-15|563[2n]bp|Hemiptera|Corixidae|
Gerridae|[2693]|RRINV1445-15|658[0n]bp|Hemiptera|Gerridae|
Mesovelia|[2694]|RRINV2566-15|637[0n]bp|Hemiptera|Mesoveliidae|
Hemiptera|[2695]|RRINV1438-15|658[0n]bp|Hemiptera||
Neottiglossa undata|[2696]|RRINV3595-15|608[0n]bp|Hemiptera|Pentatomidae|
Acrosternum hilare|[2697]|RRINV1617-15|632[0n]bp|Hemiptera|Pentatomidae|
Euschistus|[2698]|RRINV1618-15|633[0n]bp|Hemiptera|Pentatomidae|
Euschistus|[2699]|RRINV3091-15|629[0n]bp|Hemiptera|Pentatomidae|
Phlegyas abbreviatus|[2700]|RRINV1648-15|628[0n]bp|Hemiptera|Pachygronthidae|
Ozophora|[2701]|RRINV3068-15|629[0n]bp|Hemiptera|Rhyparochromidae|
Arhyssus|[2702]|RRINV1653-15|637[0n]bp|Hemiptera|Rhopalidae|
Stictopleurus punctiventris|[2703]|RRINV1632-15|637[0n]bp|Hemiptera|Rhopalidae|
Harmostes reflexulus|[2704]|RRINV1640-15|632[0n]bp|Hemiptera|Rhopalidae|
Crophius|[2705]|RRINV3525-15|607[0n]bp|Hemiptera|Oxycarenidae|
Kleidocerys resedae geminatus|[2706]|RRMFC847-15|564[0n]bp|Hemiptera|Lygaeidae|BOLD:ABY8347
Hemiptera|[2707]|RRINV2600-15|610[0n]bp|Hemiptera||
Lepyronia quadrangularis|[2708]|RRINV1621-15|632[0n]bp|Hemiptera|Cercopidae|
Philaenus spumarius quadrimaculatus|[2709]|RRMFD292-15|579[0n]bp|Hemiptera|Cercopidae|BOLD:AAB1850
Clastoptera proteus|[2710]|RRINV334-15|638[0n]bp|Hemiptera|Clastopteridae|
Clastoptera obtusa|[2711]|RRINV3082-15|629[0n]bp|Hemiptera|Clastopteridae|
Aphrophora|[2712]|RRBAA598-15|534[0n]bp|Hemiptera|Cercopidae|BOLD:AAZ2091
Dikraneura mali|[2713]|RRMPB118-15|582[1n]bp|Hemiptera|Cicadellidae|BOLD:ABA5842
Forcipata acclina|[2714]|RRINV3073-15|629[0n]bp|Hemiptera|Cicadellidae|
Forcipata loca|[2715]|RRMFG2004-15|543[0n]bp|Hemiptera|Cicadellidae|BOLD:ACC8165
Empoasca|[2716]|RRMPG022-15|582[0n]bp|Hemiptera|Cicadellidae|BOLD:AAG8683
Empoasca coccinea|[2717]|RRSSA1963-15|588[3n]bp|Hemiptera|Cicadellidae|BOLD:ABA5764
Empoasca|[2718]|RRMFC836-15|579[0n]bp|Hemiptera|Cicadellidae|BOLD:ABA5771
Empoasca fabae|[2719]|RRMFG3179-15|579[0n]bp|Hemiptera|Cicadellidae|BOLD:AAG2873
Empoasca fabae|[2720]|RRMPG009-15|573[0n]bp|Hemiptera|Cicadellidae|BOLD:AAG2868
Empoasca|[2721]|RRMPG033-15|579[0n]bp|Hemiptera|Cicadellidae|BOLD:ABA5807
Empoasca|[2722]|RRMPE495-15|579[0n]bp|Hemiptera|Cicadellidae|BOLD:AAN8337
Empoasca|[2723]|RRMFE1279-15|597[1n]bp|Hemiptera|Cicadellidae|BOLD:AAN8250
Empoasca|[2724]|RRMFG2059-15|552[0n]bp|Hemiptera|Cicadellidae|BOLD:ACE5873
Empoasca|[2725]|RRMFG443-15|567[0n]bp|Hemiptera|Cicadellidae|BOLD:ACF5025
Empoasca|[2726]|RRMFG437-15|552[0n]bp|Hemiptera|Cicadellidae|BOLD:ABZ4247
Empoasca|[2727]|RRMFG3168-15|579[0n]bp|Hemiptera|Cicadellidae|BOLD:ACF5026
Typhlocybinae|[2728]|RRMFG2023-15|555[0n]bp|Hemiptera|Cicadellidae|BOLD:ABW2910
Typhlocybinae|[2729]|RRMFG409-15|582[0n]bp|Hemiptera|Cicadellidae|BOLD:ACQ9086
Empoasca decipiens|[2730]|RRMFE1320-15|582[0n]bp|Hemiptera|Cicadellidae|BOLD:AAY6741
Empoasca|[2731]|RRMFE1369-15|504[1n]bp|Hemiptera|Cicadellidae|BOLD:ABA5875
Empoasca|[2732]|RRINV3077-15|629[0n]bp|Hemiptera|Cicadellidae|
Empoasca|[2733]|RRMFC807-15|567[0n]bp|Hemiptera|Cicadellidae|BOLD:AAV0165
Empoasca|[2734]|RRMPC010-15|579[0n]bp|Hemiptera|Cicadellidae|BOLD:AAN8289
Empoasca|[2735]|RRSSA1916-15|567[0n]bp|Hemiptera|Cicadellidae|BOLD:ACL2611
Empoasca|[2736]|RRMFG429-15|567[0n]bp|Hemiptera|Cicadellidae|BOLD:AAV0159
Empoasca|[2737]|RRMFC835-15|582[0n]bp|Hemiptera|Cicadellidae|BOLD:AAG8850
Empoasca|[2738]|RRMFG3186-15|567[5n]bp|Hemiptera|Cicadellidae|BOLD:AAY6736
Empoasca|[2739]|RRINV3071-15|625[0n]bp|Hemiptera|Cicadellidae|
Erythridula wysongi|[2740]|RRMFC1360-15|579[0n]bp|Hemiptera|Cicadellidae|BOLD:AAN8287
Erythridula|[2741]|RRMFC762-15|552[0n]bp|Hemiptera|Cicadellidae|BOLD:ABA5831
Erythridula wysongi|[2742]|RRMFB473-15|579[0n]bp|Hemiptera|Cicadellidae|BOLD:ABZ1306
Erythridula|[2743]|RRMFG413-15|582[0n]bp|Hemiptera|Cicadellidae|BOLD:ACE0635
Erythridula wysongi|[2744]|RRMFC882-15|432[0n]bp|Hemiptera|Cicadellidae|
Erythridula|[2745]|RRMFD167-15|468[0n]bp|Hemiptera|Cicadellidae|

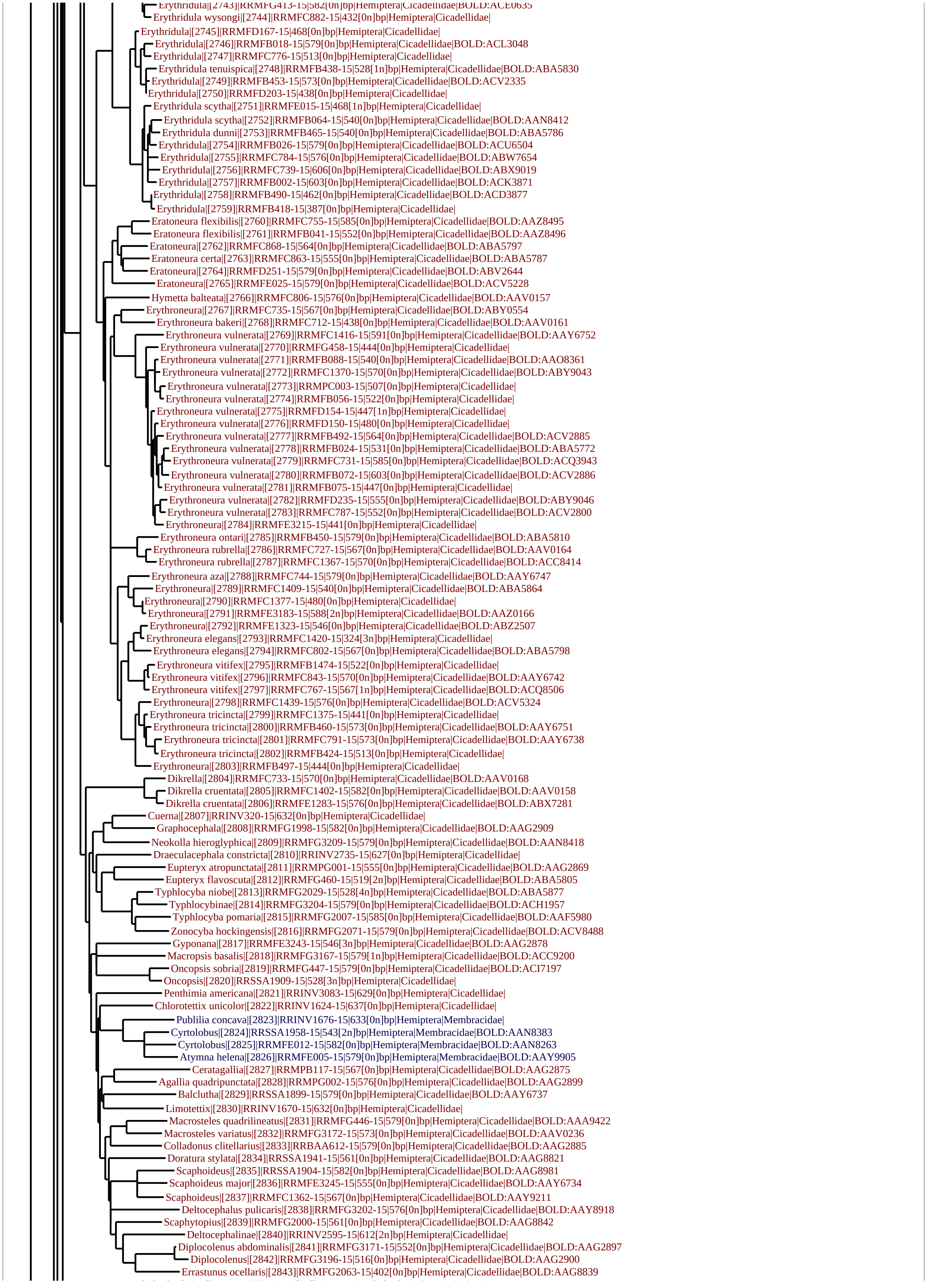

Erythridula|[2743]|RRMFG413-15|582[0n]bp|Hemiptera|Cicadellidae|BOLD:ACE0635
Erythridula wysongi|[2744]|RRMFC882-15|432[0n]bp|Hemiptera|Cicadellidae|
Erythridula|[2745]|RRMFD167-15|468[0n]bp|Hemiptera|Cicadellidae|
Erythridula|[2746]|RRMFB018-15|579[0n]bp|Hemiptera|Cicadellidae|BOLD:ACL3048
Erythridula|[2747]|RRMFC776-15|513[0n]bp|Hemiptera|Cicadellidae|
Erythridula tenuispica|[2748]|RRMFB438-15|528[1n]bp|Hemiptera|Cicadellidae|BOLD:ABA5830
Erythridula|[2749]|RRMFB453-15|573[0n]bp|Hemiptera|Cicadellidae|BOLD:ACV2335
Erythridula|[2750]|RRMFD203-15|438[0n]bp|Hemiptera|Cicadellidae|
Erythridula scytha|[2751]|RRMFE015-15|468[1n]bp|Hemiptera|Cicadellidae|
Erythridula scytha|[2752]|RRMFB064-15|540[0n]bp|Hemiptera|Cicadellidae|BOLD:AAN8412
Erythridula dunni|[2753]|RRMFB465-15|540[0n]bp|Hemiptera|Cicadellidae|BOLD:ABA5786
Erythridula|[2754]|RRMFB026-15|579[0n]bp|Hemiptera|Cicadellidae|BOLD:ACU6504
Erythridula|[2755]|RRMFC784-15|576[0n]bp|Hemiptera|Cicadellidae|BOLD:ABW7654
Erythridula|[2756]|RRMFC739-15|606[0n]bp|Hemiptera|Cicadellidae|BOLD:ABX9019
Erythridula|[2757]|RRMFB002-15|603[0n]bp|Hemiptera|Cicadellidae|BOLD:ACK3871
Erythridula|[2758]|RRMFB490-15|462[0n]bp|Hemiptera|Cicadellidae|BOLD:ACD3877
Erythridula|[2759]|RRMFB418-15|387[0n]bp|Hemiptera|Cicadellidae|
Eratoneura flexibilis|[2760]|RRMFC755-15|585[0n]bp|Hemiptera|Cicadellidae|BOLD:AAZ8495
Eratoneura flexibilis|[2761]|RRMFB041-15|552[0n]bp|Hemiptera|Cicadellidae|BOLD:AAZ8496
Eratoneura|[2762]|RRMFC868-15|564[0n]bp|Hemiptera|Cicadellidae|BOLD:ABA5797
Eratoneura certa|[2763]|RRMFC863-15|555[0n]bp|Hemiptera|Cicadellidae|BOLD:ABA5787
Eratoneura|[2764]|RRMFD251-15|579[0n]bp|Hemiptera|Cicadellidae|BOLD:ABV2644
Eratoneura|[2765]|RRMFE025-15|579[0n]bp|Hemiptera|Cicadellidae|BOLD:ACV5228
Hymetta balteata|[2766]|RRMFC806-15|576[0n]bp|Hemiptera|Cicadellidae|BOLD:AAV0157
Erythroneura|[2767]|RRMFC735-15|567[0n]bp|Hemiptera|Cicadellidae|BOLD:ABY0554
Erythroneura bakeri|[2768]|RRMFC712-15|438[0n]bp|Hemiptera|Cicadellidae|BOLD:AAV0161
Erythroneura vulnerata|[2769]|RRMFC1416-15|591[0n]bp|Hemiptera|Cicadellidae|BOLD:AAY6752
Erythroneura vulnerata|[2770]|RRMFG458-15|444[0n]bp|Hemiptera|Cicadellidae|
Erythroneura vulnerata|[2771]|RRMFB088-15|540[0n]bp|Hemiptera|Cicadellidae|BOLD:AAO8361
Erythroneura vulnerata|[2772]|RRMFC1370-15|570[0n]bp|Hemiptera|Cicadellidae|BOLD:ABY9043
Erythroneura vulnerata|[2773]|RRMPC003-15|507[0n]bp|Hemiptera|Cicadellidae|
Erythroneura vulnerata|[2774]|RRMFB056-15|522[0n]bp|Hemiptera|Cicadellidae|
Erythroneura vulnerata|[2775]|RRMFD154-15|447[1n]bp|Hemiptera|Cicadellidae|
Erythroneura vulnerata|[2776]|RRMFD150-15|480[0n]bp|Hemiptera|Cicadellidae|
Erythroneura vulnerata|[2777]|RRMFB492-15|564[0n]bp|Hemiptera|Cicadellidae|BOLD:ACV2885
Erythroneura vulnerata|[2778]|RRMFB024-15|531[0n]bp|Hemiptera|Cicadellidae|BOLD:ABA5772
Erythroneura vulnerata|[2779]|RRMFC731-15|585[0n]bp|Hemiptera|Cicadellidae|BOLD:ACQ3943
Erythroneura vulnerata|[2780]|RRMFB072-15|603[0n]bp|Hemiptera|Cicadellidae|BOLD:ACV2886
Erythroneura vulnerata|[2781]|RRMFB075-15|447[0n]bp|Hemiptera|Cicadellidae|
Erythroneura vulnerata|[2782]|RRMFD235-15|555[0n]bp|Hemiptera|Cicadellidae|BOLD:ABY9046
Erythroneura vulnerata|[2783]|RRMFC787-15|552[0n]bp|Hemiptera|Cicadellidae|BOLD:ACV2800
Erythroneura|[2784]|RRMFE3215-15|441[0n]bp|Hemiptera|Cicadellidae|
Erythroneura ontari|[2785]|RRMFB450-15|579[0n]bp|Hemiptera|Cicadellidae|BOLD:ABA5810
Erythroneura rubrella|[2786]|RRMFC727-15|567[0n]bp|Hemiptera|Cicadellidae|BOLD:AAV0164
Erythroneura rubrella|[2787]|RRMFC1367-15|570[0n]bp|Hemiptera|Cicadellidae|BOLD:ACC8414
Erythroneura aza|[2788]|RRMFC744-15|579[0n]bp|Hemiptera|Cicadellidae|BOLD:AAY6747
Erythroneura|[2789]|RRMFC1409-15|540[0n]bp|Hemiptera|Cicadellidae|BOLD:ABA5864
Erythroneura|[2790]|RRMFC1377-15|480[0n]bp|Hemiptera|Cicadellidae|
Erythroneura|[2791]|RRMFE3183-15|588[2n]bp|Hemiptera|Cicadellidae|BOLD:AAZ0166
Erythroneura|[2792]|RRMFE1323-15|546[0n]bp|Hemiptera|Cicadellidae|BOLD:ABZ2507
Erythroneura elegans|[2793]|RRMFC1420-15|324[3n]bp|Hemiptera|Cicadellidae|
Erythroneura elegans|[2794]|RRMFC802-15|567[0n]bp|Hemiptera|Cicadellidae|BOLD:ABA5798
Erythroneura vitifex|[2795]|RRMFB1474-15|522[0n]bp|Hemiptera|Cicadellidae|
Erythroneura vitifex|[2796]|RRMFC843-15|570[0n]bp|Hemiptera|Cicadellidae|BOLD:AAY6742
Erythroneura vitifex|[2797]|RRMFC767-15|567[1n]bp|Hemiptera|Cicadellidae|BOLD:ACQ8506
Erythroneura|[2798]|RRMFC1439-15|576[0n]bp|Hemiptera|Cicadellidae|BOLD:ACV5324
Erythroneura tricincta|[2799]|RRMFC1375-15|441[0n]bp|Hemiptera|Cicadellidae|
Erythroneura tricincta|[2800]|RRMFB460-15|573[0n]bp|Hemiptera|Cicadellidae|BOLD:AAY6751
Erythroneura tricincta|[2801]|RRMFC791-15|573[0n]bp|Hemiptera|Cicadellidae|BOLD:AAY6738
Erythroneura tricincta|[2802]|RRMFB424-15|513[0n]bp|Hemiptera|Cicadellidae|
Erythroneura|[2803]|RRMFB497-15|444[0n]bp|Hemiptera|Cicadellidae|
Dikrella|[2804]|RRMFC733-15|570[0n]bp|Hemiptera|Cicadellidae|BOLD:AAV0168
Dikrella cruentata|[2805]|RRMFC1402-15|582[0n]bp|Hemiptera|Cicadellidae|BOLD:AAV0158
Dikrella cruentata|[2806]|RRMFE1283-15|576[0n]bp|Hemiptera|Cicadellidae|BOLD:ABX7281
Cuerna|[2807]|RRINV320-15|632[0n]bp|Hemiptera|Cicadellidae|
Graphocephala|[2808]|RRMFG1998-15|582[0n]bp|Hemiptera|Cicadellidae|BOLD:AAG2909
Neokolla hieroglyphica|[2809]|RRMFG3209-15|579[0n]bp|Hemiptera|Cicadellidae|BOLD:AAN8418
Draeculacephala constricta|[2810]|RRINV2735-15|627[0n]bp|Hemiptera|Cicadellidae|
Eupteryx atropunctata|[2811]|RRMPG001-15|555[0n]bp|Hemiptera|Cicadellidae|BOLD:AAG2869
Eupteryx flavoscuta|[2812]|RRMFG460-15|519[2n]bp|Hemiptera|Cicadellidae|BOLD:ABA5805
Typhlocyba niobe|[2813]|RRMFG2029-15|528[4n]bp|Hemiptera|Cicadellidae|BOLD:ABA5877
Typhlocybinae|[2814]|RRMFG3204-15|579[0n]bp|Hemiptera|Cicadellidae|BOLD:ACH1957
Typhlocyba pomaria|[2815]|RRMFG2007-15|585[0n]bp|Hemiptera|Cicadellidae|BOLD:AAF5980
Zonocyba hockingensis|[2816]|RRMFG2071-15|579[0n]bp|Hemiptera|Cicadellidae|BOLD:ACV8488
Gyponana|[2817]|RRMFE3243-15|546[3n]bp|Hemiptera|Cicadellidae|BOLD:AAG2878
Macropsis basalis|[2818]|RRMFG3167-15|579[1n]bp|Hemiptera|Cicadellidae|BOLD:ACC9200
Oncopsis sobria|[2819]|RRMFG447-15|579[0n]bp|Hemiptera|Cicadellidae|BOLD:ACI7197
Oncopsis|[2820]|RRSSA1909-15|528[3n]bp|Hemiptera|Cicadellidae|
Penthimia americana|[2821]|RRINV3083-15|629[0n]bp|Hemiptera|Cicadellidae|
Chlorotettix unicolor|[2822]|RRINV1624-15|637[0n]bp|Hemiptera|Cicadellidae|
Publilia concava|[2823]|RRINV1676-15|633[0n]bp|Hemiptera|Membracidae|
Cyrtolobus|[2824]|RRSSA1958-15|543[2n]bp|Hemiptera|Membracidae|BOLD:AAN8383
Cyrtolobus|[2825]|RRMFE012-15|582[0n]bp|Hemiptera|Membracidae|BOLD:AAN8263
Atymna helena|[2826]|RRMFE005-15|579[0n]bp|Hemiptera|Membracidae|BOLD:AAY9905
Ceratagallia|[2827]|RRMPB117-15|567[0n]bp|Hemiptera|Cicadellidae|BOLD:AAG2875
Agallia quadripunctata|[2828]|RRMPG002-15|576[0n]bp|Hemiptera|Cicadellidae|BOLD:AAG2899
Balclutha|[2829]|RRSSA1899-15|579[0n]bp|Hemiptera|Cicadellidae|BOLD:AAY6737
Limotettix|[2830]|RRINV1670-15|632[0n]bp|Hemiptera|Cicadellidae|
Macrosteles quadrilineatus|[2831]|RRMFG446-15|579[0n]bp|Hemiptera|Cicadellidae|BOLD:AAA9422
Macrosteles variatus|[2832]|RRMFG3172-15|573[0n]bp|Hemiptera|Cicadellidae|BOLD:AAV0236
Colladonus clitellarius|[2833]|RRBAA612-15|579[0n]bp|Hemiptera|Cicadellidae|BOLD:AAG2885
Doratura stylata|[2834]|RRSSA1941-15|561[0n]bp|Hemiptera|Cicadellidae|BOLD:AAG8821
Scaphoideus|[2835]|RRSSA1904-15|582[0n]bp|Hemiptera|Cicadellidae|BOLD:AAG8981
Scaphoideus major|[2836]|RRMFE3245-15|555[0n]bp|Hemiptera|Cicadellidae|BOLD:AAY6734
Scaphoideus|[2837]|RRMFC1362-15|567[0n]bp|Hemiptera|Cicadellidae|BOLD:AAY9211
Deltocephalus pulicaris|[2838]|RRMFG3202-15|576[0n]bp|Hemiptera|Cicadellidae|BOLD:AAY8918
Scaphytopius|[2839]|RRMFG2000-15|561[0n]bp|Hemiptera|Cicadellidae|BOLD:AAG8842
Deltocephalinae|[2840]|RRINV2595-15|612[2n]bp|Hemiptera|Cicadellidae|
Diplocolenus abdominalis|[2841]|RRMFG3171-15|552[0n]bp|Hemiptera|Cicadellidae|BOLD:AAG2897
Diplocolenus|[2842]|RRMFG3196-15|516[0n]bp|Hemiptera|Cicadellidae|BOLD:AAG2900
Errastunus ocellaris|[2843]|RRMFG2063-15|402[0n]bp|Hemiptera|Cicadellidae|BOLD:AAG8839

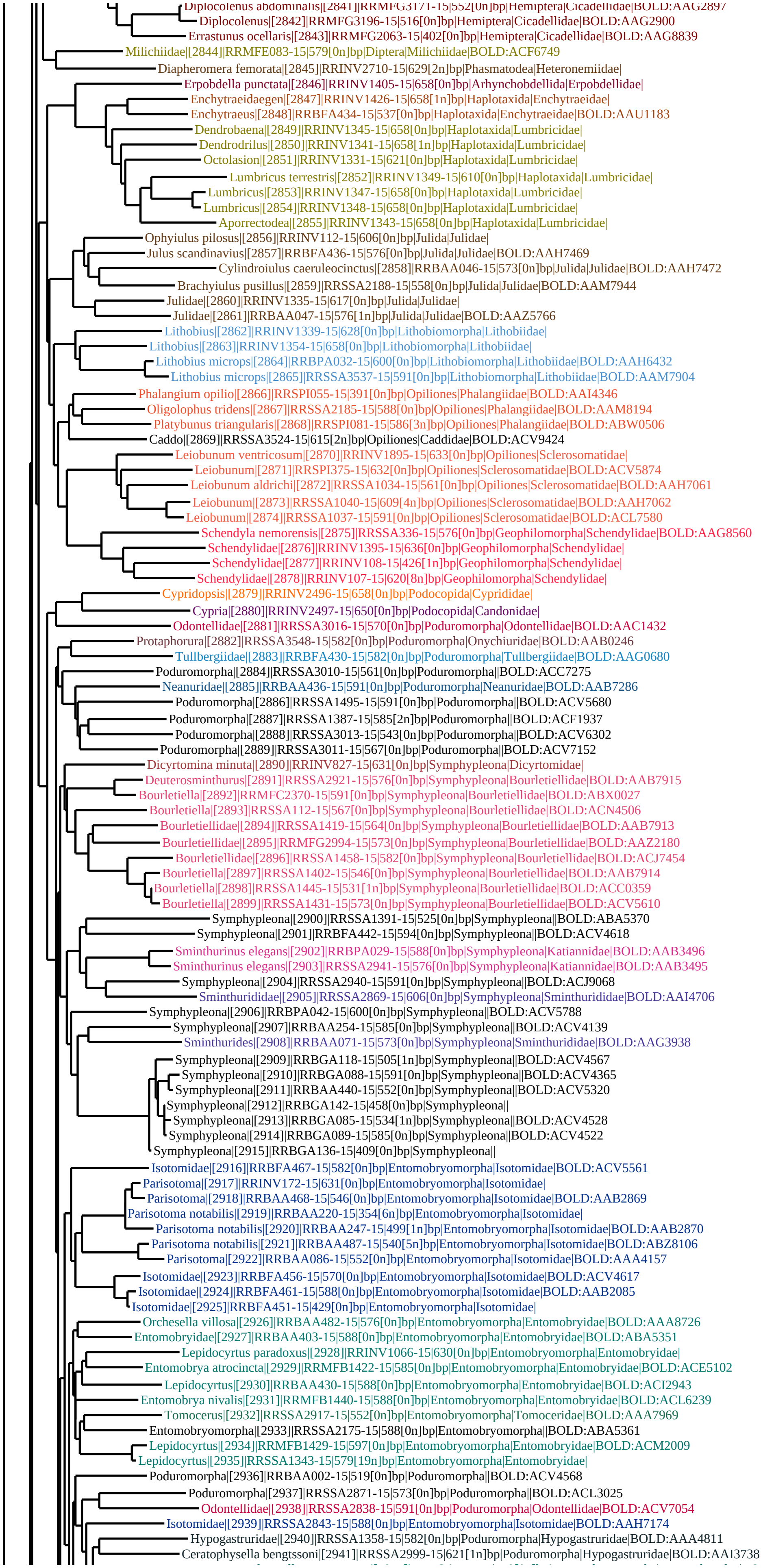

Diplocolenus abdominalis|[2841]|RRMFG3171-15|552[0n]bp|Hemiptera|Cicadellidae|BOLD:AAG2897
Diplocolenus|[2842]|RRMFG3196-15|516[0n]bp|Hemiptera|Cicadellidae|BOLD:AAG2900
Errastunus ocellaris|[2843]|RRMFG2063-15|402[0n]bp|Hemiptera|Cicadellidae|BOLD:AAG8839
Milichiidae|[2844]|RRMFE083-15|579[0n]bp|Diptera|Milichiidae|BOLD:ACF6749
Diapheromera femorata|[2845]|RRINV2710-15|629[2n]bp|Phasmatodea|Heteronemiidae|
Erpobdella punctata|[2846]|RRINV1405-15|658[0n]bp|Arhynchobdellida|Erpobdellidae|
Enchytraeidaegen|[2847]|RRINV1426-15|658[1n]bp|Haplotaxida|Enchytraeidae|
Enchytraeus|[2848]|RRBFA434-15|537[0n]bp|Haplotaxida|Enchytraeidae|BOLD:AAU1183
Dendrobaena|[2849]|RRINV1345-15|658[0n]bp|Haplotaxida|Lumbricidae|
Dendrodrilus|[2850]|RRINV1341-15|658[1n]bp|Haplotaxida|Lumbricidae|
Octolasion|[2851]|RRINV1331-15|621[0n]bp|Haplotaxida|Lumbricidae|
Lumbricus terrestris|[2852]|RRINV1349-15|610[0n]bp|Haplotaxida|Lumbricidae|
Lumbricus|[2853]|RRINV1347-15|658[0n]bp|Haplotaxida|Lumbricidae|
Lumbricus|[2854]|RRINV1348-15|658[0n]bp|Haplotaxida|Lumbricidae|
Aporrectodea|[2855]|RRINV1343-15|658[0n]bp|Haplotaxida|Lumbricidae|
Ophyiulus pilosus|[2856]|RRINV112-15|606[0n]bp|Julida|Julidae|
Julus scandinavius|[2857]|RRBFA436-15|576[0n]bp|Julida|Julidae|BOLD:AAH7469
Cylindroiulus caeruleocinctus|[2858]|RRBAA046-15|573[0n]bp|Julida|Julidae|BOLD:AAH7472
Brachyiulus pusillus|[2859]|RRSSA2188-15|558[0n]bp|Julida|Julidae|BOLD:AAM7944
Julidae|[2860]|RRINV1335-15|617[0n]bp|Julida|Julidae|
Julidae|[2861]|RRBAA047-15|576[1n]bp|Julida|Julidae|BOLD:AAZ5766
Lithobius|[2862]|RRINV1339-15|628[0n]bp|Lithobiomorpha|Lithobiidae|
Lithobius|[2863]|RRINV1354-15|658[0n]bp|Lithobiomorpha|Lithobiidae|
Lithobius microps|[2864]|RRBPA032-15|600[0n]bp|Lithobiomorpha|Lithobiidae|BOLD:AAH6432
Lithobius microps|[2865]|RRSSA3537-15|591[0n]bp|Lithobiomorpha|Lithobiidae|BOLD:AAM7904
Phalangium opilio|[2866]|RRSPI055-15|391[0n]bp|Opiliones|Phalangiidae|BOLD:AAI4346
Oligolophus tridens|[2867]|RRSSA2185-15|588[0n]bp|Opiliones|Phalangiidae|BOLD:AAM8194
Platybunus triangularis|[2868]|RRSPI081-15|586[3n]bp|Opiliones|Phalangiidae|BOLD:ABW0506
Caddo|[2869]|RRSSA3524-15|615[2n]bp|Opiliones|Caddidae|BOLD:ACV9424
Leiobunum ventricosum|[2870]|RRINV1895-15|633[0n]bp|Opiliones|Sclerosomatidae|
Leiobunum|[2871]|RRSPI375-15|632[0n]bp|Opiliones|Sclerosomatidae|BOLD:ACV5874
Leiobunum aldrichi|[2872]|RRSSA1034-15|561[0n]bp|Opiliones|Sclerosomatidae|BOLD:AAH7061
Leiobunum|[2873]|RRSSA1040-15|609[4n]bp|Opiliones|Sclerosomatidae|BOLD:AAH7062
Leiobunum|[2874]|RRSSA1037-15|591[0n]bp|Opiliones|Sclerosomatidae|BOLD:ACL7580
Schendyla nemorensis|[2875]|RRSSA336-15|576[0n]bp|Geophilomorpha|Schendylidae|BOLD:AAG8560
Schendylidae|[2876]|RRINV1395-15|636[0n]bp|Geophilomorpha|Schendylidae|
Schendylidae|[2877]|RRINV108-15|426[1n]bp|Geophilomorpha|Schendylidae|
Schendylidae|[2878]|RRINV107-15|620[8n]bp|Geophilomorpha|Schendylidae|
Cypridopsis|[2879]|RRINV2496-15|658[0n]bp|Podocopida|Cyprididae|
Cypria|[2880]|RRINV2497-15|650[0n]bp|Podocopida|Candonidae|
Odontellidae|[2881]|RRSSA3016-15|570[0n]bp|Poduromorpha|Odontellidae|BOLD:AAC1432
Protaphorura|[2882]|RRSSA3548-15|582[0n]bp|Poduromorpha|Onychiuridae|BOLD:AAB0246
Tullbergiidae|[2883]|RRBFA430-15|582[0n]bp|Poduromorpha|Tullbergiidae|BOLD:AAG0680
Poduromorpha|[2884]|RRSSA3010-15|561[0n]bp|Poduromorpha||BOLD:ACC7275
Neanuridae|[2885]|RRBAA436-15|591[0n]bp|Poduromorpha|Neanuridae|BOLD:AAB7286
Poduromorpha|[2886]|RRSSA1495-15|591[0n]bp|Poduromorpha||BOLD:ACV5680
Poduromorpha|[2887]|RRSSA1387-15|585[2n]bp|Poduromorpha||BOLD:ACF1937
Poduromorpha|[2888]|RRSSA3013-15|543[0n]bp|Poduromorpha||BOLD:ACV6302
Poduromorpha|[2889]|RRSSA3011-15|567[0n]bp|Poduromorpha||BOLD:ACV7152
Dicyrtomina minuta|[2890]|RRINV827-15|631[0n]bp|Symphypleona|Dicyrtomidae|
Deuterosminthurus|[2891]|RRSSA2921-15|576[0n]bp|Symphypleona|Bourletiellidae|BOLD:AAB7915
Bourletiella|[2892]|RRMFC2370-15|591[0n]bp|Symphypleona|Bourletiellidae|BOLD:ABX0027
Bourletiella|[2893]|RRSSA112-15|567[0n]bp|Symphypleona|Bourletiellidae|BOLD:ACN4506
Bourletiellidae|[2894]|RRSSA1419-15|564[0n]bp|Symphypleona|Bourletiellidae|BOLD:AAB7913
Bourletiellidae|[2895]|RRMFG2994-15|573[0n]bp|Symphypleona|Bourletiellidae|BOLD:AAZ2180
Bourletiellidae|[2896]|RRSSA1458-15|582[0n]bp|Symphypleona|Bourletiellidae|BOLD:ACJ7454
Bourletiella|[2897]|RRSSA1402-15|546[0n]bp|Symphypleona|Bourletiellidae|BOLD:AAB7914
Bourletiella|[2898]|RRSSA1445-15|531[1n]bp|Symphypleona|Bourletiellidae|BOLD:ACC0359
Bourletiella|[2899]|RRSSA1431-15|573[0n]bp|Symphypleona|Bourletiellidae|BOLD:ACV5610
Symphypleona|[2900]|RRSSA1391-15|525[0n]bp|Symphypleona||BOLD:ABA5370
Symphypleona|[2901]|RRBFA442-15|594[0n]bp|Symphypleona||BOLD:ACV4618
Sminthurinus elegans|[2902]|RRBPA029-15|588[0n]bp|Symphypleona|Katiannidae|BOLD:AAB3496
Sminthurinus elegans|[2903]|RRSSA2941-15|576[0n]bp|Symphypleona|Katiannidae|BOLD:AAB3495
Symphypleona|[2904]|RRSSA2940-15|591[0n]bp|Symphypleona||BOLD:ACJ9068
Sminthurididae|[2905]|RRSSA2869-15|606[0n]bp|Symphypleona|Sminthurididae|BOLD:AAI4706
Symphypleona|[2906]|RRBPA042-15|600[0n]bp|Symphypleona||BOLD:ACV5788
Symphypleona|[2907]|RRBAA254-15|585[0n]bp|Symphypleona||BOLD:ACV4139
Sminthurides|[2908]|RRBAA071-15|573[0n]bp|Symphypleona|Sminthurididae|BOLD:AAG3938
Symphypleona|[2909]|RRBGA118-15|505[1n]bp|Symphypleona||BOLD:ACV4567
Symphypleona|[2910]|RRBGA088-15|591[0n]bp|Symphypleona||BOLD:ACV4365
Symphypleona|[2911]|RRBAA440-15|552[0n]bp|Symphypleona||BOLD:ACV5320
Symphypleona|[2912]|RRBGA142-15|458[0n]bp|Symphypleona||
Symphypleona|[2913]|RRBGA085-15|534[1n]bp|Symphypleona||BOLD:ACV4528
Symphypleona|[2914]|RRBGA089-15|585[0n]bp|Symphypleona||BOLD:ACV4522
Symphypleona|[2915]|RRBGA136-15|409[0n]bp|Symphypleona||
Isotomidae|[2916]|RRBFA467-15|582[0n]bp|Entomobryomorpha|Isotomidae|BOLD:ACV5561
Parisotoma|[2917]|RRINV172-15|631[0n]bp|Entomobryomorpha|Isotomidae|
Parisotoma|[2918]|RRBAA468-15|546[0n]bp|Entomobryomorpha|Isotomidae|BOLD:AAB2869
Parisotoma notabilis|[2919]|RRBAA220-15|354[6n]bp|Entomobryomorpha|Isotomidae|
Parisotoma notabilis|[2920]|RRBAA247-15|499[1n]bp|Entomobryomorpha|Isotomidae|BOLD:AAB2870
Parisotoma notabilis|[2921]|RRBAA487-15|540[5n]bp|Entomobryomorpha|Isotomidae|BOLD:ABZ8106
Parisotoma|[2922]|RRBAA086-15|552[0n]bp|Entomobryomorpha|Isotomidae|BOLD:AAA4157
Isotomidae|[2923]|RRBFA456-15|570[0n]bp|Entomobryomorpha|Isotomidae|BOLD:ACV4617
Isotomidae|[2924]|RRBFA461-15|588[0n]bp|Entomobryomorpha|Isotomidae|BOLD:AAB2085
Isotomidae|[2925]|RRBFA451-15|429[0n]bp|Entomobryomorpha|Isotomidae|
Orchesella villosa|[2926]|RRBAA482-15|576[0n]bp|Entomobryomorpha|Entomobryidae|BOLD:AAA8726
Entomobryidae|[2927]|RRBAA403-15|588[0n]bp|Entomobryomorpha|Entomobryidae|BOLD:ABA5351
Lepidocyrtus paradoxus|[2928]|RRINV1066-15|630[0n]bp|Entomobryomorpha|Entomobryidae|
Entomobrya atrocincta|[2929]|RRMFB1422-15|585[0n]bp|Entomobryomorpha|Entomobryidae|BOLD:ACE5102
Lepidocyrtus|[2930]|RRBAA430-15|588[0n]bp|Entomobryomorpha|Entomobryidae|BOLD:ACI2943
Entomobrya nivalis|[2931]|RRMFB1440-15|588[0n]bp|Entomobryomorpha|Entomobryidae|BOLD:ACL6239
Tomocerus|[2932]|RRSSA2917-15|552[0n]bp|Entomobryomorpha|Tomoceridae|BOLD:AAA7969
Entomobryomorpha|[2933]|RRSSA2175-15|588[0n]bp|Entomobryomorpha||BOLD:ABA5361
Lepidocyrtus|[2934]|RRMFB1429-15|597[0n]bp|Entomobryomorpha|Entomobryidae|BOLD:ACM2009
Lepidocyrtus|[2935]|RRSSA1343-15|579[19n]bp|Entomobryomorpha|Entomobryidae|
Poduromorpha|[2936]|RRBAA002-15|519[0n]bp|Poduromorpha||BOLD:ACV4568
Poduromorpha|[2937]|RRSSA2871-15|573[0n]bp|Poduromorpha||BOLD:ACL3025
Odontellidae|[2938]|RRSSA2838-15|591[0n]bp|Poduromorpha|Odontellidae|BOLD:ACV7054
Isotomidae|[2939]|RRSSA2843-15|588[0n]bp|Entomobryomorpha|Isotomidae|BOLD:AAH7174
Hypogastruridae|[2940]|RRSSA1358-15|582[0n]bp|Poduromorpha|Hypogastruridae|BOLD:AAA4811
Ceratophysella bengtssoni|[2941]|RRSSA2999-15|621[1n]bp|Poduromorpha|Hypogastruridae|BOLD:AAI3738

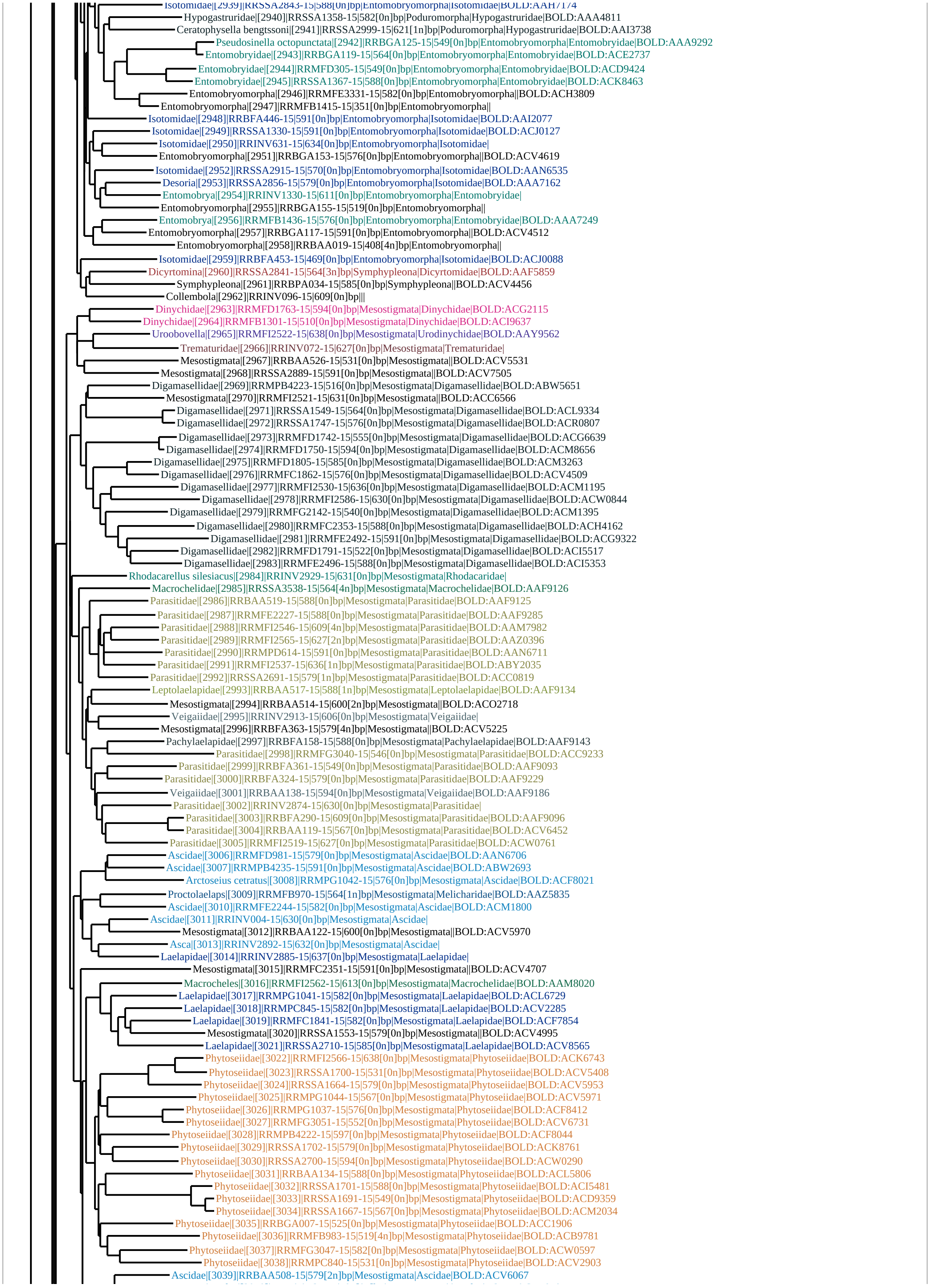
Isotomidae|[2939]|RRSSA2843-15|588[0n]bp|Entomobryomorpha|Isotomidae|BOLD:AAH7174
Hypogastruridae|[2940]|RRSSA1358-15|582[0n]bp|Poduromorpha|Hypogastruridae|BOLD:AAA4811
Ceratophysella bengtssoni|[2941]|RRSSA2999-15|621[1n]bp|Poduromorpha|Hypogastruridae|BOLD:AAI3738
Pseudosinella octopunctata|[2942]|RRBGA125-15|549[0n]bp|Entomobryomorpha|Entomobryidae|BOLD:AAA9292
Entomobryidae|[2943]|RRBGA119-15|564[0n]bp|Entomobryomorpha|Entomobryidae|BOLD:ACE2737
Entomobryidae|[2944]|RRMFD305-15|549[0n]bp|Entomobryomorpha|Entomobryidae|BOLD:ACD9424
Entomobryidae|[2945]|RRSSA1367-15|588[0n]bp|Entomobryomorpha|Entomobryidae|BOLD:ACK8463
Entomobryomorpha|[2946]|RRMFE3331-15|582[0n]bp|Entomobryomorpha||BOLD:ACH3809
Entomobryomorpha|[2947]|RRMFB1415-15|351[0n]bp|Entomobryomorpha||
Isotomidae|[2948]|RRBFA446-15|591[0n]bp|Entomobryomorpha|Isotomidae|BOLD:AAI2077
Isotomidae|[2949]|RRSSA1330-15|591[0n]bp|Entomobryomorpha|Isotomidae|BOLD:ACJ0127
Isotomidae|[2950]|RRINV631-15|634[0n]bp|Entomobryomorpha|Isotomidae|
Entomobryomorpha|[2951]|RRBGA153-15|576[0n]bp|Entomobryomorpha||BOLD:ACV4619
Isotomidae|[2952]|RRSSA2915-15|570[0n]bp|Entomobryomorpha|Isotomidae|BOLD:AAN6535
Desoria|[2953]|RRSSA2856-15|579[0n]bp|Entomobryomorpha|Isotomidae|BOLD:AAA7162
Entomobrya|[2954]|RRINV1330-15|611[0n]bp|Entomobryomorpha|Entomobryidae|
Entomobryomorpha|[2955]|RRBGA155-15|519[0n]bp|Entomobryomorpha||
Entomobrya|[2956]|RRMFB1436-15|576[0n]bp|Entomobryomorpha|Entomobryidae|BOLD:AAA7249
Entomobryomorpha|[2957]|RRBGA117-15|591[0n]bp|Entomobryomorpha||BOLD:ACV4512
Entomobryomorpha|[2958]|RRBAA019-15|408[4n]bp|Entomobryomorpha||
Isotomidae|[2959]|RRBFA453-15|469[0n]bp|Entomobryomorpha|Isotomidae|BOLD:ACJ0088
Dicyrtomina|[2960]|RRSSA2841-15|564[3n]bp|Symphypleona|Dicyrtomidae|BOLD:AAF5859
Symphypleona|[2961]|RRBPA034-15|585[0n]bp|Symphypleona||BOLD:ACV4456
Collembola|[2962]|RRINV096-15|609[0n]bp|||
Dinychidae|[2963]|RRMFD1763-15|594[0n]bp|Mesostigmata|Dinychidae|BOLD:ACG2115
Dinychidae|[2964]|RRMFB1301-15|510[0n]bp|Mesostigmata|Dinychidae|BOLD:ACI9637
Uroobovella|[2965]|RRMFI2522-15|638[0n]bp|Mesostigmata|Urodinychidae|BOLD:AAY9562
Trematuridae|[2966]|RRINV072-15|627[0n]bp|Mesostigmata|Trematuridae|
Mesostigmata|[2967]|RRBAA526-15|531[0n]bp|Mesostigmata||BOLD:ACV5531
Mesostigmata|[2968]|RRSSA2889-15|591[0n]bp|Mesostigmata||BOLD:ACV7505
Digamasellidae|[2969]|RRMPB4223-15|516[0n]bp|Mesostigmata|Digamasellidae|BOLD:ABW5651
Mesostigmata|[2970]|RRMFI2521-15|631[0n]bp|Mesostigmata||BOLD:ACC6566
Digamasellidae|[2971]|RRSSA1549-15|564[0n]bp|Mesostigmata|Digamasellidae|BOLD:ACL9334
Digamasellidae|[2972]|RRSSA1747-15|576[0n]bp|Mesostigmata|Digamasellidae|BOLD:ACR0807
Digamasellidae|[2973]|RRMFD1742-15|555[0n]bp|Mesostigmata|Digamasellidae|BOLD:ACG6639
Digamasellidae|[2974]|RRMFD1750-15|594[0n]bp|Mesostigmata|Digamasellidae|BOLD:ACM8656
Digamasellidae|[2975]|RRMFD1805-15|585[0n]bp|Mesostigmata|Digamasellidae|BOLD:ACM3263
Digamasellidae|[2976]|RRMFC1862-15|576[0n]bp|Mesostigmata|Digamasellidae|BOLD:ACV4509
Digamasellidae|[2977]|RRMFI2530-15|636[0n]bp|Mesostigmata|Digamasellidae|BOLD:ACM1195
Digamasellidae|[2978]|RRMFI2586-15|630[0n]bp|Mesostigmata|Digamasellidae|BOLD:ACW0844
Digamasellidae|[2979]|RRMFG2142-15|540[0n]bp|Mesostigmata|Digamasellidae|BOLD:ACM1395
Digamasellidae|[2980]|RRMFC2353-15|588[0n]bp|Mesostigmata|Digamasellidae|BOLD:ACH4162
Digamasellidae|[2981]|RRMFE2492-15|591[0n]bp|Mesostigmata|Digamasellidae|BOLD:ACG9322
Digamasellidae|[2982]|RRMFD1791-15|522[0n]bp|Mesostigmata|Digamasellidae|BOLD:ACI5517
Digamasellidae|[2983]|RRMFE2496-15|588[0n]bp|Mesostigmata|Digamasellidae|BOLD:ACI5353
Rhodacarellus silesiacus|[2984]|RRINV2929-15|631[0n]bp|Mesostigmata|Rhodacaridae|
Macrochelidae|[2985]|RRSSA3538-15|564[4n]bp|Mesostigmata|Macrochelidae|BOLD:AAF9126
Parasitidae|[2986]|RRBAA519-15|588[0n]bp|Mesostigmata|Parasitidae|BOLD:AAF9125
Parasitidae|[2987]|RRMFE2227-15|588[0n]bp|Mesostigmata|Parasitidae|BOLD:AAF9285
Parasitidae|[2988]|RRMFI2546-15|609[4n]bp|Mesostigmata|Parasitidae|BOLD:AAM7982
Parasitidae|[2989]|RRMFI2565-15|627[2n]bp|Mesostigmata|Parasitidae|BOLD:AAZ0396
Parasitidae|[2990]|RRMPD614-15|591[0n]bp|Mesostigmata|Parasitidae|BOLD:AAN6711
Parasitidae|[2991]|RRMFI2537-15|636[1n]bp|Mesostigmata|Parasitidae|BOLD:ABY2035
Parasitidae|[2992]|RRSSA2691-15|579[1n]bp|Mesostigmata|Parasitidae|BOLD:ACC0819
Leptolaelapidae|[2993]|RRBAA517-15|588[1n]bp|Mesostigmata|Leptolaelapidae|BOLD:AAF9134
Mesostigmata|[2994]|RRBAA514-15|600[2n]bp|Mesostigmata||BOLD:ACO2718
Veigaiidae|[2995]|RRINV2913-15|606[0n]bp|Mesostigmata|Veigaiidae|
Mesostigmata|[2996]|RRBFA363-15|579[4n]bp|Mesostigmata||BOLD:ACV5225
Pachylaelapidae|[2997]|RRBFA158-15|588[0n]bp|Mesostigmata|Pachylaelapidae|BOLD:AAF9143
Parasitidae|[2998]|RRMFG3040-15|546[0n]bp|Mesostigmata|Parasitidae|BOLD:ACC9233
Parasitidae|[2999]|RRBFA361-15|549[0n]bp|Mesostigmata|Parasitidae|BOLD:AAF9093
Parasitidae|[3000]|RRBFA324-15|579[0n]bp|Mesostigmata|Parasitidae|BOLD:AAF9229
Veigaiidae|[3001]|RRBAA138-15|594[0n]bp|Mesostigmata|Veigaiidae|BOLD:AAF9186
Parasitidae|[3002]|RRINV2874-15|630[0n]bp|Mesostigmata|Parasitidae|
Parasitidae|[3003]|RRBFA290-15|609[0n]bp|Mesostigmata|Parasitidae|BOLD:AAF9096
Parasitidae|[3004]|RRBAA119-15|567[0n]bp|Mesostigmata|Parasitidae|BOLD:ACV6452
Parasitidae|[3005]|RRMFI2519-15|627[0n]bp|Mesostigmata|Parasitidae|BOLD:ACW0761
Ascidae|[3006]|RRMFD981-15|579[0n]bp|Mesostigmata|Ascidae|BOLD:AAN6706
Ascidae|[3007]|RRMPB4235-15|591[0n]bp|Mesostigmata|Ascidae|BOLD:ABW2693
Arctoseius cetratus|[3008]|RRMPG1042-15|576[0n]bp|Mesostigmata|Ascidae|BOLD:ACF8021
Proctolaelaps|[3009]|RRMFB970-15|564[1n]bp|Mesostigmata|Melicharidae|BOLD:AAZ5835
Ascidae|[3010]|RRMFE2244-15|582[0n]bp|Mesostigmata|Ascidae|BOLD:ACM1800
Ascidae|[3011]|RRINV004-15|630[0n]bp|Mesostigmata|Ascidae|
Mesostigmata|[3012]|RRBAA122-15|600[0n]bp|Mesostigmata||BOLD:ACV5970
Asca|[3013]|RRINV2892-15|632[0n]bp|Mesostigmata|Ascidae|
Laelapidae|[3014]|RRINV2885-15|637[0n]bp|Mesostigmata|Laelapidae|
Mesostigmata|[3015]|RRMFC2351-15|591[0n]bp|Mesostigmata||BOLD:ACV4707
Macrocheles|[3016]|RRMFI2562-15|613[0n]bp|Mesostigmata|Macrochelidae|BOLD:AAM8020
Laelapidae|[3017]|RRMPG1041-15|582[0n]bp|Mesostigmata|Laelapidae|BOLD:ACL6729
Laelapidae|[3018]|RRMPC845-15|582[0n]bp|Mesostigmata|Laelapidae|BOLD:ACV2285
Laelapidae|[3019]|RRMFC1841-15|582[0n]bp|Mesostigmata|Laelapidae|BOLD:ACF7854
Mesostigmata|[3020]|RRSSA1553-15|579[0n]bp|Mesostigmata||BOLD:ACV4995
Laelapidae|[3021]|RRSSA2710-15|585[0n]bp|Mesostigmata|Laelapidae|BOLD:ACV8565
Phytoseiidae|[3022]|RRMFI2566-15|638[0n]bp|Mesostigmata|Phytoseiidae|BOLD:ACK6743
Phytoseiidae|[3023]|RRSSA1700-15|531[0n]bp|Mesostigmata|Phytoseiidae|BOLD:ACV5408
Phytoseiidae|[3024]|RRSSA1664-15|579[0n]bp|Mesostigmata|Phytoseiidae|BOLD:ACV5953
Phytoseiidae|[3025]|RRMPG1044-15|567[0n]bp|Mesostigmata|Phytoseiidae|BOLD:ACV5971
Phytoseiidae|[3026]|RRMPG1037-15|576[0n]bp|Mesostigmata|Phytoseiidae|BOLD:ACF8412
Phytoseiidae|[3027]|RRMFG3051-15|552[0n]bp|Mesostigmata|Phytoseiidae|BOLD:ACV6731
Phytoseiidae|[3028]|RRMPB4222-15|597[0n]bp|Mesostigmata|Phytoseiidae|BOLD:ACF8044
Phytoseiidae|[3029]|RRSSA1702-15|579[0n]bp|Mesostigmata|Phytoseiidae|BOLD:ACK8761
Phytoseiidae|[3030]|RRSSA2700-15|594[0n]bp|Mesostigmata|Phytoseiidae|BOLD:ACW0290
Phytoseiidae|[3031]|RRBAA134-15|588[0n]bp|Mesostigmata|Phytoseiidae|BOLD:ACL5806
Phytoseiidae|[3032]|RRSSA1701-15|588[0n]bp|Mesostigmata|Phytoseiidae|BOLD:ACI5481
Phytoseiidae|[3033]|RRSSA1691-15|549[0n]bp|Mesostigmata|Phytoseiidae|BOLD:ACD9359
Phytoseiidae|[3034]|RRSSA1667-15|567[0n]bp|Mesostigmata|Phytoseiidae|BOLD:ACM2034
Phytoseiidae|[3035]|RRBGA007-15|525[0n]bp|Mesostigmata|Phytoseiidae|BOLD:ACC1906
Phytoseiidae|[3036]|RRMFB983-15|519[4n]bp|Mesostigmata|Phytoseiidae|BOLD:ACB9781
Phytoseiidae|[3037]|RRMFG3047-15|582[0n]bp|Mesostigmata|Phytoseiidae|BOLD:ACW0597
Phytoseiidae|[3038]|RRMPC840-15|531[0n]bp|Mesostigmata|Phytoseiidae|BOLD:ACV2903
Ascidae|[3039]|RRBAA508-15|579[2n]bp|Mesostigmata|Ascidae|BOLD:ACV6067

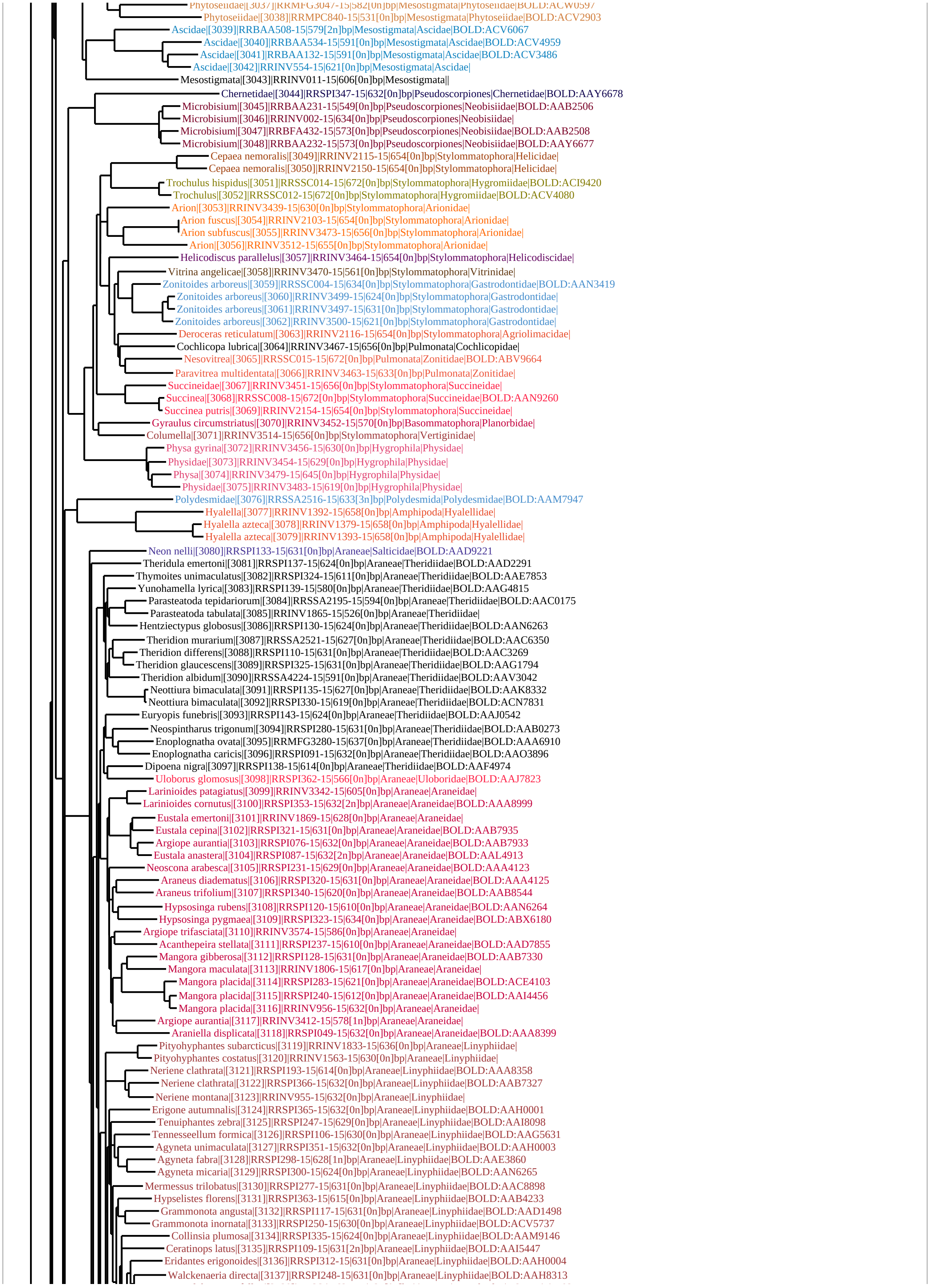
Phytoseiidae|[3037]|RRMFG3047-15|582[0n]bp|Mesostigmata|Phytoseiidae|BOLD:ACW0597
Phytoseiidae|[3038]|RRMPC840-15|531[0n]bp|Mesostigmata|Phytoseiidae|BOLD:ACV2903
Ascidae|[3039]|RRBAA508-15|579[2n]bp|Mesostigmata|Ascidae|BOLD:ACV6067
Ascidae|[3040]|RRBAA534-15|591[0n]bp|Mesostigmata|Ascidae|BOLD:ACV4959
Ascidae|[3041]|RRBAA132-15|591[0n]bp|Mesostigmata|Ascidae|BOLD:ACV3486
Ascidae|[3042]|RRINV554-15|621[0n]bp|Mesostigmata|Ascidae|
Mesostigmata|[3043]|RRINV011-15|606[0n]bp|Mesostigmata||
Chernetidae|[3044]|RRSPI347-15|632[0n]bp|Pseudoscorpiones|Chernetidae|BOLD:AAY6678
Microbisium|[3045]|RRBAA231-15|549[0n]bp|Pseudoscorpiones|Neobisiidae|BOLD:AAB2506
Microbisium|[3046]|RRINV002-15|634[0n]bp|Pseudoscorpiones|Neobisiidae|
Microbisium|[3047]|RRBFA432-15|573[0n]bp|Pseudoscorpiones|Neobisiidae|BOLD:AAB2508
Microbisium|[3048]|RRBAA232-15|573[0n]bp|Pseudoscorpiones|Neobisiidae|BOLD:AAY6677
Cepaea nemoralis|[3049]|RRINV2115-15|654[0n]bp|Stylommatophora|Helicidae|
Cepaea nemoralis|[3050]|RRINV2150-15|654[0n]bp|Stylommatophora|Helicidae|
Trochulus hispidus|[3051]|RRSSC014-15|672[0n]bp|Stylommatophora|Hygromiidae|BOLD:ACI9420
Trochulus|[3052]|RRSSC012-15|672[0n]bp|Stylommatophora|Hygromiidae|BOLD:ACV4080
Arion|[3053]|RRINV3439-15|630[0n]bp|Stylommatophora|Arionidae|
Arion fuscus|[3054]|RRINV2103-15|654[0n]bp|Stylommatophora|Arionidae|
Arion subfuscus|[3055]|RRINV3473-15|656[0n]bp|Stylommatophora|Arionidae|
Arion|[3056]|RRINV3512-15|655[0n]bp|Stylommatophora|Arionidae|
Helicodiscus parallelus|[3057]|RRINV3464-15|654[0n]bp|Stylommatophora|Helicodiscidae|
Vitrina angelicae|[3058]|RRINV3470-15|561[0n]bp|Stylommatophora|Vitrinidae|
Zonitoides arboreus|[3059]|RRSSC004-15|634[0n]bp|Stylommatophora|Gastrodontidae|BOLD:AAN3419
Zonitoides arboreus|[3060]|RRINV3499-15|624[0n]bp|Stylommatophora|Gastrodontidae|
Zonitoides arboreus|[3061]|RRINV3497-15|631[0n]bp|Stylommatophora|Gastrodontidae|
Zonitoides arboreus|[3062]|RRINV3500-15|621[0n]bp|Stylommatophora|Gastrodontidae|
Deroceras reticulatum|[3063]|RRINV2116-15|654[0n]bp|Stylommatophora|Agriolimacidae|
Cochlicopa lubrica|[3064]|RRINV3467-15|656[0n]bp|Pulmonata|Cochlicopidae|
Nesovitrea|[3065]|RRSSC015-15|672[0n]bp|Pulmonata|Zonitidae|BOLD:ABV9664
Paravitrea multidentata|[3066]|RRINV3463-15|633[0n]bp|Pulmonata|Zonitidae|
Succineidae|[3067]|RRINV3451-15|656[0n]bp|Stylommatophora|Succineidae|
Succinea|[3068]|RRSSC008-15|672[0n]bp|Stylommatophora|Succineidae|BOLD:AAN9260
Succinea putris|[3069]|RRINV2154-15|654[0n]bp|Stylommatophora|Succineidae|
Gyraulus circumstriatus|[3070]|RRINV3452-15|570[0n]bp|Basommatophora|Planorbidae|
Columella|[3071]|RRINV3514-15|656[0n]bp|Stylommatophora|Vertiginidae|
Physa gyrina|[3072]|RRINV3456-15|630[0n]bp|Hygrophila|Physidae|
Physidae|[3073]|RRINV3454-15|629[0n]bp|Hygrophila|Physidae|
Physa|[3074]|RRINV3479-15|645[0n]bp|Hygrophila|Physidae|
Physidae|[3075]|RRINV3483-15|619[0n]bp|Hygrophila|Physidae|
Polydesmidae|[3076]|RRSSA2516-15|633[3n]bp|Polydesmida|Polydesmidae|BOLD:AAM7947
Hyalella|[3077]|RRINV1392-15|658[0n]bp|Amphipoda|Hyalellidae|
Hyalella azteca|[3078]|RRINV1379-15|658[0n]bp|Amphipoda|Hyalellidae|
Hyalella azteca|[3079]|RRINV1393-15|658[0n]bp|Amphipoda|Hyalellidae|
Neon nelli|[3080]|RRSPI133-15|631[0n]bp|Araneae|Salticidae|BOLD:AAD9221
Theridula emertoni|[3081]|RRSPI137-15|624[0n]bp|Araneae|Theridiidae|BOLD:AAD2291
Thymoites unimaculatus|[3082]|RRSPI324-15|611[0n]bp|Araneae|Theridiidae|BOLD:AAE7853
Yunohamella lyrica|[3083]|RRSPI139-15|580[0n]bp|Araneae|Theridiidae|BOLD:AAG4815
Parasteatoda tepidariorum|[3084]|RRSSA2195-15|594[0n]bp|Araneae|Theridiidae|BOLD:AAC0175
Parasteatoda tabulata|[3085]|RRINV1865-15|526[0n]bp|Araneae|Theridiidae|
Hentziectypus globosus|[3086]|RRSPI130-15|624[0n]bp|Araneae|Theridiidae|BOLD:AAN6263
Theridion murarium|[3087]|RRSSA2521-15|627[0n]bp|Araneae|Theridiidae|BOLD:AAC6350
Theridion differens|[3088]|RRSPI110-15|631[0n]bp|Araneae|Theridiidae|BOLD:AAC3269
Theridion glaucescens|[3089]|RRSPI325-15|631[0n]bp|Araneae|Theridiidae|BOLD:AAG1794
Theridion albidum|[3090]|RRSSA4224-15|591[0n]bp|Araneae|Theridiidae|BOLD:AAV3042
Neottiura bimaculata|[3091]|RRSPI135-15|627[0n]bp|Araneae|Theridiidae|BOLD:AAK8332
Neottiura bimaculata|[3092]|RRSPI330-15|619[0n]bp|Araneae|Theridiidae|BOLD:ACN7831
Euryopis funebris|[3093]|RRSPI143-15|624[0n]bp|Araneae|Theridiidae|BOLD:AAJ0542
Neospintharus trigonum|[3094]|RRSPI280-15|631[0n]bp|Araneae|Theridiidae|BOLD:AAB0273
Enoplognatha ovata|[3095]|RRMFG3280-15|637[0n]bp|Araneae|Theridiidae|BOLD:AAA6910
Enoplognatha caricis|[3096]|RRSPI091-15|632[0n]bp|Araneae|Theridiidae|BOLD:AAO3896
Dipoena nigra|[3097]|RRSPI138-15|614[0n]bp|Araneae|Theridiidae|BOLD:AAF4974
Uloborus glomosus|[3098]|RRSPI362-15|566[0n]bp|Araneae|Uloboridae|BOLD:AAJ7823
Larinioides patagiatus|[3099]|RRINV3342-15|605[0n]bp|Araneae|Araneidae|
Larinioides cornutus|[3100]|RRSPI353-15|632[2n]bp|Araneae|Araneidae|BOLD:AAA8999
Eustala emertoni|[3101]|RRINV1869-15|628[0n]bp|Araneae|Araneidae|
Eustala cepina|[3102]|RRSPI321-15|631[0n]bp|Araneae|Araneidae|BOLD:AAB7935
Argiope aurantia|[3103]|RRSPI076-15|632[0n]bp|Araneae|Araneidae|BOLD:AAB7933
Eustala anastera|[3104]|RRSPI087-15|632[2n]bp|Araneae|Araneidae|BOLD:AAL4913
Neoscona arabesca|[3105]|RRSPI231-15|629[0n]bp|Araneae|Araneidae|BOLD:AAA4123
Araneus diadematus|[3106]|RRSPI320-15|631[0n]bp|Araneae|Araneidae|BOLD:AAA4125
Araneus trifolium|[3107]|RRSPI340-15|620[0n]bp|Araneae|Araneidae|BOLD:AAB8544
Hypsosinga rubens|[3108]|RRSPI120-15|610[0n]bp|Araneae|Araneidae|BOLD:AAN6264
Hypsosinga pygmaea|[3109]|RRSPI323-15|634[0n]bp|Araneae|Araneidae|BOLD:ABX6180
Argiope trifasciata|[3110]|RRINV3574-15|586[0n]bp|Araneae|Araneidae|
Acanthepeira stellata|[3111]|RRSPI237-15|610[0n]bp|Araneae|Araneidae|BOLD:AAD7855
Mangora gibberosa|[3112]|RRSPI128-15|631[0n]bp|Araneae|Araneidae|BOLD:AAB7330
Mangora maculata|[3113]|RRINV1806-15|617[0n]bp|Araneae|Araneidae|
Mangora placida|[3114]|RRSPI283-15|621[0n]bp|Araneae|Araneidae|BOLD:ACE4103
Mangora placida|[3115]|RRSPI240-15|612[0n]bp|Araneae|Araneidae|BOLD:AAI4456
Mangora placida|[3116]|RRINV956-15|632[0n]bp|Araneae|Araneidae|
Argiope aurantia|[3117]|RRINV3412-15|578[1n]bp|Araneae|Araneidae|
Araniella displicata|[3118]|RRSPI049-15|632[0n]bp|Araneae|Araneidae|BOLD:AAA8399
Pityohyphantes subarcticus|[3119]|RRINV1833-15|636[0n]bp|Araneae|Linyphiidae|
Pityohyphantes costatus|[3120]|RRINV1563-15|630[0n]bp|Araneae|Linyphiidae|
Neriene clathrata|[3121]|RRSPI193-15|614[0n]bp|Araneae|Linyphiidae|BOLD:AAA8358
Neriene clathrata|[3122]|RRSPI366-15|632[0n]bp|Araneae|Linyphiidae|BOLD:AAB7327
Neriene montana|[3123]|RRINV955-15|632[0n]bp|Araneae|Linyphiidae|
Erigone autumnalis|[3124]|RRSPI365-15|632[0n]bp|Araneae|Linyphiidae|BOLD:AAH0001
Tenuiphantes zebra|[3125]|RRSPI247-15|629[0n]bp|Araneae|Linyphiidae|BOLD:AAI8098
Tennesseellum formica|[3126]|RRSPI106-15|630[0n]bp|Araneae|Linyphiidae|BOLD:AAG5631
Agyneta unimaculata|[3127]|RRSPI351-15|632[0n]bp|Araneae|Linyphiidae|BOLD:AAH0003
Agyneta fabra|[3128]|RRSPI298-15|628[1n]bp|Araneae|Linyphiidae|BOLD:AAE3860
Agyneta micaria|[3129]|RRSPI300-15|624[0n]bp|Araneae|Linyphiidae|BOLD:AAN6265
Mermessus trilobatus|[3130]|RRSPI277-15|631[0n]bp|Araneae|Linyphiidae|BOLD:AAC8898
Hypselistes florens|[3131]|RRSPI363-15|615[0n]bp|Araneae|Linyphiidae|BOLD:AAB4233
Grammonota angusta|[3132]|RRSPI117-15|631[0n]bp|Araneae|Linyphiidae|BOLD:AAD1498
Grammonota inornata|[3133]|RRSPI250-15|630[0n]bp|Araneae|Linyphiidae|BOLD:ACV5737
Collinsia plumosa|[3134]|RRSPI335-15|624[0n]bp|Araneae|Linyphiidae|BOLD:AAM9146
Ceratinops latus|[3135]|RRSPI109-15|631[2n]bp|Araneae|Linyphiidae|BOLD:AAI5447
Eridantes erigonoides|[3136]|RRSPI312-15|631[0n]bp|Araneae|Linyphiidae|BOLD:AAH0004
Walckenaeria directa|[3137]|RRSPI248-15|631[0n]bp|Araneae|Linyphiidae|BOLD:AAH8313

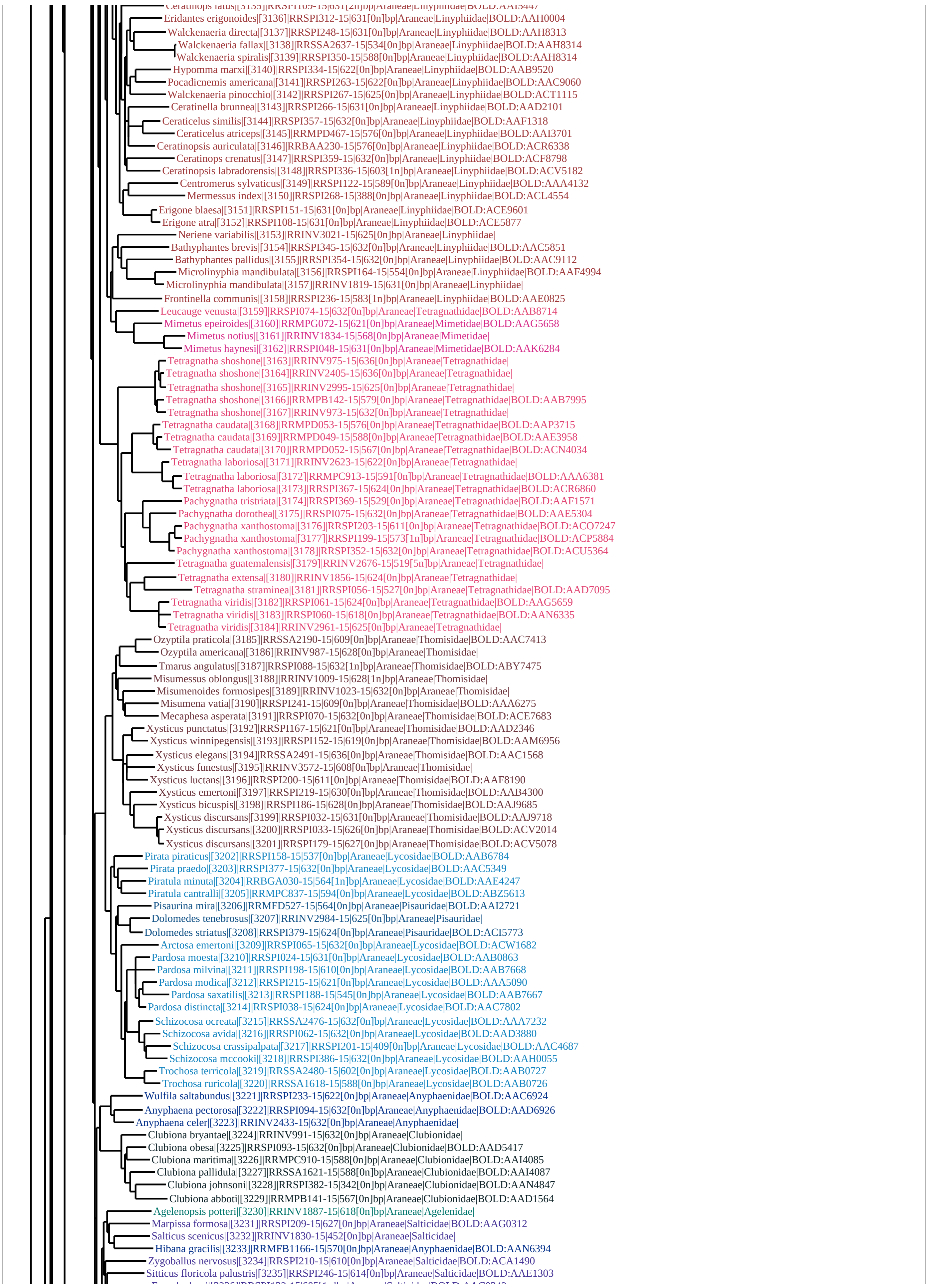

Eridantes erigonoides|[3136]|RRSPI312-15|631[0n]bp|Araneae|Linyphiidae|BOLD:AAH0004
Walckenaeria directa|[3137]|RRSPI248-15|631[0n]bp|Araneae|Linyphiidae|BOLD:AAH8313
Walckenaeria fallax|[3138]|RRSSA2637-15|534[0n]bp|Araneae|Linyphiidae|BOLD:AAH8314
Walckenaeria spiralis|[3139]|RRSPI350-15|588[0n]bp|Araneae|Linyphiidae|BOLD:AAH8314
Hypomma marxi|[3140]|RRSPI334-15|622[0n]bp|Araneae|Linyphiidae|BOLD:AAB9520
Pocadicnemis americana|[3141]|RRSPI263-15|622[0n]bp|Araneae|Linyphiidae|BOLD:AAC9060
Walckenaeria pinocchio|[3142]|RRSPI267-15|625[0n]bp|Araneae|Linyphiidae|BOLD:ACT1115
Ceratinella brunnea|[3143]|RRSPI266-15|631[0n]bp|Araneae|Linyphiidae|BOLD:AAD2101
Ceraticelus similis|[3144]|RRSPI357-15|632[0n]bp|Araneae|Linyphiidae|BOLD:AAF1318
Ceraticelus atriceps|[3145]|RRMPD467-15|576[0n]bp|Araneae|Linyphiidae|BOLD:AAI3701
Ceratinopsis auriculata|[3146]|RRBAA230-15|576[0n]bp|Araneae|Linyphiidae|BOLD:ACR6338
Ceratinops crenatus|[3147]|RRSPI359-15|632[0n]bp|Araneae|Linyphiidae|BOLD:ACF8798
Ceratinopsis labradorensis|[3148]|RRSPI336-15|603[1n]bp|Araneae|Linyphiidae|BOLD:ACV5182
Centromerus sylvaticus|[3149]|RRSPI122-15|589[0n]bp|Araneae|Linyphiidae|BOLD:AAA4132
Mermessus index|[3150]|RRSPI268-15|388[0n]bp|Araneae|Linyphiidae|BOLD:ACL4554
Erigone blaesa|[3151]|RRSPI151-15|631[0n]bp|Araneae|Linyphiidae|BOLD:ACE9601
Erigone atra|[3152]|RRSPI108-15|631[0n]bp|Araneae|Linyphiidae|BOLD:ACE5877
Neriene variabilis|[3153]|RRINV3021-15|625[0n]bp|Araneae|Linyphiidae|
Bathyphantes brevis|[3154]|RRSPI345-15|632[0n]bp|Araneae|Linyphiidae|BOLD:AAC5851
Bathyphantes pallidus|[3155]|RRSPI354-15|632[0n]bp|Araneae|Linyphiidae|BOLD:AAC9112
Microlinyphia mandibulata|[3156]|RRSPI164-15|554[0n]bp|Araneae|Linyphiidae|BOLD:AAF4994
Microlinyphia mandibulata|[3157]|RRINV1819-15|631[0n]bp|Araneae|Linyphiidae|
Frontinella communis|[3158]|RRSPI236-15|583[1n]bp|Araneae|Linyphiidae|BOLD:AAE0825
Leucauge venusta|[3159]|RRSPI074-15|632[0n]bp|Araneae|Tetragnathidae|BOLD:AAB8714
Mimetus epeiroides|[3160]|RRMPG072-15|621[0n]bp|Araneae|Mimetidae|BOLD:AAG5658
Mimetus notius|[3161]|RRINV1834-15|568[0n]bp|Araneae|Mimetidae|
Mimetus haynesi|[3162]|RRSPI048-15|631[0n]bp|Araneae|Mimetidae|BOLD:AAK6284
Tetragnatha shoshone|[3163]|RRINV975-15|636[0n]bp|Araneae|Tetragnathidae|
Tetragnatha shoshone|[3164]|RRINV2405-15|636[0n]bp|Araneae|Tetragnathidae|
Tetragnatha shoshone|[3165]|RRINV2995-15|625[0n]bp|Araneae|Tetragnathidae|
Tetragnatha shoshone|[3166]|RRMPB142-15|579[0n]bp|Araneae|Tetragnathidae|BOLD:AAB7995
Tetragnatha shoshone|[3167]|RRINV973-15|632[0n]bp|Araneae|Tetragnathidae|
Tetragnatha caudata|[3168]|RRMPD053-15|576[0n]bp|Araneae|Tetragnathidae|BOLD:AAP3715
Tetragnatha caudata|[3169]|RRMPD049-15|588[0n]bp|Araneae|Tetragnathidae|BOLD:AAE3958
Tetragnatha caudata|[3170]|RRMPD052-15|567[0n]bp|Araneae|Tetragnathidae|BOLD:ACN4034
Tetragnatha laboriosa|[3171]|RRINV2623-15|622[0n]bp|Araneae|Tetragnathidae|
Tetragnatha laboriosa|[3172]|RRMPC913-15|591[0n]bp|Araneae|Tetragnathidae|BOLD:AAA6381
Tetragnatha laboriosa|[3173]|RRSPI367-15|624[0n]bp|Araneae|Tetragnathidae|BOLD:ACR6860
Pachygnatha tristriata|[3174]|RRSPI369-15|529[0n]bp|Araneae|Tetragnathidae|BOLD:AAF1571
Pachygnatha dorothea|[3175]|RRSPI075-15|632[0n]bp|Araneae|Tetragnathidae|BOLD:AAE5304
Pachygnatha xanthostoma|[3176]|RRSPI203-15|611[0n]bp|Araneae|Tetragnathidae|BOLD:ACO7247
Pachygnatha xanthostoma|[3177]|RRSPI199-15|573[1n]bp|Araneae|Tetragnathidae|BOLD:ACP5884
Pachygnatha xanthostoma|[3178]|RRSPI352-15|632[0n]bp|Araneae|Tetragnathidae|BOLD:ACU5364
Tetragnatha guatemalensis|[3179]|RRINV2676-15|519[5n]bp|Araneae|Tetragnathidae|
Tetragnatha extensa|[3180]|RRINV1856-15|624[0n]bp|Araneae|Tetragnathidae|
Tetragnatha straminea|[3181]|RRSPI056-15|527[0n]bp|Araneae|Tetragnathidae|BOLD:AAD7095
Tetragnatha viridis|[3182]|RRSPI061-15|624[0n]bp|Araneae|Tetragnathidae|BOLD:AAG5659
Tetragnatha viridis|[3183]|RRSPI060-15|618[0n]bp|Araneae|Tetragnathidae|BOLD:AAN6335
Tetragnatha viridis|[3184]|RRINV2961-15|625[0n]bp|Araneae|Tetragnathidae|
Ozyptila praticola|[3185]|RRSSA2190-15|609[0n]bp|Araneae|Thomisidae|BOLD:AAC7413
Ozyptila americana|[3186]|RRINV987-15|628[0n]bp|Araneae|Thomisidae|
Tmarus angulatus|[3187]|RRSPI088-15|632[1n]bp|Araneae|Thomisidae|BOLD:ABY7475
Misumessus oblongus|[3188]|RRINV1009-15|628[1n]bp|Araneae|Thomisidae|
Misumenoides formosipes|[3189]|RRINV1023-15|632[0n]bp|Araneae|Thomisidae|
Misumena vatia|[3190]|RRSPI241-15|609[0n]bp|Araneae|Thomisidae|BOLD:AAA6275
Mecaphesa asperata|[3191]|RRSPI070-15|632[0n]bp|Araneae|Thomisidae|BOLD:ACE7683
Xysticus punctatus|[3192]|RRSPI167-15|621[0n]bp|Araneae|Thomisidae|BOLD:AAD2346
Xysticus winnipegensis|[3193]|RRSPI152-15|619[0n]bp|Araneae|Thomisidae|BOLD:AAM6956
Xysticus elegans|[3194]|RRSSA2491-15|636[0n]bp|Araneae|Thomisidae|BOLD:AAC1568
Xysticus funestus|[3195]|RRINV3572-15|608[0n]bp|Araneae|Thomisidae|
Xysticus luctans|[3196]|RRSPI200-15|611[0n]bp|Araneae|Thomisidae|BOLD:AAF8190
Xysticus emertoni|[3197]|RRSPI219-15|630[0n]bp|Araneae|Thomisidae|BOLD:AAB4300
Xysticus bicuspis|[3198]|RRSPI186-15|628[0n]bp|Araneae|Thomisidae|BOLD:AAJ9685
Xysticus discursans|[3199]|RRSPI032-15|631[0n]bp|Araneae|Thomisidae|BOLD:AAJ9718
Xysticus discursans|[3200]|RRSPI033-15|626[0n]bp|Araneae|Thomisidae|BOLD:ACV2014
Xysticus discursans|[3201]|RRSPI179-15|627[0n]bp|Araneae|Thomisidae|BOLD:ACV5078
Pirata piraticus|[3202]|RRSPI158-15|537[0n]bp|Araneae|Lycosidae|BOLD:AAB6784
Pirata praedo|[3203]|RRSPI377-15|632[0n]bp|Araneae|Lycosidae|BOLD:AAC5349
Piratula minuta|[3204]|RRBGA030-15|564[1n]bp|Araneae|Lycosidae|BOLD:AAE4247
Piratula cantralli|[3205]|RRMPC837-15|594[0n]bp|Araneae|Lycosidae|BOLD:ABZ5613
Pisaurina mira|[3206]|RRMFD527-15|564[0n]bp|Araneae|Pisauridae|BOLD:AAI2721
Dolomedes tenebrosus|[3207]|RRINV2984-15|625[0n]bp|Araneae|Pisauridae|
Dolomedes striatus|[3208]|RRSPI379-15|624[0n]bp|Araneae|Pisauridae|BOLD:ACI5773
Arctosa emertoni|[3209]|RRSPI065-15|632[0n]bp|Araneae|Lycosidae|BOLD:ACW1682
Pardosa moesta|[3210]|RRSPI024-15|631[0n]bp|Araneae|Lycosidae|BOLD:AAB0863
Pardosa milvina|[3211]|RRSPI198-15|610[0n]bp|Araneae|Lycosidae|BOLD:AAB7668
Pardosa modica|[3212]|RRSPI215-15|621[0n]bp|Araneae|Lycosidae|BOLD:AAA5090
Pardosa saxatilis|[3213]|RRSPI188-15|545[0n]bp|Araneae|Lycosidae|BOLD:AAB7667
Pardosa distincta|[3214]|RRSPI038-15|624[0n]bp|Araneae|Lycosidae|BOLD:AAC7802
Schizocosa ocreata|[3215]|RRSSA2476-15|632[0n]bp|Araneae|Lycosidae|BOLD:AAA7232
Schizocosa avida|[3216]|RRSPI062-15|632[0n]bp|Araneae|Lycosidae|BOLD:AAD3880
Schizocosa crassipalpata|[3217]|RRSPI201-15|409[0n]bp|Araneae|Lycosidae|BOLD:AAC4687
Schizocosa mccooki|[3218]|RRSPI386-15|632[0n]bp|Araneae|Lycosidae|BOLD:AAH0055
Trochosa terricola|[3219]|RRSSA2480-15|602[0n]bp|Araneae|Lycosidae|BOLD:AAB0727
Trochosa ruricola|[3220]|RRSSA1618-15|588[0n]bp|Araneae|Lycosidae|BOLD:AAB0726
Wulfila saltabundus|[3221]|RRSPI233-15|622[0n]bp|Araneae|Anyphaenidae|BOLD:AAC6924
Anyphaena pectorosa|[3222]|RRSPI094-15|632[0n]bp|Araneae|Anyphaenidae|BOLD:AAD6926
Anyphaena celer|[3223]|RRINV2433-15|632[0n]bp|Araneae|Anyphaenidae|
Clubiona bryantae|[3224]|RRINV991-15|632[0n]bp|Araneae|Clubionidae|
Clubiona obesa|[3225]|RRSPI093-15|632[0n]bp|Araneae|Clubionidae|BOLD:AAD5417
Clubiona maritima|[3226]|RRMPC910-15|588[0n]bp|Araneae|Clubionidae|BOLD:AAI4085
Clubiona pallidula|[3227]|RRSSA1621-15|588[0n]bp|Araneae|Clubionidae|BOLD:AAI4087
Clubiona johnsoni|[3228]|RRSPI382-15|342[0n]bp|Araneae|Clubionidae|BOLD:AAN4847
Clubiona abboti|[3229]|RRMPB141-15|567[0n]bp|Araneae|Clubionidae|BOLD:AAD1564
Agelenopsis potteri|[3230]|RRINV1887-15|618[0n]bp|Araneae|Agelenidae|
Marpissa formosa|[3231]|RRSPI209-15|627[0n]bp|Araneae|Salticidae|BOLD:AAG0312
Salticus scenicus|[3232]|RRINV1830-15|452[0n]bp|Araneae|Salticidae|
Hibana gracilis|[3233]|RRMFB1166-15|570[0n]bp|Araneae|Anyphaenidae|BOLD:AAN6394
Zygoballus nervosus|[3234]|RRSPI210-15|610[0n]bp|Araneae|Salticidae|BOLD:ACA1490
Sitticus floricola palustris|[3235]|RRSPI246-15|614[0n]bp|Araneae|Salticidae|BOLD:AAE1303

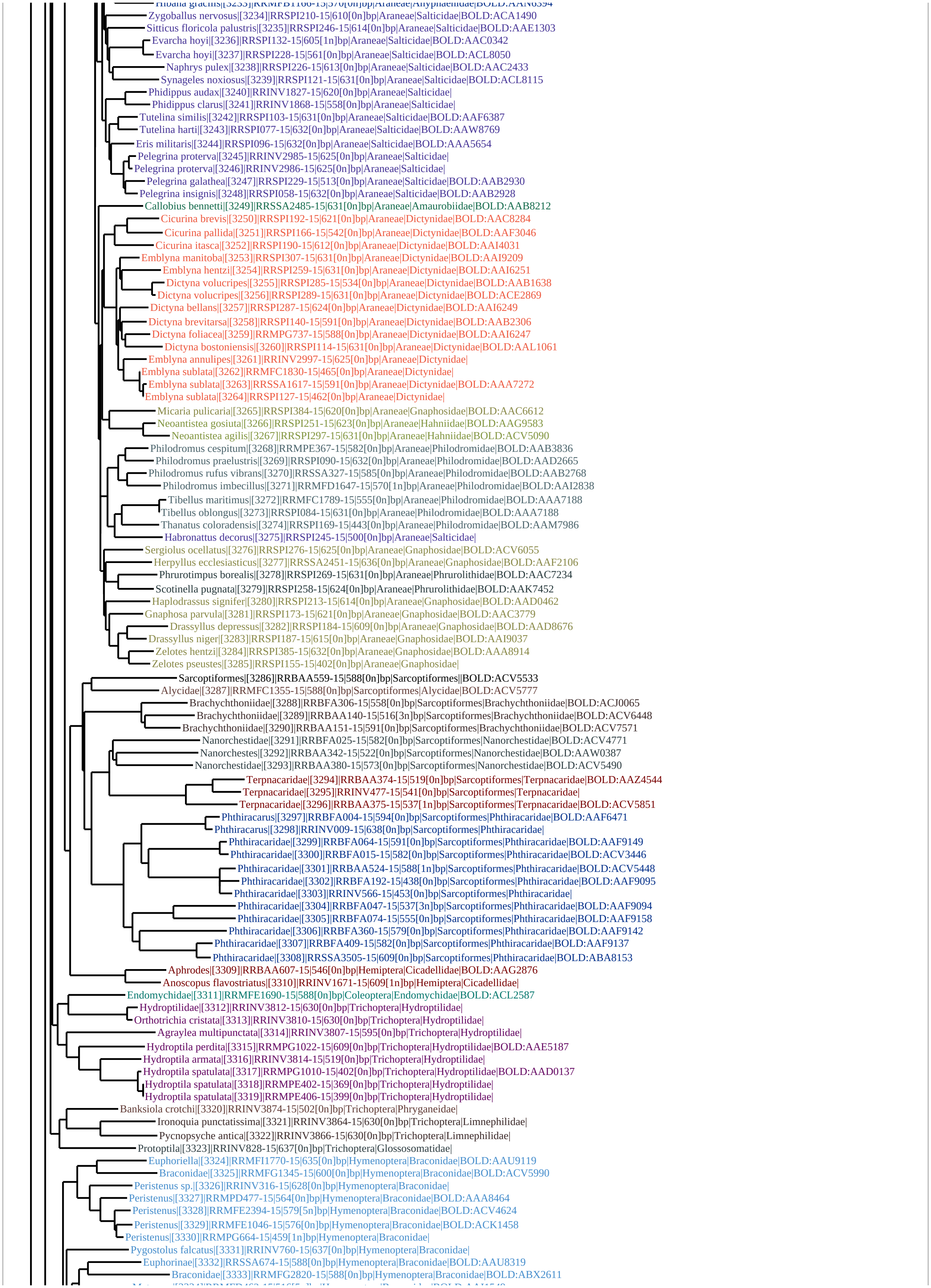
Zygoballus nervosus|[3234]|RRSPI210-15|610[0n]bp|Araneae|Salticidae|BOLD:ACA1490
Sitticus floricola palustris|[3235]|RRSPI246-15|614[0n]bp|Araneae|Salticidae|BOLD:AAE1303
Evarcha hoyi|[3236]|RRSPI132-15|605[1n]bp|Araneae|Salticidae|BOLD:AAC0342
Evarcha hoyi|[3237]|RRSPI228-15|561[0n]bp|Araneae|Salticidae|BOLD:ACL8050
Naphrys pulex|[3238]|RRSPI226-15|613[0n]bp|Araneae|Salticidae|BOLD:AAC2433
Synageles noxiosus|[3239]|RRSPI121-15|631[0n]bp|Araneae|Salticidae|BOLD:ACL8115
Phidippus audax|[3240]|RRINV1827-15|620[0n]bp|Araneae|Salticidae|
Phidippus clarus|[3241]|RRINV1868-15|558[0n]bp|Araneae|Salticidae|
Tutelina similis|[3242]|RRSPI103-15|631[0n]bp|Araneae|Salticidae|BOLD:AAF6387
Tutelina harti|[3243]|RRSPI077-15|632[0n]bp|Araneae|Salticidae|BOLD:AAW8769
Eris militaris|[3244]|RRSPI096-15|632[0n]bp|Araneae|Salticidae|BOLD:AAA5654
Pelegrina proterva|[3245]|RRINV2985-15|625[0n]bp|Araneae|Salticidae|
Pelegrina proterva|[3246]|RRINV2986-15|625[0n]bp|Araneae|Salticidae|
Pelegrina galathea|[3247]|RRSPI229-15|513[0n]bp|Araneae|Salticidae|BOLD:AAB2930
Pelegrina insignis|[3248]|RRSPI058-15|632[0n]bp|Araneae|Salticidae|BOLD:AAB2928
Callobius bennetti|[3249]|RRSSA2485-15|631[0n]bp|Araneae|Amaurobiidae|BOLD:AAB8212
Cicurina brevis|[3250]|RRSPI192-15|621[0n]bp|Araneae|Dictynidae|BOLD:AAC8284
Cicurina pallida|[3251]|RRSPI166-15|542[0n]bp|Araneae|Dictynidae|BOLD:AAF3046
Cicurina itasca|[3252]|RRSPI190-15|612[0n]bp|Araneae|Dictynidae|BOLD:AAI4031
Emblyna manitoba|[3253]|RRSPI307-15|631[0n]bp|Araneae|Dictynidae|BOLD:AAI9209
Emblyna hentzi|[3254]|RRSPI259-15|631[0n]bp|Araneae|Dictynidae|BOLD:AAI6251
Dictyna volucripes|[3255]|RRSPI285-15|534[0n]bp|Araneae|Dictynidae|BOLD:AAB1638
Dictyna volucripes|[3256]|RRSPI289-15|631[0n]bp|Araneae|Dictynidae|BOLD:ACE2869
Dictyna bellans|[3257]|RRSPI287-15|624[0n]bp|Araneae|Dictynidae|BOLD:AAI6249
Dictyna brevitarsa|[3258]|RRSPI140-15|591[0n]bp|Araneae|Dictynidae|BOLD:AAB2306
Dictyna foliacea|[3259]|RRMPG737-15|588[0n]bp|Araneae|Dictynidae|BOLD:AAI6247
Dictyna bostoniensis|[3260]|RRSPI114-15|631[0n]bp|Araneae|Dictynidae|BOLD:AAL1061
Emblyna annulipes|[3261]|RRINV2997-15|625[0n]bp|Araneae|Dictynidae|
Emblyna sublata|[3262]|RRMFC1830-15|465[0n]bp|Araneae|Dictynidae|
Emblyna sublata|[3263]|RRSSA1617-15|591[0n]bp|Araneae|Dictynidae|BOLD:AAA7272
Emblyna sublata|[3264]|RRSPI127-15|462[0n]bp|Araneae|Dictynidae|
Micaria pulicaria|[3265]|RRSPI384-15|620[0n]bp|Araneae|Gnaphosidae|BOLD:AAC6612
Neoantistea gosiuta|[3266]|RRSPI251-15|623[0n]bp|Araneae|Hahniidae|BOLD:AAG9583
Neoantistea agilis|[3267]|RRSPI297-15|631[0n]bp|Araneae|Hahniidae|BOLD:ACV5090
Philodromus cespitum|[3268]|RRMPE367-15|582[0n]bp|Araneae|Philodromidae|BOLD:AAB3836
Philodromus praelustris|[3269]|RRSPI090-15|632[0n]bp|Araneae|Philodromidae|BOLD:AAD2665
Philodromus rufus vibrans|[3270]|RRSSA327-15|585[0n]bp|Araneae|Philodromidae|BOLD:AAB2768
Philodromus imbecillus|[3271]|RRMFD1647-15|570[1n]bp|Araneae|Philodromidae|BOLD:AAI2838
Tibellus maritimus|[3272]|RRMFC1789-15|555[0n]bp|Araneae|Philodromidae|BOLD:AAA7188
Tibellus oblongus|[3273]|RRSPI084-15|631[0n]bp|Araneae|Philodromidae|BOLD:AAA7188
Thanatus coloradensis|[3274]|RRSPI169-15|443[0n]bp|Araneae|Philodromidae|BOLD:AAM7986
Habronattus decorus|[3275]|RRSPI245-15|500[0n]bp|Araneae|Salticidae|
Sergiolus ocellatus|[3276]|RRSPI276-15|625[0n]bp|Araneae|Gnaphosidae|BOLD:ACV6055
Herpyllus ecclesiasticus|[3277]|RRSSA2451-15|636[0n]bp|Araneae|Gnaphosidae|BOLD:AAF2106
Phrurotimpus borealis|[3278]|RRSPI269-15|631[0n]bp|Araneae|Phrurolithidae|BOLD:AAC7234
Scotinella pugnata|[3279]|RRSPI258-15|624[0n]bp|Araneae|Phrurolithidae|BOLD:AAK7452
Haplodrassus signifer|[3280]|RRSPI213-15|614[0n]bp|Araneae|Gnaphosidae|BOLD:AAD0462
Gnaphosa parvula|[3281]|RRSPI173-15|621[0n]bp|Araneae|Gnaphosidae|BOLD:AAC3779
Drassyllus depressus|[3282]|RRSPI184-15|609[0n]bp|Araneae|Gnaphosidae|BOLD:AAD8676
Drassyllus niger|[3283]|RRSPI187-15|615[0n]bp|Araneae|Gnaphosidae|BOLD:AAI9037
Zelotes hentzi|[3284]|RRSPI385-15|632[0n]bp|Araneae|Gnaphosidae|BOLD:AAA8914
Zelotes pseustes|[3285]|RRSPI155-15|402[0n]bp|Araneae|Gnaphosidae|
Sarcoptiformes|[3286]|RRBAA559-15|588[0n]bp|Sarcoptiformes||BOLD:ACV5533
Alycidae|[3287]|RRMFC1355-15|588[0n]bp|Sarcoptiformes|Alycidae|BOLD:ACV5777
Brachychthoniidae|[3288]|RRBFA306-15|558[0n]bp|Sarcoptiformes|Brachychthoniidae|BOLD:ACJ0065
Brachychthoniidae|[3289]|RRBAA140-15|516[3n]bp|Sarcoptiformes|Brachychthoniidae|BOLD:ACV6448
Brachychthoniidae|[3290]|RRBAA151-15|591[0n]bp|Sarcoptiformes|Brachychthoniidae|BOLD:ACV7571
Nanorchestidae|[3291]|RRBFA025-15|582[0n]bp|Sarcoptiformes|Nanorchestidae|BOLD:ACV4771
Nanorchestes|[3292]|RRBAA342-15|522[0n]bp|Sarcoptiformes|Nanorchestidae|BOLD:AAW0387
Nanorchestidae|[3293]|RRBAA380-15|573[0n]bp|Sarcoptiformes|Nanorchestidae|BOLD:ACV5490
Terpnacaridae|[3294]|RRBAA374-15|519[0n]bp|Sarcoptiformes|Terpnacaridae|BOLD:AAZ4544
Terpnacaridae|[3295]|RRINV477-15|541[0n]bp|Sarcoptiformes|Terpnacaridae|
Terpnacaridae|[3296]|RRBAA375-15|537[1n]bp|Sarcoptiformes|Terpnacaridae|BOLD:ACV5851
Phthiracarus|[3297]|RRBFA004-15|594[0n]bp|Sarcoptiformes|Phthiracaridae|BOLD:AAF6471
Phthiracarus|[3298]|RRINV009-15|638[0n]bp|Sarcoptiformes|Phthiracaridae|
Phthiracaridae|[3299]|RRBFA064-15|591[0n]bp|Sarcoptiformes|Phthiracaridae|BOLD:AAF9149
Phthiracaridae|[3300]|RRBFA015-15|582[0n]bp|Sarcoptiformes|Phthiracaridae|BOLD:ACV3446
Phthiracaridae|[3301]|RRBAA524-15|588[1n]bp|Sarcoptiformes|Phthiracaridae|BOLD:ACV5448
Phthiracaridae|[3302]|RRBFA192-15|438[0n]bp|Sarcoptiformes|Phthiracaridae|BOLD:AAF9095
Phthiracaridae|[3303]|RRINV566-15|453[0n]bp|Sarcoptiformes|Phthiracaridae|
Phthiracaridae|[3304]|RRBFA047-15|537[3n]bp|Sarcoptiformes|Phthiracaridae|BOLD:AAF9094
Phthiracaridae|[3305]|RRBFA074-15|555[0n]bp|Sarcoptiformes|Phthiracaridae|BOLD:AAF9158
Phthiracaridae|[3306]|RRBFA360-15|579[0n]bp|Sarcoptiformes|Phthiracaridae|BOLD:AAF9142
Phthiracaridae|[3307]|RRBFA409-15|582[0n]bp|Sarcoptiformes|Phthiracaridae|BOLD:AAF9137
Phthiracaridae|[3308]|RRSSA3505-15|609[0n]bp|Sarcoptiformes|Phthiracaridae|BOLD:ABA8153
Aphrodes|[3309]|RRBAA607-15|546[0n]bp|Hemiptera|Cicadellidae|BOLD:AAG2876
Anoscopus flavostriatus|[3310]|RRINV1671-15|609[1n]bp|Hemiptera|Cicadellidae|
Endomychidae|[3311]|RRMFE1690-15|588[0n]bp|Coleoptera|Endomychidae|BOLD:ACL2587
Hydroptilidae|[3312]|RRINV3812-15|630[0n]bp|Trichoptera|Hydroptilidae|
Orthotrichia cristata|[3313]|RRINV3810-15|630[0n]bp|Trichoptera|Hydroptilidae|
Agraylea multipunctata|[3314]|RRINV3807-15|595[0n]bp|Trichoptera|Hydroptilidae|
Hydroptila perdita|[3315]|RRMPG1022-15|609[0n]bp|Trichoptera|Hydroptilidae|BOLD:AAE5187
Hydroptila armata|[3316]|RRINV3814-15|519[0n]bp|Trichoptera|Hydroptilidae|
Hydroptila spatulata|[3317]|RRMPG1010-15|402[0n]bp|Trichoptera|Hydroptilidae|BOLD:AAD0137
Hydroptila spatulata|[3318]|RRMPE402-15|369[0n]bp|Trichoptera|Hydroptilidae|
Hydroptila spatulata|[3319]|RRMPE406-15|399[0n]bp|Trichoptera|Hydroptilidae|
Banksiola crotchi|[3320]|RRINV3874-15|502[0n]bp|Trichoptera|Phryganeidae|
Ironoquia punctatissima|[3321]|RRINV3864-15|630[0n]bp|Trichoptera|Limnephilidae|
Pycnopsyche antica|[3322]|RRINV3866-15|630[0n]bp|Trichoptera|Limnephilidae|
Protoptila|[3323]|RRINV828-15|637[0n]bp|Trichoptera|Glossosomatidae|
Euphoriella|[3324]|RRMFI1770-15|635[0n]bp|Hymenoptera|Braconidae|BOLD:AAU9119
Braconidae|[3325]|RRMFG1345-15|600[0n]bp|Hymenoptera|Braconidae|BOLD:ACV5990
Peristenus sp.|[3326]|RRINV316-15|628[0n]bp|Hymenoptera|Braconidae|
Peristenus|[3327]|RRMPD477-15|564[0n]bp|Hymenoptera|Braconidae|BOLD:AAA8464
Peristenus|[3328]|RRMFE2394-15|579[5n]bp|Hymenoptera|Braconidae|BOLD:ACV4624
Peristenus|[3329]|RRMFE1046-15|576[0n]bp|Hymenoptera|Braconidae|BOLD:ACK1458
Peristenus|[3330]|RRMPG664-15|459[1n]bp|Hymenoptera|Braconidae|
Pygostolus falcatus|[3331]|RRINV760-15|637[0n]bp|Hymenoptera|Braconidae|
Euphorinae|[3332]|RRSSA674-15|588[0n]bp|Hymenoptera|Braconidae|BOLD:AAU8319
Braconidae|[3333]|RRMFG2820-15|588[0n]bp|Hymenoptera|Braconidae|BOLD:ABX2611

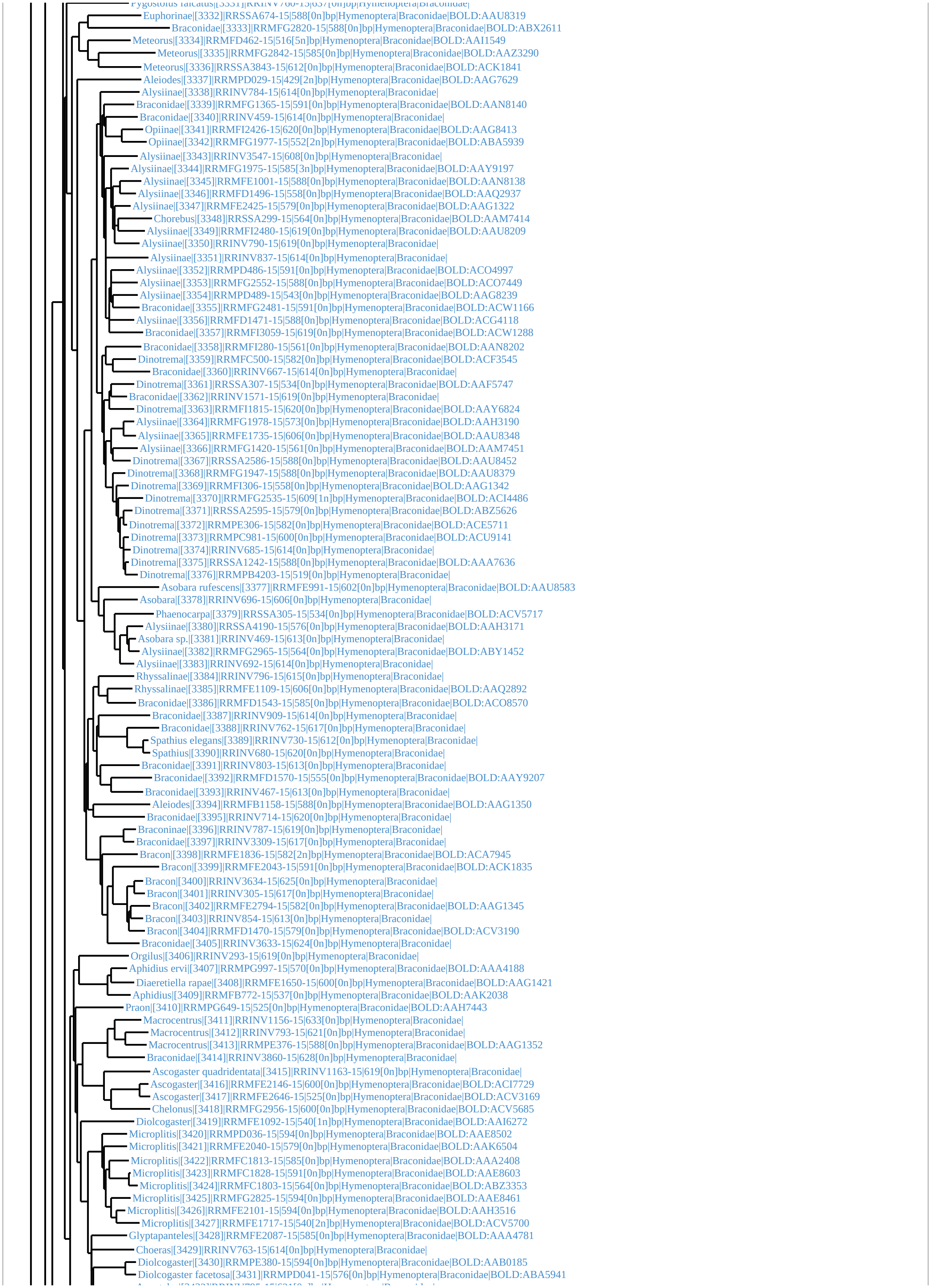
Euphorinae|[3332]|RRSSA674-15|588[0n]bp|Hymenoptera|Braconidae|BOLD:AAU8319
Braconidae|[3333]|RRMFG2820-15|588[0n]bp|Hymenoptera|Braconidae|BOLD:ABX2611
Meteorus|[3334]|RRMFD462-15|516[5n]bp|Hymenoptera|Braconidae|BOLD:AAI1549
Meteorus|[3335]|RRMFG2842-15|585[0n]bp|Hymenoptera|Braconidae|BOLD:AAZ3290
Meteorus|[3336]|RRSSA3843-15|612[0n]bp|Hymenoptera|Braconidae|BOLD:ACK1841
Aleiodes|[3337]|RRMPD029-15|429[2n]bp|Hymenoptera|Braconidae|BOLD:AAG7629
Alysiinae|[3338]|RRINV784-15|614[0n]bp|Hymenoptera|Braconidae|
Braconidae|[3339]|RRMFG1365-15|591[0n]bp|Hymenoptera|Braconidae|BOLD:AAN8140
Braconidae|[3340]|RRINV459-15|614[0n]bp|Hymenoptera|Braconidae|
Opiinae|[3341]|RRMFI2426-15|620[0n]bp|Hymenoptera|Braconidae|BOLD:AAG8413
Opiinae|[3342]|RRMFG1977-15|552[2n]bp|Hymenoptera|Braconidae|BOLD:ABA5939
Alysiinae|[3343]|RRINV3547-15|608[0n]bp|Hymenoptera|Braconidae|
Alysiinae|[3344]|RRMFG1975-15|585[3n]bp|Hymenoptera|Braconidae|BOLD:AAY9197
Alysiinae|[3345]|RRMFE1001-15|588[0n]bp|Hymenoptera|Braconidae|BOLD:AAN8138
Alysiinae|[3346]|RRMFD1496-15|558[0n]bp|Hymenoptera|Braconidae|BOLD:AAQ2937
Alysiinae|[3347]|RRMFE2425-15|579[0n]bp|Hymenoptera|Braconidae|BOLD:AAG1322
Chorebus|[3348]|RRSSA299-15|564[0n]bp|Hymenoptera|Braconidae|BOLD:AAM7414
Alysiinae|[3349]|RRMFI2480-15|619[0n]bp|Hymenoptera|Braconidae|BOLD:AAU8209
Alysiinae|[3350]|RRINV790-15|619[0n]bp|Hymenoptera|Braconidae|
Alysiinae|[3351]|RRINV837-15|614[0n]bp|Hymenoptera|Braconidae|
Alysiinae|[3352]|RRMPD486-15|591[0n]bp|Hymenoptera|Braconidae|BOLD:ACO4997
Alysiinae|[3353]|RRMFG2552-15|588[0n]bp|Hymenoptera|Braconidae|BOLD:ACO7449
Alysiinae|[3354]|RRMPD489-15|543[0n]bp|Hymenoptera|Braconidae|BOLD:AAG8239
Braconidae|[3355]|RRMFG2481-15|591[0n]bp|Hymenoptera|Braconidae|BOLD:ACW1166
Alysiinae|[3356]|RRMFD1471-15|588[0n]bp|Hymenoptera|Braconidae|BOLD:ACG4118
Braconidae|[3357]|RRMFI3059-15|619[0n]bp|Hymenoptera|Braconidae|BOLD:ACW1288
Braconidae|[3358]|RRMFI280-15|561[0n]bp|Hymenoptera|Braconidae|BOLD:AAN8202
Dinotrema|[3359]|RRMFC500-15|582[0n]bp|Hymenoptera|Braconidae|BOLD:ACF3545
Braconidae|[3360]|RRINV667-15|614[0n]bp|Hymenoptera|Braconidae|
Dinotrema|[3361]|RRSSA307-15|534[0n]bp|Hymenoptera|Braconidae|BOLD:AAF5747
Braconidae|[3362]|RRINV1571-15|619[0n]bp|Hymenoptera|Braconidae|
Dinotrema|[3363]|RRMFI1815-15|620[0n]bp|Hymenoptera|Braconidae|BOLD:AAY6824
Alysiinae|[3364]|RRMFG1978-15|573[0n]bp|Hymenoptera|Braconidae|BOLD:AAH3190
Alysiinae|[3365]|RRMFE1735-15|606[0n]bp|Hymenoptera|Braconidae|BOLD:AAU8348
Alysiinae|[3366]|RRMFG1420-15|561[0n]bp|Hymenoptera|Braconidae|BOLD:AAM7451
Dinotrema|[3367]|RRSSA2586-15|588[0n]bp|Hymenoptera|Braconidae|BOLD:AAU8452
Dinotrema|[3368]|RRMFG1947-15|588[0n]bp|Hymenoptera|Braconidae|BOLD:AAU8379
Dinotrema|[3369]|RRMFI306-15|558[0n]bp|Hymenoptera|Braconidae|BOLD:AAG1342
Dinotrema|[3370]|RRMFG2535-15|609[1n]bp|Hymenoptera|Braconidae|BOLD:ACI4486
Dinotrema|[3371]|RRSSA2595-15|579[0n]bp|Hymenoptera|Braconidae|BOLD:ABZ5626
Dinotrema|[3372]|RRMPE306-15|582[0n]bp|Hymenoptera|Braconidae|BOLD:ACE5711
Dinotrema|[3373]|RRMPC981-15|600[0n]bp|Hymenoptera|Braconidae|BOLD:ACU9141
Dinotrema|[3374]|RRINV685-15|614[0n]bp|Hymenoptera|Braconidae|
Dinotrema|[3375]|RRSSA1242-15|588[0n]bp|Hymenoptera|Braconidae|BOLD:AAA7636
Dinotrema|[3376]|RRMPB4203-15|519[0n]bp|Hymenoptera|Braconidae|
Asobara rufescens|[3377]|RRMFE991-15|602[0n]bp|Hymenoptera|Braconidae|BOLD:AAU8583
Asobara|[3378]|RRINV696-15|606[0n]bp|Hymenoptera|Braconidae|
Phaenocarpa|[3379]|RRSSA305-15|534[0n]bp|Hymenoptera|Braconidae|BOLD:ACV5717
Alysiinae|[3380]|RRSSA4190-15|576[0n]bp|Hymenoptera|Braconidae|BOLD:AAH3171
Asobara sp.|[3381]|RRINV469-15|613[0n]bp|Hymenoptera|Braconidae|
Alysiinae|[3382]|RRMFG2965-15|564[0n]bp|Hymenoptera|Braconidae|BOLD:ABY1452
Alysiinae|[3383]|RRINV692-15|614[0n]bp|Hymenoptera|Braconidae|
Rhyssalinae|[3384]|RRINV796-15|615[0n]bp|Hymenoptera|Braconidae|
Rhyssalinae|[3385]|RRMFE1109-15|606[0n]bp|Hymenoptera|Braconidae|BOLD:AAQ2892
Braconidae|[3386]|RRMFD1543-15|585[0n]bp|Hymenoptera|Braconidae|BOLD:ACO8570
Braconidae|[3387]|RRINV909-15|614[0n]bp|Hymenoptera|Braconidae|
Braconidae|[3388]|RRINV762-15|617[0n]bp|Hymenoptera|Braconidae|
Spathius elegans|[3389]|RRINV730-15|612[0n]bp|Hymenoptera|Braconidae|
Spathius|[3390]|RRINV680-15|620[0n]bp|Hymenoptera|Braconidae|
Braconidae|[3391]|RRINV803-15|613[0n]bp|Hymenoptera|Braconidae|
Braconidae|[3392]|RRMFD1570-15|555[0n]bp|Hymenoptera|Braconidae|BOLD:AAY9207
Braconidae|[3393]|RRINV467-15|613[0n]bp|Hymenoptera|Braconidae|
Aleiodes|[3394]|RRMFB1158-15|588[0n]bp|Hymenoptera|Braconidae|BOLD:AAG1350
Braconidae|[3395]|RRINV714-15|620[0n]bp|Hymenoptera|Braconidae|
Braconinae|[3396]|RRINV787-15|619[0n]bp|Hymenoptera|Braconidae|
Braconidae|[3397]|RRINV3309-15|617[0n]bp|Hymenoptera|Braconidae|
Bracon|[3398]|RRMFE1836-15|582[2n]bp|Hymenoptera|Braconidae|BOLD:ACA7945
Bracon|[3399]|RRMFE2043-15|591[0n]bp|Hymenoptera|Braconidae|BOLD:ACK1835
Bracon|[3400]|RRINV3634-15|625[0n]bp|Hymenoptera|Braconidae|
Bracon|[3401]|RRINV305-15|617[0n]bp|Hymenoptera|Braconidae|
Bracon|[3402]|RRMFE2794-15|582[0n]bp|Hymenoptera|Braconidae|BOLD:AAG1345
Bracon|[3403]|RRINV854-15|613[0n]bp|Hymenoptera|Braconidae|
Bracon|[3404]|RRMFD1470-15|579[0n]bp|Hymenoptera|Braconidae|BOLD:ACV3190
Braconidae|[3405]|RRINV3633-15|624[0n]bp|Hymenoptera|Braconidae|
Orgilus|[3406]|RRINV293-15|619[0n]bp|Hymenoptera|Braconidae|
Aphidius ervi|[3407]|RRMPG997-15|570[0n]bp|Hymenoptera|Braconidae|BOLD:AAA4188
Diaeretiella rapae|[3408]|RRMFE1650-15|600[0n]bp|Hymenoptera|Braconidae|BOLD:AAG1421
Aphidius|[3409]|RRMFB772-15|537[0n]bp|Hymenoptera|Braconidae|BOLD:AAK2038
Praon|[3410]|RRMPG649-15|525[0n]bp|Hymenoptera|Braconidae|BOLD:AAH7443
Macrocentrus|[3411]|RRINV1156-15|633[0n]bp|Hymenoptera|Braconidae|
Macrocentrus|[3412]|RRINV793-15|621[0n]bp|Hymenoptera|Braconidae|
Macrocentrus|[3413]|RRMPE376-15|588[0n]bp|Hymenoptera|Braconidae|BOLD:AAG1352
Braconidae|[3414]|RRINV3860-15|628[0n]bp|Hymenoptera|Braconidae|
Ascogaster quadridentata|[3415]|RRINV1163-15|619[0n]bp|Hymenoptera|Braconidae|
Ascogaster|[3416]|RRMFE2146-15|600[0n]bp|Hymenoptera|Braconidae|BOLD:ACI7729
Ascogaster|[3417]|RRMFE2646-15|525[0n]bp|Hymenoptera|Braconidae|BOLD:ACV3169
Chelonus|[3418]|RRMFG2956-15|600[0n]bp|Hymenoptera|Braconidae|BOLD:ACV5685
Diolcogaster|[3419]|RRMFE1092-15|540[1n]bp|Hymenoptera|Braconidae|BOLD:AAI6272
Microplitis|[3420]|RRMPD036-15|594[0n]bp|Hymenoptera|Braconidae|BOLD:AAE8502
Microplitis|[3421]|RRMFE2040-15|579[0n]bp|Hymenoptera|Braconidae|BOLD:AAK6504
Microplitis|[3422]|RRMFC1813-15|585[0n]bp|Hymenoptera|Braconidae|BOLD:AAA2408
Microplitis|[3423]|RRMFC1828-15|591[0n]bp|Hymenoptera|Braconidae|BOLD:AAE8603
Microplitis|[3424]|RRMFC1803-15|564[0n]bp|Hymenoptera|Braconidae|BOLD:ABZ3353
Microplitis|[3425]|RRMFG2825-15|594[0n]bp|Hymenoptera|Braconidae|BOLD:AAE8461
Microplitis|[3426]|RRMFE2101-15|594[0n]bp|Hymenoptera|Braconidae|BOLD:AAH3516
Microplitis|[3427]|RRMFE1717-15|540[2n]bp|Hymenoptera|Braconidae|BOLD:ACV5700
Glyptapanteles|[3428]|RRMFE2087-15|585[0n]bp|Hymenoptera|Braconidae|BOLD:AAA4781
Choeras|[3429]|RRINV763-15|614[0n]bp|Hymenoptera|Braconidae|
Diolcogaster|[3430]|RRMPE380-15|594[0n]bp|Hymenoptera|Braconidae|BOLD:AAB0185
Diolcogaster facetosa|[3431]|RRMPD041-15|576[0n]bp|Hymenoptera|Braconidae|BOLD:ABA5941

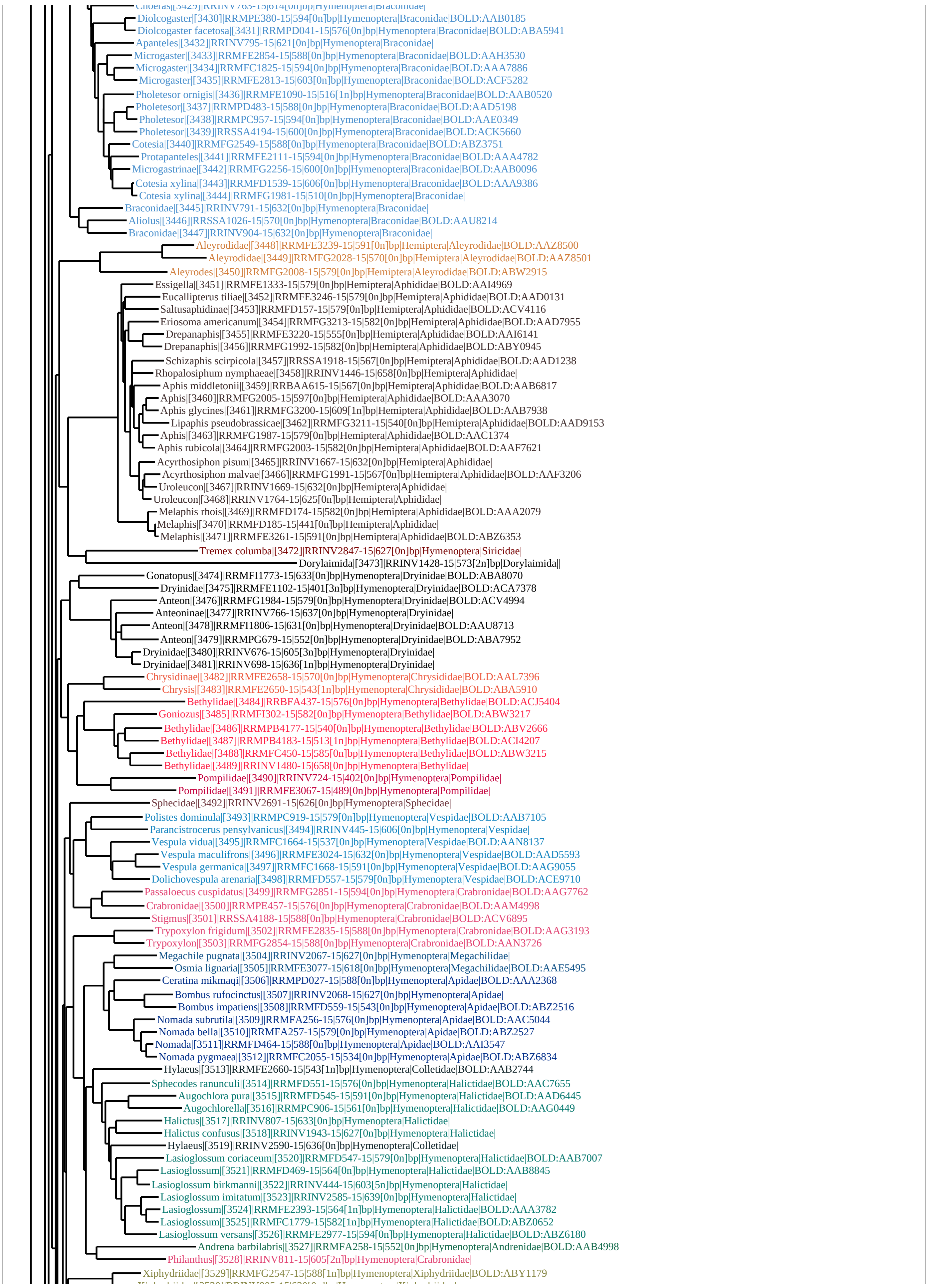
Diolcogaster|[3430]|RRMPE380-15|594[0n]bp|Hymenoptera|Braconidae|BOLD:AAB0185
Diolcogaster facetosa|[3431]|RRMPD041-15|576[0n]bp|Hymenoptera|Braconidae|BOLD:ABA5941
Apanteles|[3432]|RRINV795-15|621[0n]bp|Hymenoptera|Braconidae|
Microgaster|[3433]|RRMFE2854-15|588[0n]bp|Hymenoptera|Braconidae|BOLD:AAH3530
Microgaster|[3434]|RRMFC1825-15|594[0n]bp|Hymenoptera|Braconidae|BOLD:AAA7886
Microgaster|[3435]|RRMFE2813-15|603[0n]bp|Hymenoptera|Braconidae|BOLD:ACF5282
Pholetesor ornigis|[3436]|RRMFE1090-15|516[1n]bp|Hymenoptera|Braconidae|BOLD:AAB0520
Pholetesor|[3437]|RRMPD483-15|588[0n]bp|Hymenoptera|Braconidae|BOLD:AAD5198
Pholetesor|[3438]|RRMPC957-15|594[0n]bp|Hymenoptera|Braconidae|BOLD:AAE0349
Pholetesor|[3439]|RRSSA4194-15|600[0n]bp|Hymenoptera|Braconidae|BOLD:ACK5660
Cotesia|[3440]|RRMFG2549-15|588[0n]bp|Hymenoptera|Braconidae|BOLD:ABZ3751
Protapanteles|[3441]|RRMFE2111-15|594[0n]bp|Hymenoptera|Braconidae|BOLD:AAA4782
Microgastrinae|[3442]|RRMFG2256-15|600[0n]bp|Hymenoptera|Braconidae|BOLD:AAB0096
Cotesia xylina|[3443]|RRMFD1539-15|606[0n]bp|Hymenoptera|Braconidae|BOLD:AAA9386
Cotesia xylina|[3444]|RRMFG1981-15|510[0n]bp|Hymenoptera|Braconidae|
Braconidae|[3445]|RRINV791-15|632[0n]bp|Hymenoptera|Braconidae|
Aliolus|[3446]|RRSSA1026-15|570[0n]bp|Hymenoptera|Braconidae|BOLD:AAU8214
Braconidae|[3447]|RRINV904-15|632[0n]bp|Hymenoptera|Braconidae|
Aleyrodidae|[3448]|RRMFE3239-15|591[0n]bp|Hemiptera|Aleyrodidae|BOLD:AAZ8500
Aleyrodidae|[3449]|RRMFG2028-15|570[0n]bp|Hemiptera|Aleyrodidae|BOLD:AAZ8501
Aleyrodes|[3450]|RRMFG2008-15|579[0n]bp|Hemiptera|Aleyrodidae|BOLD:ABW2915
Essigella|[3451]|RRMFE1333-15|579[0n]bp|Hemiptera|Aphididae|BOLD:AAI4969
Eucallipterus tiliae|[3452]|RRMFE3246-15|579[0n]bp|Hemiptera|Aphididae|BOLD:AAD0131
Saltusaphidinae|[3453]|RRMFD157-15|579[0n]bp|Hemiptera|Aphididae|BOLD:ACV4116
Eriosoma americanum|[3454]|RRMFG3213-15|582[0n]bp|Hemiptera|Aphididae|BOLD:AAD7955
Drepanaphis|[3455]|RRMFE3220-15|555[0n]bp|Hemiptera|Aphididae|BOLD:AAI6141
Drepanaphis|[3456]|RRMFG1992-15|582[0n]bp|Hemiptera|Aphididae|BOLD:ABY0945
Schizaphis scirpicola|[3457]|RRSSA1918-15|567[0n]bp|Hemiptera|Aphididae|BOLD:AAD1238
Rhopalosiphum nymphaeae|[3458]|RRINV1446-15|658[0n]bp|Hemiptera|Aphididae|
Aphis middletonii|[3459]|RRBAA615-15|567[0n]bp|Hemiptera|Aphididae|BOLD:AAB6817
Aphis|[3460]|RRMFG2005-15|597[0n]bp|Hemiptera|Aphididae|BOLD:AAA3070
Aphis glycines|[3461]|RRMFG3200-15|609[1n]bp|Hemiptera|Aphididae|BOLD:AAB7938
Lipaphis pseudobrassicae|[3462]|RRMFG3211-15|540[0n]bp|Hemiptera|Aphididae|BOLD:AAD9153
Aphis|[3463]|RRMFG1987-15|579[0n]bp|Hemiptera|Aphididae|BOLD:AAC1374
Aphis rubicola|[3464]|RRMFG2003-15|582[0n]bp|Hemiptera|Aphididae|BOLD:AAF7621
Acyrthosiphon pisum|[3465]|RRINV1667-15|632[0n]bp|Hemiptera|Aphididae|
Acyrthosiphon malvae|[3466]|RRMFG1991-15|567[0n]bp|Hemiptera|Aphididae|BOLD:AAF3206
Uroleucon|[3467]|RRINV1669-15|632[0n]bp|Hemiptera|Aphididae|
Uroleucon|[3468]|RRINV1764-15|625[0n]bp|Hemiptera|Aphididae|
Melaphis rhois|[3469]|RRMFD174-15|582[0n]bp|Hemiptera|Aphididae|BOLD:AAA2079
Melaphis|[3470]|RRMFD185-15|441[0n]bp|Hemiptera|Aphididae|
Melaphis|[3471]|RRMFE3261-15|591[0n]bp|Hemiptera|Aphididae|BOLD:ABZ6353
Tremex columba|[3472]|RRINV2847-15|627[0n]bp|Hymenoptera|Siricidae|
Dorylaimida|[3473]|RRINV1428-15|573[2n]bp|Dorylaimida||
Gonatopus|[3474]|RRMFI1773-15|633[0n]bp|Hymenoptera|Dryinidae|BOLD:ABA8070
Dryinidae|[3475]|RRMFE1102-15|401[3n]bp|Hymenoptera|Dryinidae|BOLD:ACA7378
Anteon|[3476]|RRMFG1984-15|579[0n]bp|Hymenoptera|Dryinidae|BOLD:ACV4994
Anteoninae|[3477]|RRINV766-15|637[0n]bp|Hymenoptera|Dryinidae|
Anteon|[3478]|RRMFI1806-15|631[0n]bp|Hymenoptera|Dryinidae|BOLD:AAU8713
Anteon|[3479]|RRMPG679-15|552[0n]bp|Hymenoptera|Dryinidae|BOLD:ABA7952
Dryinidae|[3480]|RRINV676-15|605[3n]bp|Hymenoptera|Dryinidae|
Dryinidae|[3481]|RRINV698-15|636[1n]bp|Hymenoptera|Dryinidae|
Chrysidinae|[3482]|RRMFE2658-15|570[0n]bp|Hymenoptera|Chrysididae|BOLD:AAL7396
Chrysis|[3483]|RRMFE2650-15|543[1n]bp|Hymenoptera|Chrysididae|BOLD:ABA5910
Bethylidae|[3484]|RRBFA437-15|576[0n]bp|Hymenoptera|Bethylidae|BOLD:ACJ5404
Goniozus|[3485]|RRMFI302-15|582[0n]bp|Hymenoptera|Bethylidae|BOLD:ABW3217
Bethylidae|[3486]|RRMPB4177-15|540[0n]bp|Hymenoptera|Bethylidae|BOLD:ABV2666
Bethylidae|[3487]|RRMPB4183-15|513[1n]bp|Hymenoptera|Bethylidae|BOLD:ACI4207
Bethylidae|[3488]|RRMFC450-15|585[0n]bp|Hymenoptera|Bethylidae|BOLD:ABW3215
Bethylidae|[3489]|RRINV1480-15|658[0n]bp|Hymenoptera|Bethylidae|
Pompilidae|[3490]|RRINV724-15|402[0n]bp|Hymenoptera|Pompilidae|
Pompilidae|[3491]|RRMFE3067-15|489[0n]bp|Hymenoptera|Pompilidae|
Sphecidae|[3492]|RRINV2691-15|626[0n]bp|Hymenoptera|Sphecidae|
Polistes dominula|[3493]|RRMPC919-15|579[0n]bp|Hymenoptera|Vespidae|BOLD:AAB7105
Parancistrocerus pensylvanicus|[3494]|RRINV445-15|606[0n]bp|Hymenoptera|Vespidae|
Vespula vidua|[3495]|RRMFC1664-15|537[0n]bp|Hymenoptera|Vespidae|BOLD:AAN8137
Vespula maculifrons|[3496]|RRMFE3024-15|632[0n]bp|Hymenoptera|Vespidae|BOLD:AAD5593
Vespula germanica|[3497]|RRMFC1668-15|591[0n]bp|Hymenoptera|Vespidae|BOLD:AAG9055
Dolichovespula arenaria|[3498]|RRMFD557-15|579[0n]bp|Hymenoptera|Vespidae|BOLD:ACE9710
Passaloecus cuspidatus|[3499]|RRMFG2851-15|594[0n]bp|Hymenoptera|Crabronidae|BOLD:AAG7762
Crabronidae|[3500]|RRMPE457-15|576[0n]bp|Hymenoptera|Crabronidae|BOLD:AAM4998
Stigmus|[3501]|RRSSA4188-15|588[0n]bp|Hymenoptera|Crabronidae|BOLD:ACV6895
Trypoxylon frigidum|[3502]|RRMFE2835-15|588[0n]bp|Hymenoptera|Crabronidae|BOLD:AAG3193
Trypoxylon|[3503]|RRMFG2854-15|588[0n]bp|Hymenoptera|Crabronidae|BOLD:AAN3726
Megachile pugnata|[3504]|RRINV2067-15|627[0n]bp|Hymenoptera|Megachilidae|
Osmia lignaria|[3505]|RRMFE3077-15|618[0n]bp|Hymenoptera|Megachilidae|BOLD:AAE5495
Ceratina mikmaqi|[3506]|RRMPD027-15|588[0n]bp|Hymenoptera|Apidae|BOLD:AAA2368
Bombus rufocinctus|[3507]|RRINV2068-15|627[0n]bp|Hymenoptera|Apidae|
Bombus impatiens|[3508]|RRMFD559-15|543[0n]bp|Hymenoptera|Apidae|BOLD:ABZ2516
Nomada subrutila|[3509]|RRMFA256-15|576[0n]bp|Hymenoptera|Apidae|BOLD:AAC5044
Nomada bella|[3510]|RRMFA257-15|579[0n]bp|Hymenoptera|Apidae|BOLD:ABZ2527
Nomada|[3511]|RRMFD464-15|588[0n]bp|Hymenoptera|Apidae|BOLD:AAI3547
Nomada pygmaea|[3512]|RRMFC2055-15|534[0n]bp|Hymenoptera|Apidae|BOLD:ABZ6834
Hylaeus|[3513]|RRMFE2660-15|543[1n]bp|Hymenoptera|Colletidae|BOLD:AAB2744
Sphecodes ranunculi|[3514]|RRMFD551-15|576[0n]bp|Hymenoptera|Halictidae|BOLD:AAC7655
Augochlora pura|[3515]|RRMFD545-15|591[0n]bp|Hymenoptera|Halictidae|BOLD:AAD6445
Augochlorella|[3516]|RRMPC906-15|561[0n]bp|Hymenoptera|Halictidae|BOLD:AAG0449
Halictus|[3517]|RRINV807-15|633[0n]bp|Hymenoptera|Halictidae|
Halictus confusus|[3518]|RRINV1943-15|627[0n]bp|Hymenoptera|Halictidae|
Hylaeus|[3519]|RRINV2590-15|636[0n]bp|Hymenoptera|Colletidae|
Lasioglossum coriaceum|[3520]|RRMFD547-15|579[0n]bp|Hymenoptera|Halictidae|BOLD:AAB7007
Lasioglossum|[3521]|RRMFD469-15|564[0n]bp|Hymenoptera|Halictidae|BOLD:AAB8845
Lasioglossum birkmanni|[3522]|RRINV444-15|603[5n]bp|Hymenoptera|Halictidae|
Lasioglossum imitatum|[3523]|RRINV2585-15|639[0n]bp|Hymenoptera|Halictidae|
Lasioglossum|[3524]|RRMFE2393-15|564[1n]bp|Hymenoptera|Halictidae|BOLD:AAA3782
Lasioglossum|[3525]|RRMFC1779-15|582[1n]bp|Hymenoptera|Halictidae|BOLD:ABZ0652
Lasioglossum versans|[3526]|RRMFE2977-15|594[0n]bp|Hymenoptera|Halictidae|BOLD:ABZ6180
Andrena barbilabris|[3527]|RRMFA258-15|552[0n]bp|Hymenoptera|Andrenidae|BOLD:AAB4998
Philanthus|[3528]|RRINV811-15|605[2n]bp|Hymenoptera|Crabronidae|
Xiphydriidae|[3529]|RRMFG2547-15|588[1n]bp|Hymenoptera|Xiphydriidae|BOLD:ABY1179

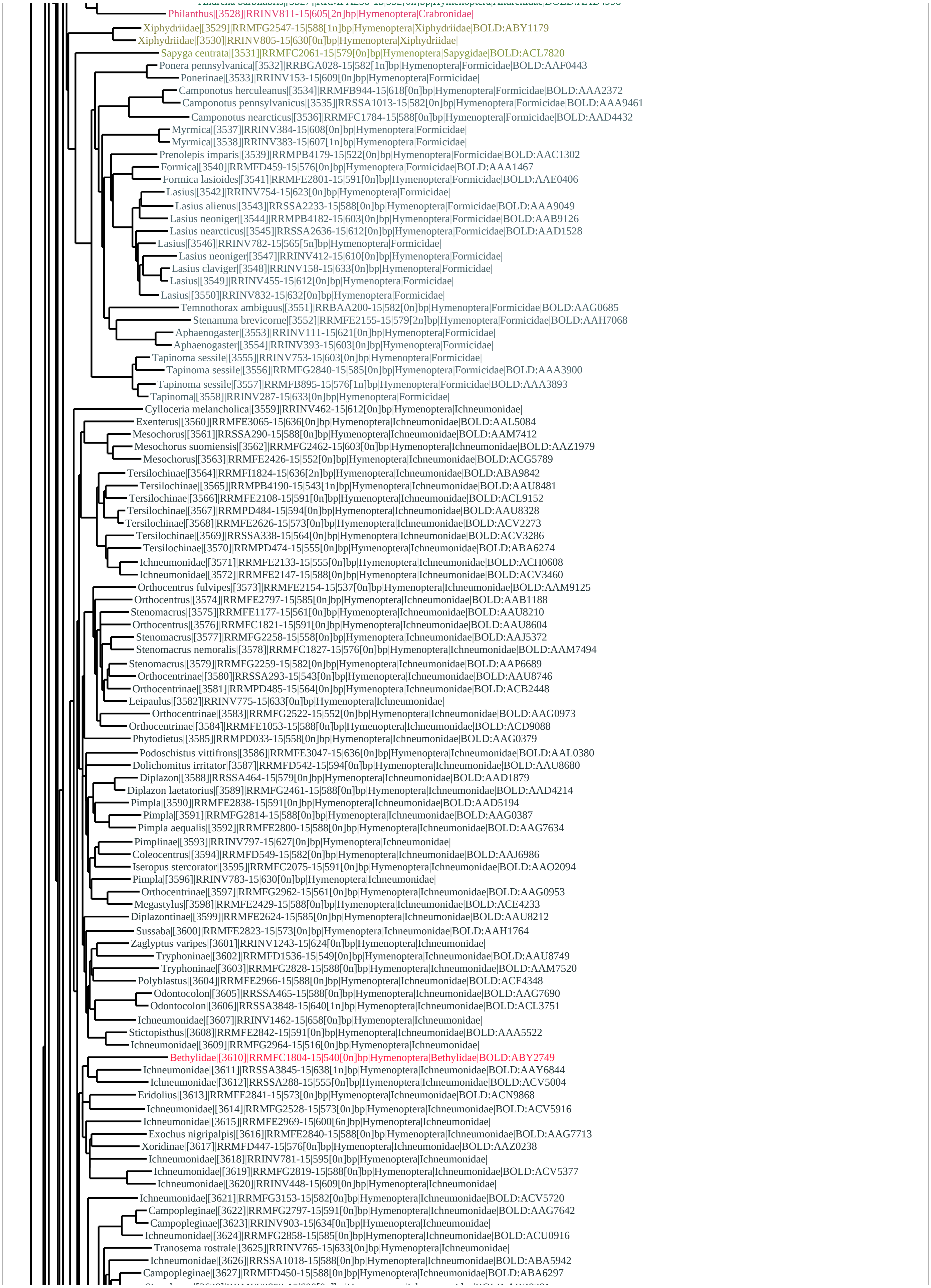
Philanthus|[3528]|RRINV811-15|605[2n]bp|Hymenoptera|Crabronidae|
Xiphydriidae|[3529]|RRMFG2547-15|588[1n]bp|Hymenoptera|Xiphydriidae|BOLD:ABY1179
Xiphydriidae|[3530]|RRINV805-15|630[0n]bp|Hymenoptera|Xiphydriidae|
Sapyga centrata|[3531]|RRMFC2061-15|579[0n]bp|Hymenoptera|Sapygidae|BOLD:ACL7820
Ponera pennsylvanica|[3532]|RRBGA028-15|582[1n]bp|Hymenoptera|Formicidae|BOLD:AAF0443
Ponerinae|[3533]|RRINV153-15|609[0n]bp|Hymenoptera|Formicidae|
Camponotus herculeanus|[3534]|RRMFB944-15|618[0n]bp|Hymenoptera|Formicidae|BOLD:AAA2372
Camponotus pennsylvanicus|[3535]|RRSSA1013-15|582[0n]bp|Hymenoptera|Formicidae|BOLD:AAA9461
Camponotus nearcticus|[3536]|RRMFC1784-15|588[0n]bp|Hymenoptera|Formicidae|BOLD:AAD4432
Myrmica|[3537]|RRINV384-15|608[0n]bp|Hymenoptera|Formicidae|
Myrmica|[3538]|RRINV383-15|607[1n]bp|Hymenoptera|Formicidae|
Prenolepis imparis|[3539]|RRMPB4179-15|522[0n]bp|Hymenoptera|Formicidae|BOLD:AAC1302
Formica|[3540]|RRMFD459-15|576[0n]bp|Hymenoptera|Formicidae|BOLD:AAA1467
Formica lasioides|[3541]|RRMFE2801-15|591[0n]bp|Hymenoptera|Formicidae|BOLD:AAE0406
Lasius|[3542]|RRINV754-15|623[0n]bp|Hymenoptera|Formicidae|
Lasius alienus|[3543]|RRSSA2233-15|588[0n]bp|Hymenoptera|Formicidae|BOLD:AAA9049
Lasius neoniger|[3544]|RRMPB4182-15|603[0n]bp|Hymenoptera|Formicidae|BOLD:AAB9126
Lasius nearcticus|[3545]|RRSSA2636-15|612[0n]bp|Hymenoptera|Formicidae|BOLD:AAD1528
Lasius|[3546]|RRINV782-15|565[5n]bp|Hymenoptera|Formicidae|
Lasius neoniger|[3547]|RRINV412-15|610[0n]bp|Hymenoptera|Formicidae|
Lasius claviger|[3548]|RRINV158-15|633[0n]bp|Hymenoptera|Formicidae|
Lasius|[3549]|RRINV455-15|612[0n]bp|Hymenoptera|Formicidae|
Lasius|[3550]|RRINV832-15|632[0n]bp|Hymenoptera|Formicidae|
Temnothorax ambiguus|[3551]|RRBAA200-15|582[0n]bp|Hymenoptera|Formicidae|BOLD:AAG0685
Stenamma brevicorne|[3552]|RRMFE2155-15|579[2n]bp|Hymenoptera|Formicidae|BOLD:AAH7068
Aphaenogaster|[3553]|RRINV111-15|621[0n]bp|Hymenoptera|Formicidae|
Aphaenogaster|[3554]|RRINV393-15|603[0n]bp|Hymenoptera|Formicidae|
Tapinoma sessile|[3555]|RRINV753-15|603[0n]bp|Hymenoptera|Formicidae|
Tapinoma sessile|[3556]|RRMFG2840-15|585[0n]bp|Hymenoptera|Formicidae|BOLD:AAA3900
Tapinoma sessile|[3557]|RRMFB895-15|576[1n]bp|Hymenoptera|Formicidae|BOLD:AAA3893
Tapinoma|[3558]|RRINV287-15|633[0n]bp|Hymenoptera|Formicidae|
Cylloceria melancholica|[3559]|RRINV462-15|612[0n]bp|Hymenoptera|Ichneumonidae|
Exenterus|[3560]|RRMFE3065-15|636[0n]bp|Hymenoptera|Ichneumonidae|BOLD:AAL5084
Mesochorus|[3561]|RRSSA290-15|588[0n]bp|Hymenoptera|Ichneumonidae|BOLD:AAM7412
Mesochorus suomiensis|[3562]|RRMFG2462-15|603[0n]bp|Hymenoptera|Ichneumonidae|BOLD:AAZ1979
Mesochorus|[3563]|RRMFE2426-15|552[0n]bp|Hymenoptera|Ichneumonidae|BOLD:ACG5789
Tersilochinae|[3564]|RRMFI1824-15|636[2n]bp|Hymenoptera|Ichneumonidae|BOLD:ABA9842
Tersilochinae|[3565]|RRMPB4190-15|543[1n]bp|Hymenoptera|Ichneumonidae|BOLD:AAU8481
Tersilochinae|[3566]|RRMFE2108-15|591[0n]bp|Hymenoptera|Ichneumonidae|BOLD:ACL9152
Tersilochinae|[3567]|RRMPD484-15|594[0n]bp|Hymenoptera|Ichneumonidae|BOLD:AAU8328
Tersilochinae|[3568]|RRMFE2626-15|573[0n]bp|Hymenoptera|Ichneumonidae|BOLD:ACV2273
Tersilochinae|[3569]|RRSSA338-15|564[0n]bp|Hymenoptera|Ichneumonidae|BOLD:ACV3286
Tersilochinae|[3570]|RRMPD474-15|555[0n]bp|Hymenoptera|Ichneumonidae|BOLD:ABA6274
Ichneumonidae|[3571]|RRMFE2133-15|555[0n]bp|Hymenoptera|Ichneumonidae|BOLD:ACH0608
Ichneumonidae|[3572]|RRMFE2147-15|588[0n]bp|Hymenoptera|Ichneumonidae|BOLD:ACV3460
Orthocentrus fulvipes|[3573]|RRMFE2154-15|537[0n]bp|Hymenoptera|Ichneumonidae|BOLD:AAM9125
Orthocentrus|[3574]|RRMFE2797-15|585[0n]bp|Hymenoptera|Ichneumonidae|BOLD:AAB1188
Stenomacrus|[3575]|RRMFE1177-15|561[0n]bp|Hymenoptera|Ichneumonidae|BOLD:AAU8210
Orthocentrus|[3576]|RRMFC1821-15|591[0n]bp|Hymenoptera|Ichneumonidae|BOLD:AAU8604
Stenomacrus|[3577]|RRMFG2258-15|558[0n]bp|Hymenoptera|Ichneumonidae|BOLD:AAJ5372
Stenomacrus nemoralis|[3578]|RRMFC1827-15|576[0n]bp|Hymenoptera|Ichneumonidae|BOLD:AAM7494
Stenomacrus|[3579]|RRMFG2259-15|582[0n]bp|Hymenoptera|Ichneumonidae|BOLD:AAP6689
Orthocentrinae|[3580]|RRSSA293-15|543[0n]bp|Hymenoptera|Ichneumonidae|BOLD:AAU8746
Orthocentrinae|[3581]|RRMPD485-15|564[0n]bp|Hymenoptera|Ichneumonidae|BOLD:ACB2448
Leipaulus|[3582]|RRINV775-15|633[0n]bp|Hymenoptera|Ichneumonidae|
Orthocentrinae|[3583]|RRMFG2522-15|552[0n]bp|Hymenoptera|Ichneumonidae|BOLD:AAG0973
Orthocentrinae|[3584]|RRMFE1053-15|588[0n]bp|Hymenoptera|Ichneumonidae|BOLD:ACD9088
Phytodietus|[3585]|RRMPD033-15|558[0n]bp|Hymenoptera|Ichneumonidae|BOLD:AAG0379
Podoschistus vittifrons|[3586]|RRMFE3047-15|636[0n]bp|Hymenoptera|Ichneumonidae|BOLD:AAL0380
Dolichomitus irritator|[3587]|RRMFD542-15|594[0n]bp|Hymenoptera|Ichneumonidae|BOLD:AAU8680
Diplazon|[3588]|RRSSA464-15|579[0n]bp|Hymenoptera|Ichneumonidae|BOLD:AAD1879
Diplazon laetatorius|[3589]|RRMFG2461-15|588[0n]bp|Hymenoptera|Ichneumonidae|BOLD:AAD4214
Pimpla|[3590]|RRMFE2838-15|591[0n]bp|Hymenoptera|Ichneumonidae|BOLD:AAD5194
Pimpla|[3591]|RRMFG2814-15|588[0n]bp|Hymenoptera|Ichneumonidae|BOLD:AAG0387
Pimpla aequalis|[3592]|RRMFE2800-15|588[0n]bp|Hymenoptera|Ichneumonidae|BOLD:AAG7634
Pimplinae|[3593]|RRINV797-15|627[0n]bp|Hymenoptera|Ichneumonidae|
Coleocentrus|[3594]|RRMFD549-15|582[0n]bp|Hymenoptera|Ichneumonidae|BOLD:AAJ6986
Iseropus stercorator|[3595]|RRMFC2075-15|591[0n]bp|Hymenoptera|Ichneumonidae|BOLD:AAO2094
Pimpla|[3596]|RRINV783-15|630[0n]bp|Hymenoptera|Ichneumonidae|
Orthocentrinae|[3597]|RRMFG2962-15|561[0n]bp|Hymenoptera|Ichneumonidae|BOLD:AAG0953
Megastylus|[3598]|RRMFE2429-15|588[0n]bp|Hymenoptera|Ichneumonidae|BOLD:ACE4233
Diplazontinae|[3599]|RRMFE2624-15|585[0n]bp|Hymenoptera|Ichneumonidae|BOLD:AAU8212
Sussaba|[3600]|RRMFE2823-15|573[0n]bp|Hymenoptera|Ichneumonidae|BOLD:AAH1764
Zaglyptus varipes|[3601]|RRINV1243-15|624[0n]bp|Hymenoptera|Ichneumonidae|
Tryphoninae|[3602]|RRMFD1536-15|549[0n]bp|Hymenoptera|Ichneumonidae|BOLD:AAU8749
Tryphoninae|[3603]|RRMFG2828-15|588[0n]bp|Hymenoptera|Ichneumonidae|BOLD:AAM7520
Polyblastus|[3604]|RRMFE2966-15|588[0n]bp|Hymenoptera|Ichneumonidae|BOLD:ACF4348
Odontocolon|[3605]|RRSSA465-15|588[0n]bp|Hymenoptera|Ichneumonidae|BOLD:AAG7690
Odontocolon|[3606]|RRSSA3848-15|640[1n]bp|Hymenoptera|Ichneumonidae|BOLD:ACL3751
Ichneumonidae|[3607]|RRINV1462-15|658[0n]bp|Hymenoptera|Ichneumonidae|
Stictopisthus|[3608]|RRMFE2842-15|591[0n]bp|Hymenoptera|Ichneumonidae|BOLD:AAA5522
Ichneumonidae|[3609]|RRMFG2964-15|516[0n]bp|Hymenoptera|Ichneumonidae|
Bethylidae|[3610]|RRMFC1804-15|540[0n]bp|Hymenoptera|Bethylidae|BOLD:ABY2749
Ichneumonidae|[3611]|RRSSA3845-15|638[1n]bp|Hymenoptera|Ichneumonidae|BOLD:AAY6844
Ichneumonidae|[3612]|RRSSA288-15|555[0n]bp|Hymenoptera|Ichneumonidae|BOLD:ACV5004
Eridolius|[3613]|RRMFE2841-15|573[0n]bp|Hymenoptera|Ichneumonidae|BOLD:ACN9868
Ichneumonidae|[3614]|RRMFG2528-15|573[0n]bp|Hymenoptera|Ichneumonidae|BOLD:ACV5916
Ichneumonidae|[3615]|RRMFE2969-15|600[6n]bp|Hymenoptera|Ichneumonidae|
Exochus nigripalpis|[3616]|RRMFE2840-15|588[0n]bp|Hymenoptera|Ichneumonidae|BOLD:AAG7713
Xoridinae|[3617]|RRMFD447-15|576[0n]bp|Hymenoptera|Ichneumonidae|BOLD:AAZ0238
Ichneumonidae|[3618]|RRINV781-15|595[0n]bp|Hymenoptera|Ichneumonidae|
Ichneumonidae|[3619]|RRMFG2819-15|588[0n]bp|Hymenoptera|Ichneumonidae|BOLD:ACV5377
Ichneumonidae|[3620]|RRINV448-15|609[0n]bp|Hymenoptera|Ichneumonidae|
Ichneumonidae|[3621]|RRMFG3153-15|582[0n]bp|Hymenoptera|Ichneumonidae|BOLD:ACV5720
Campopleginae|[3622]|RRMFG2797-15|591[0n]bp|Hymenoptera|Ichneumonidae|BOLD:AAG7642
Campopleginae|[3623]|RRINV903-15|634[0n]bp|Hymenoptera|Ichneumonidae|
Ichneumonidae|[3624]|RRMFG2858-15|585[0n]bp|Hymenoptera|Ichneumonidae|BOLD:ACU0916
Tranosema rostrale|[3625]|RRINV765-15|633[0n]bp|Hymenoptera|Ichneumonidae|
Ichneumonidae|[3626]|RRSSA1018-15|588[0n]bp|Hymenoptera|Ichneumonidae|BOLD:ABA5942
Campopleginae|[3627]|RRMFD450-15|588[0n]bp|Hymenoptera|Ichneumonidae|BOLD:ABA6297

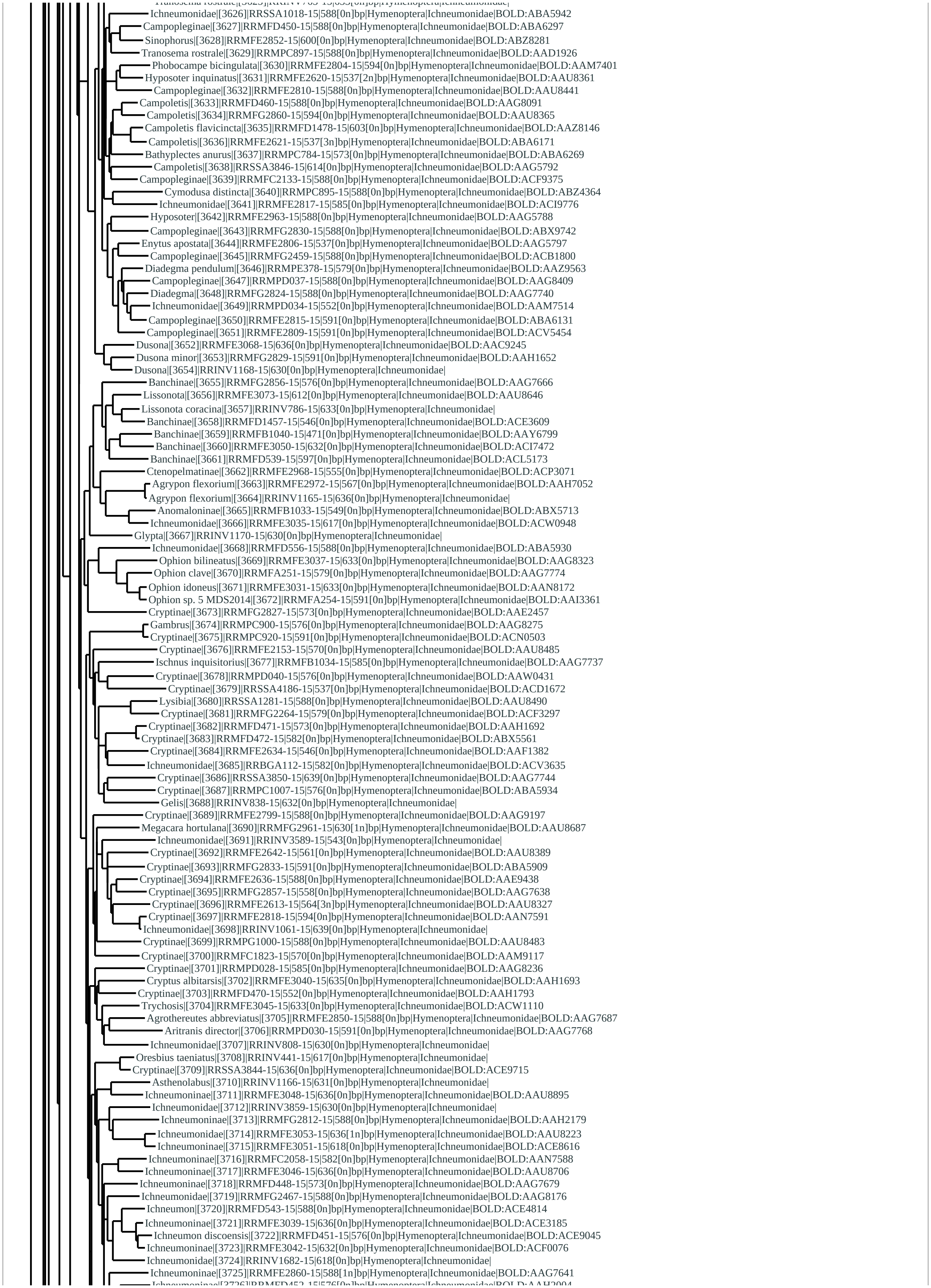

Ichneumonidae|[3626]|RRSSA1018-15|588[0n]bp|Hymenoptera|Ichneumonidae|BOLD:ABA5942
Campopleginae|[3627]|RRMFD450-15|588[0n]bp|Hymenoptera|Ichneumonidae|BOLD:ABA6297
Sinophorus|[3628]|RRMFE2852-15|600[0n]bp|Hymenoptera|Ichneumonidae|BOLD:ABZ8281
Tranosema rostrale|[3629]|RRMPC897-15|588[0n]bp|Hymenoptera|Ichneumonidae|BOLD:AAD1926
Phobocampe bicingulata|[3630]|RRMFE2804-15|594[0n]bp|Hymenoptera|Ichneumonidae|BOLD:AAM7401
Hyposoter inquinatus|[3631]|RRMFE2620-15|537[2n]bp|Hymenoptera|Ichneumonidae|BOLD:AAU8361
Campopleginae|[3632]|RRMFE2810-15|588[0n]bp|Hymenoptera|Ichneumonidae|BOLD:AAU8441
Campoletis|[3633]|RRMFD460-15|588[0n]bp|Hymenoptera|Ichneumonidae|BOLD:AAG8091
Campoletis|[3634]|RRMFG2860-15|594[0n]bp|Hymenoptera|Ichneumonidae|BOLD:AAU8365
Campoletis flavicincta|[3635]|RRMFD1478-15|603[0n]bp|Hymenoptera|Ichneumonidae|BOLD:AAZ8146
Campoletis|[3636]|RRMFE2621-15|537[3n]bp|Hymenoptera|Ichneumonidae|BOLD:ABA6171
Bathyplectes anurus|[3637]|RRMPC784-15|573[0n]bp|Hymenoptera|Ichneumonidae|BOLD:ABA6269
Campoletis|[3638]|RRSSA3846-15|614[0n]bp|Hymenoptera|Ichneumonidae|BOLD:AAG5792
Campopleginae|[3639]|RRMFC2133-15|588[0n]bp|Hymenoptera|Ichneumonidae|BOLD:ACF9375
Cymodusa distincta|[3640]|RRMPC895-15|588[0n]bp|Hymenoptera|Ichneumonidae|BOLD:ABZ4364
Ichneumonidae|[3641]|RRMFE2817-15|585[0n]bp|Hymenoptera|Ichneumonidae|BOLD:ACI9776
Hyposoter|[3642]|RRMFE2963-15|588[0n]bp|Hymenoptera|Ichneumonidae|BOLD:AAG5788
Campopleginae|[3643]|RRMFG2830-15|588[0n]bp|Hymenoptera|Ichneumonidae|BOLD:ABX9742
Enytus apostata|[3644]|RRMFE2806-15|537[0n]bp|Hymenoptera|Ichneumonidae|BOLD:AAG5797
Campopleginae|[3645]|RRMFG2459-15|588[0n]bp|Hymenoptera|Ichneumonidae|BOLD:ACB1800
Diadegma pendulum|[3646]|RRMPE378-15|579[0n]bp|Hymenoptera|Ichneumonidae|BOLD:AAZ9563
Campopleginae|[3647]|RRMPD037-15|588[0n]bp|Hymenoptera|Ichneumonidae|BOLD:AAG8409
Diadegma|[3648]|RRMFG2824-15|588[0n]bp|Hymenoptera|Ichneumonidae|BOLD:AAG7740
Ichneumonidae|[3649]|RRMPD034-15|552[0n]bp|Hymenoptera|Ichneumonidae|BOLD:AAM7514
Campopleginae|[3650]|RRMFE2815-15|591[0n]bp|Hymenoptera|Ichneumonidae|BOLD:ABA6131
Campopleginae|[3651]|RRMFE2809-15|591[0n]bp|Hymenoptera|Ichneumonidae|BOLD:ACV5454
Dusona|[3652]|RRMFE3068-15|636[0n]bp|Hymenoptera|Ichneumonidae|BOLD:AAC9245
Dusona minor|[3653]|RRMFG2829-15|591[0n]bp|Hymenoptera|Ichneumonidae|BOLD:AAH1652
Dusona|[3654]|RRINV1168-15|630[0n]bp|Hymenoptera|Ichneumonidae|
Banchinae|[3655]|RRMFG2856-15|576[0n]bp|Hymenoptera|Ichneumonidae|BOLD:AAG7666
Lissonota|[3656]|RRMFE3073-15|612[0n]bp|Hymenoptera|Ichneumonidae|BOLD:AAU8646
Lissonota coracina|[3657]|RRINV786-15|633[0n]bp|Hymenoptera|Ichneumonidae|
Banchinae|[3658]|RRMFD1457-15|546[0n]bp|Hymenoptera|Ichneumonidae|BOLD:ACE3609
Banchinae|[3659]|RRMFB1040-15|471[0n]bp|Hymenoptera|Ichneumonidae|BOLD:AAY6799
Banchinae|[3660]|RRMFE3050-15|632[0n]bp|Hymenoptera|Ichneumonidae|BOLD:ACI7472
Banchinae|[3661]|RRMFD539-15|597[0n]bp|Hymenoptera|Ichneumonidae|BOLD:ACL5173
Ctenopelmatinae|[3662]|RRMFE2968-15|555[0n]bp|Hymenoptera|Ichneumonidae|BOLD:ACP3071
Agrypon flexorium|[3663]|RRMFE2972-15|567[0n]bp|Hymenoptera|Ichneumonidae|BOLD:AAH7052
Agrypon flexorium|[3664]|RRINV1165-15|636[0n]bp|Hymenoptera|Ichneumonidae|
Anomaloninae|[3665]|RRMFB1033-15|549[0n]bp|Hymenoptera|Ichneumonidae|BOLD:ABX5713
Ichneumonidae|[3666]|RRMFE3035-15|617[0n]bp|Hymenoptera|Ichneumonidae|BOLD:ACW0948
Glypta|[3667]|RRINV1170-15|630[0n]bp|Hymenoptera|Ichneumonidae|
Ichneumonidae|[3668]|RRMFD556-15|588[0n]bp|Hymenoptera|Ichneumonidae|BOLD:ABA5930
Ophion bilineatus|[3669]|RRMFE3037-15|633[0n]bp|Hymenoptera|Ichneumonidae|BOLD:AAG8323
Ophion clave|[3670]|RRMFA251-15|579[0n]bp|Hymenoptera|Ichneumonidae|BOLD:AAG7774
Ophion idoneus|[3671]|RRMFE3031-15|633[0n]bp|Hymenoptera|Ichneumonidae|BOLD:AAN8172
Ophion sp. 5 MDS2014|[3672]|RRMFA254-15|591[0n]bp|Hymenoptera|Ichneumonidae|BOLD:AAI3361
Cryptinae|[3673]|RRMFG2827-15|573[0n]bp|Hymenoptera|Ichneumonidae|BOLD:AAE2457
Gambrus|[3674]|RRMPC900-15|576[0n]bp|Hymenoptera|Ichneumonidae|BOLD:AAG8275
Cryptinae|[3675]|RRMPC920-15|591[0n]bp|Hymenoptera|Ichneumonidae|BOLD:ACN0503
Cryptinae|[3676]|RRMFE2153-15|570[0n]bp|Hymenoptera|Ichneumonidae|BOLD:AAU8485
Ischnus inquisitorius|[3677]|RRMFB1034-15|585[0n]bp|Hymenoptera|Ichneumonidae|BOLD:AAG7737
Cryptinae|[3678]|RRMPD040-15|576[0n]bp|Hymenoptera|Ichneumonidae|BOLD:AAW0431
Cryptinae|[3679]|RRSSA4186-15|537[0n]bp|Hymenoptera|Ichneumonidae|BOLD:ACD1672
Lysibia|[3680]|RRSSA1281-15|588[0n]bp|Hymenoptera|Ichneumonidae|BOLD:AAU8490
Cryptinae|[3681]|RRMFG2264-15|579[0n]bp|Hymenoptera|Ichneumonidae|BOLD:ACF3297
Cryptinae|[3682]|RRMFD471-15|573[0n]bp|Hymenoptera|Ichneumonidae|BOLD:AAH1692
Cryptinae|[3683]|RRMFD472-15|582[0n]bp|Hymenoptera|Ichneumonidae|BOLD:ABX5561
Cryptinae|[3684]|RRMFE2634-15|546[0n]bp|Hymenoptera|Ichneumonidae|BOLD:AAF1382
Ichneumonidae|[3685]|RRBGA112-15|582[0n]bp|Hymenoptera|Ichneumonidae|BOLD:ACV3635
Cryptinae|[3686]|RRSSA3850-15|639[0n]bp|Hymenoptera|Ichneumonidae|BOLD:AAG7744
Cryptinae|[3687]|RRMPC1007-15|576[0n]bp|Hymenoptera|Ichneumonidae|BOLD:ABA5934
Gelis|[3688]|RRINV838-15|632[0n]bp|Hymenoptera|Ichneumonidae|
Cryptinae|[3689]|RRMFE2799-15|588[0n]bp|Hymenoptera|Ichneumonidae|BOLD:AAG9197
Megacara hortulana|[3690]|RRMFG2961-15|630[1n]bp|Hymenoptera|Ichneumonidae|BOLD:AAU8687
Ichneumonidae|[3691]|RRINV3589-15|543[0n]bp|Hymenoptera|Ichneumonidae|
Cryptinae|[3692]|RRMFE2642-15|561[0n]bp|Hymenoptera|Ichneumonidae|BOLD:AAU8389
Cryptinae|[3693]|RRMFG2833-15|591[0n]bp|Hymenoptera|Ichneumonidae|BOLD:ABA5909
Cryptinae|[3694]|RRMFE2636-15|588[0n]bp|Hymenoptera|Ichneumonidae|BOLD:AAE9438
Cryptinae|[3695]|RRMFG2857-15|558[0n]bp|Hymenoptera|Ichneumonidae|BOLD:AAG7638
Cryptinae|[3696]|RRMFE2613-15|564[3n]bp|Hymenoptera|Ichneumonidae|BOLD:AAU8327
Cryptinae|[3697]|RRMFE2818-15|594[0n]bp|Hymenoptera|Ichneumonidae|BOLD:AAN7591
Ichneumonidae|[3698]|RRINV1061-15|639[0n]bp|Hymenoptera|Ichneumonidae|
Cryptinae|[3699]|RRMPG1000-15|588[0n]bp|Hymenoptera|Ichneumonidae|BOLD:AAU8483
Cryptinae|[3700]|RRMFC1823-15|570[0n]bp|Hymenoptera|Ichneumonidae|BOLD:AAM9117
Cryptinae|[3701]|RRMPD028-15|585[0n]bp|Hymenoptera|Ichneumonidae|BOLD:AAG8236
Cryptus albitarsis|[3702]|RRMFE3040-15|635[0n]bp|Hymenoptera|Ichneumonidae|BOLD:AAH1693
Cryptinae|[3703]|RRMFD470-15|552[0n]bp|Hymenoptera|Ichneumonidae|BOLD:AAH1793
Trychosis|[3704]|RRMFE3045-15|633[0n]bp|Hymenoptera|Ichneumonidae|BOLD:ACW1110
Agrothereutes abbreviatus|[3705]|RRMFE2850-15|588[0n]bp|Hymenoptera|Ichneumonidae|BOLD:AAG7687
Aritranis director|[3706]|RRMPD030-15|591[0n]bp|Hymenoptera|Ichneumonidae|BOLD:AAG7768
Ichneumonidae|[3707]|RRINV808-15|630[0n]bp|Hymenoptera|Ichneumonidae|
Oresbius taeniatus|[3708]|RRINV441-15|617[0n]bp|Hymenoptera|Ichneumonidae|
Cryptinae|[3709]|RRSSA3844-15|636[0n]bp|Hymenoptera|Ichneumonidae|BOLD:ACE9715
Asthenolabus|[3710]|RRINV1166-15|631[0n]bp|Hymenoptera|Ichneumonidae|
Ichneumoninae|[3711]|RRMFE3048-15|636[0n]bp|Hymenoptera|Ichneumonidae|BOLD:AAU8895
Ichneumonidae|[3712]|RRINV3859-15|630[0n]bp|Hymenoptera|Ichneumonidae|
Ichneumoninae|[3713]|RRMFG2812-15|588[0n]bp|Hymenoptera|Ichneumonidae|BOLD:AAH2179
Ichneumonidae|[3714]|RRMFE3053-15|636[1n]bp|Hymenoptera|Ichneumonidae|BOLD:AAU8223
Ichneumoninae|[3715]|RRMFE3051-15|618[0n]bp|Hymenoptera|Ichneumonidae|BOLD:ACE8616
Ichneumoninae|[3716]|RRMFC2058-15|582[0n]bp|Hymenoptera|Ichneumonidae|BOLD:AAN7588
Ichneumoninae|[3717]|RRMFE3046-15|636[0n]bp|Hymenoptera|Ichneumonidae|BOLD:AAU8706
Ichneumoninae|[3718]|RRMFD448-15|573[0n]bp|Hymenoptera|Ichneumonidae|BOLD:AAG7679
Ichneumonidae|[3719]|RRMFG2467-15|588[0n]bp|Hymenoptera|Ichneumonidae|BOLD:AAG8176
Ichneumon|[3720]|RRMFD543-15|588[0n]bp|Hymenoptera|Ichneumonidae|BOLD:ACE4814
Ichneumoninae|[3721]|RRMFE3039-15|636[0n]bp|Hymenoptera|Ichneumonidae|BOLD:ACE3185
Ichneumon discoensis|[3722]|RRMFD451-15|576[0n]bp|Hymenoptera|Ichneumonidae|BOLD:ACE9045
Ichneumoninae|[3723]|RRMFE3042-15|632[0n]bp|Hymenoptera|Ichneumonidae|BOLD:ACF0076
Ichneumonidae|[3724]|RRINV1682-15|618[0n]bp|Hymenoptera|Ichneumonidae|
Ichneumoninae|[3725]|RRMFE2860-15|588[1n]bp|Hymenoptera|Ichneumonidae|BOLD:AAG7641

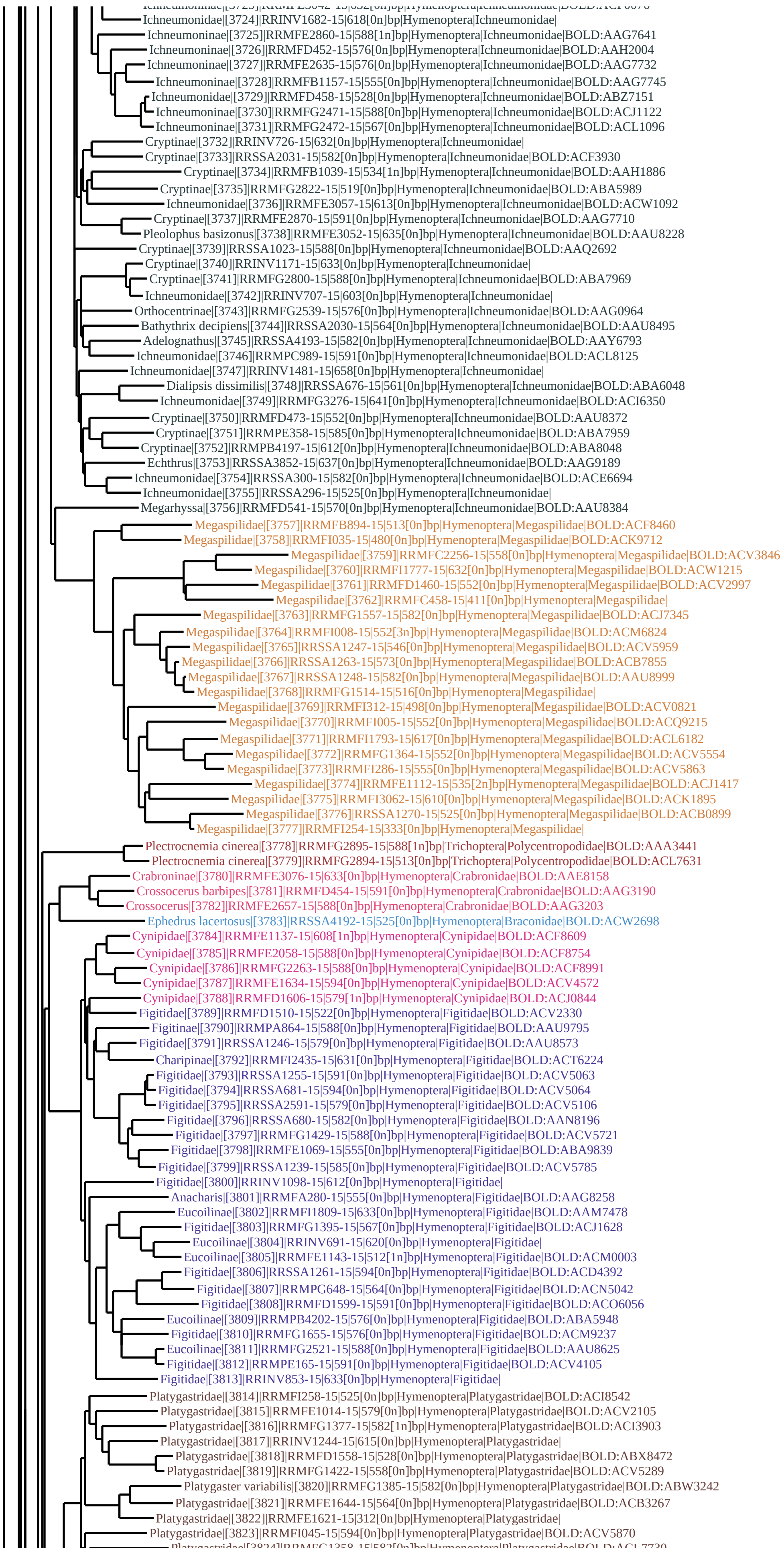
Ichneumonidae|[3724]|RRINV1682-15|618[0n]bp|Hymenoptera|Ichneumonidae|
Ichneumoninae|[3725]|RRMFE2860-15|588[1n]bp|Hymenoptera|Ichneumonidae|BOLD:AAG7641
Ichneumoninae|[3726]|RRMFD452-15|576[0n]bp|Hymenoptera|Ichneumonidae|BOLD:AAH2004
Ichneumoninae|[3727]|RRMFE2635-15|576[0n]bp|Hymenoptera|Ichneumonidae|BOLD:AAG7732
Ichneumoninae|[3728]|RRMFB1157-15|555[0n]bp|Hymenoptera|Ichneumonidae|BOLD:AAG7745
Ichneumoninae|[3729]|RRMFD458-15|528[0n]bp|Hymenoptera|Ichneumonidae|BOLD:ABZ7151
Ichneumoninae|[3730]|RRMFG2471-15|588[0n]bp|Hymenoptera|Ichneumonidae|BOLD:ACJ1122
Ichneumoninae|[3731]|RRMFG2472-15|567[0n]bp|Hymenoptera|Ichneumonidae|BOLD:ACL1096
Cryptinae|[3732]|RRINV726-15|632[0n]bp|Hymenoptera|Ichneumonidae|
Cryptinae|[3733]|RRSSA2031-15|582[0n]bp|Hymenoptera|Ichneumonidae|BOLD:ACF3930
Cryptinae|[3734]|RRMFB1039-15|534[1n]bp|Hymenoptera|Ichneumonidae|BOLD:AAH1886
Cryptinae|[3735]|RRMFG2822-15|519[0n]bp|Hymenoptera|Ichneumonidae|BOLD:ABA5989
Ichneumonidae|[3736]|RRMFE3057-15|613[0n]bp|Hymenoptera|Ichneumonidae|BOLD:ACW1092
Cryptinae|[3737]|RRMFE2870-15|591[0n]bp|Hymenoptera|Ichneumonidae|BOLD:AAG7710
Pleolophus basizonus|[3738]|RRMFE3052-15|635[0n]bp|Hymenoptera|Ichneumonidae|BOLD:AAU8228
Cryptinae|[3739]|RRSSA1023-15|588[0n]bp|Hymenoptera|Ichneumonidae|BOLD:AAQ2692
Cryptinae|[3740]|RRINV1171-15|633[0n]bp|Hymenoptera|Ichneumonidae|
Cryptinae|[3741]|RRMFG2800-15|588[0n]bp|Hymenoptera|Ichneumonidae|BOLD:ABA7969
Ichneumonidae|[3742]|RRINV707-15|603[0n]bp|Hymenoptera|Ichneumonidae|
Orthocentrinae|[3743]|RRMFG2539-15|576[0n]bp|Hymenoptera|Ichneumonidae|BOLD:AAG0964
Bathythrix decipiens|[3744]|RRSSA2030-15|564[0n]bp|Hymenoptera|Ichneumonidae|BOLD:AAU8495
Adelognathus|[3745]|RRSSA4193-15|582[0n]bp|Hymenoptera|Ichneumonidae|BOLD:AAY6793
Ichneumonidae|[3746]|RRMPC989-15|591[0n]bp|Hymenoptera|Ichneumonidae|BOLD:ACL8125
Ichneumonidae|[3747]|RRINV1481-15|658[0n]bp|Hymenoptera|Ichneumonidae|
Dialipsis dissimilis|[3748]|RRSSA676-15|561[0n]bp|Hymenoptera|Ichneumonidae|BOLD:ABA6048
Ichneumonidae|[3749]|RRMFG3276-15|641[0n]bp|Hymenoptera|Ichneumonidae|BOLD:ACI6350
Cryptinae|[3750]|RRMFD473-15|552[0n]bp|Hymenoptera|Ichneumonidae|BOLD:AAU8372
Cryptinae|[3751]|RRMPE358-15|585[0n]bp|Hymenoptera|Ichneumonidae|BOLD:ABA7959
Cryptinae|[3752]|RRMPB4197-15|612[0n]bp|Hymenoptera|Ichneumonidae|BOLD:ABA8048
Echthrus|[3753]|RRSSA3852-15|637[0n]bp|Hymenoptera|Ichneumonidae|BOLD:AAG9189
Ichneumonidae|[3754]|RRSSA300-15|582[0n]bp|Hymenoptera|Ichneumonidae|BOLD:ACE6694
Ichneumonidae|[3755]|RRSSA296-15|525[0n]bp|Hymenoptera|Ichneumonidae|
Megarhyssa|[3756]|RRMFD541-15|570[0n]bp|Hymenoptera|Ichneumonidae|BOLD:AAU8384
Megaspilidae|[3757]|RRMFB894-15|513[0n]bp|Hymenoptera|Megaspilidae|BOLD:ACF8460
Megaspilidae|[3758]|RRMFI035-15|480[0n]bp|Hymenoptera|Megaspilidae|BOLD:ACK9712
Megaspilidae|[3759]|RRMFC2256-15|558[0n]bp|Hymenoptera|Megaspilidae|BOLD:ACV3846
Megaspilidae|[3760]|RRMFI1777-15|632[0n]bp|Hymenoptera|Megaspilidae|BOLD:ACW1215
Megaspilidae|[3761]|RRMFD1460-15|552[0n]bp|Hymenoptera|Megaspilidae|BOLD:ACV2997
Megaspilidae|[3762]|RRMFC458-15|411[0n]bp|Hymenoptera|Megaspilidae|
Megaspilidae|[3763]|RRMFG1557-15|582[0n]bp|Hymenoptera|Megaspilidae|BOLD:ACJ7345
Megaspilidae|[3764]|RRMFI008-15|552[3n]bp|Hymenoptera|Megaspilidae|BOLD:ACM6824
Megaspilidae|[3765]|RRSSA1247-15|546[0n]bp|Hymenoptera|Megaspilidae|BOLD:ACV5959
Megaspilidae|[3766]|RRSSA1263-15|573[0n]bp|Hymenoptera|Megaspilidae|BOLD:ACB7855
Megaspilidae|[3767]|RRSSA1248-15|582[0n]bp|Hymenoptera|Megaspilidae|BOLD:AAU8999
Megaspilidae|[3768]|RRMFG1514-15|516[0n]bp|Hymenoptera|Megaspilidae|
Megaspilidae|[3769]|RRMFI312-15|498[0n]bp|Hymenoptera|Megaspilidae|BOLD:ACV0821
Megaspilidae|[3770]|RRMFI005-15|552[0n]bp|Hymenoptera|Megaspilidae|BOLD:ACQ9215
Megaspilidae|[3771]|RRMFI1793-15|617[0n]bp|Hymenoptera|Megaspilidae|BOLD:ACL6182
Megaspilidae|[3772]|RRMFG1364-15|552[0n]bp|Hymenoptera|Megaspilidae|BOLD:ACV5554
Megaspilidae|[3773]|RRMFI286-15|555[0n]bp|Hymenoptera|Megaspilidae|BOLD:ACV5863
Megaspilidae|[3774]|RRMFE1112-15|535[2n]bp|Hymenoptera|Megaspilidae|BOLD:ACJ1417
Megaspilidae|[3775]|RRMFI3062-15|610[0n]bp|Hymenoptera|Megaspilidae|BOLD:ACK1895
Megaspilidae|[3776]|RRSSA1270-15|525[0n]bp|Hymenoptera|Megaspilidae|BOLD:ACB0899
Megaspilidae|[3777]|RRMFI254-15|333[0n]bp|Hymenoptera|Megaspilidae|
Plectrocnemia cinerea|[3778]|RRMFG2895-15|588[1n]bp|Trichoptera|Polycentropodidae|BOLD:AAA3441
Plectrocnemia cinerea|[3779]|RRMFG2894-15|513[0n]bp|Trichoptera|Polycentropodidae|BOLD:ACL7631
Crabroninae|[3780]|RRMFE3076-15|633[0n]bp|Hymenoptera|Crabronidae|BOLD:AAE8158
Crossocerus barbipes|[3781]|RRMFD454-15|591[0n]bp|Hymenoptera|Crabronidae|BOLD:AAG3190
Crossocerus|[3782]|RRMFE2657-15|588[0n]bp|Hymenoptera|Crabronidae|BOLD:AAG3203
Ephedrus lacertosus|[3783]|RRSSA4192-15|525[0n]bp|Hymenoptera|Braconidae|BOLD:ACW2698
Cynipidae|[3784]|RRMFE1137-15|608[1n]bp|Hymenoptera|Cynipidae|BOLD:ACF8609
Cynipidae|[3785]|RRMFE2058-15|588[0n]bp|Hymenoptera|Cynipidae|BOLD:ACF8754
Cynipidae|[3786]|RRMFG2263-15|588[0n]bp|Hymenoptera|Cynipidae|BOLD:ACF8991
Cynipidae|[3787]|RRMFE1634-15|594[0n]bp|Hymenoptera|Cynipidae|BOLD:ACV4572
Cynipidae|[3788]|RRMFD1606-15|579[1n]bp|Hymenoptera|Cynipidae|BOLD:ACJ0844
Figitidae|[3789]|RRMFD1510-15|522[0n]bp|Hymenoptera|Figitidae|BOLD:ACV2330
Figitinae|[3790]|RRMPA864-15|588[0n]bp|Hymenoptera|Figitidae|BOLD:AAU9795
Figitidae|[3791]|RRSSA1246-15|579[0n]bp|Hymenoptera|Figitidae|BOLD:AAU8573
Charipinae|[3792]|RRMFI2435-15|631[0n]bp|Hymenoptera|Figitidae|BOLD:ACT6224
Figitidae|[3793]|RRSSA1255-15|591[0n]bp|Hymenoptera|Figitidae|BOLD:ACV5063
Figitidae|[3794]|RRSSA681-15|594[0n]bp|Hymenoptera|Figitidae|BOLD:ACV5064
Figitidae|[3795]|RRSSA2591-15|579[0n]bp|Hymenoptera|Figitidae|BOLD:ACV5106
Figitidae|[3796]|RRSSA680-15|582[0n]bp|Hymenoptera|Figitidae|BOLD:AAN8196
Figitidae|[3797]|RRMFG1429-15|588[0n]bp|Hymenoptera|Figitidae|BOLD:ACV5721
Figitidae|[3798]|RRMFE1069-15|555[0n]bp|Hymenoptera|Figitidae|BOLD:ABA9839
Figitidae|[3799]|RRSSA1239-15|585[0n]bp|Hymenoptera|Figitidae|BOLD:ACV5785
Figitidae|[3800]|RRINV1098-15|612[0n]bp|Hymenoptera|Figitidae|
Anacharis|[3801]|RRMFA280-15|555[0n]bp|Hymenoptera|Figitidae|BOLD:AAG8258
Eucoilinae|[3802]|RRMFI1809-15|633[0n]bp|Hymenoptera|Figitidae|BOLD:AAM7478
Figitidae|[3803]|RRMFG1395-15|567[0n]bp|Hymenoptera|Figitidae|BOLD:ACJ1628
Eucoilinae|[3804]|RRINV691-15|620[0n]bp|Hymenoptera|Figitidae|
Eucoilinae|[3805]|RRMFE1143-15|512[1n]bp|Hymenoptera|Figitidae|BOLD:ACM0003
Figitidae|[3806]|RRSSA1261-15|594[0n]bp|Hymenoptera|Figitidae|BOLD:ACD4392
Figitidae|[3807]|RRMPG648-15|564[0n]bp|Hymenoptera|Figitidae|BOLD:ACN5042
Figitidae|[3808]|RRMFD1599-15|591[0n]bp|Hymenoptera|Figitidae|BOLD:ACO6056
Eucoilinae|[3809]|RRMPB4202-15|576[0n]bp|Hymenoptera|Figitidae|BOLD:ABA5948
Figitidae|[3810]|RRMFG1655-15|576[0n]bp|Hymenoptera|Figitidae|BOLD:ACM9237
Eucoilinae|[3811]|RRMFG2521-15|588[0n]bp|Hymenoptera|Figitidae|BOLD:AAU8625
Figitidae|[3812]|RRMPE165-15|591[0n]bp|Hymenoptera|Figitidae|BOLD:ACV4105
Figitidae|[3813]|RRINV853-15|633[0n]bp|Hymenoptera|Figitidae|
Platygastridae|[3814]|RRMFI258-15|525[0n]bp|Hymenoptera|Platygastridae|BOLD:ACI8542
Platygastridae|[3815]|RRMFE1014-15|579[0n]bp|Hymenoptera|Platygastridae|BOLD:ACV2105
Platygastridae|[3816]|RRMFG1377-15|582[1n]bp|Hymenoptera|Platygastridae|BOLD:ACI3903
Platygastridae|[3817]|RRINV1244-15|615[0n]bp|Hymenoptera|Platygastridae|
Platygastridae|[3818]|RRMFD1558-15|528[0n]bp|Hymenoptera|Platygastridae|BOLD:ABX8472
Platygastridae|[3819]|RRMFG1422-15|558[0n]bp|Hymenoptera|Platygastridae|BOLD:ACV5289
Platygaster variabilis|[3820]|RRMFG1385-15|582[0n]bp|Hymenoptera|Platygastridae|BOLD:ABW3242
Platygastridae|[3821]|RRMFE1644-15|564[0n]bp|Hymenoptera|Platygastridae|BOLD:ACB3267
Platygastridae|[3822]|RRMFE1621-15|312[0n]bp|Hymenoptera|Platygastridae|
Platygastridae|[3823]|RRMFI045-15|594[0n]bp|Hymenoptera|Platygastridae|BOLD:ACV5870

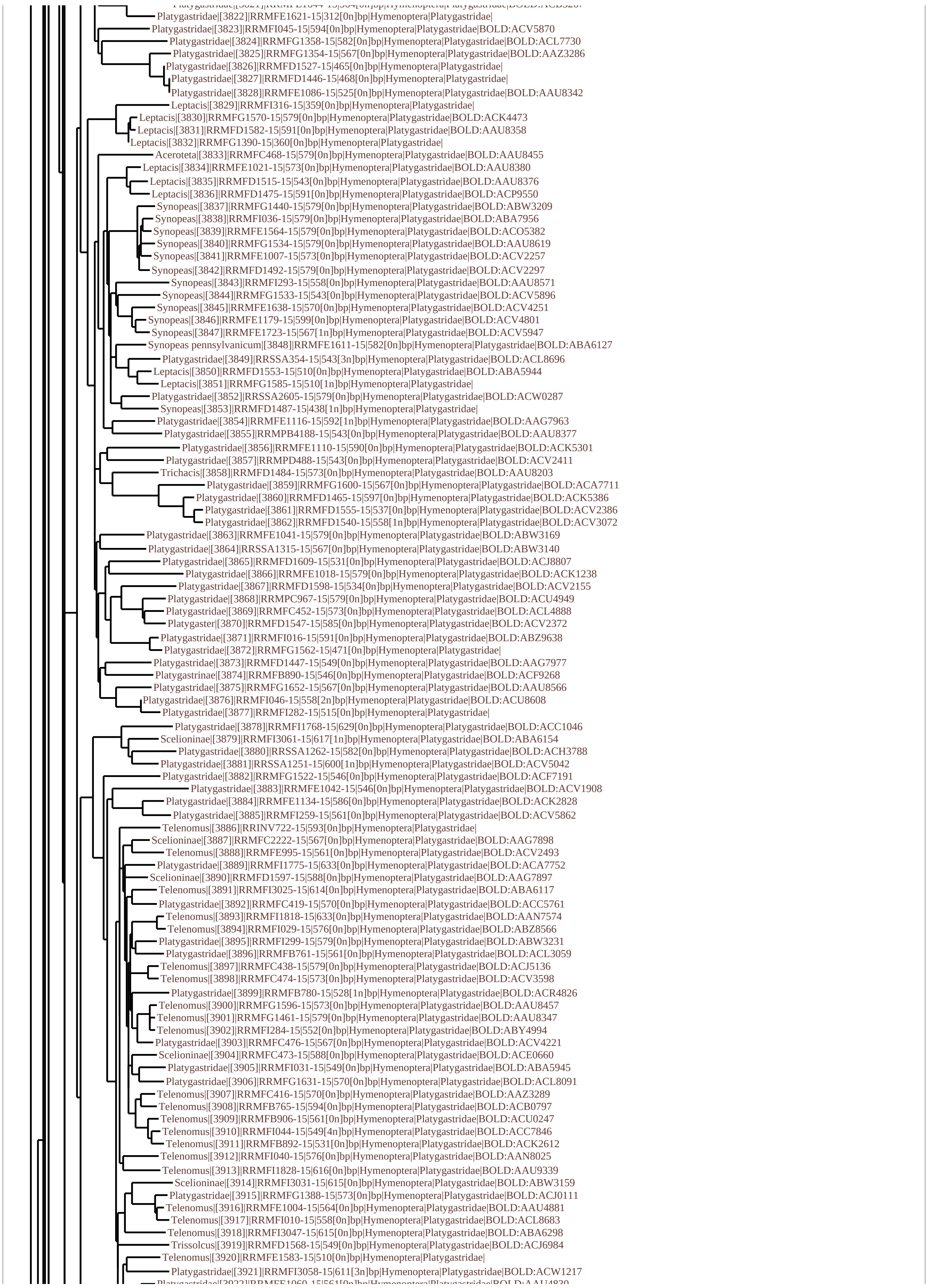
Platygastridae|[3822]|RRMFE1621-15|312[0n]bp|Hymenoptera|Platygastridae|
Platygastridae|[3823]|RRMFI045-15|594[0n]bp|Hymenoptera|Platygastridae|BOLD:ACV5870
Platygastridae|[3824]|RRMFG1358-15|582[0n]bp|Hymenoptera|Platygastridae|BOLD:ACL7730
Platygastridae|[3825]|RRMFG1354-15|567[0n]bp|Hymenoptera|Platygastridae|BOLD:AAZ3286
Platygastridae|[3826]|RRMFD1527-15|465[0n]bp|Hymenoptera|Platygastridae|
Platygastridae|[3827]|RRMFD1446-15|468[0n]bp|Hymenoptera|Platygastridae|
Platygastridae|[3828]|RRMFE1086-15|525[0n]bp|Hymenoptera|Platygastridae|BOLD:AAU8342
Leptacis|[3829]|RRMFI316-15|359[0n]bp|Hymenoptera|Platygastridae|
Leptacis|[3830]|RRMFG1570-15|579[0n]bp|Hymenoptera|Platygastridae|BOLD:ACK4473
Leptacis|[3831]|RRMFD1582-15|591[0n]bp|Hymenoptera|Platygastridae|BOLD:AAU8358
Leptacis|[3832]|RRMFG1390-15|360[0n]bp|Hymenoptera|Platygastridae|
Aceroteta|[3833]|RRMFC468-15|579[0n]bp|Hymenoptera|Platygastridae|BOLD:AAU8455
Leptacis|[3834]|RRMFE1021-15|573[0n]bp|Hymenoptera|Platygastridae|BOLD:AAU8380
Leptacis|[3835]|RRMFD1515-15|543[0n]bp|Hymenoptera|Platygastridae|BOLD:AAU8376
Leptacis|[3836]|RRMFD1475-15|591[0n]bp|Hymenoptera|Platygastridae|BOLD:ACP9550
Synopeas|[3837]|RRMFG1440-15|579[0n]bp|Hymenoptera|Platygastridae|BOLD:ABW3209
Synopeas|[3838]|RRMFI036-15|579[0n]bp|Hymenoptera|Platygastridae|BOLD:ABA7956
Synopeas|[3839]|RRMFE1564-15|579[0n]bp|Hymenoptera|Platygastridae|BOLD:ACO5382
Synopeas|[3840]|RRMFG1534-15|579[0n]bp|Hymenoptera|Platygastridae|BOLD:AAU8619
Synopeas|[3841]|RRMFE1007-15|573[0n]bp|Hymenoptera|Platygastridae|BOLD:ACV2257
Synopeas|[3842]|RRMFD1492-15|579[0n]bp|Hymenoptera|Platygastridae|BOLD:ACV2297
Synopeas|[3843]|RRMFI293-15|558[0n]bp|Hymenoptera|Platygastridae|BOLD:AAU8571
Synopeas|[3844]|RRMFG1533-15|543[0n]bp|Hymenoptera|Platygastridae|BOLD:ACV5896
Synopeas|[3845]|RRMFE1638-15|570[0n]bp|Hymenoptera|Platygastridae|BOLD:ACV4251
Synopeas|[3846]|RRMFE1179-15|599[0n]bp|Hymenoptera|Platygastridae|BOLD:ACV4801
Synopeas|[3847]|RRMFE1723-15|567[1n]bp|Hymenoptera|Platygastridae|BOLD:ACV5947
Synopeas pennsylvanicum|[3848]|RRMFE1611-15|582[0n]bp|Hymenoptera|Platygastridae|BOLD:ABA6127
Platygastridae|[3849]|RRSSA354-15|543[3n]bp|Hymenoptera|Platygastridae|BOLD:ACL8696
Leptacis|[3850]|RRMFD1553-15|510[0n]bp|Hymenoptera|Platygastridae|BOLD:ABA5944
Leptacis|[3851]|RRMFG1585-15|510[1n]bp|Hymenoptera|Platygastridae|
Platygastridae|[3852]|RRSSA2605-15|579[0n]bp|Hymenoptera|Platygastridae|BOLD:ACW0287
Synopeas|[3853]|RRMFD1487-15|438[1n]bp|Hymenoptera|Platygastridae|
Platygastridae|[3854]|RRMFE1116-15|592[1n]bp|Hymenoptera|Platygastridae|BOLD:AAG7963
Platygastridae|[3855]|RRMPB4188-15|543[0n]bp|Hymenoptera|Platygastridae|BOLD:AAU8377
Platygastridae|[3856]|RRMFE1110-15|590[0n]bp|Hymenoptera|Platygastridae|BOLD:ACK5301
Platygastridae|[3857]|RRMPD488-15|543[0n]bp|Hymenoptera|Platygastridae|BOLD:ACV2411
Trichacis|[3858]|RRMFD1484-15|573[0n]bp|Hymenoptera|Platygastridae|BOLD:AAU8203
Platygastridae|[3859]|RRMFG1600-15|567[0n]bp|Hymenoptera|Platygastridae|BOLD:ACA7711
Platygastridae|[3860]|RRMFD1465-15|597[0n]bp|Hymenoptera|Platygastridae|BOLD:ACK5386
Platygastridae|[3861]|RRMFD1555-15|537[0n]bp|Hymenoptera|Platygastridae|BOLD:ACV2386
Platygastridae|[3862]|RRMFD1540-15|558[1n]bp|Hymenoptera|Platygastridae|BOLD:ACV3072
Platygastridae|[3863]|RRMFE1041-15|579[0n]bp|Hymenoptera|Platygastridae|BOLD:ABW3169
Platygastridae|[3864]|RRSSA1315-15|567[0n]bp|Hymenoptera|Platygastridae|BOLD:ABW3140
Platygastridae|[3865]|RRMFD1609-15|531[0n]bp|Hymenoptera|Platygastridae|BOLD:ACJ8807
Platygastridae|[3866]|RRMFE1018-15|579[0n]bp|Hymenoptera|Platygastridae|BOLD:ACK1238
Platygastridae|[3867]|RRMFD1598-15|534[0n]bp|Hymenoptera|Platygastridae|BOLD:ACV2155
Platygastridae|[3868]|RRMPC967-15|579[0n]bp|Hymenoptera|Platygastridae|BOLD:ACU4949
Platygastridae|[3869]|RRMFC452-15|573[0n]bp|Hymenoptera|Platygastridae|BOLD:ACL4888
Platygaster|[3870]|RRMFD1547-15|585[0n]bp|Hymenoptera|Platygastridae|BOLD:ACV2372
Platygastridae|[3871]|RRMFI016-15|591[0n]bp|Hymenoptera|Platygastridae|BOLD:ABZ9638
Platygastridae|[3872]|RRMFG1562-15|471[0n]bp|Hymenoptera|Platygastridae|
Platygastridae|[3873]|RRMFD1447-15|549[0n]bp|Hymenoptera|Platygastridae|BOLD:AAG7977
Platygastrinae|[3874]|RRMFB890-15|546[0n]bp|Hymenoptera|Platygastridae|BOLD:ACF9268
Platygastridae|[3875]|RRMFG1652-15|567[0n]bp|Hymenoptera|Platygastridae|BOLD:AAU8566
Platygastridae|[3876]|RRMFI046-15|558[2n]bp|Hymenoptera|Platygastridae|BOLD:ACU8608
Platygastridae|[3877]|RRMFI282-15|515[0n]bp|Hymenoptera|Platygastridae|
Platygastridae|[3878]|RRMFI1768-15|629[0n]bp|Hymenoptera|Platygastridae|BOLD:ACC1046
Scelioninae|[3879]|RRMFI3061-15|617[1n]bp|Hymenoptera|Platygastridae|BOLD:ABA6154
Platygastridae|[3880]|RRSSA1262-15|582[0n]bp|Hymenoptera|Platygastridae|BOLD:ACH3788
Platygastridae|[3881]|RRSSA1251-15|600[1n]bp|Hymenoptera|Platygastridae|BOLD:ACV5042
Platygastridae|[3882]|RRMFG1522-15|546[0n]bp|Hymenoptera|Platygastridae|BOLD:ACF7191
Platygastridae|[3883]|RRMFE1042-15|546[0n]bp|Hymenoptera|Platygastridae|BOLD:ACV1908
Platygastridae|[3884]|RRMFE1134-15|586[0n]bp|Hymenoptera|Platygastridae|BOLD:ACK2828
Platygastridae|[3885]|RRMFI259-15|561[0n]bp|Hymenoptera|Platygastridae|BOLD:ACV5862
Telenomus|[3886]|RRINV722-15|593[0n]bp|Hymenoptera|Platygastridae|
Scelioninae|[3887]|RRMFC2222-15|567[0n]bp|Hymenoptera|Platygastridae|BOLD:AAG7898
Telenomus|[3888]|RRMFE995-15|561[0n]bp|Hymenoptera|Platygastridae|BOLD:ACV2493
Platygastridae|[3889]|RRMFI1775-15|633[0n]bp|Hymenoptera|Platygastridae|BOLD:ACA7752
Scelioninae|[3890]|RRMFD1597-15|588[0n]bp|Hymenoptera|Platygastridae|BOLD:AAG7897
Telenomus|[3891]|RRMFI3025-15|614[0n]bp|Hymenoptera|Platygastridae|BOLD:ABA6117
Platygastridae|[3892]|RRMFC419-15|570[0n]bp|Hymenoptera|Platygastridae|BOLD:ACC5761
Telenomus|[3893]|RRMFI1818-15|633[0n]bp|Hymenoptera|Platygastridae|BOLD:AAN7574
Telenomus|[3894]|RRMFI029-15|576[0n]bp|Hymenoptera|Platygastridae|BOLD:ABZ8566
Platygastridae|[3895]|RRMFI299-15|579[0n]bp|Hymenoptera|Platygastridae|BOLD:ABW3231
Platygastridae|[3896]|RRMFB761-15|561[0n]bp|Hymenoptera|Platygastridae|BOLD:ACL3059
Telenomus|[3897]|RRMFC438-15|579[0n]bp|Hymenoptera|Platygastridae|BOLD:ACJ5136
Telenomus|[3898]|RRMFC474-15|573[0n]bp|Hymenoptera|Platygastridae|BOLD:ACV3598
Platygastridae|[3899]|RRMFB780-15|528[1n]bp|Hymenoptera|Platygastridae|BOLD:ACR4826
Telenomus|[3900]|RRMFG1596-15|573[0n]bp|Hymenoptera|Platygastridae|BOLD:AAU8457
Telenomus|[3901]|RRMFG1461-15|579[0n]bp|Hymenoptera|Platygastridae|BOLD:AAU8347
Telenomus|[3902]|RRMFI284-15|552[0n]bp|Hymenoptera|Platygastridae|BOLD:ABY4994
Platygastridae|[3903]|RRMFC476-15|567[0n]bp|Hymenoptera|Platygastridae|BOLD:ACV4221
Scelioninae|[3904]|RRMFC473-15|588[0n]bp|Hymenoptera|Platygastridae|BOLD:ACE0660
Platygastridae|[3905]|RRMFI031-15|549[0n]bp|Hymenoptera|Platygastridae|BOLD:ABA5945
Platygastridae|[3906]|RRMFG1631-15|570[0n]bp|Hymenoptera|Platygastridae|BOLD:ACL8091
Telenomus|[3907]|RRMFC416-15|570[0n]bp|Hymenoptera|Platygastridae|BOLD:AAZ3289
Telenomus|[3908]|RRMFB765-15|594[0n]bp|Hymenoptera|Platygastridae|BOLD:ACB0797
Telenomus|[3909]|RRMFB906-15|561[0n]bp|Hymenoptera|Platygastridae|BOLD:ACU0247
Telenomus|[3910]|RRMFI044-15|549[4n]bp|Hymenoptera|Platygastridae|BOLD:ACC7846
Telenomus|[3911]|RRMFB892-15|531[0n]bp|Hymenoptera|Platygastridae|BOLD:ACK2612
Telenomus|[3912]|RRMFI040-15|576[0n]bp|Hymenoptera|Platygastridae|BOLD:AAN8025
Telenomus|[3913]|RRMFI1828-15|616[0n]bp|Hymenoptera|Platygastridae|BOLD:AAU9339
Scelioninae|[3914]|RRMFI3031-15|615[0n]bp|Hymenoptera|Platygastridae|BOLD:ABW3159
Platygastridae|[3915]|RRMFG1388-15|573[0n]bp|Hymenoptera|Platygastridae|BOLD:ACJ0111
Telenomus|[3916]|RRMFE1004-15|564[0n]bp|Hymenoptera|Platygastridae|BOLD:AAU4881
Telenomus|[3917]|RRMFI010-15|558[0n]bp|Hymenoptera|Platygastridae|BOLD:ACL8683
Telenomus|[3918]|RRMFI3047-15|615[0n]bp|Hymenoptera|Platygastridae|BOLD:ABA6298
Trissolcus|[3919]|RRMFD1568-15|549[0n]bp|Hymenoptera|Platygastridae|BOLD:ACJ6984
Telenomus|[3920]|RRMFE1583-15|510[0n]bp|Hymenoptera|Platygastridae|
Platygastridae|[3921]|RRMFI3058-15|611[3n]bp|Hymenoptera|Platygastridae|BOLD:ACW1217

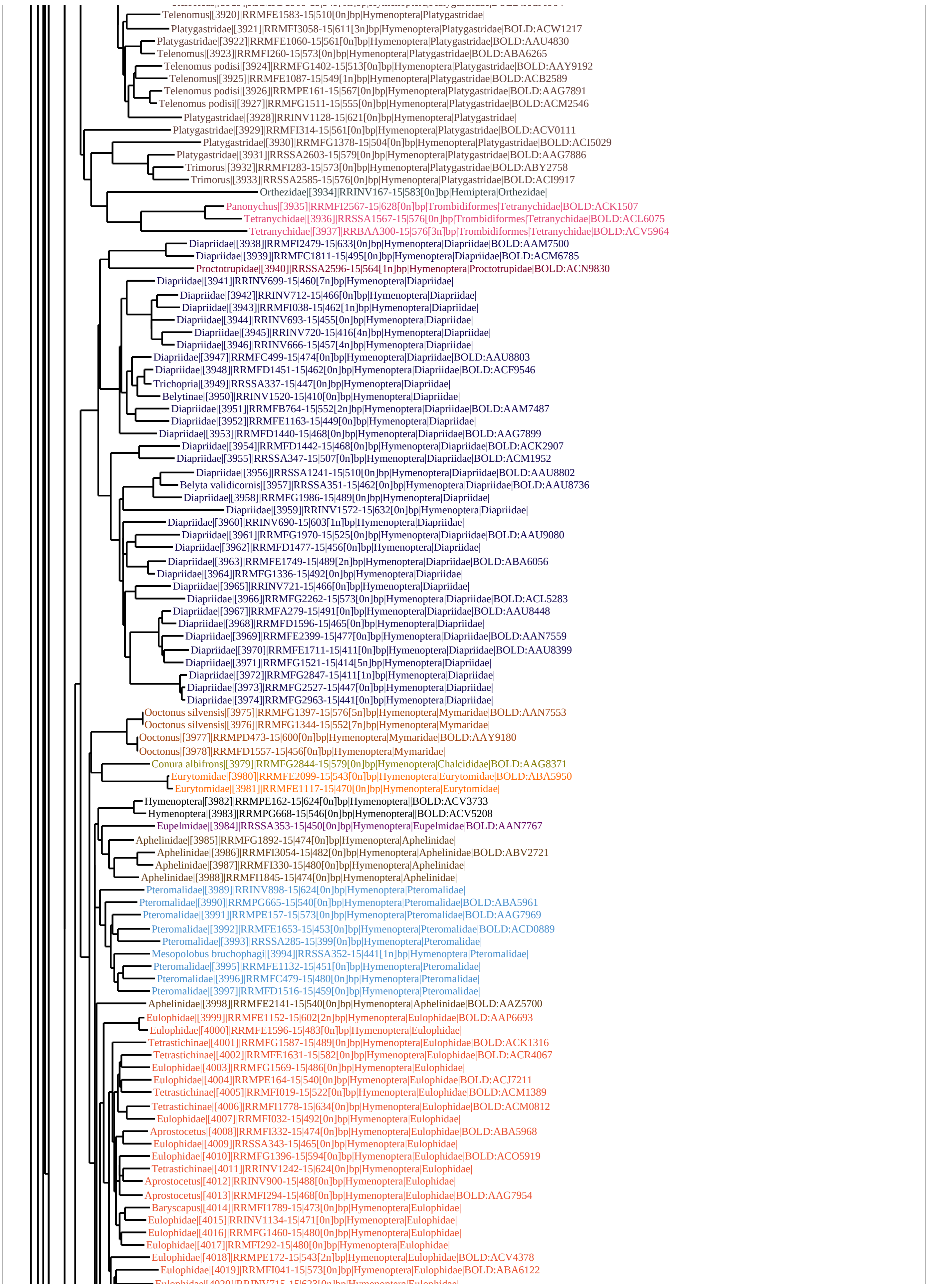

Telenomus|[3920]|RRMFE1583-15|510[0n]bp|Hymenoptera|Platygastridae|
Platygastridae|[3921]|RRMFI3058-15|611[3n]bp|Hymenoptera|Platygastridae|BOLD:ACW1217
Platygastridae|[3922]|RRMFE1060-15|561[0n]bp|Hymenoptera|Platygastridae|BOLD:AAU4830
Telenomus|[3923]|RRMFI260-15|573[0n]bp|Hymenoptera|Platygastridae|BOLD:ABA6265
Telenomus podisi|[3924]|RRMFG1402-15|513[0n]bp|Hymenoptera|Platygastridae|BOLD:AAY9192
Telenomus|[3925]|RRMFE1087-15|549[1n]bp|Hymenoptera|Platygastridae|BOLD:ACB2589
Telenomus podisi|[3926]|RRMPE161-15|567[0n]bp|Hymenoptera|Platygastridae|BOLD:AAG7891
Telenomus podisi|[3927]|RRMFG1511-15|555[0n]bp|Hymenoptera|Platygastridae|BOLD:ACM2546
Platygastridae|[3928]|RRINV1128-15|621[0n]bp|Hymenoptera|Platygastridae|
Platygastridae|[3929]|RRMFI314-15|561[0n]bp|Hymenoptera|Platygastridae|BOLD:ACV0111
Platygastridae|[3930]|RRMFG1378-15|504[0n]bp|Hymenoptera|Platygastridae|BOLD:ACI5029
Platygastridae|[3931]|RRSSA2603-15|579[0n]bp|Hymenoptera|Platygastridae|BOLD:AAG7886
Trimorus|[3932]|RRMFI283-15|573[0n]bp|Hymenoptera|Platygastridae|BOLD:ABY2758
Trimorus|[3933]|RRSSA2585-15|576[0n]bp|Hymenoptera|Platygastridae|BOLD:ACI9917
Orthezidae|[3934]|RRINV167-15|583[0n]bp|Hemiptera|Orthezidae|
Panonychus|[3935]|RRMFI2567-15|628[0n]bp|Trombidiformes|Tetranychidae|BOLD:ACK1507
Tetranychidae|[3936]|RRSSA1567-15|576[0n]bp|Trombidiformes|Tetranychidae|BOLD:ACL6075
Tetranychidae|[3937]|RRBAA300-15|576[3n]bp|Trombidiformes|Tetranychidae|BOLD:ACV5964
Diapriidae|[3938]|RRMFI2479-15|633[0n]bp|Hymenoptera|Diapriidae|BOLD:AAM7500
Diapriidae|[3939]|RRMFC1811-15|495[0n]bp|Hymenoptera|Diapriidae|BOLD:ACM6785
Proctotrupidae|[3940]|RRSSA2596-15|564[1n]bp|Hymenoptera|Proctotrupidae|BOLD:ACN9830
Diapriidae|[3941]|RRINV699-15|460[7n]bp|Hymenoptera|Diapriidae|
Diapriidae|[3942]|RRINV712-15|466[0n]bp|Hymenoptera|Diapriidae|
Diapriidae|[3943]|RRMFI038-15|462[1n]bp|Hymenoptera|Diapriidae|
Diapriidae|[3944]|RRINV693-15|455[0n]bp|Hymenoptera|Diapriidae|
Diapriidae|[3945]|RRINV720-15|416[4n]bp|Hymenoptera|Diapriidae|
Diapriidae|[3946]|RRINV666-15|457[4n]bp|Hymenoptera|Diapriidae|
Diapriidae|[3947]|RRMFC499-15|474[0n]bp|Hymenoptera|Diapriidae|BOLD:AAU8803
Diapriidae|[3948]|RRMFD1451-15|462[0n]bp|Hymenoptera|Diapriidae|BOLD:ACF9546
Trichopria|[3949]|RRSSA337-15|447[0n]bp|Hymenoptera|Diapriidae|
Belytinae|[3950]|RRINV1520-15|410[0n]bp|Hymenoptera|Diapriidae|
Diapriidae|[3951]|RRMFB764-15|552[2n]bp|Hymenoptera|Diapriidae|BOLD:AAM7487
Diapriidae|[3952]|RRMFE1163-15|449[0n]bp|Hymenoptera|Diapriidae|
Diapriidae|[3953]|RRMFD1440-15|468[0n]bp|Hymenoptera|Diapriidae|BOLD:AAG7899
Diapriidae|[3954]|RRMFD1442-15|468[0n]bp|Hymenoptera|Diapriidae|BOLD:ACK2907
Diapriidae|[3955]|RRSSA347-15|507[0n]bp|Hymenoptera|Diapriidae|BOLD:ACM1952
Diapriidae|[3956]|RRSSA1241-15|510[0n]bp|Hymenoptera|Diapriidae|BOLD:AAU8802
Belyta validicornis|[3957]|RRSSA351-15|462[0n]bp|Hymenoptera|Diapriidae|BOLD:AAU8736
Diapriidae|[3958]|RRMFG1986-15|489[0n]bp|Hymenoptera|Diapriidae|
Diapriidae|[3959]|RRINV1572-15|632[0n]bp|Hymenoptera|Diapriidae|
Diapriidae|[3960]|RRINV690-15|603[1n]bp|Hymenoptera|Diapriidae|
Diapriidae|[3961]|RRMFG1970-15|525[0n]bp|Hymenoptera|Diapriidae|BOLD:AAU9080
Diapriidae|[3962]|RRMFD1477-15|456[0n]bp|Hymenoptera|Diapriidae|
Diapriidae|[3963]|RRMFE1749-15|489[2n]bp|Hymenoptera|Diapriidae|BOLD:ABA6056
Diapriidae|[3964]|RRMFG1336-15|492[0n]bp|Hymenoptera|Diapriidae|
Diapriidae|[3965]|RRINV721-15|466[0n]bp|Hymenoptera|Diapriidae|
Diapriidae|[3966]|RRMFG2262-15|573[0n]bp|Hymenoptera|Diapriidae|BOLD:ACL5283
Diapriidae|[3967]|RRMFA279-15|491[0n]bp|Hymenoptera|Diapriidae|BOLD:AAU8448
Diapriidae|[3968]|RRMFD1596-15|465[0n]bp|Hymenoptera|Diapriidae|
Diapriidae|[3969]|RRMFE2399-15|477[0n]bp|Hymenoptera|Diapriidae|BOLD:AAN7559
Diapriidae|[3970]|RRMFE1711-15|411[0n]bp|Hymenoptera|Diapriidae|BOLD:AAU8399
Diapriidae|[3971]|RRMFG1521-15|414[5n]bp|Hymenoptera|Diapriidae|
Diapriidae|[3972]|RRMFG2847-15|411[1n]bp|Hymenoptera|Diapriidae|
Diapriidae|[3973]|RRMFG2527-15|447[0n]bp|Hymenoptera|Diapriidae|
Diapriidae|[3974]|RRMFG2963-15|441[0n]bp|Hymenoptera|Diapriidae|
Ooctonus silvensis|[3975]|RRMFG1397-15|576[5n]bp|Hymenoptera|Mymaridae|BOLD:AAN7553
Ooctonus silvensis|[3976]|RRMFG1344-15|552[7n]bp|Hymenoptera|Mymaridae|
Ooctonus|[3977]|RRMPD473-15|600[0n]bp|Hymenoptera|Mymaridae|BOLD:AAY9180
Ooctonus|[3978]|RRMFD1557-15|456[0n]bp|Hymenoptera|Mymaridae|
Conura albifrons|[3979]|RRMFG2844-15|579[0n]bp|Hymenoptera|Chalcididae|BOLD:AAG8371
Eurytomidae|[3980]|RRMFE2099-15|543[0n]bp|Hymenoptera|Eurytomidae|BOLD:ABA5950
Eurytomidae|[3981]|RRMFE1117-15|470[0n]bp|Hymenoptera|Eurytomidae|
Hymenoptera|[3982]|RRMPE162-15|624[0n]bp|Hymenoptera||BOLD:ACV3733
Hymenoptera|[3983]|RRMPG668-15|546[0n]bp|Hymenoptera||BOLD:ACV5208
Eupelmidae|[3984]|RRSSA353-15|450[0n]bp|Hymenoptera|Eupelmidae|BOLD:AAN7767
Aphelinidae|[3985]|RRMFG1892-15|474[0n]bp|Hymenoptera|Aphelinidae|
Aphelinidae|[3986]|RRMFI3054-15|482[0n]bp|Hymenoptera|Aphelinidae|BOLD:ABV2721
Aphelinidae|[3987]|RRMFI330-15|480[0n]bp|Hymenoptera|Aphelinidae|
Aphelinidae|[3988]|RRMFI1845-15|474[0n]bp|Hymenoptera|Aphelinidae|
Pteromalidae|[3989]|RRINV898-15|624[0n]bp|Hymenoptera|Pteromalidae|
Pteromalidae|[3990]|RRMPG665-15|540[0n]bp|Hymenoptera|Pteromalidae|BOLD:ABA5961
Pteromalidae|[3991]|RRMPE157-15|573[0n]bp|Hymenoptera|Pteromalidae|BOLD:AAG7969
Pteromalidae|[3992]|RRMFE1653-15|453[0n]bp|Hymenoptera|Pteromalidae|BOLD:ACD0889
Pteromalidae|[3993]|RRSSA285-15|399[0n]bp|Hymenoptera|Pteromalidae|
Mesopolobus bruchophagi|[3994]|RRSSA352-15|441[1n]bp|Hymenoptera|Pteromalidae|
Pteromalidae|[3995]|RRMFE1132-15|451[0n]bp|Hymenoptera|Pteromalidae|
Pteromalidae|[3996]|RRMFC479-15|480[0n]bp|Hymenoptera|Pteromalidae|
Pteromalidae|[3997]|RRMFD1516-15|459[0n]bp|Hymenoptera|Pteromalidae|
Aphelinidae|[3998]|RRMFE2141-15|540[0n]bp|Hymenoptera|Aphelinidae|BOLD:AAZ5700
Eulophidae|[3999]|RRMFE1152-15|602[2n]bp|Hymenoptera|Eulophidae|BOLD:AAP6693
Eulophidae|[4000]|RRMFE1596-15|483[0n]bp|Hymenoptera|Eulophidae|
Tetrastichinae|[4001]|RRMFG1587-15|489[0n]bp|Hymenoptera|Eulophidae|BOLD:ACK1316
Tetrastichinae|[4002]|RRMFE1631-15|582[0n]bp|Hymenoptera|Eulophidae|BOLD:ACR4067
Eulophidae|[4003]|RRMFG1569-15|486[0n]bp|Hymenoptera|Eulophidae|
Eulophidae|[4004]|RRMPE164-15|540[0n]bp|Hymenoptera|Eulophidae|BOLD:ACJ7211
Tetrastichinae|[4005]|RRMFI019-15|522[0n]bp|Hymenoptera|Eulophidae|BOLD:ACM1389
Tetrastichinae|[4006]|RRMFI1778-15|634[0n]bp|Hymenoptera|Eulophidae|BOLD:ACM0812
Eulophidae|[4007]|RRMFI032-15|492[0n]bp|Hymenoptera|Eulophidae|
Aprostocetus|[4008]|RRMFI332-15|474[0n]bp|Hymenoptera|Eulophidae|BOLD:ABA5968
Eulophidae|[4009]|RRSSA343-15|465[0n]bp|Hymenoptera|Eulophidae|
Eulophidae|[4010]|RRMFG1396-15|594[0n]bp|Hymenoptera|Eulophidae|BOLD:ACO5919
Tetrastichinae|[4011]|RRINV1242-15|624[0n]bp|Hymenoptera|Eulophidae|
Aprostocetus|[4012]|RRINV900-15|488[0n]bp|Hymenoptera|Eulophidae|
Aprostocetus|[4013]|RRMFI294-15|468[0n]bp|Hymenoptera|Eulophidae|BOLD:AAG7954
Baryscapus|[4014]|RRMFI1789-15|473[0n]bp|Hymenoptera|Eulophidae|
Eulophidae|[4015]|RRINV1134-15|471[0n]bp|Hymenoptera|Eulophidae|
Eulophidae|[4016]|RRMFG1460-15|480[0n]bp|Hymenoptera|Eulophidae|
Eulophidae|[4017]|RRMFI292-15|480[0n]bp|Hymenoptera|Eulophidae|
Eulophidae|[4018]|RRMPE172-15|543[2n]bp|Hymenoptera|Eulophidae|BOLD:ACV4378
Eulophidae|[4019]|RRMFI041-15|573[0n]bp|Hymenoptera|Eulophidae|BOLD:ABA6122

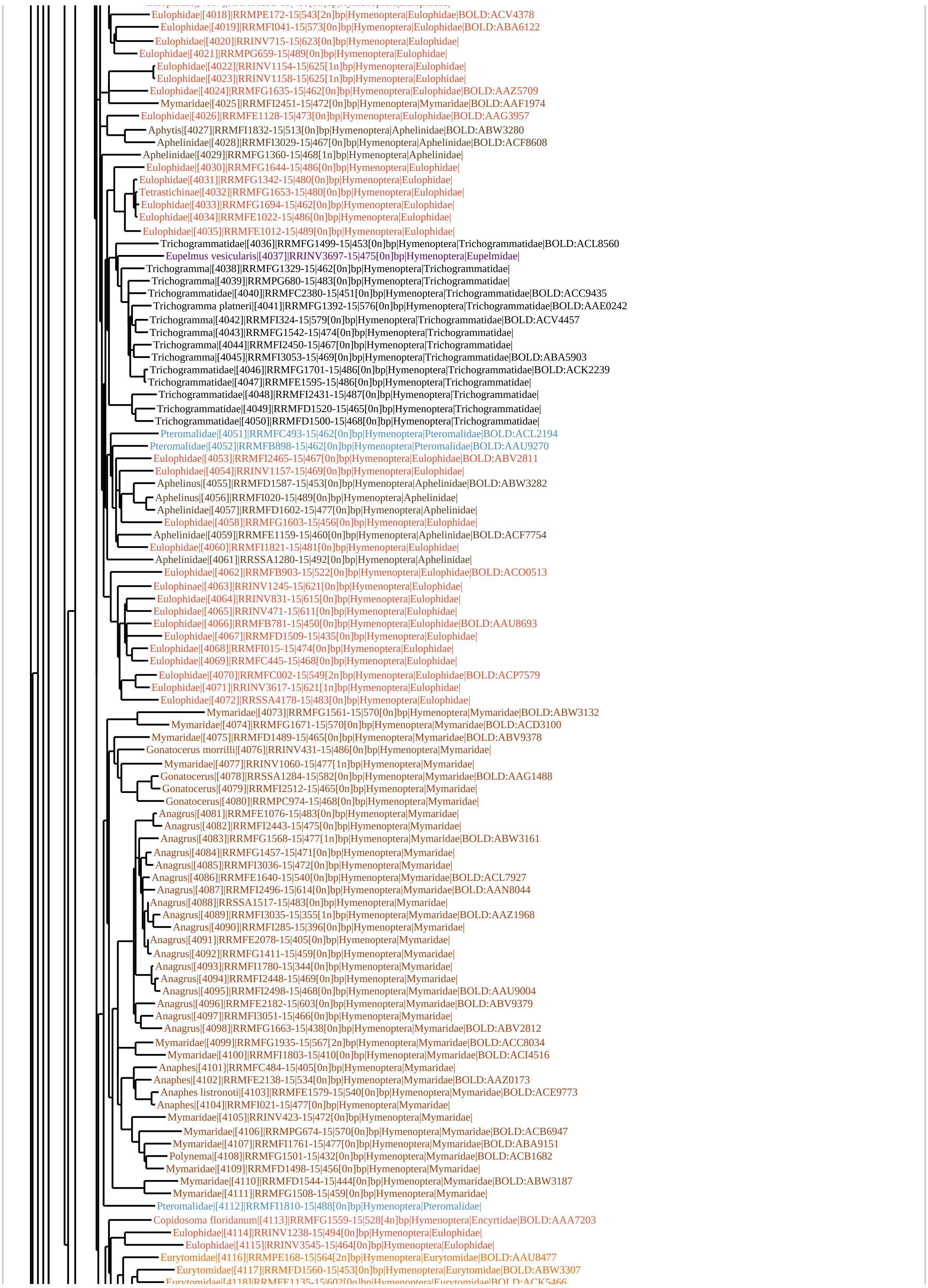
Eulophidae|[4018]|RRMPE172-15|543[2n]bp|Hymenoptera|Eulophidae|BOLD:ACV4378
Eulophidae|[4019]|RRMFI041-15|573[0n]bp|Hymenoptera|Eulophidae|BOLD:ABA6122
Eulophidae|[4020]|RRINV715-15|623[0n]bp|Hymenoptera|Eulophidae|
Eulophidae|[4021]|RRMPG659-15|489[0n]bp|Hymenoptera|Eulophidae|
Eulophidae|[4022]|RRINV1154-15|625[1n]bp|Hymenoptera|Eulophidae|
Eulophidae|[4023]|RRINV1158-15|625[1n]bp|Hymenoptera|Eulophidae|
Eulophidae|[4024]|RRMFG1635-15|462[0n]bp|Hymenoptera|Eulophidae|BOLD:AAZ5709
Mymaridae|[4025]|RRMFI2451-15|472[0n]bp|Hymenoptera|Mymaridae|BOLD:AAF1974
Eulophidae|[4026]|RRMFE1128-15|473[0n]bp|Hymenoptera|Eulophidae|BOLD:AAG3957
Aphytis|[4027]|RRMFI1832-15|513[0n]bp|Hymenoptera|Aphelinidae|BOLD:ABW3280
Aphelinidae|[4028]|RRMFI3029-15|467[0n]bp|Hymenoptera|Aphelinidae|BOLD:ACF8608
Aphelinidae|[4029]|RRMFG1360-15|468[1n]bp|Hymenoptera|Aphelinidae|
Eulophidae|[4030]|RRMFG1644-15|486[0n]bp|Hymenoptera|Eulophidae|
Eulophidae|[4031]|RRMFG1342-15|480[0n]bp|Hymenoptera|Eulophidae|
Tetrastichinae|[4032]|RRMFG1653-15|480[0n]bp|Hymenoptera|Eulophidae|
Eulophidae|[4033]|RRMFG1694-15|462[0n]bp|Hymenoptera|Eulophidae|
Eulophidae|[4034]|RRMFE1022-15|486[0n]bp|Hymenoptera|Eulophidae|
Eulophidae|[4035]|RRMFE1012-15|489[0n]bp|Hymenoptera|Eulophidae|
Trichogrammatidae|[4036]|RRMFG1499-15|453[0n]bp|Hymenoptera|Trichogrammatidae|BOLD:ACL8560
Eupelmus vesicularis|[4037]|RRINV3697-15|475[0n]bp|Hymenoptera|Eupelmidae|
Trichogramma|[4038]|RRMFG1329-15|462[0n]bp|Hymenoptera|Trichogrammatidae|
Trichogramma|[4039]|RRMPG680-15|483[0n]bp|Hymenoptera|Trichogrammatidae|
Trichogrammatidae|[4040]|RRMFC2380-15|451[0n]bp|Hymenoptera|Trichogrammatidae|BOLD:ACC9435
Trichogramma platneri|[4041]|RRMFG1392-15|576[0n]bp|Hymenoptera|Trichogrammatidae|BOLD:AAE0242
Trichogramma|[4042]|RRMFI324-15|579[0n]bp|Hymenoptera|Trichogrammatidae|BOLD:ACV4457
Trichogramma|[4043]|RRMFG1542-15|474[0n]bp|Hymenoptera|Trichogrammatidae|
Trichogramma|[4044]|RRMFI2450-15|467[0n]bp|Hymenoptera|Trichogrammatidae|
Trichogramma|[4045]|RRMFI3053-15|469[0n]bp|Hymenoptera|Trichogrammatidae|BOLD:ABA5903
Trichogrammatidae|[4046]|RRMFG1701-15|486[0n]bp|Hymenoptera|Trichogrammatidae|BOLD:ACK2239
Trichogrammatidae|[4047]|RRMFE1595-15|486[0n]bp|Hymenoptera|Trichogrammatidae|
Trichogrammatidae|[4048]|RRMFI2431-15|487[0n]bp|Hymenoptera|Trichogrammatidae|
Trichogrammatidae|[4049]|RRMFD1520-15|465[0n]bp|Hymenoptera|Trichogrammatidae|
Trichogrammatidae|[4050]|RRMFD1500-15|468[0n]bp|Hymenoptera|Trichogrammatidae|
Pteromalidae|[4051]|RRMFC493-15|462[0n]bp|Hymenoptera|Pteromalidae|BOLD:ACL2194
Pteromalidae|[4052]|RRMFB898-15|462[0n]bp|Hymenoptera|Pteromalidae|BOLD:AAU9270
Eulophidae|[4053]|RRMFI2465-15|467[0n]bp|Hymenoptera|Eulophidae|BOLD:ABV2811
Eulophidae|[4054]|RRINV1157-15|469[0n]bp|Hymenoptera|Eulophidae|
Aphelinus|[4055]|RRMFD1587-15|453[0n]bp|Hymenoptera|Aphelinidae|BOLD:ABW3282
Aphelinus|[4056]|RRMFI020-15|489[0n]bp|Hymenoptera|Aphelinidae|
Aphelinidae|[4057]|RRMFD1602-15|477[0n]bp|Hymenoptera|Aphelinidae|
Eulophidae|[4058]|RRMFG1603-15|456[0n]bp|Hymenoptera|Eulophidae|
Aphelinidae|[4059]|RRMFE1159-15|460[0n]bp|Hymenoptera|Aphelinidae|BOLD:ACF7754
Eulophidae|[4060]|RRMFI1821-15|481[0n]bp|Hymenoptera|Eulophidae|
Aphelinidae|[4061]|RRSSA1280-15|492[0n]bp|Hymenoptera|Aphelinidae|
Eulophidae|[4062]|RRMFB903-15|522[0n]bp|Hymenoptera|Eulophidae|BOLD:ACO0513
Eulophinae|[4063]|RRINV1245-15|621[0n]bp|Hymenoptera|Eulophidae|
Eulophidae|[4064]|RRINV831-15|615[0n]bp|Hymenoptera|Eulophidae|
Eulophidae|[4065]|RRINV471-15|611[0n]bp|Hymenoptera|Eulophidae|
Eulophidae|[4066]|RRMFB781-15|450[0n]bp|Hymenoptera|Eulophidae|BOLD:AAU8693
Eulophidae|[4067]|RRMFD1509-15|435[0n]bp|Hymenoptera|Eulophidae|
Eulophidae|[4068]|RRMFI015-15|474[0n]bp|Hymenoptera|Eulophidae|
Eulophidae|[4069]|RRMFC445-15|468[0n]bp|Hymenoptera|Eulophidae|
Eulophidae|[4070]|RRMFC002-15|549[2n]bp|Hymenoptera|Eulophidae|BOLD:ACP7579
Eulophidae|[4071]|RRINV3617-15|621[1n]bp|Hymenoptera|Eulophidae|
Eulophidae|[4072]|RRSSA4178-15|483[0n]bp|Hymenoptera|Eulophidae|
Mymaridae|[4073]|RRMFG1561-15|570[0n]bp|Hymenoptera|Mymaridae|BOLD:ABW3132
Mymaridae|[4074]|RRMFG1671-15|570[0n]bp|Hymenoptera|Mymaridae|BOLD:ACD3100
Mymaridae|[4075]|RRMFD1489-15|465[0n]bp|Hymenoptera|Mymaridae|BOLD:ABV9378
Gonatocerus morrilli|[4076]|RRINV431-15|486[0n]bp|Hymenoptera|Mymaridae|
Mymaridae|[4077]|RRINV1060-15|477[1n]bp|Hymenoptera|Mymaridae|
Gonatocerus|[4078]|RRSSA1284-15|582[0n]bp|Hymenoptera|Mymaridae|BOLD:AAG1488
Gonatocerus|[4079]|RRMFI2512-15|465[0n]bp|Hymenoptera|Mymaridae|
Gonatocerus|[4080]|RRMPC974-15|468[0n]bp|Hymenoptera|Mymaridae|
Anagrus|[4081]|RRMFE1076-15|483[0n]bp|Hymenoptera|Mymaridae|
Anagrus|[4082]|RRMFI2443-15|475[0n]bp|Hymenoptera|Mymaridae|
Anagrus|[4083]|RRMFG1568-15|477[1n]bp|Hymenoptera|Mymaridae|BOLD:ABW3161
Anagrus|[4084]|RRMFG1457-15|471[0n]bp|Hymenoptera|Mymaridae|
Anagrus|[4085]|RRMFI3036-15|472[0n]bp|Hymenoptera|Mymaridae|
Anagrus|[4086]|RRMFE1640-15|540[0n]bp|Hymenoptera|Mymaridae|BOLD:ACL7927
Anagrus|[4087]|RRMFI2496-15|614[0n]bp|Hymenoptera|Mymaridae|BOLD:AAN8044
Anagrus|[4088]|RRSSA1517-15|483[0n]bp|Hymenoptera|Mymaridae|
Anagrus|[4089]|RRMFI3035-15|355[1n]bp|Hymenoptera|Mymaridae|BOLD:AAZ1968
Anagrus|[4090]|RRMFI285-15|396[0n]bp|Hymenoptera|Mymaridae|
Anagrus|[4091]|RRMFE2078-15|405[0n]bp|Hymenoptera|Mymaridae|
Anagrus|[4092]|RRMFG1411-15|459[0n]bp|Hymenoptera|Mymaridae|
Anagrus|[4093]|RRMFI1780-15|344[0n]bp|Hymenoptera|Mymaridae|
Anagrus|[4094]|RRMFI2448-15|469[0n]bp|Hymenoptera|Mymaridae|
Anagrus|[4095]|RRMFI2498-15|468[0n]bp|Hymenoptera|Mymaridae|BOLD:AAU9004
Anagrus|[4096]|RRMFE2182-15|603[0n]bp|Hymenoptera|Mymaridae|BOLD:ABV9379
Anagrus|[4097]|RRMFI3051-15|466[0n]bp|Hymenoptera|Mymaridae|
Anagrus|[4098]|RRMFG1663-15|438[0n]bp|Hymenoptera|Mymaridae|BOLD:ABV2812
Mymaridae|[4099]|RRMFG1935-15|567[2n]bp|Hymenoptera|Mymaridae|BOLD:ACC8034
Mymaridae|[4100]|RRMFI1803-15|410[0n]bp|Hymenoptera|Mymaridae|BOLD:ACI4516
Anaphes|[4101]|RRMFC484-15|405[0n]bp|Hymenoptera|Mymaridae|
Anaphes|[4102]|RRMFE2138-15|534[0n]bp|Hymenoptera|Mymaridae|BOLD:AAZ0173
Anaphes listronoti|[4103]|RRMFE1579-15|540[0n]bp|Hymenoptera|Mymaridae|BOLD:ACE9773
Anaphes|[4104]|RRMFI021-15|477[0n]bp|Hymenoptera|Mymaridae|
Mymaridae|[4105]|RRINV423-15|472[0n]bp|Hymenoptera|Mymaridae|
Mymaridae|[4106]|RRMPG674-15|570[0n]bp|Hymenoptera|Mymaridae|BOLD:ACB6947
Mymaridae|[4107]|RRMFI1761-15|477[0n]bp|Hymenoptera|Mymaridae|BOLD:ABA9151
Polynema|[4108]|RRMFG1501-15|432[0n]bp|Hymenoptera|Mymaridae|BOLD:ACB1682
Mymaridae|[4109]|RRMFD1498-15|456[0n]bp|Hymenoptera|Mymaridae|
Mymaridae|[4110]|RRMFD1544-15|444[0n]bp|Hymenoptera|Mymaridae|BOLD:ABW3187
Mymaridae|[4111]|RRMFG1508-15|459[0n]bp|Hymenoptera|Mymaridae|
Pteromalidae|[4112]|RRMFI1810-15|488[0n]bp|Hymenoptera|Pteromalidae|
Copidosoma floridanum|[4113]|RRMFG1559-15|528[4n]bp|Hymenoptera|Encyrtidae|BOLD:AAA7203
Eulophidae|[4114]|RRINV1238-15|494[0n]bp|Hymenoptera|Eulophidae|
Eulophidae|[4115]|RRINV3545-15|464[0n]bp|Hymenoptera|Eulophidae|
Eurytomidae|[4116]|RRMPE168-15|564[2n]bp|Hymenoptera|Eurytomidae|BOLD:AAU8477
Eurytomidae|[4117]|RRMFD1560-15|453[0n]bp|Hymenoptera|Eurytomidae|BOLD:ABW3307
Eurytomidae|[4118]|RRMFE1135-15|602[0n]bp|Hymenoptera|Eurytomidae|BOLD:ACK5466

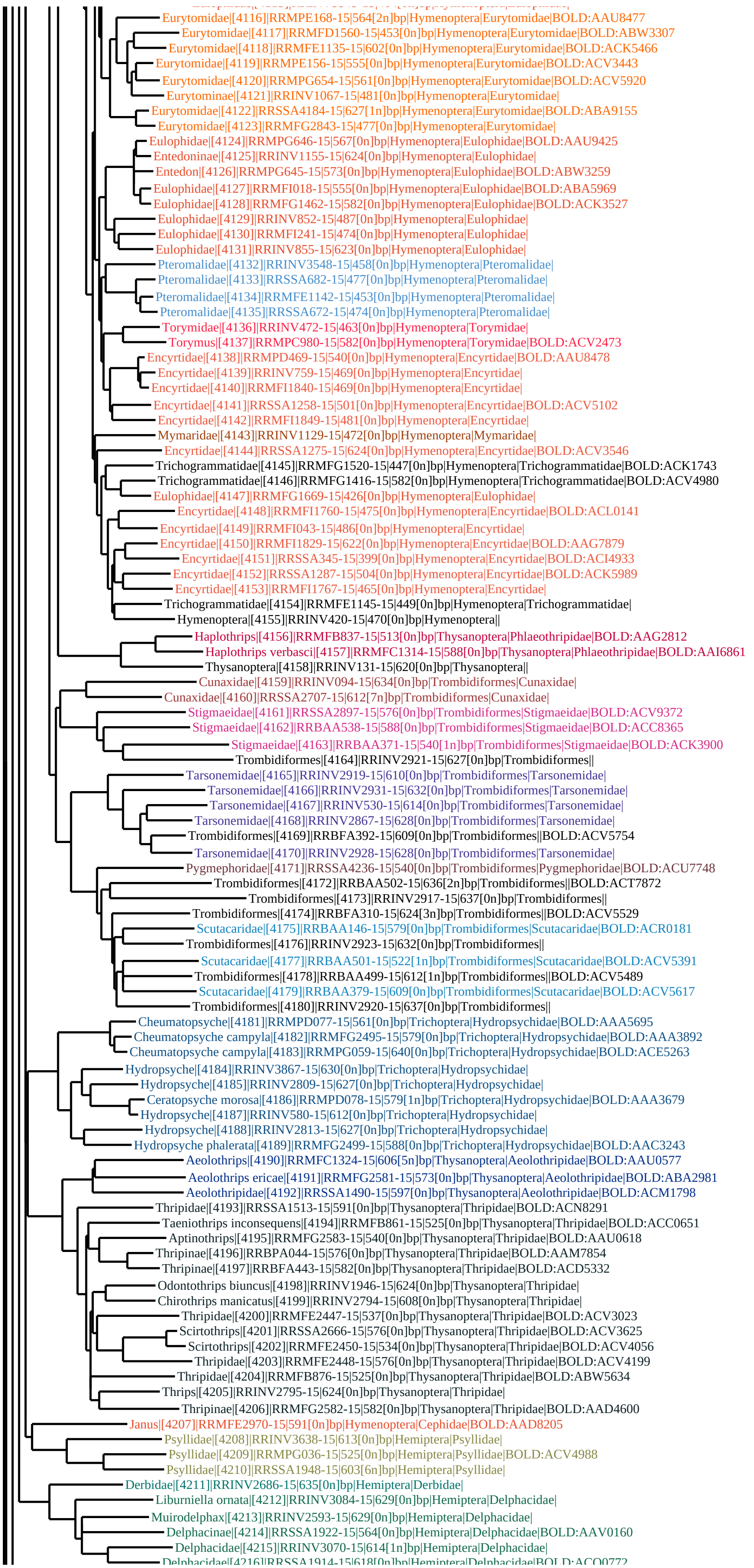

Eurytomidae|[4116]|RRMPE168-15|564[2n]bp|Hymenoptera|Eurytomidae|BOLD:AAU8477
Eurytomidae|[4117]|RRMFD1560-15|453[0n]bp|Hymenoptera|Eurytomidae|BOLD:ABW3307
Eurytomidae|[4118]|RRMFE1135-15|602[0n]bp|Hymenoptera|Eurytomidae|BOLD:ACK5466
Eurytomidae|[4119]|RRMPE156-15|555[0n]bp|Hymenoptera|Eurytomidae|BOLD:ACV3443
Eurytomidae|[4120]|RRMPG654-15|561[0n]bp|Hymenoptera|Eurytomidae|BOLD:ACV5920
Eurytominae|[4121]|RRINV1067-15|481[0n]bp|Hymenoptera|Eurytomidae|
Eurytomidae|[4122]|RRSSA4184-15|627[1n]bp|Hymenoptera|Eurytomidae|BOLD:ABA9155
Eurytomidae|[4123]|RRMFG2843-15|477[0n]bp|Hymenoptera|Eurytomidae|
Eulophidae|[4124]|RRMPG646-15|567[0n]bp|Hymenoptera|Eulophidae|BOLD:AAU9425
Entedoninae|[4125]|RRINV1155-15|624[0n]bp|Hymenoptera|Eulophidae|
Entedon|[4126]|RRMPG645-15|573[0n]bp|Hymenoptera|Eulophidae|BOLD:ABW3259
Eulophidae|[4127]|RRMFI018-15|555[0n]bp|Hymenoptera|Eulophidae|BOLD:ABA5969
Eulophidae|[4128]|RRMFG1462-15|582[0n]bp|Hymenoptera|Eulophidae|BOLD:ACK3527
Eulophidae|[4129]|RRINV852-15|487[0n]bp|Hymenoptera|Eulophidae|
Eulophidae|[4130]|RRMFI241-15|474[0n]bp|Hymenoptera|Eulophidae|
Eulophidae|[4131]|RRINV855-15|623[0n]bp|Hymenoptera|Eulophidae|
Pteromalidae|[4132]|RRINV3548-15|458[0n]bp|Hymenoptera|Pteromalidae|
Pteromalidae|[4133]|RRSSA682-15|477[0n]bp|Hymenoptera|Pteromalidae|
Pteromalidae|[4134]|RRMFE1142-15|453[0n]bp|Hymenoptera|Pteromalidae|
Pteromalidae|[4135]|RRSSA672-15|474[0n]bp|Hymenoptera|Pteromalidae|
Torymidae|[4136]|RRINV472-15|463[0n]bp|Hymenoptera|Torymidae|
Torymus|[4137]|RRMPC980-15|582[0n]bp|Hymenoptera|Torymidae|BOLD:ACV2473
Encyrtidae|[4138]|RRMPD469-15|540[0n]bp|Hymenoptera|Encyrtidae|BOLD:AAU8478
Encyrtidae|[4139]|RRINV759-15|469[0n]bp|Hymenoptera|Encyrtidae|
Encyrtidae|[4140]|RRMFI1840-15|469[0n]bp|Hymenoptera|Encyrtidae|
Encyrtidae|[4141]|RRSSA1258-15|501[0n]bp|Hymenoptera|Encyrtidae|BOLD:ACV5102
Encyrtidae|[4142]|RRMFI1849-15|481[0n]bp|Hymenoptera|Encyrtidae|
Mymaridae|[4143]|RRINV1129-15|472[0n]bp|Hymenoptera|Mymaridae|
Encyrtidae|[4144]|RRSSA1275-15|624[0n]bp|Hymenoptera|Encyrtidae|BOLD:ACV3546
Trichogrammatidae|[4145]|RRMFG1520-15|447[0n]bp|Hymenoptera|Trichogrammatidae|BOLD:ACK1743
Trichogrammatidae|[4146]|RRMFG1416-15|582[0n]bp|Hymenoptera|Trichogrammatidae|BOLD:ACV4980
Eulophidae|[4147]|RRMFG1669-15|426[0n]bp|Hymenoptera|Eulophidae|
Encyrtidae|[4148]|RRMFI1760-15|475[0n]bp|Hymenoptera|Encyrtidae|BOLD:ACL0141
Encyrtidae|[4149]|RRMFI043-15|486[0n]bp|Hymenoptera|Encyrtidae|
Encyrtidae|[4150]|RRMFI1829-15|622[0n]bp|Hymenoptera|Encyrtidae|BOLD:AAG7879
Encyrtidae|[4151]|RRSSA345-15|399[0n]bp|Hymenoptera|Encyrtidae|BOLD:ACI4933
Encyrtidae|[4152]|RRSSA1287-15|504[0n]bp|Hymenoptera|Encyrtidae|BOLD:ACK5989
Encyrtidae|[4153]|RRMFI1767-15|465[0n]bp|Hymenoptera|Encyrtidae|
Trichogrammatidae|[4154]|RRMFE1145-15|449[0n]bp|Hymenoptera|Trichogrammatidae|
Hymenoptera|[4155]|RRINV420-15|470[0n]bp|Hymenoptera||
Haplothrips|[4156]|RRMFB837-15|513[0n]bp|Thysanoptera|Phlaeothripidae|BOLD:AAG2812
Haplothrips verbasci|[4157]|RRMFC1314-15|588[0n]bp|Thysanoptera|Phlaeothripidae|BOLD:AAI6861
Thysanoptera|[4158]|RRINV131-15|620[0n]bp|Thysanoptera||
Cunaxidae|[4159]|RRINV094-15|634[0n]bp|Trombidiformes|Cunaxidae|
Cunaxidae|[4160]|RRSSA2707-15|612[7n]bp|Trombidiformes|Cunaxidae|
Stigmaeidae|[4161]|RRSSA2897-15|576[0n]bp|Trombidiformes|Stigmaeidae|BOLD:ACV9372
Stigmaeidae|[4162]|RRBAA538-15|588[0n]bp|Trombidiformes|Stigmaeidae|BOLD:ACC8365
Stigmaeidae|[4163]|RRBAA371-15|540[1n]bp|Trombidiformes|Stigmaeidae|BOLD:ACK3900
Trombidiformes|[4164]|RRINV2921-15|627[0n]bp|Trombidiformes||
Tarsonemidae|[4165]|RRINV2919-15|610[0n]bp|Trombidiformes|Tarsonemidae|
Tarsonemidae|[4166]|RRINV2931-15|632[0n]bp|Trombidiformes|Tarsonemidae|
Tarsonemidae|[4167]|RRINV530-15|614[0n]bp|Trombidiformes|Tarsonemidae|
Tarsonemidae|[4168]|RRINV2867-15|628[0n]bp|Trombidiformes|Tarsonemidae|
Trombidiformes|[4169]|RRBFA392-15|609[0n]bp|Trombidiformes||BOLD:ACV5754
Tarsonemidae|[4170]|RRINV2928-15|628[0n]bp|Trombidiformes|Tarsonemidae|
Pygmephoridae|[4171]|RRSSA4236-15|540[0n]bp|Trombidiformes|Pygmephoridae|BOLD:ACU7748
Trombidiformes|[4172]|RRBAA502-15|636[2n]bp|Trombidiformes||BOLD:ACT7872
Trombidiformes|[4173]|RRINV2917-15|637[0n]bp|Trombidiformes||
Trombidiformes|[4174]|RRBFA310-15|624[3n]bp|Trombidiformes||BOLD:ACV5529
Scutacaridae|[4175]|RRBAA146-15|579[0n]bp|Trombidiformes|Scutacaridae|BOLD:ACR0181
Trombidiformes|[4176]|RRINV2923-15|632[0n]bp|Trombidiformes||
Scutacaridae|[4177]|RRBAA501-15|522[1n]bp|Trombidiformes|Scutacaridae|BOLD:ACV5391
Trombidiformes|[4178]|RRBAA499-15|612[1n]bp|Trombidiformes||BOLD:ACV5489
Scutacaridae|[4179]|RRBAA379-15|609[0n]bp|Trombidiformes|Scutacaridae|BOLD:ACV5617
Trombidiformes|[4180]|RRINV2920-15|637[0n]bp|Trombidiformes||
Cheumatopsyche|[4181]|RRMPD077-15|561[0n]bp|Trichoptera|Hydropsychidae|BOLD:AAA5695
Cheumatopsyche campyla|[4182]|RRMFG2495-15|579[0n]bp|Trichoptera|Hydropsychidae|BOLD:AAA3892
Cheumatopsyche campyla|[4183]|RRMPG059-15|640[0n]bp|Trichoptera|Hydropsychidae|BOLD:ACE5263
Hydropsyche|[4184]|RRINV3867-15|630[0n]bp|Trichoptera|Hydropsychidae|
Hydropsyche|[4185]|RRINV2809-15|627[0n]bp|Trichoptera|Hydropsychidae|
Ceratopsyche morosa|[4186]|RRMPD078-15|579[1n]bp|Trichoptera|Hydropsychidae|BOLD:AAA3679
Hydropsyche|[4187]|RRINV580-15|612[0n]bp|Trichoptera|Hydropsychidae|
Hydropsyche|[4188]|RRINV2813-15|627[0n]bp|Trichoptera|Hydropsychidae|
Hydropsyche phalerata|[4189]|RRMFG2499-15|588[0n]bp|Trichoptera|Hydropsychidae|BOLD:AAC3243
Aeolothrips|[4190]|RRMFC1324-15|606[5n]bp|Thysanoptera|Aeolothripidae|BOLD:AAU0577
Aeolothrips ericae|[4191]|RRMFG2581-15|573[0n]bp|Thysanoptera|Aeolothripidae|BOLD:ABA2981
Aeolothripidae|[4192]|RRSSA1490-15|597[0n]bp|Thysanoptera|Aeolothripidae|BOLD:ACM1798
Thripidae|[4193]|RRSSA1513-15|591[0n]bp|Thysanoptera|Thripidae|BOLD:ACN8291
Taeniothrips inconsequens|[4194]|RRMFB861-15|525[0n]bp|Thysanoptera|Thripidae|BOLD:ACC0651
Aptinothrips|[4195]|RRMFG2583-15|540[0n]bp|Thysanoptera|Thripidae|BOLD:AAU0618
Thripinae|[4196]|RRBPA044-15|576[0n]bp|Thysanoptera|Thripidae|BOLD:AAM7854
Thripinae|[4197]|RRBFA443-15|582[0n]bp|Thysanoptera|Thripidae|BOLD:ACD5332
Odontothrips biuncus|[4198]|RRINV1946-15|624[0n]bp|Thysanoptera|Thripidae|
Chirothrips manicatus|[4199]|RRINV2794-15|608[0n]bp|Thysanoptera|Thripidae|
Thripidae|[4200]|RRMFE2447-15|537[0n]bp|Thysanoptera|Thripidae|BOLD:ACV3023
Scirtothrips|[4201]|RRSSA2666-15|576[0n]bp|Thysanoptera|Thripidae|BOLD:ACV3625
Scirtothrips|[4202]|RRMFE2450-15|534[0n]bp|Thysanoptera|Thripidae|BOLD:ACV4056
Thripidae|[4203]|RRMFE2448-15|576[0n]bp|Thysanoptera|Thripidae|BOLD:ACV4199
Thripidae|[4204]|RRMFB876-15|525[0n]bp|Thysanoptera|Thripidae|BOLD:ABW5634
Thrips|[4205]|RRINV2795-15|624[0n]bp|Thysanoptera|Thripidae|
Thripinae|[4206]|RRMFG2582-15|582[0n]bp|Thysanoptera|Thripidae|BOLD:AAD4600
Janus|[4207]|RRMFE2970-15|591[0n]bp|Hymenoptera|Cephidae|BOLD:AAD8205
Psyllidae|[4208]|RRINV3638-15|613[0n]bp|Hemiptera|Psyllidae|
Psyllidae|[4209]|RRMPG036-15|525[0n]bp|Hemiptera|Psyllidae|BOLD:ACV4988
Psyllidae|[4210]|RRSSA1948-15|603[6n]bp|Hemiptera|Psyllidae|
Derbidae|[4211]|RRINV2686-15|635[0n]bp|Hemiptera|Derbidae|
Liburniella ornata|[4212]|RRINV3084-15|629[0n]bp|Hemiptera|Delphacidae|
Muirodelphax|[4213]|RRINV2593-15|629[0n]bp|Hemiptera|Delphacidae|
Delphacinae|[4214]|RRSSA1922-15|564[0n]bp|Hemiptera|Delphacidae|BOLD:AAV0160
Delphacidae|[4215]|RRINV3070-15|614[1n]bp|Hemiptera|Delphacidae|

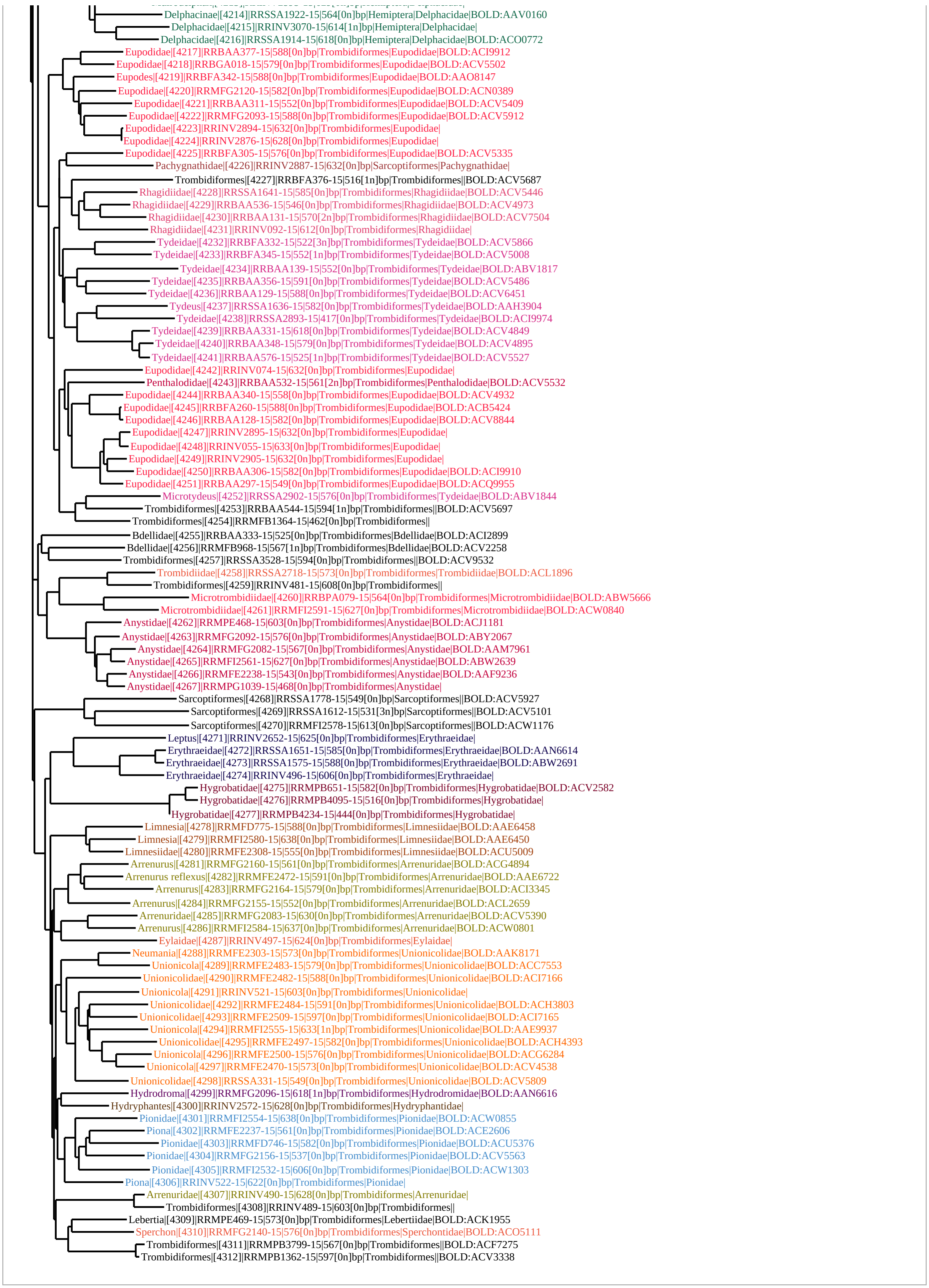
Delphacinae|[4214]|RRSSA1922-15|564[0n]bp|Hemiptera|Delphacidae|BOLD:AAV0160
Delphacidae|[4215]|RRINV3070-15|614[1n]bp|Hemiptera|Delphacidae|
Delphacidae|[4216]|RRSSA1914-15|618[0n]bp|Hemiptera|Delphacidae|BOLD:ACO0772
Eupodidae|[4217]|RRBAA377-15|588[0n]bp|Trombidiformes|Eupodidae|BOLD:ACI9912
Eupodidae|[4218]|RRBGA018-15|579[0n]bp|Trombidiformes|Eupodidae|BOLD:ACV5502
Eupodes|[4219]|RRBFA342-15|588[0n]bp|Trombidiformes|Eupodidae|BOLD:AAO8147
Eupodidae|[4220]|RRMFG2120-15|582[0n]bp|Trombidiformes|Eupodidae|BOLD:ACN0389
Eupodidae|[4221]|RRBAA311-15|552[0n]bp|Trombidiformes|Eupodidae|BOLD:ACV5409
Eupodidae|[4222]|RRMFG2093-15|588[0n]bp|Trombidiformes|Eupodidae|BOLD:ACV5912
Eupodidae|[4223]|RRINV2894-15|632[0n]bp|Trombidiformes|Eupodidae|
Eupodidae|[4224]|RRINV2876-15|628[0n]bp|Trombidiformes|Eupodidae|
Eupodidae|[4225]|RRBFA305-15|576[0n]bp|Trombidiformes|Eupodidae|BOLD:ACV5335
Pachygnathidae|[4226]|RRINV2887-15|632[0n]bp|Sarcoptiformes|Pachygnathidae|
Trombidiformes|[4227]|RRBFA376-15|516[1n]bp|Trombidiformes||BOLD:ACV5687
Rhagidiidae|[4228]|RRSSA1641-15|585[0n]bp|Trombidiformes|Rhagidiidae|BOLD:ACV5446
Rhagidiidae|[4229]|RRBAA536-15|546[0n]bp|Trombidiformes|Rhagidiidae|BOLD:ACV4973
Rhagidiidae|[4230]|RRBAA131-15|570[2n]bp|Trombidiformes|Rhagidiidae|BOLD:ACV7504
Rhagidiidae|[4231]|RRINV092-15|612[0n]bp|Trombidiformes|Rhagidiidae|
Tydeidae|[4232]|RRBFA332-15|522[3n]bp|Trombidiformes|Tydeidae|BOLD:ACV5866
Tydeidae|[4233]|RRBFA345-15|552[1n]bp|Trombidiformes|Tydeidae|BOLD:ACV5008
Tydeidae|[4234]|RRBAA139-15|552[0n]bp|Trombidiformes|Tydeidae|BOLD:ABV1817
Tydeidae|[4235]|RRBAA356-15|591[0n]bp|Trombidiformes|Tydeidae|BOLD:ACV5486
Tydeidae|[4236]|RRBAA129-15|588[0n]bp|Trombidiformes|Tydeidae|BOLD:ACV6451
Tydeus|[4237]|RRSSA1636-15|582[0n]bp|Trombidiformes|Tydeidae|BOLD:AAH3904
Tydeidae|[4238]|RRSSA2893-15|417[0n]bp|Trombidiformes|Tydeidae|BOLD:ACI9974
Tydeidae|[4239]|RRBAA331-15|618[0n]bp|Trombidiformes|Tydeidae|BOLD:ACV4849
Tydeidae|[4240]|RRBAA348-15|579[0n]bp|Trombidiformes|Tydeidae|BOLD:ACV4895
Tydeidae|[4241]|RRBAA576-15|525[1n]bp|Trombidiformes|Tydeidae|BOLD:ACV5527
Eupodidae|[4242]|RRINV074-15|632[0n]bp|Trombidiformes|Eupodidae|
Penthalodidae|[4243]|RRBAA532-15|561[2n]bp|Trombidiformes|Penthalodidae|BOLD:ACV5532
Eupodidae|[4244]|RRBAA340-15|558[0n]bp|Trombidiformes|Eupodidae|BOLD:ACV4932
Eupodidae|[4245]|RRBFA260-15|588[0n]bp|Trombidiformes|Eupodidae|BOLD:ACB5424
Eupodidae|[4246]|RRBAA128-15|582[0n]bp|Trombidiformes|Eupodidae|BOLD:ACV8844
Eupodidae|[4247]|RRINV2895-15|632[0n]bp|Trombidiformes|Eupodidae|
Eupodidae|[4248]|RRINV055-15|633[0n]bp|Trombidiformes|Eupodidae|
Eupodidae|[4249]|RRINV2905-15|632[0n]bp|Trombidiformes|Eupodidae|
Eupodidae|[4250]|RRBAA306-15|582[0n]bp|Trombidiformes|Eupodidae|BOLD:ACI9910
Eupodidae|[4251]|RRBAA297-15|549[0n]bp|Trombidiformes|Eupodidae|BOLD:ACQ9955
Microtydeus|[4252]|RRSSA2902-15|576[0n]bp|Trombidiformes|Tydeidae|BOLD:ABV1844
Trombidiformes|[4253]|RRBAA544-15|594[1n]bp|Trombidiformes||BOLD:ACV5697
Trombidiformes|[4254]|RRMFB1364-15|462[0n]bp|Trombidiformes||
Bdellidae|[4255]|RRBAA333-15|525[0n]bp|Trombidiformes|Bdellidae|BOLD:ACI2899
Bdellidae|[4256]|RRMFB968-15|567[1n]bp|Trombidiformes|Bdellidae|BOLD:ACV2258
Trombidiformes|[4257]|RRSSA3528-15|594[0n]bp|Trombidiformes||BOLD:ACV9532
Trombidiidae|[4258]|RRSSA2718-15|573[0n]bp|Trombidiformes|Trombidiidae|BOLD:ACL1896
Trombidiformes|[4259]|RRINV481-15|608[0n]bp|Trombidiformes||
Microtrombidiidae|[4260]|RRBPA079-15|564[0n]bp|Trombidiformes|Microtrombidiidae|BOLD:ABW5666
Microtrombidiidae|[4261]|RRMFI2591-15|627[0n]bp|Trombidiformes|Microtrombidiidae|BOLD:ACW0840
Anystidae|[4262]|RRMPE468-15|603[0n]bp|Trombidiformes|Anystidae|BOLD:ACJ1181
Anystidae|[4263]|RRMFG2092-15|576[0n]bp|Trombidiformes|Anystidae|BOLD:ABY2067
Anystidae|[4264]|RRMFG2082-15|567[0n]bp|Trombidiformes|Anystidae|BOLD:AAM7961
Anystidae|[4265]|RRMFI2561-15|627[0n]bp|Trombidiformes|Anystidae|BOLD:ABW2639
Anystidae|[4266]|RRMFE2238-15|543[0n]bp|Trombidiformes|Anystidae|BOLD:AAF9236
Anystidae|[4267]|RRMPG1039-15|468[0n]bp|Trombidiformes|Anystidae|
Sarcoptiformes|[4268]|RRSSA1778-15|549[0n]bp|Sarcoptiformes||BOLD:ACV5927
Sarcoptiformes|[4269]|RRSSA1612-15|531[3n]bp|Sarcoptiformes||BOLD:ACV5101
Sarcoptiformes|[4270]|RRMFI2578-15|613[0n]bp|Sarcoptiformes||BOLD:ACW1176
Leptus|[4271]|RRINV2652-15|625[0n]bp|Trombidiformes|Erythraeidae|
Erythraeidae|[4272]|RRSSA1651-15|585[0n]bp|Trombidiformes|Erythraeidae|BOLD:AAN6614
Erythraeidae|[4273]|RRSSA1575-15|588[0n]bp|Trombidiformes|Erythraeidae|BOLD:ABW2691
Erythraeidae|[4274]|RRINV496-15|606[0n]bp|Trombidiformes|Erythraeidae|
Hygrobatidae|[4275]|RRMPB651-15|582[0n]bp|Trombidiformes|Hygrobatidae|BOLD:ACV2582
Hygrobatidae|[4276]|RRMPB4095-15|516[0n]bp|Trombidiformes|Hygrobatidae|
Hygrobatidae|[4277]|RRMPB4234-15|444[0n]bp|Trombidiformes|Hygrobatidae|
Limnesia|[4278]|RRMFD775-15|588[0n]bp|Trombidiformes|Limnesiidae|BOLD:AAE6458
Limnesia|[4279]|RRMFI2580-15|638[0n]bp|Trombidiformes|Limnesiidae|BOLD:AAE6450
Limnesiidae|[4280]|RRMFE2308-15|555[0n]bp|Trombidiformes|Limnesiidae|BOLD:ACU5009
Arrenurus|[4281]|RRMFG2160-15|561[0n]bp|Trombidiformes|Arrenuridae|BOLD:ACG4894
Arrenurus reflexus|[4282]|RRMFE2472-15|591[0n]bp|Trombidiformes|Arrenuridae|BOLD:AAE6722
Arrenurus|[4283]|RRMFG2164-15|579[0n]bp|Trombidiformes|Arrenuridae|BOLD:ACI3345
Arrenurus|[4284]|RRMFG2155-15|552[0n]bp|Trombidiformes|Arrenuridae|BOLD:ACL2659
Arrenuridae|[4285]|RRMFG2083-15|630[0n]bp|Trombidiformes|Arrenuridae|BOLD:ACV5390
Arrenurus|[4286]|RRMFI2584-15|637[0n]bp|Trombidiformes|Arrenuridae|BOLD:ACW0801
Eylaidae|[4287]|RRINV497-15|624[0n]bp|Trombidiformes|Eylaidae|
Neumania|[4288]|RRMFE2303-15|573[0n]bp|Trombidiformes|Unionicolidae|BOLD:AAK8171
Unionicola|[4289]|RRMFE2483-15|579[0n]bp|Trombidiformes|Unionicolidae|BOLD:ACC7553
Unionicolidae|[4290]|RRMFE2482-15|588[0n]bp|Trombidiformes|Unionicolidae|BOLD:ACI7166
Unionicola|[4291]|RRINV521-15|603[0n]bp|Trombidiformes|Unionicolidae|
Unionicolidae|[4292]|RRMFE2484-15|591[0n]bp|Trombidiformes|Unionicolidae|BOLD:ACH3803
Unionicolidae|[4293]|RRMFE2509-15|597[0n]bp|Trombidiformes|Unionicolidae|BOLD:ACI7165
Unionicola|[4294]|RRMFI2555-15|633[1n]bp|Trombidiformes|Unionicolidae|BOLD:AAE9937
Unionicolidae|[4295]|RRMFE2497-15|582[0n]bp|Trombidiformes|Unionicolidae|BOLD:ACH4393
Unionicola|[4296]|RRMFE2500-15|576[0n]bp|Trombidiformes|Unionicolidae|BOLD:ACG6284
Unionicola|[4297]|RRMFE2470-15|573[0n]bp|Trombidiformes|Unionicolidae|BOLD:ACV4538
Unionicolidae|[4298]|RRSSA331-15|549[0n]bp|Trombidiformes|Unionicolidae|BOLD:ACV5809
Hydrodroma|[4299]|RRMFG2096-15|618[1n]bp|Trombidiformes|Hydrodromidae|BOLD:AAN6616
Hydryphantes|[4300]|RRINV2572-15|628[0n]bp|Trombidiformes|Hydryphantidae|
Pionidae|[4301]|RRMFI2554-15|638[0n]bp|Trombidiformes|Pionidae|BOLD:ACW0855
Piona|[4302]|RRMFE2237-15|561[0n]bp|Trombidiformes|Pionidae|BOLD:ACE2606
Pionidae|[4303]|RRMFD746-15|582[0n]bp|Trombidiformes|Pionidae|BOLD:ACU5376
Pionidae|[4304]|RRMFG2156-15|537[0n]bp|Trombidiformes|Pionidae|BOLD:ACV5563
Pionidae|[4305]|RRMFI2532-15|606[0n]bp|Trombidiformes|Pionidae|BOLD:ACW1303
Piona|[4306]|RRINV522-15|622[0n]bp|Trombidiformes|Pionidae|
Arrenuridae|[4307]|RRINV490-15|628[0n]bp|Trombidiformes|Arrenuridae|
Trombidiformes|[4308]|RRINV489-15|603[0n]bp|Trombidiformes||
Lebertia|[4309]|RRMPE469-15|573[0n]bp|Trombidiformes|Lebertiidae|BOLD:ACK1955
Sperchon|[4310]|RRMFG2140-15|576[0n]bp|Trombidiformes|Sperchontidae|BOLD:ACO5111
Trombidiformes|[4311]|RRMPB3799-15|567[0n]bp|Trombidiformes||BOLD:ACF7275
Trombidiformes|[4312]|RRMPB1362-15|597[0n]bp|Trombidiformes||BOLD:ACV3338
