# Supplementary material for: Biodiversity inventories in high gear: DNA barcoding facilitates a rapid biotic survey of a temperate nature reserve
Source: Biodivers Data J. 2015 Aug 30;(3):e6313. doi: 10.3897/BDJ.3.e6313 (PMC4568406; doi:10.3897/BDJ.3.e6313)
Supplement: Supplementary material 9 — BIN image library [file biodiversity_data_journal-3-e6313-s009.pdf]

Image Library - SEARCH: Process ids(4312 ids)

Print

Parameters

Records Included All records
Filter Image by Orientation: None
Aspect Ratio: Original

Result Summary

Total Records: 4312

Re-render images: 4 per row

5000 Records Per Page - Pages: 1

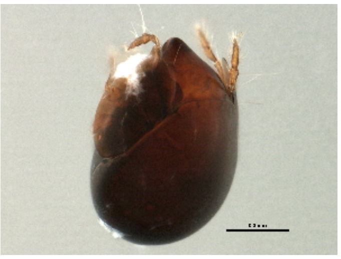

BIOUG08018-H05 [Dorsal]
Galumnidae
Family: Galumnidae
BIN URI: BOLD:ACJ0288

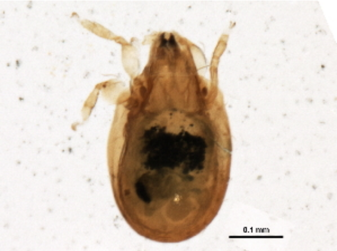

BIOUG01951-C03 [Dorsal]
Oripodidae
Family: Oripodidae
BIN URI: BOLD:ABW2696

IMAGE NOT AVAILABLE

BIOUG24005-F03
Hypochthonius
Family: Hypochthoniidae

IMAGE NOT AVAILABLE

BIOUG24005-H05
Sarcoptiformes

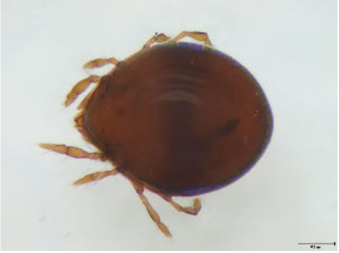

FINOR-20120134 [Dorsal]
Gustavia microcephala
Family: Gustaviidae
BIN URI: BOLD:ACE3149

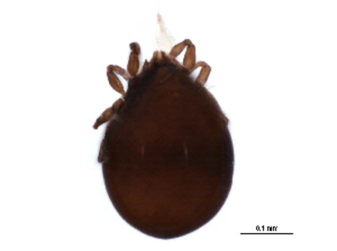

BIOUG21882-A02 [Lateral]
Gustavia
Family: Gustaviidae
BIN URI: BOLD:ACV6005

IMAGE NOT AVAILABLE

BIOUG21899-D07
Sarcoptiformes

BIN URI: BOLD:ACV4851

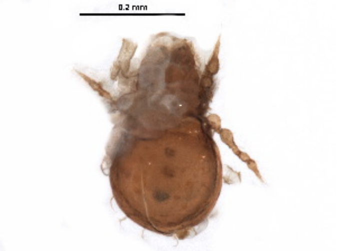

BIOUG21899-D02 [Dorsal]
Sarcoptiformes

BIN URI: BOLD:ACV5637

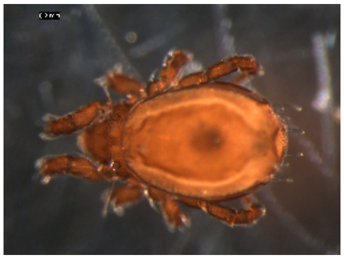

08DPMIT-0831 [Dorsal]
Nothrus
Family: Nothridae
BIN URI: BOLD:AAF9189

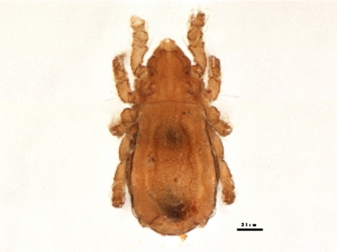

BIOUG08057-C01 [Dorsal]
Nothrus anauniensis
Family: Nothridae
BIN URI: BOLD:AAI2381

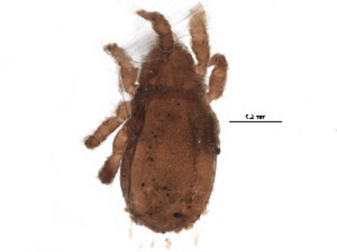

BIOUG21899-B01 [Dorsal]
Nothrus
Family: Nothridae
BIN URI: BOLD:ACV6028

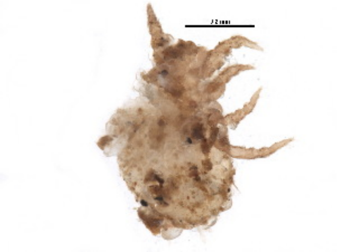

BIOUG21899-B09 [Dorsal]
Nothrus
Family: Nothridae
BIN URI: BOLD:ACV4908

IMAGE NOT AVAILABLE

DPP2.5

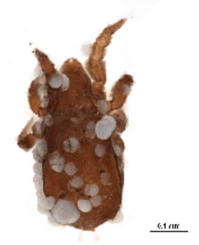

IMAGE NOT AVAILABLE

BIOUG24005-G09

IMAGE NOT AVAILABLE

BIOUG24030-H05

Nothrus cf. anauniensis
Family: Nothridae
BIN URI: BOLD:AAO0965

BIOUG21882-B08 [Lateral]
Nothrus
Family: Nothridae
BIN URI: BOLD:ACV6053

Sarcoptiformes

Sarcoptiformes

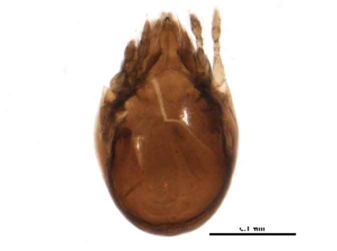

BIOUG21897-B02 [Dorsal]
Ceratozetidae
Family: Ceratozetidae
BIN URI: BOLD:ACV5485

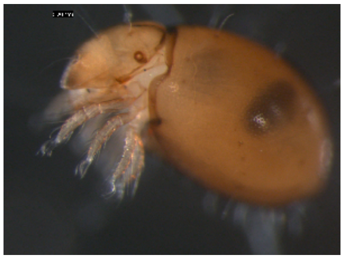

08MIONT-0178 [Dorsal]
Euphthiracaridae
Family: Euphthiracaridae
BIN URI: BOLD:AAF9146

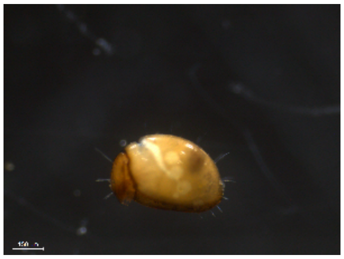

12038-G07 [Lateral]
Arachnida

BIN URI: BOLD:AAF9157

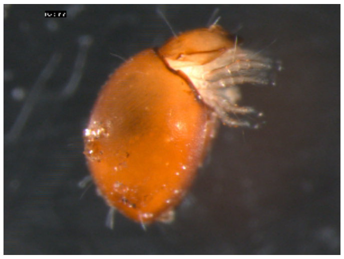

08MIONT-0151 [Dorsal]
Euphthiracaridae
Family: Euphthiracaridae
BIN URI: BOLD:AAF9140

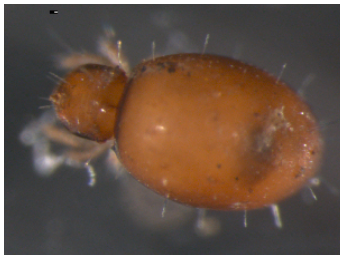

08MIONT-0250 [Dorsal]
Euphthiracaridae
Family: Euphthiracaridae
BIN URI: BOLD:AAM7951

IMAGE NOT AVAILABLE

BIOUG21882-B04
Euphthiracaridae
Family: Euphthiracaridae

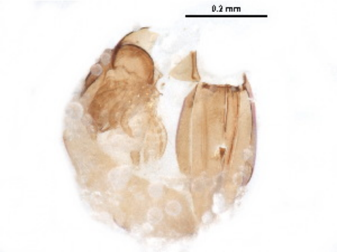

BIOUG21899-A01 [Dorsal]
Euphthiracaridae
Family: Euphthiracaridae
BIN URI: BOLD:ACV5462

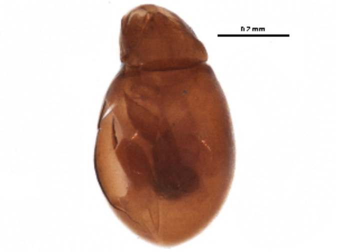

BIOUG24005-B12 [Dorsal]
Phthiracaridae
Family: Phthiracaridae

IMAGE NOT AVAILABLE

BIOUG21899-F06
Tectocepheidae
Family: Tectocepheidae
BIN URI: BOLD:ACV5626

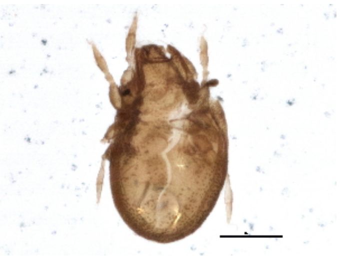

BIOUG06270-B12 [Dorsal]
Tectocepheus sarekensis
Family: Tectocepheidae
BIN URI: BOLD:AAM3402

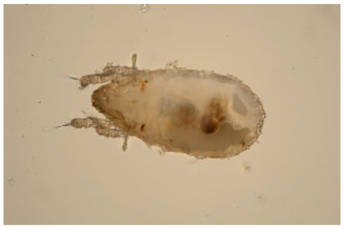

GBOL13631 [Lateral]
Tectocepheus
Family: Tectocepheidae
BIN URI: BOLD:AAM4355

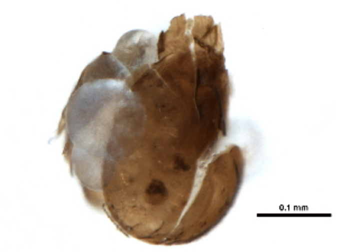

BIOUG12820-D07 [Dorsal]
Arachnida

BIN URI: BOLD:ACO1660

IMAGE NOT AVAILABLE

BIOUG21884-C03
Tectocepheidae
Family: Tectocepheidae

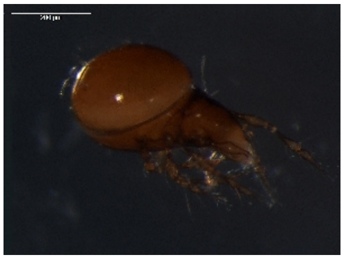

DPMIT-20-08 [Lateral]
Oppia nitens
Family: Oppiidae
BIN URI: BOLD:AAF0868

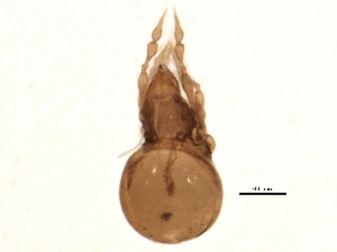

BIOUG15586-F06 [Dorsal]
Arachnida

BIN URI: BOLD:AAH6510

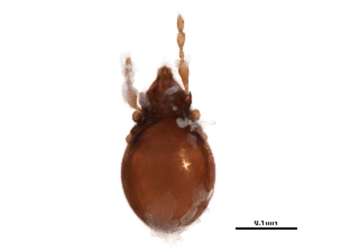

BIOUG20568-F09 [Dorsal]
Suctobelbidae
Family: Suctobelbidae
BIN URI: BOLD:ACV7259

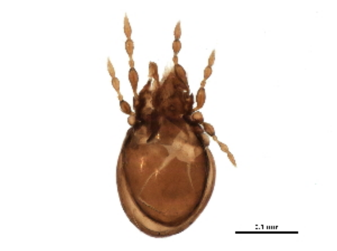

BIOUG20567-C11 [Dorsal]
Suctobelbidae
Family: Suctobelbidae
BIN URI: BOLD:ACV7260

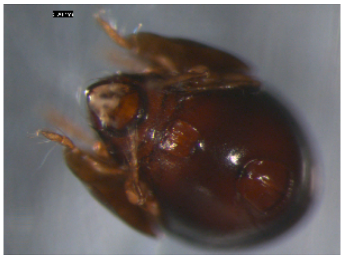

08MIONT-0133 [Ventral]
Galumnidae
Family: Galumnidae
BIN URI: BOLD:AAF9138

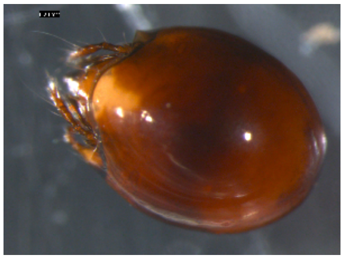

08MIONT-0138 [Dorsal]
Euzetes globulus
Family: Euzetidae
BIN URI: BOLD:AAF9090

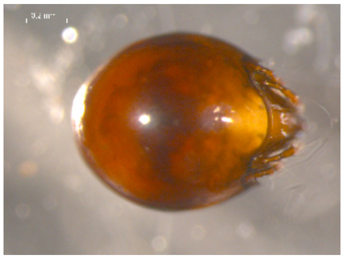

08MIONT-0277 [Dorsal]
Euzetidae
Family: Euzetidae
BIN URI: BOLD:AAH6519

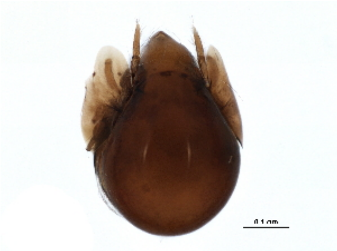

BIOUG20568-C06 [Dorsal]
Galumnidae
Family: Galumnidae
BIN URI: BOLD:ACV7095

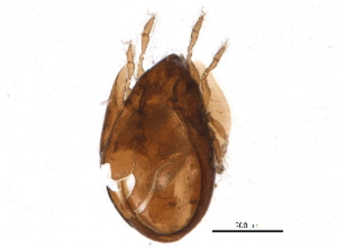

BIOUG06925-B10 [Dorsal]
Sarcoptiformes

BIN URI: BOLD:ACI5993

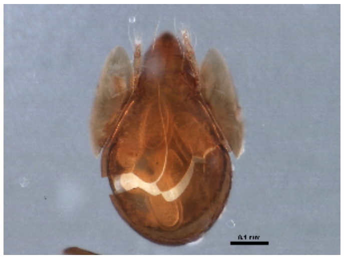

BIOUG06813-B12 [Dorsal]
Parakalummidae
Family: Parakalummidae
BIN URI: BOLD:ACI7088

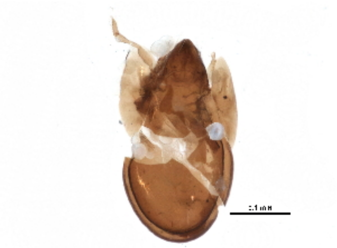

BIOUG22631-H08 [Dorsal]
Parakalummidae
Family: Parakalummidae
BIN URI: BOLD:ACV5542

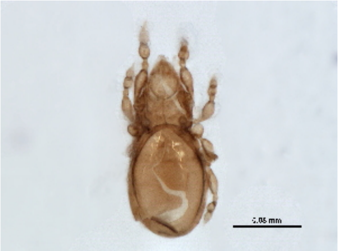

BIOUG20568-F07 [Dorsal]
Oppiidae
Family: Oppiidae
BIN URI: BOLD:ACV6645

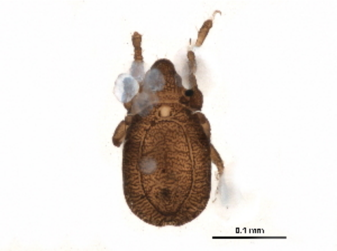

BIOUG22631-G12 [Dorsal]
Cymbaeremaeidae
Family: Cymbaeremaeidae
BIN URI: BOLD:ACV5273

IMAGE NOT AVAILABLE

BIOUG20568-F11
Sarcoptiformes

BIN URI: BOLD:ACV6449

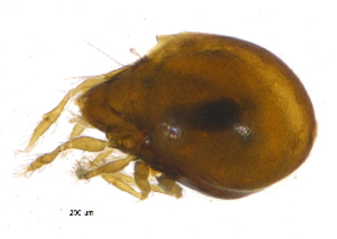

BIOUG01954-D05 [Lateral]
Podoribates pratensis
Family: Mochlozetidae
BIN URI: BOLD:AAF9173

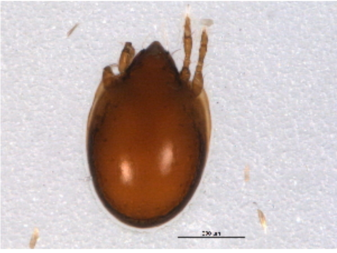

08DPMIT-0737 [Dorsal]
Scheloribates clavilanceolatus
Family: Scheloribatidae
BIN URI: BOLD:AAF9097

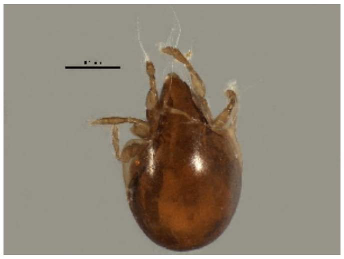

BIOUG08036-B07 [Dorsal]
Scheloribates clavilanceolatus
Family: Scheloribatidae
BIN URI: BOLD:AAI0689

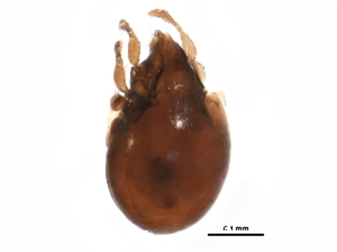

BIOUG21884-B10 [Dorsal]
Scheloribates
Family: Scheloribatidae
BIN URI: BOLD:ACV5484

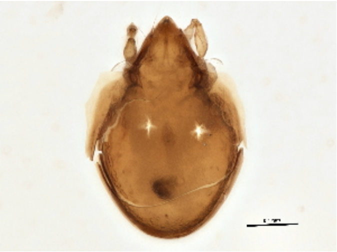

09DPMIT-0019 [Dorsal]
Scheloribates
Family: Scheloribatidae
BIN URI: BOLD:AAF9195

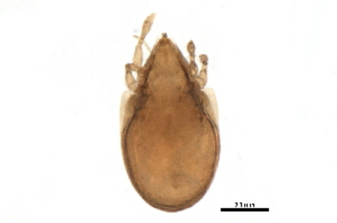

BIOUG11815-A10 [Dorsal]
Scheloribatidae
Family: Scheloribatidae
BIN URI: BOLD:ACG4502

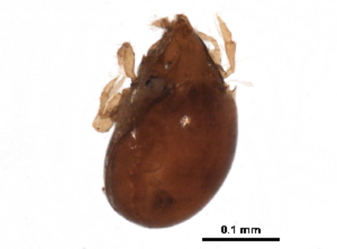

BIOUG08057-H09 [Dorsal]
Scheloribates
Family: Scheloribatidae
BIN URI: BOLD:ACI9964

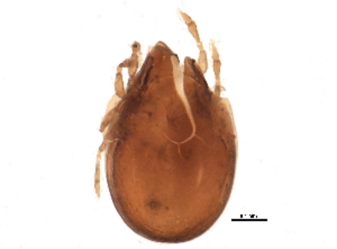

BIOUG08057-C12 [Dorsal]
Scheloribates
Family: Scheloribatidae
BIN URI: BOLD:ACV5039

IMAGE NOT AVAILABLE

BIOUG21886-E06
Scheloribates
Family: Scheloribatidae

IMAGE NOT AVAILABLE

BIOUG24030-G04
Scheloribatidae
Family: Scheloribatidae

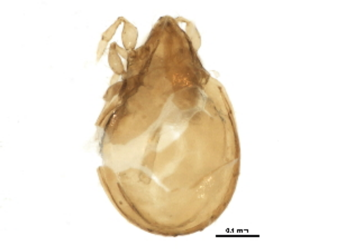

BIOUG01954-A01 [Dorsal]
Scheloribatidae
Family: Scheloribatidae
BIN URI: BOLD:AAH6515

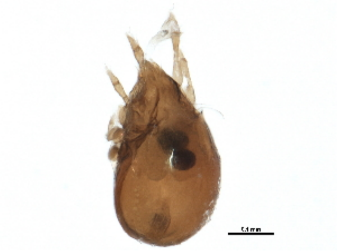

BIOUG13140-H08 [Dorsal]
Arachnida

BIN URI: BOLD:ACV4431

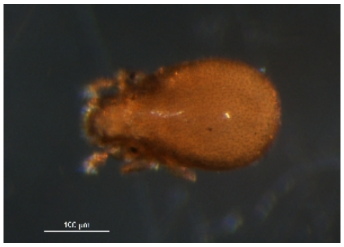

BIOUG01183-19 [Dorsal]
Tectocepheus
Family: Tectocepheidae
BIN URI: BOLD:ACV6209

IMAGE NOT AVAILABLE

BIOUG24005-E08
Sarcoptiformes

IMAGE NOT AVAILABLE

BIOUG24005-E01
Sarcoptiformes

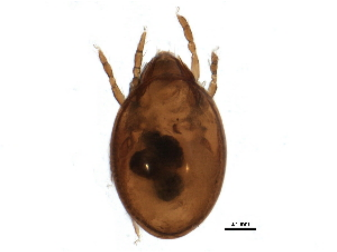

BIOUG10982-E03 [Dorsal]
Sarcoptiformes

BIN URI: BOLD:AAF9291

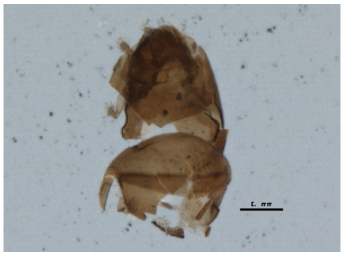

BIOUG10703-E08 [Dorsal]
Tegoribatidae
Family: Tegoribatidae
BIN URI: BOLD:ACL8086

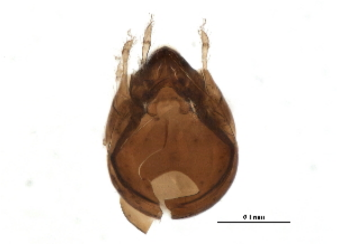

BIOUG11206-G10 [Dorsal]
Sarcoptiformes

BIN URI: BOLD:ACL7966

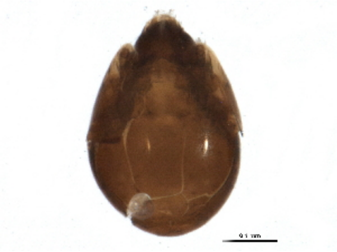

BIOUG13475-A01 [Dorsal]
Arachnida

BIN URI: BOLD:ACO1528

IMAGE NOT AVAILABLE

BIOUG21897-F02
Ceratozetidae
Family: Ceratozetidae
BIN URI: BOLD:ACV5233

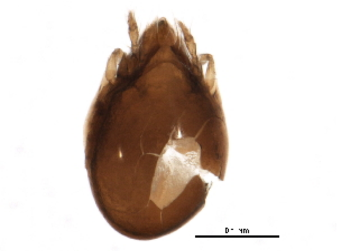

BIOUG21897-B07 [Dorsal]
Ceratozetidae
Family: Ceratozetidae
BIN URI: BOLD:ACV5252

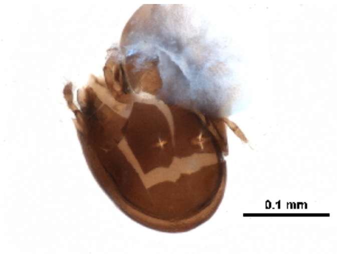

BIOUG20565-E02 [Dorsal]
Ceratozetidae
Family: Ceratozetidae
BIN URI: BOLD:ACV3447

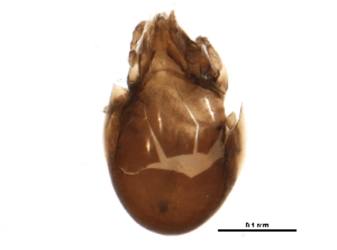

BIOUG21897-B06 [Dorsal]
Ceratozetidae
Family: Ceratozetidae
BIN URI: BOLD:ACV5672

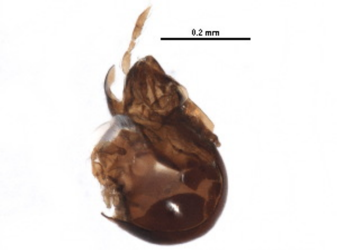

BIOUG22837-A01 [Dorsal]
Punctoribates punctum
Family: Mycobatidae
BIN URI: BOLD:AAH6516

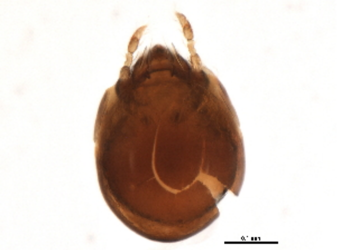

BIOUG12820-D05 [Dorsal]
Arachnida

BIN URI: BOLD:ACB6310

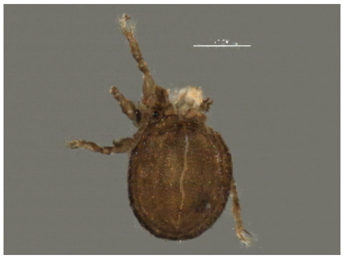

BIOUG09600-F01 [Dorsal]
Sarcoptiformes

BIN URI: BOLD:ACL0449

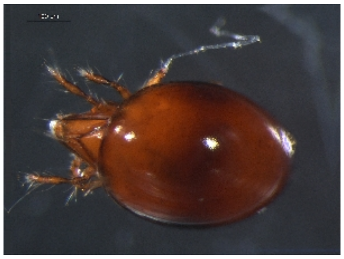

DPMIT-32-56 [Dorsal]
Dorycranosus acutidens
Family: Liacaridae
BIN URI: BOLD:AAF9274

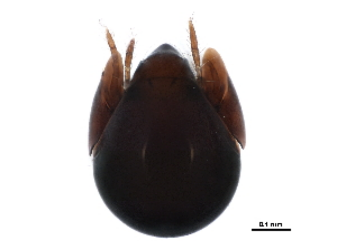

BIOUG20567-F01 [Dorsal]
Galumnidae
Family: Galumnidae
BIN URI: BOLD:ACW0678

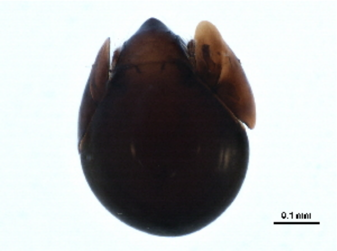

BIOUG20565-A03 [Dorsal]
Galumnidae
Family: Galumnidae
BIN URI: BOLD:ACV4708

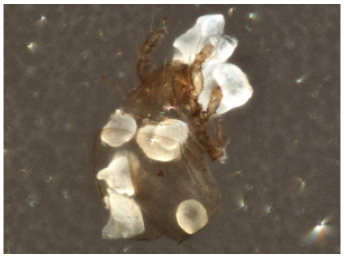

09DPMIT-0010 [Dorsal]
Galumnidae
Family: Galumnidae
BIN URI: BOLD:AAF9192

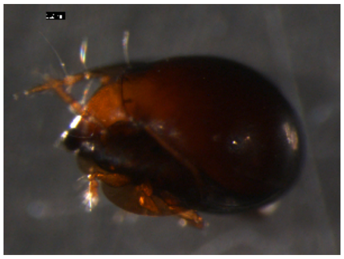

08DPMIT-0900 [Dorsal]
Galumnidae
Family: Galumnidae
BIN URI: BOLD:ACP1171

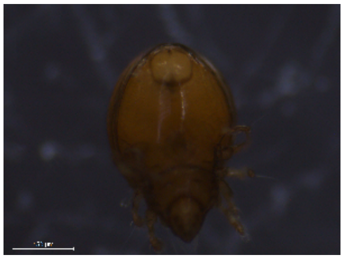

11982-H09 [Ventral]
Arachnida

BIN URI: BOLD:AAM4805

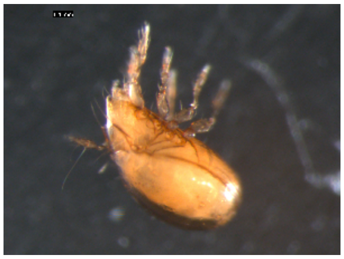

08MIONT-0159 [Dorsal]
Oribatulidae
Family: Oribatulidae
BIN URI: BOLD:ACI4357

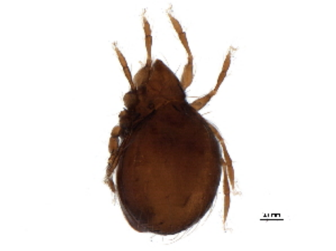

BIOUG10061-F05 [Dorsal]
Oribatulidae
Family: Oribatulidae
BIN URI: BOLD:ACT6033

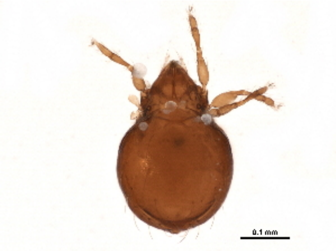

BIOUG22631-F12 [Dorsal]
Oribatulidae
Family: Oribatulidae
BIN URI: BOLD:AAL8123

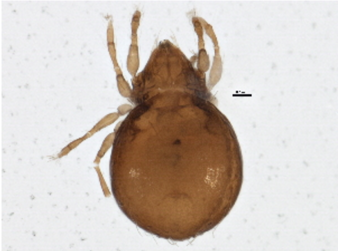

BIOUG05589-B05 [Dorsal]
Oribatulidae
Family: Oribatulidae
BIN URI: BOLD:ACF9074

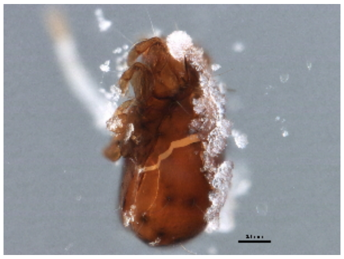

BIOUG11527-F12 [Dorsal]
Ceratozetidae
Family: Ceratozetidae
BIN URI: BOLD:ACM4402

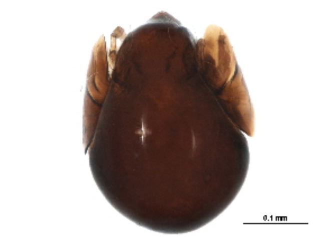

BIOUG21882-G01 [Lateral]
Galumnidae
Family: Galumnidae
BIN URI: BOLD:ACV4882

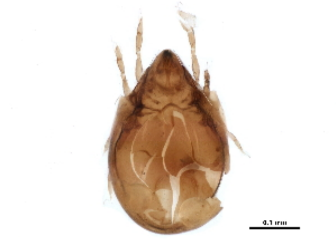

BIOUG20567-D09 [Dorsal]
Ceratozetidae
Family: Ceratozetidae
BIN URI: BOLD:ACV6450

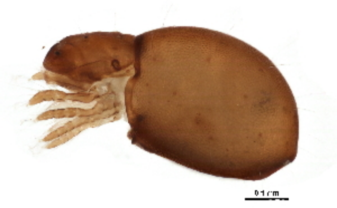

BIOUG20567-D01 [Lateral]
Ceratozetidae
Family: Ceratozetidae
BIN URI: BOLD:ACV7094

IMAGE NOT AVAILABLE

BIOUG24030-E10
Sarcoptiformes

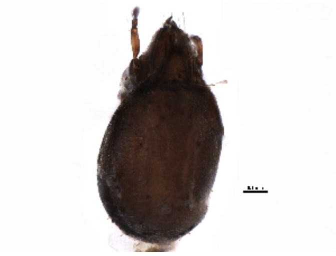

BIOUG14143-D09 [Dorsal]
Arachnida

BIN URI: BOLD:ACP1000

IMAGE NOT AVAILABLE

BIOUG24000-B08
Sarcoptiformes

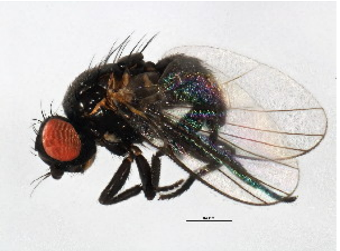

BIOUG00819-D06 [Lateral]
Agromyzidae
Family: Agromyzidae
BIN URI: BOLD:AAG6907

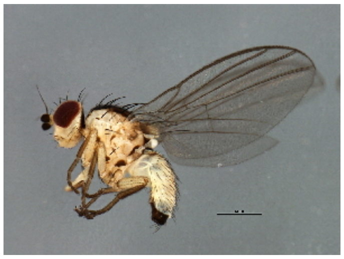

MTDIC-0260 [Lateral]
Phytoliriomyza dorsata
Family: Agromyzidae
BIN URI: BOLD:AAG4751

IMAGE NOT AVAILABLE

BIOUG22416-C05
Agromyzidae
Family: Agromyzidae

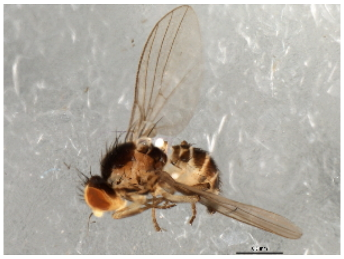

08TTML-1445 [Lateral]
Agromyzidae
Family: Agromyzidae
BIN URI: BOLD:AAG4781

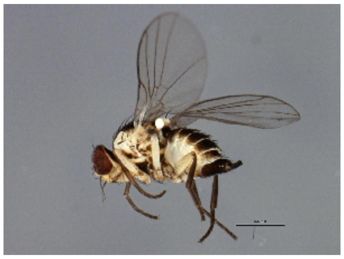

08TTML-0977 [Lateral]
Liriomyza brassicae
Family: Agromyzidae
BIN URI: BOLD:AAF6806

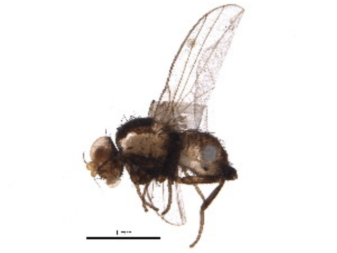

BIOUG20570-D12 [Lateral]
Agromyzidae
Family: Agromyzidae
BIN URI: BOLD:ACV5125

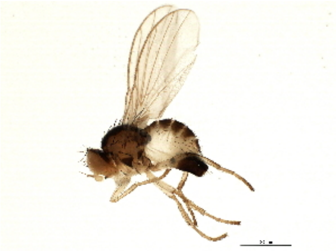

BIOUG01589-E08 [Lateral]
Liriomyza fricki
Family: Agromyzidae
BIN URI: BOLD:AAK8756

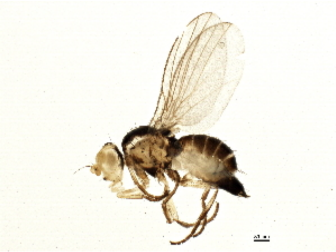

BIOUG03410-A05 [Lateral]
Liriomyza
Family: Agromyzidae
BIN URI: BOLD:ACE7414

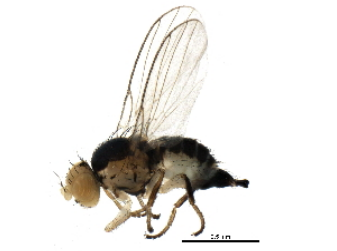

BIOUG24002-B10 [Lateral]
Agromyzidae
Family: Agromyzidae

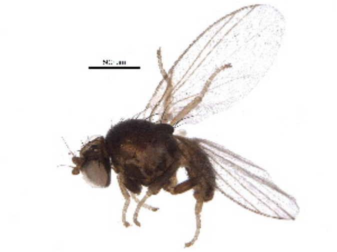

BIOUG22356-E03 [Lateral]
Agromyzidae
Family: Agromyzidae
BIN URI: BOLD:AAN5429

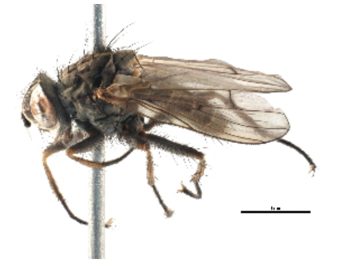

BIOUG08978-F07 [Lateral]
Anthomyiidae
Family: Anthomyiidae
BIN URI: BOLD:ACL0478

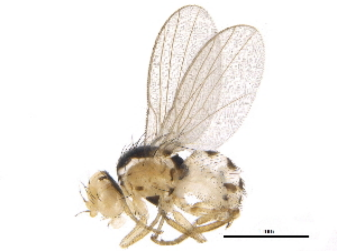

BIOUG22330-D05 [Lateral]
Agromyzidae
Family: Agromyzidae
BIN URI: BOLD:AAV4836

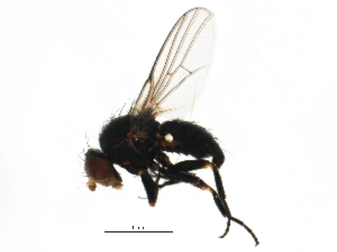

BIOUG22361-B06 [Lateral]
Agromyzidae
Family: Agromyzidae
BIN URI: BOLD:ACV2367

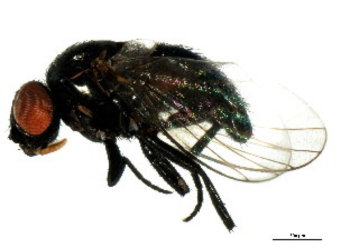

BIOUG01348-B10 [Lateral]
Ophiomyia nasuta
Family: Agromyzidae
BIN URI: BOLD:AAK5607

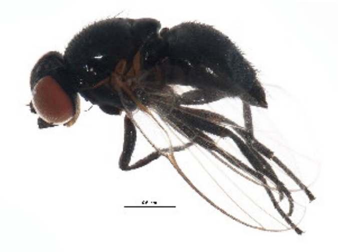

BIOUG01360-B04 [Lateral]
Agromyzidae
Family: Agromyzidae
BIN URI: BOLD:AAN5441

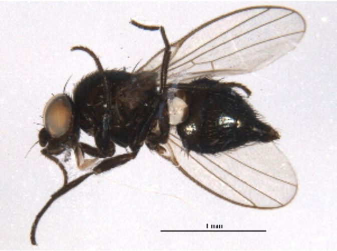

BIOUG04247-A03 [Lateral]
Agromyzidae
Family: Agromyzidae
BIN URI: BOLD:AAP6781

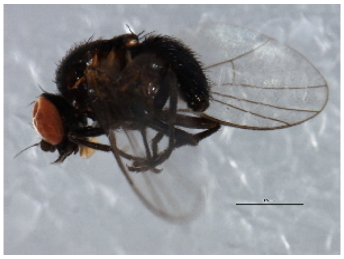

08TTML-0966 [Lateral]
Ophiomyia
Family: Agromyzidae
BIN URI: BOLD:AAG6954

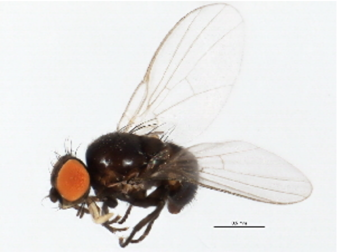

08TTML-2475 [Lateral]
Ophiomyia quinta
Family: Agromyzidae
BIN URI: BOLD:AAI3360

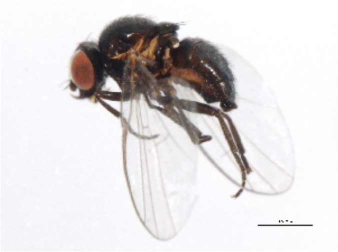

09BBEDI-0613 [Lateral]
Ophiomyia
Family: Agromyzidae
BIN URI: BOLD:ABZ1036

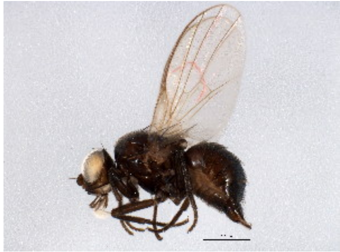

BIOUG00896-C10 [Lateral]
Agromyzidae
Family: Agromyzidae
BIN URI: BOLD:AAN6235

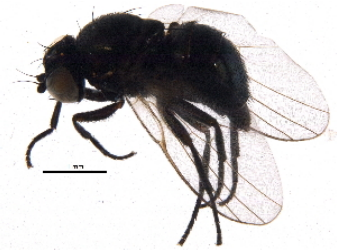

BIOUG10528-G05 [Lateral]
Ophiomyia
Family: Agromyzidae
BIN URI: BOLD:ACL6244

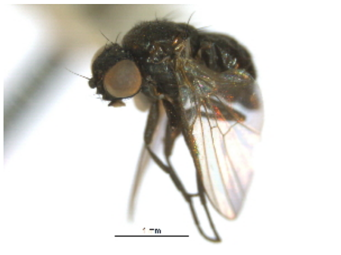

CCDB-21422-B06 [Lateral]
Ophiomyia labiatarum
Family: Agromyzidae
BIN URI: BOLD:ACL9630

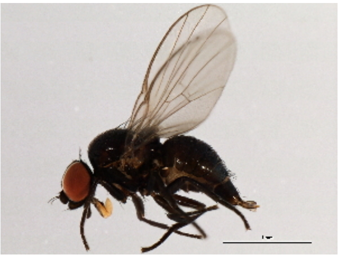

08TTML-1856 [Lateral]
Agromyzidae
Family: Agromyzidae
BIN URI: BOLD:AAN5434

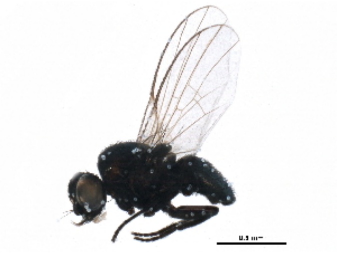

BIOUG21780-H08 [Lateral]
Diptera

BIN URI: BOLD:ACV1282

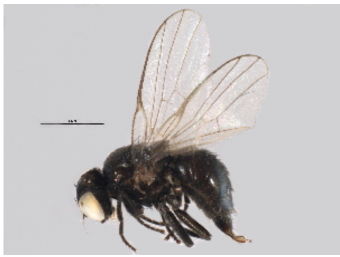

BIOUG11389-D09 [Lateral]
Agromyzidae
Family: Agromyzidae
BIN URI: BOLD:ACM7529

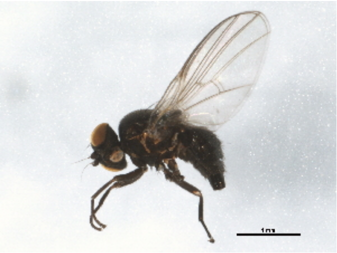

BIOUG01685-G11 [Lateral]
Agromyzidae
Family: Agromyzidae
BIN URI: BOLD:ACV3095

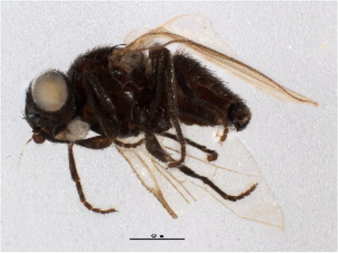

BIOUG01882-G12 [Lateral]
Agromyzidae
Family: Agromyzidae
BIN URI: BOLD:AAP8823

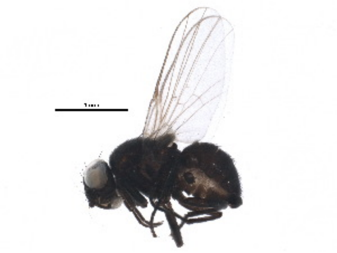

BIOUG23076-E11 [Lateral]
Ophiomyia similata
Family: Agromyzidae
BIN URI: BOLD:ACV5944

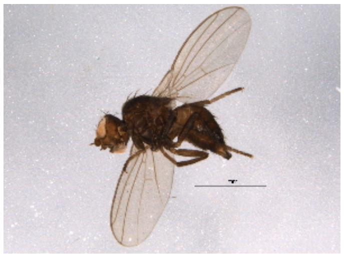

BIOUG06462-E04 [Lateral]
Agromyza frontella
Family: Agromyzidae
BIN URI: BOLD:AAJ7105

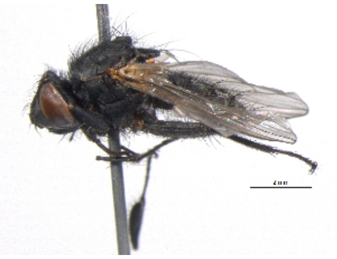

BIOUG05523-E08 [Lateral]
Anthomyiidae
Family: Anthomyiidae
BIN URI: BOLD:ABW1310

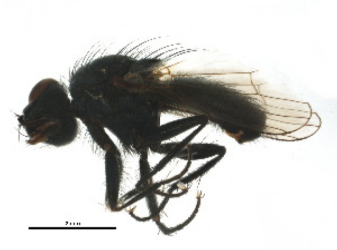

BIOUG21873-D04 [Lateral]
Anthomyiidae
Family: Anthomyiidae
BIN URI: BOLD:ACF8096

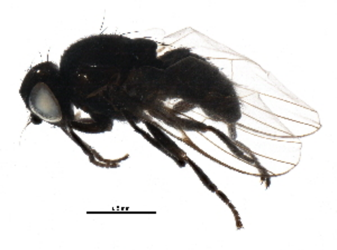

BIOUG22462-H09 [Lateral]
Hexomyza
Family: Agromyzidae
BIN URI: BOLD:ACV2366

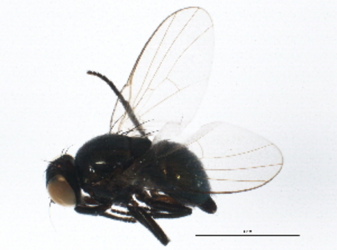

BIOUG03345-D02 [Lateral]
Agromyzidae
Family: Agromyzidae
BIN URI: BOLD:ACC8572

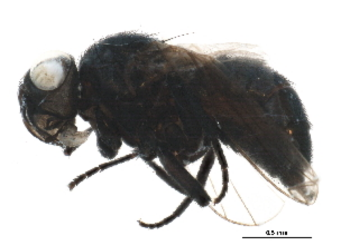

BIOUG21985-C12 [Lateral]
Agromyzidae
Family: Agromyzidae
BIN URI: BOLD:ACU4080

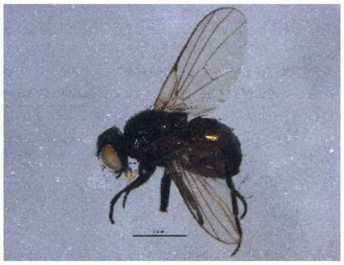

BIOUG12062-C07 [Lateral]
Agromyzidae
Family: Agromyzidae
BIN URI: BOLD:ACM8243

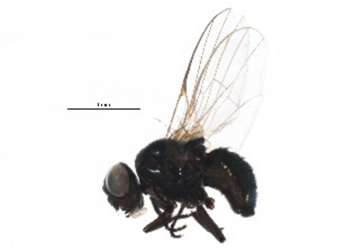

BIOUG21250-F07 [Lateral]
Diptera

BIN URI: BOLD:ACU8028

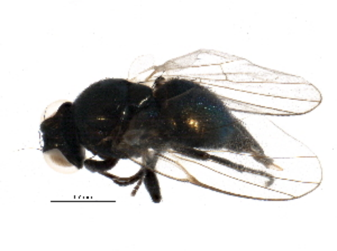

BIOUG22462-D10 [Lateral]
Agromyzidae
Family: Agromyzidae
BIN URI: BOLD:ACV3260

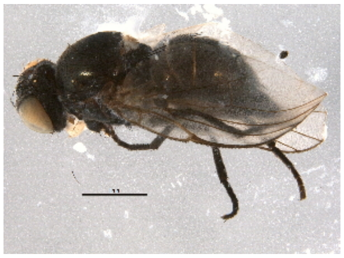

BIOUG05750-A07 [Lateral]
Agromyzidae
Family: Agromyzidae
BIN URI: BOLD:ACG3390

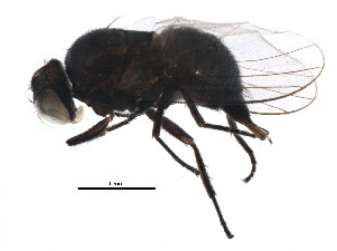

BIOUG22467-C05 [Lateral]
Melanagromyza
Family: Agromyzidae
BIN URI: BOLD:ACV3482

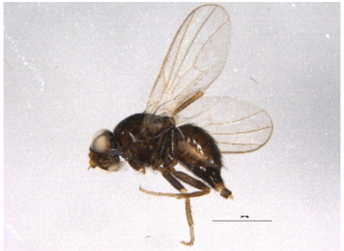

BIOUG05994-E10 [Lateral]
Agromyzidae
Family: Agromyzidae
BIN URI: BOLD:ACG5862

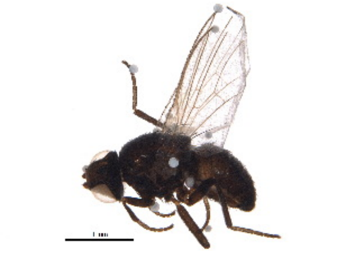

BIOUG20569-G02 [Lateral]
Agromyzidae
Family: Agromyzidae
BIN URI: BOLD:ACV5956

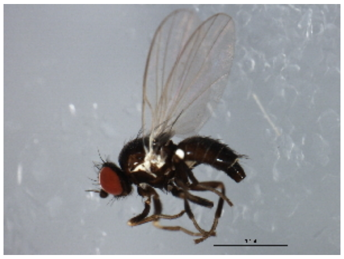

08TTML-0817 [Lateral]
Agromyzidae
Family: Agromyzidae
BIN URI: BOLD:AAN5430

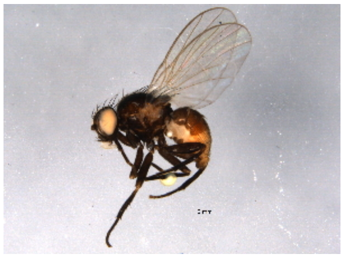

BIOUG05151-A07 [Lateral]
Agromyzidae
Family: Agromyzidae
BIN URI: BOLD:AAG4743

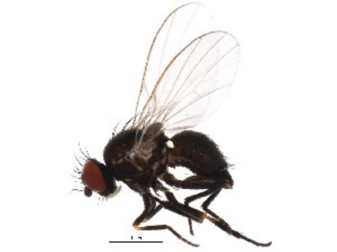

BIOUG01400-A05 [Lateral]
Agromyzidae
Family: Agromyzidae
BIN URI: BOLD:AAG9234

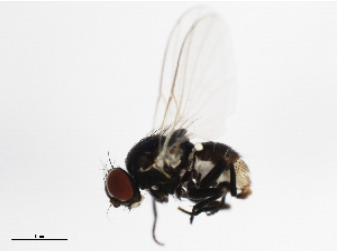

10JSROW-1341 [Lateral]
Nemorimyza posticata
Family: Agromyzidae
BIN URI: BOLD:ACJ0616

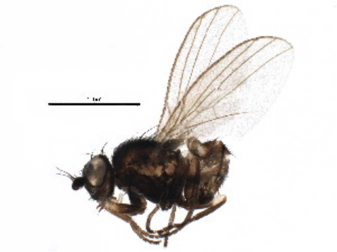

BIOUG22416-A01 [Lateral]
Cerodontha muscina
Family: Agromyzidae
BIN URI: BOLD:AAF1051

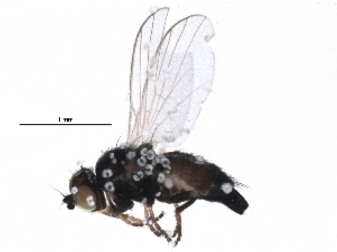

BIOUG24038-H09 [Lateral]
Agromyzidae
Family: Agromyzidae

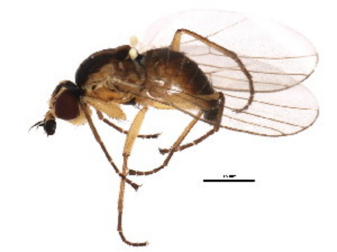

BIOUG01460-G02 [Lateral]
Agromyzidae
Family: Agromyzidae
BIN URI: BOLD:AAD1945

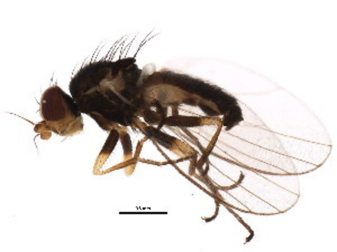

BIOUG01400-B02 [Lateral]
Agromyzidae
Family: Agromyzidae
BIN URI: BOLD:AAG4782

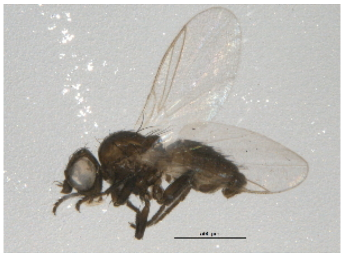

BIOUG08682-B09 [Lateral]
Agromyzidae
Family: Agromyzidae
BIN URI: BOLD:AAM6324

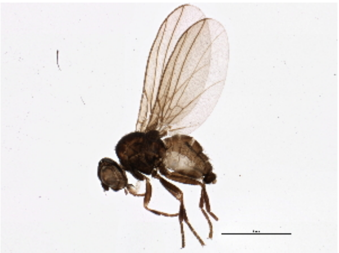

BIOUG04864-D09 [Lateral]
Phytoliriomyza robiniae
Family: Agromyzidae
BIN URI: BOLD:AAY1337

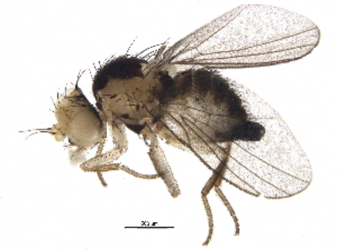

BIOUG08666-B10 [Lateral]
Agromyzidae
Family: Agromyzidae
BIN URI: BOLD:ACK1935

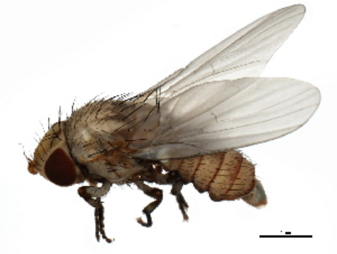

10PHMAL-0881 [Lateral]
Phytobia
Family: Agromyzidae
BIN URI: BOLD:AAM7338

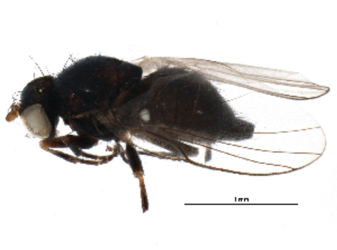

BIOUG22084-D07 [Lateral]
Phytobia
Family: Agromyzidae
BIN URI: BOLD:ACV3622

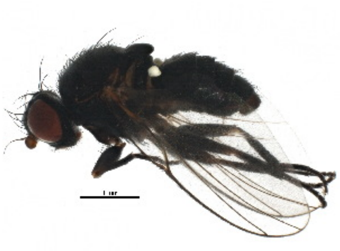

BIOUG22289-E06 [Lateral]
Phytobia
Family: Agromyzidae
BIN URI: BOLD:ACV5074

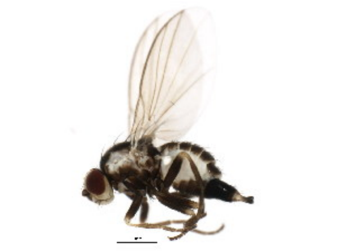

BIOUG01360-H09 [Lateral]
Agromyzidae
Family: Agromyzidae
BIN URI: BOLD:AAG4775

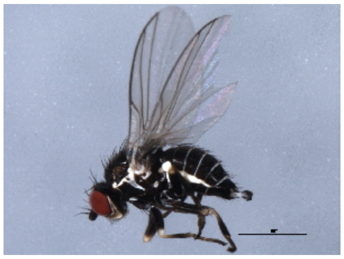

10JSROW-0012 [Lateral]
Phytomyza solidaginophaga
Family: Agromyzidae
BIN URI: BOLD:AAL4176

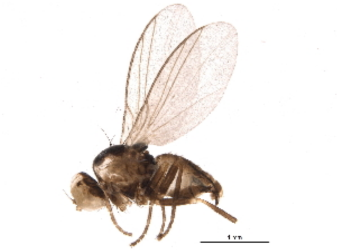

BIOUG05992-F06 [Lateral]
Phytomyza
Family: Agromyzidae
BIN URI: BOLD:ACG5827

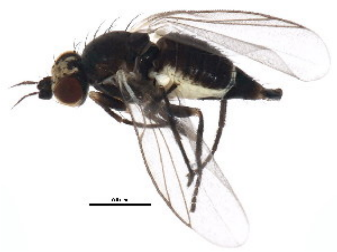

BIOUG01360-D06 [Lateral]
Phytomyza crassiseta
Family: Agromyzidae
BIN URI: BOLD:AAL4268

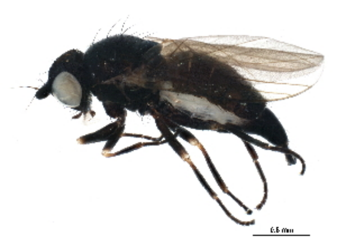

BIOUG22356-E11 [Lateral]
Phytomyza
Family: Agromyzidae
BIN URI: BOLD:ACC4458

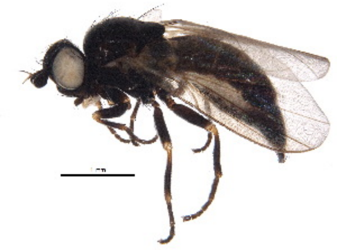

BIOUG22352-D09 [Lateral]
Phytomyza
Family: Agromyzidae
BIN URI: BOLD:ACV4673

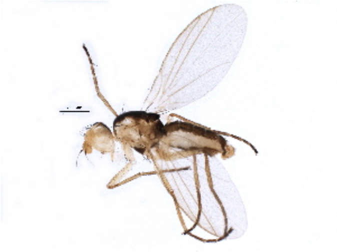

BIOUG15652-C11 [Lateral]
Phytomyza flavicornis
Family: Agromyzidae
BIN URI: BOLD:AAH9376

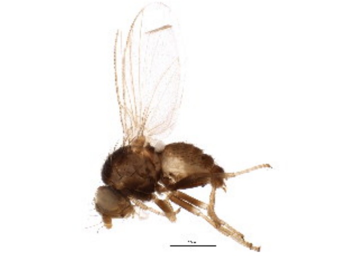

BIOUG01610-D06 [Lateral]
Aulagromyza luteoscutellata
Family: Agromyzidae
BIN URI: BOLD:AAJ9681

IMAGE NOT AVAILABLE

BIOUG22417-D09
Chromatomyia lactuca
Family: Agromyzidae

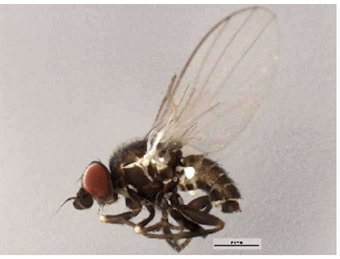

10JSROW-0693 [Lateral]
Cerodontha fasciata
Family: Agromyzidae
BIN URI: BOLD:AAF1049

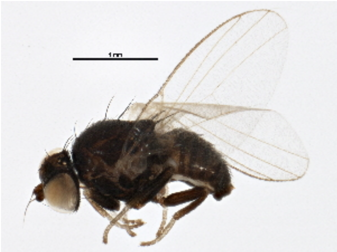

BIOUG03066-F06 [Lateral]
Cerodontha
Family: Agromyzidae
BIN URI: BOLD:ACC7605

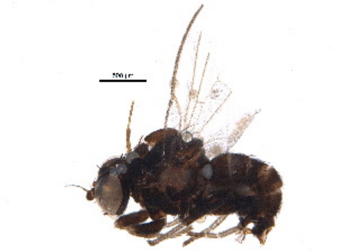

BIOUG20566-E07 [Lateral]
Agromyzidae
Family: Agromyzidae
BIN URI: BOLD:ACV3831

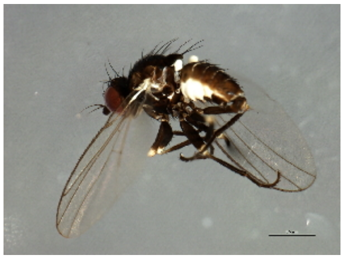

08BBDIP-2752 [Lateral]
Agromyzidae
Family: Agromyzidae
BIN URI: BOLD:AAG4741

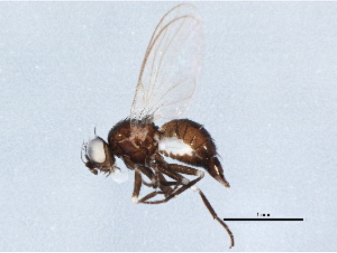

BIOUG01609-C07 [Lateral]
Agromyzidae
Family: Agromyzidae
BIN URI: BOLD:AAV4870

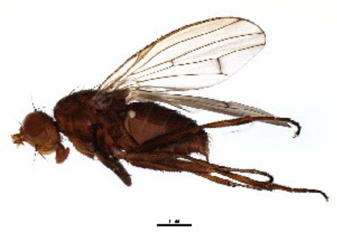

BIOUG01349-G07 [Lateral]
Heleomyzidae
Family: Heleomyzidae
BIN URI: BOLD:AAG0464

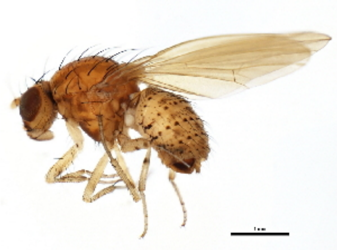

08TTML-1963 [Lateral]
Lauxaniidae
Family: Lauxaniidae
BIN URI: BOLD:AAH3532

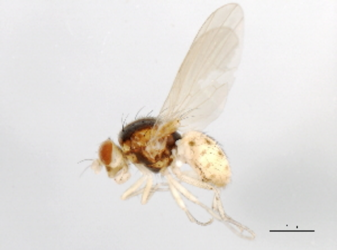

09BBEDI-0491 [Lateral]
Lauxaniidae
Family: Lauxaniidae
BIN URI: BOLD:AAH3541

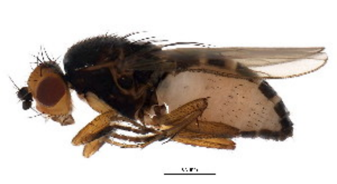

BIOUG01389-C04 [Lateral]
Spelobia ochripes
Family: Sphaeroceridae
BIN URI: BOLD:AAG7279

IMAGE NOT AVAILABLE

BIOUG22084-E06
Poecilominettia puncticeps
Family: Lauxaniidae

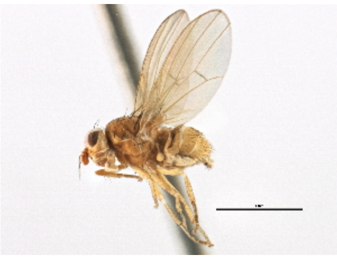

CCDB-21321-B08 [Lateral]
Poecilominettia puncticeps
Family: Lauxaniidae
BIN URI: BOLD:AAN8633

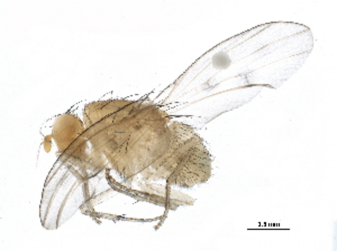

BIOUG24029-A08 [Lateral]
Homoneura
Family: Lauxaniidae

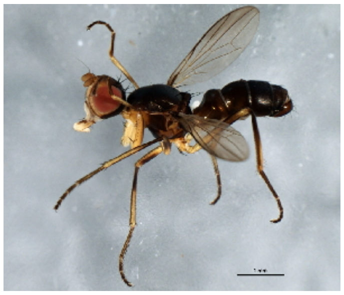

08TTML-1546 [Lateral]
Sepsidae
Family: Sepsidae
BIN URI: BOLD:AAG5640

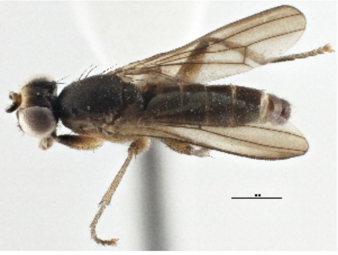

CCDB-21320-B11 [Lateral]
Pteromicra similis
Family: Sciomyzidae
BIN URI: BOLD:AAG6869

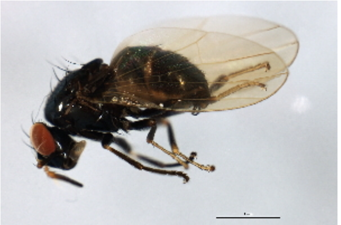

MTDIC-0039 [Lateral]
Lauxania shewelli
Family: Lauxaniidae
BIN URI: BOLD:AAH3531

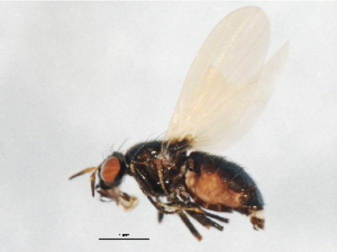

09BBEDI-0761 [Lateral]
Lauxania shewelli
Family: Lauxaniidae
BIN URI: BOLD:ACF4012

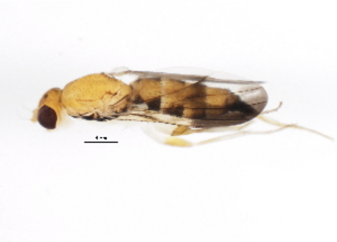

10JSROW-1504 [Lateral]
Clusia czernyi
Family: Clusiidae
BIN URI: BOLD:AAF4394

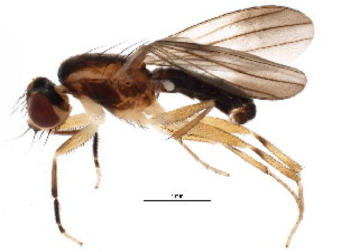

BIOUG01422-F10 [Lateral]
Clusiodes
Family: Clusiidae
BIN URI: BOLD:AAJ4032

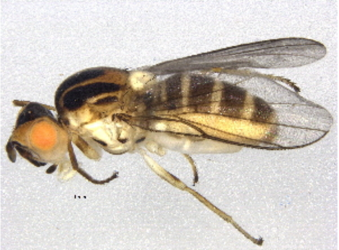

BIOUG08423-B05 [Lateral]
Chloropidae
Family: Chloropidae
BIN URI: BOLD:AAH4204

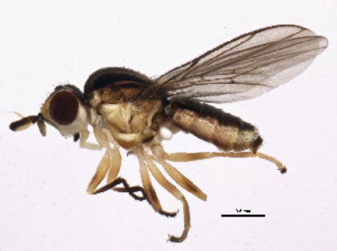

BIOUG00860-G12 [Lateral]
Chloropidae
Family: Chloropidae
BIN URI: BOLD:AAH4211

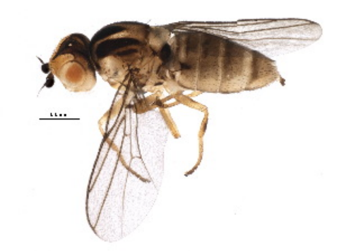

BIOUG20832-A08 [Lateral]
Diptera

BIN URI: BOLD:ACU5909

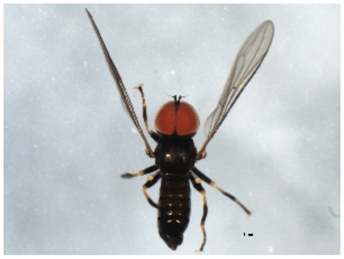

08TTML-1326 [Dorsal]
Pipunculus hertzogi
Family: Pipunculidae
BIN URI: BOLD:AAE4793

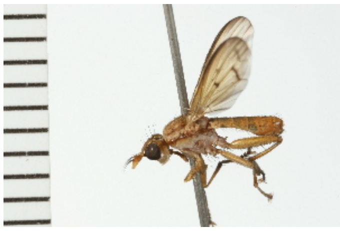

08BBDIP-0995 [Lateral]

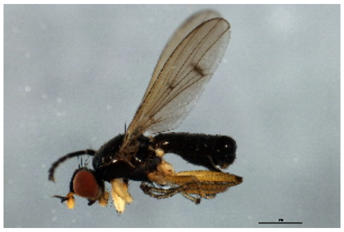

PROBE-TW0042 [Lateral]

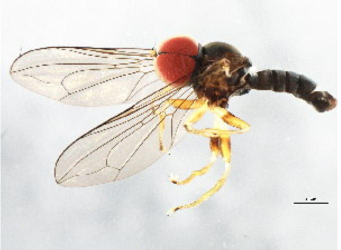

08TTML-2527 [Lateral]
Pipunculus sp. ON11
Family: Pipunculidae
BIN URI: BOLD:AAF1615

Tetanocera plumosa
Family: Sciomyzidae
BIN URI: BOLD:AAC2266

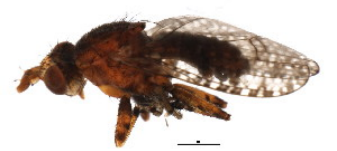

BIOUG00938-H04 [Lateral]
Dictya sp. TAW1
Family: Sciomyzidae
BIN URI: BOLD:AAG3634

Anticheta sp.
Family: Sciomyzidae
BIN URI: BOLD:AAG6971

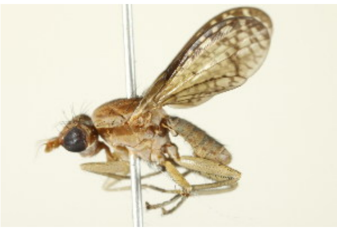

PCPP10-0116 [Lateral]
Trypetoptera canadensis
Family: Sciomyzidae
BIN URI: BOLD:AAD7383

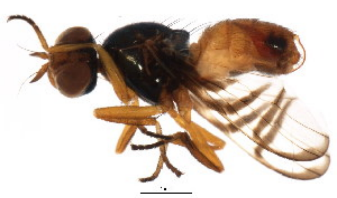

BIOUG01411-G05 [Lateral]
Platystomatidae
Family: Platystomatidae
BIN URI: BOLD:AAP8739

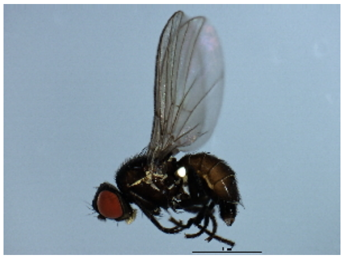

09BBEDI-2378 [Lateral]
Cerodontha biseta
Family: Agromyzidae
BIN URI: BOLD:AAG4780

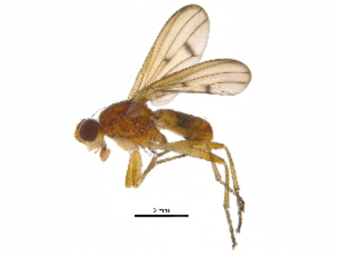

BIOUG22325-H08 [Lateral]
Renocera
Family: Sciomyzidae
BIN URI: BOLD:ACV3909

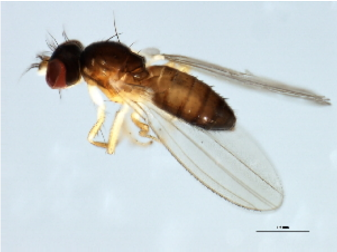

09BBDIP-1642 [Lateral]
Mumetopia occipitalis
Family: Anthomyzidae
BIN URI: BOLD:AAG4827

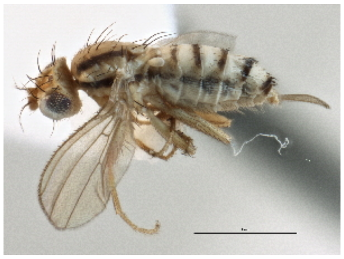

CCDB-21420-D04 [Lateral]
Stiphrosoma balteatum
Family: Anthomyzidae
BIN URI: BOLD:ABA0747

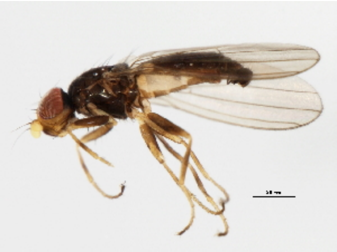

09BBEDI-0664 [Lateral]
Anthomyza sp. OL126DEBU
Family: Anthomyzidae
BIN URI: BOLD:AAD6605

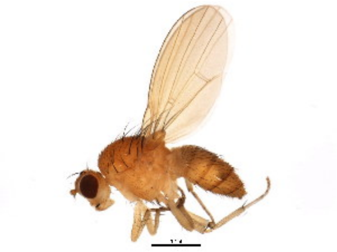

BIOUG01459-H04 [Lateral]
Lauxaniidae
Family: Lauxaniidae
BIN URI: BOLD:AAG6753

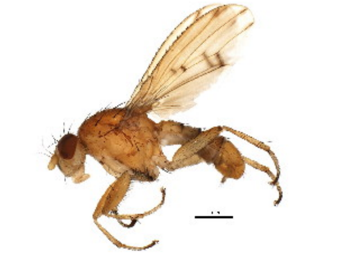

BIOUG01349-B01 [Lateral]
Suillia quinquepunctata
Family: Heleomyzidae
BIN URI: BOLD:AAC8595

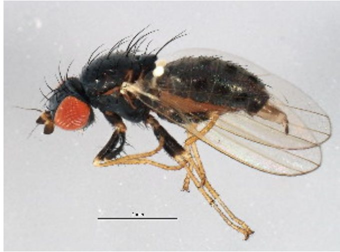

BIOUG01543-D06 [Lateral]
Chamaemyiidae
Family: Chamaemyiidae
BIN URI: BOLD:AAG6951

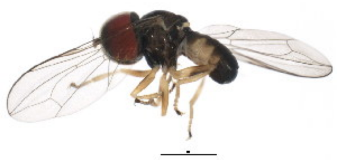

BIOUG00942-B12 [Lateral]
Cephalops varius
Family: Pipunculidae
BIN URI: BOLD:AAP1931

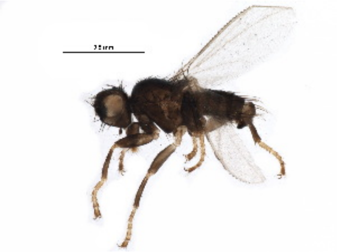

BIOUG22722-A04 [Lateral]
Anthomyiidae
Family: Anthomyiidae
BIN URI: BOLD:ACV5822

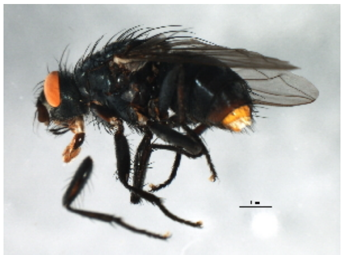

09BBEDI-1710 [Lateral]
Boettcheria cimbicis
Family: Sarcophagidae
BIN URI: BOLD:AAG6756

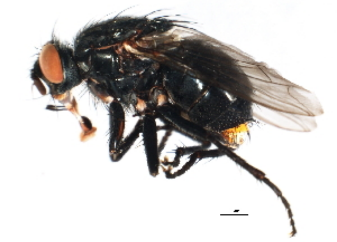

09BBEDI-0828 [Lateral]
Boettcheria bisetosa
Family: Sarcophagidae
BIN URI: BOLD:AAH7139

IMAGE NOT AVAILABLE

BIOUG22580-A09
Helicobia
Family: Sarcophagidae
BIN URI: BOLD:AAA1962

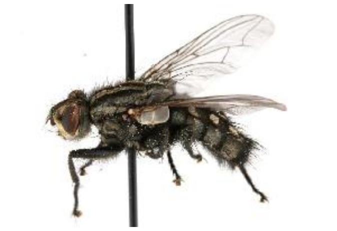

GUE06-FLY-074 [Lateral]
Diptera

BIN URI: BOLD:AAG6743

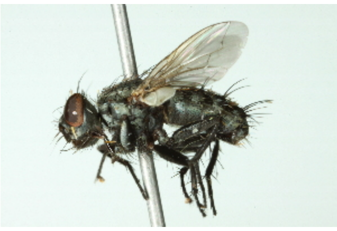

BIOUG01816-A07 [Lateral]
Sarcophagidae
Family: Sarcophagidae
BIN URI: BOLD:AAP1218

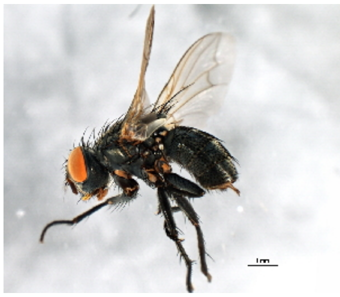

08TTML-1722 [Lateral]
Sarcophagidae
Family: Sarcophagidae
BIN URI: BOLD:AAM9291

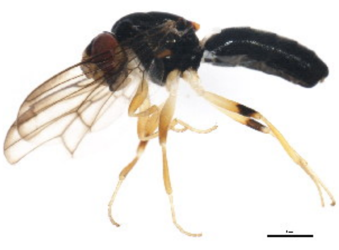

BIOUG01347-D07 [Lateral]
Chyliza
Family: Psilidae
BIN URI: BOLD:AAU4506

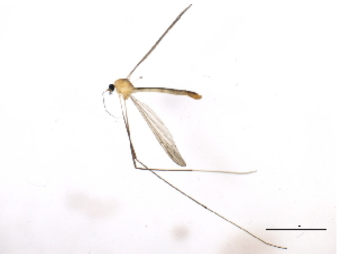

BIOUG00867-E12 [Lateral]
Limoniidae
Family: Limoniidae
BIN URI: BOLD:AAN5881

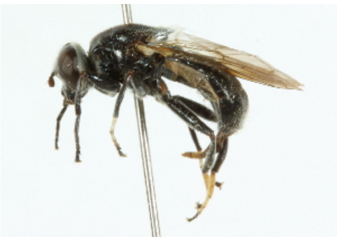

CNC DIPTERA 43733 [Lateral]
Myolepta nigra
Family: Syrphidae
BIN URI: BOLD:AAV0836

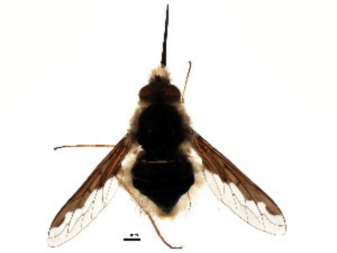

BIOUG00992-C02 [Dorsal]
Bombyliidae
Family: Bombyliidae
BIN URI: BOLD:ABV0388

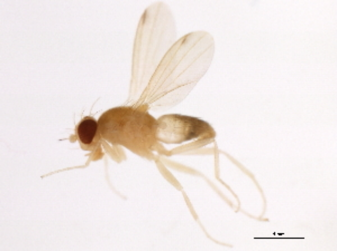

08TTML-1807 [Lateral]
Clusiodes albimanus
Family: Clusiidae
BIN URI: BOLD:AAN5648

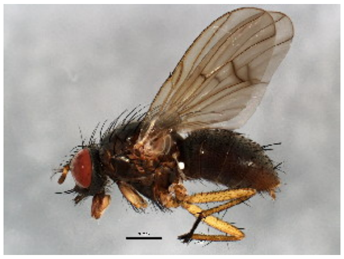

BIOUG01402-A09 [Lateral]
Phaonia
Family: Muscidae
BIN URI: BOLD:ACU3950

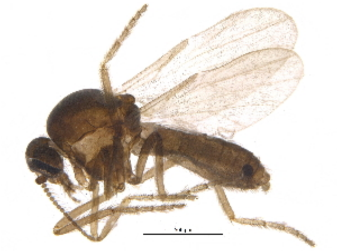

BIOUG20105-E09 [Lateral]
Diptera

BIN URI: BOLD:ACW3173

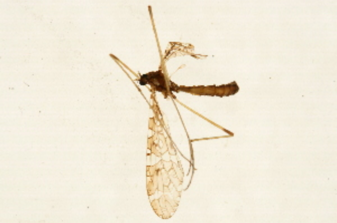

08BBTIP-059 [Lateral]
Epiphragma
Family: Limoniidae
BIN URI: BOLD:ACL8650

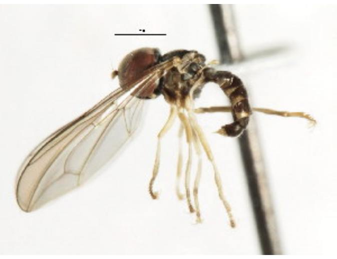

JSS 15760 [Lateral]
Cephalops pallidivittipes
Family: Pipunculidae
BIN URI: BOLD:AAG1658

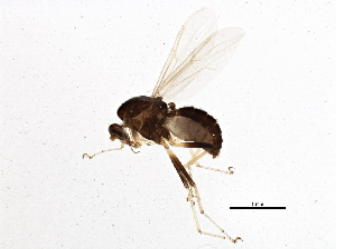

BIOUG04678-E09 [Lateral]
Stilobezzia
Family: Ceratopogonidae
BIN URI: BOLD:ACA7683

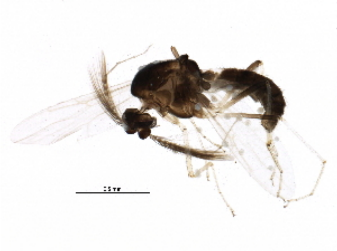

BIOUG21891-E12 [Lateral]
Stilobezzia antennalis
Family: Ceratopogonidae
BIN URI: BOLD:ACV4869

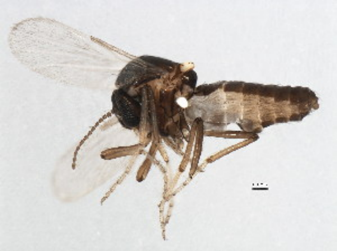

BIOUG01460-F02 [Lateral]
Ceratopogonidae
Family: Ceratopogonidae
BIN URI: BOLD:AAG6475

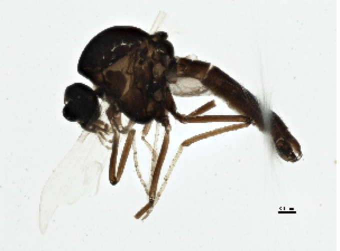

BIOUG05788-D06 [Lateral]
Ceratopogonidae
Family: Ceratopogonidae
BIN URI: BOLD:ACG8210

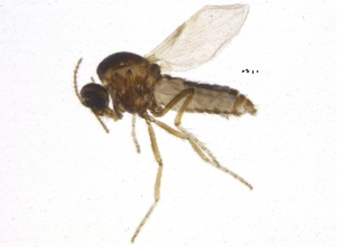

BIOUG03345-F12 [Lateral]
Dasyhelea
Family: Ceratopogonidae
BIN URI: BOLD:AAV5177

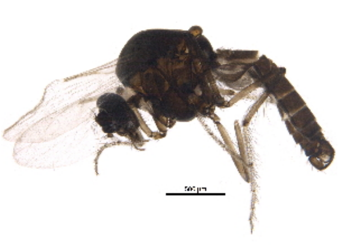

BIOUG22328-A10 [Lateral]

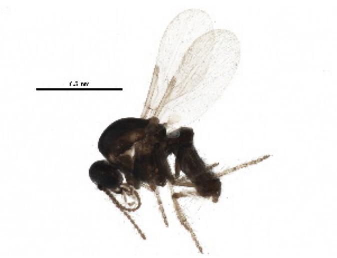

BIOUG22467-B06 [Lateral]

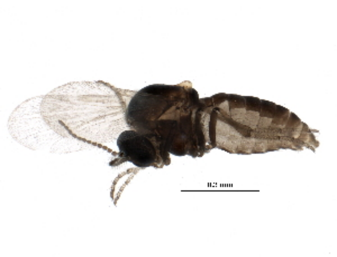

BIOUG22084-G03 [Lateral]

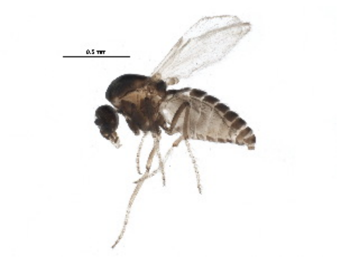

BIOUG22291-C09 [Lateral]

Ceratopogonidae
Family: Ceratopogonidae
BIN URI: BOLD:AAO7716

Ceratopogonidae
Family: Ceratopogonidae
BIN URI: BOLD:ACV3172

Ceratopogonidae
Family: Ceratopogonidae
BIN URI: BOLD:ACV3306

Dasyhelea
Family: Ceratopogonidae
BIN URI: BOLD:AAN5161

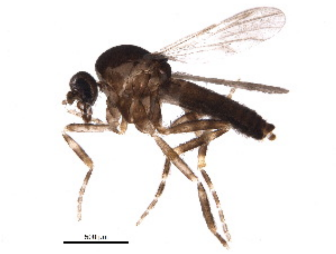

BIOUG22454-D08 [Lateral]
Ceratopogonidae
Family: Ceratopogonidae
BIN URI: BOLD:ACV4324

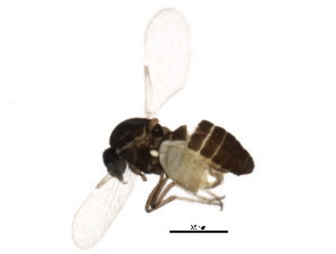

BIOUG01345-E02 [Lateral]
Dasyhelea
Family: Ceratopogonidae
BIN URI: BOLD:AAU6592

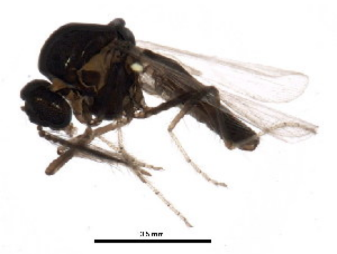

BIOUG01348-H05 [Lateral]
Dasyhelea
Family: Ceratopogonidae
BIN URI: BOLD:AAN5154

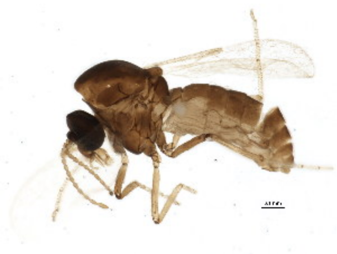

BIOUG01611-E08 [Lateral]
Dasyhelea
Family: Ceratopogonidae
BIN URI: BOLD:ABV1354

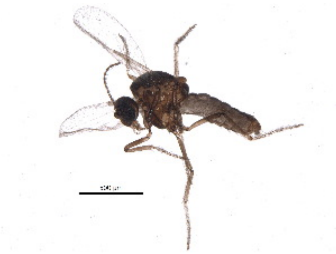

BIOUG22454-C11 [Lateral]
Ceratopogonidae
Family: Ceratopogonidae
BIN URI: BOLD:ACV4557

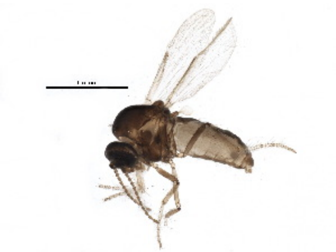

BIOUG21794-D04 [Lateral]
Diptera

BIN URI: BOLD:ACV3366

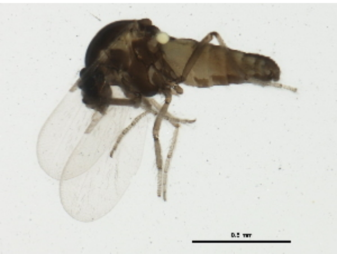

BIOUG01410-F08 [Lateral]
Ceratopogonidae
Family: Ceratopogonidae
BIN URI: BOLD:AAP6703

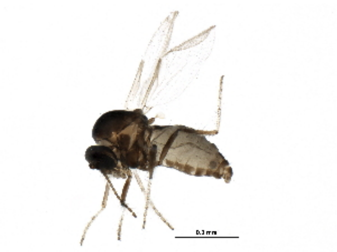

BIOUG22365-B12 [Lateral]
Ceratopogonidae
Family: Ceratopogonidae
BIN URI: BOLD:AAQ2556

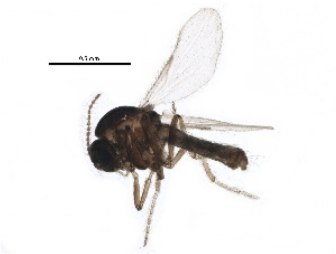

BIOUG22733-B10 [Lateral]
Ceratopogonidae
Family: Ceratopogonidae
BIN URI: BOLD:ACV4888

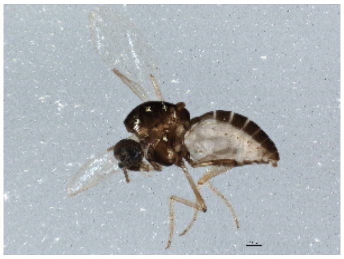

BIOUG06085-D02 [Lateral]
Dasyhelea
Family: Ceratopogonidae
BIN URI: BOLD:ACG3349

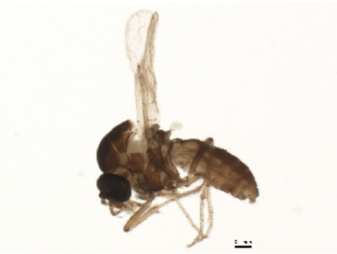

BIOUG01132-A11 [Lateral]
Dasyhelea
Family: Ceratopogonidae
BIN URI: BOLD:ACV5193

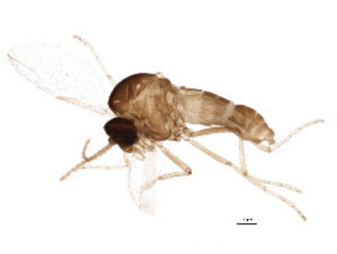

BIOUG01611-A09 [Lateral]
Ceratopogonidae
Family: Ceratopogonidae
BIN URI: BOLD:ABV1356

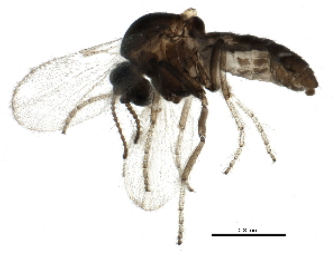

BIOUG22359-D08 [Lateral]
Dasyhelea
Family: Ceratopogonidae
BIN URI: BOLD:AAV5098

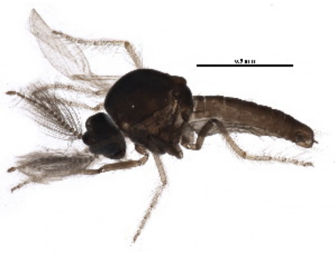

BIOUG22716-D09 [Lateral]
Ceratopogonidae
Family: Ceratopogonidae
BIN URI: BOLD:ACV5326

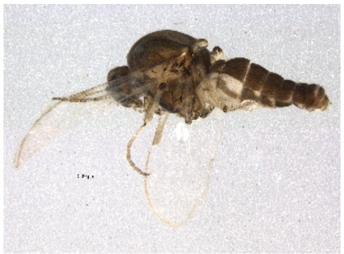

BIOUG03164-H11 [Lateral]
Dasyhelea
Family: Ceratopogonidae
BIN URI: BOLD:AAN5172

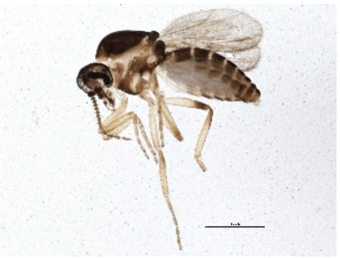

BIOUG05000-G11 [Lateral]
Ceratopogonidae
Family: Ceratopogonidae
BIN URI: BOLD:ACD9562

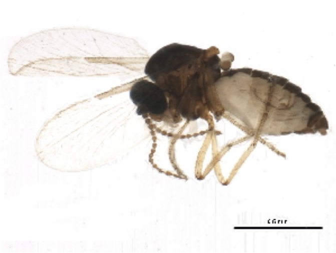

BIOUG01130-A10 [Lateral]
Dasyhelea
Family: Ceratopogonidae
BIN URI: BOLD:AAU6535

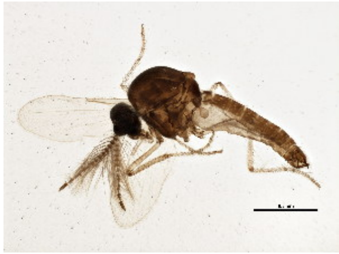

BIOUG02755-F11 [Lateral]
Dasyhelea
Family: Ceratopogonidae
BIN URI: BOLD:ACA7494

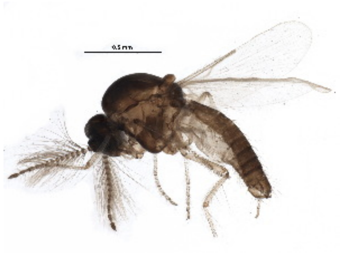

BIOUG22417-H06 [Lateral]
Dasyhelea
Family: Ceratopogonidae
BIN URI: BOLD:AAY6464

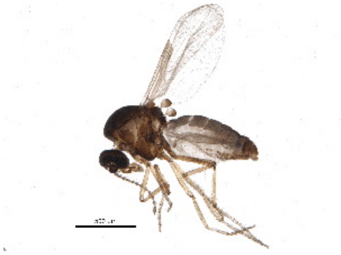

BIOUG10967-H06 [Lateral]
Ceratopogonidae
Family: Ceratopogonidae
BIN URI: BOLD:ABX7385

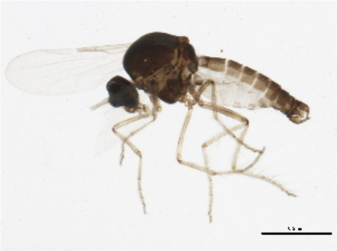

10PHMAL-3455 [Lateral]
Dasyhelea
Family: Ceratopogonidae
BIN URI: BOLD:AAU6477

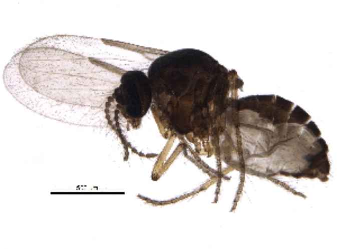

BIOUG08653-G06 [Lateral]
Ceratopogonidae
Family: Ceratopogonidae
BIN URI: BOLD:ACK2715

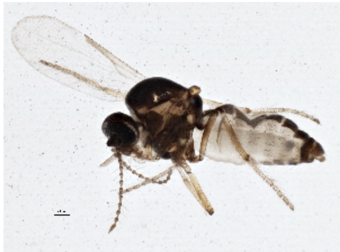

BIOUG03028-A07 [Lateral]
Ceratopogonidae
Family: Ceratopogonidae
BIN URI: BOLD:AAN5151

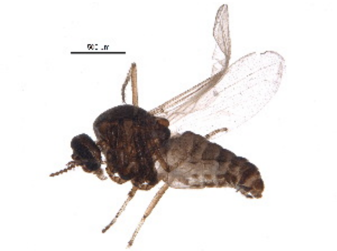

BIOUG20566-E01 [Lateral]
Ceratopogonidae
Family: Ceratopogonidae
BIN URI: BOLD:ACV5946

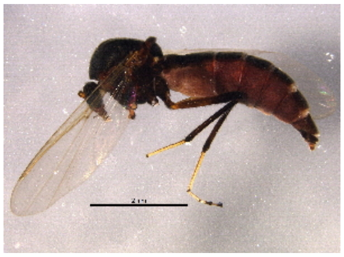

BIOUG10174-B02 [Lateral]
Ceratopogonidae
Family: Ceratopogonidae
BIN URI: BOLD:ACL5068

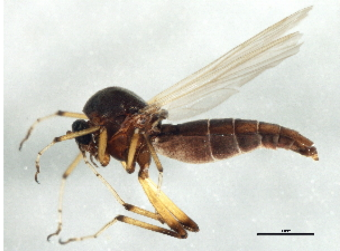

BIOUG01544-E08 [Lateral]
Ceratopogonidae
Family: Ceratopogonidae
BIN URI: BOLD:ACV4442

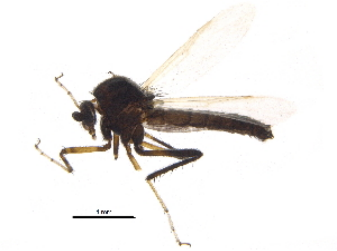

BIOUG22573-D03 [Lateral]
Ceratopogoninae
Family: Ceratopogonidae
BIN URI: BOLD:AAV5157

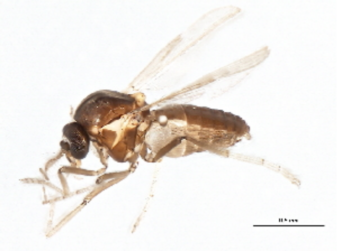

08TTML-2482 [Lateral]
Forcipomyia
Family: Ceratopogonidae
BIN URI: BOLD:AAN5149

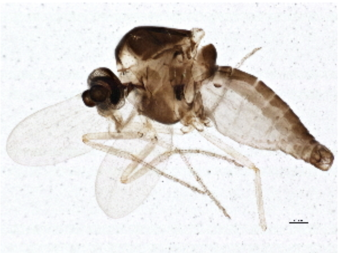

BIOUG02892-E06 [Lateral]
Ceratopogonidae
Family: Ceratopogonidae
BIN URI: BOLD:ACB5406

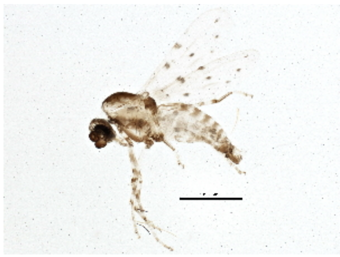

BIOUG06082-E09 [Lateral]
Alluaudomyia
Family: Ceratopogonidae
BIN URI: BOLD:ACG4727

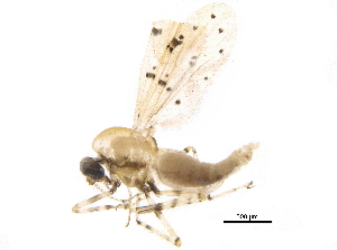

BIOUG22718-A01 [Lateral]
Ceratopogoninae
Family: Ceratopogonidae
BIN URI: BOLD:ABW9876

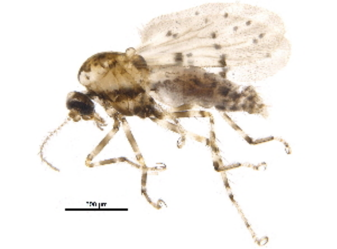

BIOUG22719-D08 [Lateral]
Ceratopogoninae
Family: Ceratopogonidae
BIN URI: BOLD:ACV4258

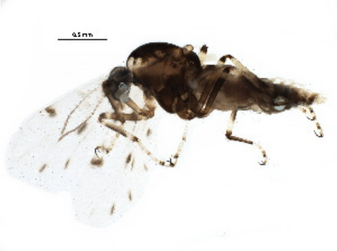

BIOUG22725-E03 [Lateral]
Ceratopogonidae
Family: Ceratopogonidae
BIN URI: BOLD:ACV5978

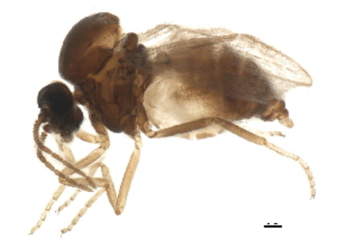

BIOUG01116-H06 [Lateral]
Forcipomyia
Family: Ceratopogonidae
BIN URI: BOLD:AAQ0307

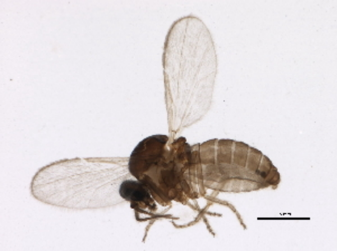

10PHMAL-2926 [Lateral]
Forcipomyia
Family: Ceratopogonidae
BIN URI: BOLD:AAU6519

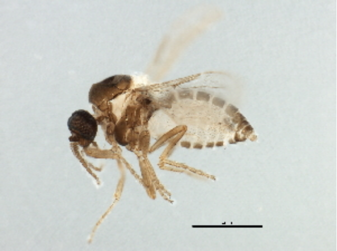

BIOUG01120-A12 [Lateral]
Forcipomyia
Family: Ceratopogonidae
BIN URI: BOLD:AAN5153

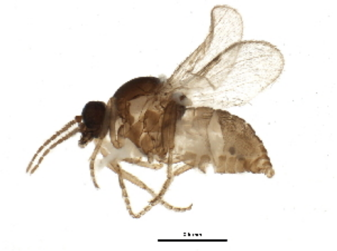

BIOUG01127-E12 [Lateral]
Forcipomyia
Family: Ceratopogonidae
BIN URI: BOLD:ABZ3626

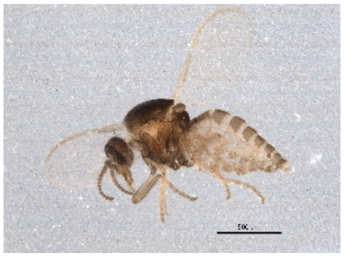

BIOUG05800-A09 [Lateral]
Forcipomyia
Family: Ceratopogonidae
BIN URI: BOLD:AAQ0594

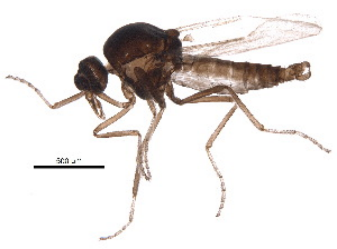

BIOUG22353-B08 [Lateral]
Forcipomyia
Family: Ceratopogonidae
BIN URI: BOLD:AAM6191

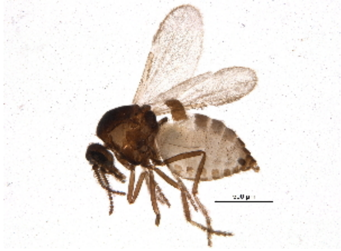

BIOUG06690-A01 [Lateral]
Forcipomyia
Family: Ceratopogonidae
BIN URI: BOLD:AAN5165

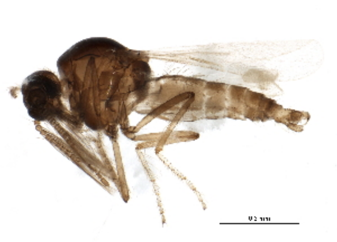

BIOUG08142-C08 [Lateral]
Ceratopogonidae
Family: Ceratopogonidae
BIN URI: BOLD:AAN5155

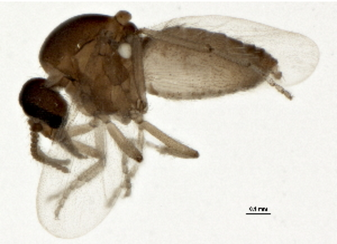

BIOUG02056-F06 [Lateral]
Forcipomyia
Family: Ceratopogonidae
BIN URI: BOLD:AAV5144

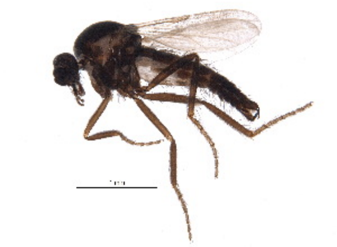

BIOUG22356-G06 [Lateral]
Forcipomyia
Family: Ceratopogonidae
BIN URI: BOLD:AAV5212

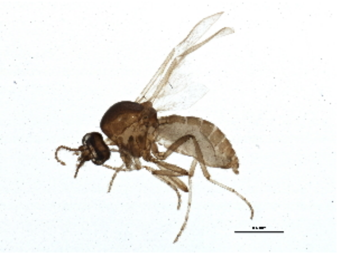

BIOUG05788-D04 [Lateral]
Ceratopogonidae
Family: Ceratopogonidae
BIN URI: BOLD:ACG3798

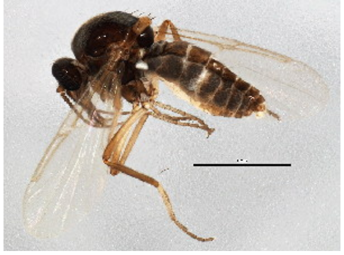

BIOUG01923-A04 [Lateral]
Atrichopogon
Family: Ceratopogonidae
BIN URI: BOLD:AAG6519

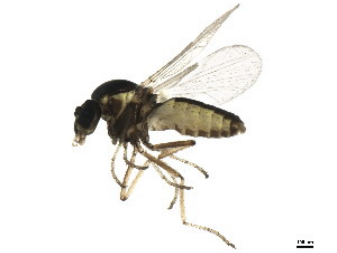

BIOUG01348-E03 [Lateral]
Dasyhelea
Family: Ceratopogonidae
BIN URI: BOLD:AAN5162

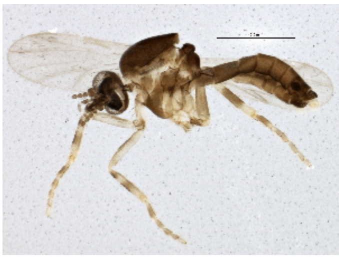

BIOUG04698-D04 [Lateral]
Forcipomyia
Family: Ceratopogonidae
BIN URI: BOLD:ACC4125

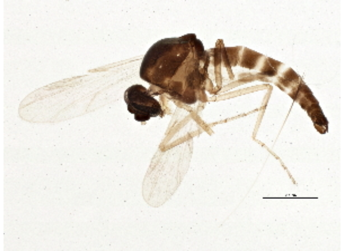

BIOUG02662-E11 [Lateral]
Ceratopogonidae
Family: Ceratopogonidae
BIN URI: BOLD:ACQ9076

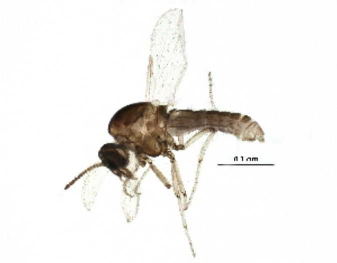

BIOUG05781-A04 [Lateral]
Dasyhelea
Family: Ceratopogonidae
BIN URI: BOLD:ACN5021

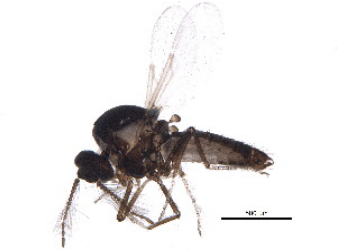

BIOUG22454-B06 [Lateral]
Ceratopogonidae
Family: Ceratopogonidae
BIN URI: BOLD:ACV4507

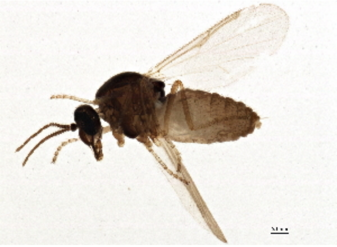

BIOUG03907-F06 [Lateral]
Atrichopogon
Family: Ceratopogonidae
BIN URI: BOLD:AAG6431

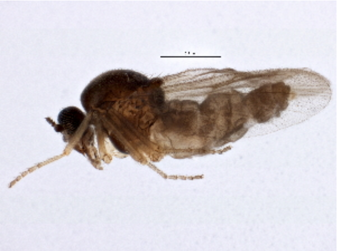

BIOUG02759-C01 [Lateral]
Atrichopogon
Family: Ceratopogonidae
BIN URI: BOLD:AAG3631

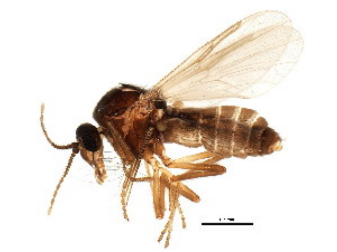

BIOUG01915-H03 [Lateral]
Atrichopogon
Family: Ceratopogonidae
BIN URI: BOLD:AAG6494

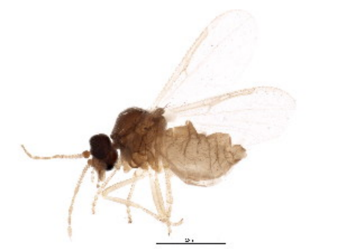

BIOUG01695-E08 [Lateral]
Atrichopogon
Family: Ceratopogonidae
BIN URI: BOLD:ABA0806

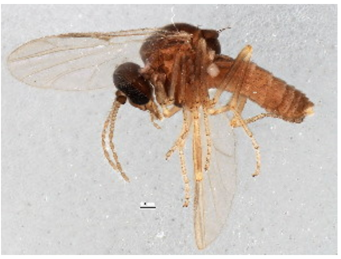

BIOUG01929-D04 [Lateral]
Atrichopogon
Family: Ceratopogonidae
BIN URI: BOLD:AAG6452

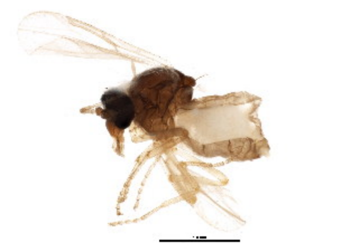

BIOUG01885-A07 [Lateral]
Atrichopogon
Family: Ceratopogonidae
BIN URI: BOLD:ABV9306

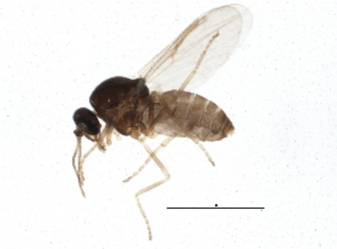

BIOUG02976-C11 [Lateral]
Atrichopogon
Family: Ceratopogonidae
BIN URI: BOLD:ACA3626

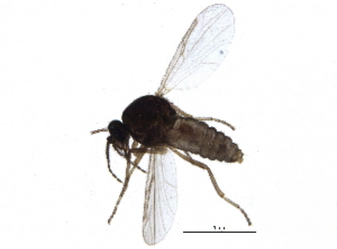

BIOUG10373-D01 [Lateral]
Atrichopogon
Family: Ceratopogonidae
BIN URI: BOLD:ACL4764

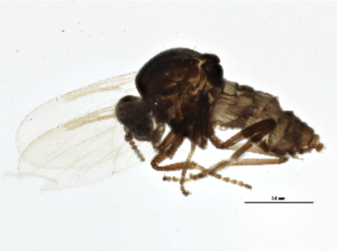

BIOUG05788-E02 [Lateral]
Atrichopogon
Family: Ceratopogonidae
BIN URI: BOLD:AAP6915

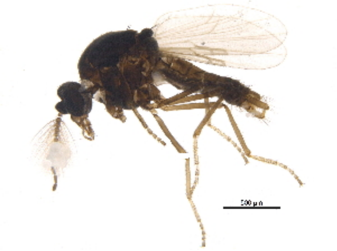

BIOUG22457-F08 [Lateral]
Atrichopogon
Family: Ceratopogonidae
BIN URI: BOLD:ACV5558

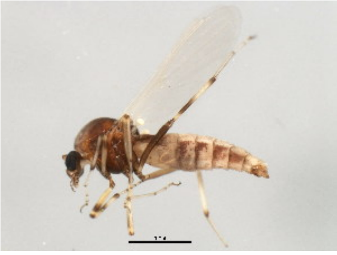

BIOUG01543-F10 [Lateral]
Ceratopogonidae
Family: Ceratopogonidae
BIN URI: BOLD:ABW1518

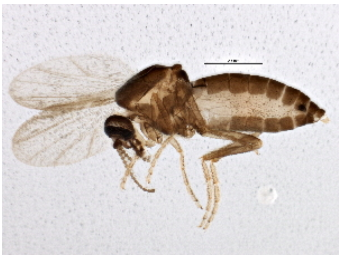

BIOUG03287-G01 [Lateral]
Forcipomyia
Family: Ceratopogonidae
BIN URI: BOLD:ACA7885

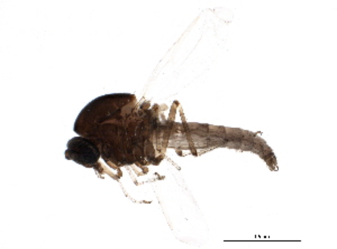

BIOUG22459-F04 [Lateral]
Ceratopogonidae
Family: Ceratopogonidae
BIN URI: BOLD:AAN5158

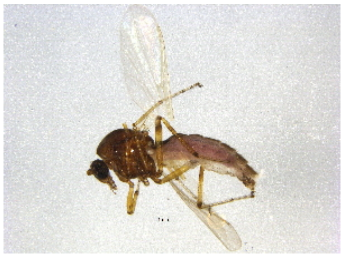

BIOUG03899-F12 [Lateral]
Ceratopogonidae
Family: Ceratopogonidae
BIN URI: BOLD:ACR1960

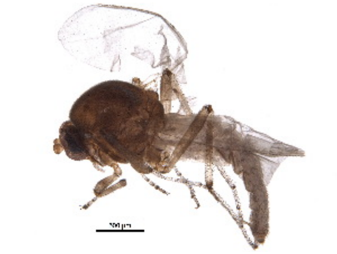

BIOUG21890-F05 [Lateral]
Bezzia
Family: Ceratopogonidae
BIN URI: BOLD:ACV5708

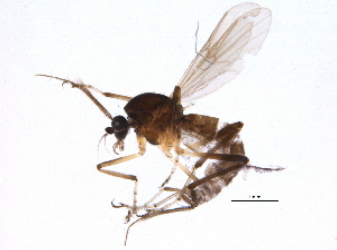

BIOUG04067-H11 [Lateral]
Clinohelea
Family: Ceratopogonidae
BIN URI: BOLD:AAG6451

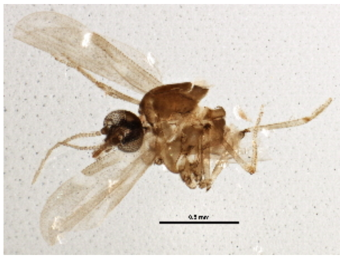

BIOUG02849-G12 [Lateral]
Culicoides
Family: Ceratopogonidae
BIN URI: BOLD:AAM6184

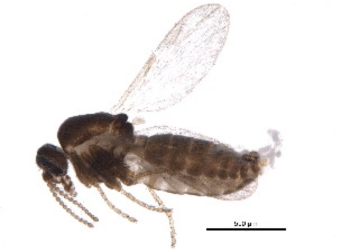

BIOUG22454-B02 [Lateral]
Ceratopogonidae
Family: Ceratopogonidae
BIN URI: BOLD:ACA4406

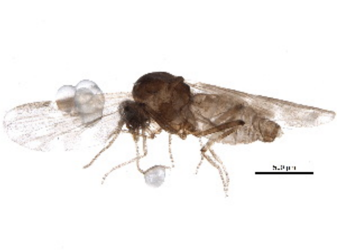

BIOUG11859-B09 [Lateral]
Culicoides
Family: Ceratopogonidae
BIN URI: BOLD:AAN5166

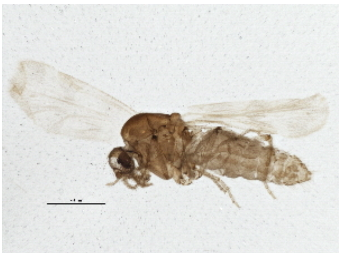

BIOUG03327-H11 [Lateral]
Culicoides
Family: Ceratopogonidae
BIN URI: BOLD:ACC3892

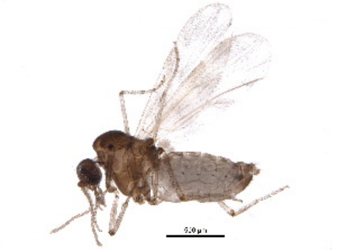

BIOUG22454-B11 [Lateral]
Culicoides
Family: Ceratopogonidae
BIN URI: BOLD:ACV4326

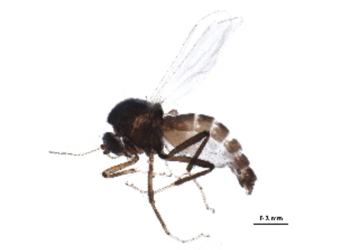

BIOUG20729-B09 [Lateral]
Diptera

BIN URI: BOLD:ACU4023

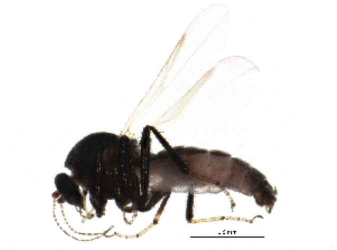

BIOUG24002-A05 [Lateral]
Bezzia
Family: Ceratopogonidae

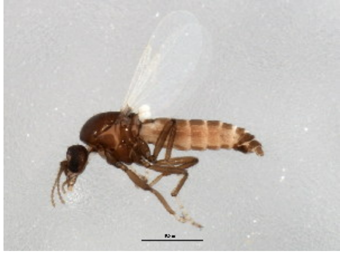

BIOUG01924-F07 [Lateral]
Brachypogon
Family: Ceratopogonidae
BIN URI: BOLD:AAL7405

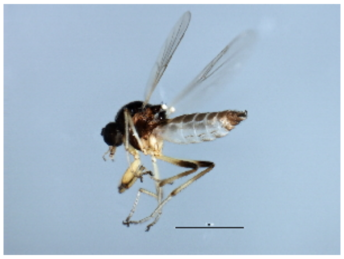

09BBEDI-2381 [Lateral]
Palpomyia
Family: Ceratopogonidae
BIN URI: BOLD:AAM6196

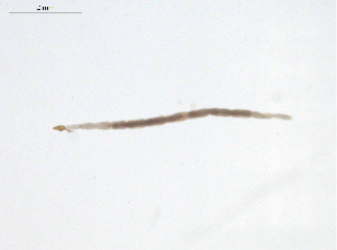

BIOUG02700-A12 [Lateral]
Ceratopogonidae
Family: Ceratopogonidae
BIN URI: BOLD:ACH0425

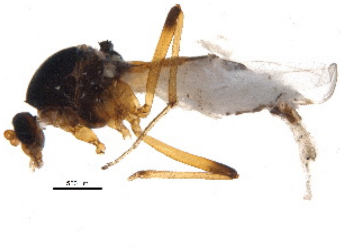

BIOUG24024-D02 [Lateral]
Palpomyia
Family: Ceratopogonidae

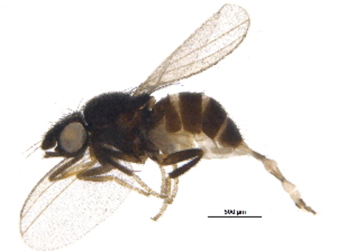

BIOUG23076-A02 [Lateral]
Paramyia nitens
Family: Milichiidae
BIN URI: BOLD:AAG0166

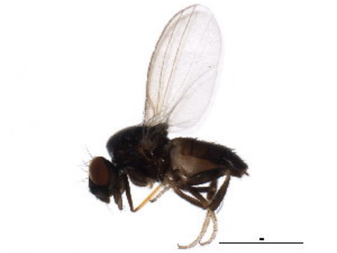

BIOUG01360-C05 [Lateral]
Paramyia nitens
Family: Milichiidae
BIN URI: BOLD:AAG0169

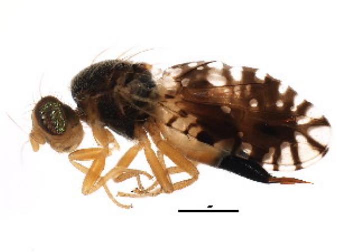

BIOUG01408-B03 [Lateral]
Tephritidae
Family: Tephritidae
BIN URI: BOLD:AAC6384

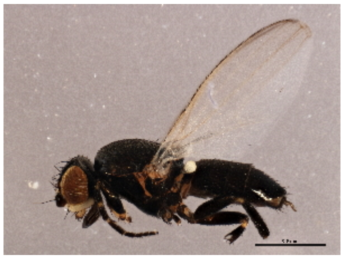

BIOUG02713-C10 [Lateral]
Leptometopa latipes
Family: Milichiidae
BIN URI: BOLD:AAP8985

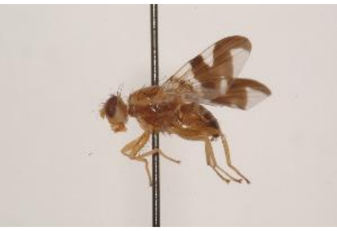

TEPH165 [Lateral]
Rhagoletis suavis
Family: Tephritidae
BIN URI: BOLD:AAY0136

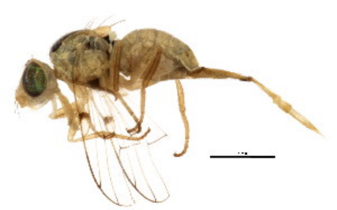

BIOUG01244-H02 [Lateral]
Tephritidae
Family: Tephritidae
BIN URI: BOLD:ABX6599

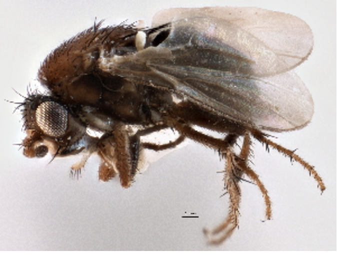

CCDB-21403-A01 [Lateral]
Coproica ferruginata
Family: Sphaeroceridae
BIN URI: BOLD:AAN6407

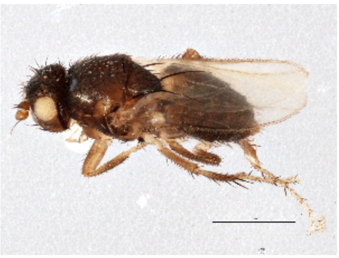

BIOUG01677-H01 [Lateral]
Coproica hirtula
Family: Sphaeroceridae
BIN URI: BOLD:ACF7714

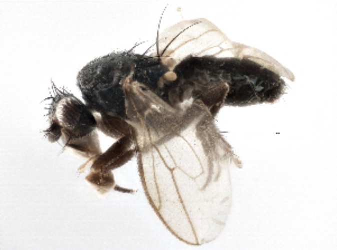

CCDB-21402-D03 [Lateral]
Spelobia clunipes
Family: Sphaeroceridae
BIN URI: BOLD:AAG7312

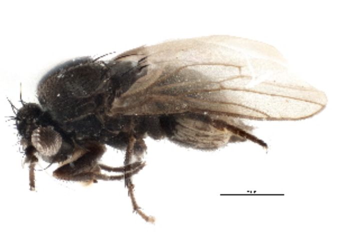

CCDB-21402-C12 [Lateral]
Spelobia clunipes
Family: Sphaeroceridae
BIN URI: BOLD:AAN6405

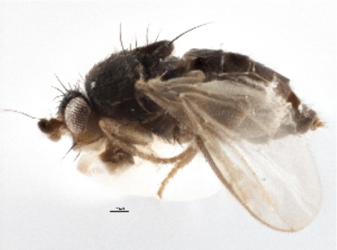

CCDB-21402-C04 [Lateral]
Spelobia bifrons
Family: Sphaeroceridae
BIN URI: BOLD:AAN6408

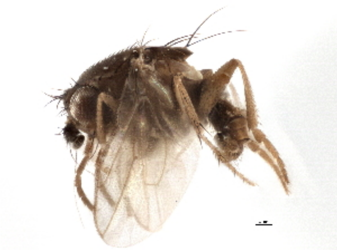

CCDB-21402-F01 [Lateral]
Spelobia semioculata
Family: Sphaeroceridae
BIN URI: BOLD:AAL7752

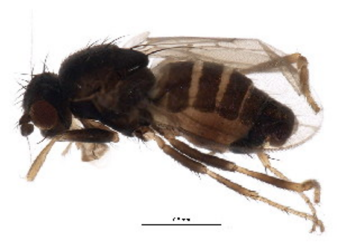

BIOUG01360-C08 [Lateral]
Sphaeroceridae
Family: Sphaeroceridae
BIN URI: BOLD:AAG7308

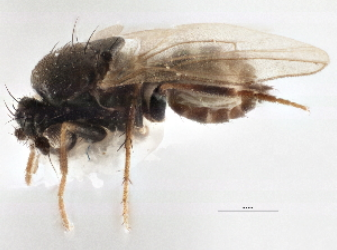

CCDB-21402-E06 [Lateral]
Spelobia quinata
Family: Sphaeroceridae
BIN URI: BOLD:AAN6415

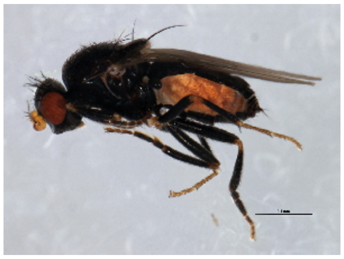

08TTML-1015 [Lateral]
Sphaeroceridae
Family: Sphaeroceridae
BIN URI: BOLD:AAG7309

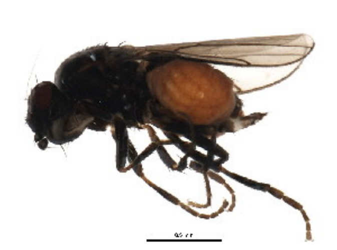

BIOUG01337-E05 [Lateral]
Sphaeroceridae
Family: Sphaeroceridae
BIN URI: BOLD:AAN6409

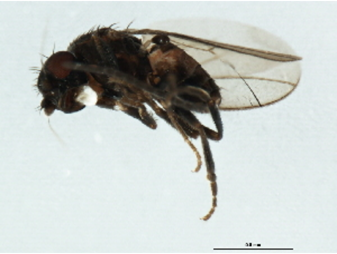

10BBCDIP-3459 [Lateral]
Sphaeroceridae
Family: Sphaeroceridae
BIN URI: BOLD:AAN6414

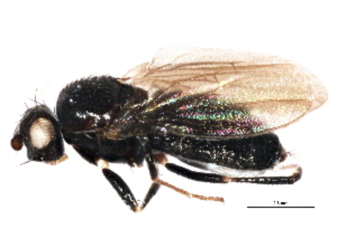

BIOUG02825-D01 [Lateral]
Sphaeroceridae
Family: Sphaeroceridae
BIN URI: BOLD:ACA4498

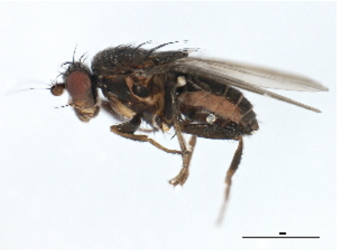

10BBCDIP-1275 [Lateral]
Leptocera erythrocera
Family: Sphaeroceridae
BIN URI: BOLD:AAG7276

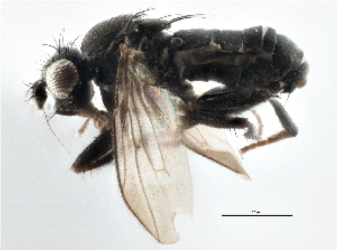

CCDB-21401-H04 [Lateral]
Apteromyia claviventris
Family: Sphaeroceridae
BIN URI: BOLD:AAG7283

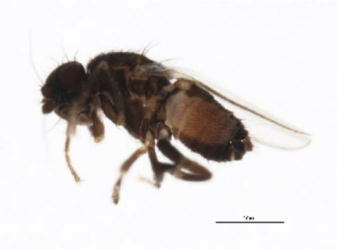

09BBDIP-1144 [Lateral]
Sphaeroceridae
Family: Sphaeroceridae
BIN URI: BOLD:AAG7292

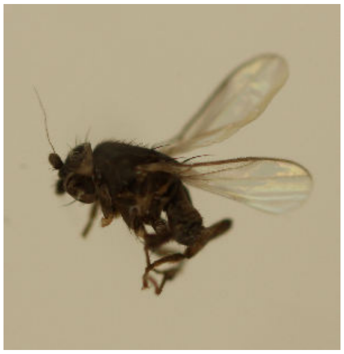

MZH_HP.1104 [Lateral]
Telomerina flavipes
Family: Sphaeroceridae
BIN URI: BOLD:ACJ1971

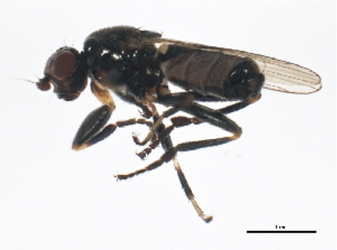

BIOUG00904-G07 [Lateral]
Sphaeroceridae
Family: Sphaeroceridae
BIN URI: BOLD:AAH3548

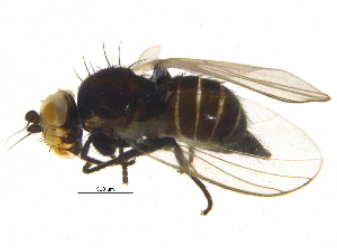

BIOUG03210-F10 [Lateral]
Agromyzidae
Family: Agromyzidae
BIN URI: BOLD:AAN5432

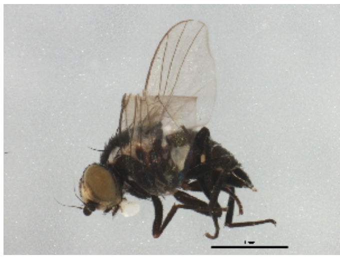

BIOUG01142-C09 [Lateral]
Calycomyza majuscula
Family: Agromyzidae
BIN URI: BOLD:AAV4861

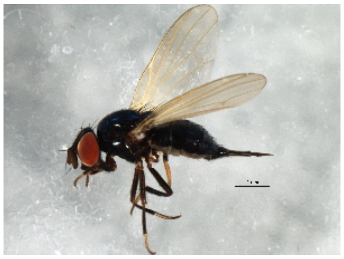

08TTML-1971 [Lateral]
Lonchaeinae
Family: Lonchaeidae
BIN URI: BOLD:AAG7064

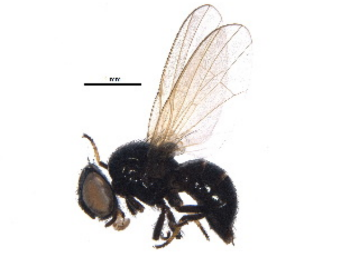

BIOUG22356-B11 [Lateral]
Lonchaeidae
Family: Lonchaeidae
BIN URI: BOLD:AAP2540

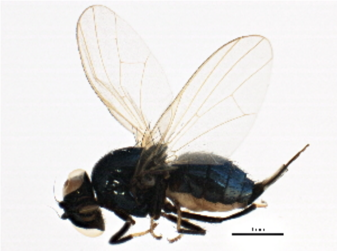

BIOUG05510-H10 [Lateral]
Lonchaea
Family: Lonchaeidae
BIN URI: BOLD:AAG7070

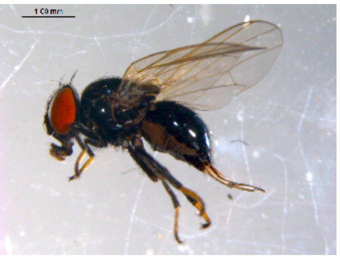

10PROBE-11259 [Lateral]
Lonchaea sp. TAW4
Family: Lonchaeidae
BIN URI: BOLD:AAP8895

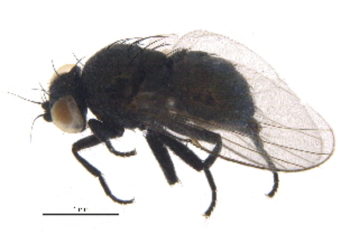

BIOUG22330-B09 [Lateral]
Japanagromyza viridula
Family: Agromyzidae
BIN URI: BOLD:AAI7960

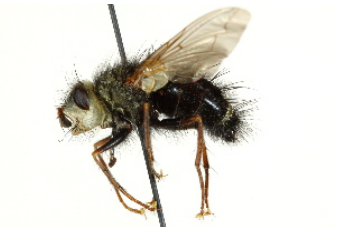

CNC DIPTERA 162362 [Lateral]
Epalpus signifier
Family: Tachinidae
BIN URI: BOLD:ABZ2493

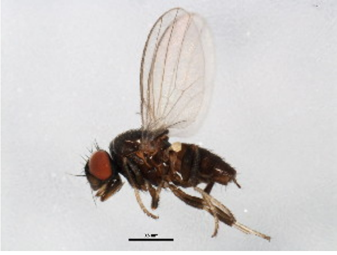

BIOUG01426-F07 [Lateral]
Milichiidae
Family: Milichiidae
BIN URI: BOLD:AAG0172

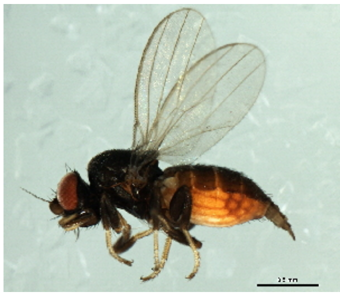

08TTML-1260 [Lateral]
Milichiidae
Family: Milichiidae
BIN URI: BOLD:AAG0174

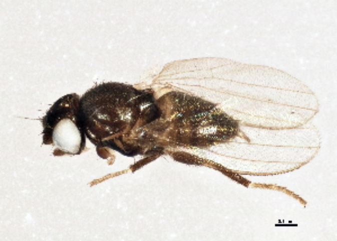

BIOUG03278-D09 [Lateral]
Milichiidae
Family: Milichiidae
BIN URI: BOLD:AAN8612

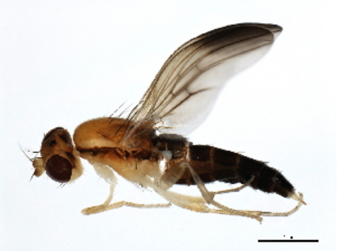

10PHMAL-1243 [Lateral]
Clusiodes melanostomus
Family: Clusiidae
BIN URI: BOLD:AAJ4031

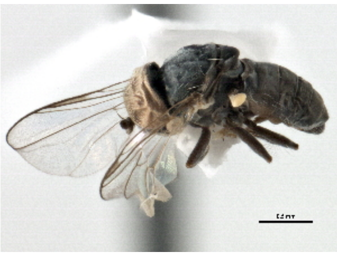

CNC DIPTERA 158661 [Lateral]
Chalarus sp. CA9
Family: Pipunculidae
BIN URI: BOLD:AAI7230

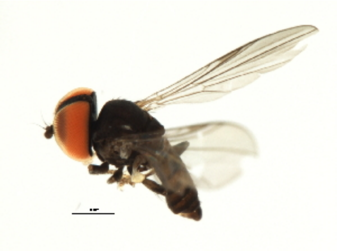

MTDIC-0104 [Lateral]
Chalarus decorus
Family: Pipunculidae
BIN URI: BOLD:AAG1657

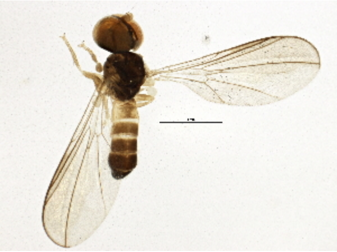

BIOUG03279-G02 [Dorsal]
Chalarus sp. OR1
Family: Pipunculidae
BIN URI: BOLD:ACI4153

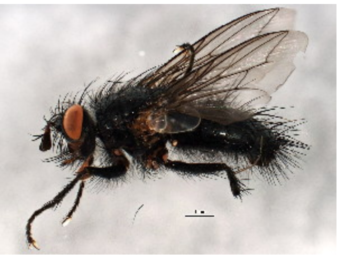

BIOUG01459-A02 [Lateral]
Tachinidae
Family: Tachinidae
BIN URI: BOLD:AAP3780

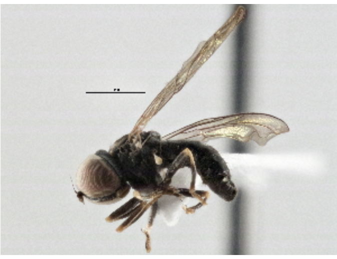

CNC DIPTERA 136934 [Lateral]
Tomosvaryella sp. ON18
Family: Pipunculidae
BIN URI: BOLD:AAG1656

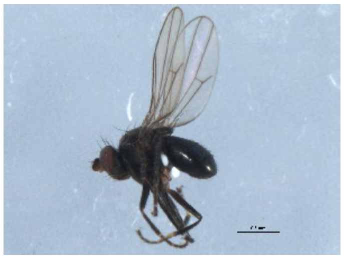

10JSROW-0178 [Lateral]
Hydrellia albilabris
Family: Ephydridae
BIN URI: BOLD:AAP6348

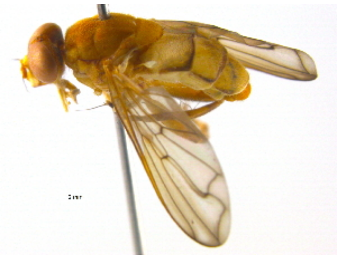

BIOUG04570-D11 [Lateral]
Brachyopa punctipennis
Family: Syrphidae
BIN URI: BOLD:ACE7625

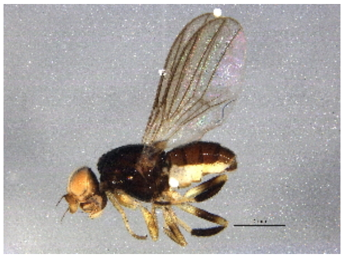

BIOUG05094-D12 [Lateral]
Psilacrum arpidia
Family: Chloropidae
BIN URI: BOLD:ACE0829

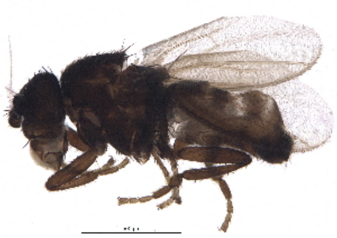

BIOUG08448-E03 [Lateral]
Sphaeroceridae
Family: Sphaeroceridae
BIN URI: BOLD:ACK0161

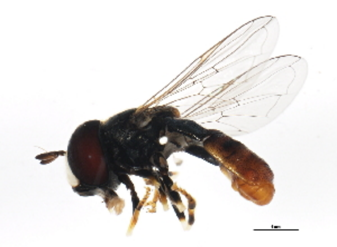

BIOUG00904-C07 [Lateral]
Paragus haemorrhous
Family: Syrphidae
BIN URI: BOLD:AAC2438

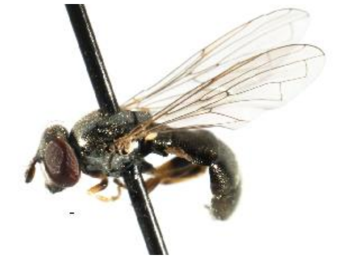

Cp 2007 - 87 [Lateral]
Neoascia distinata
Family: Syrphidae
BIN URI: BOLD:AAG6766

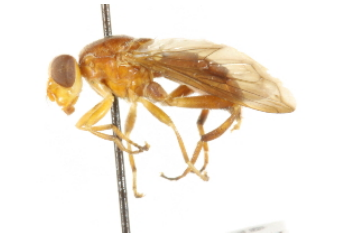

CNC DIPTERA 49268 [Lateral]
Brachyopa sedmani
Family: Syrphidae
BIN URI: BOLD:AAY8902

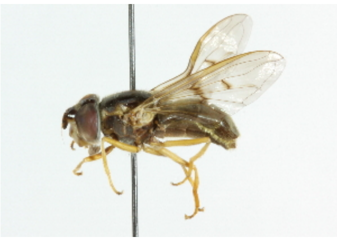

CNC DIPTERA 36580 [Lateral]
Ferdinandea buccata
Family: Syrphidae
BIN URI: BOLD:AAE0948

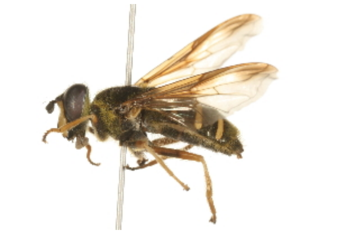

08TTML-0550 [Lateral]
Sericomyia chrysotoxoides
Family: Syrphidae
BIN URI: BOLD:ABX5395

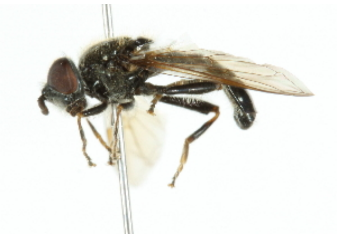

CNC DIPTERA 142 [Lateral]
Pipiza femoralis
Family: Syrphidae
BIN URI: BOLD:AAM7334

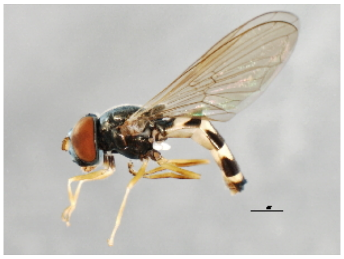

09BBEDI-1095 [Lateral]
Melanostoma
Family: Syrphidae
BIN URI: BOLD:AAB2866

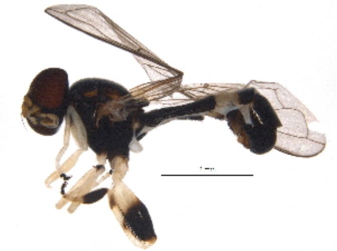

BIOUG09750-G02 [Lateral]
Sphegina keeniana
Family: Syrphidae
BIN URI: BOLD:ACR0385

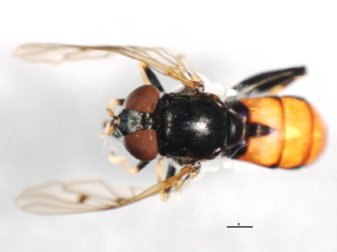

09BBEDI-1211 [Dorsal]
Chalcosyrphus libo
Family: Syrphidae
BIN URI: BOLD:AAG4679

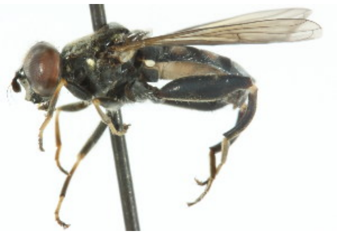

JK00623 [Lateral]
Chalcosyrphus nemorum
Family: Syrphidae
BIN URI: BOLD:AAG6762

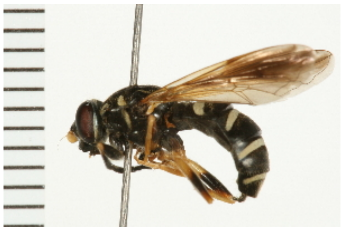

08BBDIP-0095 [Lateral]
Temnostoma balyras
Family: Syrphidae
BIN URI: BOLD:AAD2789

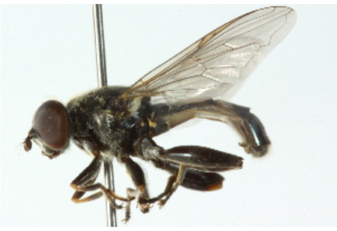

CNC DIPTERA 48709 [Lateral]
Xylota annulifera
Family: Syrphidae
BIN URI: BOLD:AAG0857

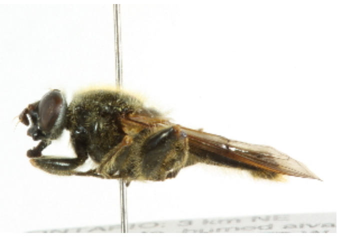

CNC DIPTERA 37752 [Lateral]
Brachypalpus oarus
Family: Syrphidae
BIN URI: BOLD:AAP8757

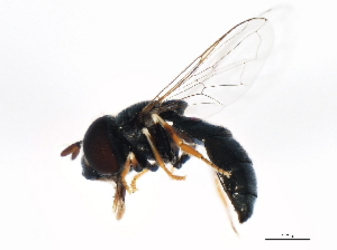

10JSROW-1685 [Lateral]
Paragus haemorrhous
Family: Syrphidae
BIN URI: BOLD:AAC2439

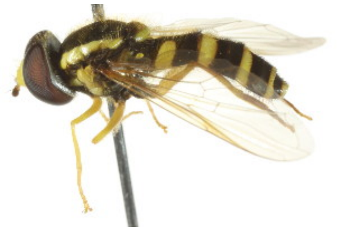

JSS 18632 [Lateral]
Xanthogramma flavipes
Family: Syrphidae
BIN URI: BOLD:AAK0114

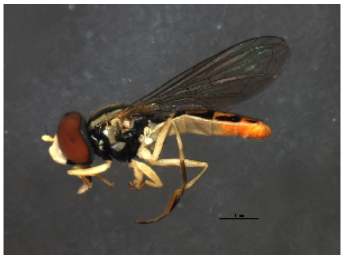

08BBDIP-2174 [Lateral]

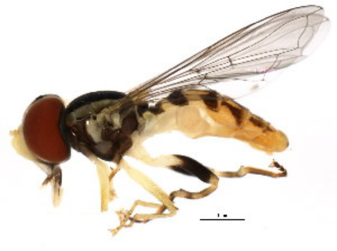

BIOUG01364-H03 [Lateral]

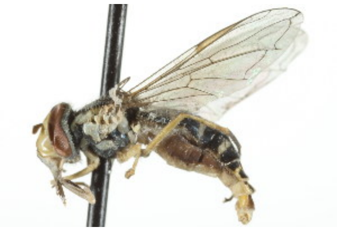

JSYKB-180 [Lateral]
Sphaerophoria
Family: Syrphidae

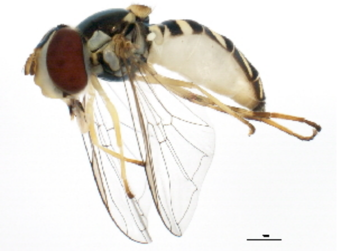

BIOUG01794-C01 [Lateral]

Toxomerus marginatus
Family: Syrphidae
BIN URI: BOLD:AAA4277

Toxomerus geminatus
Family: Syrphidae
BIN URI: BOLD:AAC1312

BIN URI: BOLD:AAA7374

Allograpta obliqua
Family: Syrphidae
BIN URI: BOLD:AAD8276

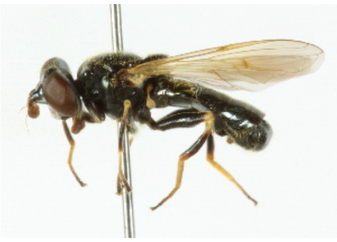

CNC DIPTERA 45250 [Lateral]
Lejota aerea
Family: Syrphidae
BIN URI: BOLD:AAY9807

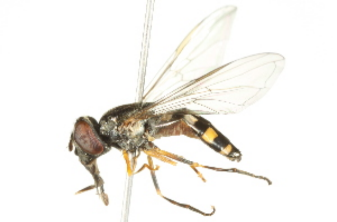

10BBCDIP-2680 [Lateral]
Platycheirus peltatoides
Family: Syrphidae
BIN URI: BOLD:AAC6630

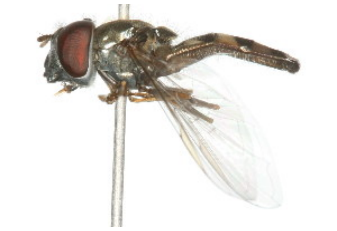

09BBDIP-0286 [Lateral]
Platycheirus obscurus
Family: Syrphidae
BIN URI: BOLD:AAF1237

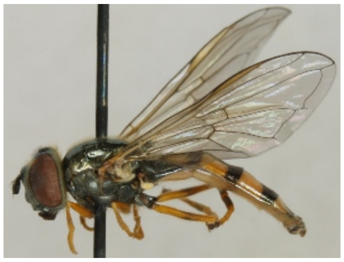

jka09-05324 [Lateral]
Platycheirus immarginatus
Family: Syrphidae
BIN URI: BOLD:AAA9506

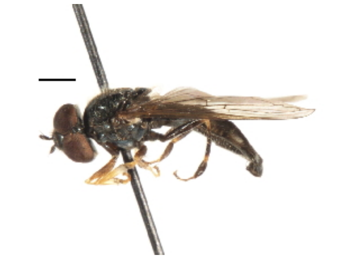

CNC DIPTERA 26022 [Lateral]
Platycheirus hyperboreus
Family: Syrphidae
BIN URI: BOLD:ACF4734

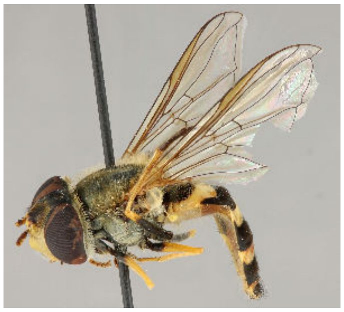

MZH_HP.15 [Lateral]
Syrphus vitripennis
Family: Syrphidae
BIN URI: BOLD:AAB5577

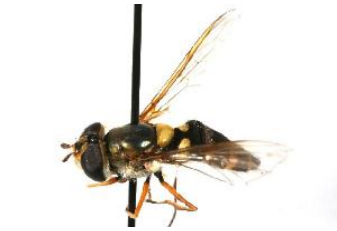

CHU06-SYR-004 [Lateral]
Syrphus ribesii
Family: Syrphidae
BIN URI: BOLD:AAA4570

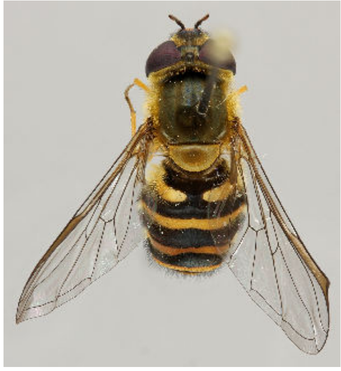

MZH_HP.25 [Dorsal]
Syrphus torvus
Family: Syrphidae
BIN URI: BOLD:AAC6088

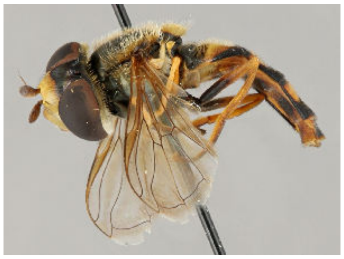

MZH_HP.35 [Lateral]
Eupeodes latifasciatus
Family: Syrphidae
BIN URI: BOLD:AAB2384

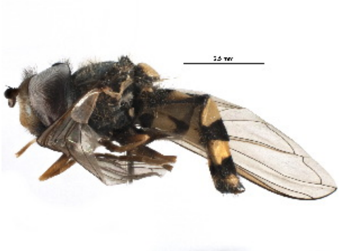

BIOUG22360-C01 [Lateral]
Syrphinae
Family: Syrphidae
BIN URI: BOLD:ACU2992

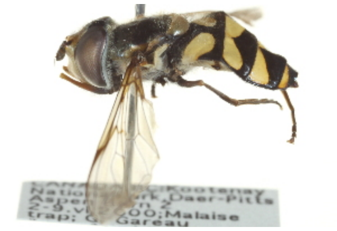

CNC DIPTERA 135215 [Lateral]
Didea fuscipes
Family: Syrphidae
BIN URI: BOLD:AAI9913

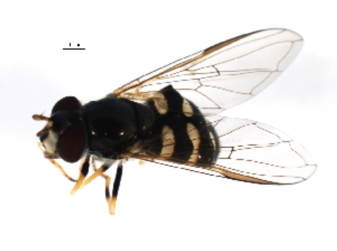

10JSROW-0562 [Dorsal]
Dasysyrphus venustus
Family: Syrphidae
BIN URI: BOLD:ACV5348

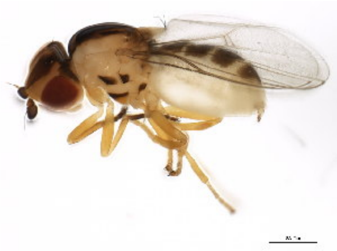

BIOUG01410-C11 [Lateral]
Chloropidae
Family: Chloropidae
BIN URI: BOLD:AAH4135

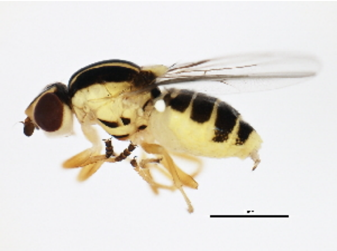

BIOUG00915-C02 [Lateral]
Thaumatomyia glabra
Family: Chloropidae
BIN URI: BOLD:ABY9688

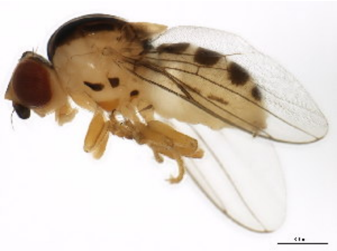

BIOUG01410-C09 [Lateral]
Chloropidae
Family: Chloropidae
BIN URI: BOLD:ABY9689

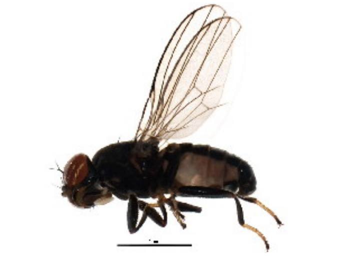

BIOUG01352-A05 [Lateral]
Aulacigaster neoleucopeza
Family: Aulacigastridae
BIN URI: BOLD:AAV0437

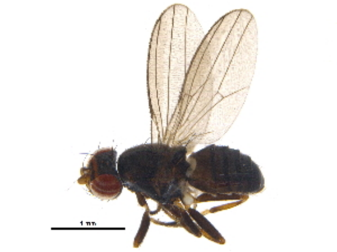

BIOUG21482-E08 [Lateral]

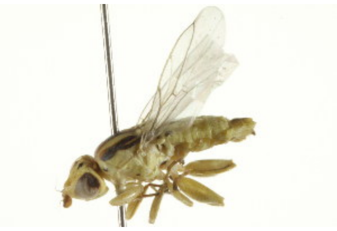

PCPP10-0159 [Lateral]
Meromyza

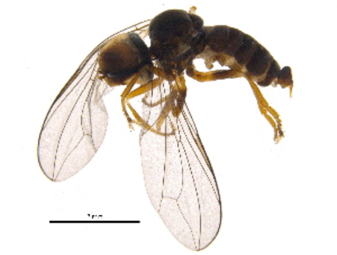

BIOUG22329-E12 [Lateral]

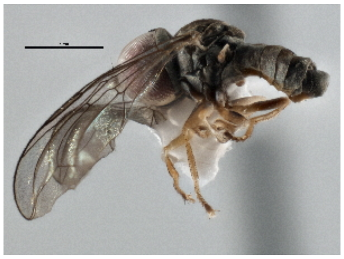

CNC DIPTERA 137523 [Lateral]

Aulacigaster neoleucopeza
Family: Aulacigastridae
BIN URI: BOLD:ABV3853

Family: Chloropidae
BIN URI: BOLD:AAH4205

Eudorylas
Family: Pipunculidae
BIN URI: BOLD:AAI5103

Eudorylas sp. ON20
Family: Pipunculidae
BIN URI: BOLD:AAJ0212

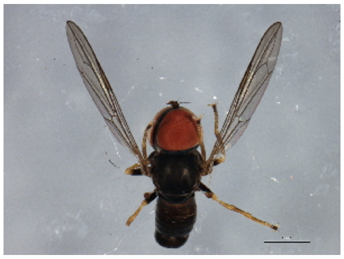

08TTML-1135 [Dorsal]
Eudorylas sp. ON7
Family: Pipunculidae
BIN URI: BOLD:AAM9392

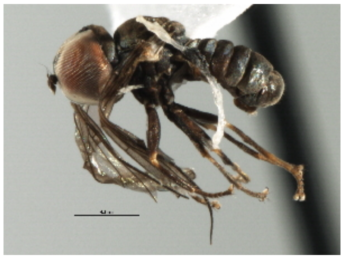

CNC DIPTERA 135982 [Lateral]
Eudorylas sp. YT3
Family: Pipunculidae
BIN URI: BOLD:AAJ0213

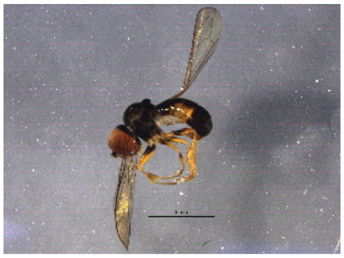

BIOUG06627-C04 [Lateral]
Dorylomorpha hardyi
Family: Pipunculidae
BIN URI: BOLD:ABY5401

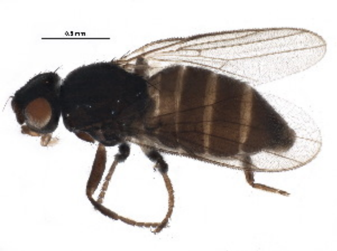

BIOUG22456-E01 [Lateral]
Chloropidae
Family: Chloropidae
BIN URI: BOLD:ACV5279

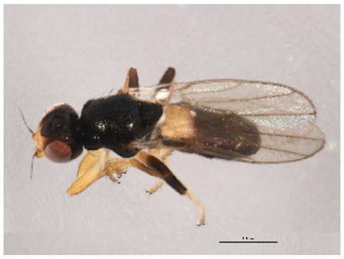

10JSROW-0652 [Dorsal]
Chloropidae
Family: Chloropidae
BIN URI: BOLD:ACE3223

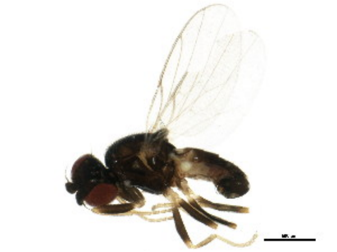

BIOUG01348-B11 [Lateral]
Gaurax pallidipes
Family: Chloropidae
BIN URI: BOLD:AAH4210

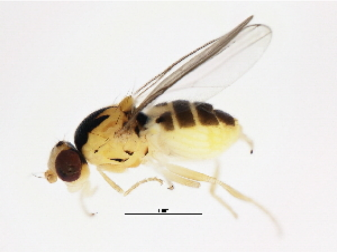

10JSROW-1531 [Lateral]
Gaurax pallidipes
Family: Chloropidae
BIN URI: BOLD:AAV6132

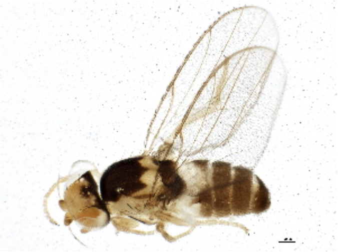

BIOUG03669-F11 [Lateral]
Gaurax dubius
Family: Chloropidae
BIN URI: BOLD:ACC7744

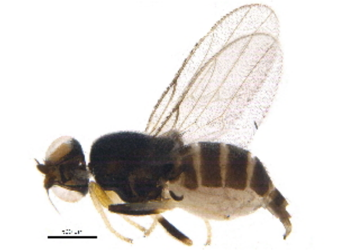

BIOUG22717-E09 [Lateral]
Gaurax
Family: Chloropidae
BIN URI: BOLD:ACV4074

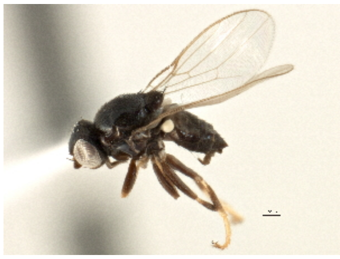

CCDB-21328-F04 [Lateral]
Hapleginella conicola
Family: Chloropidae
BIN URI: BOLD:ABA7838

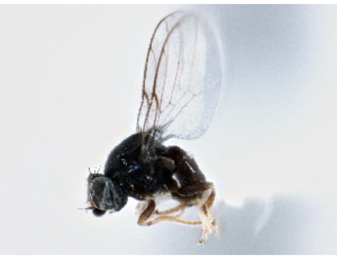

CCDB-21328-F01 [Lateral]
Gaurax varihalteratus
Family: Chloropidae
BIN URI: BOLD:ACM2340

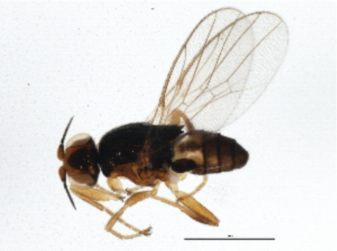

BIOUG05524-A08 [Lateral]
Elachiptera sibirica
Family: Chloropidae
BIN URI: BOLD:AAH4208

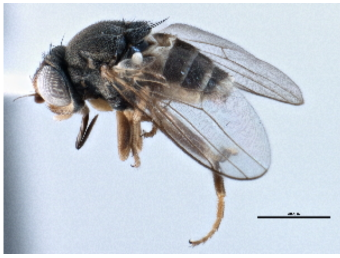

CCDB-21328-G10 [Lateral]
Olcella provocans
Family: Chloropidae
BIN URI: BOLD:ABV1245

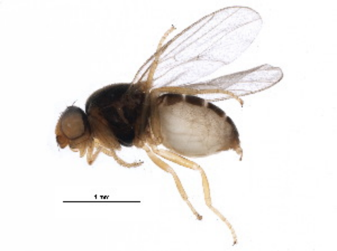

BIOUG22452-H05 [Lateral]
Tricimba
Family: Chloropidae
BIN URI: BOLD:AAN5663

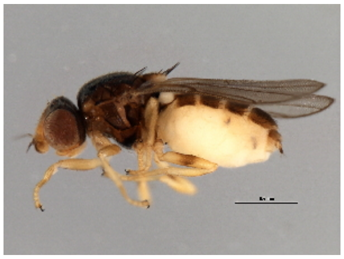

08TTML-2064 [Lateral]
Chloropidae
Family: Chloropidae
BIN URI: BOLD:AAN5667

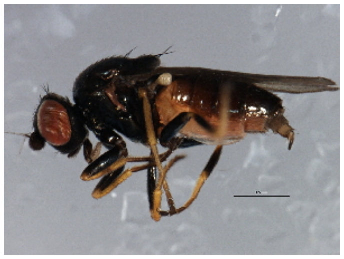

08TTML-1229 [Lateral]
Chloropidae
Family: Chloropidae
BIN URI: BOLD:AAN5659

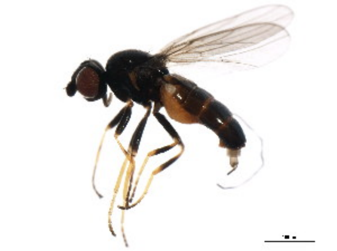

BIOUG01348-G06 [Lateral]
Oscinella sp. TAW4
Family: Chloropidae
BIN URI: BOLD:AAN5665

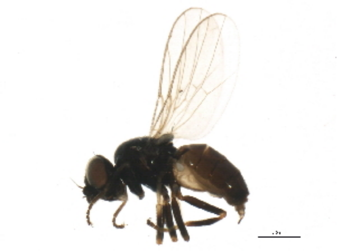

BIOUG00899-C02 [Lateral]
Oscinella sp. TAW6
Family: Chloropidae
BIN URI: BOLD:AAV6112

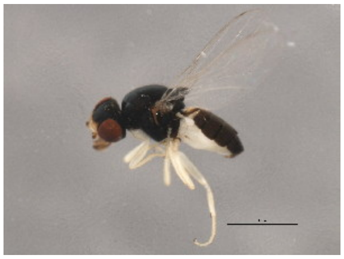

PCPP10-0953 [Lateral]
Chloropidae
Family: Chloropidae
BIN URI: BOLD:AAV6114

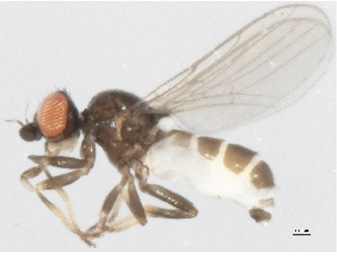

10BBDIP-1468 [Lateral]
Oscinellinae
Family: Chloropidae
BIN URI: BOLD:AAH4180

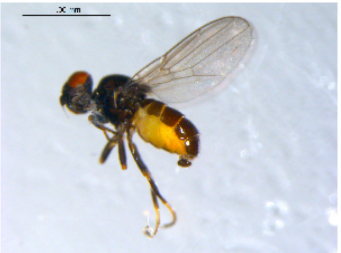

10PROBE-09607 [Lateral]
Rhopalopterum carbonarium
Family: Chloropidae
BIN URI: BOLD:AAP3813

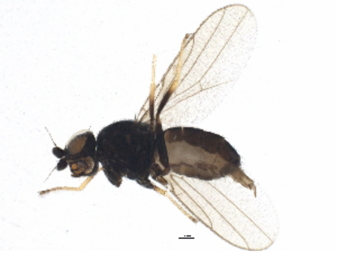

BIOUG10401-F09 [Lateral]
Oscinellinae
Family: Chloropidae
BIN URI: BOLD:AAN5662

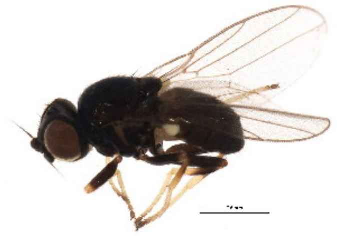

BIOUG01400-C10 [Lateral]
Chloropidae
Family: Chloropidae
BIN URI: BOLD:ABV0271

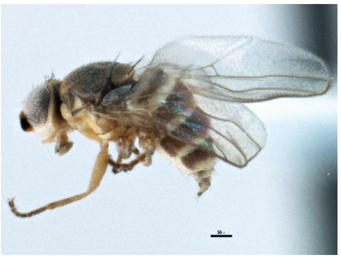

CCDB-21328-F10 [Lateral]
Incertella minor
Family: Chloropidae
BIN URI: BOLD:AAN5660

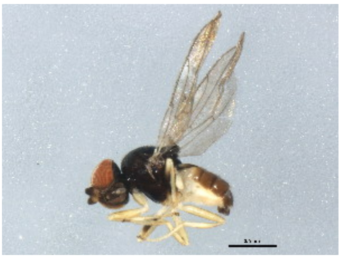

BIOUG01835-A11 [Lateral]
Incertella
Family: Chloropidae
BIN URI: BOLD:AAG6952

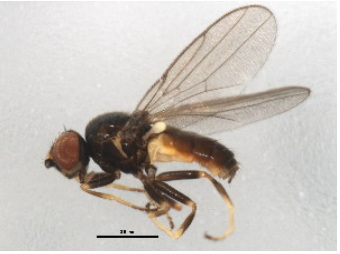

BIOUG01460-E10 [Lateral]
Chloropidae
Family: Chloropidae
BIN URI: BOLD:ABY5318

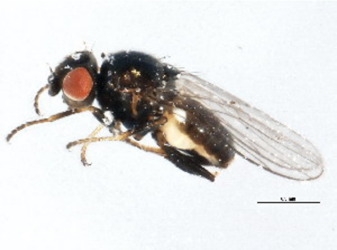

10BBDIP-0706 [Lateral]
Chloropidae
Family: Chloropidae
BIN URI: BOLD:AAH4171

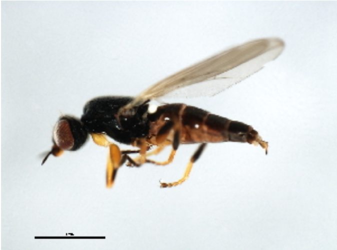

09BBDIP-0431 [Lateral]
Eribolus nana
Family: Chloropidae
BIN URI: BOLD:AAH4175

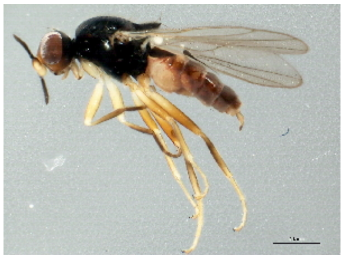

10BBDIP-2139 [Lateral]
Chloropidae
Family: Chloropidae
BIN URI: BOLD:AAP5169

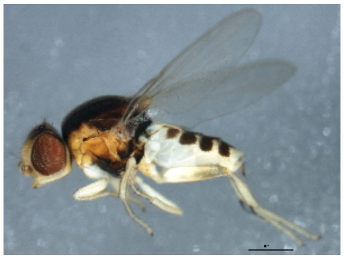

10BBDIP-1602 [Lateral]
Chloropidae
Family: Chloropidae
BIN URI: BOLD:AAH4182

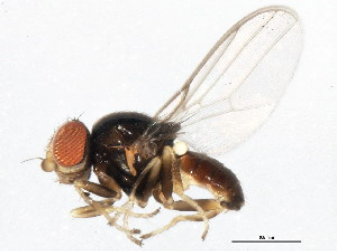

10BBDIP-1458 [Lateral]
Chloropidae
Family: Chloropidae
BIN URI: BOLD:AAP3776

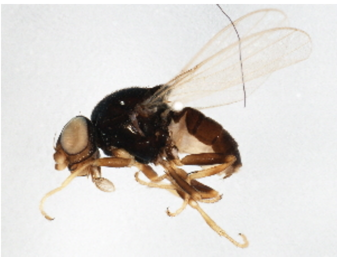

BIOUG01620-C10 [Lateral]
Chloropidae
Family: Chloropidae
BIN URI: BOLD:ABV0266

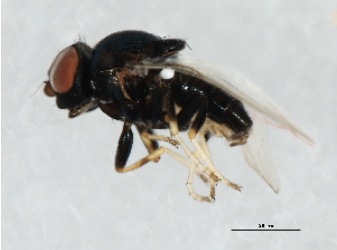

09BBDIP-1977 [Lateral]
Chloropidae
Family: Chloropidae
BIN URI: BOLD:ABZ4644

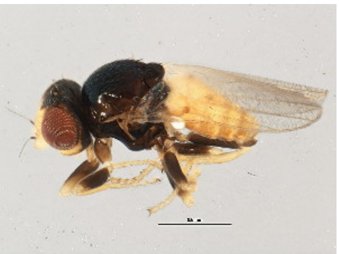

BIOUG01543-E09 [Lateral]
Chloropidae
Family: Chloropidae
BIN URI: BOLD:ABW1379

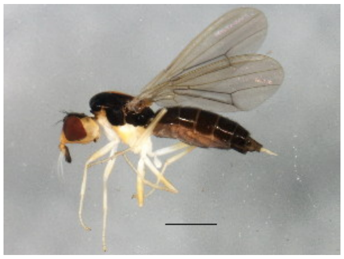

BIOUG00825-C06 [Lateral]
Psilidae
Family: Psilidae
BIN URI: BOLD:AAF9707

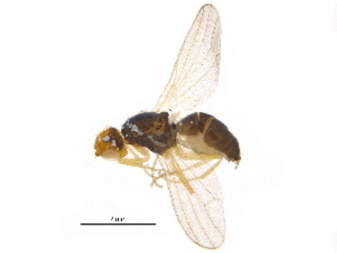

BIOUG22581-E07 [Lateral]
Psila rosae
Family: Psilidae
BIN URI: BOLD:AAP6388

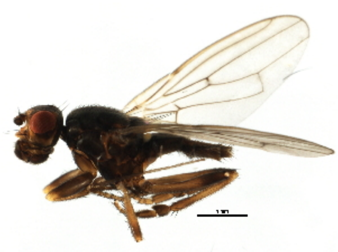

BIOUG01475-E04 [Lateral]
Sphaeroceridae
Family: Sphaeroceridae
BIN URI: BOLD:AAJ7412

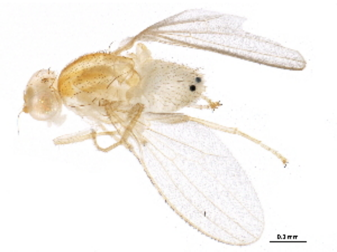

BIOUG22733-G12 [Lateral]
Gymnochiromyia concolor
Family: Chyromyidae
BIN URI: BOLD:ACV5890

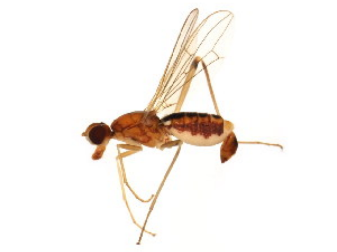

BIOUG01427-F05 [Lateral]
Micropezidae
Family: Micropezidae
BIN URI: BOLD:AAP8989

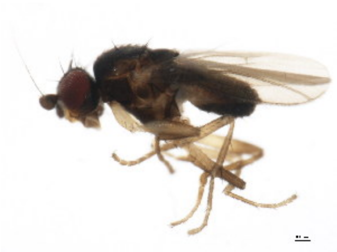

BIOUG01343-E08 [Lateral]
Sphaeroceridae
Family: Sphaeroceridae
BIN URI: BOLD:AAG7284

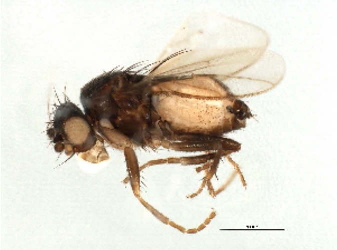

BIOUG01587-F05 [Lateral]
Sphaeroceridae
Family: Sphaeroceridae
BIN URI: BOLD:AAG7275

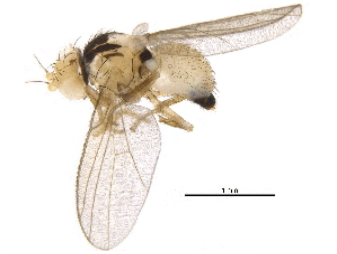

BIOUG22719-D03 [Lateral]
Phytoliriomyza
Family: Agromyzidae
BIN URI: BOLD:AAL4236

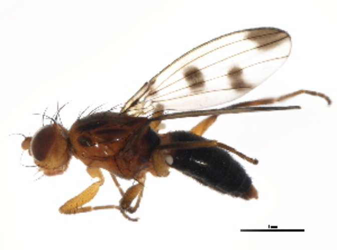

BIOUG01544-B10 [Lateral]
Opomyzidae
Family: Opomyzidae
BIN URI: BOLD:AAG3915

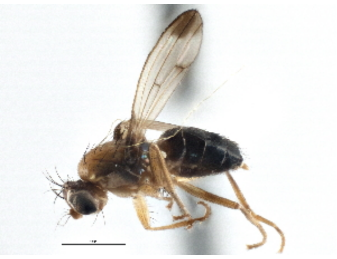

CCDB-21320-E08 [Lateral]
Geomyza apicalis
Family: Opomyzidae
BIN URI: BOLD:ACM2703

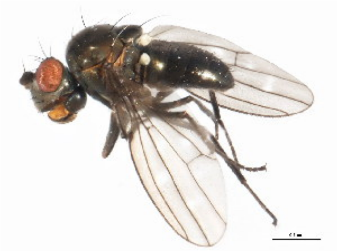

10BBDIP-0894 [Lateral]
Ephydridae
Family: Ephydridae
BIN URI: BOLD:AAG2738

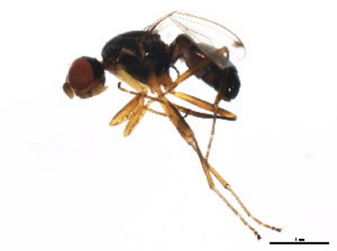

BIOUG01336-F05 [Lateral]
Sepsis punctum
Family: Sepsidae
BIN URI: BOLD:AAG5639

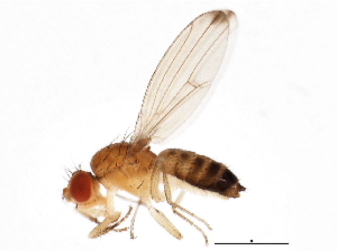

08TTML-1004 [Lateral]
Drosophilidae
Family: Drosophilidae
BIN URI: BOLD:AAG8491

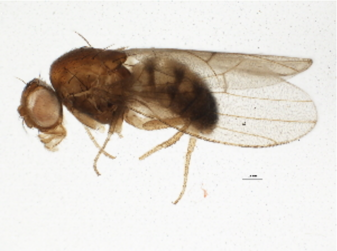

BIOUG05509-A02 [Lateral]
Drosophila putrida
Family: Drosophilidae
BIN URI: BOLD:AAF6986

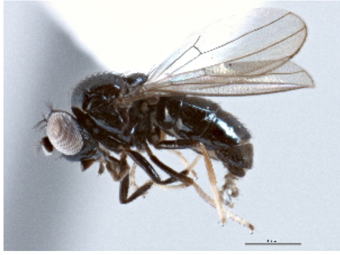

CCDB-21412-A05 [Lateral]
Athyroglossa granulosa
Family: Ephydridae
BIN URI: BOLD:ABY0801

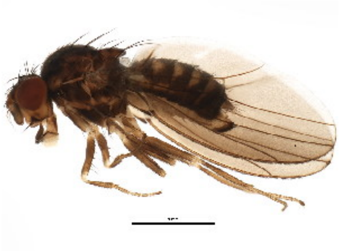

BIOUG01426-C09 [Lateral]
Drosophilidae
Family: Drosophilidae
BIN URI: BOLD:AAN5542

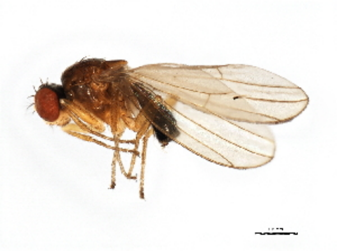

08BBDIP-0637 [Lateral]
Drosophila
Family: Drosophilidae
BIN URI: BOLD:AAG8493

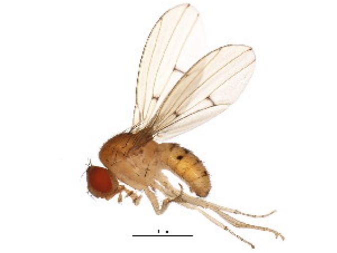

BIOUG01352-H11 [Lateral]
Drosophila falleni
Family: Drosophilidae
BIN URI: BOLD:AAB7507

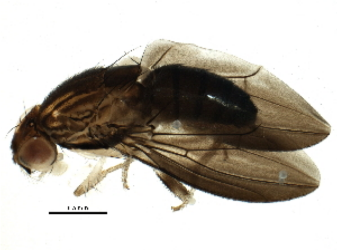

BIOUG22462-A07 [Lateral]
Stegana
Family: Drosophilidae
BIN URI: BOLD:AAN9209

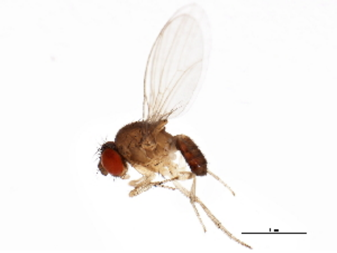

08TTML-0991 [Lateral]
Drosophilidae
Family: Drosophilidae
BIN URI: BOLD:AAB8851

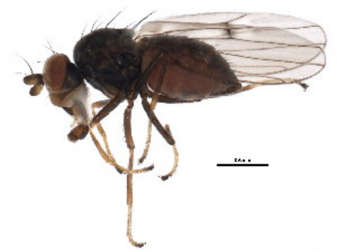

BIOUG01360-C12 [Lateral]
Philygria
Family: Ephydridae
BIN URI: BOLD:AAG2740

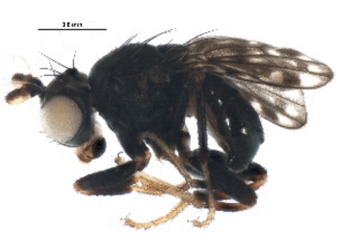

BIOUG23078-H01 [Lateral]
Ephydridae
Family: Ephydridae
BIN URI: BOLD:ACV4971

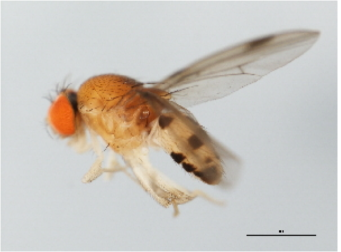

09BBDIP-1343 [Lateral]
Drosophilidae
Family: Drosophilidae
BIN URI: BOLD:AAG8500

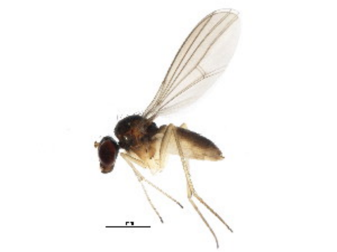

BIOUG01338-E10 [Lateral]
Dolichopodidae
Family: Dolichopodidae
BIN URI: BOLD:AAG9713

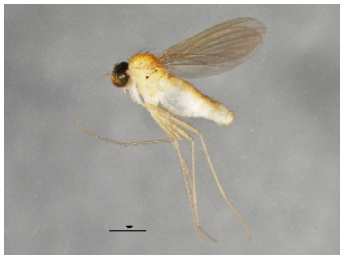

09BBEDI-1293 [Lateral]
Xanthochlorus helvinus
Family: Dolichopodidae
BIN URI: BOLD:AAG9735

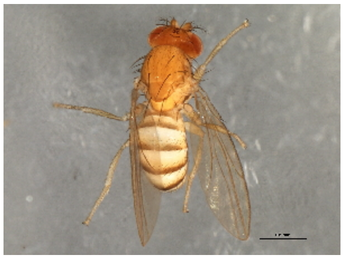

08TTML-1669 [Dorsal]
Drosophilidae
Family: Drosophilidae
BIN URI: BOLD:AAN5543

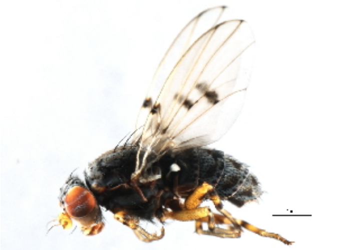

10BBCDIP-3419 [Lateral]
Odinia betulae
Family: Odiniidae
BIN URI: BOLD:AAP8071

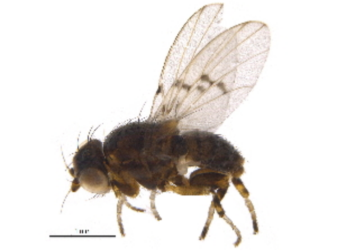

BIOUG22323-H10 [Lateral]
Odinia meijerei
Family: Odiniidae
BIN URI: BOLD:ACV3828

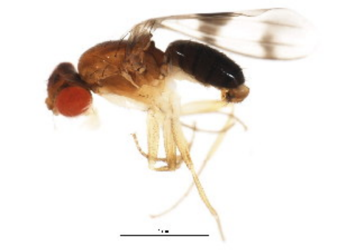

BIOUG01345-E11 [Lateral]
Chymomyza
Family: Drosophilidae
BIN URI: BOLD:AAE2703

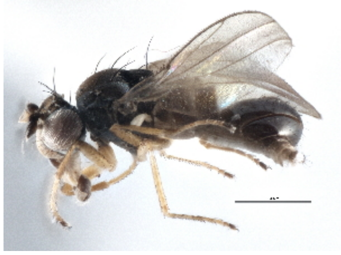

CCDB-21410-B03 [Lateral]
Nostima approximata
Family: Ephydridae
BIN URI: BOLD:AAG2754

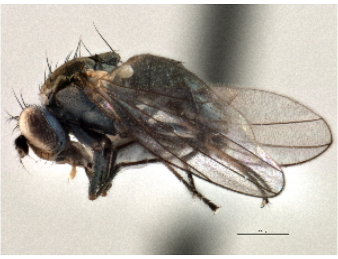

CCDB-21412-G04 [Lateral]
Hydrellia notata
Family: Ephydridae
BIN URI: BOLD:AAG9656

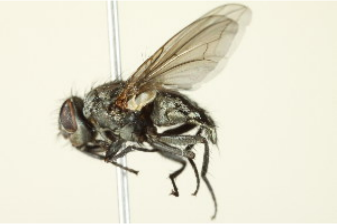

PCPP10-0126 [Lateral]
Pollenia griseotomentosa
Family: Calliphoridae
BIN URI: BOLD:AAI2766

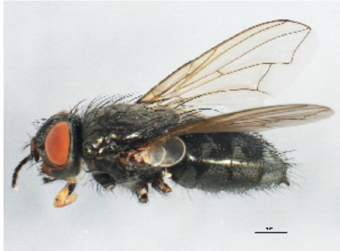

BIOUG01143-H05 [Lateral]
Pollenia labialis
Family: Calliphoridae
BIN URI: BOLD:AAI2765

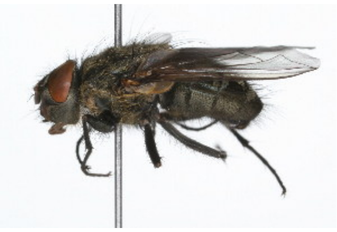

08TTML-0587 [Lateral]
Pollenia pediculata
Family: Calliphoridae
BIN URI: BOLD:AAG6745

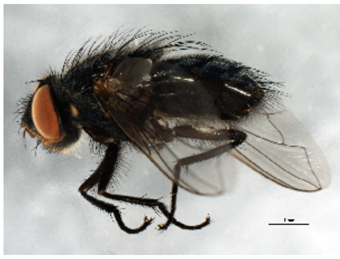

08TTML-1727 [Lateral]
Pollenia rudis
Family: Calliphoridae
BIN URI: BOLD:AAH3035

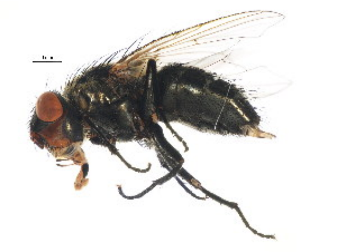

BIOUG00855-H10 [Lateral]
Pollenia rudis
Family: Calliphoridae
BIN URI: BOLD:AAP2825

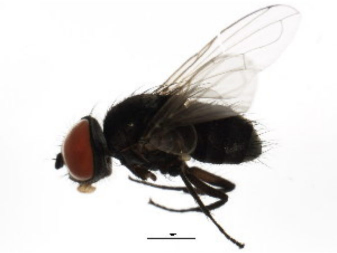

BIOUG01344-F05 [Lateral]
Strongygaster triangulifera
Family: Tachinidae
BIN URI: BOLD:AAG2355

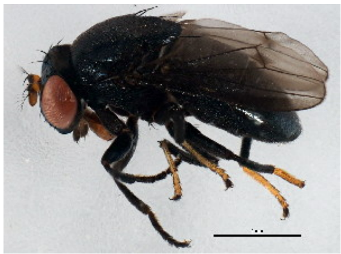

BIOUG01408-D07 [Lateral]
Ephydridae
Family: Ephydridae
BIN URI: BOLD:ABA8754

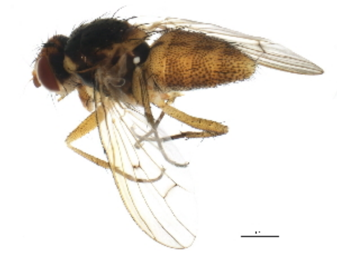

BIOUG00892-D03 [Lateral]
Anthomyiidae
Family: Anthomyiidae
BIN URI: BOLD:AAV4975

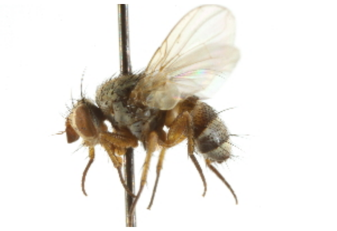

CNC DIPTERA 104991 [Lateral]
Siphona flavipes
Family: Tachinidae
BIN URI: BOLD:AAM7892

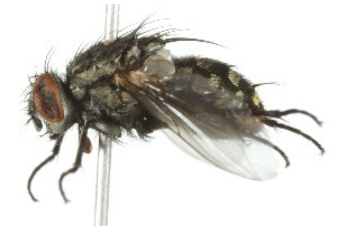

09BBDIP-2196 [Lateral]
Sarcophagidae
Family: Sarcophagidae
BIN URI: BOLD:AAA1962

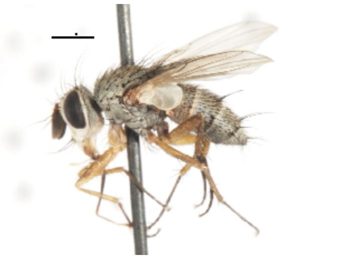

CNC DIPTERA 105063 [Lateral]
Siphona intrudens
Family: Tachinidae
BIN URI: BOLD:AAP2721

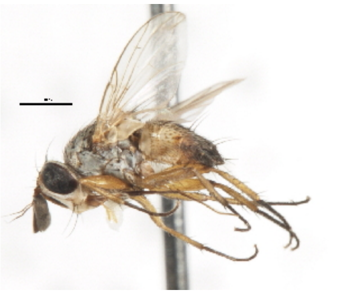

CNC DIPTERA 105061 [Lateral]
Siphona pisinnia
Family: Tachinidae
BIN URI: BOLD:AAZ4865

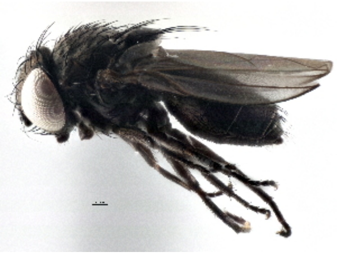

CCDB-21327-E03 [Lateral]
Pholeomyia indecora
Family: Milichiidae
BIN URI: BOLD:AAU6554

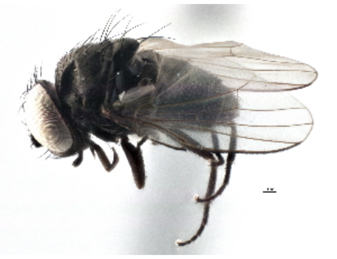

CCDB-21327-E05 [Lateral]
Pholeomyia indecora
Family: Milichiidae
BIN URI: BOLD:AAZ5488

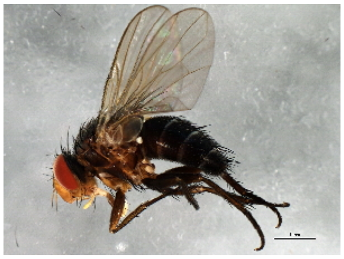

08TTML-1948 [Lateral]
Actia interrupta
Family: Tachinidae
BIN URI: BOLD:AAB8437

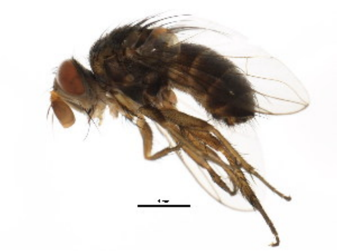

BIOUG01344-A12 [Lateral]
Ceromya
Family: Tachinidae
BIN URI: BOLD:AAP4828

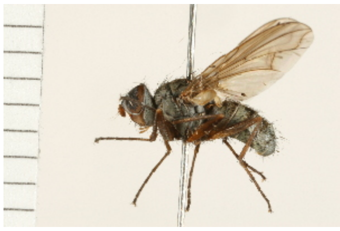

08BBDIP-1783 [Lateral]
Tachinidae
Family: Tachinidae
BIN URI: BOLD:AAG2155

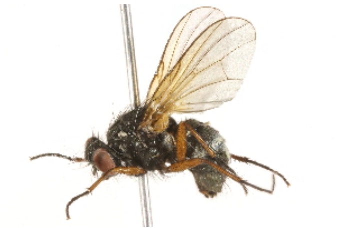

BIOUG05440-D10 [Lateral]
Mydaea urbana
Family: Muscidae
BIN URI: BOLD:ACB9959

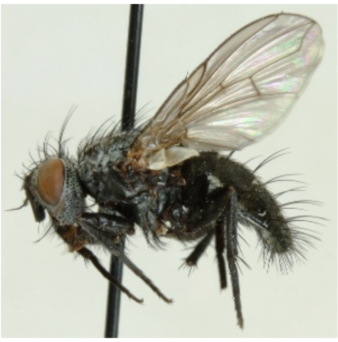

JP00245 [Lateral]
Triarthria setipennis
Family: Tachinidae
BIN URI: BOLD:ACO3992

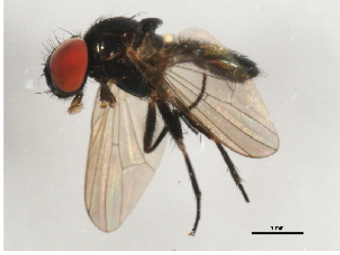

BIOUG00866-A03 [Lateral]
Fannia immaculata
Family: Fanniidae
BIN URI: BOLD:AAG4630

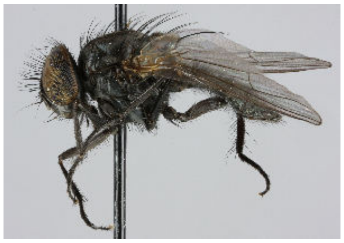

MZH_HP.249 [Lateral]
Fannia armata
Family: Fanniidae
BIN URI: BOLD:AAU6630

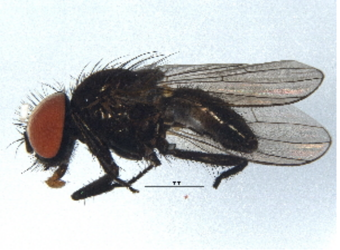

BIOUG03097-E07 [Lateral]
Fannia
Family: Fanniidae
BIN URI: BOLD:ACC1224

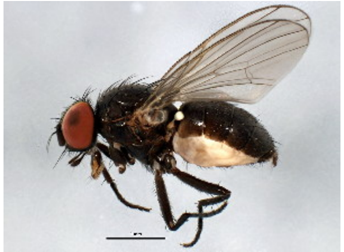

BIOUG01422-C10 [Lateral]
Fanniidae
Family: Fanniidae
BIN URI: BOLD:AAG6810

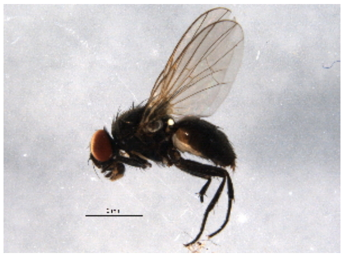

BIOUG04877-A07 [Lateral]
Fanniidae
Family: Fanniidae
BIN URI: BOLD:ACF8801

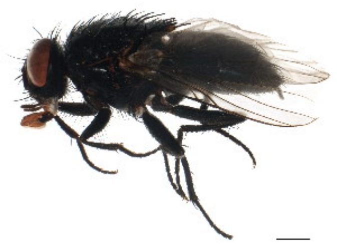

BIOUG01347-H02 [Lateral]
Muscina levida
Family: Muscidae
BIN URI: BOLD:AAB8817

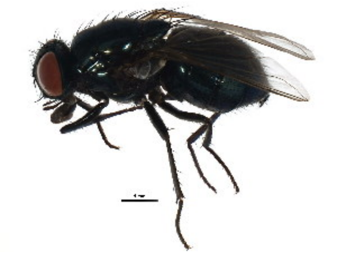

BIOUG01349-F02 [Lateral]
Eudasyphora
Family: Muscidae
BIN URI: BOLD:AAG6757

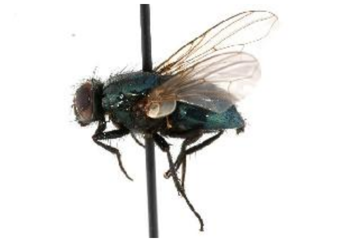

GUE06-FLY-091 [Lateral]
Diptera

BIN URI: BOLD:ABZ1424

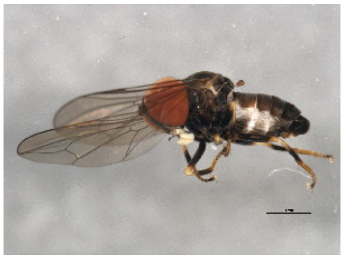

10BBCDIP-0562 [Lateral]
Eudorylas alternatus
Family: Pipunculidae
BIN URI: BOLD:AAF1875

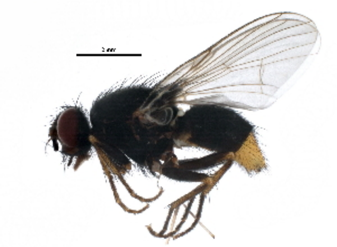

BIOUG21873-E07 [Lateral]
Muscidae
Family: Muscidae
BIN URI: BOLD:ACV4140

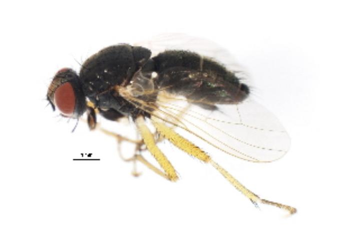

10JSROW-1465 [Lateral]
Macrorchis ausoba
Family: Muscidae
BIN URI: BOLD:AAG1701

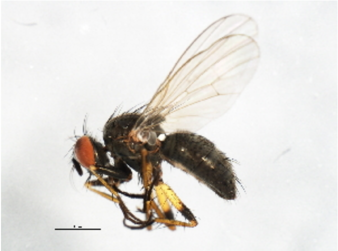

09BBDIP-0721 [Lateral]
Coenosia
Family: Muscidae
BIN URI: BOLD:AAG1741

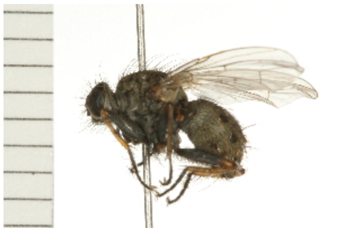

08OEC-121 [Lateral]
Coenosia tigrina
Family: Muscidae
BIN URI: BOLD:AAB5609

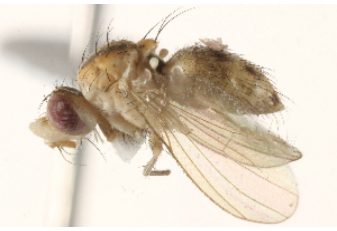

BIOUG03689-F10 [Lateral]
Coenosia
Family: Muscidae
BIN URI: BOLD:AAG1759

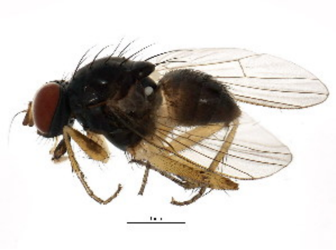

BIOUG01364-F10 [Lateral]
Coenosia
Family: Muscidae
BIN URI: BOLD:AAG1769

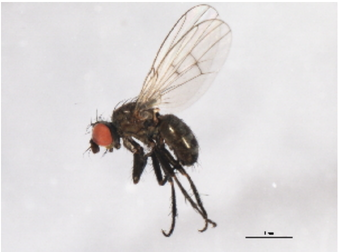

08BBDIP-2622 [Lateral]
Schoenomyza
Family: Muscidae
BIN URI: BOLD:AAG4622

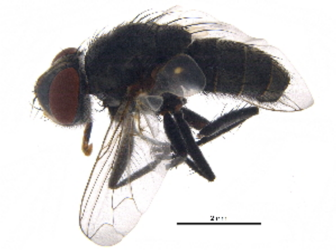

BIOUG22325-H02 [Lateral]
Senotainia trilineata
Family: Sarcophagidae
BIN URI: BOLD:AAG6744

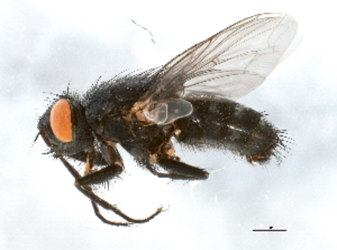

BIOUG01639-B12 [Lateral]
Sarcophagidae
Family: Sarcophagidae
BIN URI: BOLD:ABV1243

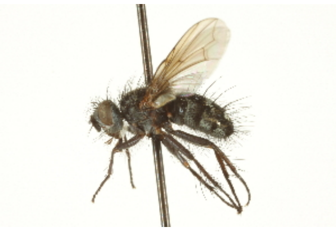

CNC DIPTERA 162295 [Lateral]
Lypha fumipennis
Family: Tachinidae
BIN URI: BOLD:AAF6259

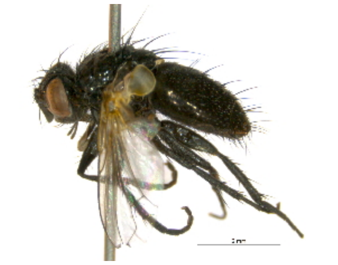

BIOUG04342-F09 [Lateral]
Lydina americana
Family: Tachinidae
BIN URI: BOLD:AAG2432

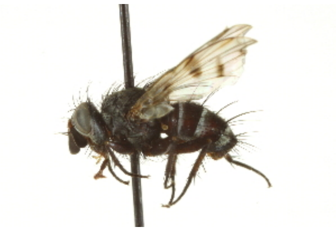

CNC DIPTERA 162267 [Lateral]
Homalactia harringtoni
Family: Tachinidae
BIN URI: BOLD:AAP2717

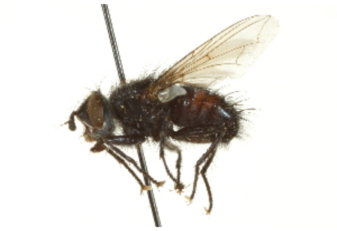

CNC DIPTERA 162146 [Lateral]
Panzeria arcuata
Family: Tachinidae
BIN URI: BOLD:ABX8463

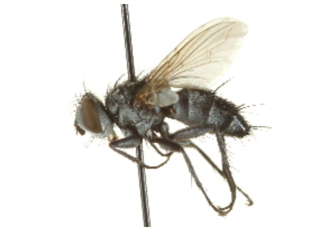

CNC DIPTERA 162002 [Lateral]
Ceracia dentata
Family: Tachinidae
BIN URI: BOLD:ABX6290

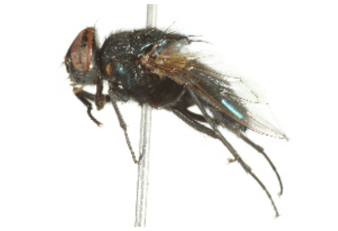

10BBDIP-0331 [Lateral]
Phormia regina
Family: Calliphoridae
BIN URI: BOLD:AAB9140

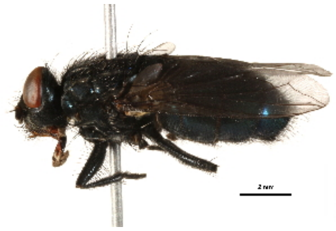

BIOUG04921-F06 [Lateral]
Protocalliphora tundrae
Family: Calliphoridae
BIN URI: BOLD:AAH3037

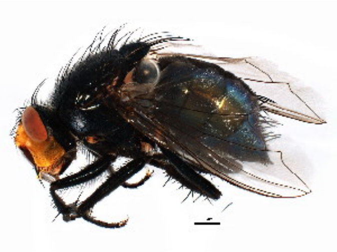

BIOUG01914-B10 [Lateral]
Cynomya mortuorum
Family: Calliphoridae
BIN URI: BOLD:AAB0868

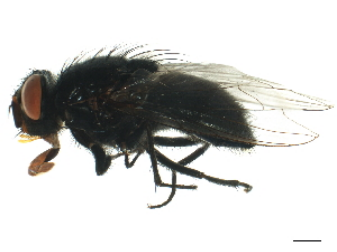

BIOUG01347-H08 [Lateral]
Calliphora livida
Family: Calliphoridae
BIN URI: BOLD:ABY7153

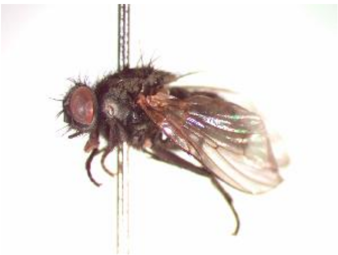

BUIC-CHU0556 [Lateral]
Myospila meditabunda
Family: Muscidae
BIN URI: BOLD:AAD7145

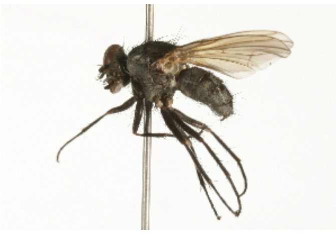

08TTML-0524 [Lateral]
Anthomyiidae
Family: Anthomyiidae
BIN URI: BOLD:AAG1712

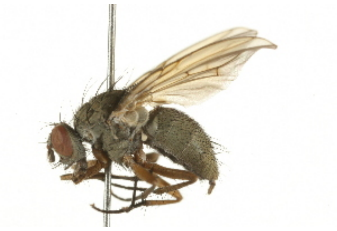

08TTML-0730 [Lateral]
Helina depuncta
Family: Muscidae
BIN URI: BOLD:AAG1711

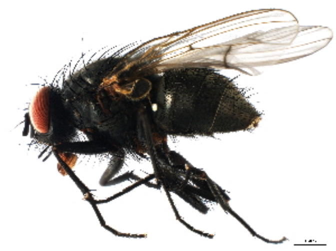

BIOUG01392-A04 [Lateral]
Helina evecta
Family: Muscidae
BIN URI: BOLD:AAC2498

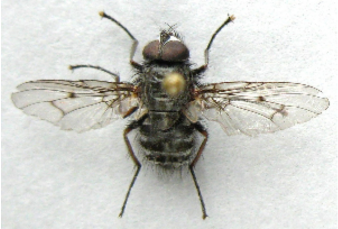

BIOUG06723-H08 [Dorsal]
Diptera

BIN URI: BOLD:AAG1742

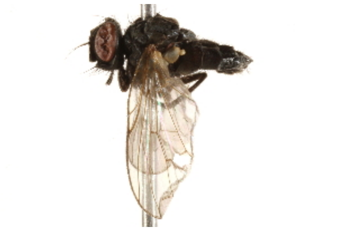

BIOUG13659-F08 [Lateral]
Phaonia
Family: Muscidae
BIN URI: BOLD:AAP2512

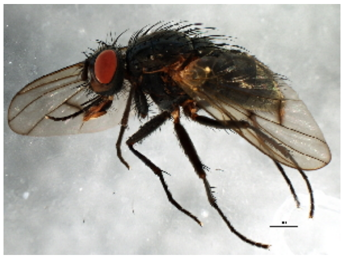

08TTML-1945 [Lateral]
Phaonia
Family: Muscidae
BIN URI: BOLD:AAM9107

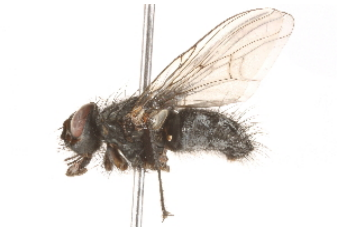

BIOUG13061-A02 [Lateral]
Phaonia
Family: Muscidae
BIN URI: BOLD:ABU9891

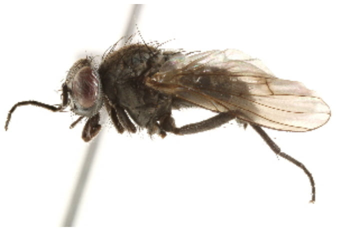

BIOUG04288-G12 [Lateral]
Phaonia
Family: Muscidae
BIN URI: BOLD:ABV1241

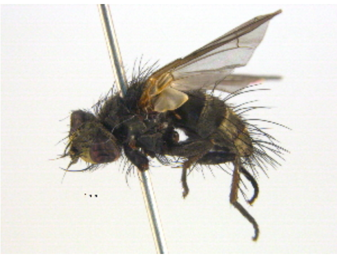

BIOUG03215-D01 [Lateral]
Blepharomyia pagana
Family: Tachinidae
BIN URI: BOLD:AAV0903

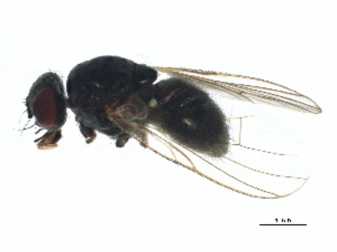

BIOUG16073-D03 [Lateral]
Spilogona
Family: Muscidae
BIN URI: BOLD:ACP7541

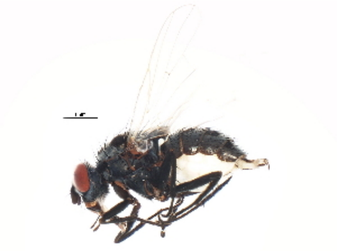

10JSROW-1443 [Lateral]
Anthomyia pluvialis
Family: Anthomyiidae
BIN URI: BOLD:AAP2970

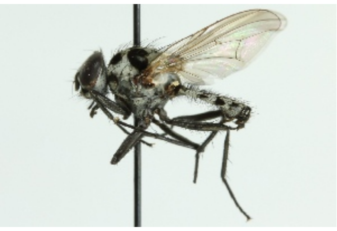

MZH_HP.437 [Lateral]
Anthomyia pluvialis
Family: Anthomyiidae
BIN URI: BOLD:AAQ0583

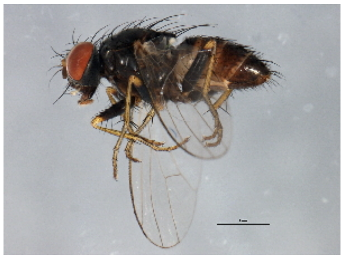

08TTML-1053 [Lateral]
Lispocephala erythrocera
Family: Muscidae
BIN URI: BOLD:AAG1704

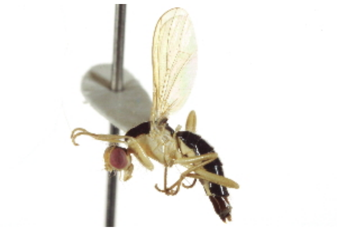

CCDB-21400-D05 [Lateral]
Cordilura gracilipes
Family: Scathophagidae
BIN URI: BOLD:AAH4229

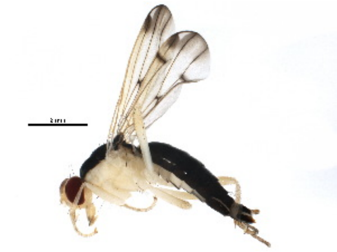

BIOUG21873-G07 [Lateral]
Cordilura
Family: Scathophagidae
BIN URI: BOLD:AAH4225

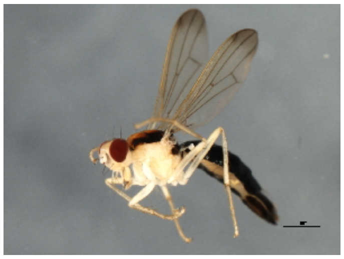

09BBEDI-1932 [Lateral]
Scathophagidae
Family: Scathophagidae
BIN URI: BOLD:AAH4236

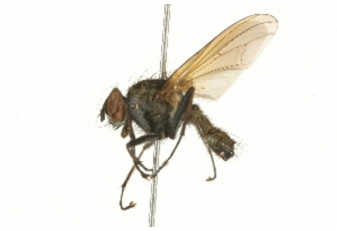

08TTML-0739 [Lateral]
Anthomyiidae
Family: Anthomyiidae
BIN URI: BOLD:AAG2460

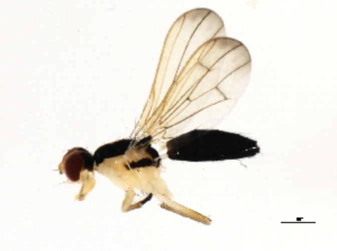

09BBEDI-1899 [Lateral]
Americina adusta
Family: Scathophagidae
BIN URI: BOLD:AAH4235

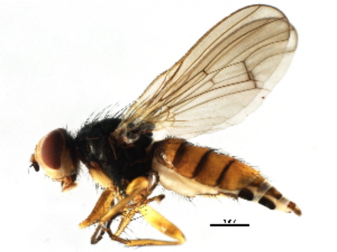

BIOUG01475-E01 [Lateral]
Anthomyiidae
Family: Anthomyiidae
BIN URI: BOLD:ABW1307

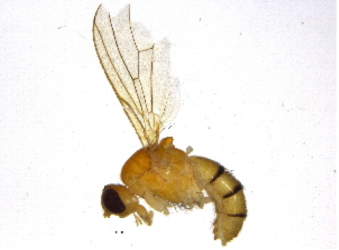

BIOUG04331-A07 [Lateral]
Scathophagidae
Family: Scathophagidae
BIN URI: BOLD:ACM2222

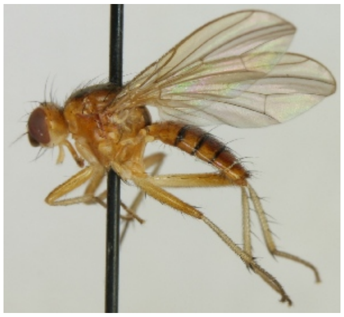

jka10-00851 [Lateral]
Megaphthalma pallida

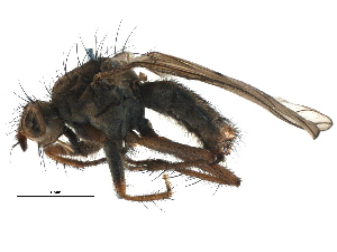

BIOUG08898-F10 [Lateral]
Scathophaga furcata
Family: Scathophagidae
BIN URI: BOLD:AAH0022

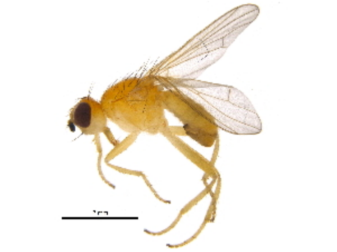

BIOUG22241-E12 [Lateral]
Scathophaga
Family: Scathophagidae
BIN URI: BOLD:ACU9724

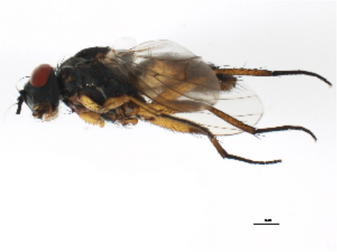

BIOUG01373-A10 [Lateral]
Anthomyiidae
Family: Anthomyiidae
BIN URI: BOLD:AAG2513

Family: Scathophagidae
BIN URI: BOLD:AAH4234

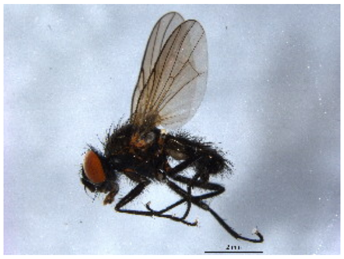

BIOUG03018-C05 [Lateral]
Anthomyiidae
Family: Anthomyiidae
BIN URI: BOLD:AAP8831

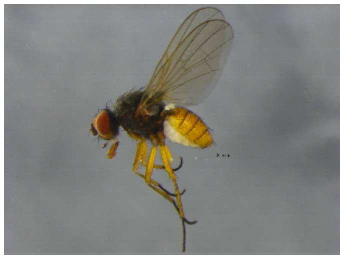

BIOUG03381-H10 [Lateral]
Anthomyiidae
Family: Anthomyiidae
BIN URI: BOLD:AAG2479

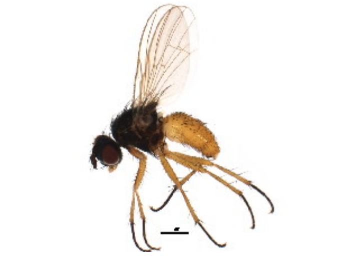

BIOUG01411-C06 [Lateral]
Anthomyiidae
Family: Anthomyiidae
BIN URI: BOLD:AAN5497

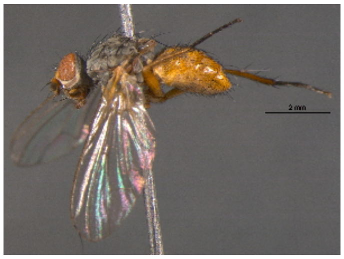

BIOUG03400-H08 [Lateral]
Pegomya
Family: Anthomyiidae
BIN URI: BOLD:ACB2221

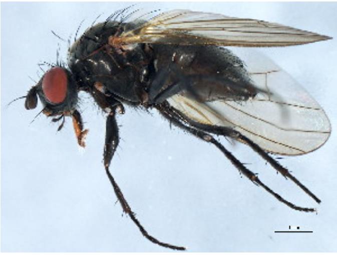

10JSROW-0273 [Lateral]
Anthomyiidae
Family: Anthomyiidae
BIN URI: BOLD:AAP2968

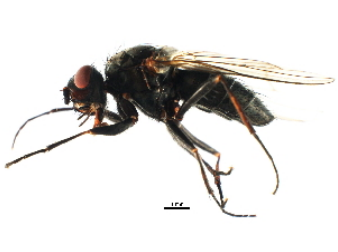

BIOUG01475-C02 [Lateral]
Anthomyiidae
Family: Anthomyiidae
BIN URI: BOLD:AAP2967

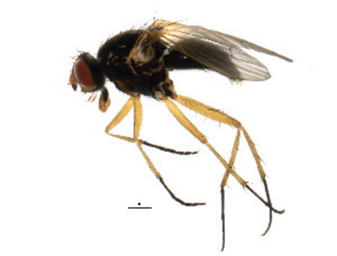

BIOUG01344-F01 [Lateral]
Anthomyiidae
Family: Anthomyiidae
BIN URI: BOLD:ABW1309

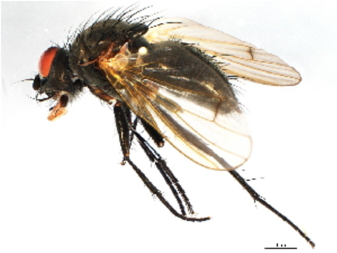

10PHMAL-0954 [Lateral]
Anthomyiidae
Family: Anthomyiidae
BIN URI: BOLD:AAM7339

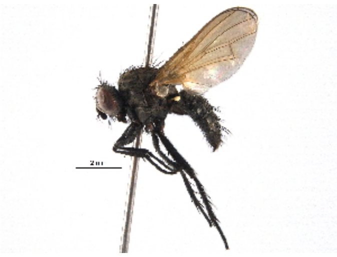

BIOUG10177-C10 [Lateral]
Anthomyiidae
Family: Anthomyiidae
BIN URI: BOLD:ACL8023

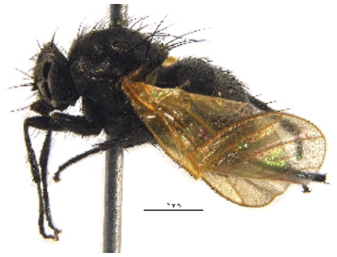

BIOUG13702-F04 [Lateral]
Anthomyiidae
Family: Anthomyiidae
BIN URI: BOLD:ACP3345

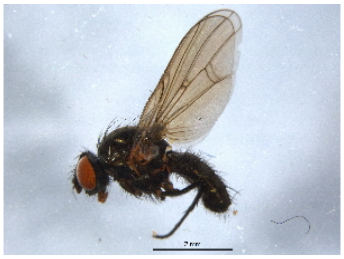

BIOUG02989-F07 [Lateral]
Hylemya
Family: Anthomyiidae
BIN URI: BOLD:ABW2407

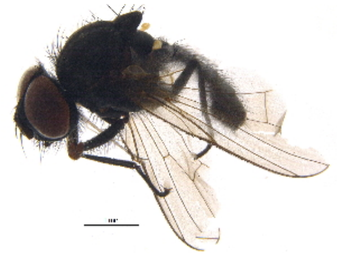

BIOUG22325-A12 [Lateral]
Lasiomma
Family: Anthomyiidae
BIN URI: BOLD:AAL7525

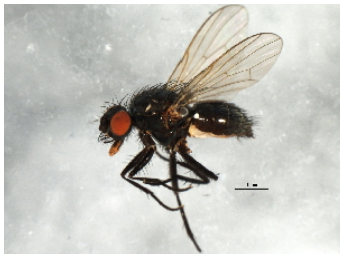

08TTML-1946 [Lateral]
Anthomyiidae
Family: Anthomyiidae
BIN URI: BOLD:AAG2463

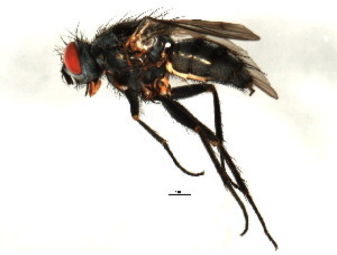

BIOUG00938-F04 [Lateral]
Anthomyiidae
Family: Anthomyiidae
BIN URI: BOLD:AAP8833

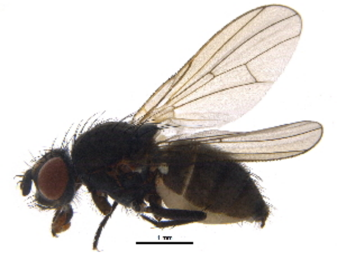

BIOUG22325-A03 [Lateral]
Delia
Family: Anthomyiidae
BIN URI: BOLD:AAP2973

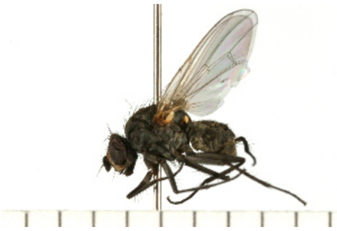

08FAYTT-0085 [Lateral]
Delia florilega
Family: Anthomyiidae
BIN URI: BOLD:ACR4394

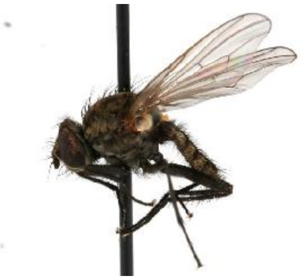

GUE06-FLY-069 [Lateral]
Diptera

BIN URI: BOLD:AAA3453

IMAGE NOT AVAILABLE

BIOUG22843-D03
Delia antiqua
Family: Anthomyiidae

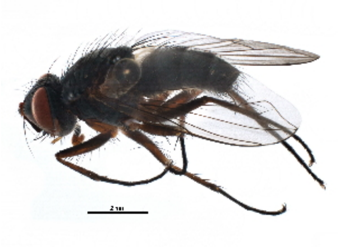

BIOUG21873-D07 [Lateral]
Cryptomeigenia
Family: Tachinidae
BIN URI: BOLD:AAG2128

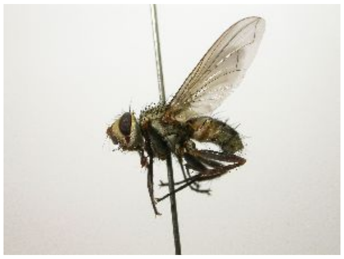

Haber 000797 [Lateral]
Cryptomeigenia
Family: Tachinidae
BIN URI: BOLD:ABZ1975

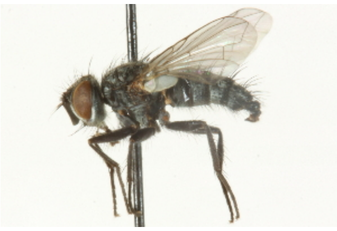

CNC DIPTERA 104565 [Lateral]
Oswaldia minor
Family: Tachinidae
BIN URI: BOLD:ACF1129

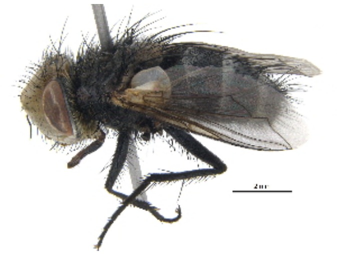

BIOUG21607-B12 [Lateral]
Gonia
Family: Tachinidae
BIN URI: BOLD:ACF7092

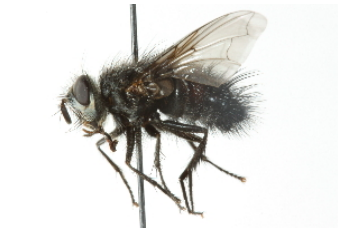

CNC DIPTERA 103928 [Lateral]
Leschenaultia exul
Family: Tachinidae
BIN URI: BOLD:ACE2864

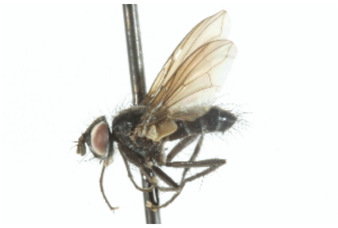

CNC DIPTERA 104287 [Lateral]
Medina barbata
Family: Tachinidae
BIN URI: BOLD:AAG6902

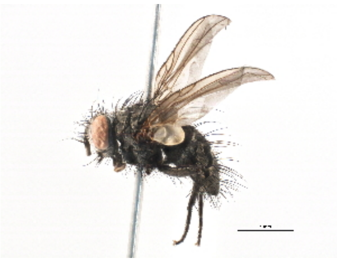

BIOUG08956-F01 [Lateral]
Eucelatoria
Family: Tachinidae
BIN URI: BOLD:ACG6047

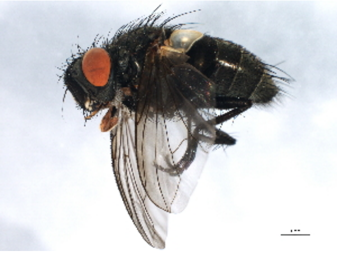

BIOUG01544-B02 [Lateral]
Tachinidae
Family: Tachinidae
BIN URI: BOLD:AAG2348

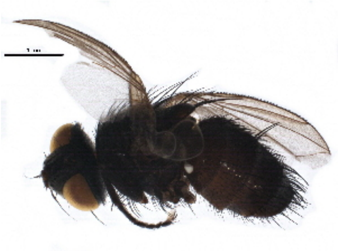

BIOUG24012-E10 [Lateral]
Lixophaga
Family: Tachinidae

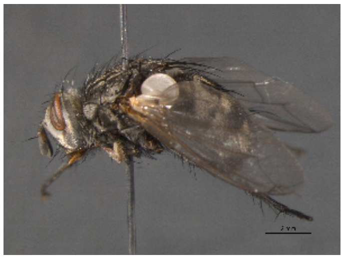

BIOUG05615-E11 [Lateral]
Phorocera
Family: Tachinidae
BIN URI: BOLD:AAG2146

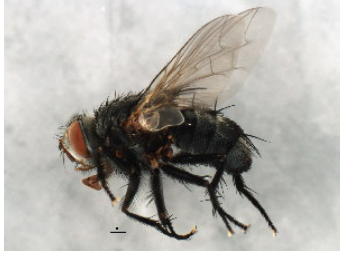

BIOUG01427-G04 [Lateral]
Tachinidae
Family: Tachinidae
BIN URI: BOLD:AAP8654

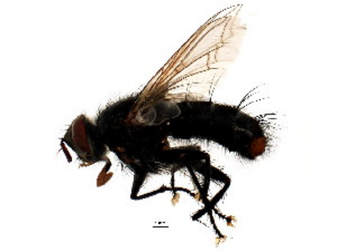

BIOUG00992-E09 [Lateral]
Phorocera obscura
Family: Tachinidae
BIN URI: BOLD:ABY8575

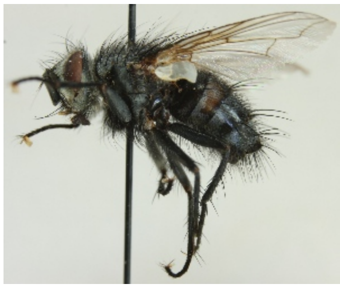

JP00114 [Lateral]
Parasetigena silvestris
Family: Tachinidae
BIN URI: BOLD:ABV1657

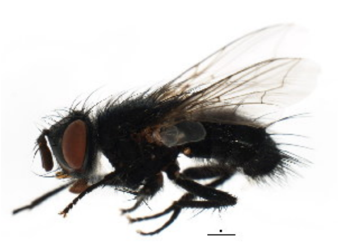

BIOUG01344-H01 [Lateral]
Phorocera obscura
Family: Tachinidae
BIN URI: BOLD:AAP4830

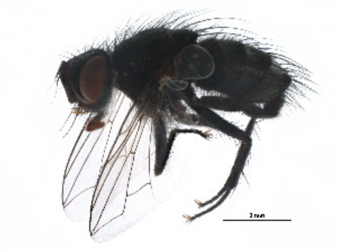

BIOUG21873-C06 [Lateral]
Tachinidae
Family: Tachinidae
BIN URI: BOLD:ACV4527

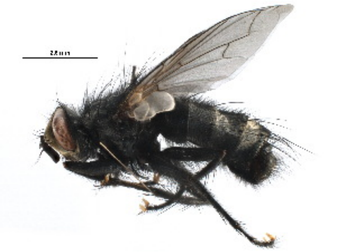

BIOUG22360-E02 [Lateral]
Exoristinae
Family: Tachinidae
BIN URI: BOLD:ACQ2060

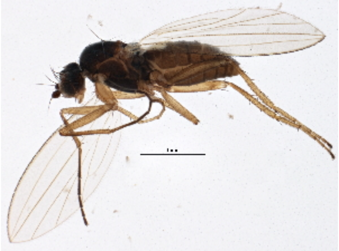

BIOUG05636-E02 [Lateral]
Lonchoptera bifurcata
Family: Lonchopteridae
BIN URI: BOLD:AAG9974

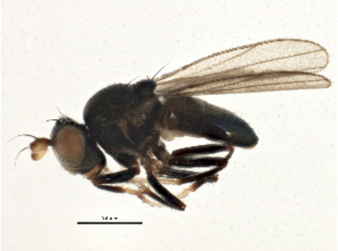

BIOUG05535-E10 [Lateral]
Ephydridae
Family: Ephydridae
BIN URI: BOLD:ABX2181

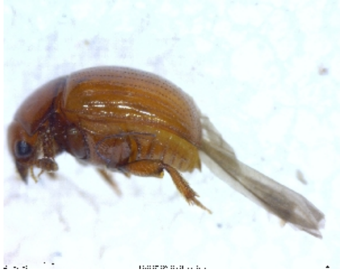

BIOUG01043_D05 [Lateral]
Leiodidae
Family: Leiodidae
BIN URI: BOLD:ABA6310

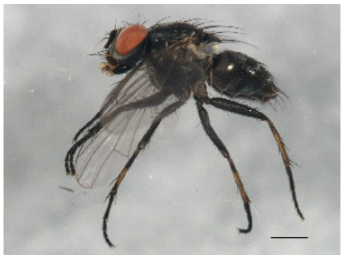

10BBCDIP-1257 [Lateral]
Lispe albitarsis
Family: Muscidae
BIN URI: BOLD:AAP1125

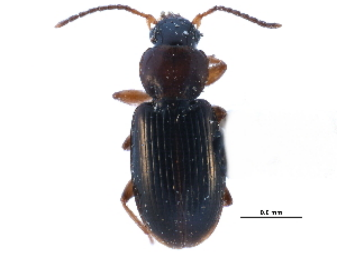

CCDB-23051-F10 [Dorsal]
Bradycellus neglectus
Family: Carabidae
BIN URI: BOLD:AAE3092

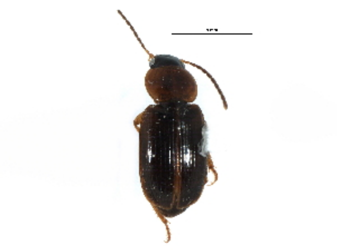

CCDB-22962-B11 [Dorsal]
Stenolophus conjunctus
Family: Carabidae
BIN URI: BOLD:AAE9008

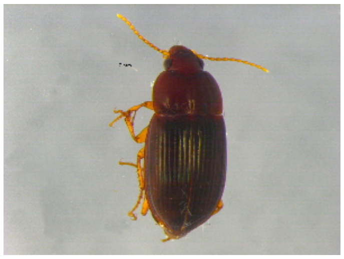

BIOUG03138-C03 [Dorsal]
Amara rubrica
Family: Carabidae
BIN URI: BOLD:AAM7658

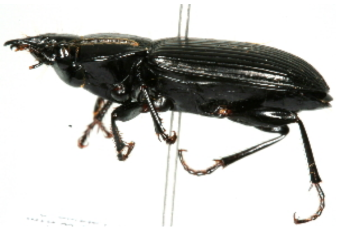

09BBECO-0078 [Lateral]
Pterostichus melanarius
Family: Carabidae
BIN URI: BOLD:AAC0661

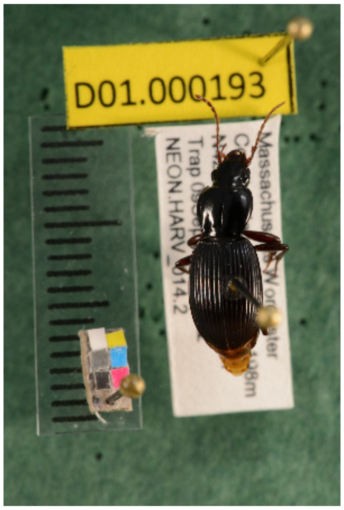

D01.000193 [Dorsal]
Carabidae D01morph_B
Family: Carabidae
BIN URI: BOLD:AAN6180

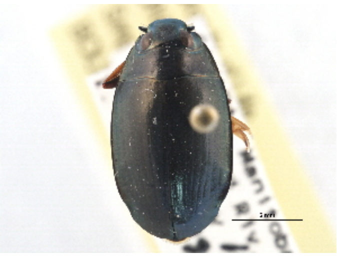

CCDB-21430-F05 [Dorsal]
Dineutus assimilis
Family: Gyrinidae
BIN URI: BOLD:ABX0920

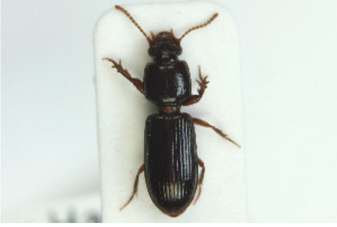

ZMUO.000458 [Dorsal]
Clivina fossor
Family: Carabidae
BIN URI: BOLD:AAH0274

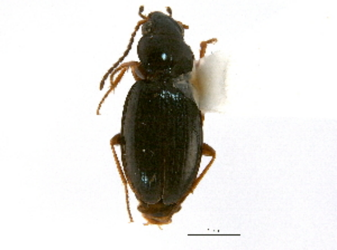

CCDB-21405-D12 [Dorsal]
Bembidion obtusum
Family: Carabidae
BIN URI: BOLD:AAP9490

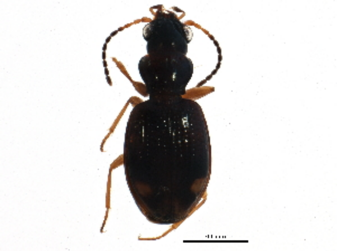

BIOUG21883-E04 [Dorsal]
Bembidion frontale
Family: Carabidae
BIN URI: BOLD:AAU7150

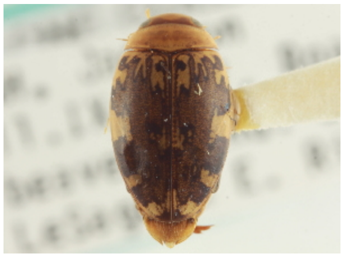

CNC COLEO 00252387 [Dorsal]
Laccophilus maculosus maculosus
Family: Dytiscidae
BIN URI: BOLD:AAH0221

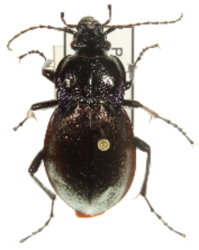

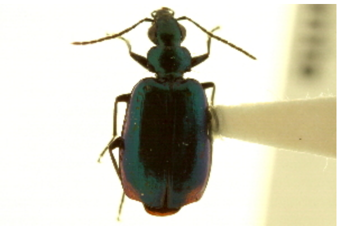

CNC COLEO 00119446 [Dorsal]

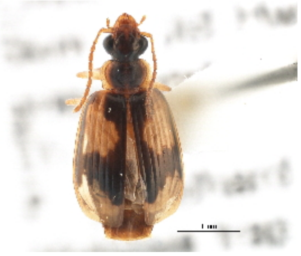

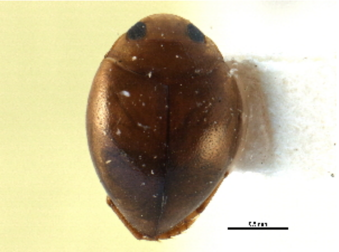

BIOUG09887-A03 [Dorsal]
Carabus
Family: Carabidae
BIN URI: BOLD:AAH2826

Lebia viridis
Family: Carabidae
BIN URI: BOLD:AAH0141

CCDB-21430-B11 [Dorsal]
Lebia fasciata
Family: Carabidae
BIN URI: BOLD:AAH0212

CCDB-21428-G12 [Dorsal]
Desmopachria convexa
Family: Dytiscidae
BIN URI: BOLD:ACO4071

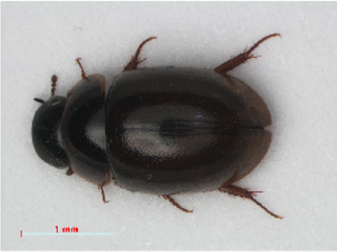

RMNH.INS.536361 [Dorsal]
Anacaena lutescens
Family: Hydrophilidae
BIN URI: BOLD:AAF4596

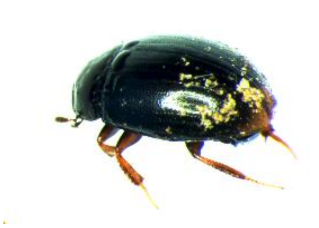

GBOL_Col_FK_8339 [Lateral]
Cercyon haemorrhoidalis
Family: Hydrophilidae
BIN URI: BOLD:ABV1545

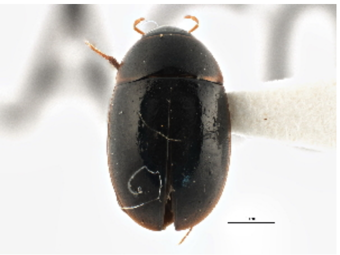

BIOUG09883-A12 [Dorsal]
Cymbiodyta vindicata
Family: Hydrophilidae
BIN URI: BOLD:ACM5968

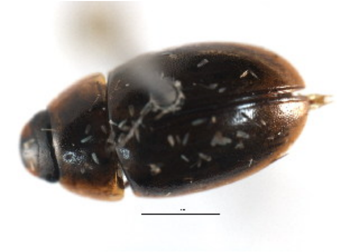

08SOCOL-0247 [Dorsal]
Enochrus ochraceus
Family: Hydrophilidae
BIN URI: BOLD:AAH2910

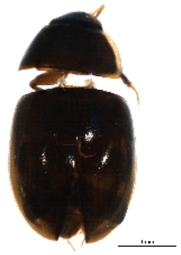

BIOUG24040-H03 [Dorsal]
Enochrus
Family: Hydrophilidae

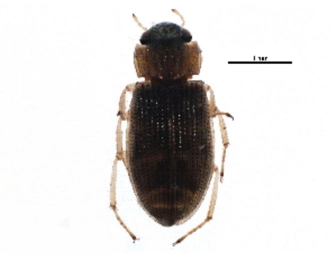

BIOUG24027-A05 [Dorsal]
Helophorinae
Family: Hydrophilidae

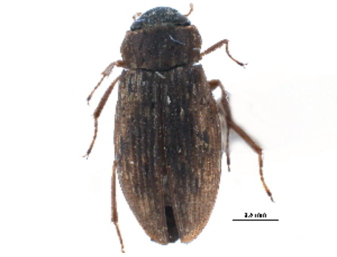

CCDB-23054-F03 [Dorsal]
Helophorus lacustris
Family: Hydrophilidae
BIN URI: BOLD:ACR8815

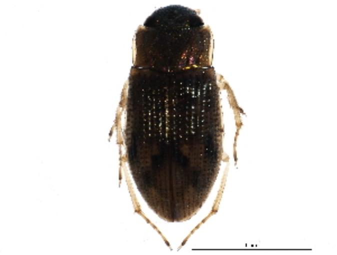

BIOUG24040-H09 [Dorsal]
Hydrophilidae
Family: Hydrophilidae

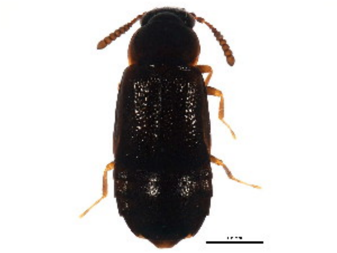

BIOUG01144-B06 [Dorsal]
Phyllodrepa
Family: Staphylinidae
BIN URI: BOLD:ABW5502

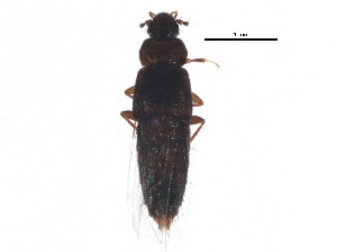

BIOUG22867-A07 [Dorsal]
Trichophya pilicornis
Family: Staphylinidae
BIN URI: BOLD:ABW9580

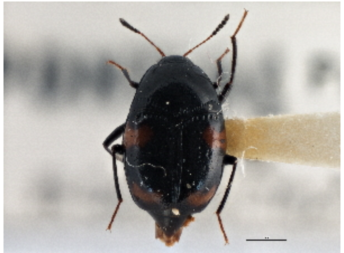

CNC COLEO 00162916 [Dorsal]
Scaphidium quadriguttatum
Family: Staphylinidae
BIN URI: BOLD:ACP0011

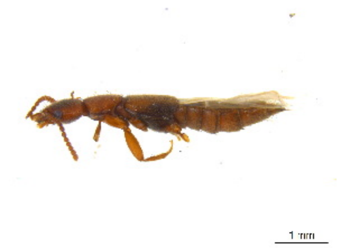

BIOUG06758-E02 [Lateral]
Staphylinidae
Family: Staphylinidae
BIN URI: BOLD:AAY6538

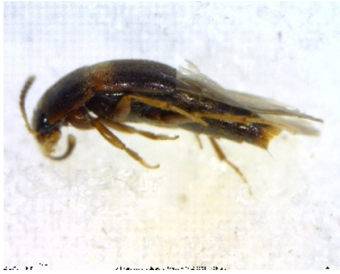

BIOUG02612-B07 [Lateral]
Staphylinidae
Family: Staphylinidae
BIN URI: BOLD:ACC1294

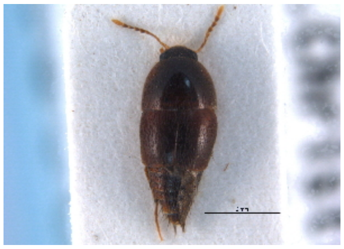

CNC COLEO 00161467 [Dorsal]
Sepedophilus versicolor
Family: Staphylinidae
BIN URI: BOLD:AAP6950

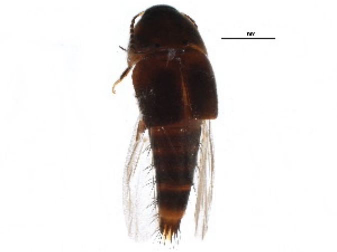

BIOUG22422-D02 [Dorsal]
Staphylinidae
Family: Staphylinidae
BIN URI: BOLD:ACJ3516

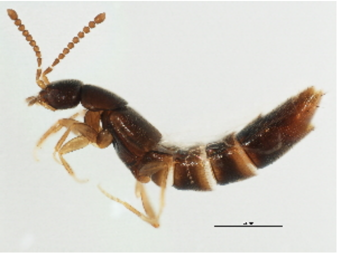

BIOUG01593-D03 [Lateral]
Amischa analis
Family: Staphylinidae
BIN URI: BOLD:ABA5313

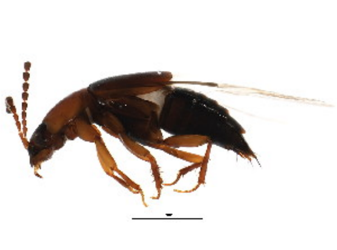

BIOUG01144-E10 [Lateral]
Tachinus corticinus
Family: Staphylinidae
BIN URI: BOLD:AAH0107

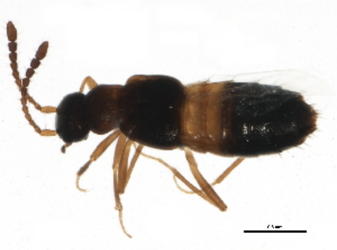

BIOUG01502-C11 [Lateral]
Meronera
Family: Staphylinidae
BIN URI: BOLD:ABW2870

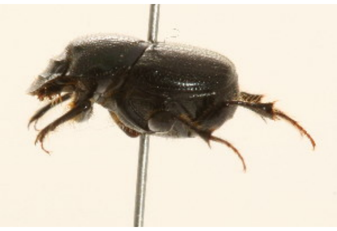

09BBCOL-0754 [Lateral]
Onthophagus orpheus orpheus
Family: Scarabaeidae
BIN URI: BOLD:AAH6827

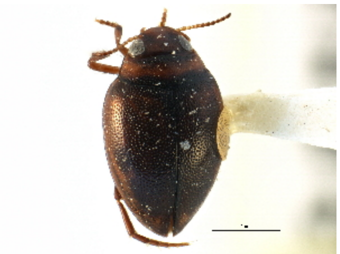

CCDB-21428-H08 [Dorsal]
Hygrotus intermedius
Family: Dytiscidae
BIN URI: BOLD:AAN3354

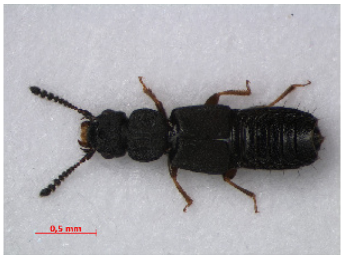

RMNH.INS.535818 [Dorsal]
Anotylus tetracarinatus
Family: Staphylinidae
BIN URI: BOLD:AAN1738

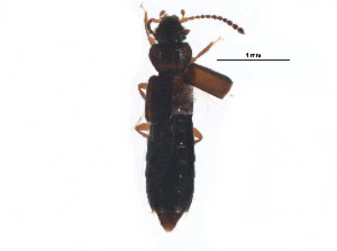

BIOUG22867-B04 [Dorsal]
Carpelimus fuliginosus
Family: Staphylinidae
BIN URI: BOLD:AAO0558

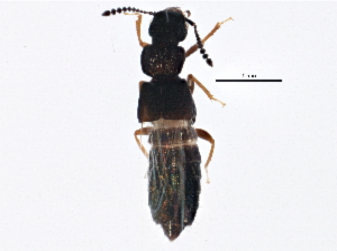

BIOUG05586-D02 [Dorsal]
Anotylus
Family: Staphylinidae
BIN URI: BOLD:AAH0104

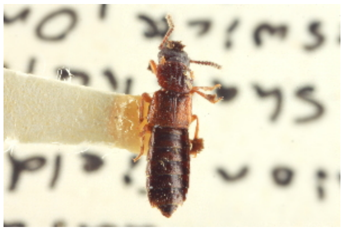

CNC COLEO 00250918 [Dorsal]
Anotylus insecatus
Family: Staphylinidae
BIN URI: BOLD:AAR3352

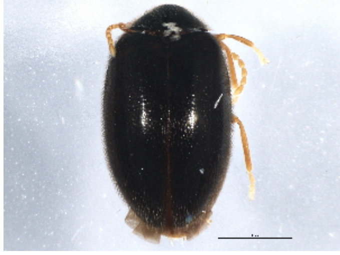

BIOUG10131-A12 [Dorsal]
Cyphon
Family: Scirtidae
BIN URI: BOLD:AAM7653

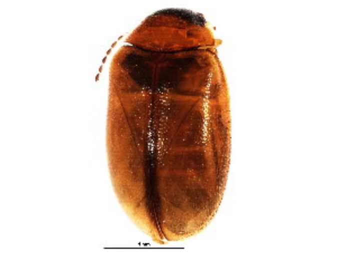

BIOUG00993-G03 [Dorsal]
Cyphon laevipennis
Family: Scirtidae
BIN URI: BOLD:AAG3633

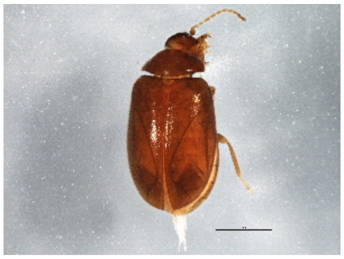

BIOUG02856-D06 [Dorsal]
Cyphon obscurus
Family: Scirtidae
BIN URI: BOLD:AAG7259

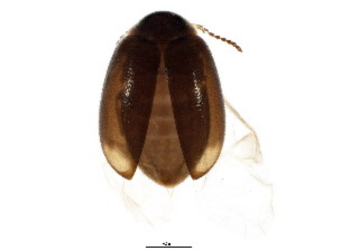

BIOUG01502-B02 [Dorsal]
Cyphon pusillus
Family: Scirtidae
BIN URI: BOLD:AAP7021

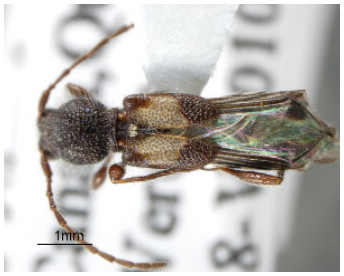

CNCCOLVG00001507 [Dorsal]
Molorchus bimaculatus
Family: Cerambycidae
BIN URI: BOLD:AAH0019

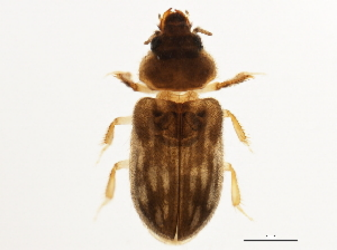

BIOUG00670-B06 [Dorsal]
Lapsus tristis
Family: Heteroceridae
BIN URI: BOLD:AAP7099

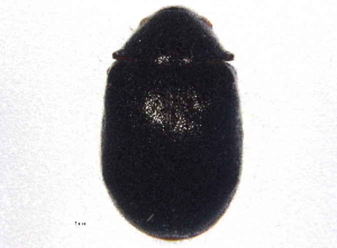

BIOUG07429-H02 [Dorsal]
Ptinidae
Family: Ptinidae
BIN URI: BOLD:ACC7074

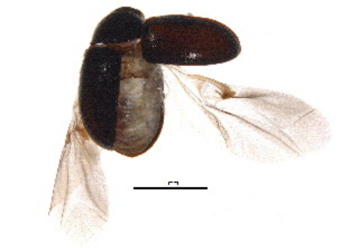

BIOUG20950-A11 [Dorsal]
Coleoptera

BIN URI: BOLD:ACV1604

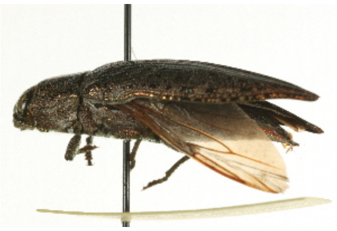

AY165645 [Lateral]
Dicerca divaricata
Family: Buprestidae
BIN URI: BOLD:AAC3543

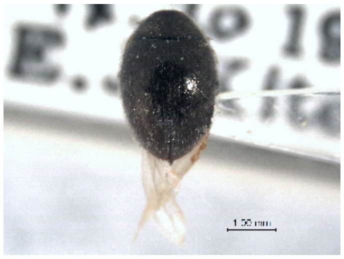

CNC COLEO 00159480 [Dorsal]
Simplocaria semistriata
Family: Byrrhidae
BIN URI: BOLD:ABW1696

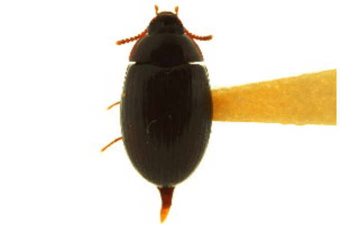

CNC COLEO 00124899 [Dorsal]
Platydema oregonense
Family: Tenebrionidae
BIN URI: BOLD:AAY9734

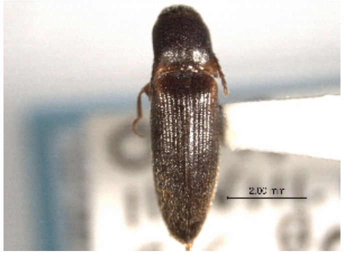

CNC COLEO 00158240 [Dorsal]
Dalopius vagus
Family: Elateridae
BIN URI: BOLD:AAH2416

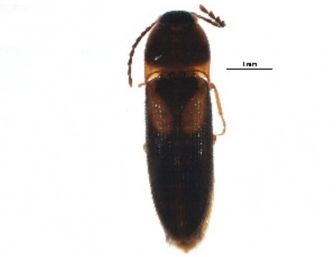

BIOUG22422-E08 [Dorsal]
Ampedus areolatus
Family: Elateridae
BIN URI: BOLD:ACM2015

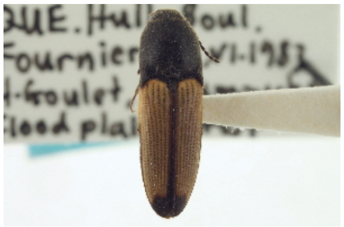

CNC COLEO 00157941 [Dorsal]
Ampedus linteus
Family: Elateridae
BIN URI: BOLD:AAU7141

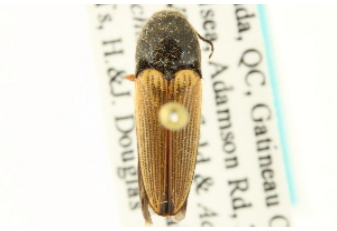

CNC COLEO 00158596 [Dorsal]
Ampedus nigricollis
Family: Elateridae
BIN URI: BOLD:AAH2376

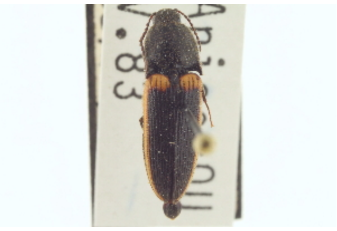

CNC COLEO 00157969 [Dorsal]
Ampedus oblessus
Family: Elateridae
BIN URI: BOLD:ACA3849

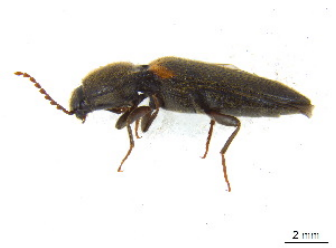

BIOUG06758-D10 [Lateral]
Ampedus protervus
Family: Elateridae
BIN URI: BOLD:ACR3975

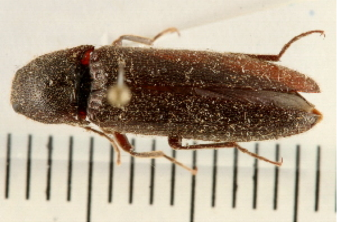

08BBCOL-0026 [Dorsal]
Melanotus castanipes
Family: Elateridae
BIN URI: BOLD:AAH2378

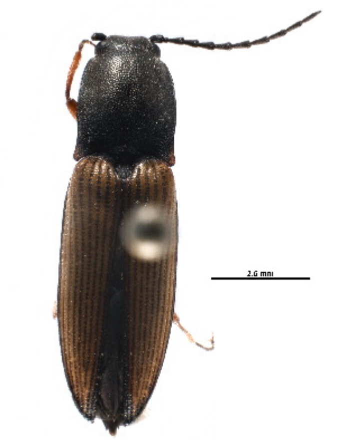

BIOUG22326-C09 [Dorsal]
Corymbitodes tarsalis
Family: Elateridae
BIN URI: BOLD:ACV5201

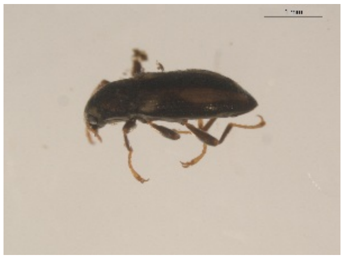

BIOUG01315-C05 [Lateral]
Dubiraphia sp. BOLDAAF6867
Family: Elmidae
BIN URI: BOLD:AAF6867

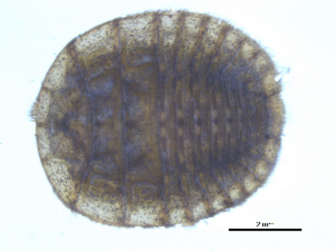

BIOUG06975-C12 [Dorsal]
Psephenus herricki
Family: Psephenidae
BIN URI: BOLD:AAB6713

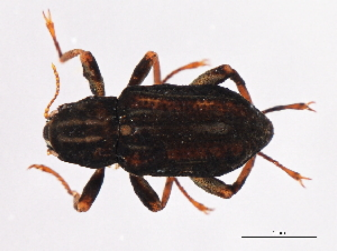

09BBECO-0512 [Dorsal]
Stenelmis crenata
Family: Elmidae
BIN URI: BOLD:AAC4909

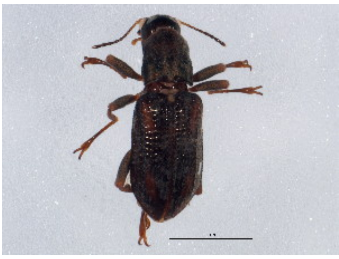

BIOUG02273-C09 [Dorsal]
Stenelmis
Family: Elmidae
BIN URI: BOLD:ABZ5521

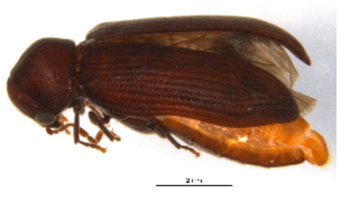

BIOUG07026-E02 [Lateral]
Ptinidae
Family: Ptinidae
BIN URI: BOLD:ACH2408

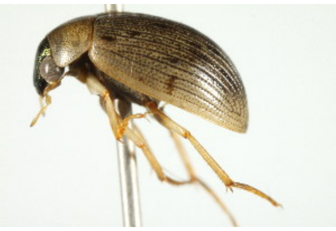

PCPP10-0060 [Lateral]
Berosus
Family: Hydrophilidae
BIN URI: BOLD:AAH0308

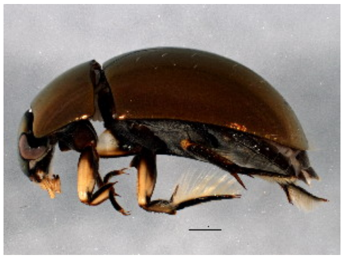

BIOUG02274-C04 [Lateral]
Hydrophilidae
Family: Hydrophilidae
BIN URI: BOLD:ABY1765

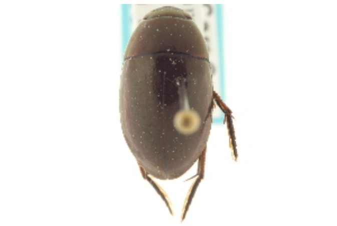

CNC COLEO 00150769 [Dorsal]
Tropisternus natator
Family: Hydrophilidae
BIN URI: BOLD:ACA3026

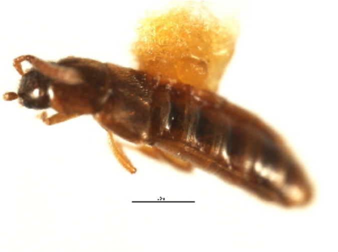

05-CTATBI-0642 [Dorsal]
Aleocharinae
Family: Staphylinidae
BIN URI: BOLD:AAH0119

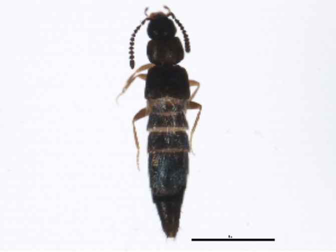

BIOUG00873-E10 [Dorsal]
Staphylinidae

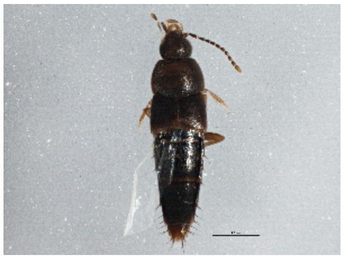

BIOUG08688-A05 [Dorsal]
Staphylinidae

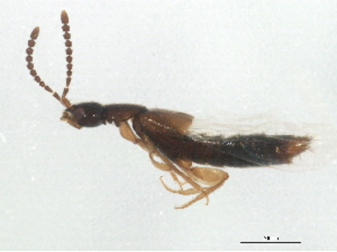

BIOUG01593-E05 [Lateral]
Atheta

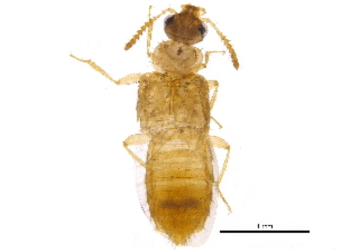

BIOUG22838-G10 [Dorsal]
Staphylinidae

Family: Staphylinidae
BIN URI: BOLD:AAU6968

Family: Staphylinidae
BIN URI: BOLD:ACJ6804

Family: Staphylinidae
BIN URI: BOLD:ABW2820

Family: Staphylinidae
BIN URI: BOLD:ACV6600

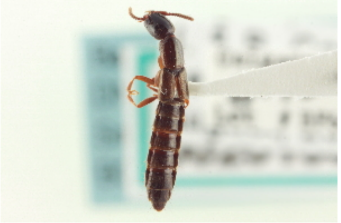

CNC COLEO 00251951 [Dorsal]
Xantholinus linearis
Family: Staphylinidae
BIN URI: BOLD:AAG4333

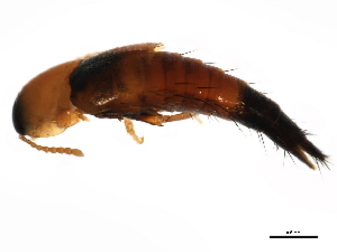

10PHMAL-1775 [Lateral]
Tachyporus elegans
Family: Staphylinidae
BIN URI: BOLD:AAU6934

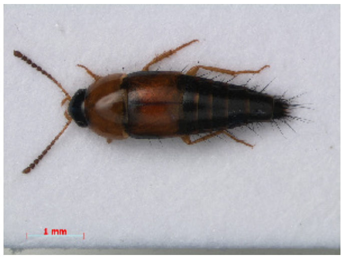

RMNH.INS.535739 [Dorsal]
Tachyporus dispar
Family: Staphylinidae
BIN URI: BOLD:AAN9511

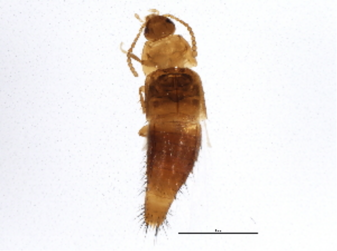

BIOUG03260-A02 [Dorsal]
Tachyporus nitidulus
Family: Staphylinidae
BIN URI: BOLD:ABA9096

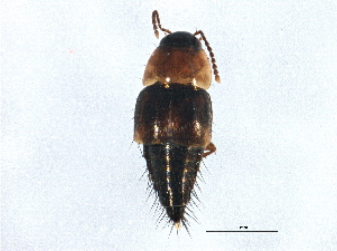

BIOUG01309-G02 [Dorsal]
Tachyporus atriceps
Family: Staphylinidae
BIN URI: BOLD:ABX2484

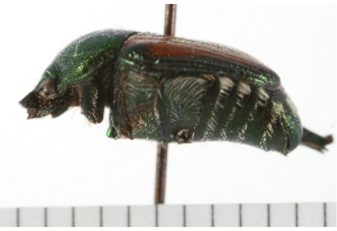

COLP 0085.02 [Lateral]
Popillia japonica
Family: Scarabaeidae
BIN URI: BOLD:AAF0418

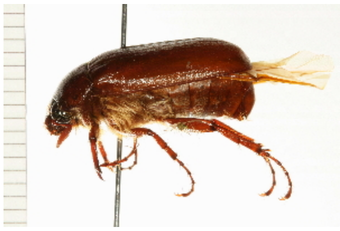

08OEC-054 [Lateral]
Phyllophaga futilis
Family: Scarabaeidae
BIN URI: BOLD:AAD1098

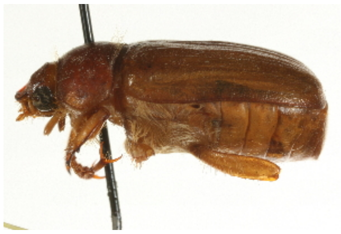

AY165642 [Lateral]
Phyllophaga rugosa
Family: Scarabaeidae
BIN URI: BOLD:AAJ2312

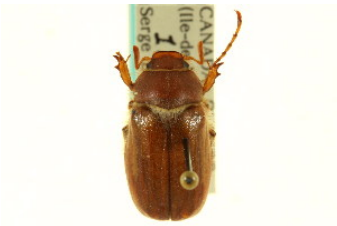

CNC COLEO 00094284 [Dorsal]
Amphimallon majale
Family: Scarabaeidae
BIN URI: BOLD:AAR3859

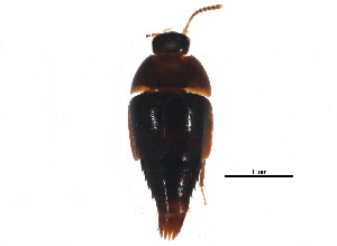

BIOUG06798-F07 [Dorsal]
Coproporus ventriculus
Family: Staphylinidae
BIN URI: BOLD:ACV1788

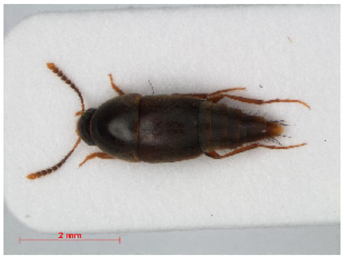

RMNH.INS.536208 [Dorsal]
Sepedophilus testaceus
Family: Staphylinidae
BIN URI: BOLD:AAH0108

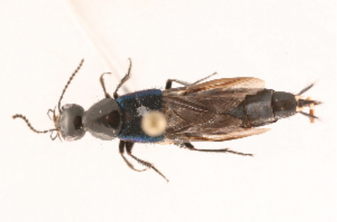

09BBECO-0215 [Dorsal]
Philonthus caeruleipennis
Family: Staphylinidae
BIN URI: BOLD:AAF6824

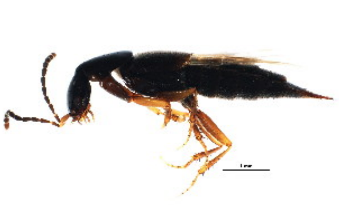

BIOUG00993-H01 [Lateral]
Philonthus flavibasis
Family: Staphylinidae
BIN URI: BOLD:AAH0113

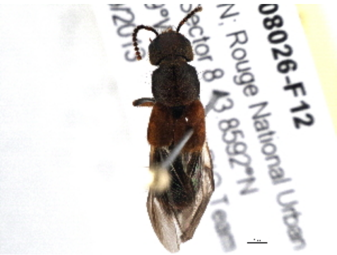

BIOUG08026-F12 [Dorsal]
Platydracus cinnamopterus
Family: Staphylinidae
BIN URI: BOLD:ACJ0017

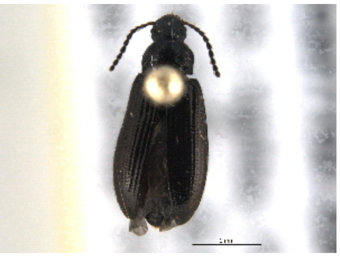

BIOUG07704-G08 [Dorsal]
Alleculinae
Family: Tenebrionidae
BIN URI: BOLD:AAY6541

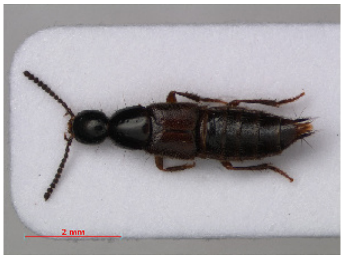

RMNH.INS.535853 [Dorsal]
Philonthus debilis
Family: Staphylinidae
BIN URI: BOLD:ABV1529

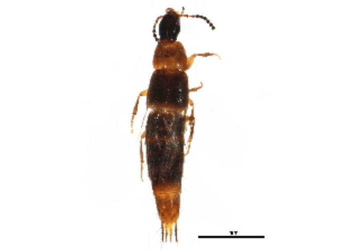

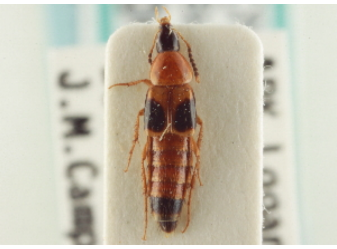

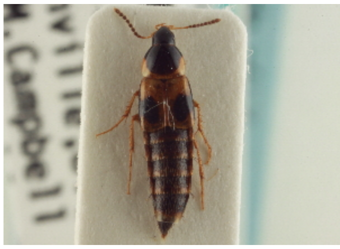

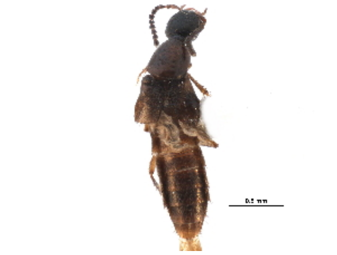

BIOUG22362-G06 [Dorsal]
Staphylinidae
Family: Staphylinidae
BIN URI: BOLD:ACV3870

CNC COLEO 00161500 [Dorsal]
Lordithon cinctus
Family: Staphylinidae
BIN URI: BOLD:ABA6370

CNC COLEO 00161517 [Dorsal]
Lordithon appalachianus
Family: Staphylinidae
BIN URI: BOLD:ABA6331

CCDB-23048-D11 [Dorsal]
Gabrius microphthalmus
Family: Staphylinidae
BIN URI: BOLD:ABW2822

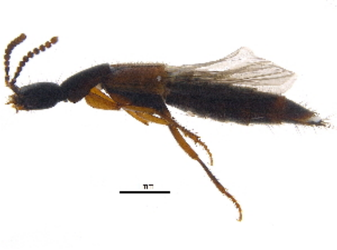

BIOUG22876-D02 [Lateral]
Staphylinidae
Family: Staphylinidae
BIN URI: BOLD:ACW0850

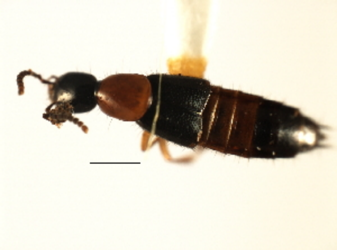

05-CTATBI-0650 [Dorsal]
Bisnius blandus
Family: Staphylinidae
BIN URI: BOLD:AAH0121

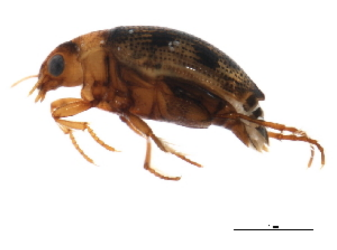

10PCCOL-0191 [Lateral]
Haliplus immaculicollis
Family: Haliplidae
BIN URI: BOLD:AAI6895

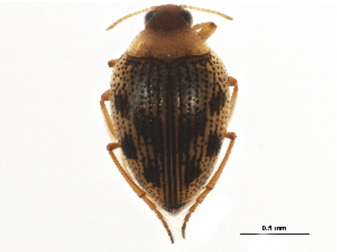

BIOUG24029-G01 [Dorsal]
Haliplus
Family: Haliplidae

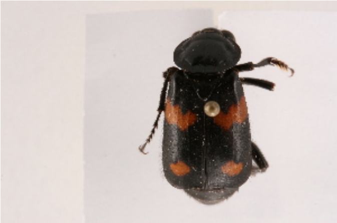

09BBCOL-0075 [Dorsal]
Nicrophorus orbicollis
Family: Silphidae
BIN URI: BOLD:AAE1939

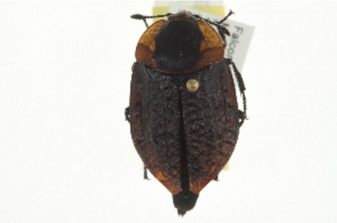

JBWM0211465 [Dorsal]
Necrophila americana
Family: Silphidae
BIN URI: BOLD:AAG2066

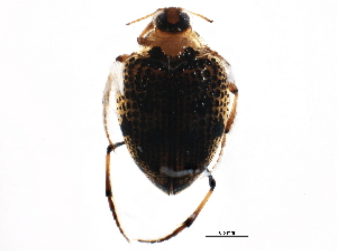

BIOUG21000-H01 [Lateral]
Coleoptera

BIN URI: BOLD:ACU8601

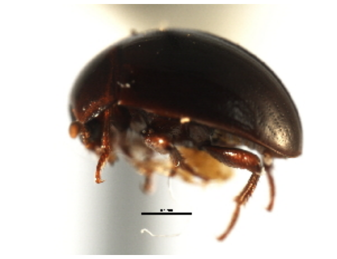

05-CTATBI-0652 [Lateral]
Anisotoma obsoleta
Family: Leiodidae
BIN URI: BOLD:AAR3435

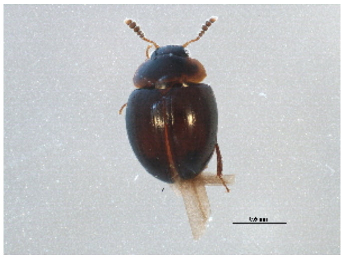

BIOUG10440-G02 [Dorsal]
Leiodinae
Family: Leiodidae
BIN URI: BOLD:ACL8841

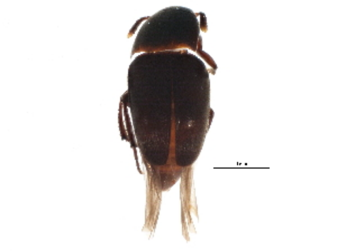

BIOUG15940-F07 [Dorsal]
Coleoptera

BIN URI: BOLD:ACQ7563

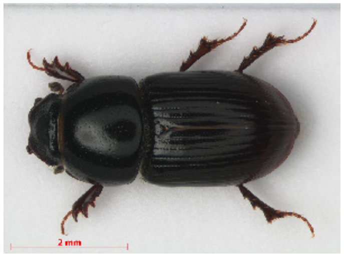

RMNH.INS.535945 [Dorsal]
Aphodius granarius
Family: Scarabaeidae
BIN URI: BOLD:AAM7733

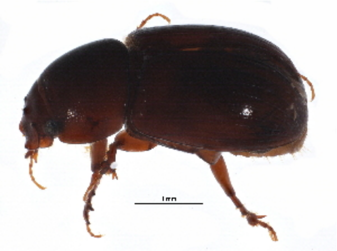

BIOUG24028-E01 [Lateral]
Aphodius granarius
Family: Scarabaeidae

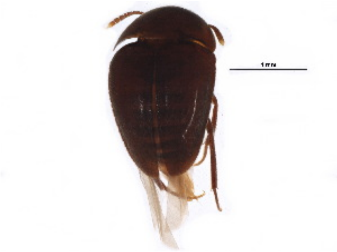

BIOUG22288-H03 [Dorsal]
Leiodidae
Family: Leiodidae
BIN URI: BOLD:ACI6050

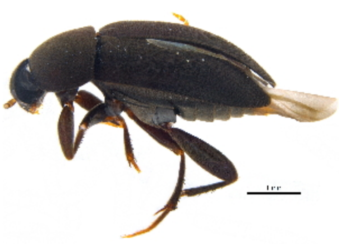

BIOUG22325-C01 [Lateral]
Prionochaeta opaca
Family: Leiodidae
BIN URI: BOLD:AAP6949

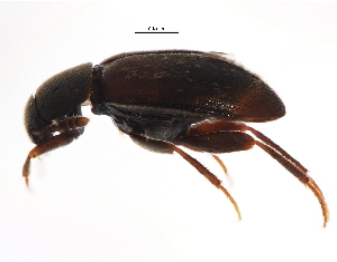

BIOUG00781-D12 [Lateral]
Catops paramericanus
Family: Leiodidae
BIN URI: BOLD:AAH3504

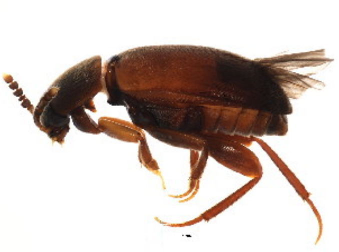

BIOUG00971-E05 [Lateral]
Catops
Family: Leiodidae
BIN URI: BOLD:ABW6289

IMAGE NOT AVAILABLE

BIOUG22867-B05

Cholevinae
Family: Leiodidae

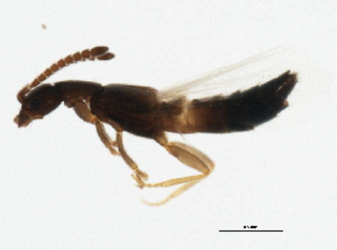

BIOUG00828-C04 [Lateral]
Staphylinidae
Family: Staphylinidae
BIN URI: BOLD:AAY6554

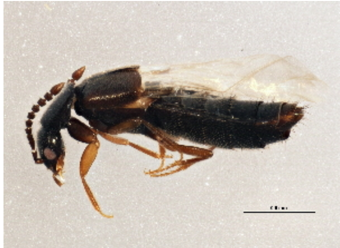

BIOUG03521-E06 [Lateral]
Atheta brunneipennis
Family: Staphylinidae
BIN URI: BOLD:ABA9094

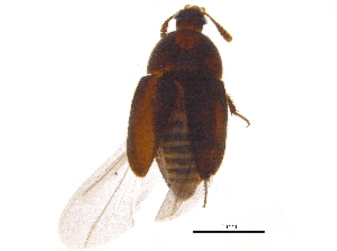

BIOUG22838-F08 [Dorsal]
Leiodidae
Family: Leiodidae
BIN URI: BOLD:ACV8702

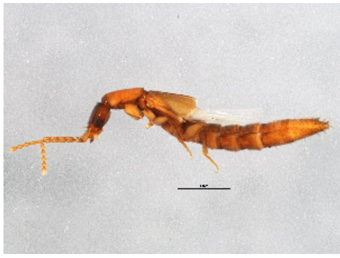

BIOUG02279-F12 [Lateral]
Coleoptera

BIN URI: BOLD:ABX3618

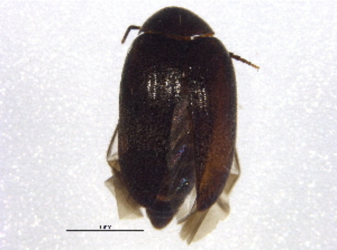

BIOUG07429-H04 [Dorsal]
Eucinetidae
Family: Eucinetidae
BIN URI: BOLD:ABW2834

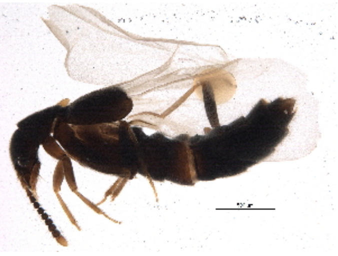

BIOUG10519-D02 [Lateral]
Staphylinidae
Family: Staphylinidae
BIN URI: BOLD:ACC7094

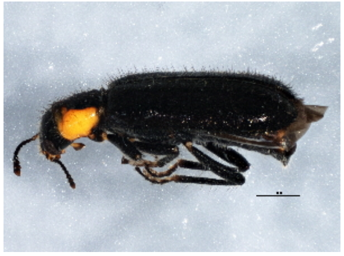

BIOUG02862-A12 [Lateral]
Placopterus thoracicus
Family: Cleridae
BIN URI: BOLD:AAP8584

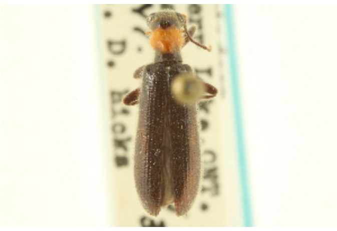

CNC COLEO 00154865 [Dorsal]
Cymatodera bicolor
Family: Cleridae
BIN URI: BOLD:AAU6910

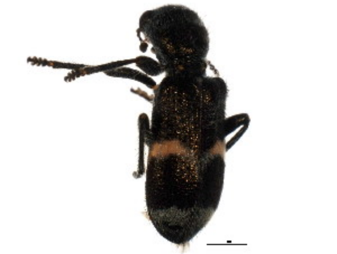

BIOUG00874-D07 [Dorsal]
Enoclerus nigripes rufiventris
Family: Cleridae
BIN URI: BOLD:AAU6970

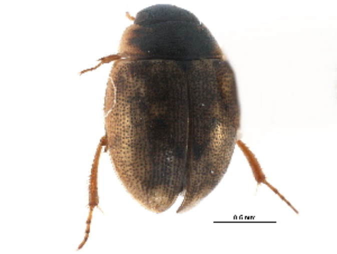

CCDB-23054-G09 [Dorsal]
Laccobius spangleri
Family: Hydrophilidae
BIN URI: BOLD:ACA3199

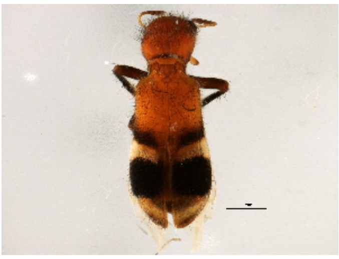

BIOUG08803-C10 [Dorsal]
Enoclerus
Family: Cleridae
BIN URI: BOLD:ACI9790

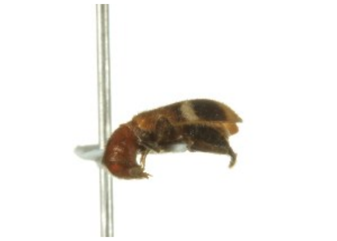

52-4-032 [Lateral]
Enoclerus nigripes dubius
Family: Cleridae
BIN URI: BOLD:ACK1620

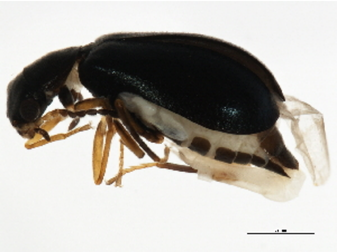

BIOUG00828-C07 [Lateral]
Hypebaeus apicalis
Family: Melyridae
BIN URI: BOLD:AAN5932

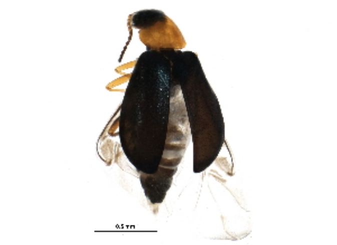

BIOUG21892-B09 [Dorsal]
Melyridae
Family: Melyridae
BIN URI: BOLD:ACV5054

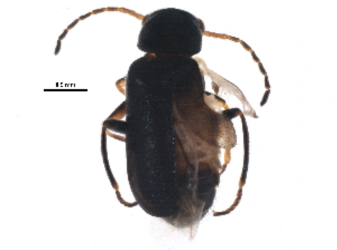

BIOUG24003-C06 [Dorsal]
Melyridae
Family: Melyridae

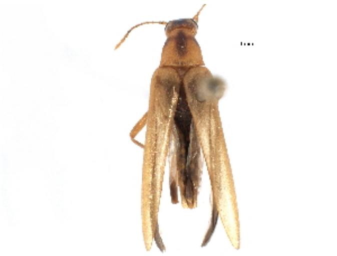

CCDB-22970-D03 [Dorsal]
Cephaloon lepturoides
Family: Stenotrachelidae
BIN URI: BOLD:AAI3796

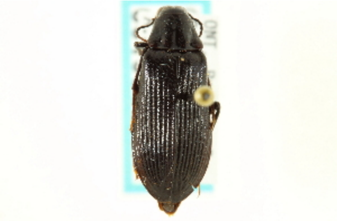

CNC COLEO 00160359 [Dorsal]
Melandrya striata
Family: Melandryidae
BIN URI: BOLD:AAK7242

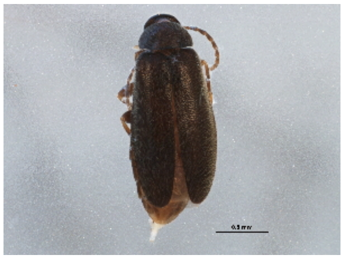

BIOUG11724-D09 [Dorsal]
Canifa
Family: Scraptiidae
BIN URI: BOLD:ACM5031

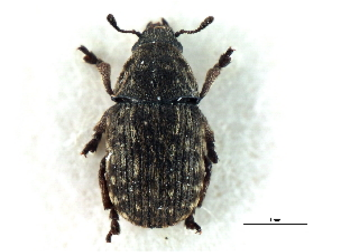

CNC COLEO 00109593 [Dorsal]
Anthribus nebulosus
Family: Anthribidae
BIN URI: BOLD:AAO1339

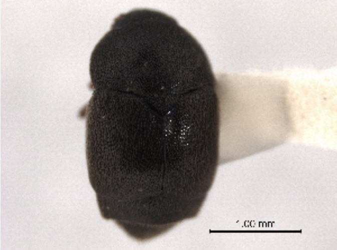

CNC COLEO 00159686 [Dorsal]
Brachypterolus pulicarius
Family: Kateretidae
BIN URI: BOLD:ABA9077

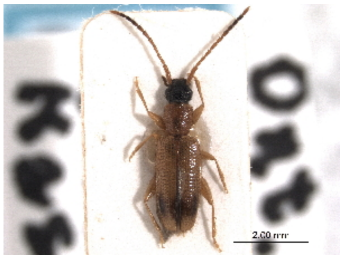

CNC COLEO 00159854 [Dorsal]
Telephanus velox
Family: Silvanidae
BIN URI: BOLD:AAW6380

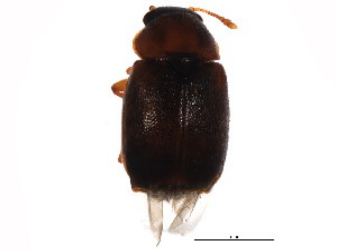

BIOUG00971-B05 [Dorsal]
Kateretidae
Family: Kateretidae
BIN URI: BOLD:ABA9071

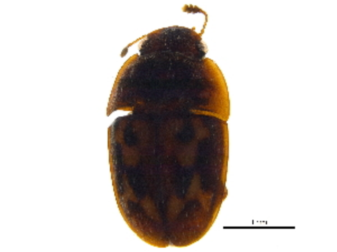

BIOUG22867-B01 [Dorsal]
Nitidulidae
Family: Nitidulidae
BIN URI: BOLD:ACV5474

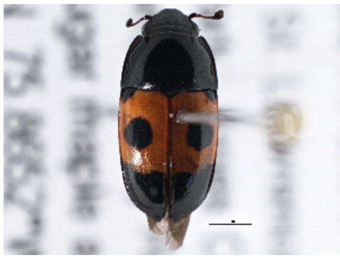

BIOUG06614-E04 [Dorsal]
Glischrochilus sanguinolentus sanguinolentus
Family: Nitidulidae
BIN URI: BOLD:AAP8585

IMAGE NOT AVAILABLE

BIOUG22324-H11
Glischrochilus fasciatus
Family: Nitidulidae

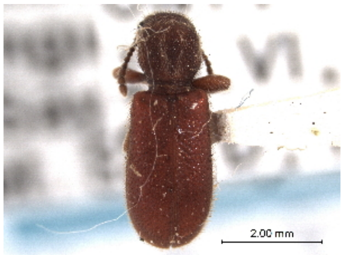

CNC COLEO 00154897 [Dorsal]
Zenodosus sanguineus
Family: Cleridae
BIN URI: BOLD:ABA6311

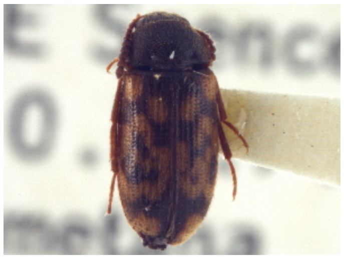

CNC COLEO 00160184 [Dorsal]
Mycetophagus pluripunctatus
Family: Mycetophagidae
BIN URI: BOLD:ACB0681

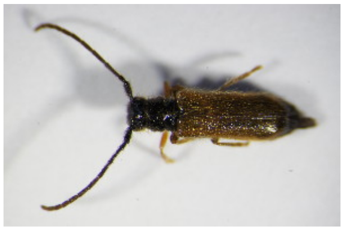

BFB_Col_FK_9283 [Dorsal]
Tetrops starkii
Family: Cerambycidae
BIN URI: BOLD:AAE9431

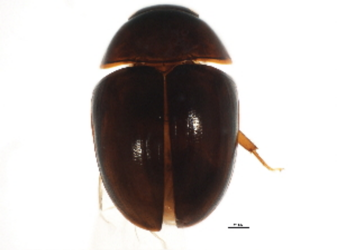

BIOUG05559-F08 [Dorsal]
Phalacridae
Family: Phalacridae
BIN URI: BOLD:ACF7672

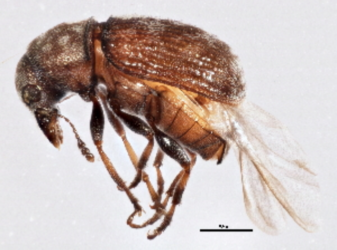

BIOUG02789-C05 [Dorsal]
Ormiscus walshii
Family: Anthribidae
BIN URI: BOLD:AAU7341

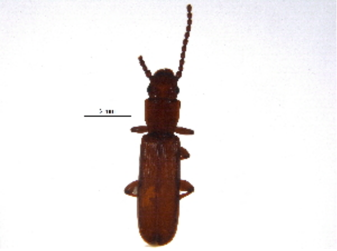

BIOUG09831-G12 [Dorsal]
Passandridae
Family: Passandridae
BIN URI: BOLD:ACL2308

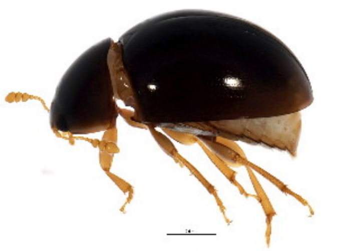

BIOUG00971-H09 [Lateral]
Phalacridae
Family: Phalacridae
BIN URI: BOLD:AAG4848

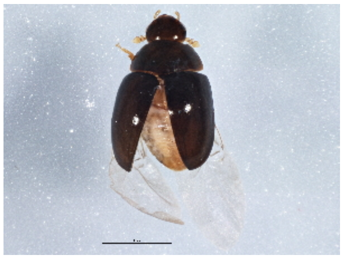

BIOUG07916-G06 [Dorsal]
Olibrus semistriatus
Family: Phalacridae
BIN URI: BOLD:AAK9463

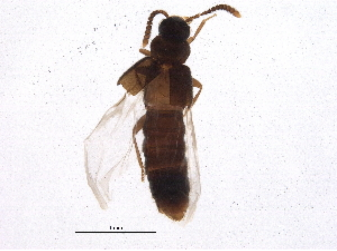

BIOUG09870-C08 [Dorsal]
Staphylinidae
Family: Staphylinidae
BIN URI: BOLD:ACD9884

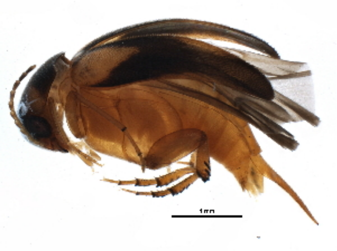

BIOUG08611-B06 [Lateral]
Mordellistena
Family: Mordellidae
BIN URI: BOLD:ACK1250

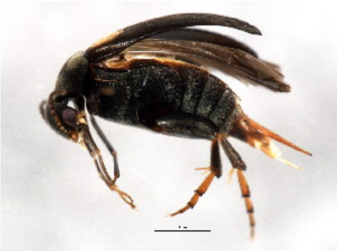

BIOUG00818-E09 [Lateral]
Mordellidae
Family: Mordellidae
BIN URI: BOLD:AAU6912

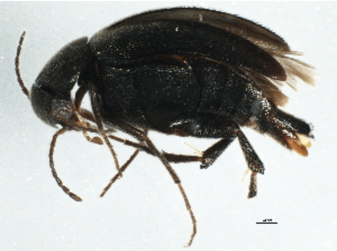

BIOUG03515-G05 [Lateral]
Mordellistena
Family: Mordellidae
BIN URI: BOLD:AAH0125

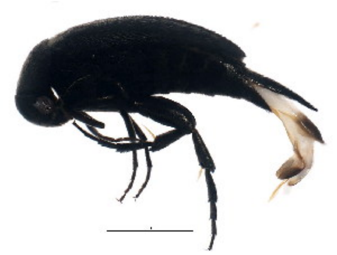

BIOUG00971-E02 [Lateral]
Mordellistena
Family: Mordellidae
BIN URI: BOLD:ABA9087

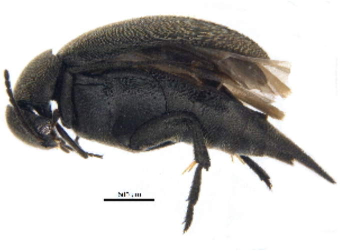

BIOUG22927-D02 [Lateral]
Mordellidae
Family: Mordellidae
BIN URI: BOLD:ABA5303

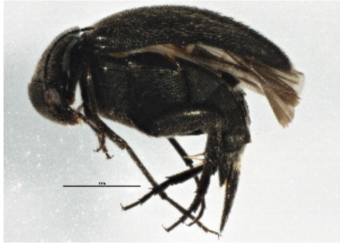

BIOUG09009-A02 [Lateral]
Mordellidae
Family: Mordellidae
BIN URI: BOLD:ACK6134

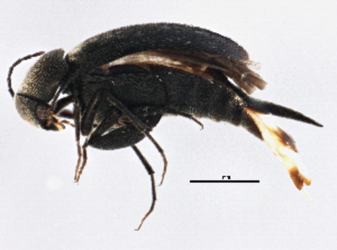

BIOUG03065-H10 [Lateral]
Mordellistena
Family: Mordellidae
BIN URI: BOLD:AAU7291

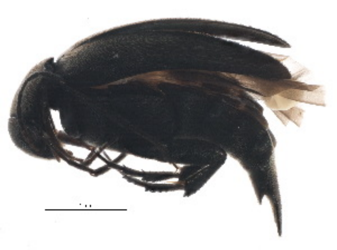

BIOUG21328-C09 [Lateral]
Coleoptera

BIN URI: BOLD:ACU9444

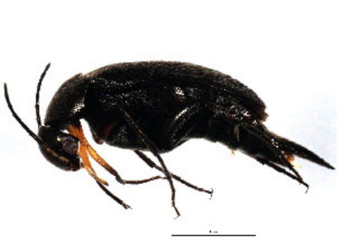

BIOUG00971-F12 [Lateral]
Mordellistena
Family: Mordellidae
BIN URI: BOLD:ABA9078

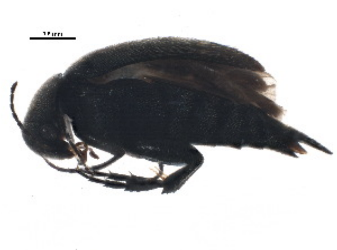

BIOUG22729-A06 [Lateral]
Mordellidae
Family: Mordellidae
BIN URI: BOLD:ACV5549

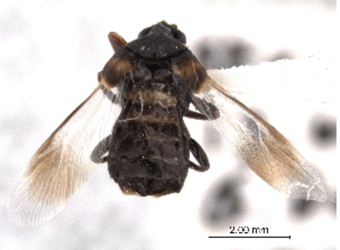

CNC COLEO 00160522 [Dorsal]
Ripiphorus fasciatus
Family: Ripiphoridae
BIN URI: BOLD:ABA6346

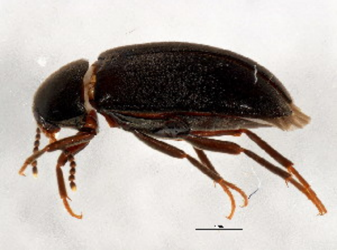

BIOUG00818-H11 [Lateral]
Mallodrya subaenea
Family: Synchroidae
BIN URI: BOLD:AAK7440

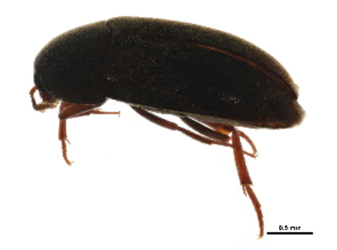

BIOUG22289-B10 [Lateral]
Eustrophus tomentosus
Family: Tetratomidae
BIN URI: BOLD:ACI7017

IMAGE NOT AVAILABLE

BIOUG22729-C02
Mycetophagidae
Family: Mycetophagidae

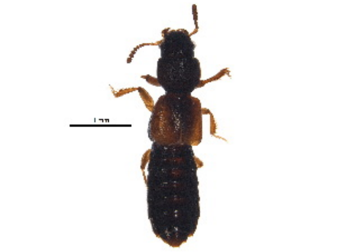

BIOUG20566-C10 [Dorsal]
Staphylinidae
Family: Staphylinidae
BIN URI: BOLD:ACV4933

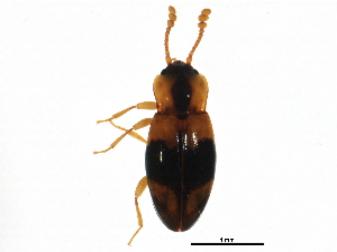

BIOUG22361-F04 [Dorsal]
Phymaphora pulchella
Family: Endomychidae
BIN URI: BOLD:ACI7114

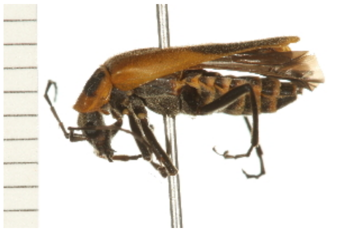

COLP 0183.02 [Lateral]
Chauliognathus pensylvanicus
Family: Cantharidae
BIN URI: BOLD:AAH0916

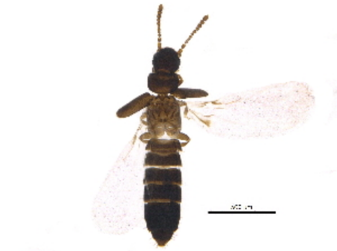

BIOUG22867-A08 [Dorsal]
Staphylinidae
Family: Staphylinidae
BIN URI: BOLD:ACV4901

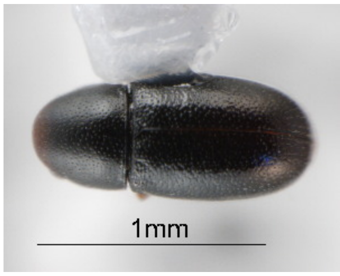

CNCCOLVG00001304 [Dorsal]
Ceracis thoracicornis
Family: Ciidae
BIN URI: BOLD:AAK0893

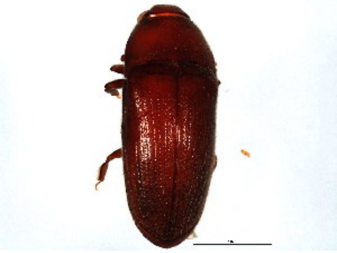

BIOUG00971-C12 [Dorsal]
Aulonothroscus distans
Family: Throscidae
BIN URI: BOLD:ABA9083

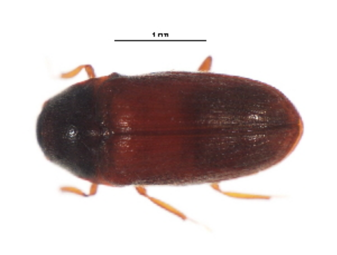

BIOUG00781-E04 [Dorsal]
Coleoptera

BIN URI: BOLD:AAU7339

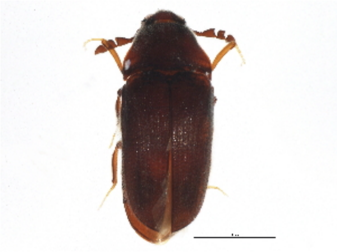

BIOUG05507-E08 [Dorsal]
Trixagus carinicollis
Family: Throscidae
BIN URI: BOLD:AAN6148

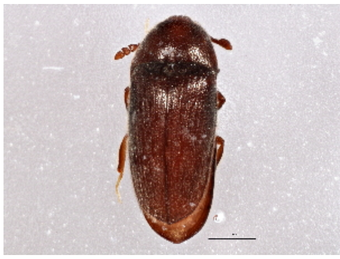

BIOUG02832-H01 [Dorsal]
Trixagus
Family: Throscidae
BIN URI: BOLD:AAU6966

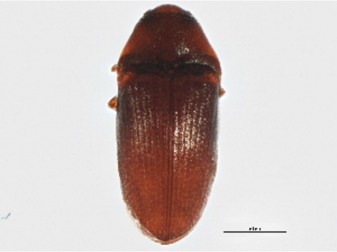

BIOUG01649-A09 [Dorsal]
Trixagus
Family: Throscidae
BIN URI: BOLD:ABW2869

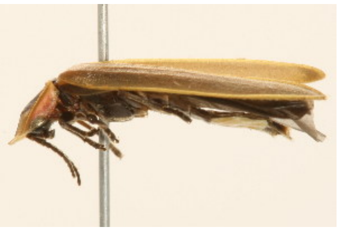

09BBCOL-0687 [Lateral]
Pyractomena
Family: Lampyridae
BIN URI: BOLD:AAH6661

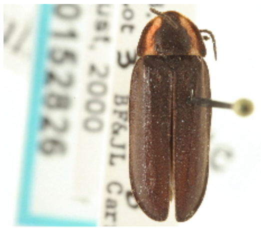

CNC COLEO 00152826 [Lateral]
Ellychnia facula
Family: Lampyridae
BIN URI: BOLD:ACV4844

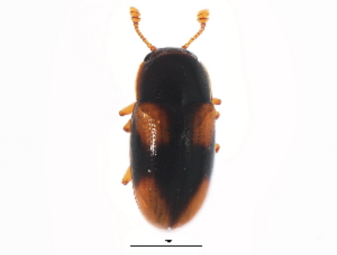

BIOUG00781-H03 [Dorsal]
Coleoptera

BIN URI: BOLD:AAU7330

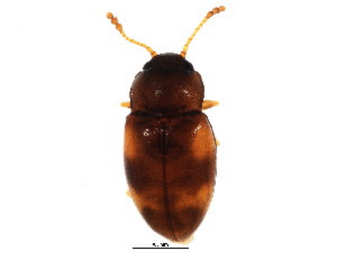

BIOUG00993-F07 [Dorsal]
Erotylidae
Family: Erotylidae
BIN URI: BOLD:ABA6320

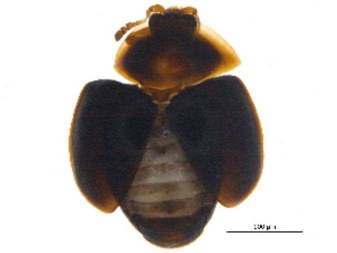

BIOUG21887-G10 [Dorsal]
Corylophidae
Family: Corylophidae
BIN URI: BOLD:ACJ0061

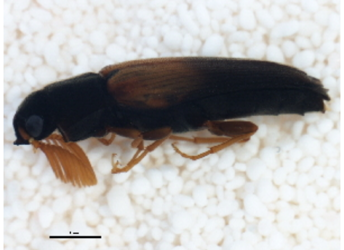

BIOUG00824-B08 [Lateral]
Eucnemidae
Family: Eucnemidae
BIN URI: BOLD:AAY6530

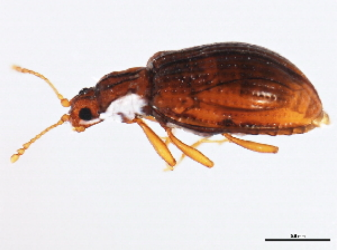

10PCCOL-0092 [Lateral]
Cartodere
Family: Latridiidae
BIN URI: BOLD:ABA9100

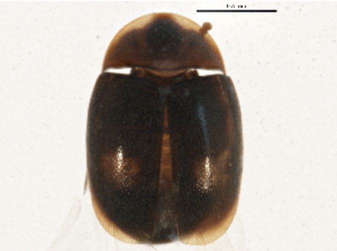

BIOUG05539-D01 [Lateral]
Clypastraea
Family: Corylophidae
BIN URI: BOLD:AAN6155

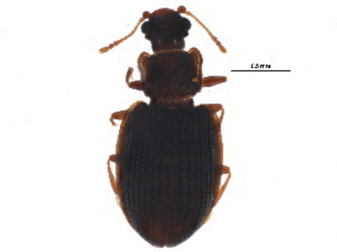

BIOUG22729-B09 [Dorsal]
Latridiidae
Family: Latridiidae
BIN URI: BOLD:ACV4877

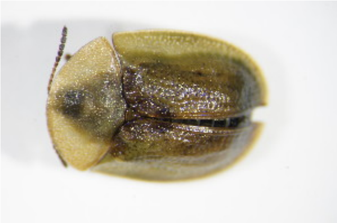

GBOL_Col_FK_2792 [Dorsal]
Cassida rubiginosa
Family: Chrysomelidae
BIN URI: BOLD:AAO0522

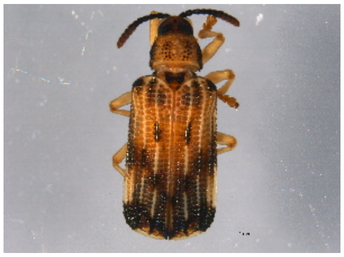

BIOUG07914-G12 [Dorsal]
Anisostena
Family: Chrysomelidae
BIN URI: BOLD:AAY6537

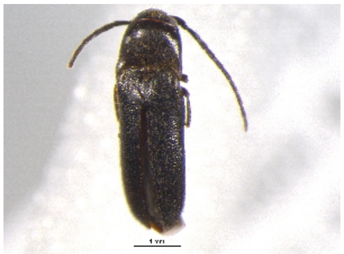

BIOUG02947-A05 [Dorsal]
Eucnemidae
Family: Eucnemidae
BIN URI: BOLD:ACA7831

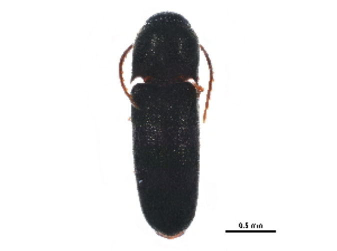

BIOUG23320-B07 [Dorsal]
Eucnemidae
Family: Eucnemidae
BIN URI: BOLD:ACW1152

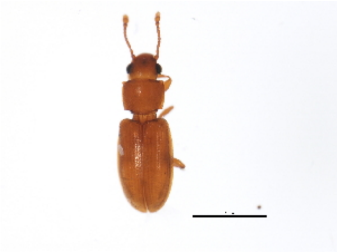

BIOUG00873-F03 [Dorsal]
Ahasverus advena
Family: Silvanidae
BIN URI: BOLD:AAJ2005

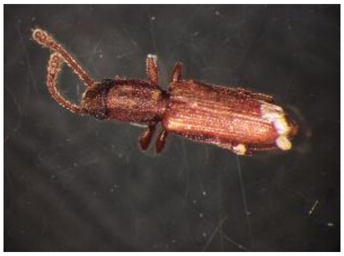

GBOL_Col_FK_5621 [Dorsal]
Silvanus bidentatus
Family: Silvanidae
BIN URI: BOLD:AAO0157

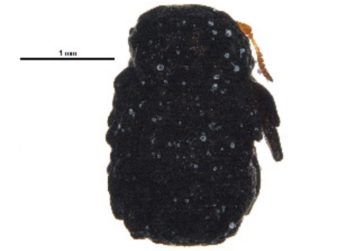

BIOUG24020-B02 [Dorsal]
Exema canadensis
Family: Chrysomelidae

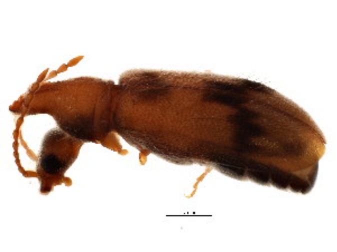

BIOUG02281-G07 [Lateral]
Anthicidae
Family: Anthicidae
BIN URI: BOLD:ABX0657

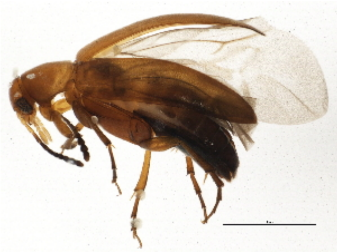

BIOUG03260-A01 [Lateral]
Anaspis rufa
Family: Scraptiidae
BIN URI: BOLD:AAH0469

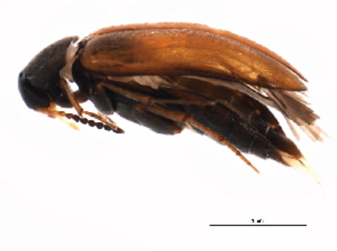

10PCCOL-0427 [Lateral]
Scraptiidae
Family: Scraptiidae
BIN URI: BOLD:AAP7841

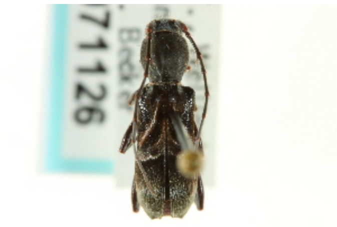

CNC COLEO 00071126 [Dorsal]
Cyrtophorus verrucosus
Family: Cerambycidae
BIN URI: BOLD:AAD4513

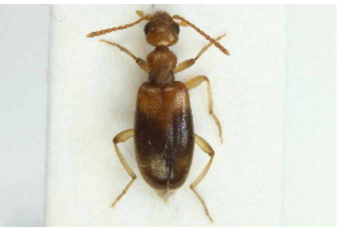

ZMUO.000427 [Dorsal]
Stricticollis tobias
Family: Anthicidae
BIN URI: BOLD:AAQ1028

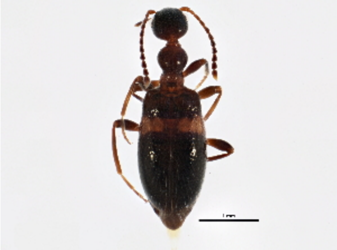

BIOUG05529-G04 [Dorsal]
Malporus formicarius
Family: Anthicidae
BIN URI: BOLD:ACF8552

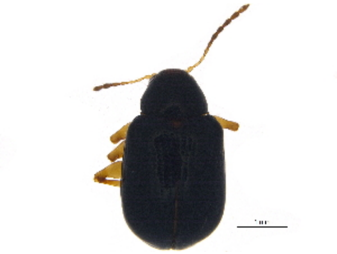

BIOUG22325-B11 [Dorsal]
Paria fragariae
Family: Chrysomelidae
BIN URI: BOLD:ABA3960

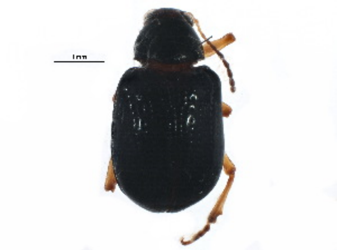

BIOUG22422-F01 [Dorsal]
Paria fragariae
Family: Chrysomelidae
BIN URI: BOLD:ACF6671

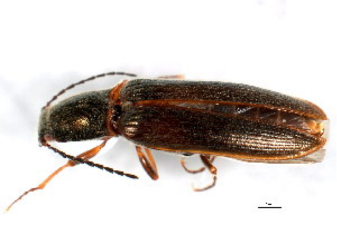

BIOUG00818-F09 [Dorsal]
Ctenicera cylindriformis
Family: Elateridae
BIN URI: BOLD:AAH2370

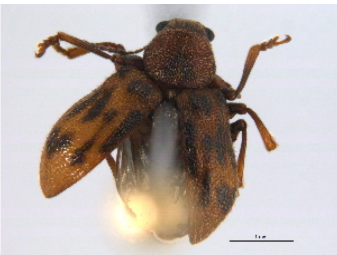

BIOUG08060-G03 [Dorsal]
Xanthonia decemnotata
Family: Chrysomelidae
BIN URI: BOLD:ACJ0239

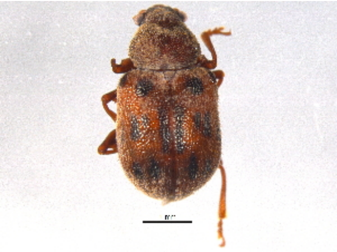

BIOUG05721-C01 [Dorsal]
Xanthonia decemnotata
Family: Chrysomelidae
BIN URI: BOLD:ABA6335

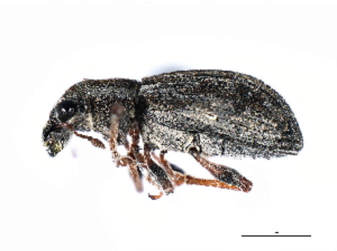

08BBCOL-0798 [Lateral]
Sitona lineellus
Family: Curculionidae
BIN URI: BOLD:AAH0257

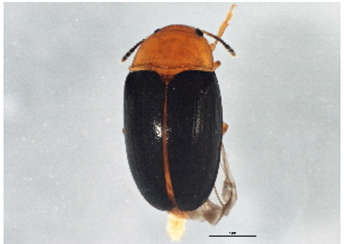

BIOUG07122-D11 [Dorsal]
Triplax thoracica
Family: Erotylidae
BIN URI: BOLD:ACI9012

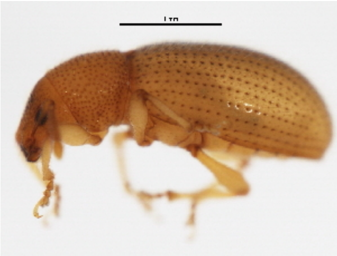

BIOUG00781-E12 [Lateral]
Barypeithes pellucidus
Family: Curculionidae
BIN URI: BOLD:AAG5192

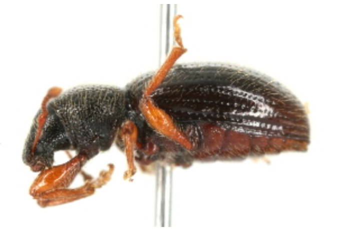

09BBECO-0236 [Lateral]
Barypeithes pellucidus
Family: Curculionidae
BIN URI: BOLD:AAG5242

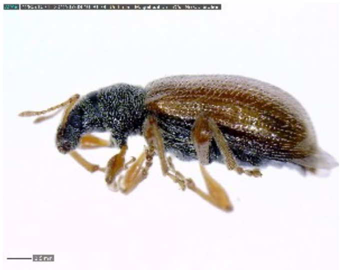

BIOUG02612-B03 [Lateral]
Phyllobius oblongus
Family: Curculionidae
BIN URI: BOLD:AAF9187

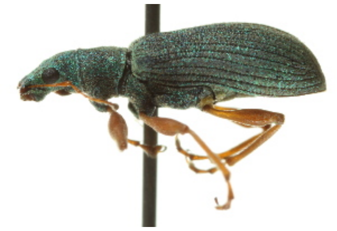

10PHMAL-2451 [Lateral]
Polydrusus impressifrons
Family: Curculionidae
BIN URI: BOLD:AAO4332

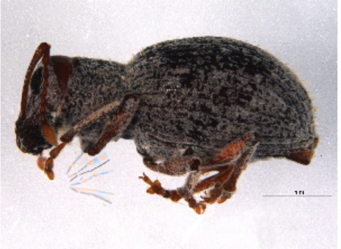

BIOUG08040-E02 [Lateral]
Curculionidae
Family: Curculionidae
BIN URI: BOLD:AAU7125

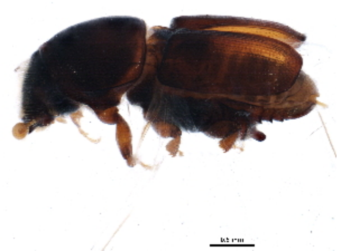

BIOUG16047-B07 [Lateral]
Scolytus
Family: Curculionidae
BIN URI: BOLD:AAD1195

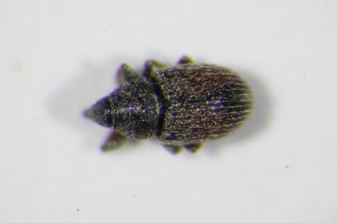

GBOL_Col_FK_2585 [Dorsal]
Gymnetron pascuorum
Family: Curculionidae
BIN URI: BOLD:AAO0328

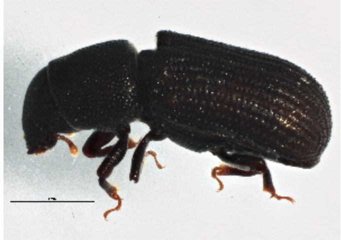

BIOUG05829-D07 [Lateral]
Curculionidae
Family: Curculionidae
BIN URI: BOLD:ACA5840

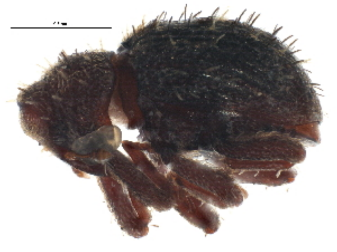

BIOUG16340-D11 [Lateral]
Coleoptera

BIN URI: BOLD:ACQ9042

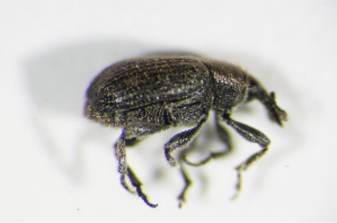

GBOL_Col_FK_4611 [Dorsal]
Gymnetron antirrhini
Family: Curculionidae
BIN URI: BOLD:ACE2245

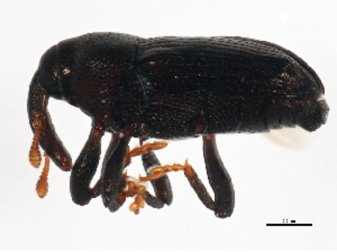

10PHMAL-1755 [Lateral]
Curculionidae
Family: Curculionidae
BIN URI: BOLD:AAU6928

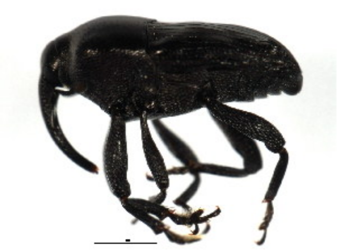

BIOUG00874-E06 [Lateral]
Madarellus undulatus
Family: Curculionidae
BIN URI: BOLD:AAY6533

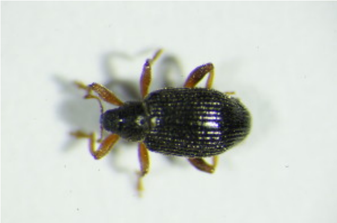

GBOL03301 [Lateral]
Isochnus
Family: Curculionidae
BIN URI: BOLD:ACA3052

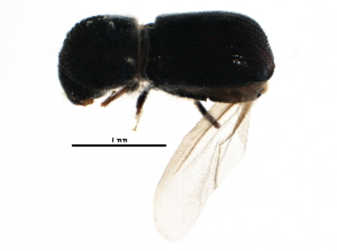

BIOUG21769-C12 [Lateral]
Xyleborus dispar
Family: Curculionidae
BIN URI: BOLD:AAD0158

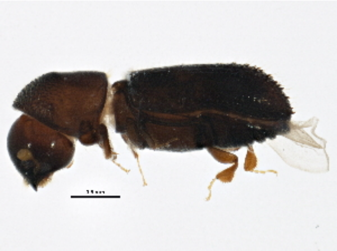

BIOUG05510-A03 [Lateral]
Xyleborinus alni
Family: Curculionidae
BIN URI: BOLD:AAB2754

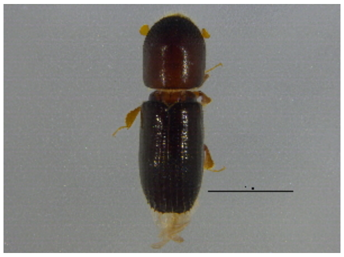

BIOUG02856-E12 [Dorsal]
Xyleborinus saxeseni
Family: Curculionidae
BIN URI: BOLD:AAB9578

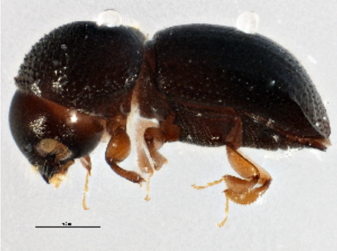

BIOUG03065-F12 [Dorsal]
Xylosandrus germanus
Family: Curculionidae
BIN URI: BOLD:AAF7523

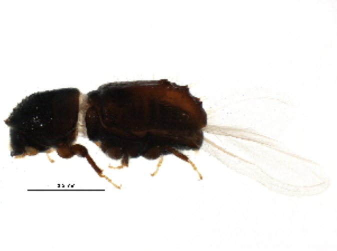

BIOUG21769-A01 [Lateral]
Pityogenes hopkinsi
Family: Curculionidae
BIN URI: BOLD:ABW5076

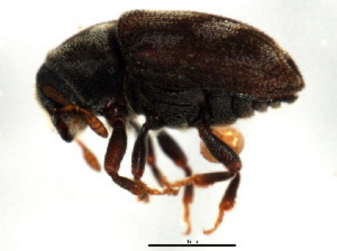

BIOUG00874-D11 [Lateral]
Hylesinus aculeatus
Family: Curculionidae
BIN URI: BOLD:AAU7331

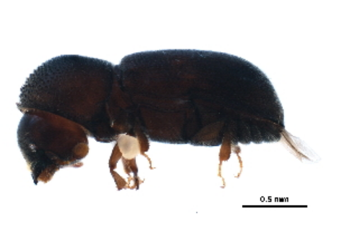

BIOUG22354-D07 [Lateral]
Curculionidae
Family: Curculionidae
BIN URI: BOLD:ACK1847

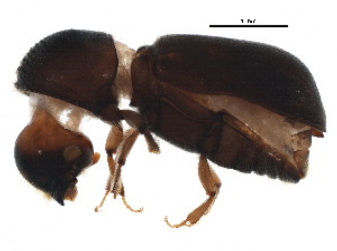

BIOUG22422-C05 [Lateral]
Curculionidae
Family: Curculionidae
BIN URI: BOLD:ACV3407

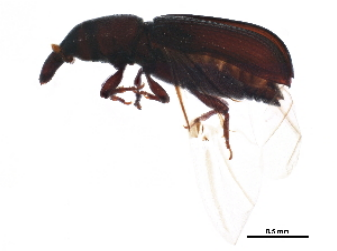

BIOUG23320-C10 [Lateral]
Curculionidae
Family: Curculionidae
BIN URI: BOLD:ACW1207

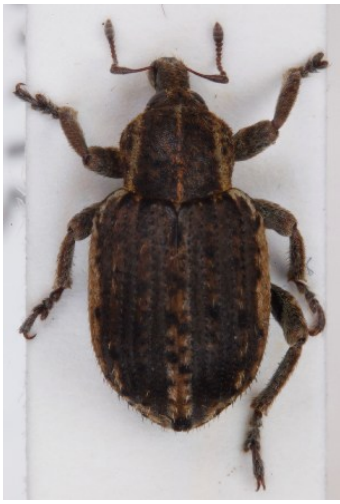

ZMUO.010653 [Dorsal]
Hypera zoilus
Family: Curculionidae
BIN URI: BOLD:ABA6345

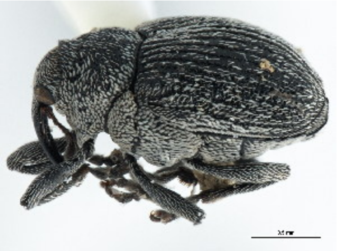

CNC COLEO 00126878 [Lateral]
Ceutorhynchus typhae
Family: Curculionidae
BIN URI: BOLD:AAN9807

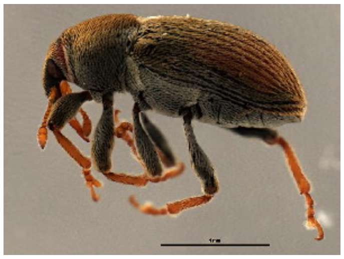

BIOUG00669-E09 [Lateral]
Tychius meliloti
Family: Curculionidae
BIN URI: BOLD:AAM7740

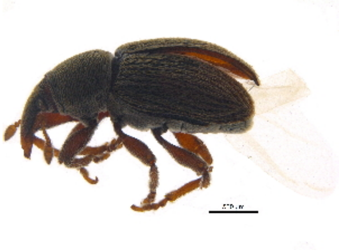

BIOUG22578-B09 [Lateral]
Curculionidae
Family: Curculionidae
BIN URI: BOLD:ACV3774

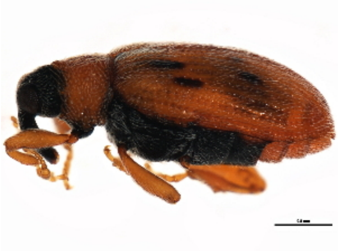

10BBCOL-0013 [Lateral]
Curculionidae
Family: Curculionidae
BIN URI: BOLD:AAM7726

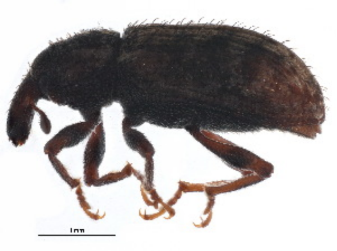

BIOUG22580-F02 [Lateral]
Curculionidae
Family: Curculionidae
BIN URI: BOLD:ABW8698

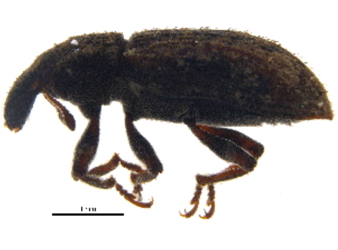

BIOUG21883-E05 [Lateral]
Curculionidae
Family: Curculionidae
BIN URI: BOLD:ACJ0010

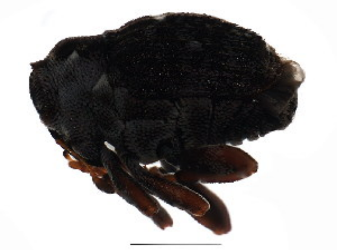

BIOUG00874-F08 [Lateral]
Curculionidae
Family: Curculionidae
BIN URI: BOLD:ABA6338

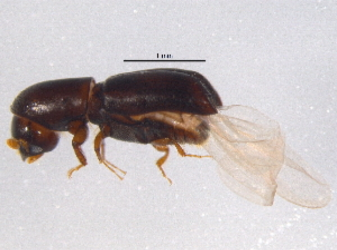

BIOUG05699-H07 [Lateral]
Monarthrum mali
Family: Curculionidae
BIN URI: BOLD:ACD0202

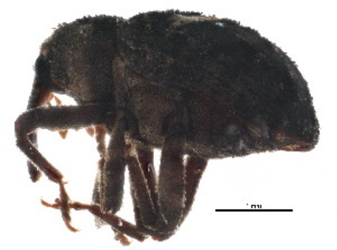

BIOUG22354-C08 [Lateral]
Curculionidae
Family: Curculionidae
BIN URI: BOLD:ACV4031

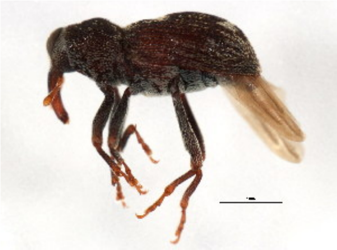

BIOUG00818-B01 [Lateral]
Acoptus suturalis
Family: Curculionidae
BIN URI: BOLD:AAU6930

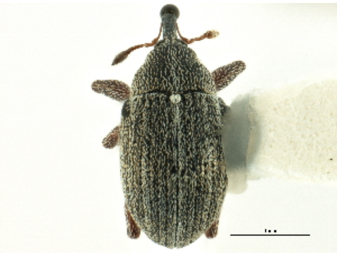

CCDB-22983-A02 [Dorsal]
Anthonomus
Family: Curculionidae
BIN URI: BOLD:AAZ2616

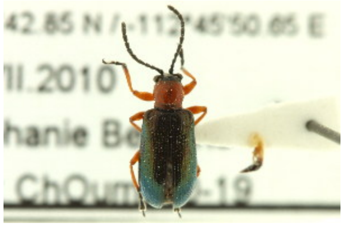

CNC COLEO 00123332 [Dorsal]
Oulema duftschmidi
Family: Chrysomelidae
BIN URI: BOLD:AAK5928

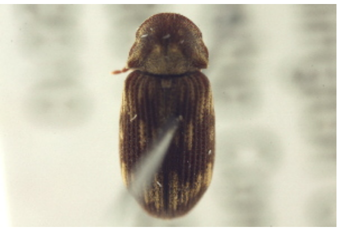

10PHMAL-2424 [Dorsal]
Hadrobregmus notatus
Family: Ptinidae
BIN URI: BOLD:AAP8586

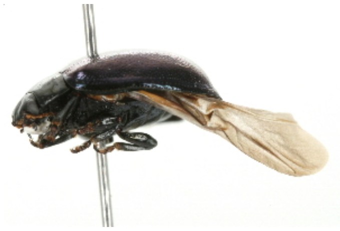

09BBECO-0271 [Lateral]
Plagiodera versicolora
Family: Chrysomelidae
BIN URI: BOLD:AAG4477

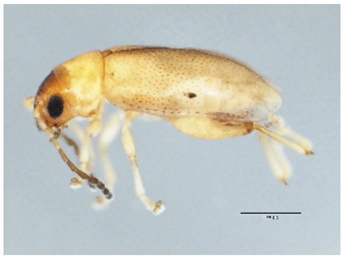

09BBECO-0399 [Lateral]
Longitarsus testaceus
Family: Chrysomelidae
BIN URI: BOLD:AAP8332

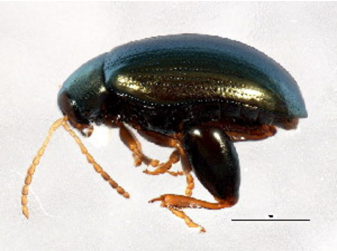

BIOUG00818-E08 [Lateral]
Dibolia borealis
Family: Chrysomelidae
BIN URI: BOLD:AAL0908

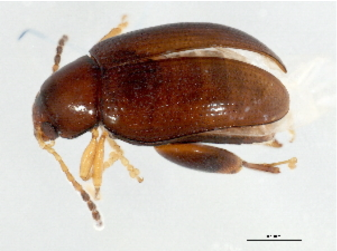

BIOUG01593-A01 [Lateral]
Psylliodes picinus
Family: Chrysomelidae
BIN URI: BOLD:AAP8162

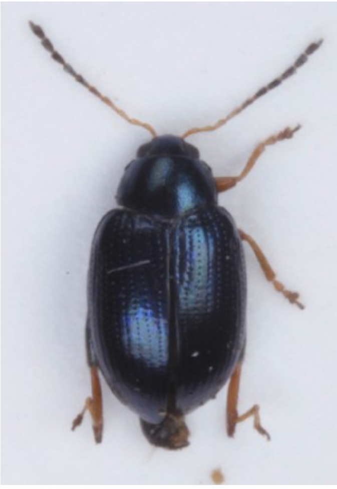

ZMUO.007494 [Dorsal]
Psylliodes napi
Family: Chrysomelidae
BIN URI: BOLD:ABA4532

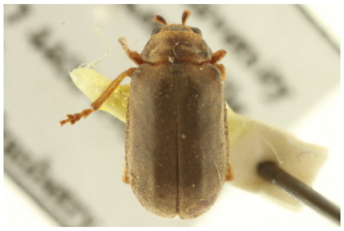

CNC COLEO 00151503 [Dorsal]
Pyrrhalta viburni
Family: Chrysomelidae
BIN URI: BOLD:AAL1083

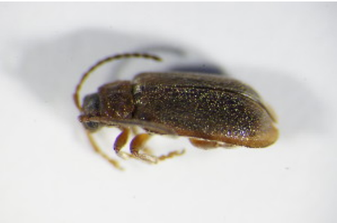

BFB_Col_FK_9282 [Dorsal]
Neogalerucella pusilla
Family: Chrysomelidae
BIN URI: BOLD:AAL2945

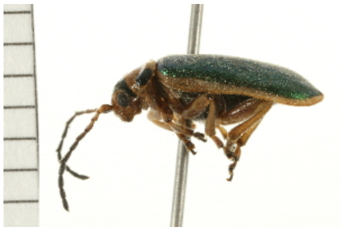

08BBCOL-0455 [Lateral]
Trirhabda borealis
Family: Chrysomelidae
BIN URI: BOLD:AAG4458

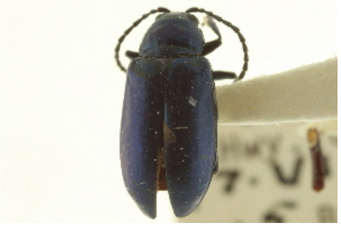

CNC COLEO 00151711 [Dorsal]
Altica subplicata
Family: Chrysomelidae
BIN URI: BOLD:AAG4462

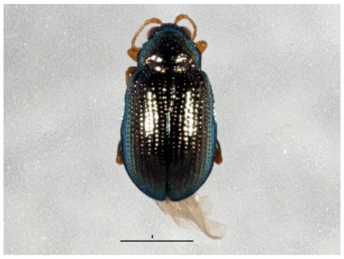

BIOUG01641-A09 [Dorsal]
Crepidodera
Family: Chrysomelidae
BIN URI: BOLD:ABA9095

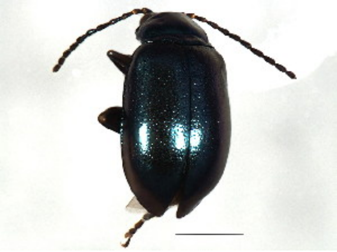

BIOUG00874-B12 [Dorsal]
Altica chalybea
Family: Chrysomelidae
BIN URI: BOLD:AAH0273

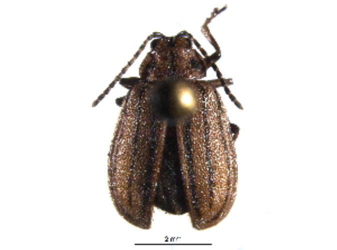

BIOUG05513-F07 [Dorsal]
Ophraella conferta
Family: Chrysomelidae
BIN URI: BOLD:ACF8270

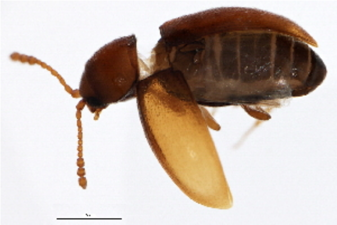

PROBE-TW0487 [Dorsal]
Atomaria CHU2
Family: Cryptophagidae
BIN URI: BOLD:AAG5060

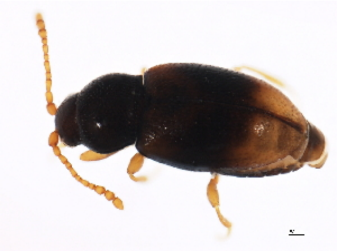

10PCCOL-0069 [Dorsal]
Atomaria ephippiata
Family: Cryptophagidae
BIN URI: BOLD:AAP7030

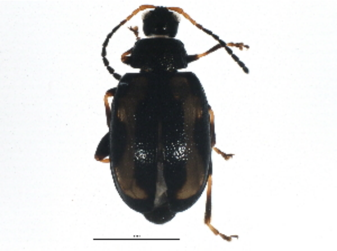

BIOUG05520-C10 [Dorsal]
Phyllotreta striolata
Family: Chrysomelidae
BIN URI: BOLD:AAL5267

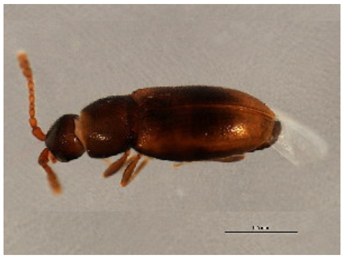

BIOUG00669-B08 [Dorsal]
Atomaria
Family: Cryptophagidae
BIN URI: BOLD:AAU7170

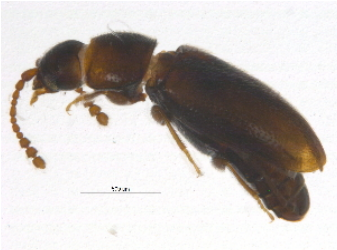

BIOUG05032-A03 [Lateral]
Atomaria
Family: Cryptophagidae
BIN URI: BOLD:ACG3394

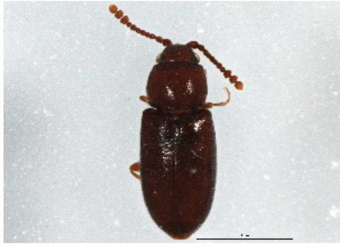

BIOUG06180-H01 [Dorsal]
Atomaria
Family: Cryptophagidae
BIN URI: BOLD:ACI5062

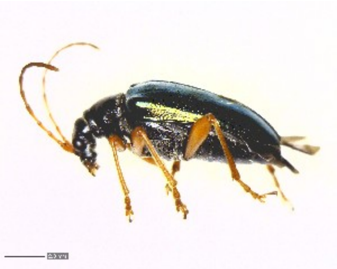

BIOUG06758-H09 [Lateral]
Gaurotes cyanipennis
Family: Cerambycidae
BIN URI: BOLD:AAI7042

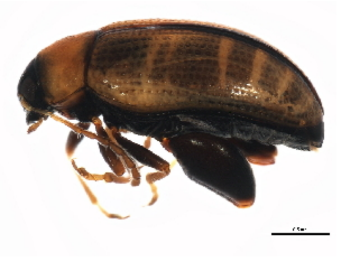

10PHMAL-0281 [Lateral]
Psylliodes affinis
Family: Chrysomelidae
BIN URI: BOLD:AAU6967

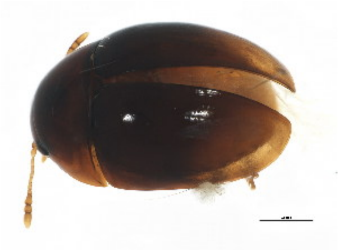

BIOUG02321-H08 [Lateral]
Stilbus apicialis
Family: Phalacridae
BIN URI: BOLD:AAH0134

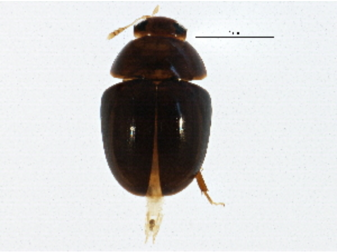

BIOUG05600-F04 [Dorsal]
Acylomus pugetanus
Family: Phalacridae
BIN URI: BOLD:AAH0135

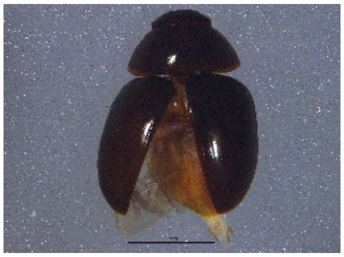

BIOUG12062-F10 [Dorsal]
Phalacridae
Family: Phalacridae
BIN URI: BOLD:ACM7465

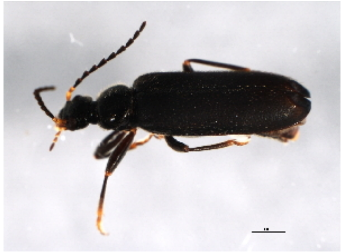

AY165670 [Dorsal]
Pedilus sp. SLB-2003
Family: Pyrochroidae
BIN URI: BOLD:AAH0127

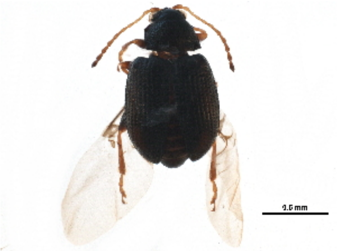

BIOUG22575-B07 [Dorsal]
Epitrix cucumeris
Family: Chrysomelidae
BIN URI: BOLD:ABA9101

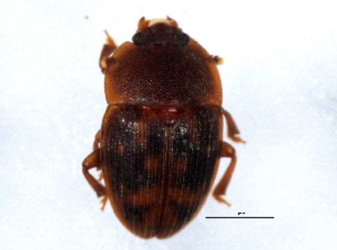

TDWG-0664 [Dorsal]
Nitidulidae
Family: Nitidulidae
BIN URI: BOLD:AAH0115

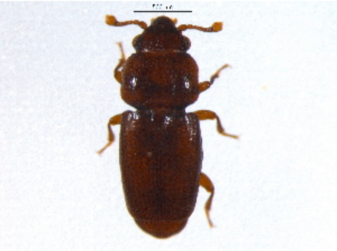

BIOUG02943-B05 [Dorsal]
Monotomidae
Family: Monotomidae
BIN URI: BOLD:ACA5543

IMAGE NOT AVAILABLE

BIOUG22422-D11
Monotomidae
Family: Monotomidae

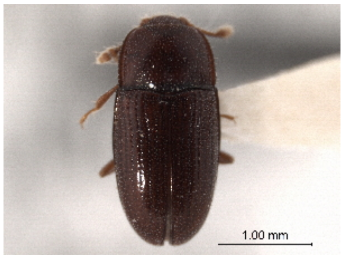

CNC COLEO 00160046 [Dorsal]
Philothermus glabriculus
Family: Cerylonidae
BIN URI: BOLD:ABX9329

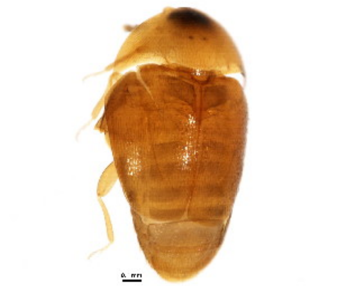

BIOUG00993-F05 [Dorsal]
Sericoderus lateralis
Family: Corylophidae
BIN URI: BOLD:ABA2914

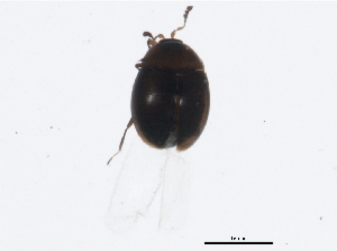

BIOUG00873-D09 [Dorsal]
Corylophidae
Family: Corylophidae
BIN URI: BOLD:AAU7040

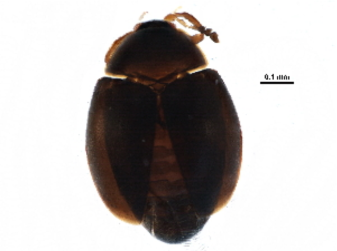

BIOUG22354-D08 [Dorsal]
Orthoperus
Family: Corylophidae
BIN URI: BOLD:ACC5439

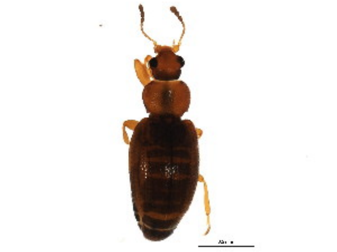

BIOUG01144-C03 [Dorsal]
Corticarina
Family: Latridiidae
BIN URI: BOLD:AAH0256

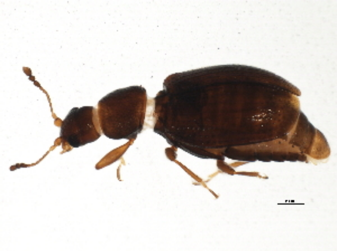

BIOUG05559-F11 [Lateral]
Latridiidae
Family: Latridiidae
BIN URI: BOLD:ACF8198

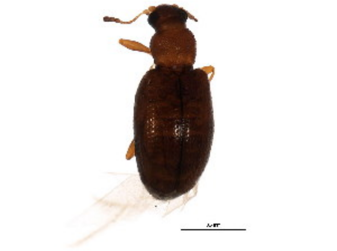

BIOUG01144-A11 [Dorsal]
Cortinicara gibbosa
Family: Latridiidae
BIN URI: BOLD:AAI8935

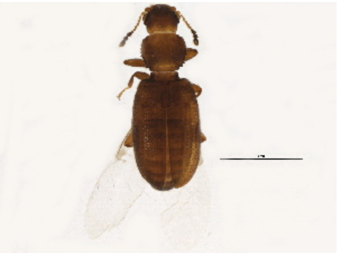

BIOUG08721-B02 [Dorsal]
Latridiidae
Family: Latridiidae
BIN URI: BOLD:ACK2360

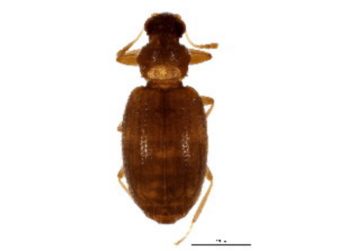

BIOUG01502-A08 [Dorsal]
Melanophthalma
Family: Latridiidae
BIN URI: BOLD:AAM7680

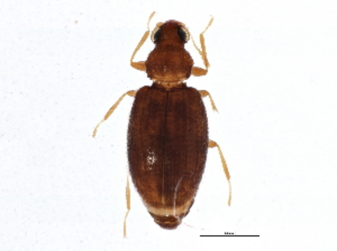

BIOUG03260-D04 [Dorsal]
Melanophthalma
Family: Latridiidae
BIN URI: BOLD:ABX1677

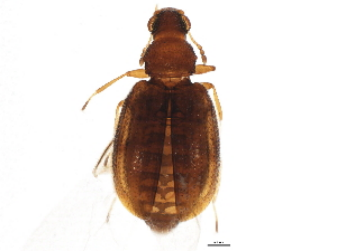

BIOUG05509-H09 [Dorsal]
Corticaria
Family: Latridiidae
BIN URI: BOLD:AAN6154

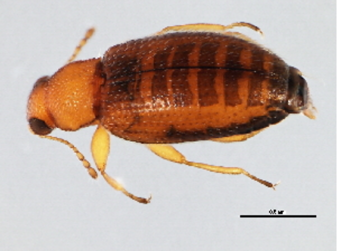

10PCCOL-0062 [Dorsal]
Melanophthalma
Family: Latridiidae
BIN URI: BOLD:AAP7026

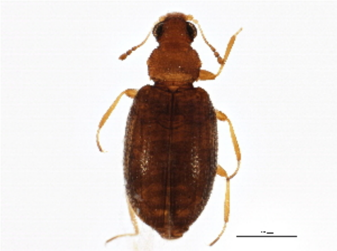

BIOUG02418-G05 [Dorsal]
Corticaria
Family: Latridiidae
BIN URI: BOLD:ABA9093

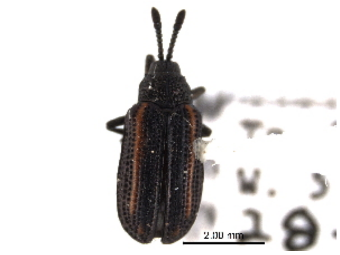

CNC COLEO 00152017 [Dorsal]
Microrhopala vittata
Family: Chrysomelidae
BIN URI: BOLD:AAH0112

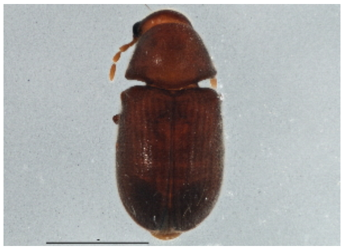

BIOUG07026-G02 [Dorsal]
Curculionidae
Family: Curculionidae
BIN URI: BOLD:ACC1644

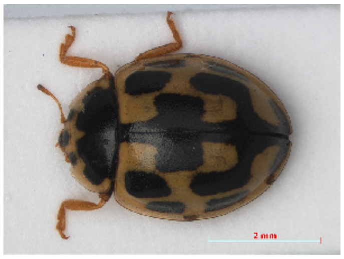

RMNH.INS.535971 [Dorsal]
Propylaea quatuordecimpunctata
Family: Coccinellidae
BIN URI: BOLD:AAF6935

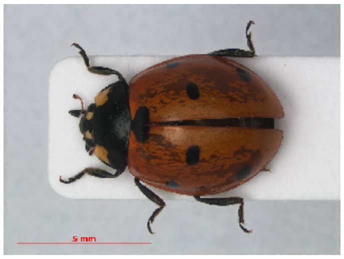

RMNH.INS.535983 [Dorsal]
Coccinella septempunctata
Family: Coccinellidae
BIN URI: BOLD:AAA8933

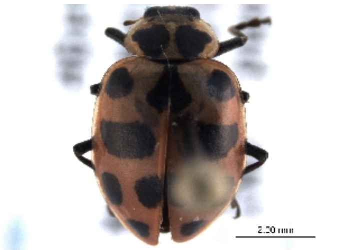

CNC COLEO 00154256 [Dorsal]
Coleomegilla maculata lengi
Family: Coccinellidae
BIN URI: BOLD:AAD7604

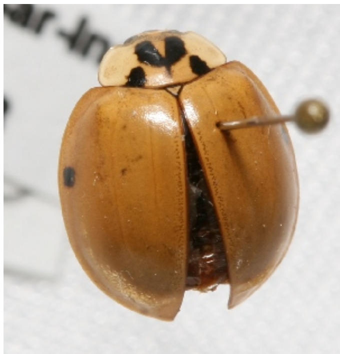

MACN-Bar-Ins-ct 288 [Dorsal]
Coccinellidae
Family: Coccinellidae
BIN URI: BOLD:AAB5640

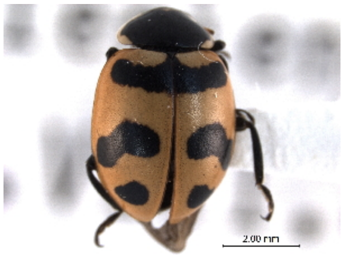

CNC COLEO 00154299 [Dorsal]
Hippodamia glacialis glacialis
Family: Coccinellidae
BIN URI: BOLD:AAH3305

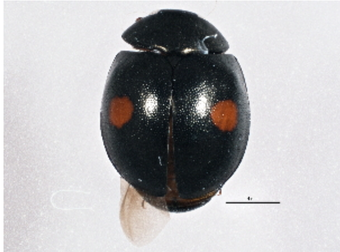

BIOUG05673-A07 [Dorsal]
Hyperaspis binotata
Family: Coccinellidae
BIN URI: BOLD:AAN6147

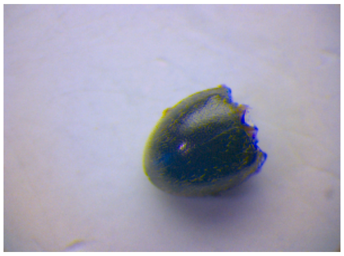

BFB_Col_FK_4209 [Dorsal]
Stethorus punctillum
Family: Coccinellidae
BIN URI: BOLD:AAN6149

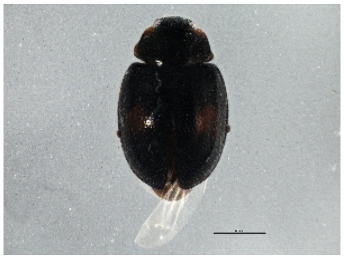

BIOUG03648-D03 [Dorsal]
Scymnus
Family: Coccinellidae
BIN URI: BOLD:ACC1509

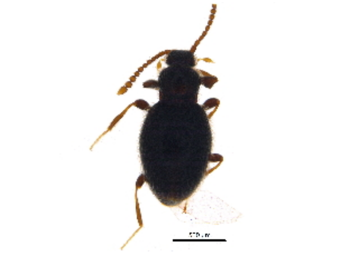

BIOUG22296-G02 [Dorsal]
Stenichnus scutellaris
Family: Staphylinidae
BIN URI: BOLD:AAN9916

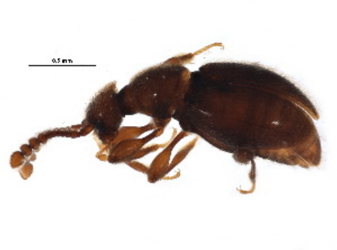

BIOUG20572-F04 [Lateral]
Staphylinidae
Family: Staphylinidae
BIN URI: BOLD:ACV4799

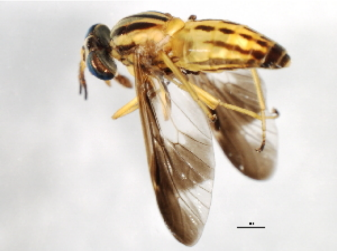

09BBEDI-1210 [Lateral]
Chrysops vittatus
Family: Tabanidae
BIN URI: BOLD:AAB5367

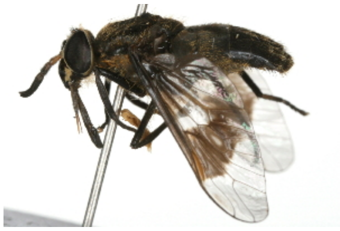

09BBEDI-0332 [Lateral]
Chrysops ater
Family: Tabanidae
BIN URI: BOLD:ACE5640

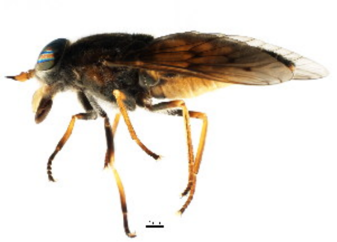

BIOUG01427-C09 [Lateral]
Tabanidae
Family: Tabanidae
BIN URI: BOLD:AAF0889

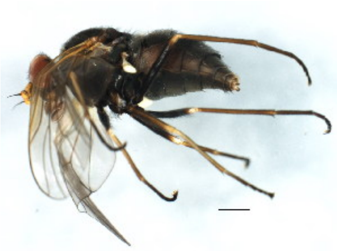

BIOUG00825-B01 [Lateral]
Symphoromyia
Family: Rhagionidae
BIN URI: BOLD:AAG5647

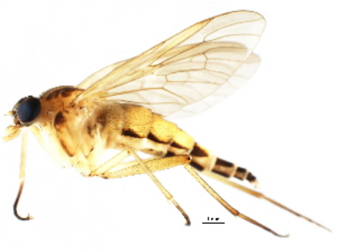

BIOUG01475-A11 [Lateral]
Rhagionidae
Family: Rhagionidae
BIN URI: BOLD:AAH2953

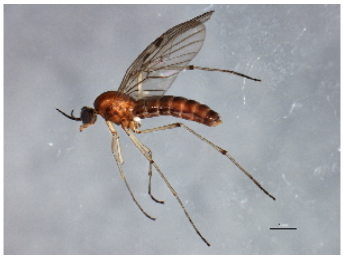

08TTML-1134 [Lateral]
Anisopodidae
Family: Anisopodidae
BIN URI: BOLD:AAG1998

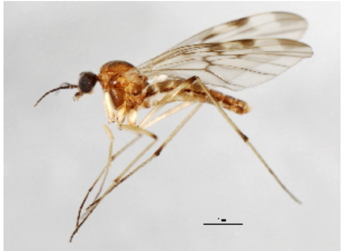

DIPNO-0102 [Lateral]
Anisopodidae
Family: Anisopodidae
BIN URI: BOLD:AAG1996

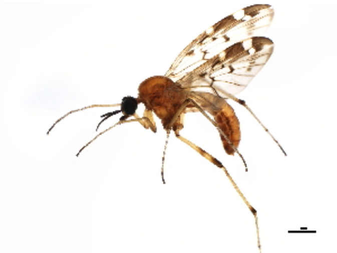

BIOUG01544-C02 [Lateral]
Anisopodidae
Family: Anisopodidae
BIN URI: BOLD:AAG2000

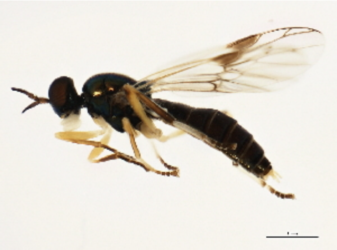

10BBCDIP-3036 [Lateral]
Beridinae
Family: Stratiomyidae
BIN URI: BOLD:AAP7640

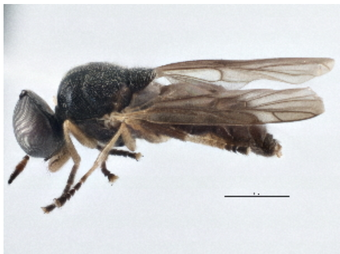

CCDB-21411-G01 [Lateral]
Allognosta fuscitarsis
Family: Stratiomyidae
BIN URI: BOLD:AAV1055

IMAGE NOT AVAILABLE

BIOUG24002-H06
Allognosta obscuriventris
Family: Stratiomyidae

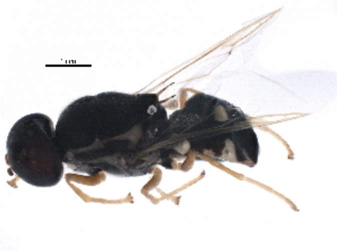

BIOUG24020-D02 [Lateral]
Stratiomyidae
Family: Stratiomyidae

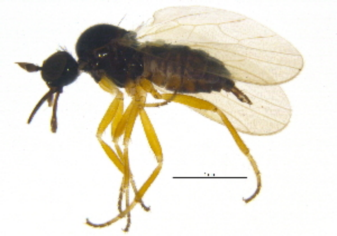

BIOUG03246-H07 [Lateral]
Hybotidae
Family: Hybotidae
BIN URI: BOLD:AAF9826

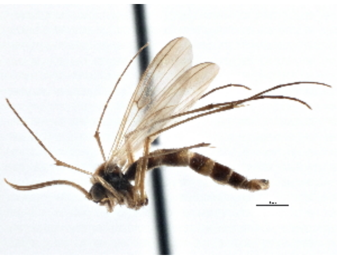

CCDB-21402-G09 [Lateral]
Aglaomyia gatineau
Family: Mycetophilidae
BIN URI: BOLD:ABV3010

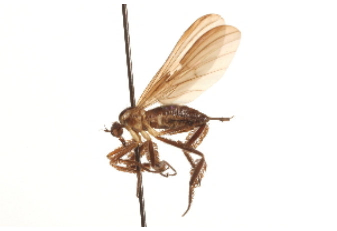

CNC DIPTERA 161599 [Lateral]
Rhamphomyia longicauda
Family: Empididae
BIN URI: BOLD:AAF8462

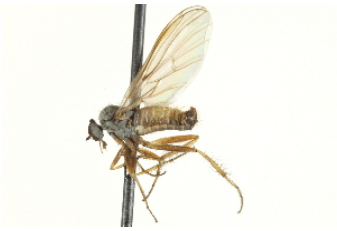

CNC DIPTERA 161541 [Lateral]
Rhamphomyia sp. 9
Family: Empididae
BIN URI: BOLD:AAP2854

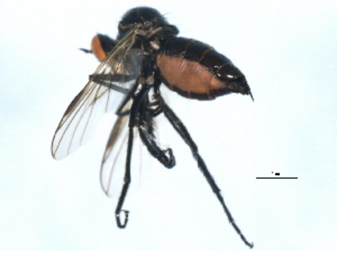

10JSROW-0457 [Lateral]
Empididae
Family: Empididae
BIN URI: BOLD:AAP2857

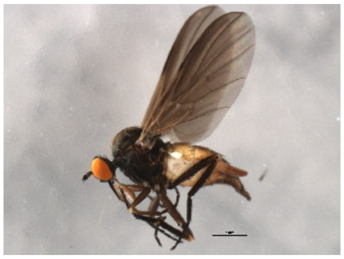

10JSROW-1168 [Lateral]
Empididae
Family: Empididae
BIN URI: BOLD:AAQ0819

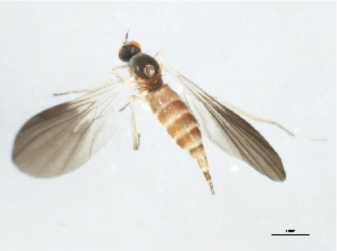

10JSROW-1776 [Dorsal]
Rhamphomyia nr. depilis grp. sp. BJS8
Family: Empididae
BIN URI: BOLD:AAP2858

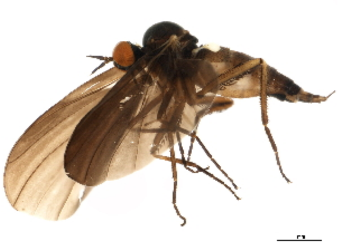

BIOUG01428-G04 [Lateral]
Empididae
Family: Empididae
BIN URI: BOLD:ABW1189

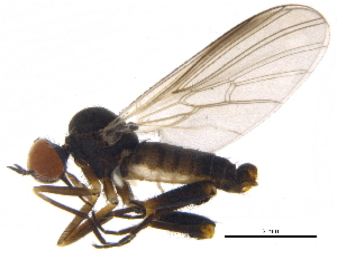

BIOUG22325-G07 [Lateral]
Empididae
Family: Empididae
BIN URI: BOLD:AAF9756

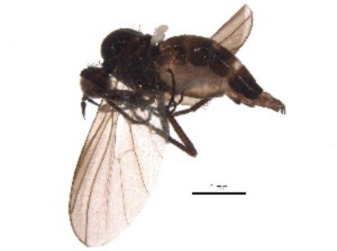

BIOUG22356-H08 [Lateral]
Rhamphomyia
Family: Empididae
BIN URI: BOLD:ACV4054

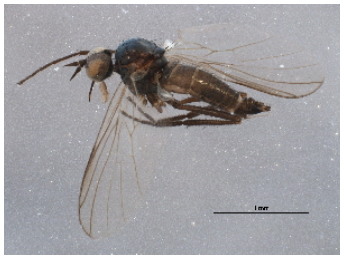

BIOUG09586-G05 [Lateral]
Rhamphomyia
Family: Empididae
BIN URI: BOLD:ACL1493

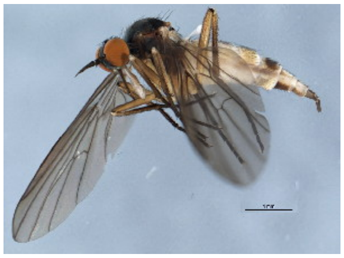

10JSROW-0312 [Lateral]
Empididae
Family: Empididae
BIN URI: BOLD:AAP2855

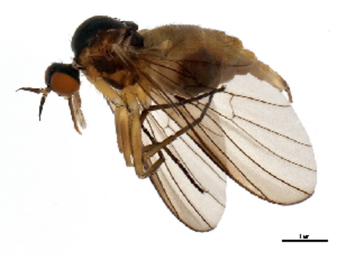

10PHMAL-0871 [Lateral]
Rhamphomyia versicolor
Family: Empididae
BIN URI: BOLD:AAM7337

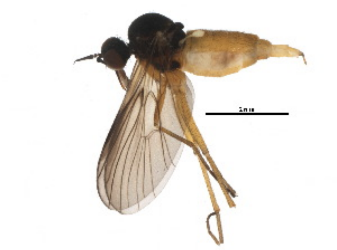

BIOUG22324-B08 [Lateral]
Rhamphomyia
Family: Empididae
BIN URI: BOLD:ACV5478

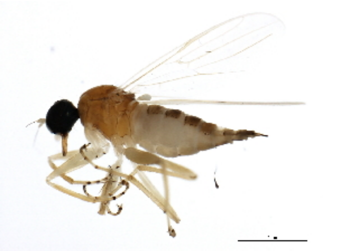

PCPP10-0904 [Lateral]
Platypalpus melleus
Family: Hybotidae
BIN URI: BOLD:AAV3697

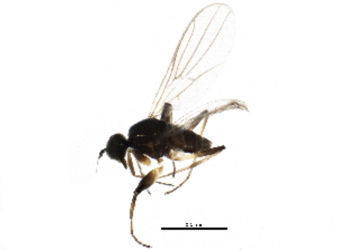

BIOUG24027-D08 [Lateral]
Platypalpus niger
Family: Hybotidae

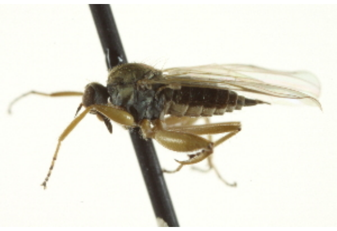

CNC DIPTERA 163527 [Lateral]
Platypalpus sp. 17
Family: Hybotidae
BIN URI: BOLD:AAF9771

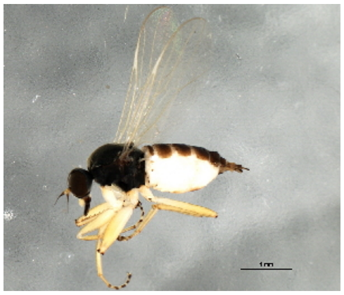

08TTML-1569 [Lateral]
Platypalpus stabilis
Family: Hybotidae
BIN URI: BOLD:AAM6655

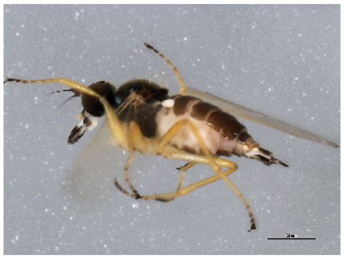

10BBCDIP-0605 [Lateral]
Hybotidae
Family: Hybotidae
BIN URI: BOLD:AAF9789

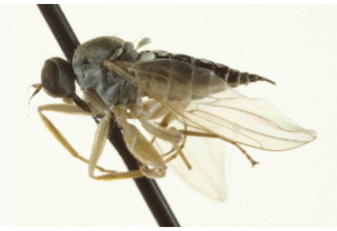

CNC DIPTERA 163547 [Lateral]
Platypalpus sp. 22
Family: Hybotidae
BIN URI: BOLD:AAF9768

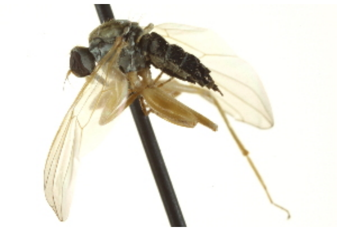

CNC DIPTERA 163557 [Lateral]
Platypalpus sp. 24
Family: Hybotidae
BIN URI: BOLD:AAF9772

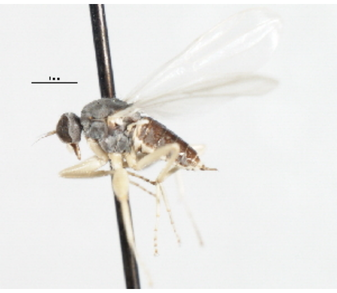

CNC DIPTERA 105448 [Lateral]
Platypalpus holosericus
Family: Hybotidae
BIN URI: BOLD:AAP6357

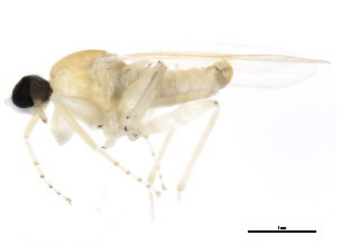

BIOUG01347-B04 [Lateral]
Hybotidae
Family: Hybotidae
BIN URI: BOLD:AAG6934

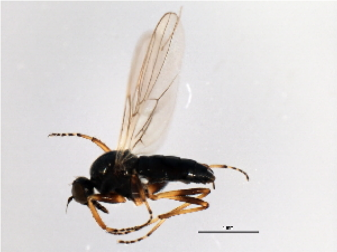

08TTML-2020 [Lateral]
Platypalpus
Family: Hybotidae
BIN URI: BOLD:AAN5505

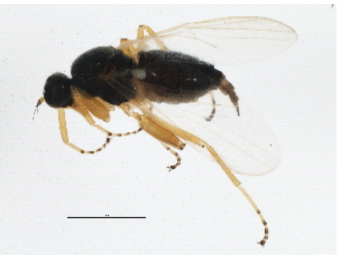

BIOUG03341-B05 [Lateral]
Hybotidae
Family: Hybotidae
BIN URI: BOLD:ACA7165

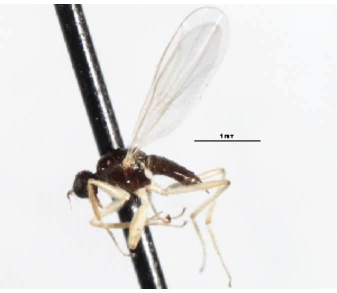

CNC DIPTERA 105481 [Lateral]
Platypalpus unguiculatus
Family: Hybotidae
BIN URI: BOLD:ABA0579

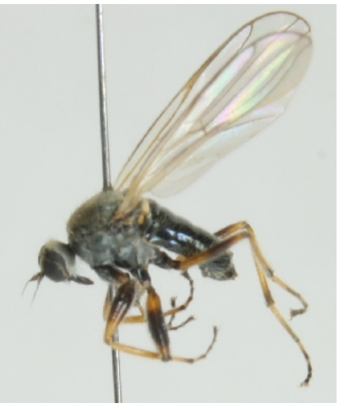

jka10-00287 [Lateral]
Platypalpus annulatus
Family: Hybotidae
BIN URI: BOLD:ACD3012

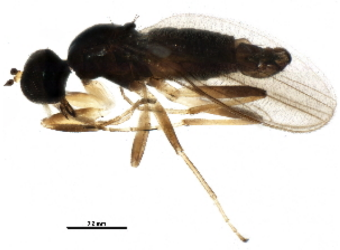

BIOUG22366-A04 [Lateral]
Hybotidae
Family: Hybotidae
BIN URI: BOLD:ACK3535

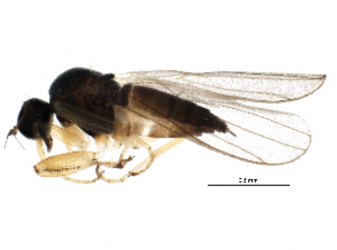

BIOUG22084-E05 [Lateral]
Platypalpus pulicarius
Family: Hybotidae
BIN URI: BOLD:AAQ0265

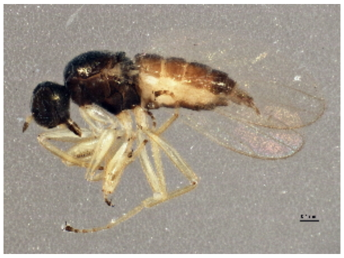

BIOUG03451-D01 [Lateral]
Hybotidae
Family: Hybotidae
BIN URI: BOLD:AAV3695

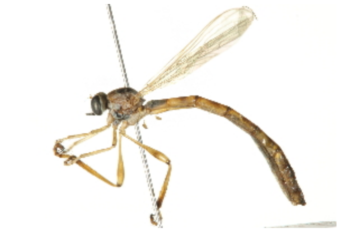

PCPP10-0394 [Lateral]
Asilidae
Family: Asilidae
BIN URI: BOLD:AAH2295

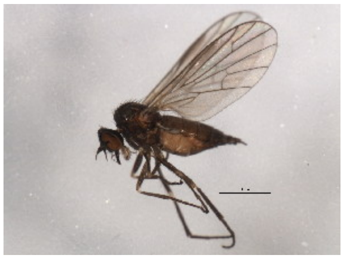

10JSROW-1219 [Lateral]
Empididae
Family: Empididae
BIN URI: BOLD:AAQ0818

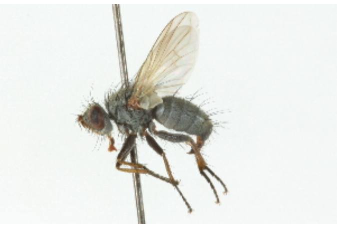

CNC DIPTERA 104335 [Lateral]
Campylocheta teliosis
Family: Tachinidae
BIN URI: BOLD:AAZ4002

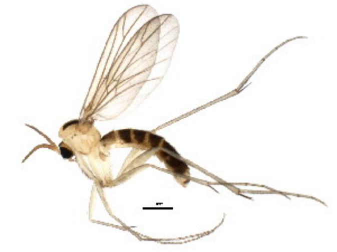

BIOUG01356-B09 [Lateral]
Ditomyiidae
Family: Ditomyiidae
BIN URI: BOLD:AAM8982

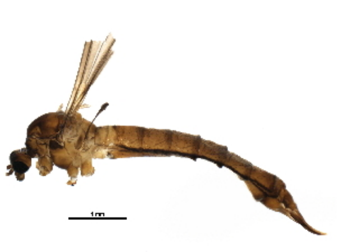

BIOUG21895-C01 [Lateral]
Limnophila
Family: Limoniidae
BIN URI: BOLD:ACV5288

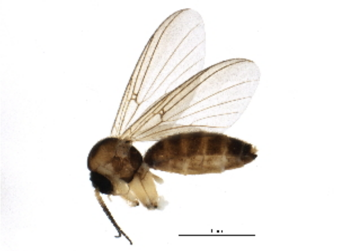

BIOUG21589-D01 [Lateral]
Mycetophilidae

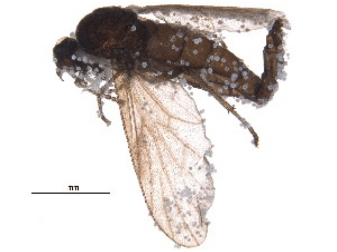

BIOUG20569-G03 [Lateral]
Bolbomyia nana

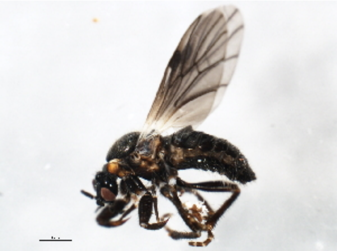

09BBDIP-0719 [Lateral]
Bibionidae

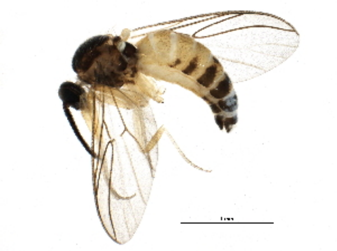

BIOUG22362-D09 [Lateral]
Mycetobia

Family: Mycetophilidae
BIN URI: BOLD:ABV9017

Family: Rhagionidae
BIN URI: BOLD:ACV5660

Family: Bibionidae
BIN URI: BOLD:AAG6647

Family: Anisopodidae
BIN URI: BOLD:AAV4005

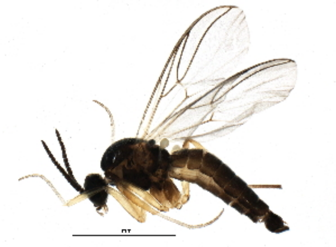

BIOUG22362-E01 [Lateral]
Mycetobia
Family: Anisopodidae
BIN URI: BOLD:AAV4004

IMAGE NOT AVAILABLE

BIOUG22415-A12
Mycetobia
Family: Anisopodidae

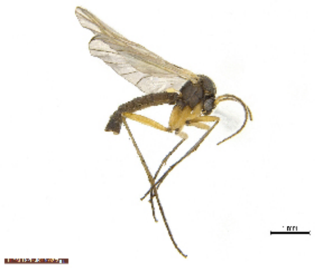

TROM-TSZD-JKJ-100273 [Insect specimen]
Boletina palmata
Family: Mycetophilidae
BIN URI: BOLD:AAG4924

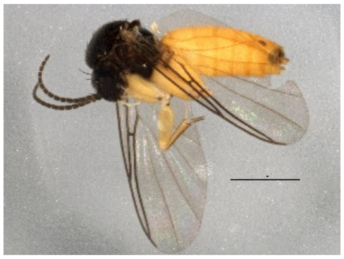

BIOUG01645-H07 [Lateral]
Mycetophilidae
Family: Mycetophilidae
BIN URI: BOLD:AAG4923

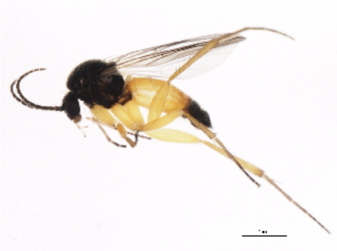

09BBDIP-1684 [Lateral]
Mycetophilidae
Family: Mycetophilidae
BIN URI: BOLD:AAL9138

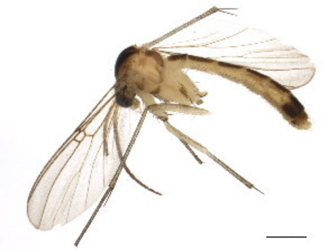

BIOUG01983-C04 [Lateral]
Mycetophilidae
Family: Mycetophilidae
BIN URI: BOLD:AAG4890

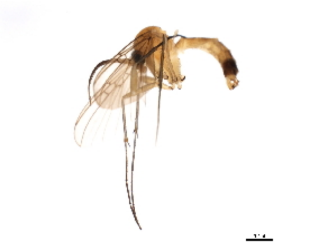

09BBEDI-1515 [Lateral]
Mycetophilidae
Family: Mycetophilidae
BIN URI: BOLD:AAG4960

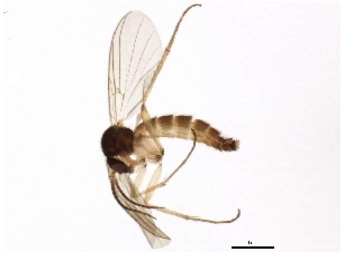

BIOUG01659-E09 [Lateral]
Mycetophilidae
Family: Mycetophilidae
BIN URI: BOLD:AAG4871

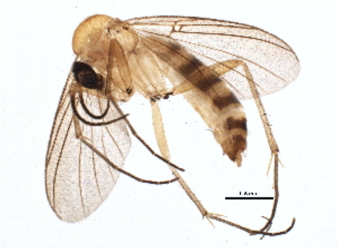

BIOUG11150-G08 [Lateral]
Mycetophilidae
Family: Mycetophilidae
BIN URI: BOLD:ACM0350

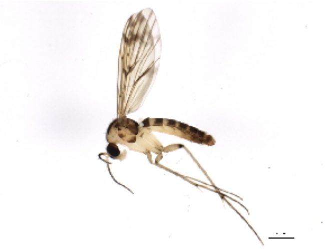

BIOUG01373-D12 [Lateral]
Mycetophilidae
Family: Mycetophilidae
BIN URI: BOLD:AAG4898

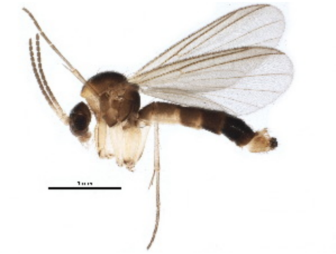

BIOUG22291-E01 [Lateral]
Trichonta submaculata
Family: Mycetophilidae
BIN URI: BOLD:AAU4912

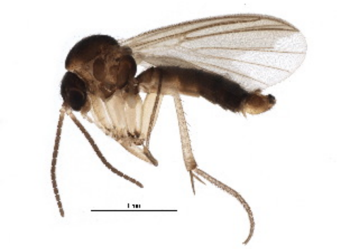

BIOUG21770-E02 [Lateral]
Mycetophilinae
Family: Mycetophilidae
BIN URI: BOLD:ACD2208

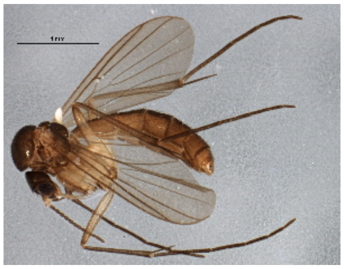

BIOUG02965-F12 [Lateral]
Mycetophilidae
Family: Mycetophilidae
BIN URI: BOLD:ACA3795

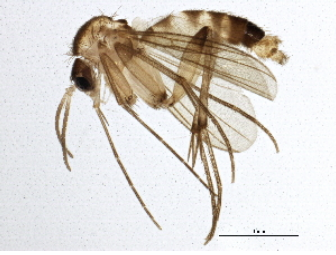

BIOUG03069-C12 [Lateral]
Mycetophilidae
Family: Mycetophilidae
BIN URI: BOLD:ABV3021

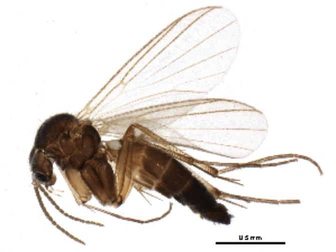

BIOUG21590-F01 [Lateral]
Phronia
Family: Mycetophilidae
BIN URI: BOLD:AAL4861

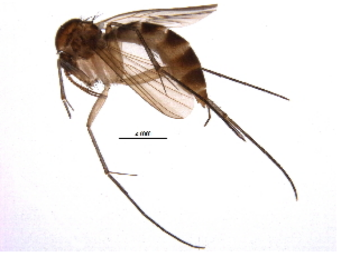

BIOUG09307-F12 [Lateral]
Mycetophilidae
Family: Mycetophilidae
BIN URI: BOLD:ACL0109

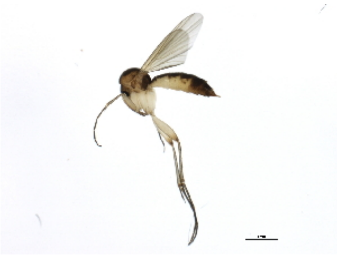

10JSROW-1772 [Lateral]
Mycetophilidae
Family: Mycetophilidae
BIN URI: BOLD:AAP2527

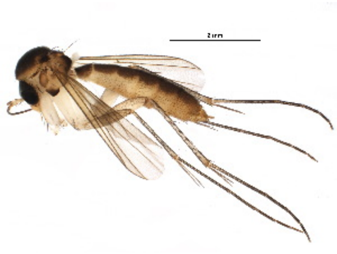

BIOUG22324-G09 [Lateral]
Mycetophilidae
Family: Mycetophilidae
BIN URI: BOLD:AAP4733

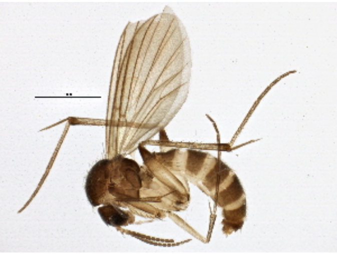

BIOUG03030-H11 [Lateral]
Mycetophilidae
Family: Mycetophilidae
BIN URI: BOLD:AAN8585

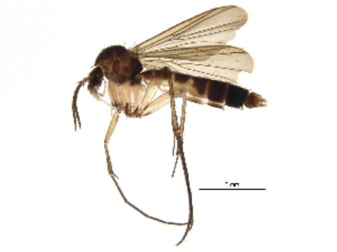

BIOUG06129-F02 [Lateral]
Mycetophilidae
Family: Mycetophilidae
BIN URI: BOLD:AAG4972

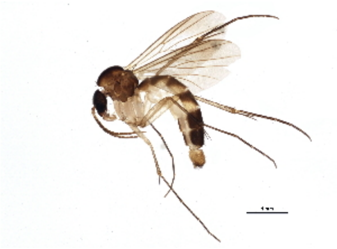

BIOUG04070-B01 [Lateral]
Mycetophilidae
Family: Mycetophilidae
BIN URI: BOLD:ACD0959

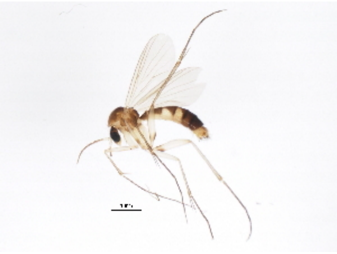

10JSROW-1626 [Lateral]
Mycetophilidae
Family: Mycetophilidae
BIN URI: BOLD:AAM8991

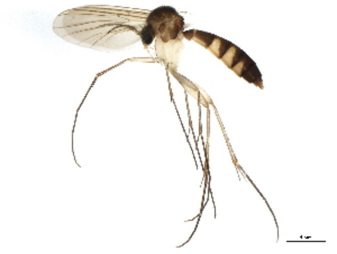

BIOUG01392-H11 [Lateral]
Mycetophilidae
Family: Mycetophilidae
BIN URI: BOLD:AAN8592

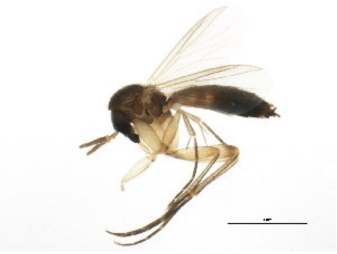

BIOUG01343-B01 [Lateral]
Mycetophilidae
Family: Mycetophilidae
BIN URI: BOLD:ABU5545

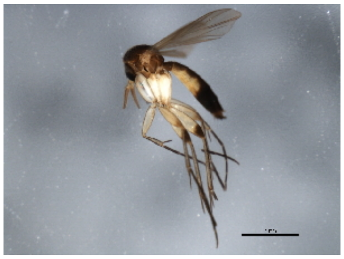

09BBEDI-2498 [Lateral]
Cordyla
Family: Mycetophilidae
BIN URI: BOLD:ABV9015

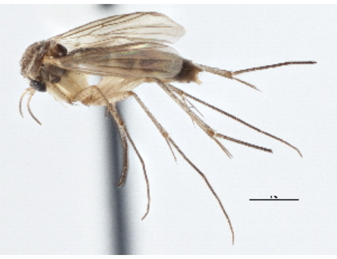

CCDB-21411-A04 [Lateral]
Exechia attrita
Family: Mycetophilidae
BIN URI: BOLD:ACM3454

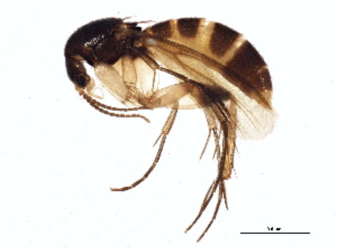

BIOUG02965-E11 [Lateral]
Sceptonia
Family: Mycetophilidae
BIN URI: BOLD:AAG4967

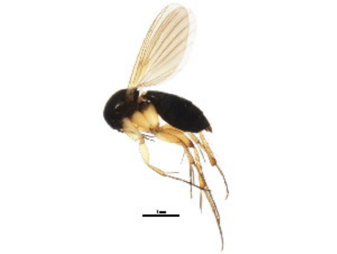

BIOUG01931-F04 [Lateral]
Mycetophilidae
Family: Mycetophilidae
BIN URI: BOLD:AAJ0565

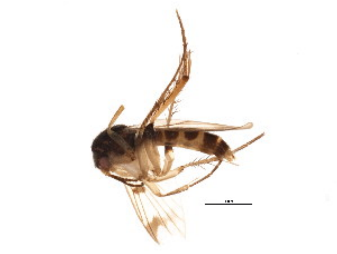

BIOUG01670-A03 [Lateral]
Mycetophilidae
Family: Mycetophilidae
BIN URI: BOLD:AAG4953

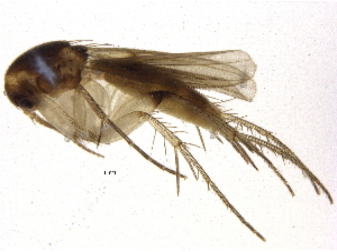

BIOUG03638-H11 [Lateral]
Mycetophilidae
Family: Mycetophilidae
BIN URI: BOLD:AAN8587

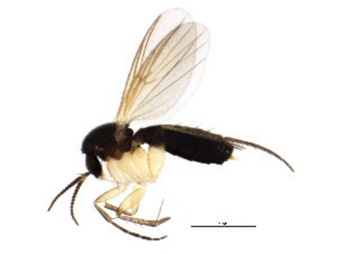

BIOUG01689-C10 [Lateral]
Mycetophilidae
Family: Mycetophilidae
BIN URI: BOLD:AAU4913

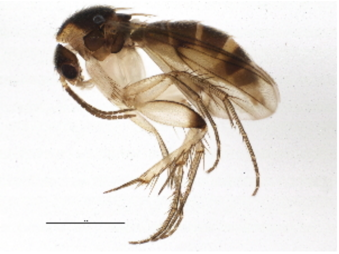

BIOUG02936-C11 [Lateral]
Mycetophila caudata
Family: Mycetophilidae
BIN URI: BOLD:AAI3260

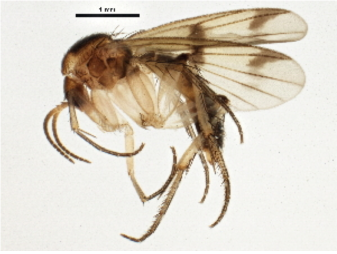

BIOUG05581-H09 [Lateral]
Mycetophila
Family: Mycetophilidae
BIN URI: BOLD:AAP8158

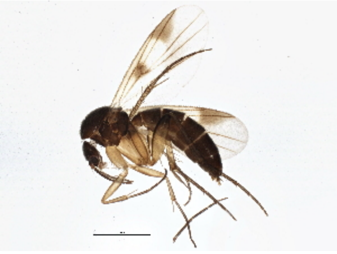

BIOUG06361-F02 [Lateral]
Zygomyia zaitzevi
Family: Mycetophilidae
BIN URI: BOLD:ABW9135

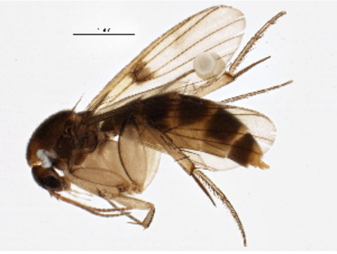

BIOUG03066-G09 [Lateral]
Epicypta
Family: Mycetophilidae
BIN URI: BOLD:ACA3426

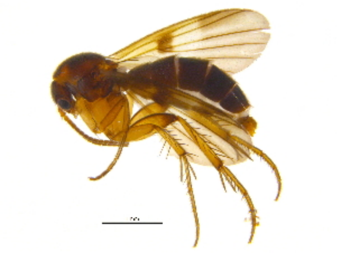

BIOUG04655-H05 [Lateral]
Epicypta
Family: Mycetophilidae
BIN URI: BOLD:ACC5656

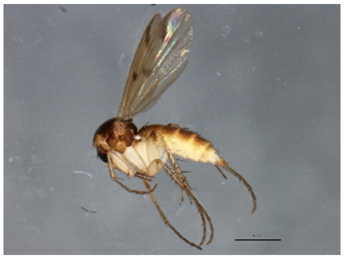

08TTML-1057 [Lateral]
Mycetophilidae
Family: Mycetophilidae
BIN URI: BOLD:AAG4922

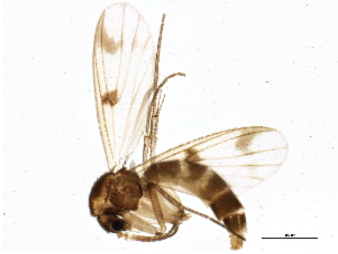

BIOUG03669-E10 [Lateral]
Mycetophilidae
Family: Mycetophilidae
BIN URI: BOLD:ACC2284

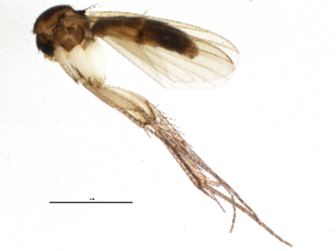

BIOUG02975-E01 [Lateral]
Mycetophilidae
Family: Mycetophilidae
BIN URI: BOLD:AAM8964

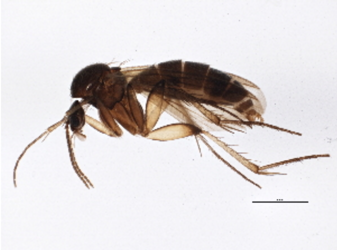

BIOUG05033-H06 [Lateral]
Mycetophila
Family: Mycetophilidae
BIN URI: BOLD:ABA6478

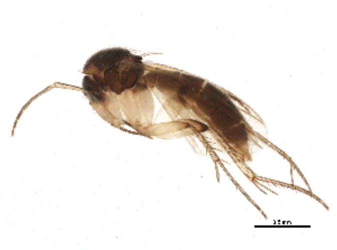

BIOUG10842-H10 [Lateral]
Mycetophila
Family: Mycetophilidae
BIN URI: BOLD:ACK1664

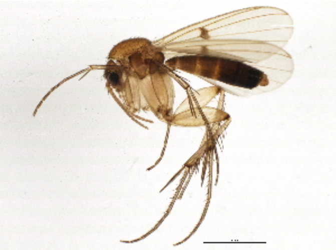

BIOUG03039-A10 [Lateral]
Mycetophilidae
Family: Mycetophilidae
BIN URI: BOLD:AAM8980

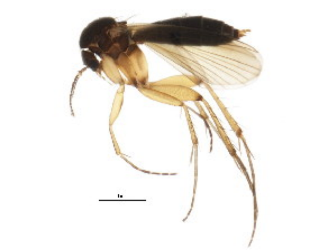

BIOUG01931-E12 [Lateral]
Mycetophilidae
Family: Mycetophilidae
BIN URI: BOLD:AAG4893

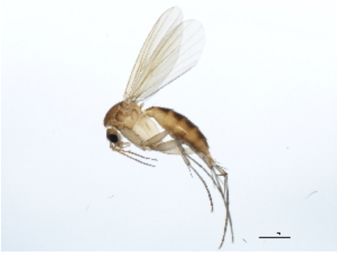

10BBDIP-2271 [Lateral]
Mycetophilidae
Family: Mycetophilidae
BIN URI: BOLD:AAG4908

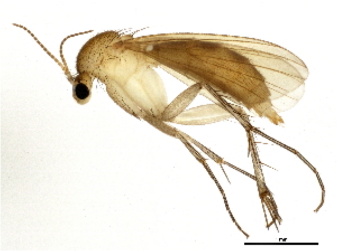

BIOUG02739-G07 [Lateral]
Mycetophila fungorum
Family: Mycetophilidae
BIN URI: BOLD:ACF2821

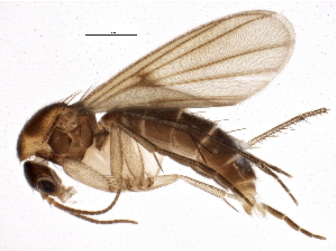

BIOUG02987-A04 [Lateral]
Mycetophilidae
Family: Mycetophilidae
BIN URI: BOLD:ACA4089

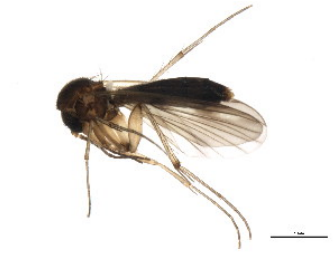

BIOUG01347-G09 [Lateral]
Mycetophilidae
Family: Mycetophilidae
BIN URI: BOLD:AAP4734

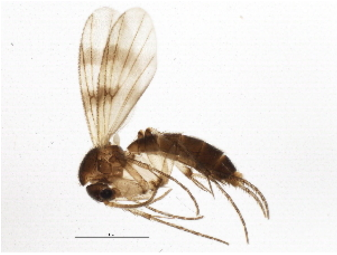

BIOUG02936-E07 [Lateral]
Mycetophila
Family: Mycetophilidae
BIN URI: BOLD:AAG4907

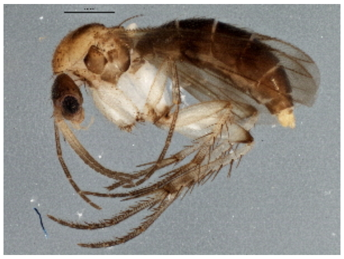

BIOUG02987-B05 [Lateral]
Mycetophila
Family: Mycetophilidae
BIN URI: BOLD:ABV9019

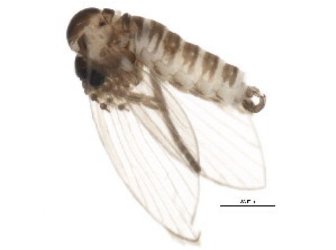

BIOUG01307-E03 [Lateral]
Psychodidae
Family: Psychodidae
BIN URI: BOLD:AAN8769

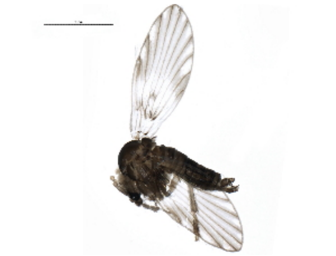

10JSROW-1107 [Lateral]
Psychodidae
Family: Psychodidae
BIN URI: BOLD:ACQ6228

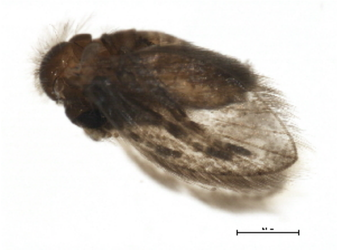

09BBDIP-1459 [Lateral]
Psychodidae
Family: Psychodidae
BIN URI: BOLD:AAF9310

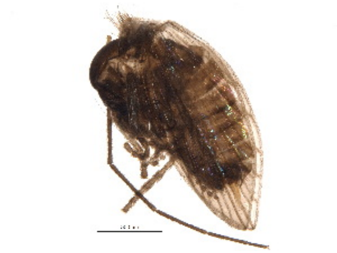

BIOUG20764-A01 [Lateral]
Diptera

BIN URI: BOLD:ACU3012

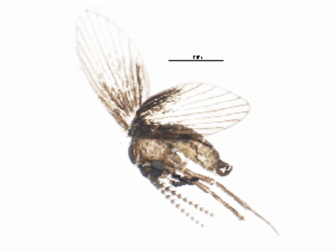

10JSROW-1769 [Lateral]
Psychodidae
Family: Psychodidae
BIN URI: BOLD:AAN8771

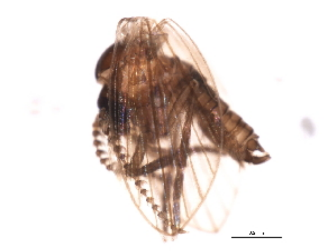

09BBEDI-2826 [Lateral]
Psychodidae
Family: Psychodidae
BIN URI: BOLD:AAF9319

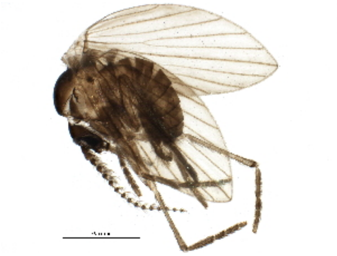

BIOUG22366-C06 [Lateral]
Psychodidae
Family: Psychodidae
BIN URI: BOLD:AAU6540

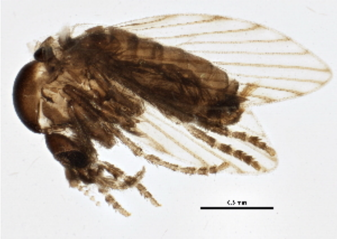

BIOUG03369-F09 [Lateral]
Psychodidae
Family: Psychodidae
BIN URI: BOLD:ABW1323

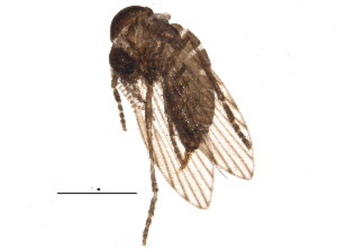

BIOUG20846-A07 [Lateral]
Diptera

BIN URI: BOLD:ACU7066

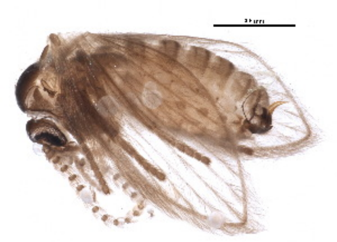

BIOUG08005-H09 [Lateral]
Psychoda sp.
Family: Psychodidae
BIN URI: BOLD:AAF9314

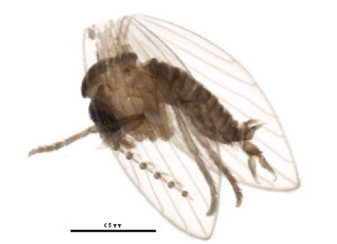

BIOUG01345-G01 [Lateral]
Psychodidae
Family: Psychodidae
BIN URI: BOLD:ABV1314

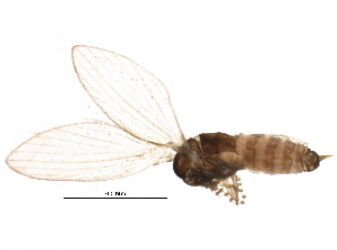

BIOUG22299-E06 [Lateral]
Psychoda
Family: Psychodidae
BIN URI: BOLD:AAF9316

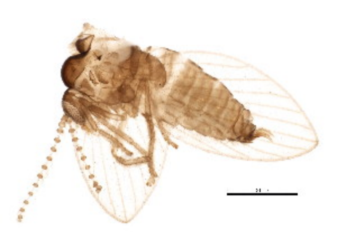

BIOUG01666-C01 [Lateral]
Psychodidae
Family: Psychodidae
BIN URI: BOLD:AAF9311

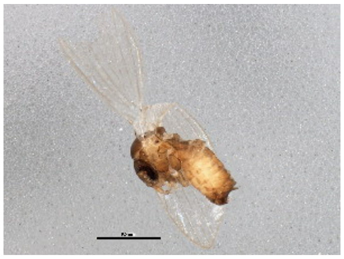

BIOUG01929-D07 [Lateral]
Psychodidae
Family: Psychodidae
BIN URI: BOLD:AAL7815

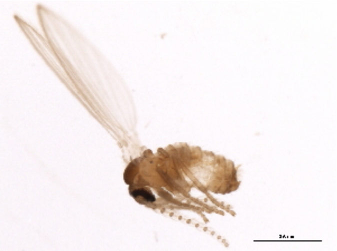

08TTML-2230 [Lateral]
Psychodidae
Family: Psychodidae
BIN URI: BOLD:AAN8770

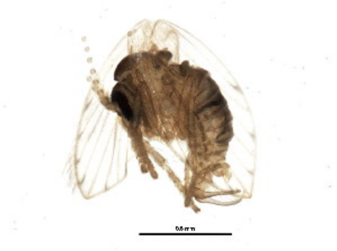

BIOUG01307-F03 [Lateral]
Psychodidae
Family: Psychodidae
BIN URI: BOLD:AAP4581

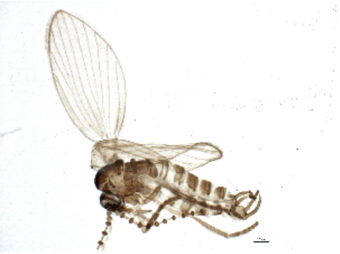

BIOUG04254-B06 [Lateral]
Psychoda
Family: Psychodidae
BIN URI: BOLD:AAF9317

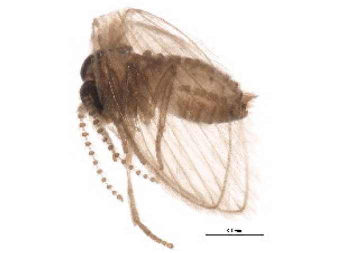

BIOUG01400-E12 [Lateral]
Psychodidae
Family: Psychodidae
BIN URI: BOLD:ABU5546

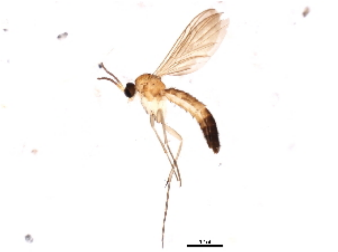

09BBEDI-2897 [Lateral]
Keroplatidae
Family: Keroplatidae
BIN URI: BOLD:AAG4979

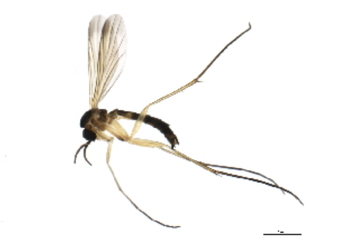

10JSROW-1778 [Lateral]
Keroplatidae
Family: Keroplatidae
BIN URI: BOLD:AAP2528

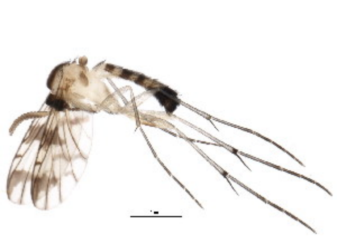

BIOUG01356-C06 [Lateral]
Keroplatidae
Family: Keroplatidae
BIN URI: BOLD:AAG4951

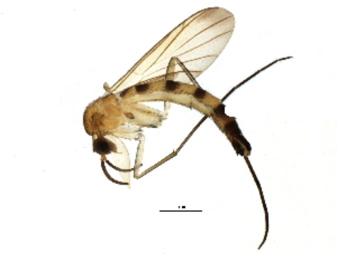

BIOUG10369-C10 [Lateral]
Keroplatidae
Family: Keroplatidae
BIN URI: BOLD:ACL5302

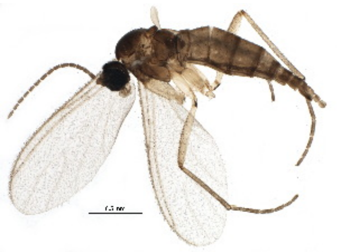

BIOUG22467-A09 [Lateral]
Sciaridae
Family: Sciaridae
BIN URI: BOLD:ACC2619

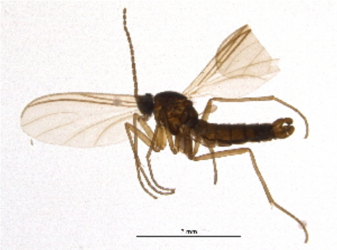

BIOUG04563-C10 [Lateral]
Sciaridae
Family: Sciaridae
BIN URI: BOLD:ACD8833

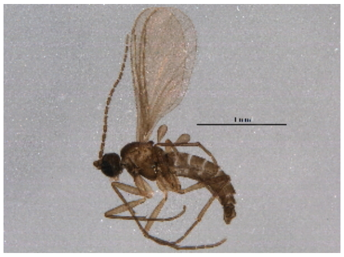

BIOUG10575-C09 [Lateral]
Sciaridae
Family: Sciaridae
BIN URI: BOLD:ACL7902

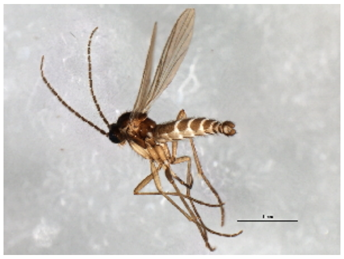

08TTML-1508 [Lateral]
Sciaridae
Family: Sciaridae
BIN URI: BOLD:AAN6439

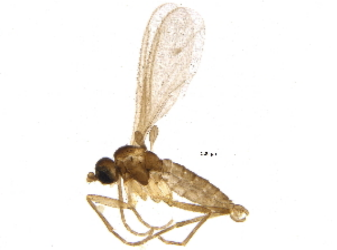

BIOUG03647-D10 [Lateral]
Sciaridae
Family: Sciaridae
BIN URI: BOLD:AAU6616

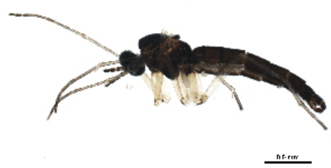

BIOUG21893-G10 [Lateral]
Sciaridae
Family: Sciaridae
BIN URI: BOLD:ACC9223

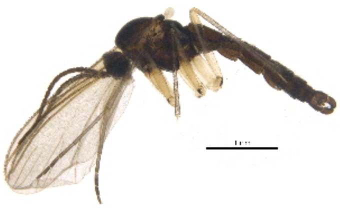

BIOUG22573-E07 [Lateral]
Sciaridae
Family: Sciaridae
BIN URI: BOLD:AAP6466

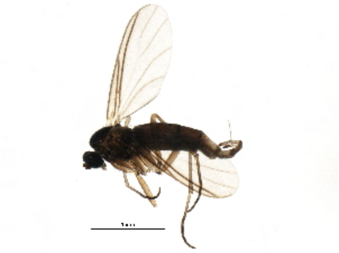

BIOUG22362-F01 [Lateral]
Sciaridae
Family: Sciaridae
BIN URI: BOLD:ACV4825

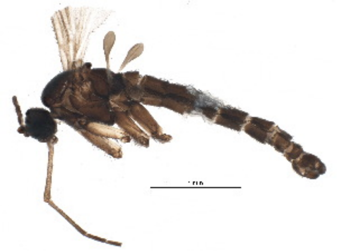

BIOUG22418-F09 [Lateral]
Sciaridae
Family: Sciaridae
BIN URI: BOLD:ACJ7008

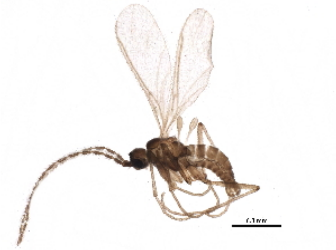

BIOUG08481-F11 [Lateral]
Sciaridae
Family: Sciaridae
BIN URI: BOLD:ACV8240

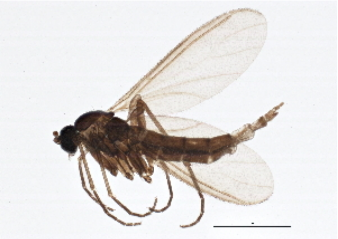

BIOUG03062-D12 [Lateral]
Sciaridae
Family: Sciaridae
BIN URI: BOLD:ACC8493

IMAGE NOT AVAILABLE

BIOUG22729-G08
Sciaridae
Family: Sciaridae

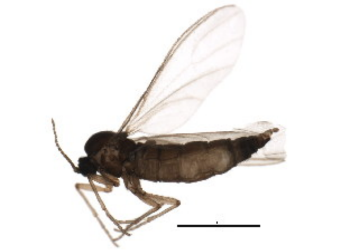

BIOUG01337-G08 [Lateral]
Sciaridae
Family: Sciaridae
BIN URI: BOLD:AAN6770

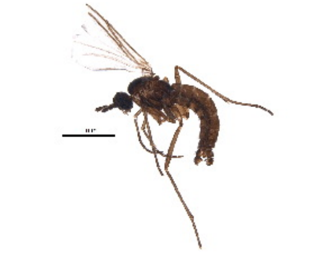

BIOUG20569-D12 [Lateral]
Sciaridae
Family: Sciaridae
BIN URI: BOLD:ACG7300

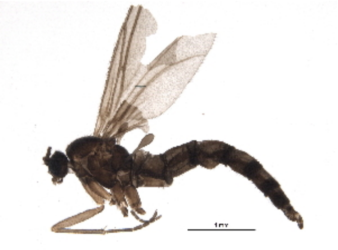

BIOUG10693-C08 [Lateral]
Sciaridae
Family: Sciaridae
BIN URI: BOLD:ACL3186

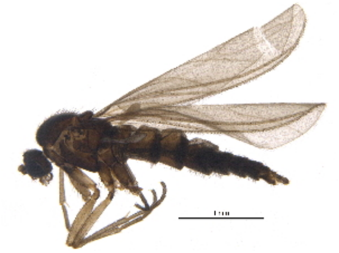

BIOUG22330-B05 [Lateral]
Sciaridae
Family: Sciaridae
BIN URI: BOLD:ACB3271

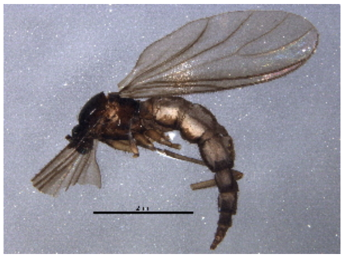

BIOUG08515-E03 [Lateral]
Sciaridae
Family: Sciaridae
BIN URI: BOLD:ACK1571

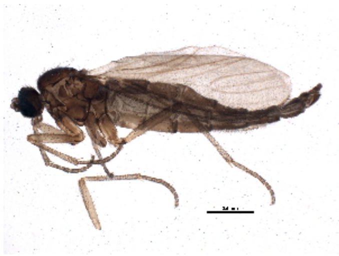

BIOUG08530-D02 [Lateral]
Sciaridae
Family: Sciaridae
BIN URI: BOLD:ACK1679

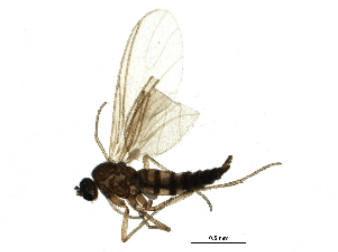

BIOUG22462-B09 [Lateral]
Sciaridae
Family: Sciaridae
BIN URI: BOLD:ACA9051

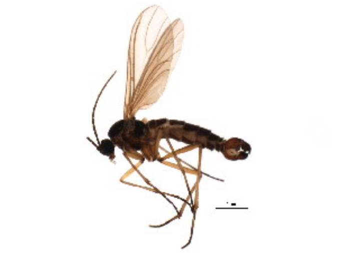

BIOUG01408-G03 [Lateral]
Sciaridae
Family: Sciaridae
BIN URI: BOLD:AAH3914

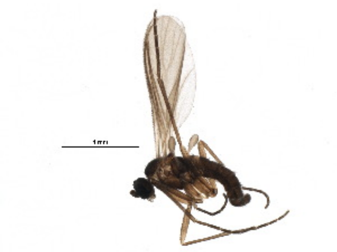

BIOUG22466-A09 [Lateral]
Cratyna ambigua
Family: Sciaridae
BIN URI: BOLD:AAH3968

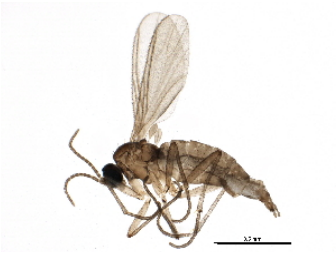

BIOUG22084-F10 [Lateral]
Sciaridae
Family: Sciaridae
BIN URI: BOLD:ACA7685

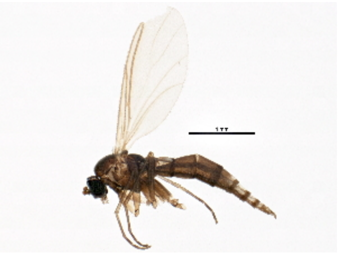

BIOUG02754-G12 [Lateral]
Sciaridae
Family: Sciaridae
BIN URI: BOLD:ACB1266

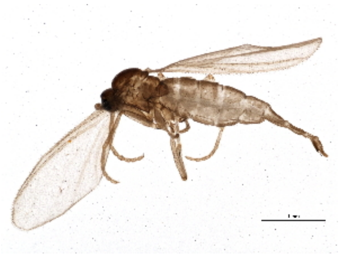

BIOUG03109-H06 [Lateral]
Sciaridae
Family: Sciaridae
BIN URI: BOLD:ACC1769

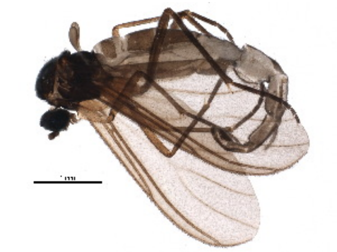

BIOUG22419-C05 [Lateral]
Sciaridae
Family: Sciaridae
BIN URI: BOLD:ACK1586

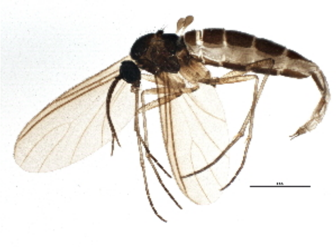

BIOUG04307-H09 [Lateral]
Sciaridae
Family: Sciaridae
BIN URI: BOLD:ACC8275

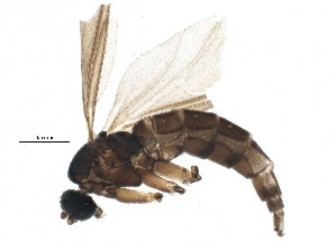

BIOUG20695-C11 [Lateral]
Sciaridae
Family: Sciaridae
BIN URI: BOLD:ACU2906

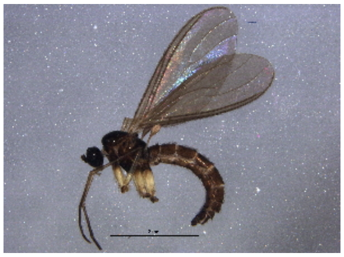

BIOUG08523-H02 [Lateral]
Sciaridae
Family: Sciaridae
BIN URI: BOLD:ACL3853

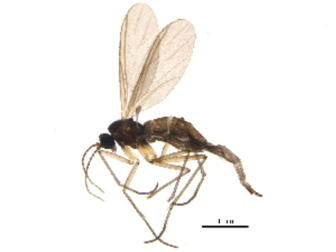

BIOUG22865-E04 [Lateral]
Sciaridae
Family: Sciaridae
BIN URI: BOLD:ACL8389

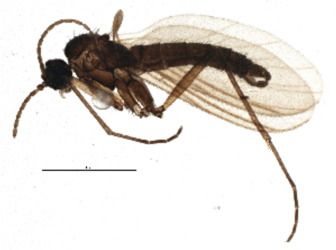

BIOUG04290-G02 [Lateral]
Sciaridae
Family: Sciaridae
BIN URI: BOLD:ACD0718

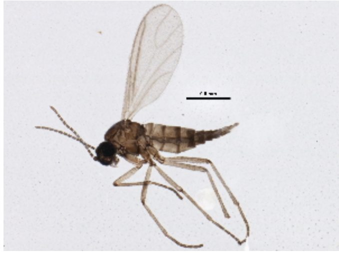

BIOUG05665-C05 [Lateral]
Sciaridae
Family: Sciaridae
BIN URI: BOLD:AAU6614

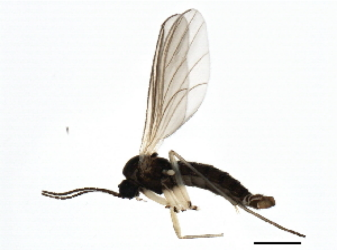

10PHMAL-1271 [Lateral]
Sciaridae
Family: Sciaridae
BIN URI: BOLD:AAU6626

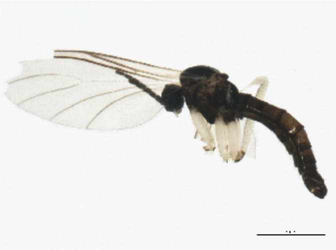

BIOUG01485-H01 [Lateral]
Sciaridae
Family: Sciaridae
BIN URI: BOLD:ACG4218

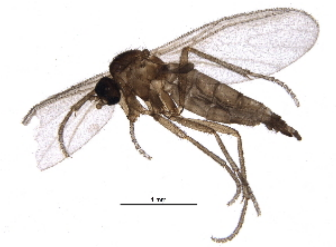

BIOUG12068-F12 [Lateral]
Sciaridae
Family: Sciaridae
BIN URI: BOLD:ACM4794

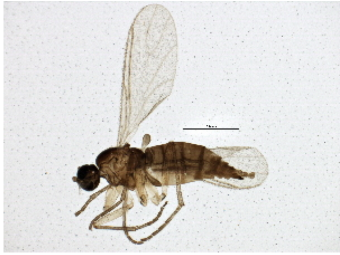

BIOUG02981-F02 [Lateral]
Sciaridae
Family: Sciaridae
BIN URI: BOLD:AAP6465

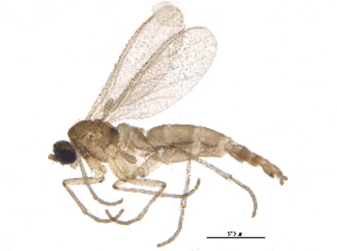

BIOUG20508-H06 [Lateral]
Diptera

BIN URI: BOLD:ACU3040

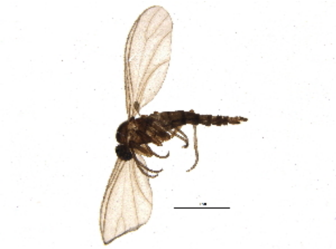

BIOUG05873-F12 [Lateral]
Sciaridae
Family: Sciaridae
BIN URI: BOLD:ACG6762

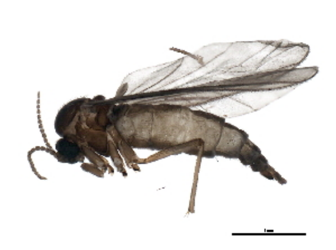

10PHMAL-1000 [Lateral]
Sciaridae
Family: Sciaridae
BIN URI: BOLD:AAU6627

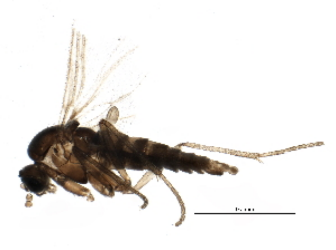

BIOUG22084-H08 [Lateral]
Sciaridae
Family: Sciaridae
BIN URI: BOLD:AAQ0299

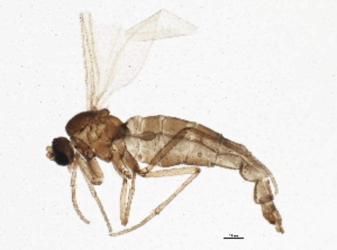

BIOUG02660-G08 [Lateral]
Sciaridae
Family: Sciaridae
BIN URI: BOLD:AAZ0294

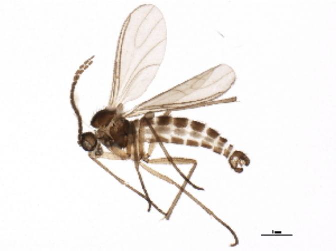

BIOUG01659-D12 [Lateral]
Sciaridae
Family: Sciaridae
BIN URI: BOLD:ABA6407

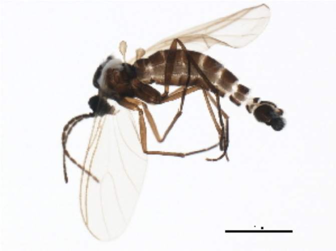

BIOUG01662-B01 [Lateral]
Sciaridae
Family: Sciaridae
BIN URI: BOLD:ABA6474

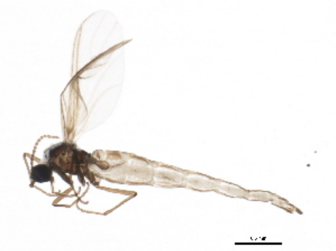

BIOUG01302-G05 [Lateral]
Sciaridae
Family: Sciaridae
BIN URI: BOLD:AAZ5612

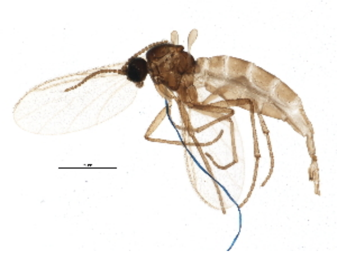

BIOUG03507-H06 [Lateral]
Sciaridae
Family: Sciaridae
BIN URI: BOLD:ACC1855

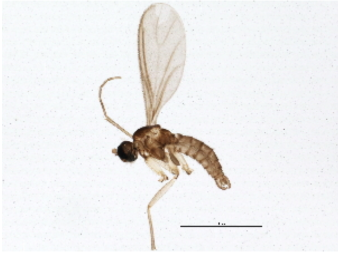

BIOUG02859-A06 [Lateral]
Sciaridae
Family: Sciaridae
BIN URI: BOLD:ACA5184

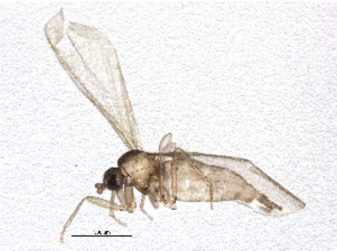

BIOUG07524-A09 [Lateral]
Sciaridae
Family: Sciaridae
BIN URI: BOLD:ACI7288

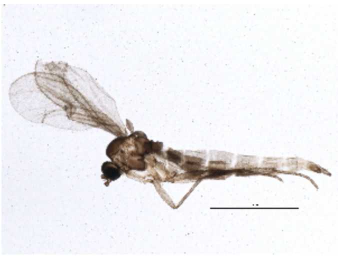

BIOUG10816-A04 [Lateral]
Sciaridae
Family: Sciaridae
BIN URI: BOLD:ACM0501

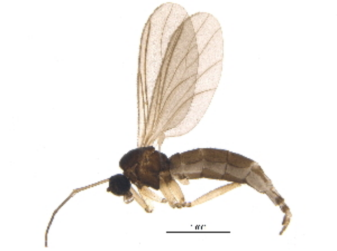

BIOUG22329-H08 [Lateral]
Sciaridae
Family: Sciaridae
BIN URI: BOLD:ACV3335

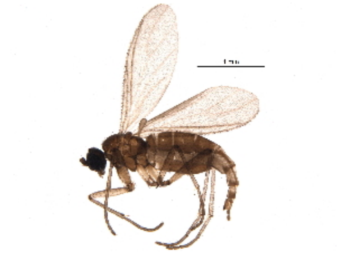

BIOUG10647-B05 [Lateral]
Sciaridae
Family: Sciaridae
BIN URI: BOLD:ACC1288

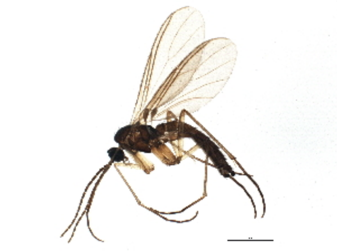

BIOUG04307-G10 [Lateral]
Leptosciarella scutellata
Family: Sciaridae
BIN URI: BOLD:ACD1218

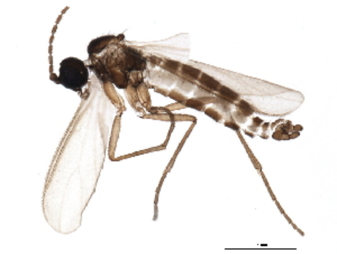

10PHMAL-1172 [Lateral]
Sciaridae
Family: Sciaridae
BIN URI: BOLD:AAU6629

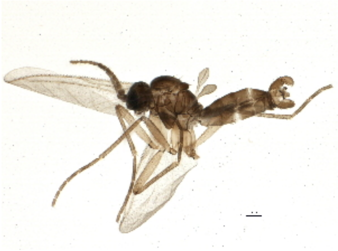

BIOUG11247-G04 [Lateral]
Sciaridae
Family: Sciaridae
BIN URI: BOLD:ACK7034

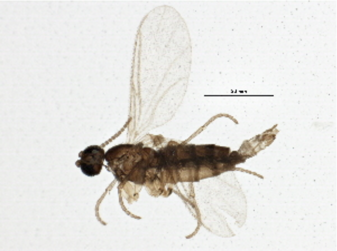

BIOUG03371-G02 [Lateral]
Sciaridae
Family: Sciaridae
BIN URI: BOLD:ABY0363

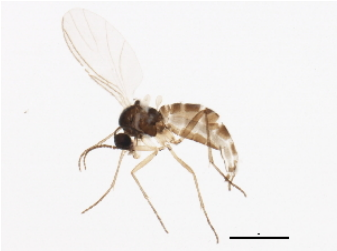

BIOUG01656-F03 [Lateral]
Sciaridae
Family: Sciaridae
BIN URI: BOLD:AAN6438

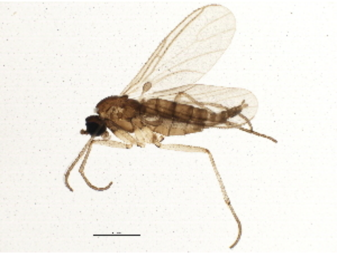

BIOUG02958-E01 [Lateral]
Sciaridae
Family: Sciaridae
BIN URI: BOLD:AAM9228

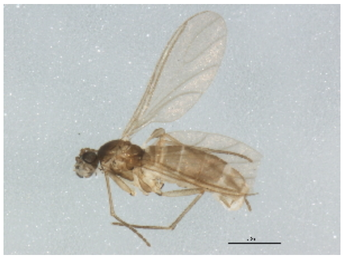

BIOUG01478-E03 [Lateral]
Sciaridae
Family: Sciaridae
BIN URI: BOLD:AAN6442

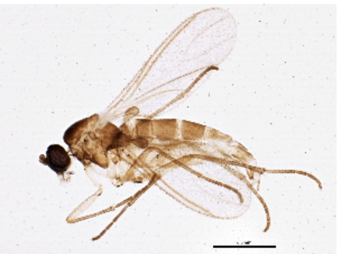

BIOUG01609-A08 [Lateral]
Sciaridae
Family: Sciaridae
BIN URI: BOLD:AAN6452

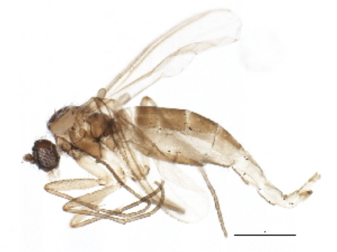

BIOUG01117-D12 [Lateral]
Sciaridae
Family: Sciaridae
BIN URI: BOLD:AAU6513

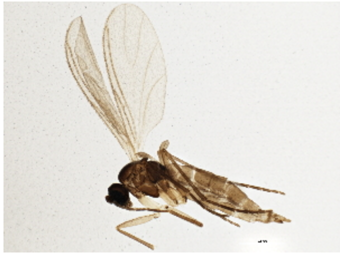

BIOUG03156-D09 [Lateral]
Sciaridae
Family: Sciaridae
BIN URI: BOLD:AAP8784

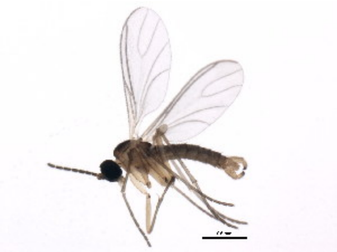

BIOUG00860-D10 [Lateral]
Sciaridae
Family: Sciaridae
BIN URI: BOLD:AAV1302

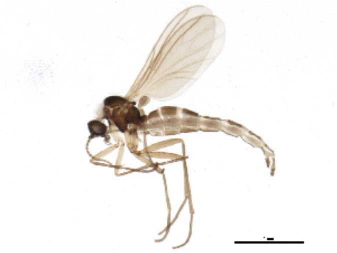

BIOUG01664-H03 [Lateral]
Sciaridae
Family: Sciaridae
BIN URI: BOLD:AAN6445

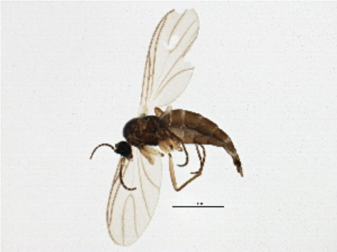

BIOUG02987-C06 [Lateral]
Lycoriella castanescens
Family: Sciaridae
BIN URI: BOLD:ABA1215

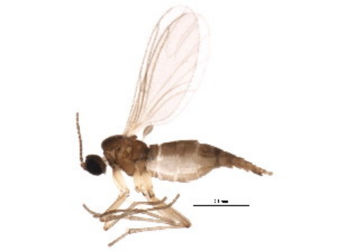

BIOUG01423-D01 [Lateral]
Sciaridae
Family: Sciaridae
BIN URI: BOLD:AAN6447

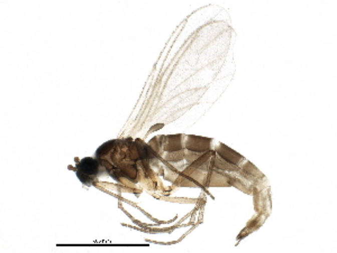

BIOUG22459-A03 [Lateral]
Sciaridae
Family: Sciaridae
BIN URI: BOLD:AAU6595

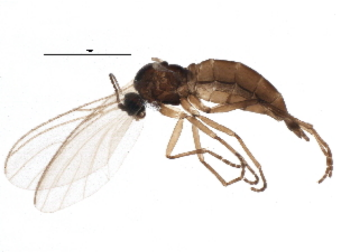

BIOUG07887-E12 [Lateral]
Scatopsciara atomaria
Family: Sciaridae
BIN URI: BOLD:AAH3920

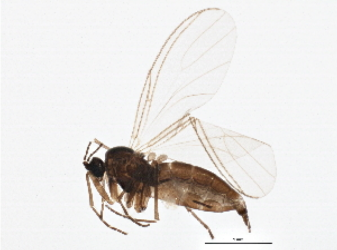

BIOUG02823-D08 [Lateral]
Scatopsciara
Family: Sciaridae
BIN URI: BOLD:AAN6431

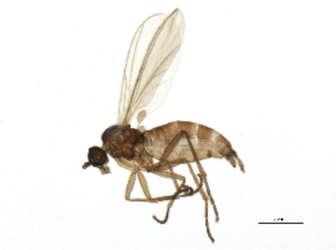

BIOUG00898-H06 [Lateral]
Sciaridae
Family: Sciaridae
BIN URI: BOLD:AAH3951

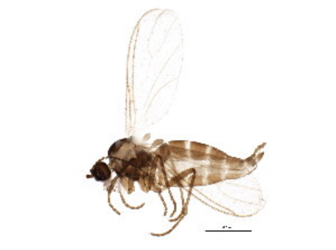

BIOUG01662-G03 [Lateral]
Sciaridae
Family: Sciaridae
BIN URI: BOLD:AAQ2559

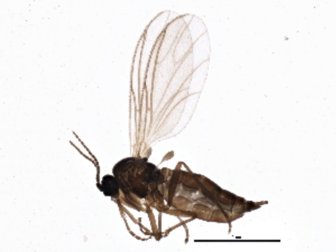

BIOUG05516-H07 [Lateral]
Sciaridae
Family: Sciaridae
BIN URI: BOLD:ABV1265

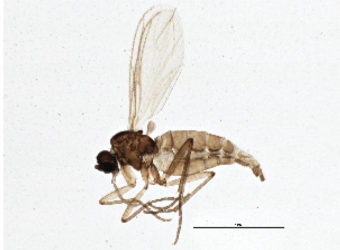

BIOUG04309-C09 [Lateral]
Sciaridae
Family: Sciaridae
BIN URI: BOLD:ACD0200

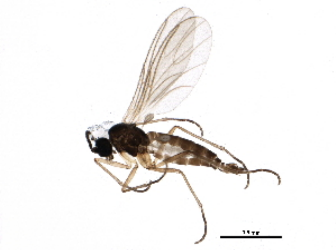

BIOUG22366-G05 [Lateral]
Sciaridae
Family: Sciaridae
BIN URI: BOLD:ACG8695

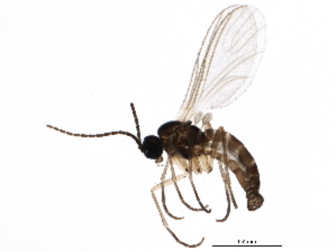

BIOUG22366-F09 [Lateral]
Lycoriella
Family: Sciaridae
BIN URI: BOLD:ACG9235

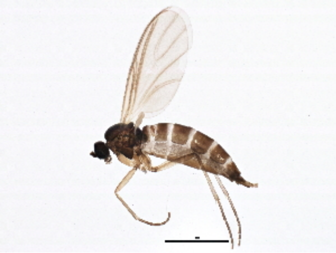

BIOUG05514-D03 [Lateral]
Sciaridae
Family: Sciaridae
BIN URI: BOLD:ABA6415

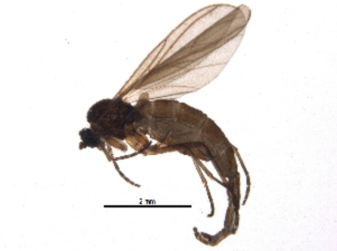

BIOUG10606-E06 [Lateral]
Sciaridae
Family: Sciaridae
BIN URI: BOLD:ACL6671

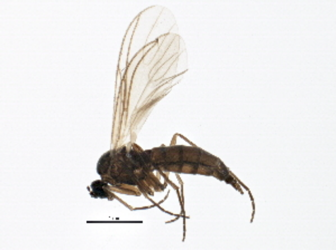

BIOUG05508-B05 [Lateral]
Sciaridae
Family: Sciaridae
BIN URI: BOLD:ACR7948

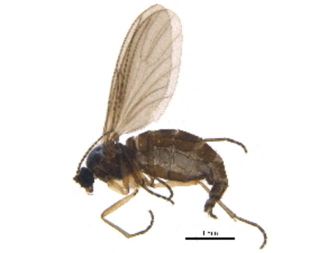

BIOUG22323-G03 [Lateral]
Sciaridae
Family: Sciaridae
BIN URI: BOLD:ACV4024

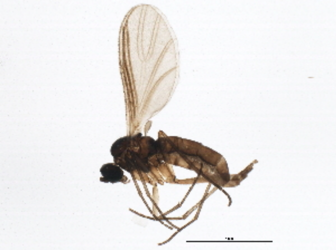

BIOUG02750-H07 [Lateral]
Sciaridae
Family: Sciaridae
BIN URI: BOLD:ACA5246

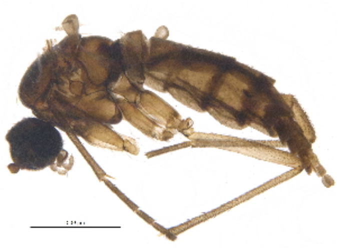

BIOUG20500-D03 [Lateral]
Diptera

BIN URI: BOLD:ACU3044

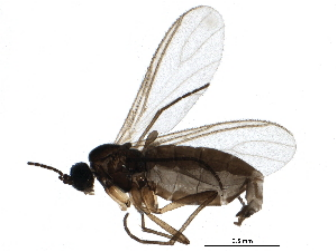

BIOUG21894-F05 [Lateral]
Sciaridae
Family: Sciaridae
BIN URI: BOLD:ACV5075

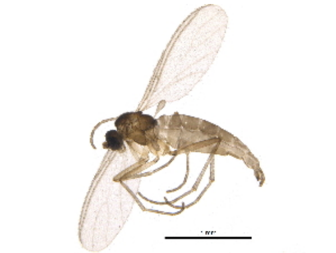

BIOUG22719-D09 [Lateral]
Sciaridae
Family: Sciaridae
BIN URI: BOLD:ACV4812

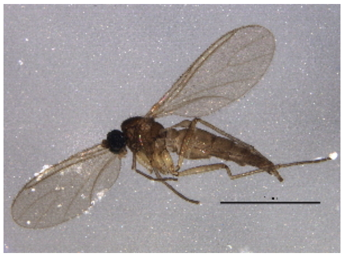

BIOUG09938-A05 [Lateral]
Sciaridae
Family: Sciaridae
BIN URI: BOLD:ACL3441

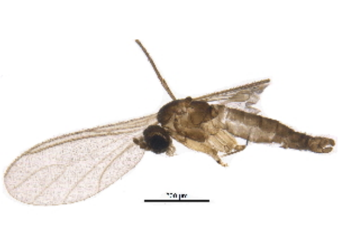

BIOUG21333-E03 [Lateral]
Diptera

BIN URI: BOLD:AAN6440

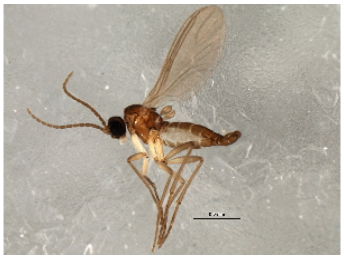

08TTML-1623 [Lateral]
Sciaridae
Family: Sciaridae
BIN URI: BOLD:AAN6433

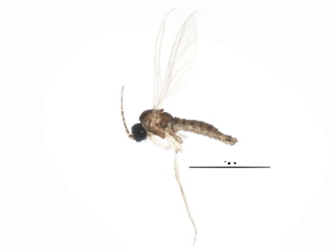

10JSROW-1092 [Lateral]
Sciaridae
Family: Sciaridae
BIN URI: BOLD:AAP1834

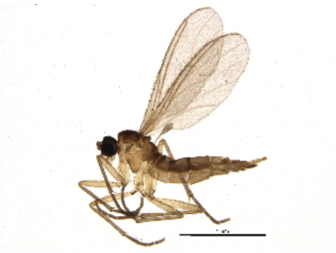

BIOUG05873-F11 [Lateral]
Sciaridae
Family: Sciaridae
BIN URI: BOLD:ACE0982

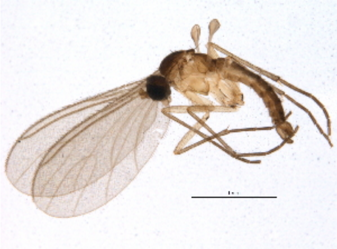

BIOUG04666-F06 [Lateral]
Sciaridae
Family: Sciaridae
BIN URI: BOLD:ACE1034

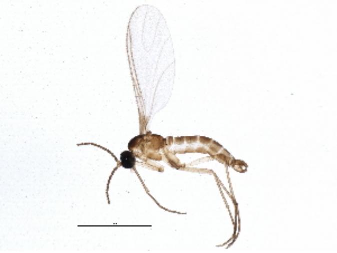

BIOUG06518-C06 [Lateral]
Sciaridae
Family: Sciaridae
BIN URI: BOLD:ABY2036

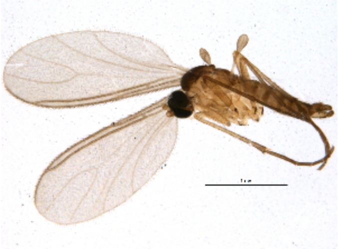

BIOUG04666-F03 [Lateral]
Sciaridae
Family: Sciaridae
BIN URI: BOLD:ACE0960

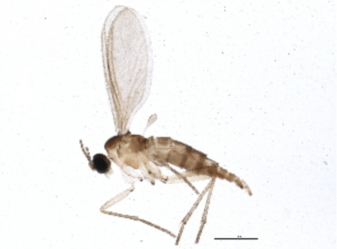

BIOUG10815-F10 [Lateral]
Sciaridae
Family: Sciaridae
BIN URI: BOLD:ACE0959

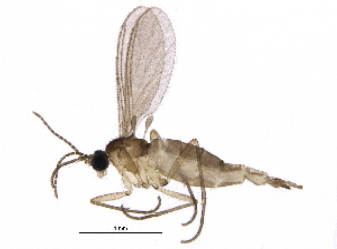

BIOUG09937-G12 [Lateral]
Sciaridae
Family: Sciaridae
BIN URI: BOLD:ACL3134

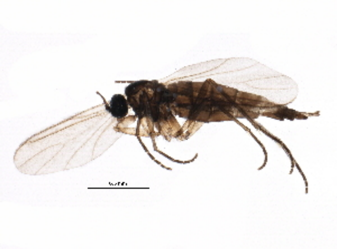

BIOUG22861-D10 [Lateral]
Sciaridae
Family: Sciaridae
BIN URI: BOLD:ACV5524

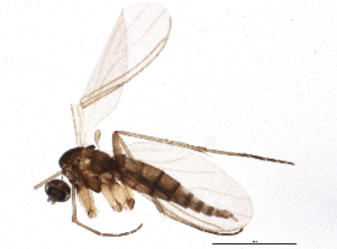

BIOUG04090-E04 [Lateral]
Sciaridae
Family: Sciaridae
BIN URI: BOLD:ACA4924

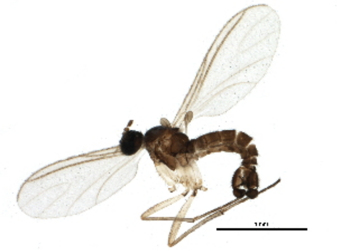

BIOUG08738-E08 [Lateral]
Sciaridae
Family: Sciaridae
BIN URI: BOLD:ACK2160

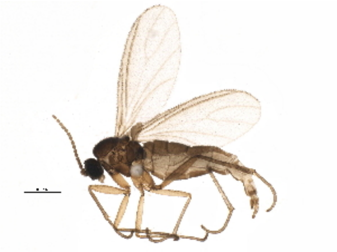

BIOUG08875-D03 [Lateral]
Sciaridae
Family: Sciaridae
BIN URI: BOLD:ACK7315

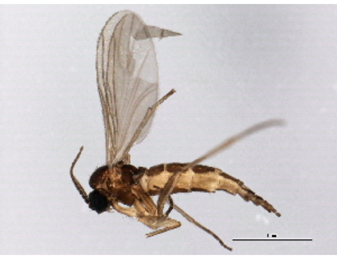

08TTML-2263 [Lateral]
Sciaridae
Family: Sciaridae
BIN URI: BOLD:AAN6446

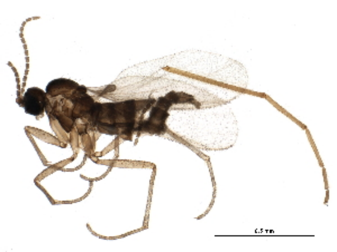

BIOUG22299-E03 [Lateral]
Sciaridae
Family: Sciaridae
BIN URI: BOLD:ACA3384

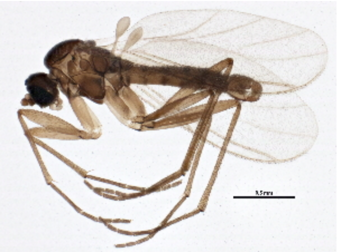

BIOUG02753-A10 [Lateral]
Sciaridae
Family: Sciaridae
BIN URI: BOLD:ACA4900

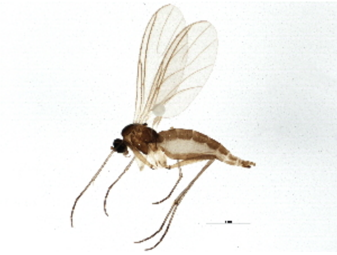

BIOUG04309-G08 [Lateral]
Sciaridae
Family: Sciaridae
BIN URI: BOLD:AAP1210

IMAGE NOT AVAILABLE

BIOUG22732-E01
Sciaridae
Family: Sciaridae

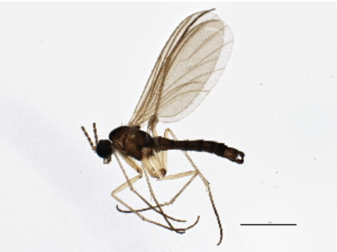

BIOUG05826-H08 [Lateral]
Sciaridae
Family: Sciaridae
BIN URI: BOLD:ACB8873

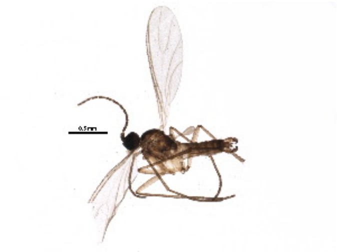

BIOUG21207-G04 [Lateral]
Diptera

BIN URI: BOLD:ACU7230

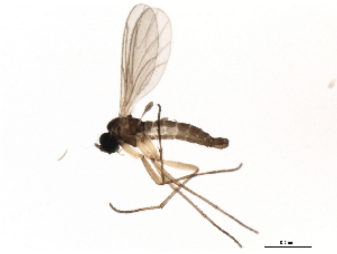

10JSROW-1722 [Lateral]
Sciaridae
Family: Sciaridae
BIN URI: BOLD:AAM9242

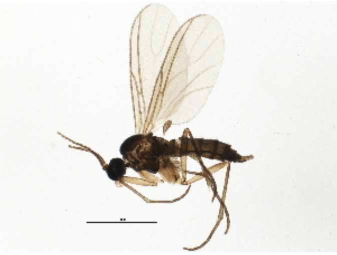

BIOUG02936-E01 [Lateral]
Bradysia difformis
Family: Sciaridae
BIN URI: BOLD:AAV1295

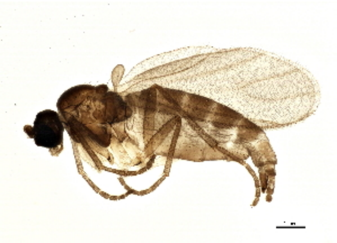

BIOUG03248-H04 [Lateral]
Sciaridae
Family: Sciaridae
BIN URI: BOLD:ABV1277

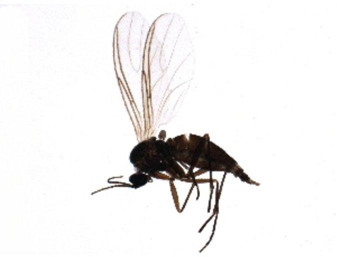

BIOUG22084-C04 [Lateral]
Bradysia vagans
Family: Sciaridae
BIN URI: BOLD:AAM9252

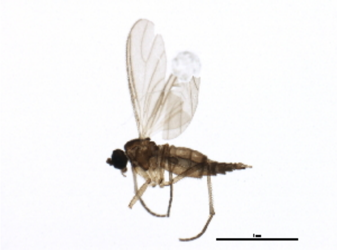

10PHMAL-3054 [Lateral]
Sciaridae
Family: Sciaridae
BIN URI: BOLD:AAN6435

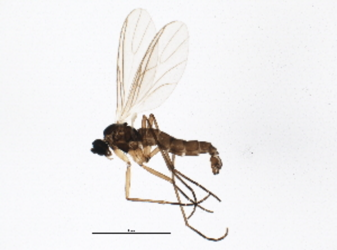

BIOUG05505-H10 [Lateral]
Sciaridae
Family: Sciaridae
BIN URI: BOLD:ABA1231

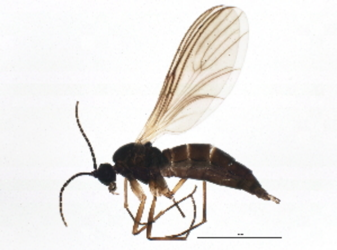

BIOUG05591-F05 [Lateral]
Sciaridae
Family: Sciaridae
BIN URI: BOLD:ABW1417

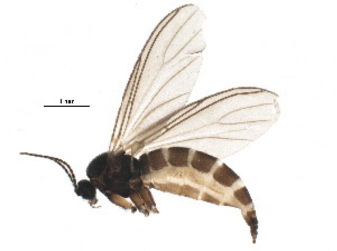

BIOUG22359-H06 [Lateral]
Bradysia pallipes
Family: Sciaridae
BIN URI: BOLD:AAM9254

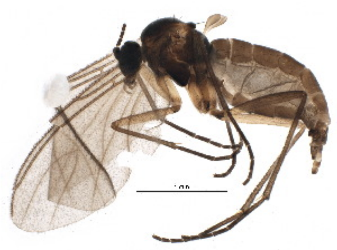

BIOUG22419-A01 [Lateral]
Sciaridae
Family: Sciaridae
BIN URI: BOLD:AAH4013

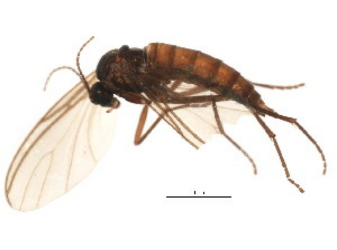

BIOUG01914-H10 [Lateral]
Sciaridae
Family: Sciaridae
BIN URI: BOLD:AAH3910

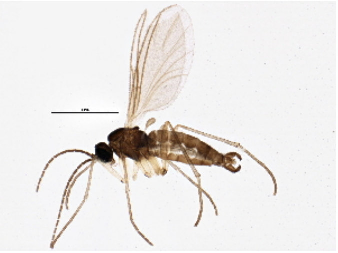

BIOUG02631-E03 [Lateral]
Sciaridae
Family: Sciaridae
BIN URI: BOLD:AAN6429

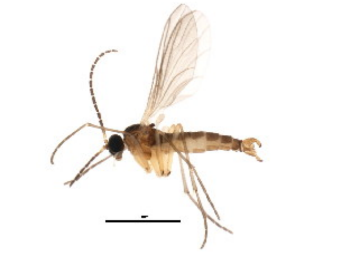

BIOUG01553-A02 [Lateral]
Sciaridae
Family: Sciaridae
BIN URI: BOLD:ABA0929

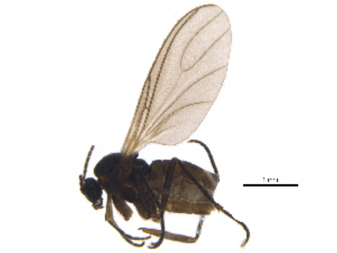

BIOUG22323-C01 [Lateral]
Sciaridae
Family: Sciaridae
BIN URI: BOLD:ACV2795

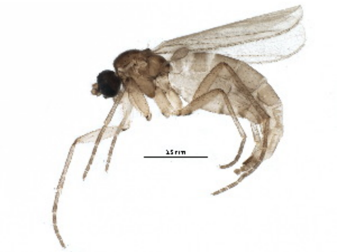

BIOUG22291-H08 [Lateral]
Lycoriella stylata
Family: Sciaridae
BIN URI: BOLD:AAN6430

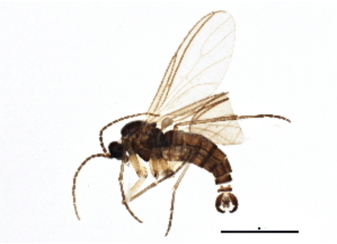

BIOUG03288-C09 [Lateral]
Sciaridae
Family: Sciaridae
BIN URI: BOLD:ACA9720

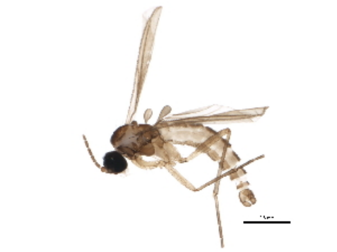

BIOUG01299-A05 [Lateral]
Sciaridae
Family: Sciaridae
BIN URI: BOLD:AAU6537

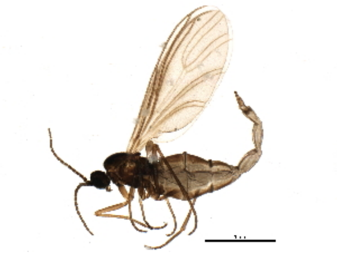

BIOUG08689-C07 [Lateral]
Sciaridae
Family: Sciaridae
BIN URI: BOLD:ACM2497

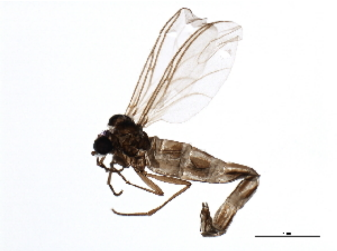

10PHMAL-1457 [Lateral]
Sciaridae
Family: Sciaridae
BIN URI: BOLD:AAU6615

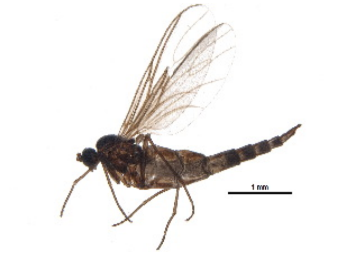

BIOUG22330-H06 [Lateral]
Sciaridae
Family: Sciaridae
BIN URI: BOLD:AAU6615

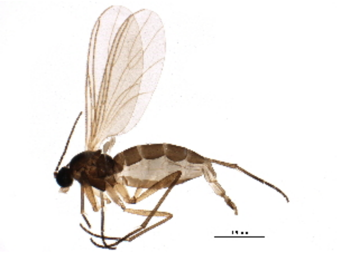

BIOUG13237-B05 [Lateral]
Sciaridae
Family: Sciaridae
BIN URI: BOLD:AAH3983

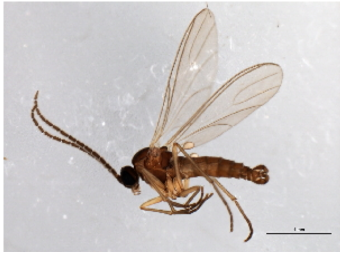

08TTML-2214 [Lateral]
Sciaridae
Family: Sciaridae
BIN URI: BOLD:ACR4350

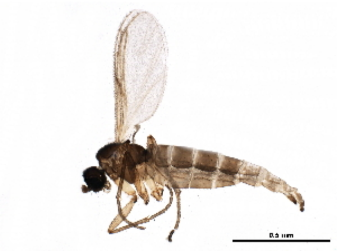

BIOUG22364-F12 [Lateral]
Sciaridae
Family: Sciaridae
BIN URI: BOLD:ACV4127

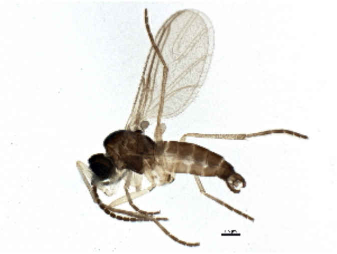

BIOUG04460-A12 [Lateral]
Sciaridae
Family: Sciaridae
BIN URI: BOLD:ACD3278

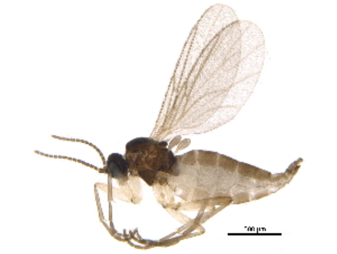

BIOUG22329-C12 [Lateral]
Sciaridae
Family: Sciaridae
BIN URI: BOLD:ABV1443

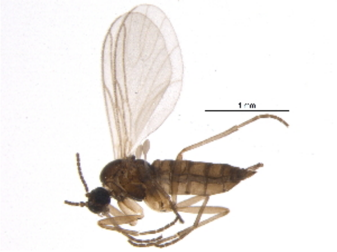

BIOUG05640-C03 [Lateral]
Sciaridae
Family: Sciaridae
BIN URI: BOLD:ABV1201

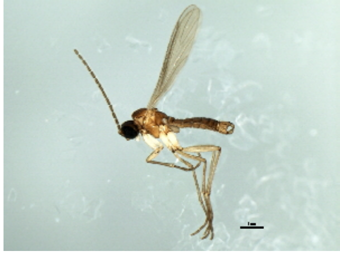

08TTML-1238 [Lateral]
Sciaridae
Family: Sciaridae
BIN URI: BOLD:AAN6437

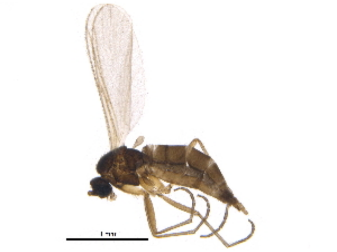

BIOUG22328-C12 [Lateral]
Sciaridae
Family: Sciaridae
BIN URI: BOLD:AAY6412

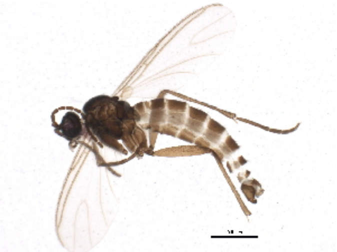

BIOUG01444-D03 [Lateral]
Sciaridae
Family: Sciaridae
BIN URI: BOLD:AAZ5626

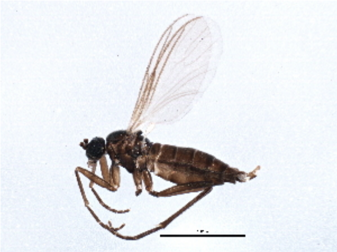

BIOUG05546-B10 [Lateral]
Sciaridae
Family: Sciaridae
BIN URI: BOLD:AAV1261

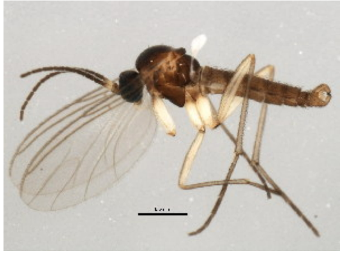

BIOUG01543-H04 [Lateral]
Sciaridae
Family: Sciaridae
BIN URI: BOLD:AAN6444

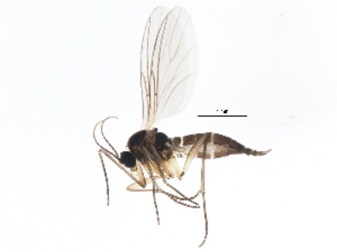

10JSROW-1558 [Lateral]
Sciaridae
Family: Sciaridae
BIN URI: BOLD:AAV1366

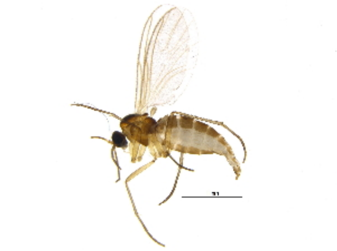

BIOUG02308-B08 [Lateral]
Sciaridae
Family: Sciaridae
BIN URI: BOLD:AAH3947

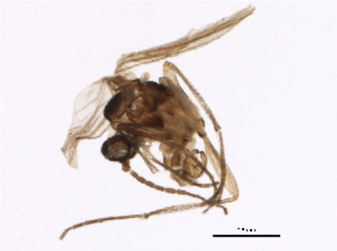

BIOUG01752-G07 [Lateral]
Sciaridae
Family: Sciaridae
BIN URI: BOLD:ACE7580

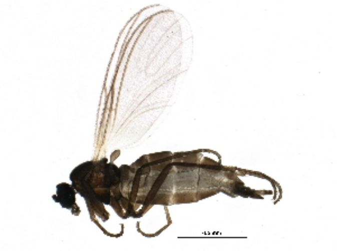

BIOUG22365-F03 [Lateral]
Bradysia
Family: Sciaridae
BIN URI: BOLD:AAM9243

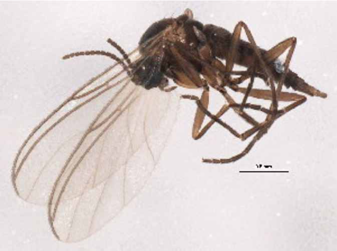

BIOUG01460-A04 [Lateral]
Sciaridae
Family: Sciaridae
BIN URI: BOLD:ABA6471

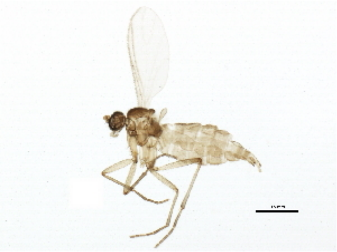

BIOUG01449-G10 [Lateral]
Sciaridae
Family: Sciaridae
BIN URI: BOLD:AAU6542

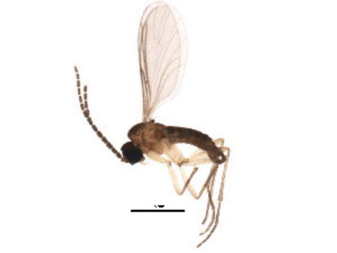

BIOUG01423-C11 [Lateral]
Sciaridae
Family: Sciaridae
BIN URI: BOLD:ABU5521

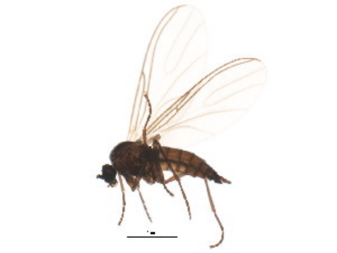

08WOLVES-01300 [Lateral]
Sciaridae
Family: Sciaridae
BIN URI: BOLD:AAU6764

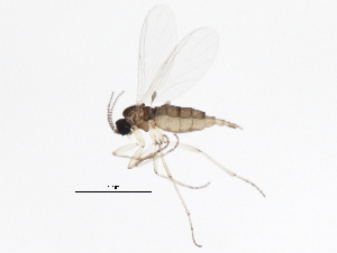

10JSROW-1590 [Lateral]
Sciaridae
Family: Sciaridae
BIN URI: BOLD:AAU6622

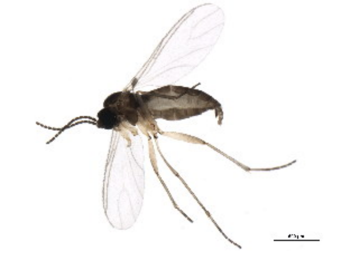

BIOUG01348-E06 [Lateral]
Sciaridae
Family: Sciaridae
BIN URI: BOLD:ACE3123

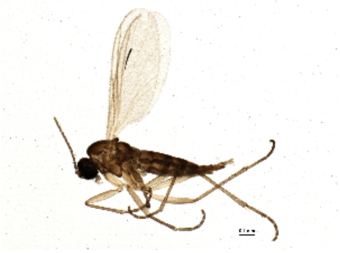

BIOUG02965-F06 [Lateral]
Sciaridae
Family: Sciaridae
BIN URI: BOLD:ABU5520

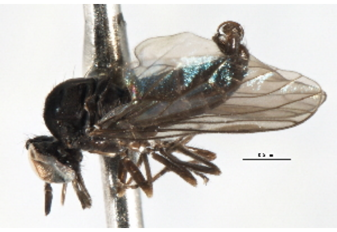

CNC DIPTERA 103398 [Lateral]
Iteaphila nitidula
Family: Empididae
BIN URI: BOLD:AAF9884

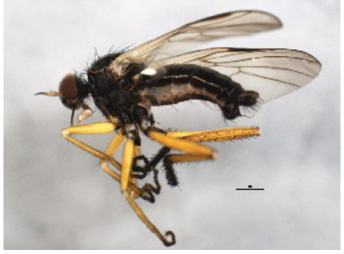

10JSROW-1205 [Lateral]
Rhamphomyia
Family: Empididae
BIN URI: BOLD:AAP6354

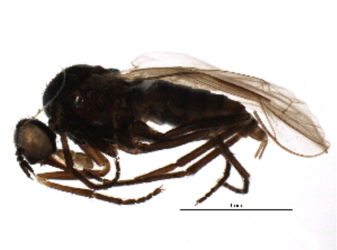

BIOUG10645-D05 [Lateral]
Rhamphomyia
Family: Empididae
BIN URI: BOLD:ACV2134

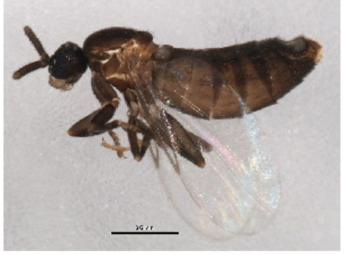

BIOUG01423-E05 [Lateral]
Scatopsidae
Family: Scatopsidae
BIN URI: BOLD:AAN8523

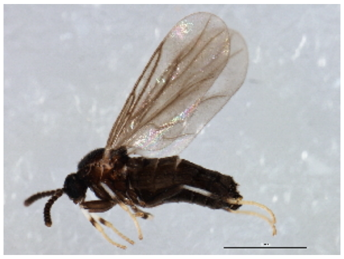

08TTML-1162 [Lateral]
Scatopsidae
Family: Scatopsidae
BIN URI: BOLD:AAH4123

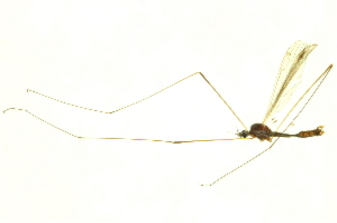

10BBTIP-0002 [Lateral]
Limoniidae
Family: Limoniidae
BIN URI: BOLD:AAO3939

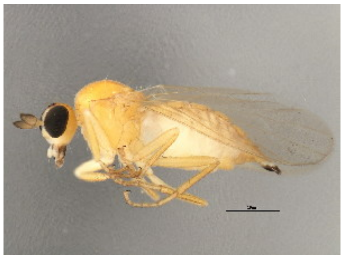

10JSROW-0857 [Lateral]
Allanthalia sp.
Family: Hybotidae
BIN URI: BOLD:AAL8961

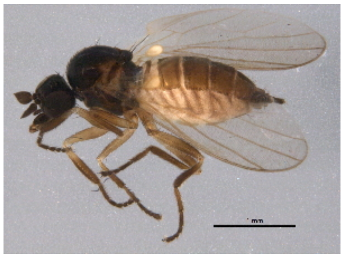

BIOUG07522-G06 [Lateral]
Anthalia sp. 5
Family: Hybotidae
BIN URI: BOLD:ACA7284

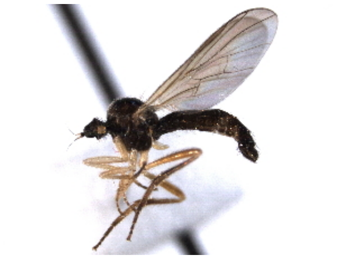

JSS21187 [Lateral]
Leptopeza compta
Family: Hybotidae
BIN URI: BOLD:AAF9779

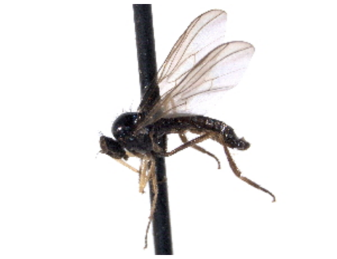

CNC DIPTERA 161673 [Lateral]
Leptopeza disparilia
Family: Hybotidae
BIN URI: BOLD:AAF9791

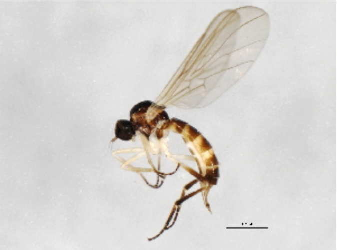

09BBEDI-0872 [Lateral]
Leptopeza flavipes
Family: Hybotidae
BIN URI: BOLD:ACE5974

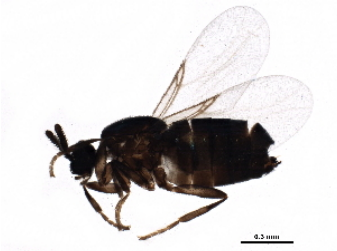

BIOUG22364-G04 [Lateral]
Scatopsidae
Family: Scatopsidae
BIN URI: BOLD:ACV4134

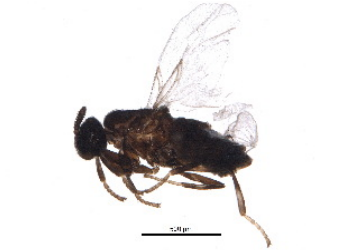

BIOUG22351-E11 [Lateral]
Scatopsidae
Family: Scatopsidae
BIN URI: BOLD:AAV1136

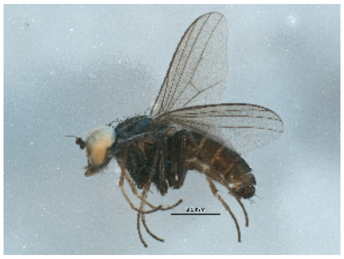

BIOUG12201-F06 [Lateral]
Dolichopodidae
Family: Dolichopodidae
BIN URI: BOLD:ACN2360

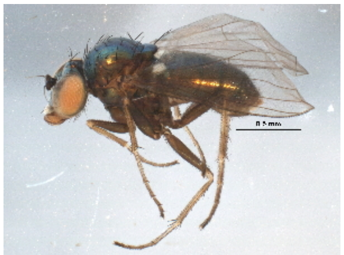

BIOUG12201-G08 [Lateral]
Dolichopodidae
Family: Dolichopodidae
BIN URI: BOLD:ACR8420

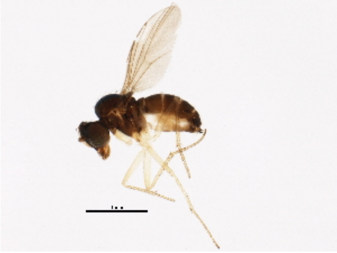

BIOUG03024-A01 [Lateral]
Dolichopodidae
Family: Dolichopodidae
BIN URI: BOLD:ACA6204

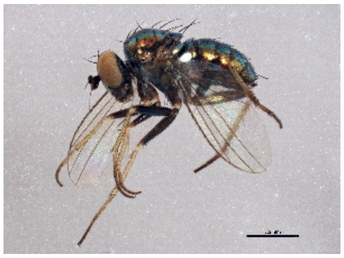

BIOUG02965-H09 [Lateral]
Chrysotus
Family: Dolichopodidae
BIN URI: BOLD:AAG9668

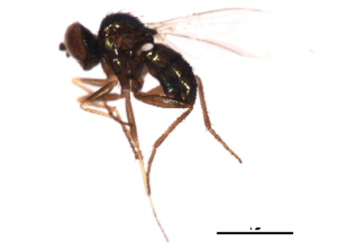

09BBEDI-2784 [Lateral]
Dolichopodidae
Family: Dolichopodidae
BIN URI: BOLD:AAN5531

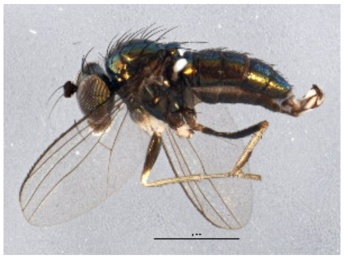

BIOUG01687-C07 [Lateral]
Chrysotus
Family: Dolichopodidae
BIN URI: BOLD:AAV3883

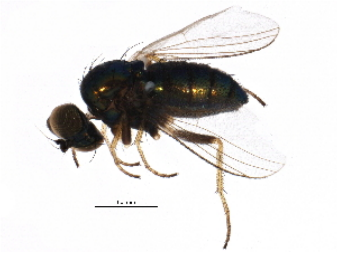

BIOUG22732-G09 [Lateral]
Chrysotus
Family: Dolichopodidae
BIN URI: BOLD:ACV5249

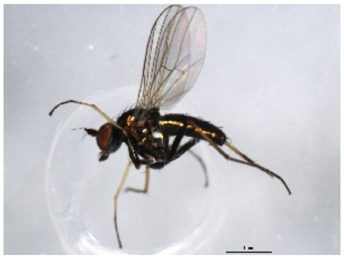

10BBDIP-1674 [Lateral]
Dolichopodidae
Family: Dolichopodidae
BIN URI: BOLD:AAG9699

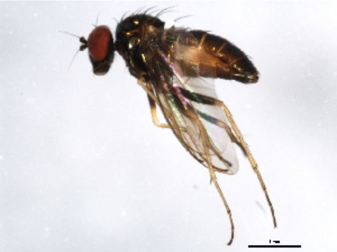

10BBDIP-2082 [Lateral]
Dolichopodidae
Family: Dolichopodidae
BIN URI: BOLD:AAP5019

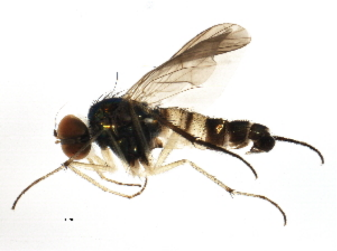

BIOUG21007-A12 [Lateral]
Diptera

BIN URI: BOLD:ACU9078

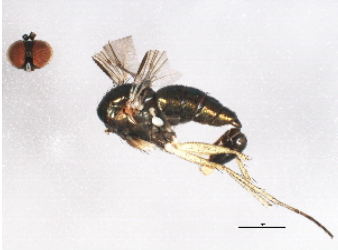

BIOUG04259-F09 [Lateral]
Gymnopternus celer
Family: Dolichopodidae
BIN URI: BOLD:ACB3156

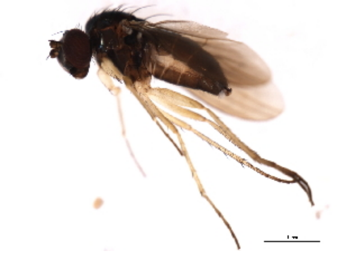

08TTML-0945 [Lateral]
Dolichopodidae
Family: Dolichopodidae
BIN URI: BOLD:AAM6783

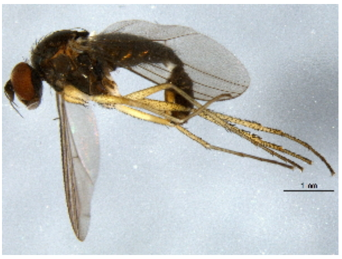

BIOUG03010-D02 [Lateral]
Dolichopodidae
Family: Dolichopodidae
BIN URI: BOLD:ACB1149

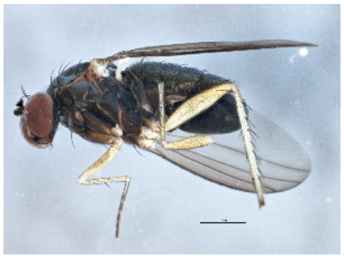

BIOUG04288-F11 [Lateral]
Dolichopodidae
Family: Dolichopodidae
BIN URI: BOLD:ACD8765

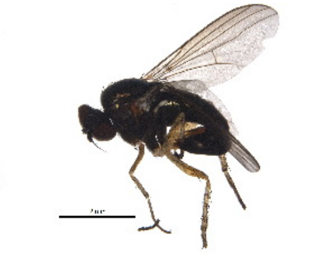

BIOUG22844-E04 [Lateral]
Gymnopternus
Family: Dolichopodidae
BIN URI: BOLD:ACW0834

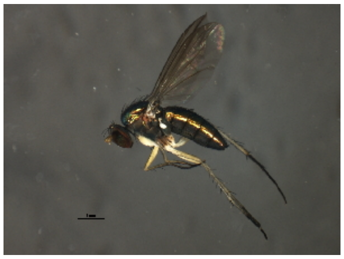

08BBDIP-2448 [Lateral]
Dolichopus
Family: Dolichopodidae
BIN URI: BOLD:AAG9652

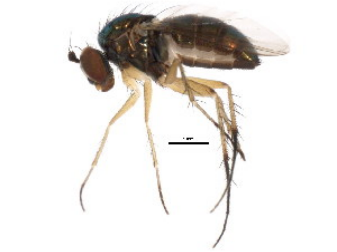

BIOUG01411-D11 [Lateral]
Dolichopodidae
Family: Dolichopodidae
BIN URI: BOLD:AAV3887

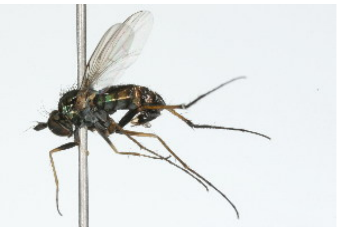

09BBDIP-0265 [Lateral]
Dolichopodidae
Family: Dolichopodidae
BIN URI: BOLD:AAG9691

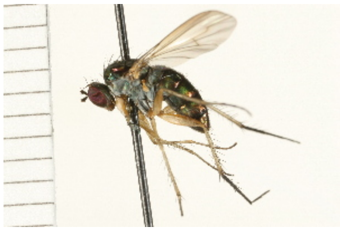

08MZPP-106 [Lateral]
Dolichopodidae
Family: Dolichopodidae
BIN URI: BOLD:AAG9641

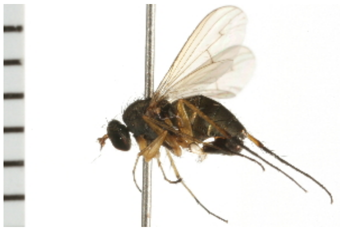

08BBDIP-0006 [Lateral]
Dolichopodidae
Family: Dolichopodidae
BIN URI: BOLD:AAG9626

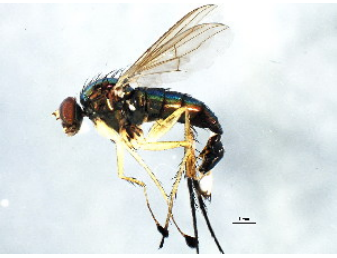

08TTML-2538 [Lateral]
Dolichopodidae
Family: Dolichopodidae
BIN URI: BOLD:AAG9629

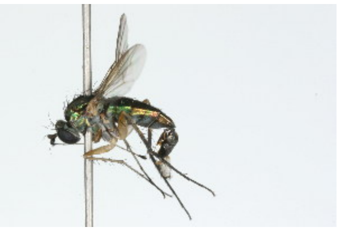

09BBDIP-0264 [Lateral]
Dolichopus
Family: Dolichopodidae
BIN URI: BOLD:AAG9690

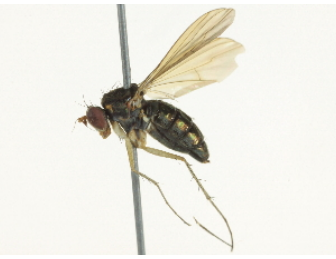

BIOUG03681-E05 [Lateral]
Dolichopodidae
Family: Dolichopodidae
BIN URI: BOLD:ABA4798

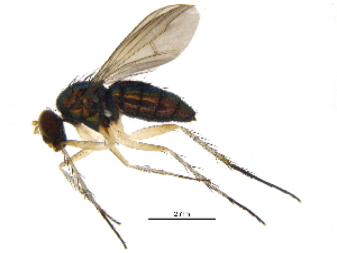

BIOUG21529-B08 [Lateral]
Diptera

BIN URI: BOLD:ACU7943

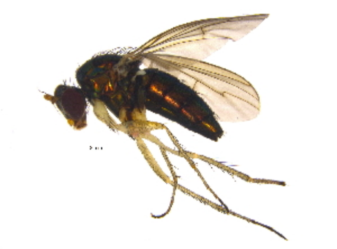

BIOUG01604-E04 [Lateral]
Dolichopodidae
Family: Dolichopodidae
BIN URI: BOLD:AAG9657

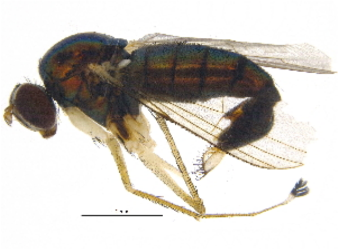

BIOUG16076-G10 [Lateral]
Dolichopodidae
Family: Dolichopodidae
BIN URI: BOLD:ACU5946

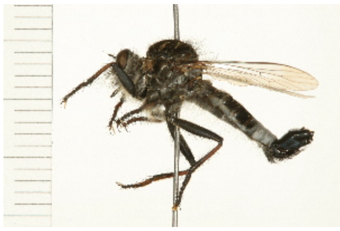

DIPT 0041.02 [Lateral]
Asilidae
Family: Asilidae
BIN URI: BOLD:AAH2306

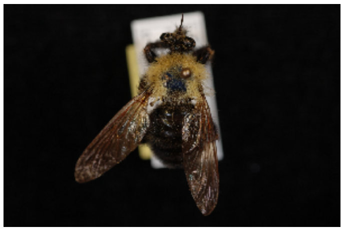

10-SKAS-088 [Dorsal]
Laphria thoracica
Family: Asilidae
BIN URI: BOLD:AAW9901

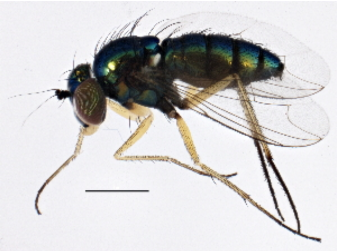

BIOUG03138-F07 [Lateral]
Dolichopodidae
Family: Dolichopodidae
BIN URI: BOLD:AAD2041

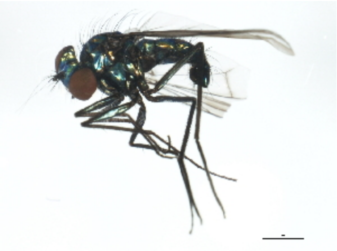

08BBDIP-0787 [Lateral]
Dolichopodidae
Family: Dolichopodidae
BIN URI: BOLD:AAG9640

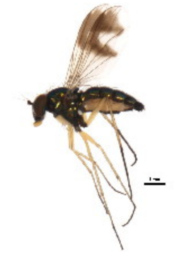

BIOUG01408-A08 [Lateral]
Dolichopodidae
Family: Dolichopodidae
BIN URI: BOLD:AAG9693

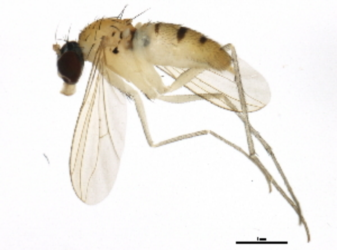

BIOUG01121-E02 [Lateral]
Dolichopodidae
Family: Dolichopodidae
BIN URI: BOLD:AAQ0271

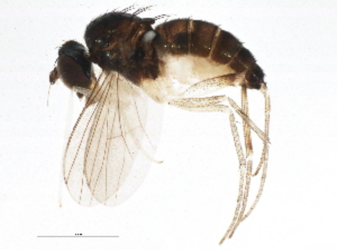

BIOUG03829-D02 [Lateral]
Dolichopodidae
Family: Dolichopodidae
BIN URI: BOLD:ACC8120

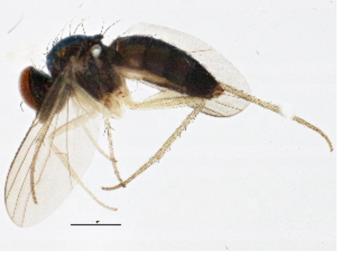

BIOUG03261-H04 [Lateral]
Dolichopodidae
Family: Dolichopodidae
BIN URI: BOLD:AAG9744

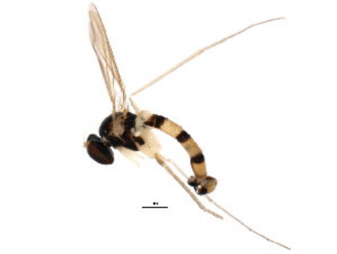

BIOUG01459-D10 [Lateral]
Dolichopodidae
Family: Dolichopodidae
BIN URI: BOLD:ABW1193

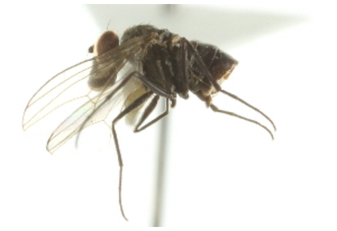

CNC DIPTERA 105362 [Lateral]
Medetera signaticornis
Family: Dolichopodidae
BIN URI: BOLD:AAZ3931

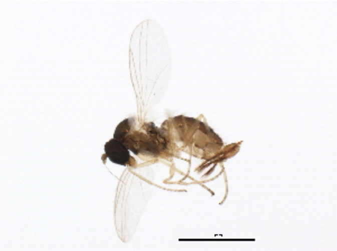

BIOUG01445-B09 [Lateral]
Dolichopodidae
Family: Dolichopodidae
BIN URI: BOLD:AAN5528

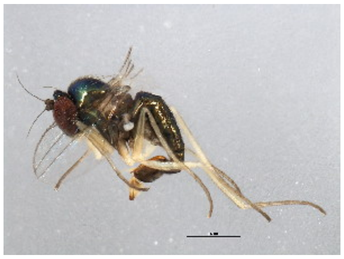

10JSROW-0841 [Lateral]
Dolichopodidae
Family: Dolichopodidae
BIN URI: BOLD:AAU6620

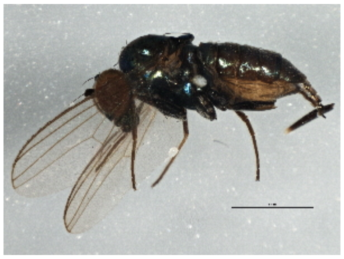

BIOUG03154-D02 [Lateral]
Dolichopodidae
Family: Dolichopodidae
BIN URI: BOLD:AAZ6708

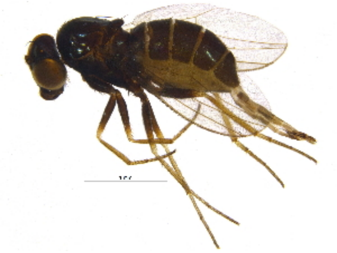

BIOUG02843-G02 [Lateral]
Dolichopodidae
Family: Dolichopodidae
BIN URI: BOLD:ACG9324

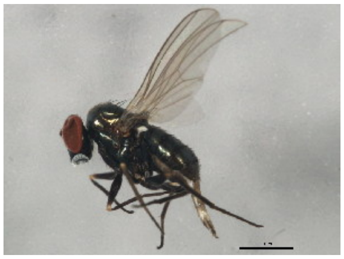

10JSROW-1038 [Lateral]
Dolichopodidae
Family: Dolichopodidae
BIN URI: BOLD:AAP6322

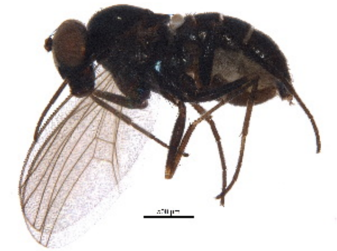

BIOUG22356-C09 [Lateral]
Dolichopodidae
Family: Dolichopodidae
BIN URI: BOLD:AAQ0833

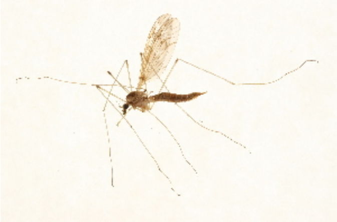

09BBTEC-058 [Lateral]
Tipula (Beringotipula) coloradensis
Family: Tipulidae
BIN URI: BOLD:AAF9052

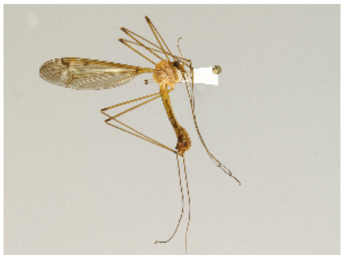

CMNH475159 [Lateral]
Tipula mallochi
Family: Tipulidae
BIN URI: BOLD:AAE0035

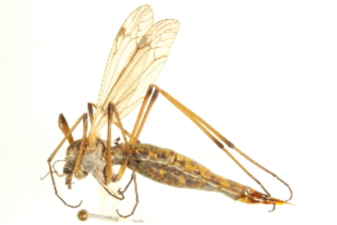

CNC DIPTERA 192300 [Lateral]
Tipula dorsimacula
Family: Tipulidae
BIN URI: BOLD:AAF8990

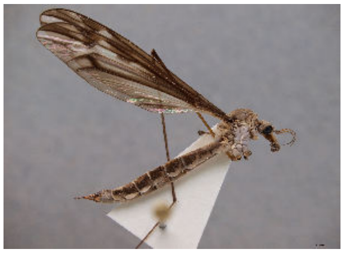

JES-20120069 [Lateral]
Tipula furca
Family: Tipulidae
BIN URI: BOLD:ABX5321

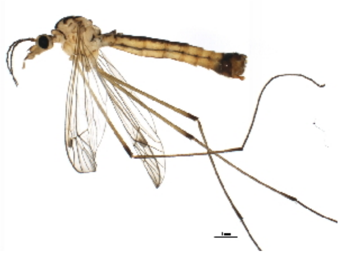

BIOUG01121-C01 [Lateral]
Nephrotoma cornicina
Family: Tipulidae
BIN URI: BOLD:AAZ0266

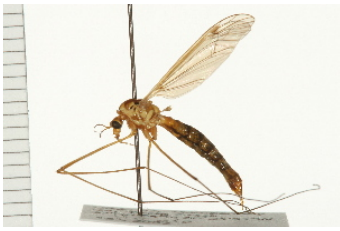

Hcmisc-095 [Lateral]
Tipulidae
Family: Tipulidae
BIN URI: BOLD:ABX6186

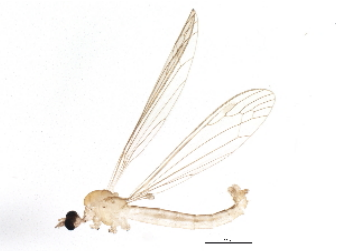

BIOUG02976-A01 [Lateral]
Dicranomyia
Family: Limoniidae
BIN URI: BOLD:ABW4424

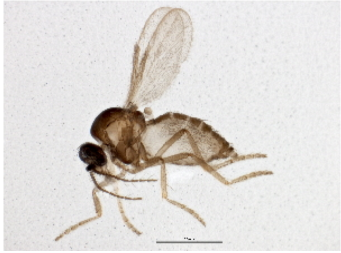

BIOUG03636-F12 [Lateral]
Forcipomyia
Family: Ceratopogonidae
BIN URI: BOLD:AAN5147

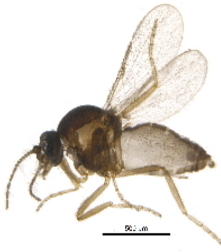

BIOUG22719-E03 [Lateral]
Forcipomyia
Family: Ceratopogonidae
BIN URI: BOLD:ACC7974

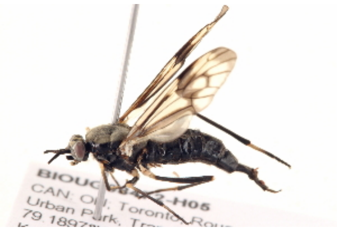

BIOUG08472-H05 [Lateral]
Xylophagus
Family: Xylophagidae
BIN URI: BOLD:AAJ9649

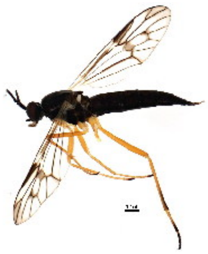

BIOUG00992-F04 [Lateral]
Xylophagus
Family: Xylophagidae
BIN URI: BOLD:AAM7333

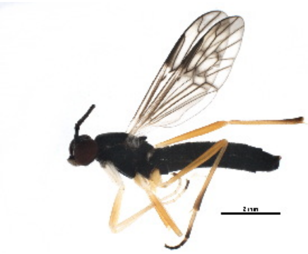

BIOUG21873-D01 [Lateral]
Xylophagus reflectens
Family: Xylophagidae
BIN URI: BOLD:AAP7637

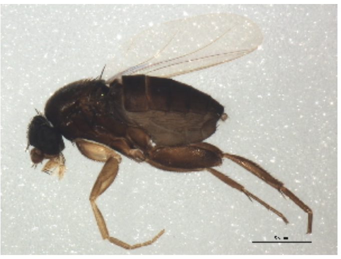

BIOUG01434-C12 [Lateral]
Phoridae
Family: Phoridae
BIN URI: BOLD:AAU6541

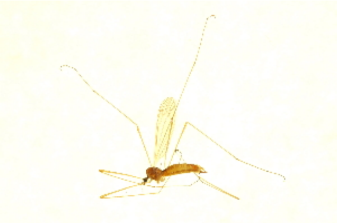

10BBTIP-0224 [Lateral]
Limoniidae
Family: Limoniidae
BIN URI: BOLD:AAV1796

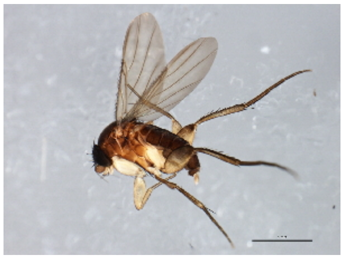

08TTML-1200 [Lateral]
Phoridae
Family: Phoridae
BIN URI: BOLD:AAM7379

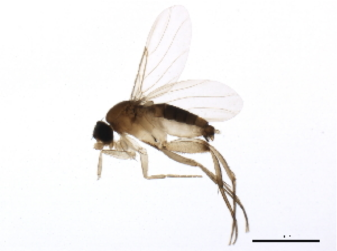

BIOUG01448-E06 [Lateral]
Phoridae
Family: Phoridae
BIN URI: BOLD:AAP6420

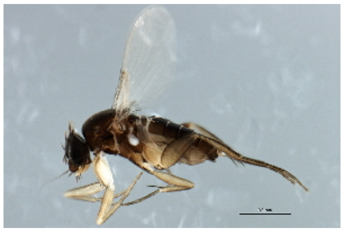

09BBDIP-1742 [Lateral]
Phoridae
Family: Phoridae
BIN URI: BOLD:AAG3286

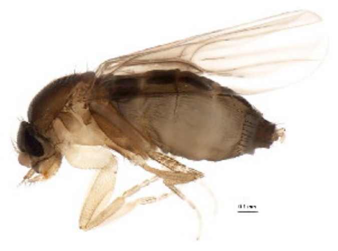

BIOUG01426-H09 [Lateral]
Phoridae
Family: Phoridae
BIN URI: BOLD:AAN8682

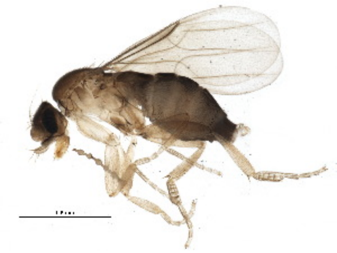

BIOUG21410-D01 [Lateral]
Diptera

BIN URI: BOLD:ACU8783

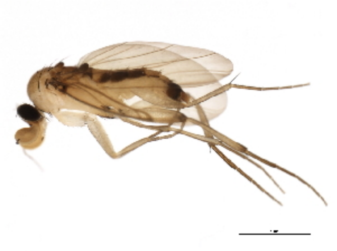

09BBEDI-1844 [Lateral]
Phoridae
Family: Phoridae
BIN URI: BOLD:AAG3338

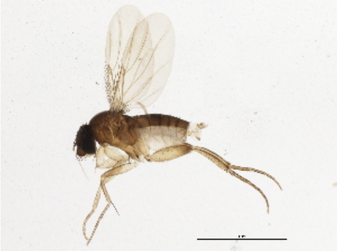

BIOUG02936-G12 [Lateral]
Phoridae
Family: Phoridae
BIN URI: BOLD:AAG3273

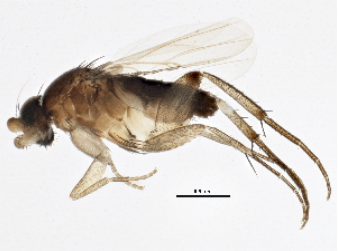

BIOUG03437-C04 [Lateral]
Phoridae
Family: Phoridae
BIN URI: BOLD:AAU5598

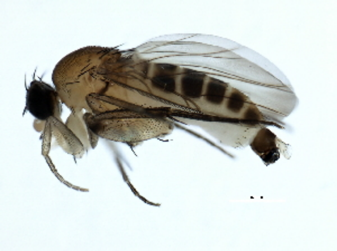

09BBEDI-2391 [Lateral]
Phoridae
Family: Phoridae
BIN URI: BOLD:AAG3320

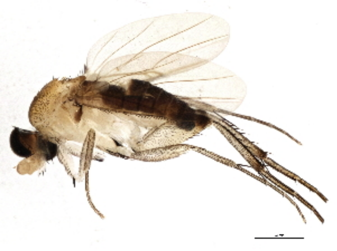

BIOUG02892-A02 [Lateral]
Phoridae
Family: Phoridae
BIN URI: BOLD:AAU6526

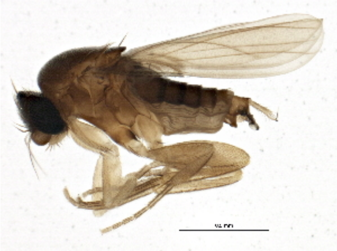

BIOUG02056-C06 [Lateral]
Phoridae
Family: Phoridae
BIN URI: BOLD:ABW5540

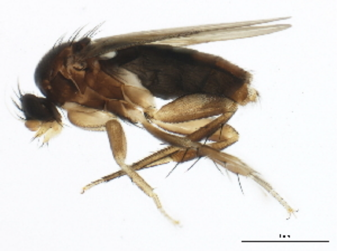

BIOUG01403-D12 [Lateral]
Phoridae
Family: Phoridae
BIN URI: BOLD:ABV3316

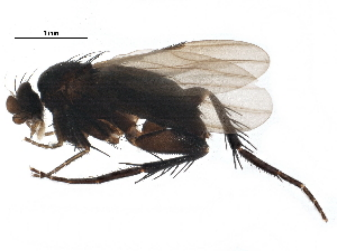

BIOUG21613-F04 [Lateral]
Phoridae
Family: Phoridae
BIN URI: BOLD:ACV2632

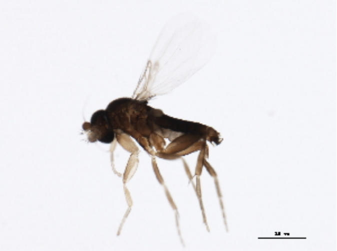

09PROBE-JW0701 [Lateral]
Phoridae
Family: Phoridae
BIN URI: BOLD:AAG3314

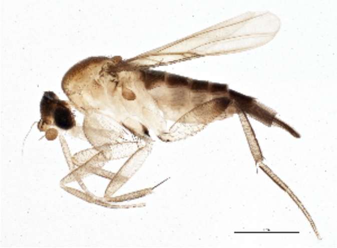

BIOUG02928-G09 [Lateral]
Phoridae
Family: Phoridae
BIN URI: BOLD:ACA4351

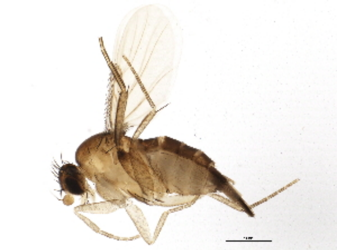

BIOUG02958-C11 [Lateral]
Phoridae
Family: Phoridae
BIN URI: BOLD:AAP4676

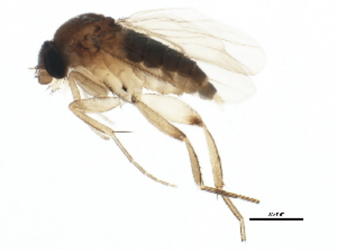

BIOUG01306-A03 [Lateral]
Phoridae
Family: Phoridae
BIN URI: BOLD:AAU6511

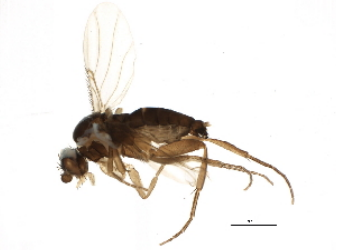

BIOUG01299-G04 [Lateral]
Phoridae
Family: Phoridae
BIN URI: BOLD:AAM9355

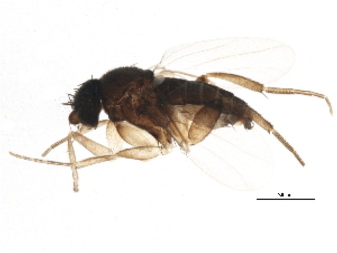

BIOUG01493-D10 [Lateral]
Phoridae
Family: Phoridae
BIN URI: BOLD:ABW8037

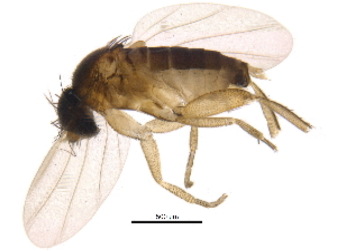

BIOUG22329-B08 [Lateral]
Phoridae
Family: Phoridae
BIN URI: BOLD:AAG3241

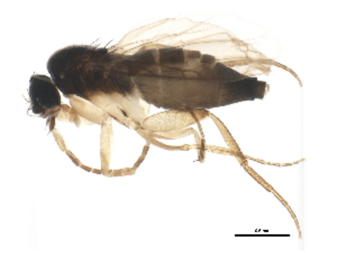

BIOUG01130-C08 [Lateral]
Phoridae
Family: Phoridae
BIN URI: BOLD:AAM9364

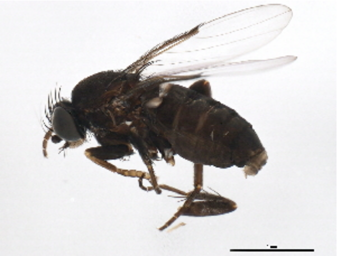

10PHMAL-1216 [Lateral]
Phoridae
Family: Phoridae
BIN URI: BOLD:AAM9376

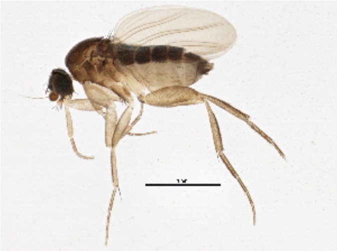

BIOUG03934-C09 [Lateral]
Phoridae
Family: Phoridae
BIN URI: BOLD:ACA6461

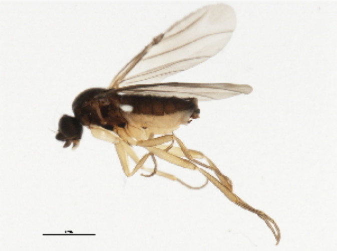

09BBDIP-1288 [Lateral]
Phoridae
Family: Phoridae
BIN URI: BOLD:AAG3292

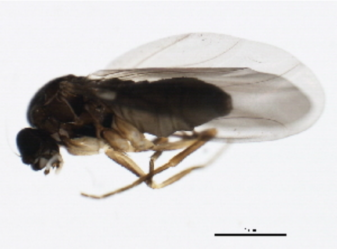

10BBCDIP-0933 [Lateral]
Phoridae
Family: Phoridae
BIN URI: BOLD:AAP8118

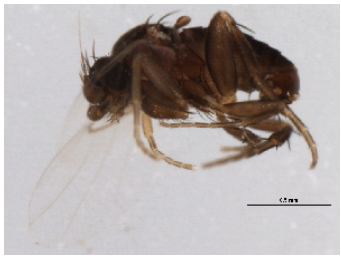

08TTML-2200 [Lateral]
Phoridae
Family: Phoridae
BIN URI: BOLD:AAG3331

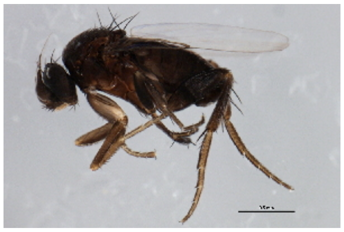

08TTML-0782 [Lateral]
Phoridae
Family: Phoridae
BIN URI: BOLD:AAN8685

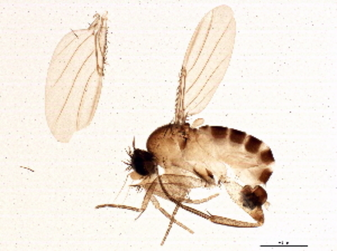

BIOUG02624-F04 [Lateral]
Phoridae
Family: Phoridae
BIN URI: BOLD:AAN8687

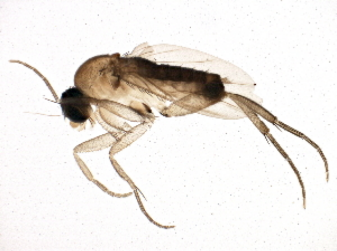

BIOUG02511-E08 [Lateral]
Phoridae
Family: Phoridae
BIN URI: BOLD:ACT8182

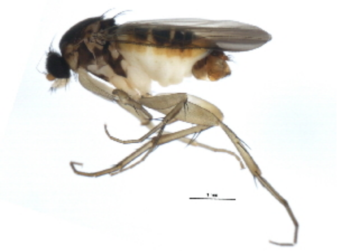

10JSROW-0499 [Lateral]
Phoridae
Family: Phoridae
BIN URI: BOLD:AAP2487

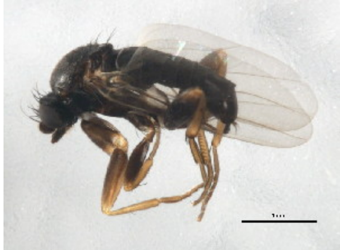

10JSROW-0141 [Lateral]
Phoridae
Family: Phoridae
BIN URI: BOLD:AAP4697

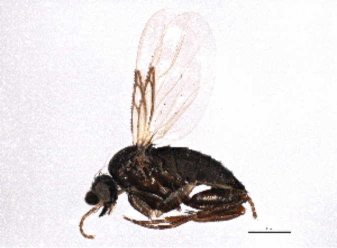

BIOUG05643-D09 [Lateral]
Phoridae
Family: Phoridae
BIN URI: BOLD:AAP6409

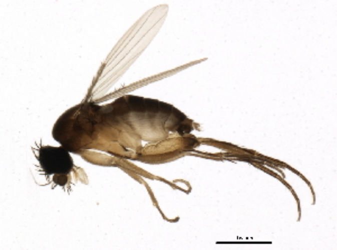

BIOUG01122-E06 [Lateral]
Phoridae
Family: Phoridae
BIN URI: BOLD:AAM7996

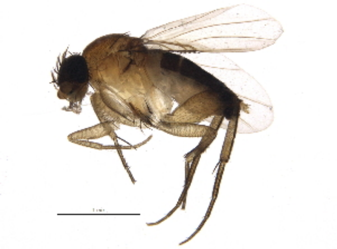

BIOUG11609-F03 [Lateral]
Phoridae
Family: Phoridae
BIN URI: BOLD:AAU8534

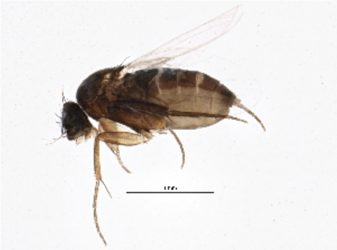

BIOUG02828-D12 [Lateral]
Phoridae
Family: Phoridae
BIN URI: BOLD:ABX8608

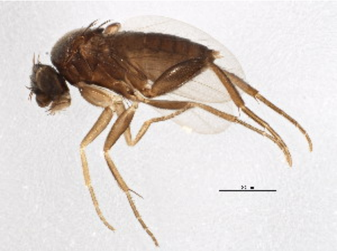

BIOUG01511-G03 [Lateral]
Phoridae
Family: Phoridae
BIN URI: BOLD:ABW8053

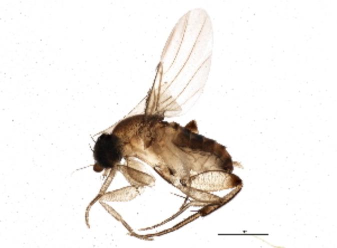

BIOUG03027-E10 [Lateral]
Phoridae
Family: Phoridae
BIN URI: BOLD:ACA6021

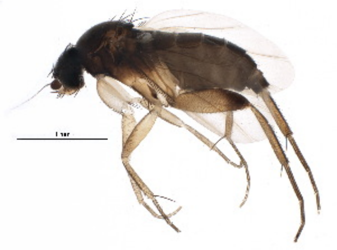

BIOUG22295-E03 [Lateral]
Megaselia
Family: Phoridae
BIN URI: BOLD:AAN8693

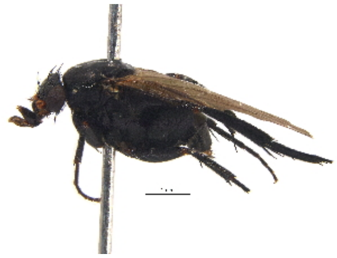

BIOUG13609-H11 [Lateral]
Hypocera
Family: Phoridae
BIN URI: BOLD:ACO8914

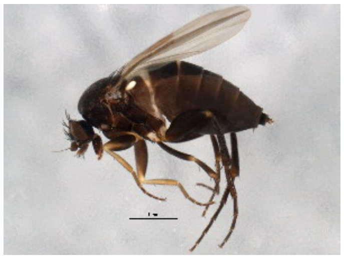

BIOUG01408-B08 [Lateral]
Phoridae
Family: Phoridae
BIN URI: BOLD:AAG3236

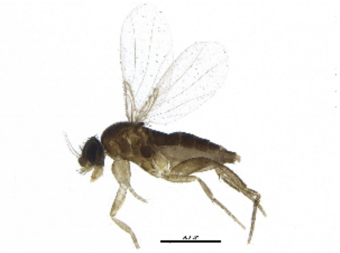

BIOUG08193-E02 [Lateral]
Megaselia
Family: Phoridae
BIN URI: BOLD:ACJ5224

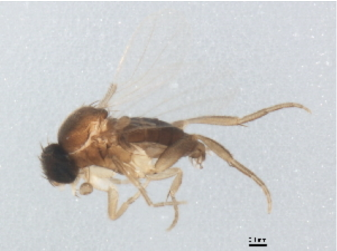

BIOUG01132-F02 [Lateral]
Phoridae
Family: Phoridae
BIN URI: BOLD:AAZ0308

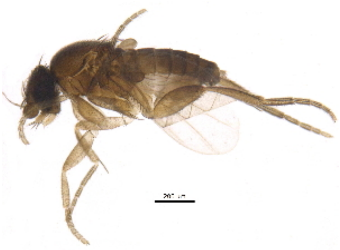

BIOUG23078-D08 [Lateral]
Phoridae
Family: Phoridae
BIN URI: BOLD:ACV5239

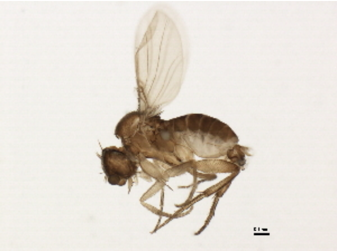

BIOUG01126-F06 [Lateral]
Phoridae
Family: Phoridae
BIN URI: BOLD:AAU6600

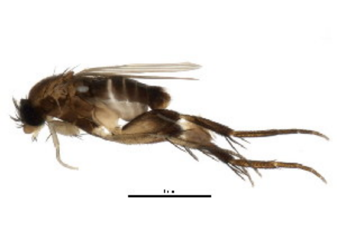

BIOUG00978-E02 [Lateral]
Phoridae
Family: Phoridae
BIN URI: BOLD:AAN8699

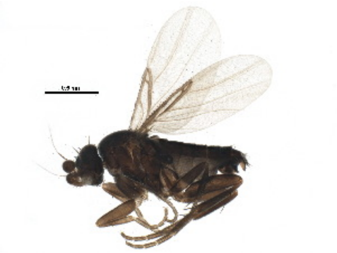

BIOUG22293-G12 [Lateral]
Phoridae
Family: Phoridae
BIN URI: BOLD:ACV3950

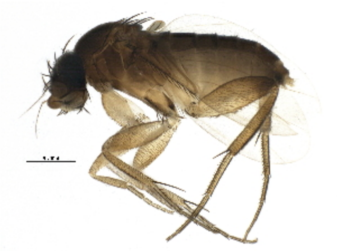

BIOUG08937-F07 [Lateral]
Megaselia
Family: Phoridae
BIN URI: BOLD:ACL5005

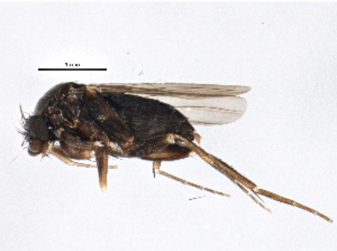

BIOUG02590-B01 [Lateral]
Phoridae
Family: Phoridae
BIN URI: BOLD:ABA1214

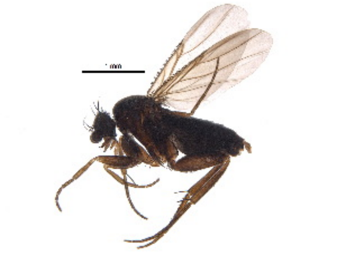

BIOUG22357-A01 [Lateral]
Phoridae
Family: Phoridae
BIN URI: BOLD:ABA1225

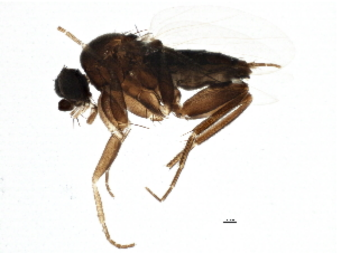

BIOUG05913-H11 [Lateral]
Phoridae
Family: Phoridae
BIN URI: BOLD:AAG3316

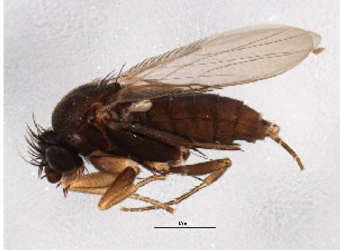

BIOUG01923-G05 [Lateral]
Phoridae
Family: Phoridae
BIN URI: BOLD:AAG3235

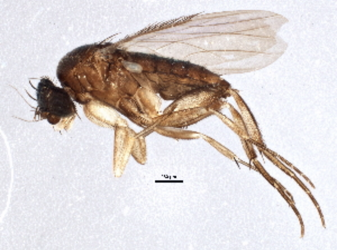

BIOUG06382-B07 [Lateral]
Phoridae
Family: Phoridae
BIN URI: BOLD:AAG3237

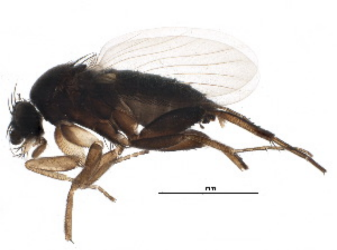

BIOUG22416-B09 [Lateral]
Megaselia
Family: Phoridae
BIN URI: BOLD:AAP8724

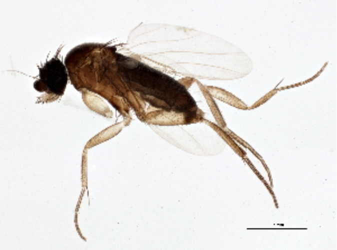

BIOUG02873-C12 [Lateral]
Phoridae
Family: Phoridae
BIN URI: BOLD:ABY1910

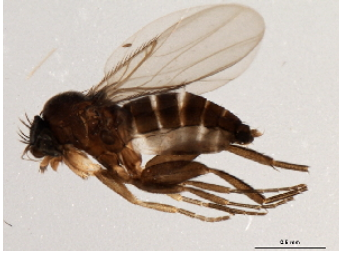

08TTML-1876 [Lateral]
Phoridae
Family: Phoridae
BIN URI: BOLD:AAN8700

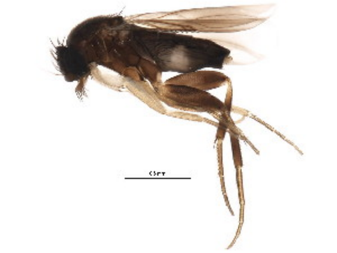

BIOUG01389-E03 [Lateral]
Phoridae
Family: Phoridae
BIN URI: BOLD:ABU5533

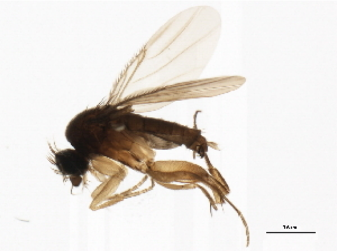

BIOUG01122-B08 [Lateral]
Phoridae
Family: Phoridae
BIN URI: BOLD:AAM9346

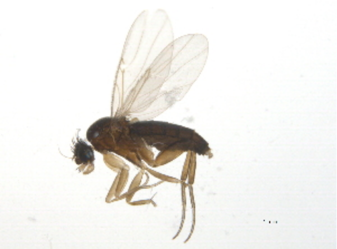

BIOUG03695-H01 [Lateral]
Phoridae
Family: Phoridae
BIN URI: BOLD:ACB9689

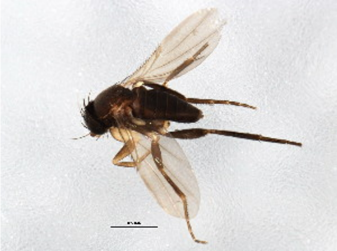

BIOUG01953-A05 [Lateral]
Phoridae
Family: Phoridae
BIN URI: BOLD:AAL9079

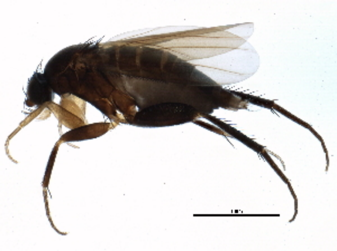

BIOUG11828-C05 [Lateral]
Phoridae
Family: Phoridae
BIN URI: BOLD:ACM9794

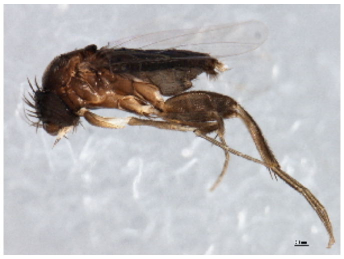

08TTML-1181 [Lateral]
Phoridae
Family: Phoridae
BIN URI: BOLD:AAN8692

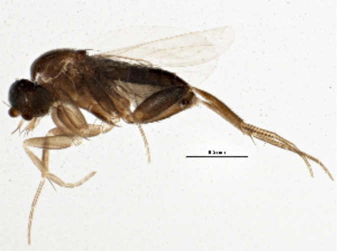

BIOUG02850-G12 [Lateral]
Phoridae
Family: Phoridae
BIN URI: BOLD:ABY9755

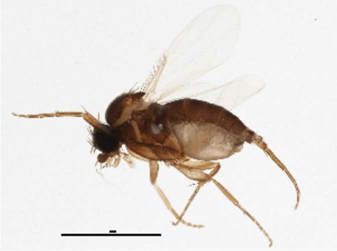

BIOUG01618-F05 [Lateral]
Phoridae
Family: Phoridae
BIN URI: BOLD:ACW3172

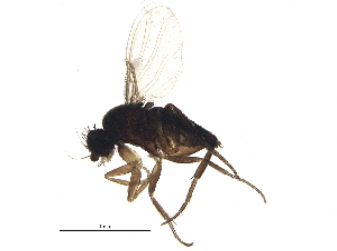

BIOUG09634-F08 [Lateral]
Phoridae
Family: Phoridae
BIN URI: BOLD:ACL7279

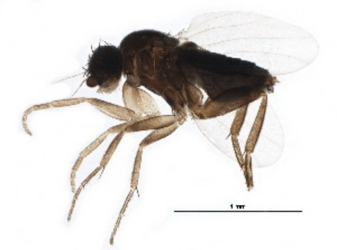

BIOUG22416-D09 [Lateral]
Phoridae
Family: Phoridae
BIN URI: BOLD:ACV3899

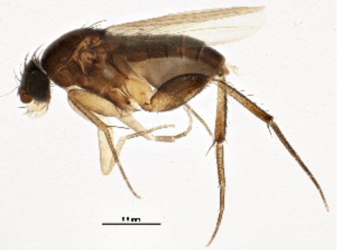

BIOUG05535-E08 [Lateral]
Phoridae
Family: Phoridae
BIN URI: BOLD:AAG3323

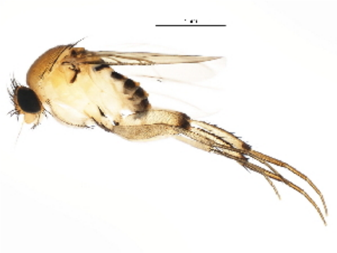

10JSROW-1069 [Lateral]
Phoridae
Family: Phoridae
BIN URI: BOLD:AAG3343

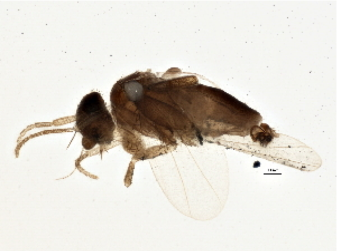

BIOUG02670-G11 [Lateral]
Phoridae
Family: Phoridae
BIN URI: BOLD:AAU6510

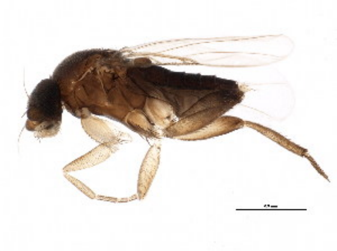

BIOUG01512-G04 [Lateral]
Phoridae
Family: Phoridae
BIN URI: BOLD:AAY6426

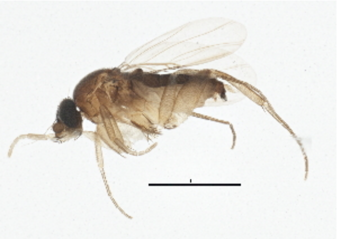

BIOUG01505-H01 [Lateral]
Phoridae
Family: Phoridae
BIN URI: BOLD:ABW7895

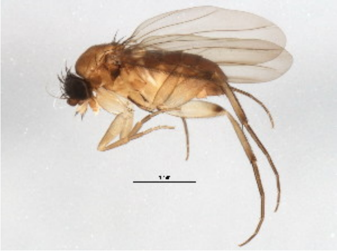

BIOUG01914-D12 [Lateral]
Phoridae
Family: Phoridae
BIN URI: BOLD:AAG3304

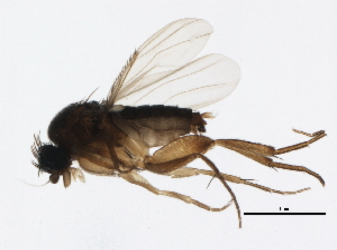

BIOUG01123-D12 [Lateral]
Phoridae
Family: Phoridae
BIN URI: BOLD:AAY6384

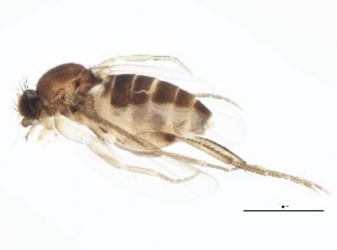

10BBDIP-0620 [Lateral]
Phoridae
Family: Phoridae
BIN URI: BOLD:AAN8711

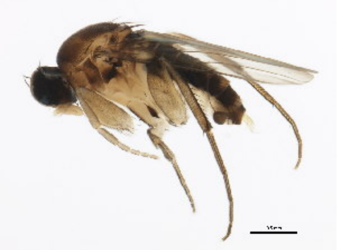

10PHMAL-2791 [Lateral]
Phoridae
Family: Phoridae
BIN URI: BOLD:AAU6538

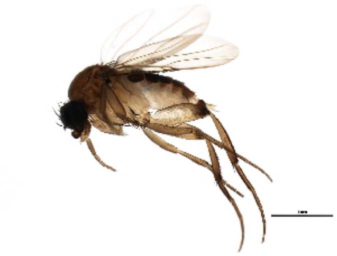

10PHMAL-1325 [Lateral]
Phoridae
Family: Phoridae
BIN URI: BOLD:AAM9378

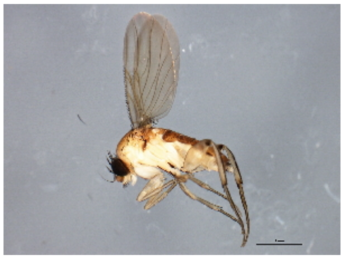

08TTML-1129 [Lateral]
Phoridae
Family: Phoridae
BIN URI: BOLD:AAM9375

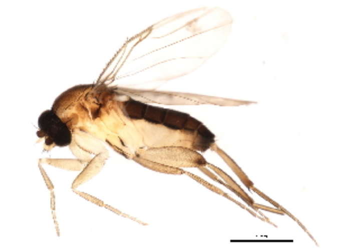

09BBEDI-2707 [Lateral]
Phoridae
Family: Phoridae
BIN URI: BOLD:AAG3261

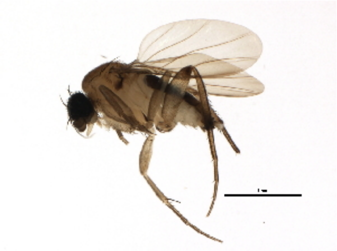

BIOUG01122-B07 [Lateral]
Phoridae
Family: Phoridae
BIN URI: BOLD:AAN8705

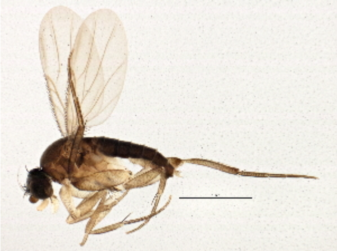

BIOUG03277-E08 [Lateral]
Phoridae
Family: Phoridae
BIN URI: BOLD:ACA8351

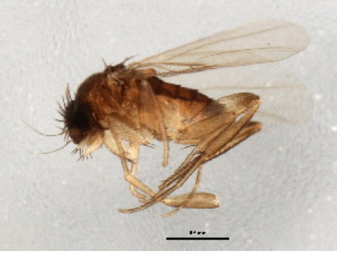

BIOUG01929-C05 [Lateral]
Phoridae
Family: Phoridae
BIN URI: BOLD:AAU5599

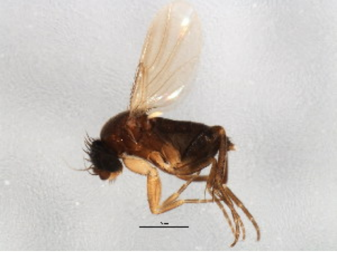

BIOUG01920-B11 [Lateral]
Phoridae
Family: Phoridae
BIN URI: BOLD:AAL9075

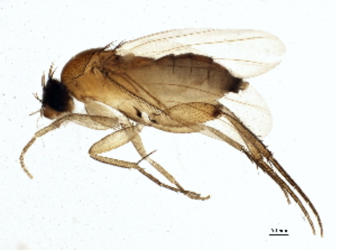

BIOUG02965-E03 [Lateral]
Phoridae
Family: Phoridae
BIN URI: BOLD:ABU5535

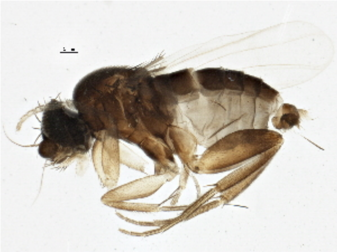

BIOUG03343-F12 [Lateral]
Phoridae
Family: Phoridae
BIN URI: BOLD:ACA5842

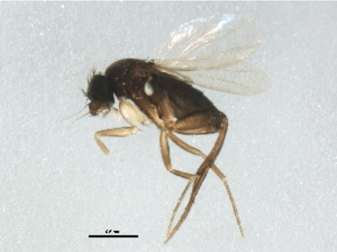

BIOUG01479-E05 [Lateral]
Phoridae
Family: Phoridae
BIN URI: BOLD:AAU6533

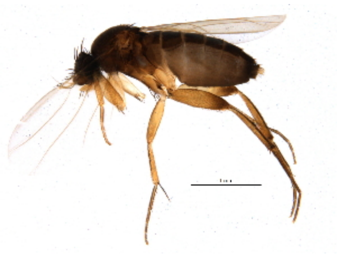

BIOUG04791-F12 [Lateral]
Phoridae
Family: Phoridae
BIN URI: BOLD:ACE1572

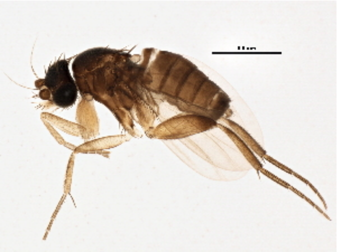

BIOUG05742-C08 [Lateral]
Phoridae
Family: Phoridae
BIN URI: BOLD:AAO8579

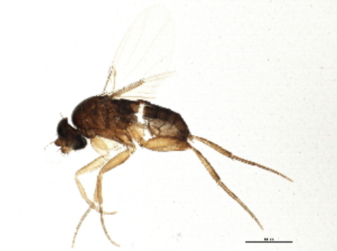

BIOUG05870-B09 [Lateral]
Phoridae
Family: Phoridae
BIN URI: BOLD:ACG7292

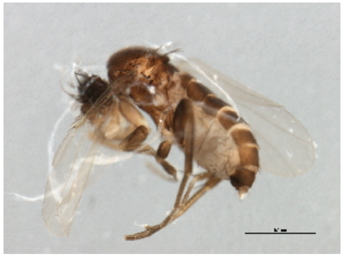

09BBEDI-0970 [Lateral]
Megaselia
Family: Phoridae
BIN URI: BOLD:AAG3318

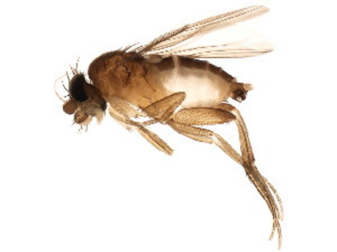

BIOUG01426-F09 [Lateral]
Phoridae
Family: Phoridae
BIN URI: BOLD:ABU5538

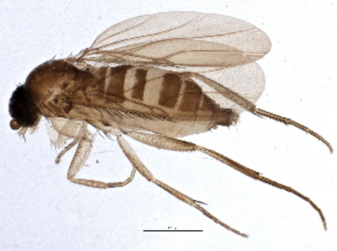

BIOUG05986-H09 [Lateral]
Phoridae
Family: Phoridae
BIN URI: BOLD:AAM9365

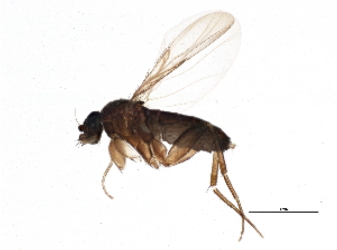

BIOUG02647-H09 [Lateral]
Megaselia
Family: Phoridae
BIN URI: BOLD:AAG3266

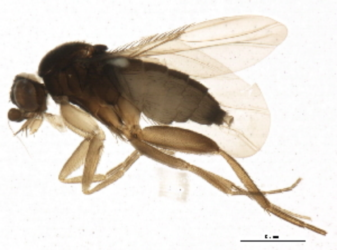

BIOUG01302-C02 [Lateral]
Phoridae
Family: Phoridae
BIN URI: BOLD:AAG3340

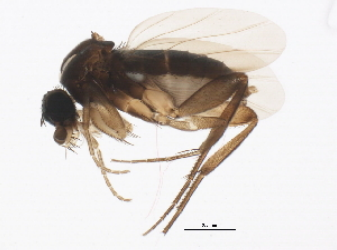

BIOUG01445-G07 [Lateral]
Phoridae
Family: Phoridae
BIN URI: BOLD:AAU6524

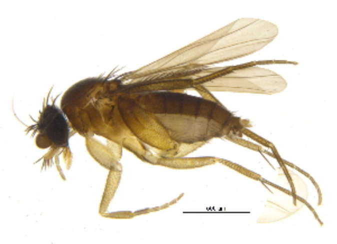

BIOUG03249-H08 [Lateral]
Megaselia
Family: Phoridae
BIN URI: BOLD:ABU5528

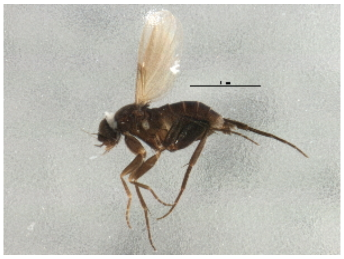

BIOUG03736-A05 [Lateral]
Phoridae
Family: Phoridae
BIN URI: BOLD:AAG3302

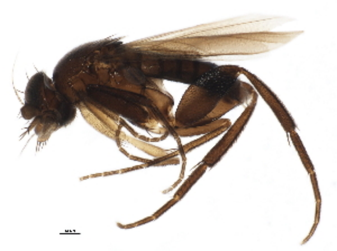

BIOUG03824-H03 [Lateral]
Megaselia
Family: Phoridae
BIN URI: BOLD:AAG3275

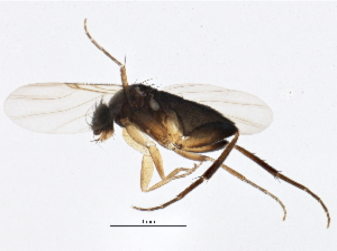

BIOUG03736-G09 [Lateral]
Megaselia
Family: Phoridae
BIN URI: BOLD:AAP6410

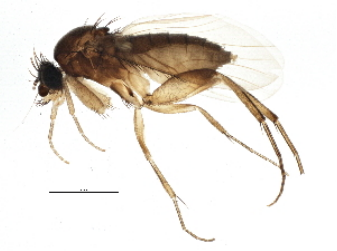

BIOUG04290-A04 [Lateral]
Megaselia
Family: Phoridae
BIN URI: BOLD:AAG3274

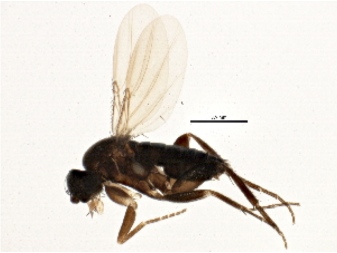

BIOUG05521-A04 [Lateral]
Phoridae
Family: Phoridae
BIN URI: BOLD:ABU5529

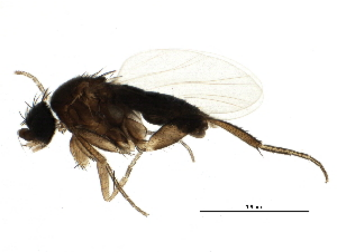

BIOUG22366-D02 [Lateral]
Phoridae
Family: Phoridae
BIN URI: BOLD:ACD8848

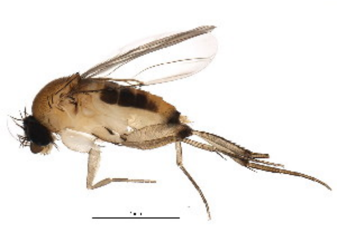

BIOUG01352-C08 [Lateral]
Phoridae
Family: Phoridae
BIN URI: BOLD:AAG3259

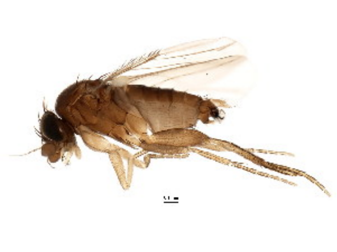

BIOUG01915-G08 [Lateral]
Phoridae
Family: Phoridae
BIN URI: BOLD:AAG3260

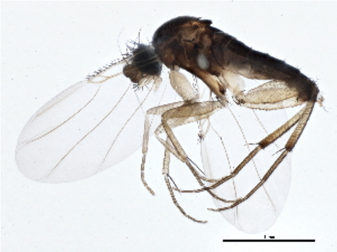

BIOUG05544-F03 [Lateral]
Phoridae
Family: Phoridae
BIN URI: BOLD:AAL9076

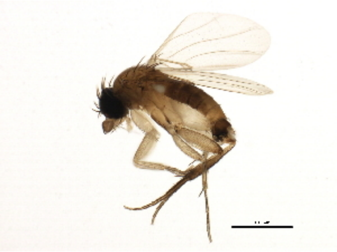

BIOUG01302-G01 [Lateral]
Phoridae
Family: Phoridae
BIN URI: BOLD:AAG3248

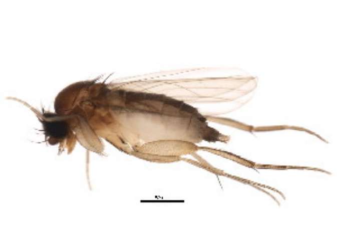

BIOUG01400-F05 [Lateral]
Phoridae
Family: Phoridae
BIN URI: BOLD:AAM9347

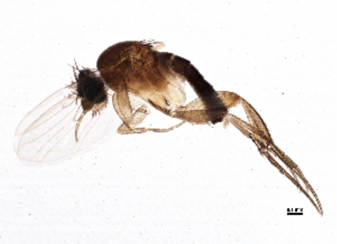

BIOUG02979-G05 [Lateral]
Phoridae
Family: Phoridae
BIN URI: BOLD:AAN8679

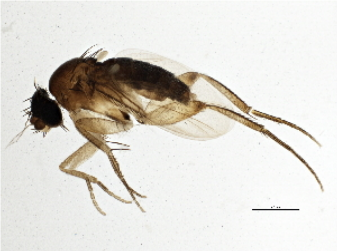

BIOUG03908-E03 [Lateral]
Phoridae
Family: Phoridae
BIN URI: BOLD:AAZ6701

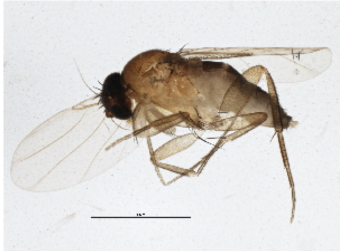

BIOUG05720-B11 [Lateral]
Phoridae
Family: Phoridae
BIN URI: BOLD:AAP4687

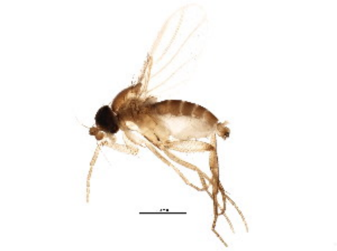

BIOUG01664-E08 [Lateral]
Phoridae
Family: Phoridae
BIN URI: BOLD:AAU5682

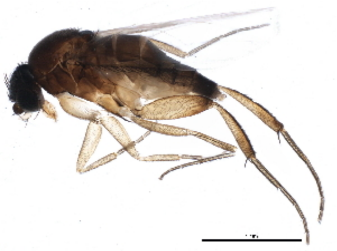

BIOUG05545-H07 [Lateral]
Phoridae
Family: Phoridae
BIN URI: BOLD:AAU6624

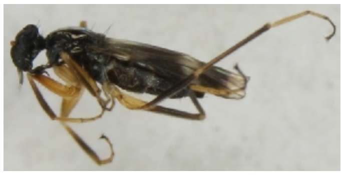

jka11-00966 [Lateral]
Tachydromia aemula
Family: Hybotidae
BIN URI: BOLD:AAN5500

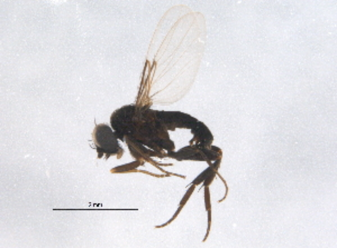

BIOUG03479-E05 [Lateral]
Phoridae
Family: Phoridae
BIN URI: BOLD:ACB0980

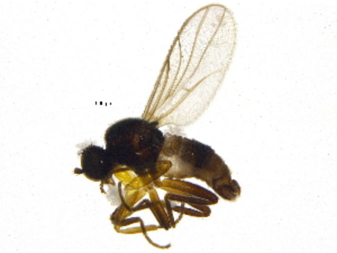

BIOUG03345-A07 [Lateral]
Platypalpus
Family: Hybotidae
BIN URI: BOLD:AAN5503

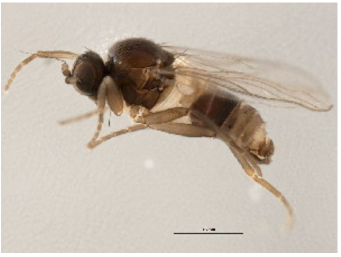

10BBCDDIP-3328 [Lateral]
Hybotidae
Family: Hybotidae
BIN URI: BOLD:AAV3698

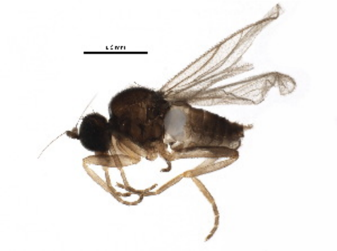

BIOUG23073-C04 [Lateral]
Hybotidae
Family: Hybotidae
BIN URI: BOLD:ACV5183

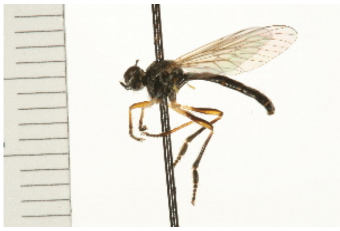

DIPT 0006.02 [Lateral]
Asilidae
Family: Asilidae
BIN URI: BOLD:AAH2293

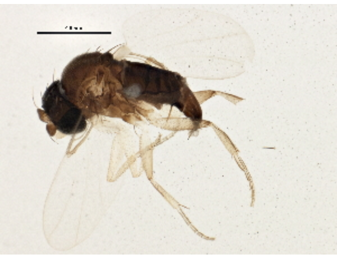

BIOUG03910-B02 [Lateral]
Phoridae
Family: Phoridae
BIN URI: BOLD:ABA8289

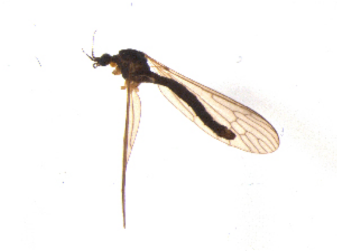

10PROBE-10299 [Lateral]
Pseudolimnophila inornata
Family: Limoniidae
BIN URI: BOLD:AAI1351

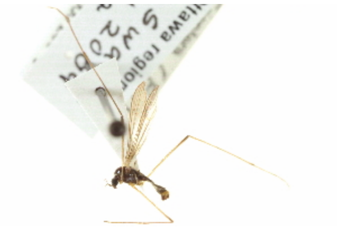

CNC DIPTERA 192405 [Lateral]
Erioptera ebenina
Family: Limoniidae
BIN URI: BOLD:ACB0353

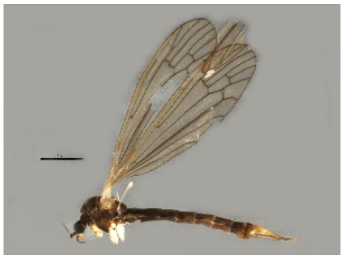

BIOUG09060-A01 [Lateral]
Limoniidae
Family: Limoniidae
BIN URI: BOLD:ACL7587

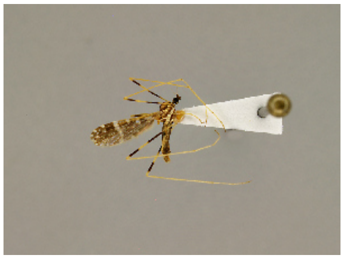

CMNH576021 [Lateral]
Erioptera caliptera
Family: Limoniidae
BIN URI: BOLD:AAN5882

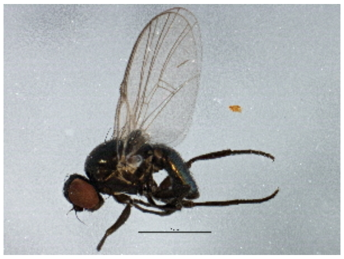

BIOUG03057-D08 [Lateral]
Agromyzidae
Family: Agromyzidae
BIN URI: BOLD:AAQ0692

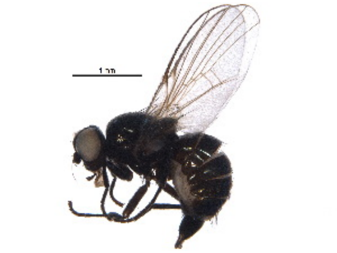

BIOUG22454-D11 [Lateral]
Agromyzidae
Family: Agromyzidae
BIN URI: BOLD:ACK1565

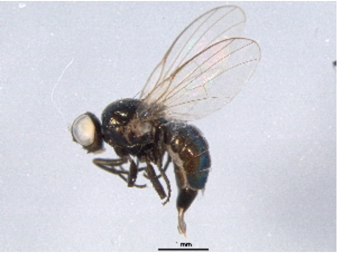

BIOUG05666-C03 [Lateral]
Agromyzidae
Family: Agromyzidae
BIN URI: BOLD:ACK1603

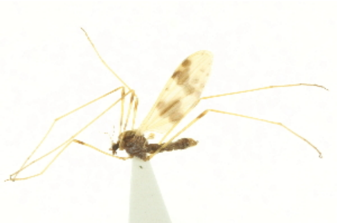

CNC DIPTERA 192426 [Lateral]
Ilisia venusta
Family: Limoniidae
BIN URI: BOLD:ACA9724

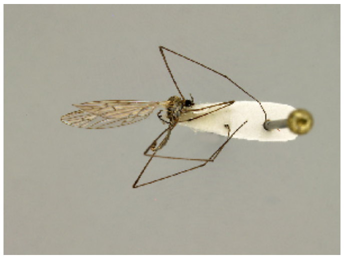

CMNH576084 [Lateral]
Symplecta
Family: Limoniidae
BIN URI: BOLD:AAF9014

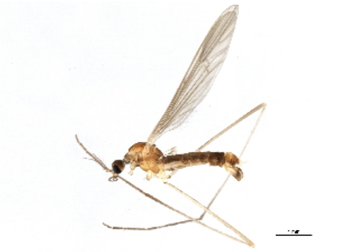

BIOUG01392-A08 [Lateral]
Ormosia affinis
Family: Limoniidae
BIN URI: BOLD:AAU6544

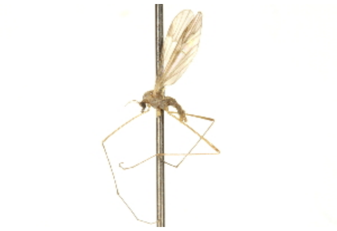

CNC DIPTERA 192458 [Lateral]
Ormosia meigenii
Family: Limoniidae
BIN URI: BOLD:ACA9818

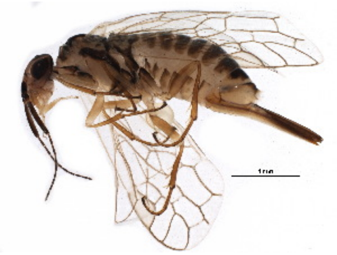

BIOUG22420-G09 [Lateral]
Xyelidae
Family: Xyelidae
BIN URI: BOLD:ACS9433

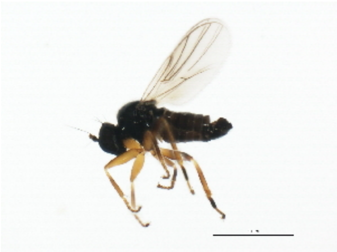

BIOUG01403-H07 [Lateral]
Hybotidae
Family: Hybotidae
BIN URI: BOLD:AAN5501

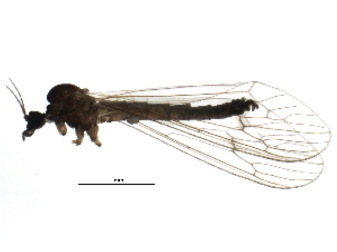

BIOUG21589-C09 [Lateral]
Trichocera
Family: Trichoceridae
BIN URI: BOLD:ACF7745

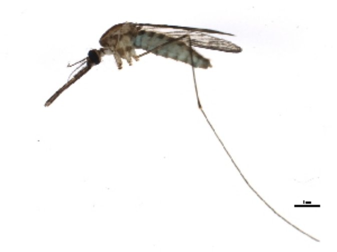

BIOUG00904-D11 [Lateral]
Anopheles quadrimaculatus
Family: Culicidae
BIN URI: BOLD:AAC2281

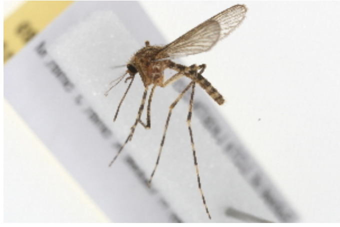

09BBEDI-1089 [Lateral]
Coquillettidia perturbans
Family: Culicidae
BIN URI: BOLD:AAB2539

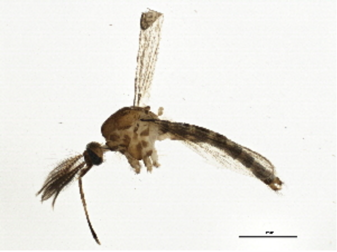

BIOUG03219-B11 [Lateral]
Coquillettidia perturbans
Family: Culicidae
BIN URI: BOLD:AAI1618

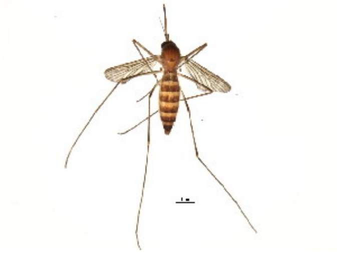

BIOUG00938-E09 [Dorsal]
Culex restuans
Family: Culicidae
BIN URI: BOLD:AAA7661

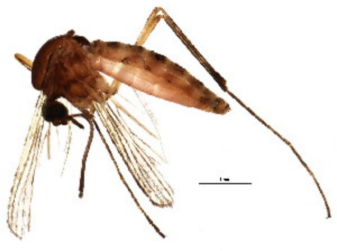

BIOUG01349-H05 [Lateral]
Culicidae
Family: Culicidae
BIN URI: BOLD:AAB6943

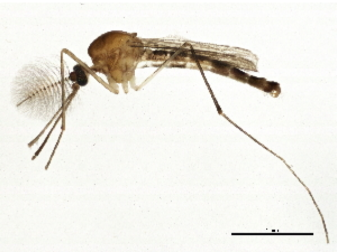

BIOUG03219-F12 [Lateral]
Culex territans
Family: Culicidae
BIN URI: BOLD:ABY7666

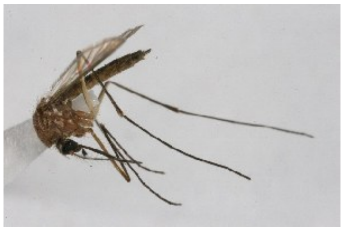

JE010 [Lateral]
Aedes cinereus
Family: Culicidae
BIN URI: BOLD:AAC1222

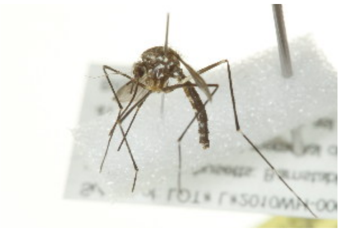

TDWG-0239 [Lateral]
Aedes japonicus
Family: Culicidae
BIN URI: BOLD:AAC5210

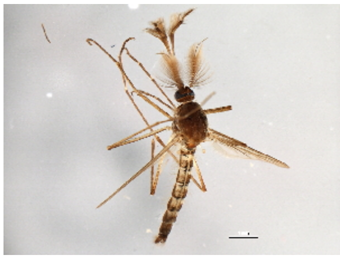

08TTML-2120 [Dorsal]
Culicidae
Family: Culicidae
BIN URI: BOLD:AAA7067

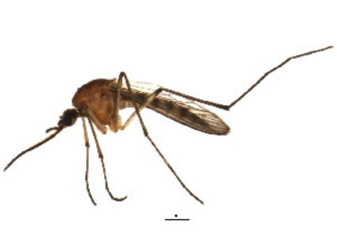

BIOUG01344-D02 [Lateral]
Culicidae
Family: Culicidae
BIN URI: BOLD:AAD4355

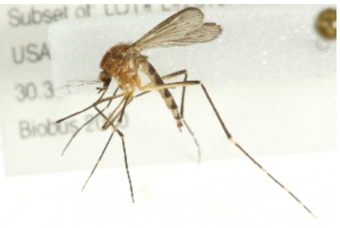

10BBDIP-0254 [Lateral]
Culicidae
Family: Culicidae
BIN URI: BOLD:AAB5696

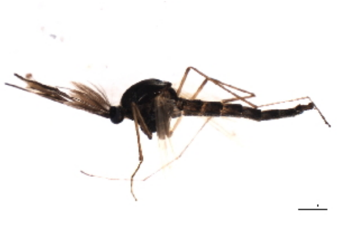

09PROBE-JW0432 [Lateral]
Aedes punctor subgroup
Family: Culicidae
BIN URI: BOLD:AAA3748

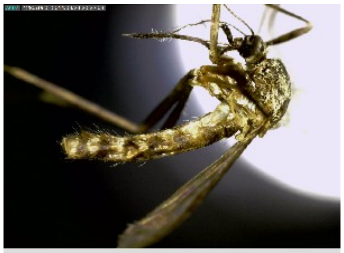

AIOM-055 [Specimen]
Aedes triseriatus
Family: Culicidae
BIN URI: BOLD:AAC9476

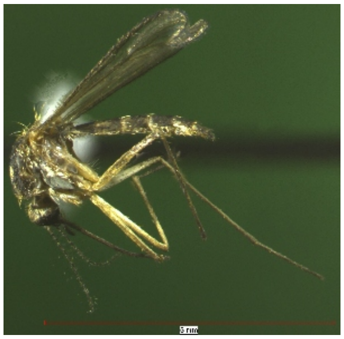

NEONTculicid853 [Lateral]
Aedes trivittatus
Family: Culicidae
BIN URI: BOLD:AAC9486

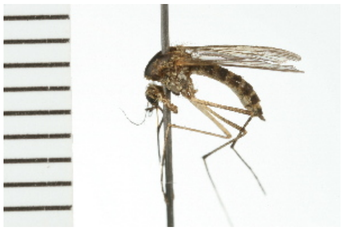

08BBDIP-0938 [Lateral]
Ochlerotatus fitchii
Family: Culicidae
BIN URI: BOLD:AAC9062

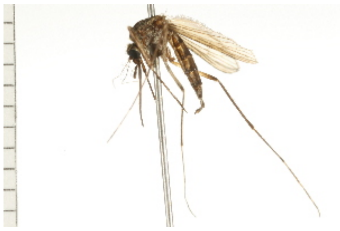

08BBDIP-1669 [Lateral]
Aedes fitchii
Family: Culicidae
BIN URI: BOLD:AAD8027

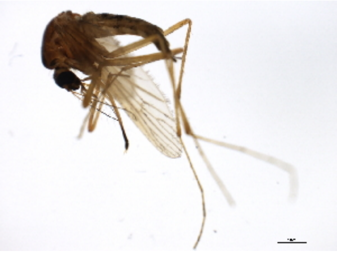

10BBCDIP-0952 [Lateral]
Aedes euedes
Family: Culicidae
BIN URI: BOLD:AAD4406

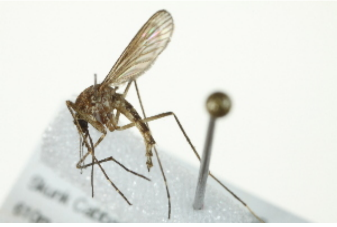

10BBCDIP-2303 [Lateral]
Ochlerotatus excrucians
Family: Culicidae
BIN URI: BOLD:AAB1098

IMAGE NOT AVAILABLE

BIOUG22843-G04
Aedes stimulans
Family: Culicidae

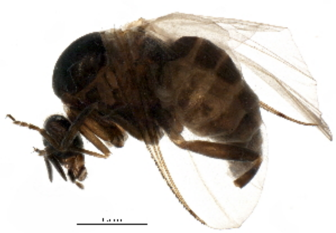

BIOUG22359-B10 [Lateral]
Stegopterna
Family: Simuliidae
BIN URI: BOLD:AAB7514

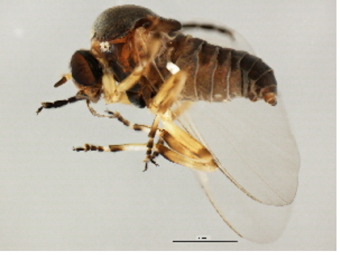

10BBCDIP-3050 [Lateral]
Simulium decorum
Family: Simuliidae
BIN URI: BOLD:AAB7749

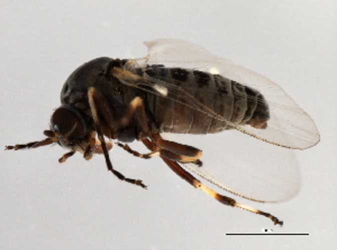

08TTML-2051 [Lateral]
Simuliidae
Family: Simuliidae
BIN URI: BOLD:AAA4121

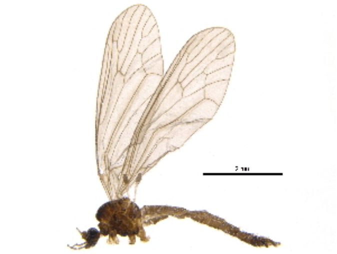

BIOUG21482-B07 [Lateral]
Trichocera
Family: Trichoceridae
BIN URI: BOLD:ABW7619

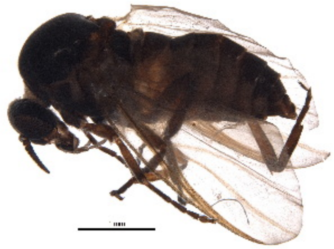

BIOUG22356-H05 [Lateral]
Prosimulium arvum
Family: Simuliidae
BIN URI: BOLD:AAD4764

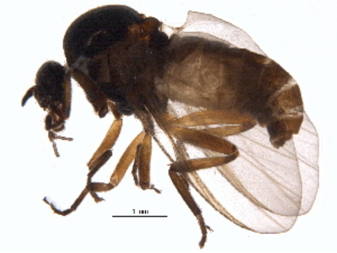

BIOUG11175-B12 [Lateral]
Prosimulium mixtum
Family: Simuliidae
BIN URI: BOLD:AAG7032

IMAGE NOT AVAILABLE

BIOUG22237-C07
Prosimulium mixtum
Family: Simuliidae

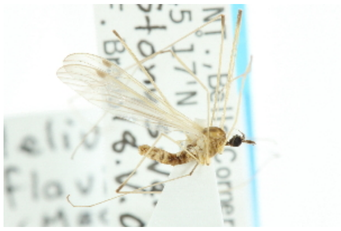

CNC DIPTERA 106614 [Lateral]
Helius flavipes
Family: Limoniidae
BIN URI: BOLD:AAF9008

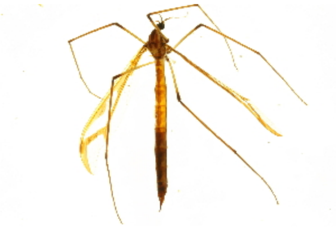

08TTML-2418 [Dorsal]
Pedicia
Family: Pediciidae
BIN URI: BOLD:AAF9045

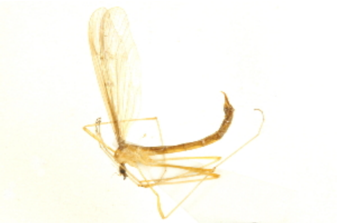

CNC DIPTERA 192485 [Lateral]
Tricyphona inconstans
Family: Pediciidae
BIN URI: BOLD:ACV1010

IMAGE NOT AVAILABLE

BIOUG24024-C03
Tricyphona
Family: Pediciidae

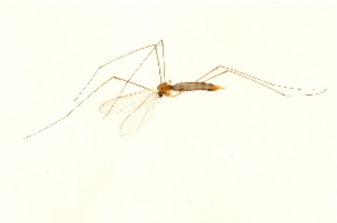

09ELEPT-045 [Lateral]
Limoniidae
Family: Limoniidae
BIN URI: BOLD:AAF9040

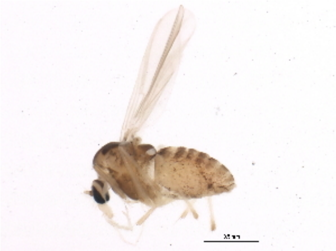

BIOUG01403-C01 [Lateral]
Tanypodinae
Family: Chironomidae
BIN URI: BOLD:ABV0256

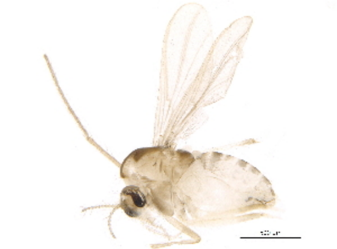

BIOUG22719-H02 [Lateral]
Chironomidae
Family: Chironomidae
BIN URI: BOLD:AAO7630

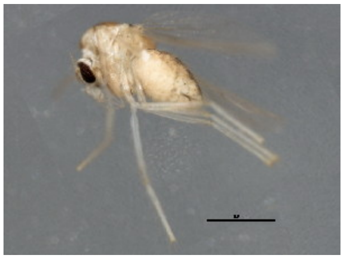

10BBCDIP-1014 [Lateral]
Chironomidae
Family: Chironomidae
BIN URI: BOLD:ACM1999

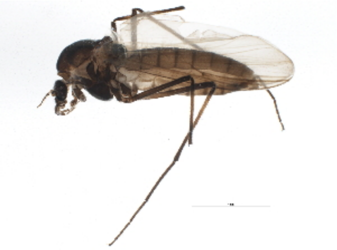

BIOUG05519-H04 [Lateral]
Pagastia orthogonia
Family: Chironomidae
BIN URI: BOLD:AAI2601

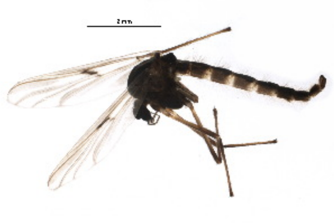

BIOUG22289-F05 [Lateral]
Chironomidae
Family: Chironomidae
BIN URI: BOLD:ACV5706

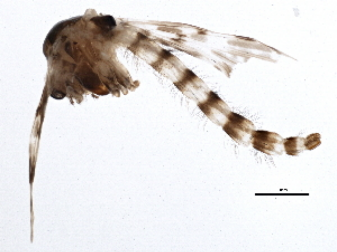

BIOUG05644-A07 [Lateral]
Psectrotanypus sp. ES01
Family: Chironomidae
BIN URI: BOLD:AAG0314

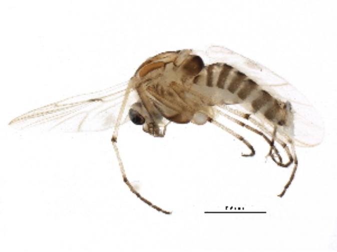

BIOUG22084-H03 [Lateral]
Procladius
Family: Chironomidae
BIN URI: BOLD:AAL7370

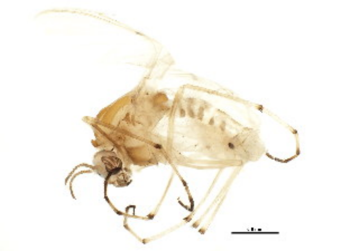

BIOUG01700-E06 [Lateral]
Chironomidae
Family: Chironomidae
BIN URI: BOLD:ABV9319

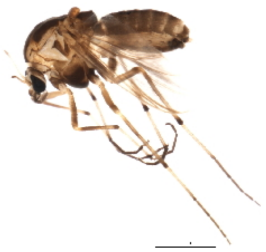

10BBDIP-1373 [Frontal]
Chironomidae
Family: Chironomidae
BIN URI: BOLD:AAG3918

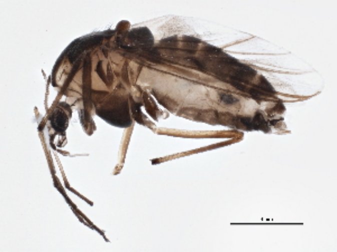

BIOUG05522-E05 [Lateral]
Procladius
Family: Chironomidae
BIN URI: BOLD:AAM6227

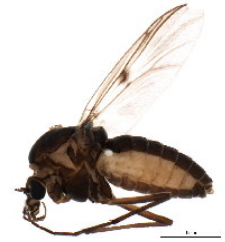

BIOUG01459-B05 [Lateral]
Chironomidae
Family: Chironomidae
BIN URI: BOLD:AAP3007

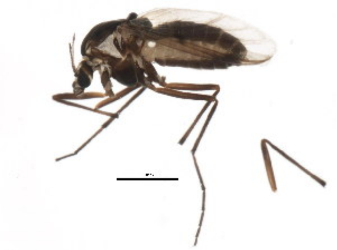

BIOUG01344-E09 [Lateral]
Procladius
Family: Chironomidae
BIN URI: BOLD:AAQ0606

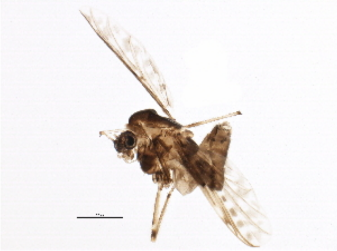

BIOUG20653-B02 [Lateral]
Diptera

BIN URI: BOLD:ACU4727

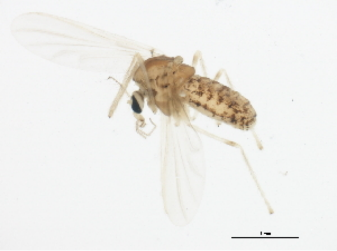

BIOUG01403-G08 [Lateral]
Chironomidae
Family: Chironomidae
BIN URI: BOLD:ACJ6513

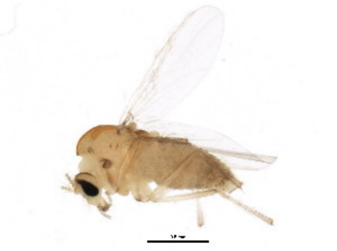

BIOUG01337-A06 [Lateral]
Monopelopia tenuicalcar
Family: Chironomidae
BIN URI: BOLD:AAM6277

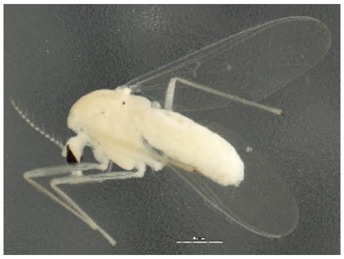

10JSROW-0880 [Lateral]
Krenopelopia
Family: Chironomidae
BIN URI: BOLD:AAC9199

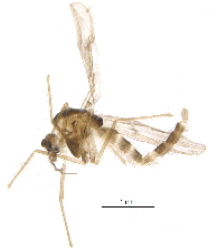

BIOUG21767-C06 [Lateral]
Chironomidae
Family: Chironomidae
BIN URI: BOLD:AAN5326

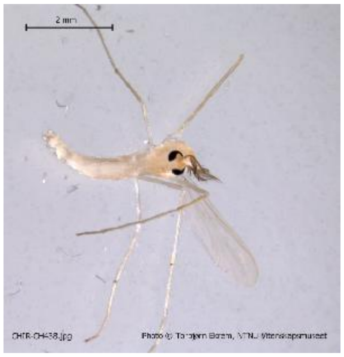

CHIR_CH438 [Ventral]
Conchapelopia telema
Family: Chironomidae
BIN URI: BOLD:AAC4802

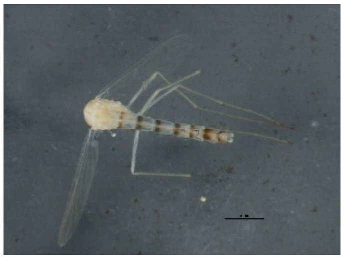

10JSROW-0447 [Dorsal]
Conchapelopia telema
Family: Chironomidae
BIN URI: BOLD:AAN5351

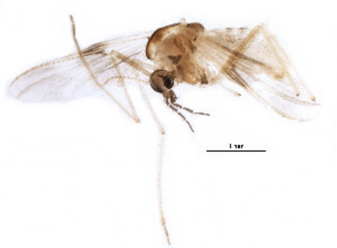

BIOUG21773-C02 [Lateral]
Chironomidae
Family: Chironomidae
BIN URI: BOLD:AAM6281

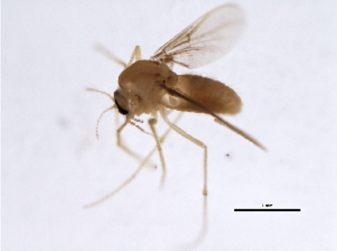

08TTML-1497 [Lateral]
Chironomidae
Family: Chironomidae
BIN URI: BOLD:AAN5335

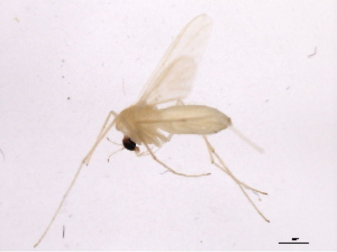

09BBDIP-1839 [Lateral]
Chironomidae
Family: Chironomidae
BIN URI: BOLD:AAG5468

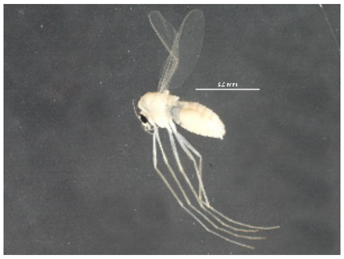

BIOUG22875-D09 [Lateral]
Chironomidae
Family: Chironomidae
BIN URI: BOLD:ACV4670

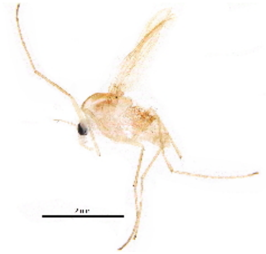

BIOUG22930-C09 [Lateral]
Tanypodinae
Family: Chironomidae
BIN URI: BOLD:AAP6883

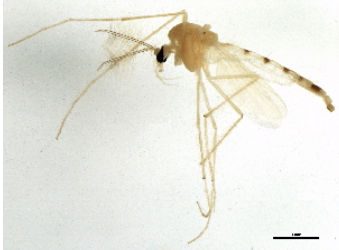

BIOUG01544-D11 [Lateral]
Tanypodinae
Family: Chironomidae
BIN URI: BOLD:ABW4240

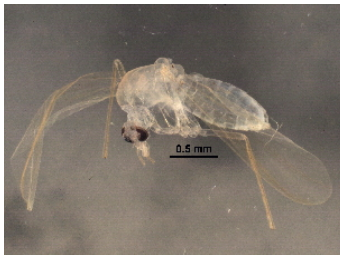

BIOUG16069-G08 [Lateral]
Tanypodinae
Family: Chironomidae
BIN URI: BOLD:ACP8795

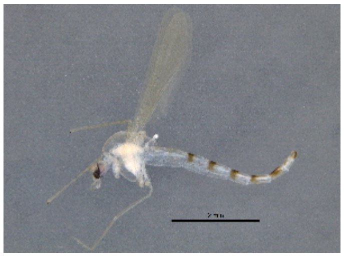

BIOUG22468-C11 [Lateral]
Chironomidae
Family: Chironomidae
BIN URI: BOLD:ACV5536

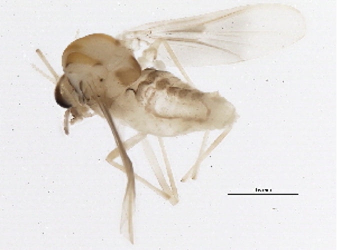

10BBDIP-0579 [Lateral]
Chironomidae
Family: Chironomidae
BIN URI: BOLD:AAG5531

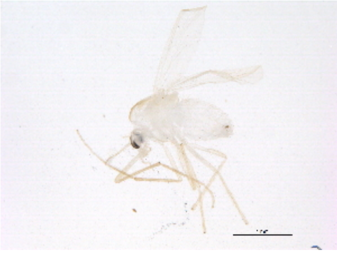

BIOUG03752-G10 [Lateral]
Chironomidae
Family: Chironomidae
BIN URI: BOLD:ACC7559

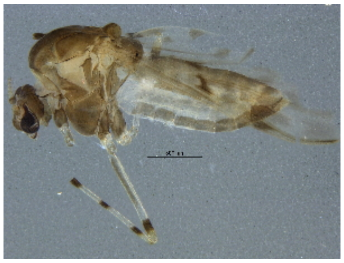

BIOUG08639-B03 [Lateral]
Ablabesmyia
Family: Chironomidae
BIN URI: BOLD:ACH2330

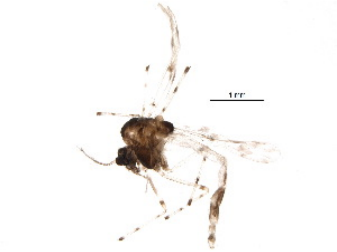

BIOUG21180-F10 [Lateral]
Diptera

BIN URI: BOLD:ACW0823

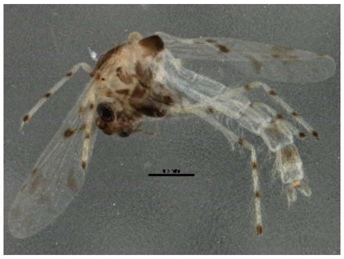

BIOUG03910-A04 [Lateral]
Chironomidae
Family: Chironomidae
BIN URI: BOLD:AAN7576

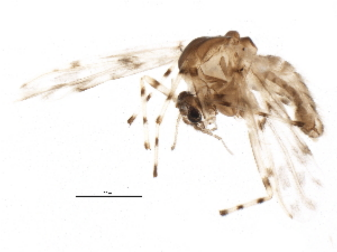

BIOUG20653-H08 [Lateral]
Diptera

BIN URI: BOLD:AAC8567

IMAGE NOT AVAILABLE

BIOUG21773-C11
Ablabesmyia
Family: Chironomidae

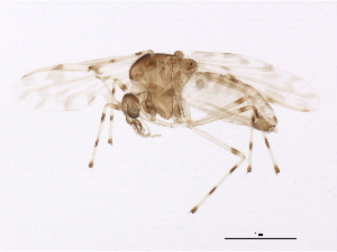

BIOUG01675-H04 [Lateral]
Chironomidae
Family: Chironomidae
BIN URI: BOLD:AAP3003

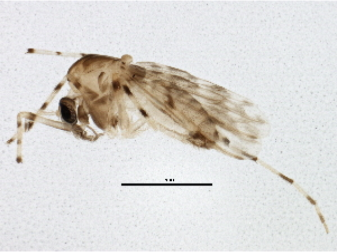

BIOUG03907-D07 [Lateral]
Chironomidae
Family: Chironomidae
BIN URI: BOLD:ACC7822

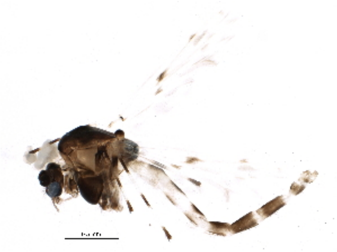

BIOUG22084-B12 [Lateral]
Chironomidae
Family: Chironomidae
BIN URI: BOLD:AAP5113

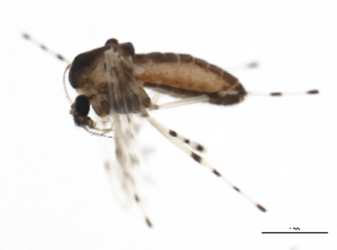

10BBCDIP-1078 [Lateral]
Tanypodinae
Family: Chironomidae
BIN URI: BOLD:AAP8999

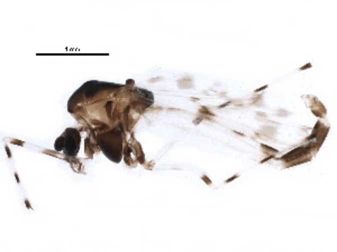

BIOUG22467-A01 [Lateral]
Ablabesmyia
Family: Chironomidae
BIN URI: BOLD:ABV1232

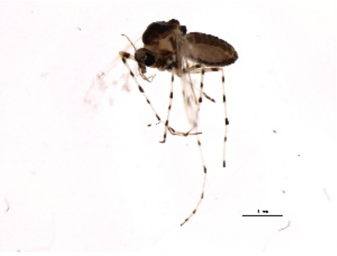

09BBDIP-1868 [Lateral]
Chironomidae
Family: Chironomidae
BIN URI: BOLD:AAM6234

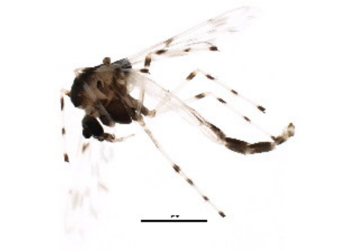

BIOUG01352-C09 [Lateral]
Chironomidae
Family: Chironomidae
BIN URI: BOLD:ABW7322

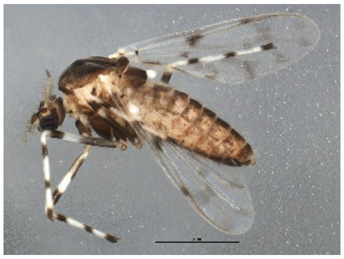

10JSROW-0876 [Lateral]
Chironomidae
Family: Chironomidae
BIN URI: BOLD:AAM6293

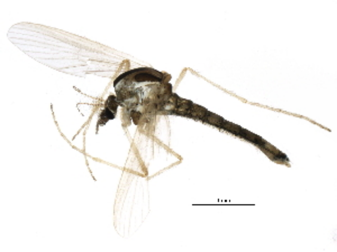

BIOUG21589-B12 [Lateral]
Chaoboridae
Family: Chaoboridae
BIN URI: BOLD:AAG5471

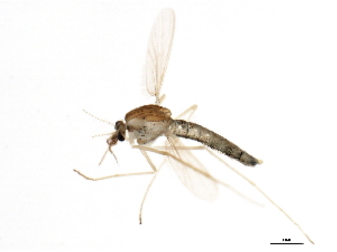

08TTML-1111 [Lateral]
Chaoboridae
Family: Chaoboridae
BIN URI: BOLD:AAM6295

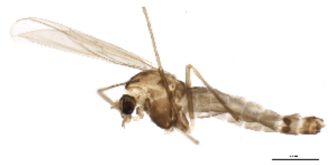

BIOUG01490-C08 [Lateral]
Chironomidae
Family: Chironomidae
BIN URI: BOLD:ABA0774

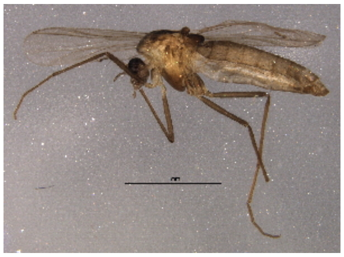

BIOUG10176-F06 [Lateral]
Chironomidae
Family: Chironomidae
BIN URI: BOLD:ACK4095

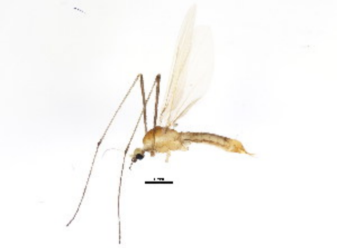

BIOUG00855-C07 [Lateral]
Limoniidae
Family: Limoniidae
BIN URI: BOLD:AAZ5967

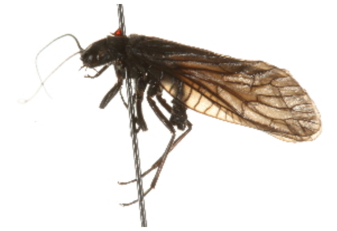

09CBCAD-139 [Lateral]
Sialis BIO6
Family: Sialidae
BIN URI: BOLD:AAG9766

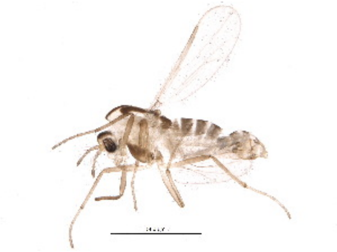

BIOUG20609-H07 [Lateral]
Diptera

BIN URI: BOLD:ACU1761

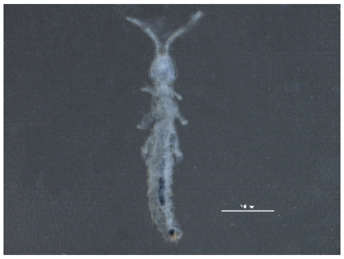

BIOUG14144-C11 [Dorsal]
Campodeidae
Family: Campodeidae
BIN URI: BOLD:ACP3162

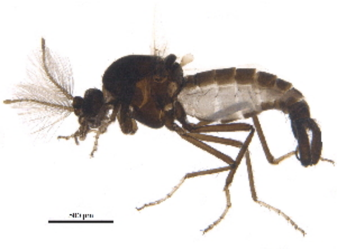

BIOUG22842-E07 [Lateral]
Ceratopogon
Family: Ceratopogonidae
BIN URI: BOLD:ACV9101

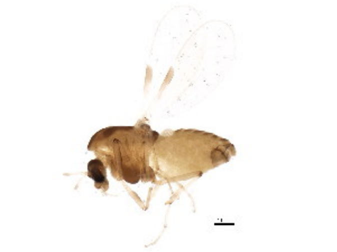

BIOUG01924-E10 [Lateral]
Chironomidae
Family: Chironomidae
BIN URI: BOLD:ABY2015

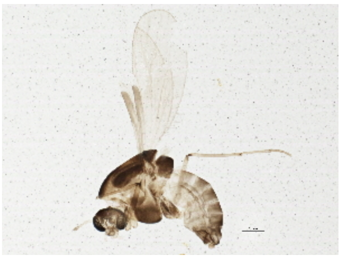

BIOUG05597-F04 [Lateral]
Corynoneura
Family: Chironomidae
BIN URI: BOLD:AAG0994

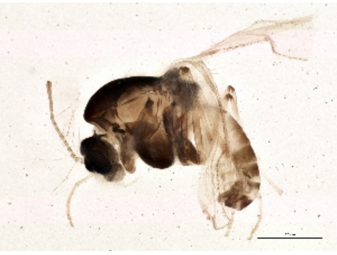

BIOUG05714-G07 [Lateral]
Corynoneura
Family: Chironomidae
BIN URI: BOLD:ACF9451

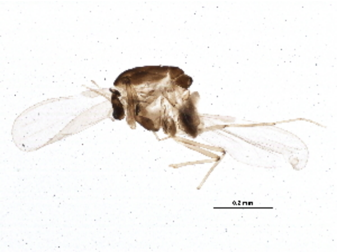

BIOUG09240-G02 [Lateral]
Orthocladiinae
Family: Chironomidae
BIN URI: BOLD:ACK8001

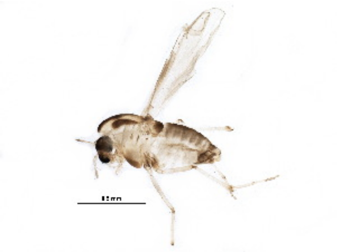

BIOUG22417-C05 [Lateral]
Corynoneura scutellata
Family: Chironomidae
BIN URI: BOLD:AAN5330

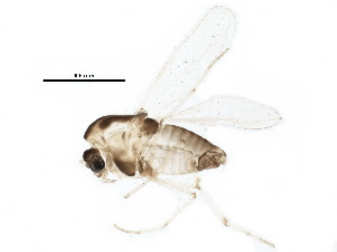

BIOUG22466-E02 [Lateral]
Corynoneura
Family: Chironomidae
BIN URI: BOLD:ABY3407

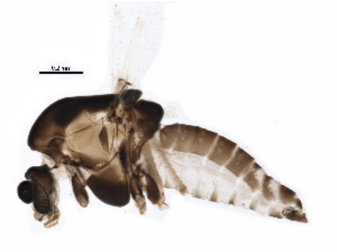

BIOUG21778-E01 [Lateral]
Chironomidae
Family: Chironomidae
BIN URI: BOLD:ACV2298

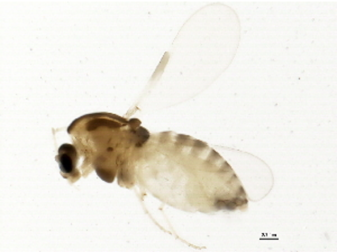

10BBDIP-2156 [Lateral]
Chironomidae
Family: Chironomidae
BIN URI: BOLD:AAO7623

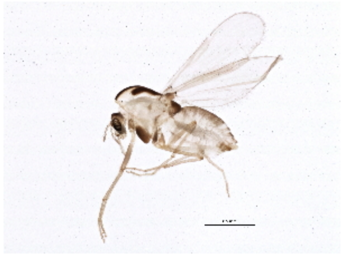

BIOUG05517-D05 [Lateral]
Thienemanniella xena
Family: Chironomidae
BIN URI: BOLD:AAD5254

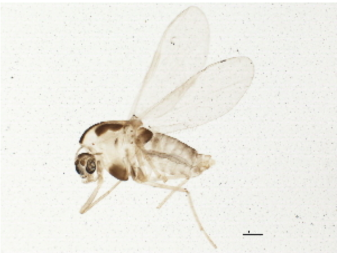

BIOUG05573-F10 [Lateral]
Chironomidae
Family: Chironomidae
BIN URI: BOLD:ACF7512

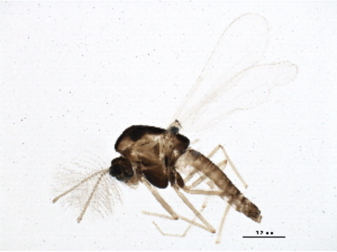

BIOUG10507-B03 [Lateral]
Thienemanniella
Family: Chironomidae
BIN URI: BOLD:AAD5253

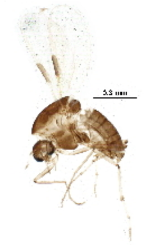

BIOUG12683-B04 [Lateral]
Chironomidae
Family: Chironomidae
BIN URI: BOLD:ACN5657

IMAGE NOT AVAILABLE

BIOUG22716-C12
Corynoneura
Family: Chironomidae

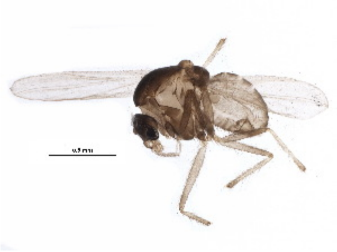

BIOUG22577-E09 [Lateral]
Nilotanypus fimbriatus
Family: Chironomidae
BIN URI: BOLD:AAE5762

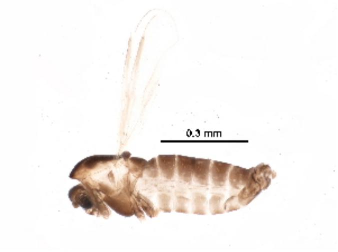

BIOUG02927-E05 [Lateral]
Chironomidae
Family: Chironomidae
BIN URI: BOLD:ACP2501

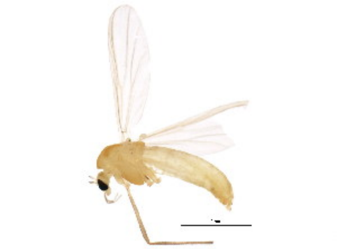

BIOUG01345-D03 [Lateral]
Bryophaenocladius ictericus
Family: Chironomidae
BIN URI: BOLD:AAM6273

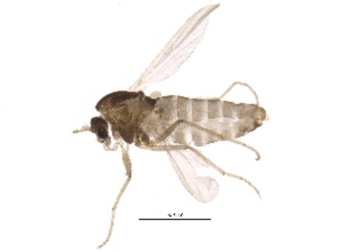

BIOUG22329-H03 [Lateral]
Chironomidae
Family: Chironomidae
BIN URI: BOLD:AAQ0599

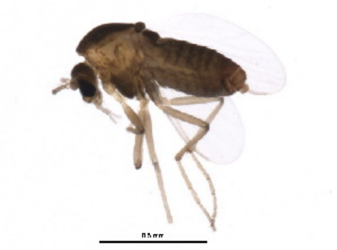

BIOUG01360-A10 [Lateral]
Chironomidae
Family: Chironomidae
BIN URI: BOLD:ABW5473

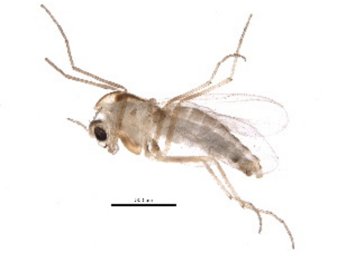

BIOUG22351-H07 [Lateral]
Limnophyes natalensis
Family: Chironomidae
BIN URI: BOLD:AAB7361

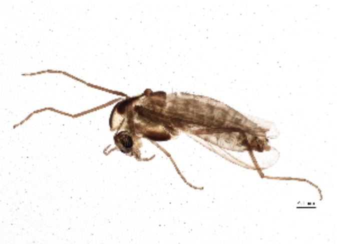

BIOUG02979-C04 [Lateral]
Limnophyes
Family: Chironomidae
BIN URI: BOLD:ABU5525

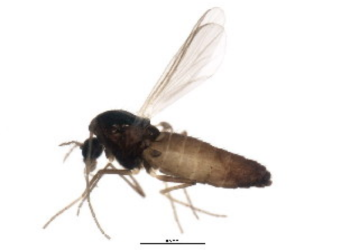

BIOUG00942-G04 [Lateral]
Limnophyes
Family: Chironomidae
BIN URI: BOLD:AAN5339

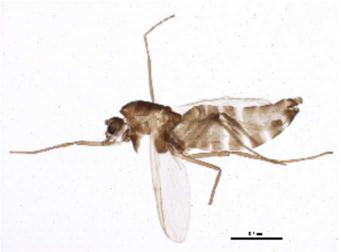

BIOUG01440-B08 [Lateral]
Chironomidae
Family: Chironomidae
BIN URI: BOLD:ABV0255

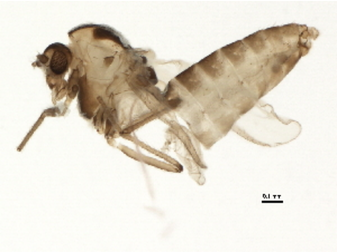

BIOUG01302-A08 [Lateral]
Chironomidae
Family: Chironomidae
BIN URI: BOLD:AAU6603

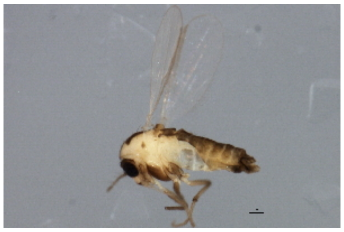

08TTML-1034 [Lateral]
Chironomidae
Family: Chironomidae
BIN URI: BOLD:AAN5336

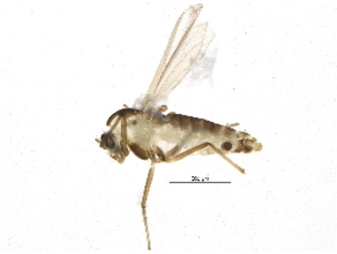

BIOUG02917-D08 [Lateral]
Chironomidae
Family: Chironomidae
BIN URI: BOLD:AAO7619

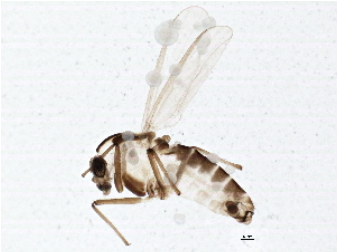

BIOUG02927-D01 [Lateral]
Chironomidae
Family: Chironomidae
BIN URI: BOLD:ACA4980

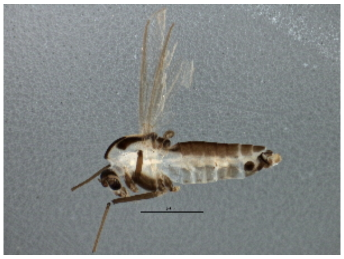

BIOUG04540-C01 [Lateral]
Chironomidae
Family: Chironomidae
BIN URI: BOLD:ACA5153

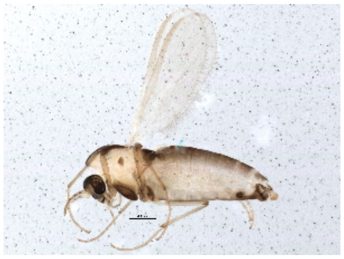

BIOUG09969-B02 [Lateral]
Orthocladiinae
Family: Chironomidae
BIN URI: BOLD:ACG2963

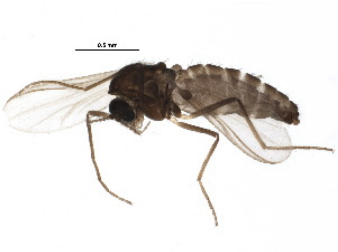

BIOUG22293-A11 [Lateral]
Limnophyes
Family: Chironomidae
BIN URI: BOLD:AAG5542

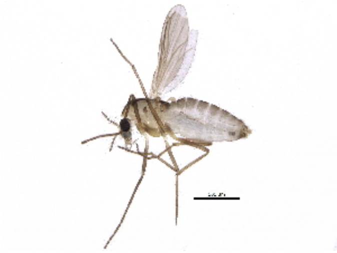

BIOUG10233-B01 [Lateral]
Limnophyes
Family: Chironomidae
BIN URI: BOLD:ACL7003

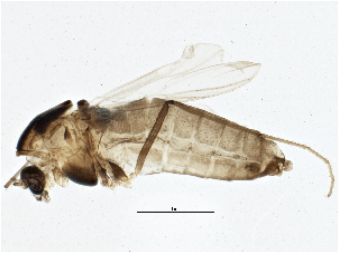

BIOUG05631-G04 [Lateral]
Chironomidae
Family: Chironomidae
BIN URI: BOLD:ACE0699

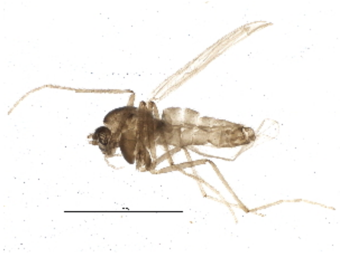

BIOUG09472-D08 [Lateral]
Chironomidae
Family: Chironomidae
BIN URI: BOLD:ACK8285

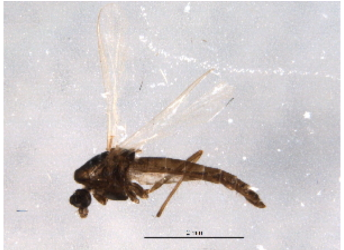

BIOUG07129-B09 [Lateral]
Metriocnemus
Family: Chironomidae
BIN URI: BOLD:ACM6798

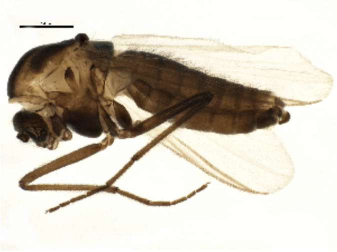

BIOUG13104-B01 [Lateral]
Metriocnemus
Family: Chironomidae
BIN URI: BOLD:ABA6408

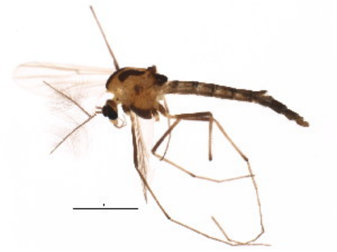

BIOUG01426-G06 [Lateral]
Orthocladiinae
Family: Chironomidae
BIN URI: BOLD:ABV0257

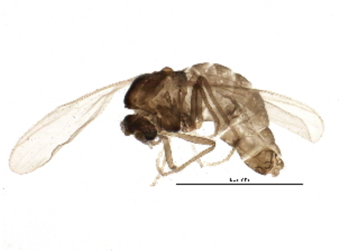

BIOUG21590-H09 [Lateral]
Orthocladiinae
Family: Chironomidae
BIN URI: BOLD:ABA1222

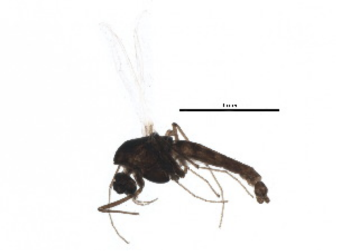

BIOUG22292-G10 [Lateral]
Chironomidae
Family: Chironomidae
BIN URI: BOLD:AAP3767

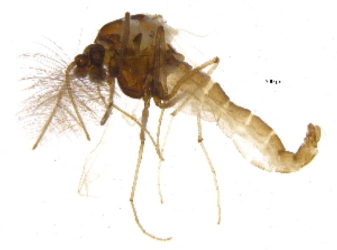

BIOUG01801-F04 [Lateral]
Chironomidae
Family: Chironomidae
BIN URI: BOLD:ACP6694

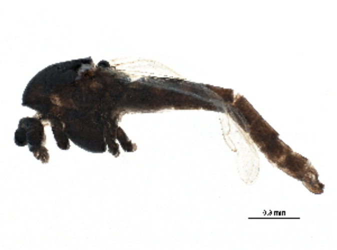

BIOUG21946-F01 [Lateral]
Orthocladiinae
Family: Chironomidae
BIN URI: BOLD:ACU4327

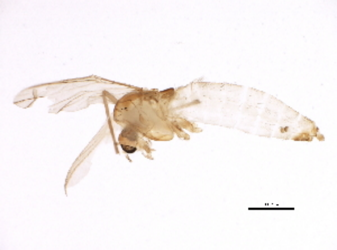

BIOUG01444-G10 [Lateral]
Chironomidae
Family: Chironomidae
BIN URI: BOLD:ABV0261

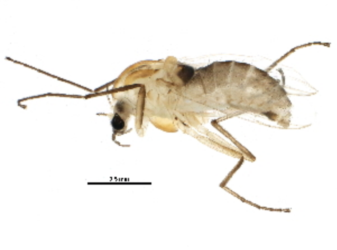

BIOUG22716-A11 [Lateral]
Psectrocladius obvius
Family: Chironomidae
BIN URI: BOLD:AAF6432

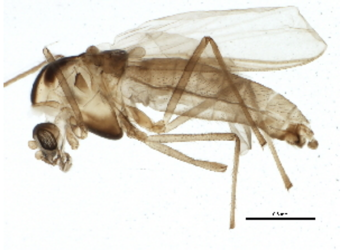

BIOUG01437-H08 [Lateral]
Chironomidae
Family: Chironomidae
BIN URI: BOLD:AAU6612

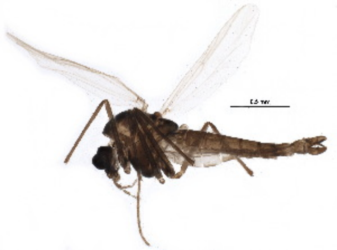

BIOUG21778-F01 [Lateral]
Orthocladiinae
Family: Chironomidae
BIN URI: BOLD:ACF7017

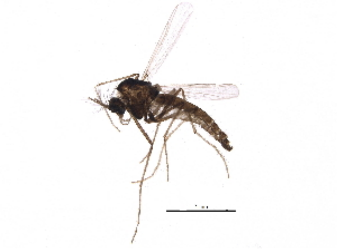

BIOUG07960-F03 [Lateral]
Orthocladiinae
Family: Chironomidae
BIN URI: BOLD:ACI9992

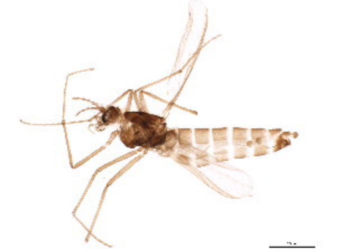

BIOUG01774-F03 [Lateral]
Chironomidae
Family: Chironomidae
BIN URI: BOLD:ABA6501

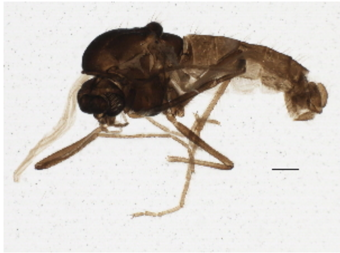

BIOUG02750-C02 [Lateral]
Orthocladiinae
Family: Chironomidae
BIN URI: BOLD:AAV5942

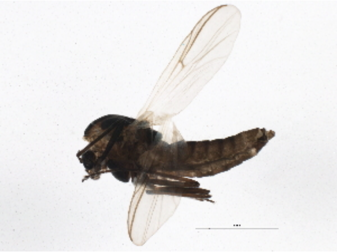

BIOUG05563-D12 [Lateral]
Chironomidae
Family: Chironomidae
BIN URI: BOLD:ABV1186

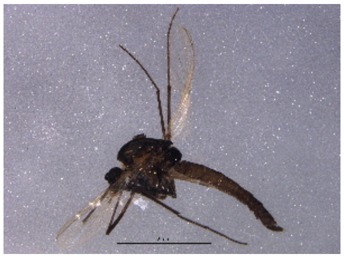

BIOUG11202-D07 [Lateral]
Rheocricotopus
Family: Chironomidae
BIN URI: BOLD:ACM1766

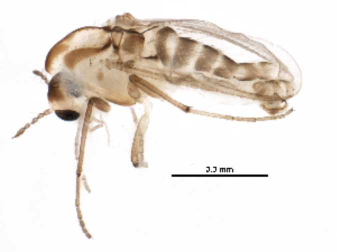

BIOUG22460-C10 [Lateral]
Chironomidae
Family: Chironomidae
BIN URI: BOLD:ACV2911

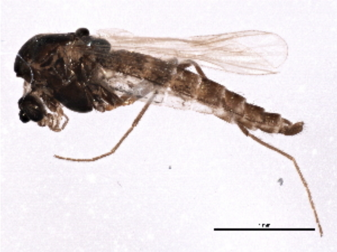

BIOUG05514-B03 [Lateral]
Cricotopus tremulus
Family: Chironomidae
BIN URI: BOLD:AAE4299

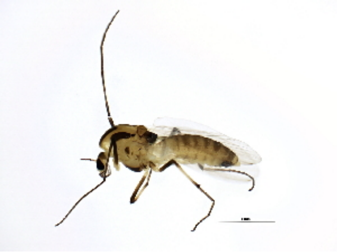

08BBDIP-2663 [Lateral]
Cricotopus
Family: Chironomidae
BIN URI: BOLD:ACH0948

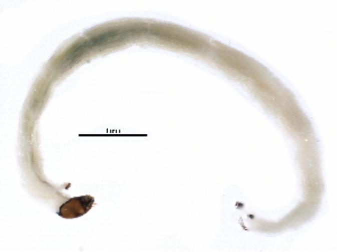

BIOUG24006-F08 [Larva]
Chironomidae
Family: Chironomidae

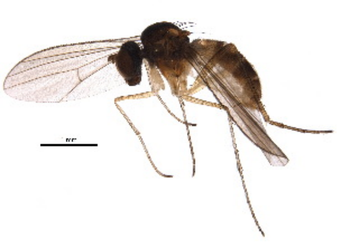

BIOUG22357-H07 [Lateral]
Chironomidae
Family: Chironomidae
BIN URI: BOLD:AAM6289

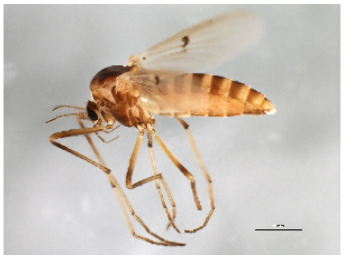

08TTML-1577 [Lateral]
Chironomidae
Family: Chironomidae
BIN URI: BOLD:AAP2998

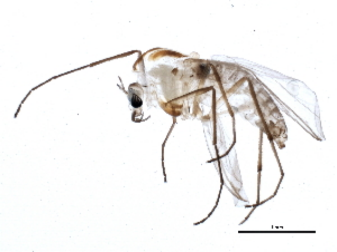

BIOUG05546-A02 [Lateral]
Psectrocladius
Family: Chironomidae
BIN URI: BOLD:AAL7382

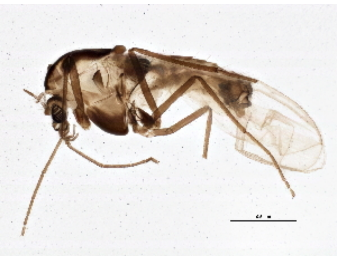

BIOUG05517-B10 [Lateral]
Chironomidae
Family: Chironomidae
BIN URI: BOLD:ABA6503

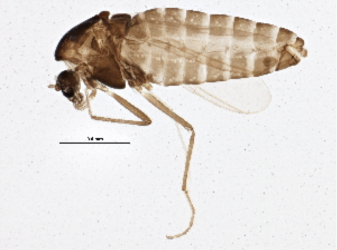

BIOUG02822-B05 [Lateral]
Chironomidae
Family: Chironomidae
BIN URI: BOLD:ABA1226

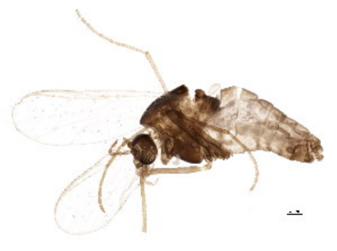

BIOUG00897-D09 [Lateral]
Chironomidae
Family: Chironomidae
BIN URI: BOLD:ABX0273

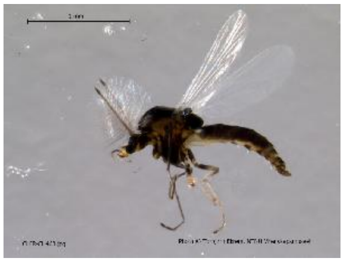

CHIR_CH463 [Lateral]
Nanocladius anderseni
Family: Chironomidae
BIN URI: BOLD:ACW4831

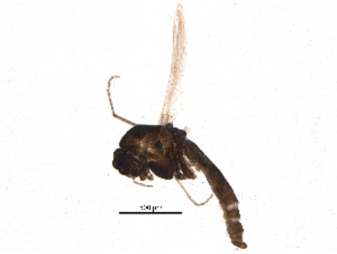

BIOUG16044-D10 [Lateral]
Metriocnemus
Family: Chironomidae
BIN URI: BOLD:ACP7395

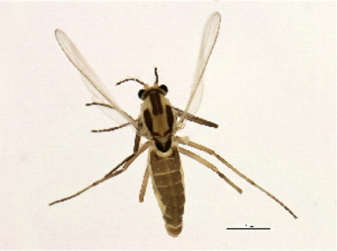

HLC-26992 [Dorsal]
Paraphaenocladius impensus
Family: Chironomidae
BIN URI: BOLD:AAC4197

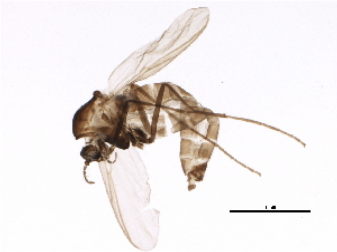

BIOUG01440-H05 [Lateral]
Bryophaenocladius sp. 8ES
Family: Chironomidae
BIN URI: BOLD:AAG1021

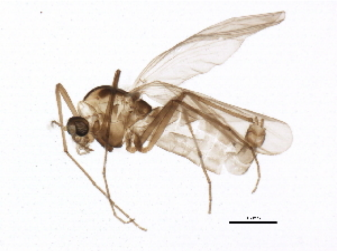

BIOUG01660-G08 [Lateral]
Chironomidae
Family: Chironomidae
BIN URI: BOLD:ABA6465

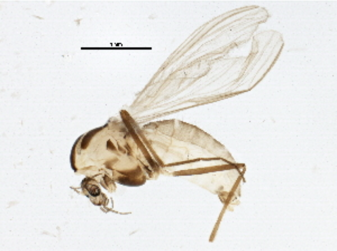

BIOUG05618-D07 [Lateral]
Heterotrissocladius
Family: Chironomidae
BIN URI: BOLD:AAN5369

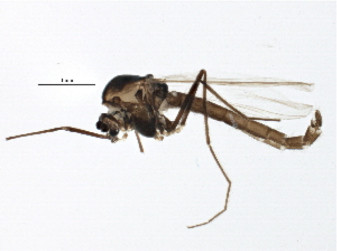

BIOUG05506-C04 [Lateral]
Chironomidae
Family: Chironomidae
BIN URI: BOLD:ACA2954

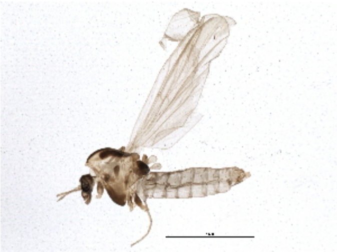

BIOUG05572-G07 [Lateral]
Chironomidae
Family: Chironomidae
BIN URI: BOLD:ACF6271

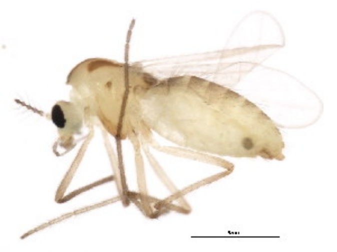

BIOUG00942-C01 [Lateral]
Chironomidae
Family: Chironomidae
BIN URI: BOLD:AAG5518

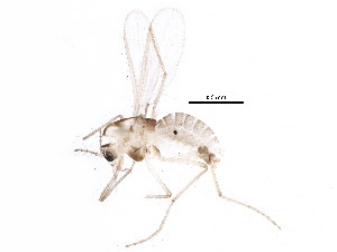

BIOUG21892-E10 [Lateral]
Chironomidae
Family: Chironomidae
BIN URI: BOLD:AAM6276

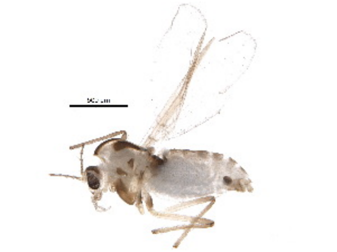

BIOUG20857-C02 [Lateral]
Diptera

BIN URI: BOLD:ACU5088

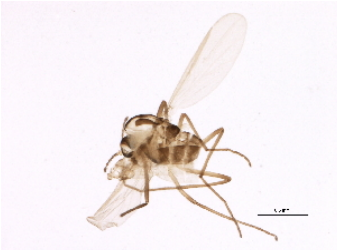

BIOUG01670-F09 [Lateral]
Chironomidae
Family: Chironomidae
BIN URI: BOLD:ACU7906

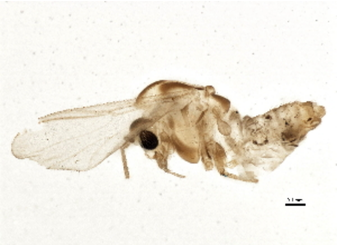

BIOUG07577-C12 [Lateral]
Chironomidae
Family: Chironomidae
BIN URI: BOLD:ACI7237

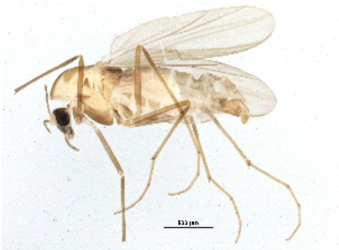

BIOUG05830-G09 [Lateral]
Orthocladiinae
Family: Chironomidae
BIN URI: BOLD:ACN1964

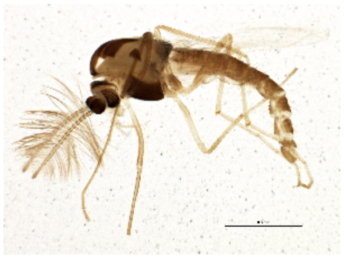

BIOUG01648-H11 [Lateral]
Chironomidae
Family: Chironomidae
BIN URI: BOLD:ABV1206

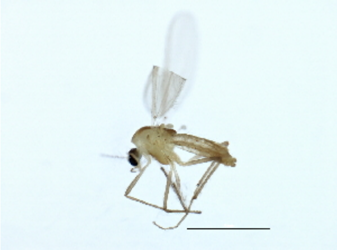

09BBEDI-2560 [Lateral]
Chironomidae
Family: Chironomidae
BIN URI: BOLD:AAM6270

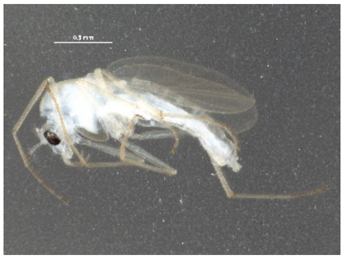

BIOUG22288-A04 [Lateral]
Chironomidae
Family: Chironomidae
BIN URI: BOLD:ACV4067

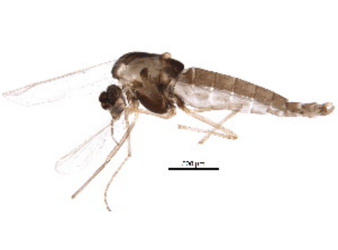

BIOUG22352-D06 [Lateral]
Paraphaenocladius
Family: Chironomidae
BIN URI: BOLD:AAV5888

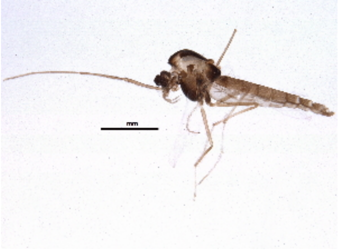

BIOUG09359-B08 [Lateral]
Paraphaenocladius
Family: Chironomidae
BIN URI: BOLD:ACL0119

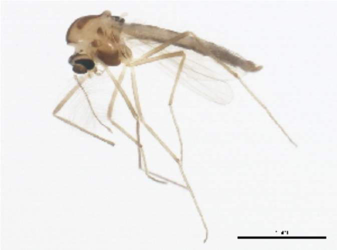

BIOUG01403-D08 [Lateral]
Chironomidae
Family: Chironomidae
BIN URI: BOLD:AAI2688

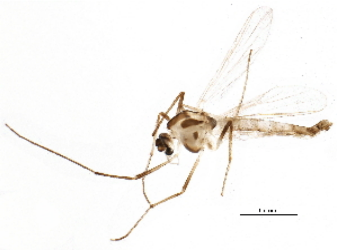

BIOUG13341-E05 [Lateral]
Parametriocnemus
Family: Chironomidae
BIN URI: BOLD:AAI2689

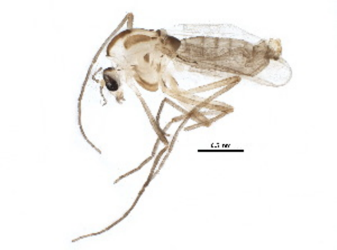

BIOUG22291-C11 [Lateral]
Parametriocnemus
Family: Chironomidae
BIN URI: BOLD:AAN5348

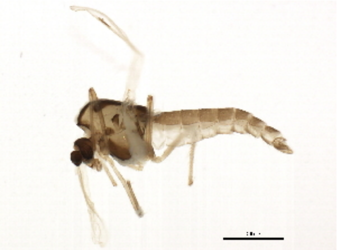

BIOUG01302-G07 [Lateral]
Chironomidae
Family: Chironomidae
BIN URI: BOLD:AAZ5599

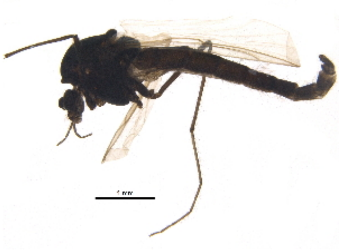

BIOUG21483-B02 [Lateral]
Chironomidae
Family: Chironomidae
BIN URI: BOLD:ACA4749

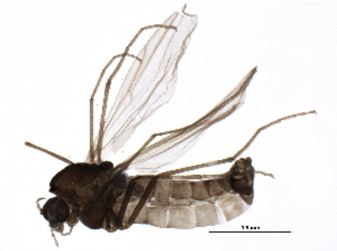

BIOUG22162-A05 [Lateral]
Chironomidae
Family: Chironomidae
BIN URI: BOLD:ACV2557

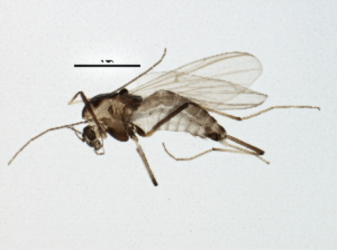

BIOUG05506-B08 [Lateral]
Cricotopus
Family: Chironomidae
BIN URI: BOLD:AAP5921

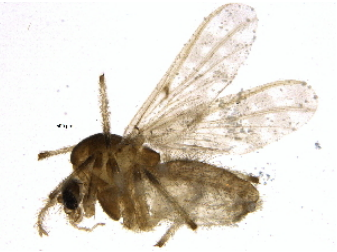

BIOUG03802-C02 [Lateral]
Chironomidae
Family: Chironomidae
BIN URI: BOLD:AAN5352

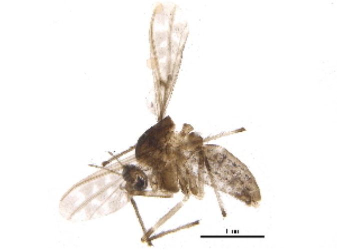

BIOUG23314-D03 [Lateral]
Chironomidae
Family: Chironomidae
BIN URI: BOLD:ACW1301

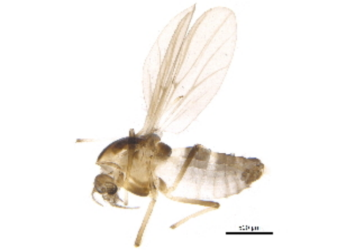

BIOUG22234-C11 [Lateral]
Cricotopus vierriensis
Family: Chironomidae
BIN URI: BOLD:ACV5403

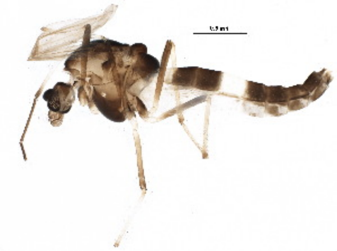

BIOUG21778-B07 [Lateral]
Cricotopus vierriensis
Family: Chironomidae
BIN URI: BOLD:ACV5404

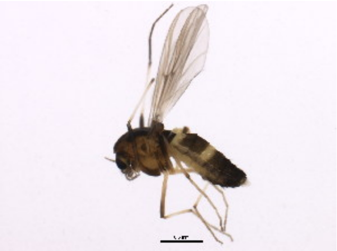

BIOUG00860-C06 [Lateral]
Cricotopus vierriensis
Family: Chironomidae
BIN URI: BOLD:AAG1005

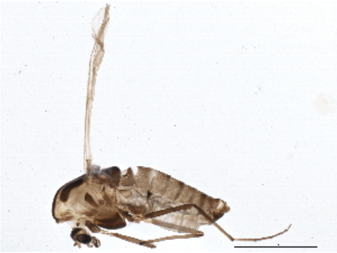

BIOUG13107-E10 [Lateral]
Eukiefferiella
Family: Chironomidae
BIN URI: BOLD:ABV1192

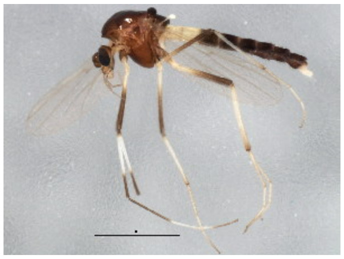

BIOUG01406-H11 [Lateral]
Chironomidae
Family: Chironomidae
BIN URI: BOLD:AAG1002

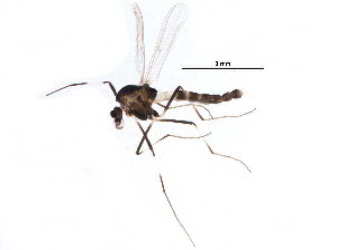

BIOUG21773-C06 [Lateral]
Cricotopus
Family: Chironomidae
BIN URI: BOLD:ABY9141

IMAGE NOT AVAILABLE

BIOUG22215-B03
Cricotopus annulator cmplx
Family: Chironomidae

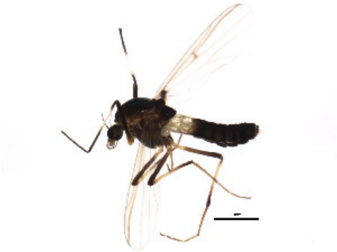

BIOUG00965-F08 [Lateral]
Cricotopus
Family: Chironomidae
BIN URI: BOLD:ABA4431

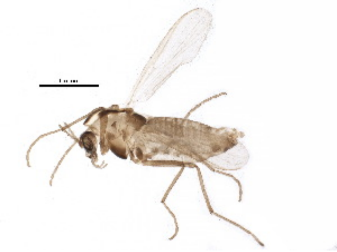

BIOUG21770-C03 [Lateral]
Gymnometriocnemus
Family: Chironomidae
BIN URI: BOLD:AAI1981

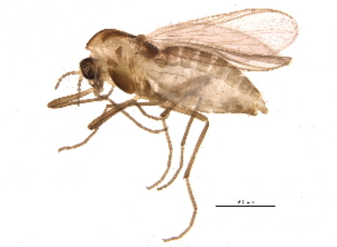

BIOUG09869-B11 [Lateral]
Gymnometriocnemus brumalis
Family: Chironomidae
BIN URI: BOLD:AAP6873

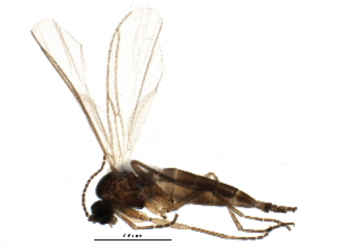

BIOUG22327-D03 [Lateral]
Sciaridae
Family: Sciaridae

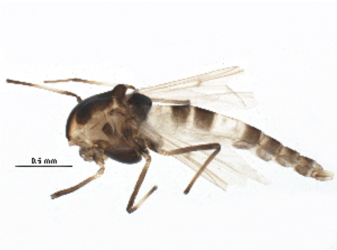

BIOUG12202-G03 [Lateral]
Cricotopus trifascia
Family: Chironomidae
BIN URI: BOLD:ACS9429

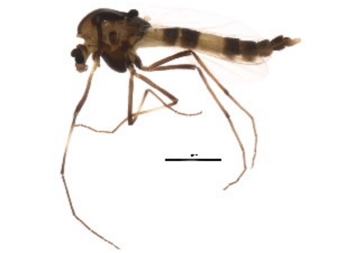

BIOUG01402-B03 [Lateral]
Chironomidae
Family: Chironomidae
BIN URI: BOLD:ACT0257

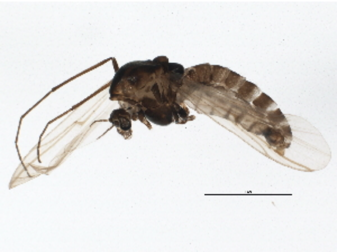

BIOUG05512-E08 [Lateral]
Cricotopus tremulus
Family: Chironomidae
BIN URI: BOLD:AAE4298

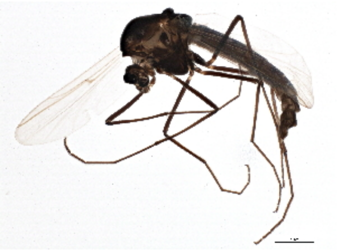

BIOUG05655-A07 [Lateral]
Cricotopus
Family: Chironomidae
BIN URI: BOLD:ACF9756

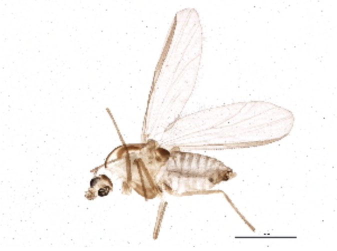

BIOUG05638-C11 [Lateral]
Parakiefferiella
Family: Chironomidae
BIN URI: BOLD:ACF7232

IMAGE NOT AVAILABLE

BIOUG22169-B09
Parakiefferiella
Family: Chironomidae

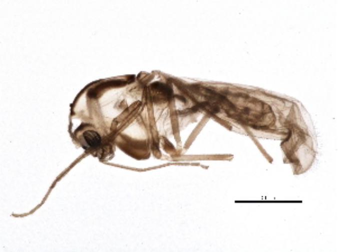

BIOUG05514-B04 [Lateral]
Parakiefferiella
Family: Chironomidae
BIN URI: BOLD:AAI2683

IMAGE NOT AVAILABLE

BIOUG22218-A10
Parakiefferiella
Family: Chironomidae

IMAGE NOT AVAILABLE

BIOUG22215-C07
Parakiefferiella
Family: Chironomidae

IMAGE NOT AVAILABLE

BIOUG22322-C08
Parakiefferiella
Family: Chironomidae

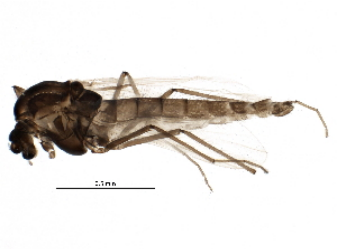

BIOUG22273-G09 [Lateral]
Parakiefferiella
Family: Chironomidae
BIN URI: BOLD:AAI2681

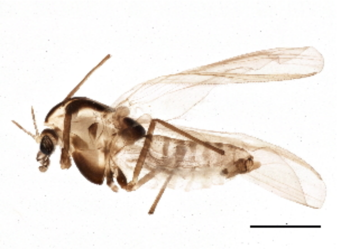

BIOUG05688-H09 [Lateral]
Chironomidae
Family: Chironomidae
BIN URI: BOLD:AAQ0604

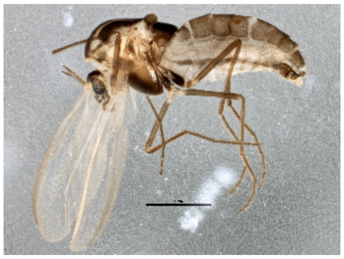

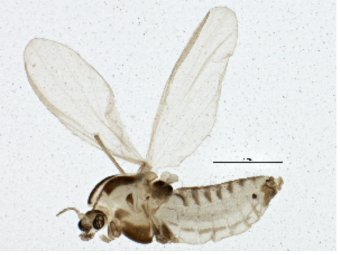

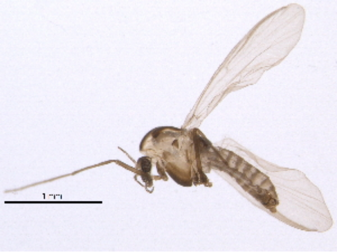

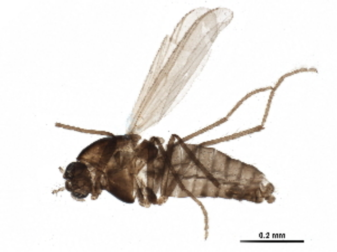

BIOUG05686-E03 [Lateral]
Chironomidae
Family: Chironomidae
BIN URI: BOLD:ACF6272

BIOUG05589-H02 [Lateral]
Chironomidae
Family: Chironomidae
BIN URI: BOLD:ACF9570

BIOUG05514-E03 [Lateral]
Orthocladiinae
Family: Chironomidae
BIN URI: BOLD:ACF8420

BIOUG22364-G01 [Lateral]
Chironomidae
Family: Chironomidae
BIN URI: BOLD:AAQ0601

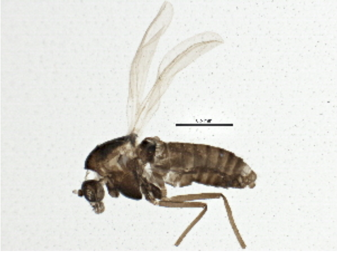

BIOUG05508-C09 [Lateral]
Chironomidae
Family: Chironomidae
BIN URI: BOLD:ABU5526

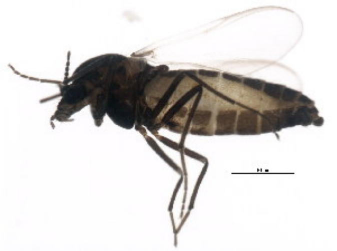

BIOUG00942-H01 [Lateral]
Chironomidae
Family: Chironomidae
BIN URI: BOLD:AAN5341

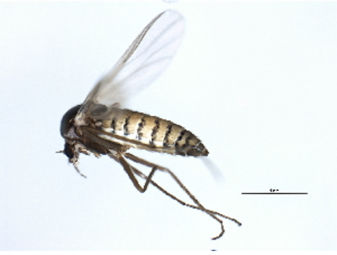

10JSROW-0382 [Lateral]
Metriocnemus sp. 4ES
Family: Chironomidae
BIN URI: BOLD:ABX5809

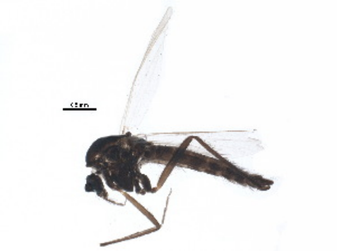

BIOUG22015-E11 [Lateral]
Chironomidae
Family: Chironomidae
BIN URI: BOLD:ACU6896

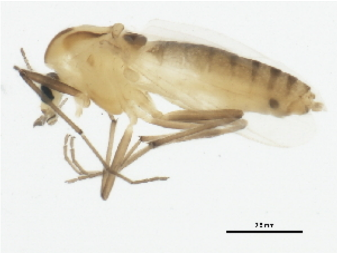

BIOUG01403-H10 [Lateral]
Orthocladiinae
Family: Chironomidae
BIN URI: BOLD:AAN5342

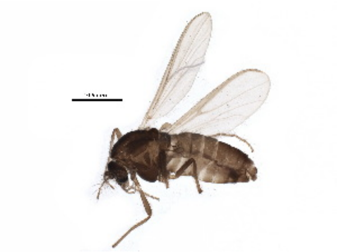

BIOUG22726-E06 [Lateral]
Chironomidae
Family: Chironomidae
BIN URI: BOLD:ACV5807

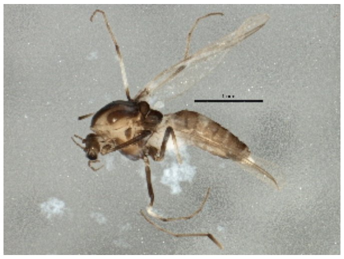

BIOUG05616-F07 [Lateral]
Cricotopus sp. 19ES
Family: Chironomidae
BIN URI: BOLD:AAP5141

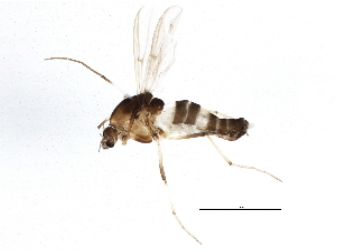

BIOUG03345-D12 [Lateral]
Cricotopus bicinctus
Family: Chironomidae
BIN URI: BOLD:ACC7282

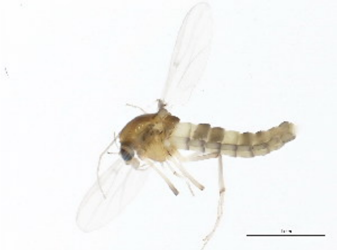

10BBDIP-0939 [Lateral]
Chironomidae
Family: Chironomidae
BIN URI: BOLD:ABY6869

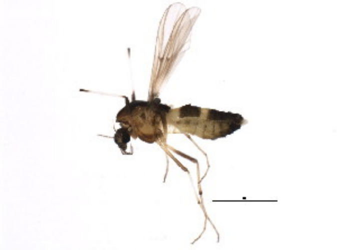

BIOUG00942-C10 [Lateral]
Cricotopus sp. 18ES
Family: Chironomidae
BIN URI: BOLD:AAG0996

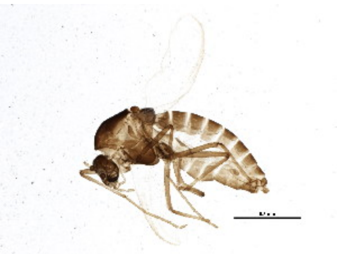

BIOUG01623-D05 [Lateral]
Smittia sp. 8ES
Family: Chironomidae
BIN URI: BOLD:ACP4736

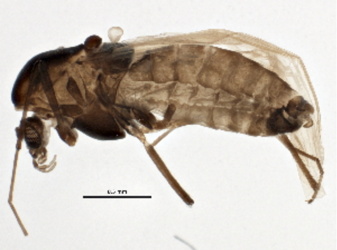

BIOUG05521-F01 [Lateral]
Chironomidae
Family: Chironomidae
BIN URI: BOLD:ACF9311

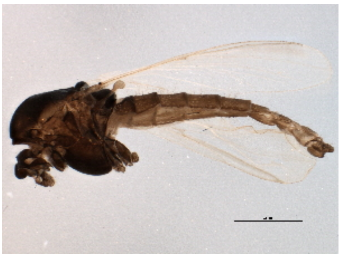

BIOUG13137-B12 [Lateral]
Eukiefferiella
Family: Chironomidae
BIN URI: BOLD:ABX5317

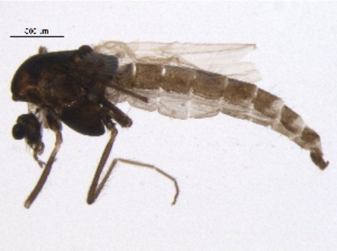

BIOUG05546-A08 [Lateral]
Eukiefferiella
Family: Chironomidae
BIN URI: BOLD:ACV1437

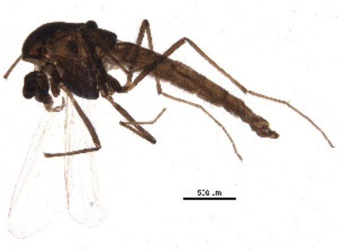

BIOUG22321-D12 [Lateral]
Eukiefferiella
Family: Chironomidae
BIN URI: BOLD:AAI5126

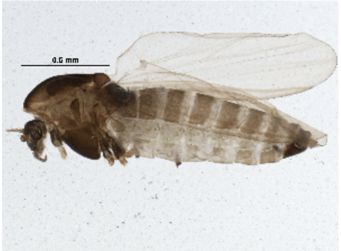

BIOUG13117-G12 [Lateral]
Eukiefferiella
Family: Chironomidae
BIN URI: BOLD:ACM6904

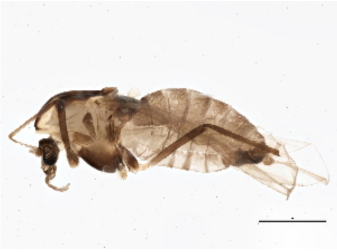

BIOUG05590-C12 [Lateral]
Eukiefferiella
Family: Chironomidae
BIN URI: BOLD:ACV1438

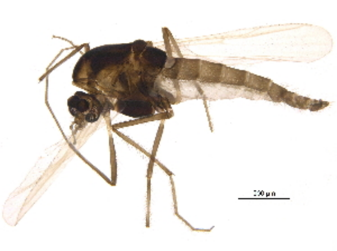

BIOUG22175-A05 [Lateral]
Eukiefferiella
Family: Chironomidae
BIN URI: BOLD:ACV2786

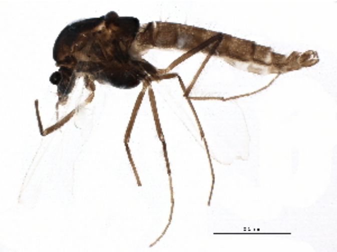

BIOUG21969-G10 [Lateral]
Eukiefferiella
Family: Chironomidae
BIN URI: BOLD:ABA1245

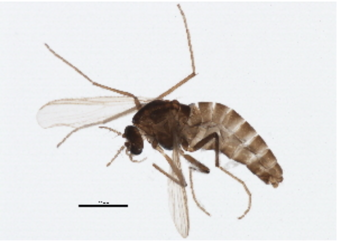

BIOUG02616-E05 [Lateral]
Chironomidae
Family: Chironomidae
BIN URI: BOLD:ABA1235

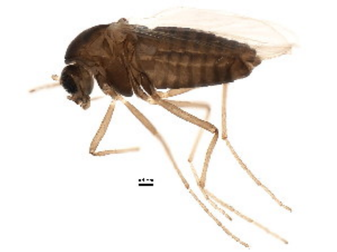

BIOUG01426-H01 [Lateral]
Orthocladiinae
Family: Chironomidae
BIN URI: BOLD:ABA1217

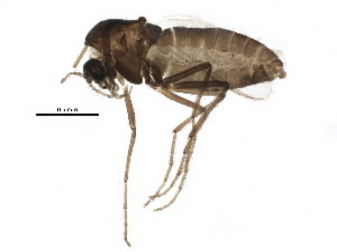

BIOUG22293-F08 [Lateral]
Chironomidae
Family: Chironomidae
BIN URI: BOLD:ACJ8377

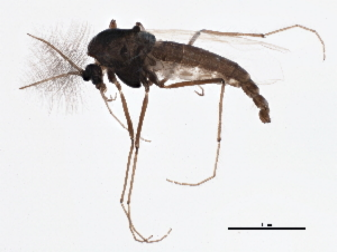

BIOUG05503-A02 [Lateral]
Smittia sp. 23ES
Family: Chironomidae
BIN URI: BOLD:AAH9641

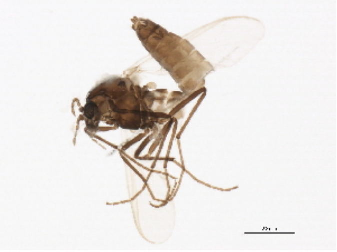

BIOUG01656-G07 [Lateral]
Smittia
Family: Chironomidae
BIN URI: BOLD:AAN5355

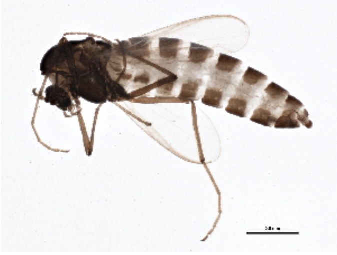

BIOUG05503-E10 [Lateral]
Smittia sp. 22ES
Family: Chironomidae
BIN URI: BOLD:AAN5358

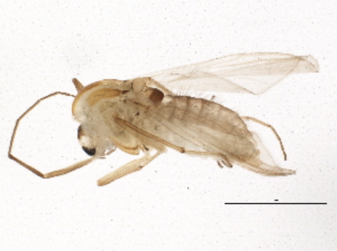

BIOUG05555-G07 [Lateral]
Chironomidae
Family: Chironomidae
BIN URI: BOLD:ACA2964

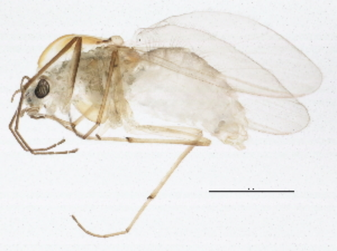

BIOUG05538-C05 [Lateral]
Orthocladiinae
Family: Chironomidae
BIN URI: BOLD:ACF8472

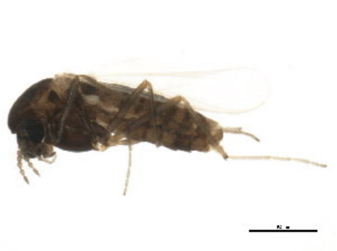

BIOUG01343-A05 [Lateral]
Smittia
Family: Chironomidae
BIN URI: BOLD:ABW7321

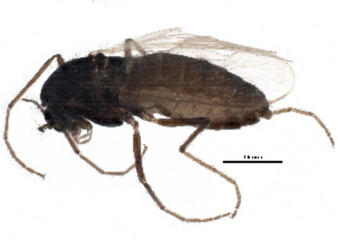

BIOUG21770-A06 [Lateral]
Smittia sp. 14ES
Family: Chironomidae
BIN URI: BOLD:AAM7064

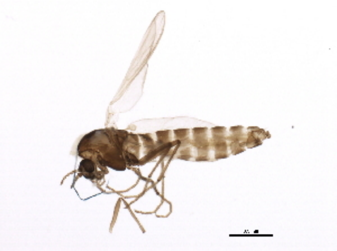

BIOUG01439-E01 [Lateral]
Chironomidae
Family: Chironomidae
BIN URI: BOLD:AAN5356

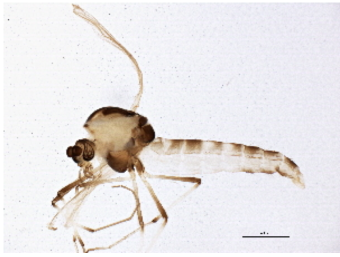

BIOUG01682-C02 [Lateral]
Cricotopus triannulatus
Family: Chironomidae
BIN URI: BOLD:AAP5920

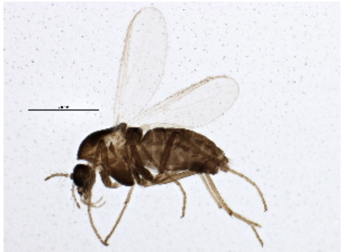

BIOUG03763-D07 [Lateral]
Smittia edwardsi
Family: Chironomidae
BIN URI: BOLD:AAF4817

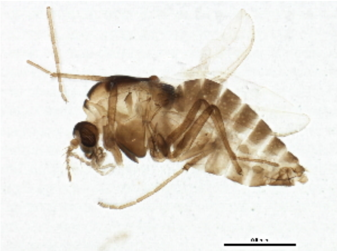

BIOUG01435-B03 [Lateral]
Chironomidae
Family: Chironomidae
BIN URI: BOLD:AAZ5601

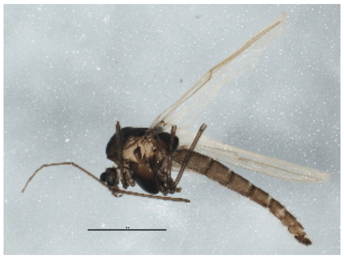

BIOUG05505-F06 [Lateral]
Orthocladius rivulorum
Family: Chironomidae
BIN URI: BOLD:AAB3988

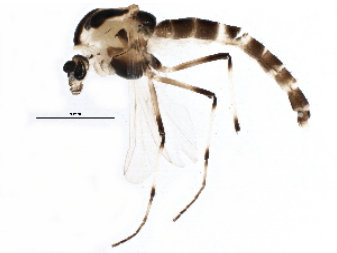

BIOUG18255-F12 [Lateral]
Cricotopus
Family: Chironomidae
BIN URI: BOLD:AAA5299

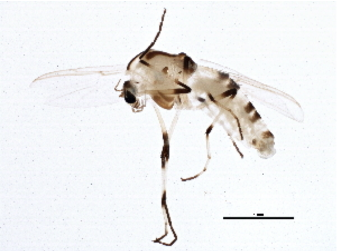

BIOUG02762-F06 [Lateral]
Chironomidae
Family: Chironomidae
BIN URI: BOLD:ACA1840

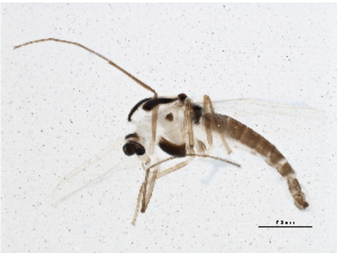

BIOUG09121-C04 [Lateral]
Chironomidae
Family: Chironomidae
BIN URI: BOLD:ACK7313

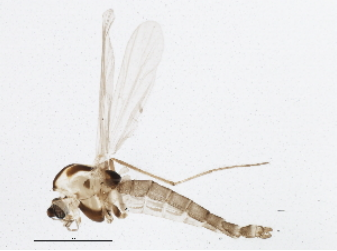

BIOUG05538-E07 [Lateral]
Orthocladius carlatus
Family: Chironomidae
BIN URI: BOLD:AAG1000

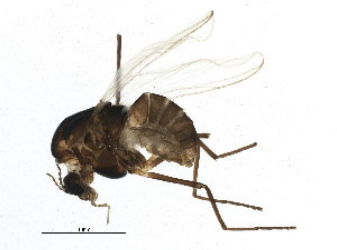

BIOUG02190-H01 [Lateral]
Chironomidae
Family: Chironomidae
BIN URI: BOLD:AAM6249

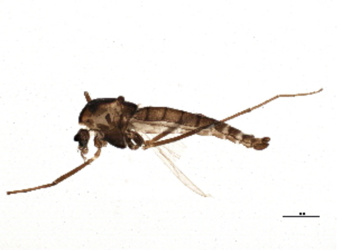

BIOUG05591-H07 [Lateral]
Orthocladius oliveri
Family: Chironomidae
BIN URI: BOLD:AAB7872

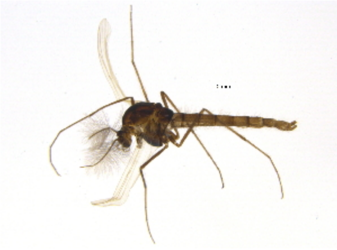

BIOUG03069-A02 [Lateral]
Orthocladius mallochi
Family: Chironomidae
BIN URI: BOLD:AAB2644

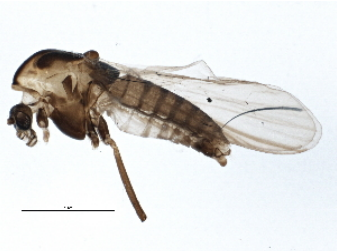

BIOUG05699-E04 [Lateral]
Orthocladius
Family: Chironomidae
BIN URI: BOLD:ACV3368

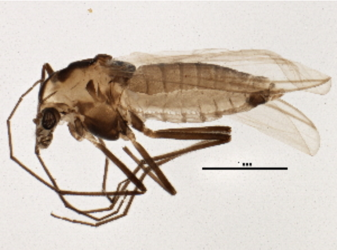

BIOUG05635-B07 [Lateral]
Orthocladius dorenus
Family: Chironomidae
BIN URI: BOLD:AAB2641

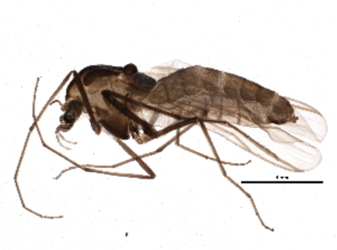

BIOUG05503-A06 [Lateral]
Orthocladius
Family: Chironomidae
BIN URI: BOLD:AAG0991

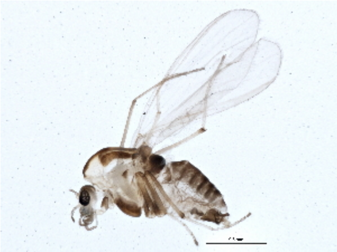

BIOUG05544-D02 [Lateral]
Chironomidae
Family: Chironomidae
BIN URI: BOLD:ACF6510

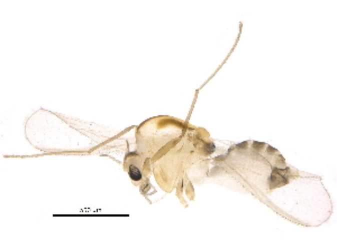

BIOUG22717-G08 [Lateral]
Chironomidae
Family: Chironomidae
BIN URI: BOLD:ACL4089

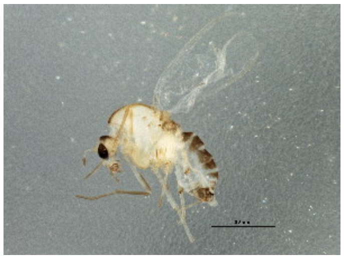

BIOUG01623-F04 [Lateral]
Chironomidae
Family: Chironomidae
BIN URI: BOLD:ABV1203

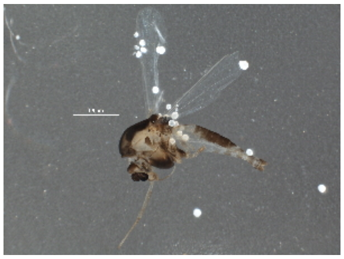

BIOUG13416-A05 [Lateral]
Chironomidae
Family: Chironomidae
BIN URI: BOLD:ACU1897

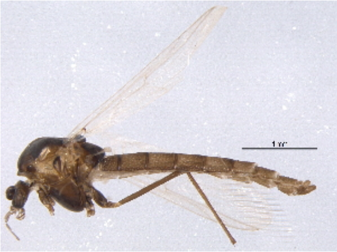

BIOUG05638-E04 [Lateral]
Orthocladius dorenus
Family: Chironomidae
BIN URI: BOLD:AAB2645

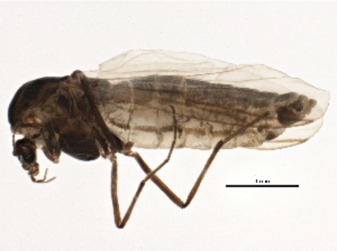

BIOUG05510-G12 [Lateral]
Chironomidae
Family: Chironomidae
BIN URI: BOLD:ACF9627

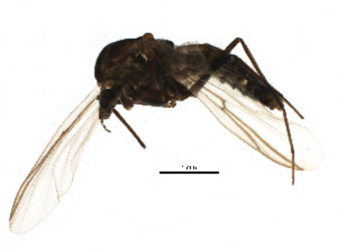

BIOUG21613-C09 [Lateral]
Orthocladius
Family: Chironomidae
BIN URI: BOLD:ACF8117

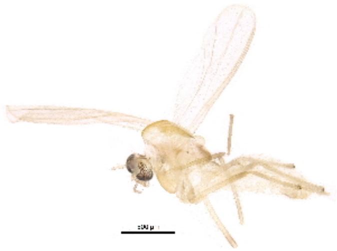

BIOUG22323-E10 [Lateral]
Chironomidae
Family: Chironomidae
BIN URI: BOLD:ABV1455

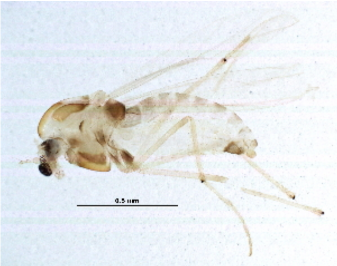

BIOUG08123-B11 [Lateral]
Chironomidae
Family: Chironomidae
BIN URI: BOLD:ACJ5130

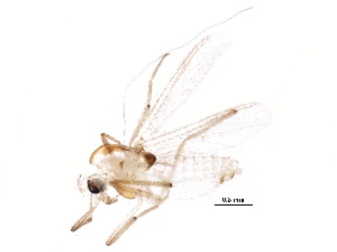

BIOUG06795-G10 [Lateral]
Chironominae
Family: Chironomidae
BIN URI: BOLD:ACR6131

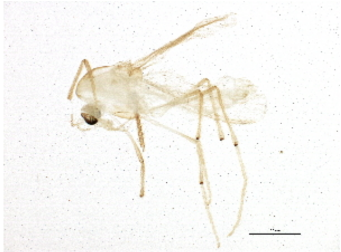

BIOUG01644-G04 [Lateral]
Chironomidae
Family: Chironomidae
BIN URI: BOLD:ACR6132

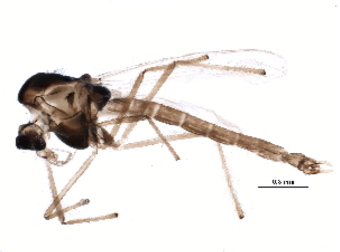

BIOUG10220-G10 [Lateral]
Chironomidae
Family: Chironomidae
BIN URI: BOLD:ACR6101

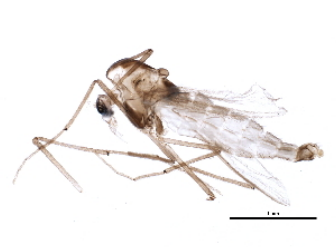

BIOUG05546-D03 [Lateral]
Paratanytarsus
Family: Chironomidae
BIN URI: BOLD:AAP2907

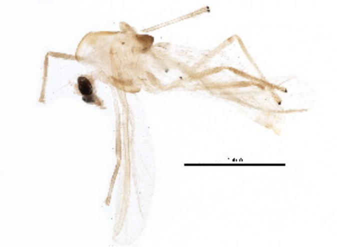

BIOUG22466-D04 [Lateral]
Paratanytarsus sp. TE03
Family: Chironomidae
BIN URI: BOLD:AAE3675

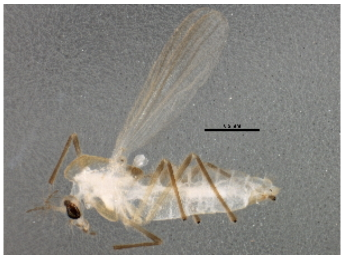

BIOUG03750-E07 [Lateral]
Paratanytarsus
Family: Chironomidae
BIN URI: BOLD:ACE7452

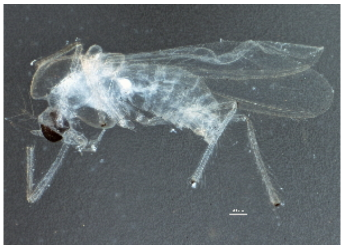

BIOUG05794-G06 [Lateral]
Chironomidae
Family: Chironomidae
BIN URI: BOLD:ACG2927

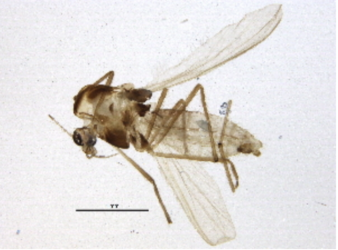

BIOUG05880-E07 [Lateral]
Paratanytarsus
Family: Chironomidae
BIN URI: BOLD:ACF7858

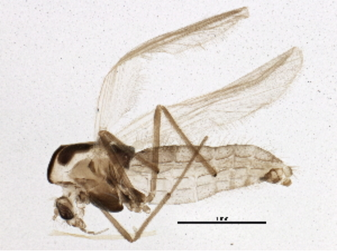

BIOUG05516-G08 [Lateral]
Chironomidae
Family: Chironomidae
BIN URI: BOLD:ACF7859

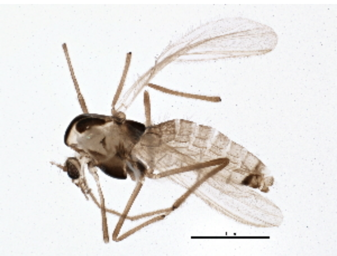

BIOUG05514-C06 [Lateral]
Chironomidae
Family: Chironomidae
BIN URI: BOLD:ABV9028

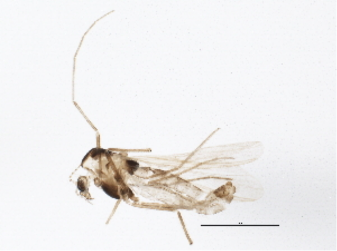

BIOUG05547-A09 [Lateral]
Chironomidae
Family: Chironomidae
BIN URI: BOLD:ACV1253

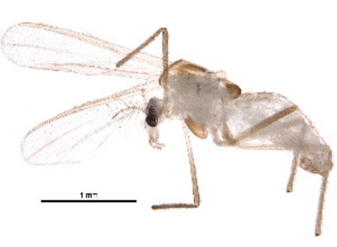

BIOUG22352-D11 [Lateral]
Paratanytarsus dissimilis
Family: Chironomidae
BIN URI: BOLD:AAE3698

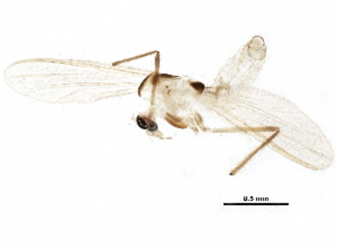

BIOUG21909-A06 [Lateral]
Paratanytarsus
Family: Chironomidae
BIN URI: BOLD:AAI2606

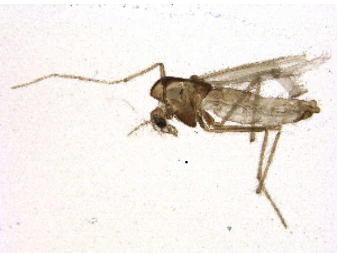

BIOUG03983-A09 [Lateral]
Paratanytarsus
Family: Chironomidae
BIN URI: BOLD:ACC1156

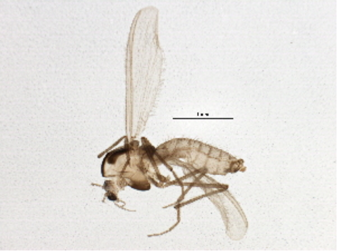

BIOUG05510-F06 [Lateral]
Paratanytarsus grimmii
Family: Chironomidae
BIN URI: BOLD:AAD1485

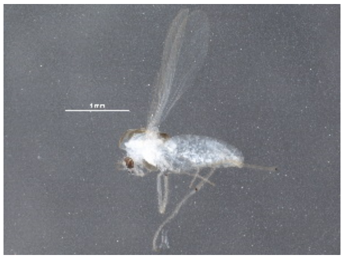

BIOUG22295-E09 [Lateral]
Paratanytarsus laccophilus
Family: Chironomidae
BIN URI: BOLD:ACF2457

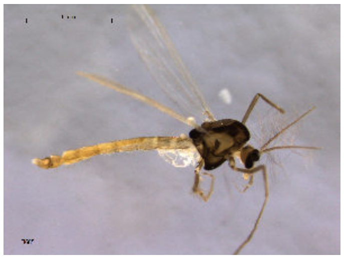

Finnmark685 [Lateral]
Paratanytarsus laccophilus
Family: Chironomidae
BIN URI: BOLD:AAC8842

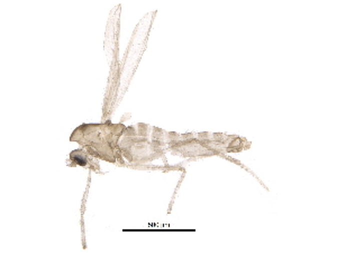

BIOUG23312-F12 [Lateral]
Chironomidae
Family: Chironomidae
BIN URI: BOLD:ACW1260

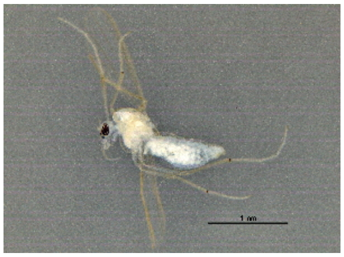

BIOUG22717-E03 [Lateral]
Tanytarsus guerlus
Family: Chironomidae
BIN URI: BOLD:AAC4523

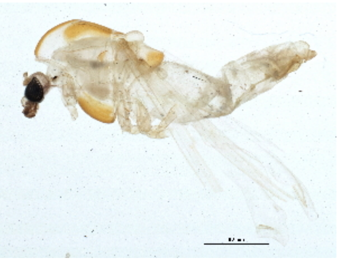

BIOUG06542-B10 [Lateral]
Chironomidae
Family: Chironomidae
BIN URI: BOLD:AAG5465

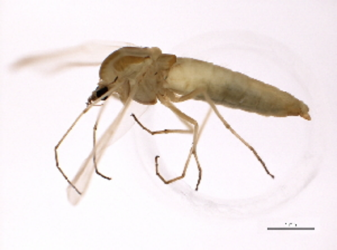

09BBDIP-1691 [Lateral]
Chironomidae
Family: Chironomidae
BIN URI: BOLD:AAM6209

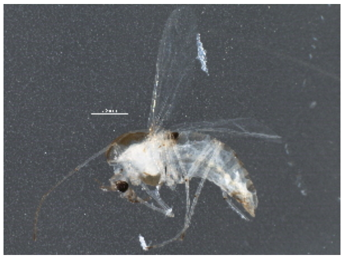

BIOUG12303-D11 [Lateral]
Chironominae
Family: Chironomidae
BIN URI: BOLD:ACM9846

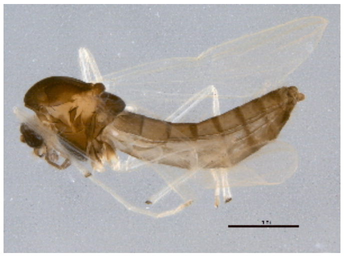

BIOUG04684-A10 [Lateral]
Chironomidae
Family: Chironomidae
BIN URI: BOLD:ACD9755

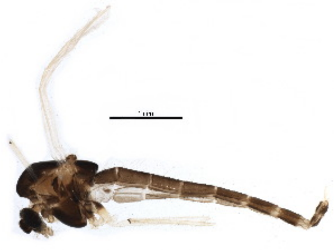

BIOUG22467-G05 [Lateral]
Phaenopsectra
Family: Chironomidae
BIN URI: BOLD:AAM6287

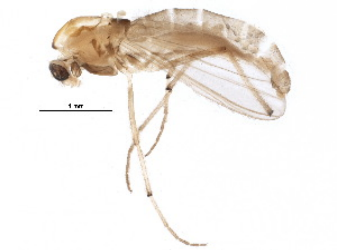

BIOUG22415-H06 [Lateral]
Chironomidae
Family: Chironomidae
BIN URI: BOLD:ACI3882

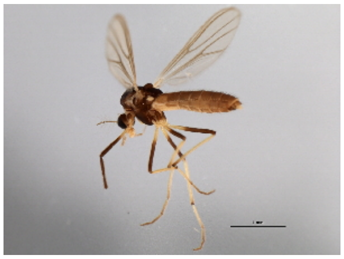

08TTML-2077 [Lateral]
Paratendipes
Family: Chironomidae
BIN URI: BOLD:AAG5473

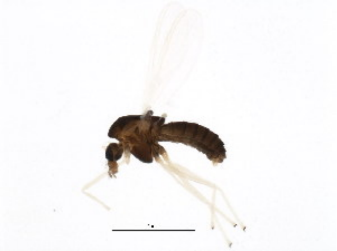

BIOUG00978-G04 [Lateral]
Lauterborniella agrayloides
Family: Chironomidae
BIN URI: BOLD:AAN5343

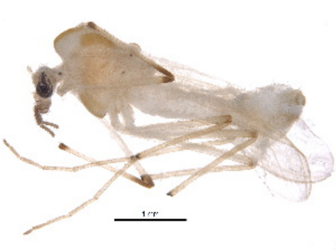

BIOUG22356-C06 [Lateral]
Microtendipes pedellus
Family: Chironomidae
BIN URI: BOLD:AAE0707

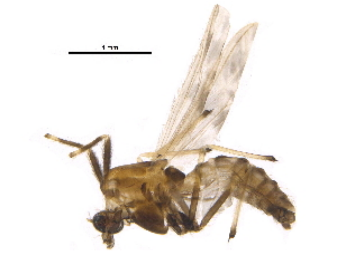

BIOUG23078-H08 [Lateral]
Chironominae
Family: Chironomidae
BIN URI: BOLD:ABV1452

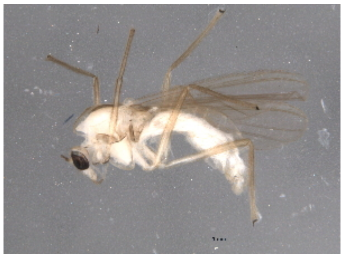

BIOUG08642-C02 [Lateral]
Polypedilum
Family: Chironomidae
BIN URI: BOLD:ACJ7070

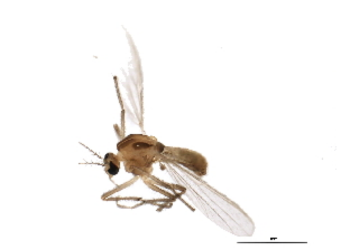

10JSROW-1726 [Lateral]
Chironomidae
Family: Chironomidae
BIN URI: BOLD:AAP8069

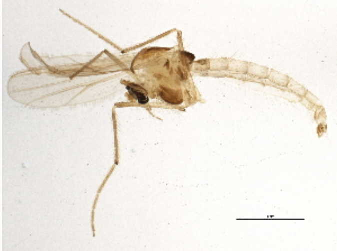

BIOUG05172-G01 [Lateral]
Chironomidae
Family: Chironomidae
BIN URI: BOLD:ACG4079

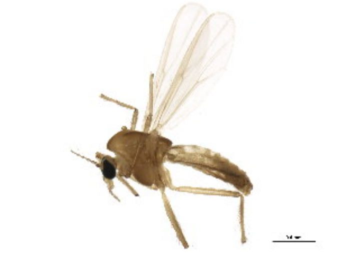

BIOUG01835-D10 [Lateral]
Chironomidae
Family: Chironomidae
BIN URI: BOLD:ACM1304

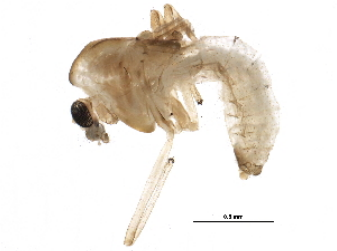

BIOUG21891-H08 [Lateral]
Chironomidae
Family: Chironomidae
BIN URI: BOLD:ACV5080

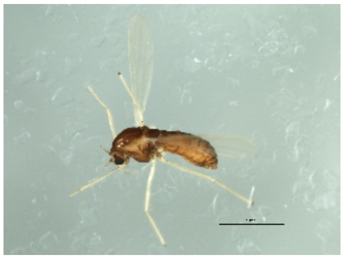

08TTML-1257 [Lateral]
Chironomidae
Family: Chironomidae
BIN URI: BOLD:AAN5332

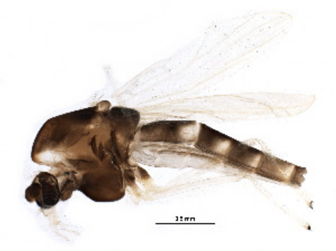

BIOUG22416-G05 [Lateral]
Polypedilum
Family: Chironomidae
BIN URI: BOLD:AAG5541

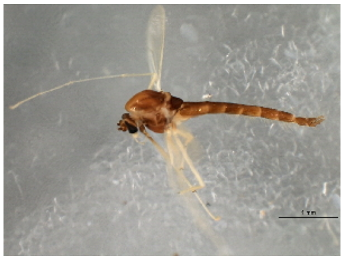

08TTML-1484 [Lateral]
Chironomidae
Family: Chironomidae
BIN URI: BOLD:AAN5334

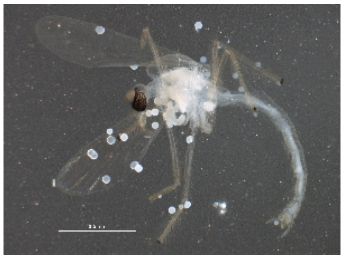

BIOUG22730-H11 [Lateral]
Polypedilum
Family: Chironomidae
BIN URI: BOLD:ABA6440

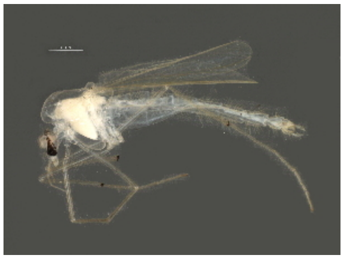

BIOUG08875-C10 [Lateral]
Chironominae
Family: Chironomidae
BIN URI: BOLD:ACK7314

IMAGE NOT AVAILABLE

BIOUG22330-D06
Polypedilum convictum
Family: Chironomidae

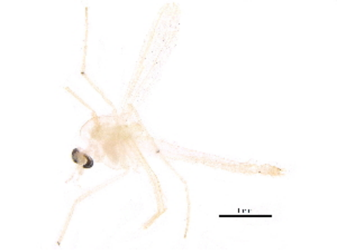

BIOUG22323-G01 [Lateral]
Polypedilum
Family: Chironomidae
BIN URI: BOLD:AAD1395

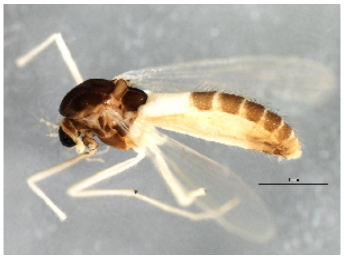

08TTML-1800 [Lateral]
Chironomidae
Family: Chironomidae
BIN URI: BOLD:AAG5516

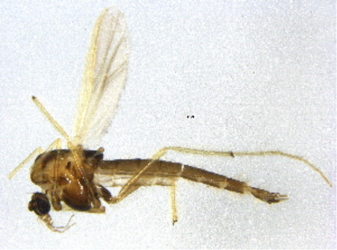

BIOUG03899-C02 [Lateral]
Chironominae
Family: Chironomidae
BIN URI: BOLD:ACB9392

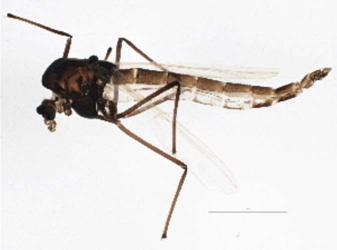

BIOUG05715-G06 [Lateral]
Chironomidae
Family: Chironomidae
BIN URI: BOLD:ACF8174

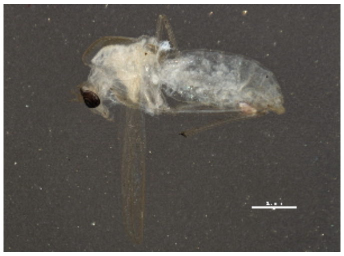

BIOUG07641-D06 [Lateral]
Chironominae
Family: Chironomidae
BIN URI: BOLD:AAN5344

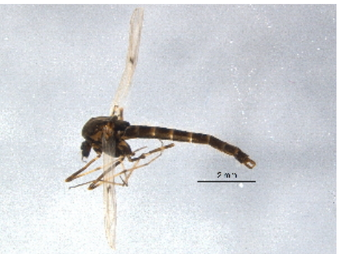

BIOUG05700-H03 [Lateral]
Chironomidae
Family: Chironomidae
BIN URI: BOLD:AAP3005

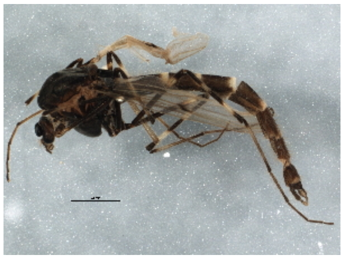

BIOUG05679-G02 [Lateral]
Chironomidae
Family: Chironomidae
BIN URI: BOLD:AAQ3439

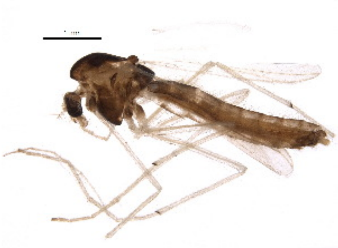

BIOUG09961-E12 [Lateral]
Chironomidae
Family: Chironomidae
BIN URI: BOLD:AAZ5511

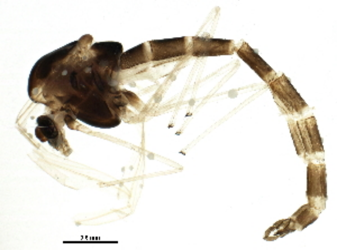

BIOUG21891-B07 [Lateral]
Chironomidae
Family: Chironomidae
BIN URI: BOLD:ACV5306

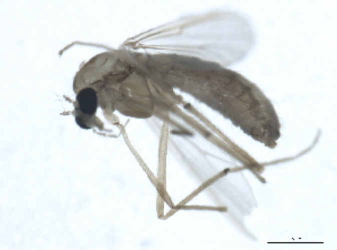

10BBDIP-1535 [Lateral]
Chironomidae
Family: Chironomidae
BIN URI: BOLD:AAD1397

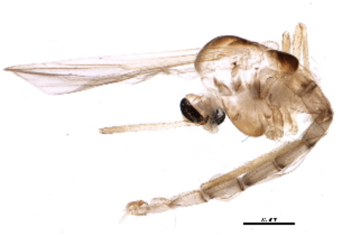

BIOUG16168-F03 [Lateral]
Polypedilum
Family: Chironomidae
BIN URI: BOLD:ACA3244

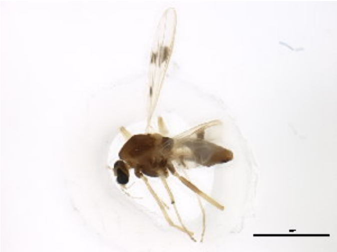

BIOUG01410-E06 [Lateral]
Polypedilum
Family: Chironomidae
BIN URI: BOLD:ABA0772

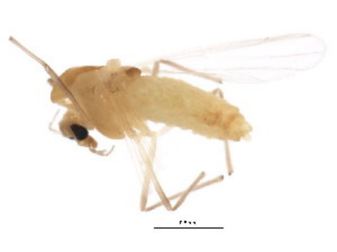

BIOUG01400-A04 [Lateral]
Chironomidae
Family: Chironomidae
BIN URI: BOLD:AAH0042

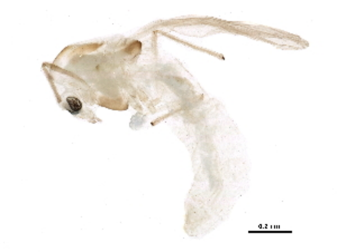

BIOUG22235-D05 [Lateral]
Cladotanytarsus
Family: Chironomidae
BIN URI: BOLD:AAI4121

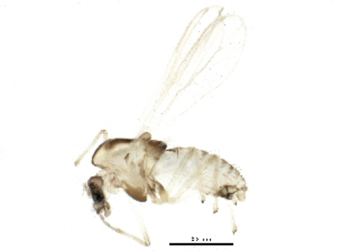

BIOUG22716-C11 [Lateral]
Chironomidae
Family: Chironomidae
BIN URI: BOLD:ACC8280

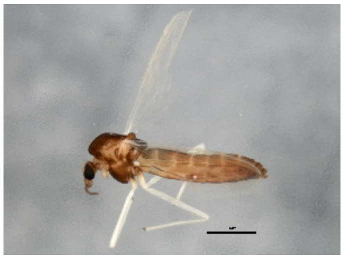

BIOUG01422-B11 [Lateral]
Chironomidae
Family: Chironomidae
BIN URI: BOLD:AAG0920

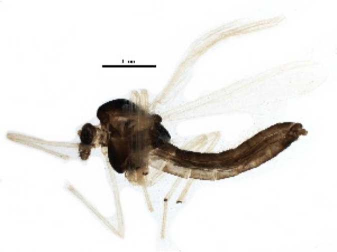

BIOUG22239-F10 [Lateral]
Tribelos
Family: Chironomidae
BIN URI: BOLD:ACN2444

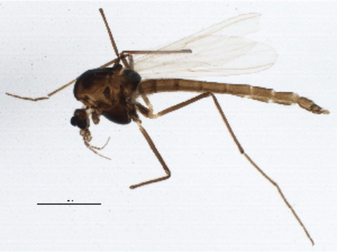

BIOUG03279-E12 [Lateral]
Chironomidae
Family: Chironomidae
BIN URI: BOLD:ACA7493

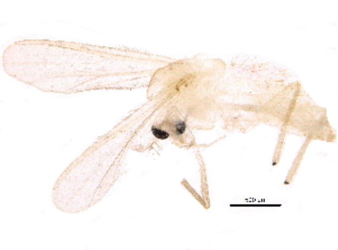

BIOUG22329-B05 [Lateral]
Tanytarsus wirthi
Family: Chironomidae
BIN URI: BOLD:AAD2144

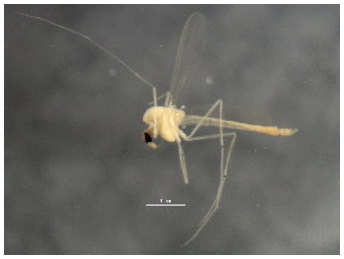

BIOUG01389-E07 [Lateral]
Chironomidae
Family: Chironomidae
BIN URI: BOLD:ACJ3722

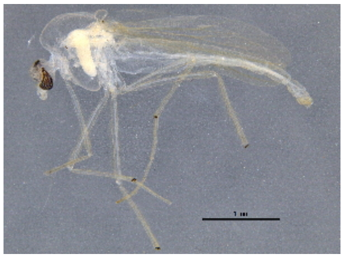

BIOUG22237-E12 [Lateral]
Tanytarsus mendax
Family: Chironomidae
BIN URI: BOLD:ACV3832

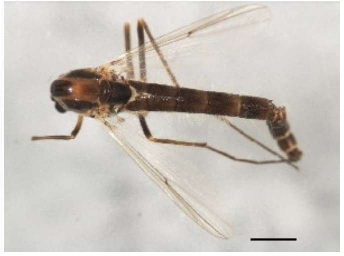

10JSROW-1043 [Dorsal]
Chironomidae
Family: Chironomidae
BIN URI: BOLD:AAP6878

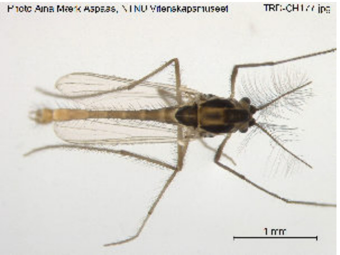

TRD-CH177 [Dorsal]
Cladotanytarsus atridorsum
Family: Chironomidae
BIN URI: BOLD:AAJ3263

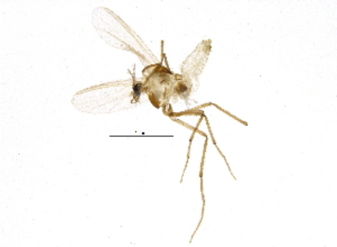

BIOUG03122-A10 [Lateral]
Cladotanytarsus
Family: Chironomidae
BIN URI: BOLD:ACA3036

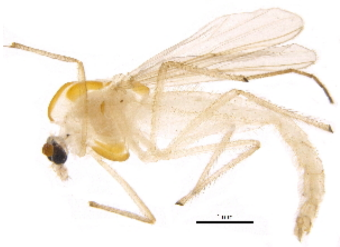

BIOUG22323-E04 [Lateral]
Chironomidae
Family: Chironomidae
BIN URI: BOLD:AAM6286

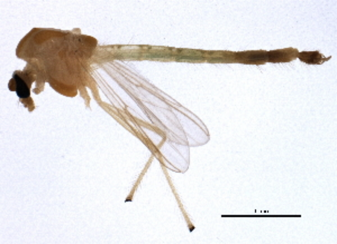

BIOUG01686-F07 [Lateral]
Dicrotendipes
Family: Chironomidae
BIN URI: BOLD:ABV9293

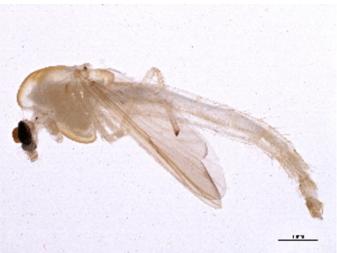

BIOUG03125-F03 [Lateral]
Chironominae
Family: Chironomidae
BIN URI: BOLD:ACA7555

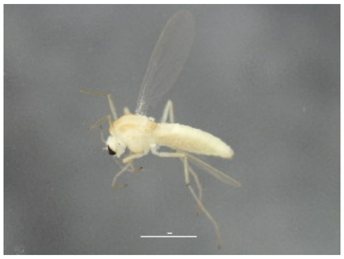

10JSROW-0838 [Lateral]
Chironomidae
Family: Chironomidae
BIN URI: BOLD:AAV5938

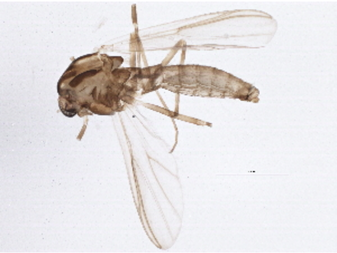

BIOUG08643-C02 [Lateral]
Dicrotendipes
Family: Chironomidae
BIN URI: BOLD:AAG5423

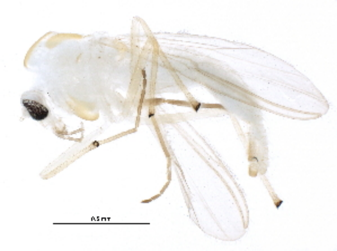

BIOUG22084-D08 [Lateral]
Dicrotendipes tritomus
Family: Chironomidae
BIN URI: BOLD:AAC0706

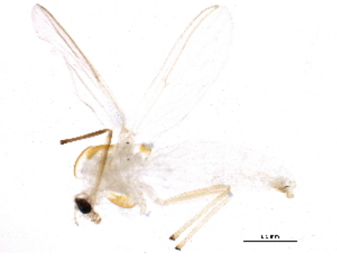

BIOUG22084-E01 [Lateral]
Chironomidae
Family: Chironomidae
BIN URI: BOLD:AAG5464

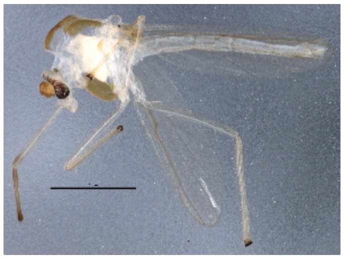

BIOUG03554-G02 [Lateral]
Dicrotendipes
Family: Chironomidae
BIN URI: BOLD:AAN5383

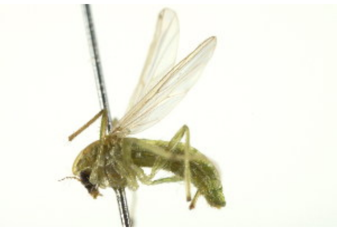

PCPP10-0284 [Lateral]
Dicrotendipes
Family: Chironomidae
BIN URI: BOLD:AAP6882

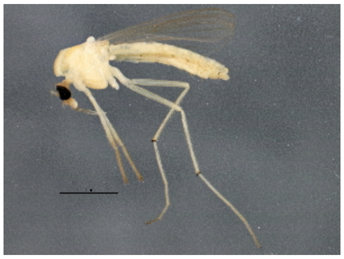

BIOUG03138-D03 [Lateral]
Chironomidae
Family: Chironomidae
BIN URI: BOLD:AAL7329

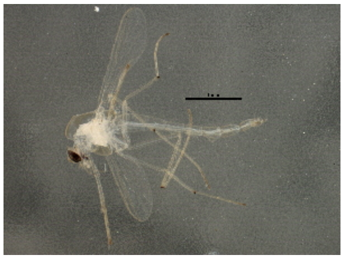

BIOUG03909-H11 [Lateral]
Chironomidae
Family: Chironomidae
BIN URI: BOLD:AAN5354

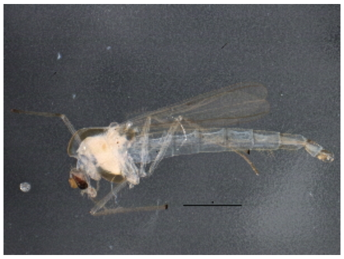

BIOUG03346-D03 [Lateral]
Chironomidae
Family: Chironomidae
BIN URI: BOLD:AAQ0607

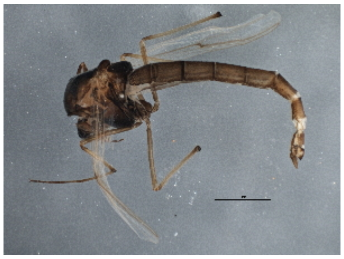

BIOUG08376-E02 [Lateral]
Dicrotendipes
Family: Chironomidae
BIN URI: BOLD:AAI6244

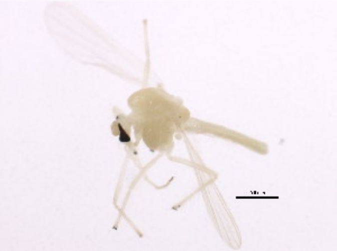

BIOUG00860-D01 [Lateral]
Chironomidae
Family: Chironomidae
BIN URI: BOLD:ABA0771

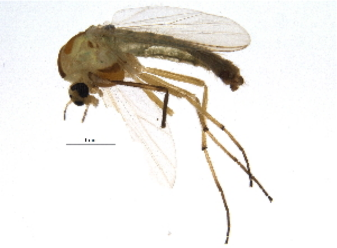

BIOUG02758-C08 [Dorsal]
Chironominae
Family: Chironomidae
BIN URI: BOLD:AAG5506

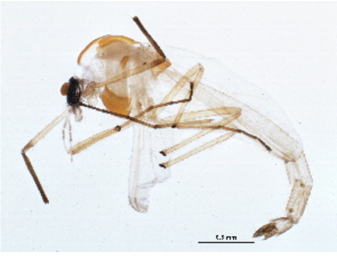

BIOUG12412-H11 [Lateral]
Chironominae
Family: Chironomidae
BIN URI: BOLD:ACN5893

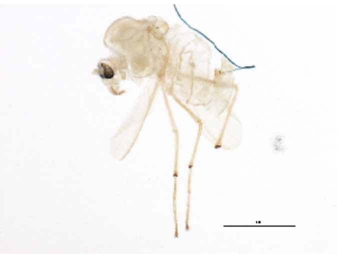

BIOUG01774-D08 [Lateral]
Parachironomus
Family: Chironomidae
BIN URI: BOLD:ACE5981

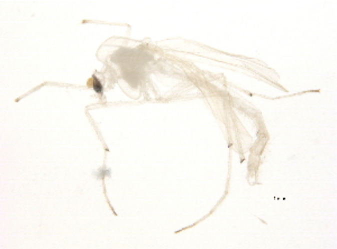

BIOUG03695-A05 [Lateral]
Parachironomus
Family: Chironomidae
BIN URI: BOLD:ACB9399

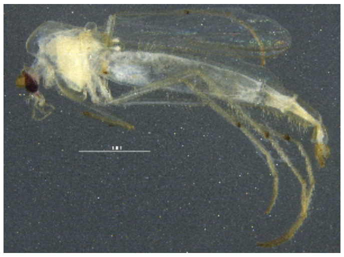

BIOUG03103-C04 [Lateral]
Parachironomus
Family: Chironomidae
BIN URI: BOLD:ABX7479

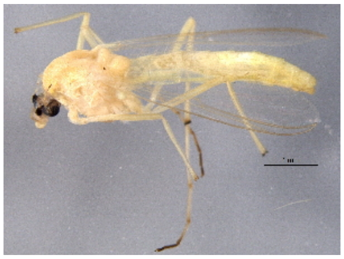

BIOUG02757-B06 [Lateral]
Chironominae
Family: Chironomidae
BIN URI: BOLD:ACA8801

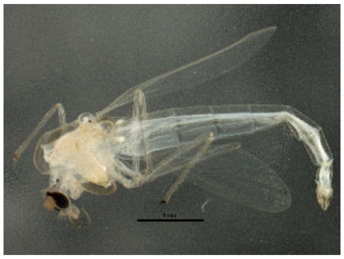

BIOUG02864-F06 [Lateral]
Chironomidae
Family: Chironomidae
BIN URI: BOLD:ACA9312

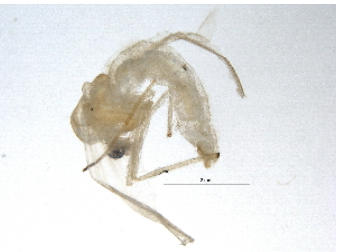

BIOUG03167-F04 [Lateral]
Einfeldia
Family: Chironomidae
BIN URI: BOLD:AAG5475

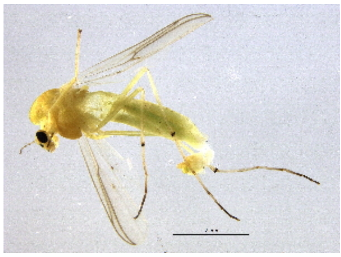

BIOUG04935-F10 [Lateral]
Einfeldia
Family: Chironomidae
BIN URI: BOLD:ACP6588

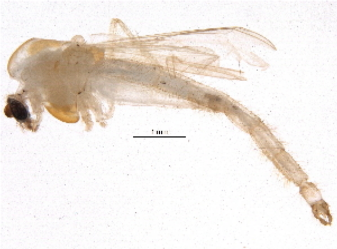

BIOUG09961-F06 [Lateral]
Einfeldia
Family: Chironomidae
BIN URI: BOLD:ACV5157

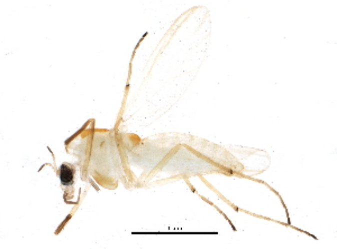

BIOUG08608-C12 [Lateral]
Chironomidae
Family: Chironomidae
BIN URI: BOLD:ACK1520

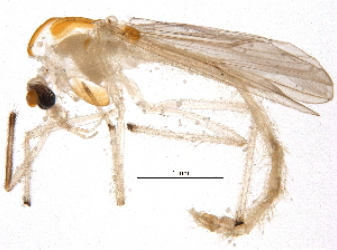

BIOUG06890-H09 [Lateral]
Chironominae
Family: Chironomidae
BIN URI: BOLD:ACL5434

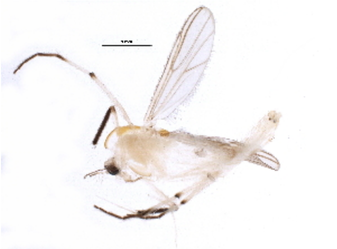

BIOUG09955-G10 [Lateral]
Chironomidae
Family: Chironomidae
BIN URI: BOLD:ACI4243

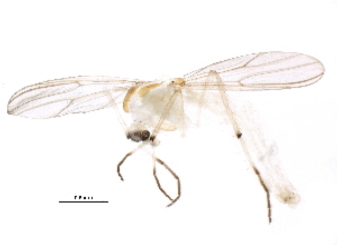

BIOUG22462-D03 [Lateral]
Chironomidae
Family: Chironomidae
BIN URI: BOLD:ACV2188

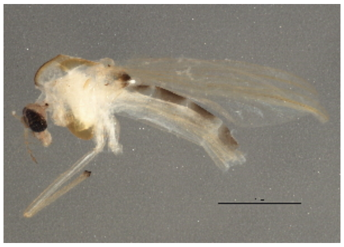

BIOUG02622-F07 [Lateral]
Chironomidae
Family: Chironomidae
BIN URI: BOLD:AAM6230

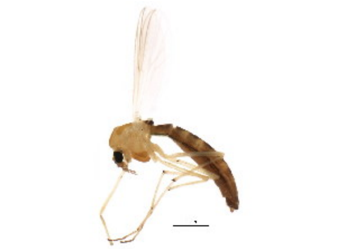

BIOUG01411-H05 [Lateral]
Chironomidae
Family: Chironomidae
BIN URI: BOLD:AAZ0144

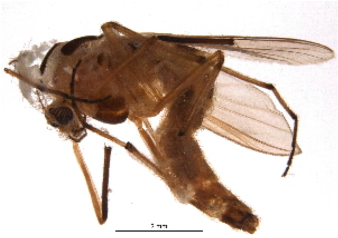

BIOUG10470-A06 [Lateral]
Chironomus
Family: Chironomidae
BIN URI: BOLD:ACL4512

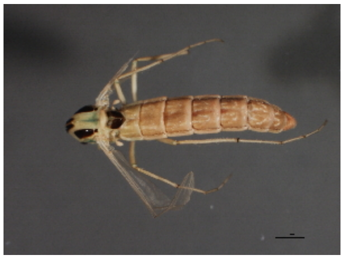

08BBDIP-1925 [Dorsal]
Chironomus
Family: Chironomidae
BIN URI: BOLD:AAG5478

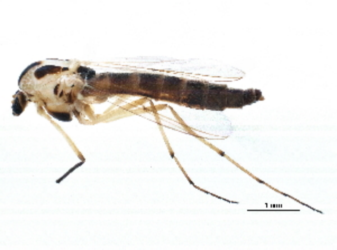

BIOUG21931-C06 [Lateral]
Chironomus nr. atroviridis 2i IP2013
Family: Chironomidae
BIN URI: BOLD:ACQ6990

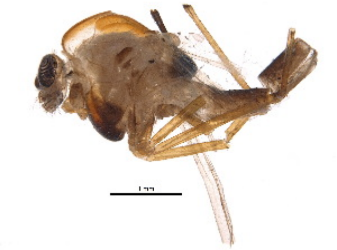

BIOUG20569-F11 [Lateral]
Chironomus ochreatus
Family: Chironomidae
BIN URI: BOLD:ACV5571

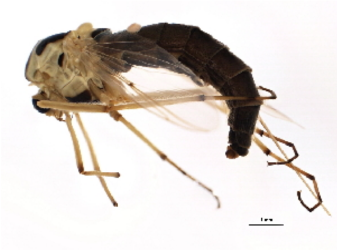

08BBDIP-2632 [Lateral]
Chironomus dilutus
Family: Chironomidae
BIN URI: BOLD:AAB4658

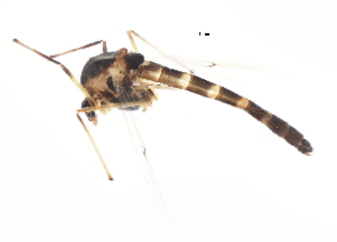

10JSROW-1494 [Lateral]
Chironomus melanescens
Family: Chironomidae
BIN URI: BOLD:AAI4303

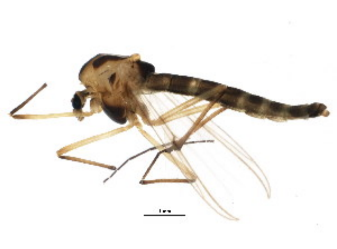

BIOUG01338-F02 [Lateral]
Chironomus acidophilus
Family: Chironomidae
BIN URI: BOLD:AAJ4295

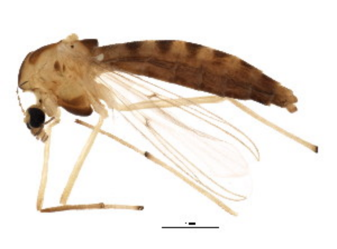

BIOUG01645-A11 [Lateral]
Chironomus
Family: Chironomidae
BIN URI: BOLD:ABV1236

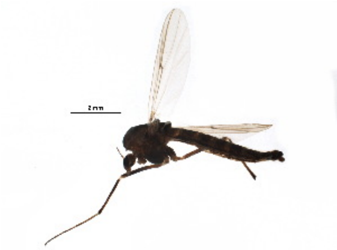

BIOUG22289-E12 [Lateral]
Chironomidae
Family: Chironomidae
BIN URI: BOLD:ACV5328

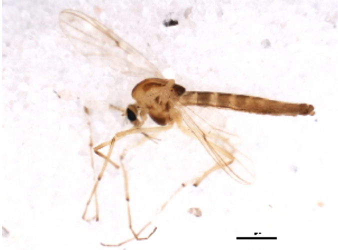

08TTML-0864 [Lateral]
Chironomus maturus
Family: Chironomidae
BIN URI: BOLD:AAB4657

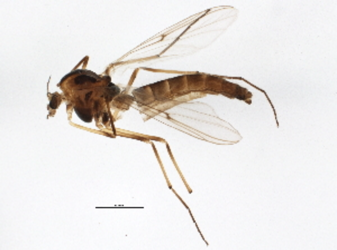

BIOUG05668-B11 [Lateral]
Chironomidae
Family: Chironomidae
BIN URI: BOLD:AAG5455

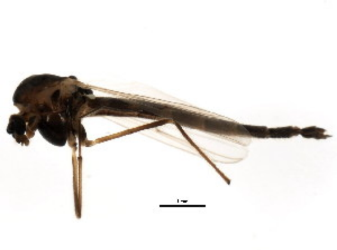

BIOUG01344-B07 [Lateral]
Chironomidae
Family: Chironomidae
BIN URI: BOLD:AAM6288

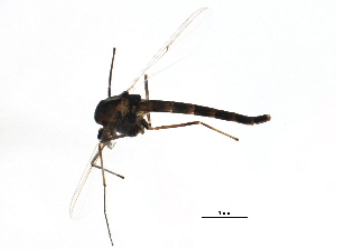

BIOUG21589-D10 [Lateral]
Chironomus
Family: Chironomidae
BIN URI: BOLD:ACA9275

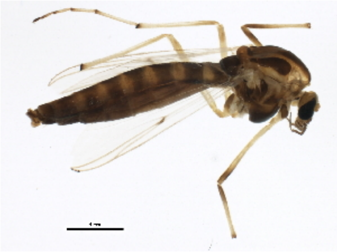

BIOUG00893-H06 [Lateral]
Chironomus
Family: Chironomidae
BIN URI: BOLD:ABV1458

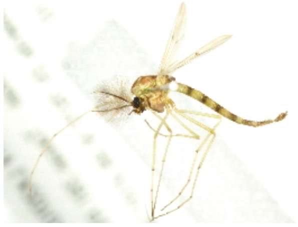

10BBDIP-0007 [Lateral]
Chironomidae
Family: Chironomidae
BIN URI: BOLD:AAB7030

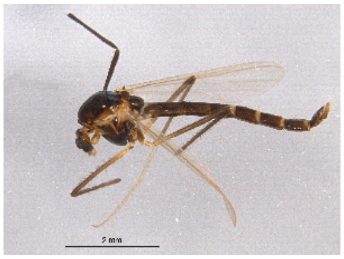

BIOUG02752-B02 [Lateral]
Chironominae
Family: Chironomidae
BIN URI: BOLD:AAN5311

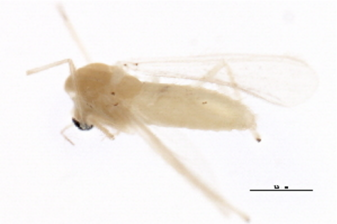

08TTML-0774 [Lateral]
Chironomidae
Family: Chironomidae
BIN URI: BOLD:AAN5329

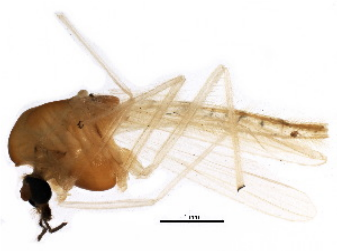

BIOUG22324-G07 [Lateral]
Chironomidae
Family: Chironomidae
BIN URI: BOLD:AAG5457

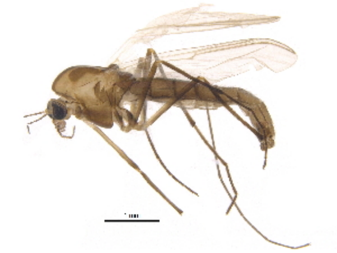

BIOUG22330-D04 [Lateral]
Chironomidae
Family: Chironomidae
BIN URI: BOLD:ACV3991

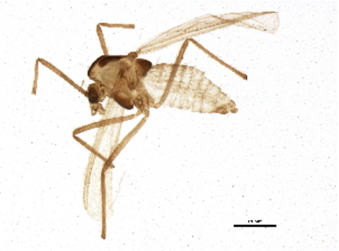

BIOUG01648-B04 [Lateral]
Tanytarsus
Family: Chironomidae
BIN URI: BOLD:ABZ6565

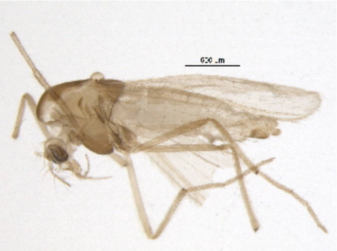

BIOUG05623-D01 [Lateral]
Chironomidae
Family: Chironomidae
BIN URI: BOLD:ACF8829

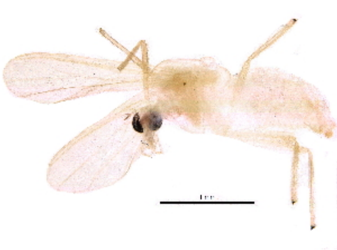

BIOUG22329-A07 [Lateral]
Tanytarsus
Family: Chironomidae
BIN URI: BOLD:AAG5523

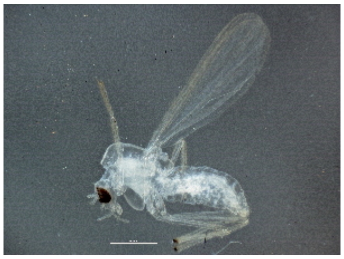

BIOUG05796-F07 [Lateral]
Chironomidae
Family: Chironomidae
BIN URI: BOLD:ACA4863

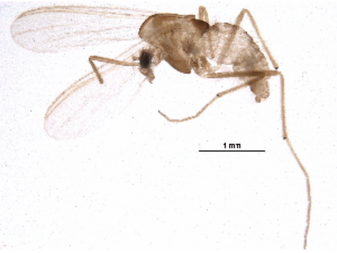

BIOUG09675-A06 [Lateral]
Chironominae
Family: Chironomidae
BIN URI: BOLD:ACL8053

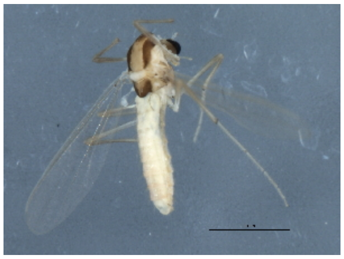

10JSROW-0174 [Dorsal]
Chironomidae
Family: Chironomidae
BIN URI: BOLD:AAP6875

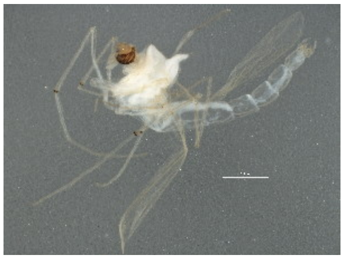

BIOUG01622-H09 [Lateral]
Chironomidae
Family: Chironomidae
BIN URI: BOLD:ABV1284

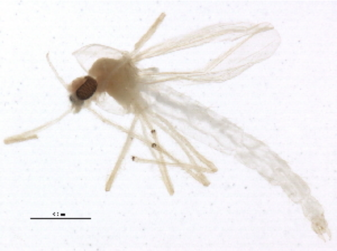

BIOUG00898-C02 [Lateral]
Chironominae
Family: Chironomidae
BIN URI: BOLD:ABV1396

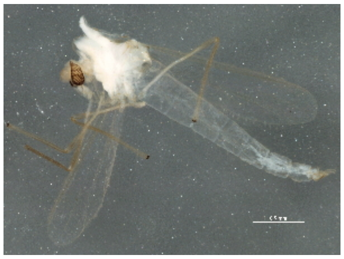

BIOUG01676-H03 [Lateral]
Chironominae
Family: Chironomidae
BIN URI: BOLD:ABV1221

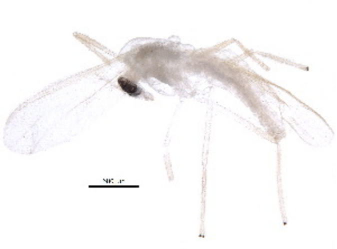

BIOUG22356-D12 [Lateral]
Rheotanytarsus
Family: Chironomidae
BIN URI: BOLD:ABA6514

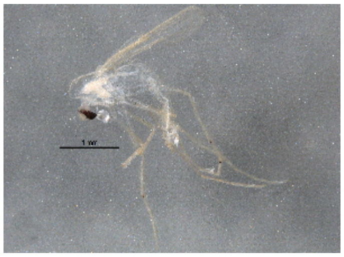

BIOUG08481-B05 [Lateral]
Rheotanytarsus
Family: Chironomidae
BIN URI: BOLD:ACJ8793

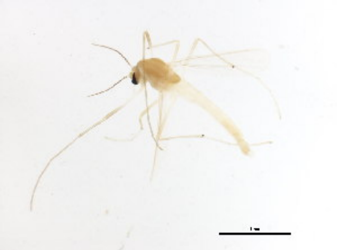

BIOUG01410-A08 [Lateral]
Chironomidae
Family: Chironomidae
BIN URI: BOLD:AAV6213

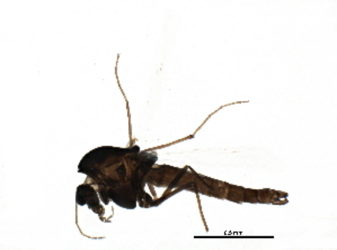

BIOUG22327-H09 [Lateral]
Rheotanytarsus pellucidus
Family: Chironomidae
BIN URI: BOLD:AAI0332

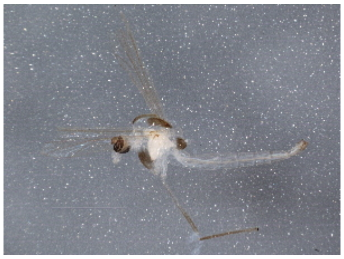

BIOUG08495-D05 [Lateral]
Chironomidae
Family: Chironomidae
BIN URI: BOLD:ACJ6550

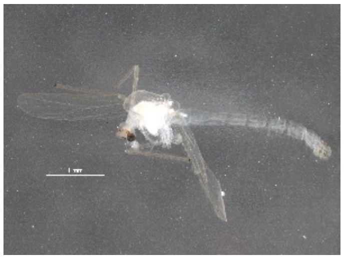

BIOUG22418-H07 [Lateral]
Tanytarsus
Family: Chironomidae
BIN URI: BOLD:ACR8658

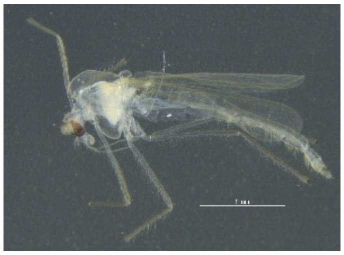

BIOUG03244-G01 [Lateral]
Tanytarsus guerlus
Family: Chironomidae
BIN URI: BOLD:AAC4525

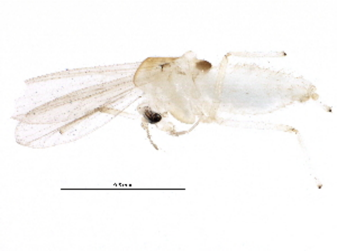

BIOUG22366-E10 [Lateral]
Stempellinella fimbriata
Family: Chironomidae
BIN URI: BOLD:AAD0300

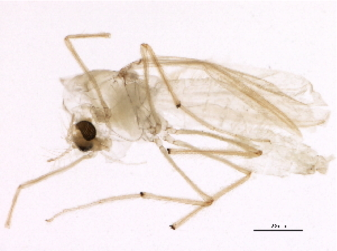

BIOUG01656-G11 [Lateral]
Chironomidae
Family: Chironomidae
BIN URI: BOLD:AAN5391

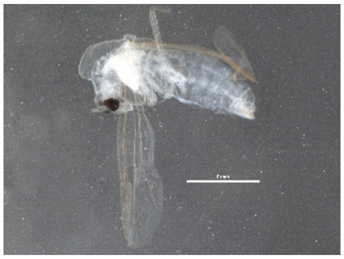

BIOUG20384-D12 [Lateral]
Diptera

BIN URI: BOLD:ABA7852

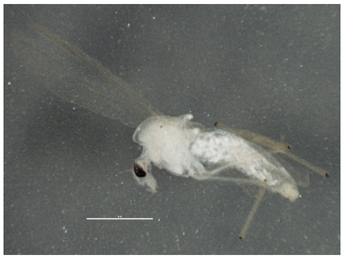

BIOUG03167-E10 [Lateral]
Tanytarsus
Family: Chironomidae
BIN URI: BOLD:ACC1283

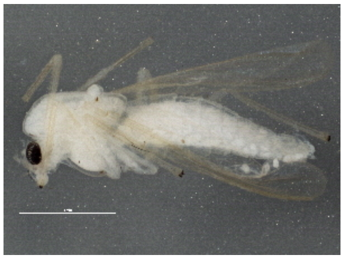

BIOUG03110-A06 [Lateral]
Chironomidae
Family: Chironomidae
BIN URI: BOLD:ACC1609

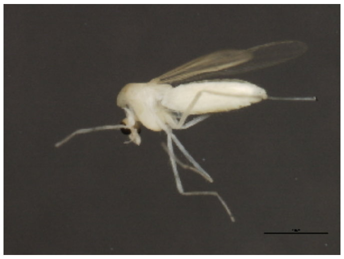

08BBDIP-1863 [Lateral]
Chironomidae
Family: Chironomidae
BIN URI: BOLD:AAG5463

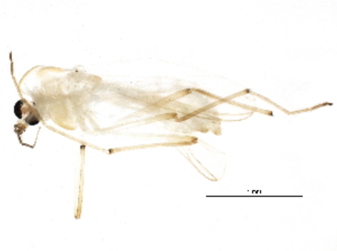

BIOUG22362-B05 [Lateral]
Tanytarsus
Family: Chironomidae
BIN URI: BOLD:ACG9026

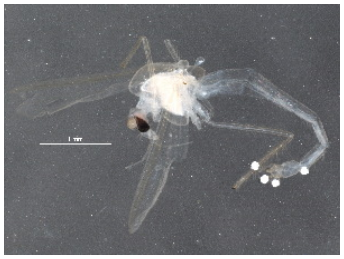

BIOUG22417-F07 [Lateral]
Tanytarsus
Family: Chironomidae
BIN URI: BOLD:ACI3514

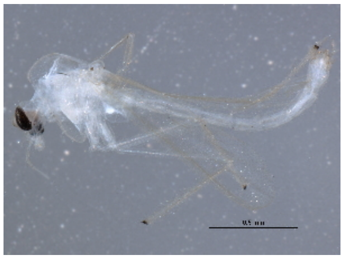

BIOUG22084-E04 [Lateral]
Tanytarsus
Family: Chironomidae
BIN URI: BOLD:ACV4276

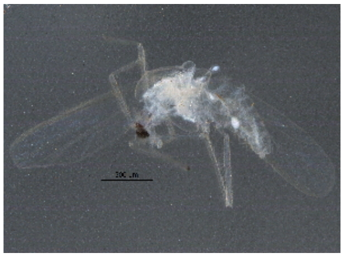

BIOUG11280-D03 [Lateral]
Tanytarsus
Family: Chironomidae
BIN URI: BOLD:ACM0828

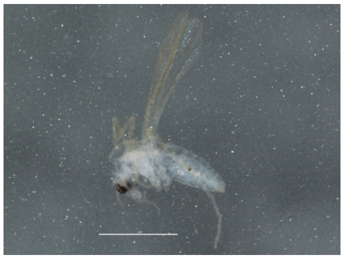

BIOUG03341-G02 [Lateral]
Chironomidae
Family: Chironomidae
BIN URI: BOLD:AAP7035

IMAGE NOT AVAILABLE

BIOUG21779-H02
Tanytarsus
Family: Chironomidae

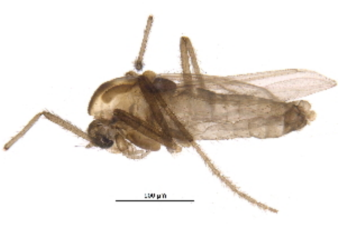

BIOUG22329-E09 [Lateral]
Stempellinella
Family: Chironomidae
BIN URI: BOLD:AAN5345

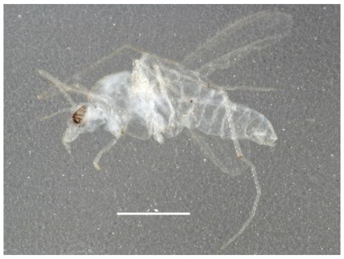

BIOUG01612-A03 [Lateral]
Chironomidae
Family: Chironomidae
BIN URI: BOLD:ABA6490

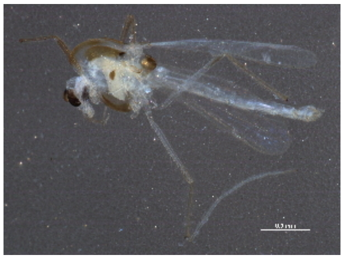

BIOUG10916-F06 [Lateral]
Cladotanytarsus
Family: Chironomidae
BIN URI: BOLD:ACM0192

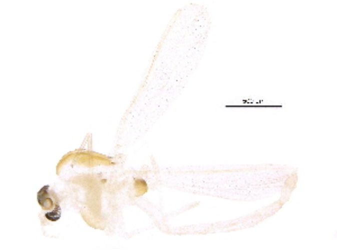

BIOUG22323-C03 [Lateral]
Tanytarsus
Family: Chironomidae
BIN URI: BOLD:ACA4847

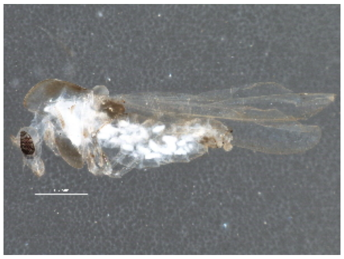

BIOUG11186-H05 [Lateral]
Tanytarsus recurvatus
Family: Chironomidae
BIN URI: BOLD:AAC3354

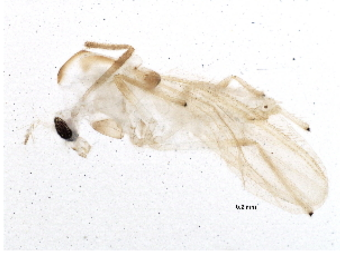

BIOUG09541-G08 [Lateral]
Tanytarsus
Family: Chironomidae
BIN URI: BOLD:AAD8854

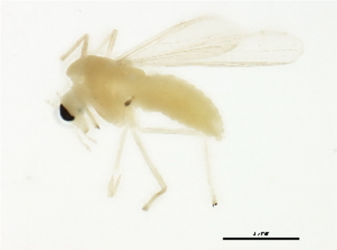

BIOUG01410-E01 [Lateral]
Chironomidae
Family: Chironomidae
BIN URI: BOLD:ACM2385

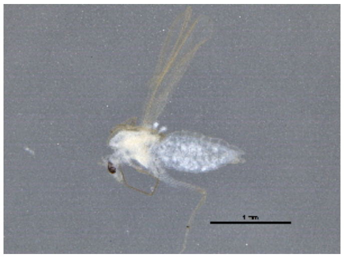

BIOUG22330-G12 [Lateral]
Chironomidae
Family: Chironomidae
BIN URI: BOLD:AAL7356

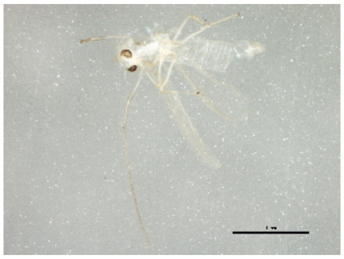

BIOUG01571-E07 [Lateral]
Chironomidae
Family: Chironomidae
BIN URI: BOLD:ACU2945

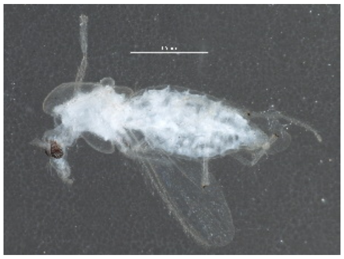

BIOUG05795-F02 [Lateral]
Chironomidae
Family: Chironomidae
BIN URI: BOLD:ACU2946

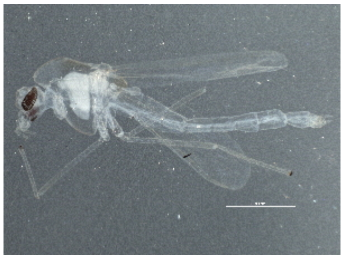

BIOUG08375-B05 [Lateral]
Tanytarsus
Family: Chironomidae
BIN URI: BOLD:AAP5870

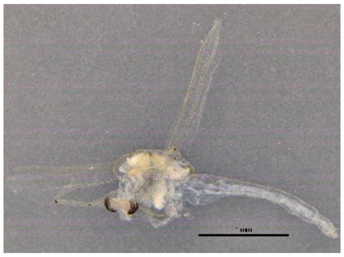

BIOUG22235-B04 [Lateral]
Tanytarsus
Family: Chironomidae
BIN URI: BOLD:ACV4333

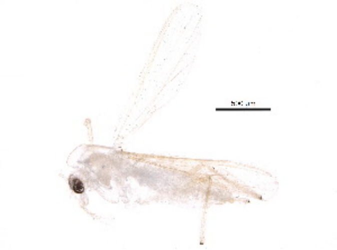

BIOUG22922-D08 [Lateral]
Tanytarsus
Family: Chironomidae
BIN URI: BOLD:ACV4943

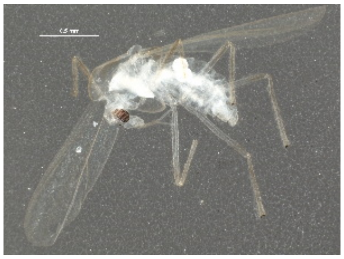

BIOUG22235-B08 [Lateral]
Tanytarsus glabrescens
Family: Chironomidae
BIN URI: BOLD:AAD8855

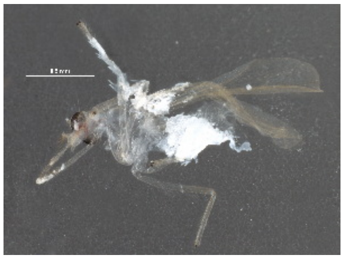

BIOUG22730-C05 [Lateral]
Tanytarsus glabrescens
Family: Chironomidae
BIN URI: BOLD:AAD8855

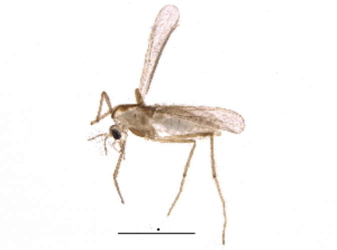

BIOUG10178-A01 [Lateral]
Chironominae
Family: Chironomidae
BIN URI: BOLD:ACL4257

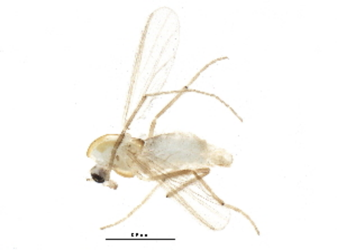

BIOUG22366-D05 [Lateral]
Micropsectra
Family: Chironomidae
BIN URI: BOLD:ACV4826

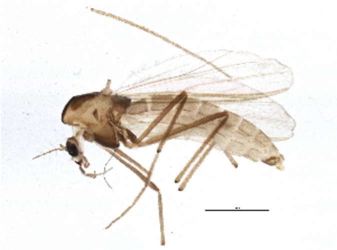

BIOUG05830-E12 [Lateral]
Micropsectra subletteorum
Family: Chironomidae
BIN URI: BOLD:AAF7088

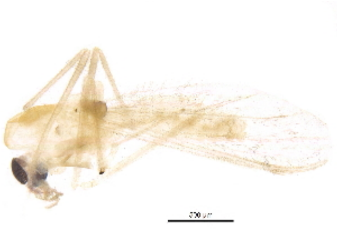

BIOUG21483-C05 [Lateral]
Chironomidae
Family: Chironomidae
BIN URI: BOLD:ACA2939

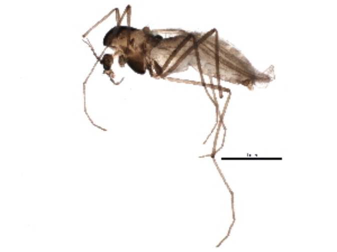

BIOUG05616-E12 [Lateral]
Micropsectra
Family: Chironomidae
BIN URI: BOLD:AAN5328

IMAGE NOT AVAILABLE

BIOUG21614-G04
Micropsectra nigripila
Family: Chironomidae

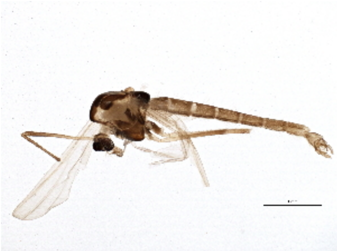

BIOUG02819-C08 [Lateral]
Chironomidae
Family: Chironomidae
BIN URI: BOLD:AAQ0602

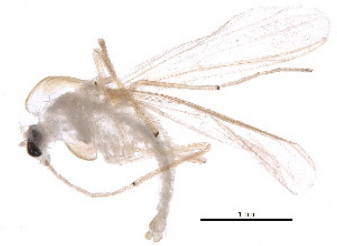

BIOUG22356-A07 [Lateral]
Tanytarsus
Family: Chironomidae
BIN URI: BOLD:AAG5467

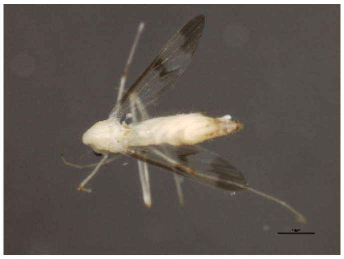

08BBDIP-1869 [Lateral]
Chironomidae
Family: Chironomidae
BIN URI: BOLD:AAG5466

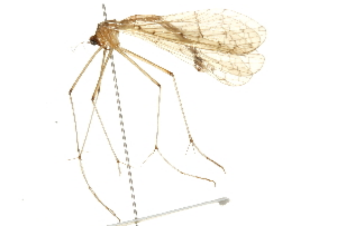

09BBMEC-0010 [Lateral]
Bittacus strigosus
Family: Bittacidae
BIN URI: BOLD:AAH3560

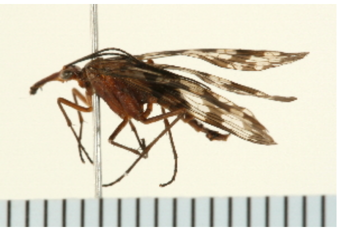

08BBMEC-005 [Lateral]
Panorpa subfurcata
Family: Panorpidae
BIN URI: BOLD:ABY6178

IMAGE NOT AVAILABLE

BIOUG24029-C11
Panorpidae
Family: Panorpidae

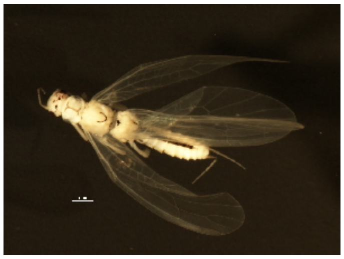

08BKEPT-194 [Dorsal]
Sweltsa onkos
Family: Chloroperlidae
BIN URI: BOLD:AAB4130

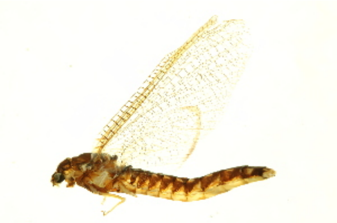

10BBEPT-0265 [Lateral]
Hexagenia limbata
Family: Ephemeridae
BIN URI: BOLD:AAA5477

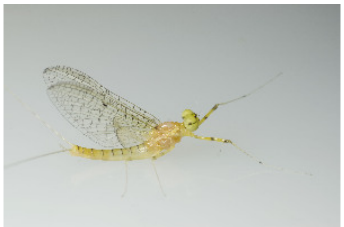

SWRC-Si_WRI9_01 [Lateral]
Stenacron interpunctatum
Family: Heptageniidae
BIN URI: BOLD:AAA8213

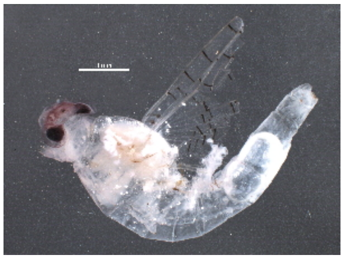

BIOUG24021-G02 [Lateral]
Maccaffertium
Family: Heptageniidae

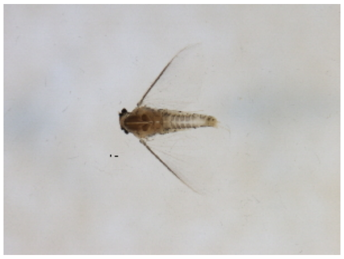

07ELEPT-388 [Dorsal]
Caenis latipennis
Family: Caenidae
BIN URI: BOLD:AAI3819

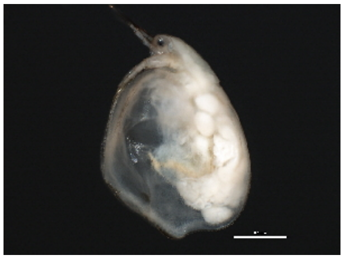

NJGS-19 [Lateral]
Simocephalus cf. punctatus sp.1 NA
Family: Daphniidae
BIN URI: BOLD:AAH3195

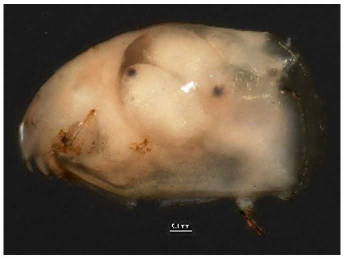

11AlgonqNJ0117 [Lateral]
Eurycercus longirostris
Family: Eurycercidae
BIN URI: BOLD:AAC8115

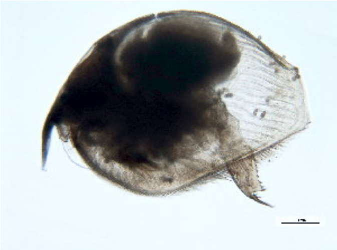

10BBPLANK-0155 [Lateral]
Chydoridae
Family: Chydoridae
BIN URI: BOLD:AAC9302

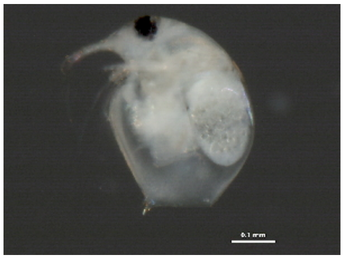

BIOUG02838-F03 [Lateral]
Branchiopoda

BIN URI: BOLD:ACA6834

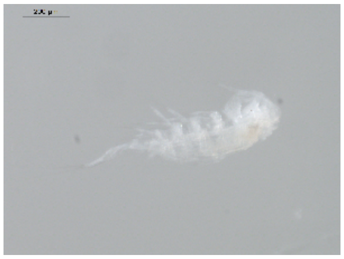

BIOUG01746-G07 [Lateral]
Maxillopoda

BIN URI: BOLD:ACN5861

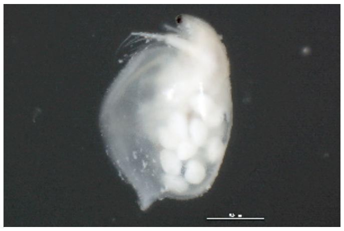

BIOUG02838-A04 [Lateral]
Branchiopoda

BIN URI: BOLD:ACF0852

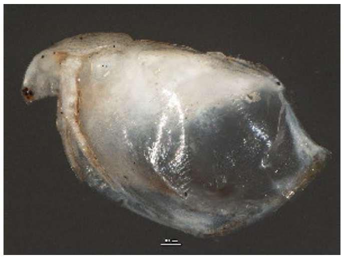

NJAK-0083 [Lateral]
Simocephalus cf. serrulatus sp. 1NA
Family: Daphniidae
BIN URI: BOLD:ACF0854

IMAGE NOT AVAILABLE

BIOUG24026-A05
Daphnia
Family: Daphniidae

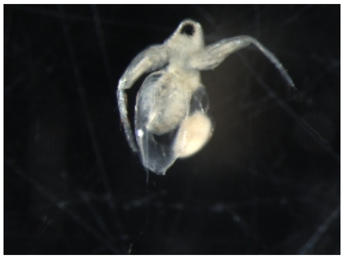

SJA-0245 [Lateral]
Diaphanosoma sp. 2
Family: Sididae
BIN URI: BOLD:AAI6310

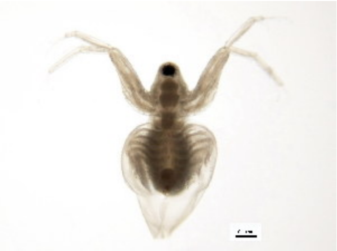

10BBCRU-0091 [Ventral]
Diaphanosoma
Family: Sididae
BIN URI: BOLD:AAI6310

IMAGE NOT AVAILABLE

BIOUG24026-B12
Diaphanosoma
Family: Sididae

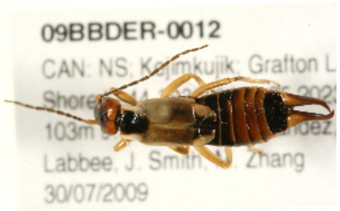

09BBDER-0012 [Dorsal]
Forficula auricularia-A
Family: Forficulidae
BIN URI: BOLD:AAG9897

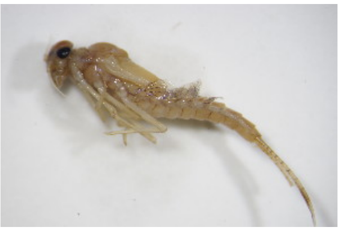

BCZSM_EPH_0133 [Lateral]
Cloeon dipterum
Family: Baetidae
BIN URI: BOLD:AAM7076

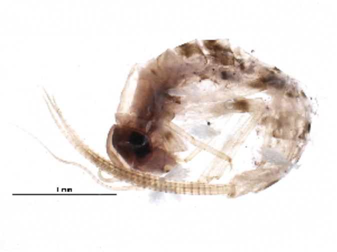

BIOUG21000-F06 [Lateral]
Ephemeroptera

BIN URI: BOLD:AAC7439

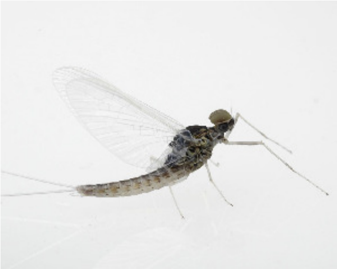

SWRC-Cf_M1_02 [Lateral]
Callibaetis fluctuans
Family: Baetidae
BIN URI: BOLD:AAD5425

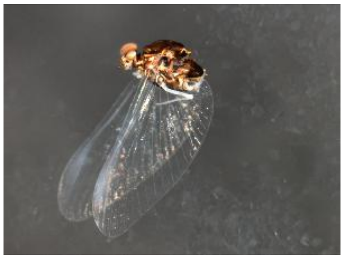

07PROBE-2257 [Lateral]
Acerpenna sp.JMW1
Family: Baetidae
BIN URI: BOLD:AAC3979

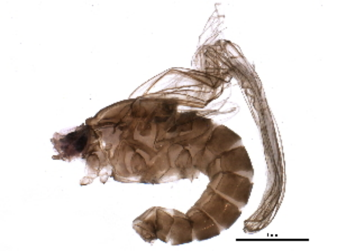

BIOUG16068-C08 [Lateral]
Baetis intercalaris
Family: Baetidae
BIN URI: BOLD:AAM2026

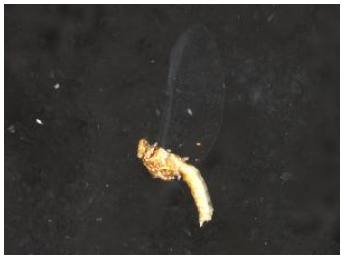

07PROBE-07104 [Lateral]
Iswaeon anoka
Family: Baetidae
BIN URI: BOLD:AAA9021

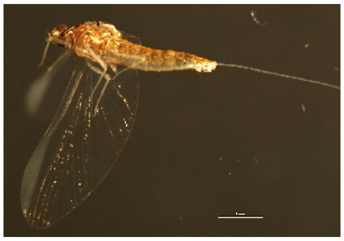

09ELEPT-019 [Lateral]
Iswaeon anoka
Family: Baetidae
BIN URI: BOLD:ACE7903

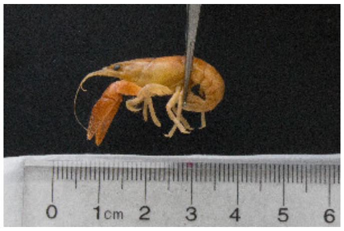

kmMR1 [Lateral full body]
Orconectes propinquus
Family: Cambaridae
BIN URI: BOLD:AAE4928

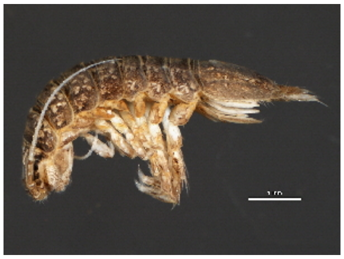

NJGS-8 [Lateral]
Caecidotea sp.
Family: Asellidae
BIN URI: BOLD:AAC7342

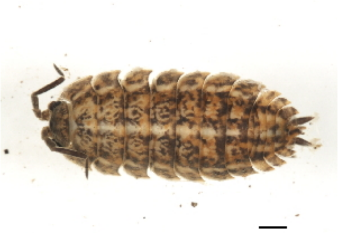

09ISOP-0001 [Dorsal]
Isopoda

BIN URI: BOLD:AAH4100

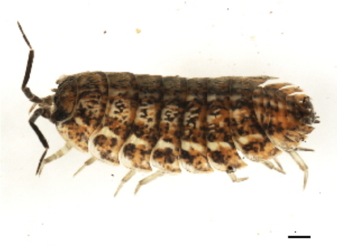

09BBIUS-0006 [Dorsal]
Isopoda

BIN URI: BOLD:AAH4102

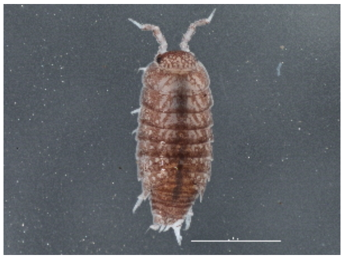

BIOUG08059-B08 [Dorsal]
Trichoniscus pusillus
Family: Trichoniscidae
BIN URI: BOLD:AAN7523

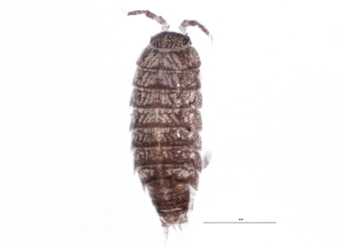

BIOUG08049-D12 [Dorsal]
Hyloniscus riparius
Family: Trichoniscidae
BIN URI: BOLD:AAV6495

IMAGE NOT AVAILABLE

BIOUG24026-G05
Maxillopoda

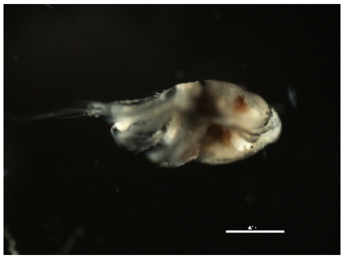

08BBCRU-053 [Lateral]
Cyclopoida

BIN URI: BOLD:AAG9779

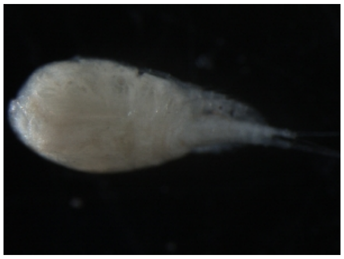

SJA-0049 [Lateral]
Cyclopoida

BIN URI: BOLD:AAG9780

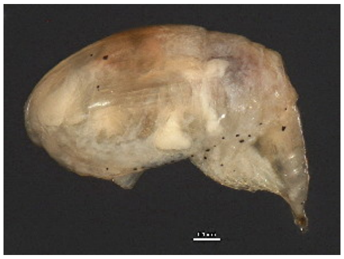

NJAK-0025 [Lateral]
Cyclopoida

BIN URI: BOLD:AAG9778

IMAGE NOT AVAILABLE

BIOUG24026-G12
Maxillopoda

IMAGE NOT AVAILABLE

BIOUG24026-D03
Cyclopidae
Family: Cyclopidae

IMAGE NOT AVAILABLE

BIOUG24026-E09
Maxillopoda

IMAGE NOT AVAILABLE

BIOUG24026-F07
Maxillopoda

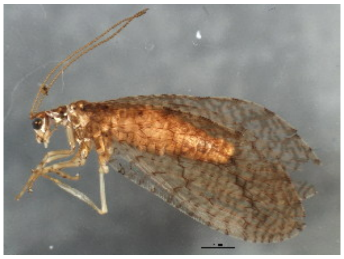

BIOUG00864-E07 [Lateral]
Micromus posticus
Family: Hemerobiidae
BIN URI: BOLD:AAG0906

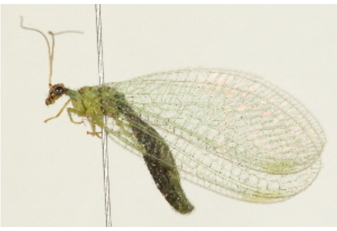

09BBNEU-0093 [Lateral]
Chrysopa oculata
Family: Chrysopidae
BIN URI: BOLD:AAG2017

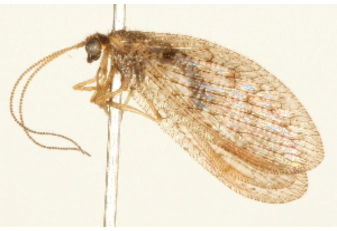

HLC-17470 [Lateral]
Hemerobius stigma
Family: Hemerobiidae
BIN URI: BOLD:AAG0891

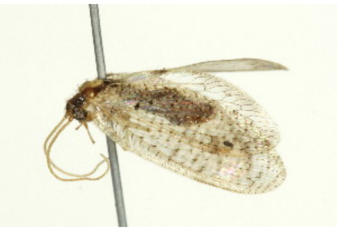

TDWG-0213 [Lateral]
Hemerobius humulinus
Family: Hemerobiidae
BIN URI: BOLD:AAG0892

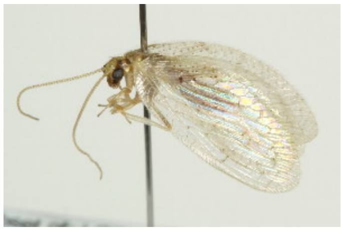

TDWG-0217 [Lateral]
Hemerobius humulinus
Family: Hemerobiidae
BIN URI: BOLD:AAN7492

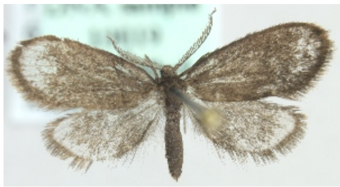

MM10019 [Dorsal]
Psyche casta
Family: Psychidae
BIN URI: BOLD:ACL8669

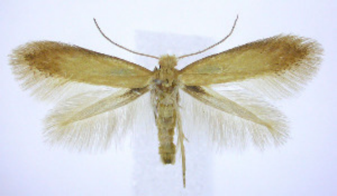

jflandry0213 [Dorsal]
Coptotriche citrinipennella
Family: Tischeriidae
BIN URI: BOLD:AAC7129

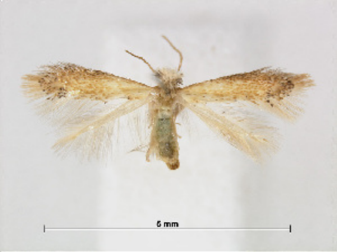

RMNH.INS.24711 [Dorsal]
Coptotriche badiiella
Family: Tischeriidae
BIN URI: BOLD:ACU4456

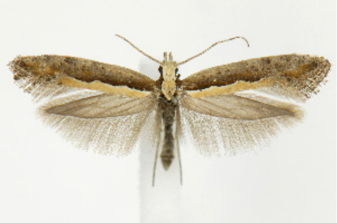

CNCLEP00040356 [Dorsal]
Plutella xylostella
Family: Plutellidae
BIN URI: BOLD:AAA1513

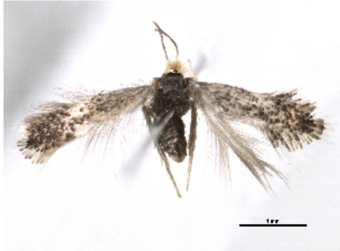

BIOUG16764-G09 [Dorsal]
Ectoedemia argyropeza
Family: Nepticulidae
BIN URI: BOLD:AAC1036

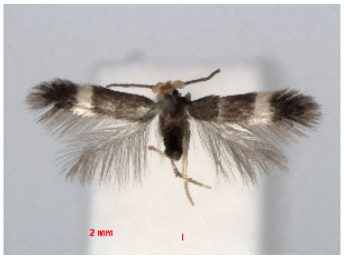

RMNH.INS.24430 [Dorsal]
Stigmella microtheriella
Family: Nepticulidae
BIN URI: BOLD:AAI0007

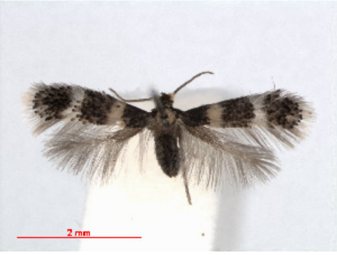

RMNH.INS.24410 [Dorsal]
Stigmella rhamnicola
Family: Nepticulidae
BIN URI: BOLD:AAU7678

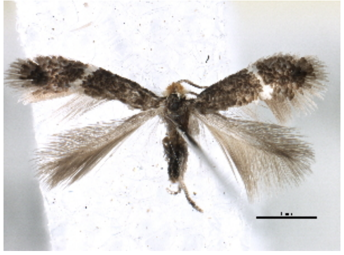

BIOUG16764-B12 [Dorsal]
Stigmella
Family: Nepticulidae
BIN URI: BOLD:AAI6169

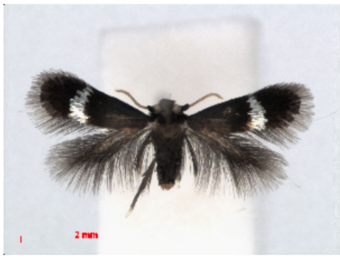

RMNH.INS.24407 [Dorsal]
Stigmella betula_usa
Family: Nepticulidae
BIN URI: BOLD:ACG9017

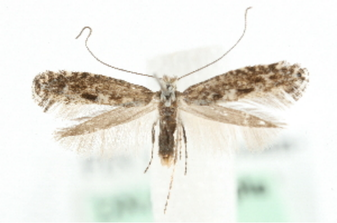

MM06299 [Dorsal]
Parornix betulae
Family: Gracillariidae
BIN URI: BOLD:AAE3418

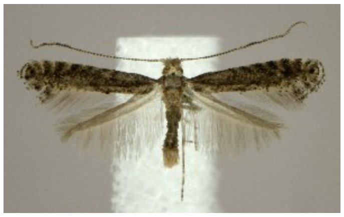

DNA-ATBI-3415 [Dorsal]
Parornix sp.
Family: Gracillariidae
BIN URI: BOLD:AAF8198

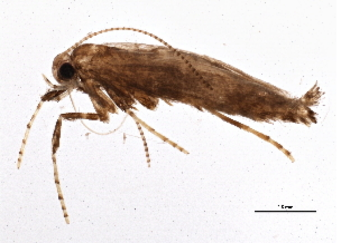

BIOUG02618-E11 [Lateral]
Parornix
Family: Gracillariidae
BIN URI: BOLD:AAG1144

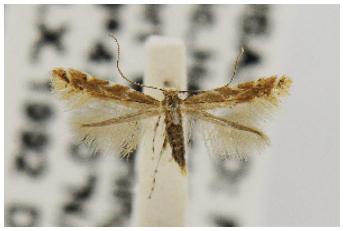

USNMENT00656251 [Dorsal]
Cremastobombycia solidaginis
Family: Gracillariidae
BIN URI: BOLD:AAC4262

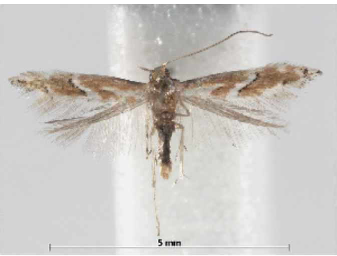

RMNH.INS.552275 [Dorsal]
Cameraria saccharella
Family: Gracillariidae
BIN URI: BOLD:AAH4493

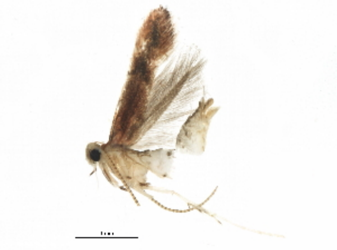

BIOUG01903-C12 [Lateral]
Cameraria
Family: Gracillariidae
BIN URI: BOLD:ABX0017

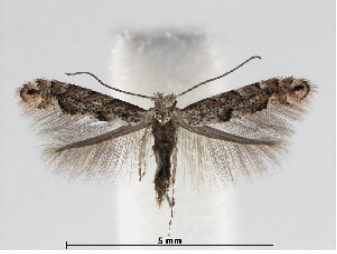

RMNH.INS.552266 [Dorsal]
Phyllonorycter salicifoliella
Family: Gracillariidae
BIN URI: BOLD:AAD4915

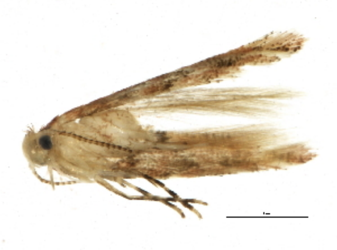

BIOUG01573-D04 [Lateral]
Phyllonorycter
Family: Gracillariidae
BIN URI: BOLD:AAI5995

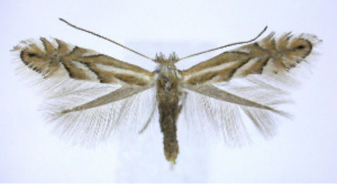

jflandry0188 [Dorsal]
Phyllonorycter ostryaefoliella
Family: Gracillariidae
BIN URI: BOLD:AAD7999

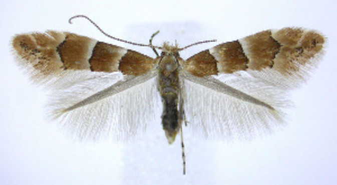

jflandry0201 [Dorsal]
Phyllonorycter tritaenianella
Family: Gracillariidae
BIN URI: BOLD:AAF6577

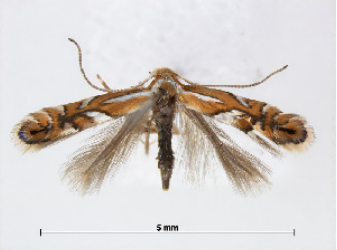

RMNH.INS.544278 [Dorsal]
Phyllonorycter propinquinella
Family: Gracillariidae
BIN URI: BOLD:AAH4497

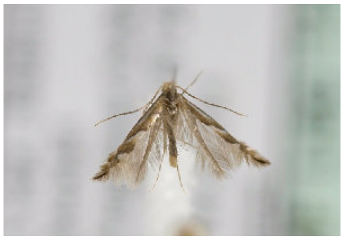

TLMF Lep 15507 [Dorsal]
Phyllonorycter maestingella
Family: Gracillariidae
BIN URI: BOLD:AAL6962

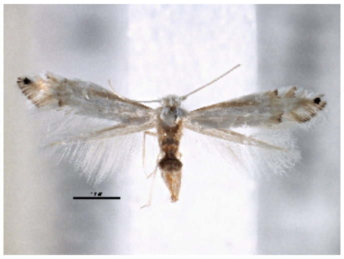

CNCLEP00101453 [Dorsal]
Phyllonorycter trinotella
Family: Gracillariidae
BIN URI: BOLD:AAG1128

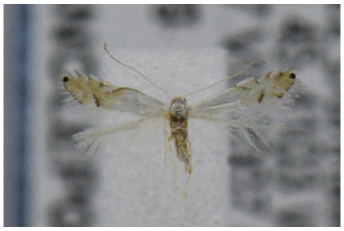

CNCLEP00038402 [Dorsal]
Phyllonorycter clemensella
Family: Gracillariidae
BIN URI: BOLD:AAN8981

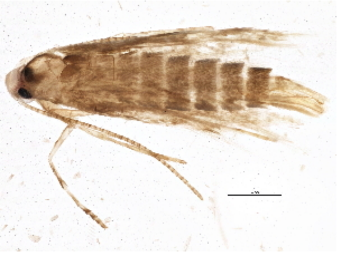

BIOUG06046-F06 [Lateral]
Phyllonorycter
Family: Gracillariidae
BIN URI: BOLD:ACB9323

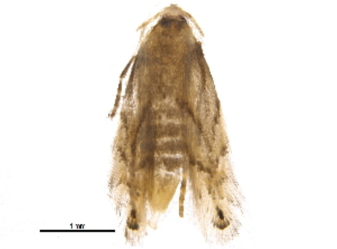

BIOUG22296-A03 [Dorsal]
Phyllonorycter
Family: Gracillariidae
BIN URI: BOLD:ACV4141

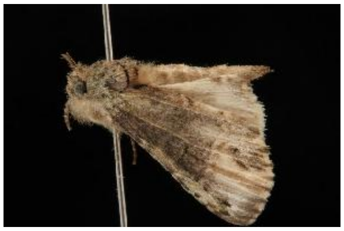

04HBL002088 [Lateral]
Schizura unicornis
Family: Notodontidae
BIN URI: BOLD:AAA3873

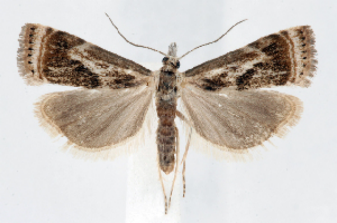

jflandry1182 [Dorsal]
Microcrambus elegans
Family: Crambidae
BIN URI: BOLD:AAA4893

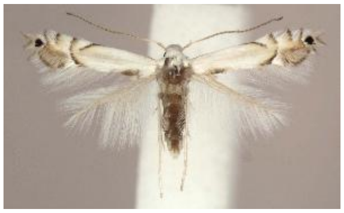

CNCLEP00027712 [Dorsal]
Phyllocnistis vitigenella
Family: Gracillariidae
BIN URI: BOLD:AAI3014

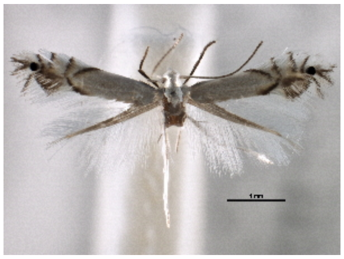

CNCLEP00101458 [Dorsal]
Phyllocnistis ampelopsiella
Family: Gracillariidae
BIN URI: BOLD:AAI3015

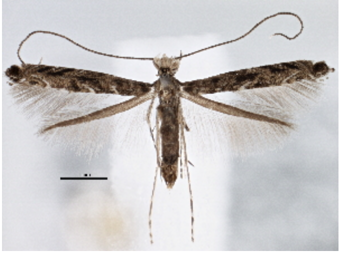

CNCLEP00006758 [Dorsal]
Neurobathra strigifinitella
Family: Gracillariidae
BIN URI: BOLD:AAR7014

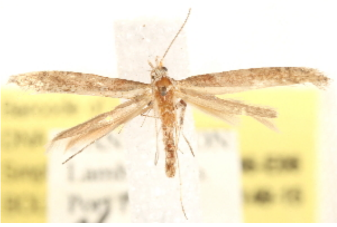

BIOUG20646-E06 [Dorsal]
Gracillariidae
Family: Gracillariidae
BIN URI: BOLD:AAE7347

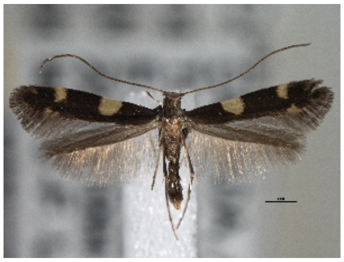

CNCLEP00101422 [Dorsal]
Caloptilia canadensisella
Family: Gracillariidae
BIN URI: BOLD:AAE7388

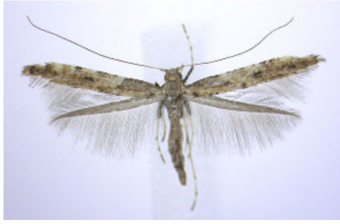

jflandry0207 [Dorsal]
Caloptilia ostryaeella
Family: Gracillariidae
BIN URI: BOLD:AAC7941

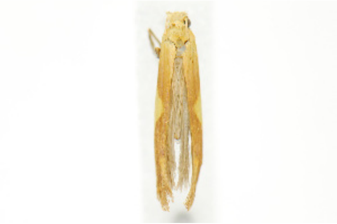

CNCLEP00035818 [Dorsal]
Caloptilia packardella
Family: Gracillariidae
BIN URI: BOLD:AAD2590

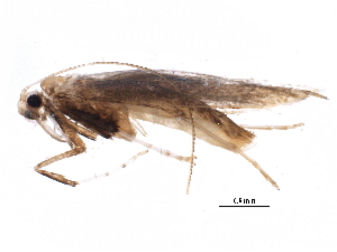

BIOUG22575-A03 [Lateral]
Caloptilia
Family: Gracillariidae
BIN URI: BOLD:ABW2631

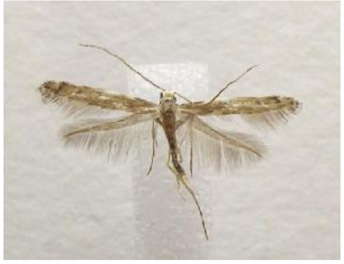

AC005127 [Dorsal]
Acrocercops astericola
Family: Gracillariidae
BIN URI: BOLD:AAD3996

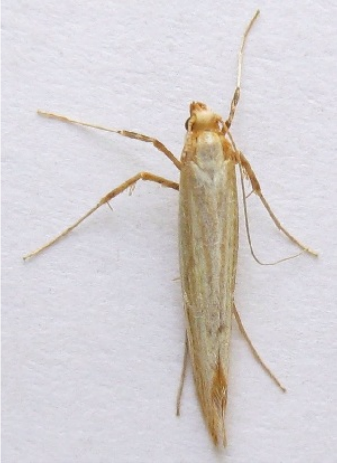

BIOUG06714-C01 [Dorsal]
Caloptilia murtfeldtella
Family: Gracillariidae
BIN URI: BOLD:AAE7367

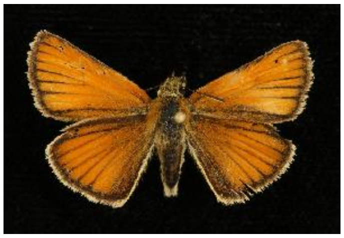

2006-ONT-1468 [Dorsal]
Thymelicus lineola
Family: Hesperiidae
BIN URI: BOLD:AAA6759

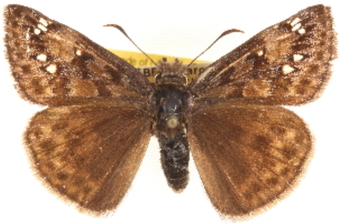

BIOUG10890-A12 [Dorsal]
Erynnis juvenalis
Family: Hesperiidae
BIN URI: BOLD:AAC6872

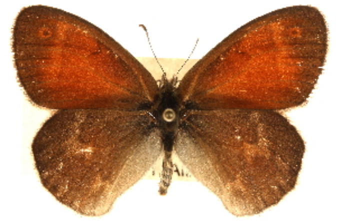

BIOUG10887-H10 [Dorsal]
Coenonympha tullia
Family: Nymphalidae
BIN URI: BOLD:AAA3561

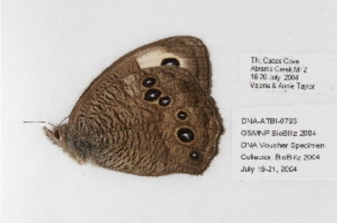

DNA-ATBI-0793 [Lateral]
Cercyonis pegala
Family: Nymphalidae
BIN URI: BOLD:AAA7277

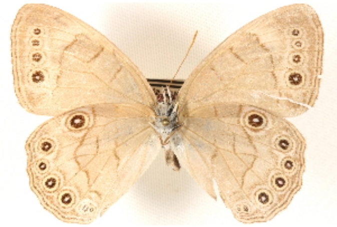

BIOUG15631-E10 [Ventral]
Lethe appalachia
Family: Nymphalidae
BIN URI: BOLD:AAB4176

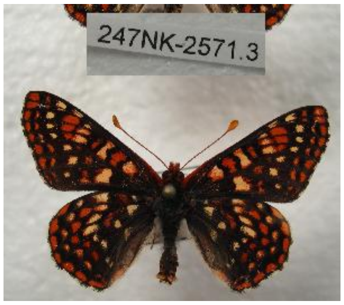

247NK-2571.3 [Dorsal]
Euphydryas anicia
Family: Nymphalidae
BIN URI: BOLD:AAA3686

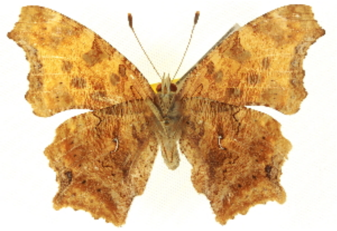

CCDB-24273-H02 [Ventral]
Polygonia comma
Family: Nymphalidae
BIN URI: BOLD:ABX6422

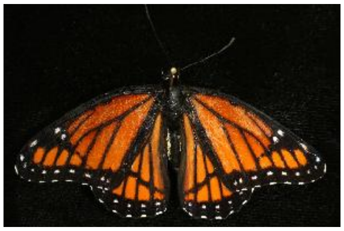

2006-ONT-1119 [Dorsal]
Limenitis archippus
Family: Nymphalidae
BIN URI: BOLD:ABZ6035

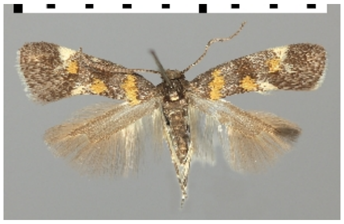

TLMF Lep 07030 [Dorsal]
Chrysoesthia sexguttella
Family: Gelechiidae
BIN URI: BOLD:AAD8505

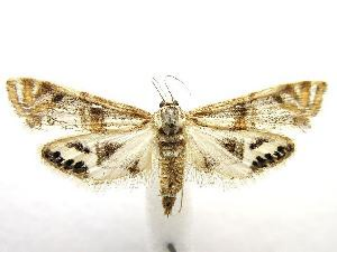

MDH002546 [Dorsal]
Petrophila bifascialis
Family: Crambidae
BIN URI: BOLD:AAC6453

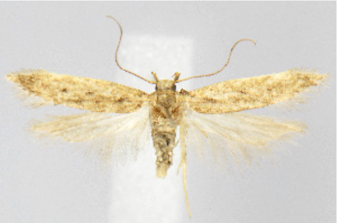

CNCLEP00040449 [Dorsal]
Bryotropha
Family: Gelechiidae
BIN URI: BOLD:AAH4276

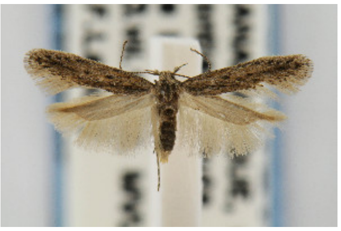

CNCLEP00067530 [Dorsal]
Scrobipalpula sacculicola
Family: Gelechiidae
BIN URI: BOLD:ABY8834

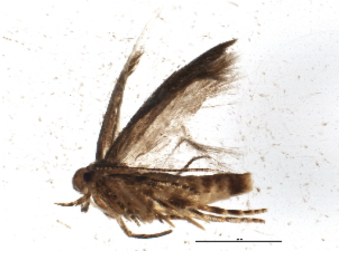

BIOUG03514-C03 [Lateral]
Scrobipalpula physaliella
Family: Gelechiidae
BIN URI: BOLD:ACB8750

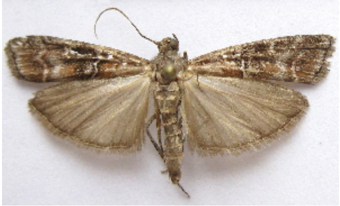

BIOUG00848-E08 [Dorsal]
Dioryctria banksiella
Family: Pyralidae
BIN URI: BOLD:AAB4478

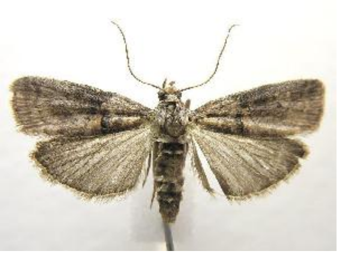

MDH000333 [Dorsal]
Acrobasis indigenella
Family: Pyralidae
BIN URI: BOLD:AAB5713

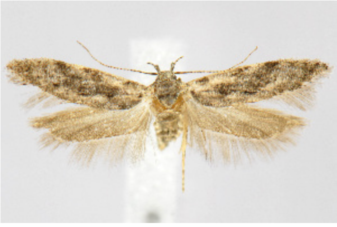

CNCLEP00040953 [Dorsal]
Xenolechia ontariensis
Family: Gelechiidae
BIN URI: BOLD:AAC6357

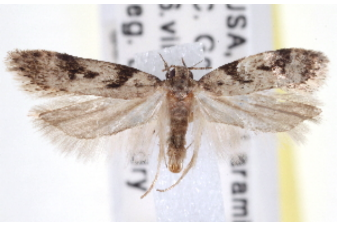

USNM ENT 00018022 [Dorsal]
Chionodes fondella
Family: Gelechiidae
BIN URI: BOLD:ABA4737

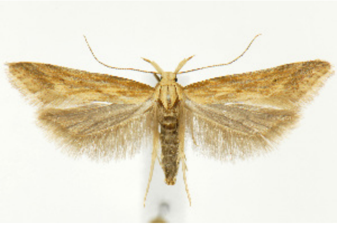

CNCLEP00040963 [Dorsal]
Metzneria lappella
Family: Gelechiidae
BIN URI: BOLD:AAB4321

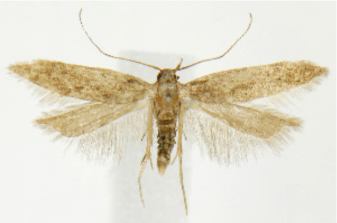

CNCLEP00040282 [Dorsal]
Monochroa
Family: Gelechiidae
BIN URI: BOLD:AAG0014

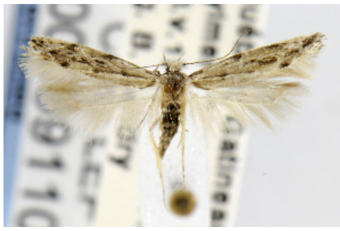

CNCLEP00099110 [Dorsal]
Sinoe chambersi
Family: Gelechiidae
BIN URI: BOLD:ACF2217

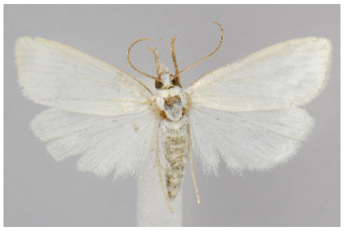

CNCLEP00040373 [Dorsal]
Urola nivalis
Family: Crambidae
BIN URI: BOLD:AAA8678

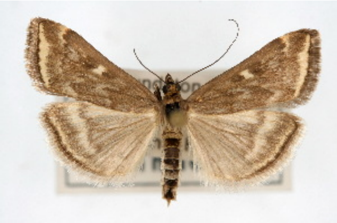

CNCLEP00074393 [Dorsal]
Loxostege sticticalis
Family: Crambidae
BIN URI: BOLD:AAB4167

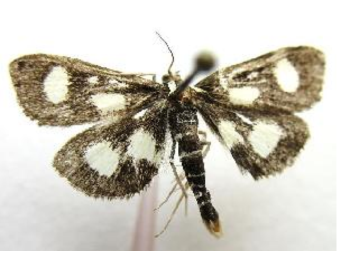

MDH002886 [Dorsal]
Anania funebris
Family: Crambidae
BIN URI: BOLD:AAB4181

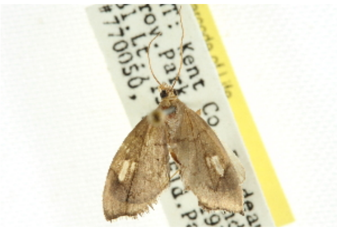

ROMPYR00001 [Dorsal]
Perispasta caeculalis
Family: Crambidae
BIN URI: BOLD:AAC0745

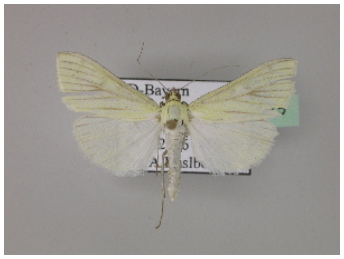

BC ZSM Lep 22975 [Dorsal]
Sitochroa palealis
Family: Crambidae
BIN URI: BOLD:AAD7889

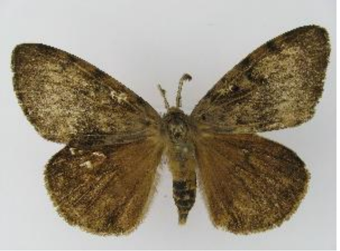

BC ZSM Lep 13352 [Dorsal]
Lymantriinae
Family: Erebidae
BIN URI: BOLD:AAA2052

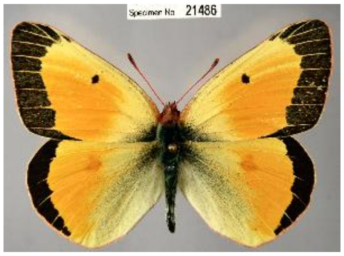

CSG21486 NE BC [Whole Specimen]
Colias christina
Family: Pieridae
BIN URI: BOLD:AAA3447

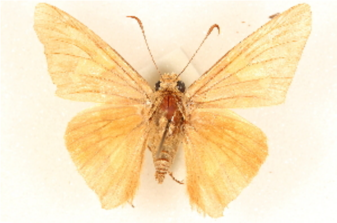

CCDB-24279-B09 [Dorsal]
Epargyreus clarus
Family: Hesperiidae
BIN URI: BOLD:ABY4698

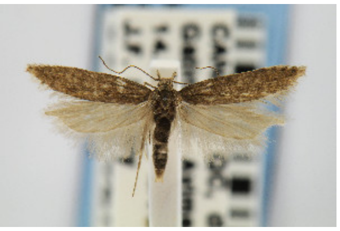

CNCLEP00067527 [Dorsal]
Scrobipalpa acuminatella
Family: Gelechiidae
BIN URI: BOLD:AAC1644

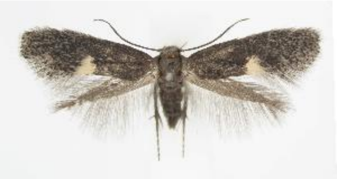

CNCLEP00027823 [Dorsal]
Perittia herrichiella
Family: Elachistidae
BIN URI: BOLD:AAC8613

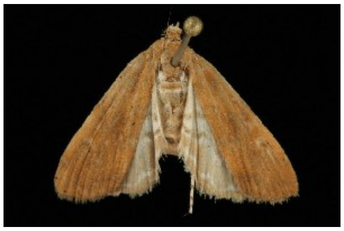

2005-ONT-1724 [Dorsal]
Elophila gyralis
Family: Crambidae
BIN URI: BOLD:AAA9838

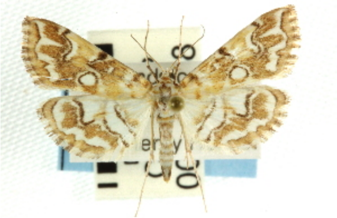

CNCLEP00087658 [Dorsal]
Elophila icciusalis
Family: Crambidae
BIN URI: BOLD:AAB0713

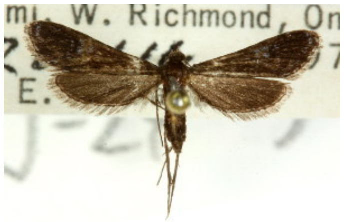

CNCLEP00087681 [Dorsal]
Elophila tinealis
Family: Crambidae
BIN URI: BOLD:AAD0306

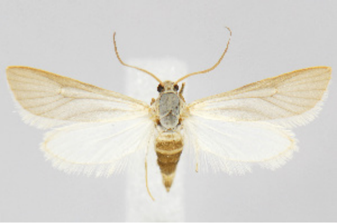

CNCLEP00040982 [Dorsal]
Acentria ephemerella
Family: Crambidae
BIN URI: BOLD:AAA8932

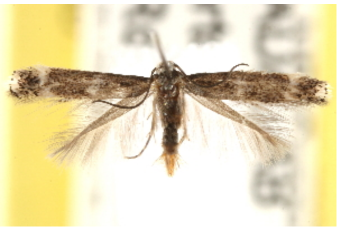

CCDB-23267-A01 [Dorsal]
Lepidoptera

BIN URI: BOLD:AAD9052

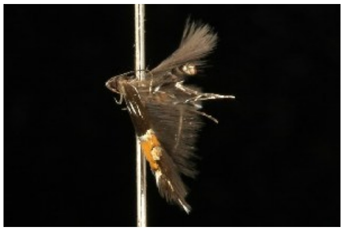

2005-ONT-2099 [Lateral]
Cosmopterix montisella
Family: Cosmopterigidae
BIN URI: BOLD:AAH4285

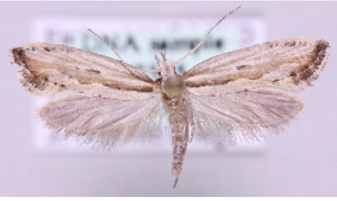

MM17535 [Dorsal]
Plutella porrectella
Family: Plutellidae
BIN URI: BOLD:ACG9804

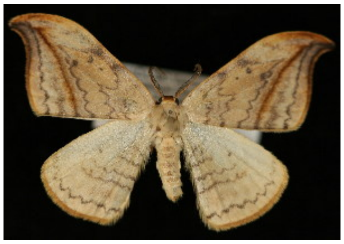

UBC-2006-0117 [Dorsal]
Drepana arcuata
Family: Drepanidae
BIN URI: BOLD:AAA3083

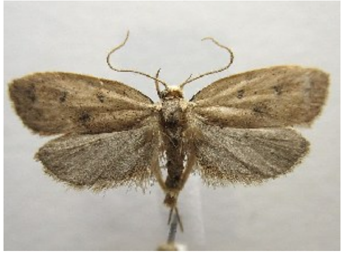

MDH000245 [Dorsal]
Machimia tentoriferella
Family: Depressariidae
BIN URI: BOLD:ABZ1624

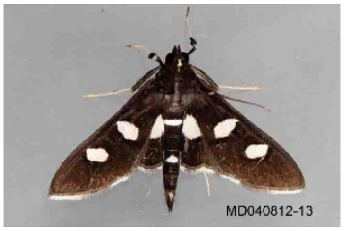

BIOUG02922-F02 [Dorsal]
Desmia maculalis
Family: Crambidae
BIN URI: BOLD:ACE8375

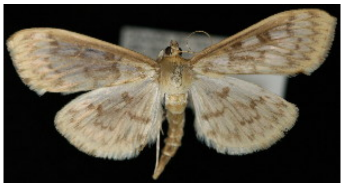

UBC-2006-0451 [Dorsal]
Herpetogramma thestealis
Family: Crambidae
BIN URI: BOLD:AAA2323

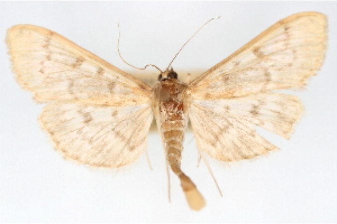

CNCLEP00075072 [Dorsal]
Herpetogramma aeglealis
Family: Crambidae
BIN URI: BOLD:AAA2324

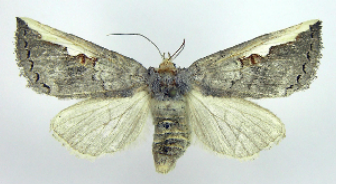

DH001502 [Dorsal]
Symmerista leucitys
Family: Notodontidae
BIN URI: BOLD:AAA7013

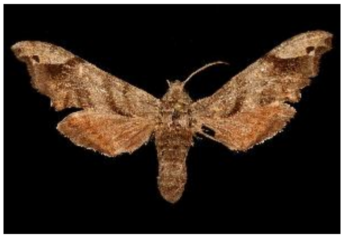

05-NCCC-794 [Dorsal]
Deidamia inscriptum
Family: Sphingidae
BIN URI: BOLD:AAB0001

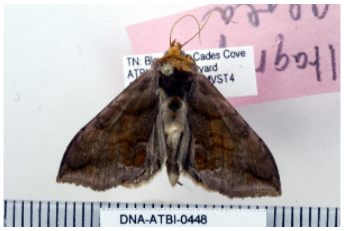

DNA-ATBI-0448 [Dorsal]
Allagrapha aerea
Family: Noctuidae
BIN URI: BOLD:AAB0752

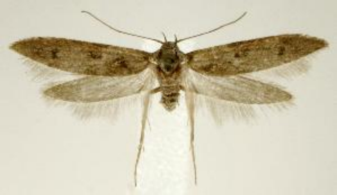

jflandry2354 [Dorsal]
Asaphocrita busckiella
Family: Blastobasidae
BIN URI: BOLD:AAA8938

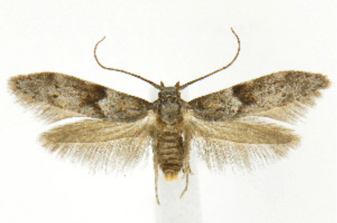

CNCLEP00040597 [Dorsal]
Blastobasis glandulella
Family: Blastobasidae
BIN URI: BOLD:AAB1096

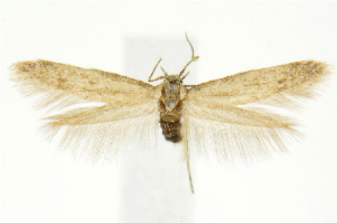

CNCLEP00040972 [Dorsal]
Teladoma helianthi
Family: Cosmopterigidae
BIN URI: BOLD:AAE1519

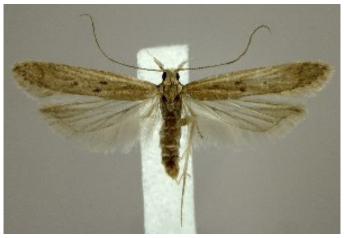

DNA-ATBI-3376 [Dorsal]
Dichomeris ligulella
Family: Gelechiidae
BIN URI: BOLD:AAA8109

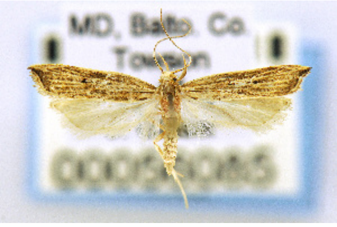

CNCLEP00056085 [Dorsal]
Helcystogramma hystricella
Family: Gelechiidae
BIN URI: BOLD:AAE7016

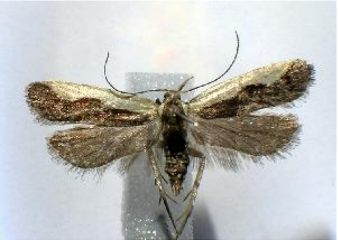

MDH003197 [Dorsal]
Dichomeris inserrata
Family: Gelechiidae
BIN URI: BOLD:AAH4488

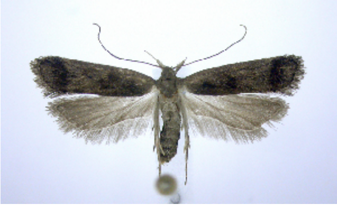

jflandry0861 [Dorsal]
Dichomeris leuconotella
Family: Gelechiidae
BIN URI: BOLD:AAB0651

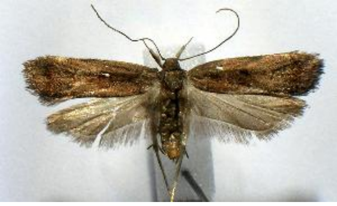

MDH001252 [Dorsal]
Dichomeris
Family: Gelechiidae
BIN URI: BOLD:AAI6258

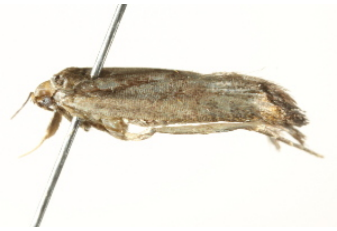

BIOUG01292-C12 [Lateral]
Dichomeris furia
Family: Gelechiidae
BIN URI: BOLD:AAI9560

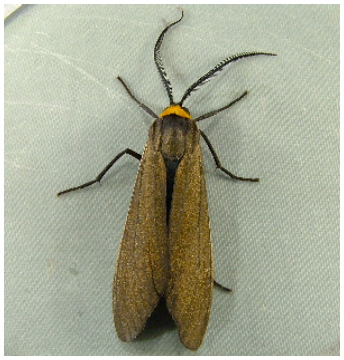

RWWA-2506 [Dorsal]
Cisseps fulvicollis
Family: Erebidae
BIN URI: BOLD:AAA4200

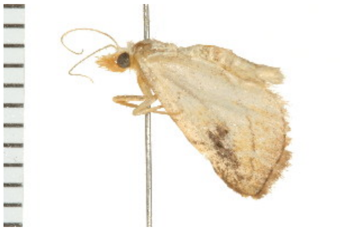

PPBP-1031 [Lateral]
Rivula propinqualis
Family: Erebidae
BIN URI: BOLD:AAA4282

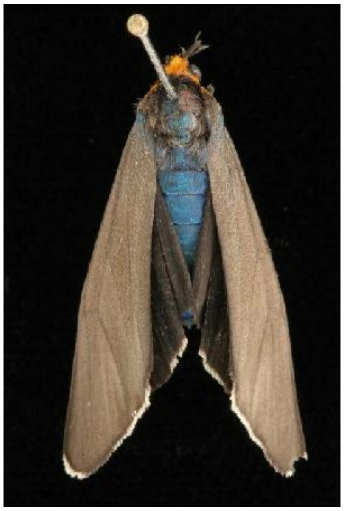

04HBL005554 [Dorsal]
Ctenucha virginica
Family: Erebidae
BIN URI: BOLD:AAA6017

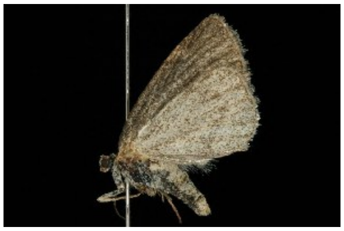

HLC-20003 [Lateral]
Protodeltote albidula
Family: Noctuidae
BIN URI: BOLD:AAA2331

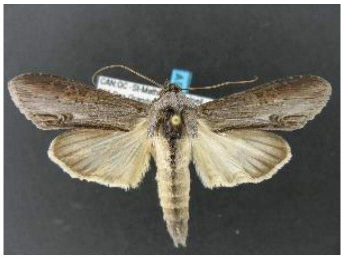

DH008842 [Dorsal]
Cucullia asteroides
Family: Noctuidae
BIN URI: BOLD:AAB9406

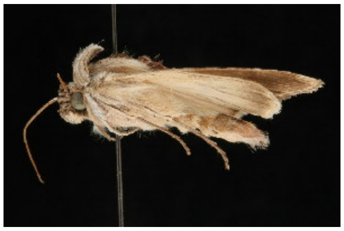

DNA-ATBI-0643 [Lateral]
Cucullia convexipennis
Family: Noctuidae
BIN URI: BOLD:AAD2762

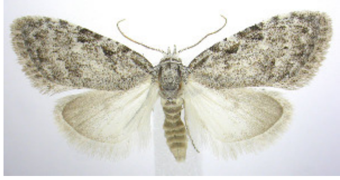

jflandry0308 [Dorsal]
Nola ovilla
Family: Nolidae
BIN URI: BOLD:AAD1810

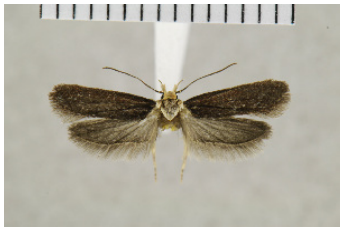

CNCLEP00097815 [Dorsal]
Depressaria depressana
Family: Depressariidae
BIN URI: BOLD:AAE7397

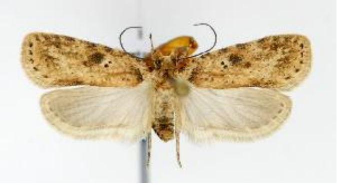

CNCLEP00020426 [Dorsal]
Agonopterix arenella
Family: Depressariidae
BIN URI: BOLD:AAC6982

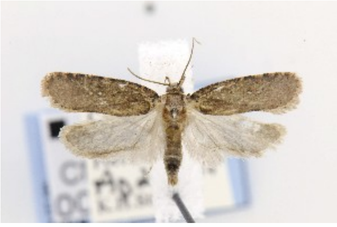

CNCLEP00121690 [Dorsal]
Agonopterix dimorphella
Family: Depressariidae
BIN URI: BOLD:AAC0205

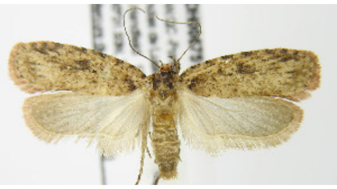

MDH006751 [Dorsal]
Agonopterix pulvipennella
Family: Depressariidae
BIN URI: BOLD:AAA7550

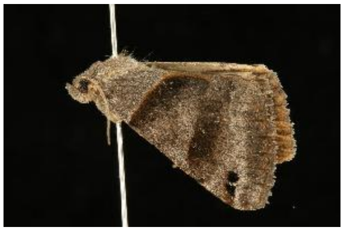

2006-ONT-1419 [Lateral]
Caenurgina crassiuscula
Family: Noctuidae
BIN URI: BOLD:AAA4171

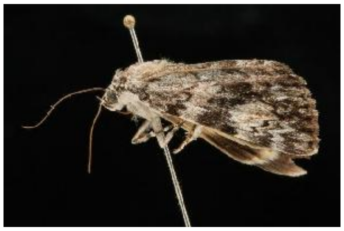

04HBL002239 [Lateral]
Catocala lineella
Family: Noctuidae
BIN URI: BOLD:AAA5644

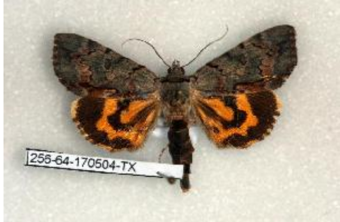

256-64-170504-TX [Dorsal]
Catocala grynea
Family: Noctuidae
BIN URI: BOLD:AAA9713

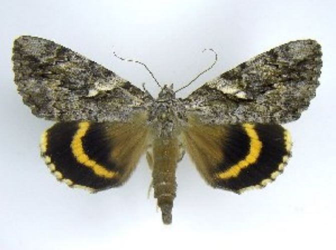

DH010565 [Dorsal]
Catocala cerogama
Family: Noctuidae
BIN URI: BOLD:AAB3383

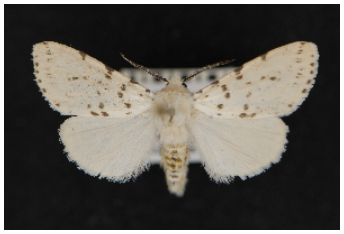

CNCLEP 73783 [Dorsal]
Hyphantria cunea
Family: Erebidae
BIN URI: BOLD:AAA2435

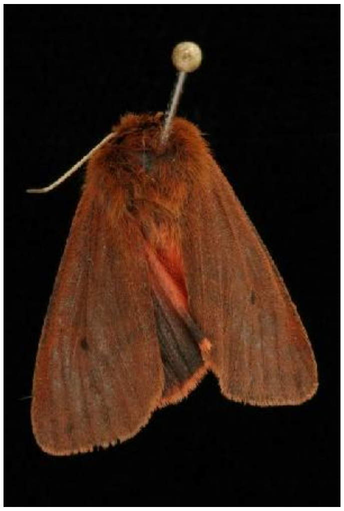

04HBL005035 [Dorsal]
Phragmatobia fuliginosa
Family: Erebidae
BIN URI: BOLD:AAA6177

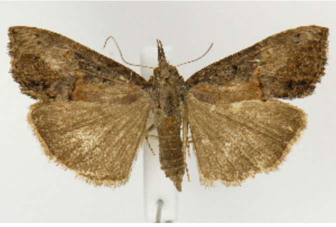

CNCLEP00040357 [Dorsal]
Hypena scabra
Family: Erebidae
BIN URI: BOLD:AAA4222

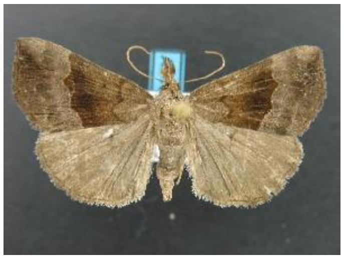

DH012080 [Dorsal]
Hypena madefactalis
Family: Erebidae
BIN URI: BOLD:ACE2873

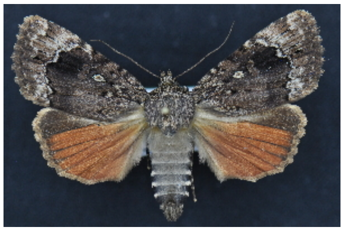

CNCLEP 94242 [Dorsal]
Amphipyra pyramidoides
Family: Noctuidae
BIN URI: BOLD:AAA8525

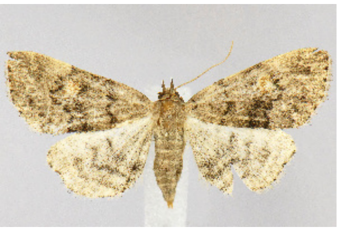

CNCLEP00041073 [Dorsal]
Idia concisa
Family: Noctuidae
BIN URI: BOLD:AAA2229

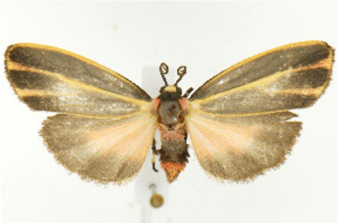

CNCLEP00040847 [Dorsal]
Hypoprepia fucosa
Family: Erebidae
BIN URI: BOLD:AAA4714

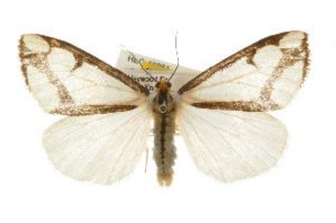

HLC-16961 [Dorsal]
Haploa lecontei
Family: Erebidae
BIN URI: BOLD:AAA8684

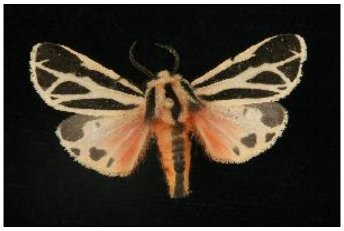

05-NCCC-737 [Dorsal]
Apantesis phalerata
Family: Erebidae
BIN URI: BOLD:ABY9321

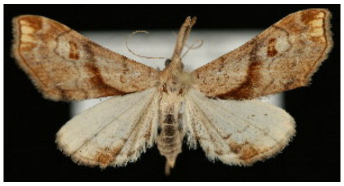

UBC-2006-0801 [Dorsal]
Palthis angulalis
Family: Noctuidae
BIN URI: BOLD:AAA3933

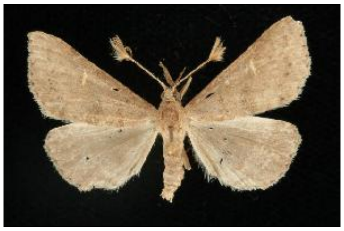

06-NCCC-595 [Dorsal]
Renia adspergillus
Family: Noctuidae
BIN URI: BOLD:AAA6692

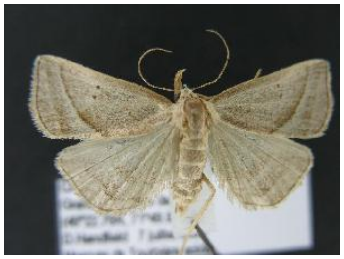

DH013195 [Dorsal]
Macrochilo absorptalis
Family: Noctuidae
BIN URI: BOLD:AAB3885

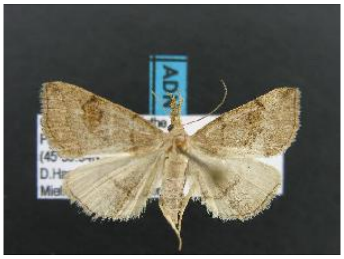

DH012266 [Dorsal]
Phalaenostola metonalis
Family: Noctuidae
BIN URI: BOLD:ACF3696

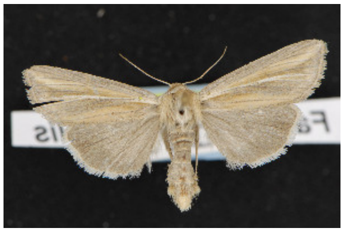

NOC14648 [Dorsal]
Dargida terrapictalis
Family: Noctuidae
BIN URI: BOLD:AAB3769

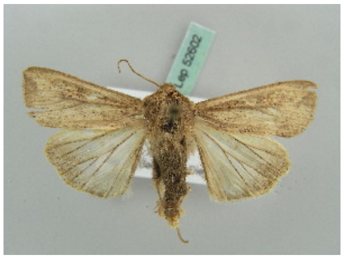

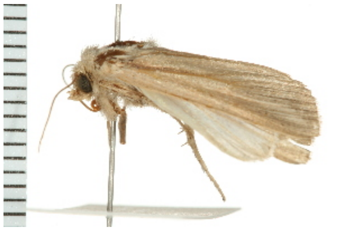

PPBP-1211 [Lateral]

BC ZSM Lep 52602 [Adult]
Mythimna sequax
Family: Noctuidae
BIN URI: BOLD:AAA2482

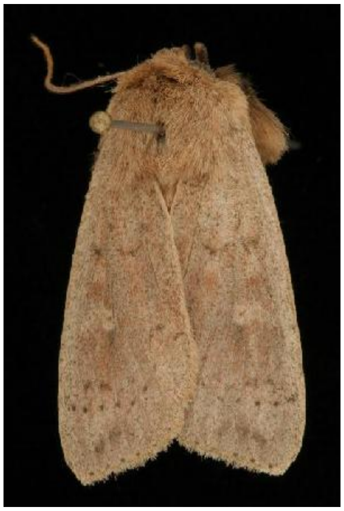

DNA-ATBI-0722 [Dorsal]
Leucania pseudargyria
Family: Noctuidae
BIN URI: BOLD:AAB6246

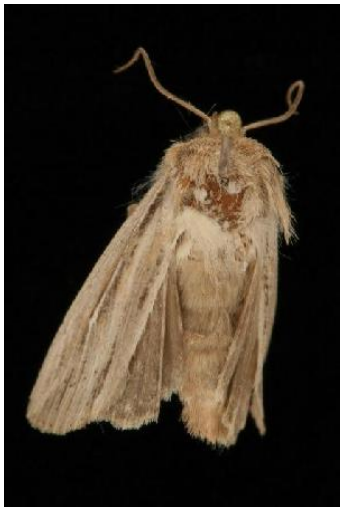

04HBL005175 [Dorsal]
Leucania commoides
Family: Noctuidae
BIN URI: BOLD:AAA8386

Leucania phragmitidicola
Family: Noctuidae
BIN URI: BOLD:ABX6101

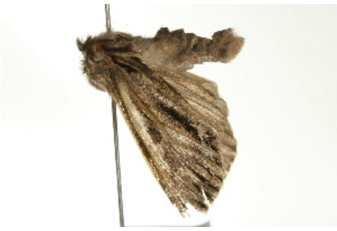

BL239 [Lateral]
Hyppa xylinoides
Family: Noctuidae
BIN URI: BOLD:ABY9574

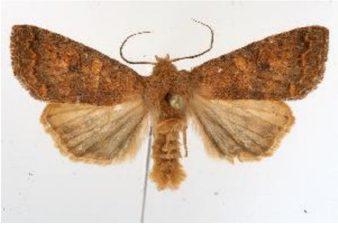

05-NCCC-454 [Dorsal]
Sunira bicolorago
Family: Noctuidae
BIN URI: BOLD:AAA4426

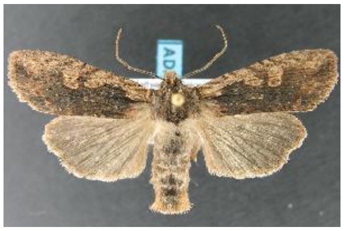

DH013268 [Dorsal]
Lithophane hemina
Family: Noctuidae
BIN URI: BOLD:AAB1070

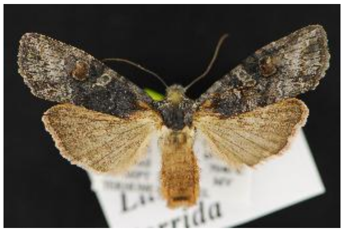

CNCNoctuoidea13876 [Dorsal]
Lithophane torrida
Family: Noctuidae
BIN URI: BOLD:AAB5821

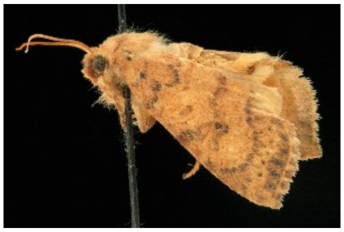

06-JKA-0125 [Lateral]
Anathix ralla
Family: Noctuidae
BIN URI: BOLD:AAC9569

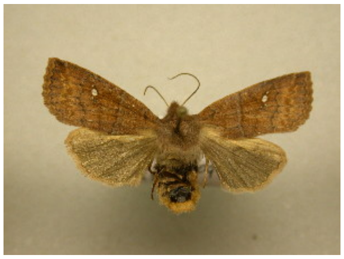

11-NCCC-0013 [Dorsal]
Eupsilia vinulenta
Family: Noctuidae
BIN URI: BOLD:AAB4640

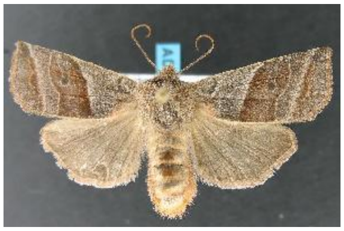

DH013349 [Dorsal]
Eupsilia devia
Family: Noctuidae
BIN URI: BOLD:AAD9847

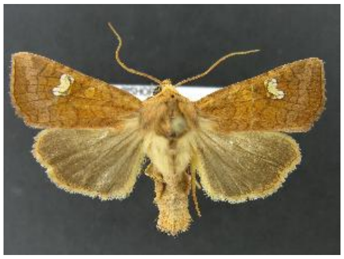

DH006295 [Dorsal]
Amphipoea americana
Family: Noctuidae
BIN URI: BOLD:AAC0644

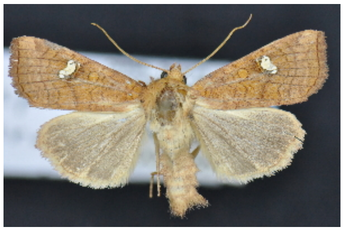

CNCLEP 92351 [Dorsal]
Amphipoea interoceanica
Family: Noctuidae
BIN URI: BOLD:ABZ0147

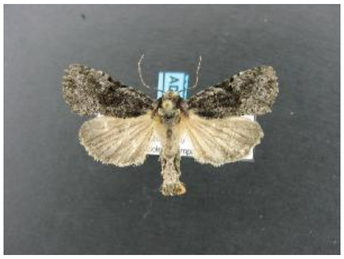

DH011988 [Dorsal]
Oligia modica
Family: Noctuidae
BIN URI: BOLD:ABZ2233

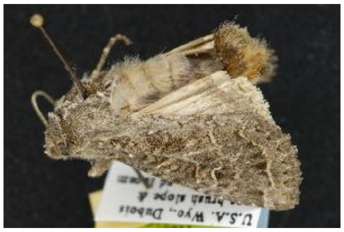

CNCNoctuoidea12311 [Lateral]
Apamea devastator
Family: Noctuidae
BIN URI: BOLD:ABY5257

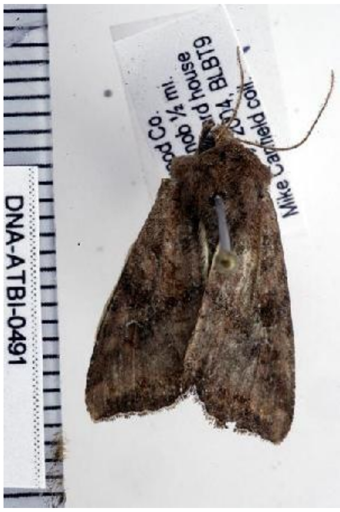

DNA-ATBI-0491 [Dorsal]
Loscopia velata
Family: Noctuidae
BIN URI: BOLD:AAB5969

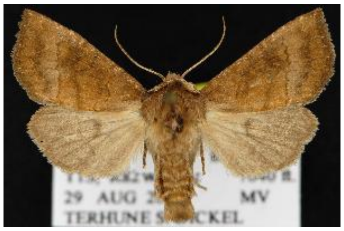

CNCNoctuoidea12932 [Dorsal]
Papaipema nelita
Family: Noctuidae
BIN URI: BOLD:AAB8711

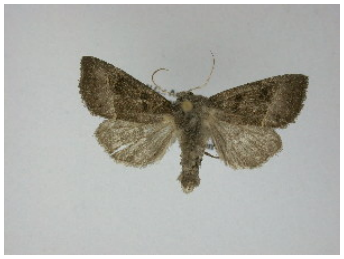

14-NCCC-660 [Dorsal]
Papaipema nebris
Family: Noctuidae
BIN URI: BOLD:ACF1624

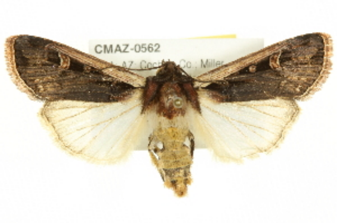

CMAZ-0562 [Dorsal]
Striacosta albicosta
Family: Noctuidae
BIN URI: BOLD:AAF3338

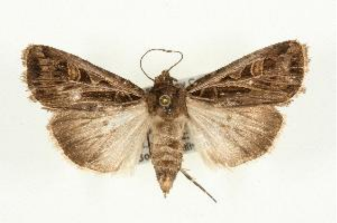

07-NCNW-0193 [Dorsal]
Feltia jaculifera
Family: Noctuidae
BIN URI: BOLD:AAA3351

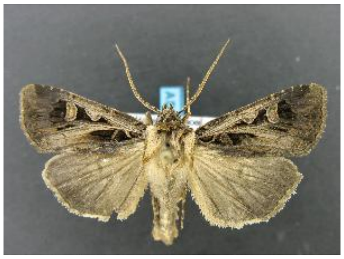

DH012203 [Dorsal]
Feltia tricosa
Family: Noctuidae
BIN URI: BOLD:ACE9865

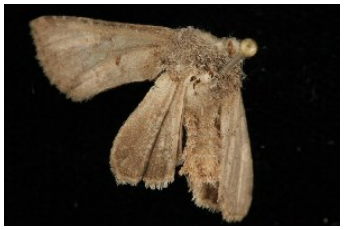

2005-ONT-1562 [Dorsal]
Lacinipolia meditata
Family: Noctuidae
BIN URI: BOLD:AAA8562

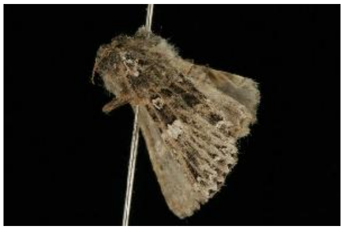

2005-ONT-197 [Lateral]
Melanchra adjuncta
Family: Noctuidae
BIN URI: BOLD:ACF4823

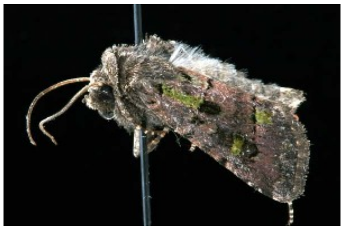

DNA-ATBI-2444 [Lateral]
Lacinipolia renigera
Family: Noctuidae
BIN URI: BOLD:ABZ4680

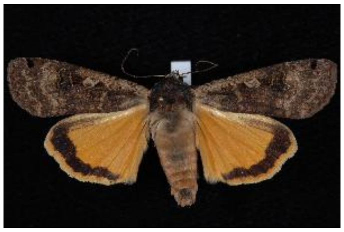

UBC-2007-0001 [Dorsal]
Noctua pronuba
Family: Noctuidae
BIN URI: BOLD:AAA2632

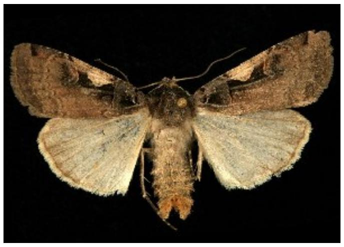

CGWC-0798 [Dorsal]
Xestia c-nigrum
Family: Noctuidae
BIN URI: BOLD:AAA2144

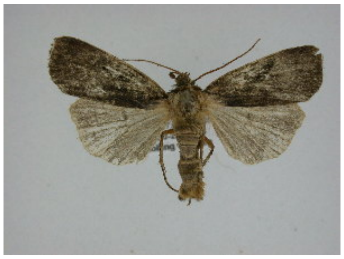

14-NCCC-696 [Dorsal]
Pseudohermonassa bicarnea
Family: Noctuidae
BIN URI: BOLD:AAB1024

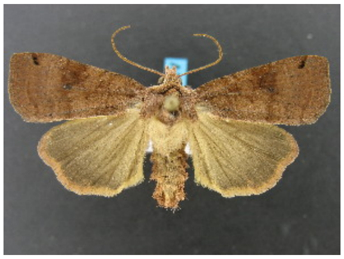

DH008022 [Dorsal]
Xestia smithii
Family: Noctuidae
BIN URI: BOLD:AAA2590

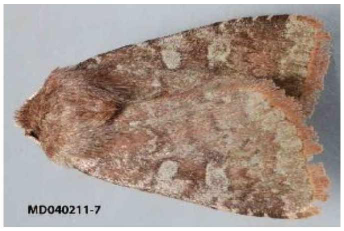

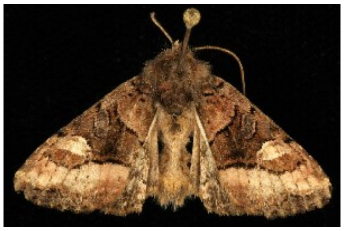

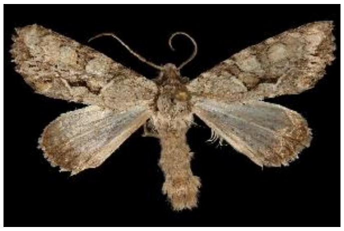

MDOK-4361 [Dorsal]
Cerastis tenebrifera
Family: Noctuidae
BIN URI: BOLD:AAC1487

HLC-22923 [Dorsal]
Euplexia benesimilis
Family: Noctuidae
BIN URI: BOLD:AAA4097

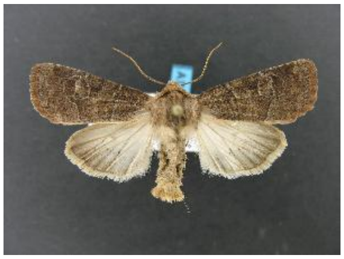

DH009278 [Dorsal]
Crocigrapha normani
Family: Noctuidae
BIN URI: BOLD:AAA6924

05-NCCC-795 [Dorsal]
Achatia distincta
Family: Noctuidae
BIN URI: BOLD:AAB7392

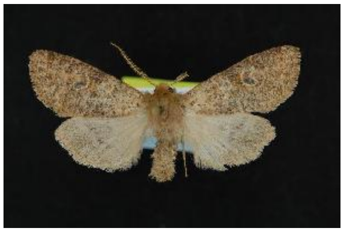

CNCNoctuoidea12098 [Dorsal]
Orthosia rubescens
Family: Noctuidae
BIN URI: BOLD:AAC0946

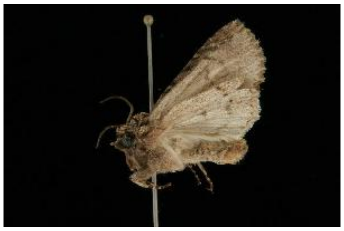

DNA-ATBI-2480 [Lateral]
Morrisonia confusa
Family: Noctuidae
BIN URI: BOLD:AAA6652

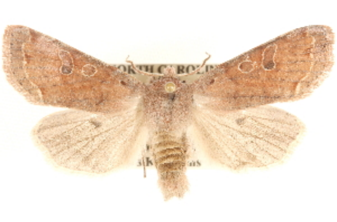

CCDB-23287-B02 [Dorsal]
Orthosia hibisci
Family: Noctuidae
BIN URI: BOLD:AAA4128

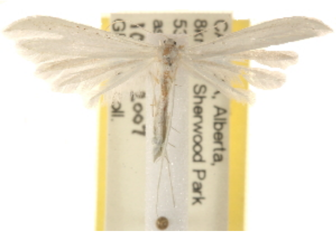

CCDB-22977-F11 [Dorsal]
Hellinsia homodactylus
Family: Pterophoridae
BIN URI: BOLD:AAB2714

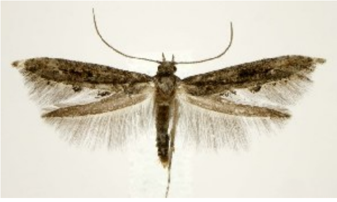

DNA-ATBI-3410 [Dorsal]
Epermenia
Family: Epermeniidae
BIN URI: BOLD:AAF0142

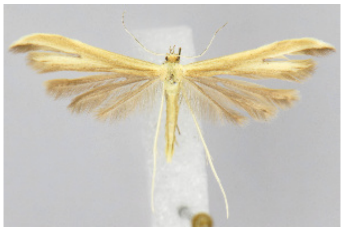

CNCLEP00040785 [Dorsal]
Hellinsia pectodactylus
Family: Pterophoridae
BIN URI: BOLD:ACF3437

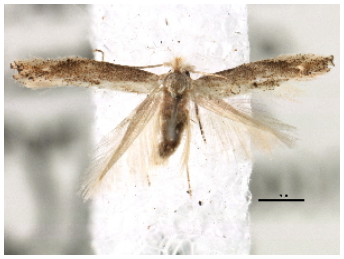

BIOUG16764-H06 [Dorsal]
Bucculatrix
Family: Bucculatricidae
BIN URI: BOLD:AAB4931

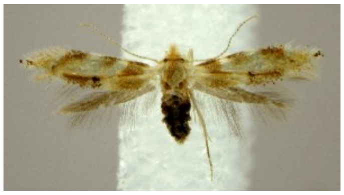

DNA-ATBI-3021 [Dorsal]
Bucculatrix
Family: Bucculatricidae
BIN URI: BOLD:AAH5592

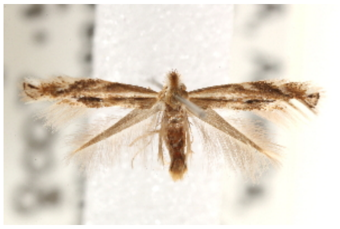

CCDB-22978-A08 [Dorsal]
Bucculatrix pomifoliella
Family: Bucculatricidae
BIN URI: BOLD:AAD2085

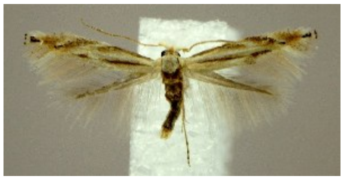

DNA-ATBI-3019 [Dorsal]
Bucculatrix
Family: Bucculatricidae
BIN URI: BOLD:AAH5599

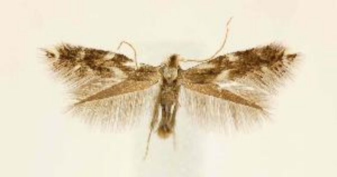

CNCLEP00027519 [Dorsal]
Bucculatrix
Family: Bucculatricidae
BIN URI: BOLD:AAI3849

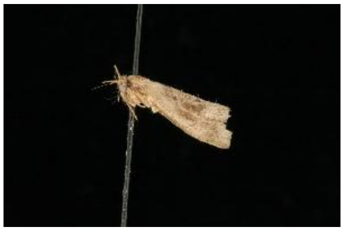

04HBL007216 [Lateral]
Eucosma similana
Family: Tortricidae
BIN URI: BOLD:AAB4297

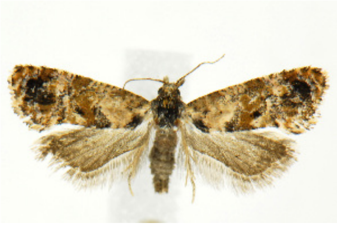

CNCLEP00040915 [Dorsal]
Cochylis hoffmanana
Family: Tortricidae
BIN URI: BOLD:AAB3571

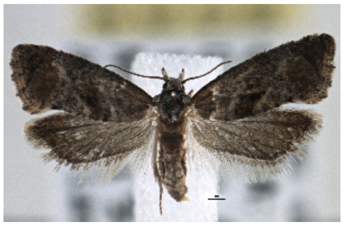

CNCLEP00101832 [Dorsal]
Phtheochroa temerana
Family: Tortricidae
BIN URI: BOLD:AAB7534

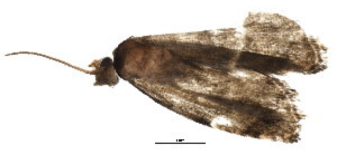

BIOUG01630-B11 [Lateral]
Phalonia nr. maiana
Family: Tortricidae
BIN URI: BOLD:AAM0777

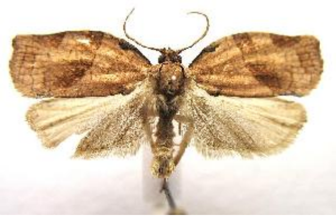

MDH001278 [Dorsal]
Choristoneura rosaceana
Family: Tortricidae
BIN URI: BOLD:AAA1517

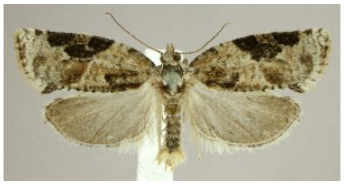

jflandry2199 [Dorsal]
Argyrotaenia mariana
Family: Tortricidae
BIN URI: BOLD:AAA4119

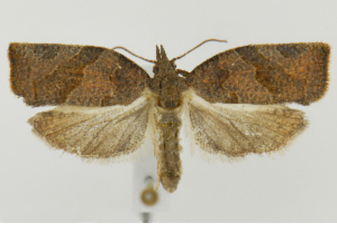

CNCLEP00038641 [Dorsal]
Pandemis limitata
Family: Tortricidae
BIN URI: BOLD:AAA3659

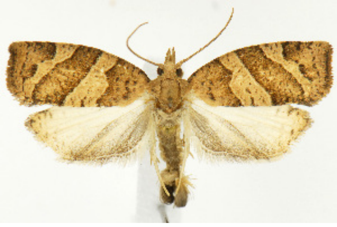

CNCLEP00041070 [Dorsal]
Pandemis lamprosana
Family: Tortricidae
BIN URI: BOLD:AAA8840

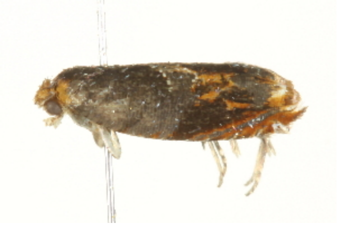

SNS10IL-01097 [Lateral]
Ancylis muricana
Family: Tortricidae
BIN URI: BOLD:AAU7760

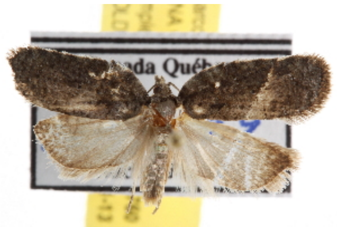

CNCLEP00101760 [Dorsal]
Acleris hudsoniana
Family: Tortricidae
BIN URI: BOLD:AAA7667

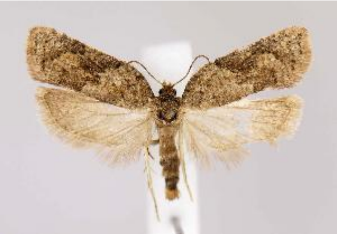

CNCLEP00029418 [Dorsal]
Acleris cornana
Family: Tortricidae
BIN URI: BOLD:ABZ7431

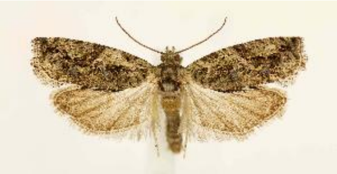

CNCLEP00025103 [Dorsal]
Proteoteras aesculana
Family: Tortricidae
BIN URI: BOLD:AAA6740

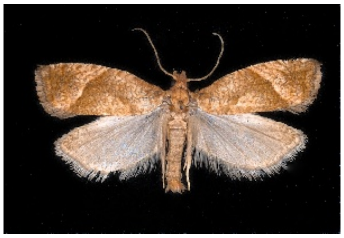

C096001A16Aug2006 [Dorsal]
Eucosma derelicta
Family: Tortricidae
BIN URI: BOLD:AAA9420

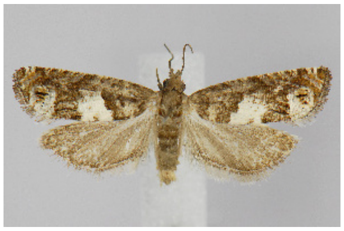

CNCLEP00040720 [Dorsal]
Phaneta parmatana
Family: Tortricidae
BIN URI: BOLD:AAA5487

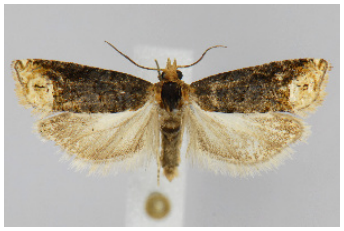

CNCLEP00040726 [Dorsal]
Phaneta ochroterminana
Family: Tortricidae
BIN URI: BOLD:AAA5486

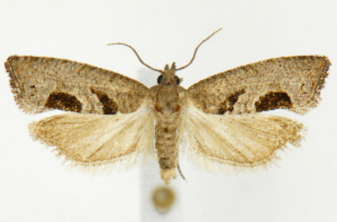

CNCLEP00040734 [Dorsal]
Phaneta tomonana
Family: Tortricidae
BIN URI: BOLD:AAB2093

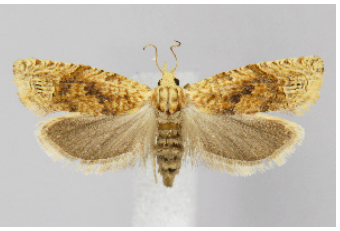

CNCLEP00040312 [Dorsal]
Phaneta ochrocephala
Family: Tortricidae
BIN URI: BOLD:AAB4433

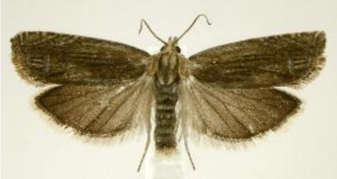

jflandry2488 [Dorsal]
Phaneta umbrastriana
Family: Tortricidae
BIN URI: BOLD:AAA8046

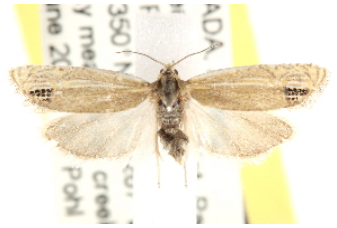

CCDB-22976-D04 [Dorsal]
Phaneta verna
Family: Tortricidae
BIN URI: BOLD:ABA8759

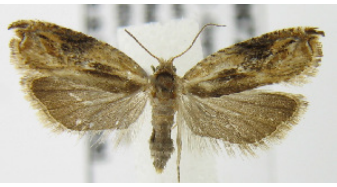

MDH006725 [Dorsal]
Ancylis spiraeifoliana
Family: Tortricidae
BIN URI: BOLD:AAC5326

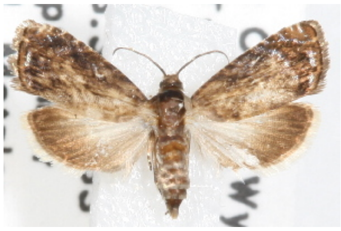

09-JBTOR-1305 [Dorsal]
Grapholita prunivora
Family: Tortricidae
BIN URI: BOLD:AAG0330

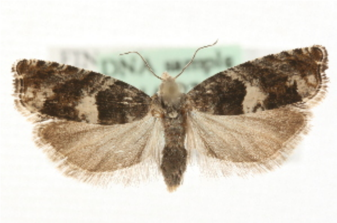

MM14227 [Dorsal]
Epinotia demarniana
Family: Tortricidae
BIN URI: BOLD:AAB0710

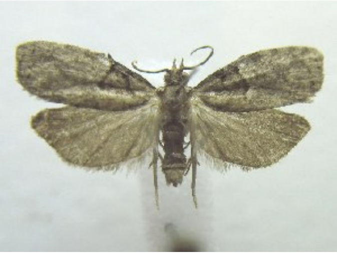

MDH000531 [Dorsal]
Epinotia medioviridana
Family: Tortricidae
BIN URI: BOLD:AAC2645

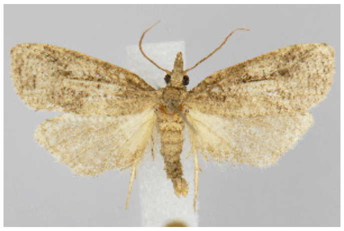

CNCLEP00040313 [Dorsal]
Platynota idaeusalis
Family: Tortricidae
BIN URI: BOLD:ABY7901

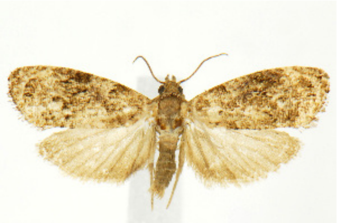

CNCLEP00040914 [Dorsal]
Endothenia hebesana
Family: Tortricidae
BIN URI: BOLD:AAA3308

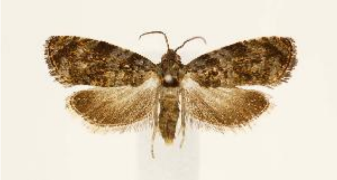

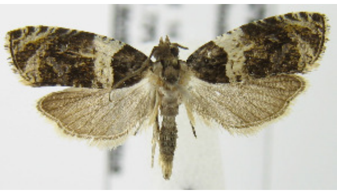

CNCLEP00026935 [Dorsal]
Pristerognatha fuligana
Family: Tortricidae
BIN URI: BOLD:AAC7661

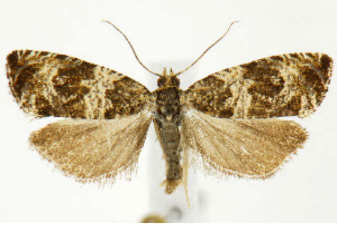

CNCLEP00041057 [Dorsal]
Olethreutes baccatanum
Family: Tortricidae
BIN URI: BOLD:AAA7669

MDH006793 [Dorsal]
Olethreutes fasciatana
Family: Tortricidae
BIN URI: BOLD:AAC2533

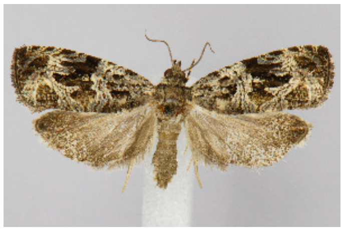

CNCLEP00040315 [Dorsal]
Olethreutes atrodentana
Family: Tortricidae
BIN URI: BOLD:ACF0606

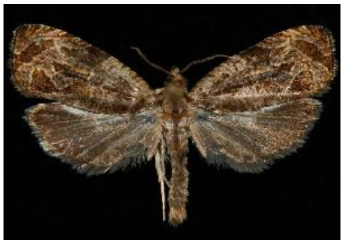

DNA-ATBI-6352 [Dorsal]
Olethreutes permundana
Family: Tortricidae
BIN URI: BOLD:ACF0608

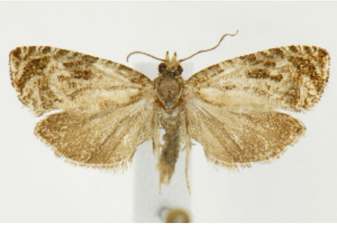

CNCLEP00040323 [Dorsal]
Olethreutes valdanum
Family: Tortricidae
BIN URI: BOLD:ACF0609

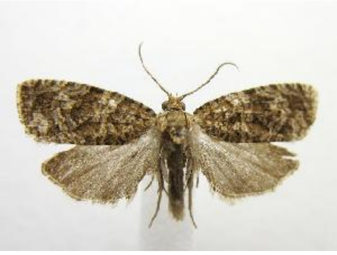

MDH002273 [Dorsal]
Olethreutes electrofuscum
Family: Tortricidae
BIN URI: BOLD:ACF4045

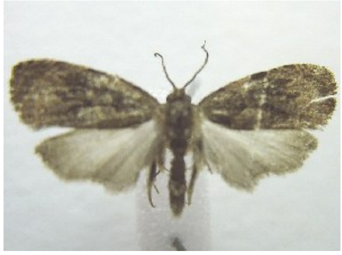

MDH001777 [Dorsal]
Olethreutes permundana
Family: Tortricidae
BIN URI: BOLD:ACE6591

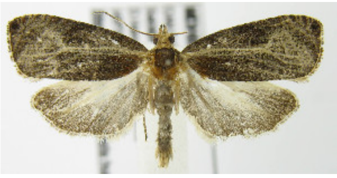

MDH006338 [Dorsal]
Olethreutes quadrifidus
Family: Tortricidae
BIN URI: BOLD:ACF4260

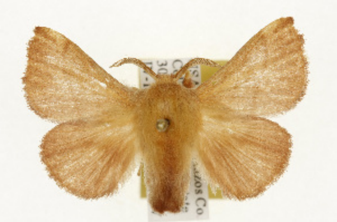

TAMUICEGR-0828 [Dorsal]
Malacosoma disstria
Family: Lasiocampidae
BIN URI: BOLD:AAA4130

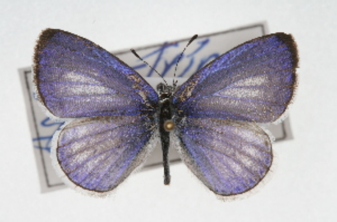

CSU-CPG-LEP001236 [Dorsal]
Celastrina gozora
Family: Lycaenidae
BIN URI: BOLD:ACF0806

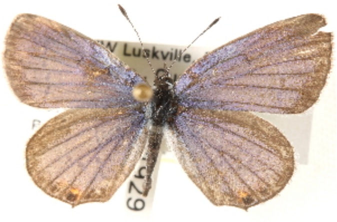

CCDB-24271-B07 [Dorsal]
Cupido comyntas
Family: Lycaenidae
BIN URI: BOLD:ACF5210

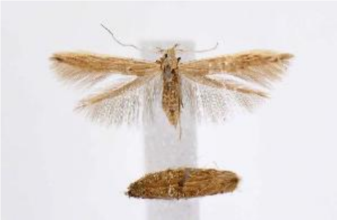

CNCLEP00010324 [Dorsal]
Coleophora intermediella
Family: Coleophoridae
BIN URI: BOLD:AAA3993

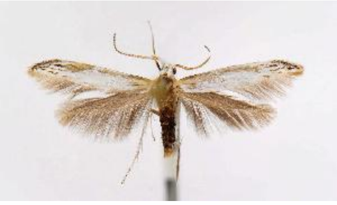

CNCLEP00027943 [Dorsal]
Coleophora tiliaefoliella
Family: Coleophoridae
BIN URI: BOLD:AAA7936

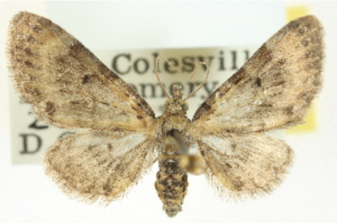

CCDB-20270-B10 [Dorsal]
Eupithecia chlorofasciata
Family: Geometridae
BIN URI: BOLD:AAA4178

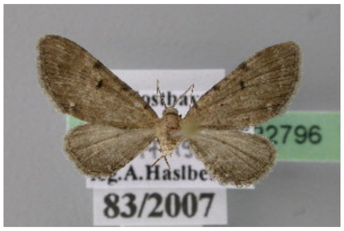

BC ZSM Lep 22796 [Dorsal]
Eupithecia goossensiata
Family: Geometridae
BIN URI: BOLD:AAA4217

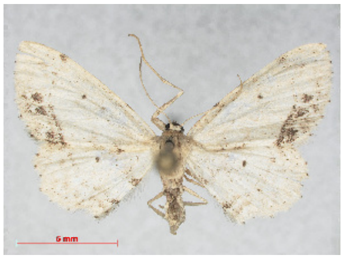

RMNH.INS.544467 [Dorsal]
Idaea dimidiata
Family: Geometridae
BIN URI: BOLD:AAA4213

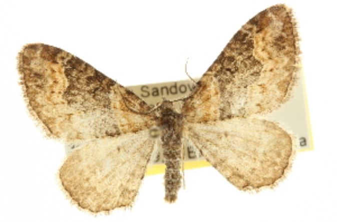

CCDB-20824-E01 [Dorsal]
Xanthorhoe ferrugata
Family: Geometridae
BIN URI: BOLD:AAA3817

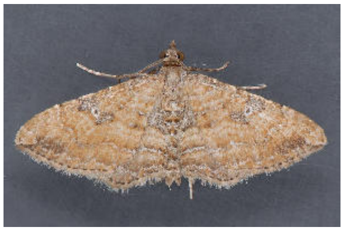

MDOK-3232 [Dorsal]
Orthonama obstipata
Family: Geometridae
BIN URI: BOLD:AAA3430

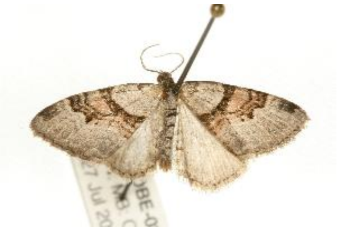

07PROBE-03851 [Dorsal]
Xanthorhoe labradorensis
Family: Geometridae
BIN URI: BOLD:AAA7001

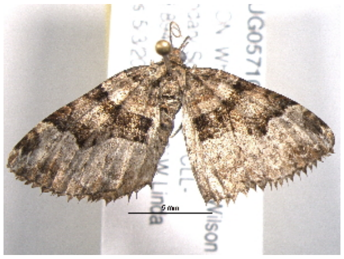

BIOUG05710-F02 [Dorsal]
Xanthorhoe lacustrata
Family: Geometridae
BIN URI: BOLD:AAA8660

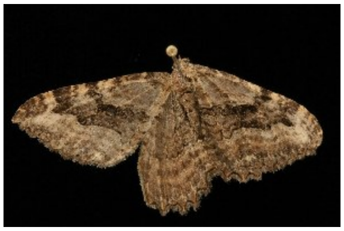

04HBL00854 [Dorsal]
Coryphista meadii
Family: Geometridae
BIN URI: BOLD:AAB1307

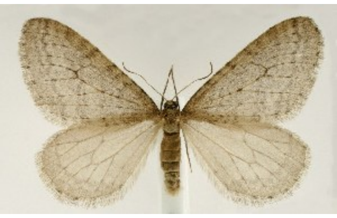

jflandry1146 [Dorsal]
Operophtera bruceata
Family: Geometridae
BIN URI: BOLD:AAA2999

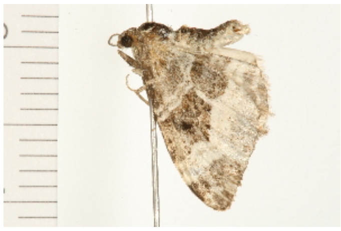

PPBP-2051 [Lateral]
Epirrhoe alternata
Family: Geometridae
BIN URI: BOLD:AAA3371

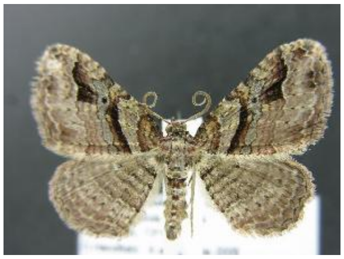

DH012776 [Dorsal]
Costaconvexa centrostrigaria
Family: Geometridae
BIN URI: BOLD:AAA4271

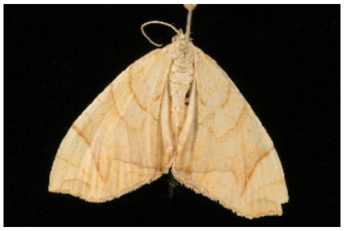

04HBL005685 [Dorsal]
Eulithis gracilineata
Family: Geometridae
BIN URI: BOLD:AAB2705

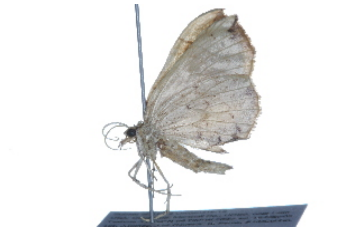

09BBLEP-01372 [Lateral]
Eulithis gracilineata
Family: Geometridae
BIN URI: BOLD:AAB2706

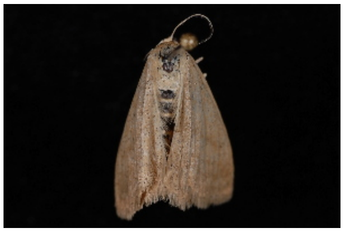

10-JDWBC-5907 []
Scopula inductata
Family: Geometridae
BIN URI: BOLD:AAA5383

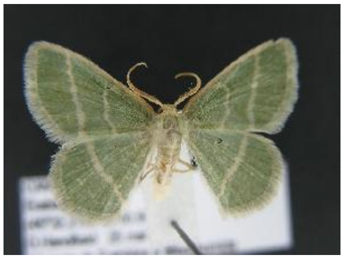

DH013036 [Dorsal]
Chlorochlamys chloroleucaria
Family: Geometridae
BIN URI: BOLD:AAA7313

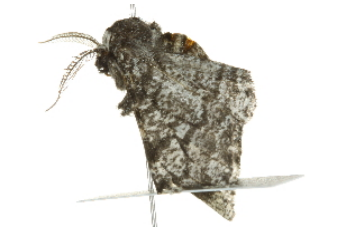

10BBCLP-1302 [Lateral]
Biston betularia
Family: Geometridae
BIN URI: BOLD:AAA1588

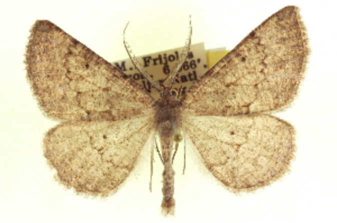

CCDB-20818-G09 [Dorsal]
Somatolophia incana
Family: Geometridae
BIN URI: BOLD:AAA9811

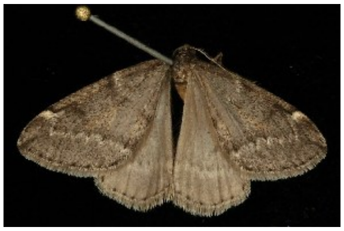

2005-ONT-2361 [Dorsal]
Alsophila pometaria
Family: Geometridae
BIN URI: BOLD:AAB0196

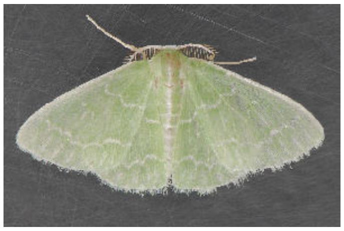

MDOK-3073 [Dorsal]
Synchlora frondaria
Family: Geometridae
BIN URI: BOLD:AAA5690

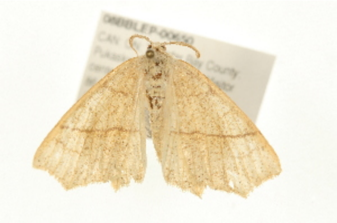

08BBLEP-00650 [Dorsal]
Besma quercivoraria
Family: Geometridae
BIN URI: BOLD:AAA7768

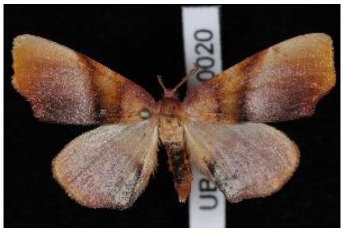

UBC-2007-0020 [Dorsal]
Plagodis phlogosaria
Family: Geometridae
BIN URI: BOLD:AAA3984

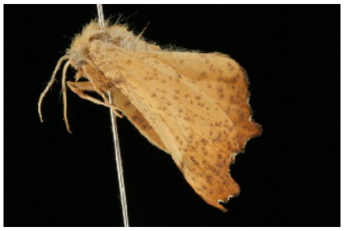

04HBL006792 [Lateral]
Ennomos magnaria
Family: Geometridae
BIN URI: BOLD:AAA5557

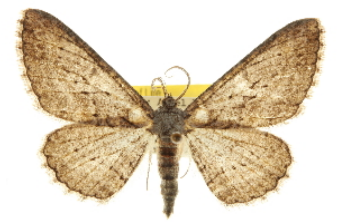

CCDB-20807-C08 [Dorsal]
Anavitrinella addendaria
Family: Geometridae
BIN URI: BOLD:AAA2278

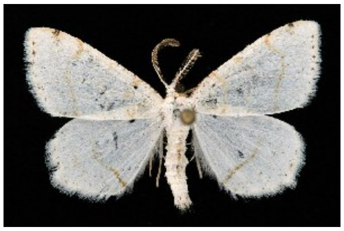

05-NCCC-168 [Dorsal]
Speranza pustularia
Family: Geometridae
BIN URI: BOLD:AAA4456

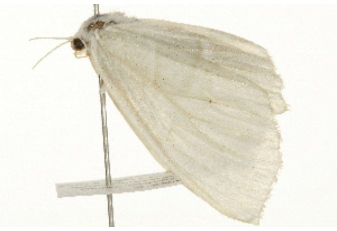

09BBELE-2043 [Lateral]
Campaea perlata
Family: Geometridae
BIN URI: BOLD:AAA2078

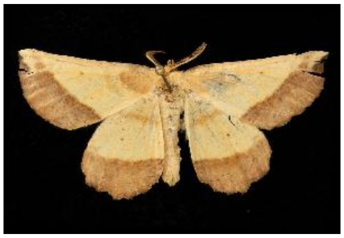

moth977.01 [Dorsal]
Euchlaena serrata
Family: Geometridae
BIN URI: BOLD:ABZ2601

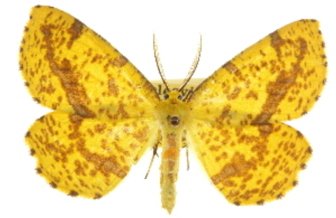

CCDB-20809-G07 [Dorsal]
Xanthotype barnesi
Family: Geometridae
BIN URI: BOLD:AAA6610

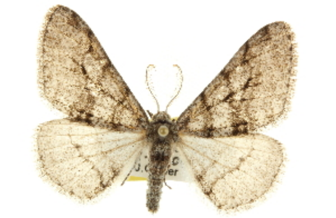

CCDB-20808-D11 [Dorsal]
Phigalia titea
Family: Geometridae
BIN URI: BOLD:AAA5234

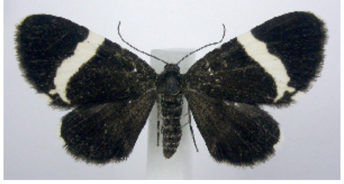

jflandry0580 [Dorsal]
Trichodezia albovittata
Family: Geometridae
BIN URI: BOLD:AAA6926

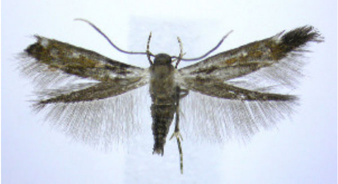

jflandry0042 [Dorsal]
Mompha terminella
Family: Momphidae
BIN URI: BOLD:AAX4784

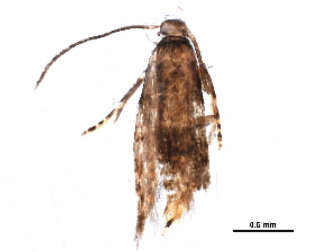

BIOUG22240-E08 [Dorsal]
Mompha
Family: Momphidae
BIN URI: BOLD:ACV2157

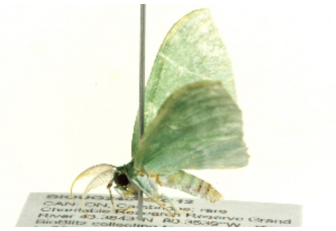

BIOUG24040-C12 [Lateral]
Dyspteris
Family: Geometridae

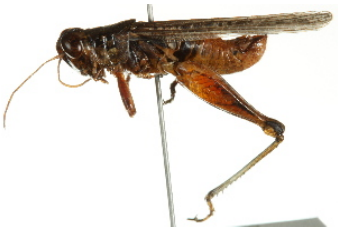

BIOUG02004-G03 [Lateral]
Melanoplus cinereus
Family: Acrididae
BIN URI: BOLD:AAA4555

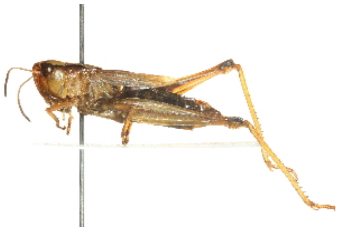

BIOUG00739-F08 [Lateral]
Chorthippus curtipennis
Family: Acrididae
BIN URI: BOLD:AAA6822

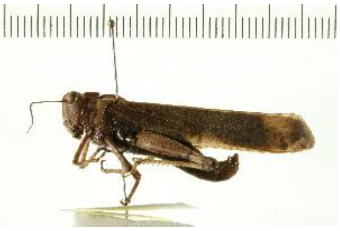

ORTH 0060.02 [Lateral]
Dissosteira carolina
Family: Acrididae
BIN URI: BOLD:AAD3251

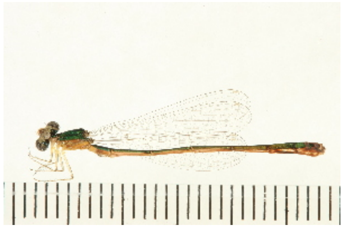

08OMSOD-0168 [Lateral]
Nehalennia irene
Family: Coenagrionidae
BIN URI: BOLD:AAA5874

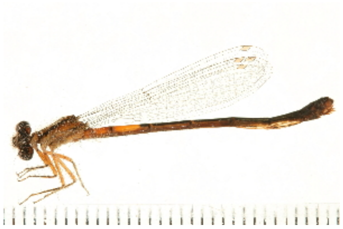

08OMSOD-0040 [Lateral]
Ischnura verticalis
Family: Coenagrionidae
BIN URI: BOLD:AAA2219

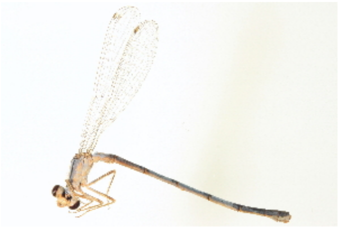

BIOUG02482-H06 [Lateral]
Ischnura
Family: Coenagrionidae
BIN URI: BOLD:AAI2160

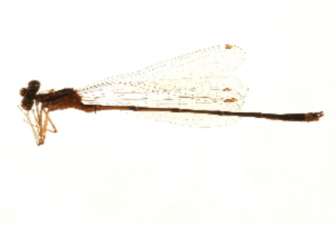

09BBEOD-0121 [Lateral]
Enallagma signatum
Family: Coenagrionidae
BIN URI: BOLD:AAD5238

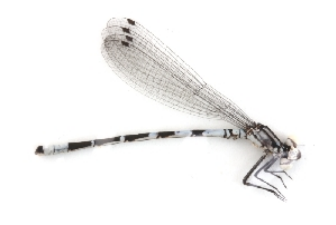

UAM:Ento:120418 [Lateral]
Enallagma annexum
Family: Coenagrionidae
BIN URI: BOLD:AAA2218

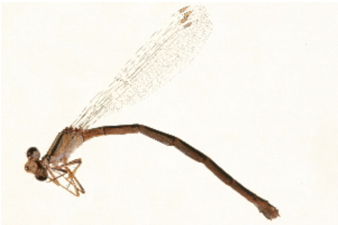

09BBEOD-0156 [Lateral]
Enallagma civile
Family: Coenagrionidae
BIN URI: BOLD:AAB3741

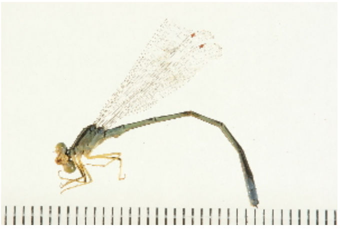

08OMSOD-0268 [Lateral]
Enallagma antennatum
Family: Coenagrionidae
BIN URI: BOLD:AAC0004

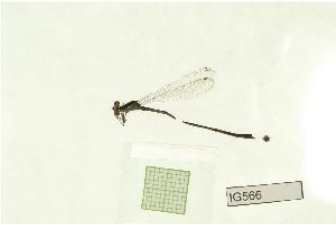

IG566 []
Enallagma exsulans
Family: Coenagrionidae
BIN URI: BOLD:AAI9242

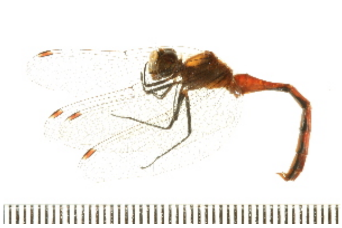

08BBODO-258 [Lateral]
Sympetrum obtrusum
Family: Libellulidae
BIN URI: BOLD:AAA5224

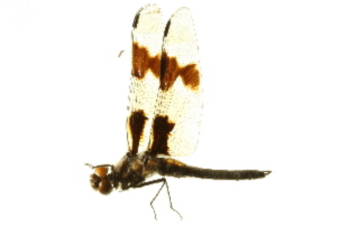

BIOUG02813-B04 [Lateral]
Libellula forensis
Family: Libellulidae
BIN URI: BOLD:AAD6885

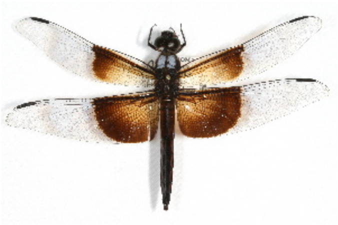

ENT-OUBS-222 [Dorsal]
Libellulidae
Family: Libellulidae
BIN URI: BOLD:ABX5734

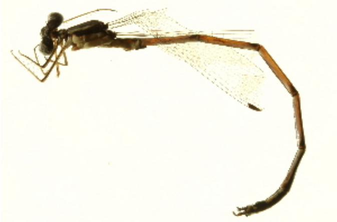

BIOUG02813-H07 [Lateral]
Lestes rectangularis
Family: Lestidae
BIN URI: BOLD:ACF4056

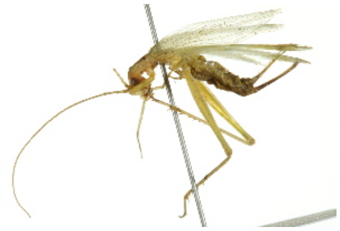

BIOUG02001-E08 [Lateral]
Oecanthus
Family: Gryllidae
BIN URI: BOLD:AAF9891

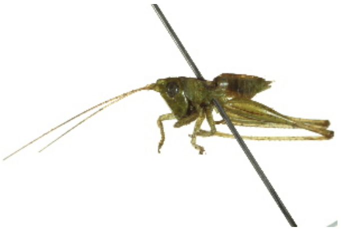

BIOUG02001-E02 [Lateral]
Conocephalus brevipennis
Family: Tettigoniidae
BIN URI: BOLD:AAK5385

IMAGE NOT AVAILABLE

BIOUG24020-G11
Conocephalus brevipennis
Family: Tettigoniidae

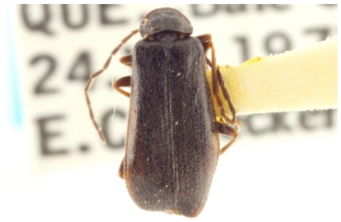

CNC COLEO 00153333 [Dorsal]
Rhagonycha mandibularis
Family: Cantharidae
BIN URI: BOLD:AAH0920

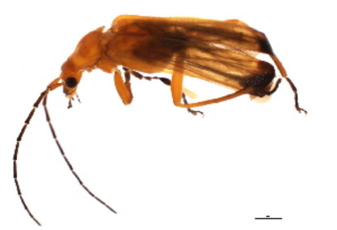

AY165676 [Lateral]
Rhagonycha fulva
Family: Cantharidae
BIN URI: BOLD:AAL1297

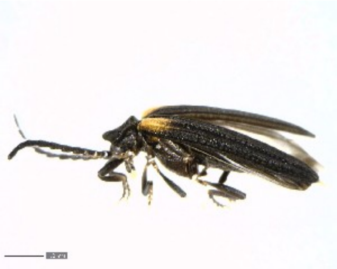

BIOUG03207-D10 [Lateral]
Lycidae
Family: Lycidae
BIN URI: BOLD:ACI6664

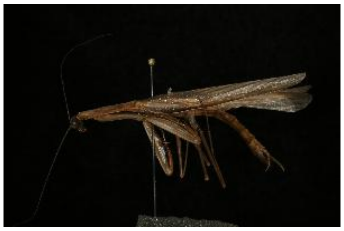

Uleth-DJ-136 [Lateral]
Mantodea

BIN URI: BOLD:AAF4833

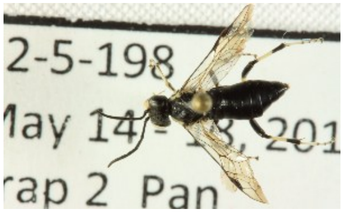

22-5-198 [Dorsal]
Macrophya flavolineata
Family: Tenthredinidae
BIN URI: BOLD:ABU8852

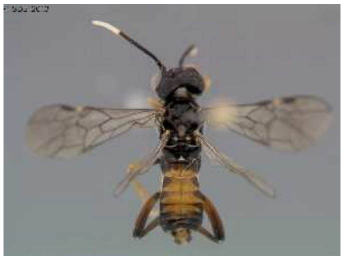

DEIGISHym15311 [Dorsal]
Taxonus terminalis
Family: Tenthredinidae
BIN URI: BOLD:AAU8702

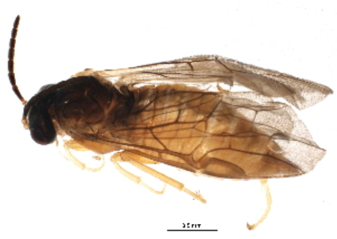

BIOUG08824-A01 [Lateral]
Caulocampus acericaulis
Family: Tenthredinidae
BIN URI: BOLD:ACJ9109

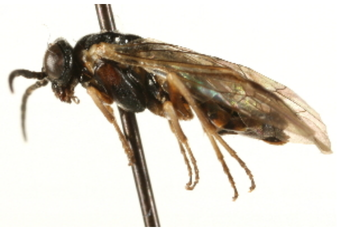

Hcmisc-143 [Lateral]
Periclista
Family: Tenthredinidae
BIN URI: BOLD:AAG3550

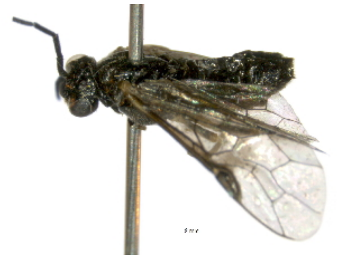

BIOUG07533-D03 [Lateral]
Priophorus compressicornis
Family: Tenthredinidae
BIN URI: BOLD:ACI7354

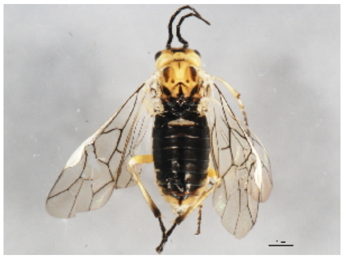

BIOUG01022-H10 [Dorsal]
Pachynematus albipennis
Family: Tenthredinidae
BIN URI: BOLD:AAN8130

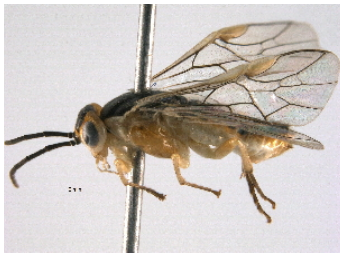

BIOUG08485-D05 [Lateral]
Pristiphora chlorea
Family: Tenthredinidae
BIN URI: BOLD:ACG2990

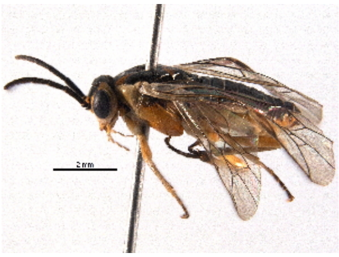

BIOUG11210-D12 [Lateral]
Pristiphora
Family: Tenthredinidae
BIN URI: BOLD:ACM9731

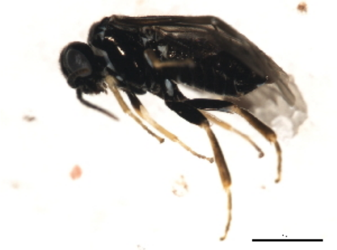

10BBHYM-0029 [Lateral]
Tenthredinidae
Family: Tenthredinidae
BIN URI: BOLD:AAN7643

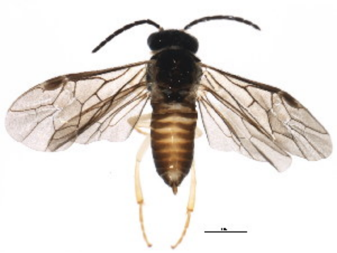

BIOUG00991-A10 [Dorsal]
Metallus lanceolatus
Family: Tenthredinidae
BIN URI: BOLD:AAP1085

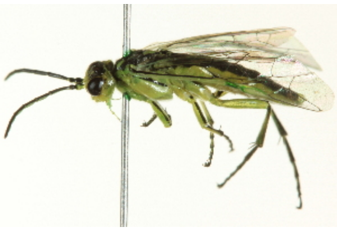

BIOUG02974-G07 [Lateral]
Rhogogaster
Family: Tenthredinidae
BIN URI: BOLD:ABZ3400

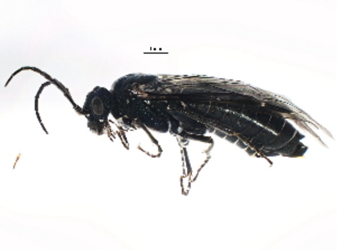

ASGLE-0442 [Lateral]
Hymenoptera

BIN URI: BOLD:AAG7773

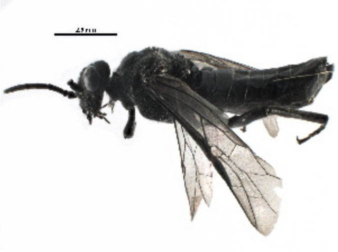

BIOUG22326-B10 [Lateral]
Dolerus nitens
Family: Tenthredinidae
BIN URI: BOLD:ACV5952

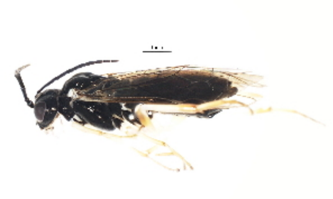

ASGLE-0396 [Lateral]
Ametastegia pallipes
Family: Tenthredinidae
BIN URI: BOLD:AAE5602

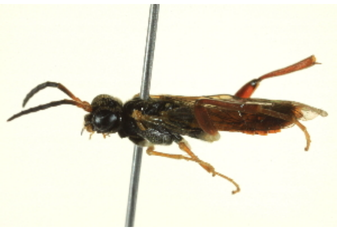

BIOUG03033-A12 [Lateral]
Tenthredinidae
Family: Tenthredinidae
BIN URI: BOLD:ACC7921

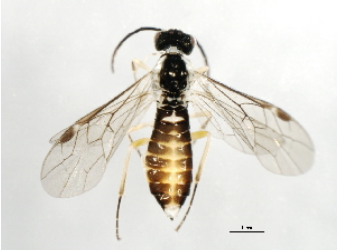

09BBEHY-1041 [Dorsal]
Ametastegia
Family: Tenthredinidae
BIN URI: BOLD:AAI4543

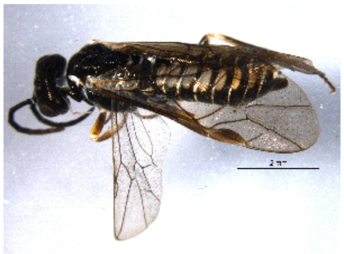

BIOUG03019-C03 [Dorsal]
Empria maculata
Family: Tenthredinidae
BIN URI: BOLD:ACC8799

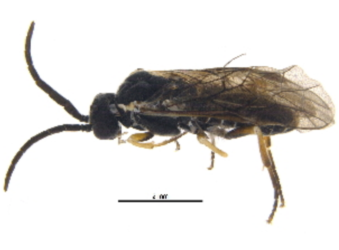

BIOUG22241-D01 [Lateral]
Empria nordica
Family: Tenthredinidae
BIN URI: BOLD:ACI4328

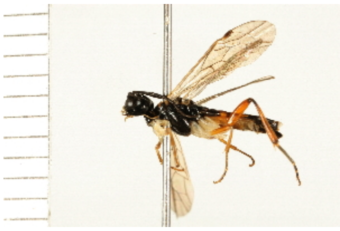

HYMN 0141.02 [Lateral]
Taxonus
Family: Tenthredinidae
BIN URI: BOLD:AAG7788

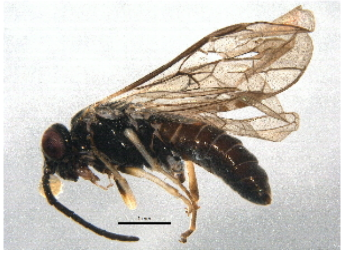

BIOUG10280-F01 [Lateral]
Monophadnus
Family: Tenthredinidae
BIN URI: BOLD:ACK2140

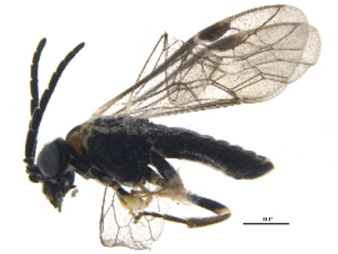

BIOUG22567-F11 [Lateral]
Tomostethus multicinctus
Family: Tenthredinidae
BIN URI: BOLD:ACV5036

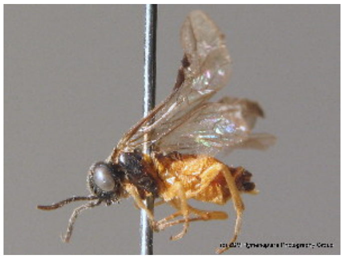

BC ZSM HYM 11702 [Lateral]
Halidamia affinis
Family: Tenthredinidae
BIN URI: BOLD:AAN7641

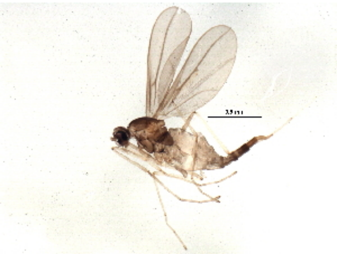

BIOUG08602-B03 [Lateral]
Cecidomyiidae
Family: Cecidomyiidae
BIN URI: BOLD:ACK1730

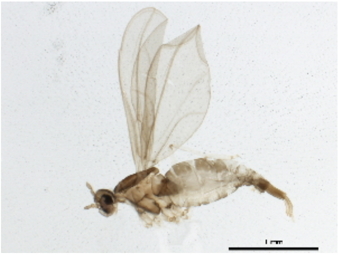

BIOUG02760-F06 [Lateral]
Cecidomyiidae
Family: Cecidomyiidae
BIN URI: BOLD:ACA7641

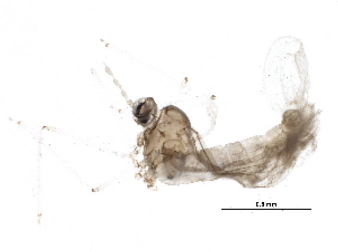

BIOUG22084-C07 [Lateral]
Cecidomyiidae
Family: Cecidomyiidae
BIN URI: BOLD:ACV4508

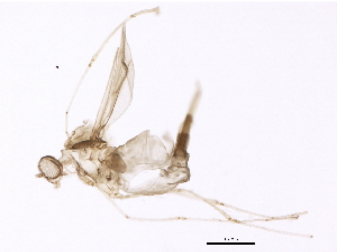

BIOUG01120-E10 [Lateral]
Cecidomyiidae
Family: Cecidomyiidae
BIN URI: BOLD:AAY6466

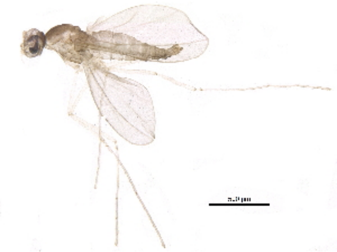

BIOUG23081-F04 [Lateral]
Cecidomyiidae
Family: Cecidomyiidae
BIN URI: BOLD:ACW0774

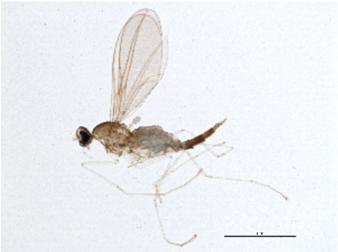

BIOUG02627-C08 [Lateral]
Cecidomyiidae
Family: Cecidomyiidae
BIN URI: BOLD:ABX9505

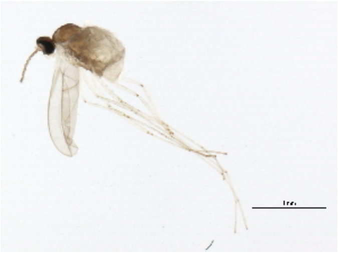

BIOUG01122-A05 [Lateral]
Cecidomyiidae
Family: Cecidomyiidae
BIN URI: BOLD:AAN5246

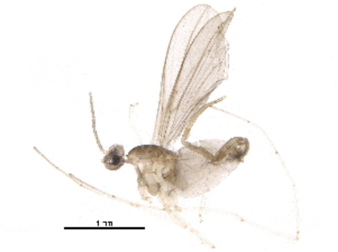

BIOUG23086-B04 [Lateral]
Cecidomyiidae
Family: Cecidomyiidae
BIN URI: BOLD:ACW1132

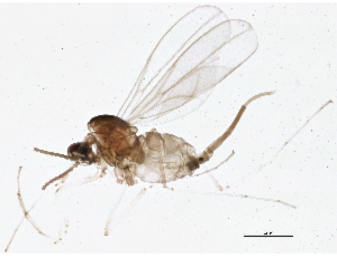

BIOUG06310-C07 [Lateral]
Cecidomyiidae
Family: Cecidomyiidae
BIN URI: BOLD:ACC8322

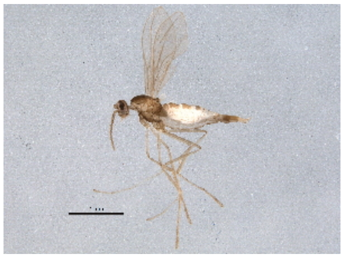

BIOUG05754-G08 [Lateral]
Cecidomyiidae
Family: Cecidomyiidae
BIN URI: BOLD:ACG3753

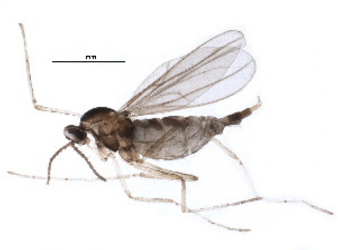

BIOUG22295-G04 [Lateral]
Cecidomyiidae
Family: Cecidomyiidae
BIN URI: BOLD:ACV3992

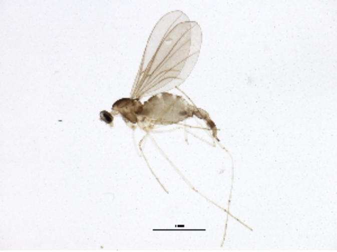

10PHMAL-3408 [Lateral]
Cecidomyiidae
Family: Cecidomyiidae
BIN URI: BOLD:AAU6478

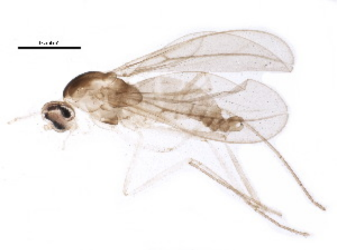

BIOUG22733-H09 [Lateral]
Cecidomyiidae
Family: Cecidomyiidae
BIN URI: BOLD:ACA7518

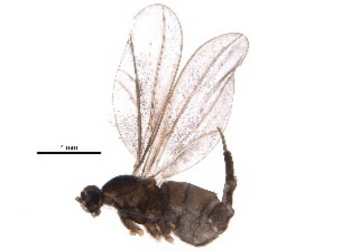

BIOUG22357-A09 [Lateral]
Cecidomyiidae
Family: Cecidomyiidae
BIN URI: BOLD:ACA8239

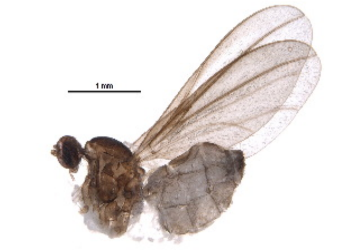

BIOUG22357-B03 [Lateral]
Cecidomyiidae
Family: Cecidomyiidae
BIN URI: BOLD:ACV4680

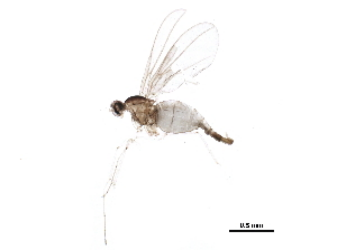

BIOUG23316-D08 [Lateral]
Cecidomyiidae
Family: Cecidomyiidae
BIN URI: BOLD:ACW0822

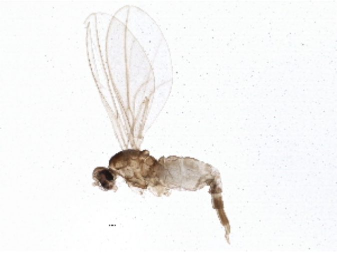

BIOUG05769-C04 [Lateral]
Cecidomyiidae
Family: Cecidomyiidae
BIN URI: BOLD:ABW6100

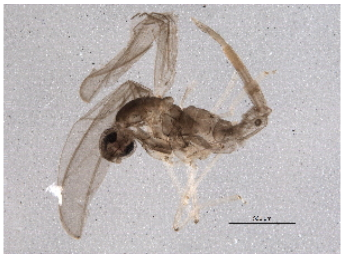

BIOUG09995-C07 [Lateral]
Cecidomyiidae
Family: Cecidomyiidae
BIN URI: BOLD:ACL3877

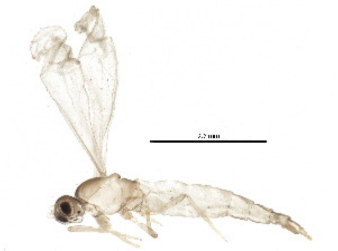

BIOUG19531-D03 [Lateral]
Cecidomyiidae
Family: Cecidomyiidae
BIN URI: BOLD:ABW1321

IMAGE NOT AVAILABLE

BIOUG23084-H11
Cecidomyiidae
Family: Cecidomyiidae
BIN URI: BOLD:ACL0151

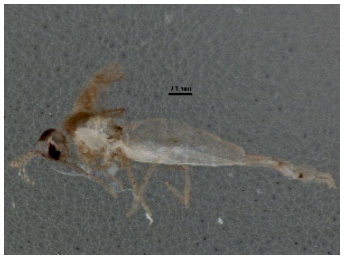

BIOUG03687-F06 [Lateral]
Cecidomyiidae
Family: Cecidomyiidae
BIN URI: BOLD:ACA6240

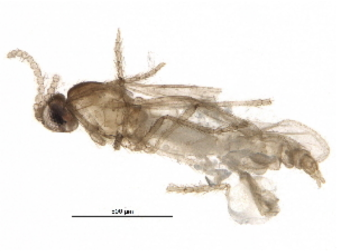

BIOUG09829-E02 [Lateral]
Cecidomyiidae
Family: Cecidomyiidae
BIN URI: BOLD:ACL6616

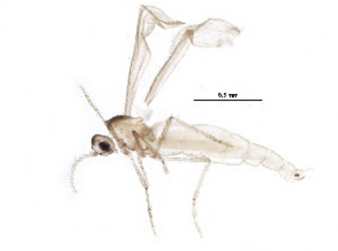

BIOUG22467-B05 [Lateral]
Cecidomyiidae
Family: Cecidomyiidae
BIN URI: BOLD:ACC7243

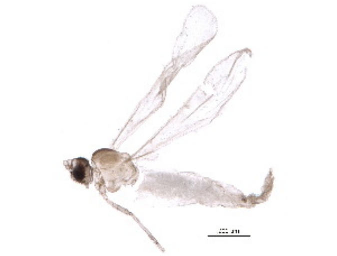

BIOUG22353-F05 [Lateral]
Cecidomyiidae
Family: Cecidomyiidae
BIN URI: BOLD:ACM1165

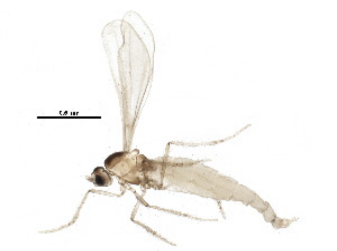

BIOUG22295-H07 [Lateral]
Cecidomyiidae
Family: Cecidomyiidae
BIN URI: BOLD:ACM1549

IMAGE NOT AVAILABLE

BIOUG22456-H04
Cecidomyiidae
Family: Cecidomyiidae

IMAGE NOT AVAILABLE

BIOUG22351-G05
Cecidomyiidae
Family: Cecidomyiidae

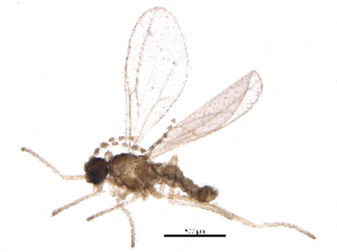

BIOUG07902-A02 [Lateral]
Cecidomyiidae
Family: Cecidomyiidae
BIN URI: BOLD:ACJ0206

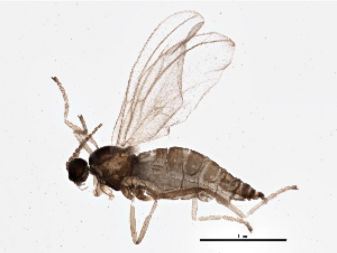

BIOUG05503-B09 [Lateral]
Cecidomyiidae
Family: Cecidomyiidae
BIN URI: BOLD:AAN5268

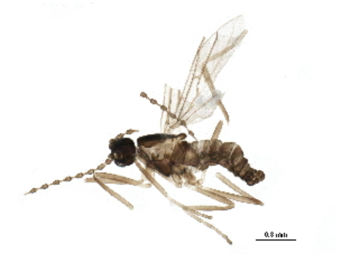

BIOUG22235-H11 [Lateral]
Cecidomyiidae
Family: Cecidomyiidae
BIN URI: BOLD:ACF7379

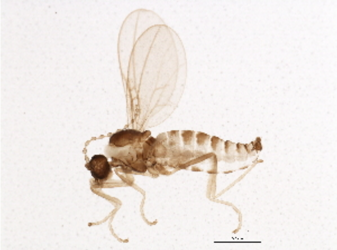

BIOUG01620-C06 [Lateral]
Cecidomyiidae
Family: Cecidomyiidae
BIN URI: BOLD:ABV1222

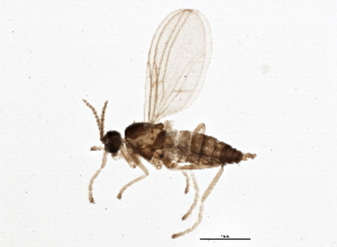

BIOUG05570-C12 [Lateral]
Cecidomyiidae
Family: Cecidomyiidae
BIN URI: BOLD:ACF7633

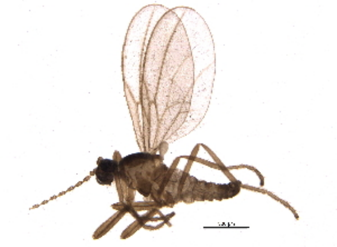

BIOUG10178-F02 [Lateral]
Cecidomyiidae
Family: Cecidomyiidae
BIN URI: BOLD:AAH3630

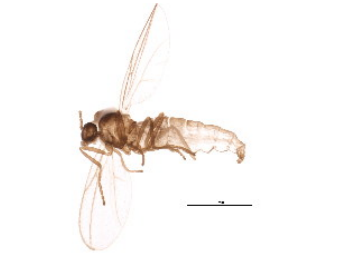

BIOUG01658-B09 [Lateral]
Cecidomyiidae
Family: Cecidomyiidae
BIN URI: BOLD:ABA1220

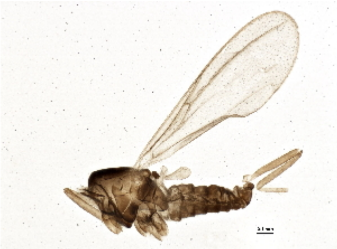

BIOUG07577-D11 [Lateral]
Cecidomyiidae
Family: Cecidomyiidae
BIN URI: BOLD:ABA0844

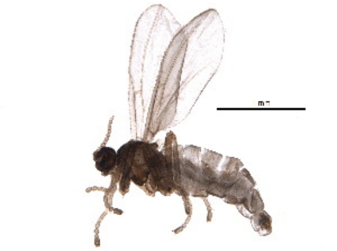

BIOUG22351-C05 [Lateral]
Cecidomyiidae
Family: Cecidomyiidae
BIN URI: BOLD:ACV3674

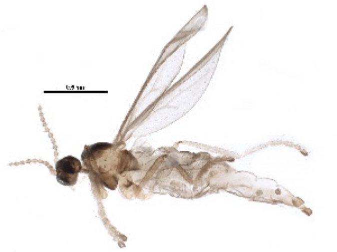

BIOUG22295-G06 [Lateral]
Cecidomyiidae
Family: Cecidomyiidae
BIN URI: BOLD:AAP5342

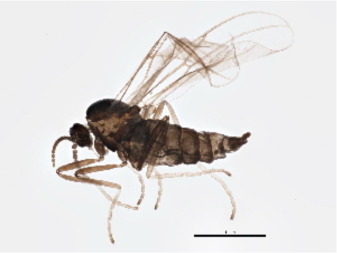

BIOUG05503-B03 [Lateral]
Cecidomyiidae
Family: Cecidomyiidae
BIN URI: BOLD:ABA1223

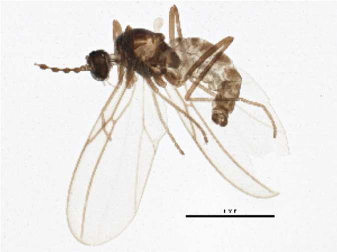

BIOUG02726-A01 [Lateral]
Cecidomyiidae
Family: Cecidomyiidae
BIN URI: BOLD:ABX8104

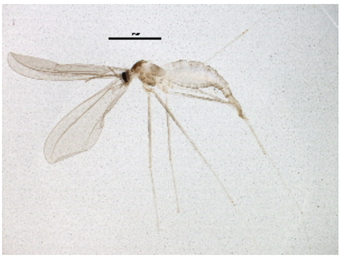

BIOUG03444-C09 [Lateral]
Cecidomyiidae
Family: Cecidomyiidae
BIN URI: BOLD:ABA0852

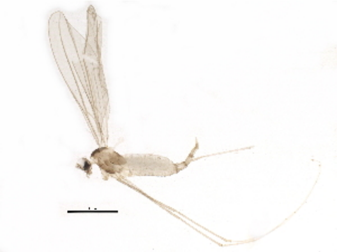

BIOUG08723-D02 [Lateral]
Cecidomyiidae
Family: Cecidomyiidae
BIN URI: BOLD:ACK3141

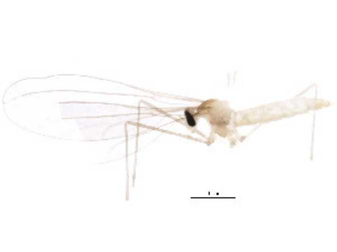

BIOUG01360-E08 [Lateral]
Cecidomyiidae
Family: Cecidomyiidae
BIN URI: BOLD:AAM6097

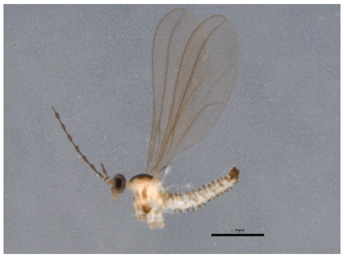

BIOUG03658-C05 [Lateral]
Cecidomyiidae
Family: Cecidomyiidae
BIN URI: BOLD:AAV5688

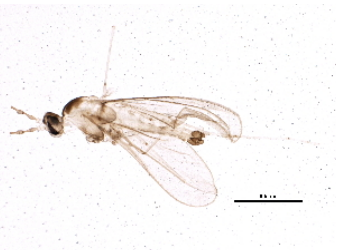

BIOUG02510-C02 [Lateral]
Cecidomyiidae
Family: Cecidomyiidae
BIN URI: BOLD:ABX9970

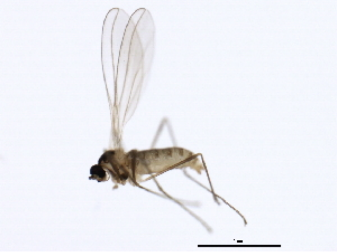

10BBCDIP-1328 [Lateral]
Cecidomyiidae
Family: Cecidomyiidae
BIN URI: BOLD:AAV5630

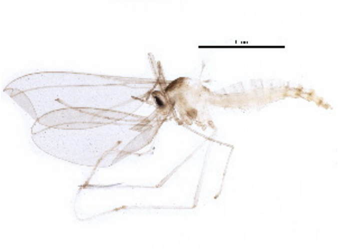

BIOUG22415-E10 [Lateral]
Cecidomyiidae
Family: Cecidomyiidae
BIN URI: BOLD:ACV2603

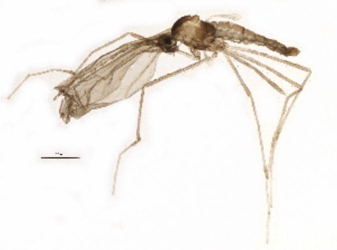

BIOUG06796-A10 [Lateral]
Cecidomyiidae
Family: Cecidomyiidae
BIN URI: BOLD:ACI6861

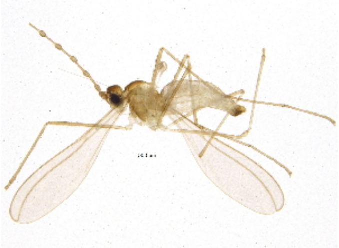

BIOUG02627-B08 [Lateral]
Cecidomyiidae
Family: Cecidomyiidae
BIN URI: BOLD:AAY6407

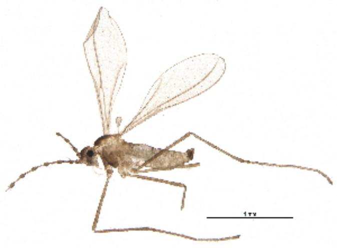

BIOUG08554-H09 [Lateral]
Cecidomyiidae
Family: Cecidomyiidae
BIN URI: BOLD:ACK1358

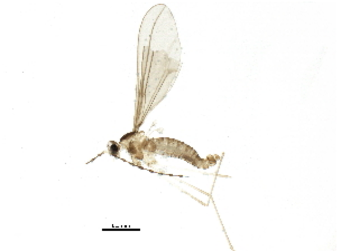

BIOUG22865-D09 [Lateral]
Cecidomyiidae
Family: Cecidomyiidae
BIN URI: BOLD:ACV5166

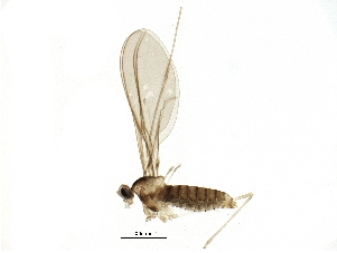

BIOUG22865-A08 [Lateral]
Cecidomyiidae
Family: Cecidomyiidae
BIN URI: BOLD:ACV5434

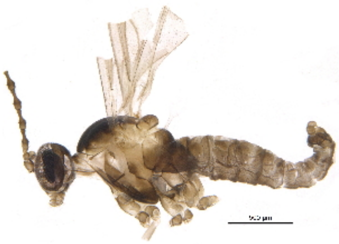

BIOUG22239-H08 [Lateral]
Cecidomyiidae
Family: Cecidomyiidae
BIN URI: BOLD:ACV2679

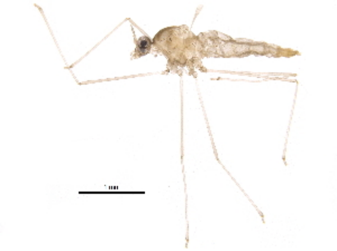

BIOUG22838-C07 [Lateral]
Cecidomyiidae
Family: Cecidomyiidae
BIN URI: BOLD:ACV9326

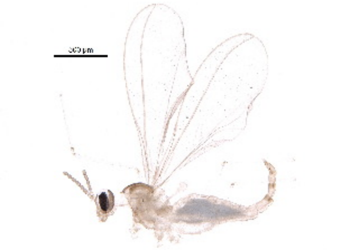

BIOUG22454-F09 [Lateral]
Cecidomyiidae
Family: Cecidomyiidae
BIN URI: BOLD:ABV1487

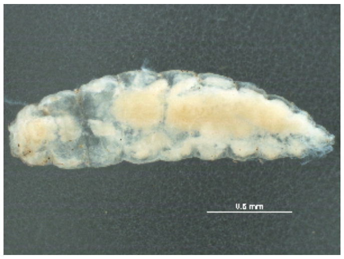

BIOUG21887-F05 [Larva]
Cecidomyiidae
Family: Cecidomyiidae
BIN URI: BOLD:ACV4255

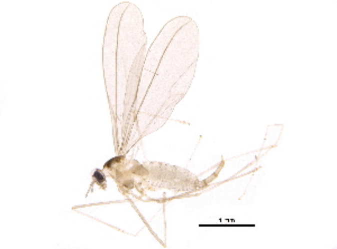

BIOUG22329-B06 [Lateral]
Cecidomyiidae
Family: Cecidomyiidae
BIN URI: BOLD:ABW0442

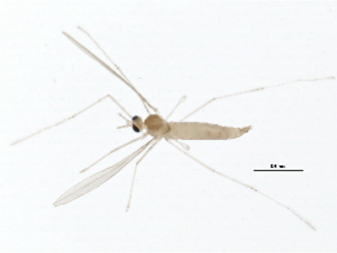

08TTML-2477 [Dorsal]
Cecidomyiidae
Family: Cecidomyiidae
BIN URI: BOLD:AAN5249

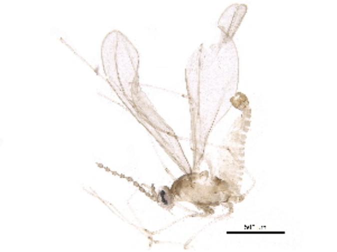

BIOUG23314-G12 [Lateral]
Cecidomyiidae
Family: Cecidomyiidae
BIN URI: BOLD:ACW0974

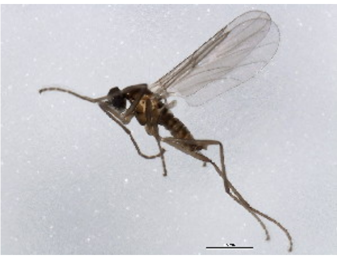

10BBCDIP-0429 [Lateral]
Cecidomyiidae
Family: Cecidomyiidae
BIN URI: BOLD:AAP6849

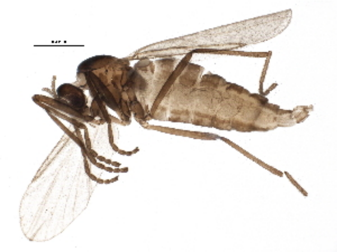

BIOUG17416-B10 [Lateral]
Cecidomyiidae
Family: Cecidomyiidae
BIN URI: BOLD:AAU6594

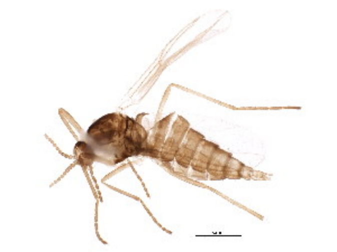

BIOUG01610-E02 [Lateral]
Cecidomyiidae
Family: Cecidomyiidae
BIN URI: BOLD:ABV1329

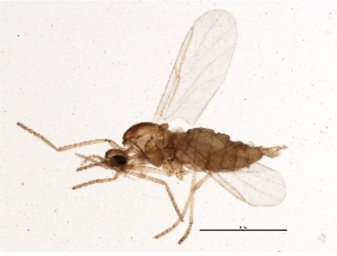

BIOUG02583-G03 [Lateral]
Cecidomyiidae
Family: Cecidomyiidae
BIN URI: BOLD:ACA1234

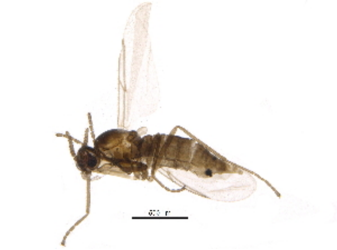

BIOUG23079-C12 [Lateral]
Cecidomyiidae
Family: Cecidomyiidae
BIN URI: BOLD:AAG6460

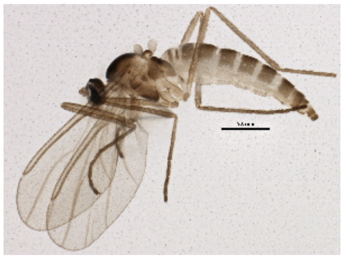

BIOUG05625-G04 [Lateral]
Cecidomyiidae
Family: Cecidomyiidae
BIN URI: BOLD:AAP9021

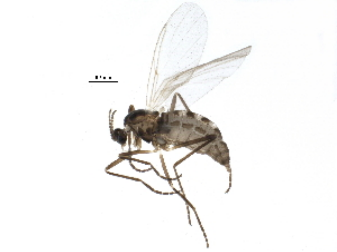

BIOUG08741-E12 [Lateral]
Cecidomyiidae
Family: Cecidomyiidae
BIN URI: BOLD:ACK3431

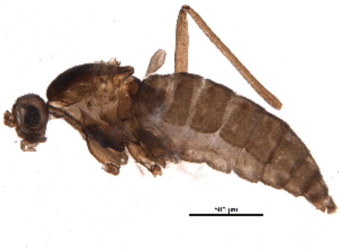

BIOUG14694-G09 [Lateral]
Cecidomyiidae
Family: Cecidomyiidae
BIN URI: BOLD:ACP5423

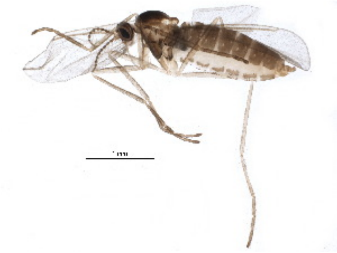

BIOUG22463-G05 [Lateral]
Cecidomyiidae
Family: Cecidomyiidae
BIN URI: BOLD:ACV2861

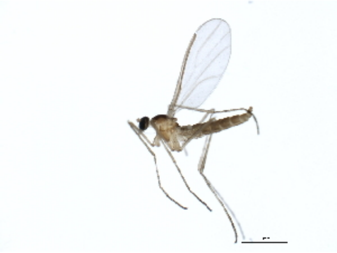

09BBDIP-1765 [Lateral]
Cecidomyiidae
Family: Cecidomyiidae
BIN URI: BOLD:AAH3671

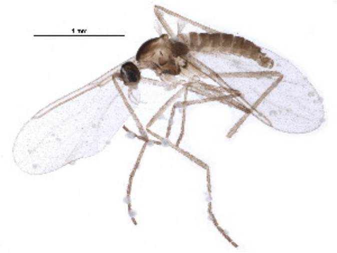

BIOUG22295-G02 [Lateral]
Cecidomyiidae
Family: Cecidomyiidae
BIN URI: BOLD:ABV0480

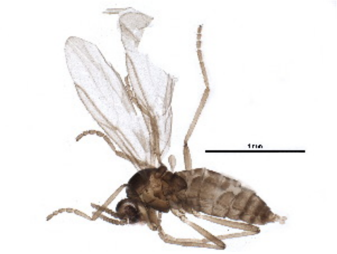

BIOUG22295-G05 [Lateral]
Cecidomyiidae
Family: Cecidomyiidae
BIN URI: BOLD:ACV3989

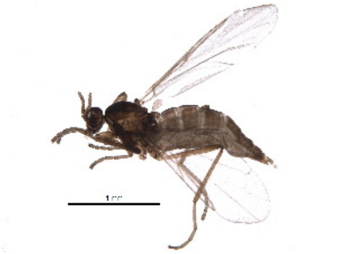

BIOUG22168-B05 [Lateral]
Cecidomyiidae
Family: Cecidomyiidae
BIN URI: BOLD:ABV1474

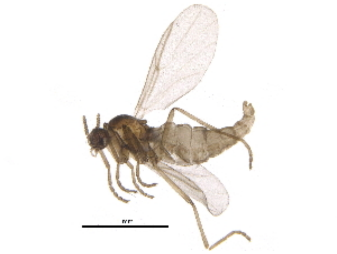

BIOUG22330-F12 [Lateral]
Cecidomyiidae
Family: Cecidomyiidae
BIN URI: BOLD:ACV4250

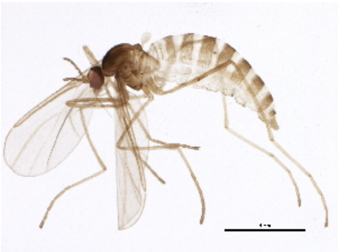

BIOUG01662-H02 [Lateral]
Cecidomyiidae
Family: Cecidomyiidae
BIN URI: BOLD:ABA6448

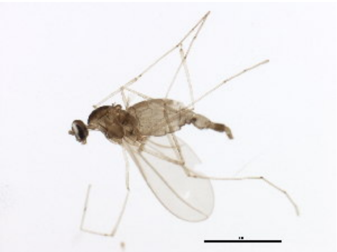

10PHMAL-3426 [Lateral]
Cecidomyiidae
Family: Cecidomyiidae
BIN URI: BOLD:AAU6483

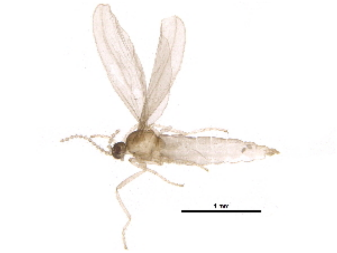

BIOUG22328-G06 [Lateral]
Cecidomyiidae
Family: Cecidomyiidae
BIN URI: BOLD:ACV2942

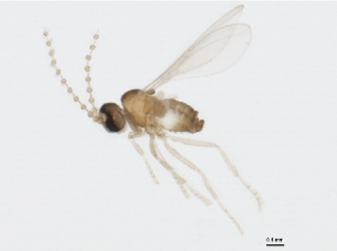

BIOUG01409-D03 [Lateral]
Cecidomyiidae
Family: Cecidomyiidae
BIN URI: BOLD:AAN5215

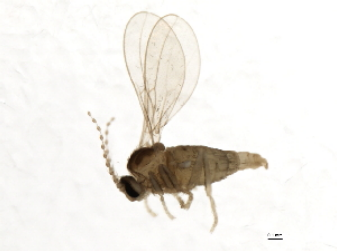

09BBDIP-1932 [Lateral]
Cecidomyiidae
Family: Cecidomyiidae
BIN URI: BOLD:AAM6043

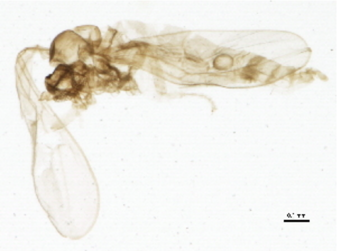

BIOUG01449-F06 [Lateral]
Cecidomyiidae
Family: Cecidomyiidae
BIN URI: BOLD:ABV0479

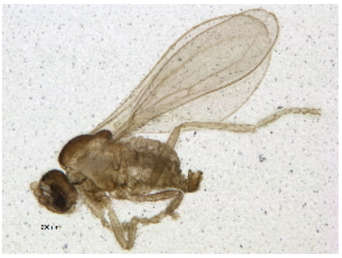

BIOUG04356-D01 [Lateral]
Cecidomyiidae
Family: Cecidomyiidae
BIN URI: BOLD:ABW6101

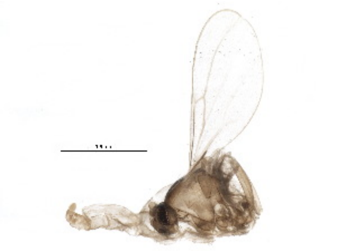

BIOUG20932-G05 [Lateral]
Diptera

BIN URI: BOLD:ACU7441

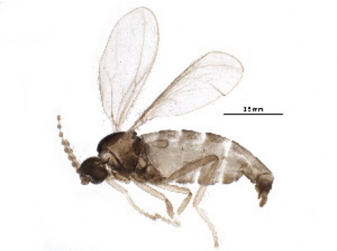

BIOUG22417-E04 [Lateral]
Cecidomyiidae
Family: Cecidomyiidae
BIN URI: BOLD:ACV4544

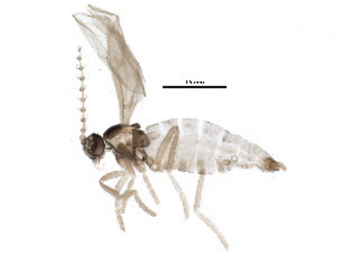

BIOUG22291-D05 [Lateral]
Cecidomyiidae
Family: Cecidomyiidae
BIN URI: BOLD:AAQ0640

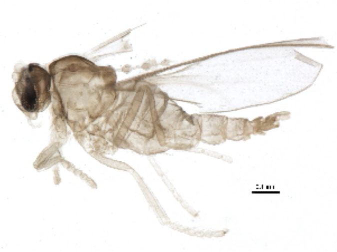

BIOUG15597-E12 [Lateral]
Cecidomyiidae
Family: Cecidomyiidae
BIN URI: BOLD:ACF7688

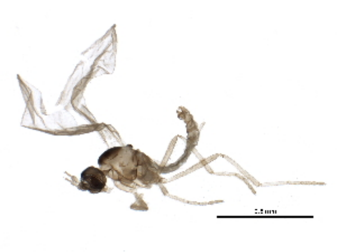

BIOUG21892-D08 [Lateral]
Cecidomyiidae
Family: Cecidomyiidae
BIN URI: BOLD:ACV4891

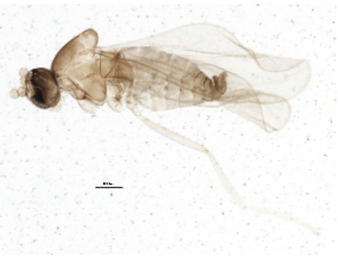

BIOUG02667-B06 [Lateral]
Cecidomyiidae
Family: Cecidomyiidae
BIN URI: BOLD:AAV5559

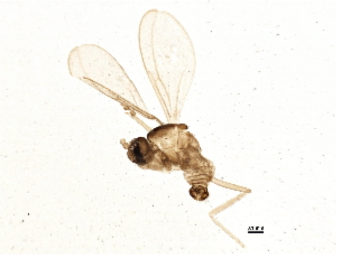

BIOUG03978-F05 [Lateral]
Cecidomyiidae
Family: Cecidomyiidae
BIN URI: BOLD:ACB9780

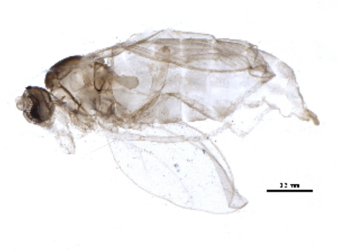

BIOUG11816-C10 [Lateral]
Cecidomyiidae
Family: Cecidomyiidae
BIN URI: BOLD:ACM6249

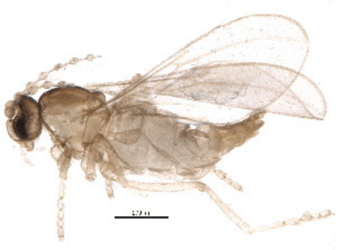

BIOUG20772-H10 [Lateral]
Diptera

BIN URI: BOLD:ACQ9861

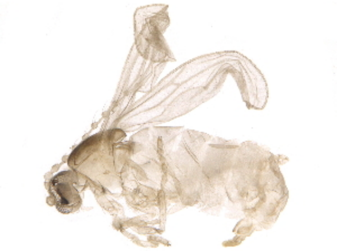

BIOUG23076-F12 [Lateral]
Cecidomyiidae
Family: Cecidomyiidae
BIN URI: BOLD:AAY6441

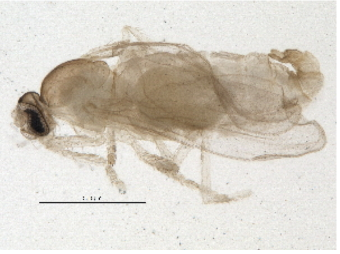

BIOUG03591-E03 [Lateral]
Cecidomyiidae
Family: Cecidomyiidae
BIN URI: BOLD:ABV9284

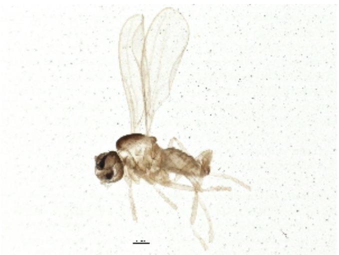

BIOUG03732-E05 [Lateral]
Cecidomyiidae
Family: Cecidomyiidae
BIN URI: BOLD:AAV5762

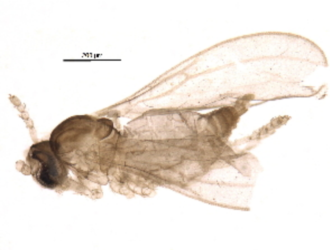

BIOUG11144-E08 [Lateral]
Cecidomyiidae
Family: Cecidomyiidae
BIN URI: BOLD:ACA4778

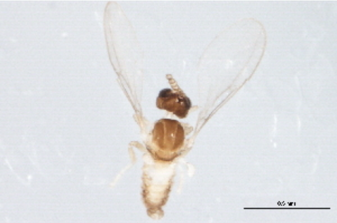

08TTML-1165 [Dorsal]
Cecidomyiidae
Family: Cecidomyiidae
BIN URI: BOLD:AAN5228

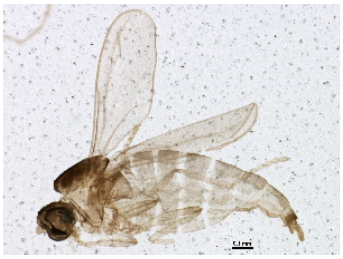

BIOUG02871-C09 [Lateral]
Cecidomyiidae
Family: Cecidomyiidae
BIN URI: BOLD:AAN5250

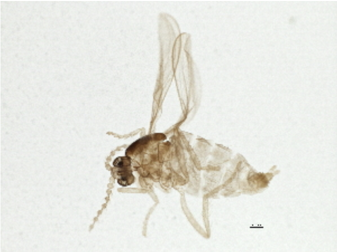

BIOUG03327-E04 [Lateral]
Cecidomyiidae
Family: Cecidomyiidae
BIN URI: BOLD:ABX8601

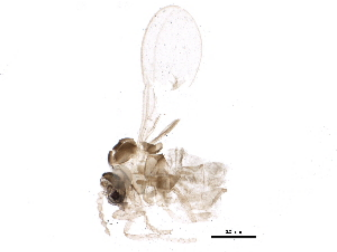

BIOUG12128-C05 [Lateral]
Cecidomyiidae
Family: Cecidomyiidae
BIN URI: BOLD:ACL6620

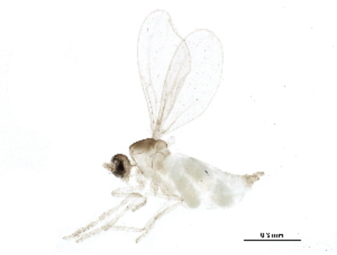

BIOUG20736-H03 [Lateral]
Diptera

BIN URI: BOLD:ACU3942

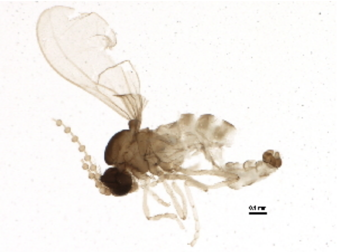

BIOUG01131-B11 [Lateral]
Cecidomyiidae
Family: Cecidomyiidae
BIN URI: BOLD:AAY6443

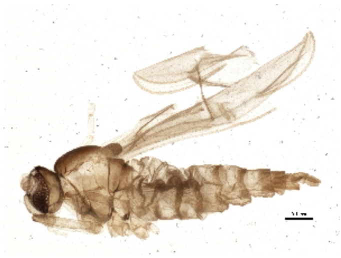

BIOUG02662-H01 [Lateral]
Cecidomyiidae
Family: Cecidomyiidae
BIN URI: BOLD:AAZ0300

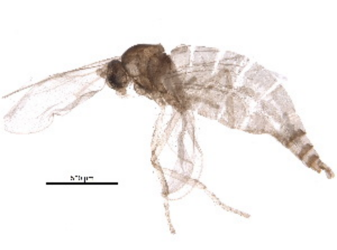

BIOUG23075-E06 [Lateral]
Cecidomyiidae
Family: Cecidomyiidae
BIN URI: BOLD:ACV5763

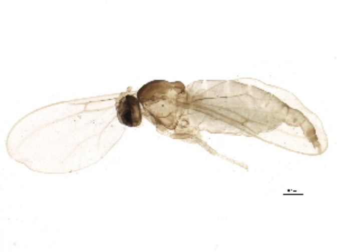

BIOUG01489-B04 [Lateral]
Cecidomyiidae
Family: Cecidomyiidae
BIN URI: BOLD:AAU6607

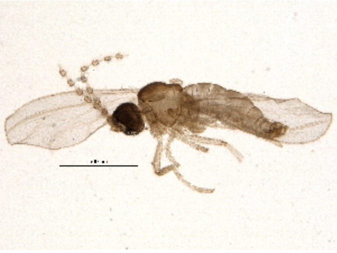

BIOUG03591-B10 [Lateral]
Cecidomyiidae
Family: Cecidomyiidae
BIN URI: BOLD:ACC7540

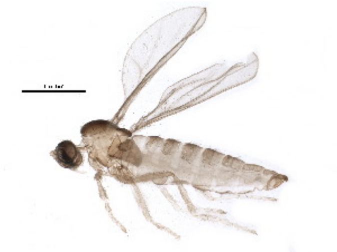

BIOUG22452-F10 [Lateral]
Cecidomyiidae
Family: Cecidomyiidae
BIN URI: BOLD:ABX9178

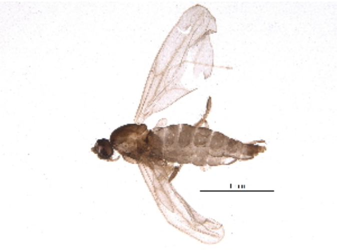

BIOUG11738-D02 [Lateral]
Cecidomyiidae
Family: Cecidomyiidae
BIN URI: BOLD:ACM6155

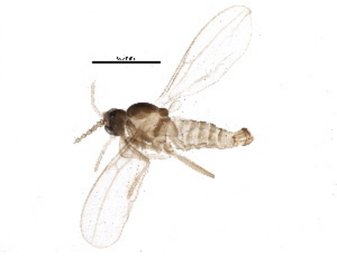

BIOUG19538-A08 [Lateral]
Cecidomyiidae
Family: Cecidomyiidae
BIN URI: BOLD:ACT3394

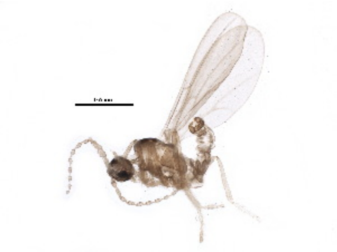

BIOUG22452-G04 [Lateral]
Cecidomyiidae
Family: Cecidomyiidae
BIN URI: BOLD:ACV5392

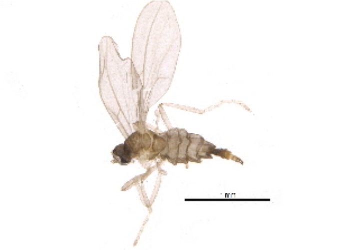

BIOUG22328-A11 [Lateral]
Cecidomyiidae
Family: Cecidomyiidae
BIN URI: BOLD:ACA8939

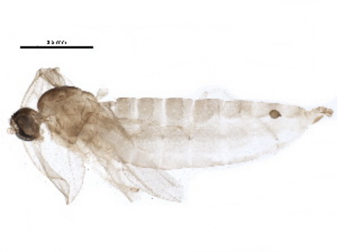

BIOUG22467-H09 [Lateral]
Cecidomyiidae
Family: Cecidomyiidae
BIN URI: BOLD:ACV3777

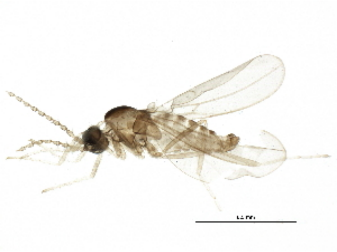

BIOUG22459-E07 [Lateral]
Cecidomyiidae
Family: Cecidomyiidae
BIN URI: BOLD:AAN5229

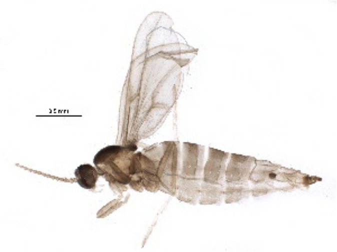

BIOUG22716-C01 [Lateral]
Cecidomyiidae
Family: Cecidomyiidae
BIN URI: BOLD:ACV5162

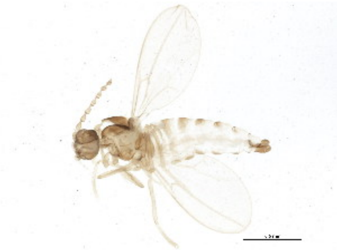

BIOUG01696-C02 [Lateral]
Cecidomyiidae
Family: Cecidomyiidae
BIN URI: BOLD:ABV1292

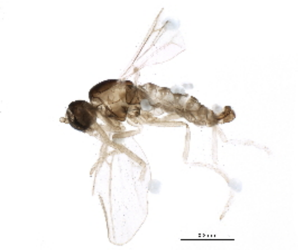

BIOUG22084-H01 [Lateral]
Cecidomyiidae
Family: Cecidomyiidae
BIN URI: BOLD:ABX8236

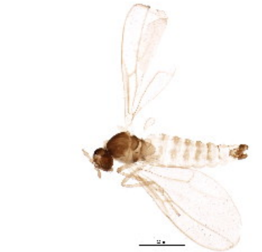

BIOUG01450-H07 [Lateral]
Cecidomyiidae
Family: Cecidomyiidae
BIN URI: BOLD:ACF0765

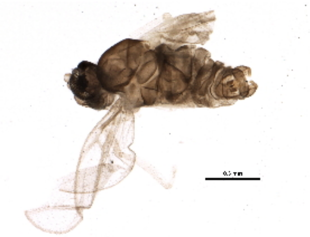

BIOUG16051-E08 [Lateral]
Cecidomyiidae
Family: Cecidomyiidae
BIN URI: BOLD:ACK1651

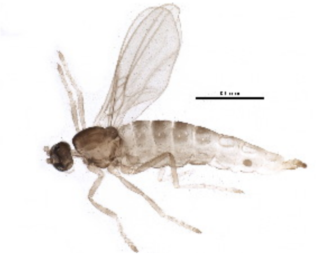

BIOUG22721-B11 [Lateral]
Cecidomyiidae
Family: Cecidomyiidae
BIN URI: BOLD:ACV5841

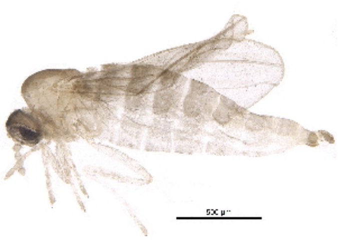

BIOUG23086-G08 [Lateral]
Cecidomyiidae
Family: Cecidomyiidae
BIN URI: BOLD:ACW1120

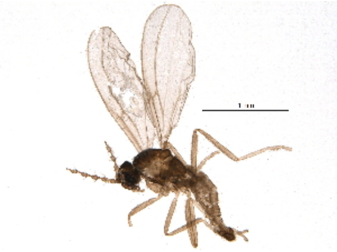

BIOUG08481-C04 [Lateral]
Cecidomyiidae
Family: Cecidomyiidae
BIN URI: BOLD:ACJ9045

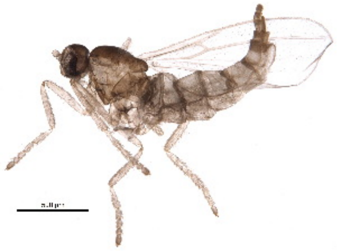

BIOUG22352-C05 [Lateral]
Cecidomyiidae
Family: Cecidomyiidae
BIN URI: BOLD:ACV3604

IMAGE NOT AVAILABLE

BIOUG23316-B10
Cecidomyiidae
Family: Cecidomyiidae

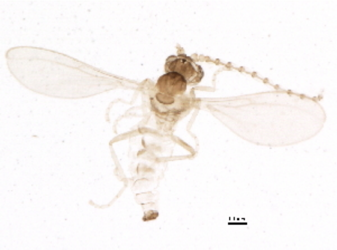

BIOUG01667-A02 [Dorsal]
Cecidomyiidae
Family: Cecidomyiidae
BIN URI: BOLD:ABA6489

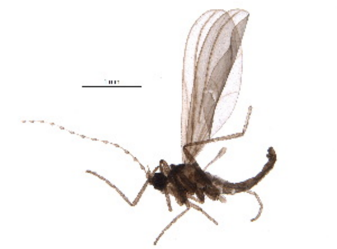

BIOUG22353-G01 [Lateral]
Cecidomyiidae
Family: Cecidomyiidae
BIN URI: BOLD:AAP5343

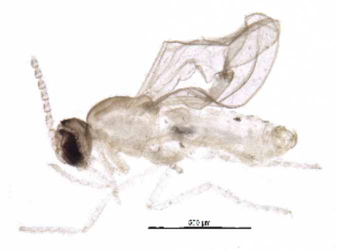

BIOUG09483-D12 [Lateral]
Cecidomyiidae
Family: Cecidomyiidae
BIN URI: BOLD:ACK2532

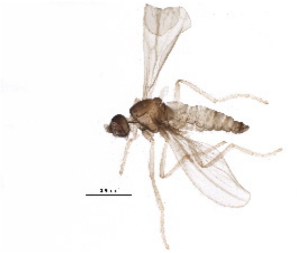

BIOUG19792-F09 [Lateral]
Cecidomyiidae
Family: Cecidomyiidae
BIN URI: BOLD:ACS9522

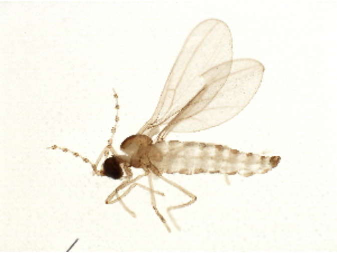

BIOUG01589-G09 [Lateral]
Cecidomyiidae
Family: Cecidomyiidae
BIN URI: BOLD:AAN5204

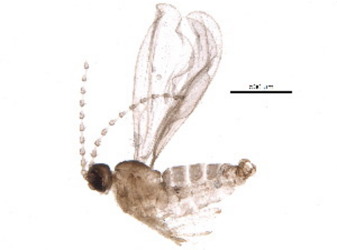

BIOUG23075-F12 [Lateral]
Cecidomyiidae
Family: Cecidomyiidae
BIN URI: BOLD:ACV5281

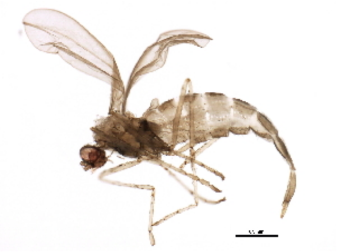

BIOUG01123-D03 [Lateral]
Cecidomyiidae
Family: Cecidomyiidae
BIN URI: BOLD:AAY6383

IMAGE NOT AVAILABLE

BIOUG23188-G07
Cecidomyiidae
Family: Cecidomyiidae

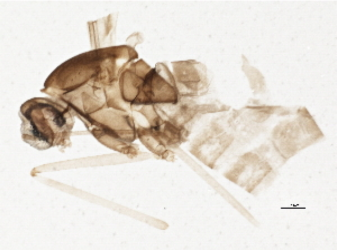

BIOUG02853-A02 [Lateral]
Cecidomyiidae
Family: Cecidomyiidae
BIN URI: BOLD:ACB1654

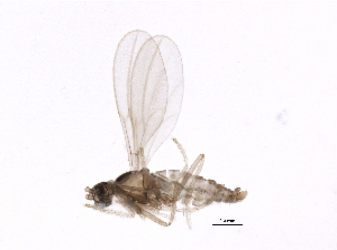

BIOUG16095-H05 [Lateral]
Cecidomyiidae
Family: Cecidomyiidae
BIN URI: BOLD:ACK3803

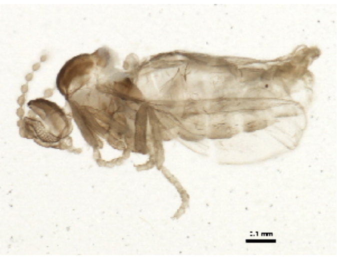

BIOUG01128-D04 [Lateral]
Cecidomyiidae
Family: Cecidomyiidae
BIN URI: BOLD:AAV5796

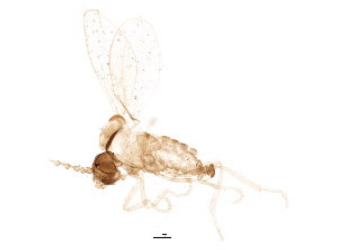

BIOUG01613-E04 [Lateral]
Cecidomyiidae
Family: Cecidomyiidae
BIN URI: BOLD:ABV1310

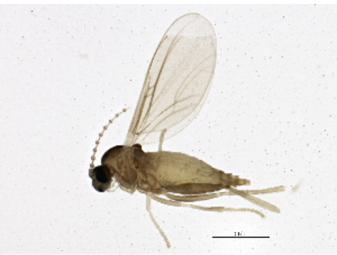

BIOUG02006-B04 [Lateral]
Cecidomyiidae
Family: Cecidomyiidae
BIN URI: BOLD:ACB3033

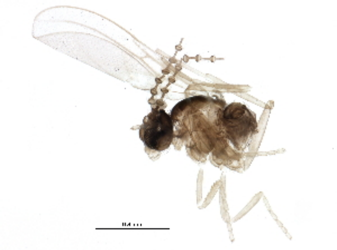

BIOUG22366-F03 [Lateral]
Cecidomyiidae
Family: Cecidomyiidae
BIN URI: BOLD:ACV3619

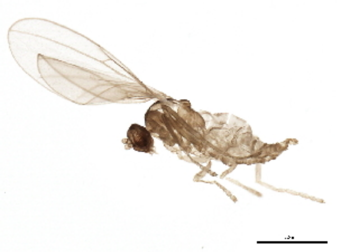

10PHMAL-1611 [Lateral]
Cecidomyiidae
Family: Cecidomyiidae
BIN URI: BOLD:AAN5283

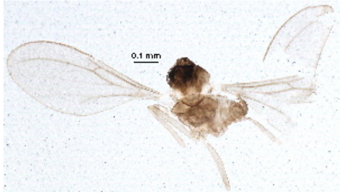

BIOUG06993-C10 [Lateral]
Cecidomyiidae
Family: Cecidomyiidae
BIN URI: BOLD:ACI6072

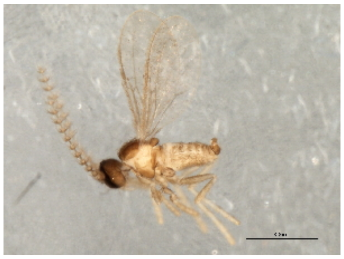

08TTML-1674 [Lateral]
Cecidomyiidae
Family: Cecidomyiidae
BIN URI: BOLD:AAN5259

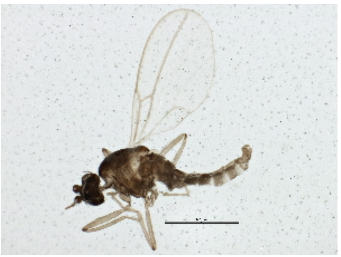

BIOUG03440-B08 [Lateral]
Cecidomyiidae
Family: Cecidomyiidae
BIN URI: BOLD:ABV1330

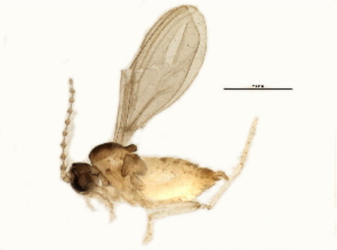

BIOUG06926-H09 [Lateral]
Cecidomyiidae
Family: Cecidomyiidae
BIN URI: BOLD:ACI5626

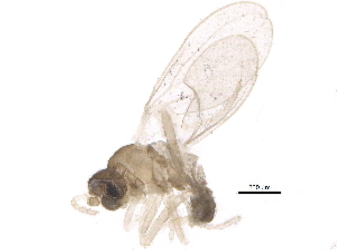

BIOUG23308-D03 [Lateral]
Cecidomyiidae
Family: Cecidomyiidae
BIN URI: BOLD:ACW1306

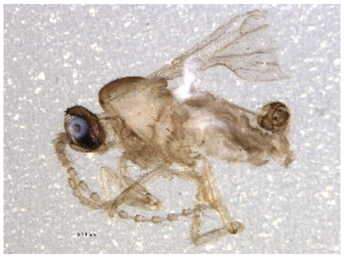

BIOUG04313-G06 [Lateral]
Cecidomyiidae
Family: Cecidomyiidae
BIN URI: BOLD:ACC8700

IMAGE NOT AVAILABLE

BIOUG22299-D11
Cecidomyiidae
Family: Cecidomyiidae

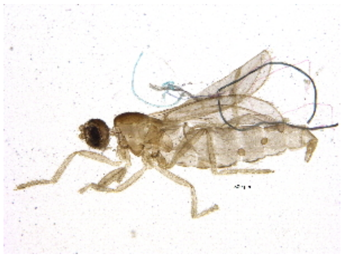

BIOUG03733-G11 [Lateral]
Cecidomyiidae
Family: Cecidomyiidae
BIN URI: BOLD:ACC9182

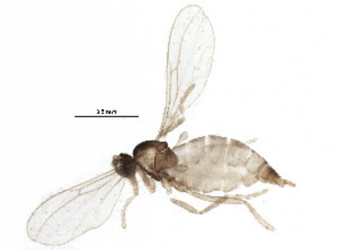

BIOUG22291-E10 [Lateral]
Cecidomyiidae
Family: Cecidomyiidae
BIN URI: BOLD:AAN5267

IMAGE NOT AVAILABLE

BIOUG22463-E04
Cecidomyiidae
Family: Cecidomyiidae

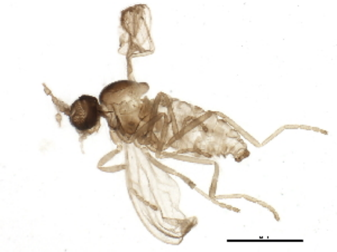

BIOUG01117-H04 [Lateral]
Cecidomyiidae
Family: Cecidomyiidae
BIN URI: BOLD:AAU6597

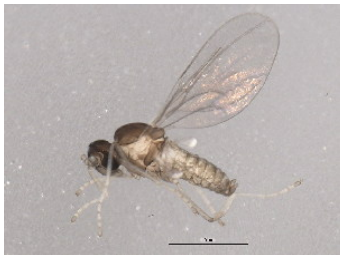

10JSROW-0688 [Lateral]
Cecidomyiidae
Family: Cecidomyiidae
BIN URI: BOLD:AAV5355

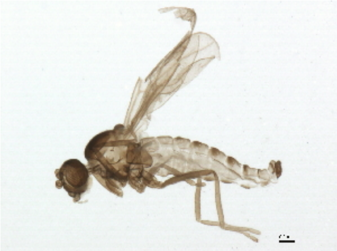

BIOUG01448-F08 [Lateral]
Cecidomyiidae
Family: Cecidomyiidae
BIN URI: BOLD:ABA7887

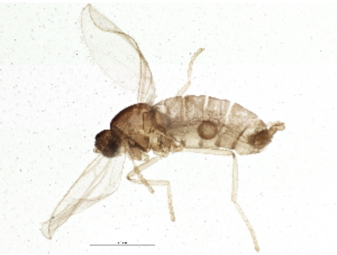

BIOUG04290-H04 [Lateral]
Cecidomyiidae
Family: Cecidomyiidae
BIN URI: BOLD:ACD0690

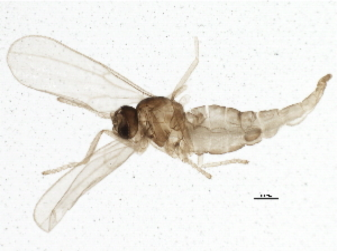

BIOUG02668-D12 [Lateral]
Cecidomyiidae
Family: Cecidomyiidae
BIN URI: BOLD:ABV9278

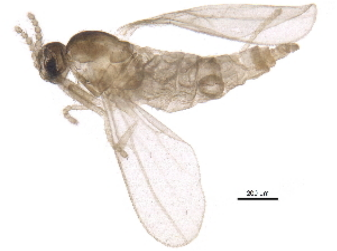

BIOUG23080-E09 [Lateral]
Cecidomyiidae
Family: Cecidomyiidae
BIN URI: BOLD:ACW0708

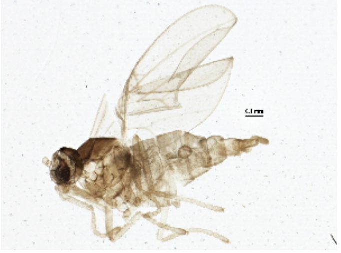

BIOUG03371-H07 [Lateral]
Cecidomyiidae
Family: Cecidomyiidae
BIN URI: BOLD:ACC8238

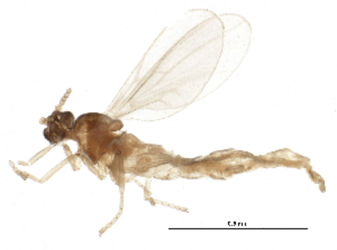

BIOUG23188-H01 [Lateral]
Cecidomyiidae
Family: Cecidomyiidae
BIN URI: BOLD:ACB3240

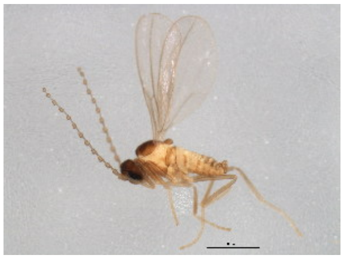

BIOUG00826-A11 [Lateral]
Cecidomyiidae
Family: Cecidomyiidae
BIN URI: BOLD:AAN5269

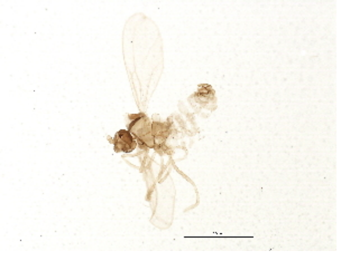

BIOUG01592-D08 [Lateral]
Cecidomyiidae
Family: Cecidomyiidae
BIN URI: BOLD:AAH2876

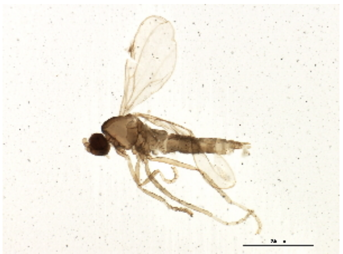

BIOUG01142-A07 [Lateral]
Cecidomyiidae
Family: Cecidomyiidae
BIN URI: BOLD:AAU6598

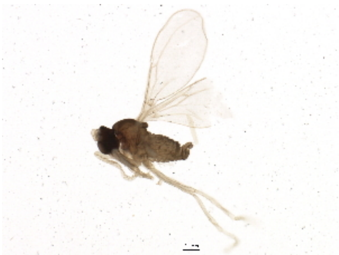

BIOUG01125-A05 [Lateral]
Cecidomyiidae
Family: Cecidomyiidae
BIN URI: BOLD:AAV5788

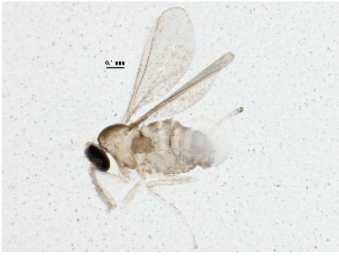

BIOUG03440-A05 [Lateral]
Cecidomyiidae
Family: Cecidomyiidae
BIN URI: BOLD:ACC8554

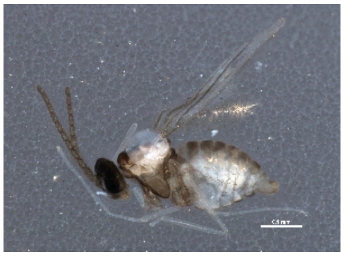

BIOUG11220-F03 [Lateral]
Cecidomyiidae
Family: Cecidomyiidae
BIN URI: BOLD:ACD1623

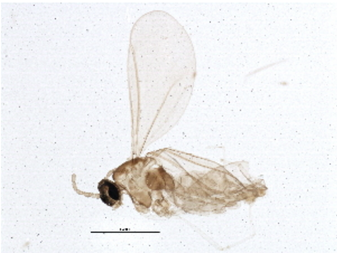

BIOUG06265-E09 [Lateral]
Cecidomyiidae
Family: Cecidomyiidae
BIN URI: BOLD:ACA8582

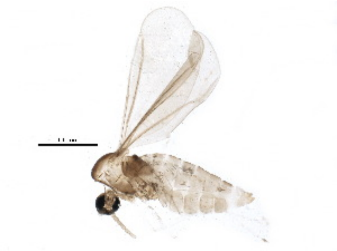

BIOUG22470-E10 [Lateral]
Cecidomyiidae
Family: Cecidomyiidae
BIN URI: BOLD:ACG3539

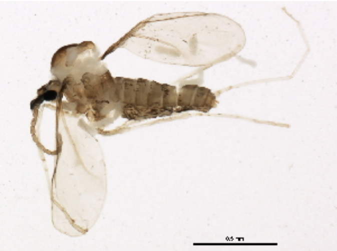

BIOUG01130-H07 [Lateral]
Cecidomyiidae
Family: Cecidomyiidae
BIN URI: BOLD:ABY9434

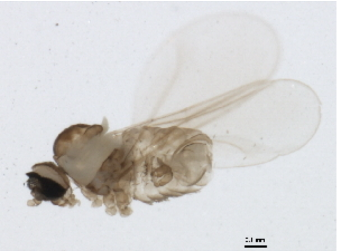

BIOUG01305-C01 [Lateral]
Cecidomyiidae
Family: Cecidomyiidae
BIN URI: BOLD:AAY6430

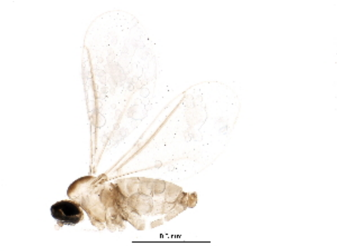

BIOUG22861-A04 [Lateral]
Cecidomyiidae
Family: Cecidomyiidae
BIN URI: BOLD:ACN2134

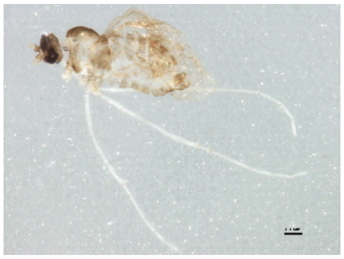

BIOUG01482-G08 [Lateral]
Cecidomyiidae
Family: Cecidomyiidae
BIN URI: BOLD:ABV1470

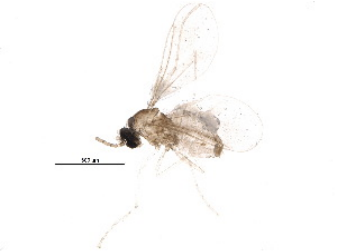

BIOUG20846-E08 [Lateral]
Diptera

BIN URI: BOLD:ACU5703

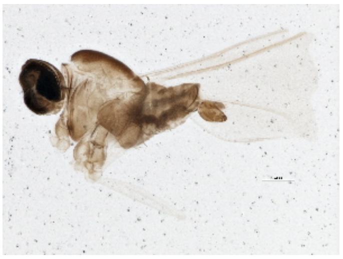

BIOUG02939-F04 [Lateral]
Cecidomyiidae
Family: Cecidomyiidae
BIN URI: BOLD:ACA5795

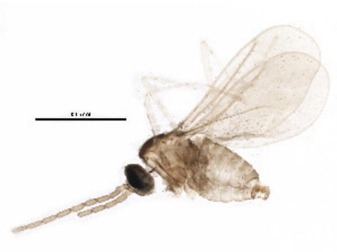

BIOUG23073-E03 [Lateral]
Cecidomyiidae
Family: Cecidomyiidae
BIN URI: BOLD:ACV0232

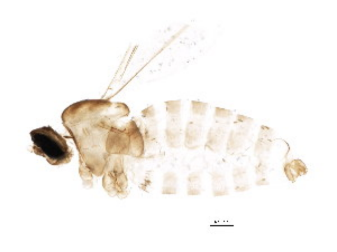

BIOUG01510-A04 [Lateral]
Cecidomyiidae
Family: Cecidomyiidae
BIN URI: BOLD:ABW8020

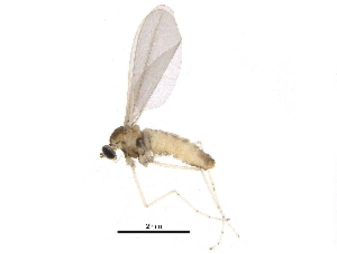

BIOUG22876-E03 [Lateral]
Cecidomyiidae
Family: Cecidomyiidae
BIN URI: BOLD:ACW0970

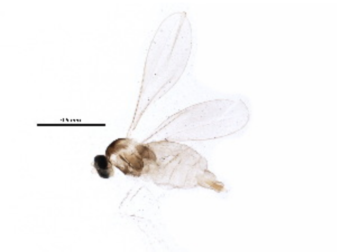

BIOUG22466-F05 [Lateral]
Cecidomyiidae
Family: Cecidomyiidae
BIN URI: BOLD:AAM6039

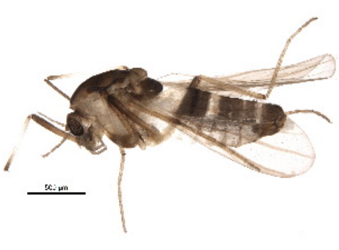

BIOUG22237-A07 [Lateral]
Cecidomyiidae
Family: Cecidomyiidae
BIN URI: BOLD:ACW0899

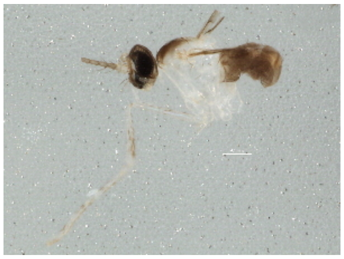

BIOUG03825-D02 [Lateral]
Cecidomyiidae
Family: Cecidomyiidae
BIN URI: BOLD:ACB3003

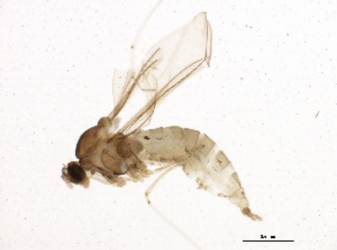

BIOUG01128-A08 [Lateral]
Cecidomyiidae
Family: Cecidomyiidae
BIN URI: BOLD:AAZ0283

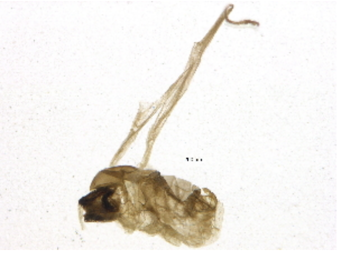

BIOUG02917-G01 [Lateral]
Cecidomyiidae
Family: Cecidomyiidae
BIN URI: BOLD:ACA5223

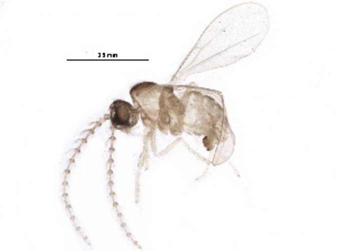

BIOUG23077-H11 [Lateral]
Cecidomyiidae
Family: Cecidomyiidae
BIN URI: BOLD:ACD0161

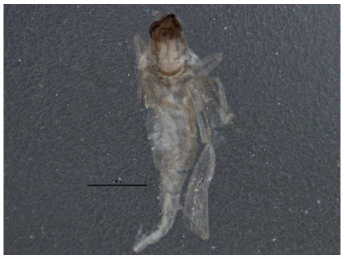

BIOUG02918-G02 [Dorsal]
Cecidomyiidae
Family: Cecidomyiidae
BIN URI: BOLD:ACA4968

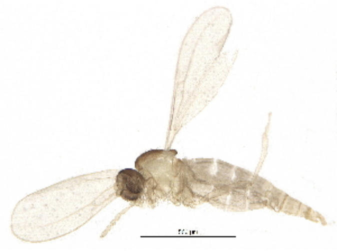

BIOUG08234-A12 [Lateral]
Cecidomyiidae
Family: Cecidomyiidae
BIN URI: BOLD:ACR6587

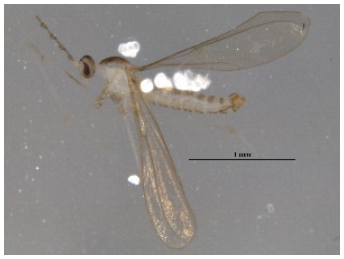

BIOUG03272-B02 [Lateral]
Cecidomyiidae
Family: Cecidomyiidae
BIN URI: BOLD:AAV5570

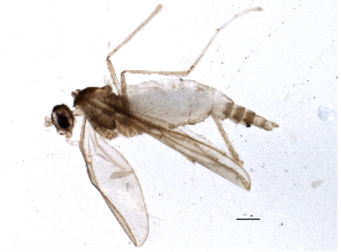

BIOUG04314-F11 [Lateral]
Cecidomyiidae
Family: Cecidomyiidae
BIN URI: BOLD:ACD1979

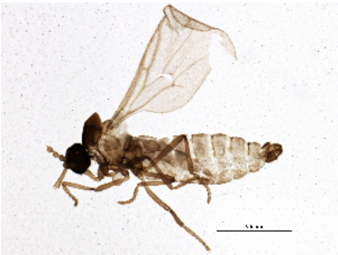

BIOUG01886-H04 [Lateral]
Cecidomyiidae
Family: Cecidomyiidae
BIN URI: BOLD:ABW2703

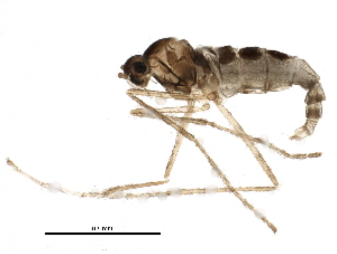

BIOUG23073-C02 [Lateral]
Cecidomyiidae
Family: Cecidomyiidae
BIN URI: BOLD:ACV5271

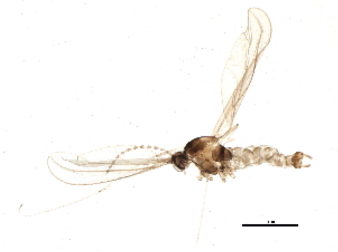

BIOUG10320-B01 [Lateral]
Cecidomyiidae
Family: Cecidomyiidae
BIN URI: BOLD:ACL4997

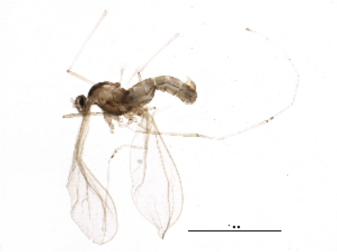

BIOUG22359-B06 [Lateral]
Cecidomyiidae
Family: Cecidomyiidae
BIN URI: BOLD:ACV3779

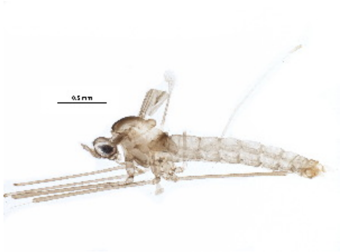

BIOUG22466-E12 [Lateral]
Cecidomyiidae
Family: Cecidomyiidae
BIN URI: BOLD:ACV4682

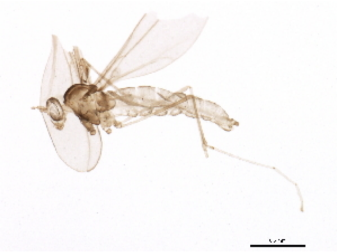

BIOUG01444-D05 [Lateral]
Cecidomyiidae
Family: Cecidomyiidae
BIN URI: BOLD:ABV0470

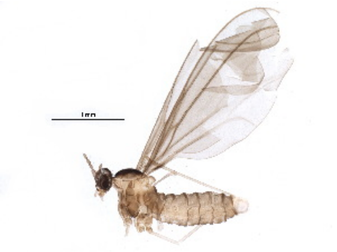

BIOUG22730-B09 [Lateral]
Cecidomyiidae
Family: Cecidomyiidae
BIN URI: BOLD:ACV6013

IMAGE NOT AVAILABLE

BIOUG23083-G11
Cecidomyiidae
Family: Cecidomyiidae

IMAGE NOT AVAILABLE

BIOUG23315-C08
Cecidomyiidae
Family: Cecidomyiidae

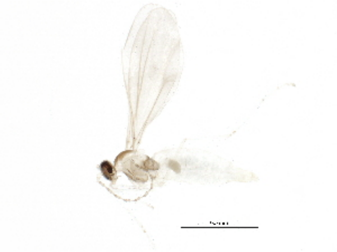

BIOUG22729-F07 [Lateral]
Cecidomyiidae
Family: Cecidomyiidae
BIN URI: BOLD:ABV0498

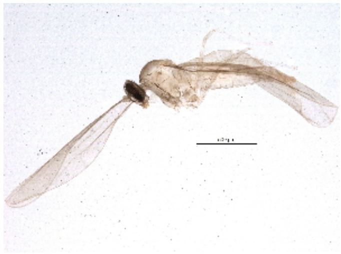

BIOUG06486-B04 [Lateral]
Cecidomyiidae
Family: Cecidomyiidae
BIN URI: BOLD:ACI5405

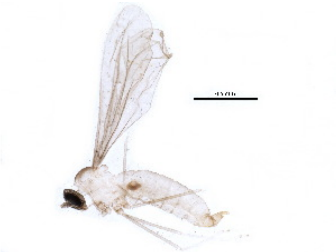

BIOUG23077-D02 [Lateral]
Cecidomyiidae
Family: Cecidomyiidae
BIN URI: BOLD:ACV5829

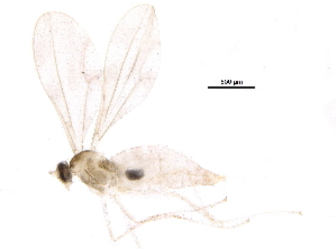

BIOUG23080-H05 [Lateral]
Cecidomyiidae
Family: Cecidomyiidae
BIN URI: BOLD:ABV1389

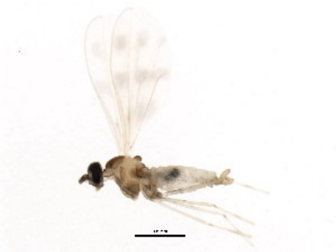

BIOUG01345-E04 [Lateral]
Cecidomyiidae
Family: Cecidomyiidae
BIN URI: BOLD:AAN5235

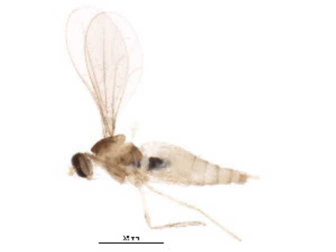

BIOUG01389-E06 [Lateral]
Cecidomyiidae
Family: Cecidomyiidae
BIN URI: BOLD:ABX3660

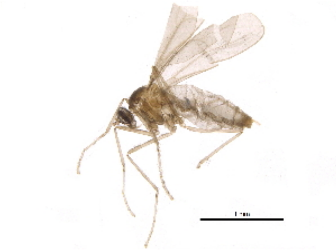

BIOUG22328-D05 [Lateral]
Cecidomyiidae
Family: Cecidomyiidae
BIN URI: BOLD:ACV2090

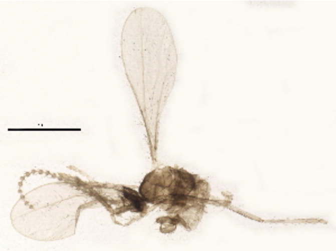

BIOUG08624-B01 [Lateral]
Cecidomyiidae
Family: Cecidomyiidae
BIN URI: BOLD:ABW8049

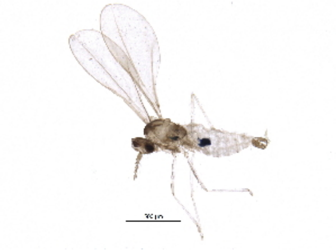

BIOUG08607-C05 [Lateral]
Cecidomyiidae
Family: Cecidomyiidae
BIN URI: BOLD:ACK2390

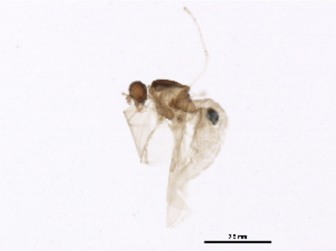

BIOUG01120-H01 [Lateral]
Cecidomyiidae
Family: Cecidomyiidae
BIN URI: BOLD:AAY6471

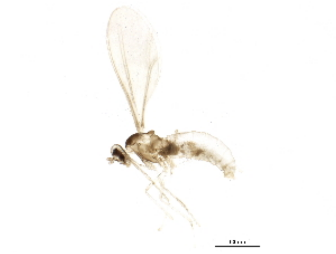

BIOUG22365-A03 [Lateral]
Cecidomyiidae
Family: Cecidomyiidae
BIN URI: BOLD:ACI8549

IMAGE NOT AVAILABLE

BIOUG23081-H10
Cecidomyiidae
Family: Cecidomyiidae

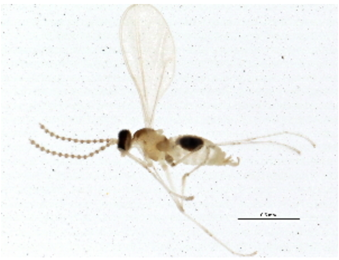

08TTML-2485 [Lateral]
Cecidomyiidae
Family: Cecidomyiidae
BIN URI: BOLD:AAN5282

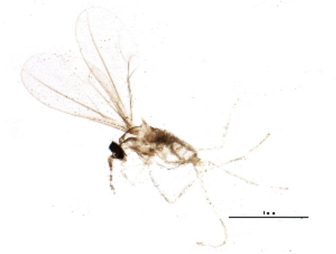

BIOUG08712-C04 [Lateral]
Cecidomyiidae
Family: Cecidomyiidae
BIN URI: BOLD:ACK2599

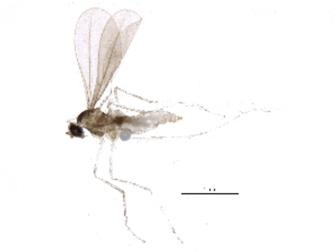

BIOUG11883-C07 [Lateral]
Cecidomyiidae
Family: Cecidomyiidae
BIN URI: BOLD:ACM9802

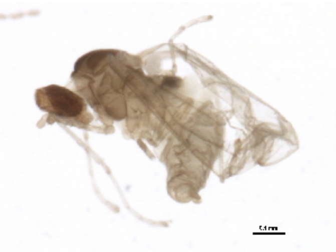

BIOUG01142-B08 [Lateral]
Cecidomyiidae
Family: Cecidomyiidae
BIN URI: BOLD:AAH3740

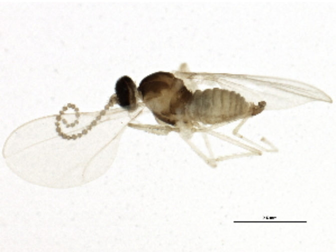

BIOUG01982-D08 [Lateral]
Cecidomyiidae
Family: Cecidomyiidae
BIN URI: BOLD:AAM6107

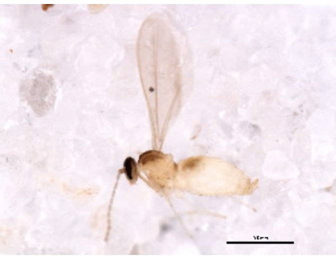

09BBEDI-2807 [Lateral]
Cecidomyiidae
Family: Cecidomyiidae
BIN URI: BOLD:AAN5186

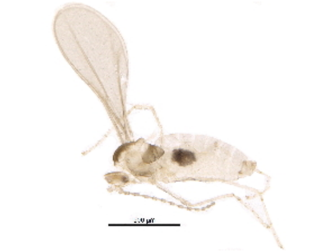

BIOUG22719-B08 [Lateral]
Cecidomyiidae
Family: Cecidomyiidae
BIN URI: BOLD:AAV5575

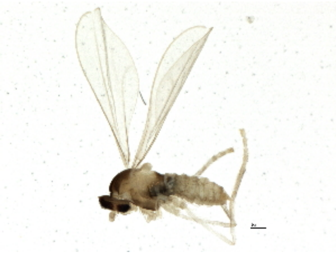

BIOUG02056-A08 [Lateral]
Cecidomyiidae
Family: Cecidomyiidae
BIN URI: BOLD:AAN5193

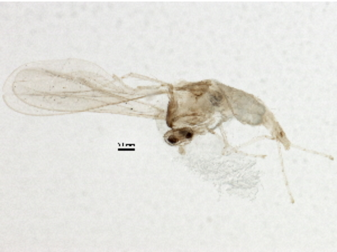

BIOUG03437-C01 [Lateral]
Cecidomyiidae
Family: Cecidomyiidae
BIN URI: BOLD:ABV0448

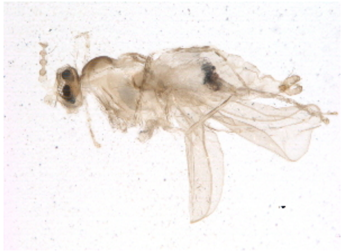

BIOUG03758-F09 [Lateral]
Cecidomyiidae
Family: Cecidomyiidae
BIN URI: BOLD:ABV1261

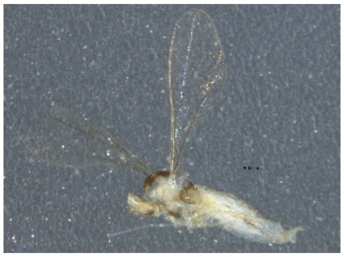

BIOUG04661-D08 [Lateral]
Cecidomyiidae
Family: Cecidomyiidae
BIN URI: BOLD:ACC8599

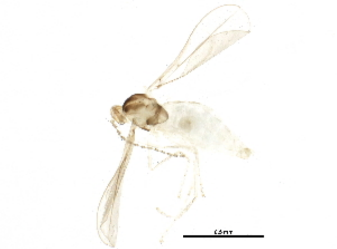

BIOUG22716-H01 [Lateral]
Cecidomyiidae
Family: Cecidomyiidae
BIN URI: BOLD:AAH3717

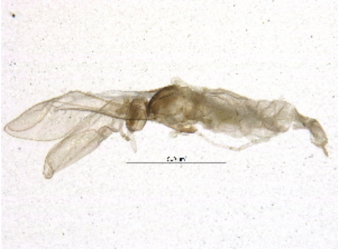

BIOUG03900-D06 [Lateral]
Cecidomyiidae
Family: Cecidomyiidae
BIN URI: BOLD:AAH3751

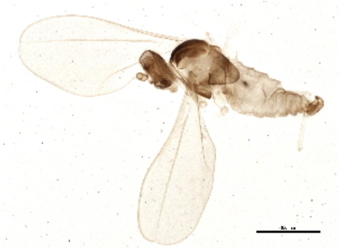

BIOUG02903-H07 [Lateral]
Cecidomyiidae
Family: Cecidomyiidae
BIN URI: BOLD:ABW8040

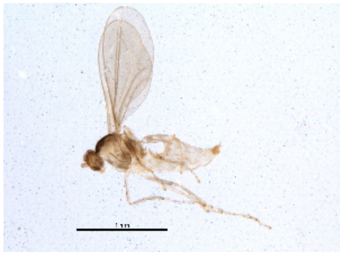

BIOUG04480-G04 [Lateral]
Cecidomyiidae
Family: Cecidomyiidae
BIN URI: BOLD:ACD1757

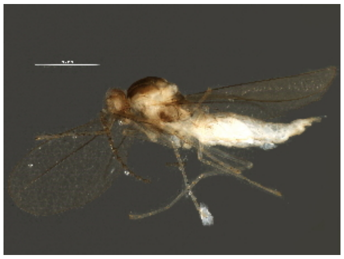

BIOUG08603-G03 [Lateral]
Cecidomyiidae
Family: Cecidomyiidae
BIN URI: BOLD:ACK1765

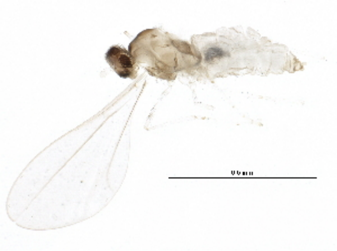

BIOUG22084-H06 [Lateral]
Cecidomyiidae
Family: Cecidomyiidae
BIN URI: BOLD:ABY0682

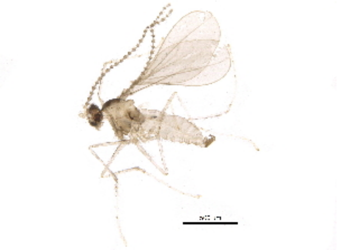

BIOUG22719-C08 [Lateral]
Cecidomyiidae
Family: Cecidomyiidae
BIN URI: BOLD:ACV3513

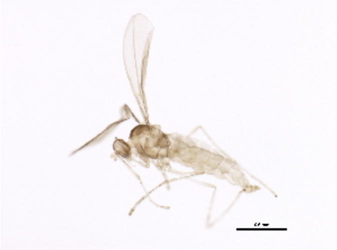

BIOUG01120-H10 [Lateral]
Cecidomyiidae
Family: Cecidomyiidae
BIN URI: BOLD:AAY6468

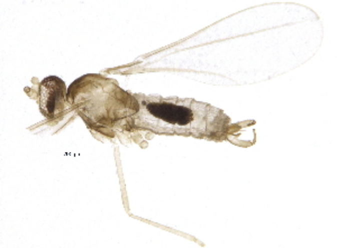

BIOUG03238-E11 [Lateral]
Cecidomyiidae
Family: Cecidomyiidae
BIN URI: BOLD:ACB0715

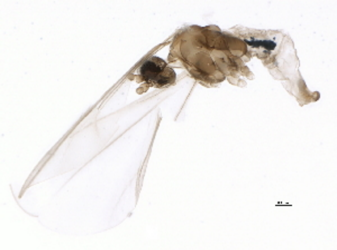

BIOUG01491-H07 [Lateral]
Cecidomyiidae
Family: Cecidomyiidae
BIN URI: BOLD:AAY6438

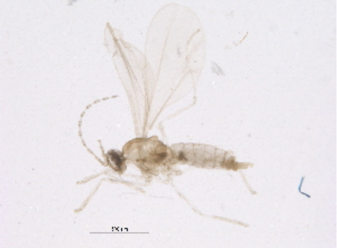

BIOUG04835-H06 [Lateral]
Cecidomyiidae
Family: Cecidomyiidae
BIN URI: BOLD:ACD6132

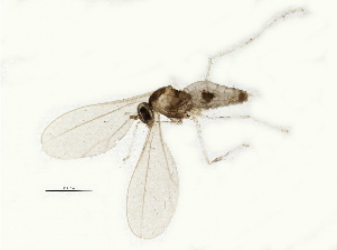

BIOUG14095-G04 [Lateral]
Cecidomyiidae
Family: Cecidomyiidae
BIN URI: BOLD:ACD3287

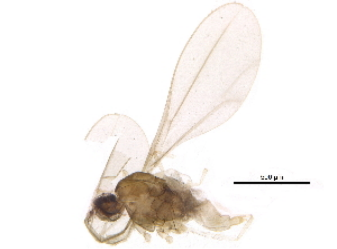

BIOUG22719-A01 [Lateral]
Cecidomyiidae
Family: Cecidomyiidae
BIN URI: BOLD:ACI4196

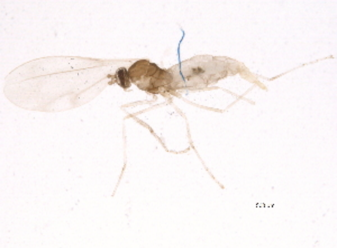

BIOUG03954-B12 [Lateral]
Cecidomyiidae
Family: Cecidomyiidae
BIN URI: BOLD:ACD0329

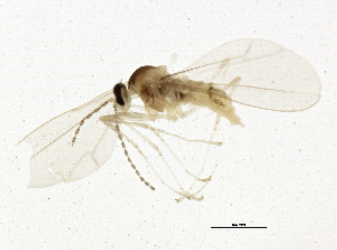

BIOUG01982-D07 [Lateral]
Cecidomyiidae
Family: Cecidomyiidae
BIN URI: BOLD:AAH3720

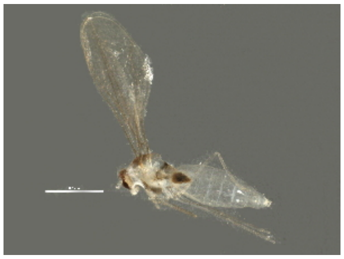

BIOUG08603-C08 [Lateral]
Cecidomyiidae
Family: Cecidomyiidae
BIN URI: BOLD:AAY6450

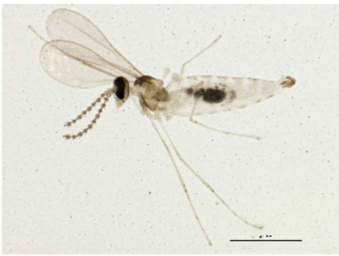

BIOUG02006-A06 [Lateral]
Cecidomyiidae
Family: Cecidomyiidae
BIN URI: BOLD:AAH3742

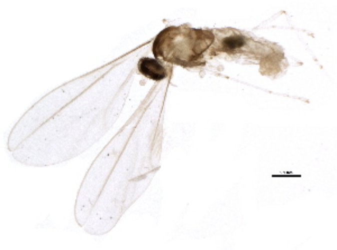

BIOUG04314-F02 [Lateral]
Cecidomyiidae
Family: Cecidomyiidae
BIN URI: BOLD:AAY6456

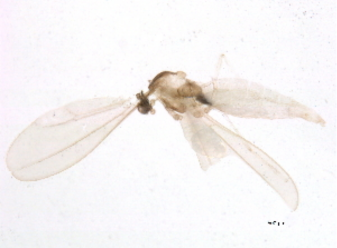

BIOUG03758-A11 [Lateral]
Cecidomyiidae
Family: Cecidomyiidae
BIN URI: BOLD:ACC8702

IMAGE NOT AVAILABLE

BIOUG22730-G04
Cecidomyiidae
Family: Cecidomyiidae
BIN URI: BOLD:ACV5340

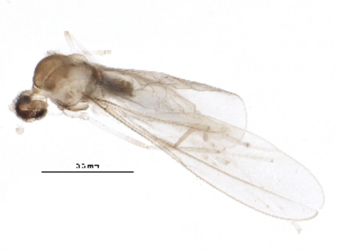

BIOUG22733-C08 [Lateral]
Cecidomyiidae
Family: Cecidomyiidae
BIN URI: BOLD:ACV5422

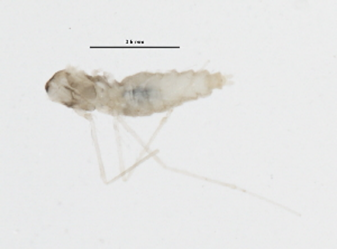

10JSROW-1752 [Lateral]
Cecidomyiidae
Family: Cecidomyiidae
BIN URI: BOLD:AAV5356

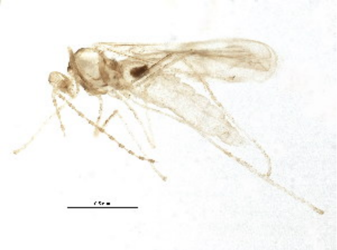

BIOUG01621-E05 [Lateral]
Cecidomyiidae
Family: Cecidomyiidae
BIN URI: BOLD:ABV1291

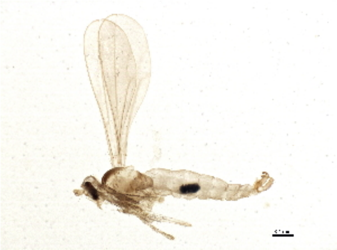

BIOUG03066-B08 [Lateral]
Cecidomyiidae
Family: Cecidomyiidae
BIN URI: BOLD:ABV1337

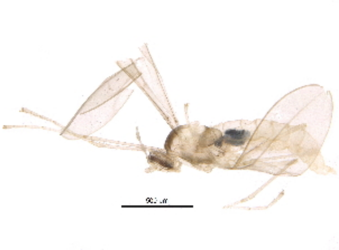

BIOUG22328-A09 [Lateral]
Cecidomyiidae
Family: Cecidomyiidae
BIN URI: BOLD:ABV1379

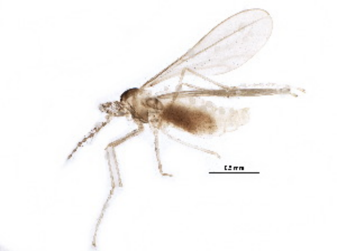

BIOUG22417-F01 [Lateral]
Cecidomyiidae
Family: Cecidomyiidae
BIN URI: BOLD:ACV4577

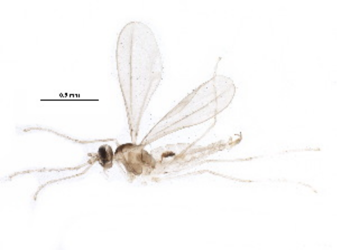

BIOUG22721-H01 [Lateral]
Cecidomyiidae
Family: Cecidomyiidae
BIN URI: BOLD:ACV5221

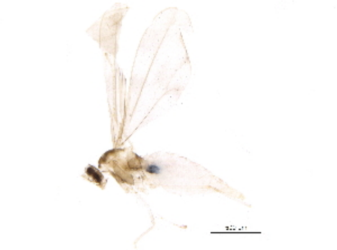

BIOUG22323-D06 [Lateral]
Cecidomyiidae
Family: Cecidomyiidae
BIN URI: BOLD:AAV5793

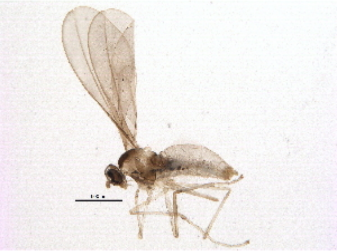

BIOUG10277-B03 [Lateral]
Cecidomyiidae
Family: Cecidomyiidae
BIN URI: BOLD:ABX7420

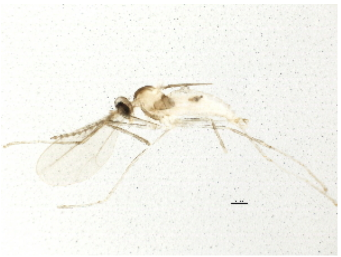

BIOUG10401-D09 [Lateral]
Cecidomyiidae
Family: Cecidomyiidae
BIN URI: BOLD:ACC6635

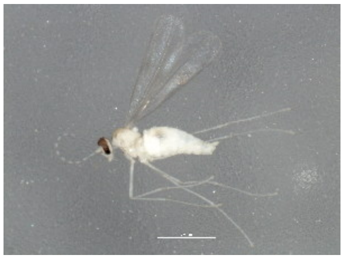

BIOUG00819-F01 [Lateral]
Cecidomyiidae
Family: Cecidomyiidae
BIN URI: BOLD:AAQ0294

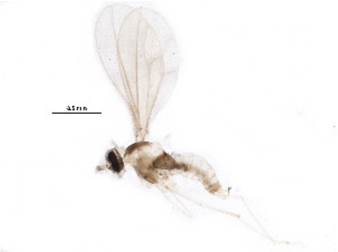

BIOUG23077-E06 [Lateral]
Cecidomyiidae
Family: Cecidomyiidae
BIN URI: BOLD:ACF3884

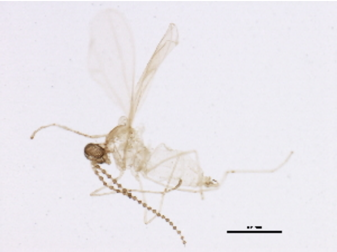

BIOUG01118-F05 [Lateral]
Cecidomyiidae
Family: Cecidomyiidae
BIN URI: BOLD:AAY6442

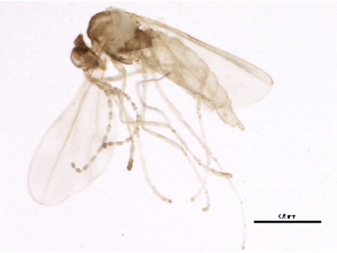

BIOUG01128-C07 [Lateral]
Cecidomyiidae
Family: Cecidomyiidae
BIN URI: BOLD:AAZ0267

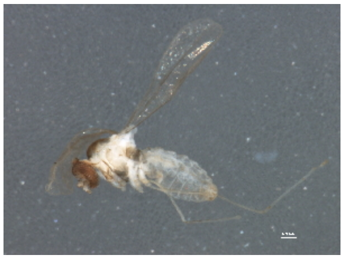

BIOUG01132-G04 [Lateral]
Cecidomyiidae
Family: Cecidomyiidae
BIN URI: BOLD:AAZ0284

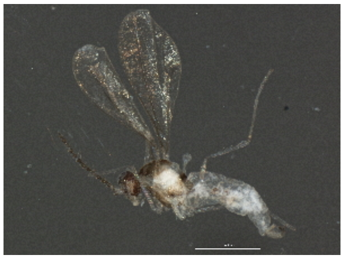

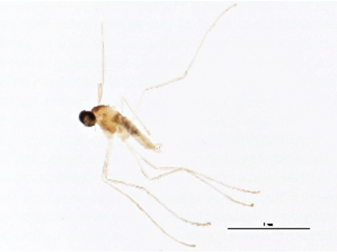

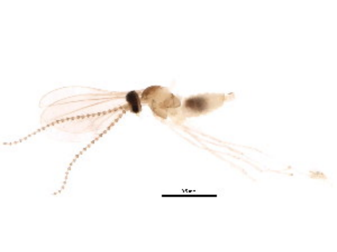

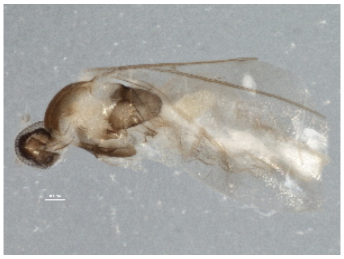

BIOUG05170-G03 [Lateral]
Cecidomyiidae
Family: Cecidomyiidae
BIN URI: BOLD:ABV1390

08TTML-2490 [Lateral]
Cecidomyiidae
Family: Cecidomyiidae
BIN URI: BOLD:AAN5285

BIOUG01400-G12 [Lateral]
Cecidomyiidae
Family: Cecidomyiidae
BIN URI: BOLD:ABX9467

BIOUG08687-F06 [Lateral]
Cecidomyiidae
Family: Cecidomyiidae
BIN URI: BOLD:ACJ6855

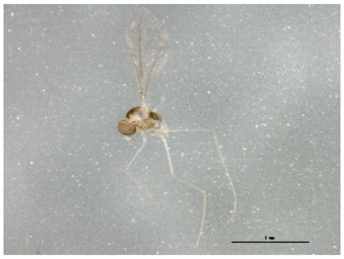

BIOUG01479-H04 [Lateral]
Cecidomyiidae
Family: Cecidomyiidae
BIN URI: BOLD:ABV9362

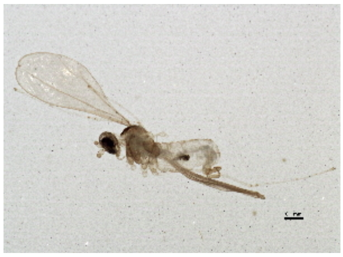

BIOUG02998-G05 [Lateral]
Cecidomyiidae
Family: Cecidomyiidae
BIN URI: BOLD:ABA0863

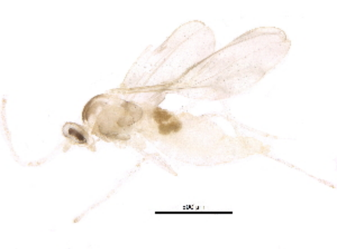

BIOUG23079-H04 [Lateral]
Cecidomyiidae
Family: Cecidomyiidae
BIN URI: BOLD:ACS9320

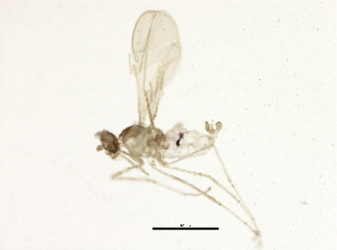

BIOUG01116-F08 [Lateral]
Cecidomyiidae
Family: Cecidomyiidae
BIN URI: BOLD:ABZ3625

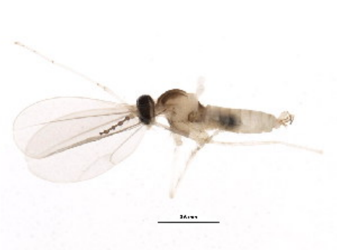

BIOUG01352-C02 [Lateral]
Cecidomyiidae
Family: Cecidomyiidae
BIN URI: BOLD:AAH3755

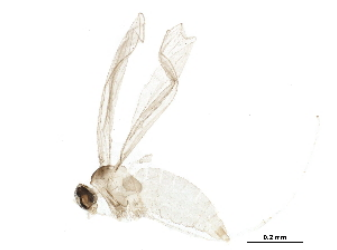

BIOUG20831-B08 [Lateral]
Diptera

BIN URI: BOLD:ACU7030

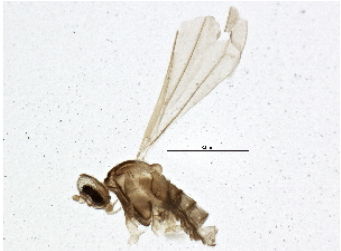

BIOUG02513-D11 [Lateral]
Cecidomyiidae
Family: Cecidomyiidae
BIN URI: BOLD:ABW7897

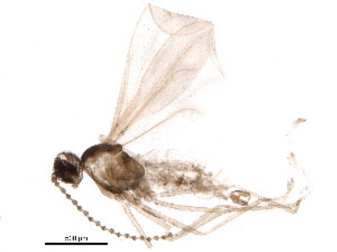

BIOUG23083-C10 [Lateral]
Cecidomyiidae
Family: Cecidomyiidae
BIN URI: BOLD:ACW0955

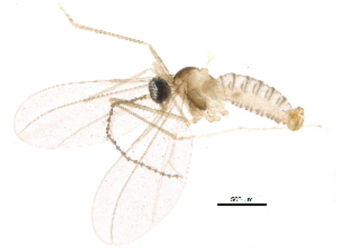

BIOUG23081-A02 [Lateral]
Cecidomyiidae
Family: Cecidomyiidae
BIN URI: BOLD:ACW1081

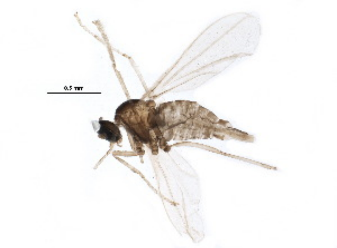

BIOUG22470-F05 [Lateral]
Cecidomyiidae
Family: Cecidomyiidae
BIN URI: BOLD:ACV3873

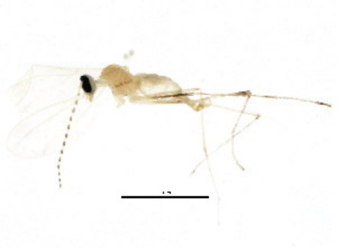

BIOUG01337-B08 [Lateral]
Cecidomyiidae
Family: Cecidomyiidae
BIN URI: BOLD:ACM1807

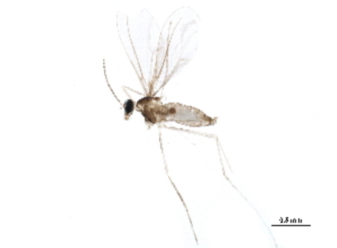

BIOUG23323-E12 [Lateral]
Cecidomyiidae
Family: Cecidomyiidae
BIN URI: BOLD:ACW1131

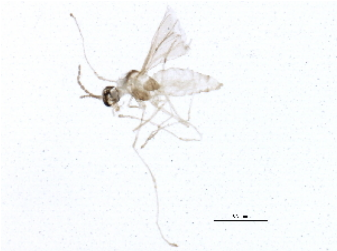

BIOUG01117-G01 [Lateral]
Cecidomyiidae
Family: Cecidomyiidae
BIN URI: BOLD:AAU6476

IMAGE NOT AVAILABLE

BIOUG23081-B02
Cecidomyiidae
Family: Cecidomyiidae

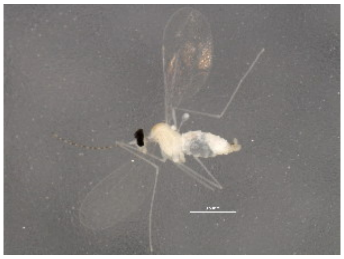

BIOUG01423-F01 [Lateral]
Cecidomyiidae
Family: Cecidomyiidae
BIN URI: BOLD:AAH3741

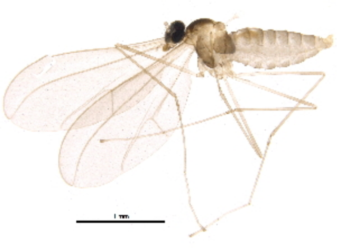

BIOUG22328-A07 [Lateral]
Cecidomyiidae
Family: Cecidomyiidae
BIN URI: BOLD:AAQ0634

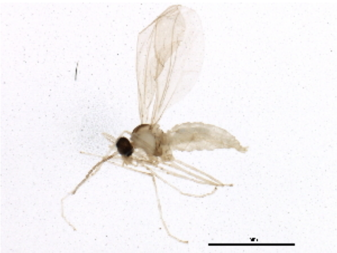

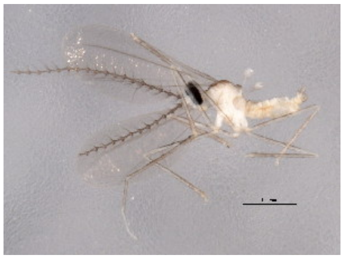

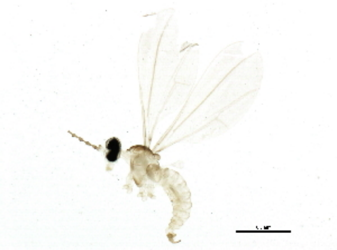

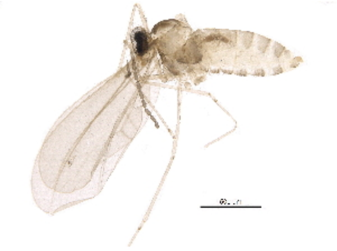

BIOUG01131-G02 [Lateral]
Cecidomyiidae
Family: Cecidomyiidae
BIN URI: BOLD:AAY6445

BIOUG00973-D07 [Lateral]
Cecidomyiidae
Family: Cecidomyiidae
BIN URI: BOLD:AAG8269

BIOUG01492-A09 [Lateral]
Cecidomyiidae
Family: Cecidomyiidae
BIN URI: BOLD:ABW6103

BIOUG23315-D04 [Lateral]
Cecidomyiidae
Family: Cecidomyiidae
BIN URI: BOLD:ACW0802

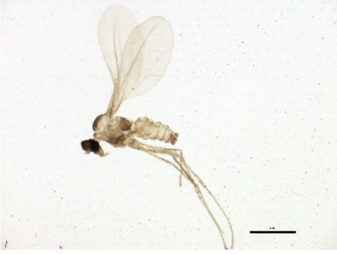

BIOUG01116-G12 [Lateral]
Cecidomyiidae
Family: Cecidomyiidae
BIN URI: BOLD:AAN5212

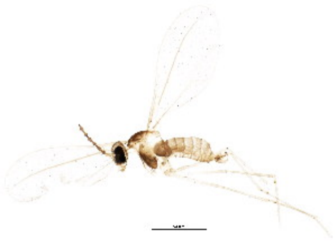

BIOUG01510-E09 [Lateral]
Cecidomyiidae
Family: Cecidomyiidae
BIN URI: BOLD:ACK1667

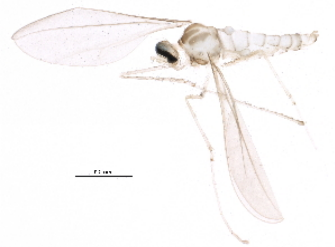

BIOUG23072-D03 [Lateral]
Cecidomyiidae
Family: Cecidomyiidae
BIN URI: BOLD:ACV5229

IMAGE NOT AVAILABLE

BIOUG23084-E03
Cecidomyiidae
Family: Cecidomyiidae

IMAGE NOT AVAILABLE

BIOUG23082-G12
Cecidomyiidae
Family: Cecidomyiidae

IMAGE NOT AVAILABLE

BIOUG23310-A01
Cecidomyiidae
Family: Cecidomyiidae

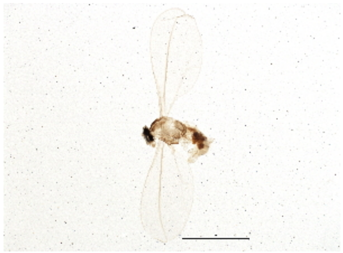

BIOUG02906-F08 [Lateral]
Cecidomyiidae
Family: Cecidomyiidae
BIN URI: BOLD:ACB3134

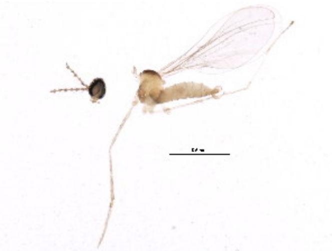

BIOUG00978-D03 [Lateral]
Cecidomyiidae
Family: Cecidomyiidae
BIN URI: BOLD:AAY6112

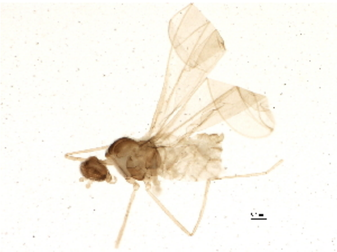

BIOUG01132-A02 [Lateral]
Cecidomyiidae
Family: Cecidomyiidae
BIN URI: BOLD:AAM6116

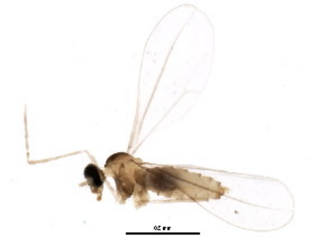

BIOUG00978-E10 [Lateral]
Cecidomyiidae
Family: Cecidomyiidae
BIN URI: BOLD:AAN5181

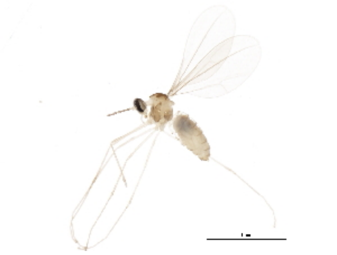

08TTML-2488 [Lateral]
Cecidomyiidae
Family: Cecidomyiidae
BIN URI: BOLD:ACE3371

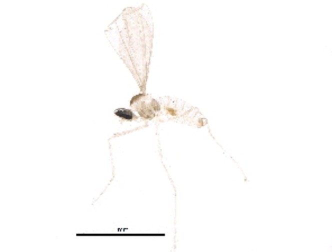

BIOUG23314-C12 [Lateral]
Asteromyia
Family: Cecidomyiidae
BIN URI: BOLD:ACW1255

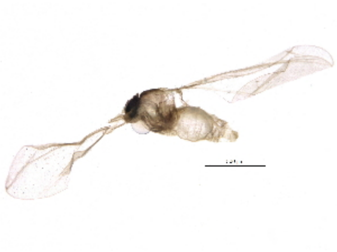

BIOUG09715-C06 [Lateral]
Asteromyia
Family: Cecidomyiidae
BIN URI: BOLD:ACL5259

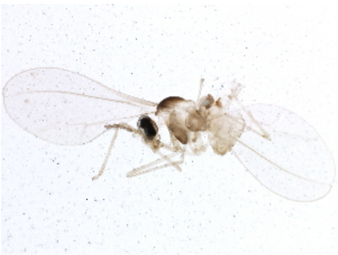

BIOUG10453-F06 [Lateral]
Asteromyia
Family: Cecidomyiidae
BIN URI: BOLD:ACL6776

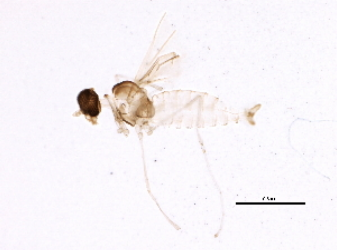

BIOUG01446-A01 [Lateral]
Cecidomyiidae
Family: Cecidomyiidae
BIN URI: BOLD:AAN5223

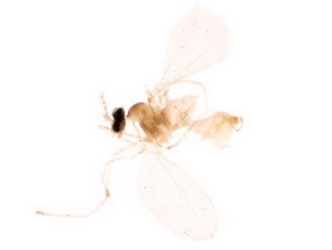

BIOUG01460-A05 [Lateral]
Cecidomyiidae
Family: Cecidomyiidae
BIN URI: BOLD:ACC5677

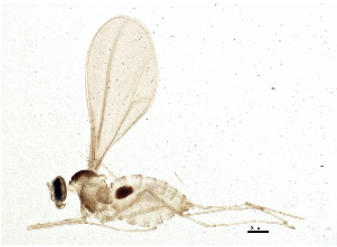

IMAGE NOT AVAILABLE

BIOUG23078-A06

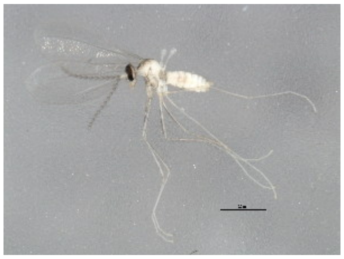

IMAGE NOT AVAILABLE

BIOUG23308-E09

BIOUG03278-B07 [Lateral]
Asteromyia
Family: Cecidomyiidae
BIN URI: BOLD:AAV5552

Cecidomyiidae
Family: Cecidomyiidae

BIOUG00819-D05 [Lateral]
Cecidomyiidae
Family: Cecidomyiidae
BIN URI: BOLD:AAM6037

Cecidomyiidae
Family: Cecidomyiidae

IMAGE NOT AVAILABLE

BIOUG22452-B07
Cecidomyiidae
Family: Cecidomyiidae

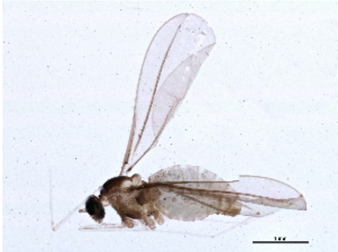

BIOUG03094-G12 [Lateral]
Cecidomyiidae
Family: Cecidomyiidae
BIN URI: BOLD:ACC1334

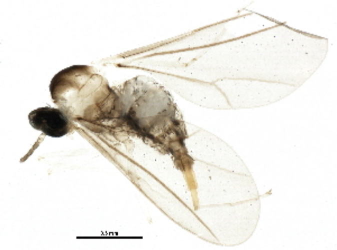

BIOUG22365-F09 [Lateral]
Cecidomyiidae
Family: Cecidomyiidae
BIN URI: BOLD:ACV4726

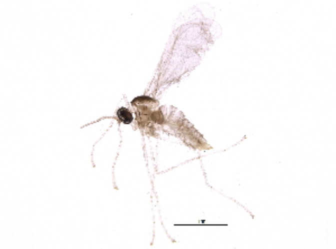

BIOUG11363-H02 [Lateral]
Cecidomyiidae
Family: Cecidomyiidae
BIN URI: BOLD:ACM2941

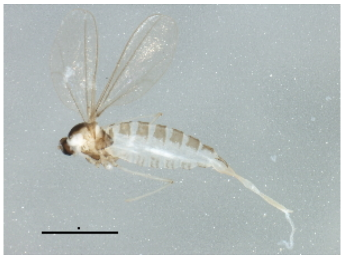

BIOUG01478-F08 [Lateral]
Cecidomyiidae
Family: Cecidomyiidae
BIN URI: BOLD:AAY6418

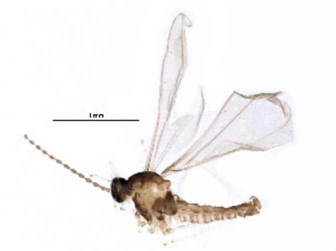

BIOUG22419-G12 [Lateral]
Dasineura
Family: Cecidomyiidae
BIN URI: BOLD:ACV3706

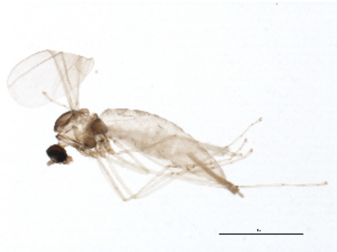

10PHMAL-1466 [Lateral]
Cecidomyiidae
Family: Cecidomyiidae
BIN URI: BOLD:AAU6618

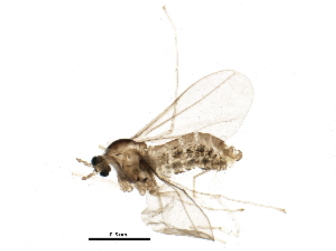

BIOUG21893-G06 [Lateral]
Cecidomyiidae
Family: Cecidomyiidae
BIN URI: BOLD:ACV5220

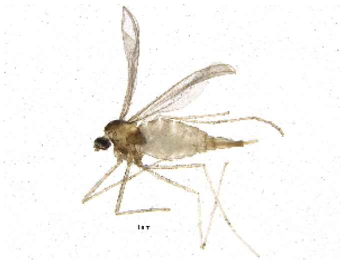

BIOUG08599-C07 [Lateral]
Cecidomyiidae
Family: Cecidomyiidae
BIN URI: BOLD:ACJ6637

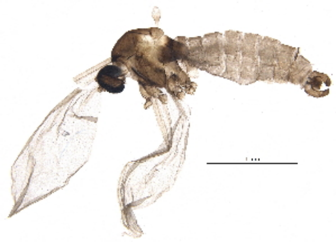

BIOUG16057-D07 [Lateral]
Cecidomyiidae
Family: Cecidomyiidae
BIN URI: BOLD:ACP9024

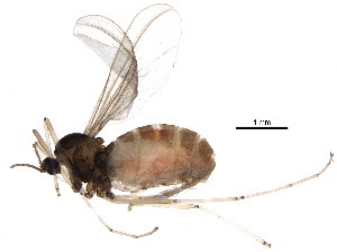

BIOUG24024-G07 [Lateral]
Cecidomyiidae
Family: Cecidomyiidae

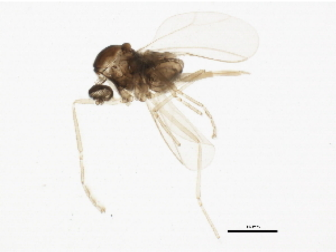

10PHMAL-3432 [Lateral]
Cecidomyiidae
Family: Cecidomyiidae
BIN URI: BOLD:AAN5178

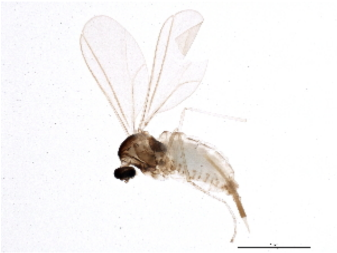

BIOUG02638-H07 [Lateral]
Cecidomyiidae
Family: Cecidomyiidae
BIN URI: BOLD:ABV0473

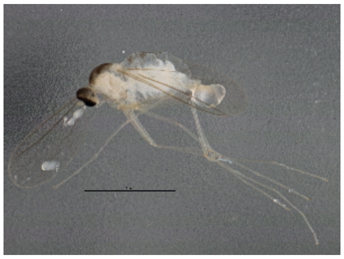

BIOUG03562-B12 [Lateral]
Cecidomyiidae
Family: Cecidomyiidae
BIN URI: BOLD:ACA9569

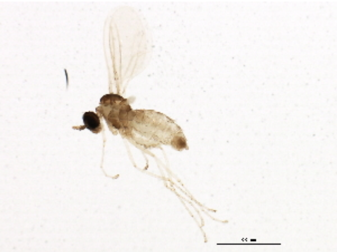

BIOUG01125-D03 [Lateral]
Cecidomyiidae
Family: Cecidomyiidae
BIN URI: BOLD:AAQ2523

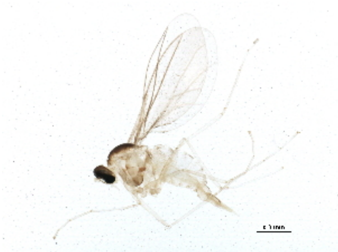

BIOUG06297-B06 [Lateral]
Cecidomyiidae
Family: Cecidomyiidae
BIN URI: BOLD:AAU6610

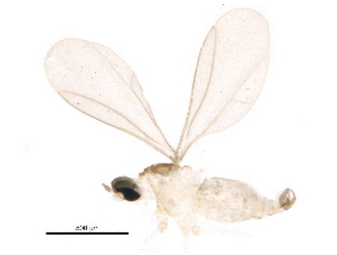

BIOUG22576-C05 [Lateral]

IMAGE NOT AVAILABLE

BIOUG23086-E11
Cecidomyiidae
Family: Cecidomyiidae

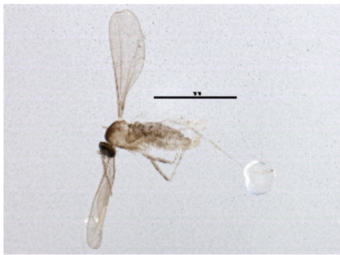

BIOUG03518-H10 [Lateral]

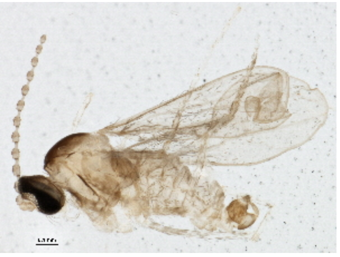

BIOUG03679-E12 [Lateral]

Cecidomyiidae
Family: Cecidomyiidae
BIN URI: BOLD:ACA4717

Cecidomyiidae
Family: Cecidomyiidae
BIN URI: BOLD:ACC8185

Cecidomyiidae
Family: Cecidomyiidae
BIN URI: BOLD:ACC8560

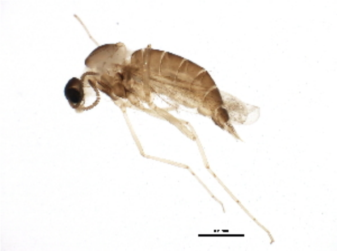

BIOUG01444-A08 [Lateral]
Cecidomyiidae
Family: Cecidomyiidae
BIN URI: BOLD:ACF4376

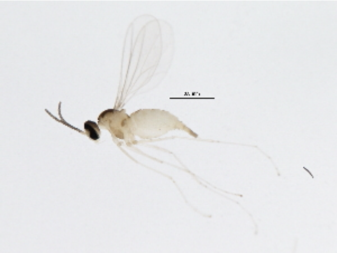

10JSROW-1425 [Lateral]
Cecidomyiidae
Family: Cecidomyiidae
BIN URI: BOLD:AAV5323

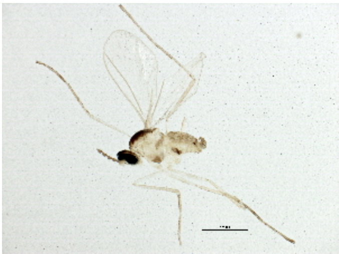

BIOUG03069-D11 [Lateral]
Cecidomyiidae
Family: Cecidomyiidae
BIN URI: BOLD:AAV5790

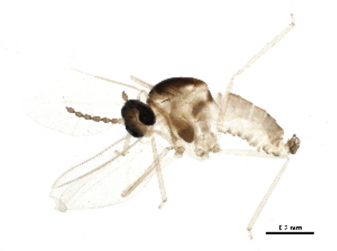

BIOUG21893-B02 [Lateral]
Cecidomyiinae
Family: Cecidomyiidae
BIN URI: BOLD:ACK5585

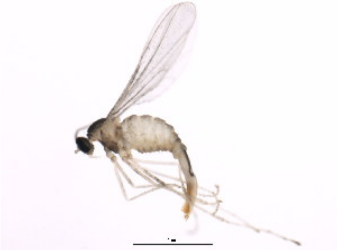

BIOUG00860-D07 [Lateral]
Cecidomyiidae
Family: Cecidomyiidae
BIN URI: BOLD:AAQ0262

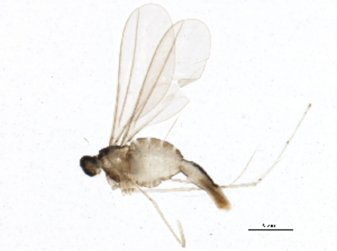

10PHMAL-2762 [Lateral]
Cecidomyiidae
Family: Cecidomyiidae
BIN URI: BOLD:ACE6955

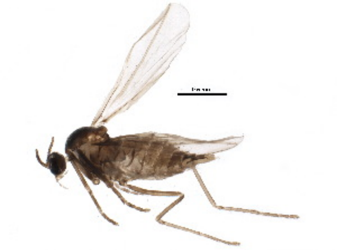

BIOUG21770-A05 [Lateral]
Cecidomyiidae
Family: Cecidomyiidae
BIN URI: BOLD:ACV2925

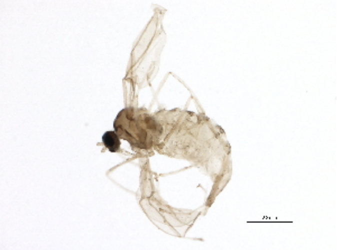

BIOUG01119-E02 [Lateral]
Cecidomyiidae
Family: Cecidomyiidae
BIN URI: BOLD:AAY6452

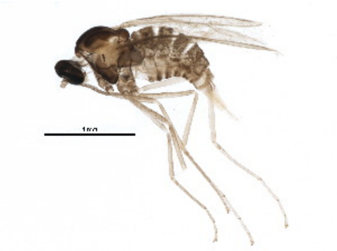

BIOUG22292-E10 [Lateral]
Cecidomyiidae
Family: Cecidomyiidae
BIN URI: BOLD:ACV2288

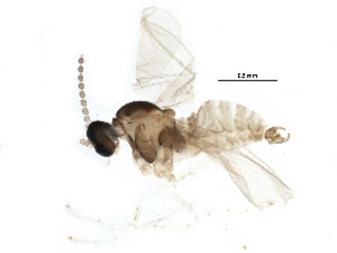

BIOUG22291-D11 [Lateral]
Cecidomyiidae
Family: Cecidomyiidae
BIN URI: BOLD:ACV3141

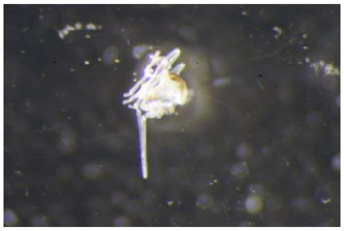

BIOUG08135-G12 [Lateral]
Cecidomyiidae
Family: Cecidomyiidae
BIN URI: BOLD:AAY6448

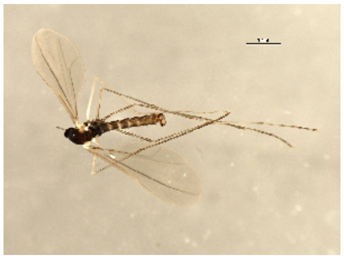

10JSROW-0813 [Dorsal]
Cecidomyiidae
Family: Cecidomyiidae
BIN URI: BOLD:AAP9022

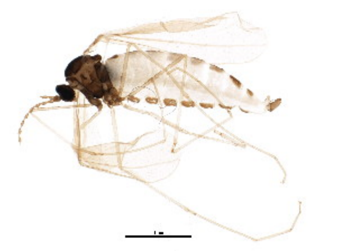

BIOUG01684-A04 [Lateral]
Mayetiola
Family: Cecidomyiidae
BIN URI: BOLD:ABV9277

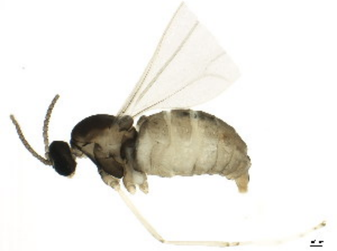

BIOUG01348-B08 [Lateral]
Cecidomyiidae
Family: Cecidomyiidae
BIN URI: BOLD:AAQ0642

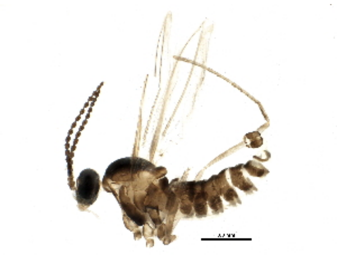

BIOUG22365-C04 [Lateral]
Cecidomyiidae
Family: Cecidomyiidae
BIN URI: BOLD:ACV4450

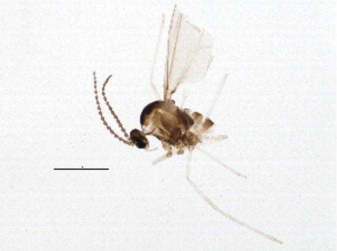

BIOUG02601-H05 [Lateral]
Cecidomyiidae
Family: Cecidomyiidae
BIN URI: BOLD:ABX7522

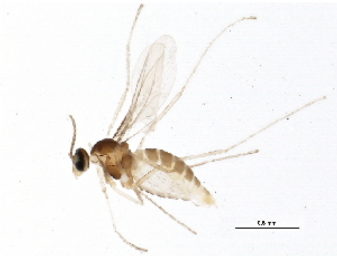

08TTML-2494 [Lateral]
Cecidomyiidae
Family: Cecidomyiidae
BIN URI: BOLD:AAN5200

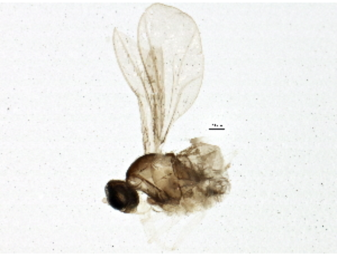

BIOUG03066-C06 [Lateral]
Cecidomyiidae
Family: Cecidomyiidae
BIN URI: BOLD:ACC7924

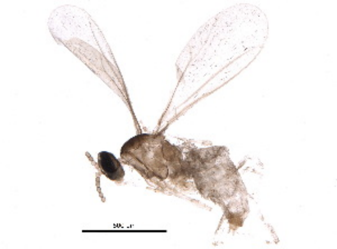

BIOUG22454-E03 [Lateral]
Cecidomyiidae
Family: Cecidomyiidae
BIN URI: BOLD:ACV4210

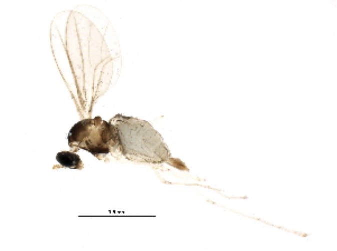

BIOUG23073-B10 [Lateral]
Cecidomyiidae
Family: Cecidomyiidae
BIN URI: BOLD:ACV5688

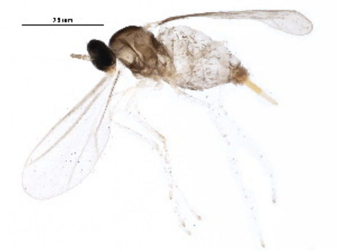

BIOUG23078-C07 [Lateral]
Cecidomyiidae
Family: Cecidomyiidae
BIN URI: BOLD:ACV5834

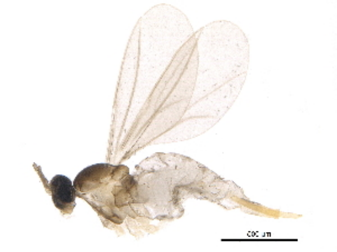

BIOUG23308-H10 [Lateral]
Cecidomyiidae
Family: Cecidomyiidae
BIN URI: BOLD:ACW1203

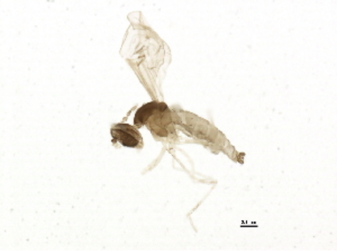

BIOUG01132-C04 [Lateral]
Cecidomyiidae
Family: Cecidomyiidae
BIN URI: BOLD:AAY6376

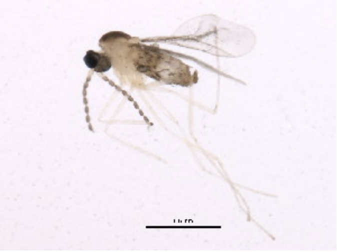

BIOUG00860-F09 [Lateral]
Cecidomyiidae
Family: Cecidomyiidae
BIN URI: BOLD:ABA0830

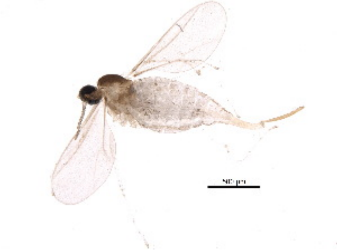

BIOUG23075-G09 [Lateral]
Cecidomyiidae
Family: Cecidomyiidae
BIN URI: BOLD:ACV5280

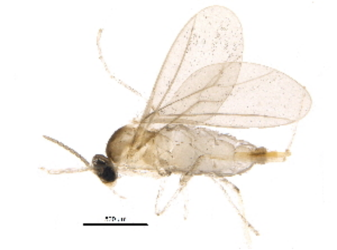

BIOUG23076-A01 [Lateral]
Cecidomyiidae
Family: Cecidomyiidae
BIN URI: BOLD:ACJ0207

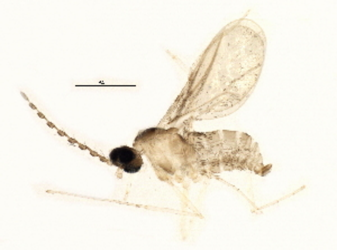

BIOUG08625-C04 [Lateral]
Cecidomyiidae
Family: Cecidomyiidae
BIN URI: BOLD:ACK2812

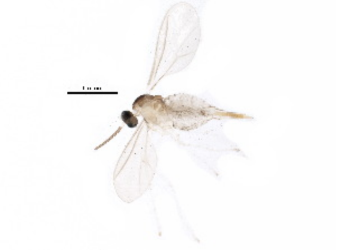

BIOUG22721-G01 [Lateral]
Cecidomyiidae
Family: Cecidomyiidae
BIN URI: BOLD:ACV5343

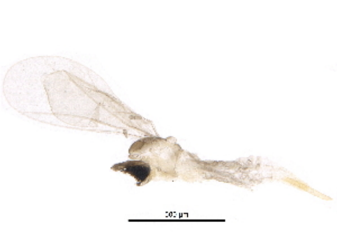

BIOUG23080-F05 [Lateral]
Cecidomyiidae
Family: Cecidomyiidae
BIN URI: BOLD:ACV5733

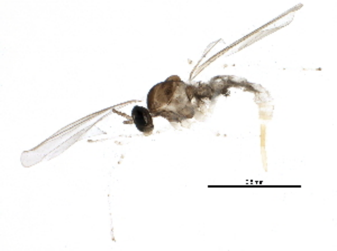

BIOUG22084-E11 [Lateral]
Cecidomyiidae
Family: Cecidomyiidae
BIN URI: BOLD:ABW7767

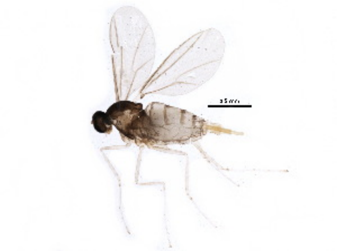

BIOUG22418-C01 [Lateral]
Cecidomyiidae
Family: Cecidomyiidae
BIN URI: BOLD:ACV4035

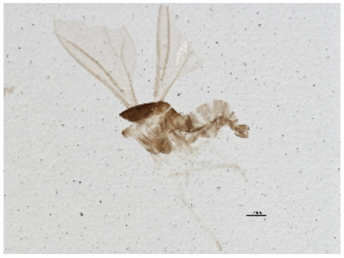

BIOUG02628-D04 [Lateral]
Cecidomyiidae
Family: Cecidomyiidae
BIN URI: BOLD:ABX7810

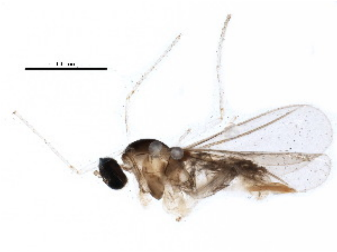

BIOUG23078-C12 [Lateral]
Cecidomyiidae
Family: Cecidomyiidae
BIN URI: BOLD:ACV4868

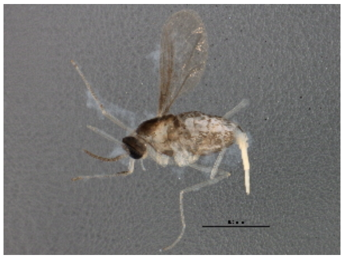

BIOUG03750-D05 [Lateral]
Cecidomyiidae
Family: Cecidomyiidae
BIN URI: BOLD:AAM6038

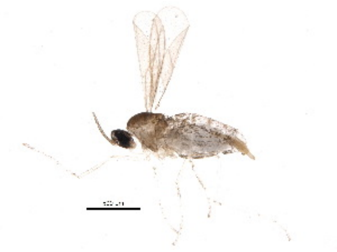

BIOUG23084-G06 [Lateral]
Cecidomyiidae
Family: Cecidomyiidae
BIN URI: BOLD:ACW0773

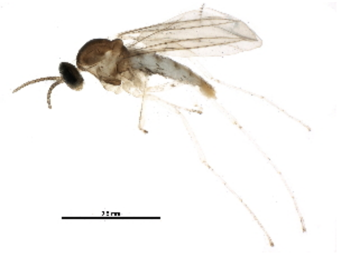

BIOUG23072-E08 [Lateral]
Cecidomyiidae
Family: Cecidomyiidae
BIN URI: BOLD:ACV5840

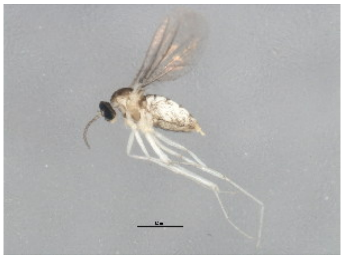

BIOUG00819-D09 [Lateral]
Cecidomyiidae
Family: Cecidomyiidae
BIN URI: BOLD:AAH3701

IMAGE NOT AVAILABLE

BIOUG22722-D02
Cecidomyiidae
Family: Cecidomyiidae

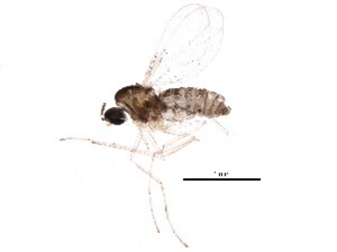

BIOUG22351-C04 [Lateral]
Cecidomyiidae
Family: Cecidomyiidae
BIN URI: BOLD:AAN5195

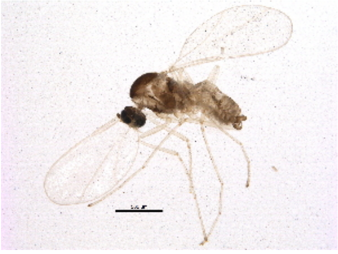

BIOUG10359-E01 [Lateral]
Cecidomyiidae
Family: Cecidomyiidae
BIN URI: BOLD:ACL4289

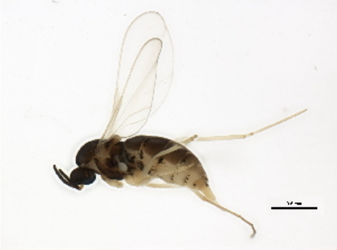

BIOUG01410-E03 [Lateral]
Cecidomyiidae
Family: Cecidomyiidae
BIN URI: BOLD:AAN5221

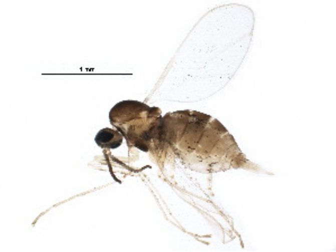

BIOUG22463-D04 [Lateral]
Cecidomyiidae
Family: Cecidomyiidae
BIN URI: BOLD:ACM2973

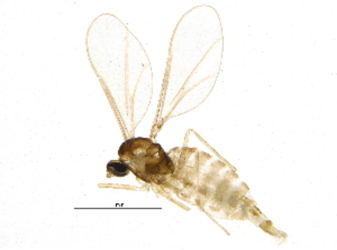

BIOUG02894-H09 [Lateral]
Asteromyia modesta
Family: Cecidomyiidae
BIN URI: BOLD:ACG8775

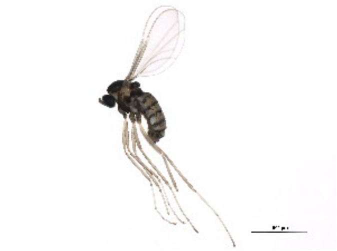

BIOUG01348-G03 [Lateral]
Cecidomyiidae
Family: Cecidomyiidae
BIN URI: BOLD:AAA2254

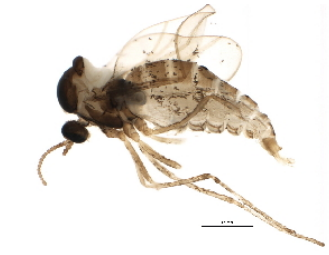

BIOUG01125-E06 [Lateral]
Asteromyia carbonifera
Family: Cecidomyiidae
BIN URI: BOLD:ABX5689

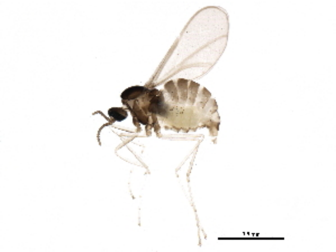

BIOUG22366-G11 [Lateral]
Asteromyia modesta
Family: Cecidomyiidae
BIN URI: BOLD:AAM1948

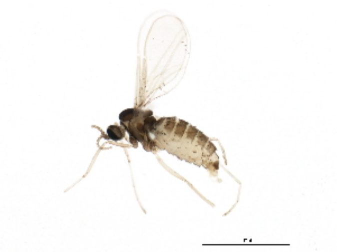

BIOUG01123-F03 [Lateral]
Asteromyia modesta
Family: Cecidomyiidae
BIN URI: BOLD:AAM1954

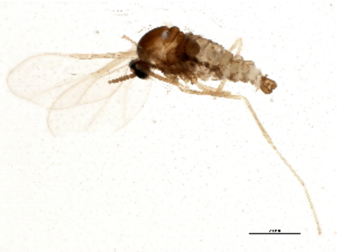

BIOUG01589-G11 [Lateral]
Asteromyia laeviana
Family: Cecidomyiidae
BIN URI: BOLD:ABV1420

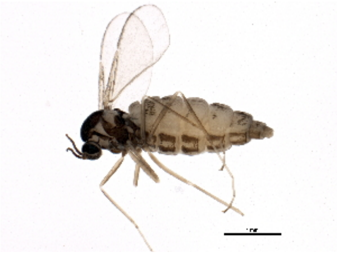

BIOUG02383-D02 [Lateral]
Asteromyia tumifica
Family: Cecidomyiidae
BIN URI: BOLD:ACL0470

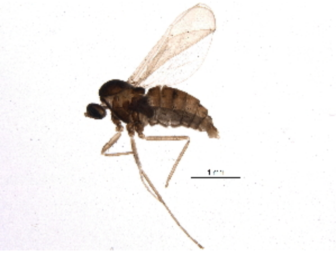

BIOUG10608-E07 [Lateral]
Asteromyia
Family: Cecidomyiidae
BIN URI: BOLD:ACL8441

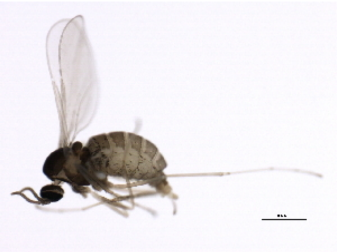

10JSROW-0358 [Lateral]
Asteromyia modesta
Family: Cecidomyiidae
BIN URI: BOLD:AAM1947

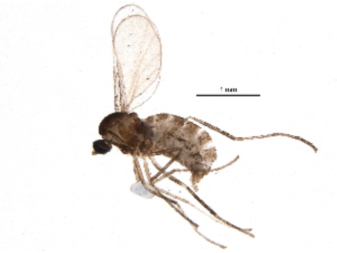

BIOUG09355-G04 [Lateral]
Asteromyia
Family: Cecidomyiidae
BIN URI: BOLD:ACN2213

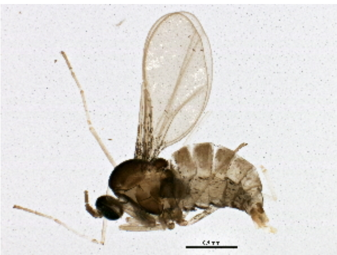

BIOUG02875-B07 [Lateral]
Asteromyia
Family: Cecidomyiidae
BIN URI: BOLD:ACB3163

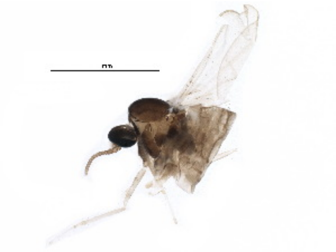

BIOUG22415-G04 [Lateral]
Asteromyia
Family: Cecidomyiidae
BIN URI: BOLD:ACV3990

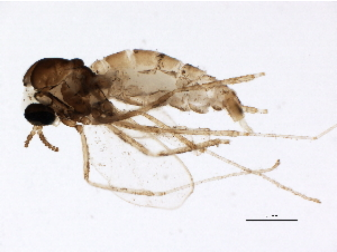

BIOUG01609-F10 [Lateral]
Cecidomyiidae
Family: Cecidomyiidae
BIN URI: BOLD:AAV5562

IMAGE NOT AVAILABLE

BIOUG22237-A11
Chironomidae
Family: Chironomidae

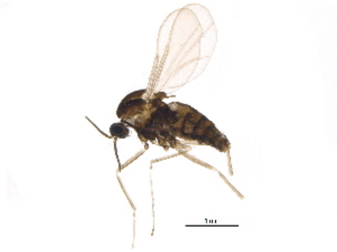

BIOUG22323-B05 [Lateral]
Cecidomyiidae
Family: Cecidomyiidae
BIN URI: BOLD:ACV3571

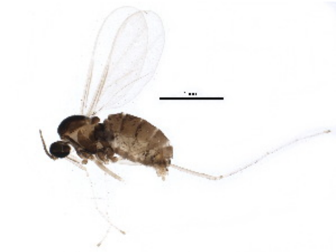

BIOUG22416-F09 [Lateral]
Cecidomyiidae
Family: Cecidomyiidae
BIN URI: BOLD:ACV4280

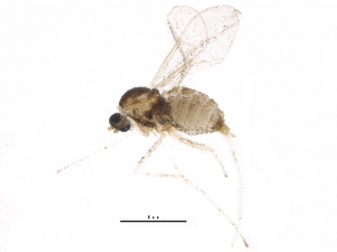

BIOUG20955-G11 [Lateral]
Diptera

BIN URI: BOLD:ACU6359

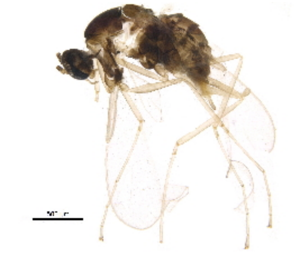

BIOUG22468-D09 [Lateral]
Asteromyia
Family: Cecidomyiidae
BIN URI: BOLD:ACV5159

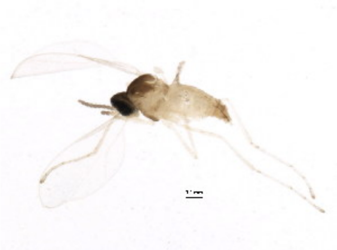

BIOUG01345-H06 [Lateral]
Cecidomyiidae
Family: Cecidomyiidae
BIN URI: BOLD:AAN5233

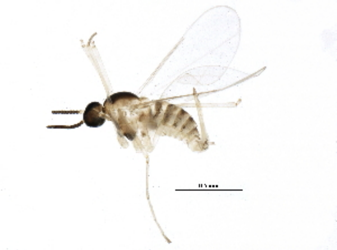

BIOUG22084-H05 [Lateral]
Cecidomyiidae
Family: Cecidomyiidae
BIN URI: BOLD:ACV4277

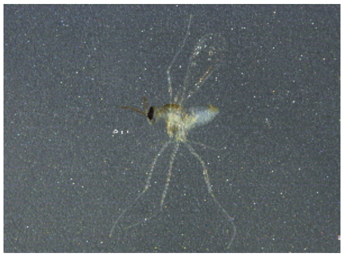

BIOUG02623-H10 [Lateral]
Cecidomyiidae
Family: Cecidomyiidae
BIN URI: BOLD:ABA0859

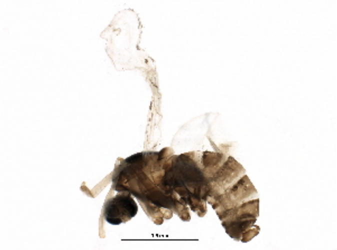

BIOUG22459-H01 [Lateral]
Cecidomyiidae
Family: Cecidomyiidae
BIN URI: BOLD:ACV4447

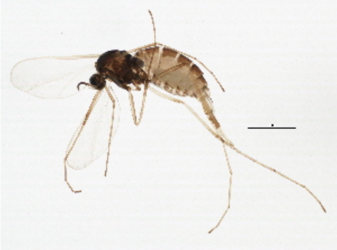

BIOUG02601-G11 [Lateral]
Cecidomyiidae
Family: Cecidomyiidae
BIN URI: BOLD:ABW7797

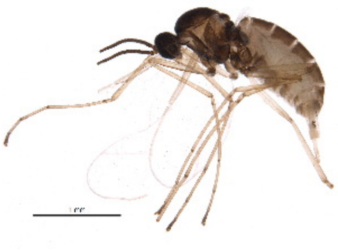

BIOUG22454-E01 [Lateral]
Cecidomyiidae
Family: Cecidomyiidae
BIN URI: BOLD:ACV4374

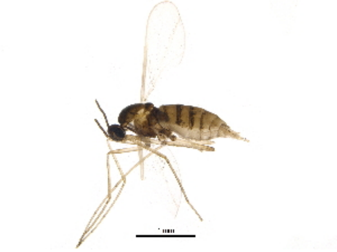

BIOUG22468-C05 [Lateral]
Cecidomyiidae
Family: Cecidomyiidae
BIN URI: BOLD:ACV6040

IMAGE NOT AVAILABLE

BIOUG22299-H01
Cecidomyiidae
Family: Cecidomyiidae

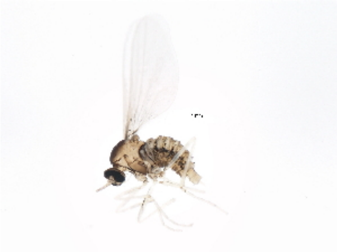

10JSROW-1593 [Lateral]
Cecidomyiidae
Family: Cecidomyiidae
BIN URI: BOLD:AAV5792

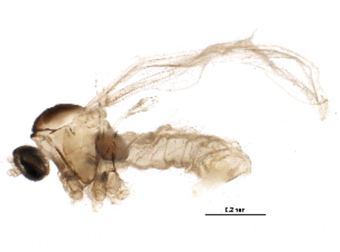

BIOUG10829-E05 [Lateral]
Cecidomyiidae
Family: Cecidomyiidae
BIN URI: BOLD:AAU6609

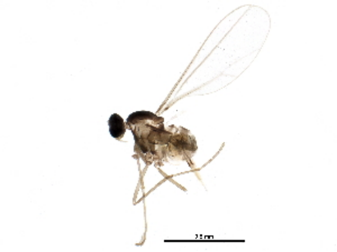

BIOUG22084-F02 [Lateral]
Cecidomyiidae
Family: Cecidomyiidae
BIN URI: BOLD:AAV5329

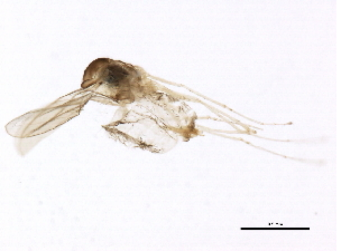

BIOUG01119-H08 [Lateral]
Cecidomyiidae
Family: Cecidomyiidae
BIN URI: BOLD:AAY6457

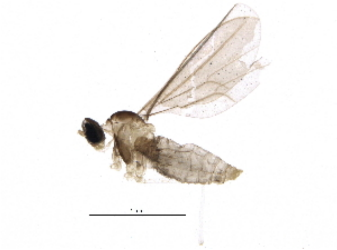

BIOUG06867-G12 [Lateral]
Cecidomyiinae
Family: Cecidomyiidae
BIN URI: BOLD:ACI5257

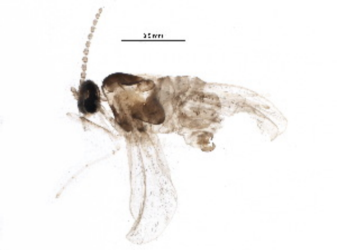

BIOUG22731-B08 [Lateral]
Cecidomyiidae
Family: Cecidomyiidae
BIN URI: BOLD:ACV5556

IMAGE NOT AVAILABLE

BIOUG22729-F11
Cecidomyiidae
Family: Cecidomyiidae

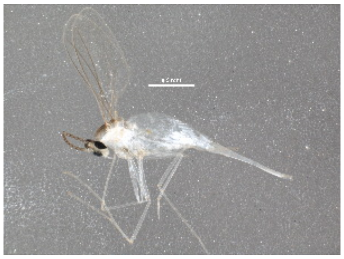

BIOUG22722-C07 [Lateral]
Cecidomyiidae
Family: Cecidomyiidae
BIN URI: BOLD:ACV5705

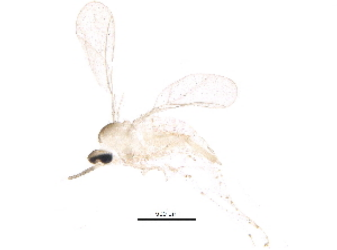

BIOUG23082-A11 [Lateral]
Cecidomyiidae
Family: Cecidomyiidae
BIN URI: BOLD:ACW1240

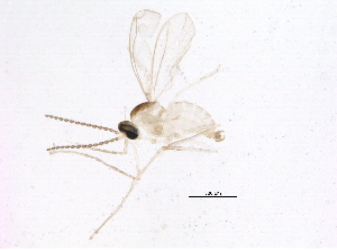

BIOUG03752-F12 [Lateral]
Cecidomyiidae
Family: Cecidomyiidae
BIN URI: BOLD:ACC8788

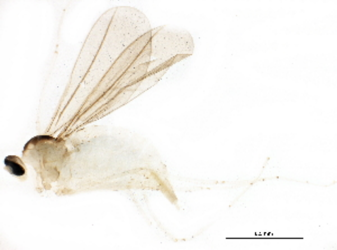

BIOUG22459-H03 [Lateral]
Dasineura
Family: Cecidomyiidae
BIN URI: BOLD:ABV0493

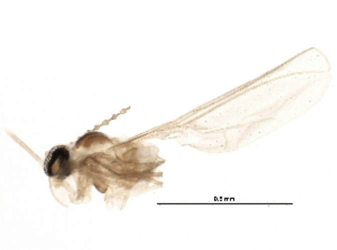

BIOUG22459-H02 [Lateral]
Cecidomyiidae
Family: Cecidomyiidae
BIN URI: BOLD:ACV3783

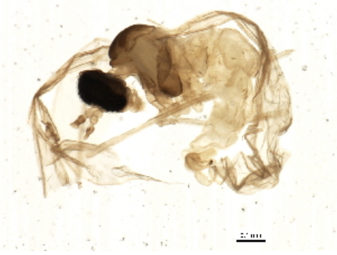

BIOUG01142-C08 [Lateral]
Cecidomyiidae
Family: Cecidomyiidae
BIN URI: BOLD:AAZ5620

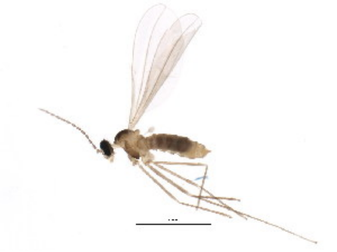

BIOUG00942-A06 [Lateral]
Cecidomyiidae
Family: Cecidomyiidae
BIN URI: BOLD:AAG3625

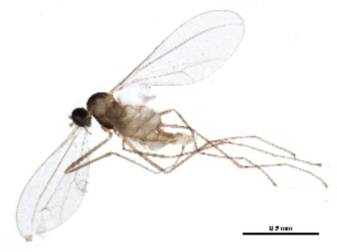

BIOUG23075-B10 [Lateral]
Cecidomyiidae
Family: Cecidomyiidae
BIN URI: BOLD:AAV5689

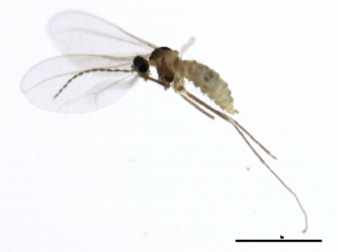

PCPP10-0820 [Lateral]
Cecidomyiidae
Family: Cecidomyiidae
BIN URI: BOLD:AAV5594

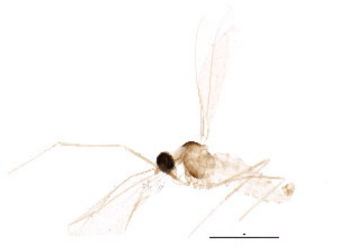

BIOUG01450-C10 [Lateral]
Cecidomyiidae
Family: Cecidomyiidae
BIN URI: BOLD:ABV9290

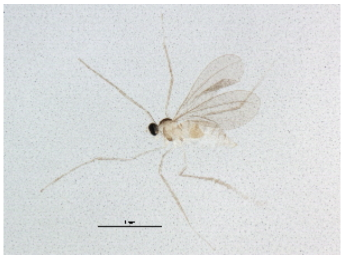

BIOUG03444-B01 [Lateral]
Cecidomyiidae
Family: Cecidomyiidae
BIN URI: BOLD:ACA6970

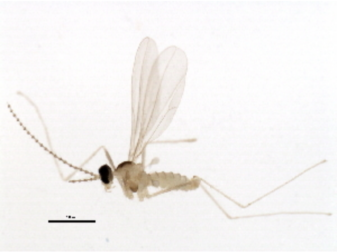

09BBDIP-0631 [Lateral]
Cecidomyiidae
Family: Cecidomyiidae
BIN URI: BOLD:AAH3655

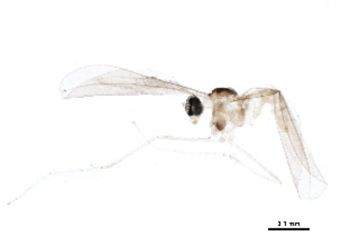

BIOUG23318-C11 [Lateral]
Cecidomyiidae
Family: Cecidomyiidae
BIN URI: BOLD:ACW1049

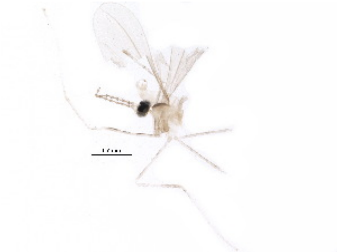

BIOUG21208-H01 [Lateral]
Diptera

BIN URI: BOLD:ABV1384

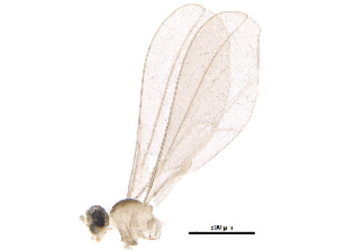

BIOUG23308-F03 [Lateral]
Cecidomyiidae
Family: Cecidomyiidae
BIN URI: BOLD:ACW1246

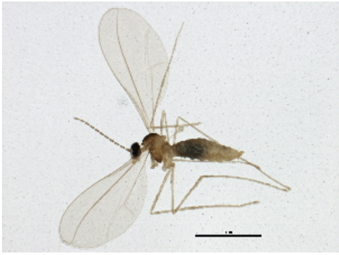

BIOUG01982-D03 [Lateral]
Cecidomyiidae
Family: Cecidomyiidae
BIN URI: BOLD:AAH3617

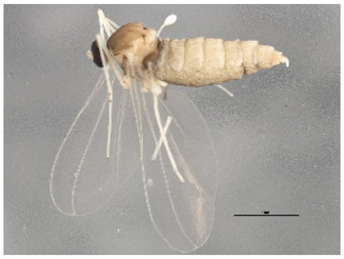

10JSROW-0681 [Lateral]
Cecidomyiidae
Family: Cecidomyiidae
BIN URI: BOLD:AAV5352

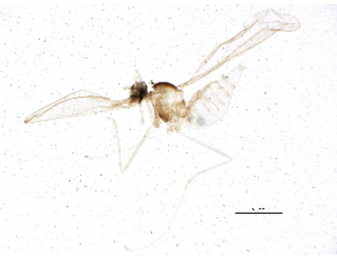

BIOUG01609-B09 [Lateral]
Cecidomyiidae
Family: Cecidomyiidae
BIN URI: BOLD:AAH3662

IMAGE NOT AVAILABLE

BIOUG23079-B11
Cecidomyiidae
Family: Cecidomyiidae

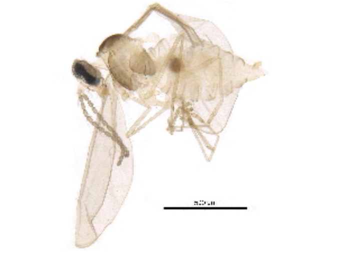

BIOUG22719-A07 [Lateral]
Cecidomyiidae
Family: Cecidomyiidae
BIN URI: BOLD:ACV4546

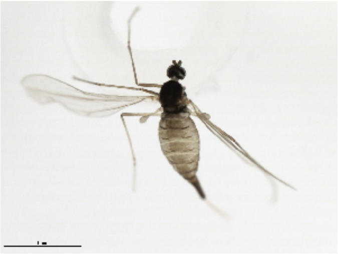

10JSROW-1395 [Dorsal]
Cecidomyiidae
Family: Cecidomyiidae
BIN URI: BOLD:AAV5319

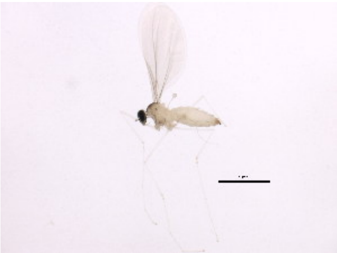

BIOUG00860-H06 [Lateral]
Cecidomyiidae
Family: Cecidomyiidae
BIN URI: BOLD:AAM7667

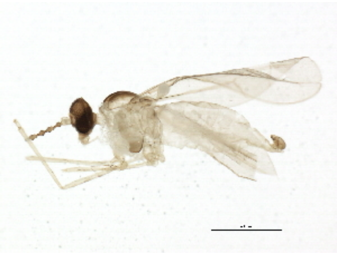

BIOUG01116-E06 [Lateral]
Cecidomyiidae
Family: Cecidomyiidae
BIN URI: BOLD:AAN5261

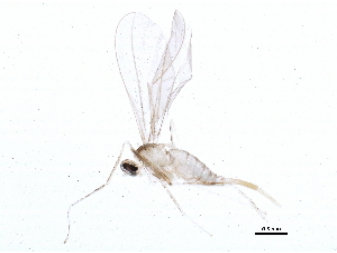

BIOUG10279-E04 [Lateral]
Cecidomyiidae
Family: Cecidomyiidae
BIN URI: BOLD:ABX8646

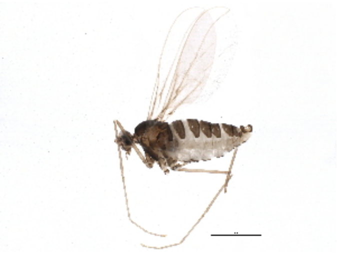

BIOUG13080-B03 [Lateral]
Cecidomyiidae
Family: Cecidomyiidae
BIN URI: BOLD:ACL0188

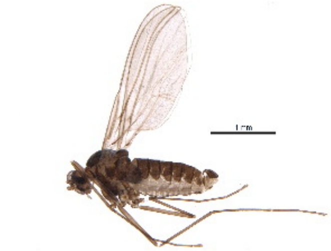

BIOUG22356-B10 [Lateral]
Cecidomyiidae
Family: Cecidomyiidae
BIN URI: BOLD:ACV4008

IMAGE NOT AVAILABLE

BIOUG22634-H07
Cecidomyiidae
Family: Cecidomyiidae

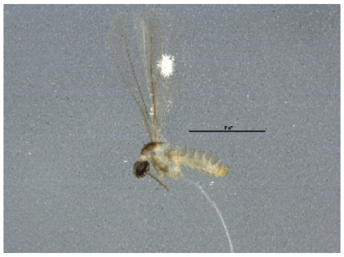

BIOUG04015-G10 [Lateral]
Cecidomyiidae
Family: Cecidomyiidae
BIN URI: BOLD:ABV0502

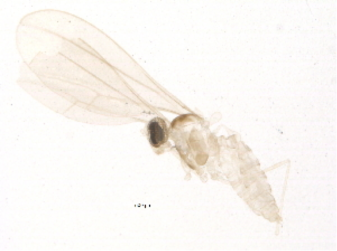

BIOUG04965-B08 [Lateral]
Cecidomyiidae
Family: Cecidomyiidae
BIN URI: BOLD:ACF6207

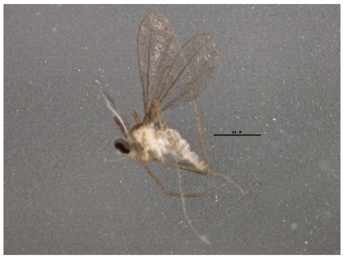

BIOUG03562-E01 [Lateral]
Cecidomyiidae
Family: Cecidomyiidae
BIN URI: BOLD:ACA9953

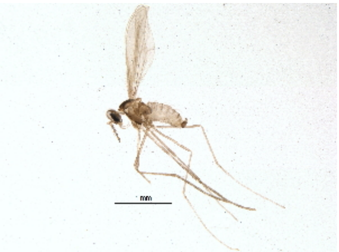

BIOUG10504-A09 [Lateral]
Cecidomyiidae
Family: Cecidomyiidae
BIN URI: BOLD:ACM2272

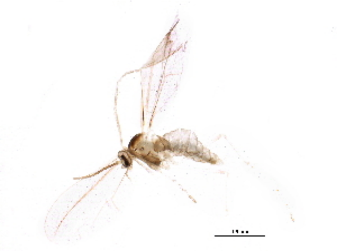

BIOUG10604-H01 [Lateral]
Cecidomyiidae
Family: Cecidomyiidae
BIN URI: BOLD:ACV4516

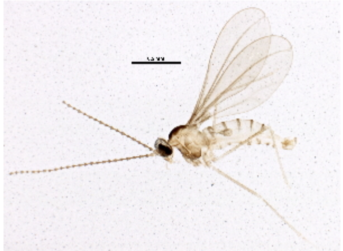

BIOUG03763-D03 [Lateral]
Cecidomyiidae
Family: Cecidomyiidae
BIN URI: BOLD:ACB2053

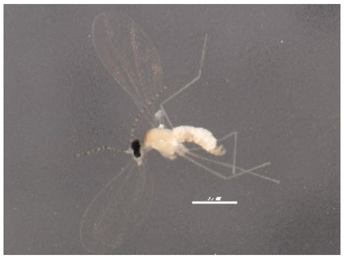

BIOUG01460-A09 [Lateral]
Cecidomyiidae
Family: Cecidomyiidae
BIN URI: BOLD:ACC5547

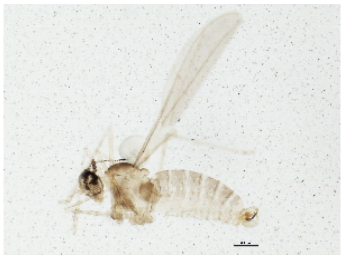

BIOUG03636-E04 [Lateral]
Cecidomyiidae
Family: Cecidomyiidae
BIN URI: BOLD:ACC7889

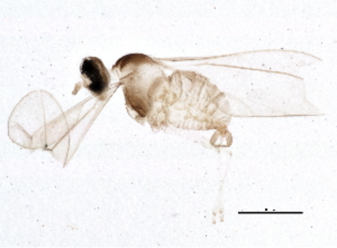

BIOUG02928-D11 [Lateral]
Cecidomyiidae
Family: Cecidomyiidae
BIN URI: BOLD:AAY6396

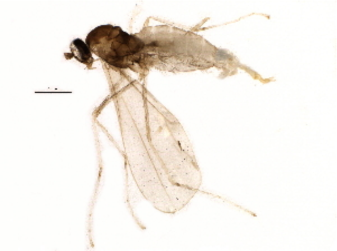

BIOUG10707-A01 [Lateral]
Cecidomyiidae
Family: Cecidomyiidae
BIN URI: BOLD:ACL8670

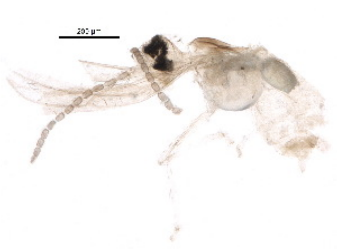

BIOUG21140-D04 [Lateral]
Diptera

BIN URI: BOLD:ACW0868

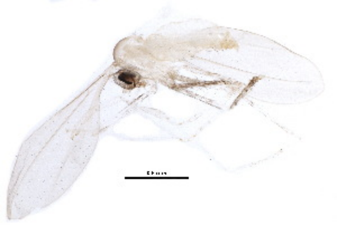

BIOUG22722-D06 [Lateral]
Cecidomyiidae
Family: Cecidomyiidae
BIN URI: BOLD:ABV1369

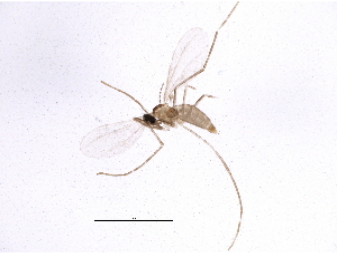

BIOUG04085-B07 [Lateral]
Cecidomyiidae
Family: Cecidomyiidae
BIN URI: BOLD:AAN5225

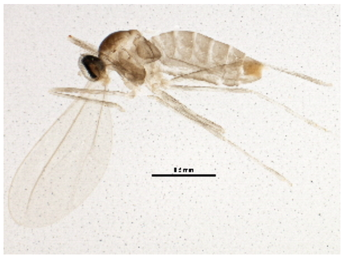

BIOUG03750-B11 [Lateral]
Cecidomyiidae
Family: Cecidomyiidae
BIN URI: BOLD:ACA6930

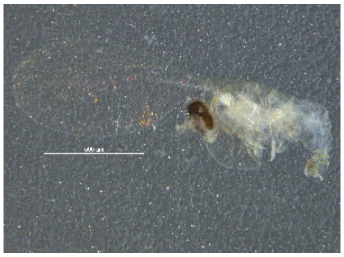

BIOUG02851-F05 [Lateral]
Cecidomyiidae
Family: Cecidomyiidae
BIN URI: BOLD:ACF3692

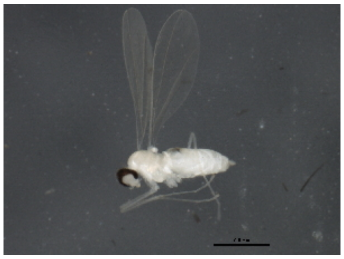

BIOUG00915-G03 [Lateral]
Cecidomyiidae
Family: Cecidomyiidae
BIN URI: BOLD:AAH3691

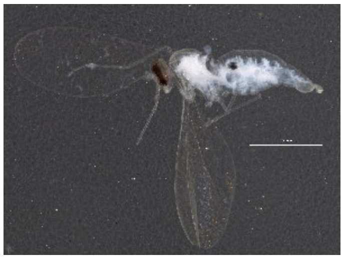

BIOUG01507-C08 [Lateral]
Cecidomyiidae
Family: Cecidomyiidae
BIN URI: BOLD:ACF3694

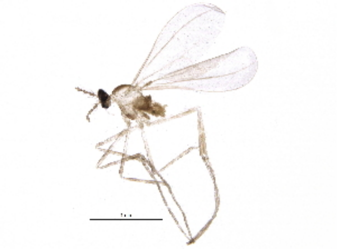

BIOUG08629-B05 [Lateral]
Cecidomyiidae
Family: Cecidomyiidae
BIN URI: BOLD:ACK2185

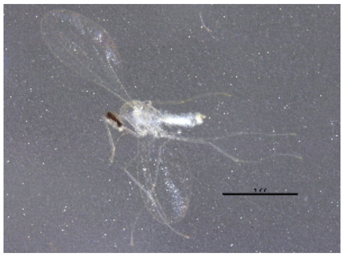

BIOUG08607-F10 [Lateral]
Cecidomyiidae
Family: Cecidomyiidae
BIN URI: BOLD:ACK3192

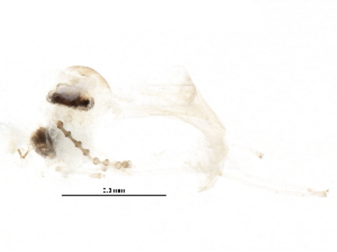

BIOUG22732-D04 [Lateral]
Cecidomyiidae
Family: Cecidomyiidae
BIN URI: BOLD:ACV4879

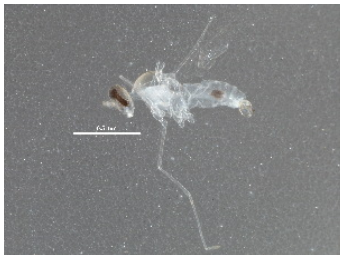

BIOUG22467-H07 [Lateral]
Cecidomyiidae
Family: Cecidomyiidae
BIN URI: BOLD:ACV4196

IMAGE NOT AVAILABLE

BIOUG22725-B11
Cecidomyiidae
Family: Cecidomyiidae
BIN URI: BOLD:ACV5488

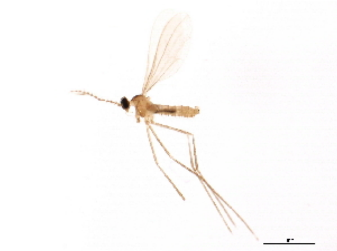

08TTML-2185 [Lateral]
Cecidomyiidae
Family: Cecidomyiidae
BIN URI: BOLD:AAN5271

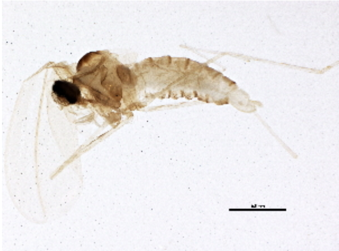

BIOUG01609-C06 [Lateral]
Cecidomyiidae
Family: Cecidomyiidae
BIN URI: BOLD:AAY6381

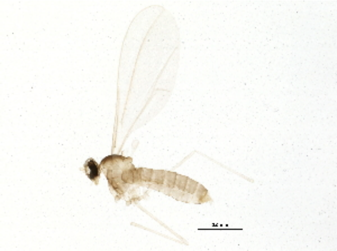

BIOUG01509-A06 [Lateral]
Cecidomyiidae
Family: Cecidomyiidae
BIN URI: BOLD:ABW8023

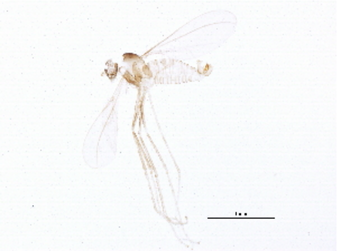

BIOUG01609-B07 [Lateral]
Cecidomyiidae
Family: Cecidomyiidae
BIN URI: BOLD:AAM6040

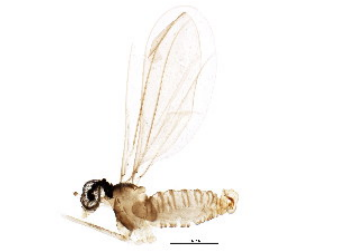

BIOUG01513-G09 [Lateral]
Cecidomyiidae
Family: Cecidomyiidae
BIN URI: BOLD:AAN5220

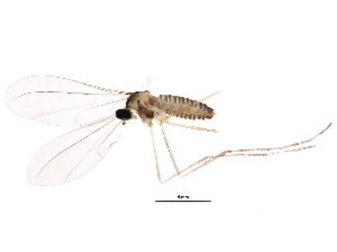

BIOUG01352-H07 [Lateral]
Cecidomyiidae
Family: Cecidomyiidae
BIN URI: BOLD:ABW7834

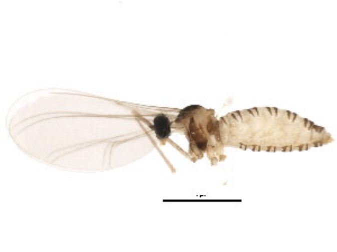

BIOUG01337-D01 [Lateral]
Cecidomyiidae
Family: Cecidomyiidae
BIN URI: BOLD:ABA7871

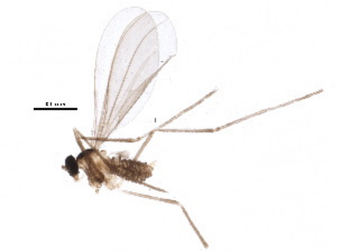

BIOUG23076-C05 [Lateral]
Cecidomyiidae
Family: Cecidomyiidae
BIN URI: BOLD:ACV5891

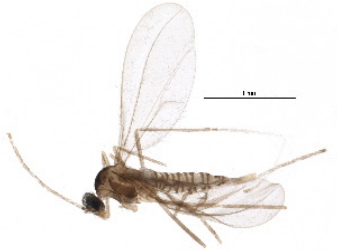

BIOUG22731-D08 [Lateral]
Cecidomyiidae
Family: Cecidomyiidae
BIN URI: BOLD:ACV5892

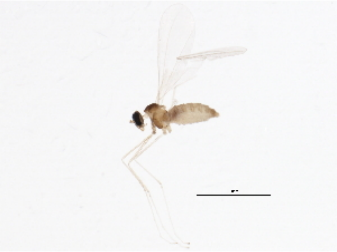

08TTML-1220 [Lateral]
Cecidomyiidae
Family: Cecidomyiidae
BIN URI: BOLD:AAN5198

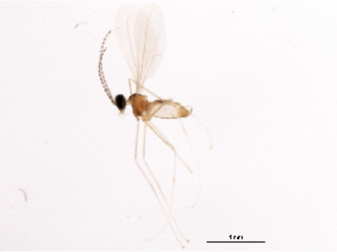

08TTML-2270 [Lateral]
Cecidomyiidae
Family: Cecidomyiidae
BIN URI: BOLD:AAN5276

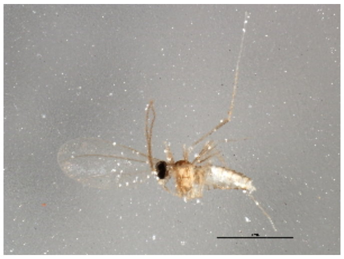

BIOUG02583-H11 [Lateral]
Cecidomyiidae
Family: Cecidomyiidae
BIN URI: BOLD:AAN5244

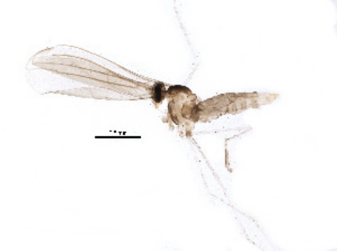

BIOUG21064-D08 [Lateral]
Diptera

BIN URI: BOLD:ACU9494

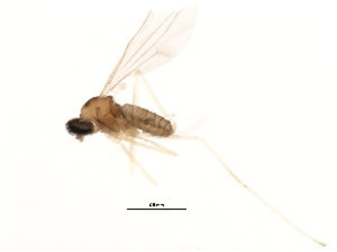

BIOUG01460-D11 [Lateral]
Cecidomyiidae
Family: Cecidomyiidae
BIN URI: BOLD:ABV9080

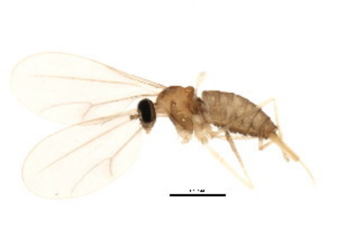

BIOUG01460-C09 [Lateral]
Cecidomyiidae
Family: Cecidomyiidae
BIN URI: BOLD:ABV0477

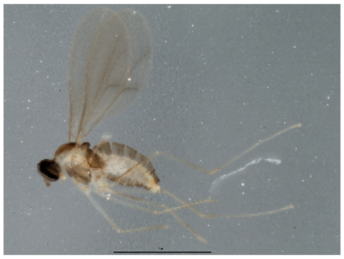

BIOUG02859-F10 [Lateral]
Cecidomyiidae
Family: Cecidomyiidae
BIN URI: BOLD:ACB3295

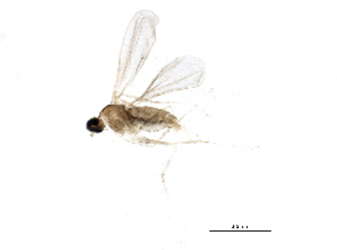

BIOUG23073-F07 [Lateral]
Cecidomyiidae
Family: Cecidomyiidae
BIN URI: BOLD:ACV5110

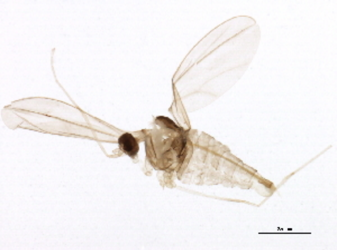

BIOUG01437-H09 [Lateral]
Cecidomyiidae
Family: Cecidomyiidae
BIN URI: BOLD:ABA1219

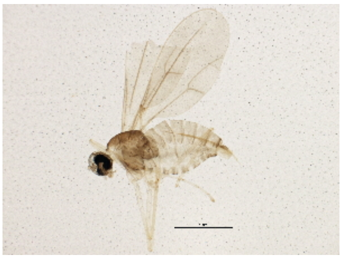

BIOUG03001-F06 [Lateral]
Cecidomyiidae
Family: Cecidomyiidae
BIN URI: BOLD:ACA6105

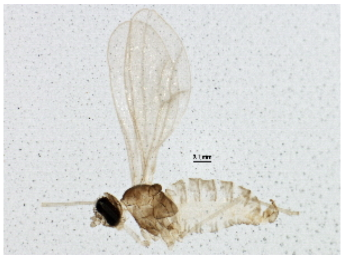

BIOUG02893-F10 [Lateral]
Cecidomyiidae
Family: Cecidomyiidae
BIN URI: BOLD:ACB5167

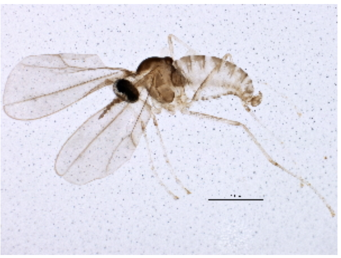

BIOUG03039-B11 [Lateral]
Cecidomyiidae
Family: Cecidomyiidae
BIN URI: BOLD:ACC8041

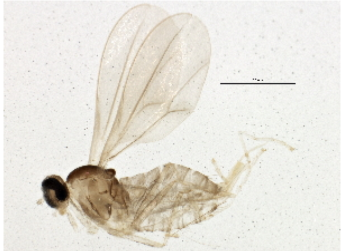

BIOUG03066-H06 [Lateral]
Cecidomyiidae
Family: Cecidomyiidae
BIN URI: BOLD:ABW2751

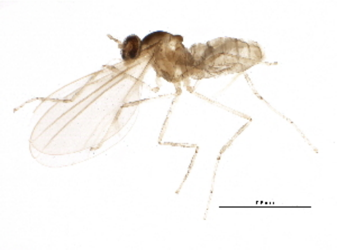

BIOUG22299-E07 [Lateral]
Cecidomyiidae
Family: Cecidomyiidae
BIN URI: BOLD:ACA3288

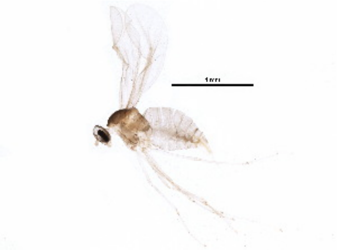

BIOUG22278-B08 [Lateral]
Cecidomyiidae
Family: Cecidomyiidae
BIN URI: BOLD:ACV3885

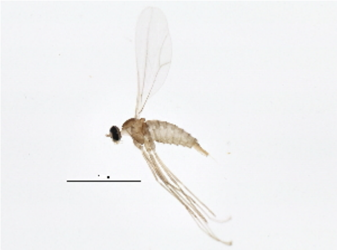

10JSROW-1756 [Lateral]
Cecidomyiidae
Family: Cecidomyiidae
BIN URI: BOLD:AAV5325

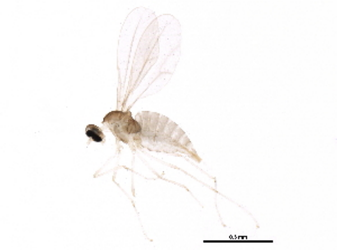

BIOUG22460-G01 [Lateral]
Cecidomyiidae
Family: Cecidomyiidae
BIN URI: BOLD:ACV3079

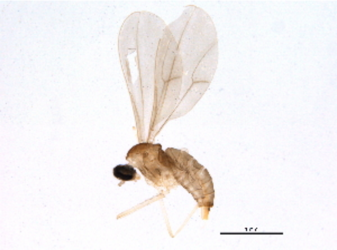

BIOUG07465-H04 [Lateral]
Cecidomyiidae
Family: Cecidomyiidae
BIN URI: BOLD:ABX7483

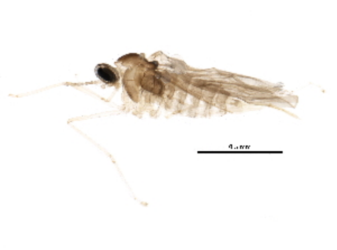

BIOUG22462-G10 [Lateral]
Cecidomyiidae
Family: Cecidomyiidae
BIN URI: BOLD:ACV3083

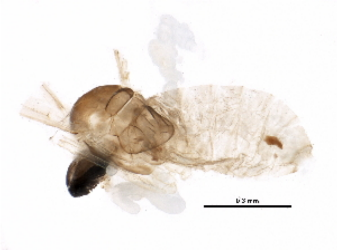

BIOUG22861-E06 [Lateral]
Cecidomyiidae
Family: Cecidomyiidae
BIN URI: BOLD:ACV4945

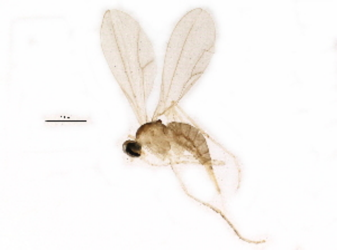

BIOUG09219-E10 [Lateral]
Cecidomyiinae
Family: Cecidomyiidae
BIN URI: BOLD:ACK8692

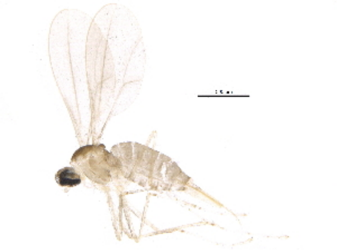

BIOUG23080-E04 [Lateral]
Cecidomyiidae
Family: Cecidomyiidae
BIN URI: BOLD:ACW1275

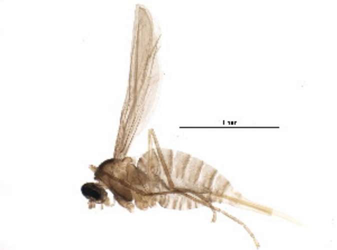

BIOUG23077-E12 [Lateral]
Cecidomyiidae
Family: Cecidomyiidae
BIN URI: BOLD:ACU6072

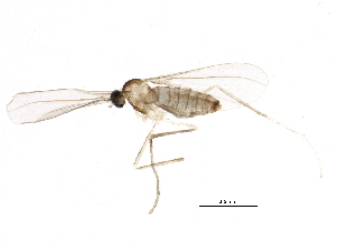

BIOUG23072-D12 [Lateral]
Cecidomyiidae
Family: Cecidomyiidae
BIN URI: BOLD:ACV5761

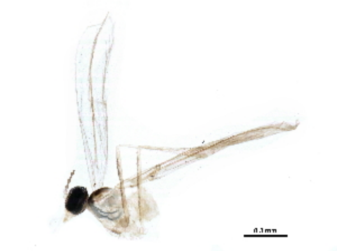

BIOUG23323-A03 [Lateral]
Cecidomyiidae
Family: Cecidomyiidae
BIN URI: BOLD:ACW1108

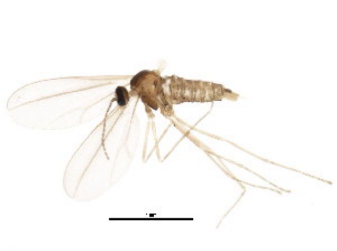

BIOUG01426-D01 [Lateral]
Cecidomyiidae
Family: Cecidomyiidae
BIN URI: BOLD:ACC5578

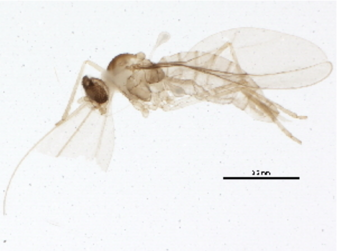

BIOUG01444-C04 [Lateral]
Cecidomyiidae
Family: Cecidomyiidae
BIN URI: BOLD:AAY6432

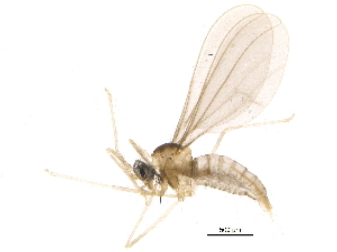

BIOUG22717-B02 [Lateral]
Cecidomyiidae
Family: Cecidomyiidae
BIN URI: BOLD:ACV4441

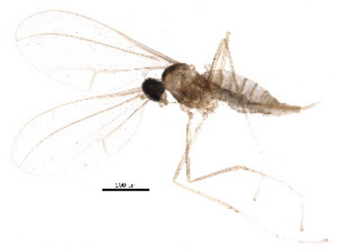

BIOUG23083-F05 [Lateral]
Cecidomyiidae
Family: Cecidomyiidae
BIN URI: BOLD:ACW1309

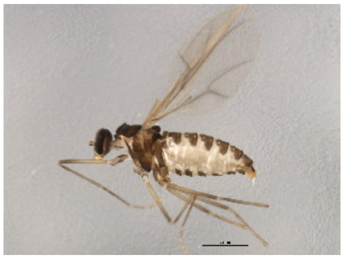

10JSROW-0712 [Lateral]
Cecidomyiidae
Family: Cecidomyiidae
BIN URI: BOLD:AAV5358

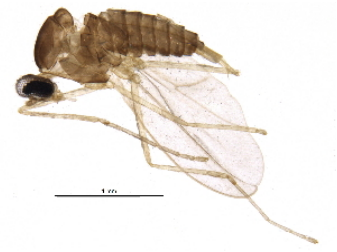

BIOUG08515-E12 [Lateral]
Cecidomyiidae
Family: Cecidomyiidae
BIN URI: BOLD:ACK1253

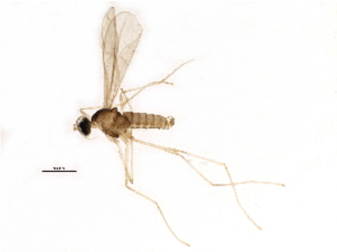

BIOUG08875-A11 [Lateral]
Cecidomyiinae
Family: Cecidomyiidae
BIN URI: BOLD:ACK5235

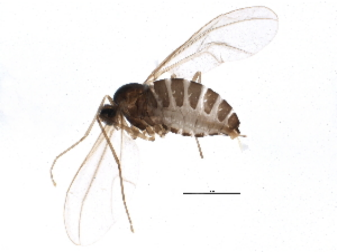

BIOUG13080-B09 [Lateral]
Cecidomyiidae
Family: Cecidomyiidae
BIN URI: BOLD:ACJ8925

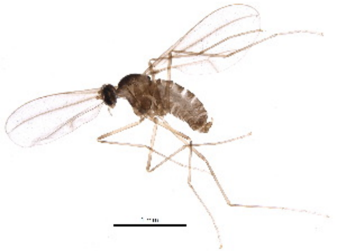

BIOUG22353-B04 [Lateral]
Cecidomyiidae
Family: Cecidomyiidae
BIN URI: BOLD:ACV4007

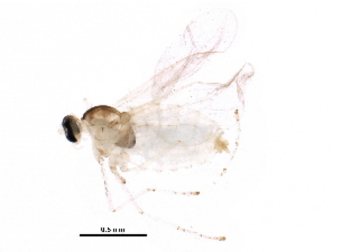

BIOUG22460-D11 [Lateral]
Cecidomyiidae
Family: Cecidomyiidae
BIN URI: BOLD:ACV3021

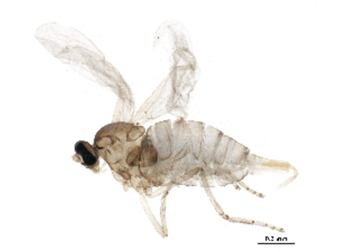

BIOUG21985-D09 [Lateral]
Cecidomyiidae
Family: Cecidomyiidae
BIN URI: BOLD:ACU5220

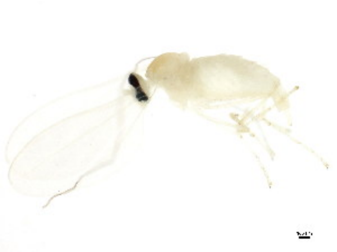

BIOUG01348-A05 [Lateral]
Cecidomyiidae
Family: Cecidomyiidae
BIN URI: BOLD:ABW5461

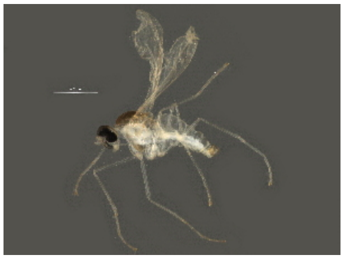

BIOUG08875-G12 [Lateral]
Cecidomyiidae
Family: Cecidomyiidae
BIN URI: BOLD:ACK7254

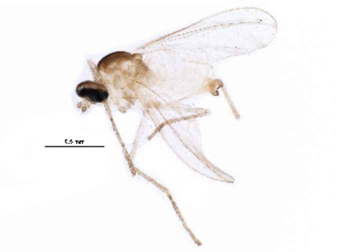

BIOUG22452-F03 [Lateral]
Cecidomyiidae
Family: Cecidomyiidae
BIN URI: BOLD:ACV5276

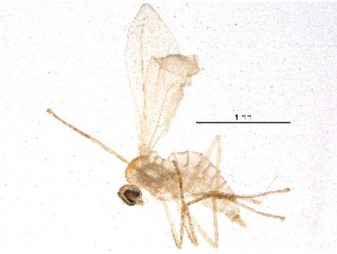

BIOUG08481-F01 [Lateral]
Cecidomyiidae
Family: Cecidomyiidae
BIN URI: BOLD:ACJ8520

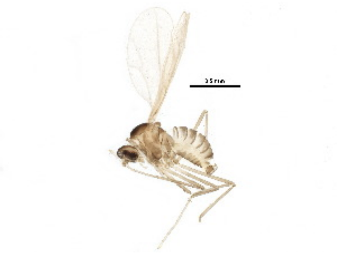

BIOUG22291-E09 [Lateral]
Cecidomyiidae
Family: Cecidomyiidae
BIN URI: BOLD:ACV3127

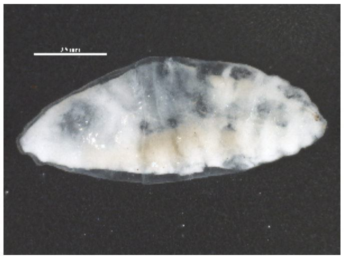

BIOUG22634-H06 [Larva]
Cecidomyiidae
Family: Cecidomyiidae
BIN URI: BOLD:ACW1780

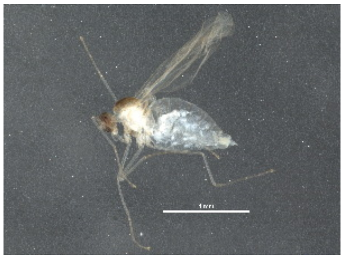

BIOUG22291-F10 [Lateral]
Cecidomyiidae
Family: Cecidomyiidae
BIN URI: BOLD:ACV1921

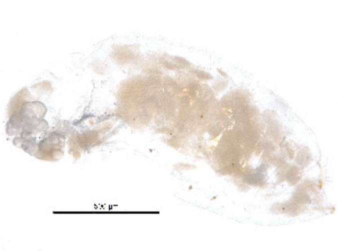

BIOUG22634-H05 [Larva]
Cecidomyiidae
Family: Cecidomyiidae
BIN URI: BOLD:ACW2016

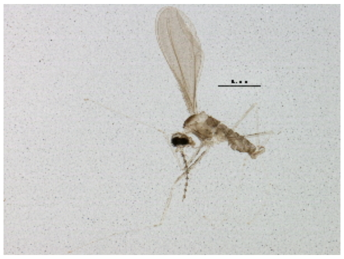

BIOUG03510-F08 [Lateral]
Cecidomyiidae
Family: Cecidomyiidae
BIN URI: BOLD:AAN5238

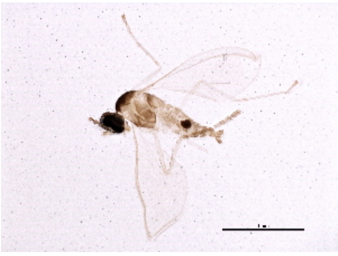

BIOUG03125-F09 [Lateral]
Cecidomyiidae
Family: Cecidomyiidae
BIN URI: BOLD:ACR5313

IMAGE NOT AVAILABLE

BIOUG23323-H05
Cecidomyiidae
Family: Cecidomyiidae
BIN URI: BOLD:ACW1135

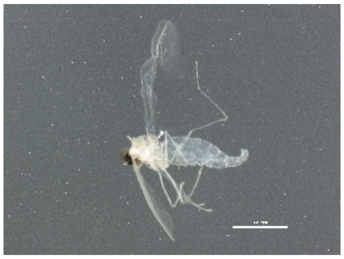
BIOUG01126-E04 [Lateral]
Cecidomyiidae
Family: Cecidomyiidae
BIN URI: BOLD:AAY6397

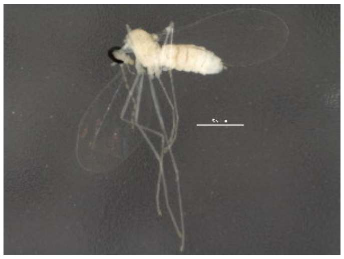
BIOUG01337-E08 [Lateral]
Cecidomyiidae
Family: Cecidomyiidae
BIN URI: BOLD:ABA7903

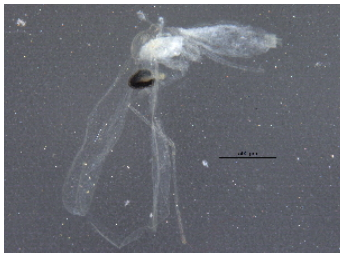
BIOUG06996-F09 [Lateral]
Cecidomyiidae
Family: Cecidomyiidae
BIN URI: BOLD:ACI6169

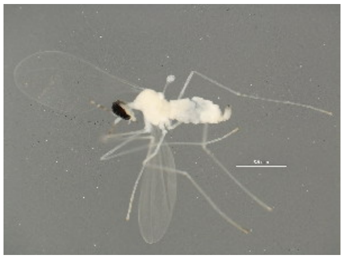
BIOUG01553-A03 [Lateral]
Cecidomyiidae
Family: Cecidomyiidae
BIN URI: BOLD:AAN5241

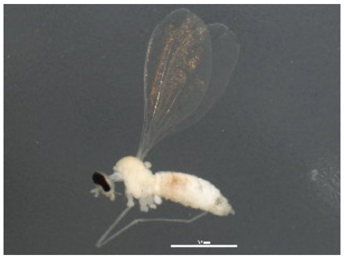
BIOUG00942-G06 [Lateral]
Cecidomyiidae
Family: Cecidomyiidae
BIN URI: BOLD:ABA0856

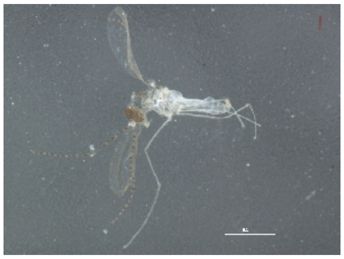
BIOUG01123-E12 [Lateral]
Cecidomyiidae
Family: Cecidomyiidae
BIN URI: BOLD:AAN5255

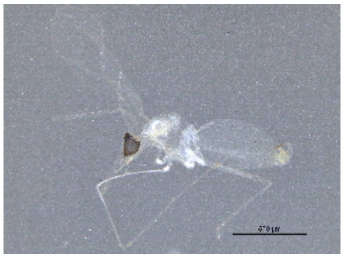
BIOUG23081-A07 [Lateral]
Cecidomyiidae
Family: Cecidomyiidae
BIN URI: BOLD:ACR0558

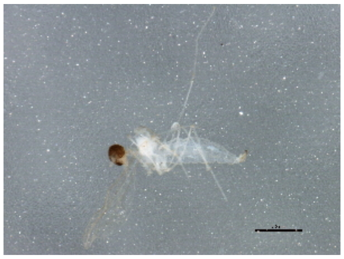
BIOUG01447-F05 [Lateral]
Cecidomyiidae
Family: Cecidomyiidae
BIN URI: BOLD:ABV0455

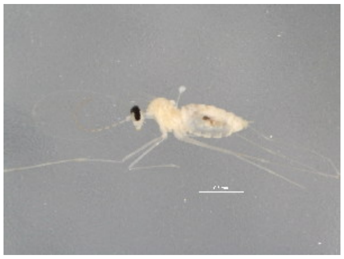
BIOUG01426-F04 [Lateral]
Cecidomyiidae
Family: Cecidomyiidae
BIN URI: BOLD:ACE7472

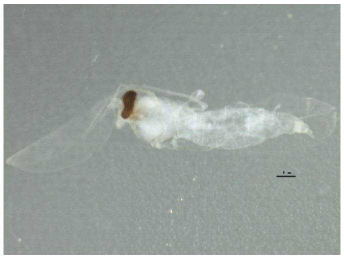
BIOUG01488-E06 [Lateral]
Cecidomyiidae
Family: Cecidomyiidae
BIN URI: BOLD:AAM6057

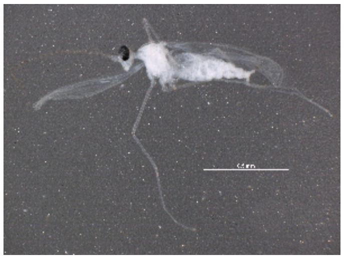
BIOUG22865-A02 [Lateral]
Cecidomyiidae
Family: Cecidomyiidae
BIN URI: BOLD:ACS1971

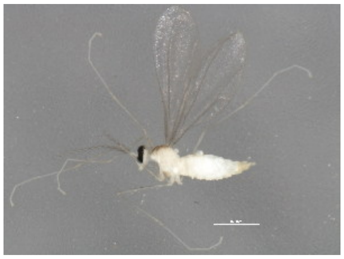
BIOUG00819-D12 [Lateral]
Cecidomyiidae
Family: Cecidomyiidae
BIN URI: BOLD:AAQ0296

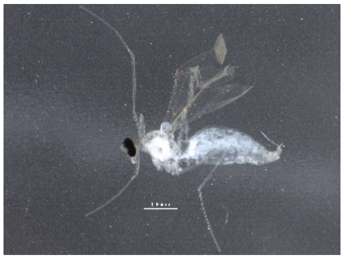
BIOUG20809-D08 [Lateral]
Diptera

BIN URI: BOLD:ACU6062

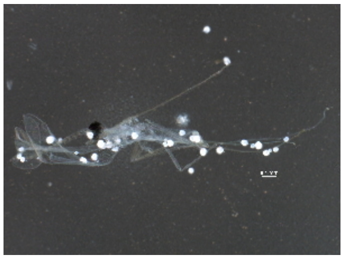
BIOUG21408-A04 [Lateral]
Diptera

BIN URI: BOLD:ACU6282

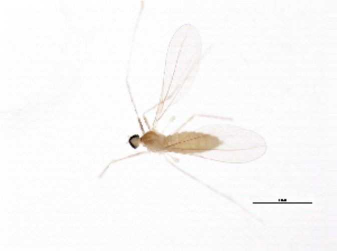
08TTML-0762 [Dorsal]
Cecidomyiidae
Family: Cecidomyiidae
BIN URI: BOLD:AAN5201

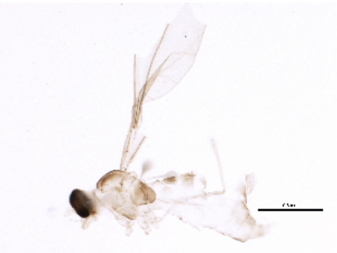
BIOUG01446-F12 [Lateral]
Cecidomyiidae
Family: Cecidomyiidae
BIN URI: BOLD:AAZ0297

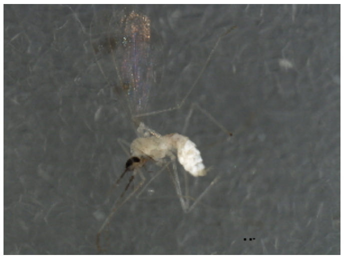
09BBEDI-1778 [Lateral]
Cecidomyiidae
Family: Cecidomyiidae
BIN URI: BOLD:AAH3734

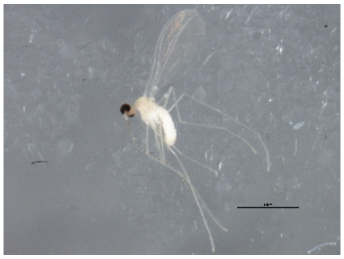
08TTML-1161 [Lateral]
Cecidomyiidae
Family: Cecidomyiidae
BIN URI: BOLD:AAN5227

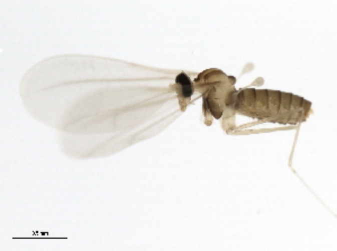
10JSROW-1370 [Lateral]
Cecidomyiidae
Family: Cecidomyiidae
BIN URI: BOLD:AAH3664

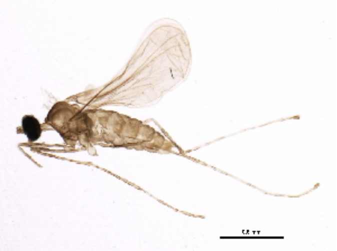
BIOUG01126-A05 [Lateral]
Cecidomyiidae
Family: Cecidomyiidae
BIN URI: BOLD:ACF4901

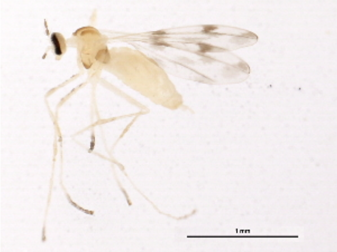

08TTML-2468 [Lateral]
Cecidomyiidae
Family: Cecidomyiidae
BIN URI: BOLD:AAN5183

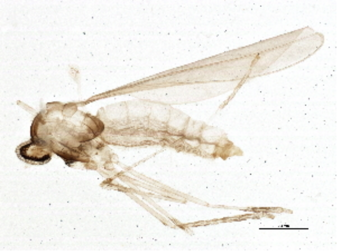

BIOUG02901-C01 [Lateral]
Cecidomyiidae
Family: Cecidomyiidae
BIN URI: BOLD:AAV5569

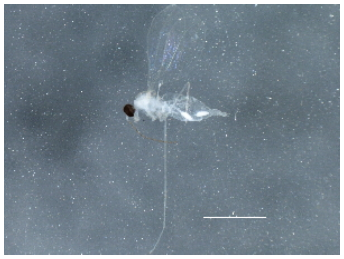

BIOUG01477-C12 [Lateral]
Cecidomyiidae
Family: Cecidomyiidae
BIN URI: BOLD:AAQ2553

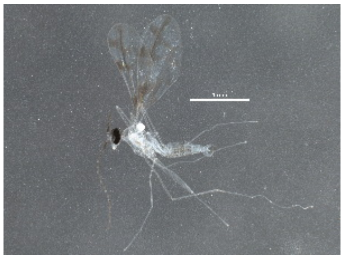

BIOUG22732-D10 [Lateral]
Cecidomyiidae
Family: Cecidomyiidae
BIN URI: BOLD:ACV4936

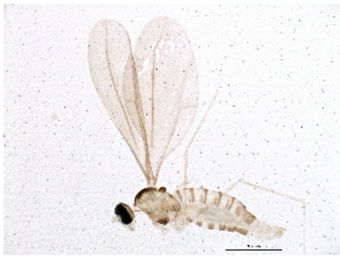

BIOUG02895-D01 [Lateral]
Cecidomyiidae
Family: Cecidomyiidae
BIN URI: BOLD:ACB4105

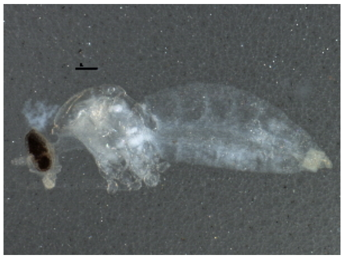

BIOUG02516-E07 [Lateral]
Cecidomyiidae
Family: Cecidomyiidae
BIN URI: BOLD:AAH3661

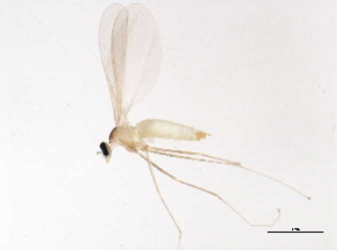

BIOUG00832-D11 [Dorsal]
Cecidomyiidae
Family: Cecidomyiidae
BIN URI: BOLD:AAV5568

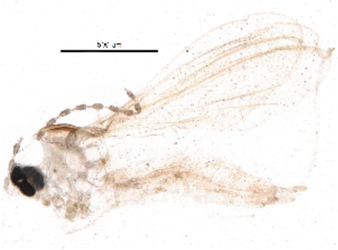

BIOUG23083-C08 [Lateral]
Cecidomyiidae
Family: Cecidomyiidae
BIN URI: BOLD:ACW0871

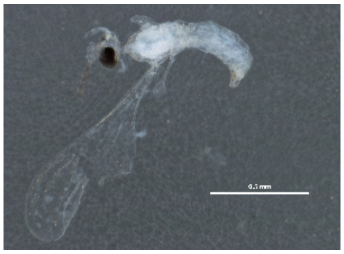

BIOUG02510-D01 [Lateral]
Cecidomyiidae
Family: Cecidomyiidae
BIN URI: BOLD:AAN5242

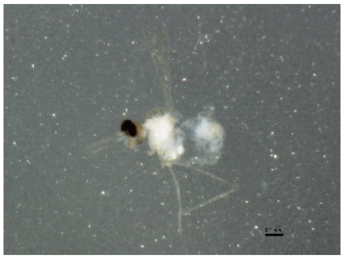

BIOUG00900-E11 [Lateral]
Cecidomyiidae
Family: Cecidomyiidae
BIN URI: BOLD:AAY6111

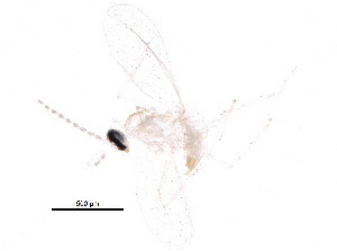

BIOUG21849-D07 [Lateral]
Diptera

BIN URI: BOLD:ACC8079

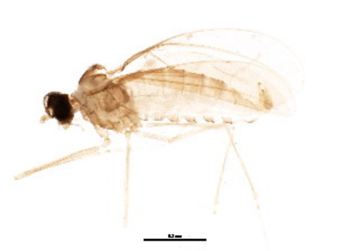

BIOUG01622-D10 [Lateral]
Cecidomyiidae
Family: Cecidomyiidae
BIN URI: BOLD:ABV1287

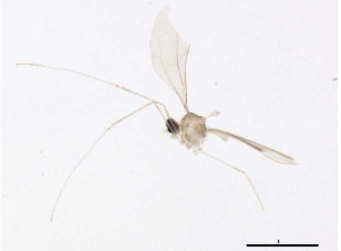

BIOUG01123-H06 [Lateral]
Cecidomyiidae
Family: Cecidomyiidae
BIN URI: BOLD:AAU6474

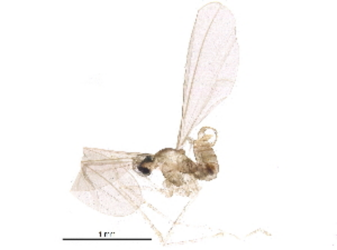

BIOUG23080-F01 [Lateral]
Cecidomyiidae
Family: Cecidomyiidae
BIN URI: BOLD:ACW1008

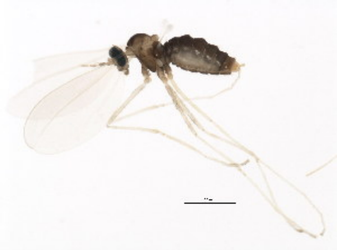

BIOUG00942-E09 [Lateral]
Cecidomyiidae
Family: Cecidomyiidae
BIN URI: BOLD:AAN5199

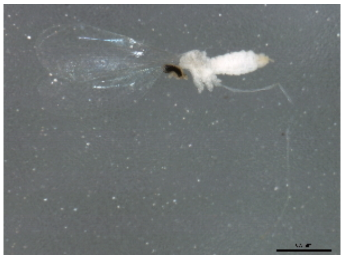

BIOUG01447-F12 [Lateral]
Cecidomyiidae
Family: Cecidomyiidae
BIN URI: BOLD:ABA0860

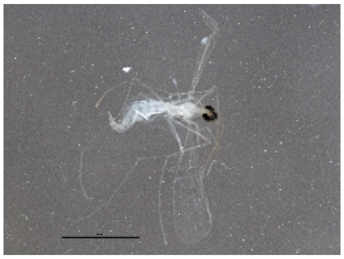

BIOUG02633-B03 [Lateral]
Cecidomyiidae
Family: Cecidomyiidae
BIN URI: BOLD:ABX7943

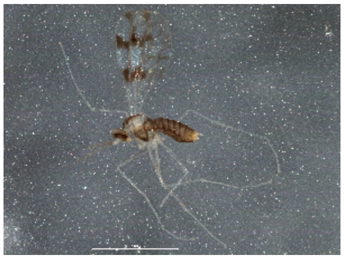

BIOUG02775-C02 [Lateral]
Cecidomyiidae
Family: Cecidomyiidae
BIN URI: BOLD:ACA1917

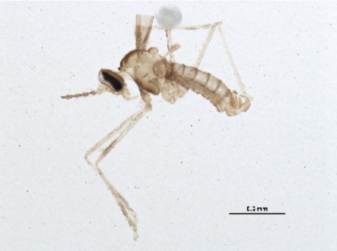

BIOUG10551-G08 [Lateral]
Cecidomyiidae
Family: Cecidomyiidae
BIN URI: BOLD:ACL6055

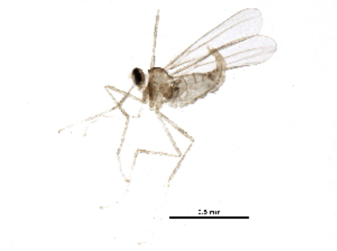

BIOUG23073-F12 [Lateral]
Cecidomyiidae
Family: Cecidomyiidae
BIN URI: BOLD:ACV5875

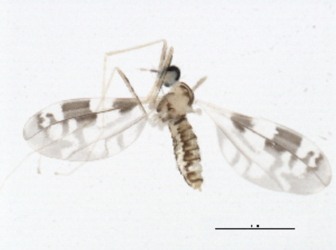

BIOUG00832-F05 [Dorsal]
Cecidomyiidae
Family: Cecidomyiidae
BIN URI: BOLD:AAH3760

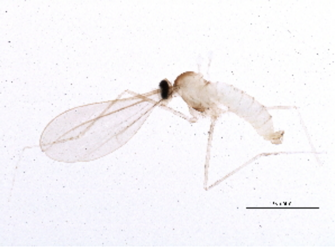

BIOUG10226-B10 [Lateral]
Cecidomyiidae
Family: Cecidomyiidae
BIN URI: BOLD:ACC5649

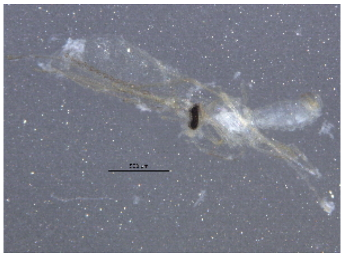

BIOUG08606-H06 [Lateral]
Cecidomyiidae
Family: Cecidomyiidae
BIN URI: BOLD:ABW5545

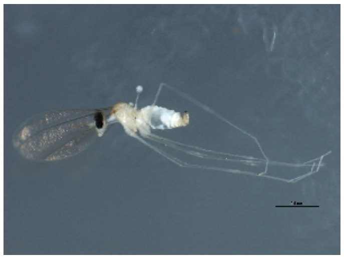

09BBEDI-2567 [Lateral]
Cecidomyiidae
Family: Cecidomyiidae
BIN URI: BOLD:ABY8401

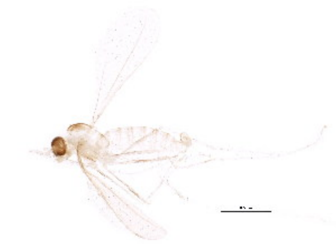

BIOUG01610-A11 [Lateral]
Cecidomyiidae
Family: Cecidomyiidae
BIN URI: BOLD:ABV1341

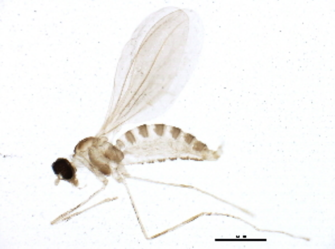

BIOUG01120-A04 [Lateral]
Cecidomyiidae
Family: Cecidomyiidae
BIN URI: BOLD:AAN5264

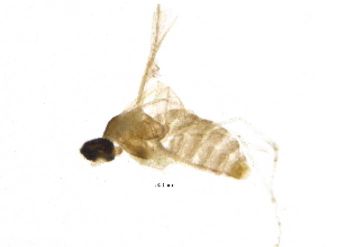

BIOUG02852-D12 [Lateral]
Cecidomyiidae
Family: Cecidomyiidae
BIN URI: BOLD:ACE6123

IMAGE NOT AVAILABLE

BIOUG10815-F12
Cecidomyiidae
Family: Cecidomyiidae
BIN URI: BOLD:ACL9909

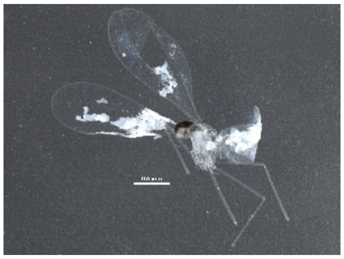

BIOUG22730-E03 [Lateral]
Cecidomyiidae
Family: Cecidomyiidae
BIN URI: BOLD:ACV6072

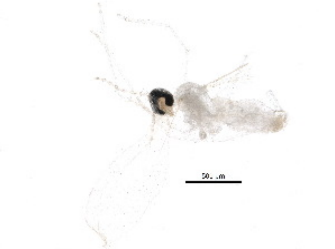

BIOUG24024-F05 [Lateral]
Cecidomyiidae
Family: Cecidomyiidae

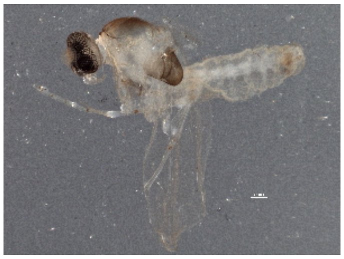

BIOUG04421-A12 [Lateral]
Cecidomyiidae
Family: Cecidomyiidae
BIN URI: BOLD:ACC8231

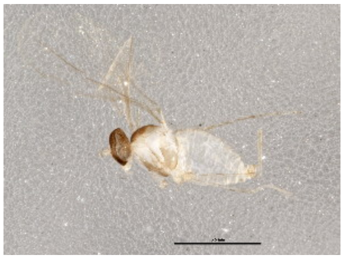

BIOUG01447-A02 [Lateral]
Cecidomyiidae
Family: Cecidomyiidae
BIN URI: BOLD:ABV9336

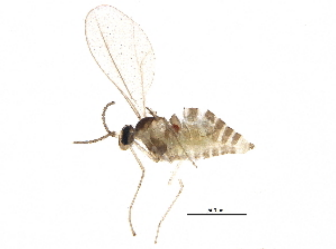

BIOUG16030-E06 [Lateral]
Cecidomyiidae
Family: Cecidomyiidae
BIN URI: BOLD:ACP9644

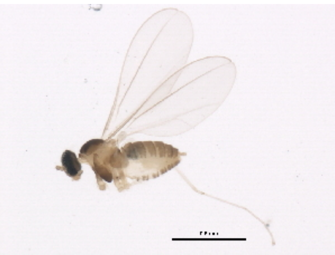

BIOUG01403-B10 [Lateral]
Cecidomyiidae
Family: Cecidomyiidae
BIN URI: BOLD:AAV5322

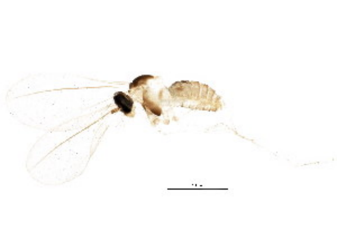

BIOUG01515-B01 [Lateral]
Cecidomyiidae
Family: Cecidomyiidae
BIN URI: BOLD:ABW8031

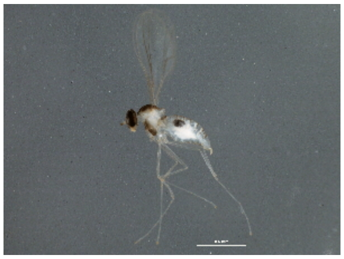

BIOUG01512-F05 [Lateral]
Cecidomyiidae
Family: Cecidomyiidae
BIN URI: BOLD:ABV1316

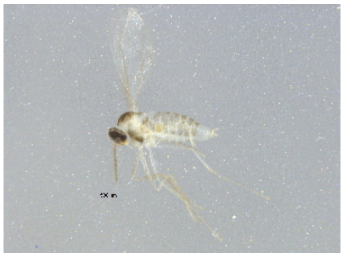

BIOUG03823-E06 [Lateral]
Cecidomyiidae
Family: Cecidomyiidae
BIN URI: BOLD:ACE9303

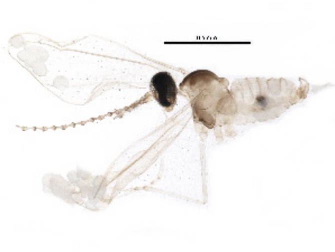

BIOUG22732-A10 [Lateral]
Cecidomyiidae
Family: Cecidomyiidae
BIN URI: BOLD:ACF4988

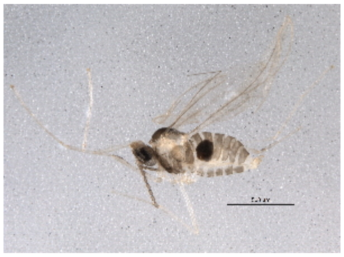

BIOUG09997-A08 [Lateral]
Cecidomyiidae
Family: Cecidomyiidae
BIN URI: BOLD:ACK2706

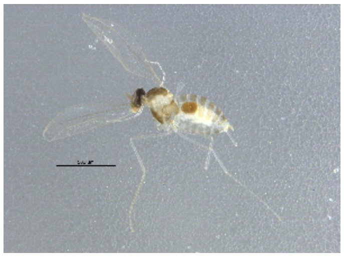

BIOUG03825-G11 [Lateral]
Cecidomyiidae
Family: Cecidomyiidae
BIN URI: BOLD:ACF0947

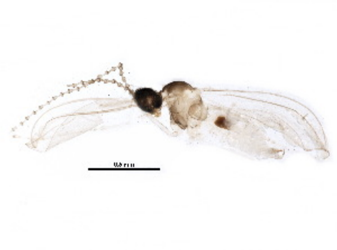

BIOUG22731-E04 [Lateral]
Cecidomyiidae
Family: Cecidomyiidae
BIN URI: BOLD:ACV5585

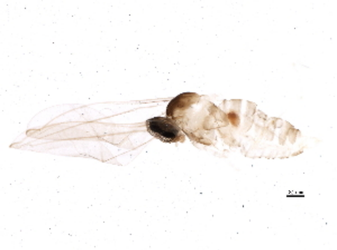

BIOUG01512-H01 [Lateral]
Cecidomyiidae
Family: Cecidomyiidae
BIN URI: BOLD:ABV0456

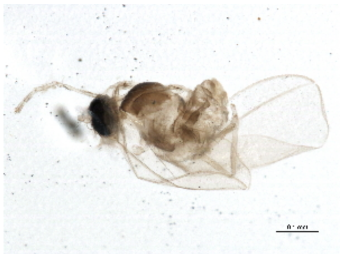

BIOUG12367-B09 [Lateral]
Cecidomyiidae
Family: Cecidomyiidae
BIN URI: BOLD:ACN1655

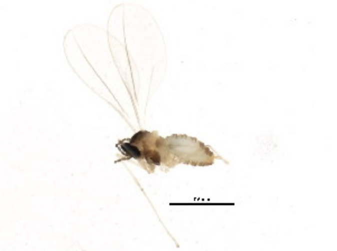

BIOUG01345-H10 [Lateral]
Cecidomyiidae
Family: Cecidomyiidae
BIN URI: BOLD:AAM6117

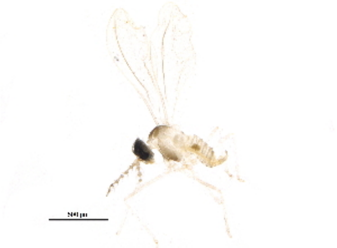

BIOUG22865-A10 [Lateral]
Cecidomyiidae
Family: Cecidomyiidae
BIN URI: BOLD:ABX8054

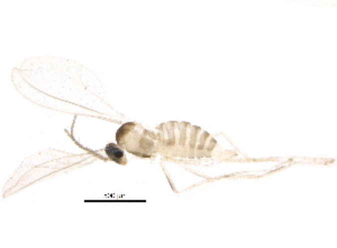

BIOUG22717-F08 [Lateral]
Cecidomyiidae
Family: Cecidomyiidae
BIN URI: BOLD:AAN5256

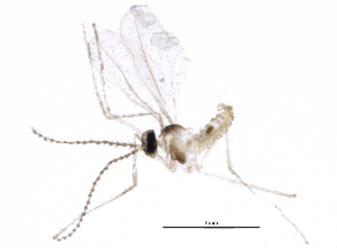

BIOUG08604-H09 [Lateral]
Cecidomyiidae
Family: Cecidomyiidae
BIN URI: BOLD:ACK2103

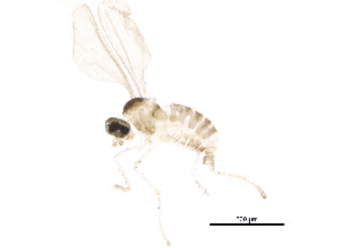

BIOUG23079-F01 [Lateral]
Cecidomyiidae
Family: Cecidomyiidae
BIN URI: BOLD:ACA7277

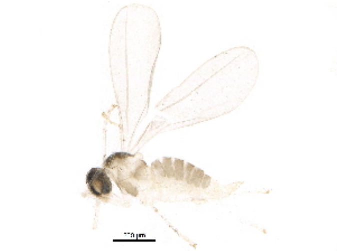

BIOUG23081-F03 [Lateral]
Cecidomyiidae
Family: Cecidomyiidae
BIN URI: BOLD:ACL9468

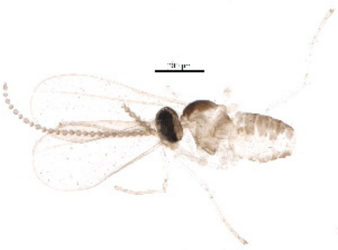

BIOUG23084-F02 [Lateral]
Cecidomyiidae
Family: Cecidomyiidae
BIN URI: BOLD:ACW0962

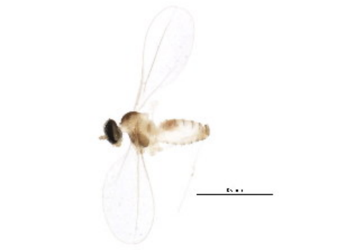

BIOUG01426-A01 [Lateral]
Cecidomyiidae
Family: Cecidomyiidae
BIN URI: BOLD:AAG8290

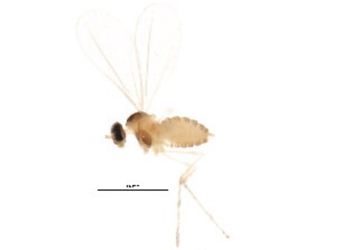

BIOUG01406-D04 [Lateral]
Cecidomyiidae
Family: Cecidomyiidae
BIN URI: BOLD:ACE3233

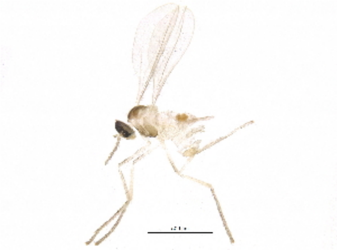

BIOUG09651-F06 [Lateral]
Cecidomyiidae
Family: Cecidomyiidae
BIN URI: BOLD:ACF3882

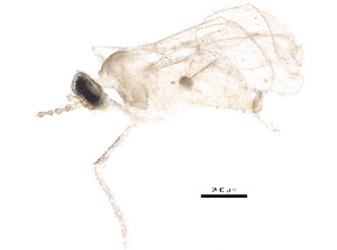

BIOUG23315-C12 [Lateral]
Cecidomyiidae
Family: Cecidomyiidae
BIN URI: BOLD:ACW1232

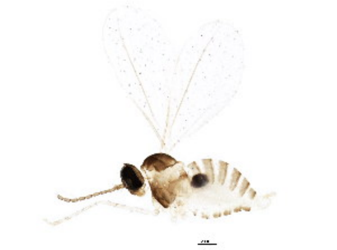

BIOUG01514-F03 [Lateral]
Cecidomyiidae
Family: Cecidomyiidae
BIN URI: BOLD:ABV9078

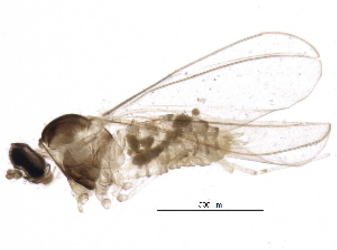

BIOUG12325-G03 [Lateral]
Cecidomyiidae
Family: Cecidomyiidae
BIN URI: BOLD:AAH3749

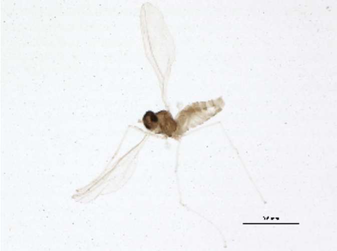

BIOUG01442-H10 [Lateral]
Cecidomyiidae
Family: Cecidomyiidae
BIN URI: BOLD:ABA0817

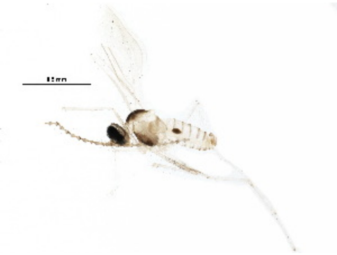

BIOUG22466-G10 [Lateral]
Cecidomyiidae
Family: Cecidomyiidae
BIN URI: BOLD:ABY8137

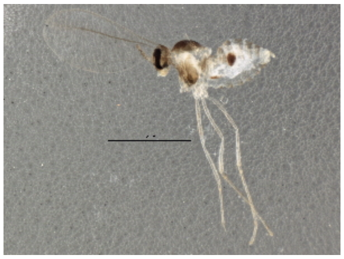

BIOUG03130-G12 [Lateral]
Cecidomyiidae
Family: Cecidomyiidae
BIN URI: BOLD:ABZ7164

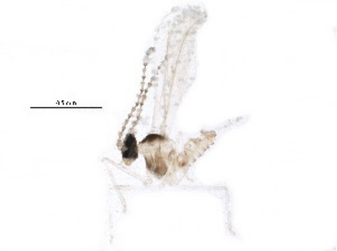

BIOUG22731-F03 [Lateral]
Cecidomyiidae
Family: Cecidomyiidae
BIN URI: BOLD:ACE3131

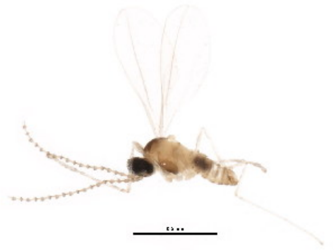

BIOUG01406-D12 [Lateral]
Cecidomyiidae
Family: Cecidomyiidae
BIN URI: BOLD:ACE8735

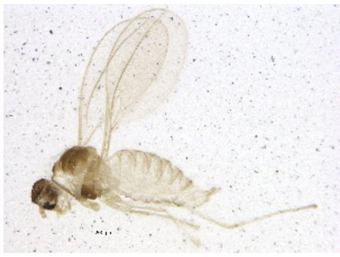

BIOUG01123-C02 [Lateral]
Cecidomyiidae
Family: Cecidomyiidae
BIN URI: BOLD:ACE5281

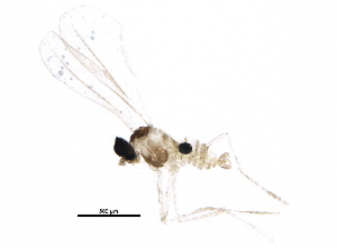

BIOUG06996-F07 [Lateral]
Cecidomyiidae
Family: Cecidomyiidae
BIN URI: BOLD:ACI3047

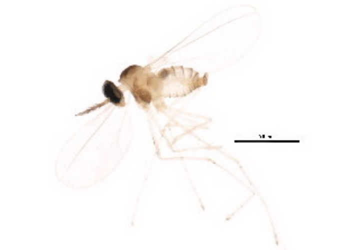

BIOUG01423-C01 [Lateral]
Cecidomyiidae
Family: Cecidomyiidae
BIN URI: BOLD:ACE5277

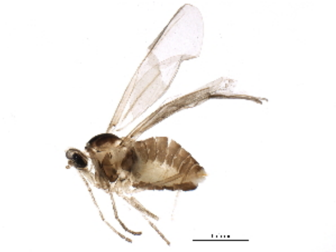

BIOUG22084-D10 [Lateral]
Cecidomyiidae
Family: Cecidomyiidae
BIN URI: BOLD:ABX6610

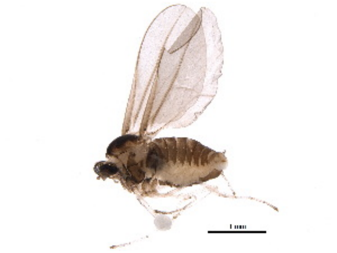

BIOUG22454-E02 [Lateral]
Cecidomyiidae
Family: Cecidomyiidae
BIN URI: BOLD:ACV3932

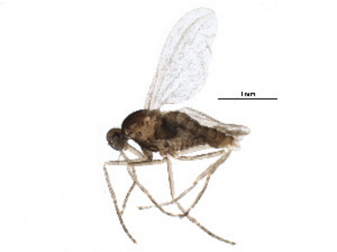

BIOUG22291-G10 [Lateral]
Cecidomyiidae
Family: Cecidomyiidae
BIN URI: BOLD:ACV1942

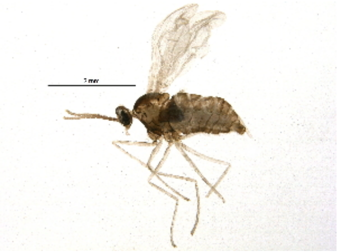

BIOUG10606-B06 [Lateral]
Cecidomyiidae
Family: Cecidomyiidae
BIN URI: BOLD:ACL6802

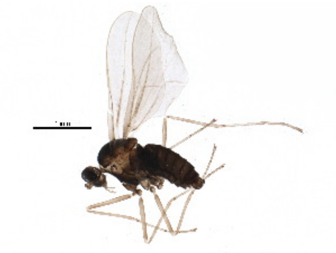

BIOUG22420-A03 [Lateral]
Cecidomyiidae
Family: Cecidomyiidae
BIN URI: BOLD:ACV1904

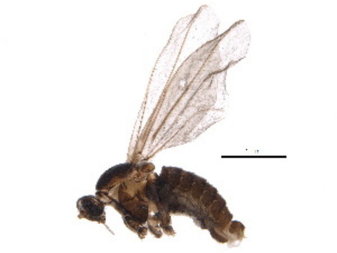

BIOUG22357-H05 [Lateral]
Cecidomyiidae
Family: Cecidomyiidae
BIN URI: BOLD:ACV3952

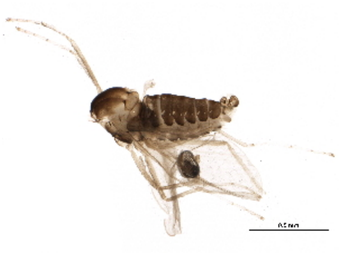

BIOUG22462-F02 [Lateral]
Cecidomyiidae
Family: Cecidomyiidae
BIN URI: BOLD:ACV3224

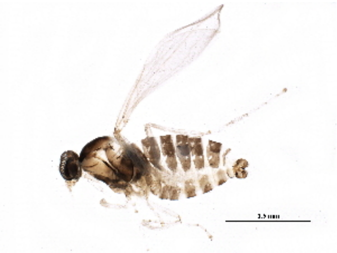

BIOUG22364-F06 [Lateral]
Cecidomyiidae
Family: Cecidomyiidae
BIN URI: BOLD:ACV4404

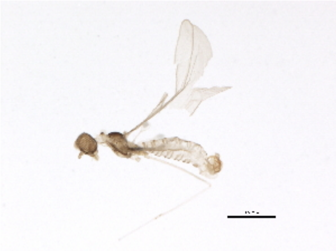

BIOUG01442-F05 [Lateral]
Cecidomyiidae
Family: Cecidomyiidae
BIN URI: BOLD:AAH3743

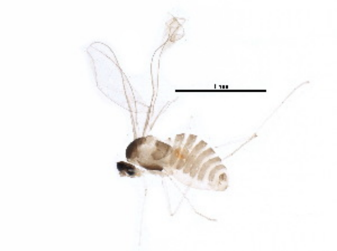

BIOUG23077-B11 [Lateral]
Orseolia
Family: Cecidomyiidae
BIN URI: BOLD:ACV5413

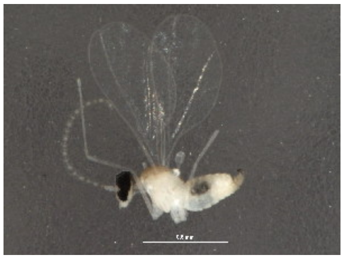

BIOUG01345-E06 [Lateral]
Cecidomyiidae
Family: Cecidomyiidae
BIN URI: BOLD:ABV1473

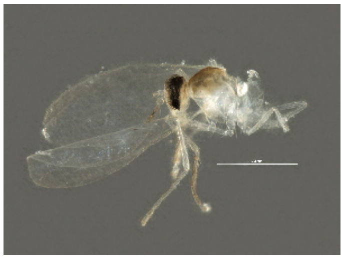

BIOUG08601-A11 [Lateral]
Cecidomyiidae
Family: Cecidomyiidae
BIN URI: BOLD:ACK1237

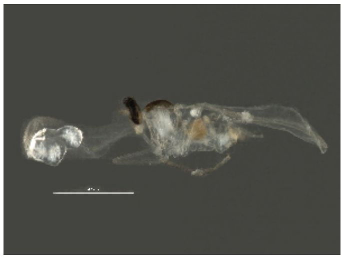

BIOUG08603-F03 [Lateral]
Cecidomyiidae
Family: Cecidomyiidae
BIN URI: BOLD:ACK1745

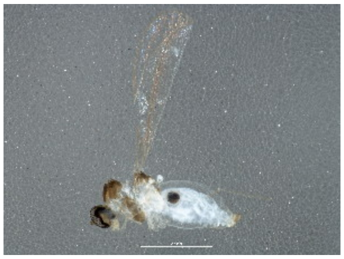

BIOUG01508-B09 [Lateral]
Cecidomyiidae
Family: Cecidomyiidae
BIN URI: BOLD:AAY6429

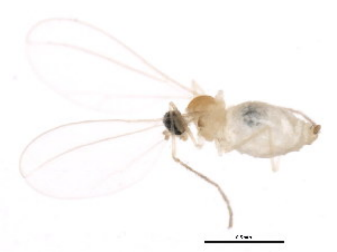

BIOUG00942-C06 [Lateral]
Cecidomyiidae
Family: Cecidomyiidae
BIN URI: BOLD:AAY6463

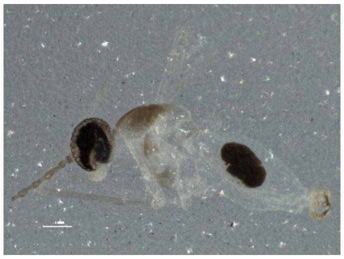

BIOUG02939-B12 [Lateral]
Cecidomyiidae
Family: Cecidomyiidae
BIN URI: BOLD:ACA5760

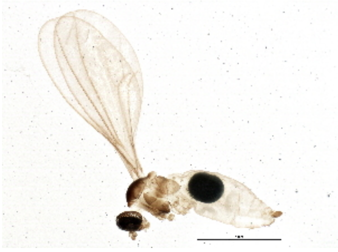

BIOUG02904-E03 [Lateral]
Cecidomyiidae
Family: Cecidomyiidae
BIN URI: BOLD:AAH3770

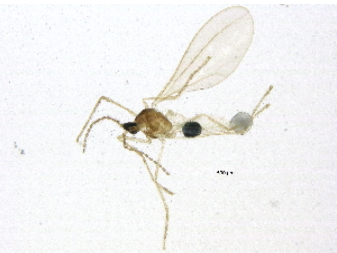

BIOUG03756-C07 [Lateral]
Cecidomyiidae
Family: Cecidomyiidae
BIN URI: BOLD:ABW8065

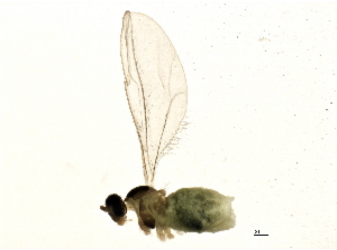

BIOUG10729-A02 [Lateral]
Cecidomyiidae
Family: Cecidomyiidae
BIN URI: BOLD:ACL1931

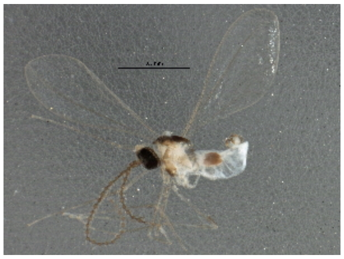

BIOUG03924-B09 [Lateral]
Cecidomyiidae
Family: Cecidomyiidae
BIN URI: BOLD:ACA6463

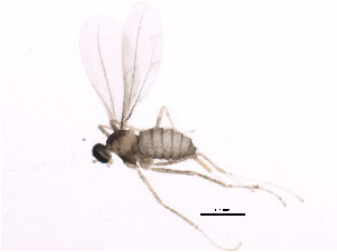

BIOUG00860-F07 [Lateral]
Cecidomyiidae
Family: Cecidomyiidae
BIN URI: BOLD:ABA0829

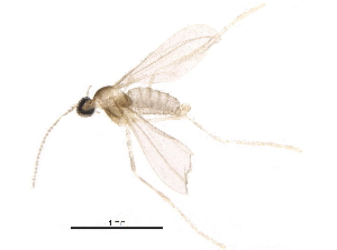

BIOUG22717-G10 [Lateral]
Cecidomyiidae
Family: Cecidomyiidae
BIN URI: BOLD:ACV3844

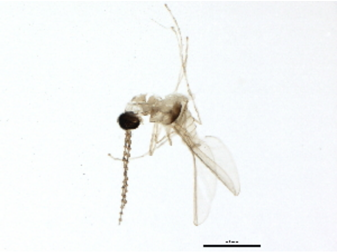

BIOUG01118-H01 [Lateral]
Cecidomyiidae
Family: Cecidomyiidae
BIN URI: BOLD:AAY6380

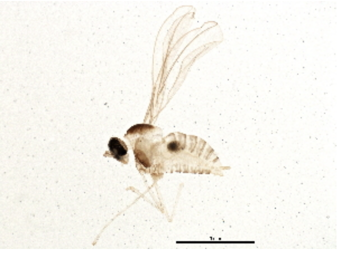

BIOUG02903-D09 [Lateral]
Cecidomyiidae
Family: Cecidomyiidae
BIN URI: BOLD:ABA0840

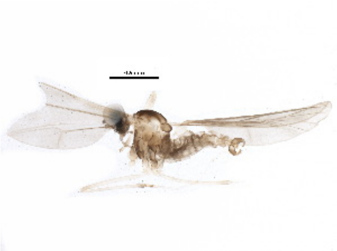

BIOUG22725-E08 [Lateral]
Cecidomyiidae
Family: Cecidomyiidae
BIN URI: BOLD:ACV5112

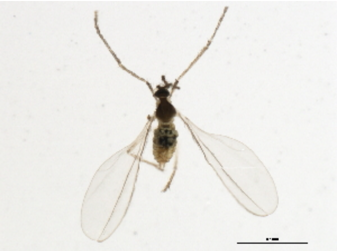

BIOUG00832-G10 [Lateral]
Cecidomyiidae
Family: Cecidomyiidae
BIN URI: BOLD:AAY6374

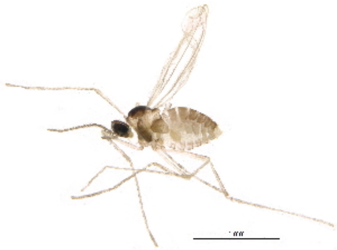

BIOUG23079-F03 [Lateral]
Cecidomyiidae
Family: Cecidomyiidae
BIN URI: BOLD:ACE9686

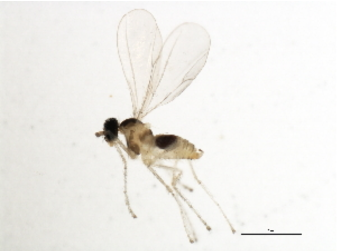

BIOUG00832-G11 [Dorsal]
Cecidomyiidae
Family: Cecidomyiidae
BIN URI: BOLD:ABA0818

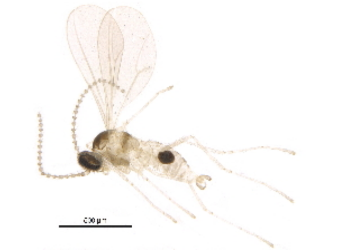

BIOUG22287-F07 [Lateral]
Cecidomyiidae
Family: Cecidomyiidae
BIN URI: BOLD:ACA6770

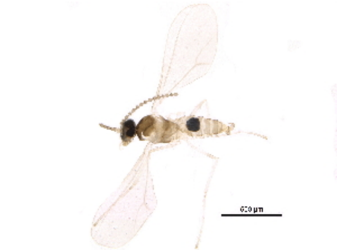

BIOUG22329-C11 [Lateral]
Cecidomyiidae
Family: Cecidomyiidae
BIN URI: BOLD:ACA9545

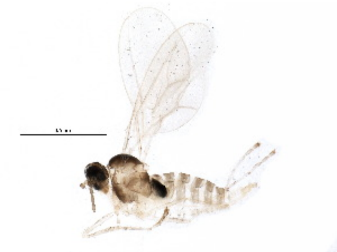

BIOUG19779-A10 [Lateral]
Cecidomyiidae
Family: Cecidomyiidae
BIN URI: BOLD:ACT3440

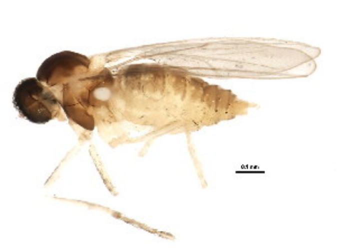

BIOUG01543-D03 [Lateral]
Cecidomyiidae
Family: Cecidomyiidae
BIN URI: BOLD:ABV0494

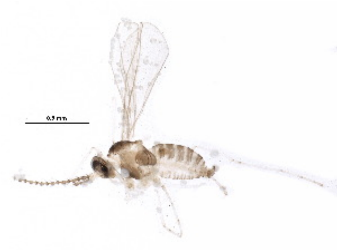

BIOUG22731-G03 [Lateral]
Cecidomyiidae
Family: Cecidomyiidae
BIN URI: BOLD:ACE8808

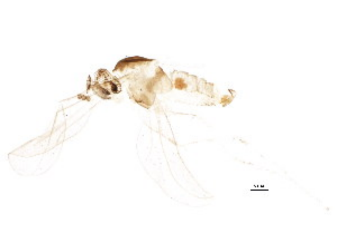

BIOUG01514-F06 [Lateral]
Cecidomyiidae
Family: Cecidomyiidae
BIN URI: BOLD:ABW8018

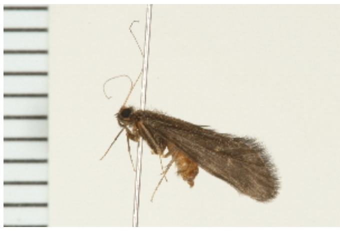

08ONCAD-0306 [Lateral]
Helicopsyche borealis
Family: Helicopsychidae
BIN URI: BOLD:AAA4316

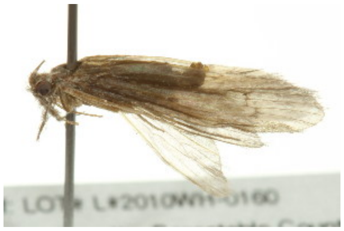

TDWG-0266 [Lateral]
Oecetis cinerascens
Family: Leptoceridae
BIN URI: BOLD:AAA5652

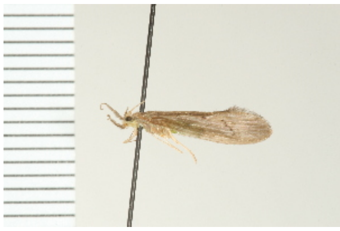

08ONCAD-0274 [Lateral]
Oecetis avara
Family: Leptoceridae
BIN URI: BOLD:AAB0303

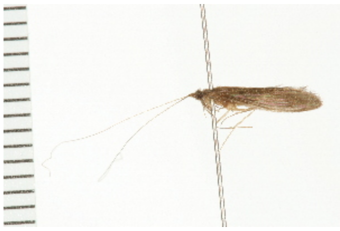

08MBCAD-0178 [Lateral]
Oecetis inconspicua
Family: Leptoceridae
BIN URI: BOLD:AAA1532

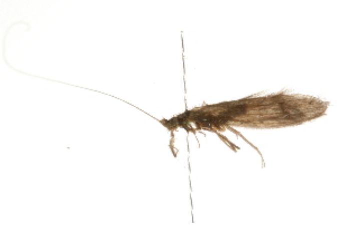

10BBCAD-067 [Lateral]
Oecetis nocturna
Family: Leptoceridae
BIN URI: BOLD:AAB4389

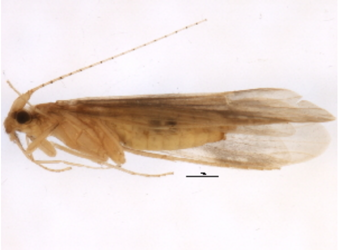

07ELEPT-315 [Lateral]
Triaenodes tardus
Family: Leptoceridae
BIN URI: BOLD:ABY4173

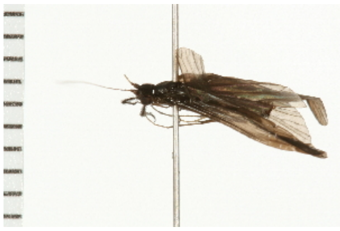

08ONCAD-0543 [Lateral]
Mystacides sepulchralis
Family: Leptoceridae
BIN URI: BOLD:ACF0896

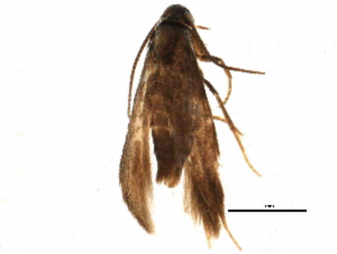

BIOUG08878-E02 [Dorsal]
Heliozela
Family: Heliozelidae
BIN URI: BOLD:ACK4960

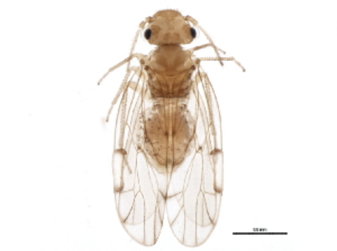

BIOUG00771-D06 [Dorsal]
Ectopsocus meridionalis
Family: Ectopsocidae
BIN URI: BOLD:AAM8931

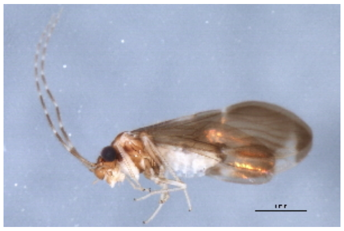

08TTML-2758 [Lateral]
Polypsocus corruptus
Family: Amphipsocidae
BIN URI: BOLD:AAM8933

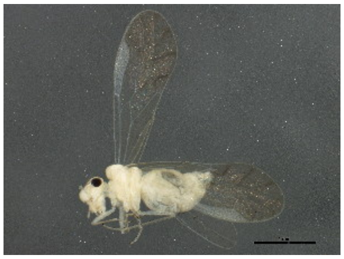

BIOUG01239-C11 [Lateral]
Valenzuela
Family: Caeciliusidae
BIN URI: BOLD:AAM8930

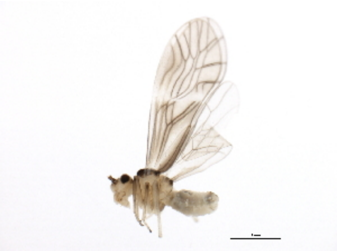

10BBSIO-0135 [Lateral]
Valenzuela flavidus
Family: Caeciliusidae
BIN URI: BOLD:AAH3228

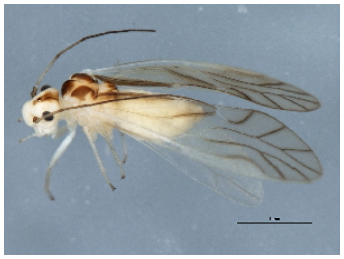

08TTML-2699 [Lateral]
Valenzuela flavidus
Family: Caeciliusidae
BIN URI: BOLD:AAN8447

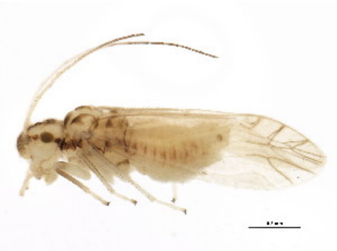

BIOUG01239-D01 [Lateral]
Caeciliusidae
Family: Caeciliusidae
BIN URI: BOLD:ACA3113

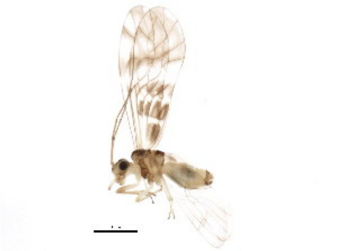

BIOUG01239-E11 [Lateral]
Graphopsocus cruciatus
Family: Stenopsocidae
BIN URI: BOLD:ACA2933

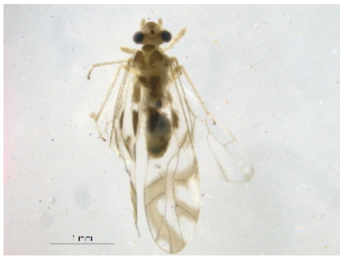

BIOUG16752-D01 [Dorsal]
Graphopsocus cruciatus
Family: Stenopsocidae
BIN URI: BOLD:ACB0984

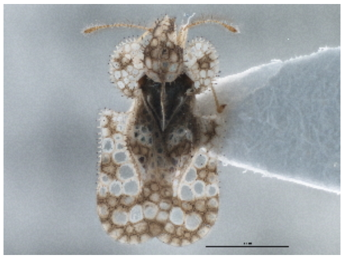

CCDB-21316-G02 [Dorsal]
Corythucha marmorata
Family: Tingidae
BIN URI: BOLD:AAR9127

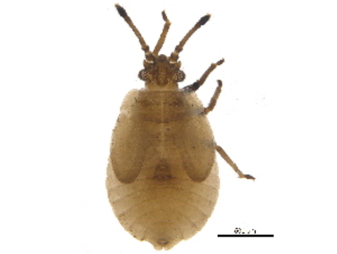

BIOUG21881-A11 [Dorsal]
Acalypta
Family: Tingidae
BIN URI: BOLD:ACW0228

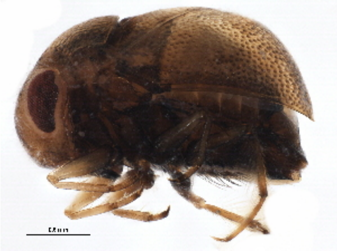

BIOUG24012-H09 [Lateral]
Neoplea
Family: Pleidae

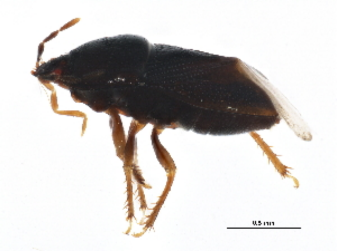

BIOUG24015-H01 [Lateral]
Cydnidae
Family: Cydnidae

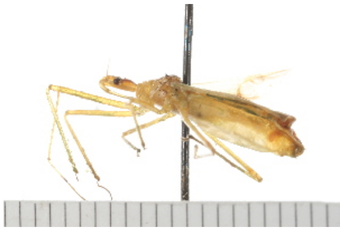

HEMI 0015.02 [Lateral]
Zelus luridus
Family: Reduviidae
BIN URI: BOLD:AAG2724

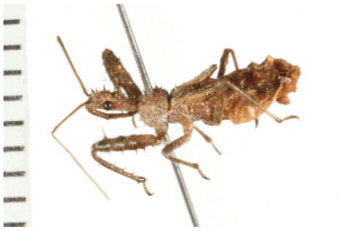

08BBHEM-287 [Dorsal]
Sinea diadema
Family: Reduviidae
BIN URI: BOLD:AAH9651

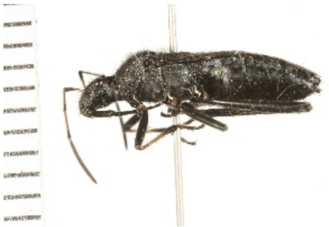

08BBHEM-144 [Lateral]
Alydus eurinus
Family: Alydidae
BIN URI: BOLD:AAC7993

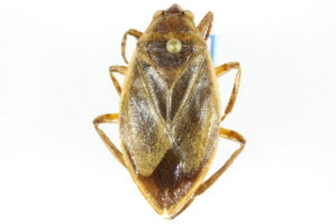

CNC#HEM301054 [Dorsal]
Belostoma
Family: Belostomatidae
BIN URI: BOLD:AAZ0987

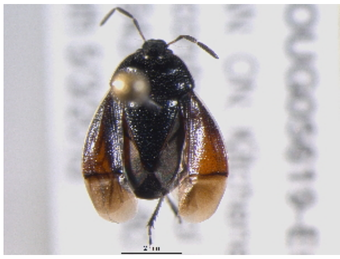

BIOUG05619-E08 [Dorsal]
Cydnidae
Family: Cydnidae
BIN URI: BOLD:AAG8897

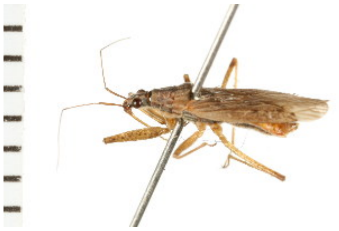

08BBHEM-247 [Lateral]
Nabis rufusculus
Family: Nabidae
BIN URI: BOLD:AAD1219

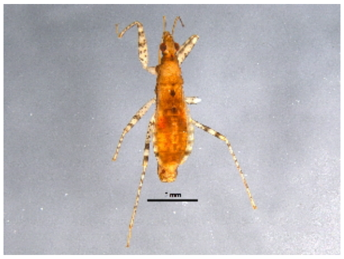

BIOUG05564-F06 [Dorsal]
Hoplistoscelis sordidus
Family: Nabidae
BIN URI: BOLD:ABV2541

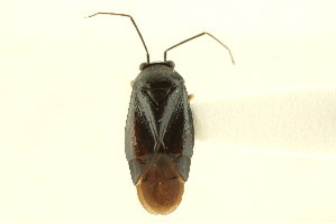

CNC#PBI00394953 [Dorsal]
Slaterocoris stygicus
Family: Miridae
BIN URI: BOLD:AAF4643

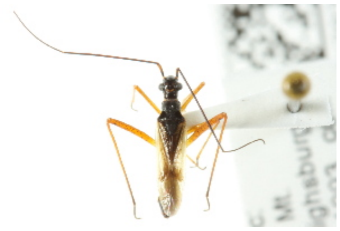

CNC#PBI00394787 [Dorsal]
Collaria meilleurii
Family: Miridae
BIN URI: BOLD:AAG8826

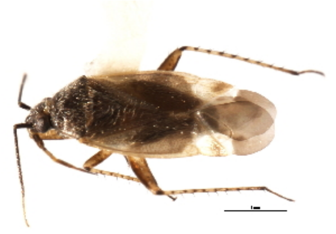

CNC-HEM-0529 [Dorsal]
Plagiognathus obscurus
Family: Miridae
BIN URI: BOLD:AAB3379

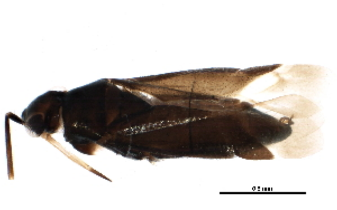

BIOUG12757-F05 [Lateral]
Chlamydatus associatus
Family: Miridae
BIN URI: BOLD:AAF3365

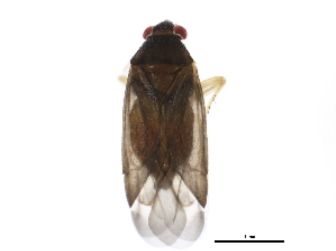

BIOUG00935-F04 [Dorsal]
Phoenicocoris strobicola
Family: Miridae
BIN URI: BOLD:AAH8507

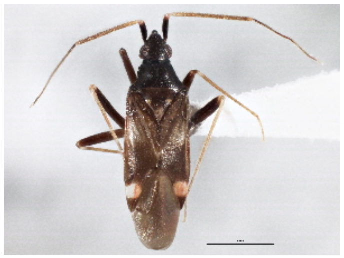

CCDB-21309-A09 [Dorsal]
Fulvius slateri
Family: Miridae
BIN URI: BOLD:ACB0077

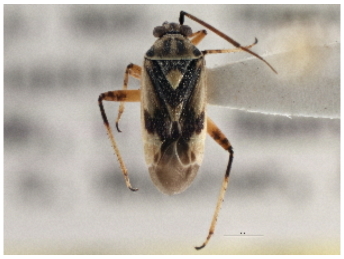

CCDB-21310-H05 [Dorsal]
Polymerus balli
Family: Miridae
BIN URI: BOLD:AAY2830

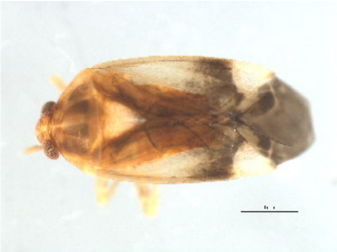

08BBHEM-407 [Dorsal]
Lygocoris pabulinus
Family: Miridae
BIN URI: BOLD:AAB2216

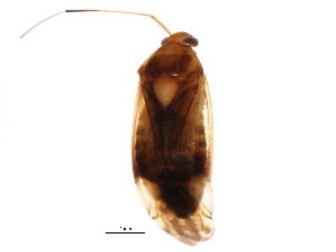

BIOUG01019-D08 [Dorsal]
Neolygus omnivagus
Family: Miridae
BIN URI: BOLD:AAJ2791

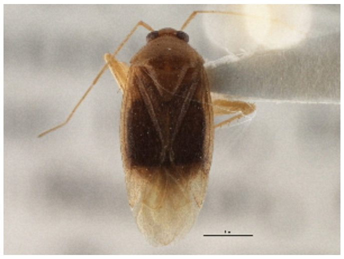

CCDB-21315-F04 [Dorsal]
Neolygus hirticulus
Family: Miridae
BIN URI: BOLD:ABY1773

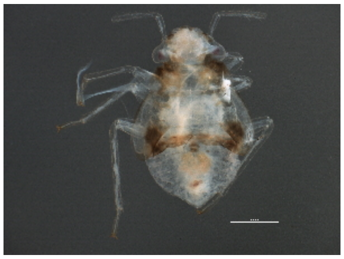

BIOUG09991-H04 [Dorsal]
Tropidosteptes
Family: Miridae
BIN URI: BOLD:ACC0381

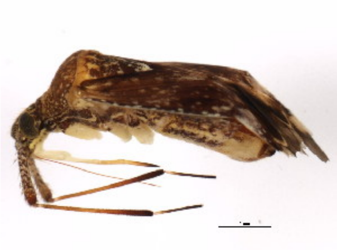

PCPP10-0643 [Lateral]
Neurocolpus nubilus
Family: Miridae
BIN URI: BOLD:AAN8390

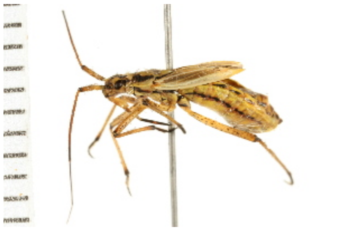

08BBHEM-046 [Lateral]
Leptopterna dolabrata
Family: Miridae
BIN URI: BOLD:AAB5081

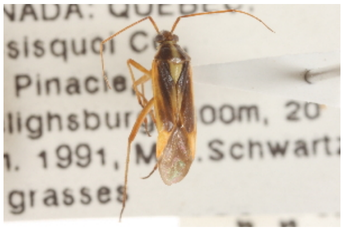

PBI 00395992 [Dorsal]
Stenotus binotatus
Family: Miridae
BIN URI: BOLD:AAC0635

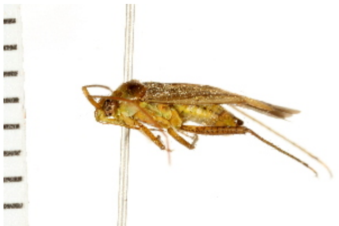

08BBHEM-058 [Lateral]
Adelphocoris lineolatus
Family: Miridae
BIN URI: BOLD:ACE7444

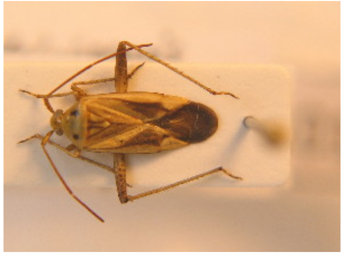

BFB_Heteroptera_Schmolke_134 [Dorsal]
Adelphocoris lineolatus
Family: Miridae
BIN URI: BOLD:ACF1257

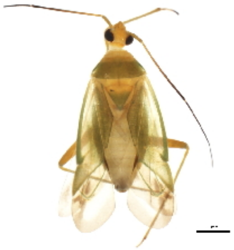

BIOUG00856-D04 [Dorsal]
Lygocoris pabulinus
Family: Miridae
BIN URI: BOLD:AAB2218

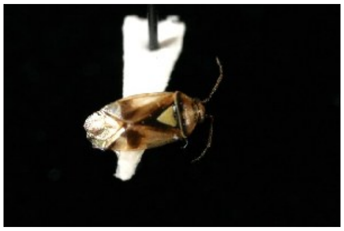

05-PK-049 [Dorsal]
Hemiptera

BIN URI: BOLD:ACE8426

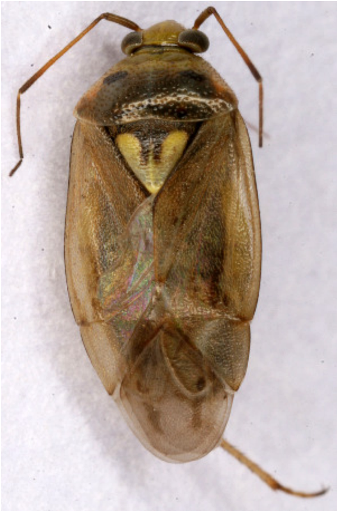

EUBUG_704_f_Lygurugu6 [Dorsal]
Lygus rugulipennis
Family: Miridae
BIN URI: BOLD:ACF4388

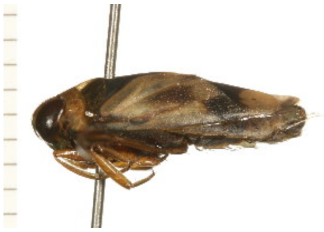

CNC-HEM-1378 [Lateral]
Notonecta undulata
Family: Notonectidae
BIN URI: BOLD:AAI2431

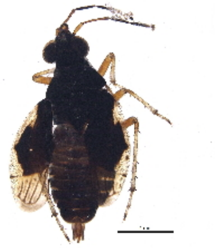

BIOUG09338-F04 [Dorsal]
Micracanthia
Family: Saldidae
BIN URI: BOLD:ACK8299

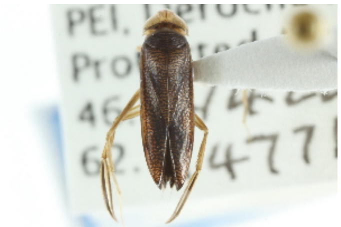

CNC#HEM300323 [Dorsal]
Palmacorixa buenoi
Family: Corixidae
BIN URI: BOLD:AAG8833

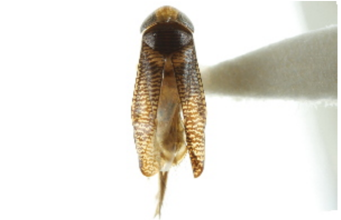

CNC#HEM302722 [Dorsal]
Trichocorixa sexcincta
Family: Corixidae
BIN URI: BOLD:AAH8079

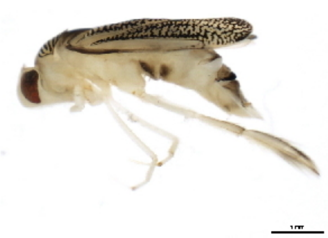

10BBCHEM-0286 [Lateral]
Trichocorixa borealis
Family: Corixidae
BIN URI: BOLD:ACU9590

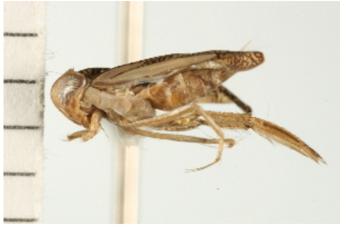

CNC-HEM-1465 [Lateral]
Trichocorixa borealis
Family: Corixidae
BIN URI: BOLD:ABZ4308

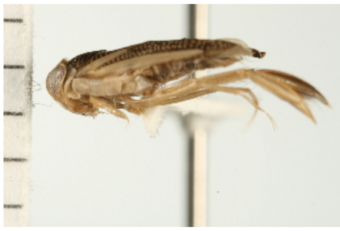

CNC-HEM-1464 [Lateral]
Trichocorixa borealis
Family: Corixidae
BIN URI: BOLD:ACU9591

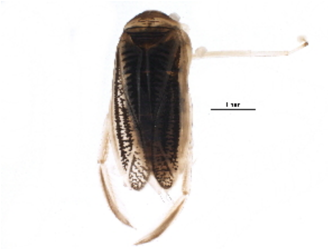

BIOUG24012-G09 [Dorsal]
Corixidae
Family: Corixidae

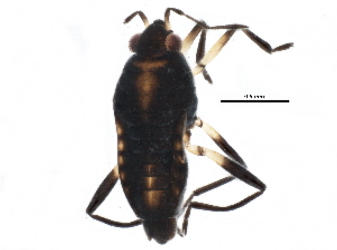

BIOUG24012-G11 [Dorsal]
Gerridae
Family: Gerridae

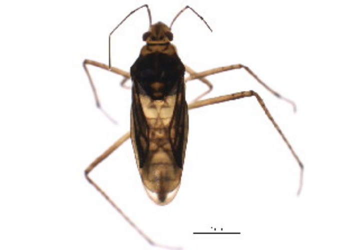

PCPP10-0598 [Dorsal]
Mesovelia
Family: Mesoveliidae
BIN URI: BOLD:ACF3552

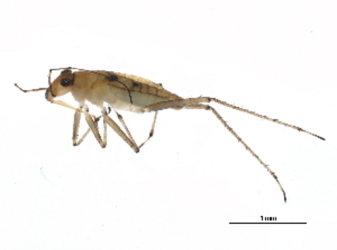

BIOUG24015-B01 [Lateral]
Hemiptera

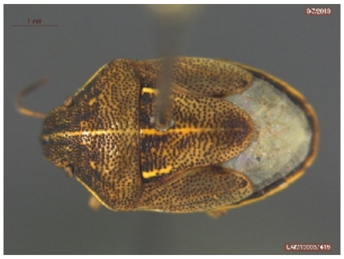

UAM:Ento:97000 [Dorsal]
Neottiglossa undata
Family: Pentatomidae
BIN URI: BOLD:AAE1308

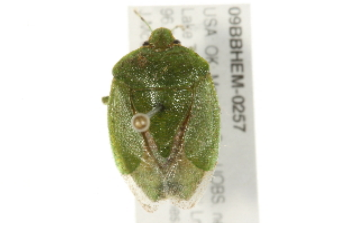

09BBHEM-0257 [Dorsal]
Acrosternum hilare
Family: Pentatomidae
BIN URI: BOLD:AAD6352

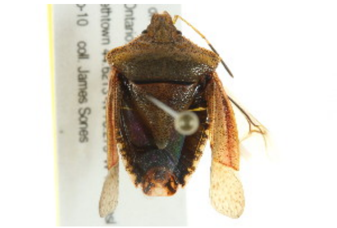

BIOUG01150-C09 [Dorsal]
Euschistus servus
Family: Pentatomidae
BIN URI: BOLD:AAE0845

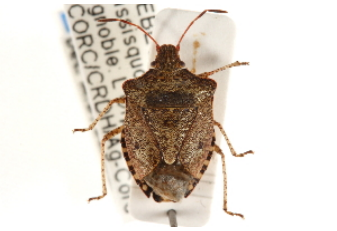

CNC#HEM305245 [Dorsal]
Euschistus tristigmus luridus
Family: Pentatomidae
BIN URI: BOLD:AAG1809

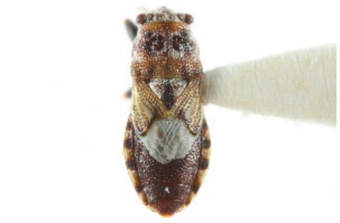

CNC#HEM303005 [Dorsal]
Phlegyas abbreviatus
Family: Pachygronthidae
BIN URI: BOLD:AAW5225

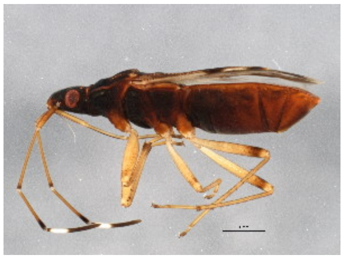

BIOUG02287-D12 [Lateral]
Ozophora sp.
Family: Rhyparochromidae
BIN URI: BOLD:ABW7692

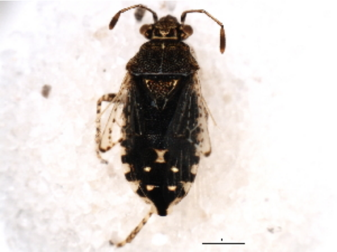

10BBHEM-297 [Dorsal]
Hemiptera

BIN URI: BOLD:AAJ1753

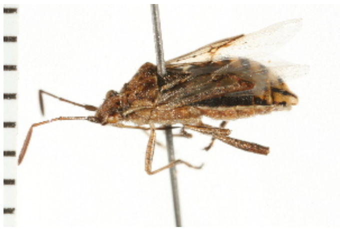

08BBHEM-369 [Lateral]
Stictopleurus punctiventris
Family: Rhopalidae
BIN URI: BOLD:AAC0814

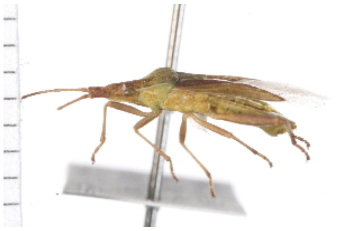

CNC-HEM-0872 [Lateral]
Harmostes reflexulus
Family: Rhopalidae
BIN URI: BOLD:AAD0591

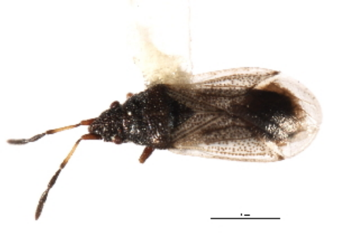

CNC-HEM-0059 [Dorsal]
Crophius disconotus
Family: Oxycarenidae
BIN URI: BOLD:AAR4786

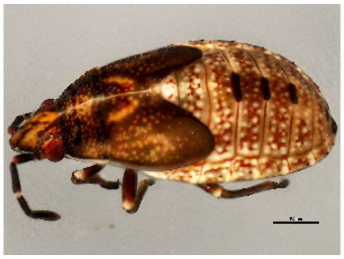

TDWG-1204 [Dorsal]
Hemiptera

BIN URI: BOLD:ABY8347

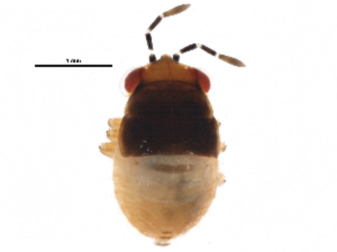

BIOUG24027-C11 [Dorsal]
Hemiptera

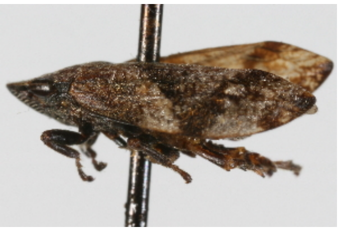

ENT-OUBS-335 [Lateral]
Cercopidae
Family: Cercopidae
BIN URI: BOLD:AAG8827

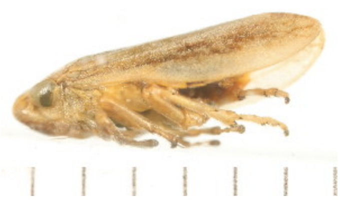

CNC-HEM-1064 [Lateral]
Philaenus spumarius
Family: Cercopidae
BIN URI: BOLD:AAB1850

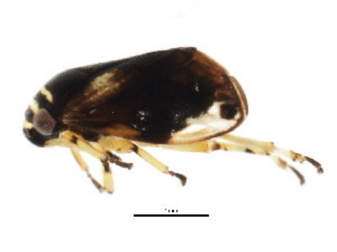

BIOUG01012-A07 [Lateral]
Clastoptera proteus
Family: Clastopteridae
BIN URI: BOLD:AAG8734

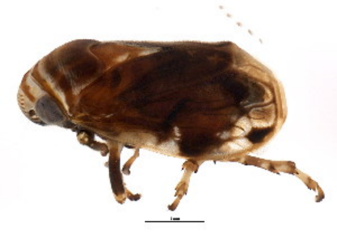

BIOUG00806-E01 [Lateral]
Clastoptera obtusa
Family: Clastopteridae
BIN URI: BOLD:AAG8823

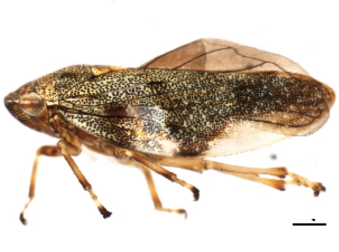

09BBEHE-194 [Lateral]
Aphrophora quadrinotata
Family: Cercopidae
BIN URI: BOLD:AAZ2091

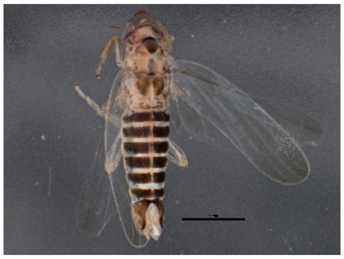

BIOUG03639-D02 [Dorsal]
Dikraneura mali
Family: Cicadellidae
BIN URI: BOLD:ABA5842

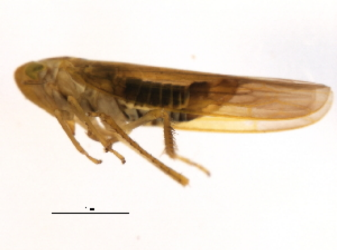

10BBCHEM-1100 [Lateral]
Forcipata acclina
Family: Cicadellidae
BIN URI: BOLD:AAN8283

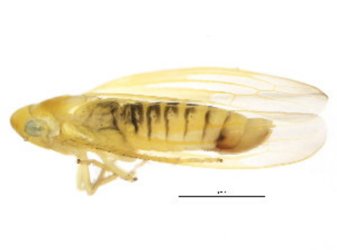

BIOUG00941-A12 [Lateral]
Forcipata loca
Family: Cicadellidae
BIN URI: BOLD:ACC8165

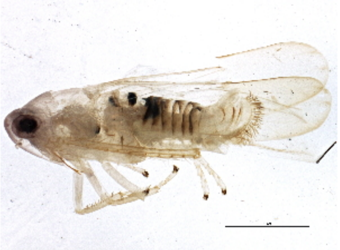

BIOUG06041-G11 [Lateral]
Empoasca
Family: Cicadellidae
BIN URI: BOLD:AAG8683

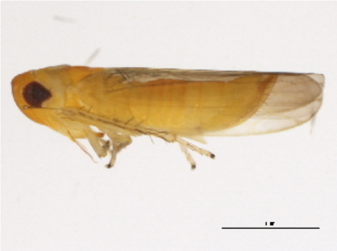

BIOUG00999-F06 [Lateral]
Empoasca coccinea
Family: Cicadellidae
BIN URI: BOLD:ABA5764

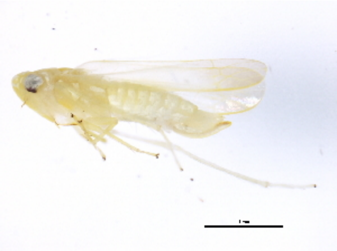

BIOUG00935-G12 [Lateral]
Empoasca
Family: Cicadellidae
BIN URI: BOLD:ABA5771

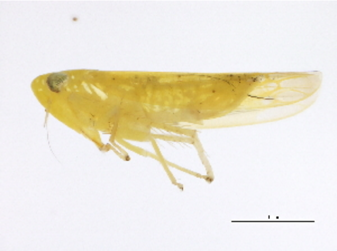

BIOUG00906-A02 [Lateral]
Empoasca
Family: Cicadellidae
BIN URI: BOLD:AAG2873

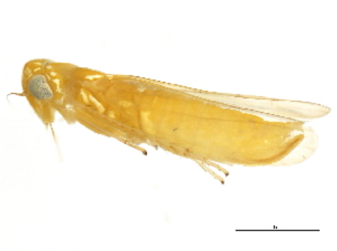

OT1_JRE_234 [Lateral]
Empoasca sp.
Family: Cicadellidae
BIN URI: BOLD:AAG2868

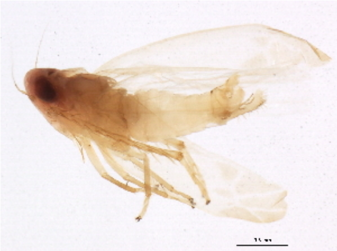

BIOUG01652-C12 [Lateral]
Empoasca
Family: Cicadellidae
BIN URI: BOLD:ABA5807

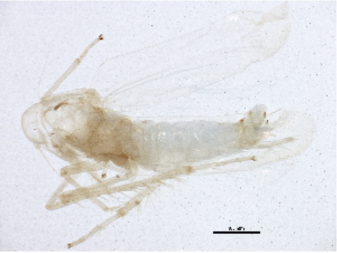

BIOUG01595-D08 [Dorsal]
Empoasca
Family: Cicadellidae
BIN URI: BOLD:AAN8337

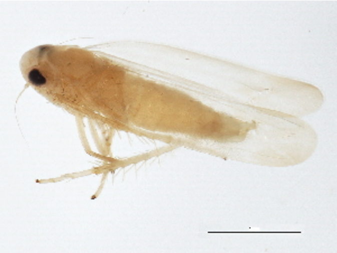

BIOUG00771-D03 [Lateral]
Empoasca
Family: Cicadellidae
BIN URI: BOLD:AAN8250

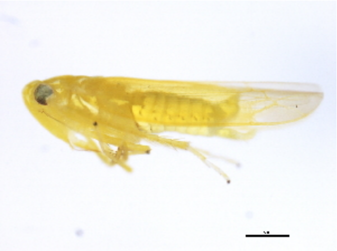

BIOUG00935-E06 [Lateral]
Empoasca
Family: Cicadellidae
BIN URI: BOLD:ACE5873

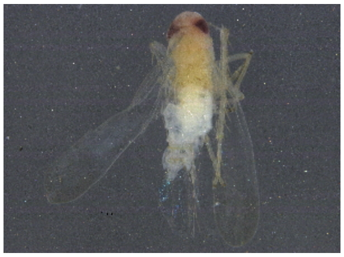

BIOUG00891-G01 [Dorsal]
Empoasca
Family: Cicadellidae
BIN URI: BOLD:ACF5025

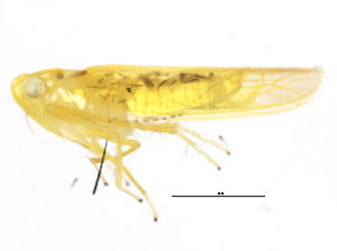

BIOUG00941-H11 [Lateral]
Empoasca
Family: Cicadellidae
BIN URI: BOLD:ABZ4247

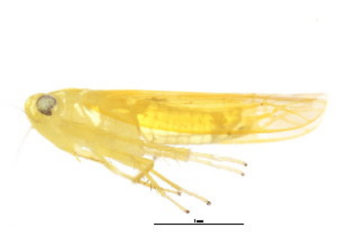

BIOUG00941-C02 [Lateral]
Empoasca
Family: Cicadellidae
BIN URI: BOLD:ACF5026

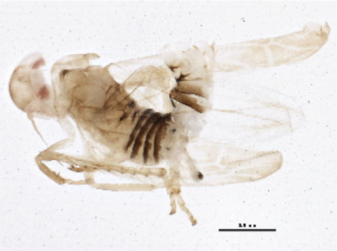

BIOUG01595-H09 [Lateral]
Cicadellidae
Family: Cicadellidae
BIN URI: BOLD:ABW2910

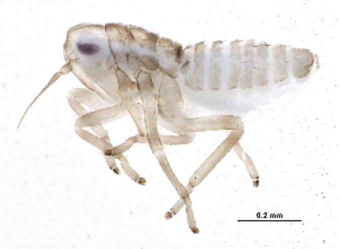

BIOUG15778-E02 [Lateral]
Typhlocybinae
Family: Cicadellidae
BIN URI: BOLD:ACQ9086

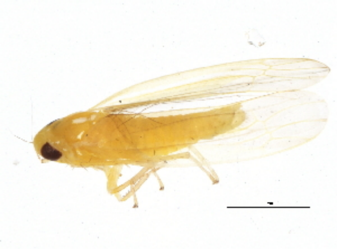

BIOUG00771-A11 [Lateral]
Empoasca decipiens
Family: Cicadellidae
BIN URI: BOLD:AAY6741

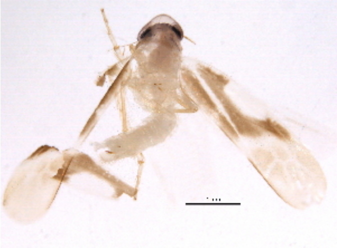

BIOUG03055-B03 [Dorsal]
Empoasca
Family: Cicadellidae
BIN URI: BOLD:ABA5875

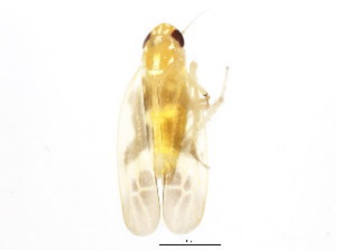

BIOUG00941-E01 [Dorsal]
Empoasca
Family: Cicadellidae
BIN URI: BOLD:AAV0166

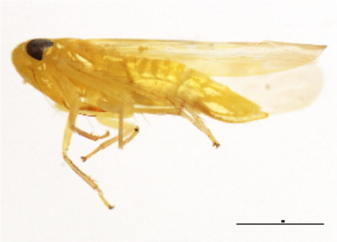

BIOUG00552-B02 [Lateral]
Empoasca
Family: Cicadellidae
BIN URI: BOLD:AAV0165

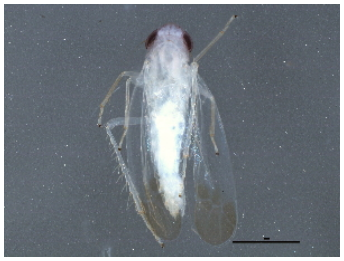

BIOUG05545-F05 [Dorsal]
Empoasca
Family: Cicadellidae
BIN URI: BOLD:AAN8289

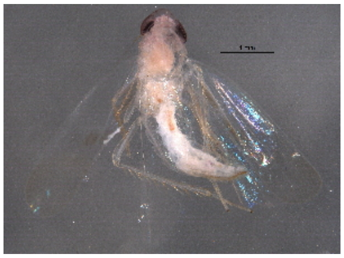

BIOUG09357-H06 [Dorsal]
Empoasca
Family: Cicadellidae
BIN URI: BOLD:ACL2611

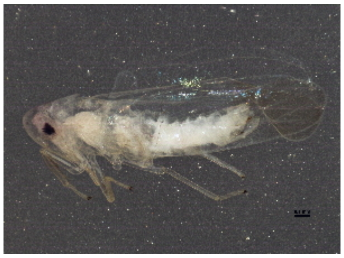

BIOUG03564-F01 [Lateral]
Empoasca
Family: Cicadellidae
BIN URI: BOLD:AAV0159

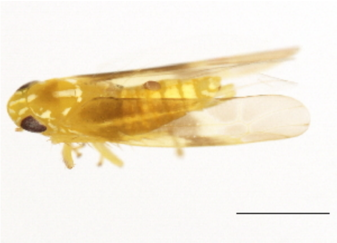

BIOUG00552-D12 [Lateral]
Empoasca
Family: Cicadellidae
BIN URI: BOLD:AAG8850

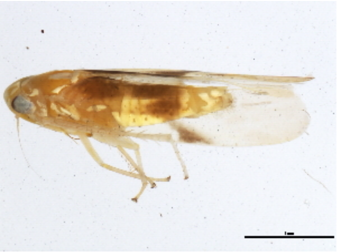

BIOUG00771-B03 [Lateral]
Empoasca
Family: Cicadellidae
BIN URI: BOLD:AAY6736

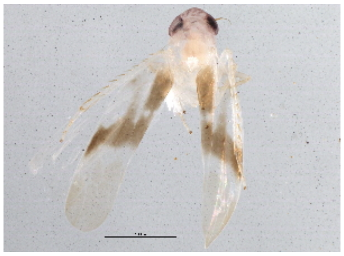

BIOUG09690-H09 [Dorsal]
Empoasca
Family: Cicadellidae
BIN URI: BOLD:ACC8521

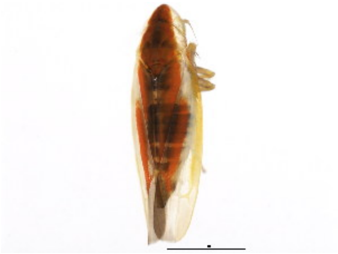

BIOUG00941-H07 [Dorsal]
Erythridula wysongi
Family: Cicadellidae
BIN URI: BOLD:AAN8287

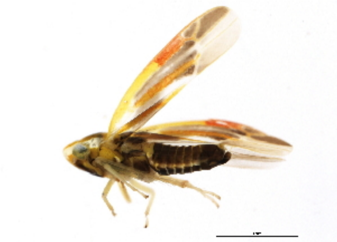

BIOUG00552-H06 [Lateral]
Arboridia bitincta
Family: Cicadellidae
BIN URI: BOLD:ABA5831

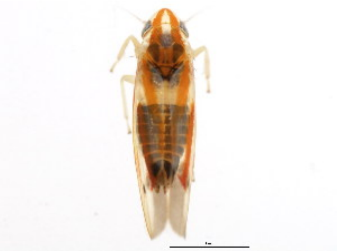

BIOUG00917-A08 [Dorsal]
Erythridula wysongi
Family: Cicadellidae
BIN URI: BOLD:ABZ1306

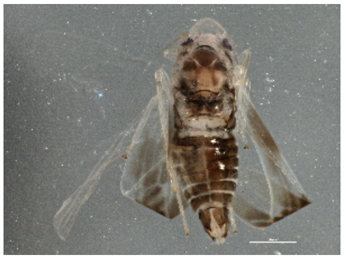

BIOUG05017-C10 [Dorsal]
Erythridula
Family: Cicadellidae
BIN URI: BOLD:ACE0635

IMAGE NOT AVAILABLE

BIOUG22298-H10
Erythridula wysongi
Family: Cicadellidae

IMAGE NOT AVAILABLE

BIOUG22082-B09
Erythridula
Family: Cicadellidae

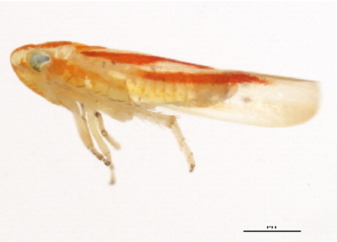

BIOUG00552-D02 [Lateral]
Erythridula
Family: Cicadellidae
BIN URI: BOLD:ACL3048

IMAGE NOT AVAILABLE

BIOUG22297-G11
Erythridula
Family: Cicadellidae

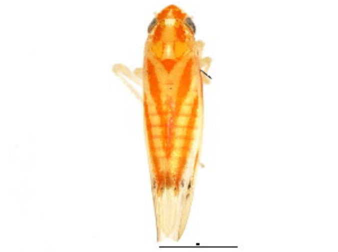

BIOUG00917-B01 [Dorsal]
Erythridula tenuispica
Family: Cicadellidae
BIN URI: BOLD:ABA5830

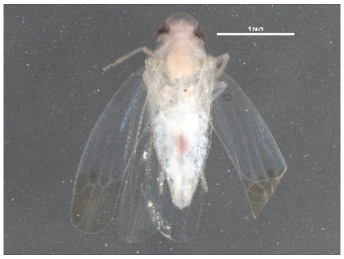

BIOUG21768-D03 [Dorsal]
Erythridula
Family: Cicadellidae
BIN URI: BOLD:ACV2335

IMAGE NOT AVAILABLE

BIOUG22082-E09
Erythridula
Family: Cicadellidae

IMAGE NOT AVAILABLE

BIOUG22083-G02
Erythridula scytha
Family: Cicadellidae

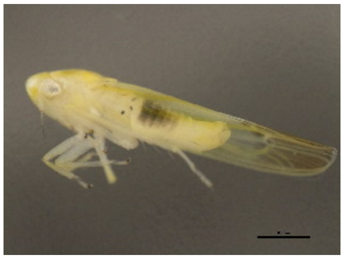

TDWG-1159 [Lateral]
Erythridula scytha
Family: Cicadellidae
BIN URI: BOLD:AAN8412

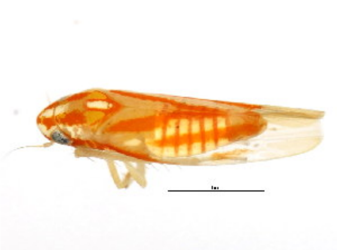

BIOUG00917-B02 [Dorsal]
Erythridula dunni
Family: Cicadellidae
BIN URI: BOLD:ABA5786

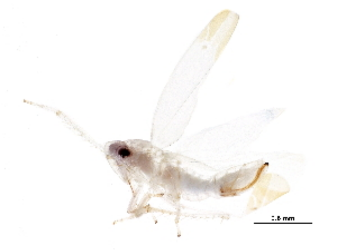

BIOUG20490-A06 [Lateral]
Hemiptera

BIN URI: BOLD:ACU6504

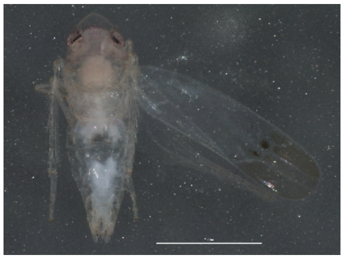

BIOUG02602-B02 [Dorsal]
Erythridula
Family: Cicadellidae
BIN URI: BOLD:ABW7654

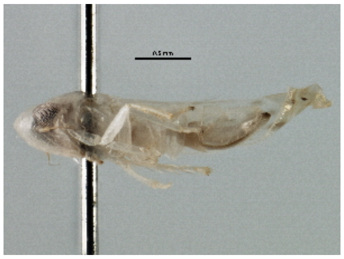

BIOUG02602-A03 [Lateral]
Erythridula
Family: Cicadellidae
BIN URI: BOLD:ABX9019

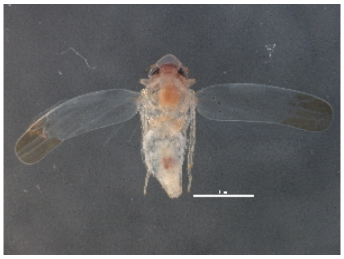

BIOUG08453-A04 [Dorsal]
Erythridula
Family: Cicadellidae
BIN URI: BOLD:ACK3871

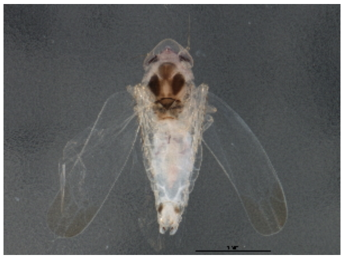

BIOUG03637-C03 [Dorsal]
Erythridula
Family: Cicadellidae
BIN URI: BOLD:ACD3877

IMAGE NOT AVAILABLE

BIOUG21768-A04
Erythridula
Family: Cicadellidae

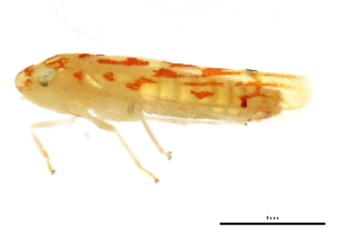

BIOUG00947-B10 [Lateral]
Eratoneura flexibilis
Family: Cicadellidae
BIN URI: BOLD:AAZ8495

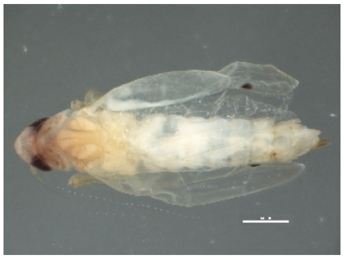

BIOUG01297-B04 [Dorsal]
Eratoneura flexibilis
Family: Cicadellidae
BIN URI: BOLD:AAZ8496

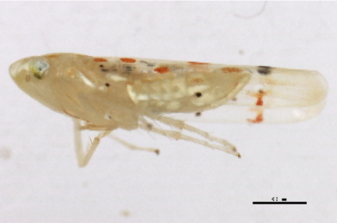

BIOUG00906-D07 [Lateral]
Hemiptera

BIN URI: BOLD:ABA5797

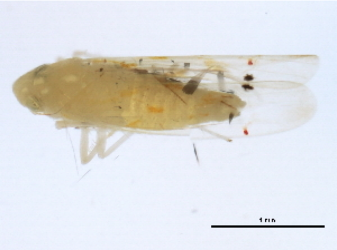

BIOUG00947-A02 [Lateral]
Erythroneura certa
Family: Cicadellidae
BIN URI: BOLD:ABA5787

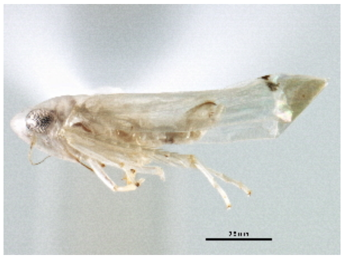

BIOUG02994-E06 [Lateral]
Eratoneura
Family: Cicadellidae
BIN URI: BOLD:ABV2644

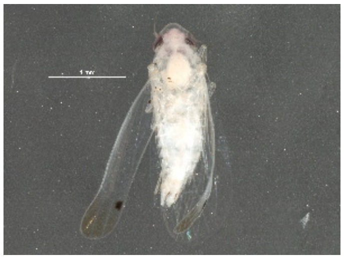

BIOUG22083-G12 [Dorsal]
Eratoneura
Family: Cicadellidae
BIN URI: BOLD:ACV5228

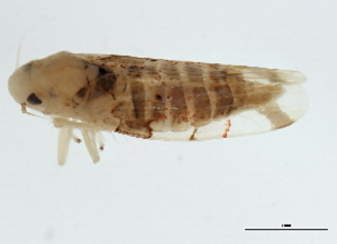

BIOUG00856-A10 [Lateral]
Hymetta balteata
Family: Cicadellidae
BIN URI: BOLD:AAV0157

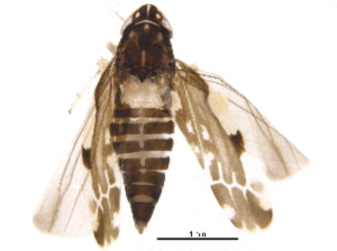

BIOUG22297-D06 [Dorsal]
Erythroneura
Family: Cicadellidae
BIN URI: BOLD:ABY0554

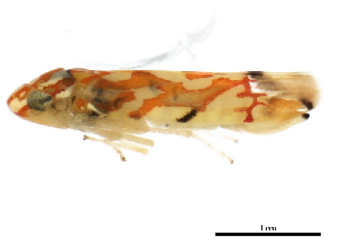

BIOUG00947-E02 [Lateral]
Erythroneura bakeri
Family: Cicadellidae
BIN URI: BOLD:AAV0161

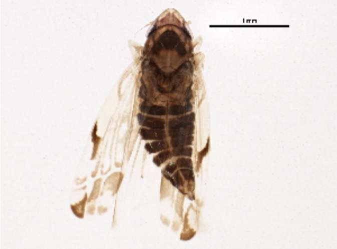

BIOUG05606-F10 [Dorsal]
Erythroneura vulnerata
Family: Cicadellidae
BIN URI: BOLD:AAY6752

IMAGE NOT AVAILABLE

BIOUG22582-H03
Erythroneura vulnerata
Family: Cicadellidae

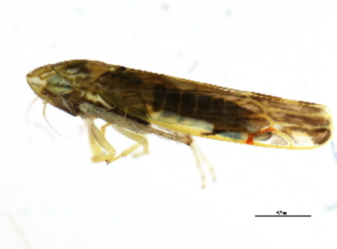

PCPP10-0567 [Lateral]
Erythroneura vulnerata
Family: Cicadellidae
BIN URI: BOLD:AAO8361

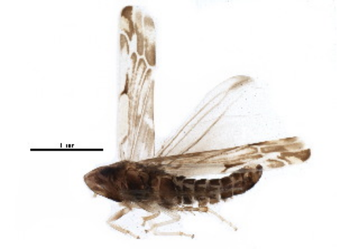

BIOUG21768-D01 [Lateral]
Erythroneura vulnerata
Family: Cicadellidae
BIN URI: BOLD:ABY9043

IMAGE NOT AVAILABLE

BIOUG21777-D09
Erythroneura vulnerata
Family: Cicadellidae

IMAGE NOT AVAILABLE

BIOUG21568-E12
Erythroneura vulnerata
Family: Cicadellidae

IMAGE NOT AVAILABLE

BIOUG22082-A08
Erythroneura vulnerata
Family: Cicadellidae

IMAGE NOT AVAILABLE

BIOUG22082-A04
Erythroneura vulnerata
Family: Cicadellidae

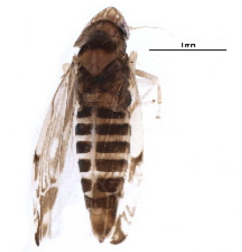

BIOUG21568-B12 [Dorsal]
Erythroneura vulnerata
Family: Cicadellidae
BIN URI: BOLD:ACV2885

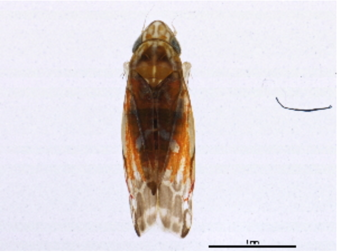

BIOUG01236-B09 [Dorsal]
Hemiptera

BIN URI: BOLD:ABA5772

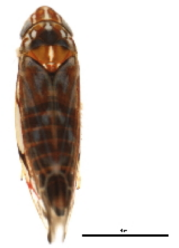

BIOUG00906-G11 [Dorsal]
Erythroneura vulnerata
Family: Cicadellidae
BIN URI: BOLD:ACQ3943

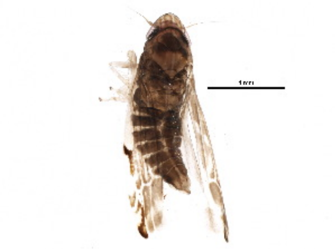

BIOUG21568-G04 [Dorsal]
Erythroneura vulnerata
Family: Cicadellidae
BIN URI: BOLD:ACV2886

IMAGE NOT AVAILABLE

BIOUG21568-G07
Erythroneura vulnerata
Family: Cicadellidae

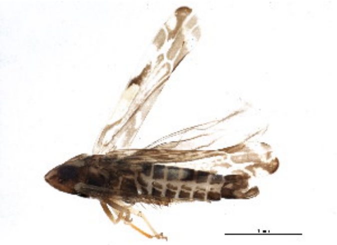

BIOUG01293-G07 [Dorsal]
Erythroneura vulnerata
Family: Cicadellidae
BIN URI: BOLD:ABY9046

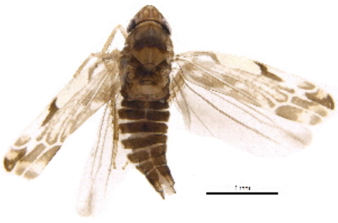

BIOUG22297-H10 [Dorsal]
Erythroneura vulnerata
Family: Cicadellidae
BIN URI: BOLD:ACV2800

IMAGE NOT AVAILABLE

BIOUG22574-D02
Erythroneura
Family: Cicadellidae

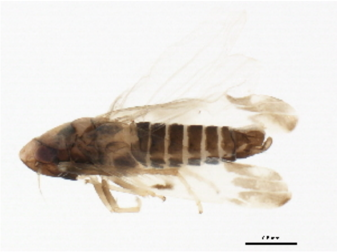

BIOUG01652-C01 [Lateral]
Erythroneura ontari
Family: Cicadellidae
BIN URI: BOLD:ABA5810

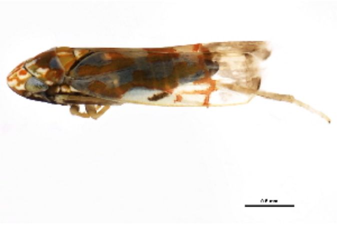

BIOUG00906-C03 [Lateral]
Erythroneura vulnerata
Family: Cicadellidae
BIN URI: BOLD:AAV0164

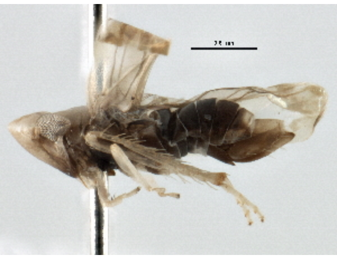

BIOUG02966-D03 [Lateral]
Erythroneura rubrella
Family: Cicadellidae
BIN URI: BOLD:ACC8414

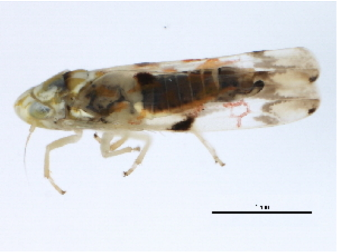

BIOUG00935-C08 [Lateral]
Erythroneura aza
Family: Cicadellidae
BIN URI: BOLD:AAY6747

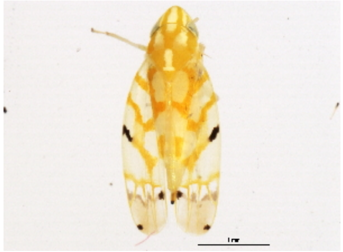

BIOUG00999-C04 [Dorsal]
Erythroneura
Family: Cicadellidae
BIN URI: BOLD:ABA5864

IMAGE NOT AVAILABLE

BIOUG22355-B07
Erythroneura
Family: Cicadellidae

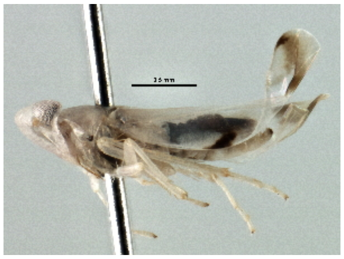

BIOUG02986-E08 [Lateral]
Erythroneura
Family: Cicadellidae
BIN URI: BOLD:AAZ0166

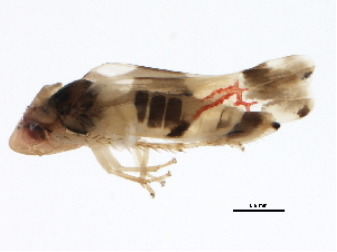

BIOUG01308-A04 [Lateral]
Erythroneura
Family: Cicadellidae
BIN URI: BOLD:ABZ2507

IMAGE NOT AVAILABLE

BIOUG22355-F02
Erythroneura elegans
Family: Cicadellidae

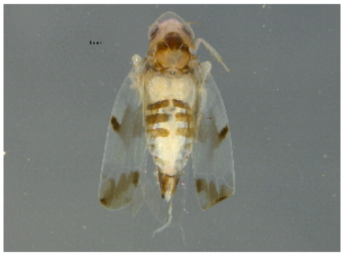

BIOUG02944-G12 [Dorsal]
Erythroneura elegans
Family: Cicadellidae
BIN URI: BOLD:ABA5798

IMAGE NOT AVAILABLE

BIOUG21777-B09
Erythroneura vitifex
Family: Cicadellidae

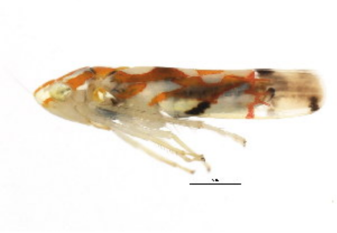

BIOUG00941-B07 [Lateral]
Erythroneura vitifex
Family: Cicadellidae
BIN URI: BOLD:AAY6742

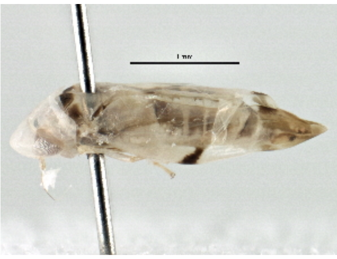

BIOUG02716-D02 [Lateral]
Erythroneura vitifex
Family: Cicadellidae
BIN URI: BOLD:ACQ8506

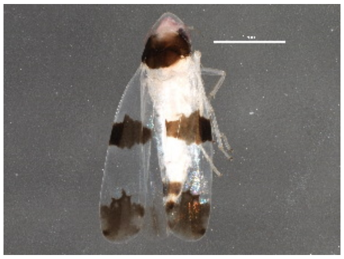

BIOUG22355-G09 [Dorsal]
Erythroneura
Family: Cicadellidae
BIN URI: BOLD:ACV5324

IMAGE NOT AVAILABLE

BIOUG22355-B05
Erythroneura tricincta
Family: Cicadellidae

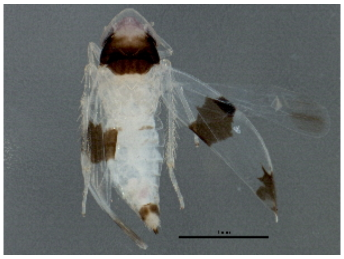

BIOUG05510-B02 [Dorsal]
Erythroneura tricincta
Family: Cicadellidae
BIN URI: BOLD:AAY6751

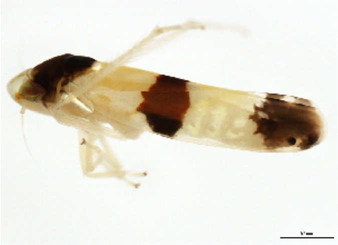

BIOUG00856-E08 [Lateral]
Erythroneura tricincta
Family: Cicadellidae
BIN URI: BOLD:AAY6738

IMAGE NOT AVAILABLE

BIOUG21768-A10
Erythroneura tricincta
Family: Cicadellidae

IMAGE NOT AVAILABLE

BIOUG21768-G11
Erythroneura
Family: Cicadellidae

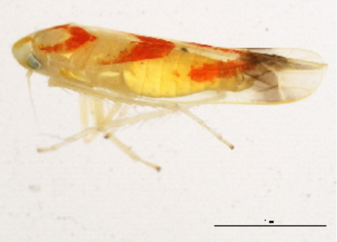

BIOUG00552-D06 [Lateral]
Dikrella
Family: Cicadellidae
BIN URI: BOLD:AAV0168

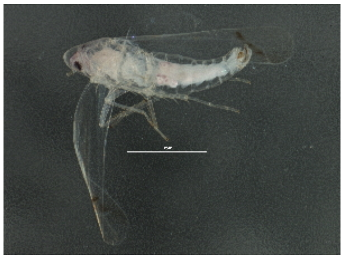

BIOUG02829-F01 [Lateral]
Dikrella cruentata
Family: Cicadellidae
BIN URI: BOLD:AAV0158

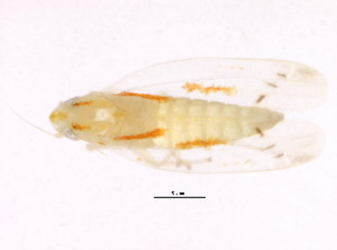

BIOUG00806-C01 [Lateral]
Dikrella
Family: Cicadellidae
BIN URI: BOLD:ABX7281

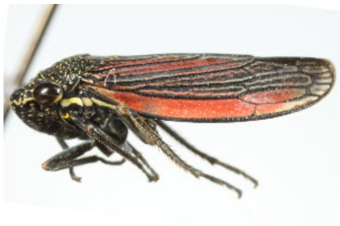

CNC#HEM400181 [Lateral]
Cuerna striata
Family: Cicadellidae
BIN URI: BOLD:AAG2877

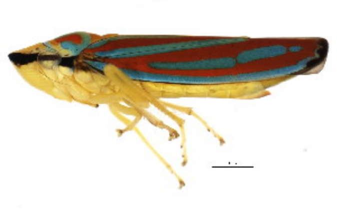

BIOUG00901-G06 [Lateral]
Graphocephala fennahi
Family: Cicadellidae
BIN URI: BOLD:AAG2909

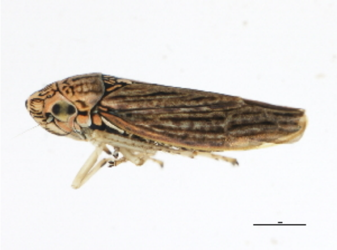

10BBCHEM-1231 [Lateral]
Neokolla hieroglyphica
Family: Cicadellidae
BIN URI: BOLD:AAN8418

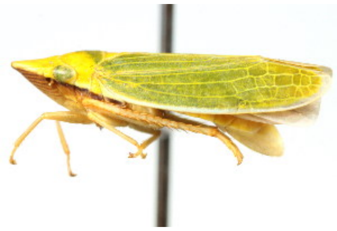

CNC#HEM400202 [Lateral]
Draeculacephala antica
Family: Cicadellidae
BIN URI: BOLD:ACE9696

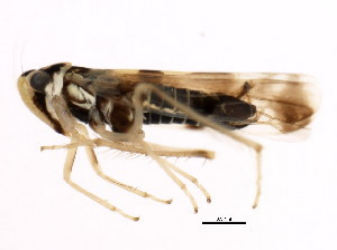

BIOUG00806-H01 [Lateral]
Eupteryx atropunctata
Family: Cicadellidae
BIN URI: BOLD:AAG2869

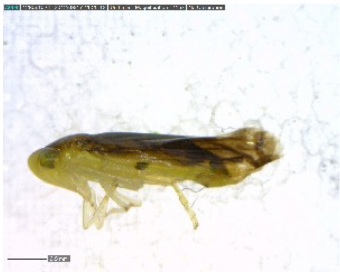

BIOUG02608-D05 [Lateral]
Eupteryx flavoscuta
Family: Cicadellidae
BIN URI: BOLD:ABA5805

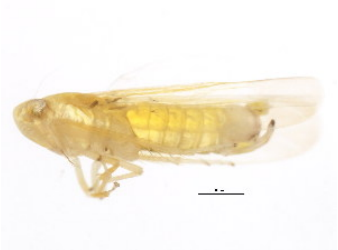

BIOUG00941-C11 [Lateral]
Typhlocyba niobe
Family: Cicadellidae
BIN URI: BOLD:ABA5877

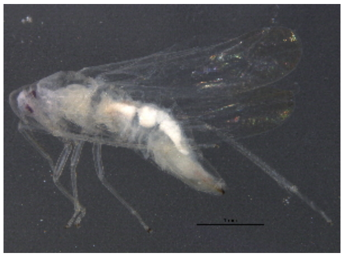

BIOUG06799-F05 [Lateral]
Typhlocybinae
Family: Cicadellidae
BIN URI: BOLD:ACH1957

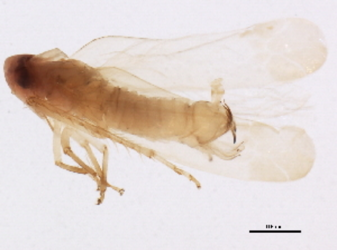

BIOUG01663-A12 [Lateral]
Typhlocyba pomaria
Family: Cicadellidae
BIN URI: BOLD:AAF5980

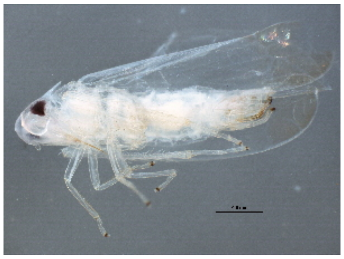

BIOUG05847-H05 [Lateral]
Typhlocyba hockingensis
Family: Cicadellidae
BIN URI: BOLD:ACV8488

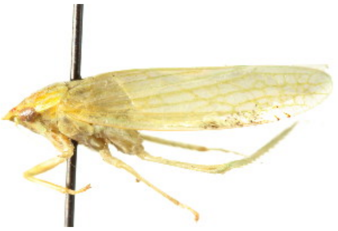

CNC#HEM400764 [Lateral]
Gyponana praelonga
Family: Cicadellidae
BIN URI: BOLD:AAG2878

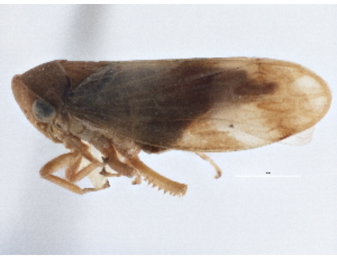

CNC#HEM305539 [Lateral]
Macropsis basalis
Family: Cicadellidae
BIN URI: BOLD:ACC9200

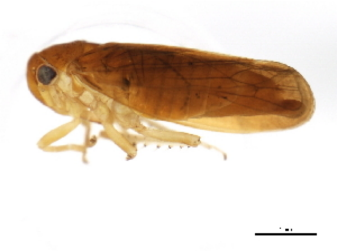

BIOUG00943-A02 [Lateral]
Oncopsis sobria
Family: Cicadellidae
BIN URI: BOLD:ACI7197

IMAGE NOT AVAILABLE

BIOUG21881-D01
Oncopsis
Family: Cicadellidae

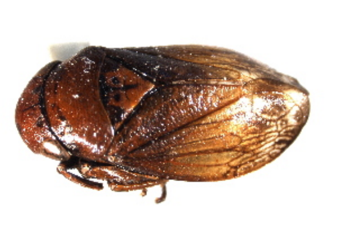

CNC#HEM405041 [Lateral]
Penthimia americana
Family: Cicadellidae
BIN URI: BOLD:ABW7659

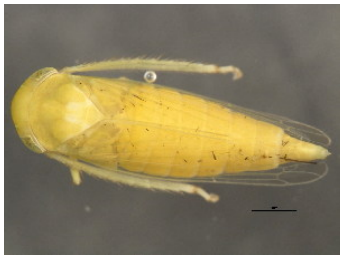

10BBCHEM-1039 [Dorsal]
Chlorotettix
Family: Cicadellidae
BIN URI: BOLD:AAG2891

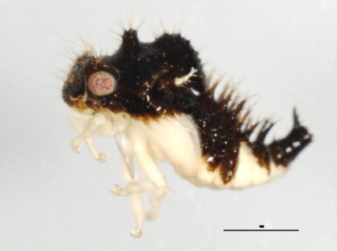

09BBEHE-086 [Lateral]
Publilia concava
Family: Membracidae
BIN URI: BOLD:AAD6344

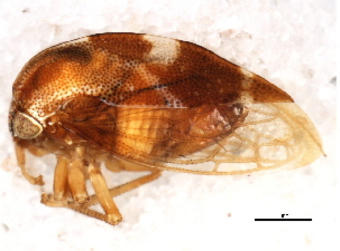

09BBEHE-204 [Lateral]
Cyrtolobus
Family: Membracidae
BIN URI: BOLD:AAN8383

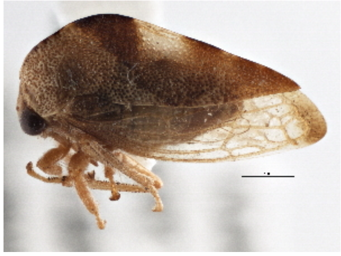

CCDB-21318-G08 [Lateral]
Cyrtolobus vau
Family: Membracidae
BIN URI: BOLD:AAN8263

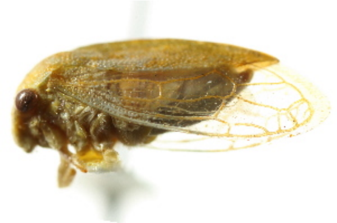

CNC#HEM401480 [Lateral]
Atymna helena
Family: Membracidae
BIN URI: BOLD:AAY9905

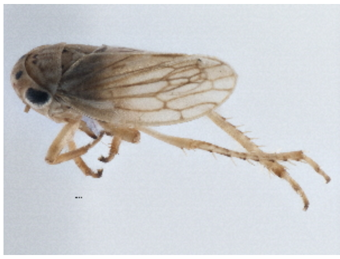

CNC#HEM305528 [Lateral]
Ceratagallia viator
Family: Cicadellidae
BIN URI: BOLD:AAG2875

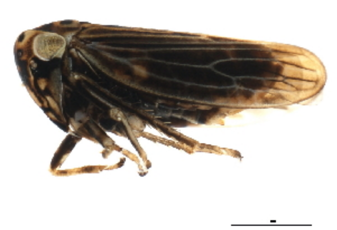

BIOUG00552-H08 [Lateral]
Agallia quadripunctata
Family: Cicadellidae
BIN URI: BOLD:AAG2899

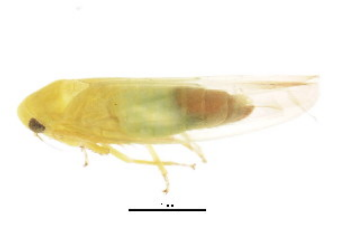

BIOUG01012-E05 [Lateral]
Balclutha
Family: Cicadellidae
BIN URI: BOLD:AAY6737

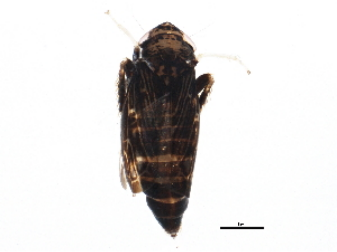

BIOUG06984-G02 [Dorsal]
Limotettix
Family: Cicadellidae
BIN URI: BOLD:ACJ8795

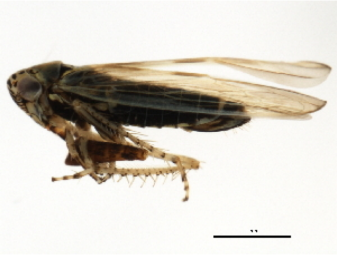

BIOUG00937-B09 [Lateral]
Macrosteles quadrilineatus
Family: Cicadellidae
BIN URI: BOLD:AAA9422

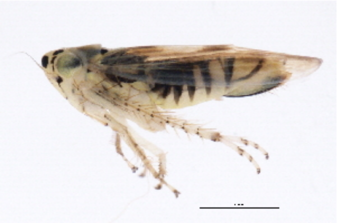

09BBEHE-127 [Lateral]
Macrosteles variatus
Family: Cicadellidae
BIN URI: BOLD:AAV0236

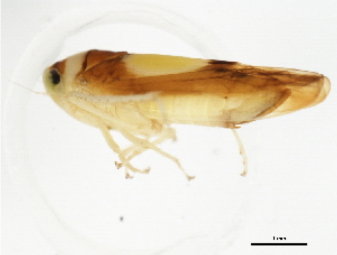

BIOUG00937-D08 [Lateral]
Colladonus clitellarius
Family: Cicadellidae
BIN URI: BOLD:AAG2885

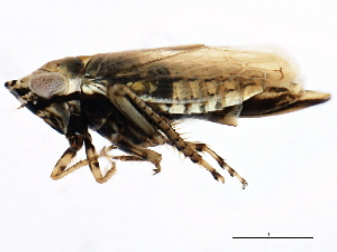

10BBCHEM-0358 [Lateral]
Doratura stylata
Family: Cicadellidae
BIN URI: BOLD:AAG8821

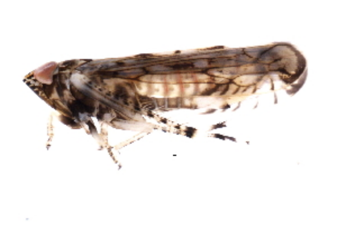

09BBEHE-151 [Lateral]
Scaphoideus
Family: Cicadellidae
BIN URI: BOLD:AAG8981

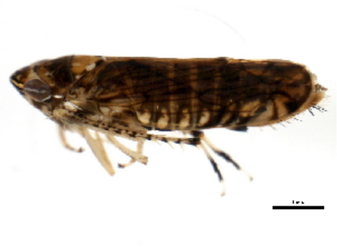

BIOUG00943-F12 [Lateral]
Scaphoideus major
Family: Cicadellidae
BIN URI: BOLD:AAY6734

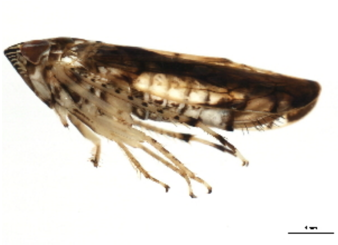

BIOUG00856-E01 [Lateral]
Scaphoideus
Family: Cicadellidae
BIN URI: BOLD:AAY9211

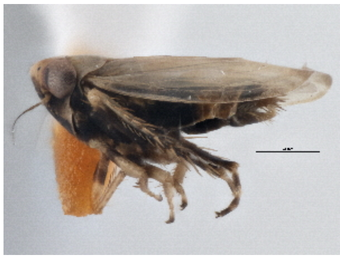

CNC#HEM305697 [Lateral]
Limotettix humidus
Family: Cicadellidae
BIN URI: BOLD:AAY8918

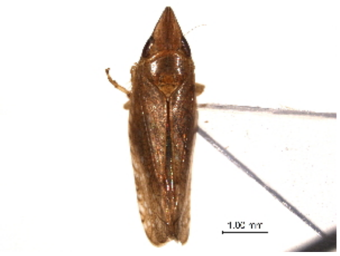

CNC#HEM405203 [Dorsal]
Scaphytopius latus
Family: Cicadellidae
BIN URI: BOLD:AAG8842

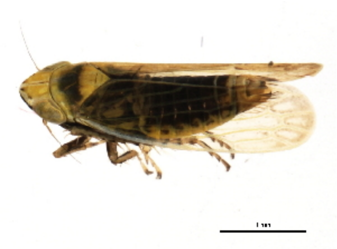

BIOUG00999-C06 [Lateral]
Sorhoanus pascuellus
Family: Cicadellidae
BIN URI: BOLD:ABV2554

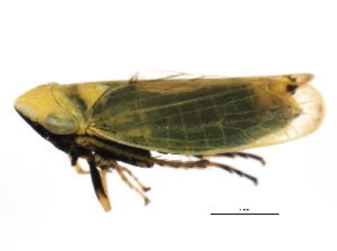

BIOUG01000-A07 [Lateral]
Diplocolenus abdominalis
Family: Cicadellidae
BIN URI: BOLD:AAG2897

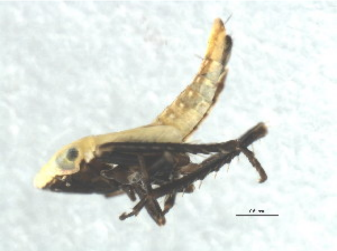

08BBHEM-417 [Lateral]
Diplocolenus
Family: Cicadellidae
BIN URI: BOLD:AAG2900

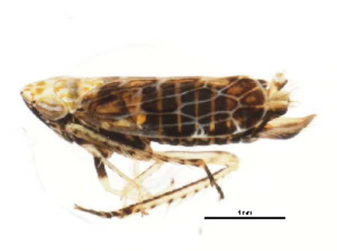

BIOUG01012-E07 [Lateral]
Errastunus ocellaris
Family: Cicadellidae
BIN URI: BOLD:AAG8839

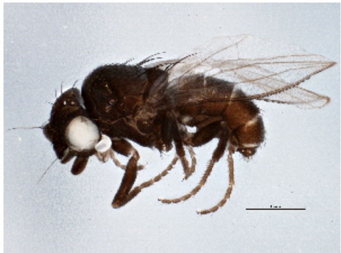

BIOUG04880-B06 [Lateral]
Milichiidae
Family: Milichiidae
BIN URI: BOLD:ACF6749

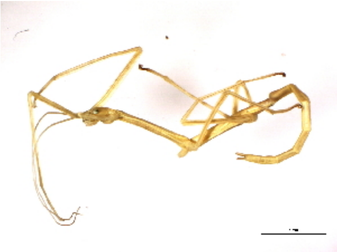

BIOUG00863-A12 [Lateral]
Diapheromera femorata
Family: Heteronemiidae
BIN URI: BOLD:AAW5365

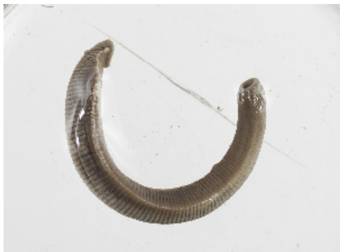

CCDB-22802 D05 [Lateral]
Clitellata

BIN URI: BOLD:ACR1438

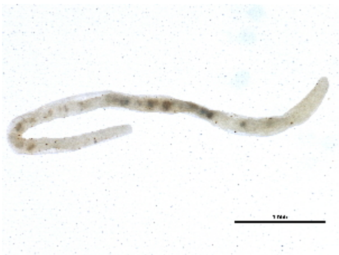

SCCWRP0264001 [Lateral]
Enchytraeidaegen
Family: Enchytraeidae
BIN URI: BOLD:AAT8916

IMAGE NOT AVAILABLE

CE6293
Enchytraeus sp.
Family: Enchytraeidae
BIN URI: BOLD:AAU1183

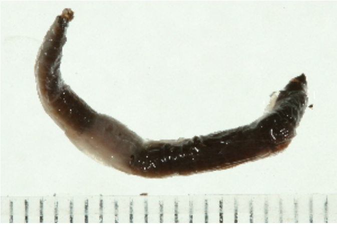

08BBANN-069 [Lateral]
Dendrobaena octaedra
Family: Lumbricidae
BIN URI: BOLD:ABX6545

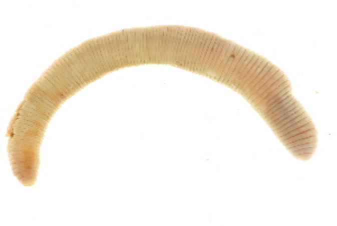

09BBANN-001 [Lateral]
Dendrodrilus rubidus
Family: Lumbricidae
BIN URI: BOLD:AAA7664

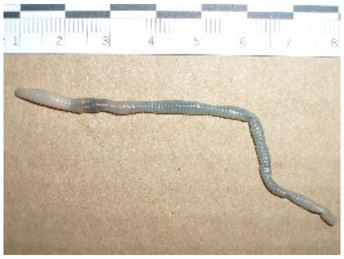

EW-CA-164 []
Octolasion tyrtaeum
Family: Lumbricidae
BIN URI: BOLD:ACF5848

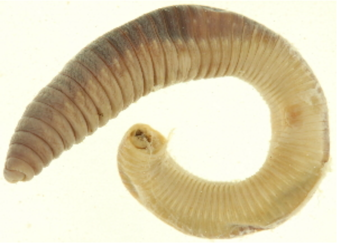

CE4366 [Lateral]
Lumbricus terrestris
Family: Lumbricidae
BIN URI: BOLD:ABZ1950

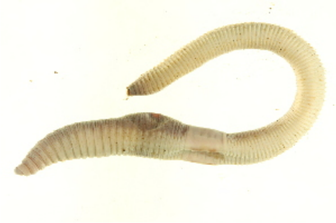

CE7111 [Lateral]
Lumbricus rubellus
Family: Lumbricidae
BIN URI: BOLD:ACE9910

IMAGE NOT AVAILABLE

BIOUG24014-A12
Lumbricus
Family: Lumbricidae

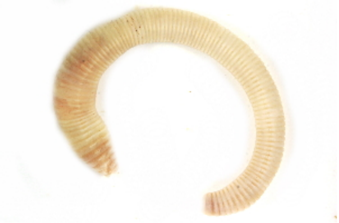

BIOUG24014-B01 [Lateral]
Aporrectodea
Family: Lumbricidae

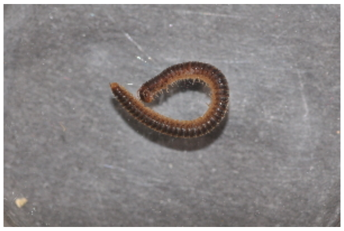

BC ZSM MYR 00156 [Lateral]
Ophyiulus pilosus
Family: Julidae
BIN URI: BOLD:AAH7470

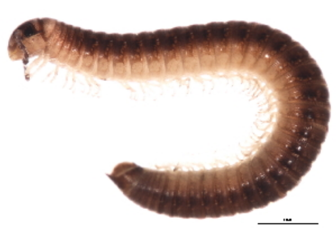

MYRAB-010 [Lateral]
Julus scandinavius
Family: Julidae
BIN URI: BOLD:AAH7469

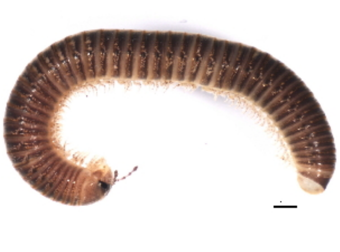

MYRAB-003 [Lateral]
Cylindroiulus caeruleocinctus
Family: Julidae
BIN URI: BOLD:AAH7472

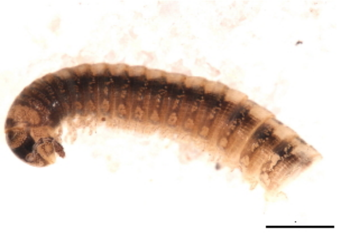

09BBMYR-083 [Lateral]
Brachyiulus pusillus
Family: Julidae
BIN URI: BOLD:AAM7944

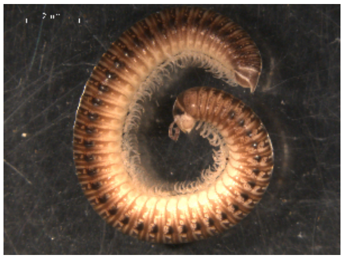

GAB-ONT-009 [Dorsolateral]
Julidae
Family: Julidae
BIN URI: BOLD:AAH7468

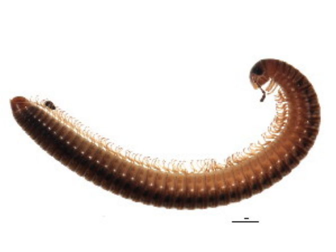

BIOUG00880-G02 [Lateral]
Julidae
Family: Julidae
BIN URI: BOLD:AAZ5766

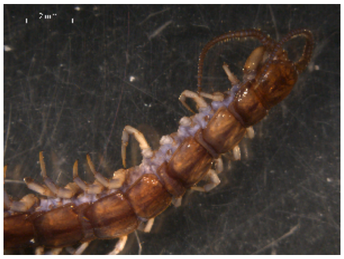

GAB-ONT-006 [Dorsal]
Lithobius
Family: Lithobiidae
BIN URI: BOLD:AAI1133

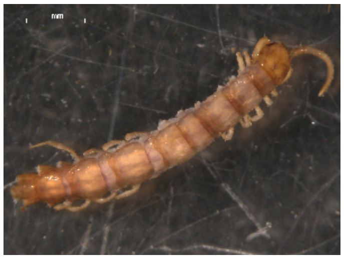

GAB-ONT-018 [Dorsal]
Lithobius
Family: Lithobiidae
BIN URI: BOLD:AAI1134

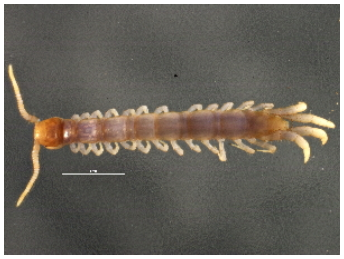

BIOUG08056-G10 [Dorsal]
Lithobius
Family: Lithobiidae
BIN URI: BOLD:AAH6432

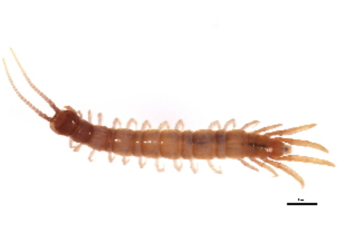

09BBMYR-086 [Dorsal]
Lithobius microps
Family: Lithobiidae
BIN URI: BOLD:AAM7904

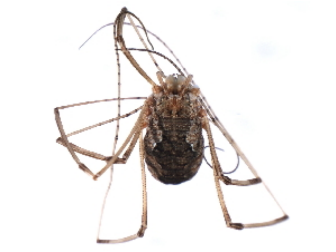

TDWG-0953 [Dorsal]
Phalangium opilio
Family: Phalangiidae
BIN URI: BOLD:AAI4346

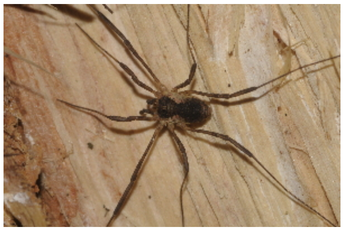

BC ZSM ARA 00045 [Adult]
Oligolophus tridens
Family: Phalangiidae
BIN URI: BOLD:AAM8194

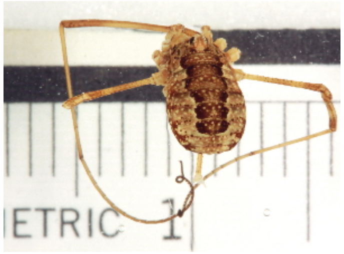

BIOUG03997-C08 [Dorsal]
Platybunus triangularis
Family: Phalangiidae
BIN URI: BOLD:ABW0506

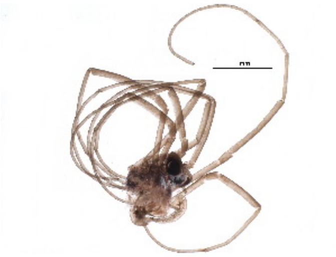

BIOUG22837-C01 [Dorsal]
Caddo
Family: Caddidae
BIN URI: BOLD:ACV9424

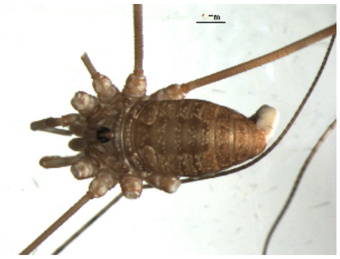

BIOUG00633-F05 [Dorsal]
Leiobunum ventricosum
Family: Sclerosomatidae
BIN URI: BOLD:AAN8497

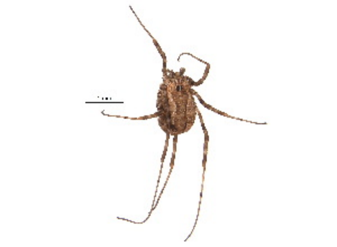

BIOUG22090-E02 [Dorsal]
Leiobunum
Family: Sclerosomatidae
BIN URI: BOLD:ACV5874

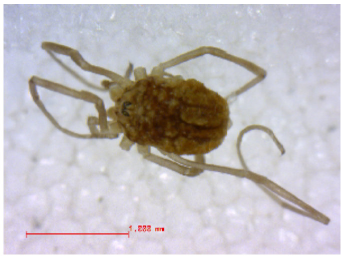

BIOUG02613-D04 [Dorsal]
Opiliones

BIN URI: BOLD:AAH7061

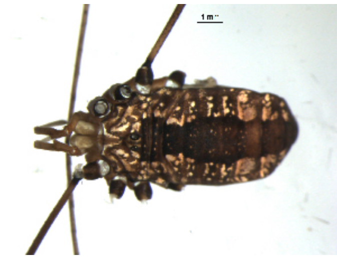

BIOUG00633-G02 [Dorsal]
Leiobunum
Family: Sclerosomatidae
BIN URI: BOLD:AAH7062

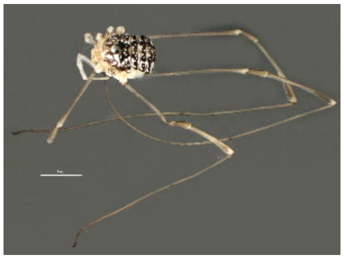

BIOUG09060-B07 [Dorsal]
Sclerosomatidae
Family: Sclerosomatidae
BIN URI: BOLD:ACL7580

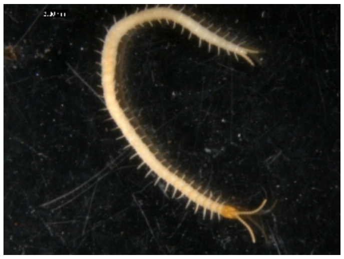

MYRNO-0010 []
Schendyla nemorensis
Family: Schendylidae
BIN URI: BOLD:AAG8560

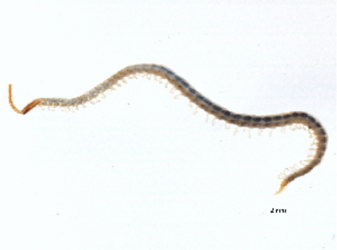

BIOUG12435-H06 [Lateral]
Geophilomorpha

BIN URI: BOLD:ACN4104

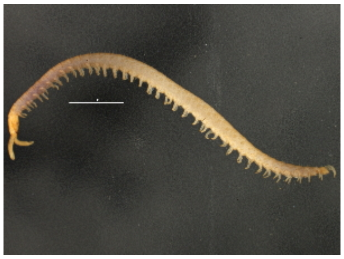

BIOUG08056-H03 [Lateral]
Geophilomorpha

BIN URI: BOLD:AAM7902

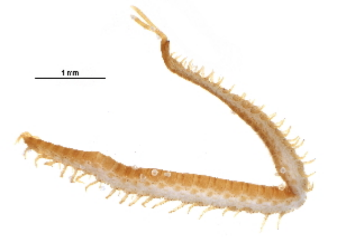

BIOUG24001-A12 [Lateral]
Schendylidae
Family: Schendylidae

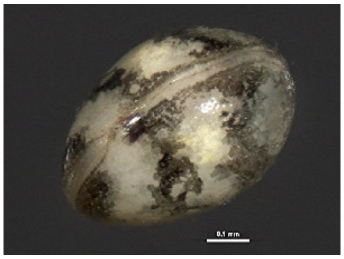

NJAK-0103 [Lateral]
Cypridopsis vidua
Family: Cyprididae
BIN URI: BOLD:AAH0892

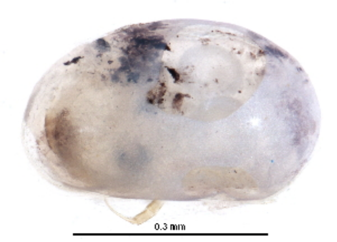

BIOUG24026-C01 [Lateral]
Cypria
Family: Candonidae

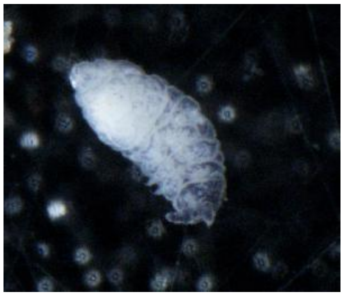

ONTCOL0050 []
Odontellidae
Family: Odontellidae
BIN URI: BOLD:AAC1432

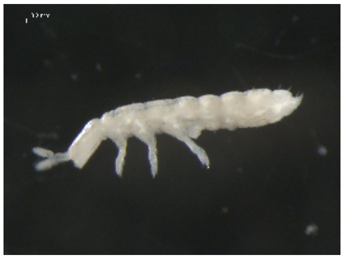

CHU06-COL-1148 [Lateral]
Protaphorura
Family: Onychiuridae
BIN URI: BOLD:AAB0246

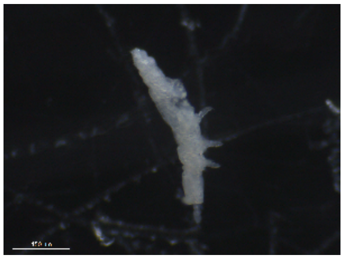

12037-D06 [Dorsal]
Tullbergiidae
Family: Tullbergiidae
BIN URI: BOLD:AAG0680

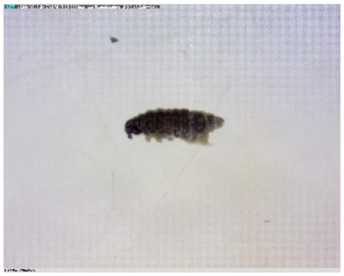

BIOUG03204-D05 [Lateral]
Collembola

BIN URI: BOLD:ACC7275

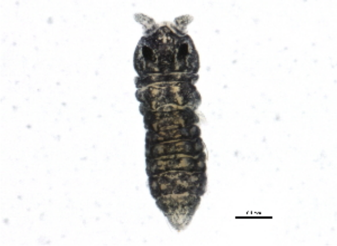

BIOUG04812-E08 [Dorsal]
Neanuridae
Family: Neanuridae
BIN URI: BOLD:AAB7286

IMAGE NOT AVAILABLE

BIOUG22629-F12
Poduromorpha

BIN URI: BOLD:ACV5680

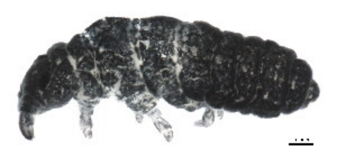

BIOUG04093-E11 [Lateral]
Poduromorpha

BIN URI: BOLD:ACF1937

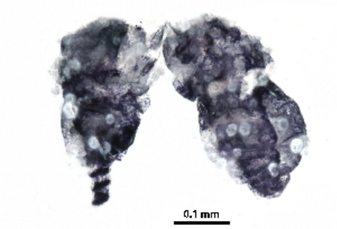

BIOUG22846-D12 [Lateral]
Poduromorpha

BIN URI: BOLD:ACV6302

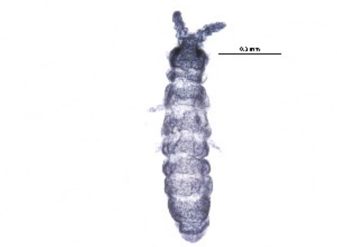

BIOUG22846-A04 [Dorsal]
Poduromorpha

BIN URI: BOLD:ACV7152

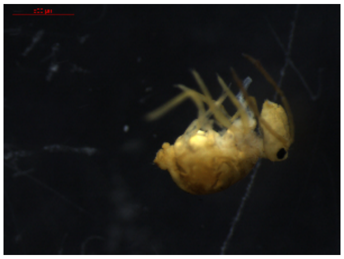

01336-C10 [Lateral]
Dicyrtomina minuta
Family: Dicyrtomidae
BIN URI: BOLD:ACQ0383

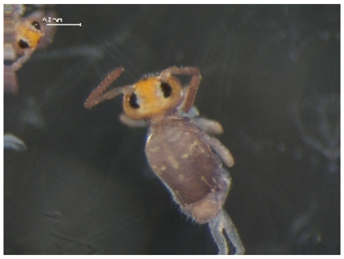

CHU06-COL-1010 [Dorsal]
Deuterosminthurus sp1
Family: Bourletiellidae
BIN URI: BOLD:AAB7915

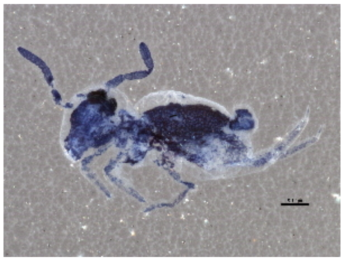

BIOUG05516-D08 [Lateral]
Bourletiella
Family: Bourletiellidae
BIN URI: BOLD:ABX0027

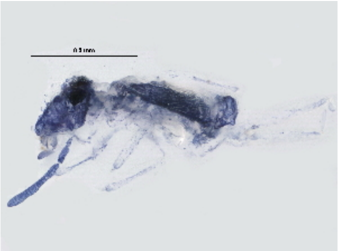

BIOUG13497-G01 [Lateral]
Collembola

BIN URI: BOLD:ACN4506

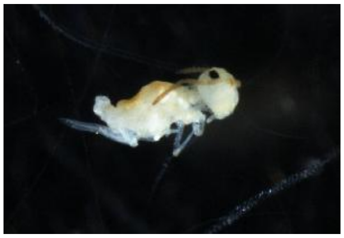

ONTCOL0051 []
Bourletiellidae
Family: Bourletiellidae
BIN URI: BOLD:AAB7913

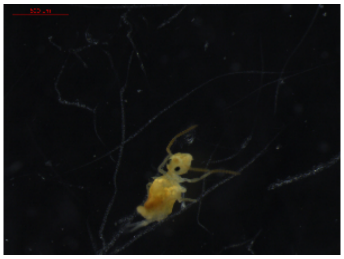

01336-B08 [Dorsal]
Bourletiellidae
Family: Bourletiellidae
BIN URI: BOLD:AAZ2180

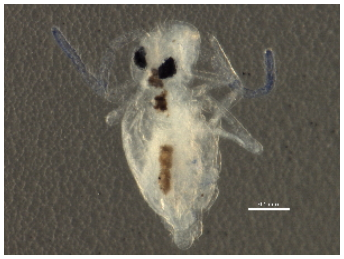

BIOUG09351-F01 [Dorsal]
Collembola

BIN URI: BOLD:ACJ7454

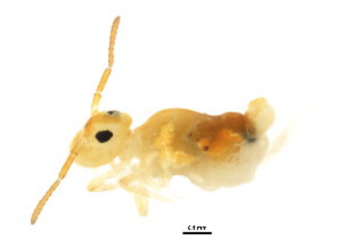

BIOUG02093-G01 [Lateral]
Collembola

BIN URI: BOLD:AAB7914

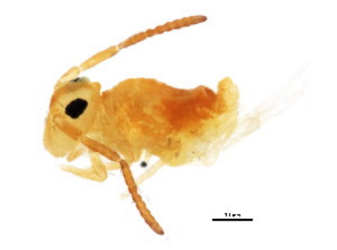

BIOUG04016-H04 [Lateral]
Bourletiellidae
Family: Bourletiellidae
BIN URI: BOLD:ACC0359

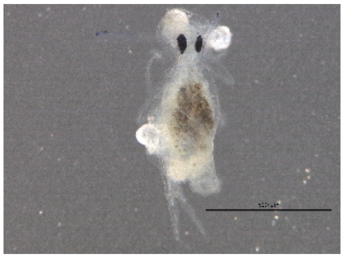

BIOUG16104-G03 [Dorsal]
Bourletiella
Family: Bourletiellidae
BIN URI: BOLD:ACV5610

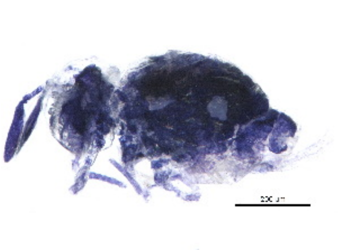

BIOUG11945-A05 [Lateral]
Collembola

BIN URI: BOLD:ABA5370

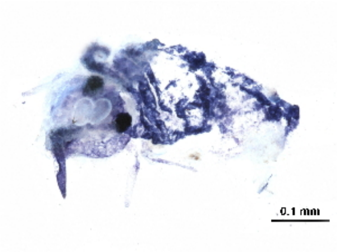

BIOUG21887-G08 [Lateral]
Symphypleona

BIN URI: BOLD:ACV4618

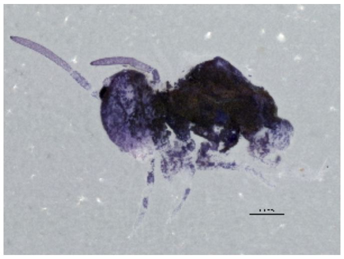

BIOUG08570-E02 [Lateral]
Sminthurinus elegans
Family: Katiannidae
BIN URI: BOLD:AAB3496

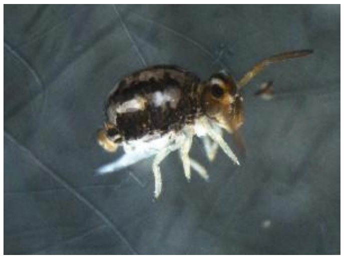

ONTCOL0071 []
Sminthurinus elegans
Family: Katiannidae
BIN URI: BOLD:AAB3495

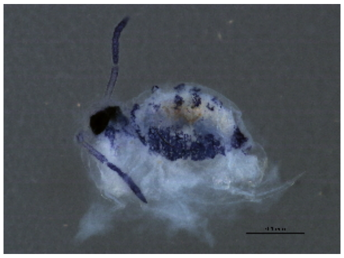

BIOUG09123-C09 [Lateral]
Collembola

BIN URI: BOLD:ACJ9068

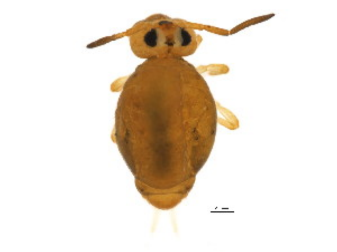

BIOUG02093-E01 [Dorsal]
Collembola

BIN URI: BOLD:AAI4706

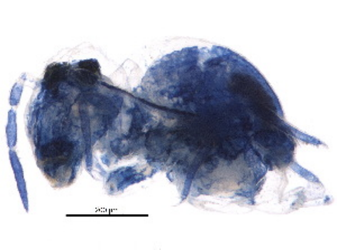

BIOUG20566-C12 [Lateral]
Symphypleona

BIN URI: BOLD:ACV5788

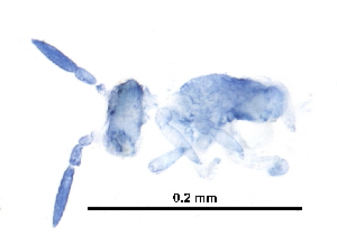

BIOUG21883-F04 [Lateral]
Symphypleona

BIN URI: BOLD:ACV4139

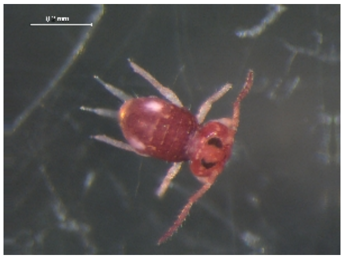

CHU06-COL-1060 [Dorsal]
Sminthurides sp1
Family: Sminthurididae
BIN URI: BOLD:AAG3938

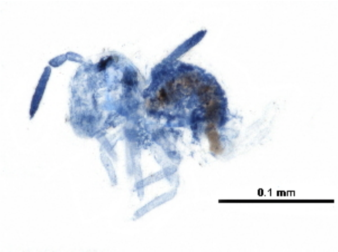

BIOUG21885-G05 [Lateral]
Symphypleona

BIN URI: BOLD:ACV4567

IMAGE NOT AVAILABLE

BIOUG21885-F07
Symphypleona

BIN URI: BOLD:ACV4365

IMAGE NOT AVAILABLE

BIOUG21898-D02
Symphypleona

BIN URI: BOLD:ACV5320

IMAGE NOT AVAILABLE

BIOUG21887-C02
Symphypleona

IMAGE NOT AVAILABLE

BIOUG21887-B09
Symphypleona

BIN URI: BOLD:ACV4528

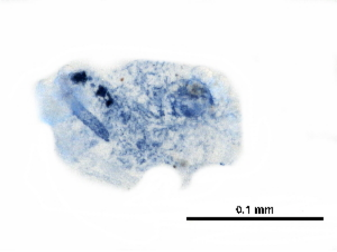

BIOUG21885-G07 [Lateral]
Symphypleona

BIN URI: BOLD:ACV4522

IMAGE NOT AVAILABLE

BIOUG21887-B08
Symphypleona

IMAGE NOT AVAILABLE

BIOUG20566-A10
Isotomidae
Family: Isotomidae
BIN URI: BOLD:ACV5561

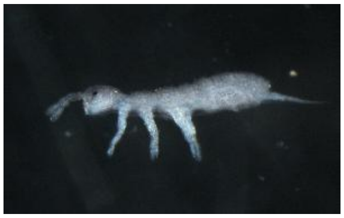

ONTCOL0066 ▯
Parisotoma notabilis L2
Family: Isotomidae
BIN URI: BOLD:AAB2868

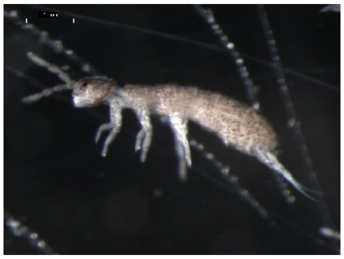

ONTCOL0477 ▯
Parisotoma notabilis L2
Family: Isotomidae
BIN URI: BOLD:AAB2869

IMAGE NOT AVAILABLE

BIOUG21883-C06
Parisotoma notabilis
Family: Isotomidae

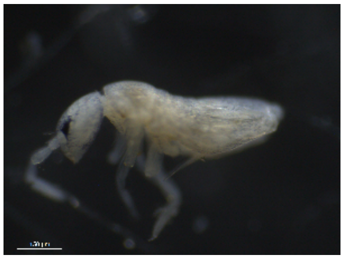

16709-D08 [Lateral]
Parisotoma notabilis L2
Family: Isotomidae
BIN URI: BOLD:AAB2870

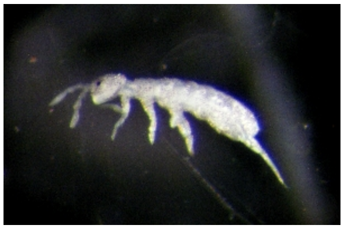

ONTCOL0378 ▯
Parisotoma notabilis L1
Family: Isotomidae
BIN URI: BOLD:ABZ8106

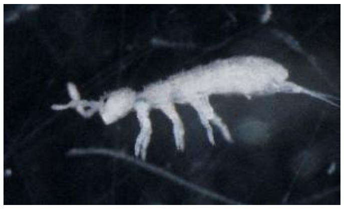

ONTCOL0040 ▯
Parisotoma notabilis L1
Family: Isotomidae
BIN URI: BOLD:AAA4157

IMAGE NOT AVAILABLE

BIOUG21887-H10
Isotomidae
Family: Isotomidae
BIN URI: BOLD:ACV4617

IMAGE NOT AVAILABLE

ONTCOL0168
Isotomidae
Family: Isotomidae
BIN URI: BOLD:AAB2085

IMAGE NOT AVAILABLE

BIOUG21887-H05
Isotomidae
Family: Isotomidae

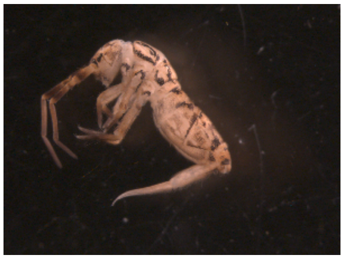

DAREK-0039 [Lateral]
Orchesella villosa
Family: Entomobryidae
BIN URI: BOLD:AAA8726

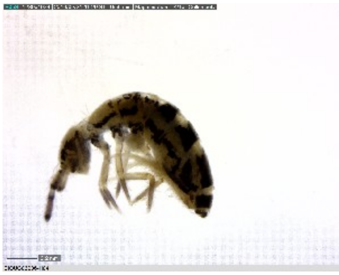

BIOUG03206-H04 [latteral]
Collembola

BIN URI: BOLD:ABA5351

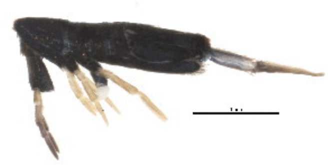

BIOUG02130-G04 [Lateral]
Collembola

BIN URI: BOLD:AAB8452

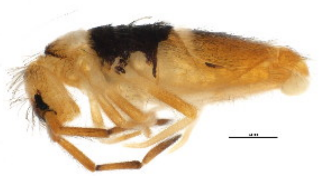

BIOUG04310-G04 [Lateral]
Entomobrya atrocincta
Family: Entomobryidae
BIN URI: BOLD:ACE5102

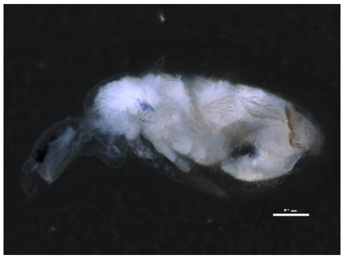

BIOUG07135-A05 [Lateral]
Lepidocyrtus
Family: Entomobryidae
BIN URI: BOLD:ACI2943

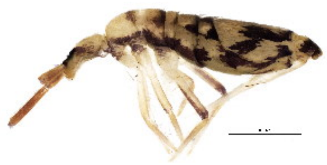

BIOUG04016-H05 [Lateral]
Entomobrya
Family: Entomobryidae
BIN URI: BOLD:ACL6239

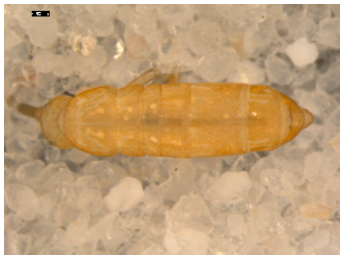

BIOUG00037-81 [Dorsal]
Tomocerus
Family: Tomoceridae
BIN URI: BOLD:AAA7969

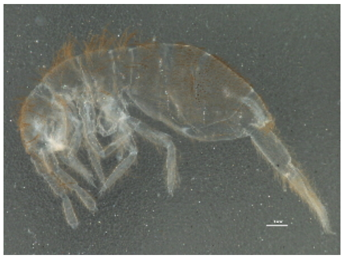

BIOUG01653-A09 [Lateral]
Collembola

BIN URI: BOLD:ABA5361

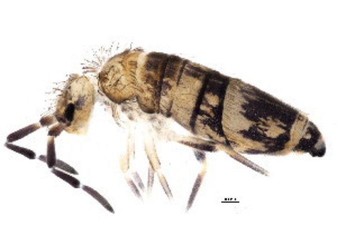

BIOUG03735-F01 [Lateral]
Lepidocyrtus
Family: Entomobryidae
BIN URI: BOLD:ACM2009

IMAGE NOT AVAILABLE

BIOUG22628-B03
Lepidocyrtus
Family: Entomobryidae

IMAGE NOT AVAILABLE

BIOUG20572-A11
Poduromorpha

BIN URI: BOLD:ACV4568

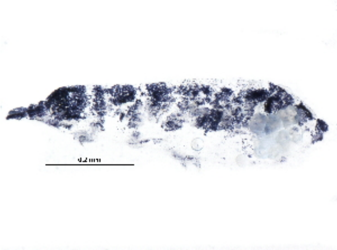

BIOUG15211-D04 [Lateral]
Collembola

BIN URI: BOLD:ACL3025

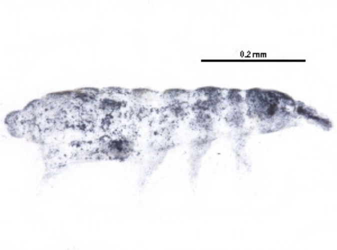

BIOUG22846-A05 [Lateral]
Odontellidae
Family: Odontellidae
BIN URI: BOLD:ACV7054

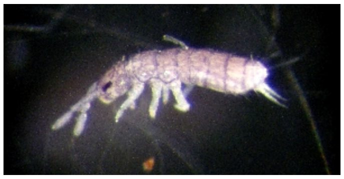

ONTCOL0430 []
Isotomidae
Family: Isotomidae
BIN URI: BOLD:AAH7174

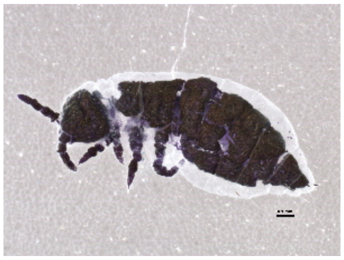

BIOUG05516-D05 [Lateral]
Poduromorpha

BIN URI: BOLD:AAA4811

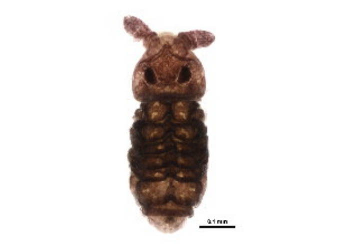

BIOUG02093-D10 [Dorsal]
Collembola

BIN URI: BOLD:AAI3738

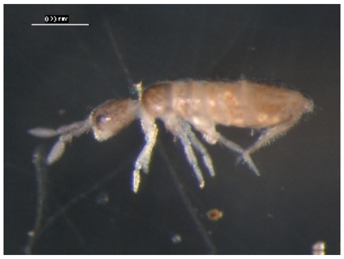

ONTCOL0588 [Lateral]
Entomobryidae
Family: Entomobryidae
BIN URI: BOLD:AAA9292

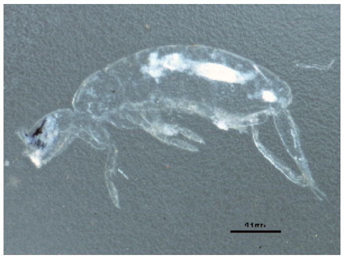

BIOUG04597-D11 [Lateral]
Entomobryidae
Family: Entomobryidae
BIN URI: BOLD:ACE2737

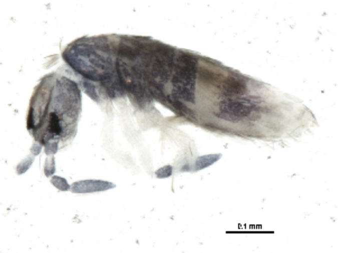

BIOUG04291-C12 [Lateral]
Entomobryidae
Family: Entomobryidae
BIN URI: BOLD:ACD9424

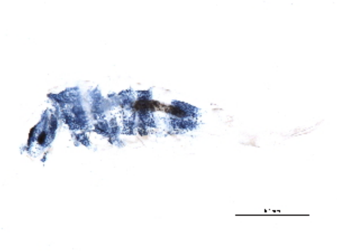

BIOUG11033-F11 [Lateral]
Entomobryidae
Family: Entomobryidae
BIN URI: BOLD:ACK8463

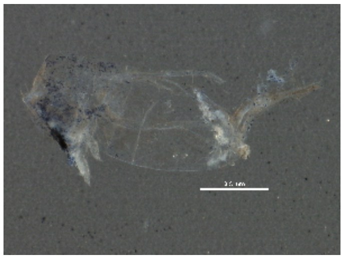

BIOUG06960-A01 [Lateral]
Entomobryomorpha

BIN URI: BOLD:ACH3809

IMAGE NOT AVAILABLE

BIOUG21775-B08
Entomobryomorpha

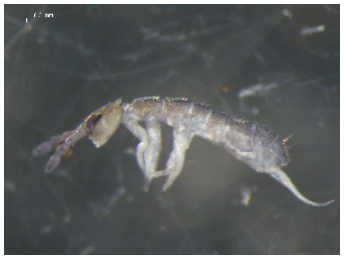

CHU06-COL-1196 [Lateral]
Isotomidae
Family: Isotomidae
BIN URI: BOLD:AAI2077

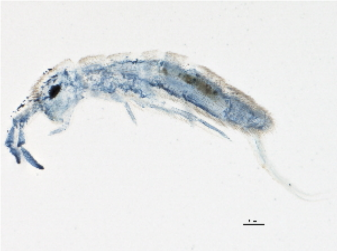

BIOUG13043-A11 [Lateral]
Isotomidae
Family: Isotomidae
BIN URI: BOLD:ACJ0127

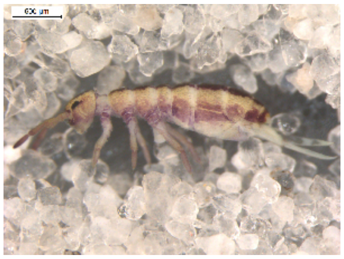

BIOUG00506-64 [Lateral]
Isotomidae
Family: Isotomidae
BIN URI: BOLD:AAN6552

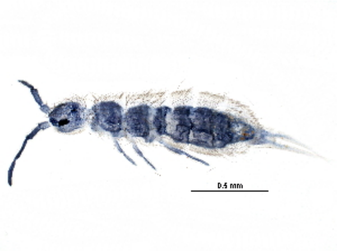

BIOUG21887-A07 [Lateral]
Entomobryomorpha

BIN URI: BOLD:ACV4619

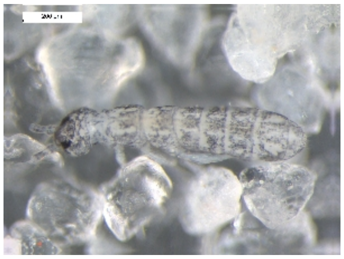

BIOUG00172-59 [Dorsal]
Isotomidae
Family: Isotomidae
BIN URI: BOLD:AAN6535

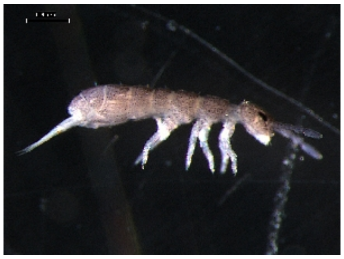

ONTCOL0478 []
Isotomidae
Family: Isotomidae
BIN URI: BOLD:AAA7162

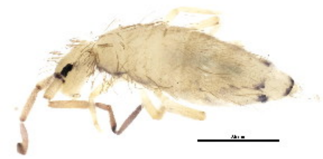

BIOUG02130-G05 [Lateral]
Collembola

BIN URI: BOLD:AAA7246

IMAGE NOT AVAILABLE

BIOUG21887-D03
Entomobryomorpha

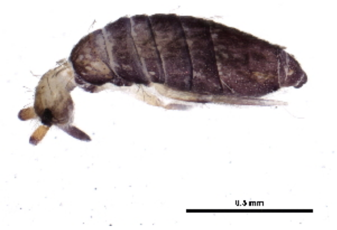

BIOUG04503-F02 [Lateral]
Entomobrya sp.
Family: Entomobryidae
BIN URI: BOLD:AAA7249

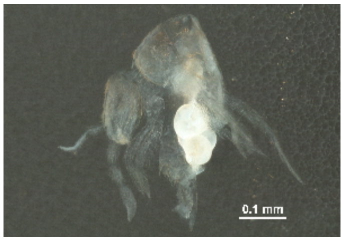

BIOUG21887-A01 [Lateral]
Entomobryomorpha

BIN URI: BOLD:ACV4512

IMAGE NOT AVAILABLE

BIOUG20572-B07
Entomobryomorpha

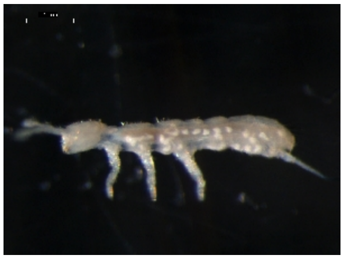

ONTCOL0472 []
Isotomidae
Family: Isotomidae
BIN URI: BOLD:ACJ0088

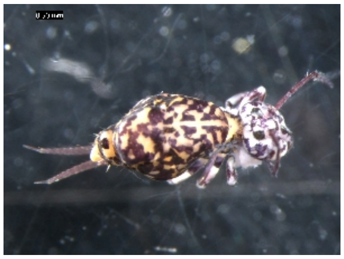

COLC-DAV46 [Lateral]
Dicyrtomina
Family: Dicyrtomidae
BIN URI: BOLD:AAF5859

IMAGE NOT AVAILABLE

BIOUG20566-C04
Symphypleona

BIN URI: BOLD:ACV4456

IMAGE NOT AVAILABLE

BIOUG24001-A01
Collembola

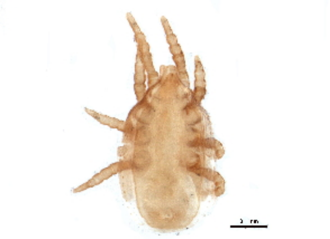

BIOUG07381-G01 [Dorsal]
Dinychidae
Family: Dinychidae
BIN URI: BOLD:ACG2115

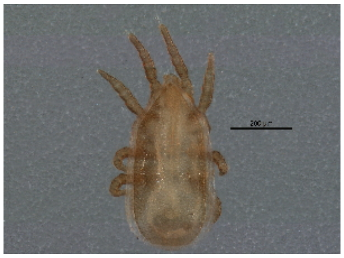

BIOUG07274-A01 [Dorsal]
Dinychidae
Family: Dinychidae
BIN URI: BOLD:ACI9637

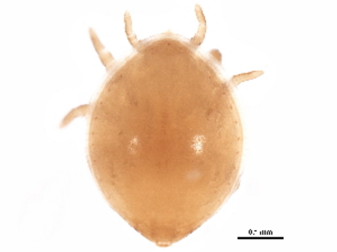

BIOUG23322-A05 [Dorsal]
Uroobovella
Family: Urodinychidae
BIN URI: BOLD:AAY9562

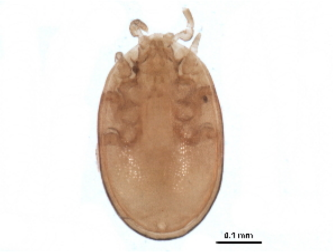

BIOUG24000-F12 [Dorsal]
Trematuridae
Family: Trematuridae

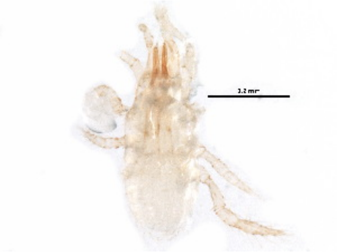

BIOUG21899-C05 [Dorsal]
Mesostigmata

BIN URI: BOLD:ACV5531

IMAGE NOT AVAILABLE

BIOUG22846-E11
Mesostigmata

BIN URI: BOLD:ACV7505

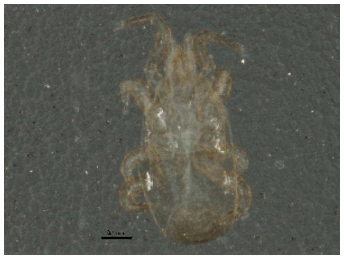

BIOUG05505-C09 [Lateral]
Digamasellidae
Family: Digamasellidae
BIN URI: BOLD:ABW5651

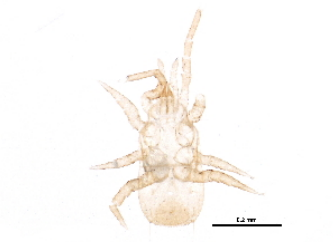

BIOUG06909-D02 [Dorsal]
Mesostigmata

BIN URI: BOLD:ACC6566

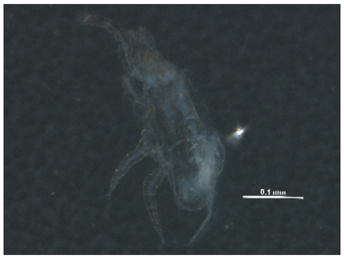

BIOUG16226-C05 [Dorsal]
Digamasellidae
Family: Digamasellidae
BIN URI: BOLD:ACL9334

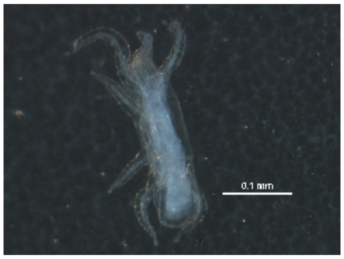

BIOUG16226-E07 [Dorsal]
Digamasellidae
Family: Digamasellidae
BIN URI: BOLD:ACR0807

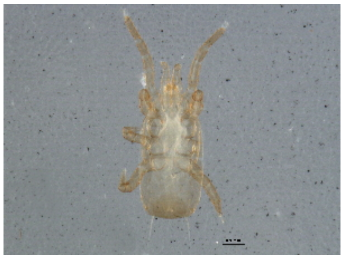

BIOUG05872-E05 [Dorsal]
Digamasellidae
Family: Digamasellidae
BIN URI: BOLD:ACG6639

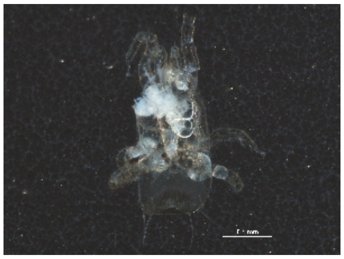

BIOUG10650-A06 [Dorsal]
Digamasellidae
Family: Digamasellidae
BIN URI: BOLD:ACM8656

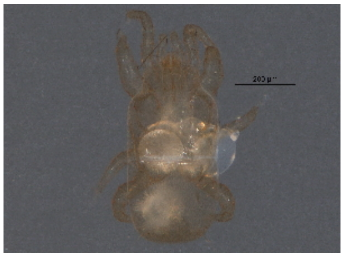

BIOUG11727-H03 [Dorsal]
Digamasellidae
Family: Digamasellidae
BIN URI: BOLD:ACM3263

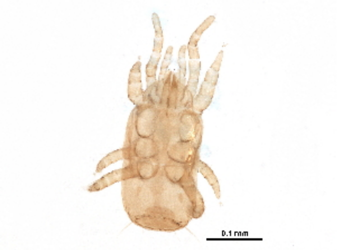

BIOUG22354-H05 [Dorsal]
Digamasellidae
Family: Digamasellidae
BIN URI: BOLD:ACV4509

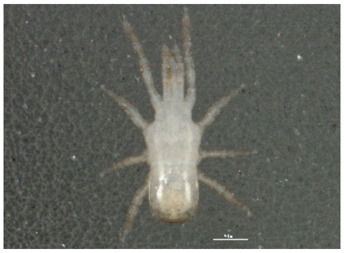

BIOUG04017-G10 [Dorsal]
Digamasellidae
Family: Digamasellidae
BIN URI: BOLD:ACM1195

IMAGE NOT AVAILABLE

BIOUG23322-F09
Digamasellidae
Family: Digamasellidae
BIN URI: BOLD:ACW0844

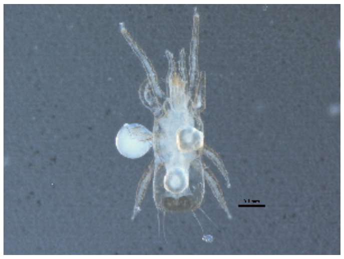

BIOUG06813-B01 [Dorsal]
Digamasellidae
Family: Digamasellidae
BIN URI: BOLD:ACM1395

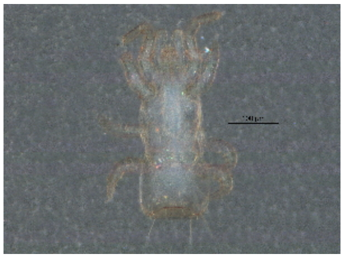

BIOUG07274-C06 [Dorsal]
Digamasellidae
Family: Digamasellidae
BIN URI: BOLD:ACH4162

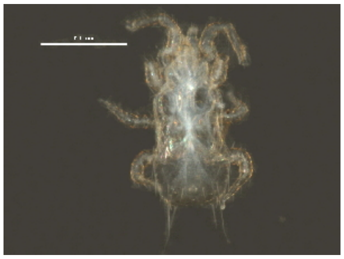

BIOUG07003-H09 [Dorsal]
Digamasellidae
Family: Digamasellidae
BIN URI: BOLD:ACG9322

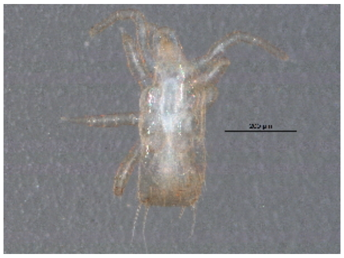

BIOUG07273-B12 [Dorsal]
Digamasellidae
Family: Digamasellidae
BIN URI: BOLD:ACI5517

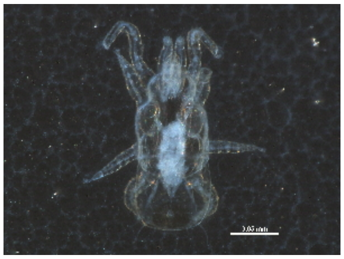

BIOUG10650-G12 [Dorsal]
Digamasellidae
Family: Digamasellidae
BIN URI: BOLD:ACI5353

IMAGE NOT AVAILABLE

BIOUG24030-G07
Rhodacarellus silesiacus
Family: Rhodacaridae

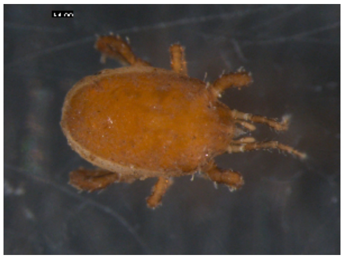

08MIONT-0091 [Dorsal]
Macrochelidae
Family: Macrochelidae
BIN URI: BOLD:AAF9126

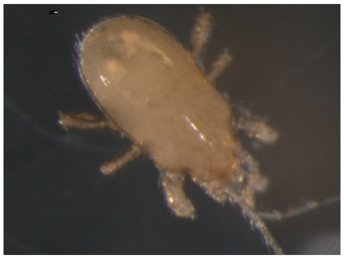

08MIONT-0246 [Dorsal]
Parasitidae
Family: Parasitidae
BIN URI: BOLD:AAF9125

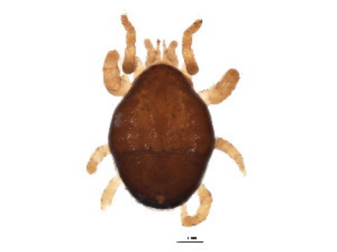

BIOUG03211-F12 [Dorsal]
Parasitidae
Family: Parasitidae
BIN URI: BOLD:AAF9285

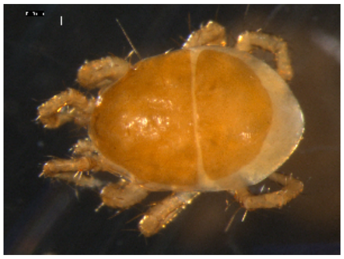

BIOUG00036-10 [Dorsal]
Parasitidae
Family: Parasitidae
BIN URI: BOLD:AAM7982

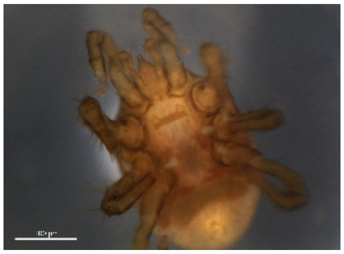

BIOUG10944-D11 [V]
Parasitidae
Family: Parasitidae
BIN URI: BOLD:AAZ0396

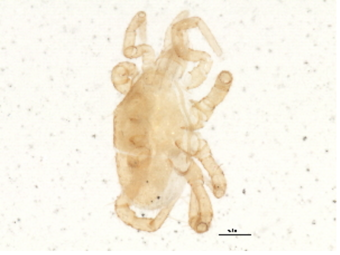

BIOUG05559-B12 [Dorsal]
Parasitidae
Family: Parasitidae
BIN URI: BOLD:AAN6711

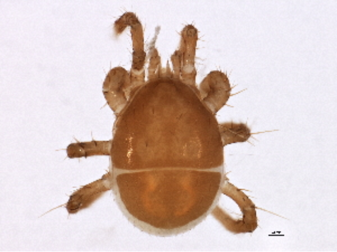

BIOUG03832-H11 [Dorsal]
Parasitidae
Family: Parasitidae
BIN URI: BOLD:ABY2035

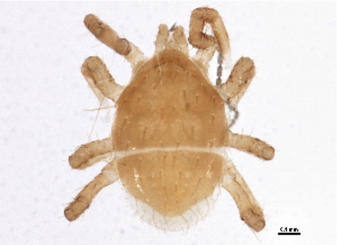

BIOUG03832-A11 [Dorsal]
Parasitidae
Family: Parasitidae
BIN URI: BOLD:ACC0819

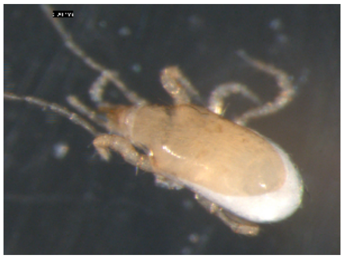

08MIONT-0120 [Dorsal]
Leptolaelapidae
Family: Leptolaelapidae
BIN URI: BOLD:AAF9134

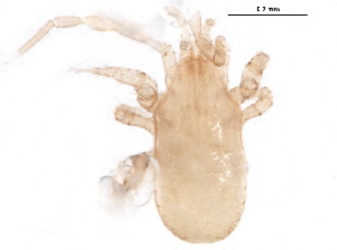

BIOUG21899-B05 [Dorsal]
Mesostigmata

BIN URI: BOLD:ACO2718

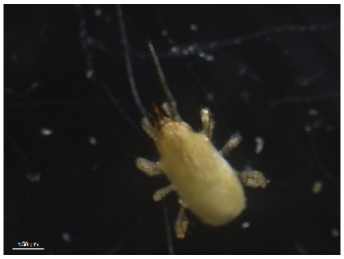

12037-A02 [Dorsal]
Arachnida

BIN URI: BOLD:ABX1977

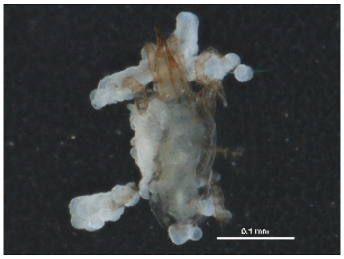

BIOUG21884-A11 [Dorsal]
Mesostigmata

BIN URI: BOLD:ACV5225

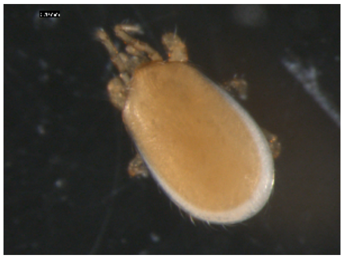

08MIONT-0165 [Dorsal]
Pachylaelapidae
Family: Pachylaelapidae
BIN URI: BOLD:AAF9143

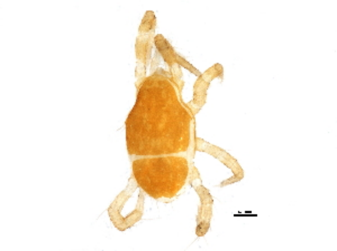

BIOUG03530-F05 [Dorsal]
Parasitidae
Family: Parasitidae
BIN URI: BOLD:ACC9233

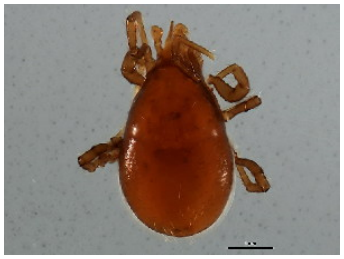

TDWG-0490 [Dorsal]
Parasitidae
Family: Parasitidae
BIN URI: BOLD:AAF9093

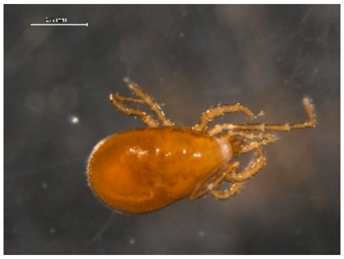

08MIONT-0415 [Dorsal]
Parasitidae
Family: Parasitidae
BIN URI: BOLD:AAF9229

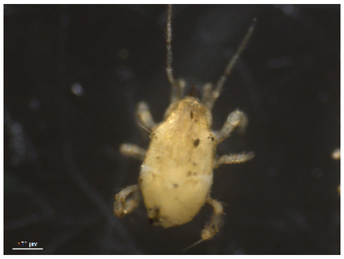

11982-C01 [Dorsal]
Arachnida

BIN URI: BOLD:AAF9186

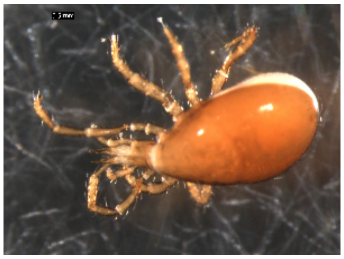

08DPMIT-0891 [Dorsal]
Parasitidae
Family: Parasitidae
BIN URI: BOLD:AAF9099

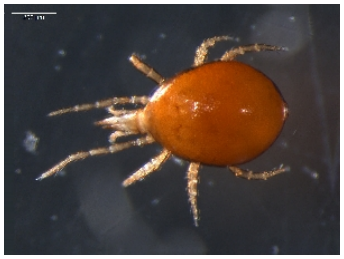

DPMIT-20-28 [Dorsal]
Parasitidae
Family: Parasitidae
BIN URI: BOLD:AAF9096

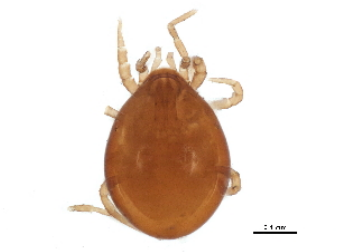

BIOUG21882-B12 [Lateral]
Parasitidae
Family: Parasitidae
BIN URI: BOLD:ACV6452

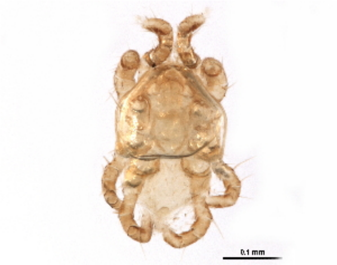

BIOUG23322-A02 [Dorsal]
Parasitidae
Family: Parasitidae
BIN URI: BOLD:ACW0761

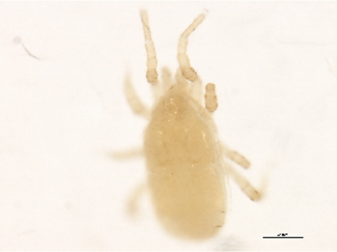

MITEPAR-0092 [Dorsal]
Ascidae
Family: Ascidae
BIN URI: BOLD:AAN6706

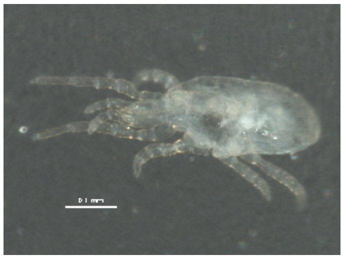

BIOUG01769-F03 [Dorsal]
Ascidae
Family: Ascidae
BIN URI: BOLD:ABW2693

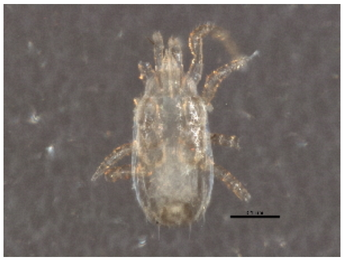

BIOUG05655-H09 [Lateral]
Ascidae
Family: Ascidae
BIN URI: BOLD:ACF8021

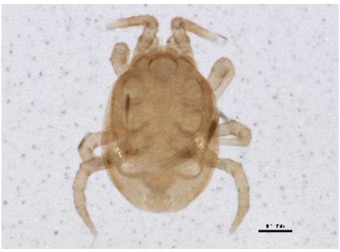

BIOUG03832-A05 [Dorsal]
Proctolaelaps
Family: Melicharidae
BIN URI: BOLD:AAZ5835

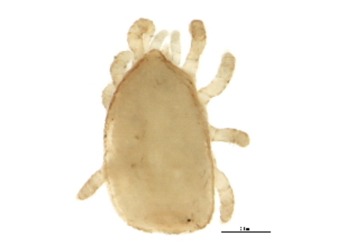

BIOUG01238-D06 [Dorsal]
Ascidae
Family: Ascidae
BIN URI: BOLD:ACM1800

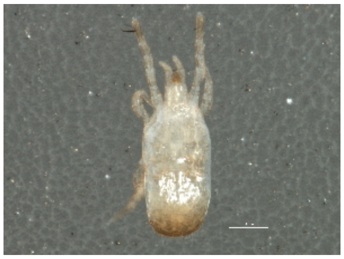

BIOUG04832-D06 [Dorsal]
Ascidae
Family: Ascidae
BIN URI: BOLD:ACD8928

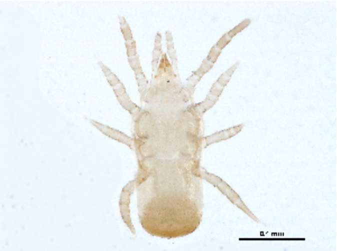

BIOUG21882-C03 [Lateral]
Mesostigmata

BIN URI: BOLD:ACV5970

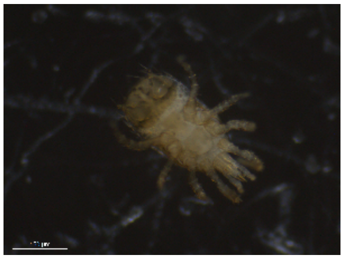

16711-G04 [Ventral]
Arachnida

BIN URI: BOLD:ABW8100

IMAGE NOT AVAILABLE

BIOUG24030-C11
Laelapidae
Family: Laelapidae

IMAGE NOT AVAILABLE

BIOUG22364-B08
Mesostigmata

BIN URI: BOLD:ACV4707

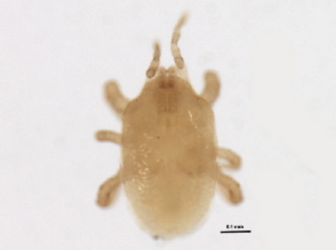

BIOUG00209-D05 [Dorsal]
Macrocheles
Family: Macrochelidae
BIN URI: BOLD:AAM8020

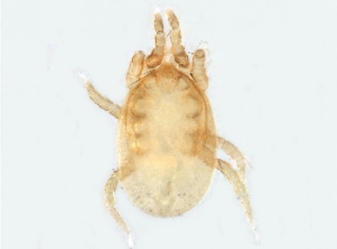

BIOUG09351-F11 [Dorsal]
Laelapidae
Family: Laelapidae
BIN URI: BOLD:ACL6729

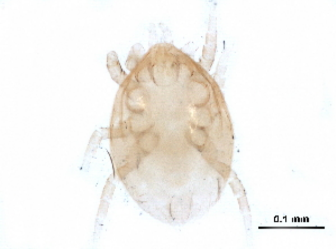

BIOUG22240-F08 [Dorsal]
Laelapidae
Family: Laelapidae
BIN URI: BOLD:ACV2285

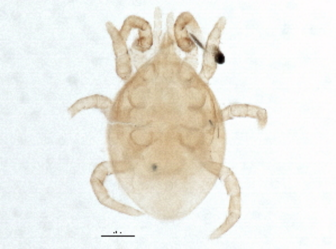

BIOUG05718-D05 [Dorsal]
Laelapidae
Family: Laelapidae
BIN URI: BOLD:ACF7854

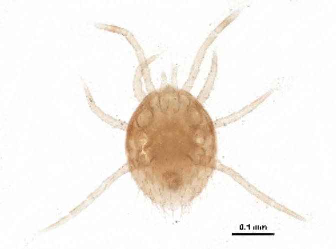

BIOUG22630-C11 [Dorsal]
Mesostigmata

BIN URI: BOLD:ACV4995

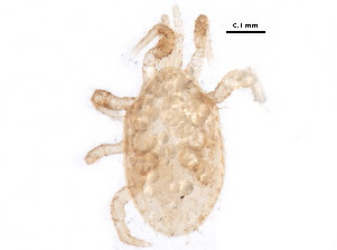

BIOUG22841-F10 [Dorsal]
Laelapidae
Family: Laelapidae
BIN URI: BOLD:ACV8565

IMAGE NOT AVAILABLE

BIOUG23322-E01
Phytoseiidae
Family: Phytoseiidae
BIN URI: BOLD:ACK6743

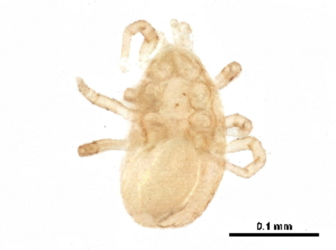

BIOUG22631-H03 [Dorsal]
Phytoseiidae
Family: Phytoseiidae
BIN URI: BOLD:ACV5408

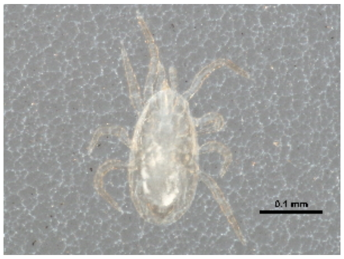

BIOUG22631-E03 [Dorsal]
Phytoseiidae
Family: Phytoseiidae
BIN URI: BOLD:ACV5953

IMAGE NOT AVAILABLE

BIOUG22931-D12
Phytoseiidae
Family: Phytoseiidae
BIN URI: BOLD:ACV5971

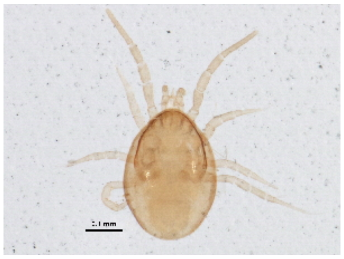

BIOUG05716-H11 [Dorsal]
Phytoseiidae
Family: Phytoseiidae
BIN URI: BOLD:ACF8412

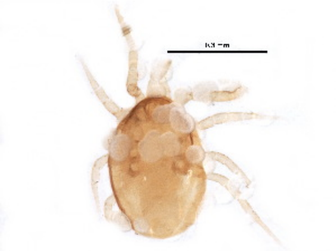

BIOUG22873-G05 [Dorsal]
Phytoseiidae
Family: Phytoseiidae
BIN URI: BOLD:ACV6731

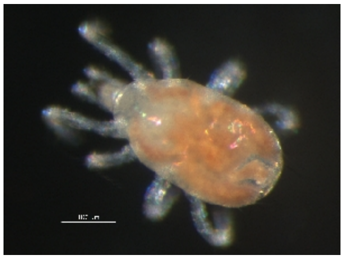

BIOUG05654-B11 [Lateral]
Phytoseiidae
Family: Phytoseiidae
BIN URI: BOLD:ACF8044

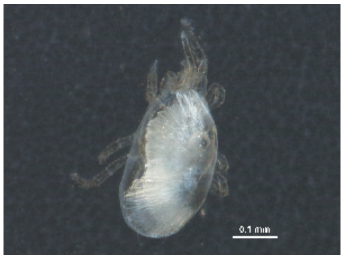

BIOUG10650-D05 [Dorsal]
Phytoseiidae
Family: Phytoseiidae
BIN URI: BOLD:ACK8761

IMAGE NOT AVAILABLE

BIOUG22841-E12
Phytoseiidae
Family: Phytoseiidae
BIN URI: BOLD:ACW0290

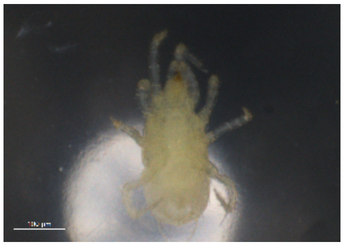

BIOUG10944-C12 [V]
Phytoseiidae
Family: Phytoseiidae
BIN URI: BOLD:ACL5806

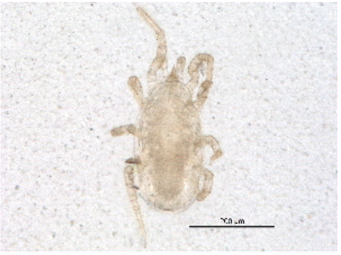

BIOUG06925-B03 [Dorsal]
Phytoseiidae
Family: Phytoseiidae
BIN URI: BOLD:ACI5481

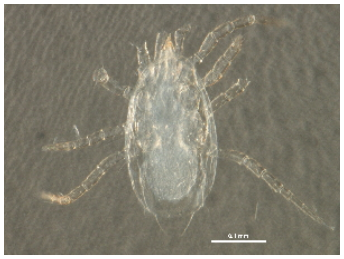

BIOUG06962-F05 [Dorsal]
Phytoseiidae
Family: Phytoseiidae
BIN URI: BOLD:ACD9359

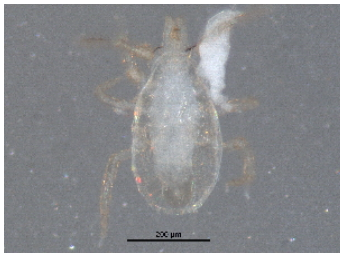

BIOUG09853-H03 [Dorsal]
Phytoseiidae
Family: Phytoseiidae
BIN URI: BOLD:ACM2034

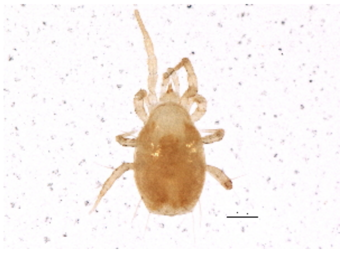

BIOUG04017-E01 [Dorsal]
Phytoseiidae
Family: Phytoseiidae
BIN URI: BOLD:ACC1906

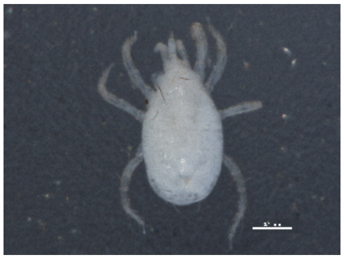

BIOUG03830-F11 [Dorsal]
Phytoseiidae
Family: Phytoseiidae
BIN URI: BOLD:ACB9781

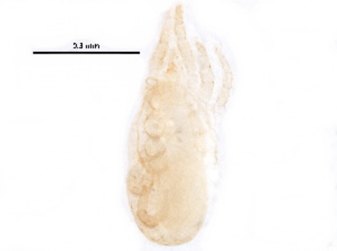

BIOUG22873-G01 [Dorsal]
Phytoseiidae
Family: Phytoseiidae
BIN URI: BOLD:ACW0597

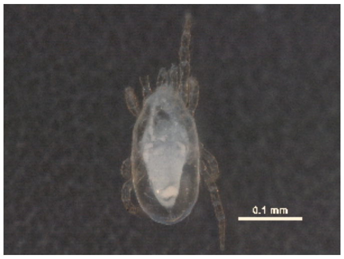

BIOUG22240-F03 [Dorsal]
Phytoseiidae
Family: Phytoseiidae
BIN URI: BOLD:ACV2903

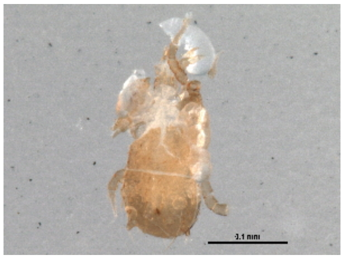

BIOUG21899-A11 [Dorsal]
Ascidae
Family: Ascidae
BIN URI: BOLD:ACV6067

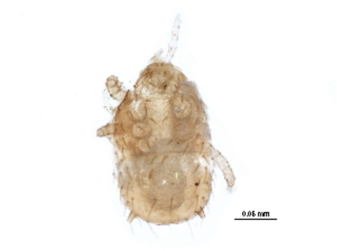

BIOUG21882-C11 [Lateral]
Ascidae
Family: Ascidae
BIN URI: BOLD:ACV4959

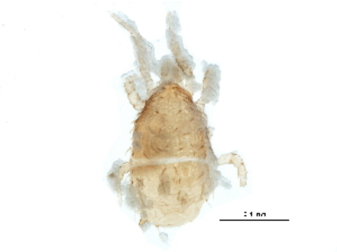

BIOUG21882-D01 [Lateral]
Ascidae
Family: Ascidae
BIN URI: BOLD:ACV3486

IMAGE NOT AVAILABLE

BIOUG24005-G05
Ascidae
Family: Ascidae

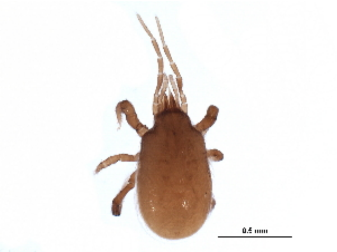

BIOUG24000-A11 [Dorsal]
Mesostigmata

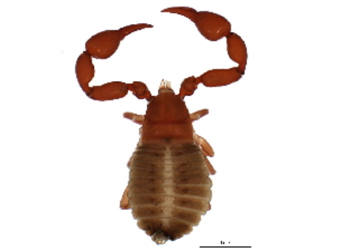

BIOUG00863-G06 [Lateral]
Chernetidae
Family: Chernetidae
BIN URI: BOLD:AAY6678

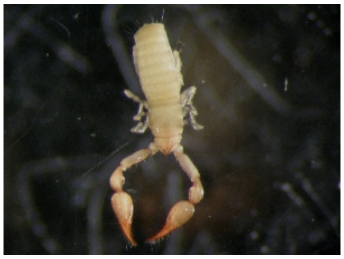

PSCA002 [Dorsal]
Microbisium
Family: Neobisiidae
BIN URI: BOLD:AAB2506

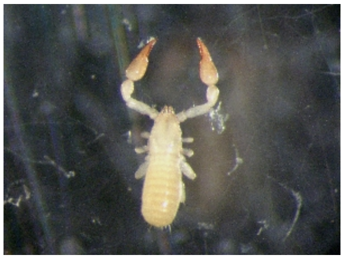

PSCA035 [Dorsal]
Microbisium
Family: Neobisiidae
BIN URI: BOLD:AAB2511

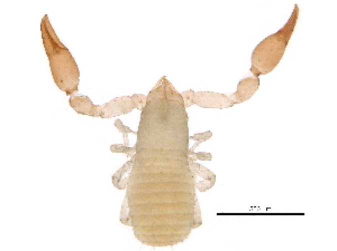

BIOUG21887-F10 [Dorsal]
Microbisium
Family: Neobisiidae
BIN URI: BOLD:AAB2508

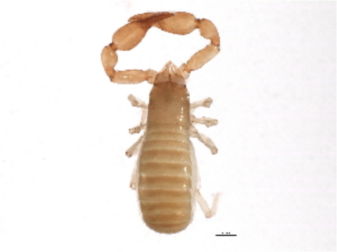

BIOUG02858-H11 [Dorsal]
Microbisium
Family: Neobisiidae
BIN URI: BOLD:AAY6677

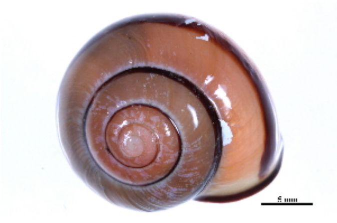

BIOUG15233-G03 [Apical]
Cepaea nemoralis
Family: Helicidae
BIN URI: BOLD:AAG1590

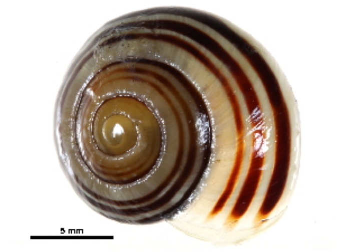

BIOUG15234-A01 [Apical]
Cepaea nemoralis
Family: Helicidae
BIN URI: BOLD:AAU0190

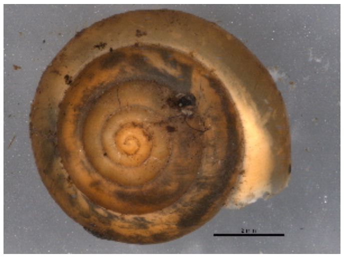

BIOUG09921-D04 [Dorsal]
Trochulus hispidus
Family: Hygromiidae
BIN URI: BOLD:ACI9420

IMAGE NOT AVAILABLE

BIOUG20546-H07
Trochulus
Family: Hygromiidae
BIN URI: BOLD:ACV4080

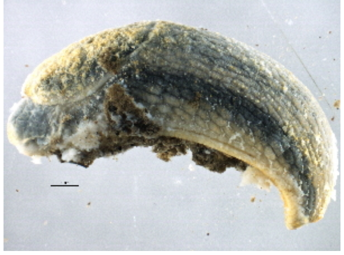

BIOUG07961-C06 [Lateral]
Arion distinctus
Family: Arionidae
BIN URI: BOLD:AAC0783

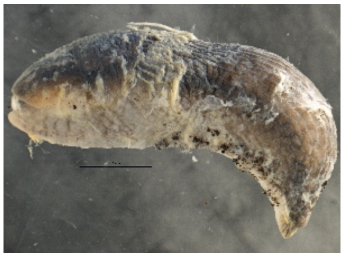

BIOUG08048-E09 [Dorsal]
Arion flagellus
Family: Arionidae
BIN URI: BOLD:AAC0334

IMAGE NOT AVAILABLE

BIOUG24036-E05
Arion subfuscus
Family: Arionidae

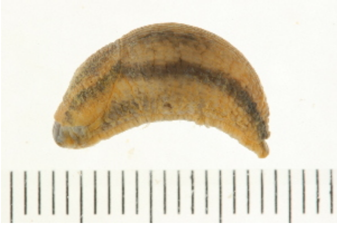

08BBMOL-0043 [Lateral]
Arion fuscus
Family: Arionidae
BIN URI: BOLD:AAC2849

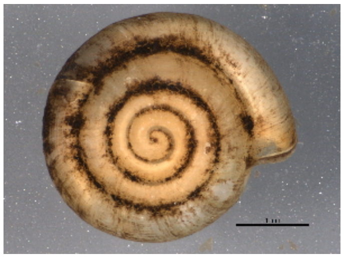

BIOUG09922-E02 [Dorsal]
Helicodiscus parallelus
Family: Helicodiscidae
BIN URI: BOLD:AAM7935

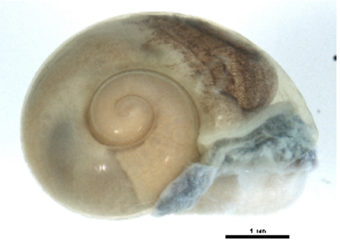

BIOUG15235-B07 [Apical]
Vitrina angelicae
Family: Vitrinidae
BIN URI: BOLD:AAN0223

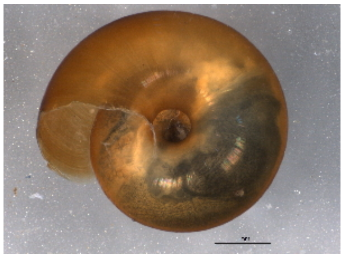

BIOUG09921-C12 [Ventral]
Zonitoides nitidus
Family: Gastrodontidae
BIN URI: BOLD:AAN3419

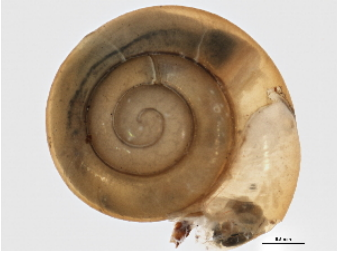

BIOUG02457-G06 [Lateral]
Zonitoides arboreus
Family: Gastrodontidae
BIN URI: BOLD:AAG1583

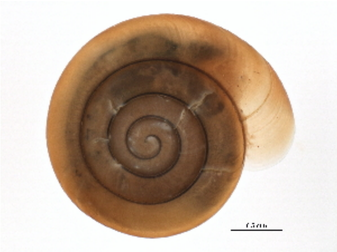

BIOUG13626-B01 [Apical]
Nesovitrea electrina
Family: Zonitidae
BIN URI: BOLD:ACO2467

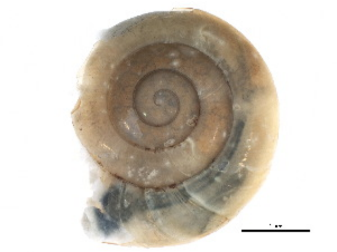

BIOUG14152-D04 [Apical]
Nesovitrea electrina
Family: Zonitidae
BIN URI: BOLD:ACP8138

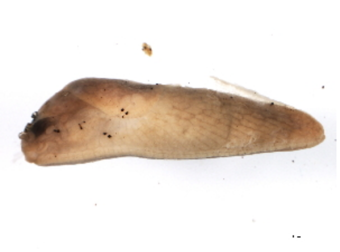

BIOUG00775-H03 [Lateral]
Deroceras reticulatum
Family: Agriolimacidae
BIN URI: BOLD:AAI9663

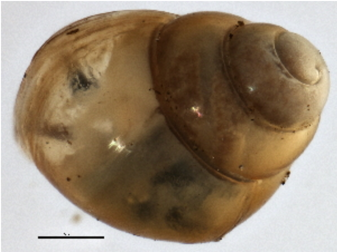

BIOUG07961-E03 [Dorsal]
Cochlicopa lubrica
Family: Cochlicopidae
BIN URI: BOLD:AAO2419

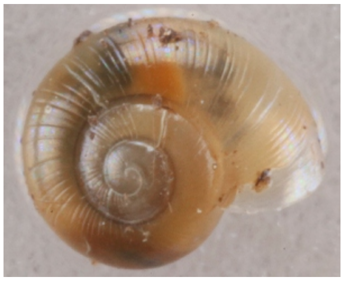

ZMUO.019943 [Dorsal]
Nesovitrea hammonis
Family: Zonitidae
BIN URI: BOLD:ABV9664

IMAGE NOT AVAILABLE

BIOUG24036-D07
Paravitrea multidentata
Family: Zonitidae

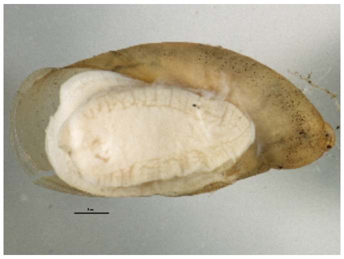

BIOUG07961-F08 [Ventral]
Succineidae
Family: Succineidae
BIN URI: BOLD:ACI9370

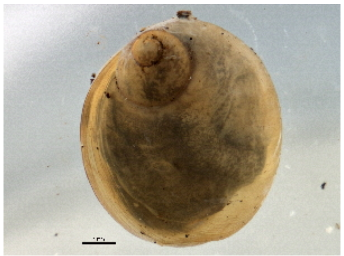

BIOUG07961-E09 [Dorsal]
Succinea putris
Family: Succineidae
BIN URI: BOLD:AAN9260

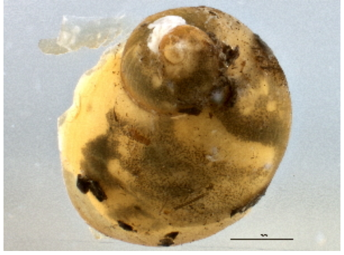

BIOUG07961-A04 [Dorsal]
Succinea putris
Family: Succineidae
BIN URI: BOLD:AAN9260

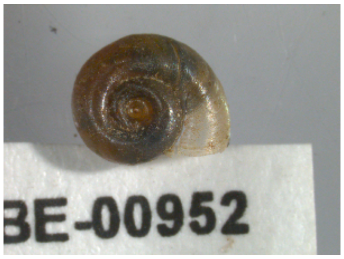

PPM265 [Dorsal]
Gyraulus circumstriatus
Family: Planorbidae
BIN URI: BOLD:AAG1567

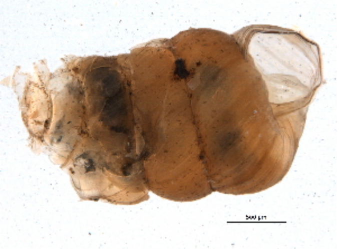

BIOUG03707-A12 [Lateral]
Columella simplex
Family: Vertiginidae
BIN URI: BOLD:AAO2405

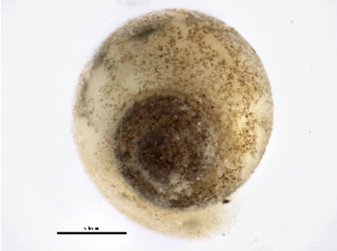

BIOUG07961-G08 [Dorsal]
Physa gyrina
Family: Physidae
BIN URI: BOLD:ACA7323

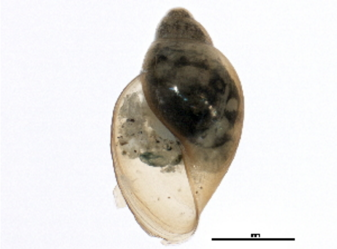

SCCWRP0039022 [Foot]
Physa heterostropha
Family: Physidae
BIN URI: BOLD:AAZ1627

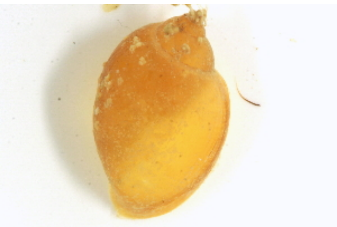

BIOUG24036-E11 [Abapertural]
Physa
Family: Physidae

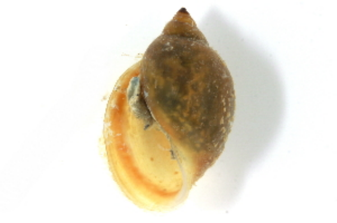

BIOUG24036-F03 [Apertural]
Physidae
Family: Physidae

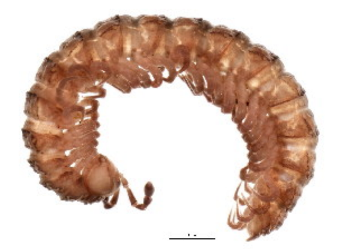

BIOUG00880-F10 [Lateral]
Polydesmidae
Family: Polydesmidae
BIN URI: BOLD:AAM7947

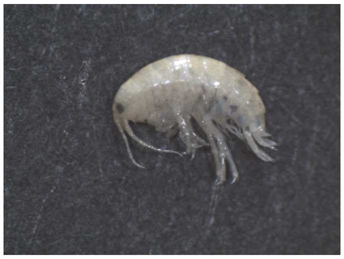

HOSO-LoWat05 [Lateral]
Hyalella azteca
Family: Hyalellidae
BIN URI: BOLD:AAA1707

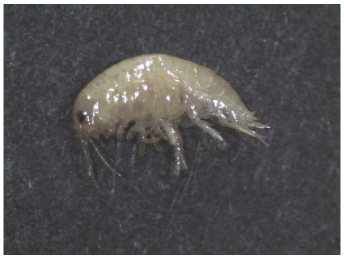

HOSO-LoWat04 [Lateral]
Hyalella azteca
Family: Hyalellidae
BIN URI: BOLD:AAA1704

IMAGE NOT AVAILABLE

BIOUG24014-F03
Hyalella azteca
Family: Hyalellidae

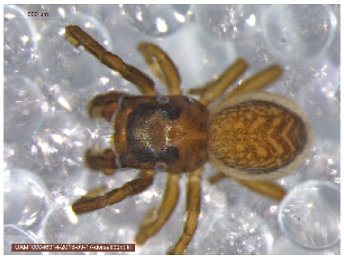

UAM:Ento:113456 [Dorsal]
Neon reticulatus
Family: Salticidae
BIN URI: BOLD:AAD9221

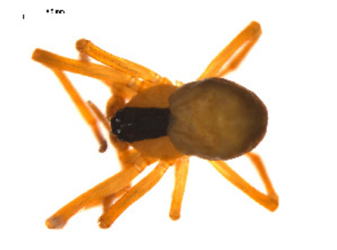

CCDB-05296-E12 [Dorsal]
Theridula emertoni
Family: Theridiidae
BIN URI: BOLD:AAD2291

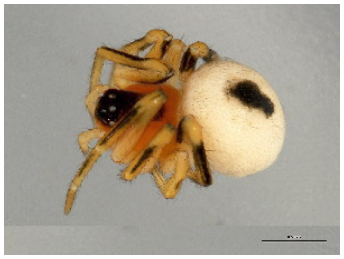

TDWG-0918 [Lateral]
Thymoites unimaculatus
Family: Theridiidae
BIN URI: BOLD:AAE7853

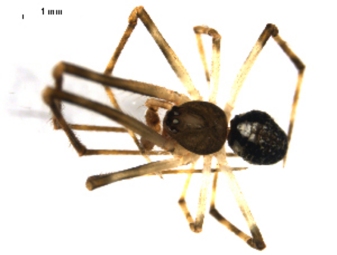

BIOUG00625-G07 [Dorsal]
Yunohamella lyrica
Family: Theridiidae
BIN URI: BOLD:AAG4815

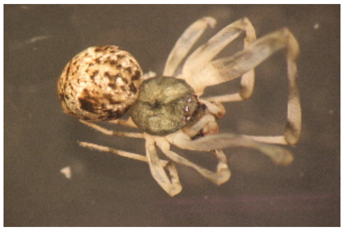

NZSPI208 [Dorsal]
Parasteatoda tepidariorum
Family: Theridiidae
BIN URI: BOLD:AAC0175

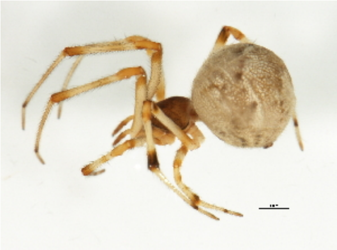

09ONTGAB-060 [Lateral]
Parasteatoda tabulata
Family: Theridiidae
BIN URI: BOLD:AAC3680

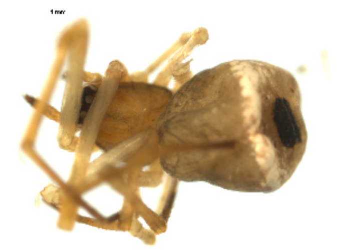

CCDB-05155-D04 [Dorsal]
Hentziectypus globosus
Family: Theridiidae
BIN URI: BOLD:AAN6263

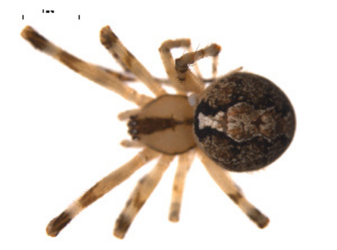

CCDB-05294-B10 [Dorsal]
Theridion murarium
Family: Theridiidae
BIN URI: BOLD:AAC6350

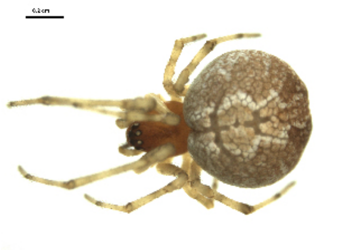

CCDB-05302-E04 [Dorsal]
Theridion differens
Family: Theridiidae
BIN URI: BOLD:AAC3269

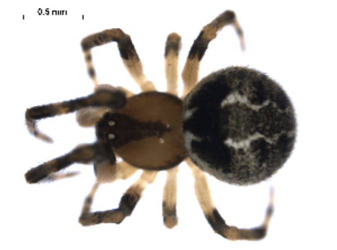

BIOUG00626-G04 [Dorsal]
Theridion glaucescens
Family: Theridiidae
BIN URI: BOLD:AAG1794

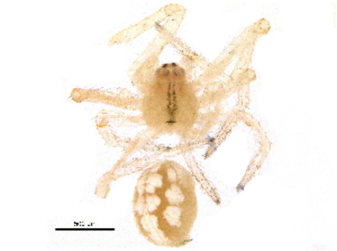

BIOUG22573-H10 [Dorsal]
Theridion albidum
Family: Theridiidae
BIN URI: BOLD:AAV3042

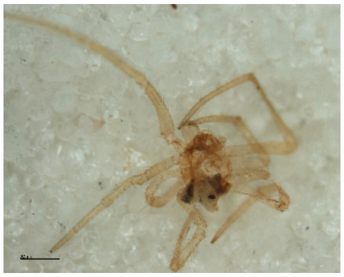

RMNH.ARA.14037 [Dorsal]
Neottiura bimaculata
Family: Theridiidae
BIN URI: BOLD:AAK8332

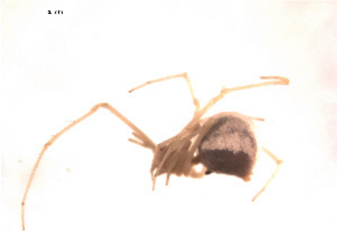

BIOUG00176-F02 [Lateral]
Neottiura bimaculata
Family: Theridiidae
BIN URI: BOLD:ACN7831

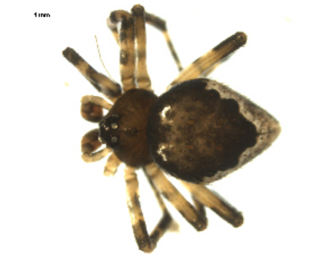

CCDB-05155-A12 [Dorsal]
Euryopis funebris
Family: Theridiidae
BIN URI: BOLD:AAJ0542

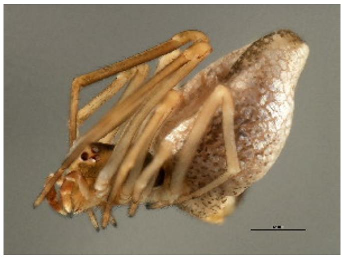

TDWG-0927 [Lateral]
Neospintharus trigonum
Family: Theridiidae
BIN URI: BOLD:AAB0273

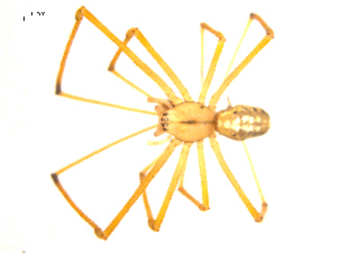

09ONTGAB-165 [Dorsal]
Enoplognatha ovata
Family: Theridiidae
BIN URI: BOLD:AAA6910

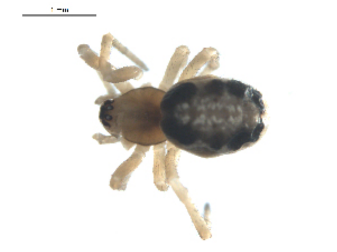

BIOUG00623-A08 [Dorsal]
Enoplognatha caricis
Family: Theridiidae
BIN URI: BOLD:AAO3896

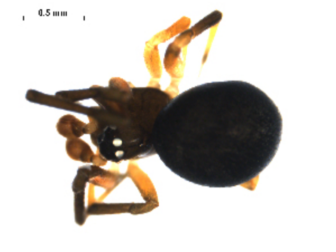

BIOUG00625-D12 [Dorsal]
Dipoena nigra
Family: Theridiidae
BIN URI: BOLD:AAF4974

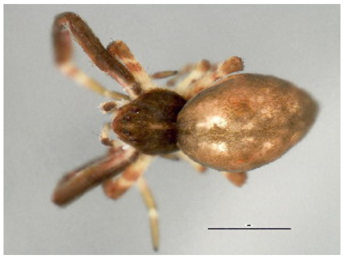

TDWG-0922 [Dorsal]
Uloborus glomosus
Family: Uloboridae
BIN URI: BOLD:AAJ7823

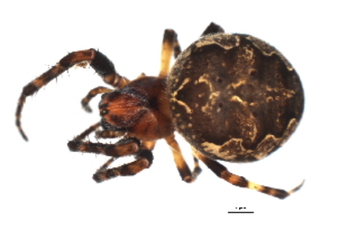

09PROBE-01583 [Dorsal]
Larinioides patagiatus
Family: Araneidae
BIN URI: BOLD:AAA3681

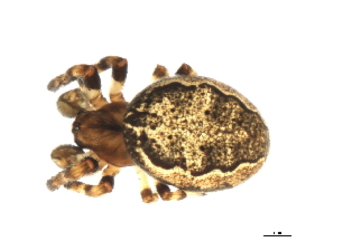

08SOAR-0035 [Dorsal]
Larinioides cornutus
Family: Araneidae
BIN URI: BOLD:AAA8999

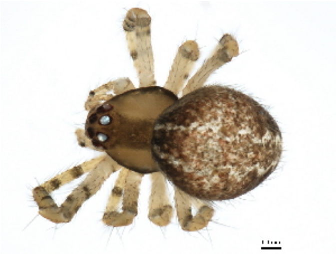

10PHMAL-2092 [Dorsal]
Eustala emertoni
Family: Araneidae
BIN URI: BOLD:AAB7934

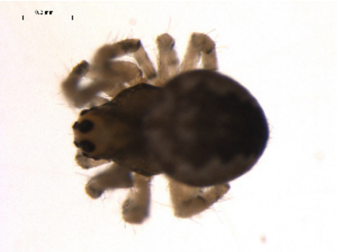

CCDB-05307-D05 [Dorsal]
Eustala cepina
Family: Araneidae
BIN URI: BOLD:AAB7935

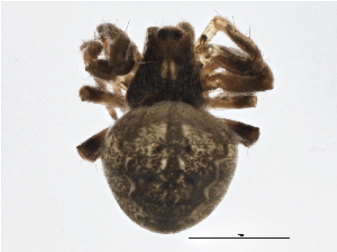

BIOUG05713-A12 [Dorsal]
Eustala anastera
Family: Araneidae
BIN URI: BOLD:AAB7933

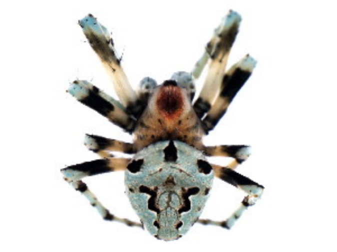

TDWG-0975 [Dorsal]
Eustala rosae
Family: Araneidae
BIN URI: BOLD:AAL4913

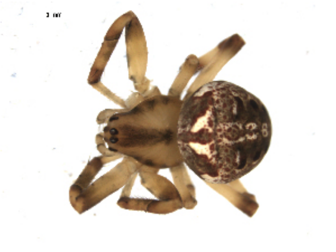

CCDB-08516-G04 [Dorsal]
Neoscona arabesca
Family: Araneidae
BIN URI: BOLD:AAA4123

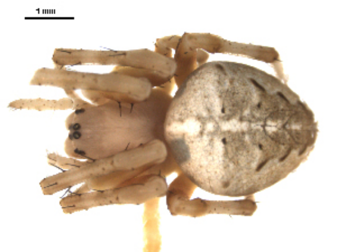

02-KADIR-G12 [Dorsal]
Araneus diadematus
Family: Araneidae
BIN URI: BOLD:AAA4125

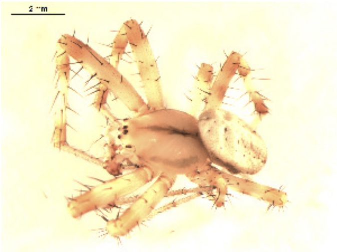

BIOUG00524-G04 [Dorsal]
Araneus trifolium
Family: Araneidae
BIN URI: BOLD:AAB8544

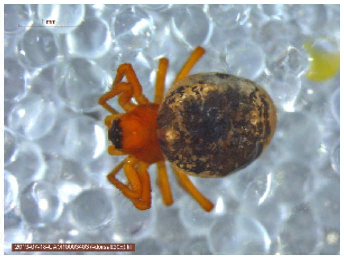

UAM:Ento:116820 [Dorsal]
Hypsosinga rubens
Family: Araneidae
BIN URI: BOLD:AAN6264

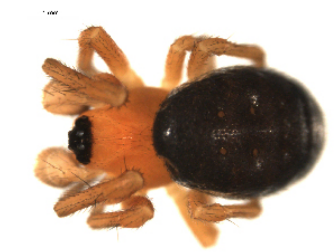

CCDB-04290-C03 [Dorsal]
Hypsosinga pygmaea
Family: Araneidae
BIN URI: BOLD:ABX6180

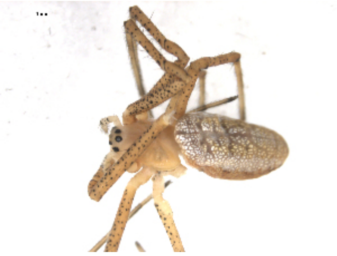

BIOUG00163-H05 [Dorsal]
Argiope trifasciata
Family: Araneidae
BIN URI: BOLD:AAC3165

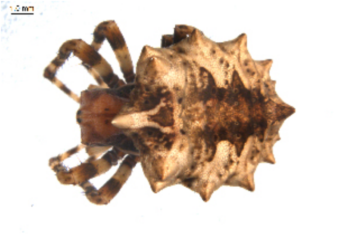

BIOUG00039-D02 [Dorsal]
Acanthepeira stellata
Family: Araneidae
BIN URI: BOLD:AAD7855

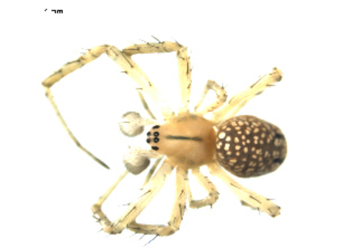

BIOUG00616-A10 [Dorsal]
Mangora gibberosa
Family: Araneidae
BIN URI: BOLD:AAB7330

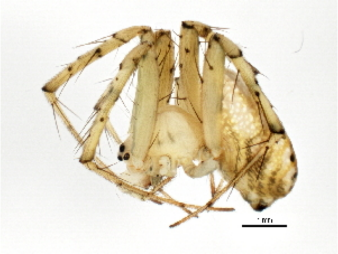

BIOUG01966-A07 [Lateral]
Mangora maculata
Family: Araneidae
BIN URI: BOLD:AAK7682

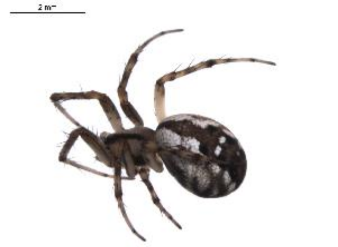

BIOUG00886-B11 [Dorsal]
Mangora placida
Family: Araneidae
BIN URI: BOLD:ACE4103

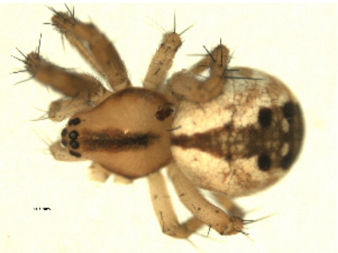

CCDB-04359-G06 [Lateral]
Mangora placida
Family: Araneidae
BIN URI: BOLD:AAI4456

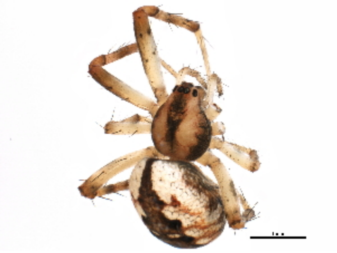

BIOUG12203-B06 [Dorsal]
Mangora placida
Family: Araneidae
BIN URI: BOLD:ACJ0225

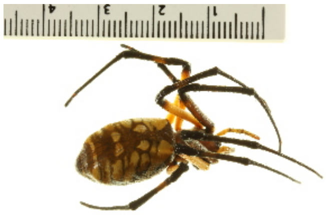

BIOUG01961-H11 [Dorsal]
Argiope aurantia
Family: Araneidae
BIN URI: BOLD:AAD2601

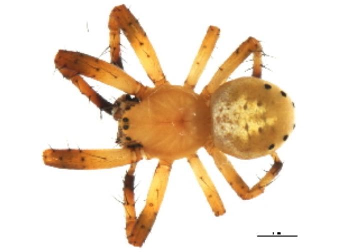

08SOAR-0103 [Dorsal]
Araniella displicata
Family: Araneidae
BIN URI: BOLD:AAA8399

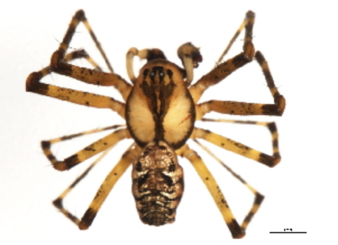

09-PROBE-08152 [Dorsal]
Pityohyphantes subarcticus
Family: Linyphiidae
BIN URI: BOLD:AAA4185

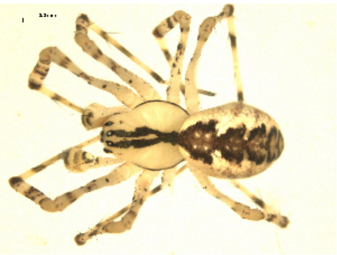

CCDB-04359-B07 [Lateral]
Pityohyphantes costatus
Family: Linyphiidae
BIN URI: BOLD:AAC6457

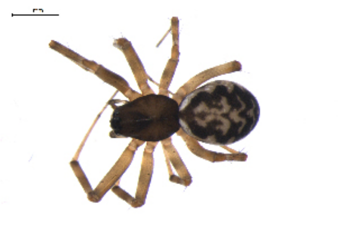

BIOUG00889-C01 [Dorsal]
Neriene clathrata
Family: Linyphiidae
BIN URI: BOLD:AAA8358

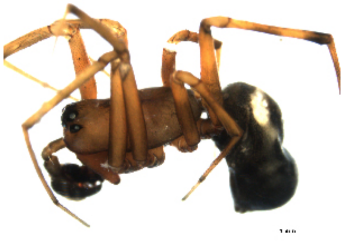

CCDB-05259-F06 [Lateral]
Neriene clathrata
Family: Linyphiidae
BIN URI: BOLD:AAB7327

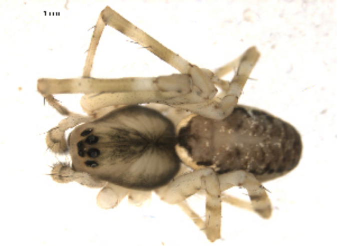

BIOUG00040-C05 [Dorsal]
Neriene montana
Family: Linyphiidae
BIN URI: BOLD:AAB7328

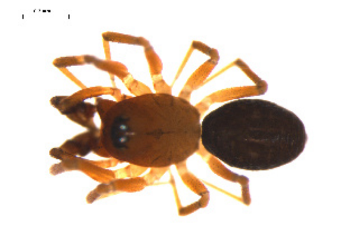

CCDB-05303-C03 [Dorsal]
Erigone autumnalis
Family: Linyphiidae
BIN URI: BOLD:AAH0001

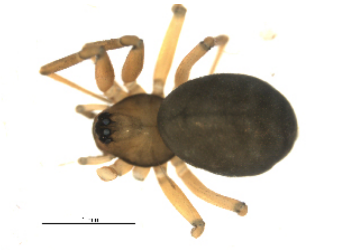

CCDB-05149-A07 [Dorsal]
Tenuiphantes zebra
Family: Linyphiidae
BIN URI: BOLD:AAI8098

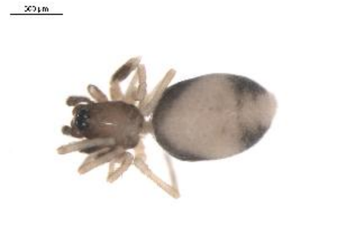

BIOUG00886-E04 [Dorsal]
Tennesseellum formica
Family: Linyphiidae
BIN URI: BOLD:AAG5631

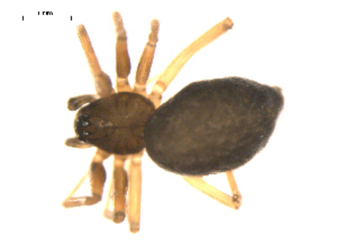

09ONTGAB-144 [Dorsal]
Agyneta unimaculata
Family: Linyphiidae
BIN URI: BOLD:AAH0003

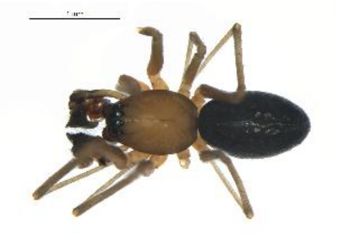

BIOUG00632-B12 [Dorsal]
Agyneta fabra
Family: Linyphiidae
BIN URI: BOLD:AAE3860

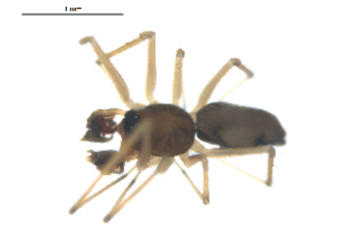

BIOUG00623-E06 [Dorsal]
Agyneta micaria
Family: Linyphiidae
BIN URI: BOLD:AAN6265

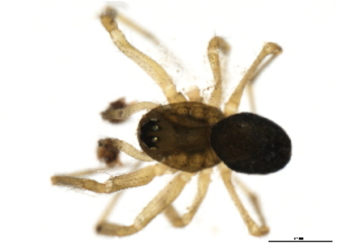

08EARGUE-0009 [Dorsal]
Mermessus trilobatus
Family: Linyphiidae
BIN URI: BOLD:AAC8898

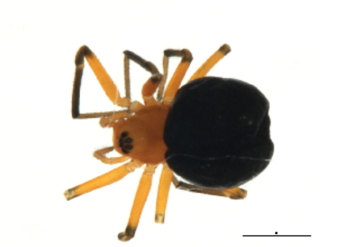

08SOAR-0082 [Dorsal]
Hypselistes florens
Family: Linyphiidae
BIN URI: BOLD:AAB4233

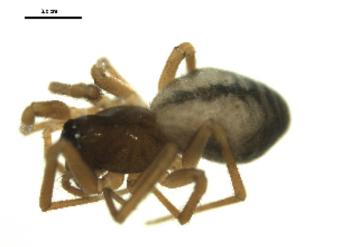

CCDB-05302-F03 [Dorsal]
Grammonota angusta
Family: Linyphiidae
BIN URI: BOLD:AAD1498

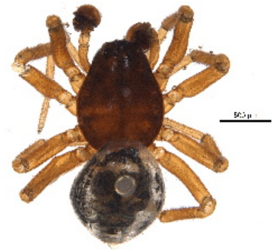

BIOUG20599-A04 [Dorsal]
Grammonota inornata
Family: Linyphiidae
BIN URI: BOLD:ACV5737

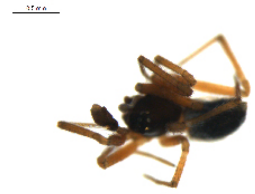

CCDB-21418-B11 [Dorsal]
Collinsia plumosa
Family: Linyphiidae
BIN URI: BOLD:AAM9146

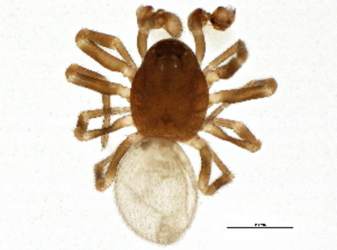

BIOUG01654-A09 [Dorsal]
Ceratinops latus
Family: Linyphiidae
BIN URI: BOLD:AAI5447

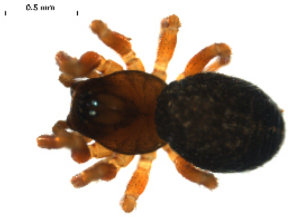

BIOUG00616-F11 [Dorsal]
Eridantes erigonoides
Family: Linyphiidae
BIN URI: BOLD:AAH0004

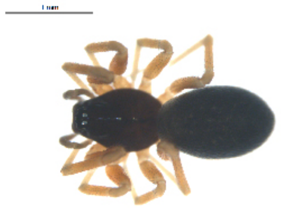

BIOUG00622-H05 [Dorsal]
Walckenaeria directa
Family: Linyphiidae
BIN URI: BOLD:AAH8313

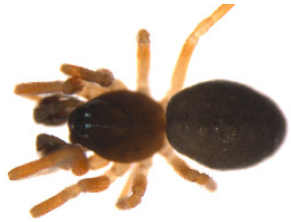

CCDB-05303-B12 [Dorsal]
Walckenaeria fallax
Family: Linyphiidae
BIN URI: BOLD:AAH8314

IMAGE NOT AVAILABLE

BIOUG20595-C03
Walckenaeria spiralis
Family: Linyphiidae
BIN URI: BOLD:AAH8314

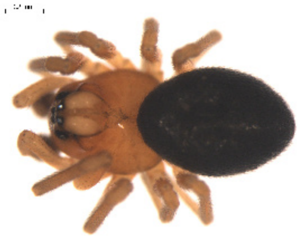

09PROBE-932 [Dorsal]
Hypomma marxi
Family: Linyphiidae
BIN URI: BOLD:AAB9520

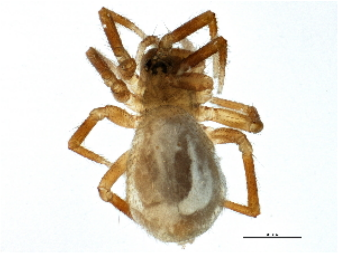

BIOUG06316-H10 [Dorsal]
Pocadicnemis americana
Family: Linyphiidae
BIN URI: BOLD:AAC9060

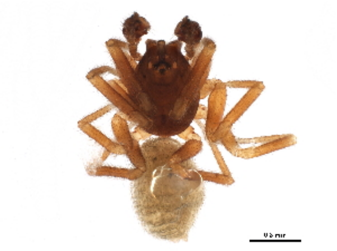

BIOUG18633-F09 [Dorsal]
Walckenaeria pinocchio
Family: Linyphiidae
BIN URI: BOLD:ACT1115

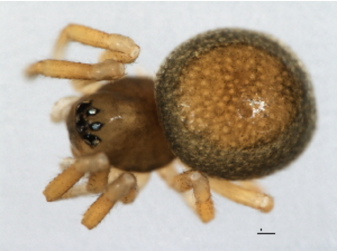

CCDB-04552-E04 [Dorsal]
Ceratinella brunnea
Family: Linyphiidae
BIN URI: BOLD:AAD2101

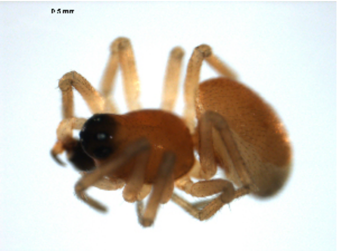

BIOUG00616-A07 [Dorsal]
Ceraticelus similis
Family: Linyphiidae
BIN URI: BOLD:AAF1318

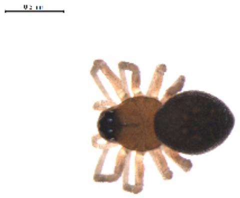

BIOUG00635-B04 [Dorsal]
Ceraticelus atriceps
Family: Linyphiidae
BIN URI: BOLD:AAI3701

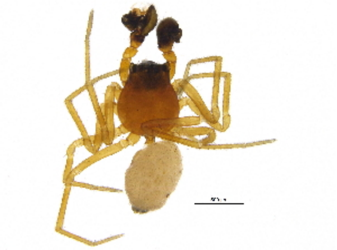

BIOUG17140-B08 [Dorsal]
Ceratinopsis auriculata
Family: Linyphiidae
BIN URI: BOLD:ACR6338

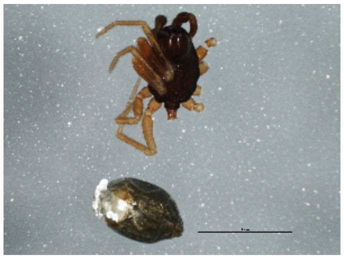

BIOUG05512-B09 [Dorsal]
Ceratinops crenatus
Family: Linyphiidae
BIN URI: BOLD:ACF8798

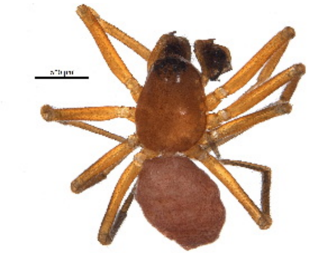

BIOUG20597-D10 [Dorsal]
Ceratinopsis labradorensis
Family: Linyphiidae
BIN URI: BOLD:ACV5182

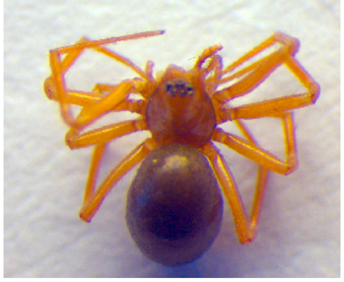

BC ZSM ARA 00186 [Dorsal]
Centromerus sylvaticus
Family: Linyphiidae
BIN URI: BOLD:AAA4132

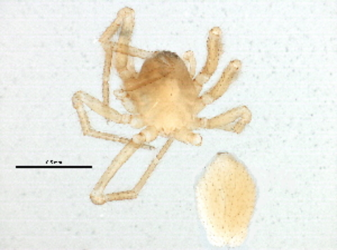

BIOUG10076-G09 [Dorsal]
Mermessus index
Family: Linyphiidae
BIN URI: BOLD:ACL4554

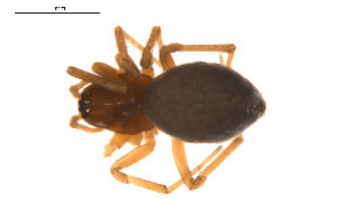

BIOUG14290-B04 [Dorsal]
Erigone blaesa
Family: Linyphiidae
BIN URI: BOLD:ACE9601

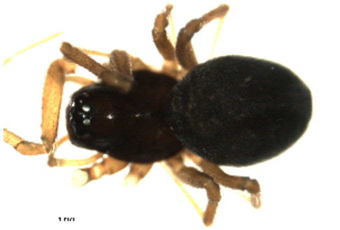

CCDB-05259-G11 [Dorsal]
Erigone atra
Family: Linyphiidae
BIN URI: BOLD:ACE5877

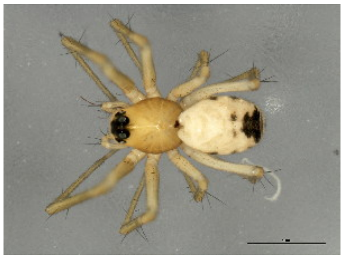

TDWG-0900 [Dorsal]
Neriene variabilis
Family: Linyphiidae
BIN URI: BOLD:AAE5990

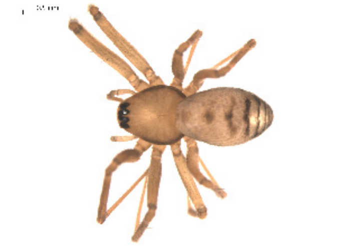

09PROBE-873 [Dorsal]
Bathyphantes brevis
Family: Linyphiidae
BIN URI: BOLD:AAC5851

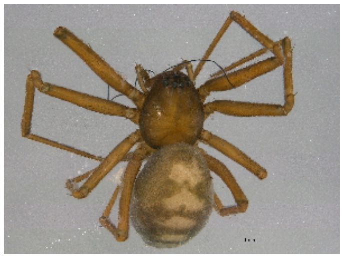

BIOUG06190-C12 [Dorsal]
Bathyphantes pallidus
Family: Linyphiidae
BIN URI: BOLD:AAC9112

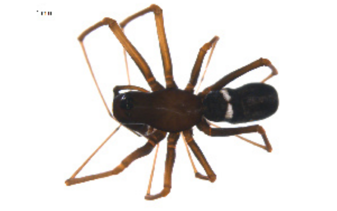

BIOUG09848-G11 [Dorsal]
Microlinyphia mandibulata
Family: Linyphiidae
BIN URI: BOLD:AAF4994

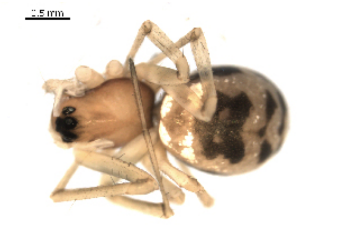

BIOUG00519-E01 [Dorsal]
Microlinyphia mandibulata
Family: Linyphiidae
BIN URI: BOLD:AAG9557

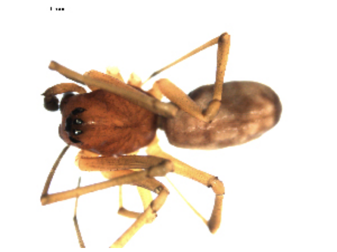

CCDB-05292-C03 [Dorsal]
Frontinella communis
Family: Linyphiidae
BIN URI: BOLD:AAE0825

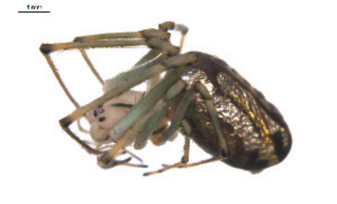

BIOUG12606-C03 [Dorsal]
Leucauge venusta
Family: Tetragnathidae
BIN URI: BOLD:AAB8714

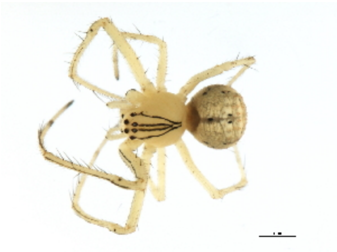

08BBARAC-0006 [Dorsal]
Mimetus epeiroides
Family: Mimetidae
BIN URI: BOLD:AAG5658

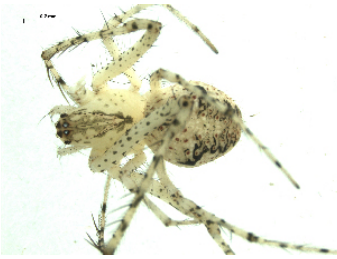

CCDB-04289-A12 [Dorsal]
Mimetus notius
Family: Mimetidae
BIN URI: BOLD:AAE0114

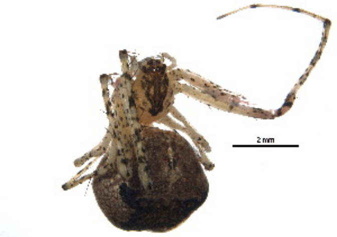

BIOUG20596-A02 [Dorsal]
Mimetus haynesi
Family: Mimetidae
BIN URI: BOLD:AAK6284

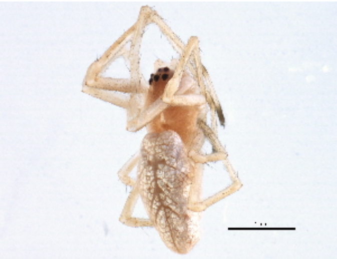

BIOUG08040-F06 [Dorsal]
Tetragnatha shoshone
Family: Tetragnathidae
BIN URI: BOLD:ACN2094

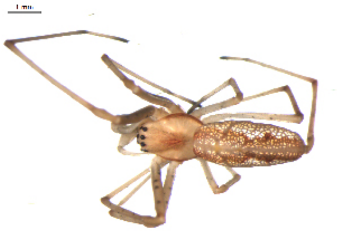

BIOUG00521-F06 [Dorsal]
Tetragnatha shoshone
Family: Tetragnathidae
BIN URI: BOLD:ACN2276

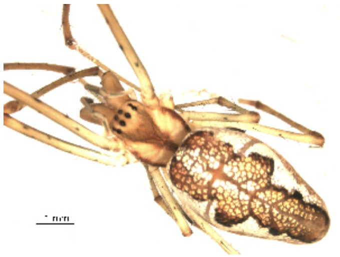

BIOUG00517-A03 [Dorsal]
Tetragnatha shoshone
Family: Tetragnathidae
BIN URI: BOLD:ACN2438

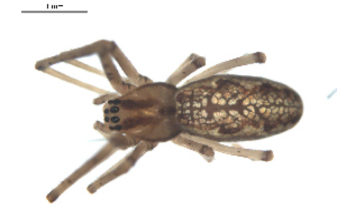

BIOUG00622-G04 [Dorsal]
Tetragnatha versicolor
Family: Tetragnathidae
BIN URI: BOLD:AAB7995

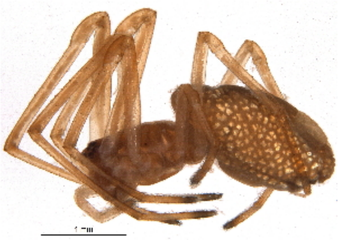

BIOUG16052-H01 [Lateral]
Tetragnatha shoshone
Family: Tetragnathidae
BIN URI: BOLD:ACP7708

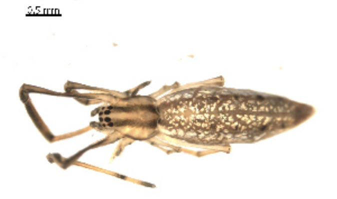

BIOUG00519-D08 [Dorsal]
Tetragnatha caudata
Family: Tetragnathidae
BIN URI: BOLD:AAP3715

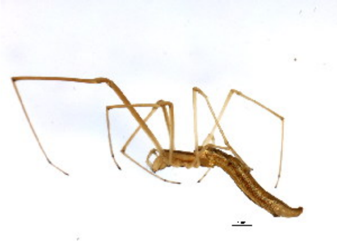

08BBARAC-0377 [Lateral]
Tetragnatha caudata
Family: Tetragnathidae
BIN URI: BOLD:AAE3958

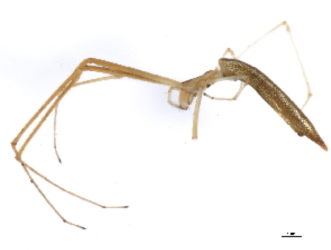

08BBARAC-0105 [Lateral]
Tetragnatha caudata
Family: Tetragnathidae
BIN URI: BOLD:ACN4034

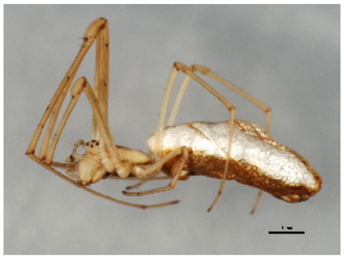

CCDB-04551-F10 [Lateral]
Tetragnatha laboriosa
Family: Tetragnathidae
BIN URI: BOLD:AAA6383

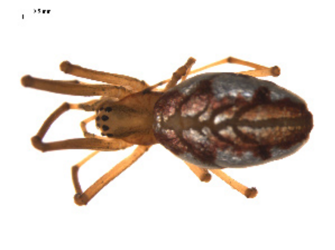

CCDB-05296-H04 [Dorsal]
Tetragnatha laboriosa
Family: Tetragnathidae
BIN URI: BOLD:AAA6381

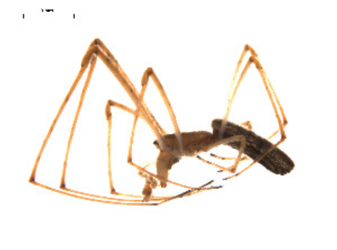

CCDB-05305-H11 [Lateral]
Tetragnatha laboriosa
Family: Tetragnathidae
BIN URI: BOLD:ACR6860

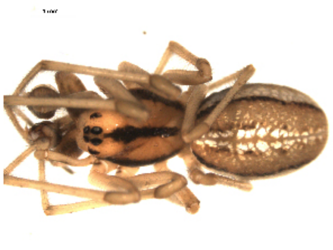

CCDB-04290-D09 [Dorsal]
Pachygnatha tristriata
Family: Tetragnathidae
BIN URI: BOLD:AAF1571

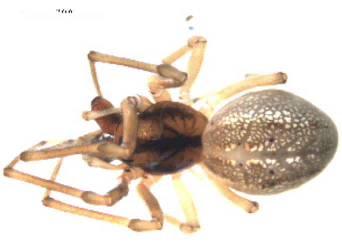

CCDB-04290-H10 [Dorsal]
Pachygnatha dorothea
Family: Tetragnathidae
BIN URI: BOLD:AAE5304

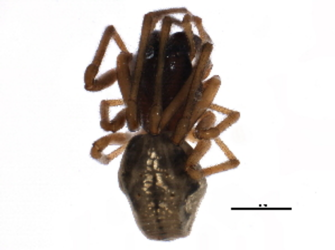

BIOUG14249-H01 [Dorsal]
Pachygnatha xanthostoma
Family: Tetragnathidae
BIN URI: BOLD:ACO7247

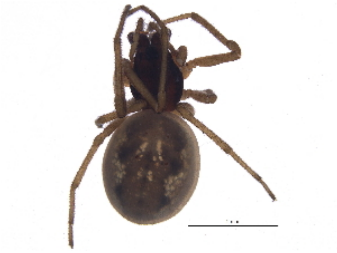

BIOUG13984-H01 [Dorsal]
Pachygnatha xanthostoma
Family: Tetragnathidae
BIN URI: BOLD:ACP5884

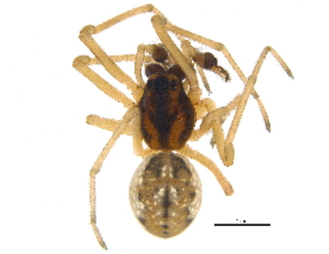

BIOUG20041-D11 [Dorsal]
Pachygnatha xanthostoma
Family: Tetragnathidae
BIN URI: BOLD:ACU5364

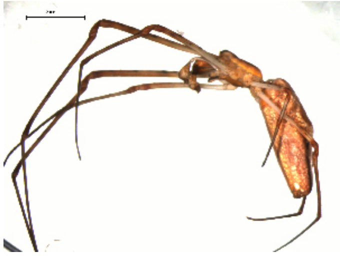

01-BB09US-D08 [Lateral]
Tetragnatha guatemalensis
Family: Tetragnathidae
BIN URI: BOLD:AAC6596

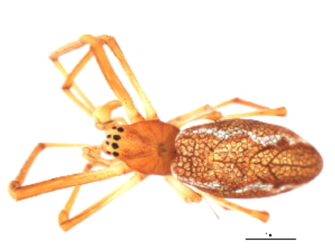

10PROBE-21174 [Dorsal]
Tetragnatha extensa
Family: Tetragnathidae
BIN URI: BOLD:AAA4940

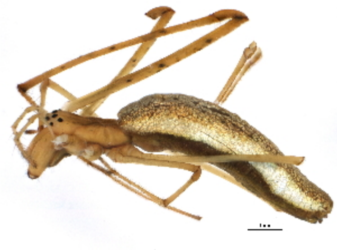

BIOUG05803-G12 [Lateral]
Tetragnatha straminea
Family: Tetragnathidae
BIN URI: BOLD:AAD7095

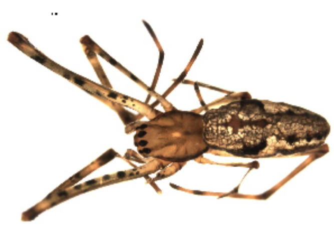

CCDB-05292-A01 [Dorsal]
Tetragnatha viridis
Family: Tetragnathidae
BIN URI: BOLD:AAG5659

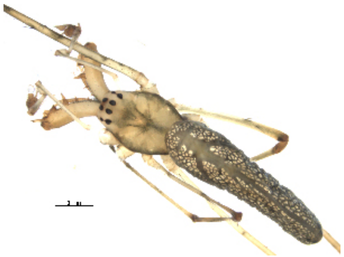

CCDB-08519-B02 [Dorsal]
Tetragnatha viridis
Family: Tetragnathidae
BIN URI: BOLD:AAN6335

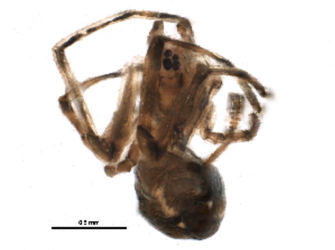

BIOUG12563-C10 [Dorsal]
Tetragnatha viridis
Family: Tetragnathidae
BIN URI: BOLD:ACN5503

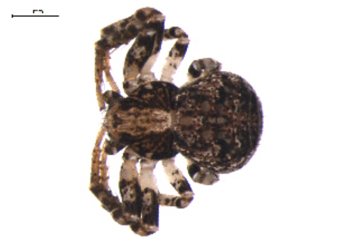

BIOUG00889-H07 [Dorsal]
Ozyptila praticola
Family: Thomisidae
BIN URI: BOLD:AAC7413

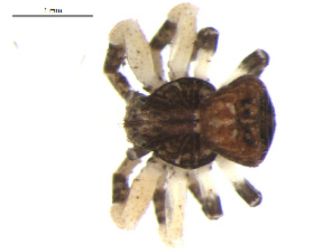

BIOUG00619-A07 [Dorsal]
Ozyptila americana
Family: Thomisidae
BIN URI: BOLD:AAV1641

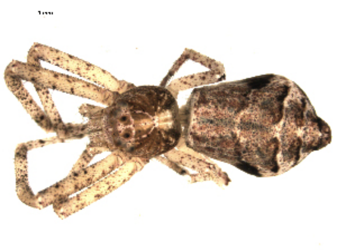

CCDB-05292-A04 [Dorsal]
Tmarus angulatus
Family: Thomisidae
BIN URI: BOLD:ABY7475

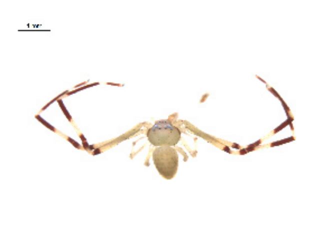

BIOUG12606-B08 [Dorsal]
Misumessus oblongus
Family: Thomisidae
BIN URI: BOLD:AAA8847

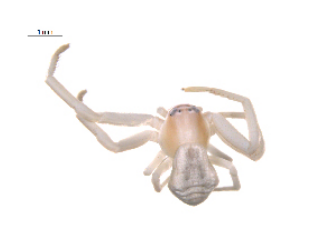

BIOUG12606-G08 [Dorsal]
Misumenoides formosipes
Family: Thomisidae
BIN URI: BOLD:AAE9994

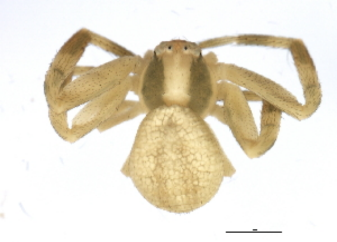

08BBARAC-0322 [Dorsal]
Misumena vatia
Family: Thomisidae
BIN URI: BOLD:AAA6275

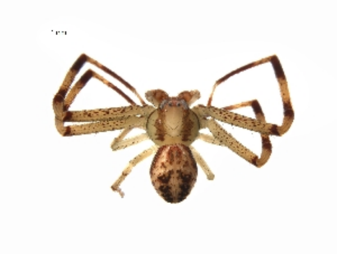

BIOUG12604-E02 [Dorsal]
Mecaphesa asperata
Family: Thomisidae
BIN URI: BOLD:ACE7683

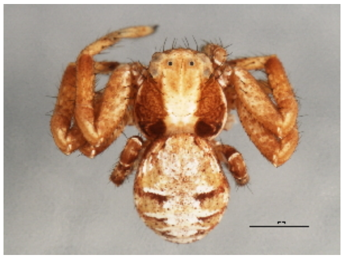

CCDB-04551-C01 [Dorsal]
Xysticus punctatus
Family: Thomisidae
BIN URI: BOLD:AAD2346

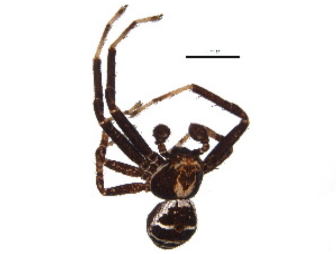

BIOUG22090-H01 [Dorsal]
Xysticus winnipegensis
Family: Thomisidae
BIN URI: BOLD:AAM6956

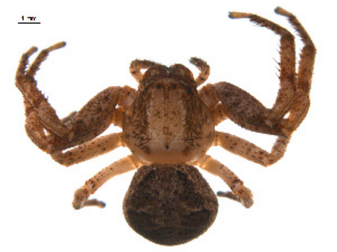

BIOUG12605-A11 [Dorsal]
Xysticus elegans
Family: Thomisidae
BIN URI: BOLD:AAC1568

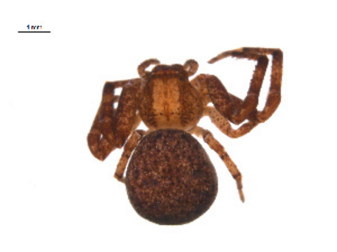

BIOUG12606-A06 [Dorsal]
Xysticus funestus
Family: Thomisidae
BIN URI: BOLD:AAC1600

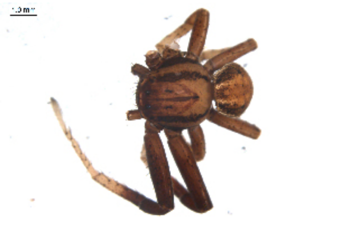

BIOUG00039-D01 [Dorsal]
Xysticus luctans
Family: Thomisidae
BIN URI: BOLD:AAF8190

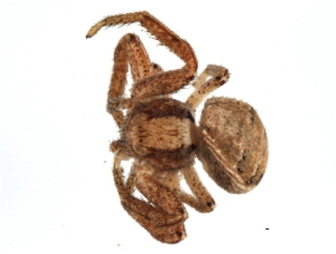

08EARGUE-0052 [Dorsal]
Xysticus emertoni
Family: Thomisidae
BIN URI: BOLD:AAB4300

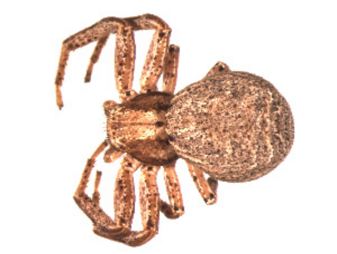

BIOUG00162-D01 [Dorsal]
Xysticus bicuspis
Family: Thomisidae
BIN URI: BOLD:AAJ9685

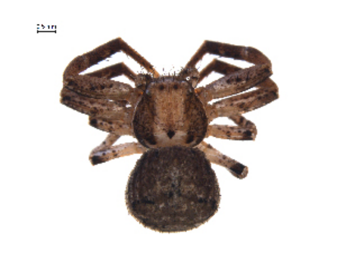

BIOUG07169-A04 [Dorsal]
Xysticus discursans
Family: Thomisidae
BIN URI: BOLD:AAJ9718

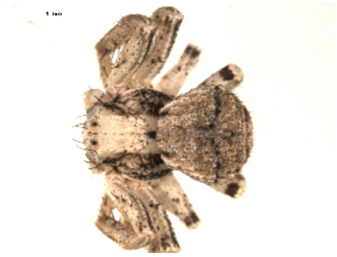

CCDB-04290-C11 [Dorsal]
Xysticus discursans
Family: Thomisidae
BIN URI: BOLD:ACV2014

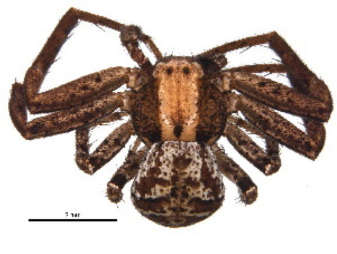

BIOUG20598-B09 [Dorsal]
Xysticus discursans
Family: Thomisidae
BIN URI: BOLD:ACV5078

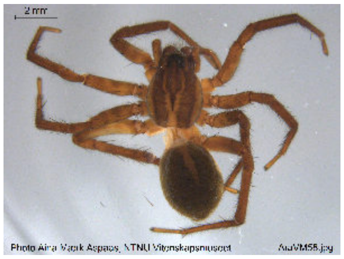

AraVM58 [Dorsal]
Pirata piraticus
Family: Lycosidae
BIN URI: BOLD:AAB6784

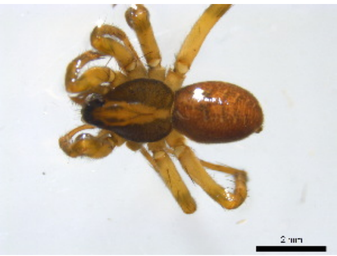

BIOUG09324-G11 [Dorsal]
Pirata praedo
Family: Lycosidae
BIN URI: BOLD:AAC5349

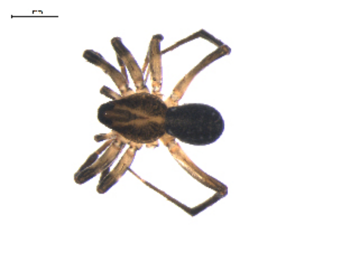

BIOUG00889-E03 [Dorsal]
Piratula minuta
Family: Lycosidae
BIN URI: BOLD:AAE4247

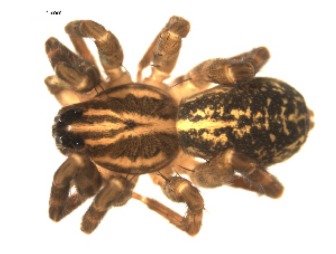

CCDB-04290-D11 [Dorsal]
Piratula cantralli
Family: Lycosidae
BIN URI: BOLD:ABZ5613

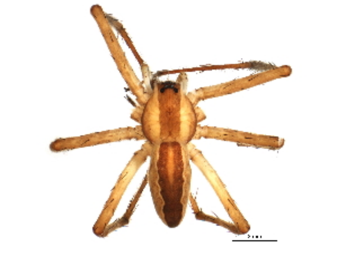

BIOUG14847-H09 [Dorsal]
Pisaurina mira
Family: Pisauridae
BIN URI: BOLD:AAI2721

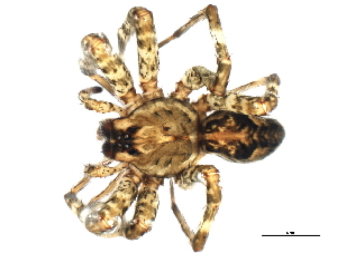

08OEC-020 [Dorsal]
Dolomedes tenebrosus
Family: Pisauridae
BIN URI: BOLD:AAC7517

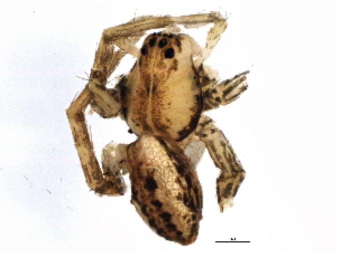

BIOUG06212-G12 [Dorsal]
Dolomedes vittatus
Family: Pisauridae
BIN URI: BOLD:ACI5773

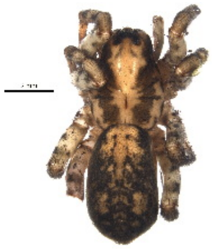

BIOUG20596-B07 [Dorsal]
Arctosa emertoni
Family: Lycosidae
BIN URI: BOLD:ACW1682

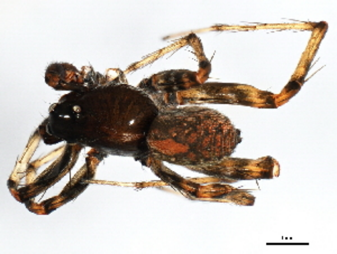

10PHMAL-1973 [Dorsal]
Pardosa moesta
Family: Lycosidae
BIN URI: BOLD:AAB0863

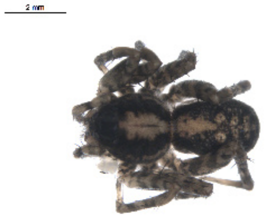

BIOUG00886-A08 [Dorsal]
Pardosa milvina
Family: Lycosidae
BIN URI: BOLD:AAB7668

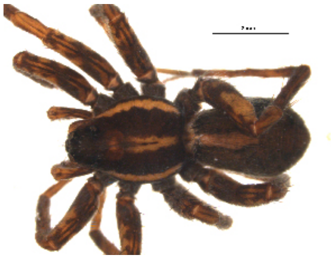

01WOLF-H05 [Dorsal]
Pardosa furcifera
Family: Lycosidae
BIN URI: BOLD:AAA5090

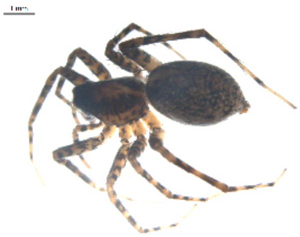

BIOUG00617-D02 [Dorsal]
Pardosa saxatilis
Family: Lycosidae
BIN URI: BOLD:AAB7667

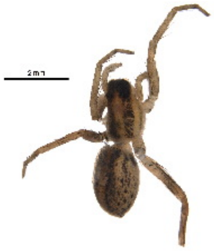

BIOUG22090-H03 [Dorsal]
Pardosa distincta
Family: Lycosidae
BIN URI: BOLD:AAC7802

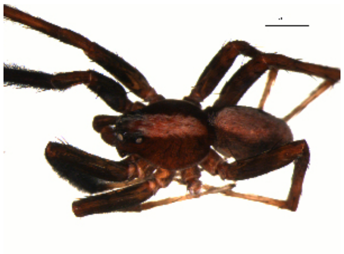

01-BB09US-A06 [Dorsal]
Schizocosa ocreata
Family: Lycosidae
BIN URI: BOLD:AAA7232

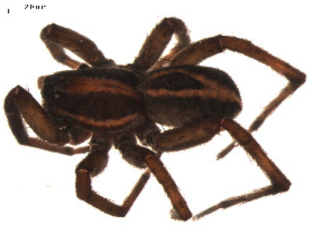

09ONTGAB-141 [Dorsal]
Schizocosa avida
Family: Lycosidae
BIN URI: BOLD:AAD3880

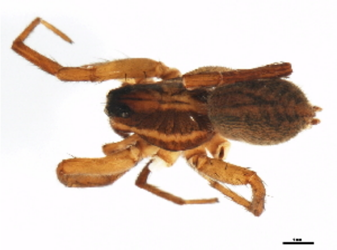

09EARGUE-0162 [Dorsal]
Schizocosa crassipalpata
Family: Lycosidae
BIN URI: BOLD:AAC4687

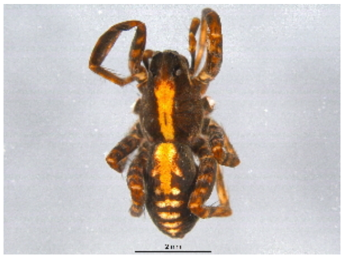

BIOUG05537-B06 [Lateral]
Schizocosa mccooki
Family: Lycosidae
BIN URI: BOLD:AAH0055

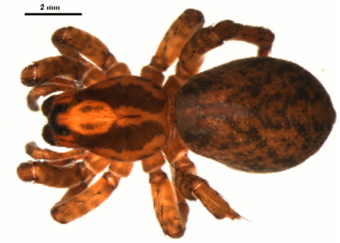

BGSPI-03-H07 [Dorsal]
Trochosa terricola
Family: Lycosidae
BIN URI: BOLD:AAB0727

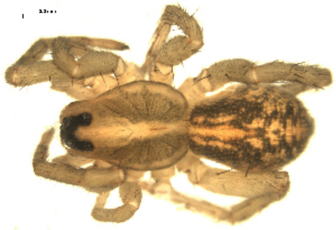

CCDB-04359-A03 [Lateral]
Trochosa ruricola
Family: Lycosidae
BIN URI: BOLD:AAB0726

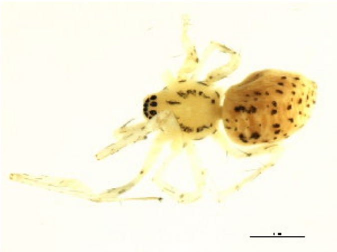

08BBARAC-0013 [Dorsal]
Wulfila saltabundus
Family: Anyphaenidae
BIN URI: BOLD:AAC6924

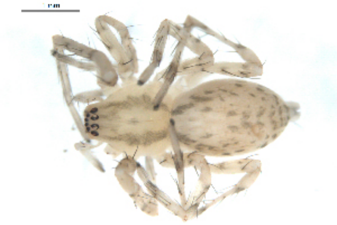

BIOUG00622-C09 [Dorsal]
Anyphaena pectorosa
Family: Anyphaenidae
BIN URI: BOLD:AAD6926

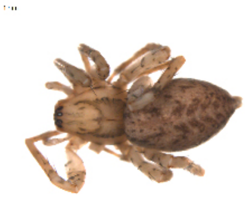

BIOUG09848-E10 [Dorsal]
Anyphaena celer
Family: Anyphaenidae
BIN URI: BOLD:AAK1749

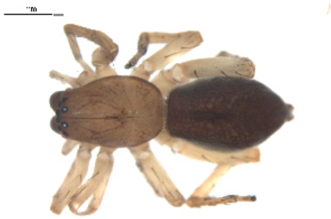

BIOUG00627-H11 [Dorsal]
Clubiona bryantae
Family: Clubionidae
BIN URI: BOLD:AAG5664

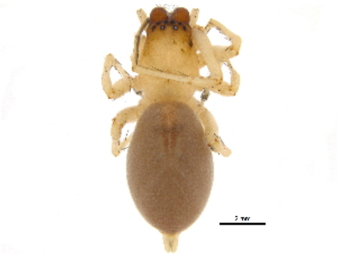

BIOUG22567-H10 [Dorsal]
Clubiona obesa
Family: Clubionidae
BIN URI: BOLD:AAD5417

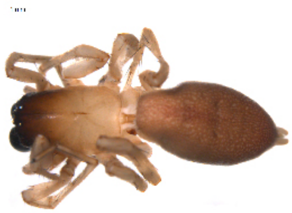

BIOUG09848-F08 [Dorsal]
Clubiona maritima
Family: Clubionidae
BIN URI: BOLD:AAI4085

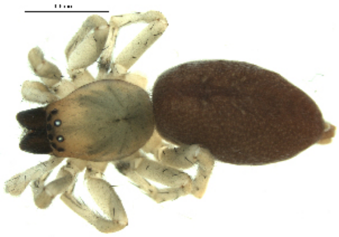

01ARONT-H08 [Dorsal]
Clubiona pallidula
Family: Clubionidae
BIN URI: BOLD:AAI4087

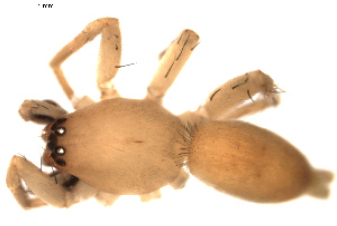

CCDB-05156-G08 [Dorsal]
Clubiona johnsoni
Family: Clubionidae
BIN URI: BOLD:AAN4847

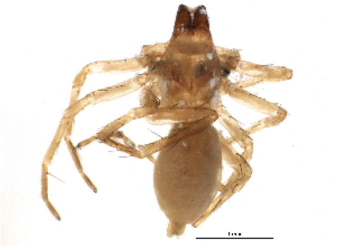

BIOUG22358-D01 [Dorsal]
Clubiona abboti
Family: Clubionidae
BIN URI: BOLD:AAD1564

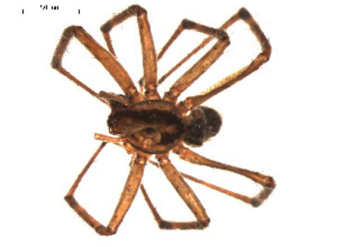

09ONTGAB-132 [Dorsal]
Agelenopsis potteri
Family: Agelenidae
BIN URI: BOLD:AAB5726

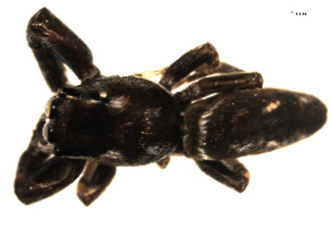

CCDB-05292-E04 [Dorsal]
Marpissa formosa
Family: Salticidae
BIN URI: BOLD:AAG0312

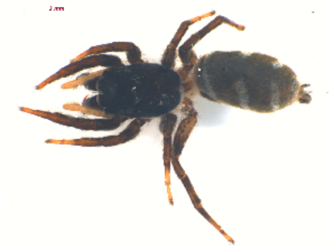

10-SKBC-0673 [Dorsal]
Salticus scenicus
Family: Salticidae
BIN URI: BOLD:AAC9044

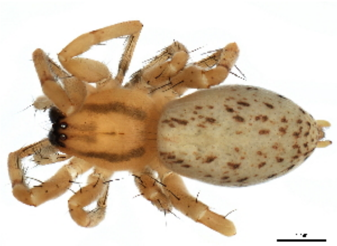

10PHMAL-2088 [Dorsal]
Hibana gracilis
Family: Anyphaenidae
BIN URI: BOLD:AAN6394

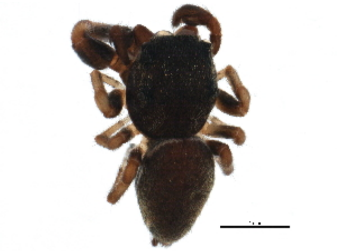

BIOUG12604-G11 [Dorsal]
Zygoballus nervosus
Family: Salticidae
BIN URI: BOLD:ACA1490

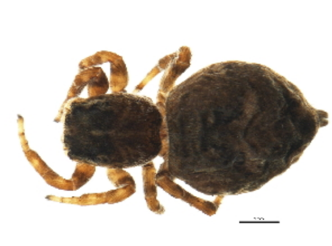

09ONTGAB-073 [Dorsal]
Sitticus floricola palustris
Family: Salticidae
BIN URI: BOLD:AAE1303

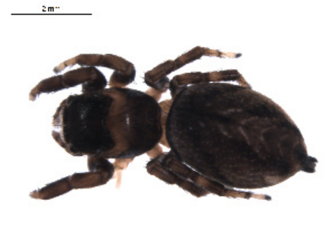

BIOUG00886-A09 [Dorsal]
Evarcha hoyi
Family: Salticidae
BIN URI: BOLD:AAC0342

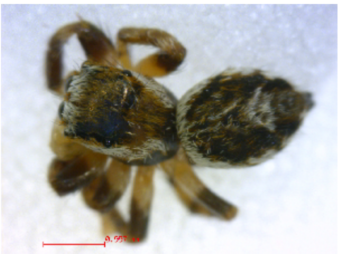

BIOUG02680-F06 [Dorsal]
Evarcha hoyi
Family: Salticidae
BIN URI: BOLD:ACL8050

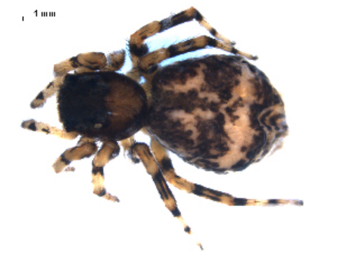

BIOUG00626-A04 [Dorsal]
Naphrys pulex
Family: Salticidae
BIN URI: BOLD:AAC2433

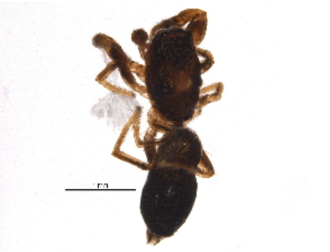

BIOUG10643-H02 [Dorsal]
Synageles noxiosus
Family: Salticidae
BIN URI: BOLD:ACL8115

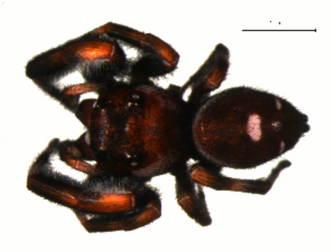

01-BB09US-C08 [Dorsal]
Phidippus audax
Family: Salticidae
BIN URI: BOLD:AAC6891

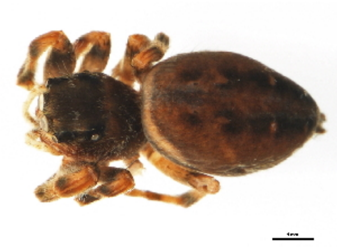

09ONTGAB-068 [Dorsal]
Phidippus clarus
Family: Salticidae
BIN URI: BOLD:AAC8083

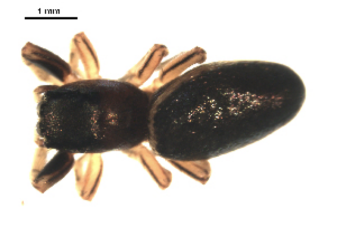

BIOUG00619-H01 [Dorsal]
Tutelina similis
Family: Salticidae
BIN URI: BOLD:AAF6387

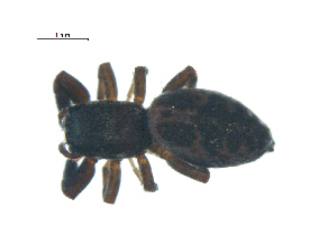

BIOUG07161-G05 [Dorsal]
Tutelina harti
Family: Salticidae
BIN URI: BOLD:AAW8769

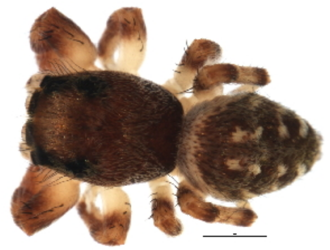

08BBARAC-0352 [Dorsal]
Eris militaris
Family: Salticidae
BIN URI: BOLD:AAA5654

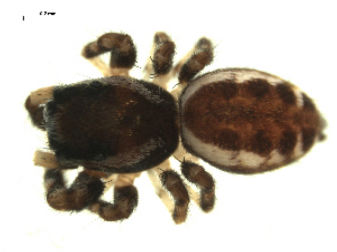

CCDB-04289-B12 [Dorsal]
Pelegrina proterva
Family: Salticidae
BIN URI: BOLD:AAB2927

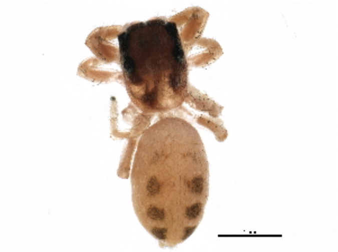

BIOUG07914-C03 [Dorsal]
Pelegrina proterva
Family: Salticidae
BIN URI: BOLD:ACI9841

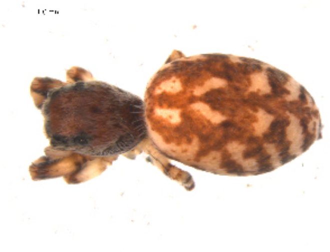

CCDB-08512-C11 [Dorsal]
Pelegrina galathea
Family: Salticidae
BIN URI: BOLD:AAB2930

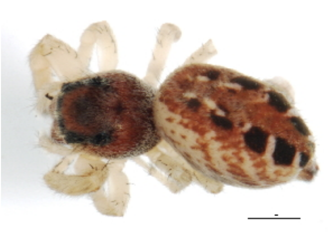

09ONTGAB-019 [Dorsal]
Pelegrina insignis
Family: Salticidae
BIN URI: BOLD:AAB2928

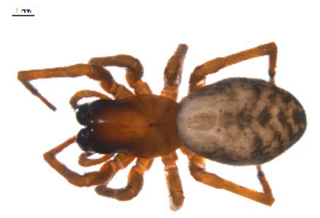

BIOUG12605-B10 [Dorsal]
Callobius bennetti
Family: Amaurobiidae
BIN URI: BOLD:AAB8212

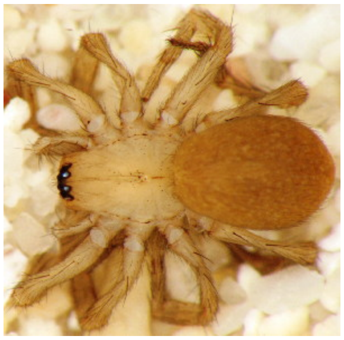

HF-2010-316 [Dorsal]
Cicurina brevis
Family: Dictynidae
BIN URI: BOLD:AAC8284

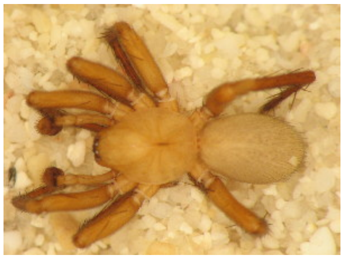

HF-2010-119pm [Dorsal]
Cicurina pallida
Family: Dictynidae
BIN URI: BOLD:AAF3046

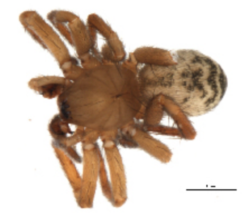

08EARGUE-0144 [Dorsal]
Cicurina itasca
Family: Dictynidae
BIN URI: BOLD:AAI4031

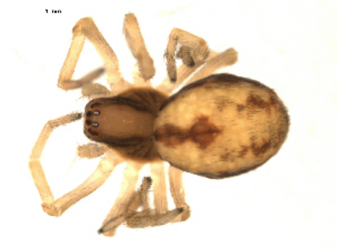

CCDB-04290-C06 [Dorsal]
Emblyna manitoba
Family: Dictynidae
BIN URI: BOLD:AAI9209

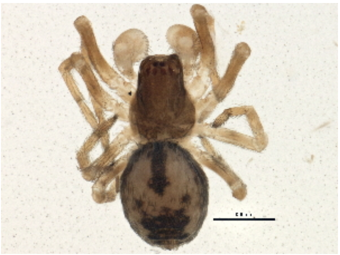

BIOUG05539-G09 [Dorsal]
Emblyna hentzi
Family: Dictynidae
BIN URI: BOLD:AAI6251

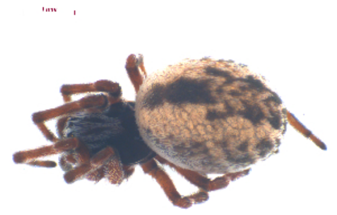

10-SKBC-0471 [Dorsal]
Dictyna volucripes
Family: Dictynidae
BIN URI: BOLD:AAB1638

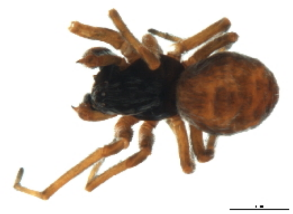

08BBARAC-0004 [Dorsal]
Dictyna volucripes
Family: Dictynidae
BIN URI: BOLD:ACE2869

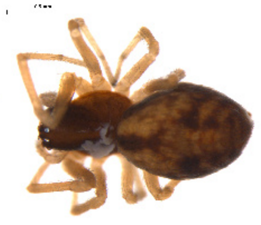

CCDB-05295-D09 [Dorsal]
Dictyna bellans
Family: Dictynidae
BIN URI: BOLD:AAI6249

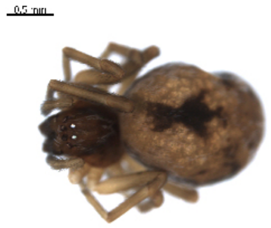

BIOUG00518-A05 [Dorsal]
Dictyna brevitarsa
Family: Dictynidae
BIN URI: BOLD:AAB2306

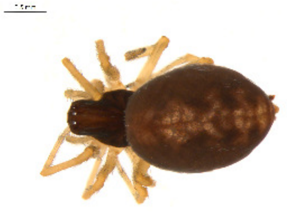

BIOUG12607-B01 [Dorsal]
Dictyna foliacea
Family: Dictynidae
BIN URI: BOLD:AAI6247

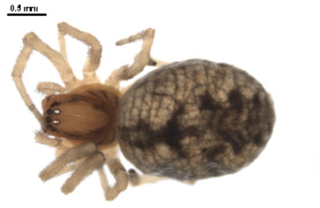

BIOUG00615-H04 [Dorsal]
Dictyna bostoniensis
Family: Dictynidae
BIN URI: BOLD:AAL1061

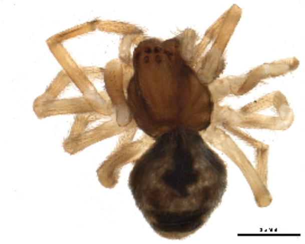

BIOUG01955-E04 [Dorsal]
Emblyna annulipes
Family: Dictynidae
BIN URI: BOLD:AAB4733

IMAGE NOT AVAILABLE

BIOUG22358-C12
Emblyna sublata
Family: Dictynidae

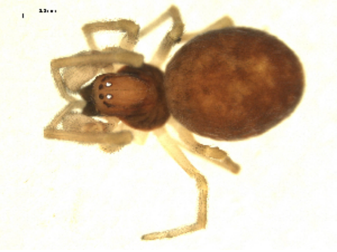

CCDB-04359-D02 [Lateral]
Emblyna sublata
Family: Dictynidae
BIN URI: BOLD:AAA7272

IMAGE NOT AVAILABLE

BIOUG20597-C05
Emblyna sublata
Family: Dictynidae

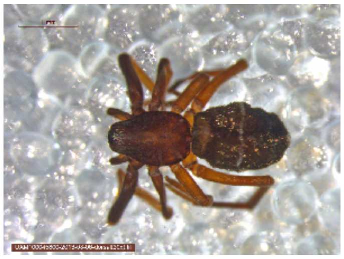

UAM:Ento:108356 [Dorsal]
Micaria pulicaria
Family: Gnaphosidae
BIN URI: BOLD:AAC6612

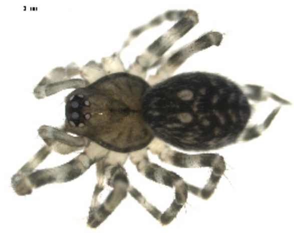

CCDB-08501-F08 [Dorsal]
Neoantistea gosiuta
Family: Hahniidae
BIN URI: BOLD:AAG9583

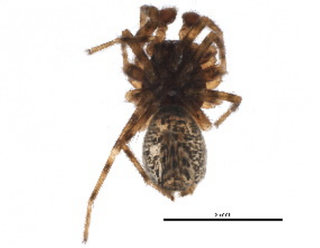

BIOUG20599-E03 [Dorsal]
Neoantistea agilis
Family: Hahniidae
BIN URI: BOLD:ACV5090

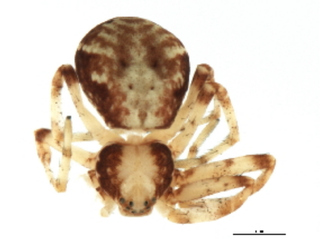

08SOAR-0097 [Dorsal]
Philodromus cespitum
Family: Philodromidae
BIN URI: BOLD:AAB3836

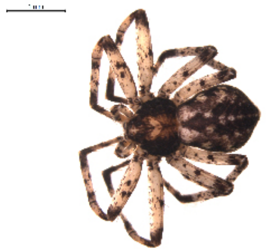

BIOUG00635-F06 [Dorsal]
Philodromus vulgaris
Family: Philodromidae
BIN URI: BOLD:AAD2665

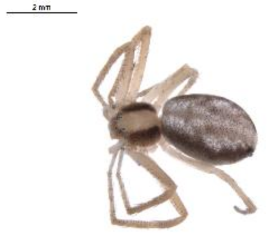

BIOUG00886-B09 [Dorsal]
Philodromus rufus vibrans
Family: Philodromidae
BIN URI: BOLD:AAB2768

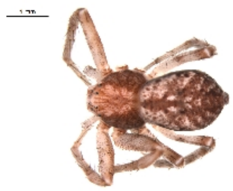

BIOUG12604-F04 [Dorsal]
Philodromus imbecillus
Family: Philodromidae
BIN URI: BOLD:AAI2838

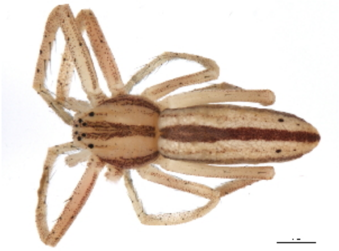

08BBARAC-0191 [Dorsal]
Tibellus maritimus
Family: Philodromidae
BIN URI: BOLD:AAA7188

IMAGE NOT AVAILABLE

BIOUG20596-D02
Tibellus oblongus
Family: Philodromidae
BIN URI: BOLD:AAA7188

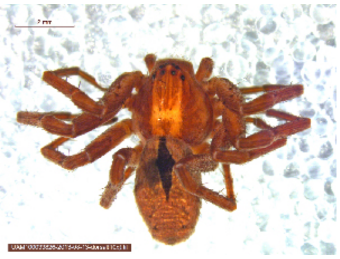

UAM:Ento:94524 [Dorsal]
Thanatus formicinus
Family: Philodromidae

IMAGE NOT AVAILABLE

BIOUG20598-H10
Habronattus decorus
Family: Salticidae

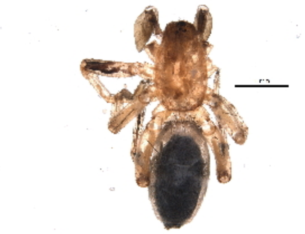

BIOUG20599-C06 [Dorsal]
Sergiolus ocellatus
Family: Gnaphosidae
BIN URI: BOLD:ACV6055

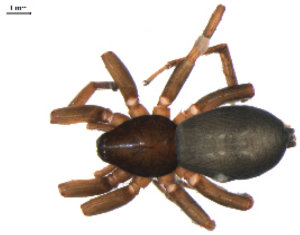

BIOUG00614-C01 [Dorsal]
Herpyllus ecclesiasticus
Family: Gnaphosidae
BIN URI: BOLD:AAF2106

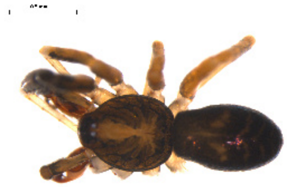

CCDB-05303-A05 [Dorsal]
Phrurotimpus borealis
Family: Phrurolithidae
BIN URI: BOLD:AAC7234

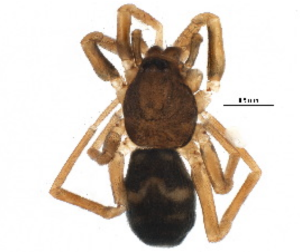

BIOUG21773-F05 [Dorsal]
Scotinella pugnata
Family: Phrurolithidae
BIN URI: BOLD:AAK7452

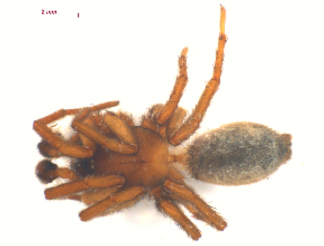

10-SKBC-0613 [Dorsal]
Haplodrassus signifer
Family: Gnaphosidae
BIN URI: BOLD:AAD0462

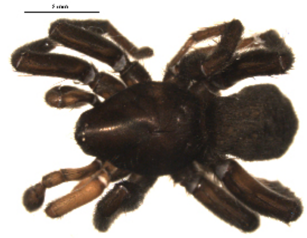

CCDB-05258-E08 [Dorsal]
Gnaphosa parvula
Family: Gnaphosidae
BIN URI: BOLD:AAC3779

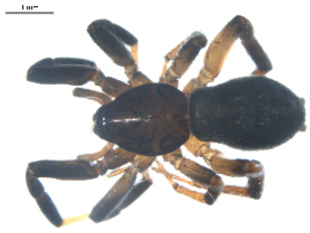

BIOUG00617-A08 [Dorsal]
Drassyllus depressus
Family: Gnaphosidae
BIN URI: BOLD:AAD8676

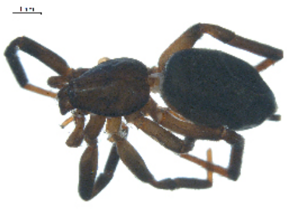

BIOUG07161-G06 [Dorsal]
Drassyllus niger
Family: Gnaphosidae
BIN URI: BOLD:AAI9037

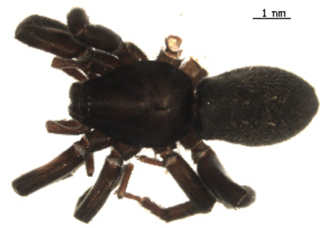

BIOUG00515-G03 [Dorsal]
Zelotes fratris
Family: Gnaphosidae
BIN URI: BOLD:AAA8914

IMAGE NOT AVAILABLE

BIOUG20598-A04
Zelotes pseustes
Family: Gnaphosidae

IMAGE NOT AVAILABLE

BIOUG21899-F02
Sarcoptiformes

BIN URI: BOLD:ACV5533

IMAGE NOT AVAILABLE

BIOUG21773-G02
Alycidae
Family: Alycidae
BIN URI: BOLD:ACV5777

IMAGE NOT AVAILABLE

BIOUG08101-C10
Brachychthoniidae
Family: Brachychthoniidae
BIN URI: BOLD:ACJ0065

IMAGE NOT AVAILABLE

BIOUG21882-D09
Brachychthoniidae
Family: Brachychthoniidae
BIN URI: BOLD:ACV6448

IMAGE NOT AVAILABLE

BIOUG21882-E08
Brachychthoniidae
Family: Brachychthoniidae
BIN URI: BOLD:ACV7571

IMAGE NOT AVAILABLE

BIOUG20565-C01
Nanorchestidae
Family: Nanorchestidae
BIN URI: BOLD:ACV4771

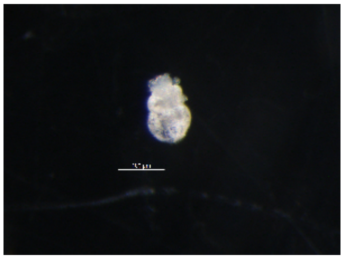

BIOUG01073-78 [Dorsal]
Nanorchestes
Family: Nanorchestidae
BIN URI: BOLD:AAW0387

IMAGE NOT AVAILABLE

BIOUG21897-H09
Nanorchestidae
Family: Nanorchestidae
BIN URI: BOLD:ACV5490

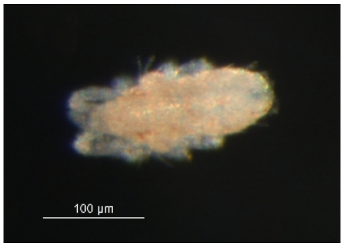

BIOUG01178-21 [Dorsal]
Terpnacaridae
Family: Terpnacaridae
BIN URI: BOLD:AAZ4544

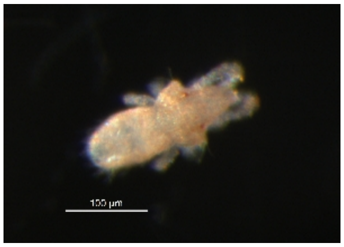

BIOUG01181-76 [Dorsal]
Terpnacaridae
Family: Terpnacaridae
BIN URI: BOLD:ABV1694

IMAGE NOT AVAILABLE

BIOUG21897-F08
Terpnacaridae
Family: Terpnacaridae
BIN URI: BOLD:ACV5851

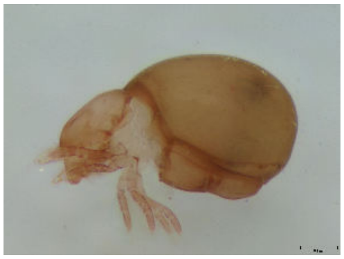

FINOR-20120366 [Lateral]
Phthiracarus crinitus
Family: Phthiracaridae
BIN URI: BOLD:AAF6471

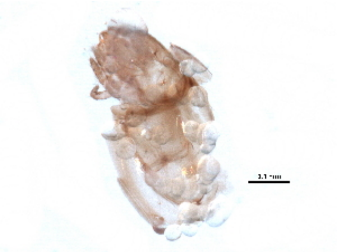

BIOUG24000-A09 [Dorsal]
Phthiracarus
Family: Phthiracaridae

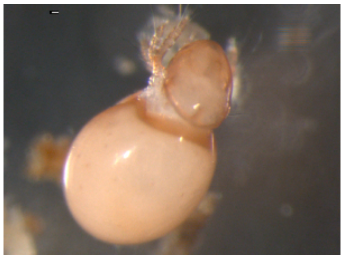

08MIONT-0216 [Dorsal]
Phthiracaridae
Family: Phthiracaridae
BIN URI: BOLD:AAF9149

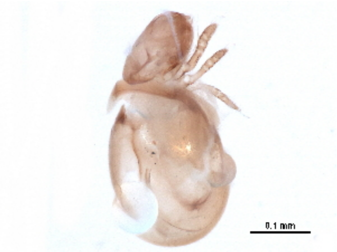

BIOUG20565-B03 [Dorsal]
Phthiracaridae
Family: Phthiracaridae
BIN URI: BOLD:ACV3446

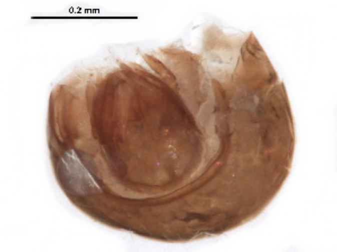

BIOUG21899-C03 [Dorsal]
Phthiracaridae
Family: Phthiracaridae
BIN URI: BOLD:ACV5448

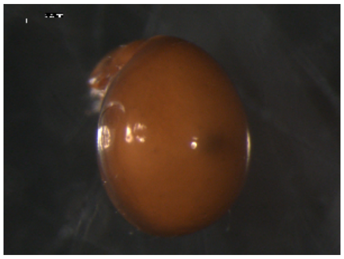

08MIONT-0066 [Dorsal]
Phthiracaridae
Family: Phthiracaridae
BIN URI: BOLD:AAF9095

IMAGE NOT AVAILABLE

BIOUG24005-H07
Phthiracaridae
Family: Phthiracaridae

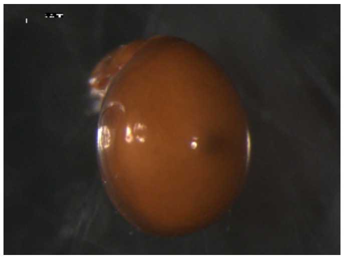

08MIONT-0065 [Dorsal]
Phthiracaridae
Family: Phthiracaridae
BIN URI: BOLD:AAF9094

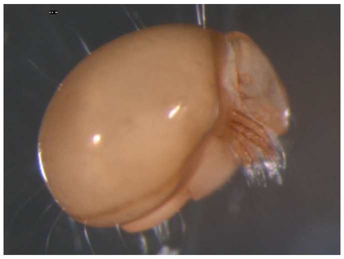

08MIONT-0251 [Dorsal]
Phthiracaridae
Family: Phthiracaridae
BIN URI: BOLD:AAF9158

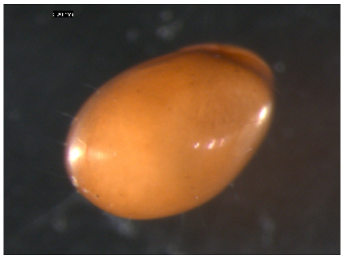

08MIONT-0160 [Dorsal]
Phthiracaridae
Family: Phthiracaridae
BIN URI: BOLD:AAF9142

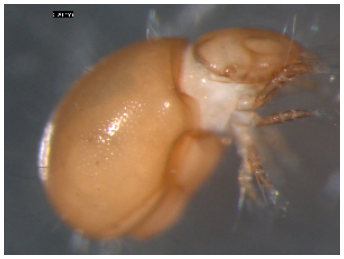

08MIONT-0128 [Dorsal]
Phthiracaridae
Family: Phthiracaridae
BIN URI: BOLD:AAF9137

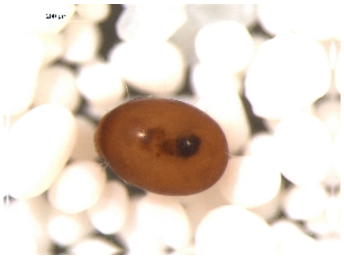

DPMIT-24-76 [Dorsal]
Phthiracaridae
Family: Phthiracaridae
BIN URI: BOLD:ABA8153

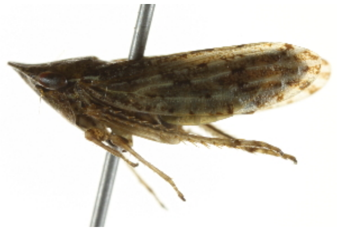

PCPP10-0684 [Dorsal]
Aphrodes makarovi
Family: Cicadellidae
BIN URI: BOLD:AAG2876

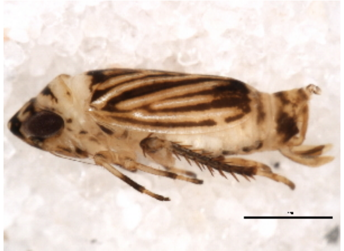

09BBEHE-218 [Lateral]
Anoscopus flavostriatus
Family: Cicadellidae
BIN URI: BOLD:AAN8385

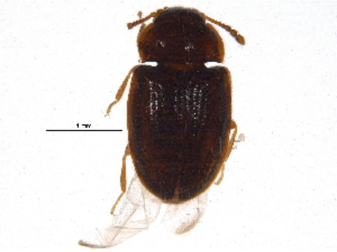

BIOUG09832-A06 [Dorsal]
Endomychidae
Family: Endomychidae
BIN URI: BOLD:ACL2587

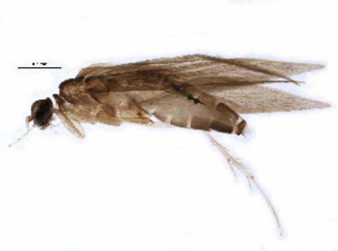

BIOUG11743-G05 [Lateral]
Hydroptilidae
Family: Hydroptilidae
BIN URI: BOLD:ACM7558

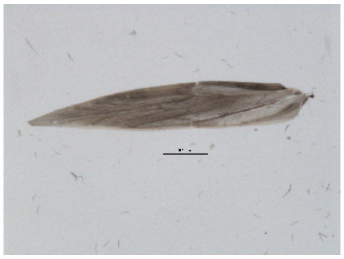

07ELEPT-167 [Lateral]
Orthotrichia cristata
Family: Hydroptilidae
BIN URI: BOLD:ACM7774

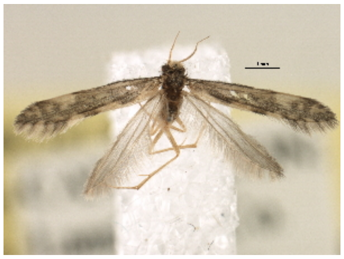

BIOUG16764-F12 [Dorsal]
Agraylea multipunctata
Family: Hydroptilidae
BIN URI: BOLD:AAA3877

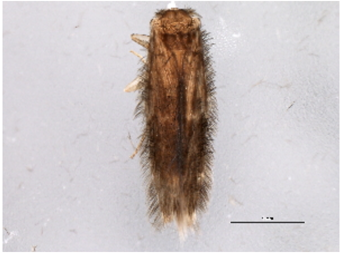

10HDT-036 [Dorsal]
Hydroptila perdita
Family: Hydroptilidae
BIN URI: BOLD:AAE5187

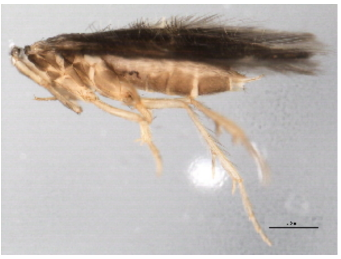

10HDT-037 [Lateral]
Hydroptila armata
Family: Hydroptilidae
BIN URI: BOLD:AAF4109

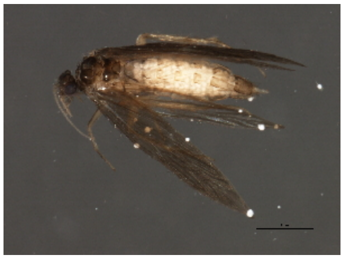

08ONCAD-0270 [Dorsal]
Hydroptila spatulata
Family: Hydroptilidae
BIN URI: BOLD:AAD0137

IMAGE NOT AVAILABLE

BIOUG22581-A11
Hydroptila spatulata
Family: Hydroptilidae

IMAGE NOT AVAILABLE

BIOUG22581-B03
Hydroptila spatulata
Family: Hydroptilidae

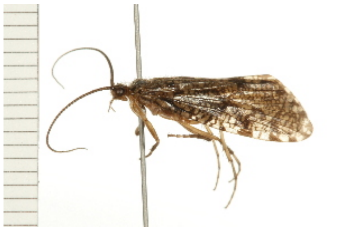

08NBEPT-1427 [Lateral]
Banksiola crotchi
Family: Phryganeidae
BIN URI: BOLD:AAA4801

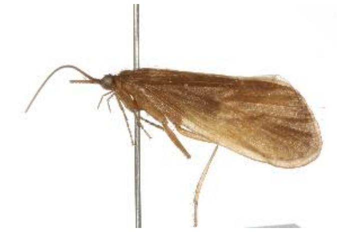

TRIC 0234.02 [Lateral]
Ironoquia punctatissima
Family: Limnephilidae
BIN URI: BOLD:AAB2190

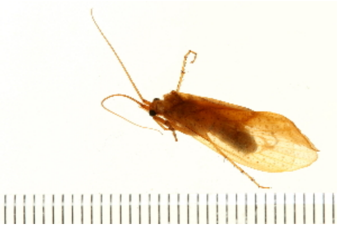

08INHST-055 [Lateral]
Pycnopsyche antica
Family: Limnephilidae
BIN URI: BOLD:ACF4302

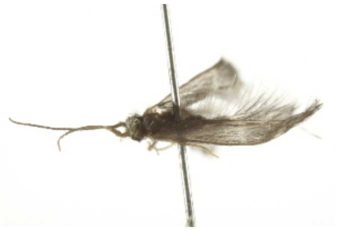

10USCAD-019 [Lateral]
Trichoptera

BIN URI: BOLD:AAO3983

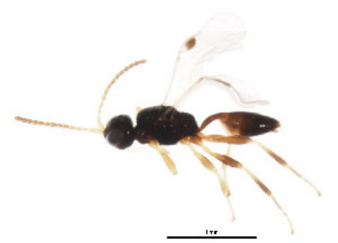

BIOUG01049-B10 [Lateral]
Euphoriella
Family: Braconidae
BIN URI: BOLD:AAU9119

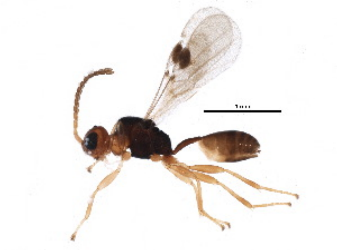

BIOUG22723-B12 [Lateral]
Braconidae
Family: Braconidae
BIN URI: BOLD:ACV5990

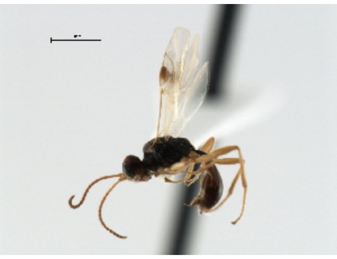

CBRA0441 [Lateral]
Peristenus sp.
Family: Braconidae
BIN URI: BOLD:AAA8469

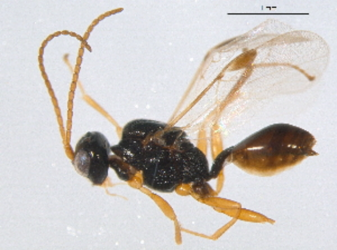

BIOUG05726-D09 [Lateral]
Peristenus
Family: Braconidae
BIN URI: BOLD:AAA8464

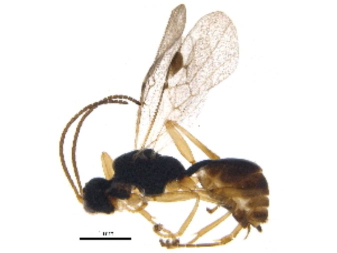

BIOUG22573-G02 [Lateral]
Peristenus
Family: Braconidae
BIN URI: BOLD:ACV4624

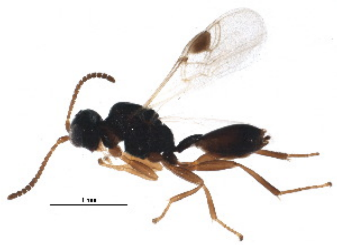

BIOUG22453-E08 [Lateral]
Peristenus
Family: Braconidae
BIN URI: BOLD:ACK1458

IMAGE NOT AVAILABLE

BIOUG22927-B09
Peristenus
Family: Braconidae

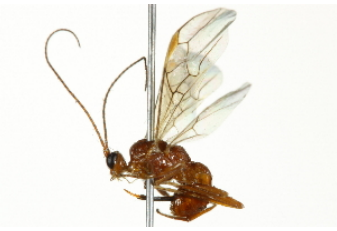

10BBCHY-3501 [Lateral]
Euphorinae
Family: Braconidae
BIN URI: BOLD:AAU8900

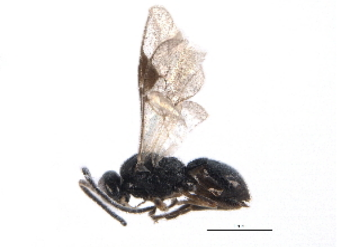

10PHMAL-0722 [Lateral]
Euphorinae
Family: Braconidae
BIN URI: BOLD:AAU8319

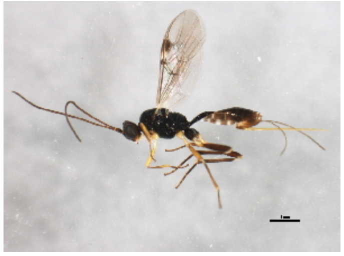

BIOUG00988-A06 [Lateral]
Hymenoptera

BIN URI: BOLD:ABX2611

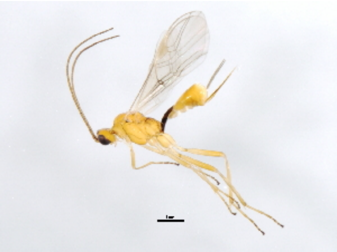

09BBEHY-0207 [Lateral]
Meteorus
Family: Braconidae
BIN URI: BOLD:AAI1549

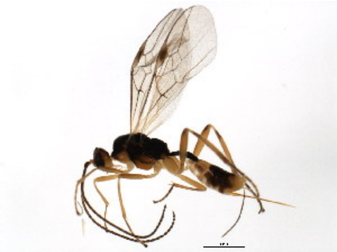

BIOUG00862-E11 [Lateral]
Meteorus
Family: Braconidae
BIN URI: BOLD:AAZ3290

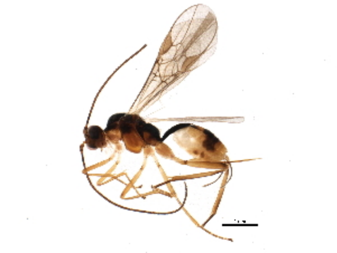

BIOUG08609-A01 [Lateral]
Meteorus
Family: Braconidae
BIN URI: BOLD:ACK1841

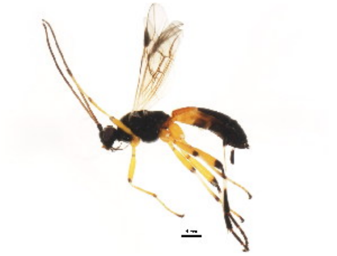

BIOUG01082-E03 [Lateral]
Aleiodes
Family: Braconidae
BIN URI: BOLD:AAG7629

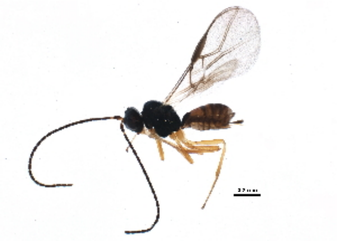

BIOUG16157-E11 [Lateral]
Opiinae
Family: Braconidae
BIN URI: BOLD:ACL6870

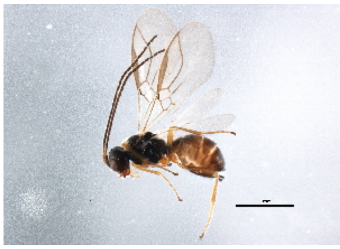

BIOUG16088-A06 [Lateral]
Alysiinae
Family: Braconidae
BIN URI: BOLD:AAN8140

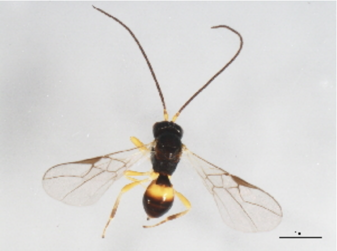

BIOUG00836-H09 [Dorsal]
Braconidae
Family: Braconidae
BIN URI: BOLD:AAY9202

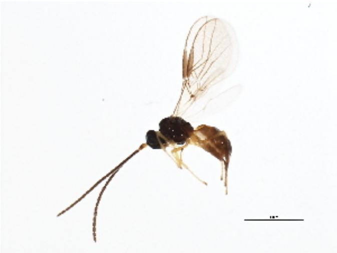

BIOUG00836-A07 [Lateral]
Opiinae
Family: Braconidae
BIN URI: BOLD:AAG8413

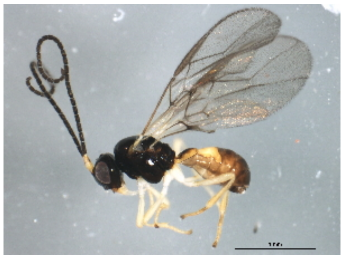

BIOUG01029-H03 [Lateral]
Opiinae
Family: Braconidae
BIN URI: BOLD:ABA5939

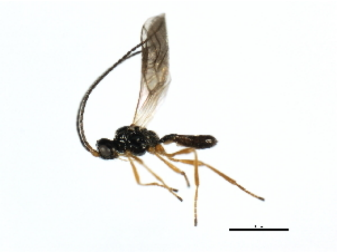

07PROBE-22199 [Lateral]
Alysiinae
Family: Braconidae
BIN URI: BOLD:AAG1289

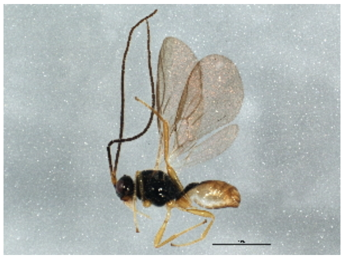

BIOUG03385-E01 [Lateral]
Alysiinae
Family: Braconidae
BIN URI: BOLD:AAY9197

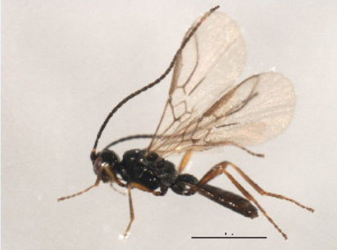

10BBCHY-2904 [Lateral]
Alysiinae
Family: Braconidae
BIN URI: BOLD:AAN8138

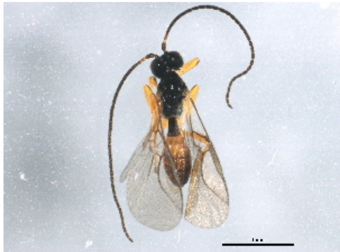

BIOUG01029-H04 [Lateral]
Alysiinae
Family: Braconidae
BIN URI: BOLD:AAQ2937

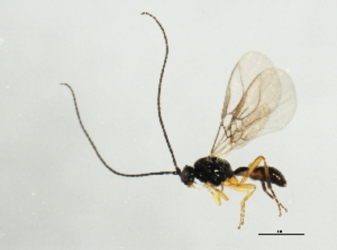

10BBCHY-1868 [Lateral]
Alysiinae
Family: Braconidae
BIN URI: BOLD:AAG1322

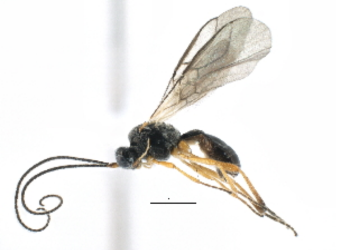

BIOUG04788-F02 [Lateral]
Chorebus 1MJS
Family: Braconidae
BIN URI: BOLD:AAM7414

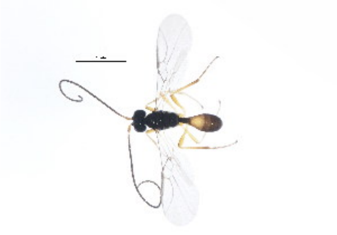

BIOUG00857-A12 [Dorsal]
Alysiinae
Family: Braconidae
BIN URI: BOLD:AAU8209

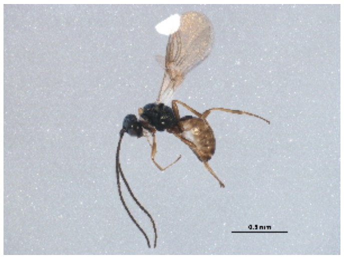

BIOUG16006-D04 [Lateral]
Alysiinae
Family: Braconidae
BIN URI: BOLD:ACP8084

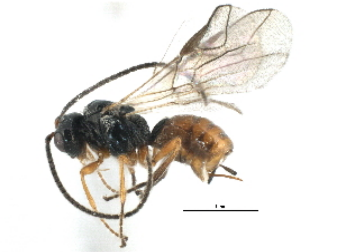

BIOUG08025-D05 [Lateral]
Opiinae
Family: Braconidae
BIN URI: BOLD:ACI9828

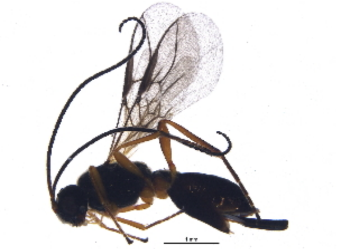

BIOUG13284-A06 [Lateral]
Alysiinae
Family: Braconidae
BIN URI: BOLD:ACO4997

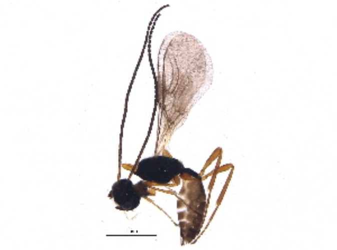

BIOUG13981-C11 [Lateral]
Alysiinae
Family: Braconidae
BIN URI: BOLD:ACO7449

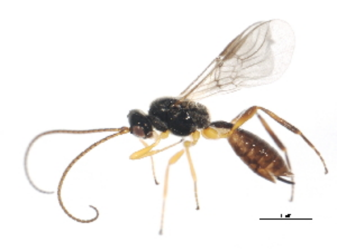

09BBHYM-651 [Lateral]
Braconidae
Family: Braconidae
BIN URI: BOLD:AAG8239

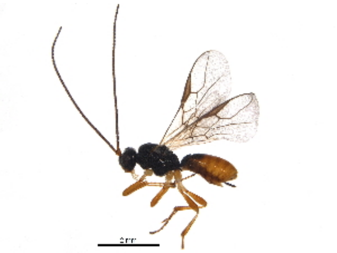

BIOUG22866-G05 [Lateral]
Braconidae
Family: Braconidae
BIN URI: BOLD:ACW1166

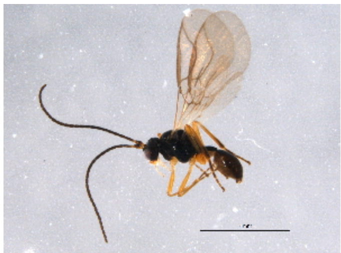

BIOUG04804-H04 [Lateral]
Alysiinae
Family: Braconidae
BIN URI: BOLD:ACG4118

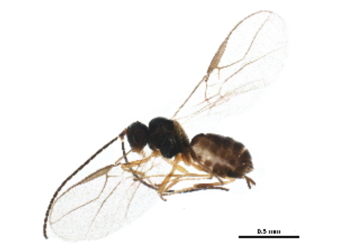

BIOUG23320-H06 [Lateral]
Braconidae
Family: Braconidae
BIN URI: BOLD:ACW1288

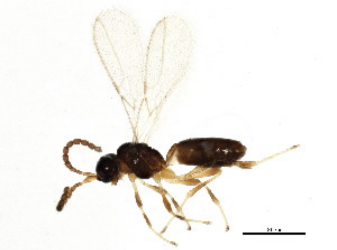

BIOUG01605-G06 [Lateral]
Alysiinae
Family: Braconidae
BIN URI: BOLD:AAN8202

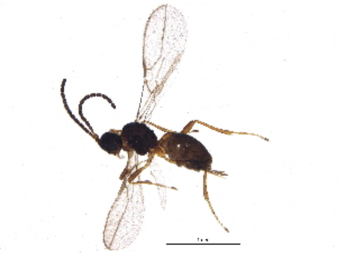

BIOUG11205-A11 [Lateral]
Dinotrema
Family: Braconidae
BIN URI: BOLD:ACF3545

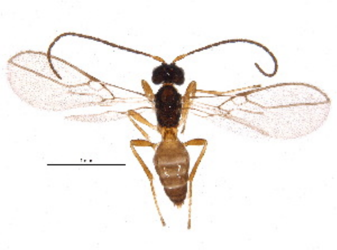

BIOUG19927-G01 [Dorsal]
Alysiinae
Family: Braconidae
BIN URI: BOLD:ACT0326

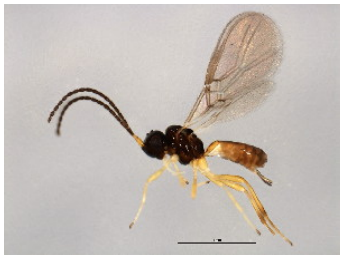

ASGLE-0229 [Lateral]
Hymenoptera

BIN URI: BOLD:AAF5747

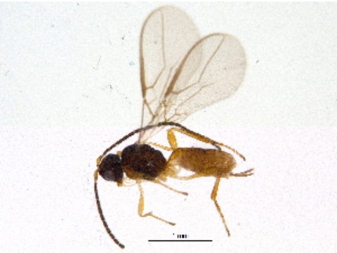

ASGLE2-0489 [Lateral]
Hymenoptera

BIN URI: BOLD:AAU8558

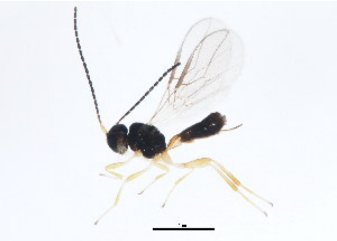

BIOUG00857-E10 [Lateral]
Dinotrema
Family: Braconidae
BIN URI: BOLD:AAY6824

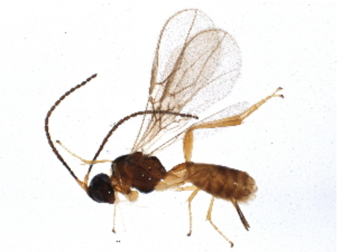

BIOUG10452-H09 [Lateral]
Alysiinae
Family: Braconidae
BIN URI: BOLD:AAH3190

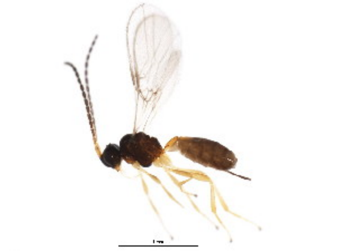

BIOUG01258-H04 [Lateral]
Dinotrema JFT23
Family: Braconidae
BIN URI: BOLD:AAU8348

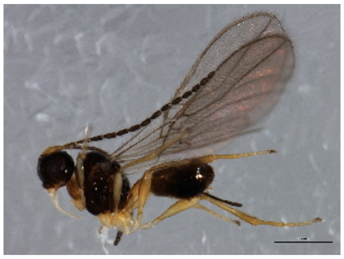

09BBEHY-1674 [Lateral]
Alysiinae
Family: Braconidae
BIN URI: BOLD:AAM7451

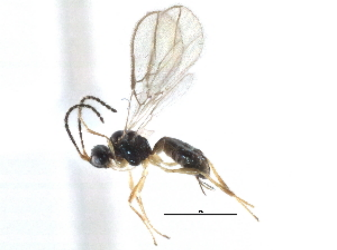

BIOUG08568-E11 [Lateral]
Dinotrema
Family: Braconidae
BIN URI: BOLD:AAU8452

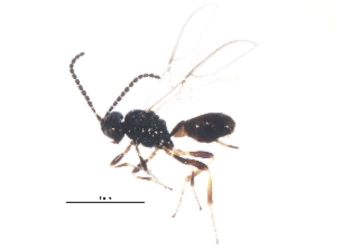

ASGLE-0576 [Lateral]
Hymenoptera

BIN URI: BOLD:AAU8379

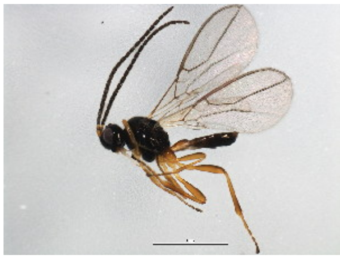

ASGLE-0545 [Lateral]
Hymenoptera

BIN URI: BOLD:AAG1342

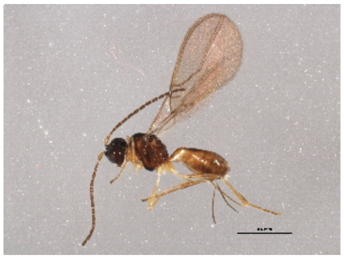

BIOUG06437-B11 [Lateral]
Dinotrema
Family: Braconidae
BIN URI: BOLD:ACI4486

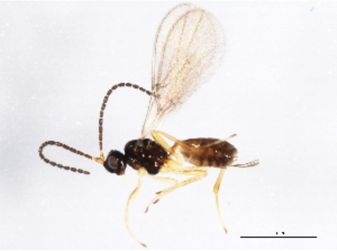

ASGLE2-0228 [Lateral]
Hymenoptera

BIN URI: BOLD:ABZ5626

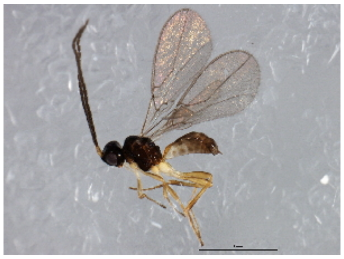

09BBEHY-1690 [Lateral]
Dinotrema
Family: Braconidae
BIN URI: BOLD:ACE5711

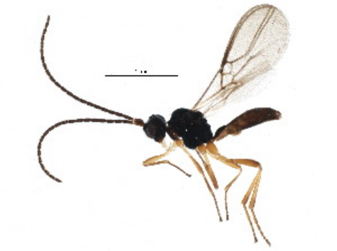

BIOUG20793-G12 [Lateral]
Alysiinae
Family: Braconidae
BIN URI: BOLD:ACU9141

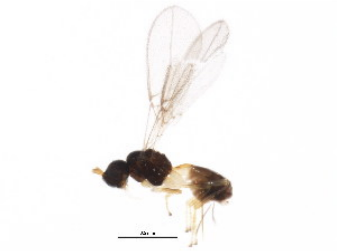

BIOUG01258-B11 [Lateral]
Dinotrema JFT01
Family: Braconidae
BIN URI: BOLD:ACE5710

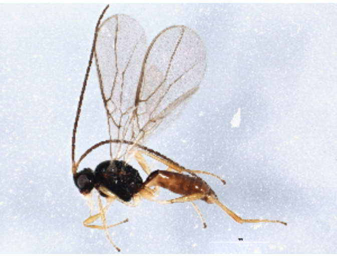

BIOUG06249-G10 [Lateral]
Dinotrema
Family: Braconidae
BIN URI: BOLD:AAA7636

IMAGE NOT AVAILABLE

BIOUG22322-F05
Dinotrema
Family: Braconidae

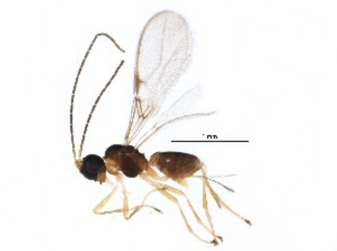

BIOUG22453-A01 [Lateral]
Asobara rufescens
Family: Braconidae
BIN URI: BOLD:AAU8583

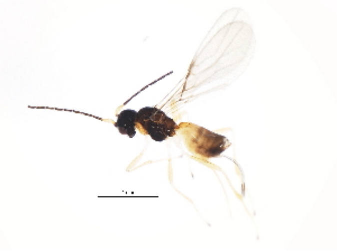

BIOUG00845-G05 [Lateral]
Asobara
Family: Braconidae
BIN URI: BOLD:AAU8579

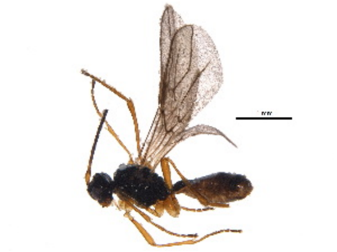

BIOUG21888-B10 [Lateral]
Phaenocarpa
Family: Braconidae
BIN URI: BOLD:ACV5717

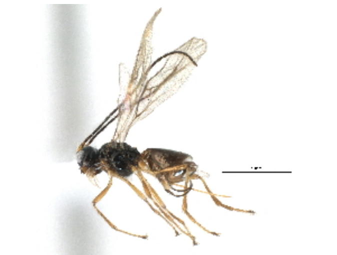

BIOUG08658-D03 [Lateral]
Asobara
Family: Braconidae
BIN URI: BOLD:AAH3171

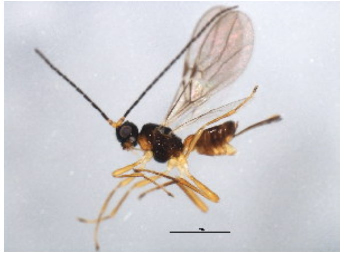

ASGLE-0237 [Lateral]
Hymenoptera

BIN URI: BOLD:AAM7459

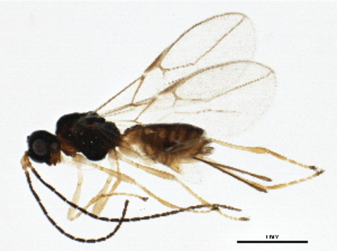

BIOUG02604-G10 [Lateral]
Asobara
Family: Braconidae
BIN URI: BOLD:ABY1452

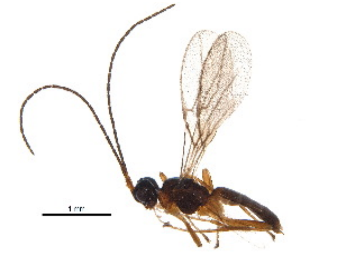

BIOUG24007-C03 [Lateral]
Alysiinae
Family: Braconidae

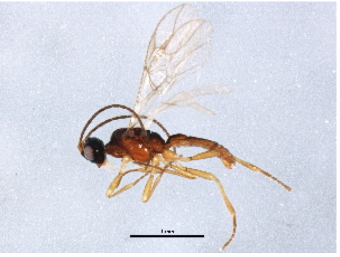

BIOUG01657-B04 [Lateral]
Rhyssalinae
Family: Braconidae
BIN URI: BOLD:AAO2112

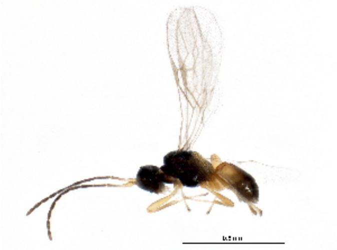
BIOUG22458-B12 [Lateral]
Rhyssalinae
Family: Braconidae
BIN URI: BOLD:AAQ2892

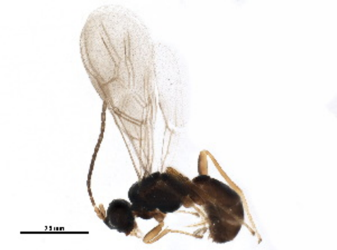
BIOUG22421-B04 [Lateral]
Braconidae
Family: Braconidae
BIN URI: BOLD:ACO8570

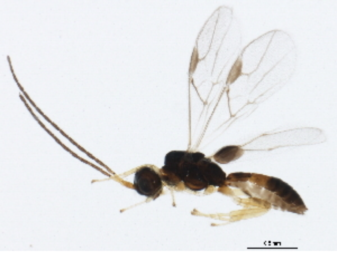
10PHMAL-3249 [Lateral]
Doryctinae
Family: Braconidae
BIN URI: BOLD:AAU8206

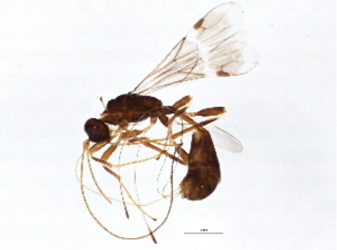
BIOUG05096-A07 [Lateral]
Doryctinae
Family: Braconidae
BIN URI: BOLD:ACG6283

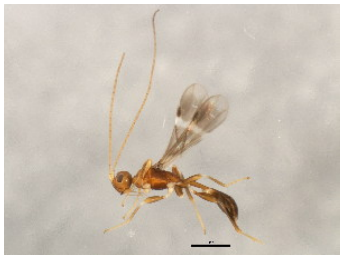
ASGLE-0857 [Lateral]
Hymenoptera

BIN URI: BOLD:ACM3076

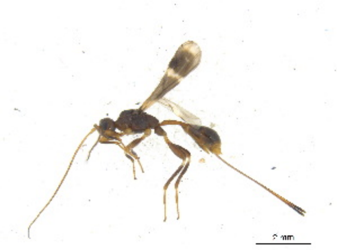
BIOUG06753-F06 [Lateral]
Hymenoptera

BIN URI: BOLD:ACM8393

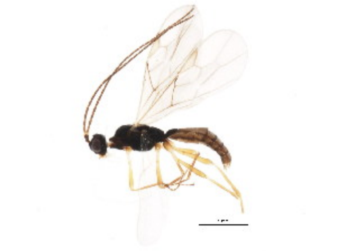
BIOUG01037-D03 [Lateral]
Hormius
Family: Braconidae
BIN URI: BOLD:ABA5915

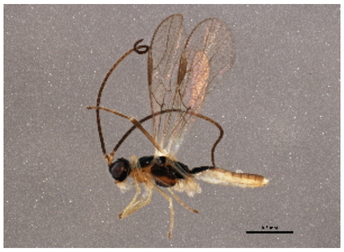
BIOUG03166-H04 [Lateral]
Braconidae
Family: Braconidae
BIN URI: BOLD:AAY9207

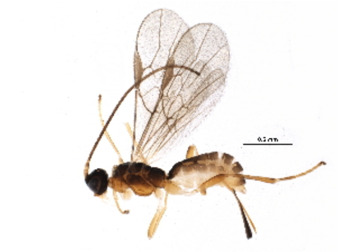
BIOUG21522-B07 [Lateral]
Hymenoptera

BIN URI: BOLD:ACU9920

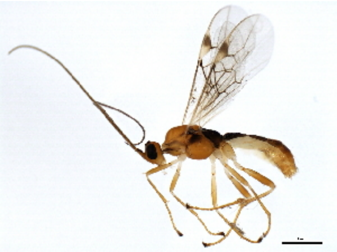
10BBHYM-1181 [Lateral]
Aleiodes
Family: Braconidae
BIN URI: BOLD:AAG1350

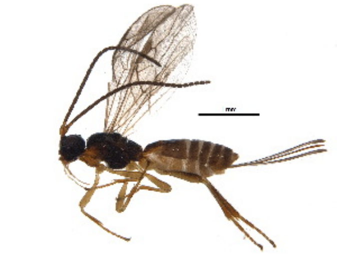
BIOUG24007-E01 [Lateral]
Braconidae
Family: Braconidae

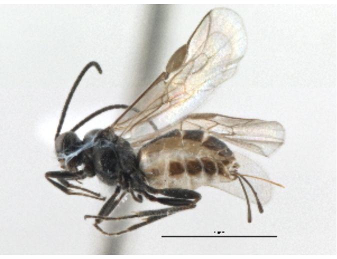
BIOUG04050-F09 [Lateral]
Habrobracon
Family: Braconidae
BIN URI: BOLD:AAG8403

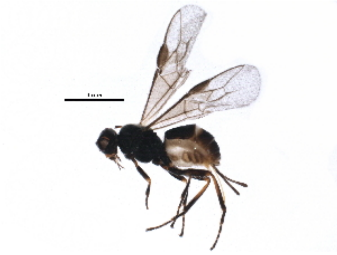
BIOUG24034-G07 [Lateral]
Braconidae
Family: Braconidae

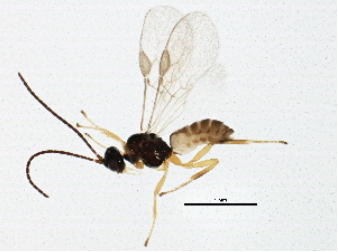
BIOUG02746-B06 [Lateral]
Bracon
Family: Braconidae
BIN URI: BOLD:ACA7945

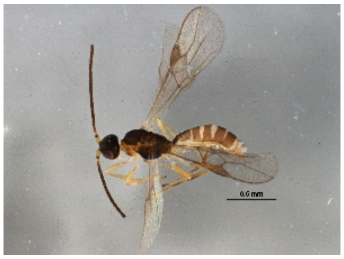
BIOUG08589-A06 [Lateral]
Bracon
Family: Braconidae
BIN URI: BOLD:ACK1835

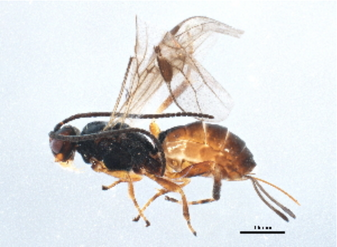
BIOUG10360-F03 [Lateral]
Bracon
Family: Braconidae
BIN URI: BOLD:ABV2692

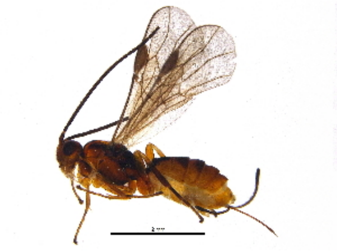
BIOUG08001-F08 [Lateral]
Bracon
Family: Braconidae
BIN URI: BOLD:ACI9921

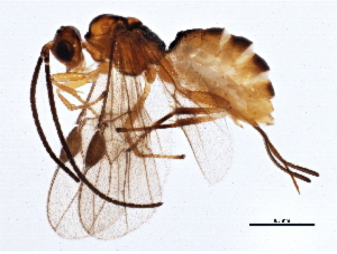
BIOUG03049-G09 [Lateral]
Bracon
Family: Braconidae
BIN URI: BOLD:AAG1345

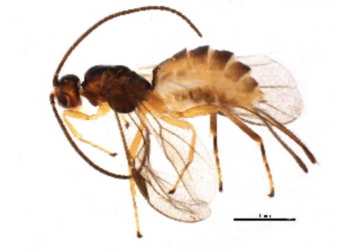
BIOUG08746-E07 [Lateral]
Bracon
Family: Braconidae
BIN URI: BOLD:ACP8803

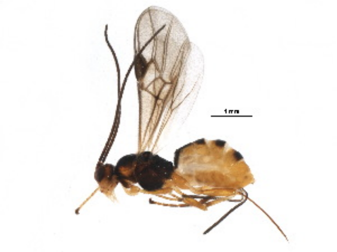
BIOUG22420-D02 [Lateral]
Bracon
Family: Braconidae
BIN URI: BOLD:ACV3190

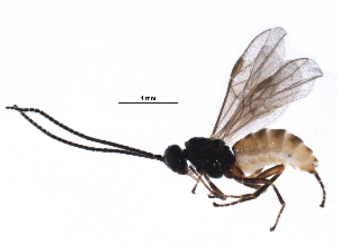

BIOUG24038-B11 [Lateral]
Braconidae
Family: Braconidae

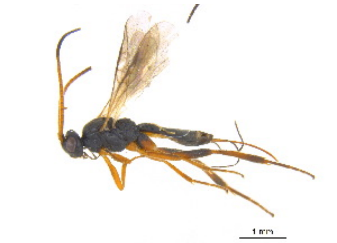

BIOUG06752-D10 [Lateral]
Hymenoptera

BIN URI: BOLD:AAG1084

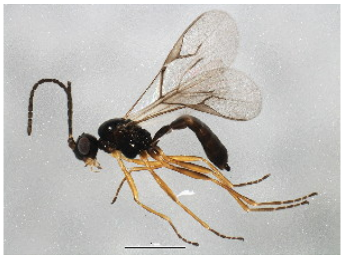

ASGLE-0915 [Lateral]
Hymenoptera

BIN URI: BOLD:AAA4188

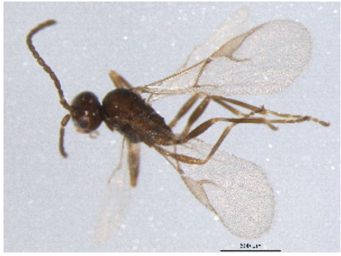

BIOUG05612-G12 [Lateral]
Diaeretiella rapae
Family: Braconidae
BIN URI: BOLD:AAG1421

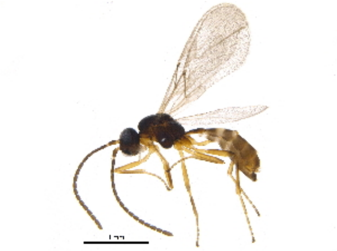

BIOUG21771-G01 [Lateral]
Aphidius
Family: Braconidae
BIN URI: BOLD:AAK2038

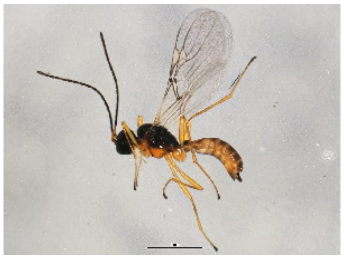

ASGLE-0569 [Lateral]
Hymenoptera

BIN URI: BOLD:AAH7443

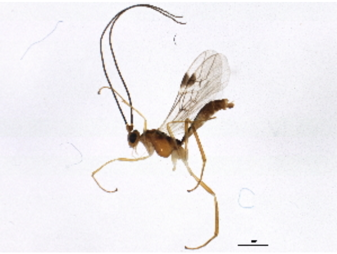

BIOUG01839-E10 [Lateral]
Macrocentrus
Family: Braconidae
BIN URI: BOLD:AAE8369

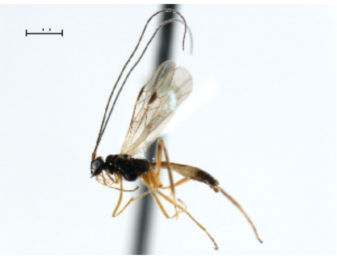

CBRA0158 [Lateral]
Macrocentrus sp.
Family: Braconidae
BIN URI: BOLD:AAE8370

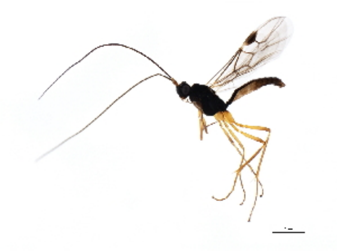

08BBHYM-0876 [Lateral]
Macrocentrus
Family: Braconidae
BIN URI: BOLD:AAG1352

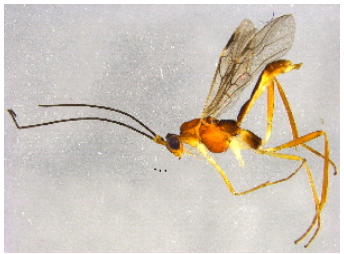

BIOUG03698-D07 [Lateral]
Macrocentrus
Family: Braconidae
BIN URI: BOLD:AAY6794

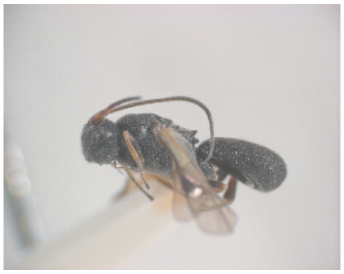

NZAC04036453 [Lateral]
Ascogaster quadridentata
Family: Braconidae
BIN URI: BOLD:AAI4826

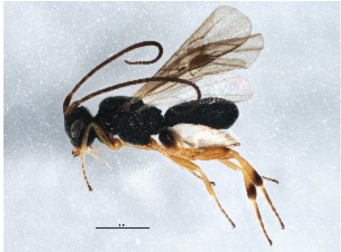

BIOUG06009-B08 [Lateral]
Ascogaster
Family: Braconidae
BIN URI: BOLD:ACI7729

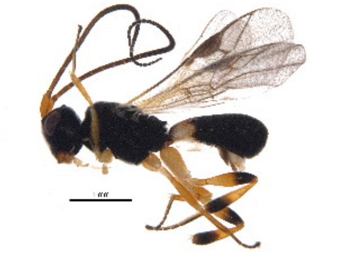

BIOUG22565-D07 [Lateral]
Ascogaster
Family: Braconidae
BIN URI: BOLD:ACV3169

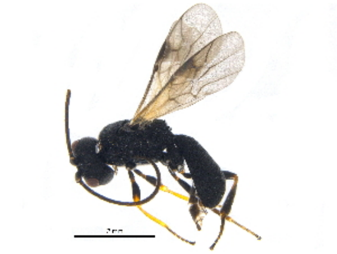

BIOUG22872-G05 [Lateral]
Chelonus
Family: Braconidae
BIN URI: BOLD:ACV5685

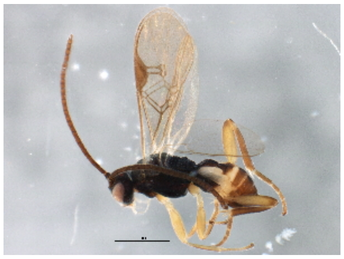

BIOUG01572-G05 [Lateral]
Diolcogaster jft32
Family: Braconidae
BIN URI: BOLD:AAI6272

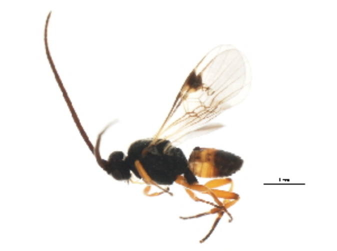

PCPP10-0449 [Lateral]
Microplitis jft53
Family: Braconidae
BIN URI: BOLD:AAE8502

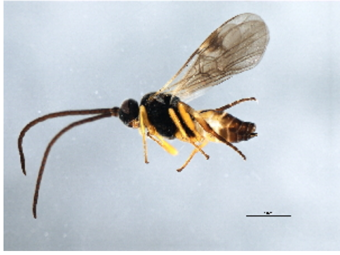

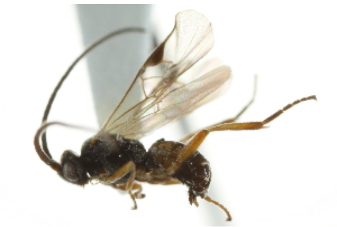

07PROBE-22352 [Lateral]
Microplitis varicolor

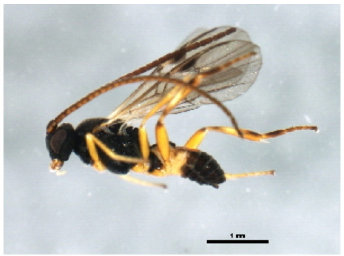

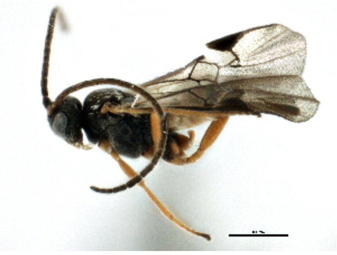

09BBHYM-904 [Lateral]
Microplitis jft28
Family: Braconidae
BIN URI: BOLD:AAK6504

Family: Braconidae
BIN URI: BOLD:AAA2408

BIOUG01029-D10 [Lateral]
Microplitis jft21
Family: Braconidae
BIN URI: BOLD:AAE8603

BIOUG01638-C10 [Lateral]
Microplitis jft23
Family: Braconidae
BIN URI: BOLD:ABZ3353

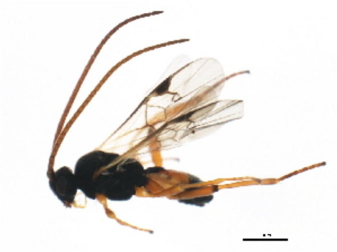

BIOUG01033-F05 [Lateral]
Microplitis jft60
Family: Braconidae
BIN URI: BOLD:AAE8461

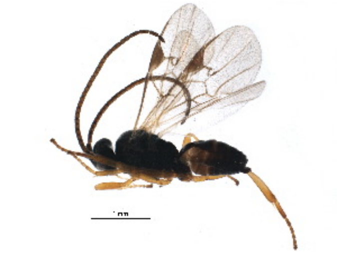

BIOUG22469-F06 [Lateral]
Microplitis
Family: Braconidae
BIN URI: BOLD:AAH3516

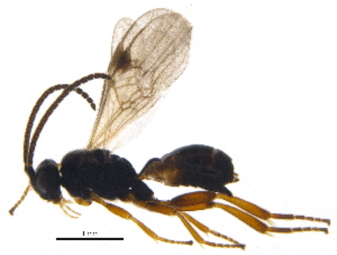

BIOUG22465-F02 [Lateral]
Microplitis
Family: Braconidae
BIN URI: BOLD:ACV5700

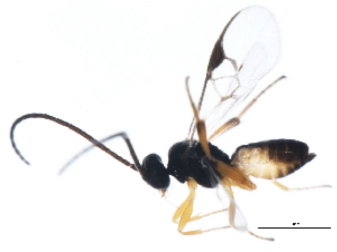

TDWG-0316 [Lateral]
Glyptapanteles sp.
Family: Braconidae
BIN URI: BOLD:AAA4781

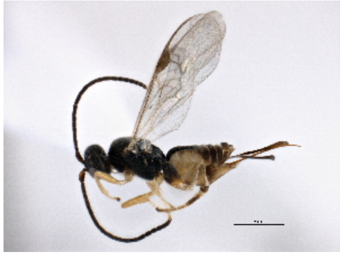

BIOUG07081-A05 [Lateral]
Choeras
Family: Braconidae
BIN URI: BOLD:AAK1835

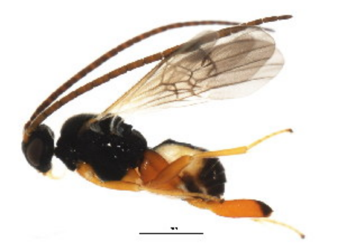

BIOUG01088-F11 [Lateral]
Diolcogaster jft30
Family: Braconidae
BIN URI: BOLD:AAB0185

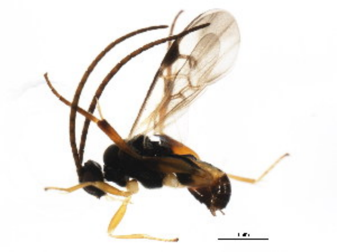

BIOUG01149-D11 [Lateral]
Diolcogaster facetosa
Family: Braconidae
BIN URI: BOLD:ABA5941

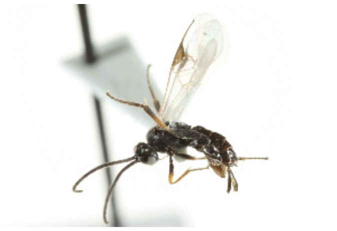

07PROBE-22434 [Lateral]
Apanteles jft01
Family: Braconidae
BIN URI: BOLD:AAA6373

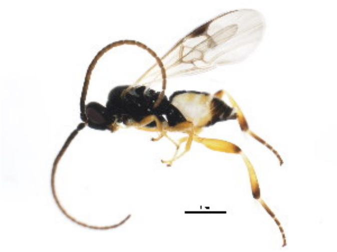

BIOUG01032-F09 [Lateral]
Microgaster jft11
Family: Braconidae
BIN URI: BOLD:AAH3530

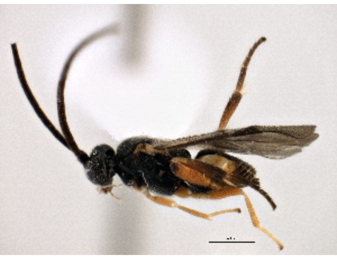

BIOUG03675-F02 [Lateral]
Microgaster jft17
Family: Braconidae
BIN URI: BOLD:AAA7886

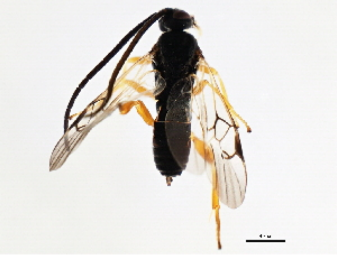

BIOUG01028-C03 [Dorsal]
Microgaster jft13
Family: Braconidae
BIN URI: BOLD:ACF5282

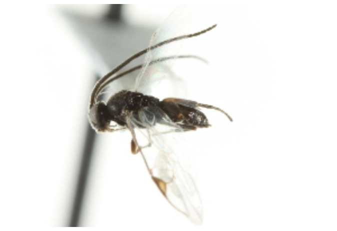

07PROBE-24235 [Lateral]
Pholetesor ornigis
Family: Braconidae
BIN URI: BOLD:AAB0520

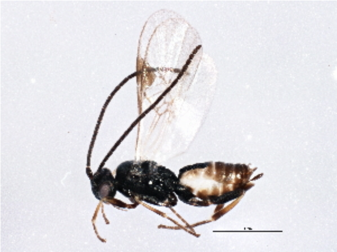

BIOUG02712-F08 [Lateral]
Pholetesor
Family: Braconidae
BIN URI: BOLD:AAD5198

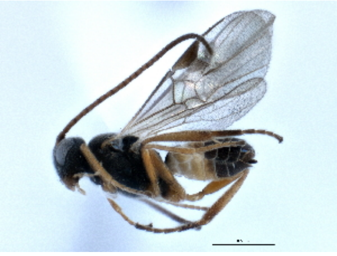

BIOUG08597-F12 [Lateral]
Pholetesor sp.
Family: Braconidae
BIN URI: BOLD:AAE0349

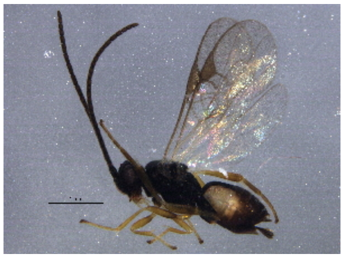

BIOUG08526-G06 [Lateral]
Pholetesor
Family: Braconidae
BIN URI: BOLD:ACK5660

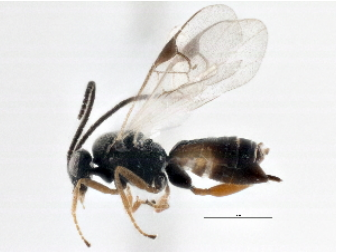

BIOUG06168-G03 [Lateral]
Cotesia sp.
Family: Braconidae
BIN URI: BOLD:ABZ3751

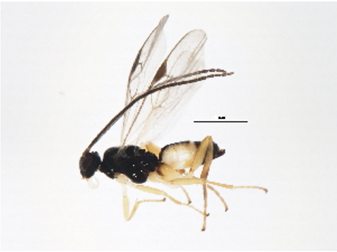

ASGLE2-0132 [Lateral]

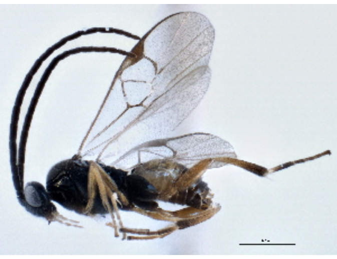

BIOUG03720-F07 [Lateral]

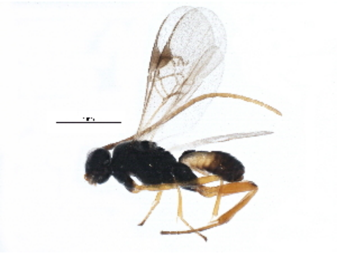

BIOUG17215-B11 [Lateral]

IMAGE NOT AVAILABLE

BIOUG22862-H06
Cotesia xylina

Protapanteles
Family: Braconidae
BIN URI: BOLD:AAA4782

Dolichogenidea sp.
Family: Braconidae
BIN URI: BOLD:AAB0096

Cotesia
Family: Braconidae
BIN URI: BOLD:AAA9386

Family: Braconidae

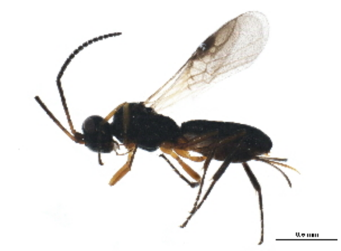

BIOUG24008-C07 [Lateral]
Braconidae
Family: Braconidae

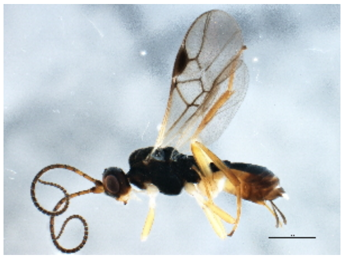

BIOUG02736-C01 [Lateral]
Aliolus
Family: Braconidae
BIN URI: BOLD:AAU8214

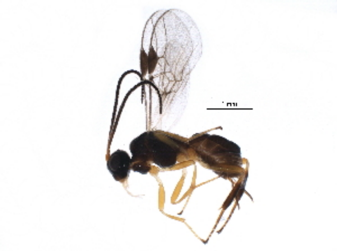

BIOUG24009-E01 [Lateral]
Braconidae
Family: Braconidae

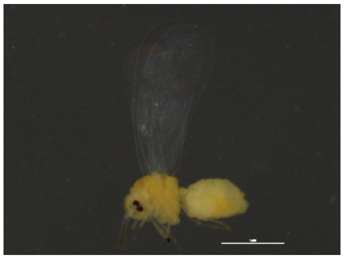

BIOUG00999-A09 [Lateral]
Aleyrodidae
Family: Aleyrodidae
BIN URI: BOLD:AAZ8500

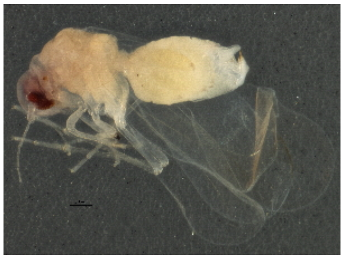

BIOUG01797-G01 [Lateral]
Aleyrodidae
Family: Aleyrodidae
BIN URI: BOLD:AAZ8501

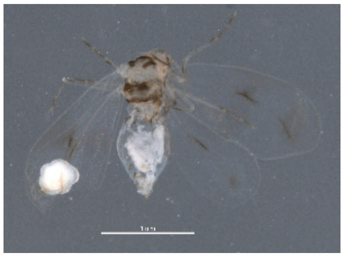

BIOUG07277-A08 [Dorsal]
Aleyrodes
Family: Aleyrodidae
BIN URI: BOLD:ABW2915

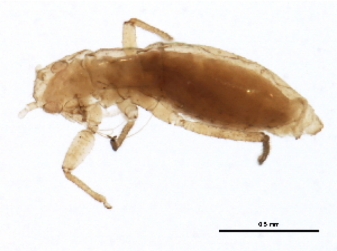

BIOUG01308-B09 [Lateral]
Essigella
Family: Aphididae
BIN URI: BOLD:AAI4969

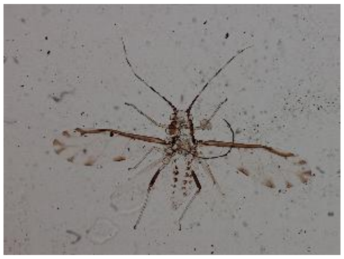

CNC#HEM006905 [Dorsal]
Eucallipterus tiliae
Family: Aphididae
BIN URI: BOLD:AAD0131

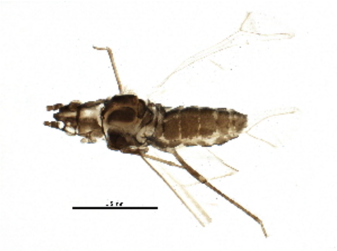

BIOUG22082-A11 [Lateral]
Saltusaphidinae
Family: Aphididae
BIN URI: BOLD:ACV4116

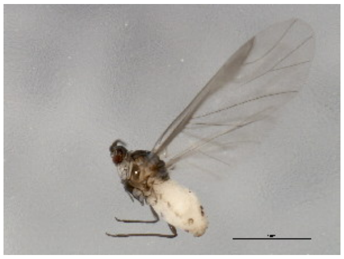

TDWG-1098 [Lateral]
Eriosoma americanum
Family: Aphididae
BIN URI: BOLD:AAD7955

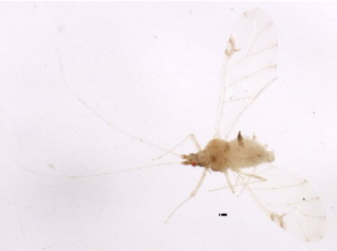

BIOUG00658-H10 [Dorsal]
Drepanaphis
Family: Aphididae
BIN URI: BOLD:AAI6141

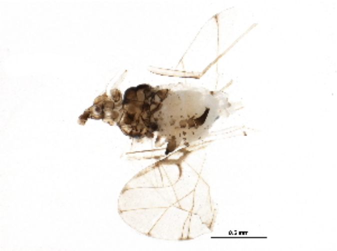

BIOUG22574-A07 [Lateral]
Drepanaphis
Family: Aphididae
BIN URI: BOLD:ABY0945

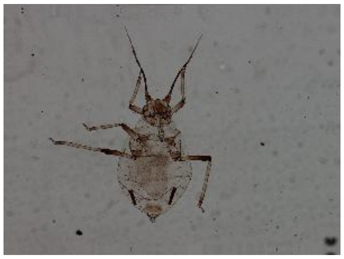

CNC#HEM012135 [Dorsal]
Schizaphis scirpicola
Family: Aphididae
BIN URI: BOLD:AAD1238

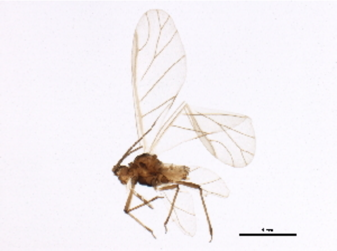

BIOUG01652-F10 [Lateral]
Rhopalosiphum nymphaeae
Family: Aphididae
BIN URI: BOLD:AAE3554

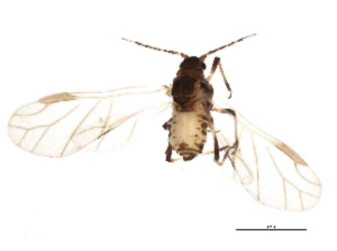

BIOUG00856-B05 [Dorsal]
Aphis middletonii
Family: Aphididae
BIN URI: BOLD:AAB6817

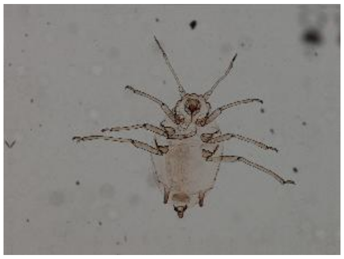

CNC#HEM049387 [Dorsal]
Aphis sp. B rgf-2008
Family: Aphididae
BIN URI: BOLD:AAA3070

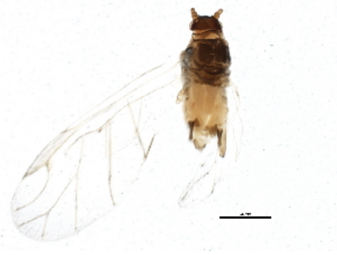

BIOUG01308-E09 [Dorsal]
Aphis glycines
Family: Aphididae
BIN URI: BOLD:AAB7938

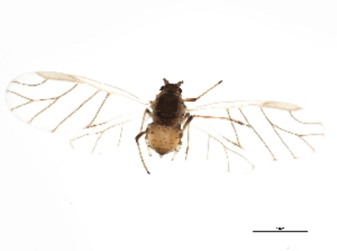

BIOUG00856-C11 [Dorsal]
Lipaphis pseudobrassicae
Family: Aphididae
BIN URI: BOLD:AAD9153

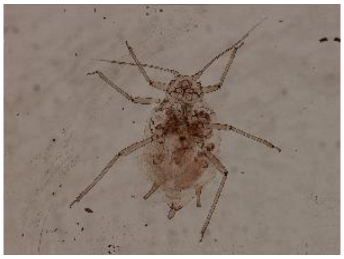

CNC#HEM007605 [Dorsal]
Aphis nasturtii
Family: Aphididae
BIN URI: BOLD:AAC1374

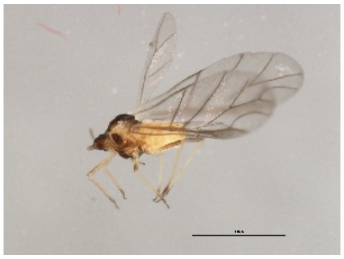

BIOUG00999-C01 [Lateral]
Aphis rubicola
Family: Aphididae
BIN URI: BOLD:AAF7621

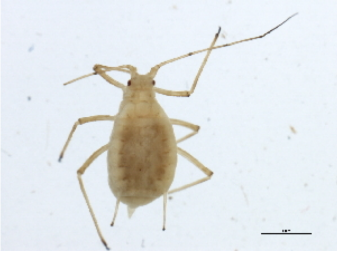

10BBCHEM-1209 [Dorsal]
Acyrthosiphon pisum
Family: Aphididae
BIN URI: BOLD:AAB1787

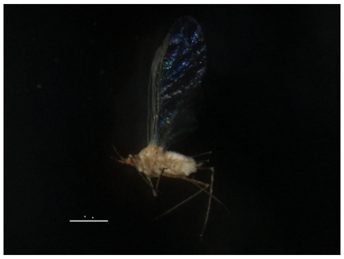

BIOUG00783-B08 [Lateral]
Hemiptera

BIN URI: BOLD:AAF3206

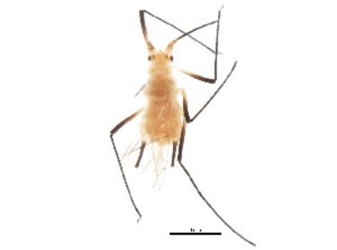

BIOUG01012-A03 [Dorsal]
Uroleucon caligatum
Family: Aphididae
BIN URI: BOLD:AAF8501

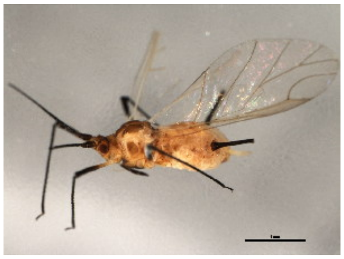

TDWG-1192 [Lateral]
Uroleucon nigrotibium
Family: Aphididae
BIN URI: BOLD:ABY4768

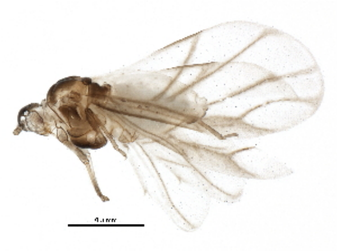

BIOUG22082-C04 [Lateral]
Melaphis rhois
Family: Aphididae
BIN URI: BOLD:AAA2079

IMAGE NOT AVAILABLE

BIOUG22082-D03
Melaphis
Family: Aphididae

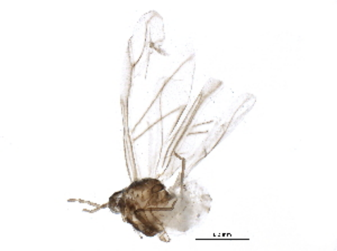

BIOUG22082-A03 [Lateral]
Melaphis
Family: Aphididae
BIN URI: BOLD:ABZ6353

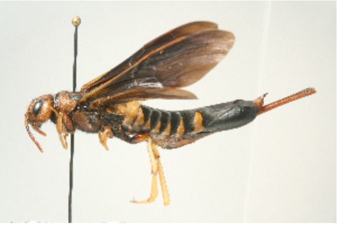

SIR 050 [Lateral]
Tremex columba
Family: Siricidae
BIN URI: BOLD:AAC2293

IMAGE NOT AVAILABLE

BIOUG24015-A03
Dorylaimida

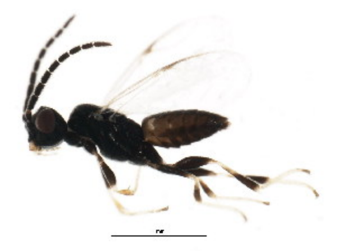

BIOUG01252-E04 [Lateral]
Gonatopus
Family: Dryinidae
BIN URI: BOLD:ABA8070

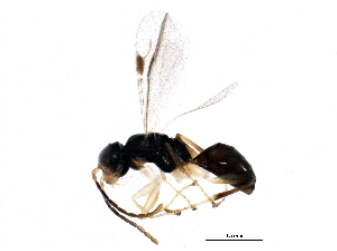

BIOUG22458-B05 [Lateral]
Dryinidae
Family: Dryinidae
BIN URI: BOLD:ACA7378

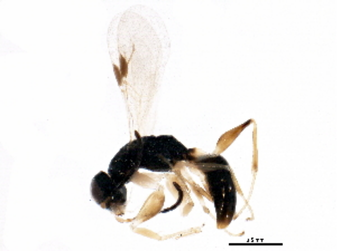

BIOUG22862-H09 [Lateral]
Anteon
Family: Dryinidae
BIN URI: BOLD:ACV4994

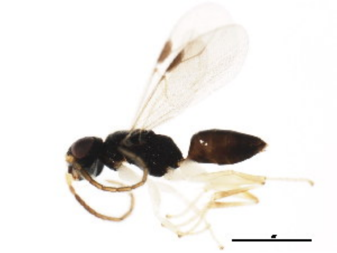

BIOUG01049-H10 [Lateral]
Anteoninae
Family: Dryinidae
BIN URI: BOLD:AAU8737

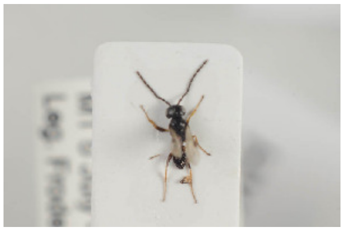

HYMNI800 [Dorsal]
Anteon cameroni
Family: Dryinidae
BIN URI: BOLD:AAU8713

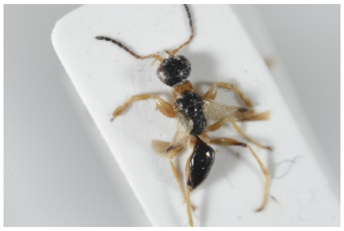

HYMNI396 [Dorsal]
Anteon gaullei
Family: Dryinidae
BIN URI: BOLD:ABA7952

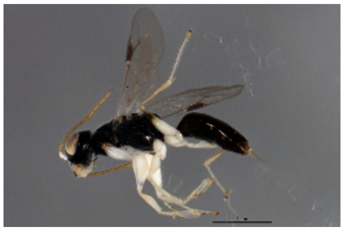

09BBEHY-1628 [Lateral]
Dryinidae
Family: Dryinidae
BIN URI: BOLD:AAM7445

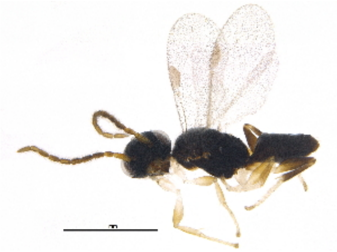

BIOUG21129-E05 [Lateral]
Hymenoptera

BIN URI: BOLD:ACU6678

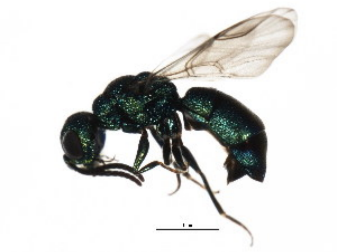

BIOUG01252-H04 [Lateral]
Pseudomalus auratus
Family: Chrysididae
BIN URI: BOLD:AAL7396

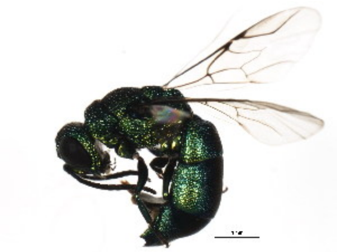

BIOUG01113-G07 [Lateral]
Chrysis
Family: Chrysididae
BIN URI: BOLD:ABA5910

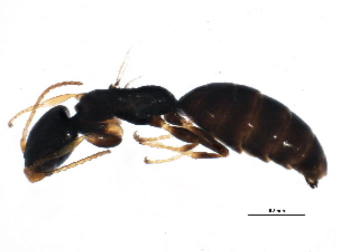

BIOUG08152-D02 [Lateral]
Bethylidae
Family: Bethylidae
BIN URI: BOLD:ACJ5404

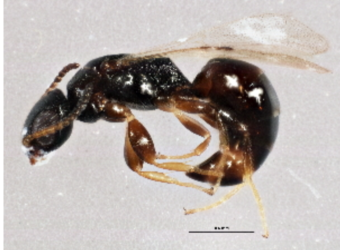

BIOUG02772-D11 [Lateral]
Goniozus
Family: Bethylidae
BIN URI: BOLD:ABW3217

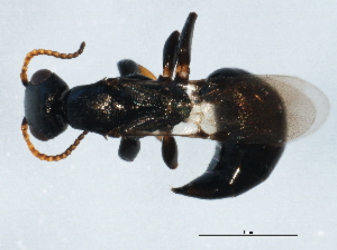

BIOUG01596-A01 [Dorsal]
Bethylidae
Family: Bethylidae
BIN URI: BOLD:ABV2666

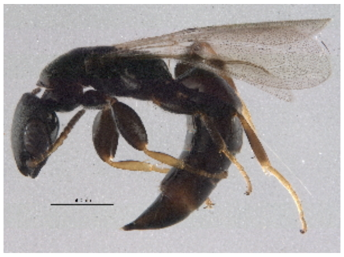

BIOUG06546-D12 [Lateral]
Bethylidae
Family: Bethylidae
BIN URI: BOLD:ACI4207

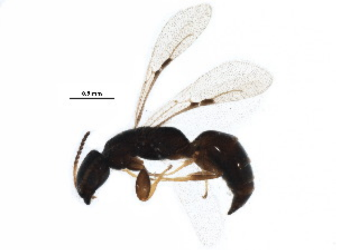

BIOUG22294-A01 [Lateral]
Bethylidae
Family: Bethylidae
BIN URI: BOLD:ABW3215

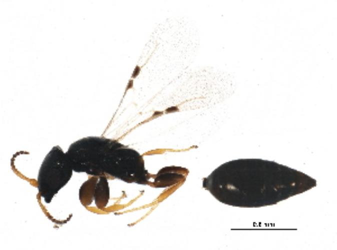

BIOUG24015-E07 [Lateral]
Bethylidae
Family: Bethylidae

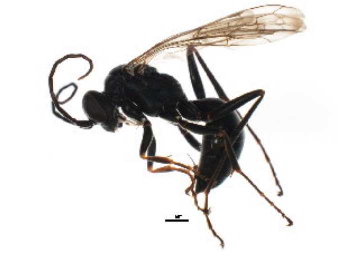

BIOUG01076-F03 [Lateral]
Pompilidae
Family: Pompilidae
BIN URI: BOLD:AAN7892

IMAGE NOT AVAILABLE

BIOUG22570-G12
Pompilidae
Family: Pompilidae

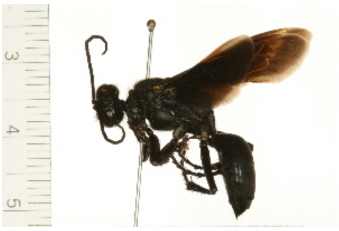

08MZPP-037 [Lateral]
Sphecidae
Family: Sphecidae
BIN URI: BOLD:AAG7748

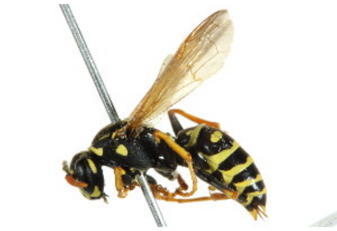

BIOUG01081-A10 [Lateral]
Polistes dominula
Family: Vespidae
BIN URI: BOLD:AAB7105

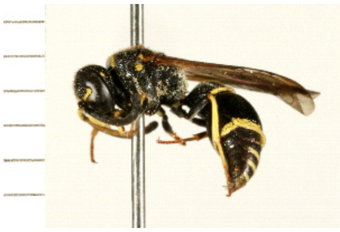

HYMN 0136.02 [Lateral]
Parancistrocerus pensylvanicus
Family: Vespidae
BIN URI: BOLD:AAD5749

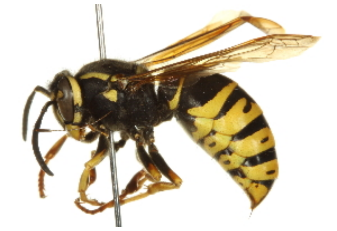

BIOUG07541-H03 [Lateral]
Vespula vidua
Family: Vespidae
BIN URI: BOLD:AAN8137

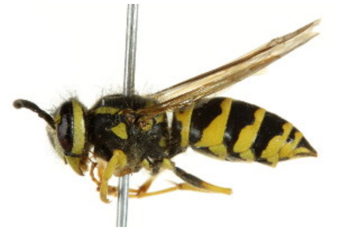

BIOUG01081-H11 [Lateral]
Vespula maculifrons
Family: Vespidae
BIN URI: BOLD:AAD5593

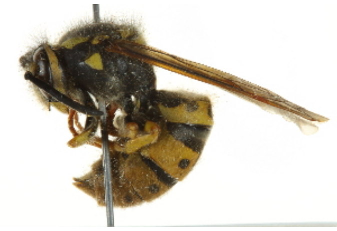

CCDB-15177-H02 [Lateral]
Vespula germanica
Family: Vespidae
BIN URI: BOLD:AAG9055

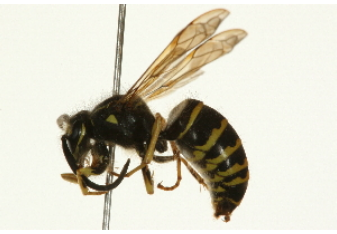

09BBEHY-0045 [Lateral]
Dolichovespula arenaria
Family: Vespidae
BIN URI: BOLD:ACE9710

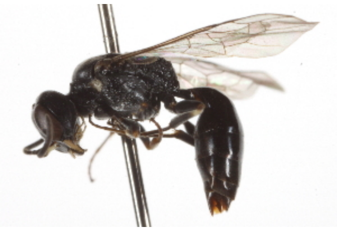

BIOUG07541-G11 [Lateral]
Passaloecus cuspidatus
Family: Crabronidae
BIN URI: BOLD:AAG7762

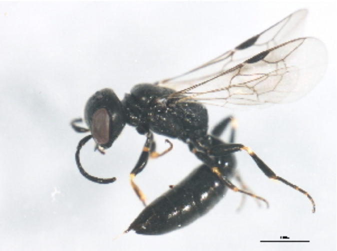

BIOUG01029-G03 [Lateral]
Passaloecus singularis
Family: Crabronidae
BIN URI: BOLD:AAM4998

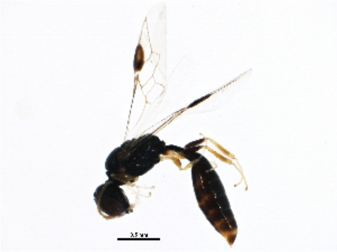

BIOUG23188-B12 [Lateral]
Stigmus
Family: Crabronidae
BIN URI: BOLD:ACV6895

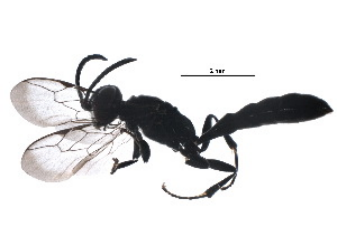

BIOUG22567-C08 [Lateral]
Trypoxylon frigidum
Family: Crabronidae
BIN URI: BOLD:AAG3193

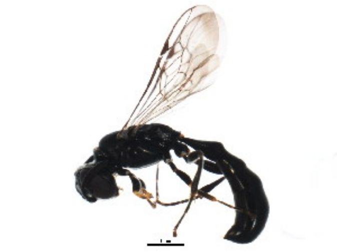

BIOUG01259-C03 [Lateral]
Trypoxylon kostylevi
Family: Crabronidae
BIN URI: BOLD:AAN3726

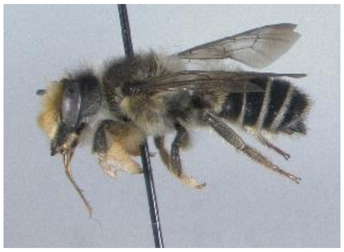

Bee13 [Lateral]
Megachile pugnata
Family: Megachilidae
BIN URI: BOLD:AAC8596

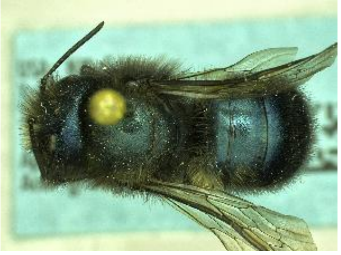
Cave 2 [Dorsal]
Osmia lignaria
Family: Megachilidae
BIN URI: BOLD:AAE5495

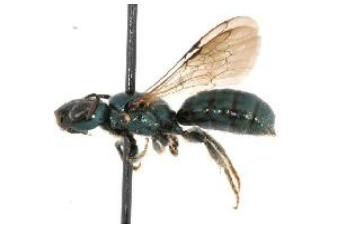
GUE06-BEES-166 [Lateral]
Ceratina calcarata
Family: Apidae
BIN URI: BOLD:AAA2368

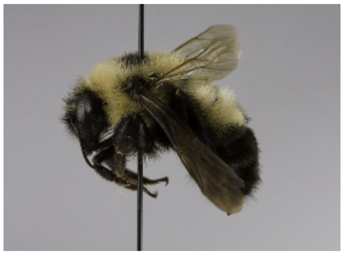
CCDB-01556 A11 [Lateral]
Bombus rufocinctus
Family: Apidae
BIN URI: BOLD:AAB0152

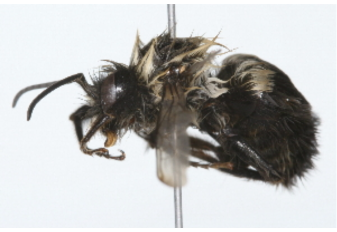
08TTML-0290 [Lateral]
Bombus impatiens
Family: Apidae
BIN URI: BOLD:ABZ2516

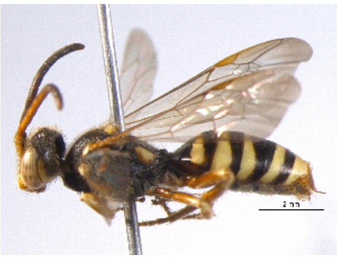
BIOUG05619-E09 [Lateral]
Nomada subrutila
Family: Apidae
BIN URI: BOLD:AAC5044

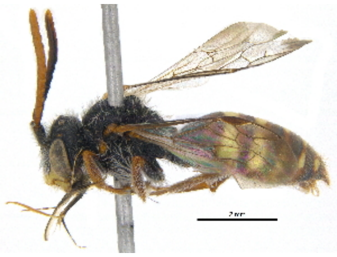
BIOUG21484-F07 [Lateral]
Nomada bella
Family: Apidae
BIN URI: BOLD:ABZ2527

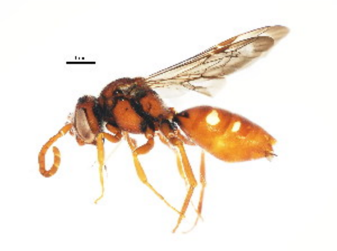
BIOUG00801-D11 [Lateral]
Nomada sp. 5
Family: Apidae
BIN URI: BOLD:AAI3547

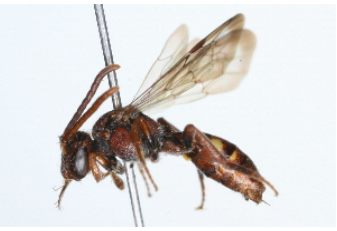
09BBHYM-318 [Lateral]
Nomada pygmaea
Family: Apidae
BIN URI: BOLD:ABZ6834

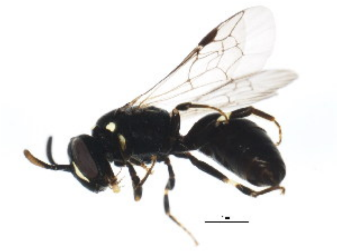
BIOUG01034-H04 [Lateral]
Hylaeus modestus
Family: Colletidae
BIN URI: BOLD:AAB2744

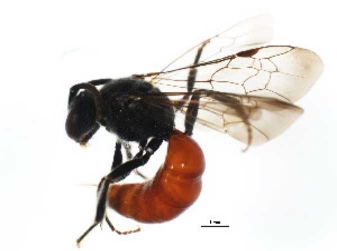
BIOUG01030-D08 [Lateral]
Sphecodes ranunculi
Family: Halictidae
BIN URI: BOLD:AAC7655

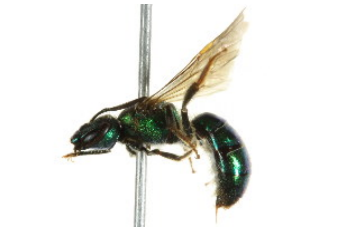
BIOUG01081-E03 [Lateral]
Augochlora pura
Family: Halictidae
BIN URI: BOLD:AAD6445

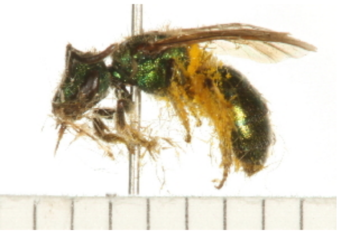
08BBHYM-0697 [Lateral]
Augochlorella
Family: Halictidae
BIN URI: BOLD:AAG0449

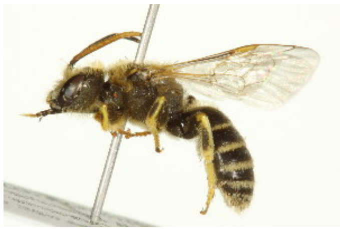
TDWG-0138 [Lateral]
Halictus ligatus
Family: Halictidae
BIN URI: BOLD:AAA1257

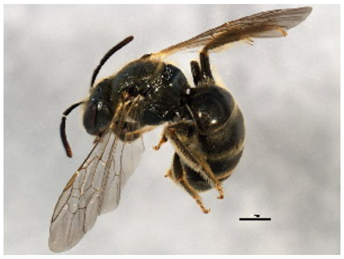
ASGLE-1011 [Lateral]
Halictus confusus
Family: Halictidae
BIN URI: BOLD:AAA2139

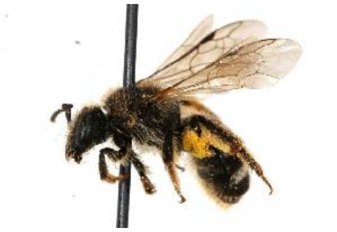
GUE06-BEES-271 [Lateral]
Lasioglossum leucozonium
Family: Halictidae
BIN URI: BOLD:AAA2322

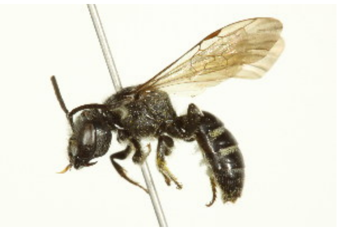
TDWG-0154 [Lateral]
Lasioglossum coriaceum
Family: Halictidae
BIN URI: BOLD:AAB7007

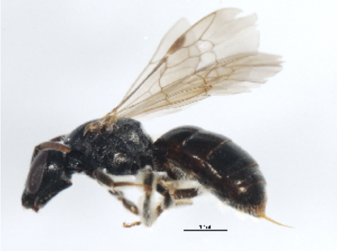
09BBEHY-0213 [Lateral]
Lasioglossum divergens
Family: Halictidae
BIN URI: BOLD:AAB8845

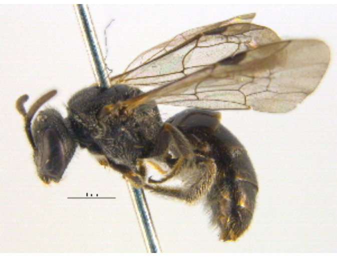
BIOUG03373-F07 [Lateral]
Lasioglossum birkmanni
Family: Halictidae
BIN URI: BOLD:AAC8293

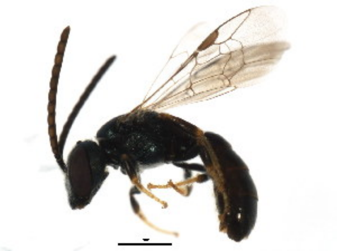
BIOUG01285-B10 [Lateral]
Lasioglossum anomalum
Family: Halictidae
BIN URI: BOLD:AAA7868

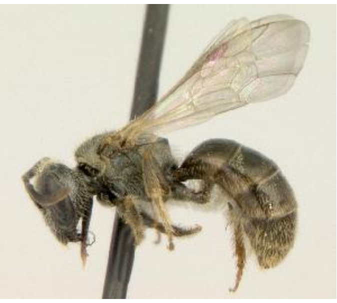
DIAL0294F01-PA [Lateral]
Lasioglossum weemsi
Family: Halictidae

BIN URI: BOLD:AAA3782

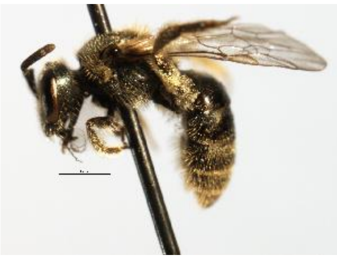

Cp 2007 - 103 [Lateral]
Lasioglossum
Family: Halictidae
BIN URI: BOLD:ABZ0652

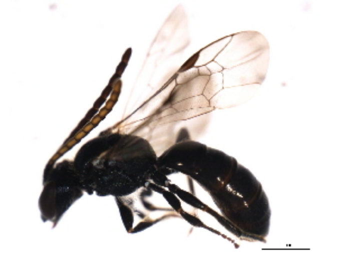

09BBEHY-1826 [Lateral]
Lasioglossum versans
Family: Halictidae
BIN URI: BOLD:ABZ6180

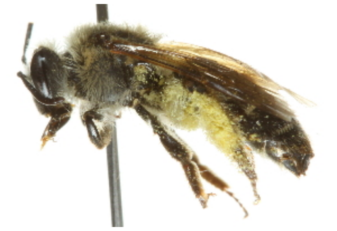

10BBCHY-3283 [Lateral]
Andrena barbilabris
Family: Andrenidae
BIN URI: BOLD:AAB4998

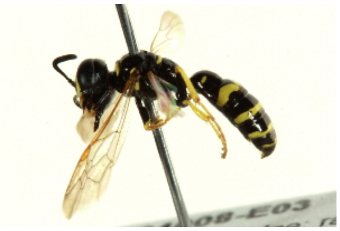

BIOUG24008-E03 [Lateral]
Philanthus
Family: Crabronidae

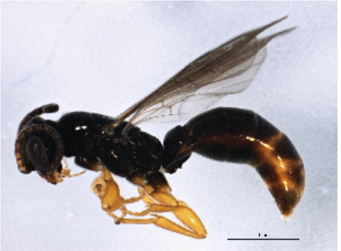

BIOUG01839-E07 [Lateral]
Xiphydriidae
Family: Xiphydriidae
BIN URI: BOLD:ABY1179

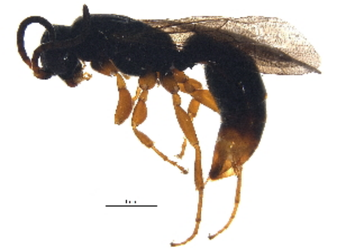

BIOUG08748-F07 [Lateral]
Hymenoptera

BIN URI: BOLD:ACL5424

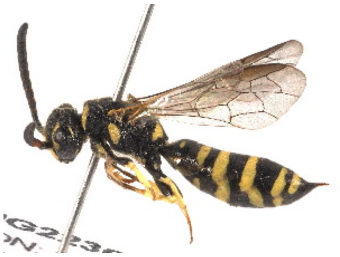

BIOUG22360-G05 [Lateral]
Sapyga centrata
Family: Sapygidae
BIN URI: BOLD:ACL7820

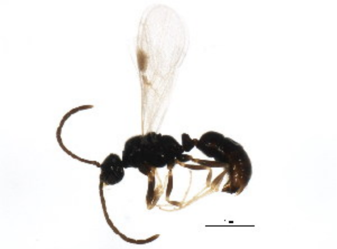

BIOUG01285-G12 [Lateral]
Ponera pennsylvanica
Family: Formicidae
BIN URI: BOLD:AAF0443

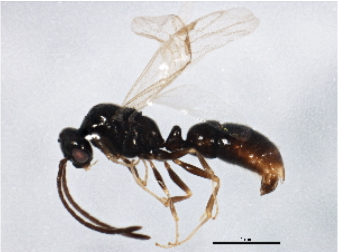

BIOUG01795-G10 [Lateral]
Hypoponera
Family: Formicidae
BIN URI: BOLD:ACU4160

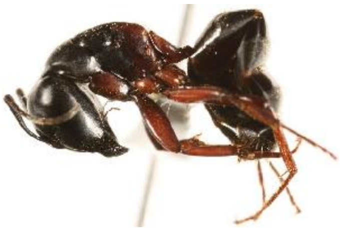

CHU06-ANT-057 [Lateral]
Camponotus herculeanus
Family: Formicidae
BIN URI: BOLD:AAA2372

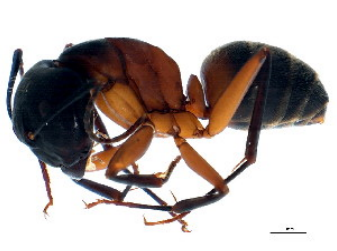

TDWG-0455 [Lateral]
Camponotus pennsylvanicus
Family: Formicidae
BIN URI: BOLD:AAA9461

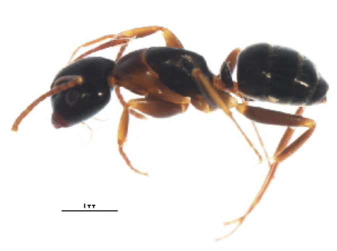

PCPP10-0474 [Lateral]
Camponotus americanus
Family: Formicidae
BIN URI: BOLD:AAD4432

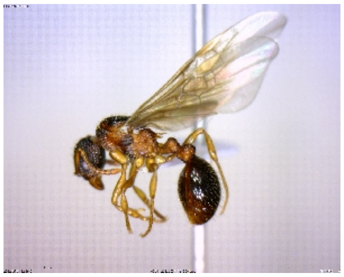

BIOUG02018-D10 [Lateral]
Myrmica detritinodis
Family: Formicidae
BIN URI: BOLD:AAA1841

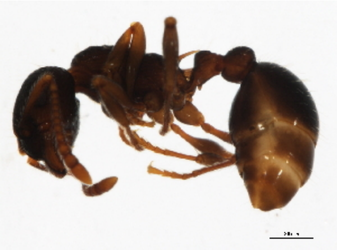

09BBEFO-0186 [Lateral]
Myrmica cf. fracticornis
Family: Formicidae
BIN URI: BOLD:AAA1863

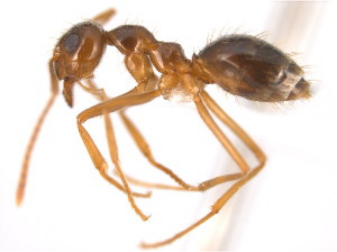

09GUANTS-304 [Lateral]
Prenolepis imparis
Family: Formicidae
BIN URI: BOLD:AAC1302

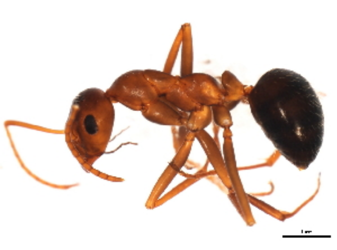

10BBUFO-0221 [Lateral]
Formica
Family: Formicidae
BIN URI: BOLD:AAA1467

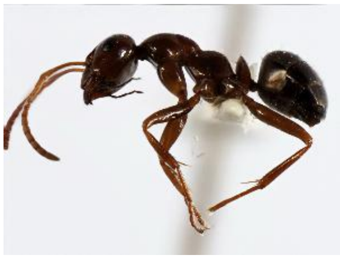

MAS036-07 [Lateral]
Formica
Family: Formicidae
BIN URI: BOLD:AAE0406

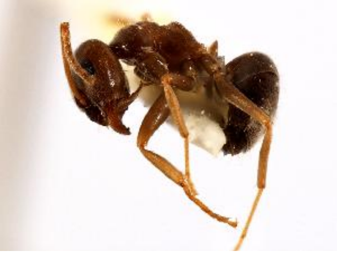

MAS206-07 [Lateral]
Lasius
Family: Formicidae
BIN URI: BOLD:AAA9048

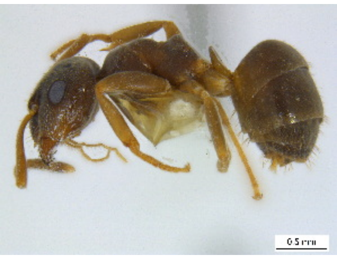

BIOUG06760-F01 [Lateral]
Lasius alienus
Family: Formicidae
BIN URI: BOLD:AAA9049

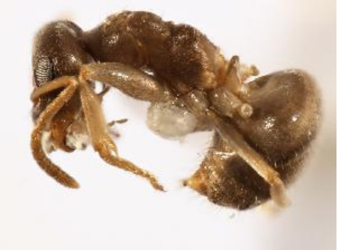

MAS145-07 [Lateral]
Lasius neoniger
Family: Formicidae
BIN URI: BOLD:AAB9126

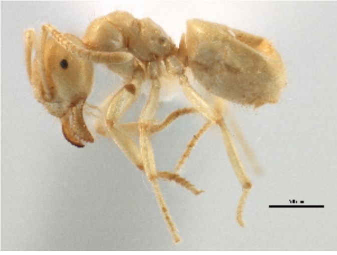

CCDB-21409-G07 [Lateral]
Lasius nearcticus
Family: Formicidae
BIN URI: BOLD:AAD1528

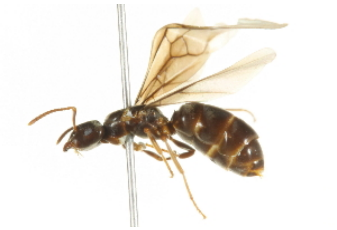

BIOUG00835-F11 [Lateral]
Lasius umbratus
Family: Formicidae
BIN URI: BOLD:AAE1553

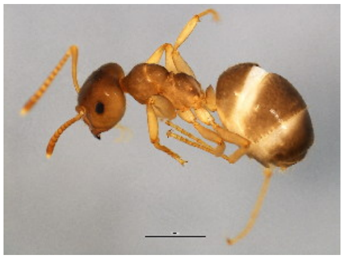

ASGLE-0021 [Lateral]
Lasius
Family: Formicidae
BIN URI: BOLD:AAI1292

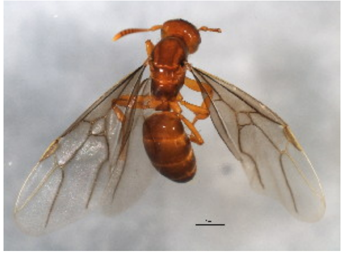

ASGLE-0014 [Dorsal]
Lasius
Family: Formicidae
BIN URI: BOLD:AAF0890

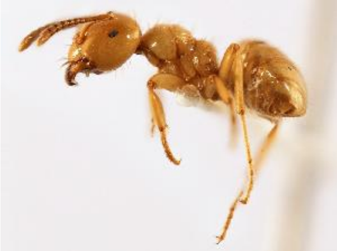

MAS186-07 [Lateral]
Lasius
Family: Formicidae
BIN URI: BOLD:ABY9254

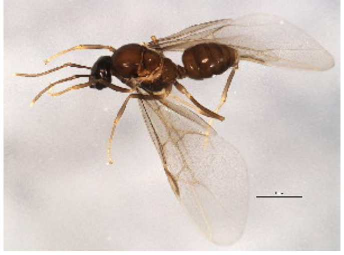

ASGLE-0071 [Lateral]
Lasius
Family: Formicidae
BIN URI: BOLD:ACW1526

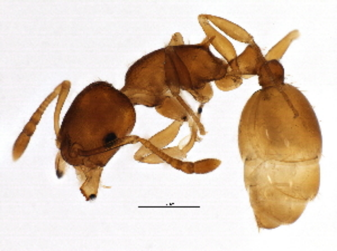

BIOUG11767-G08 [Lateral]
Temnothorax
Family: Formicidae
BIN URI: BOLD:AAG0685

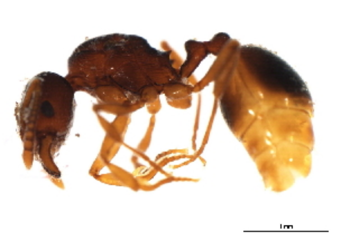

09GUANTS-208 [Lateral]
Stenamma
Family: Formicidae
BIN URI: BOLD:AAH7068

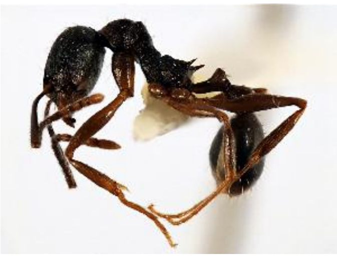

MAS205-07 [Lateral]
Aphaenogaster
Family: Formicidae
BIN URI: BOLD:AAD1929

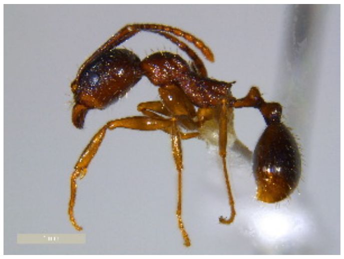

JJ-O4-01 [Lateral]
Aphaenogaster rudis-texana
Family: Formicidae
BIN URI: BOLD:AAD1927

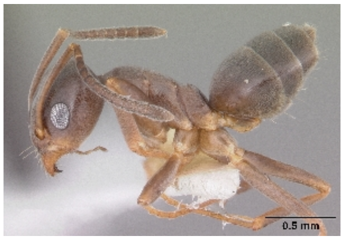

BIOUG01462-E07 [Lateral]
Tapinoma sessile
Family: Formicidae
BIN URI: BOLD:AAA3898

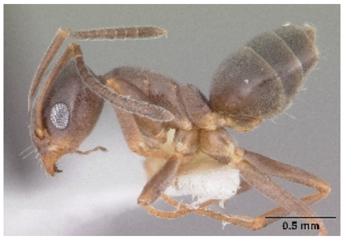

BIOUG01462-H05 [Lateral]
Tapinoma sessile
Family: Formicidae
BIN URI: BOLD:AAA3900

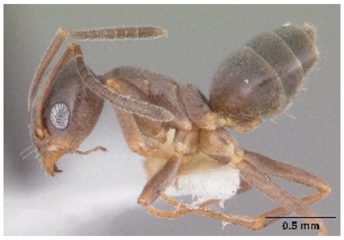

BIOUG01809-B01 [Lateral]
Tapinoma sessile
Family: Formicidae
BIN URI: BOLD:AAA3893

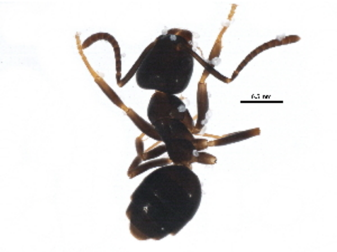

BIOUG24003-A02 [Dorsal]
Tapinoma
Family: Formicidae

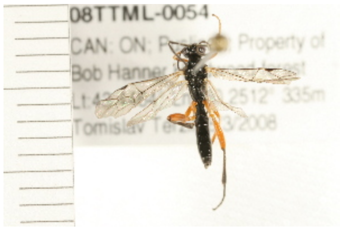

08TTML-0054 [Dorsal]
Cylloceria melancholica
Family: Ichneumonidae
BIN URI: BOLD:AAG7628

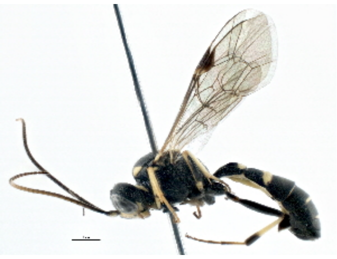

BIOUG04179-B07 [Lateral]
Exenterus confusus
Family: Ichneumonidae
BIN URI: BOLD:AAL5084

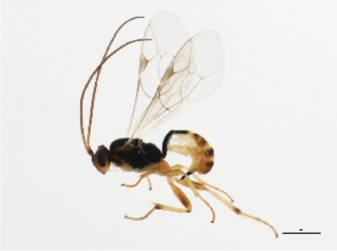

BIOUG00764-C10 [Lateral]
Mesochorus
Family: Ichneumonidae
BIN URI: BOLD:AAM7412

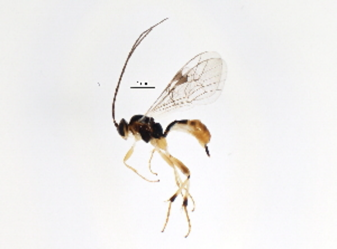

BIOUG00845-D06 [Lateral]
Mesochorus suomiensis
Family: Ichneumonidae
BIN URI: BOLD:AAZ1979

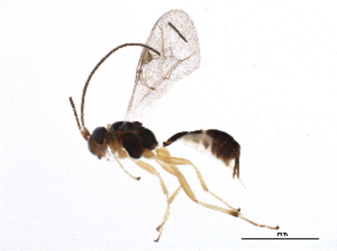

BIOUG10225-H05 [Lateral]
Mesochorus
Family: Ichneumonidae
BIN URI: BOLD:ACG5789

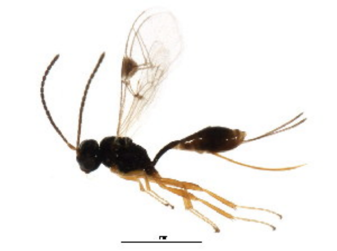

BIOUG01285-C06 [Lateral]
Tersilochinae
Family: Ichneumonidae
BIN URI: BOLD:ABA9842

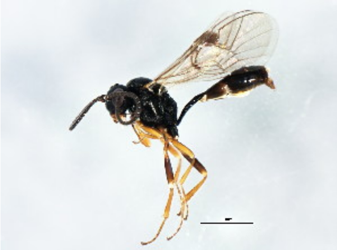

BIOUG00786-D09 [Lateral]
Tersilochinae
Family: Ichneumonidae
BIN URI: BOLD:AAU8481

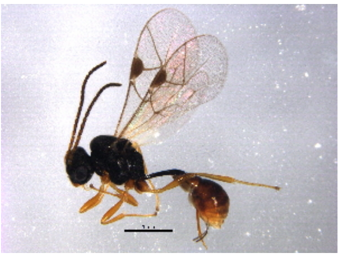

BIOUG10906-C03 [Lateral]
Tersilochinae
Family: Ichneumonidae
BIN URI: BOLD:ACL9152

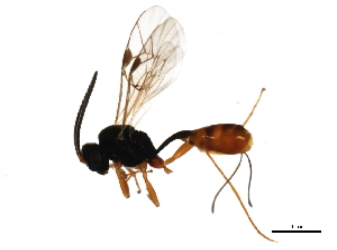

10PHMAL-1889 [Lateral]
Tersilochinae
Family: Ichneumonidae
BIN URI: BOLD:AAU8328

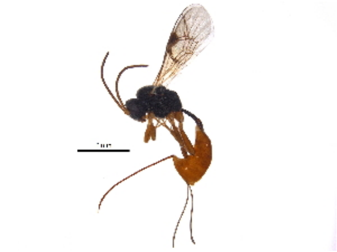

BIOUG22565-B11 [Lateral]
Tersilochinae
Family: Ichneumonidae
BIN URI: BOLD:ACV2273

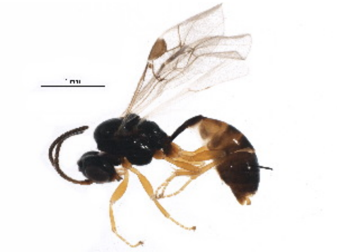

BIOUG22421-D08 [Lateral]
Tersilochinae
Family: Ichneumonidae
BIN URI: BOLD:ACV3286

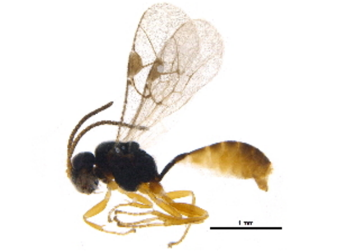

BIOUG22287-B01 [Lateral]
Tersilochinae
Family: Ichneumonidae
BIN URI: BOLD:ABA6274

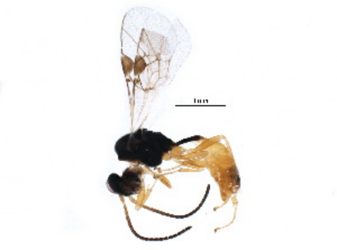

BIOUG22470-A03 [Lateral]
Ichneumonidae
Family: Ichneumonidae
BIN URI: BOLD:ACH0608

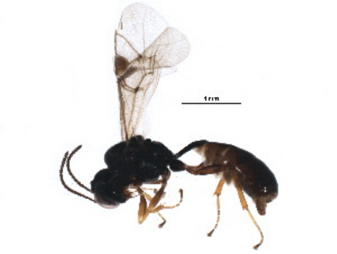

BIOUG22470-B05 [Lateral]
Ichneumonidae
Family: Ichneumonidae
BIN URI: BOLD:ACV3460

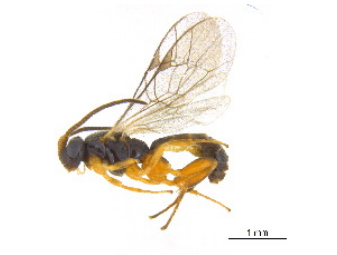

BIOUG06752-D09 [Lateral]
Hymenoptera

BIN URI: BOLD:AAM9125

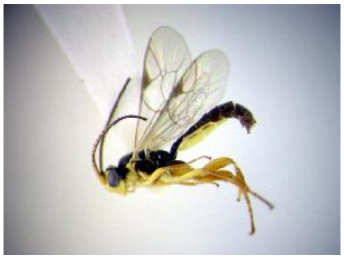

06-PROBE-3249 [Lateral]
Orthocentrus jft09
Family: Ichneumonidae
BIN URI: BOLD:AAB1188

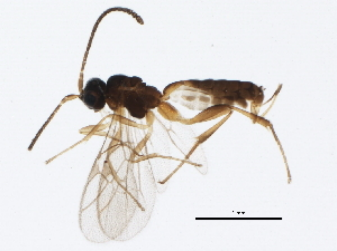

10PHMAL-3281 [Lateral]
Stenomacrus
Family: Ichneumonidae
BIN URI: BOLD:AAU8210

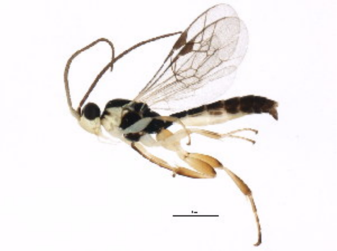

BIOUG00914-G06 [Lateral]
Hymenoptera

BIN URI: BOLD:AAU8604

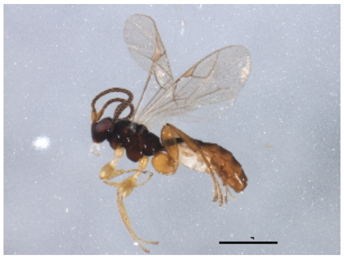

BIOUG01572-C03 [Lateral]
Stenomacrus jft14
Family: Ichneumonidae
BIN URI: BOLD:AAJ5372

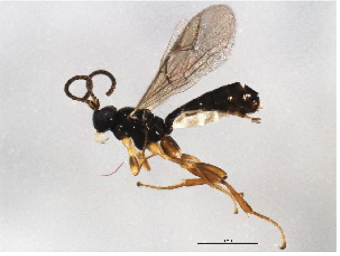

ASGLE-0532 [Lateral]
Hymenoptera

BIN URI: BOLD:AAM7494

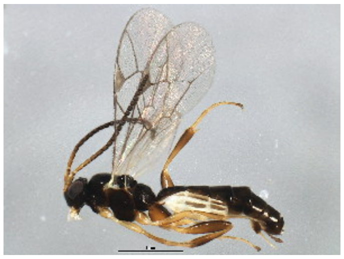

ASGLE-0907 [Lateral]
Hymenoptera

BIN URI: BOLD:AAP6689

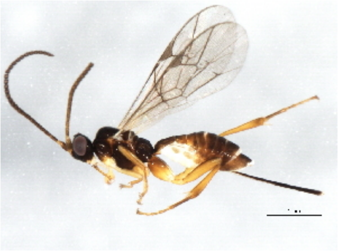

ASGLE2-0197 [Lateral]
Hymenoptera

BIN URI: BOLD:AAU8746

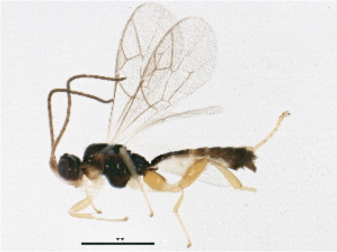

BIOUG03770-C03 [Lateral]
Orthocentrinae
Family: Ichneumonidae
BIN URI: BOLD:ACB2448

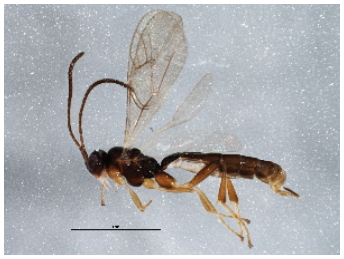

BIOUG04435-D03 [Lateral]
Leipaulus
Family: Ichneumonidae
BIN URI: BOLD:ACC8246

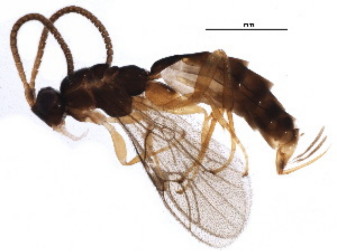

BIOUG22867-B11 [Lateral]
Orthocentrinae
Family: Ichneumonidae
BIN URI: BOLD:AAG0973

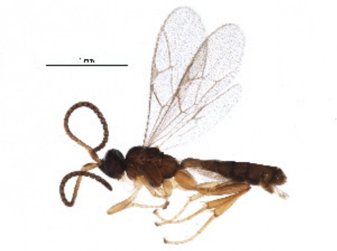

BIOUG22453-F03 [Lateral]
Orthocentrinae
Family: Ichneumonidae
BIN URI: BOLD:ACD9088

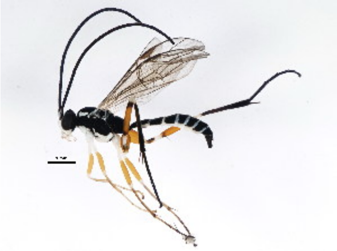

BIOUG00801-H02 [Lateral]
Phytodietus vulgaris
Family: Ichneumonidae
BIN URI: BOLD:AAG0379

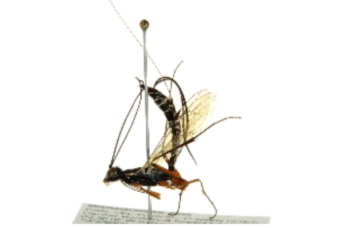

BIOUG00858-A08 [Lateral]
Podoschistus vittifrons
Family: Ichneumonidae
BIN URI: BOLD:AAL0380

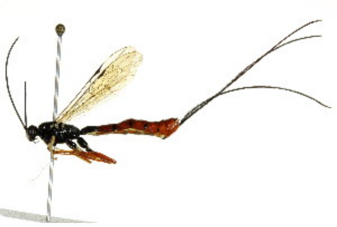

BIOUG01023-A11 [Lateral]
Dolichomitus irritator
Family: Ichneumonidae
BIN URI: BOLD:AAU8680

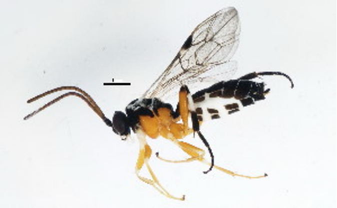

BIOUG00823-G10 [Lateral]
Diplazon
Family: Ichneumonidae
BIN URI: BOLD:AAD1879

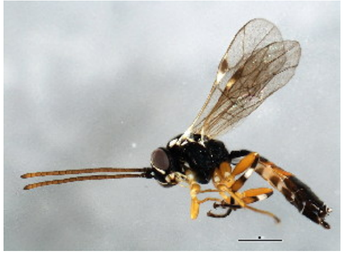

ASGLE-0955 [Lateral]
Hymenoptera

BIN URI: BOLD:AAD4214

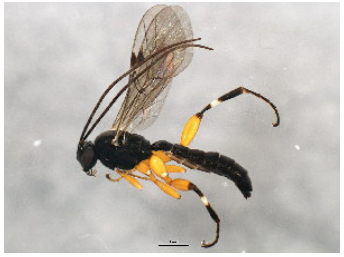

ASGLE-1025 [Lateral]
Hymenoptera

BIN URI: BOLD:AAD5194

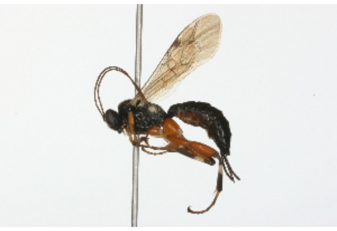

08TTML-0408 [Lateral]
Pimpla
Family: Ichneumonidae
BIN URI: BOLD:AAG0387

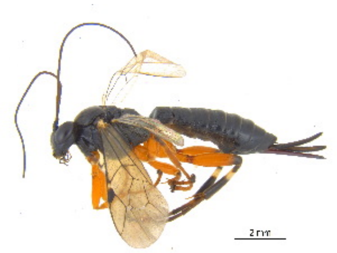

BIOUG06752-D05 [Lateral]
Hymenoptera

BIN URI: BOLD:AAG7634

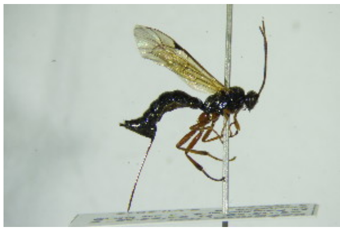

BC ZSM HYM 20692 [Lateral]
Endromopoda detrita
Family: Ichneumonidae
BIN URI: BOLD:AAD5195

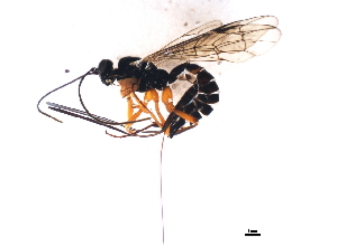

09BBEHY-1405 [Lateral]
Coleocentrus
Family: Ichneumonidae
BIN URI: BOLD:AAJ6986

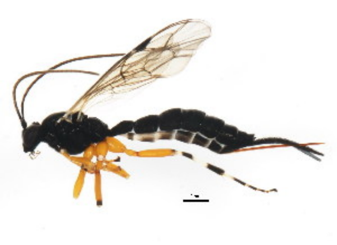

BIOUG01032-B03 [Lateral]
Iseropus stercorator
Family: Ichneumonidae
BIN URI: BOLD:AAO2094

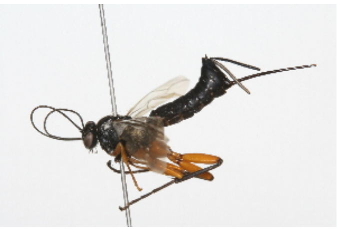

08TTML-0369 [Lateral]
Pimpla pedalis
Family: Ichneumonidae
BIN URI: BOLD:AAD5192

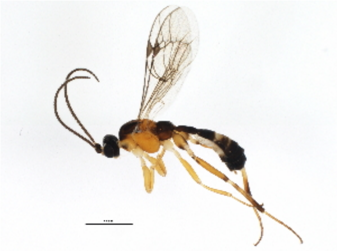

BIOUG03930-A04 [Lateral]
Orthocentrinae
Family: Ichneumonidae
BIN URI: BOLD:AAG0953

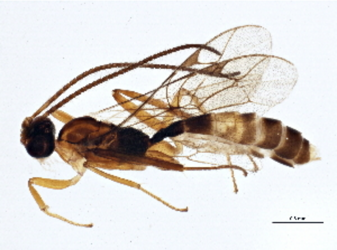

BIOUG03336-F06 [Lateral]
Megastylus
Family: Ichneumonidae
BIN URI: BOLD:ACE4233

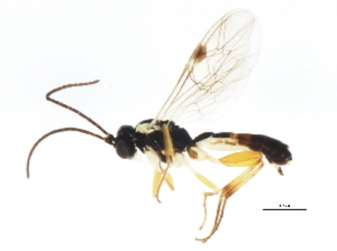

PCPP10-0497 [Lateral]
Syrphoctonus
Family: Ichneumonidae
BIN URI: BOLD:AAU8212

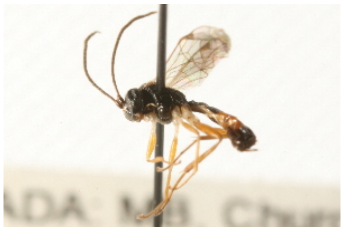

07PROBE-23120 [Lateral]
Sussaba cognata
Family: Ichneumonidae
BIN URI: BOLD:AAH1764

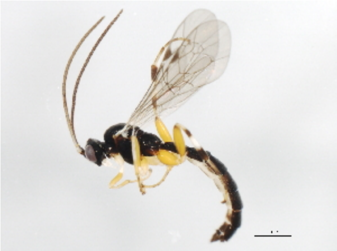

09BBHYM-646 [Lateral]
Zaglyptus varipes
Family: Ichneumonidae
BIN URI: BOLD:AAG0382

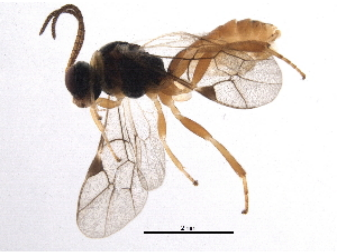

BIOUG10971-G12 [Lateral]
Tryphoninae
Family: Ichneumonidae
BIN URI: BOLD:AAU8749

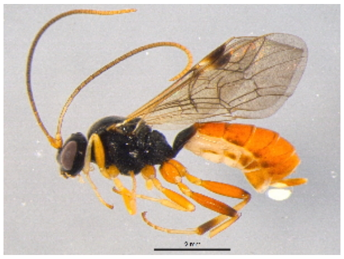

BIOUG02861-A09 [Lateral]
Tryphoninae
Family: Ichneumonidae
BIN URI: BOLD:AAM7520

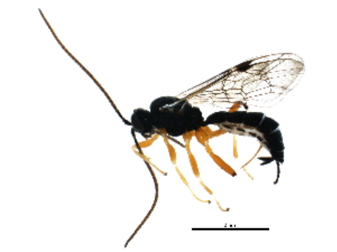

BIOUG22569-G06 [Lateral]
Polyblastus
Family: Ichneumonidae
BIN URI: BOLD:ACF4348

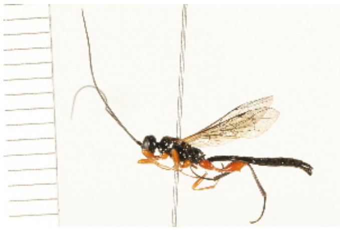

08TTML-0244 [Lateral]
Odontocolon
Family: Ichneumonidae
BIN URI: BOLD:AAG7690

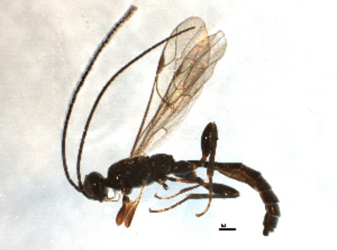

BIOUG09981-A05 [Lateral]
Odontocolon
Family: Ichneumonidae
BIN URI: BOLD:ACL3751

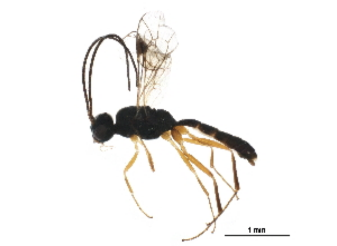

BIOUG24015-D01 [Lateral]
Ichneumonidae
Family: Ichneumonidae

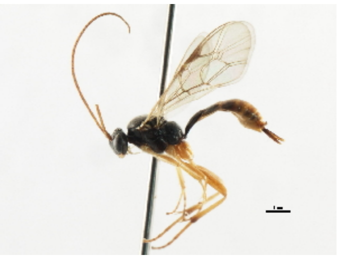

CNCHYM 03729 [Lateral]
Astiphromma strenuum
Family: Ichneumonidae
BIN URI: BOLD:AAA5522

IMAGE NOT AVAILABLE

BIOUG22872-H01
Ichneumonidae
Family: Ichneumonidae

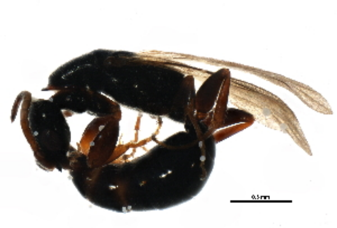

BIOUG22358-A07 [Lateral]
Bethylidae
Family: Bethylidae
BIN URI: BOLD:ABY2749

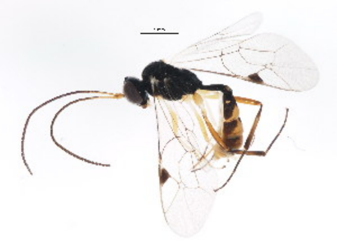

BIOUG00859-B08 [Lateral]
Cryptinae
Family: Ichneumonidae
BIN URI: BOLD:AAY6844

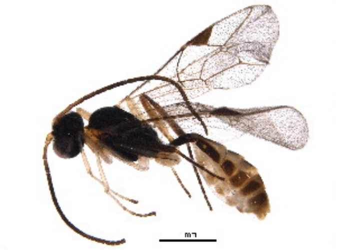

BIOUG21888-A05 [Lateral]
Ichneumonidae
Family: Ichneumonidae
BIN URI: BOLD:ACV5004

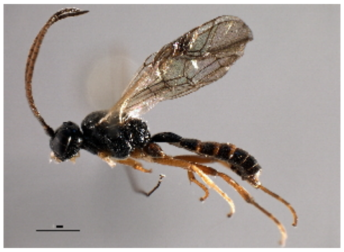

07PROBE-22704 [Lateral]
Eridolius
Family: Ichneumonidae
BIN URI: BOLD:ACN9868

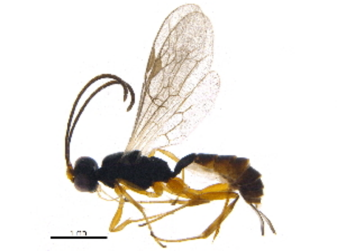

BIOUG22867-C05 [Lateral]
Ichneumonidae
Family: Ichneumonidae
BIN URI: BOLD:ACV5916

IMAGE NOT AVAILABLE

BIOUG22569-G09
Ichneumonidae
Family: Ichneumonidae

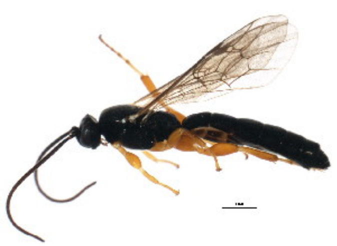

BIOUG00910-G08 [Lateral]
Hymenoptera

BIN URI: BOLD:AAG7713

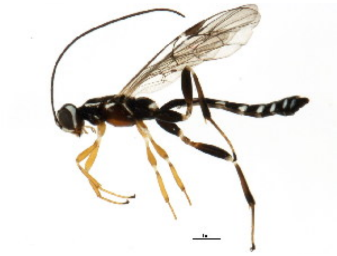

BIOUG01044-F01 [Lateral]
Xoridinae
Family: Ichneumonidae
BIN URI: BOLD:AAZ0238

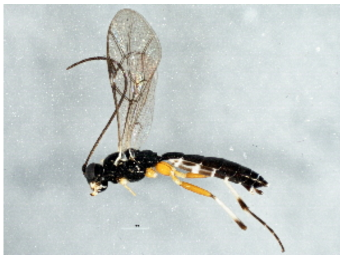

BIOUG04241-G02 [Lateral]
Ctenopelmatinae
Family: Ichneumonidae
BIN URI: BOLD:ACD9155

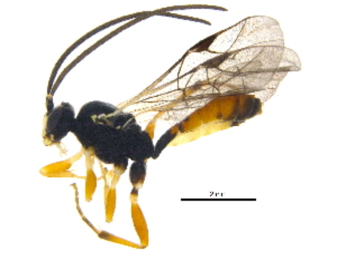

BIOUG22870-C12 [Lateral]
Ichneumonidae
Family: Ichneumonidae
BIN URI: BOLD:ACV5377

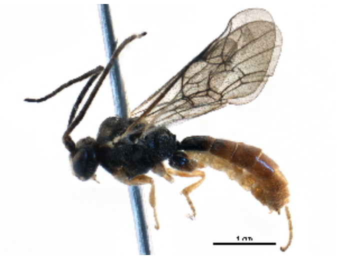

BIOUG24004-F08 [Lateral]
Ichneumonidae
Family: Ichneumonidae

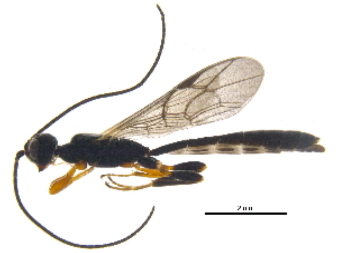

BIOUG22875-G12 [Lateral]
Ichneumonidae
Family: Ichneumonidae
BIN URI: BOLD:ACV5720

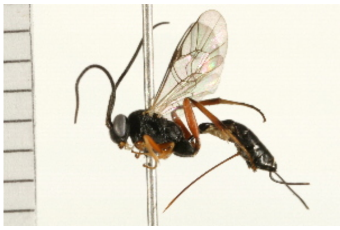

08TTML-0068 [Lateral]
Campopleginae
Family: Ichneumonidae
BIN URI: BOLD:AAG7642

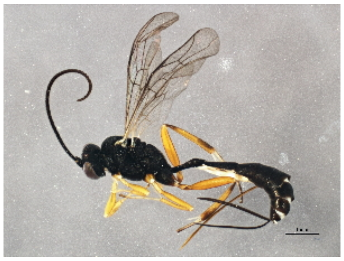

BIOUG02736-D11 [Lateral]
Campopleginae
Family: Ichneumonidae
BIN URI: BOLD:AAH1903

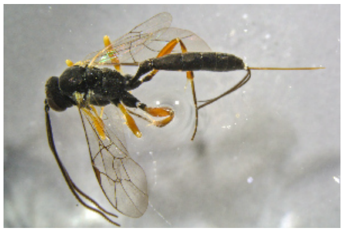

BC-ZSM-HYM-23870-C08 [Lateral]
Ichneumonidae
Family: Ichneumonidae
BIN URI: BOLD:ACU0916

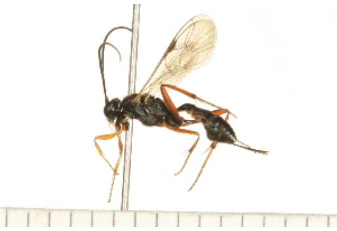

08BBHYM-0675 [Lateral]
Tranosema rostrale
Family: Ichneumonidae
BIN URI: BOLD:AAH1884

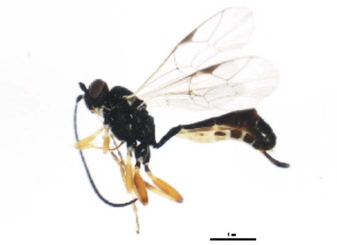

BIOUG01029-G07 [Lateral]
Ichneumonidae
Family: Ichneumonidae
BIN URI: BOLD:ABA5942

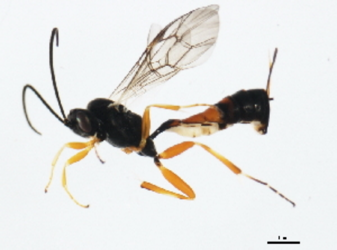

BIOUG01022-C11 [Lateral]
Campopleginae
Family: Ichneumonidae
BIN URI: BOLD:ABA6297

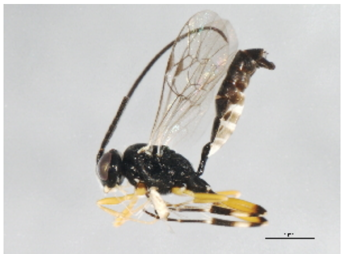

09BBEHY-0716 [Lateral]
Sinophorus
Family: Ichneumonidae
BIN URI: BOLD:ABZ8281

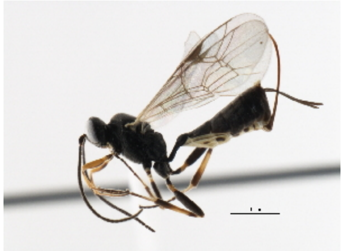

06-PROBE-4425 [Lateral]
Tranosema rostrale
Family: Ichneumonidae
BIN URI: BOLD:AAD1926

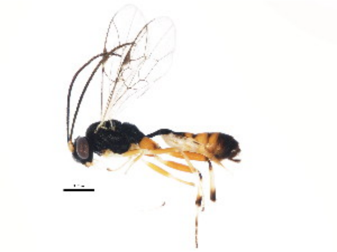

BIOUG00801-H04 [Lateral]
Phobocampe bicingulata
Family: Ichneumonidae
BIN URI: BOLD:AAM7401

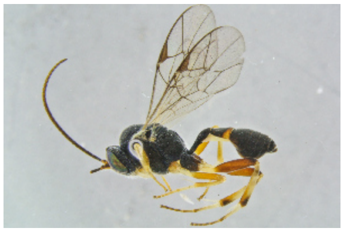

BC-ZSM-HYM-23871-C04 [Lateral]
Ichneumonidae
Family: Ichneumonidae
BIN URI: BOLD:AAU8361

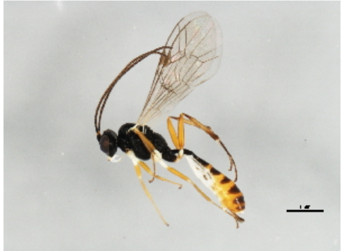

BIOUG00994-A10 [Lateral]
Hymenoptera

BIN URI: BOLD:AAU8441

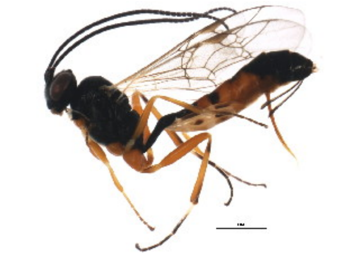

BIOUG00910-F06 [Lateral]
Hymenoptera

BIN URI: BOLD:AAG8091

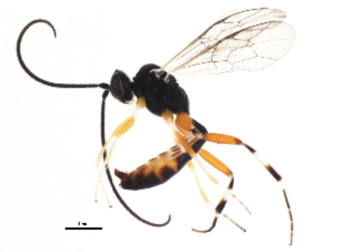

BIOUG01253-C01 [Lateral]
Campoletis
Family: Ichneumonidae
BIN URI: BOLD:AAU8365

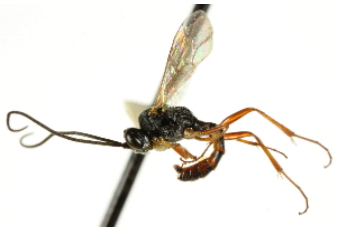

CNCHYM 05306 [Lateral]
Campoletis flavicincta
Family: Ichneumonidae
BIN URI: BOLD:AAZ8146

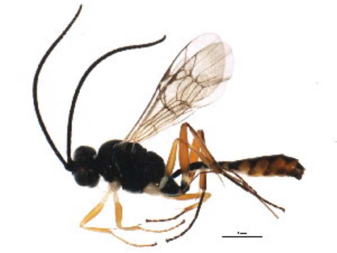

BIOUG00910-F07 [Lateral]
Hymenoptera

BIN URI: BOLD:ABA6171

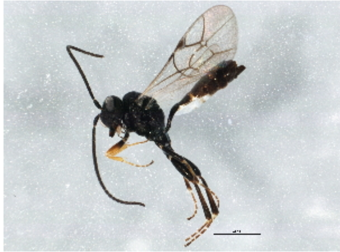

BIOUG05697-D05 [Lateral]
Ichneumonidae
Family: Ichneumonidae
BIN URI: BOLD:ABA6269

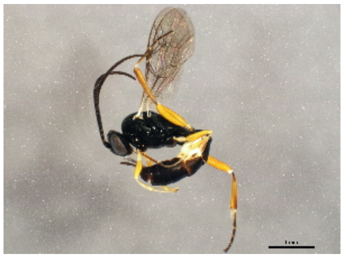

BIOUG03675-H11 [Lateral]
Campoletis sp. 17
Family: Ichneumonidae
BIN URI: BOLD:AAG5792

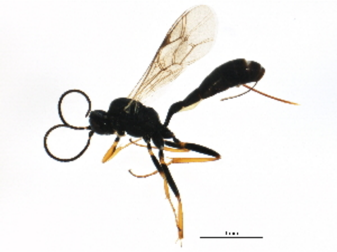

BIOUG22361-E09 [Lateral]
Campopleginae
Family: Ichneumonidae
BIN URI: BOLD:ACF9375

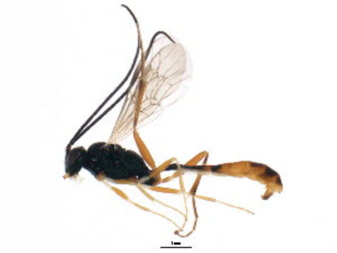

BIOUG00910-D01 [Lateral]
Hymenoptera

BIN URI: BOLD:ABZ4364

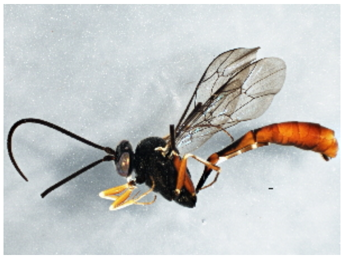

BIOUG05832-D11 [Lateral]
Ichneumonidae
Family: Ichneumonidae
BIN URI: BOLD:ACI9776

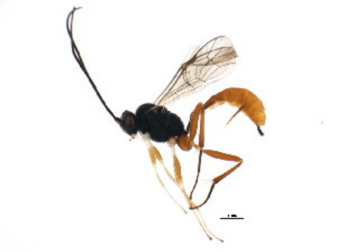

BIOUG00910-D04 [Lateral]
Hymenoptera

BIN URI: BOLD:AAG5788

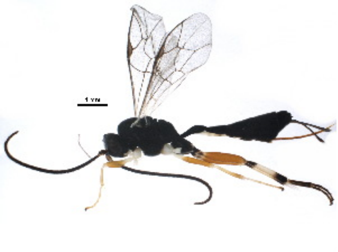

BIOUG22870-D11 [Lateral]
Campopleginae
Family: Ichneumonidae
BIN URI: BOLD:ABX9742

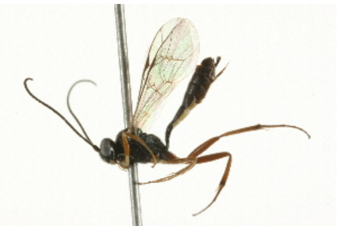

08TTML-0486 [Lateral]
Enytus apostata
Family: Ichneumonidae
BIN URI: BOLD:AAG5797

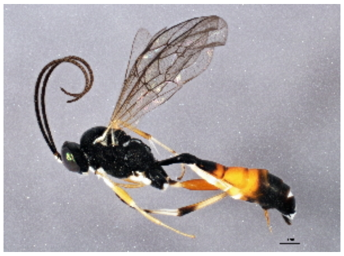

BIOUG03052-G01 [Lateral]
Campopleginae
Family: Ichneumonidae
BIN URI: BOLD:ACB1800

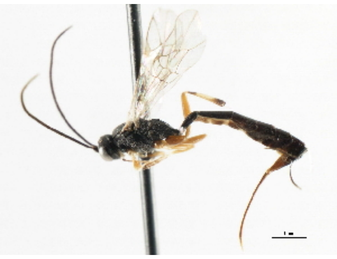

CNCHYM 07380 [Lateral]
Diadegma pendulum
Family: Ichneumonidae
BIN URI: BOLD:AAZ9563

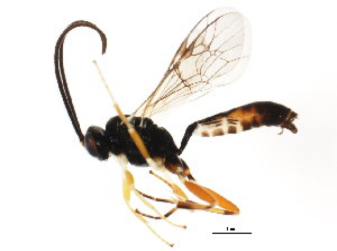

BIOUG01082-F07 [Lateral]
Campopleginae
Family: Ichneumonidae
BIN URI: BOLD:AAG8409

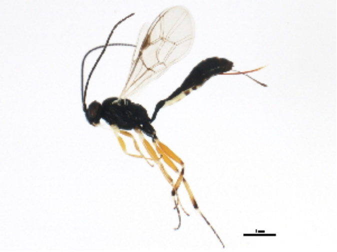

BIOUG01330-E11 [Lateral]
Diadegma nr. fenestrale
Family: Ichneumonidae
BIN URI: BOLD:AAG7740

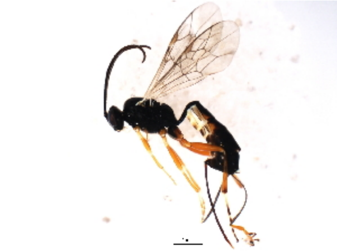

09BBEHY-2127 [Lateral]
Campopleginae
Family: Ichneumonidae
BIN URI: BOLD:AAM7514

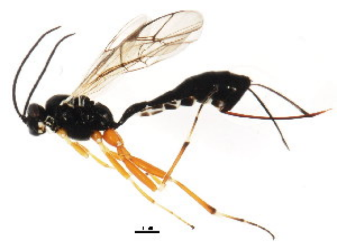

BIOUG01048-A12 [Lateral]
Campopleginae
Family: Ichneumonidae
BIN URI: BOLD:ABA6131

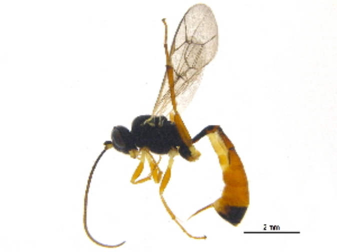

BIOUG22567-B04 [Lateral]
Campopleginae
Family: Ichneumonidae
BIN URI: BOLD:ACV5454

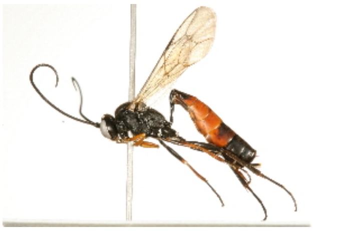

09BBEHY-0418 [Lateral]
Dusona sp.
Family: Ichneumonidae
BIN URI: BOLD:AAC9245

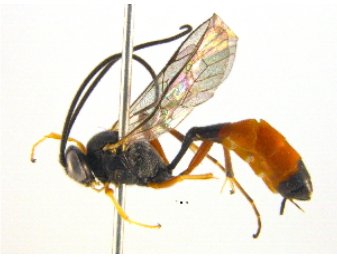

BIOUG03373-E07 [Lateral]
Dusona minor
Family: Ichneumonidae
BIN URI: BOLD:AAH1652

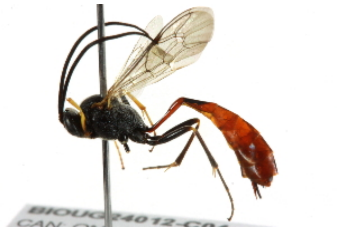

BIOUG24012-C04 [Lateral]
Dusona
Family: Ichneumonidae

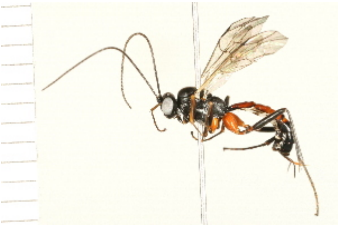

08TTML-0156 [Lateral]
Banchinae
Family: Ichneumonidae
BIN URI: BOLD:AAG7666

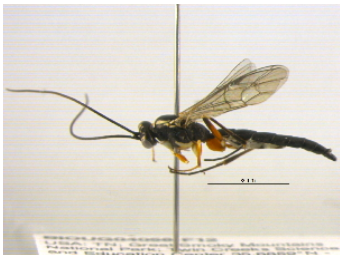

BIOUG04058-F12 [Lateral]
Lissonota
Family: Ichneumonidae
BIN URI: BOLD:AAU8646

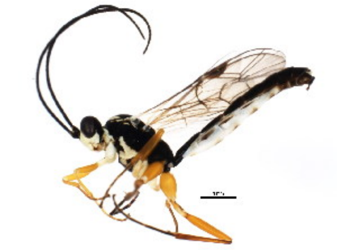

BIOUG01281-B09 [Lateral]
Lissonota coracina
Family: Ichneumonidae
BIN URI: BOLD:AAG7794

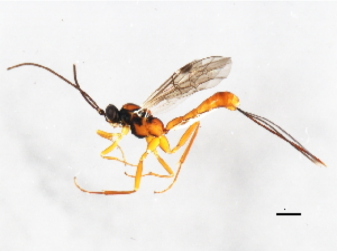

BIOUG01022-E08 [Lateral]
Banchinae
Family: Ichneumonidae
BIN URI: BOLD:ACE3609

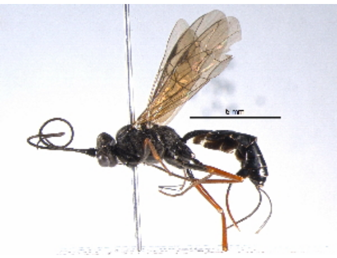

BIOUG05670-D07 [Lateral]
Banchinae
Family: Ichneumonidae
BIN URI: BOLD:AAY6799

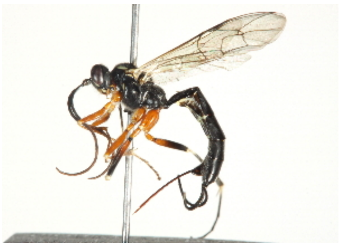

BIOUG07534-D01 [Lateral]
Banchinae
Family: Ichneumonidae
BIN URI: BOLD:ACI7472

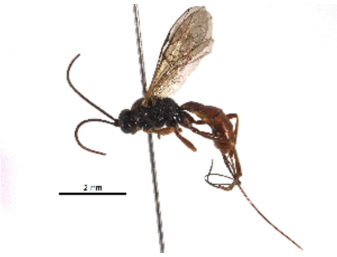

BIOUG22326-A11 [Lateral]
Banchinae
Family: Ichneumonidae
BIN URI: BOLD:ACL5173

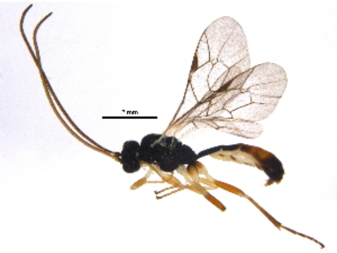

BIOUG03052-H09 [Lateral]
Ctenopelmatinae
Family: Ichneumonidae
BIN URI: BOLD:ACP3071

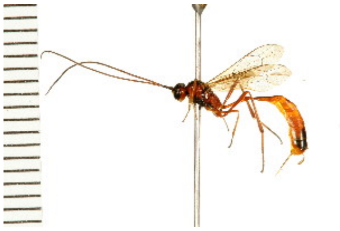

08BBHYM-0572 [Lateral]
Agrypon flexorium
Family: Ichneumonidae
BIN URI: BOLD:AAH7052

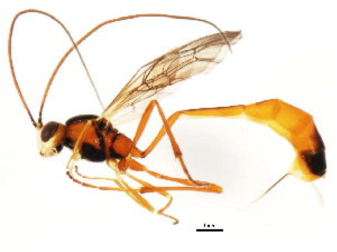

BIOUG01093-H11 [Lateral]
Agrypon flexorium
Family: Ichneumonidae
BIN URI: BOLD:ACE7001

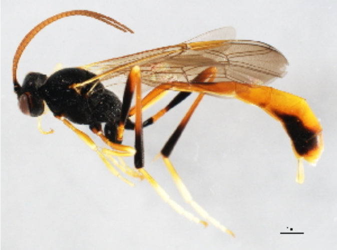

BIOUG01022-D04 [Lateral]
Anomaloninae
Family: Ichneumonidae
BIN URI: BOLD:ABX5713

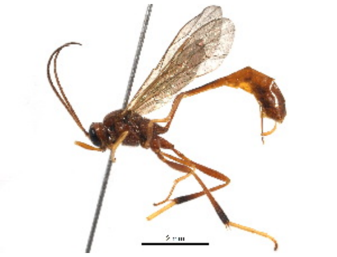

BIOUG22570-E04 [Lateral]
Ichneumonidae
Family: Ichneumonidae
BIN URI: BOLD:ACW0948

IMAGE NOT AVAILABLE

BIOUG24012-C06
Glypta
Family: Ichneumonidae

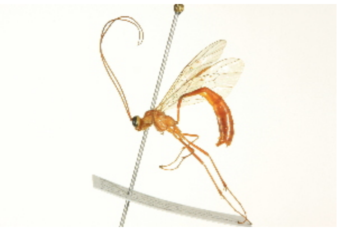

BIOUG02951-D06 [Lateral]
Ophion
Family: Ichneumonidae
BIN URI: BOLD:ABA5930

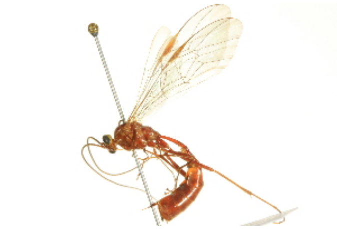

BIOUG02951-C05 [Lateral]
Ophion bilineatus
Family: Ichneumonidae
BIN URI: BOLD:AAG8323

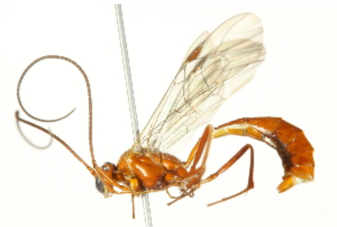

BIOUG00316-G10 [Lateral]
Ophion clave
Family: Ichneumonidae
BIN URI: BOLD:AAG7774

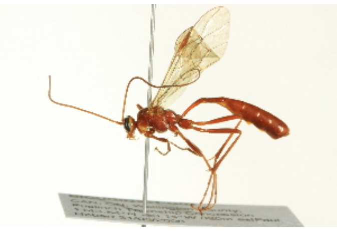

BIOUG02951-G02 [Lateral]
Ophion idoneus
Family: Ichneumonidae
BIN URI: BOLD:AAN8172

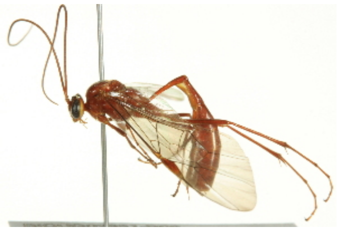

BIOUG02951-B09 [Lateral]
Ophion sp. 5 MDS2014
Family: Ichneumonidae
BIN URI: BOLD:AAI3361

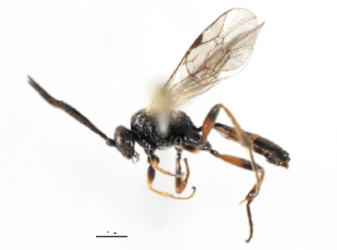

07PROBE-23738 [Lateral]
Cryptinae
Family: Ichneumonidae
BIN URI: BOLD:AAE2457

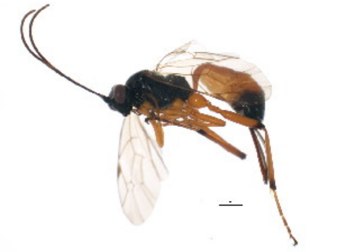

BIOUG00910-C06 [Lateral]
Hymenoptera

BIN URI: BOLD:AAG8275

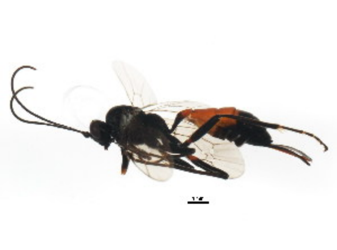

BIOUG01030-D11 [Lateral]
Cryptinae
Family: Ichneumonidae
BIN URI: BOLD:ACN0503

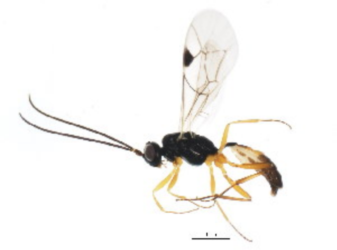

BIOUG01034-H06 [Lateral]
Cryptinae
Family: Ichneumonidae
BIN URI: BOLD:AAU8485

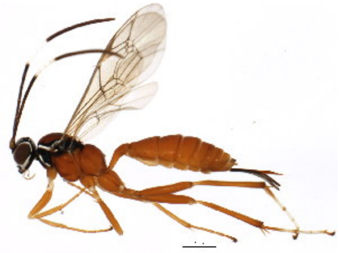

BIOUG00910-G09 [Lateral]
Hymenoptera

BIN URI: BOLD:AAG7737

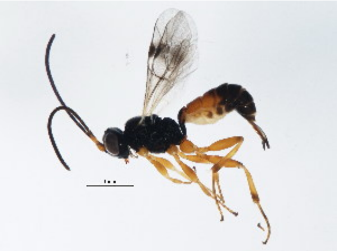

BIOUG00823-D08 [Lateral]
Cryptinae
Family: Ichneumonidae
BIN URI: BOLD:AAW0431

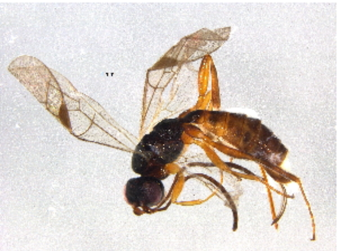

BIOUG04311-B09 [Lateral]
Cryptinae
Family: Ichneumonidae
BIN URI: BOLD:ACD1672

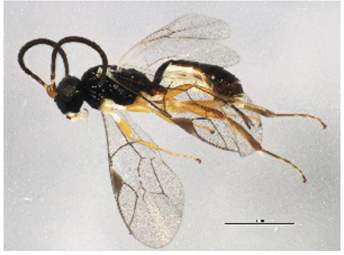

ASGLE-0528 [Lateral]
Hymenoptera

BIN URI: BOLD:AAU8490

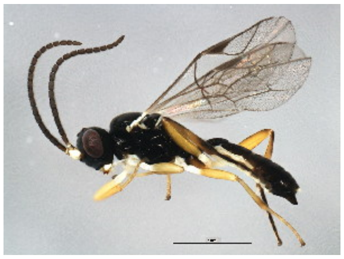

ASGLE-0540 [Lateral]
Hymenoptera

BIN URI: BOLD:ACF3297

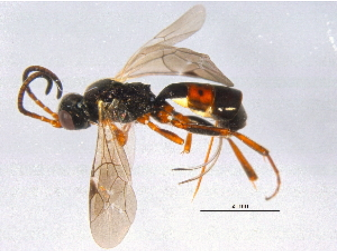

BIOUG05537-A02 [Lateral]
Cryptinae
Family: Ichneumonidae
BIN URI: BOLD:AAH1692

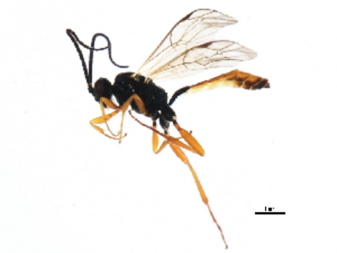

BIOUG01022-E12 [Lateral]
Cryptinae
Family: Ichneumonidae
BIN URI: BOLD:ABX5561

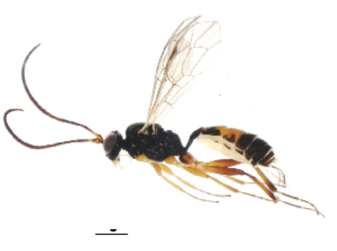

10BBCHY-2601 [Lateral]
Cryptinae
Family: Ichneumonidae
BIN URI: BOLD:AAF1382

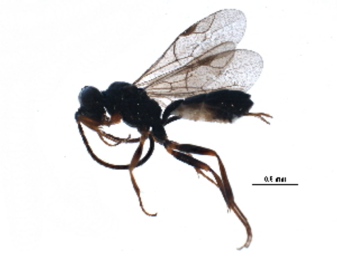

BIOUG21885-H07 [Lateral]
Ichneumonidae
Family: Ichneumonidae
BIN URI: BOLD:ACV3635

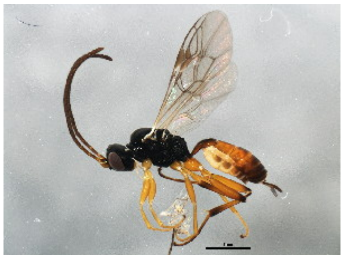

ASGLE-0939 [Lateral]
Hymenoptera

BIN URI: BOLD:AAG7744

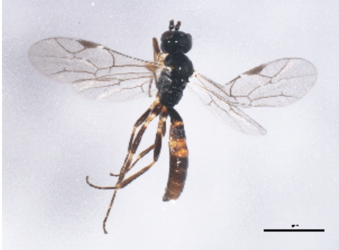

BIOUG01018-A06 [Dorsal]
Cryptinae
Family: Ichneumonidae
BIN URI: BOLD:ABA5934

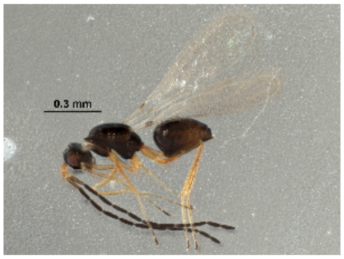

BIOUG06689-H09 [Lateral]
Gelis
Family: Ichneumonidae
BIN URI: BOLD:ACJ5069

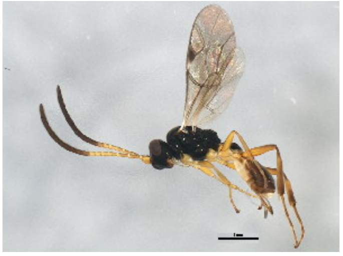

ASGLE-0913 [Lateral]
Hymenoptera

BIN URI: BOLD:AAG9197

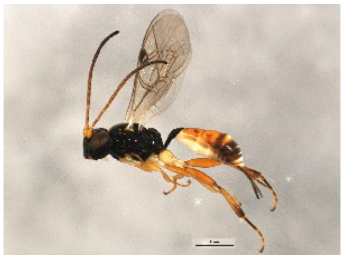

ASGLE-0860 [Lateral]
Hymenoptera

BIN URI: BOLD:AAU8687

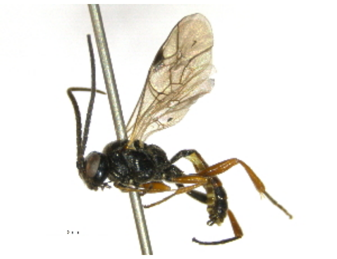

BIOUG03281-H07 [Lateral]
Cryptinae
Family: Ichneumonidae
BIN URI: BOLD:ACB3569

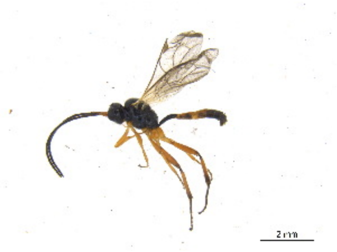

BIOUG06753-D05 [Lateral]
Hymenoptera

BIN URI: BOLD:AAU8389

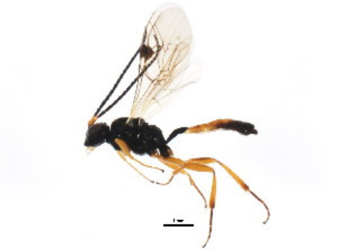

BIOUG01036-C05 [Lateral]
Cryptinae
Family: Ichneumonidae
BIN URI: BOLD:ABA5909

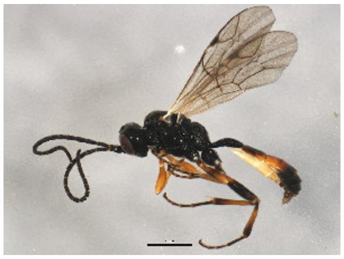

ASGLE-1123 [Lateral]
Hymenoptera

BIN URI: BOLD:AAE9438

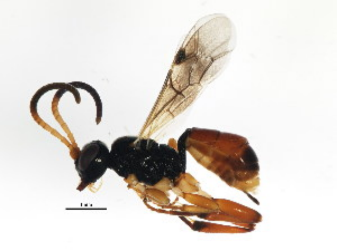

BIOUG00846-E08 [Lateral]
Cryptinae
Family: Ichneumonidae
BIN URI: BOLD:AAG7638

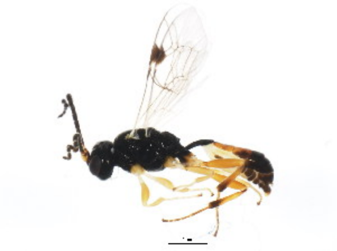

BIOUG01032-F11 [Lateral]
Cryptinae
Family: Ichneumonidae
BIN URI: BOLD:AAU8327

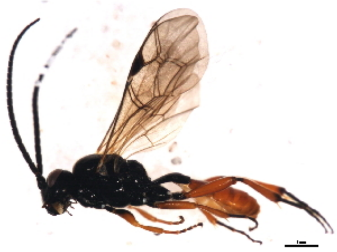

09PROBE-A0015 [Lateral]
Cryptinae
Family: Ichneumonidae
BIN URI: BOLD:AAN7591

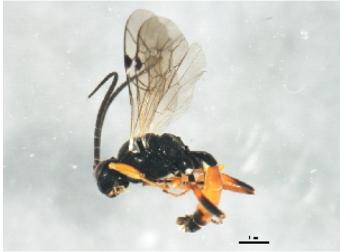

BIOUG01033-B04 [Lateral]
Cryptinae
Family: Ichneumonidae
BIN URI: BOLD:ACF2067

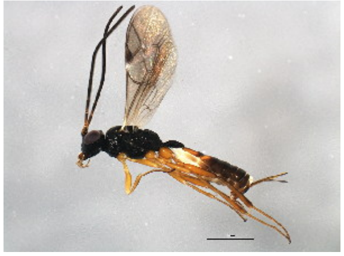

ASGLE-0495 [Lateral]
Hymenoptera

BIN URI: BOLD:AAU8483

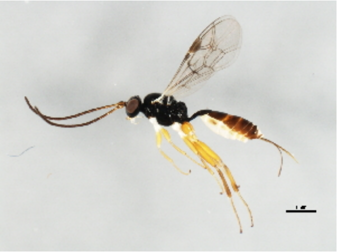

BIOUG00994-A09 [Lateral]
Hymenoptera

BIN URI: BOLD:AAM9117

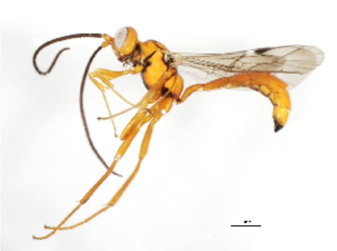

09BBHYM-644 [Lateral]
Ichneumonidae
Family: Ichneumonidae
BIN URI: BOLD:AAG8236

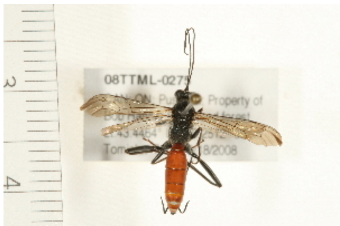

08TTML-0275 [Dorsal]
Cryptus albitarsis
Family: Ichneumonidae
BIN URI: BOLD:AAH1693

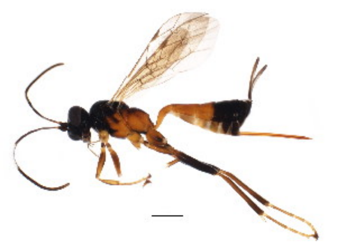

BIOUG01631-B02 [Lateral]
Cryptinae
Family: Ichneumonidae
BIN URI: BOLD:AAH1793

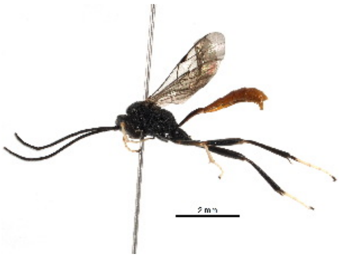

BIOUG22570-F02 [Lateral]
Trychosis
Family: Ichneumonidae
BIN URI: BOLD:ACW1110

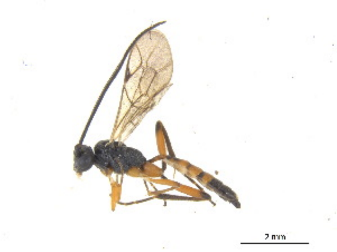

BIOUG06753-F09 [Lateral]
Hymenoptera

BIN URI: BOLD:AAG7687

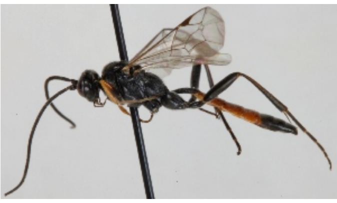

FICH-001207 [Lateral]
Agrothereutes mansuetor
Family: Ichneumonidae
BIN URI: BOLD:AAG7768

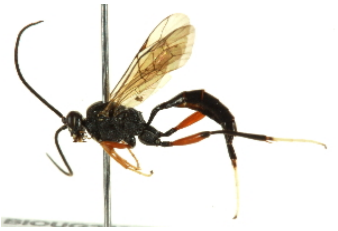

BIOUG24008-D12 [Lateral]
Ichneumonidae
Family: Ichneumonidae

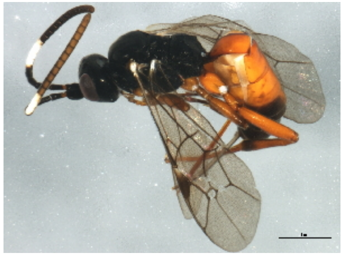

BIOUG01483-B07 [Lateral]
Oresbius taeniatus
Family: Ichneumonidae
BIN URI: BOLD:AAH1683

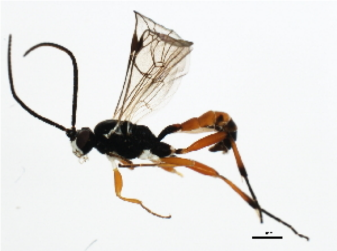

10PHMAL-0318 [Lateral]
Oresbius
Family: Ichneumonidae
BIN URI: BOLD:ACE9715

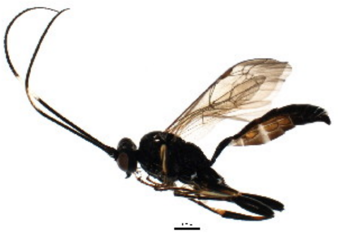

BIOUG01283-F08 [Lateral]
Asthenolabus
Family: Ichneumonidae
BIN URI: BOLD:AAU8294

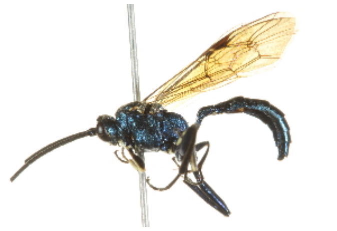

10BBCHY-3565 [Lateral]
Ichneumoninae
Family: Ichneumonidae
BIN URI: BOLD:AAU8895

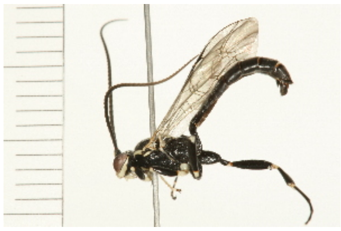

08TTML-0205 [Lateral]
Ichneumoninae
Family: Ichneumonidae
BIN URI: BOLD:AAG7676

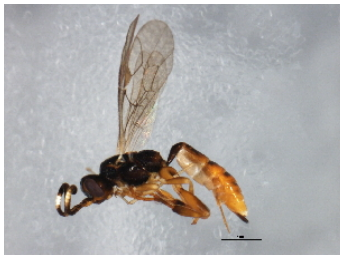

09BBEHY-1940 [Lateral]
Ichneumoninae
Family: Ichneumonidae
BIN URI: BOLD:AAH2179

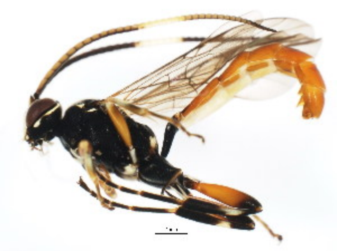

BIOUG01032-B10 [Lateral]
Ichneumoninae
Family: Ichneumonidae
BIN URI: BOLD:AAU8223

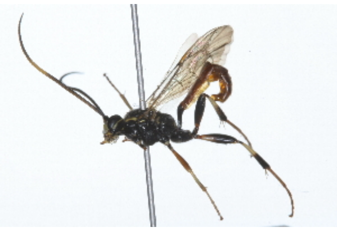

09BBHYM-198 [Lateral]
Ichneumoninae
Family: Ichneumonidae
BIN URI: BOLD:ACE8616

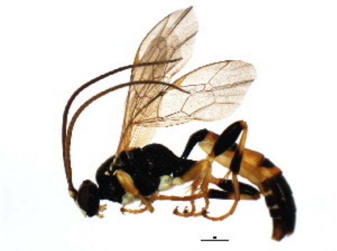

BIOUG01044-G10 [Lateral]
Ichneumoninae
Family: Ichneumonidae
BIN URI: BOLD:AAN7588

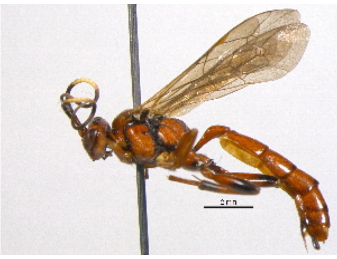

BIOUG05513-A02 [Lateral]
Ichneumoninae
Family: Ichneumonidae
BIN URI: BOLD:AAU8706

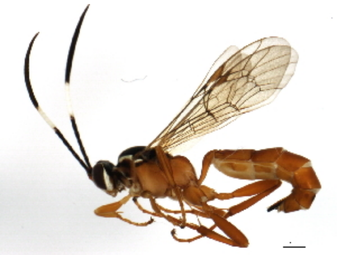

ASGLE-0678 [Lateral]
Hymenoptera

BIN URI: BOLD:AAG7679

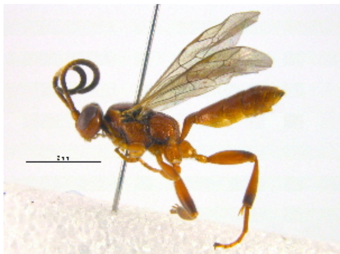

BIOUG03373-H05 [Lateral]
Ichneumoninae
Family: Ichneumonidae
BIN URI: BOLD:AAG8176

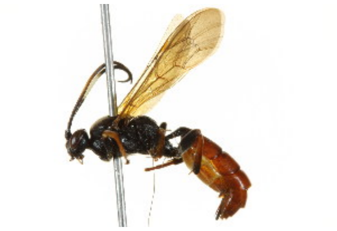

BIOUG01282-D04 [Lateral]
Ichneumon
Family: Ichneumonidae
BIN URI: BOLD:ACE4814

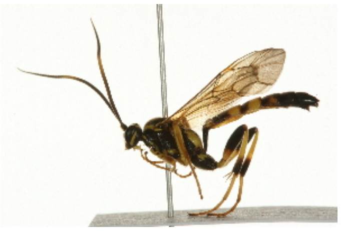

09BBEHY-0019 [Lateral]
Ichneumon computatorius
Family: Ichneumonidae
BIN URI: BOLD:ACE3185

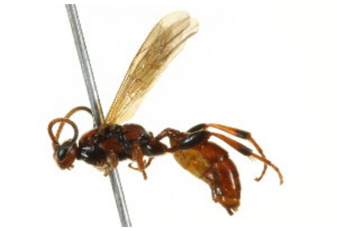

BIOUG01282-F10 [Lateral]
Ichneumon discoensis
Family: Ichneumonidae
BIN URI: BOLD:ACE9045

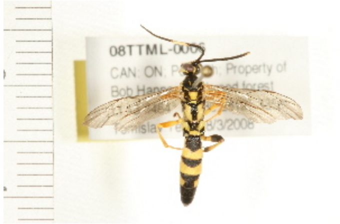

08TTML-0006 [Dorsal]
Ichneumoninae
Family: Ichneumonidae
BIN URI: BOLD:ACF0076

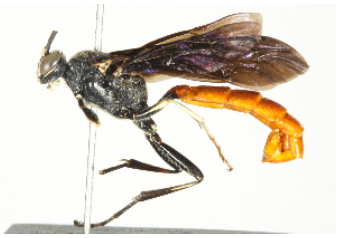

BIOUG07534-B06 [Lateral]
Ichneumoninae
Family: Ichneumonidae
BIN URI: BOLD:ACI9173

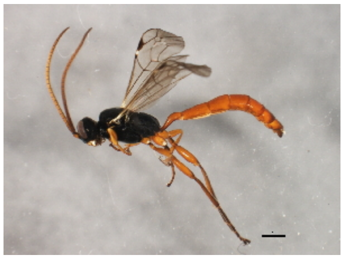

BIOUG00988-H03 [Lateral]
Hymenoptera

BIN URI: BOLD:AAG7641

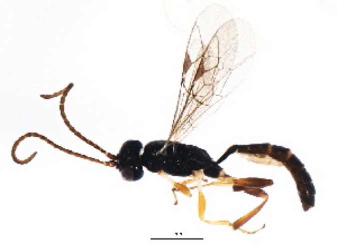

BIOUG00845-B03 [Lateral]
Ichneumoninae
Family: Ichneumonidae
BIN URI: BOLD:AAH2004

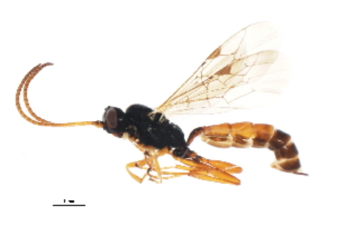

10BBCHY-3075 [Lateral]
Ichneumoninae
Family: Ichneumonidae
BIN URI: BOLD:AAG7732

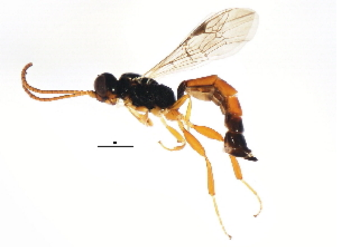

ASGLE2-0169 [Lateral]
Hymenoptera

BIN URI: BOLD:AAG7745

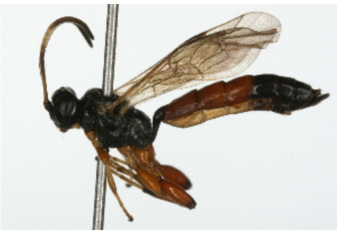

08TTML-0422 [Lateral]
Ichneumoninae
Family: Ichneumonidae
BIN URI: BOLD:ABZ7151

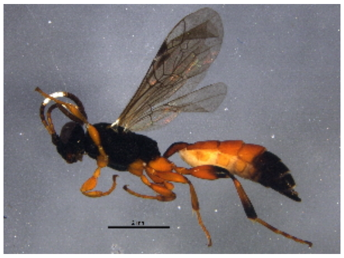

BIOUG07536-D07 [Lateral]
Ichneumoninae
Family: Ichneumonidae
BIN URI: BOLD:ACJ1122

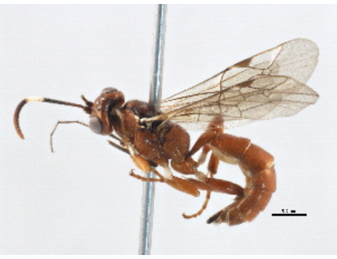

BIOUG09053-H10 [Lateral]
Ichneumoninae
Family: Ichneumonidae
BIN URI: BOLD:ACL1096

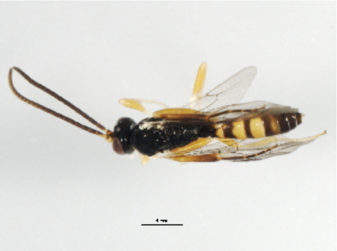

09BBEHY-0273 [Dorsal]
Cryptinae
Family: Ichneumonidae
BIN URI: BOLD:AAG9169

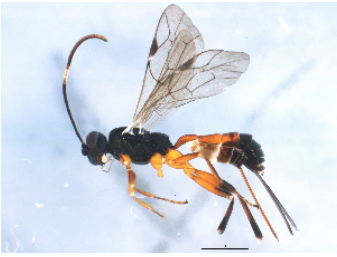

BIOUG04528-G11 [Lateral]
Cryptinae
Family: Ichneumonidae
BIN URI: BOLD:ACF3930

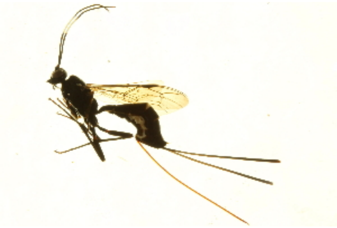

ASGLE2-0300 [Lateral]
Hymenoptera

BIN URI: BOLD:AAH1886

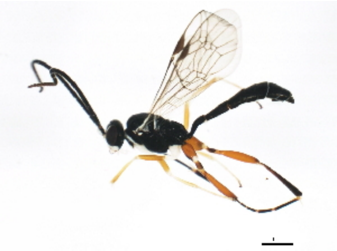

BIOUG01033-B08 [Lateral]
Cryptinae
Family: Ichneumonidae
BIN URI: BOLD:ABA5989

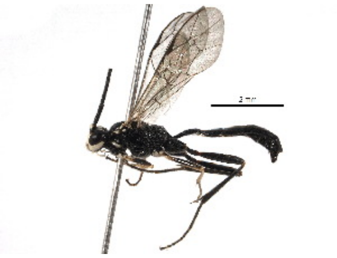

BIOUG22570-G02 [Lateral]
Ichneumonidae
Family: Ichneumonidae
BIN URI: BOLD:ACW1092

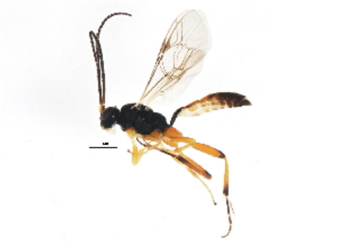

ASGLE2-0417 [Lateral]
Hymenoptera

BIN URI: BOLD:AAG7710

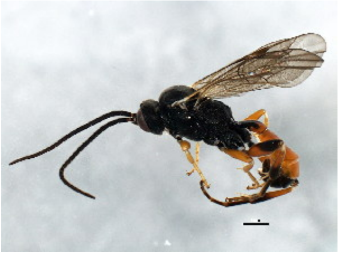

ASGLE-0986 [Lateral]
Hymenoptera

BIN URI: BOLD:AAU8228

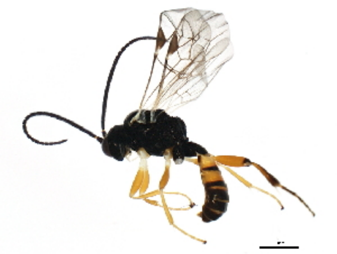

10PHMAL-0669 [Lateral]
Cryptinae
Family: Ichneumonidae
BIN URI: BOLD:AAQ2692

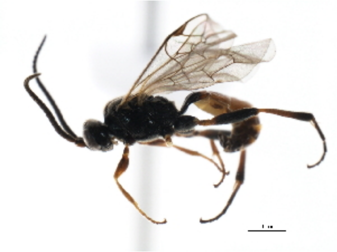

CNCH0048 [Lateral]
Cryptinae
Family: Ichneumonidae
BIN URI: BOLD:ABY7035

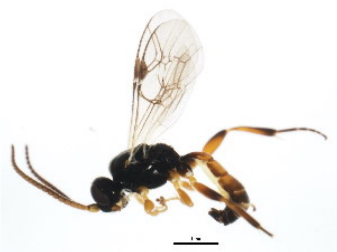

BIOUG01030-D02 [Lateral]
Cryptinae
Family: Ichneumonidae
BIN URI: BOLD:ABA7969

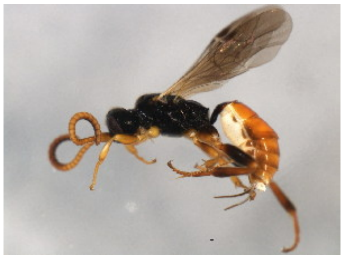

ASGLE-0054 [Lateral]
Hymenoptera

BIN URI: BOLD:ACE5353

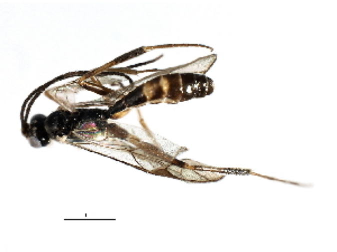

07PROBE-24343 [Lateral]
Orthocentrinae
Family: Ichneumonidae
BIN URI: BOLD:AAG0964

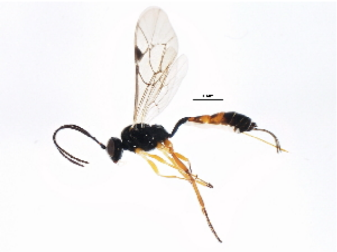

ASGLE-0475 [Lateral]
Hymenoptera

BIN URI: BOLD:AAU8495

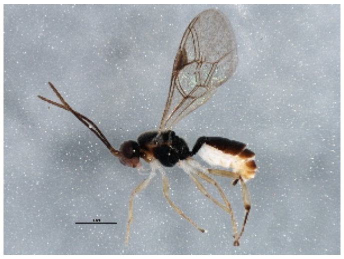

BIOUG03621-C07 [Lateral]
Adelognathus
Family: Ichneumonidae
BIN URI: BOLD:AAY6793

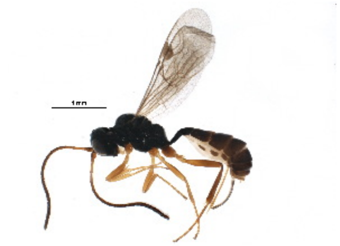

BIOUG22421-D01 [Lateral]
Ichneumonidae
Family: Ichneumonidae
BIN URI: BOLD:ACL8125

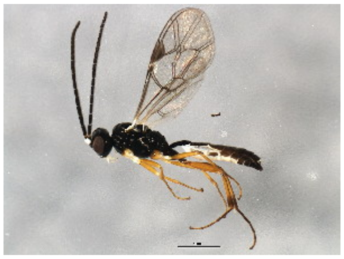

ASGLE-0506 [Lateral]
Hymenoptera

BIN URI: BOLD:AAU8487

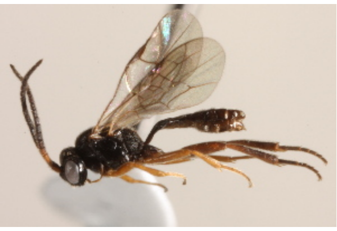

CNCHYM 08512 [Lateral]
Dialipsis dissimilis
Family: Ichneumonidae
BIN URI: BOLD:ABA6048

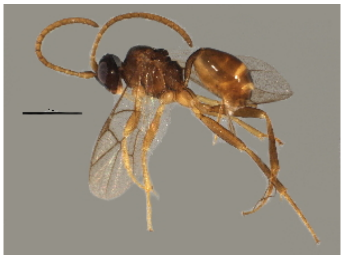

BIOUG07032-F06 [Lateral]
Ichneumonidae
Family: Ichneumonidae
BIN URI: BOLD:ACI6350

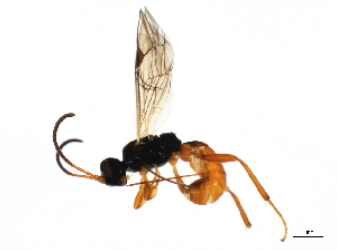

10PHMAL-0617 [Lateral]
Cryptinae
Family: Ichneumonidae
BIN URI: BOLD:AAU8372

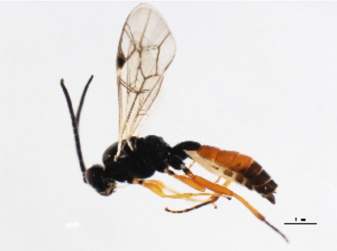

BIOUG01028-C02 [Lateral]
Cryptinae
Family: Ichneumonidae
BIN URI: BOLD:ABA7959

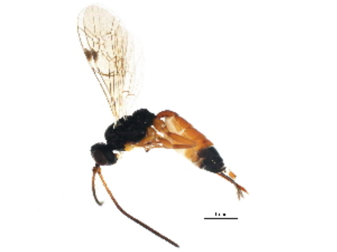

BIOUG01291-D02 [Lateral]
Cryptinae
Family: Ichneumonidae
BIN URI: BOLD:ABA8048

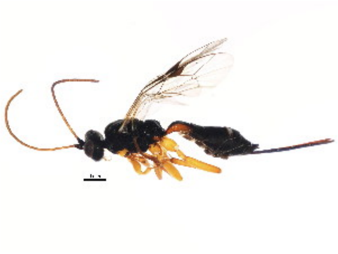

BIOUG00861-B01 [Lateral]
Echthrus sp.
Family: Ichneumonidae
BIN URI: BOLD:AAG9189

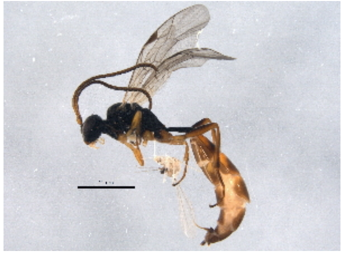

BIOUG05443-G11 [Lateral]
Mesoleptus congener
Family: Ichneumonidae
BIN URI: BOLD:ACE6694

IMAGE NOT AVAILABLE

BIOUG21888-B01
Ichneumonidae
Family: Ichneumonidae

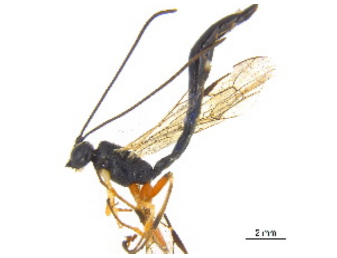

BIOUG06752-E05 [Lateral]
Hymenoptera

BIN URI: BOLD:AAU8384

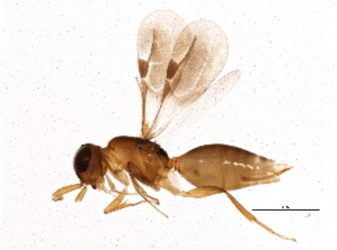

BIOUG05742-H01 [Lateral]
Megaspilidae
Family: Megaspilidae
BIN URI: BOLD:ACF8460

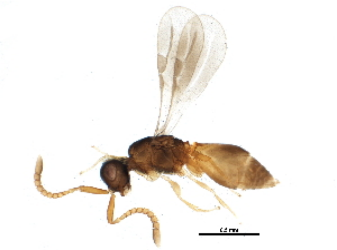

BIOUG09249-D11 [Lateral]
Megaspilidae
Family: Megaspilidae
BIN URI: BOLD:ACK9712

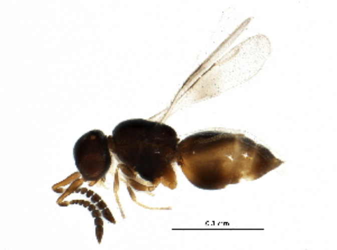

BIOUG22363-B08 [Lateral]
Megaspilidae
Family: Megaspilidae
BIN URI: BOLD:ACV3846

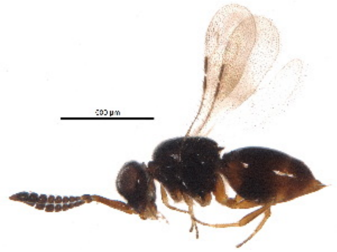

BIOUG23085-B08 [Lateral]
Megaspilidae
Family: Megaspilidae
BIN URI: BOLD:ACW1215

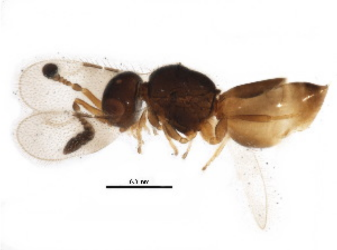

BIOUG22420-C04 [Lateral]
Megaspilidae
Family: Megaspilidae
BIN URI: BOLD:ACV2997

IMAGE NOT AVAILABLE

BIOUG22294-E02
Megaspilidae
Family: Megaspilidae

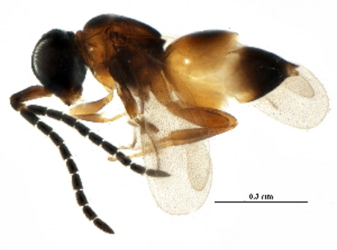

BIOUG22727-D10 [Lateral]
Megaspilidae
Family: Megaspilidae
BIN URI: BOLD:ACJ7345

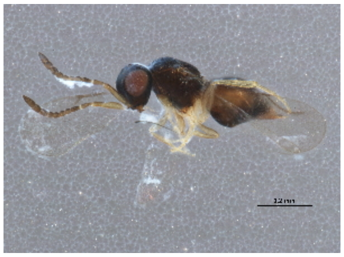

BIOUG11332-A01 [Lateral]
Megaspilidae
Family: Megaspilidae
BIN URI: BOLD:ACM6824

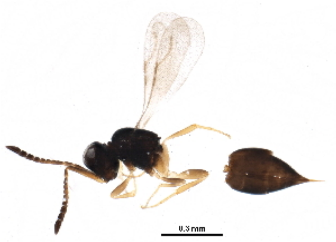

BIOUG22626-B02 [Lateral]
Megaspilidae
Family: Megaspilidae
BIN URI: BOLD:ACV5959

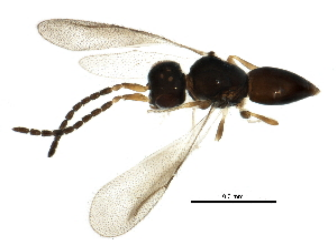

BIOUG22626-C06 [Lateral]
Megaspilidae
Family: Megaspilidae
BIN URI: BOLD:ACB7855

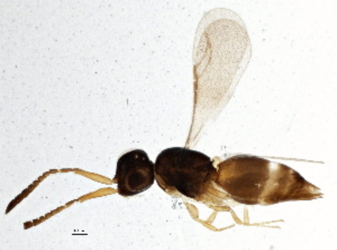

BIOUG04766-A07 [Lateral]
Hymenoptera

BIN URI: BOLD:AAU8999

IMAGE NOT AVAILABLE

BIOUG22727-A03
Megaspilidae
Family: Megaspilidae

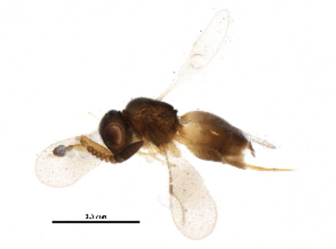

BIOUG21288-G01 [Lateral]
Hymenoptera

BIN URI: BOLD:ACV0821

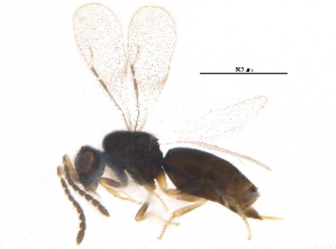

BIOUG16339-E03 [Lateral]
Hymenoptera

BIN URI: BOLD:ACQ9215

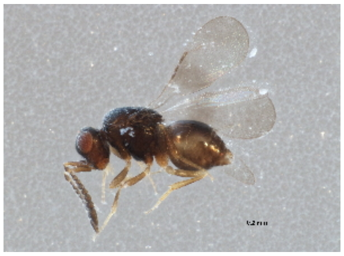

BIOUG10451-E07 [Lateral]
Megaspilidae
Family: Megaspilidae
BIN URI: BOLD:ACL6182

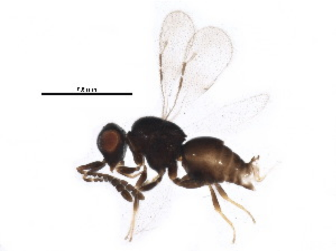

BIOUG22723-D07 [Lateral]
Megaspilidae
Family: Megaspilidae
BIN URI: BOLD:ACV5554

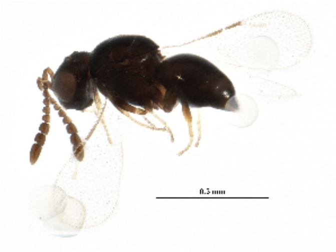

BIOUG23074-E01 [Lateral]
Megaspilidae
Family: Megaspilidae
BIN URI: BOLD:ACV5863

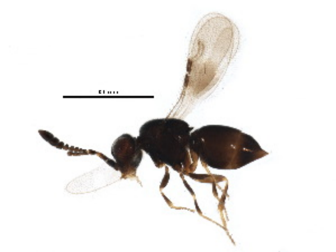

BIOUG22453-A04 [Lateral]
Megaspilidae
Family: Megaspilidae
BIN URI: BOLD:ACJ1417

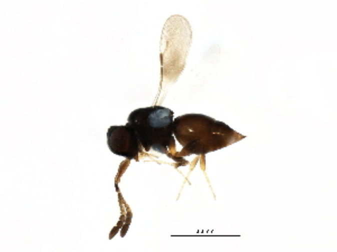

BIOUG22727-F09 [Lateral]
Megaspilidae
Family: Megaspilidae
BIN URI: BOLD:ACK1895

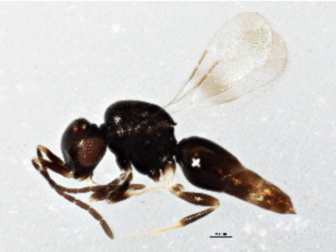

BIOUG03697-G06 [Lateral]
Megaspilidae
Family: Megaspilidae
BIN URI: BOLD:ACB0899

IMAGE NOT AVAILABLE

BIOUG23074-B05
Megaspilidae
Family: Megaspilidae

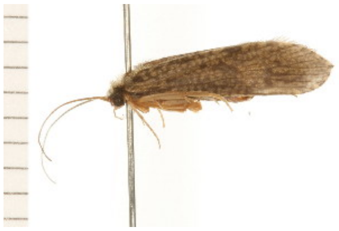

08NBEPT-0788 [Lateral]
Plectrocnemia cinerea
Family: Polycentropodidae

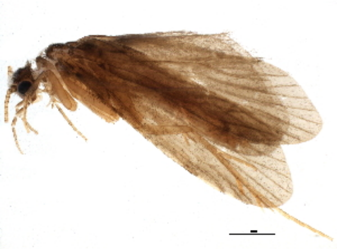

BIOUG10205-C02 [Lateral]
Plectrocnemia cinerea
Family: Polycentropodidae
BIN URI: BOLD:ACL7631

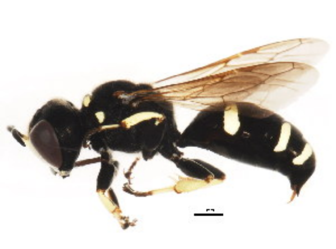

BIOUG01082-H01 [Lateral]
Ectemnius continuus
Family: Crabronidae
BIN URI: BOLD:AAE8158

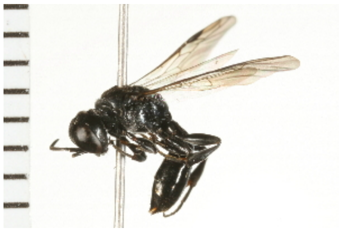

08BBHYM-0600 [Lateral]
Crossocerus barbipes
Family: Crabronidae
BIN URI: BOLD:AAG3190

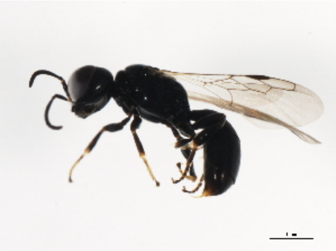

09BBEHY-0690 [Lateral]
Crossocerus
Family: Crabronidae
BIN URI: BOLD:AAG3203

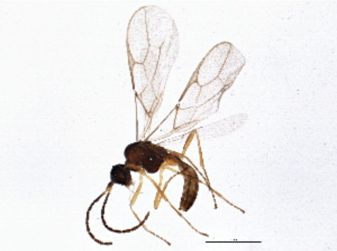

BIOUG06195-E03 [Lateral]
Ephedrus lacertosus
Family: Braconidae
BIN URI: BOLD:ACW2698

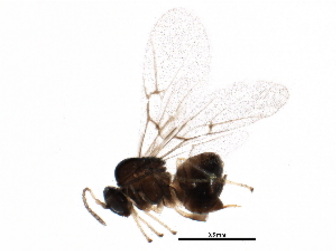

BIOUG22458-E04 [Lateral]
Cynipidae
Family: Cynipidae
BIN URI: BOLD:ACF8609

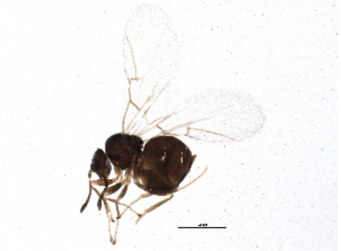

BIOUG13058-C08 [Lateral]
Cynipidae
Family: Cynipidae
BIN URI: BOLD:ACF8754

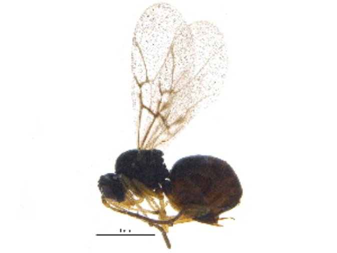

BIOUG16755-G08 [Lateral]
Cynipidae
Family: Cynipidae
BIN URI: BOLD:ACF8991

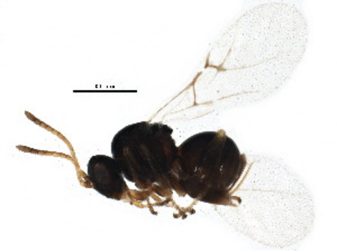

BIOUG22464-G02 [Lateral]
Cynipidae
Family: Cynipidae
BIN URI: BOLD:ACV4572

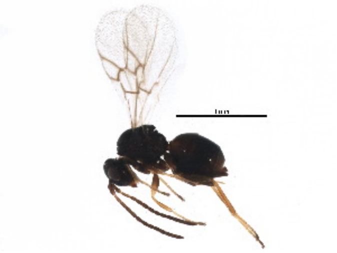

BIOUG22420-C05 [Lateral]
Cynipidae
Family: Cynipidae
BIN URI: BOLD:ACJ0844

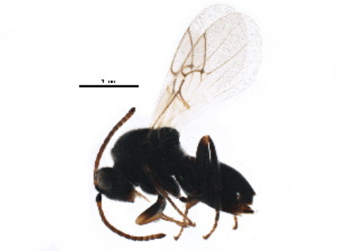

BIOUG22420-G06 [Lateral]
Figitidae
Family: Figitidae
BIN URI: BOLD:ACV2330

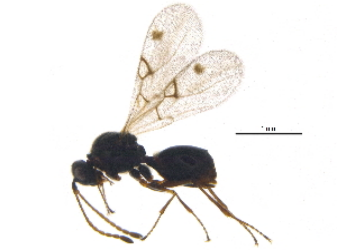

BIOUG21615-E08 [Lateral]
Figitinae
Family: Figitidae
BIN URI: BOLD:AAU9795

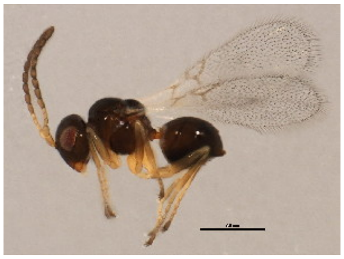

ASGLE-0338 [Lateral]
Charipinae
Family: Figitidae
BIN URI: BOLD:AAU8573

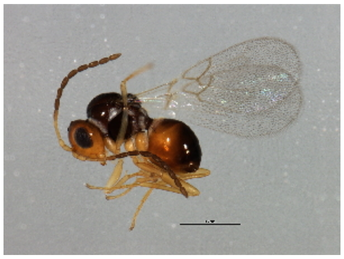

MTHYM-0002 [Lateral]
Charipinae
Family: Figitidae
BIN URI: BOLD:ACT6224

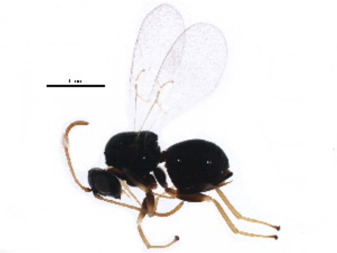

BIOUG22626-B10 [Lateral]
Figitidae
Family: Figitidae
BIN URI: BOLD:ACV5063

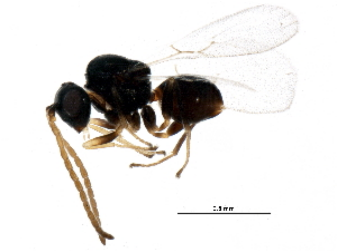

BIOUG21892-B06 [Lateral]
Figitidae
Family: Figitidae
BIN URI: BOLD:ACV5064

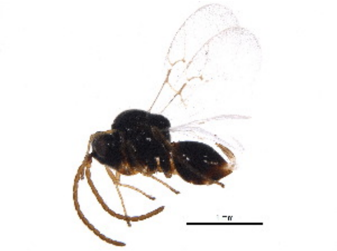

BIOUG21888-A08 [Lateral]
Figitidae
Family: Figitidae
BIN URI: BOLD:ACV5106

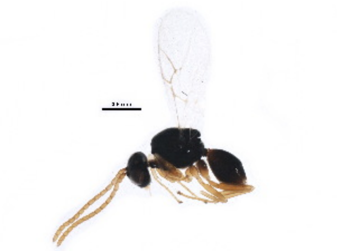

BIOUG21892-B05 [Lateral]
Figitidae
Family: Figitidae
BIN URI: BOLD:AAN8196

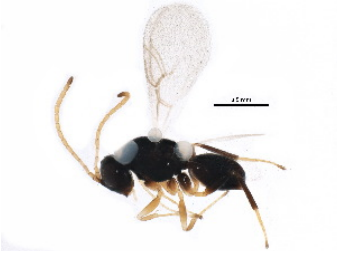

BIOUG22724-B01 [Lateral]
Figitidae
Family: Figitidae
BIN URI: BOLD:ACV5721

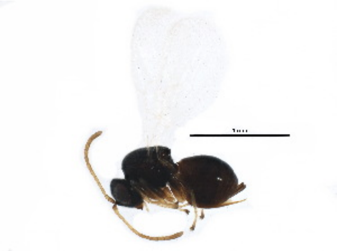

BIOUG22453-G07 [Lateral]
Figitidae
Family: Figitidae
BIN URI: BOLD:ABA9839

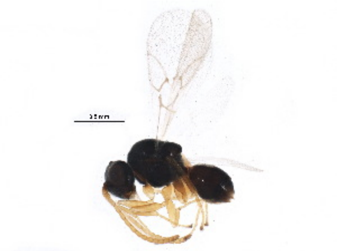

BIOUG22626-A06 [Lateral]
Figitidae
Family: Figitidae
BIN URI: BOLD:ACV5785

IMAGE NOT AVAILABLE

BIOUG24011-E05
Figitidae
Family: Figitidae

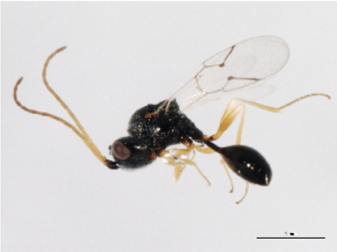

09BBHYM-707 [Lateral]
Anacharis
Family: Figitidae
BIN URI: BOLD:AAG8258

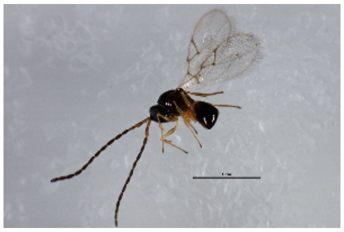

09BBEHY-1910 [Lateral]
Eucoilinae
Family: Figitidae
BIN URI: BOLD:AAM7478

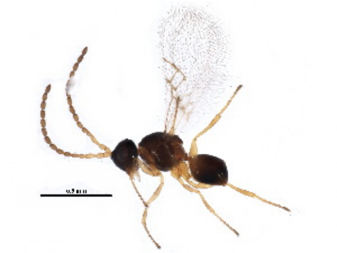

BIOUG22723-G02 [Lateral]
Figitidae
Family: Figitidae
BIN URI: BOLD:ACJ1628

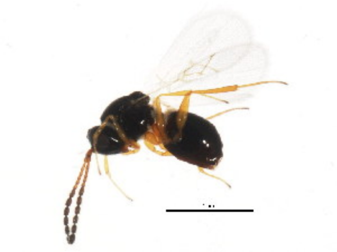

BIOUG01037-B08 [Lateral]
Eucoilinae
Family: Figitidae
BIN URI: BOLD:ABA7998

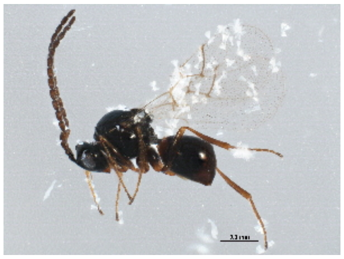

BIOUG11158-F03 [Lateral]
Eucoilinae
Family: Figitidae
BIN URI: BOLD:ACM0003

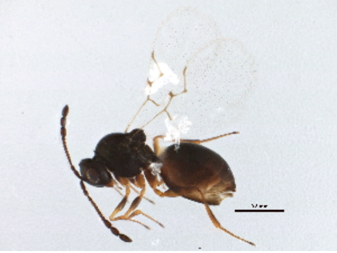

BIOUG08154-H07 [Lateral]
Ganaspis
Family: Figitidae
BIN URI: BOLD:ACD4392

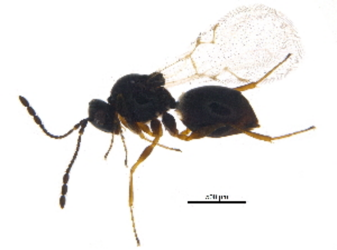

BIOUG22927-A05 [Lateral]
Figitidae
Family: Figitidae
BIN URI: BOLD:ACN5042

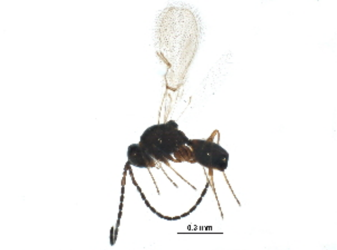

BIOUG13672-D04 [Lateral]
Hymenoptera

BIN URI: BOLD:ACO6056

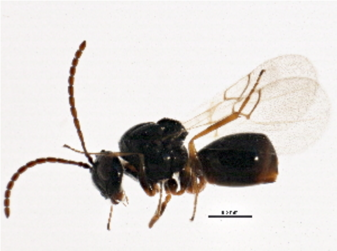

BIOUG05529-G09 [Lateral]
Eucoilinae
Family: Figitidae
BIN URI: BOLD:ABA5948

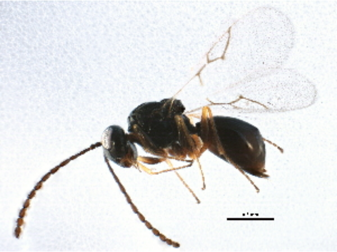

BIOUG11907-D05 [Lateral]
Hymenoptera

BIN URI: BOLD:ACM9237

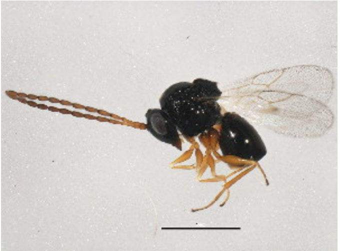

ASGLE-1124 [Lateral]
Eucoilinae
Family: Figitidae
BIN URI: BOLD:AAU8625

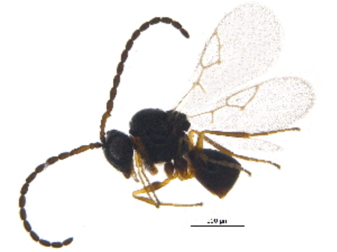

BIOUG22578-A11 [Lateral]
Figitidae
Family: Figitidae
BIN URI: BOLD:ACV4105

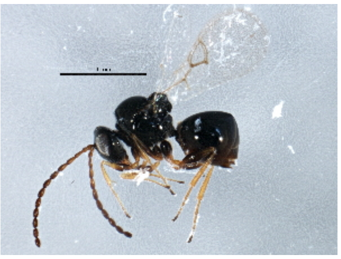

BIOUG03220-B12 [Lateral]
Eucoilinae
Family: Figitidae
BIN URI: BOLD:ACG3398

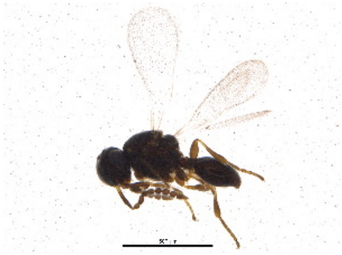

BIOUG12227-A11 [Lateral]
Hymenoptera

BIN URI: BOLD:ACI8542

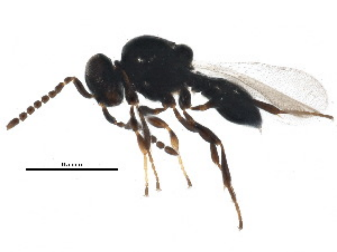

BIOUG22453-B12 [Lateral]
Platygastridae
Family: Platygastridae
BIN URI: BOLD:ACV2105

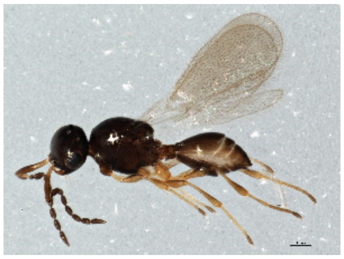

BIOUG06213-B12 [Lateral]
Platygastridae
Family: Platygastridae
BIN URI: BOLD:ACI3903

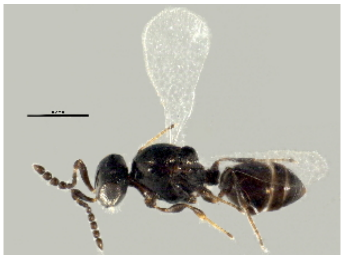

BIOUG07941-D09 [Lateral]
Platygastridae
Family: Platygastridae
BIN URI: BOLD:ACJ0034

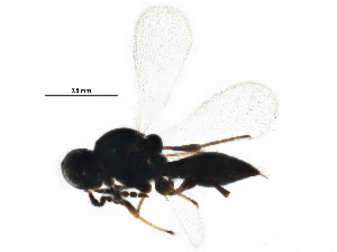

BIOUG22421-C07 [Lateral]
Platygastridae
Family: Platygastridae
BIN URI: BOLD:ABX8472

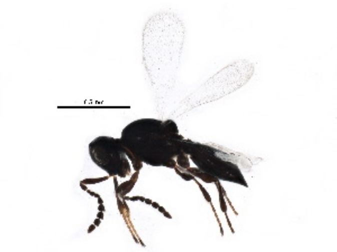

BIOUG22724-A06 [Lateral]
Platygastridae
Family: Platygastridae
BIN URI: BOLD:ACV5289

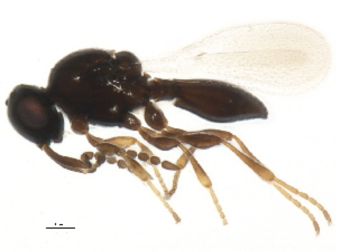

BIOUG01501-G04 [Lateral]
Platygaster variabilis
Family: Platygastridae
BIN URI: BOLD:ABW3242

IMAGE NOT AVAILABLE

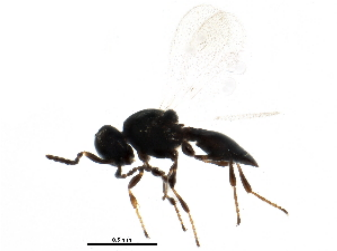

BIOUG22927-B08 [Lateral]
Platygastridae
Family: Platygastridae
BIN URI: BOLD:ACB3267

BIOUG22464-F01
Platygastridae
Family: Platygastridae

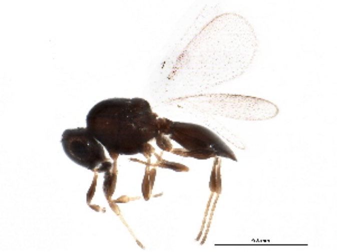

BIOUG22931-H09 [Lateral]
Platygastridae
Family: Platygastridae
BIN URI: BOLD:ACV5870

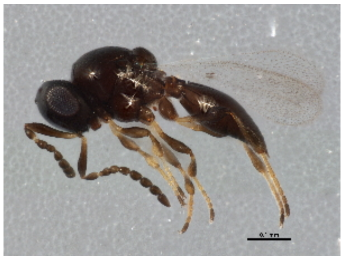

BIOUG10331-C03 [Lateral]
Platygastridae
Family: Platygastridae
BIN URI: BOLD:ACL7730

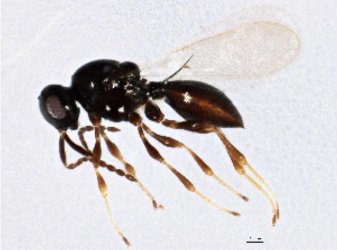

BIOUG01668-F08 [Lateral]
Platygastridae
Family: Platygastridae
BIN URI: BOLD:AAZ3286

IMAGE NOT AVAILABLE

BIOUG22420-H11
Platygastridae
Family: Platygastridae

IMAGE NOT AVAILABLE

BIOUG22420-B02
Platygastridae
Family: Platygastridae

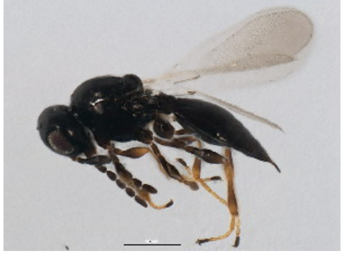

ASGLE-0350 [Lateral]
Hymenoptera

BIN URI: BOLD:AAU8342

IMAGE NOT AVAILABLE

BIOUG23074-G07
Leptacis
Family: Platygastridae

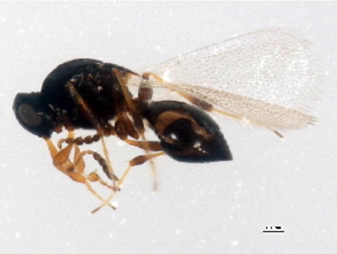

10PHMAL-0569 [Lateral]
Leptacis
Family: Platygastridae
BIN URI: BOLD:ACK4473

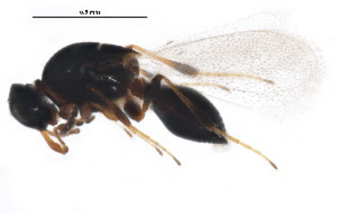

BIOUG22294-G01 [Lateral]
Leptacis
Family: Platygastridae
BIN URI: BOLD:AAU8358

IMAGE NOT AVAILABLE

BIOUG22723-F09
Leptacis
Family: Platygastridae

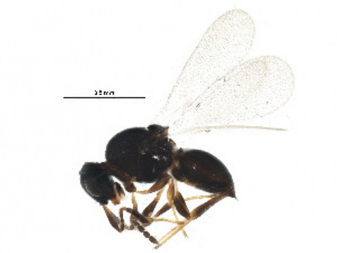

BIOUG22294-A09 [Lateral]
Aceroteta
Family: Platygastridae
BIN URI: BOLD:AAU8455

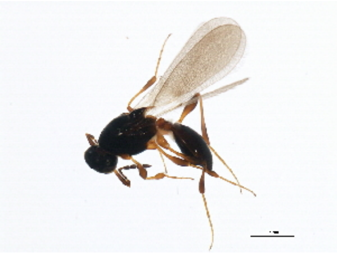

10PHMAL-0501 [Lateral]
Leptacis
Family: Platygastridae
BIN URI: BOLD:AAU8380

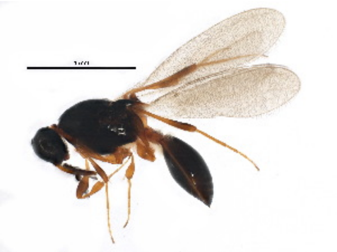

BIOUG22420-G11 [Lateral]
Leptacis
Family: Platygastridae
BIN URI: BOLD:AAU8376

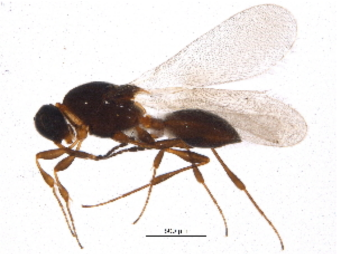

BIOUG15695-D04 [Lateral]
Platygastridae
Family: Platygastridae
BIN URI: BOLD:ACP9550

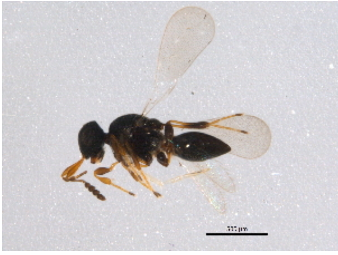

BIOUG07151-C07 [Lateral]
Platygastridae
Family: Platygastridae
BIN URI: BOLD:ABW3209

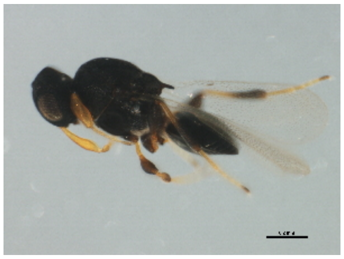

BIOUG01115-A03 [Lateral]
Platygastridae
Family: Platygastridae
BIN URI: BOLD:ABA7956

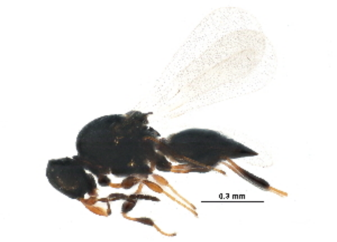

BIOUG13611-F08 [Lateral]
Platygastridae
Family: Platygastridae
BIN URI: BOLD:ACO5382

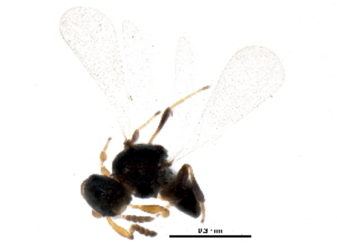

BIOUG22458-C12 [Lateral]
Synopeas
Family: Platygastridae
BIN URI: BOLD:AAU8619

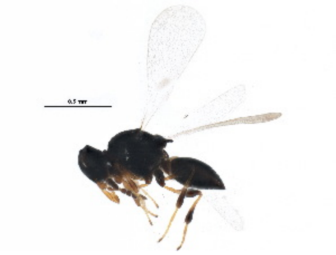

BIOUG22453-B05 [Lateral]
Synopeas
Family: Platygastridae
BIN URI: BOLD:ACV2257

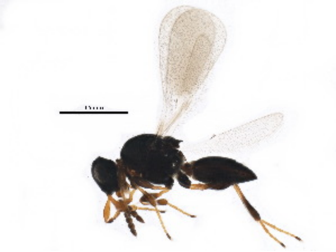

BIOUG22420-E12 [Lateral]
Synopeas
Family: Platygastridae
BIN URI: BOLD:ACV2297

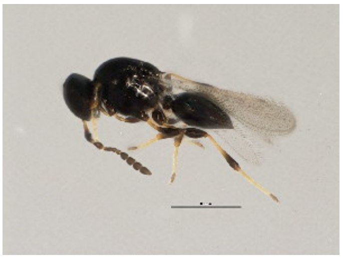

ASGLE-0326 [Lateral]
Synopeas
Family: Platygastridae
BIN URI: BOLD:AAU8571

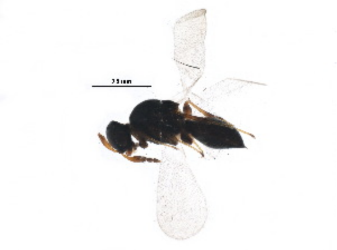

BIOUG22727-B10 [Lateral]
Synopeas
Family: Platygastridae
BIN URI: BOLD:ACV5896

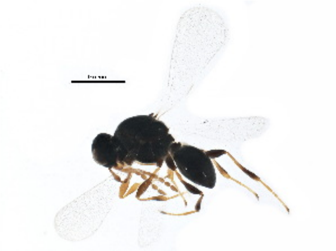

BIOUG22464-G06 [Lateral]
Synopeas
Family: Platygastridae
BIN URI: BOLD:ACV4251

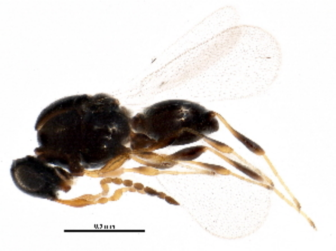

BIOUG22458-H10 [Lateral]
Synopeas
Family: Platygastridae
BIN URI: BOLD:ACV4801

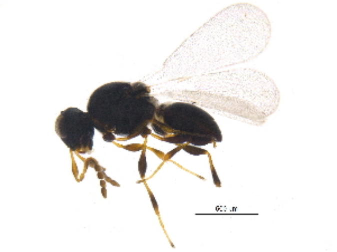

BIOUG22465-F08 [Lateral]
Synopeas
Family: Platygastridae
BIN URI: BOLD:ACV5947

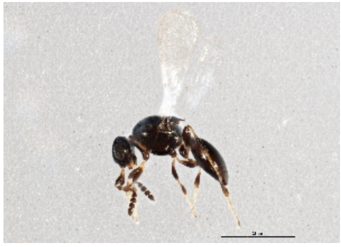

BIOUG03031-B08 [Lateral]
Synopeas pennsylvanicum
Family: Platygastridae
BIN URI: BOLD:ABA6127

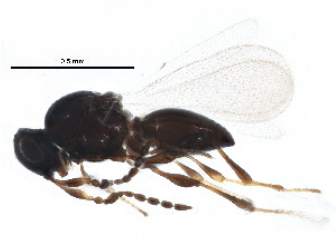

BIOUG10328-A12 [Lateral]
Platygastrinae
Family: Platygastridae
BIN URI: BOLD:ACL8696

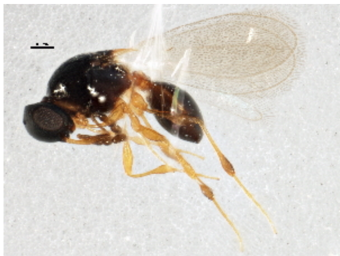

BIOUG02935-G12 [Lateral]
Leptacis
Family: Platygastridae
BIN URI: BOLD:ABA5944

IMAGE NOT AVAILABLE

BIOUG22727-G02
Leptacis
Family: Platygastridae

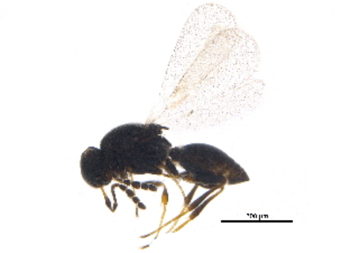

BIOUG22838-E12 [Lateral]
Platygastridae
Family: Platygastridae
BIN URI: BOLD:ACW0287

IMAGE NOT AVAILABLE

BIOUG22420-E07
Synopeas
Family: Platygastridae

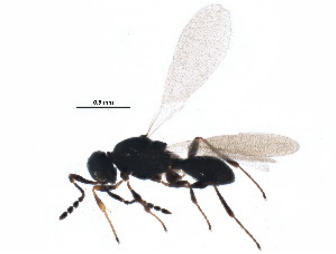

BIOUG22724-B04 [Lateral]
Platygastridae
Family: Platygastridae
BIN URI: BOLD:AAG7963

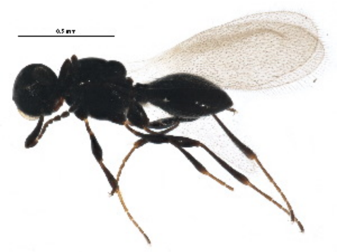

BIOUG22288-F08 [Lateral]
Platygastridae
Family: Platygastridae
BIN URI: BOLD:AAU8377

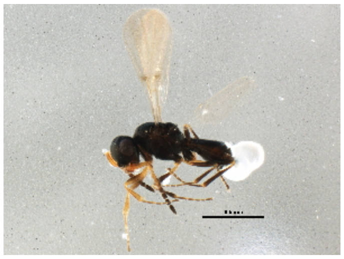

BIOUG08878-B10 [Lateral]
Platygastridae
Family: Platygastridae
BIN URI: BOLD:ACK5301

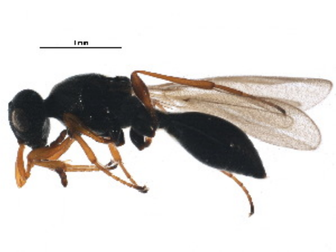

BIOUG22287-C03 [Lateral]
Platygastridae
Family: Platygastridae
BIN URI: BOLD:ACV2411

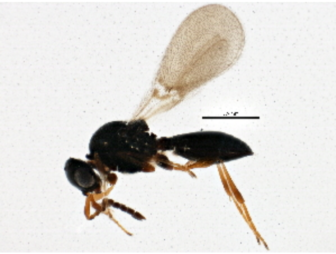

BIOUG05606-D11 [Lateral]
Trichacis
Family: Platygastridae
BIN URI: BOLD:AAU8203

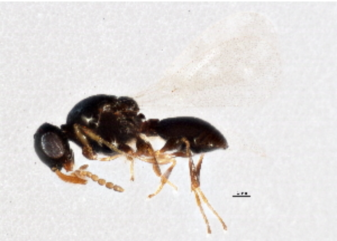

BIOUG03031-B10 [Lateral]
Platygastridae
Family: Platygastridae
BIN URI: BOLD:ACA7711

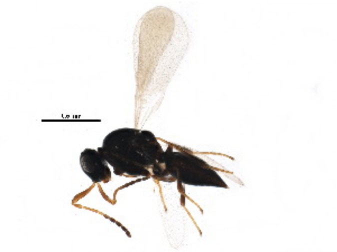

BIOUG22420-C09 [Lateral]
Platygastridae
Family: Platygastridae
BIN URI: BOLD:ACK5386

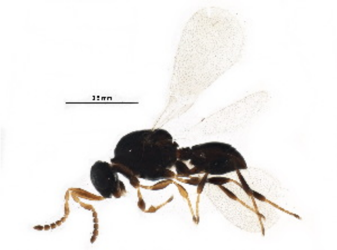

BIOUG22421-C04 [Lateral]
Platygastridae
Family: Platygastridae
BIN URI: BOLD:ACV2386

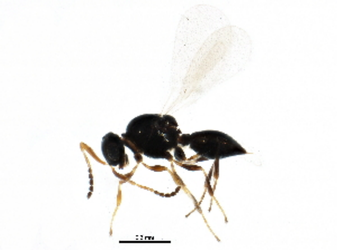

BIOUG22358-B08 [Lateral]
Platygastridae
Family: Platygastridae
BIN URI: BOLD:ACV3072

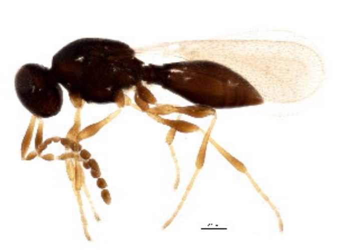

BIOUG01625-E05 [Lateral]
Platygastrinae
Family: Platygastridae
BIN URI: BOLD:ABW3169

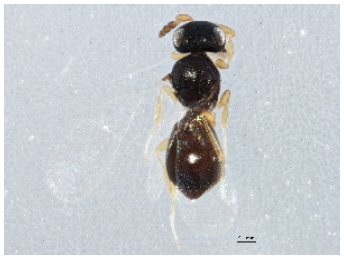

BIOUG01600-G11 [Dorsal]
Platygastridae
Family: Platygastridae
BIN URI: BOLD:ABW3140

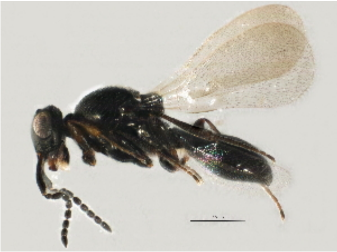

BIOUG08483-E09 [Lateral]
Platygastridae
Family: Platygastridae
BIN URI: BOLD:ACJ8807

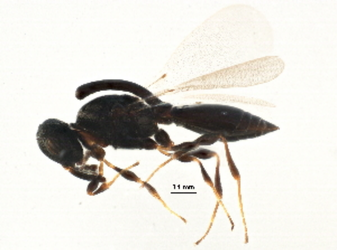

BIOUG08589-A08 [Lateral]
Platygastridae
Family: Platygastridae
BIN URI: BOLD:ACK1238

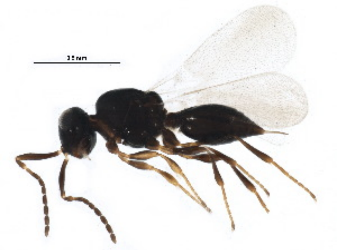

BIOUG22421-F11 [Lateral]
Platygastridae
Family: Platygastridae
BIN URI: BOLD:ACV2155

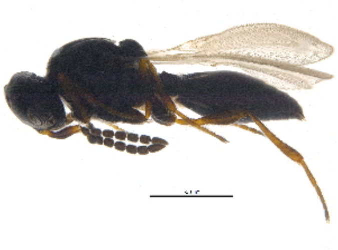

BIOUG21947-B05 [Lateral]
Platygastridae
Family: Platygastridae
BIN URI: BOLD:ACU4949

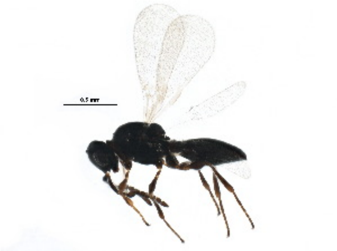

BIOUG22294-D08 [Lateral]
Platygastridae
Family: Platygastridae
BIN URI: BOLD:ACL4888

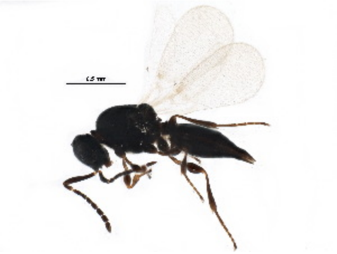

BIOUG22421-B08 [Lateral]
Platygaster
Family: Platygastridae
BIN URI: BOLD:ACV2372

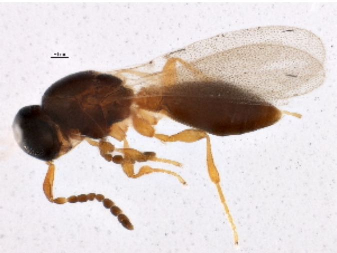

BIOUG03347-B10 [Lateral]
Platygastridae
Family: Platygastridae
BIN URI: BOLD:ABZ9638

IMAGE NOT AVAILABLE

BIOUG22727-E03
Platygastridae
Family: Platygastridae

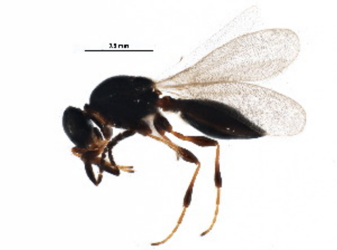

BIOUG22294-B04 [Lateral]
Platygastridae
Family: Platygastridae
BIN URI: BOLD:AAG7977

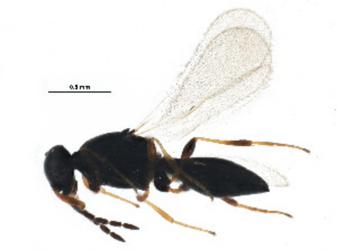

BIOUG21773-A01 [Lateral]
Platygastrinae
Family: Platygastridae
BIN URI: BOLD:ACF9268

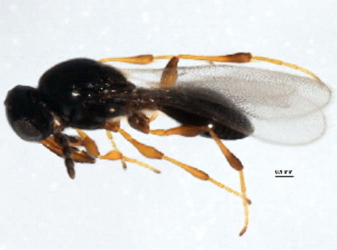

ASGLE2-0251 [Lateral]
Hymenoptera

BIN URI: BOLD:AAU8566

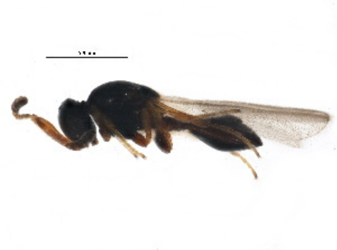

BIOUG20970-F08 [Lateral]
Hymenoptera

BIN URI: BOLD:ACU8608

IMAGE NOT AVAILABLE

BIOUG23074-D09
Platygastridae
Family: Platygastridae

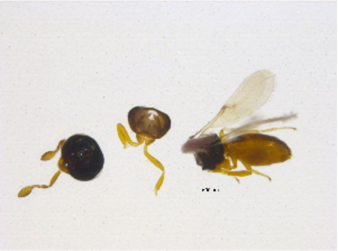

BIOUG03408-E04 [Lateral]
Platygastridae
Family: Platygastridae
BIN URI: BOLD:ACC1046

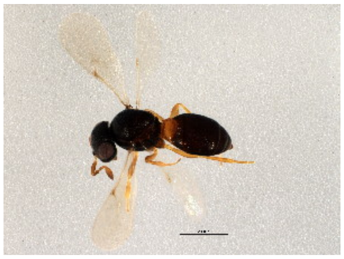

BIOUG01665-A10 [Lateral]
Scelioninae
Family: Platygastridae
BIN URI: BOLD:ABA6154

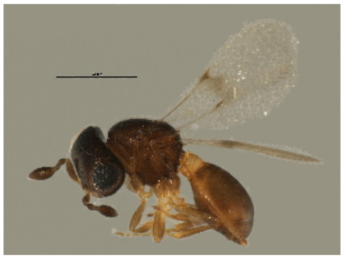

BIOUG07004-D12 [Lateral]
Platygastridae
Family: Platygastridae
BIN URI: BOLD:ACH3788

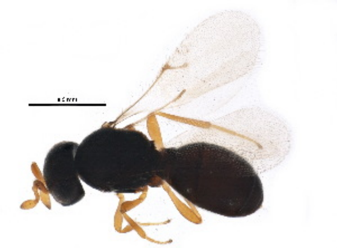

BIOUG22626-B06 [Lateral]
Platygastridae
Family: Platygastridae
BIN URI: BOLD:ACV5042

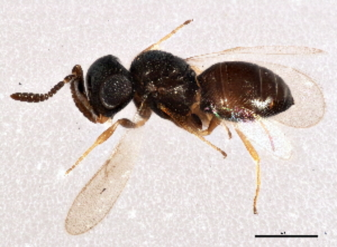

BIOUG05645-A03 [Lateral]
Platygastridae
Family: Platygastridae
BIN URI: BOLD:ACF7191

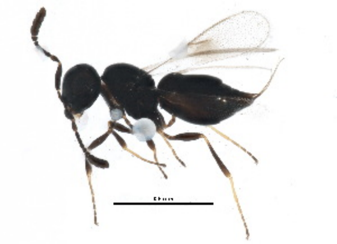

BIOUG22453-E04 [Lateral]
Platygastridae
Family: Platygastridae
BIN URI: BOLD:ACV1908

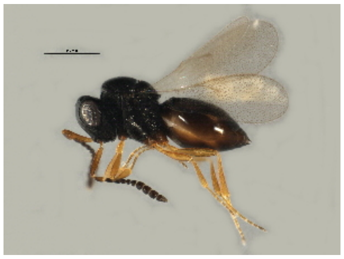

BIOUG08724-E06 [Lateral]
Platygastridae
Family: Platygastridae
BIN URI: BOLD:ACK2828

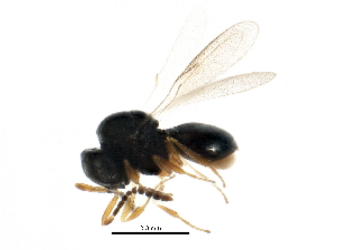

BIOUG23074-B10 [Lateral]
Platygastridae
Family: Platygastridae
BIN URI: BOLD:ACV5862

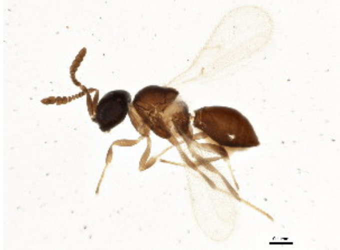

BIOUG01605-H08 [Lateral]
Telenomus
Family: Platygastridae
BIN URI: BOLD:ABW3189

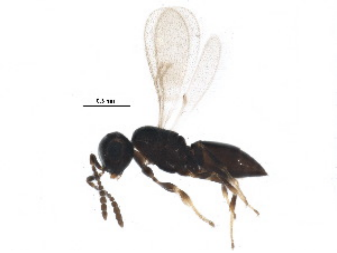

BIOUG22464-F05 [Lateral]
Scelioninae
Family: Platygastridae
BIN URI: BOLD:AAG7898

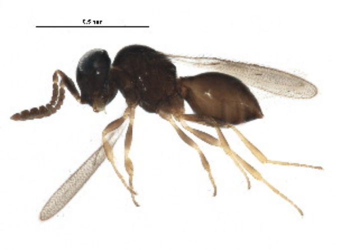

BIOUG22453-A05 [Lateral]
Telenomus
Family: Platygastridae
BIN URI: BOLD:ACV2493

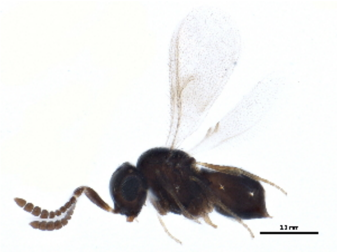

BIOUG11469-H08 [Lateral]
Scelioninae
Family: Platygastridae
BIN URI: BOLD:ACA7752

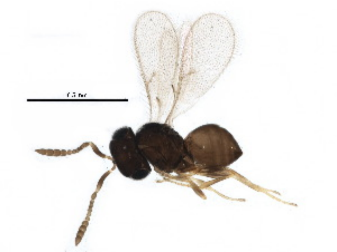

BIOUG22421-F10 [Lateral]
Scelioninae
Family: Platygastridae
BIN URI: BOLD:AAG7897

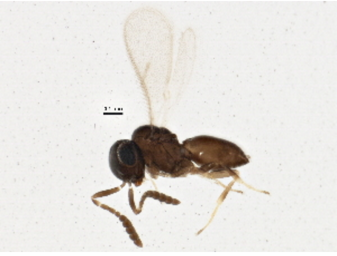

BIOUG03770-G03 [Lateral]
Scelioninae
Family: Platygastridae
BIN URI: BOLD:ABA6117

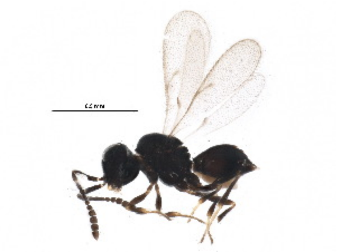

BIOUG21773-A10 [Lateral]
Platygastridae
Family: Platygastridae
BIN URI: BOLD:ACC5761

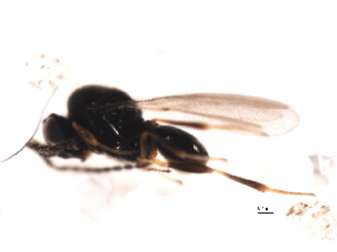

09BBEHY-1571 [Lateral]
Telenomus
Family: Platygastridae
BIN URI: BOLD:AAN7574

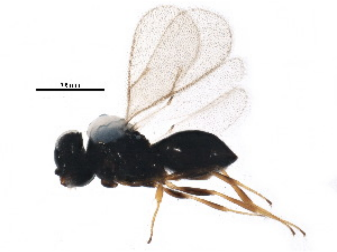

BIOUG22931-G05 [Lateral]
Telenomus
Family: Platygastridae
BIN URI: BOLD:ABZ8566

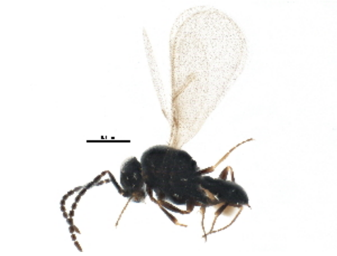

BIOUG08532-F10 [Lateral]
Platygastridae
Family: Platygastridae
BIN URI: BOLD:ABW3231

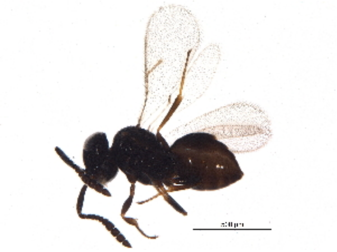

BIOUG09932-G08 [Lateral]
Platygastridae
Family: Platygastridae
BIN URI: BOLD:ACL3059

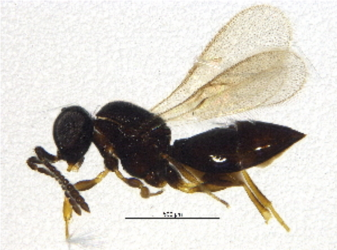

BIOUG05001-A11 [Lateral]
Telenomus
Family: Platygastridae
BIN URI: BOLD:ACJ5136

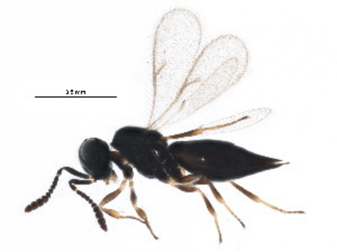

BIOUG22294-F06 [Lateral]
Telenomus
Family: Platygastridae
BIN URI: BOLD:ACV3598

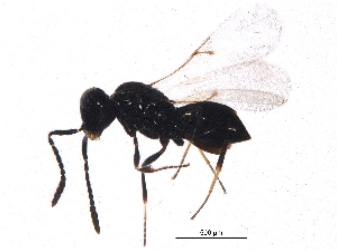

BIOUG16984-F10 [Lateral]
Platygastridae
Family: Platygastridae
BIN URI: BOLD:ACR4826

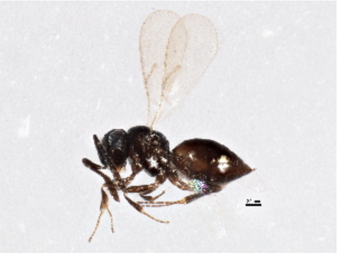

BIOUG05679-A01 [Lateral]
Telenomus
Family: Platygastridae
BIN URI: BOLD:AAU8457

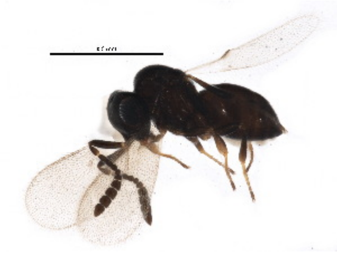

BIOUG22723-F03 [Lateral]
Telenomus
Family: Platygastridae
BIN URI: BOLD:AAU8347

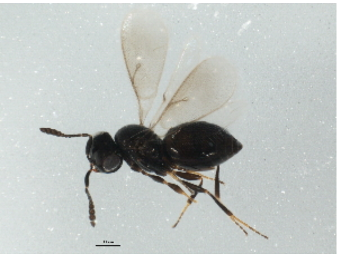

BIOUG05591-E06 [Lateral]
Telenomus
Family: Platygastridae
BIN URI: BOLD:ABY4994

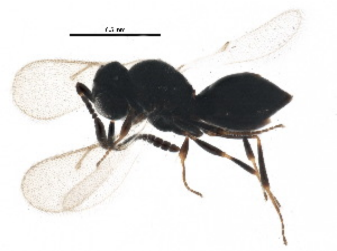

BIOUG22294-F08 [Lateral]
Platygastridae
Family: Platygastridae
BIN URI: BOLD:ACV4221

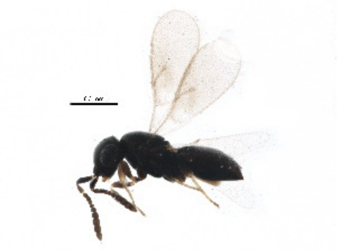

BIOUG21773-B04 [Lateral]
Scelioninae
Family: Platygastridae
BIN URI: BOLD:ACE0660

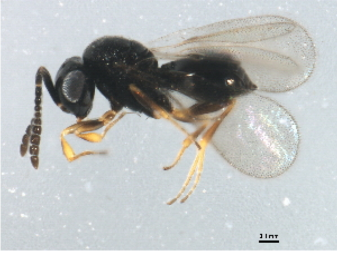

BIOUG01029-F10 [Lateral]
Platygastridae
Family: Platygastridae
BIN URI: BOLD:ABA5945

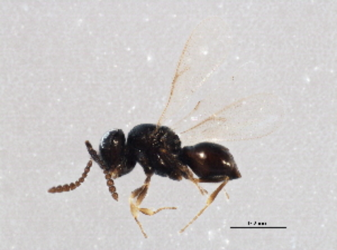

BIOUG10661-D01 [Lateral]
Platygastridae
Family: Platygastridae
BIN URI: BOLD:ACL8091

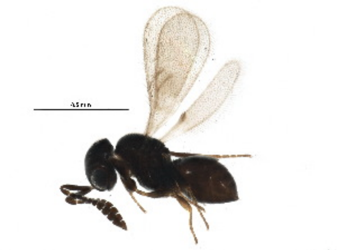

BIOUG22294-A08 [Lateral]
Telenomus
Family: Platygastridae
BIN URI: BOLD:AAZ3289

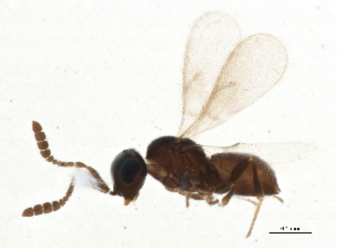

BIOUG03844-F04 [Lateral]
Platygastridae
Family: Platygastridae
BIN URI: BOLD:ACB0797

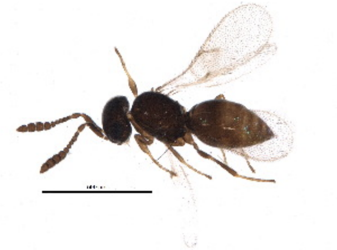

BIOUG20316-H11 [Lateral]
Telenomus
Family: Platygastridae
BIN URI: BOLD:ACU0247

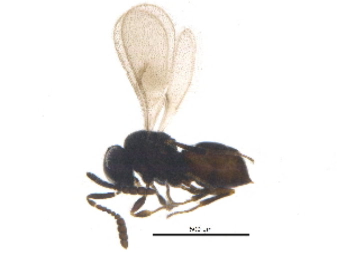

BIOUG21771-G08 [Lateral]
Telenomus
Family: Platygastridae
BIN URI: BOLD:ACC7846

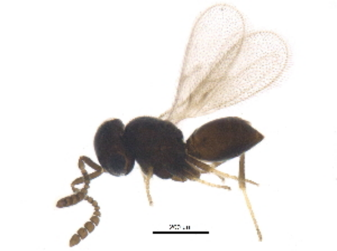

BIOUG21771-F07 [Lateral]
Telenomus
Family: Platygastridae

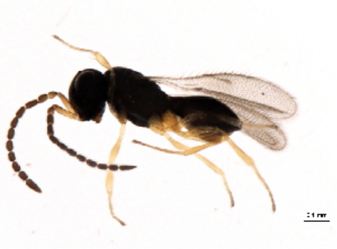

10BBHYM-1526 [Lateral]
Telenomus
Family: Platygastridae
BIN URI: BOLD:AAN8025

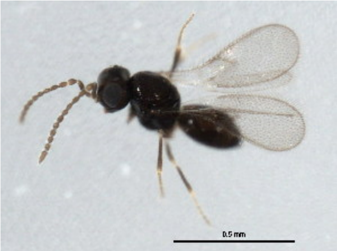

10BBCHY-1191 [Lateral]
Telenomus
Family: Platygastridae
BIN URI: BOLD:AAU9339

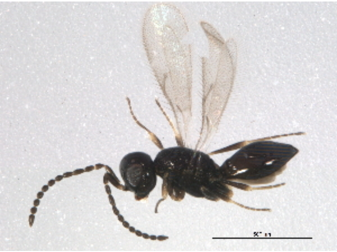

BIOUG08491-D05 [Lateral]
Scelioninae
Family: Platygastridae
BIN URI: BOLD:ABW3159

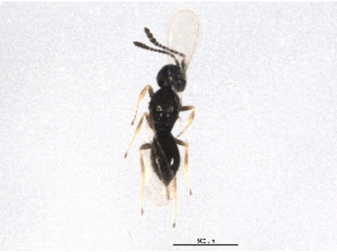

BIOUG08683-D03 [Dorsal]
Platygastridae
Family: Platygastridae
BIN URI: BOLD:ACJ0111

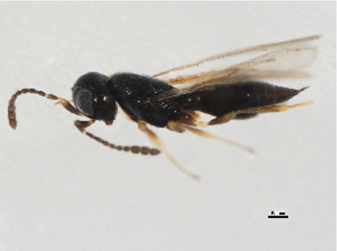

BIOUG00836-D06 [Lateral]
Telenomus
Family: Platygastridae
BIN URI: BOLD:AAU4881

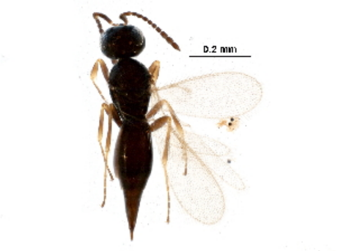

BIOUG10805-D10 [Dorsal]
Telenomus
Family: Platygastridae
BIN URI: BOLD:ACL8683

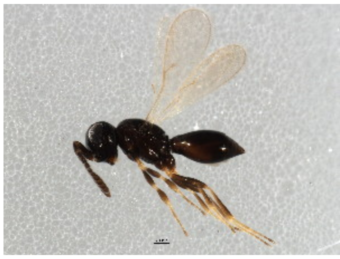

BIOUG01661-F08 [Lateral]
Telenomus
Family: Platygastridae
BIN URI: BOLD:ABA6298

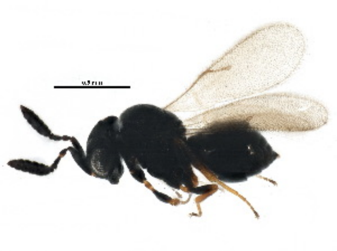

BIOUG22421-D05 [Lateral]
Trissolcus
Family: Platygastridae
BIN URI: BOLD:ACJ6984

IMAGE NOT AVAILABLE

BIOUG22464-B11
Telenomus
Family: Platygastridae

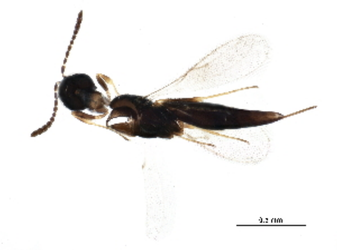

BIOUG23320-H05 [Lateral]
Platygastridae
Family: Platygastridae
BIN URI: BOLD:ACW1217

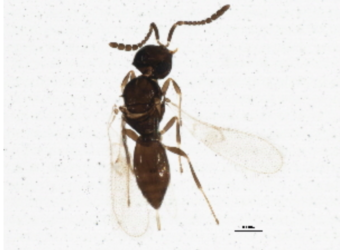

BIOUG02984-A02 [Dorsal]
Scelioninae
Family: Platygastridae
BIN URI: BOLD:AAU4830

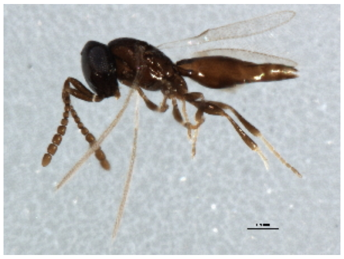

BIOUG01657-C10 [Lateral]
Telenomus
Family: Platygastridae
BIN URI: BOLD:ABA6265

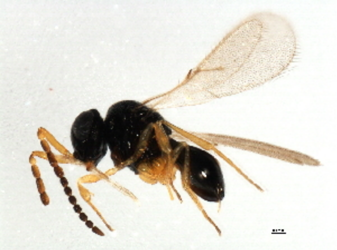

BIOUG00837-F12 [Lateral]
Telenomus
Family: Platygastridae
BIN URI: BOLD:AAY9192

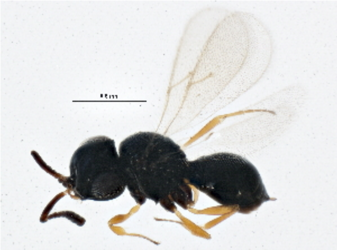

BIOUG03445-B02 [Lateral]
Telenomus podisi
Family: Platygastridae
BIN URI: BOLD:ACB2589

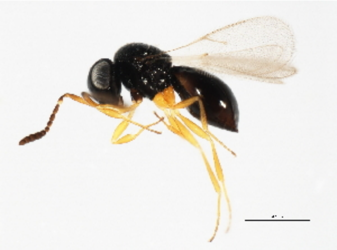

BIOUG00837-G02 [Lateral]
Telenomus podisi
Family: Platygastridae
BIN URI: BOLD:AAG7891

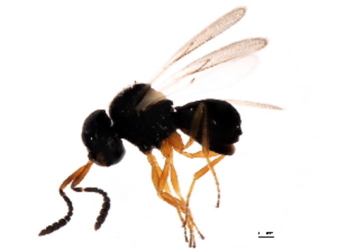

10BBHYM-1426 [Lateral]
Telenomus
Family: Platygastridae
BIN URI: BOLD:ACM2546

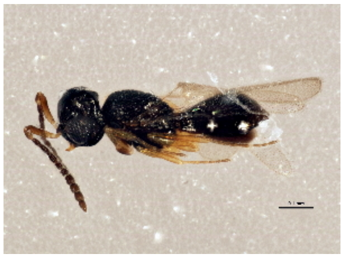

BIOUG03368-F09 [Lateral]
Platygastridae
Family: Platygastridae
BIN URI: BOLD:ABA5995

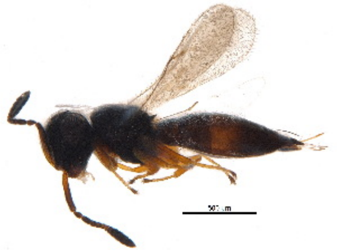

BIOUG21221-G08 [Lateral]
Hymenoptera

BIN URI: BOLD:ACV0111

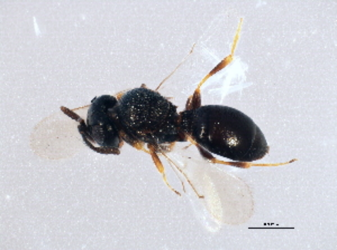

BIOUG06454-H10 [Lateral]
Platygastridae
Family: Platygastridae
BIN URI: BOLD:ACI5029

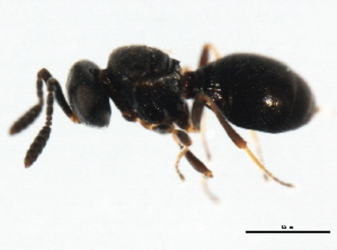

TWPARA-1026 [Lateral]
Platygastridae
Family: Platygastridae
BIN URI: BOLD:AAG7886

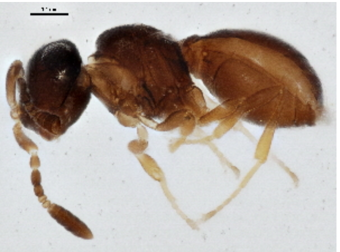

BIOUG02642-B02 [Lateral]
Trimorus
Family: Platygastridae
BIN URI: BOLD:ABY2758

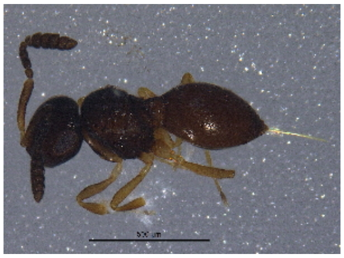

BIOUG08038-E04 [Lateral]
Trimorus
Family: Platygastridae
BIN URI: BOLD:ACI9917

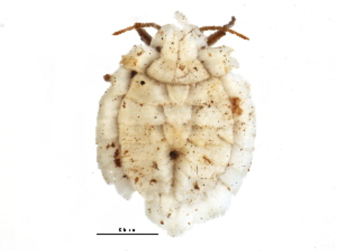

BIOUG24001-F12 [Dorsal]
Orthezidae
Family: Orthezidae

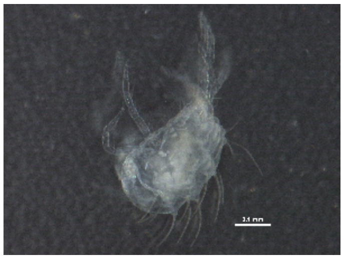

BIOUG16050-A05 [Dorsal]
Panonychus
Family: Tetranychidae
BIN URI: BOLD:ACK1507

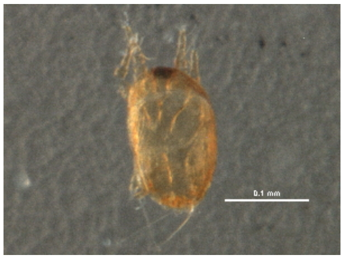

BIOUG10411-D05 [Dorsal]
Tetranychidae
Family: Tetranychidae
BIN URI: BOLD:ACL6075

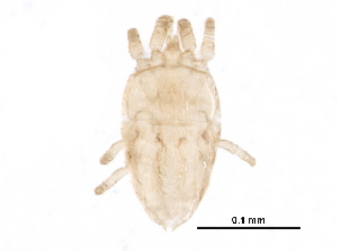

BIOUG21884-B03 [Dorsal]
Tetranychidae
Family: Tetranychidae
BIN URI: BOLD:ACV5964

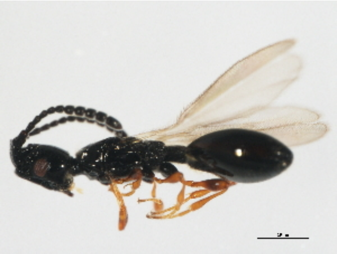

10PHMAL-0745 [Lateral]
Diapriidae
Family: Diapriidae
BIN URI: BOLD:AAM7500

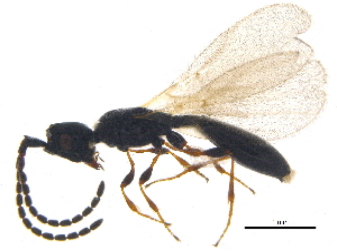

BIOUG21771-G02 [Lateral]
Diapriidae
Family: Diapriidae
BIN URI: BOLD:ACM6785

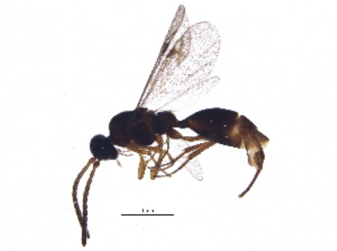

BIOUG13244-A10 [Lateral]
Proctotrupidae
Family: Proctotrupidae
BIN URI: BOLD:ACN9830

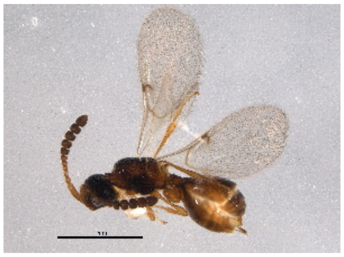

BIOUG06449-D02 [Lateral]
Diapriidae
Family: Diapriidae
BIN URI: BOLD:ACI4484

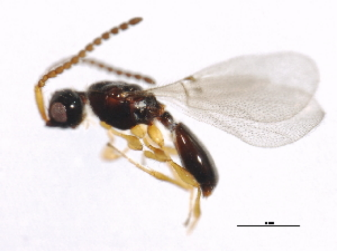

09BBEHY-0900 [Lateral]
Diapriidae
Family: Diapriidae
BIN URI: BOLD:ACC8030

IMAGE NOT AVAILABLE

BIOUG22931-H02
Diapriidae
Family: Diapriidae

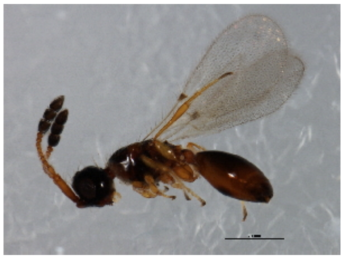

09BBEHY-1795 [Lateral]
Diapriidae
Family: Diapriidae
BIN URI: BOLD:AAM7457

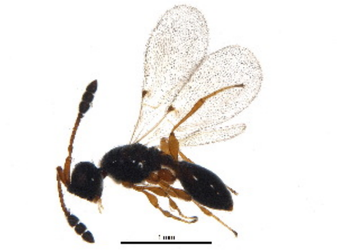

BIOUG24007-E07 [Lateral]
Diapriidae
Family: Diapriidae

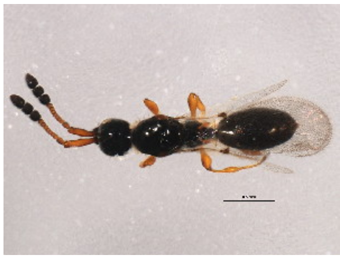

10BBCHY-2997 [Dorsal]
Diapriidae
Family: Diapriidae
BIN URI: BOLD:AAN7572

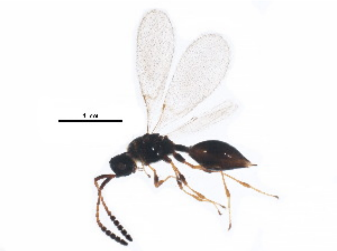

BIOUG22294-G09 [Lateral]
Diapriidae
Family: Diapriidae
BIN URI: BOLD:AAU8803

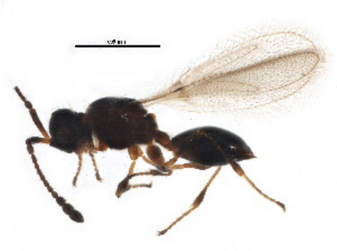

BIOUG22420-B07 [Lateral]
Diapriidae
Family: Diapriidae
BIN URI: BOLD:ACF9546

IMAGE NOT AVAILABLE

BIOUG21888-E06
Trichopria
Family: Diapriidae

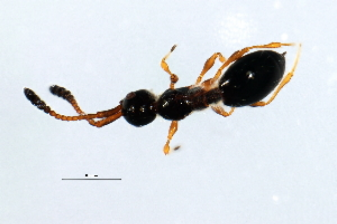

08BBHYM-1324 [Dorsal]
Diapriidae
Family: Diapriidae
BIN URI: BOLD:AAG7956

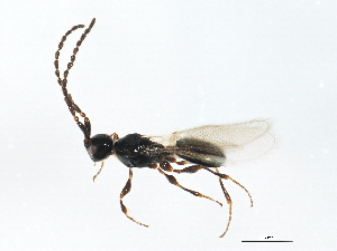

ASGLE-1229 [Lateral]
Hymenoptera

BIN URI: BOLD:AAM7487

IMAGE NOT AVAILABLE

BIOUG22458-G06
Diapriidae
Family: Diapriidae

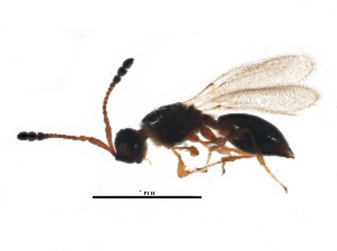

BIOUG22420-A08 [Lateral]
Diapriidae
Family: Diapriidae
BIN URI: BOLD:AAG7899

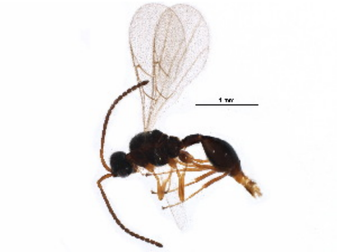

BIOUG22420-A10 [Lateral]
Diapriidae
Family: Diapriidae
BIN URI: BOLD:ACK2907

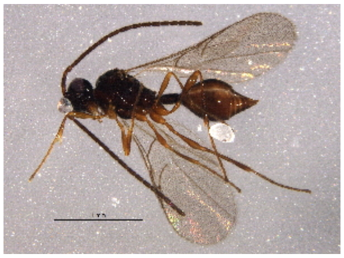

BIOUG06285-G12 [Lateral]
Diapriidae
Family: Diapriidae
BIN URI: BOLD:ACM1952

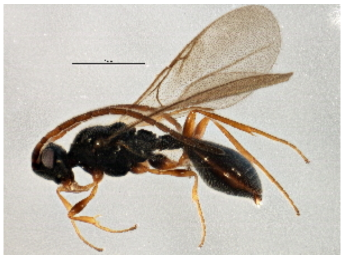

BIOUG05828-D09 [Lateral]
Diapriidae
Family: Diapriidae
BIN URI: BOLD:AAU8802

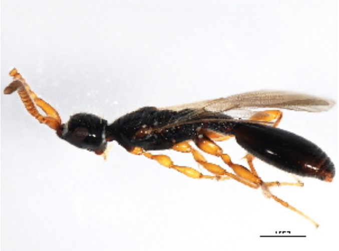

ASGLE2-0240 [Lateral]
Hymenoptera

BIN URI: BOLD:AAU8736

IMAGE NOT AVAILABLE

BIOUG22862-H11
Diapriidae
Family: Diapriidae

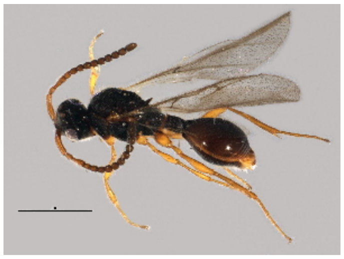

BIOUG12297-G10 [Lateral]
Diapriidae
Family: Diapriidae
BIN URI: BOLD:ACN0910

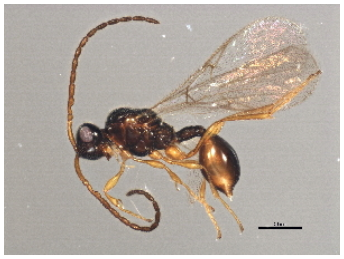

BIOUG04477-A09 [Lateral]
Diapriidae
Family: Diapriidae
BIN URI: BOLD:ABZ9639

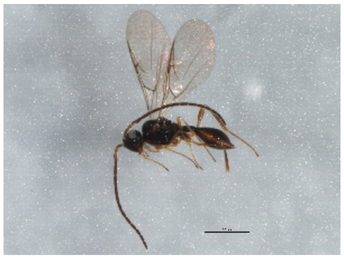

BIOUG04120-C05 [Lateral]
Diapriidae
Family: Diapriidae
BIN URI: BOLD:AAU9080

IMAGE NOT AVAILABLE

BIOUG22420-D09
Diapriidae
Family: Diapriidae

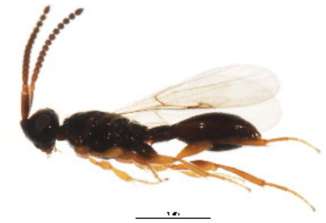

BIOUG01049-F07 [Lateral]
Diapriidae
Family: Diapriidae
BIN URI: BOLD:ABA6056

IMAGE NOT AVAILABLE

BIOUG22723-B03
Diapriidae
Family: Diapriidae

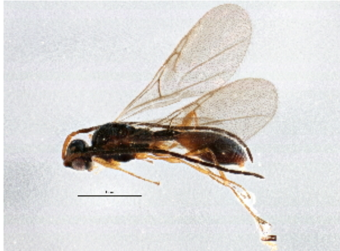

BIOUG02921-F03 [Lateral]
Diapriidae
Family: Diapriidae
BIN URI: BOLD:AAU8821

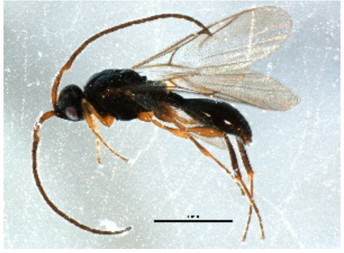

BIOUG10408-B11 [Lateral]
Diapriidae
Family: Diapriidae
BIN URI: BOLD:ACL5283

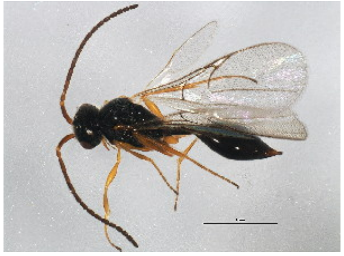

ASGLE-0930 [Dorsal]
Hymenoptera

BIN URI: BOLD:AAU8448

IMAGE NOT AVAILABLE

BIOUG22421-F09
Diapriidae
Family: Diapriidae

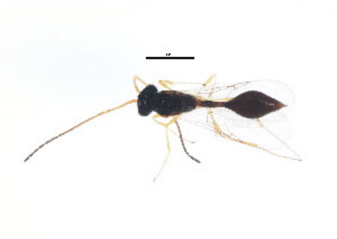

BIOUG00857-C12 [Dorsal]
Diapriidae
Family: Diapriidae
BIN URI: BOLD:AAN7559

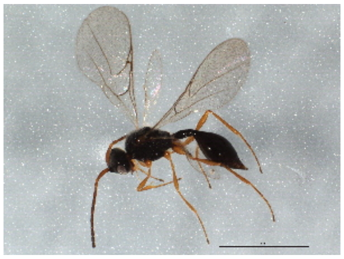

BIOUG05520-D03 [Lateral]
Diapriidae
Family: Diapriidae
BIN URI: BOLD:AAU8399

IMAGE NOT AVAILABLE

BIOUG22727-A10
Diapriidae
Family: Diapriidae

IMAGE NOT AVAILABLE

BIOUG22870-F04
Diapriidae
Family: Diapriidae

IMAGE NOT AVAILABLE

BIOUG22867-C04
Diapriidae
Family: Diapriidae

IMAGE NOT AVAILABLE

BIOUG22872-G12
Diapriidae
Family: Diapriidae

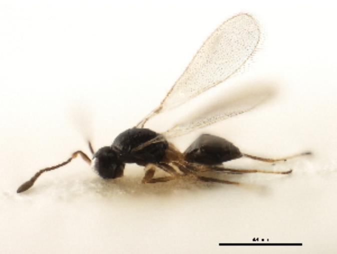

CNCHYM 07498 [Lateral]
Ooctonus silvensis
Family: Mymaridae
BIN URI: BOLD:AAN7553

IMAGE NOT AVAILABLE

BIOUG22723-B11
Ooctonus silvensis
Family: Mymaridae

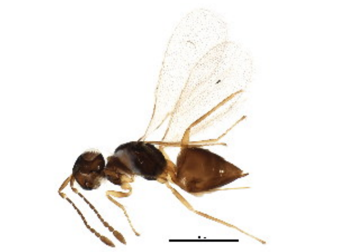

BIOUG01625-G04 [Lateral]
Ooctonus
Family: Mymaridae
BIN URI: BOLD:AAY9180

IMAGE NOT AVAILABLE

BIOUG22421-C06
Ooctonus
Family: Mymaridae

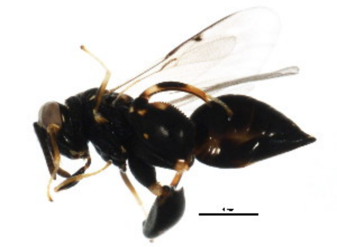

BIOUG01285-G08 [Lateral]
Conura albifrons
Family: Chalcididae
BIN URI: BOLD:AAG8371

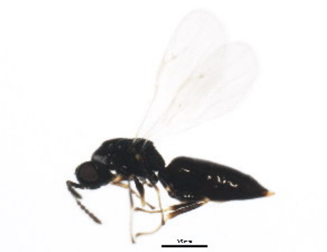

BIOUG01031-G09 [Lateral]
Eurytomidae
Family: Eurytomidae
BIN URI: BOLD:ABA5950

IMAGE NOT AVAILABLE

BIOUG22458-C08
Eurytomidae
Family: Eurytomidae

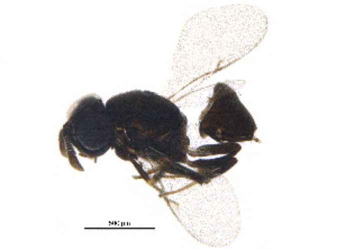

BIOUG22578-A05 [Lateral]
Hymenoptera

BIN URI: BOLD:ACV3733

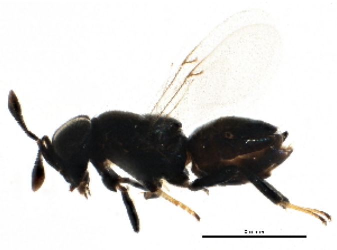

BIOUG22927-C01 [Lateral]
Hymenoptera

BIN URI: BOLD:ACV5208

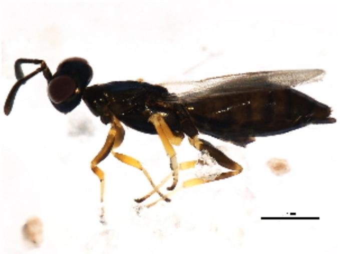

10BBHYM-0552 [Lateral]
Hymenoptera

BIN URI: BOLD:AAN7767

IMAGE NOT AVAILABLE

BIOUG22862-A01
Aphelinidae
Family: Aphelinidae

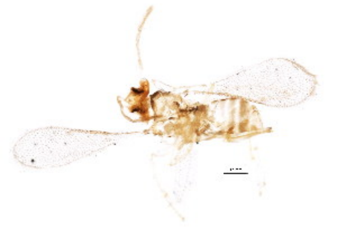

BIOUG01757-B10 [Lateral]
Aphelinidae
Family: Aphelinidae
BIN URI: BOLD:ABV2721

IMAGE NOT AVAILABLE

BIOUG23074-H09
Aphelinidae
Family: Aphelinidae

IMAGE NOT AVAILABLE

BIOUG23085-H04
Aphelinidae
Family: Aphelinidae

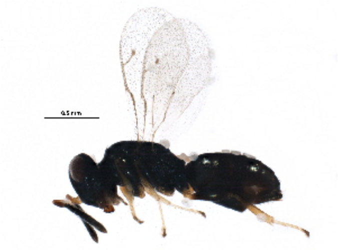

BIOUG24009-D07 [Lateral]
Pteromalidae
Family: Pteromalidae

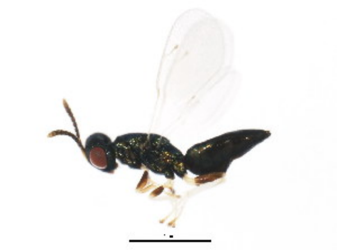

BIOUG01035-D09 [Lateral]
Pteromalidae
Family: Pteromalidae
BIN URI: BOLD:ABA5961

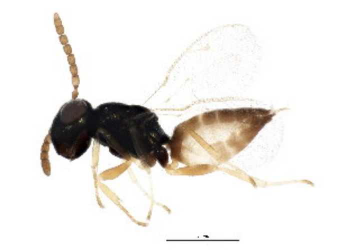

BIOUG01625-F04 [Lateral]
Pteromalidae
Family: Pteromalidae
BIN URI: BOLD:AAG7969

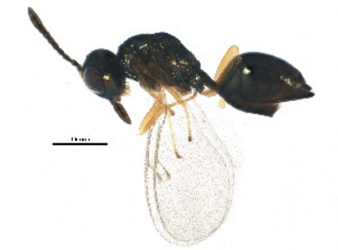

BIOUG22464-H09 [Lateral]
Pteromalidae
Family: Pteromalidae
BIN URI: BOLD:ACD0889

IMAGE NOT AVAILABLE

BIOUG21888-A02
Pteromalidae
Family: Pteromalidae

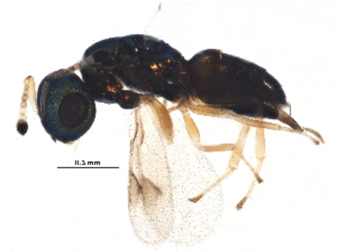

BIOUG07356-A10 [Lateral]
Mesopolobus bruchophagi
Family: Pteromalidae
BIN URI: BOLD:ACL4975

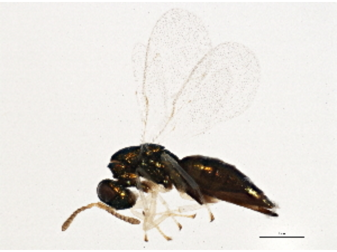

BIOUG03031-G11 [Lateral]
Pteromalidae
Family: Pteromalidae
BIN URI: BOLD:AAU9045

IMAGE NOT AVAILABLE

BIOUG22294-F11
Pteromalidae
Family: Pteromalidae

IMAGE NOT AVAILABLE

BIOUG22420-G12
Pteromalidae
Family: Pteromalidae

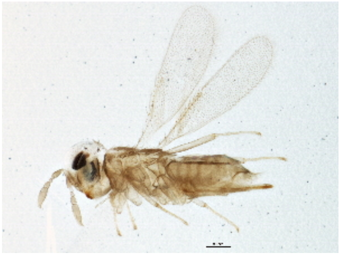

BIOUG05962-B02 [Lateral]
Aphelinidae
Family: Aphelinidae
BIN URI: BOLD:AAZ5700

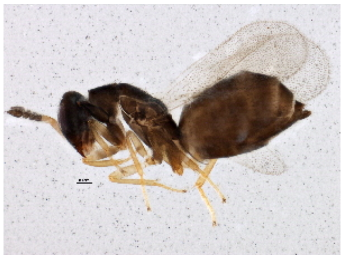

BIOUG03740-E11 [Lateral]
Eulophidae
Family: Eulophidae
BIN URI: BOLD:AAP6693

IMAGE NOT AVAILABLE

BIOUG22464-C12
Eulophidae
Family: Eulophidae

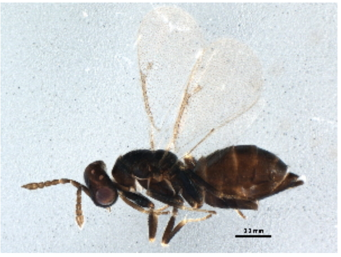

BIOUG08609-F04 [Lateral]
Tetrastichinae
Family: Eulophidae
BIN URI: BOLD:ACK1316

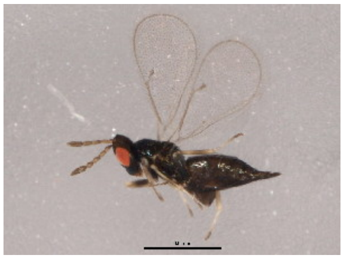

PCPP10-0539 [Lateral]
Aprostocetus sp.
Family: Eulophidae
BIN URI: BOLD:ACR4067

IMAGE NOT AVAILABLE

BIOUG22727-E10
Eulophidae
Family: Eulophidae

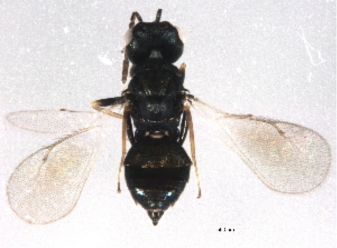

BIOUG08683-D06 [Dorsal]
Eulophidae
Family: Eulophidae
BIN URI: BOLD:ACJ7211

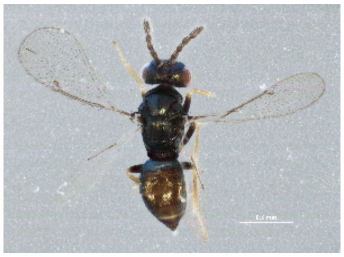

BIOUG11552-A11 [Dorsal]
Tetrastichinae
Family: Eulophidae
BIN URI: BOLD:ACM1389

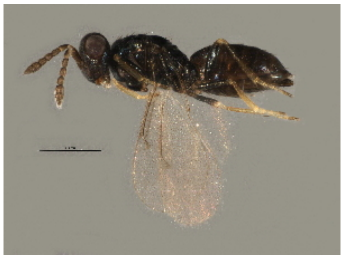

BIOUG11566-B09 [Lateral]
Tetrastichinae
Family: Eulophidae
BIN URI: BOLD:ACM0812

IMAGE NOT AVAILABLE

BIOUG22931-G08
Eulophidae
Family: Eulophidae

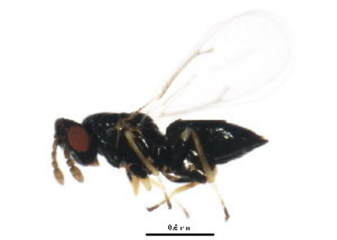

BIOUG01035-B08 [Lateral]
Aprostocetus SS0288
Family: Eulophidae
BIN URI: BOLD:ABA5968

IMAGE NOT AVAILABLE

BIOUG21888-E12
Eulophidae
Family: Eulophidae

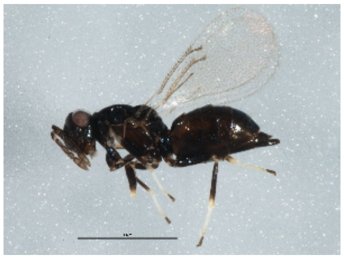

BIOUG02984-E11 [Lateral]
Eulophidae
Family: Eulophidae
BIN URI: BOLD:ACO5919

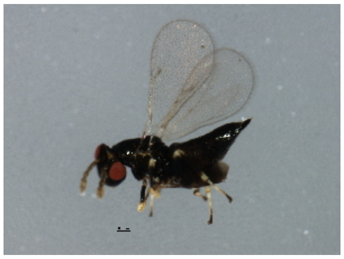

08BBHYM-1434 [Lateral]
Tetrastichinae
Family: Eulophidae
BIN URI: BOLD:AAG7984

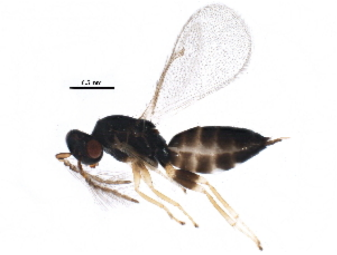

BIOUG24009-D09 [Lateral]
Aprostocetus
Family: Eulophidae

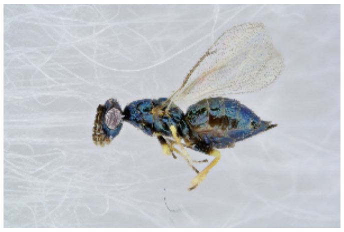

BC-ZSM-HYM-21585-H02 [Lateral]
Tetrastichinae
Family: Eulophidae
BIN URI: BOLD:AAG7954

IMAGE NOT AVAILABLE

BIOUG23085-C08
Baryscapus
Family: Eulophidae

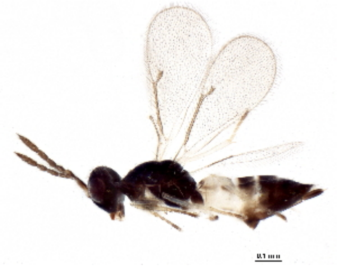

BIOUG24011-H05 [Lateral]
Eulophidae
Family: Eulophidae

IMAGE NOT AVAILABLE

BIOUG22724-D08
Eulophidae
Family: Eulophidae

IMAGE NOT AVAILABLE

BIOUG23074-E07
Eulophidae
Family: Eulophidae

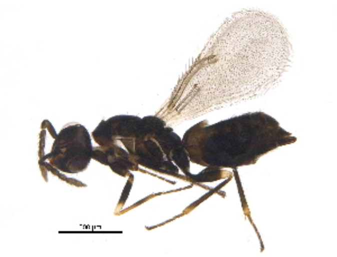

BIOUG22578-B06 [Lateral]
Eulophidae
Family: Eulophidae
BIN URI: BOLD:ACV4378

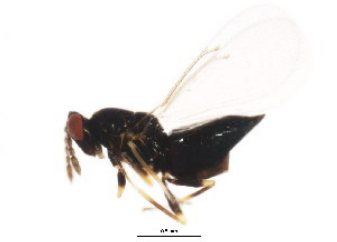

BIOUG01049-C07 [Lateral]
Eulophidae
Family: Eulophidae
BIN URI: BOLD:ABA6122

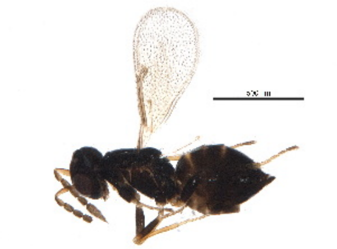

BIOUG24007-E02 [Lateral]
Eulophidae
Family: Eulophidae

IMAGE NOT AVAILABLE

BIOUG22927-B04
Eulophidae
Family: Eulophidae

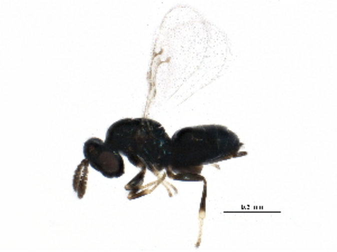

BIOUG24012-B01 [Lateral]
Eulophidae
Family: Eulophidae

IMAGE NOT AVAILABLE

BIOUG24012-B06
Eulophidae
Family: Eulophidae

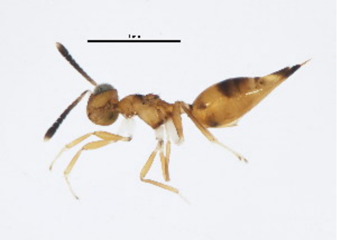

BIOUG00857-A10 [Lateral]
Eulophidae
Family: Eulophidae
BIN URI: BOLD:AAZ5709

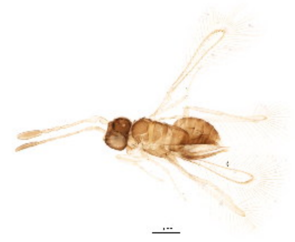

BIOUG01601-E12 [Lateral]
Mymaridae
Family: Mymaridae
BIN URI: BOLD:AAF1974

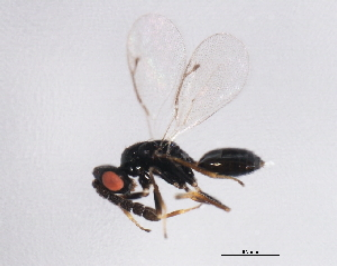

BIOUG01018-H01 [Lateral]
Tetrastichinae
Family: Eulophidae
BIN URI: BOLD:AAG3957

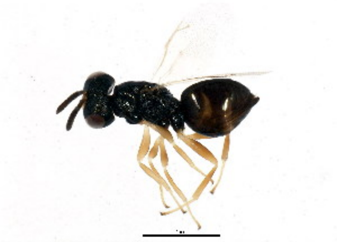

BIOUG01285-A10 [Lateral]
Aphytis
Family: Aphelinidae
BIN URI: BOLD:ABW3280

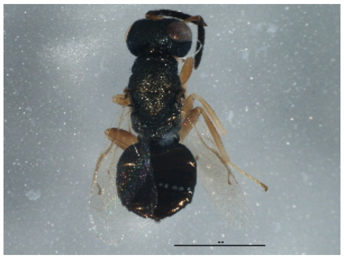

BIOUG05512-C06 [Dorsal]
Aphelinidae
Family: Aphelinidae
BIN URI: BOLD:ACF8608

IMAGE NOT AVAILABLE

BIOUG22723-D03
Aphelinidae
Family: Aphelinidae

IMAGE NOT AVAILABLE

BIOUG22728-D02
Eulophidae
Family: Eulophidae

IMAGE NOT AVAILABLE

BIOUG22723-B09
Eulophidae
Family: Eulophidae

IMAGE NOT AVAILABLE

BIOUG22728-D11
Tetrastichinae
Family: Eulophidae

IMAGE NOT AVAILABLE

BIOUG22728-H04
Eulophidae
Family: Eulophidae

IMAGE NOT AVAILABLE

BIOUG22453-C08
Eulophidae
Family: Eulophidae

IMAGE NOT AVAILABLE

BIOUG22453-B10
Eulophidae
Family: Eulophidae

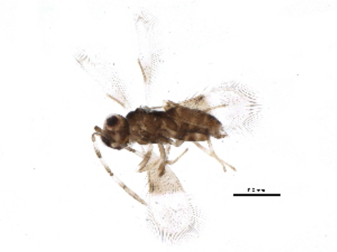

BIOUG07004-A03 [Lateral]
Trichogrammatidae
Family: Trichogrammatidae
BIN URI: BOLD:ACL8560

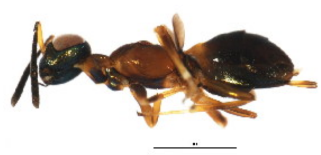

BIOUG01088-H01 [Lateral]
Eupelmus vesicularis
Family: Eupelmidae
BIN URI: BOLD:AAN8124

IMAGE NOT AVAILABLE

BIOUG22723-A08
Trichogramma
Family: Trichogrammatidae

IMAGE NOT AVAILABLE

BIOUG22927-D01
Trichogramma
Family: Trichogrammatidae

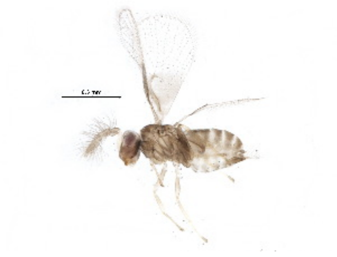

BIOUG21773-B03 [Lateral]
Trichogrammatidae
Family: Trichogrammatidae

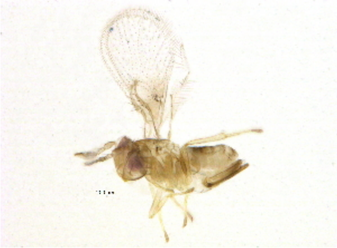

BIOUG02984-C11 [Lateral]
Trichogramma platneri
Family: Trichogrammatidae
BIN URI: BOLD:AAE0242

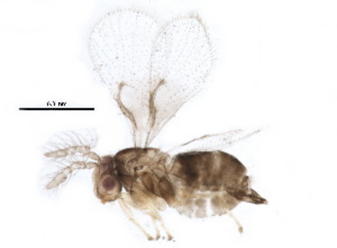

BIOUG22469-B04 [Lateral]
Trichogramma
Family: Trichogrammatidae
BIN URI: BOLD:ACV4457

IMAGE NOT AVAILABLE

BIOUG22727-C07
Trichogramma
Family: Trichogrammatidae

IMAGE NOT AVAILABLE

BIOUG23321-C04
Trichogramma
Family: Trichogrammatidae

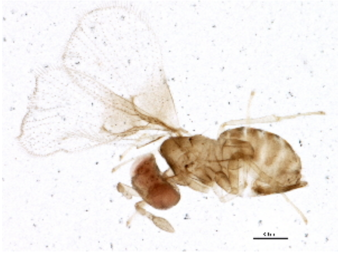

BIOUG01657-B03 [Lateral]
Trichogramma
Family: Trichogrammatidae
BIN URI: BOLD:ABA5903

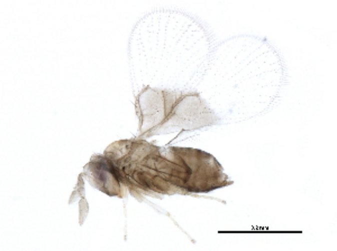

BIOUG09812-E05 [Lateral]
Trichogrammatidae
Family: Trichogrammatidae
BIN URI: BOLD:ACK2239

IMAGE NOT AVAILABLE

BIOUG22464-C11
Trichogrammatidae
Family: Trichogrammatidae

IMAGE NOT AVAILABLE

BIOUG23321-A09
Trichogrammatidae
Family: Trichogrammatidae

IMAGE NOT AVAILABLE

BIOUG22420-H04
Trichogrammatidae
Family: Trichogrammatidae

IMAGE NOT AVAILABLE

BIOUG22420-F08
Trichogrammatidae
Family: Trichogrammatidae

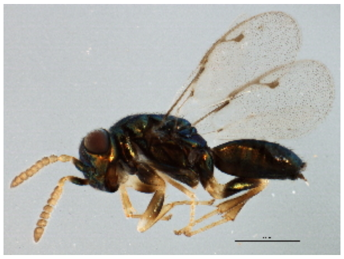

BIOUG09889-G11 [Lateral]
Hymenoptera

BIN URI: BOLD:ACL2194

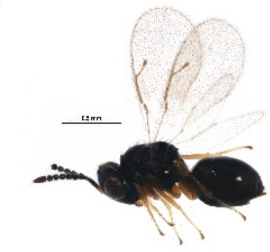

BIOUG21773-A09 [Lateral]
Pteromalidae
Family: Pteromalidae
BIN URI: BOLD:AAU9270

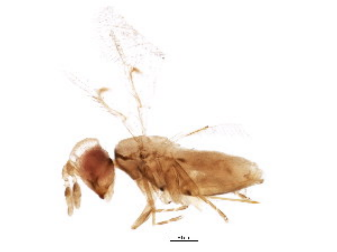

BIOUG01757-D03 [Lateral]
Trichogrammatidae
Family: Trichogrammatidae
BIN URI: BOLD:ABV2811

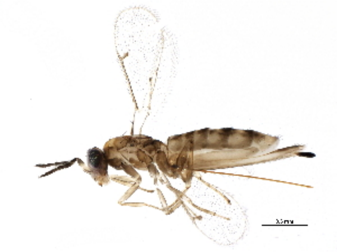

BIOUG24012-B05 [Lateral]
Eulophidae
Family: Eulophidae

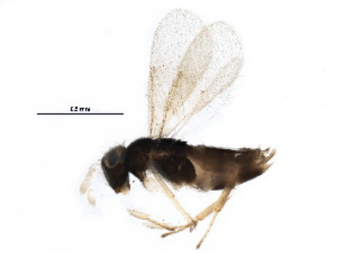

BIOUG22421-E12 [Lateral]
Aphelinus
Family: Aphelinidae
BIN URI: BOLD:ABW3282

IMAGE NOT AVAILABLE

BIOUG22931-F08
Aphelinus
Family: Aphelinidae

IMAGE NOT AVAILABLE

BIOUG22421-G03
Aphelinidae
Family: Aphelinidae

IMAGE NOT AVAILABLE

BIOUG22727-H08
Eulophidae
Family: Eulophidae

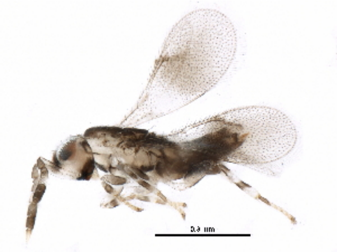

BIOUG22458-G02 [Lateral]
Aphelinidae
Family: Aphelinidae
BIN URI: BOLD:ACF7754

IMAGE NOT AVAILABLE

BIOUG23085-F04
Eulophidae
Family: Eulophidae

IMAGE NOT AVAILABLE

BIOUG22626-D11
Aphelinidae
Family: Aphelinidae

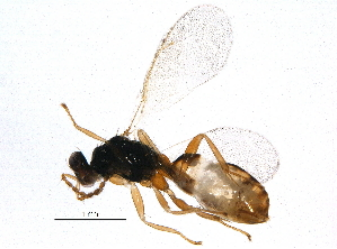

BIOUG02712-E09 [Lateral]
Eulophidae
Family: Eulophidae
BIN URI: BOLD:ACO0513

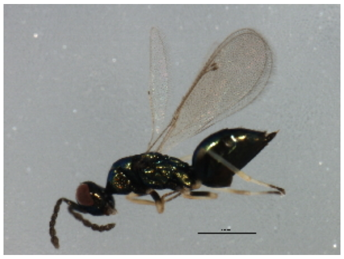

MTCHA-0047 [Lateral]
Eulophinae
Family: Eulophidae
BIN URI: BOLD:AAG3134

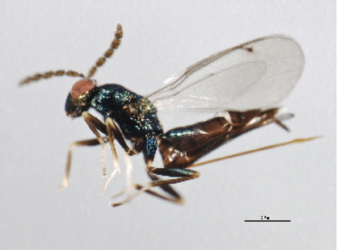

09BBEHY-0944 [Lateral]
Eulophidae
Family: Eulophidae
BIN URI: BOLD:AAG8352

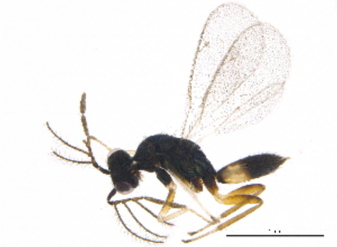

BIOUG21129-G05 [Lateral]
Hymenoptera

BIN URI: BOLD:ACU7524

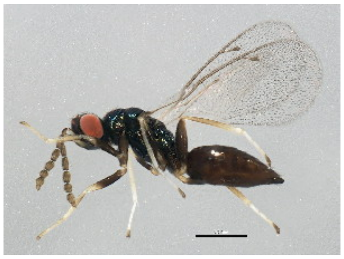

ASGLE-0941 [Lateral]
Hymenoptera

BIN URI: BOLD:AAU8693

IMAGE NOT AVAILABLE

BIOUG22420-G05
Eulophidae
Family: Eulophidae

IMAGE NOT AVAILABLE

BIOUG22931-F03
Eulophidae
Family: Eulophidae

IMAGE NOT AVAILABLE

BIOUG22294-D01
Eulophidae
Family: Eulophidae

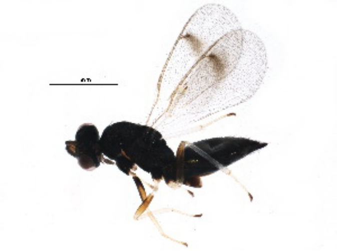

BIOUG22288-F09 [Lateral]
Eulophidae
Family: Eulophidae
BIN URI: BOLD:ACP7579

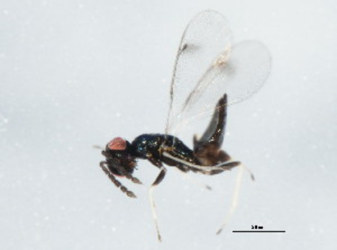

BIOUG00786-E06 [Lateral]
Eulophidae
Family: Eulophidae
BIN URI: BOLD:AAY6809

IMAGE NOT AVAILABLE

BIOUG23188-B02
Eulophidae
Family: Eulophidae

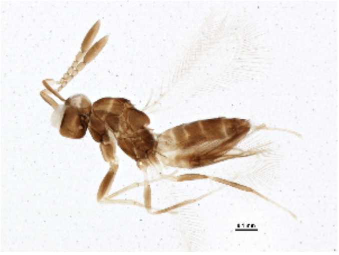

BIOUG01600-C04 [Lateral]
Mymaridae
Family: Mymaridae
BIN URI: BOLD:ABW3132

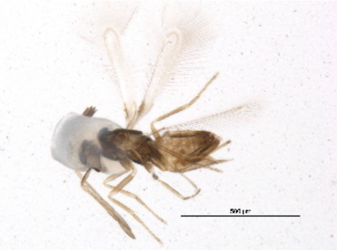

BIOUG04184-D07 [Lateral]
Mymaridae
Family: Mymaridae
BIN URI: BOLD:ACD3100

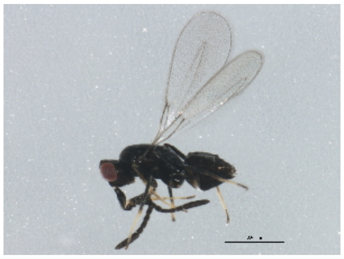

BIOUG01330-E07 [Lateral]
Mymaridae
Family: Mymaridae
BIN URI: BOLD:ABV9378

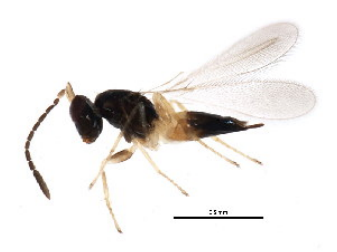

BIOUG01327-A05 [Lateral]
Gonatocerus morrilli
Family: Mymaridae
BIN URI: BOLD:AAU9165

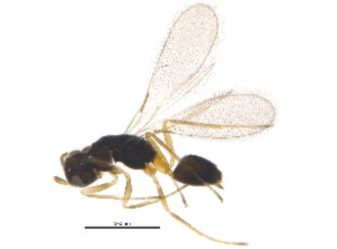

BIOUG21949-A04 [Lateral]
Hymenoptera

BIN URI: BOLD:ACU5155

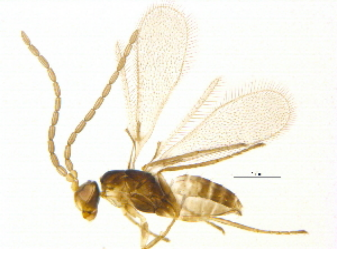

BIOUG03503-E01 [Lateral]
Gonatocerus
Family: Mymaridae
BIN URI: BOLD:AAG1488

IMAGE NOT AVAILABLE

BIOUG23321-H06
Gonatocerus
Family: Mymaridae

IMAGE NOT AVAILABLE

BIOUG22238-C03
Gonatocerus
Family: Mymaridae

IMAGE NOT AVAILABLE

BIOUG22453-H02
Anagrus
Family: Mymaridae

IMAGE NOT AVAILABLE

BIOUG23321-B09
Anagrus
Family: Mymaridae

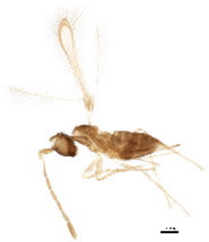

BIOUG01625-B03 [Lateral]
Anagrus
Family: Mymaridae
BIN URI: BOLD:ABW3161

IMAGE NOT AVAILABLE

BIOUG22724-D05
Anagrus
Family: Mymaridae

IMAGE NOT AVAILABLE

BIOUG23320-F07
Anagrus
Family: Mymaridae

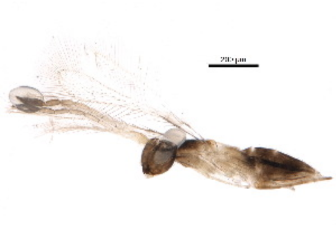

BIOUG22464-G08 [Lateral]
Anagrus
Family: Mymaridae
BIN URI: BOLD:ACL7927

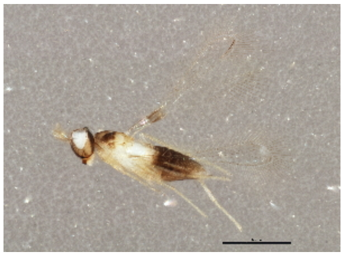

BIOUG03031-H07 [Lateral]
Anagrus
Family: Mymaridae
BIN URI: BOLD:AAN8044

IMAGE NOT AVAILABLE

BIOUG22629-H10
Anagrus
Family: Mymaridae

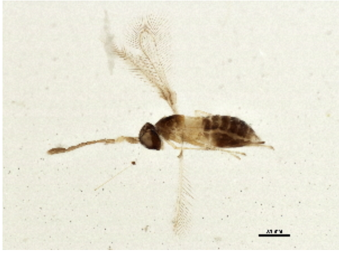

BIOUG03220-F11 [Lateral]
Anagrus
Family: Mymaridae
BIN URI: BOLD:AAZ1968

IMAGE NOT AVAILABLE

BIOUG23074-D12
Anagrus
Family: Mymaridae

IMAGE NOT AVAILABLE

BIOUG22469-D07
Anagrus
Family: Mymaridae

IMAGE NOT AVAILABLE

BIOUG22723-H06
Anagrus
Family: Mymaridae

IMAGE NOT AVAILABLE

BIOUG23085-B11
Anagrus
Family: Mymaridae

IMAGE NOT AVAILABLE

BIOUG23321-C02
Anagrus
Family: Mymaridae

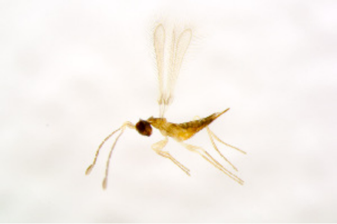

BC-ZSM-HYM-24151-E01 [adult]
Anagrus
Family: Mymaridae
BIN URI: BOLD:AAU9004

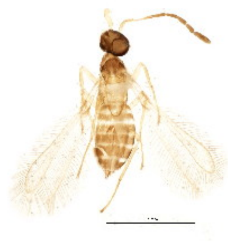

BIOUG01606-H07 [Dorsal]
Anagrus
Family: Mymaridae
BIN URI: BOLD:ABV9379

IMAGE NOT AVAILABLE

BIOUG23320-G10
Anagrus
Family: Mymaridae

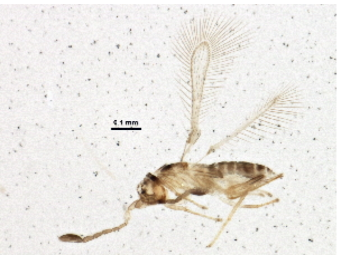

BIOUG03933-E10 [Lateral]
Anagrus
Family: Mymaridae
BIN URI: BOLD:ABV2812

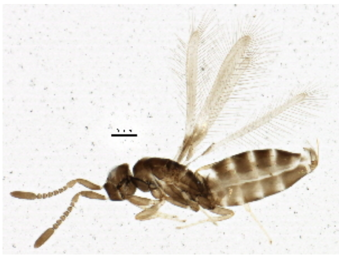

BIOUG03262-A04 [Lateral]
Mymaridae
Family: Mymaridae
BIN URI: BOLD:ACC8034

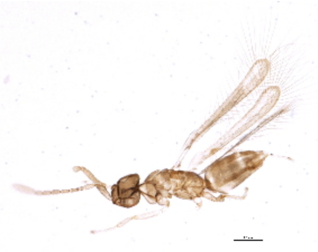

BIOUG06003-F01 [Lateral]
Mymaridae
Family: Mymaridae
BIN URI: BOLD:ACI4516

IMAGE NOT AVAILABLE

BIOUG22294-G04
Anaphes
Family: Mymaridae

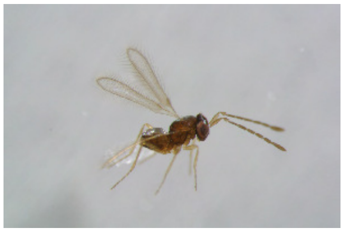

BC-ZSM-HYM-24152-E01 [adult]
Anaphes
Family: Mymaridae
BIN URI: BOLD:AAZ0173

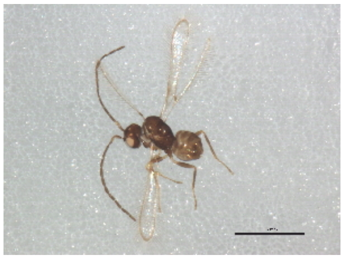

BIOUG01443-B01 [Lateral]
Anaphes anaphes listronoti
Family: Mymaridae
BIN URI: BOLD:ACE9773

IMAGE NOT AVAILABLE

BIOUG22931-F09
Anaphes
Family: Mymaridae

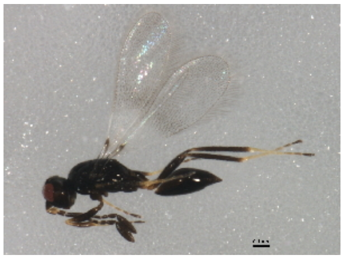

BIOUG01330-A03 [Lateral]
Mymaridae
Family: Mymaridae
BIN URI: BOLD:ABW3294

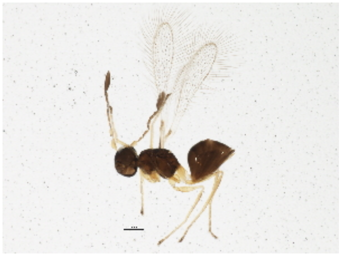

BIOUG03904-C02 [Lateral]
Mymaridae
Family: Mymaridae
BIN URI: BOLD:ACB6947

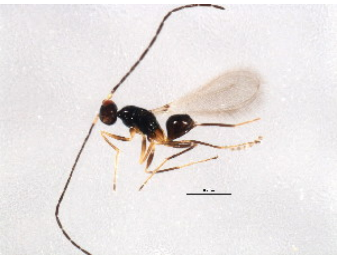

BIOUG00826-G01 [Lateral]
Mymaridae
Family: Mymaridae
BIN URI: BOLD:ABA9151

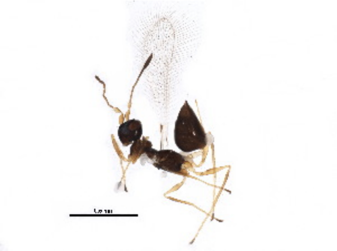

BIOUG22931-G03 [Lateral]
Polynema
Family: Mymaridae
BIN URI: BOLD:ACB1682

IMAGE NOT AVAILABLE

BIOUG22420-F06
Mymaridae
Family: Mymaridae

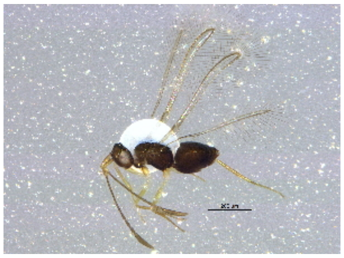

BIOUG03979-H05 [Lateral]
Mymaridae
Family: Mymaridae
BIN URI: BOLD:ABW3187

IMAGE NOT AVAILABLE

BIOUG22724-H08
Mymaridae
Family: Mymaridae

IMAGE NOT AVAILABLE

BIOUG23085-E05
Pteromalidae
Family: Pteromalidae

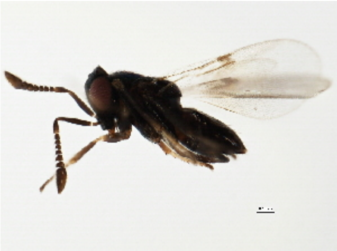

BIOUG00836-G08 [Lateral]
Copidosoma floridanum
Family: Encyrtidae
BIN URI: BOLD:AAA7203

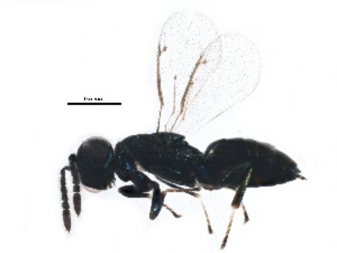

BIOUG15004-F06 [Lateral]
Hymenoptera

BIN URI: BOLD:ACV3936

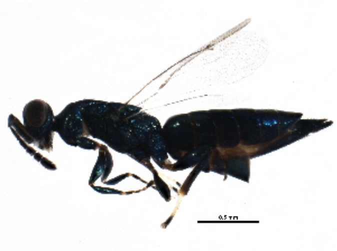

BIOUG24037-C06 [Lateral]
Eulophidae
Family: Eulophidae

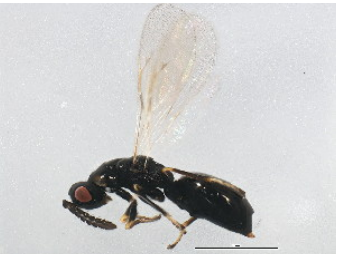

ASGLE-0546 [Lateral]
Hymenoptera

BIN URI: BOLD:AAU8477

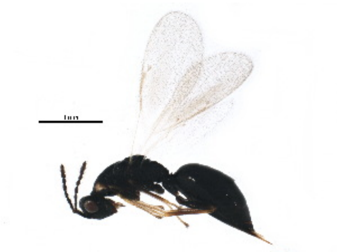

BIOUG22421-C09 [Lateral]
Eurytomidae
Family: Eurytomidae
BIN URI: BOLD:ABW3307

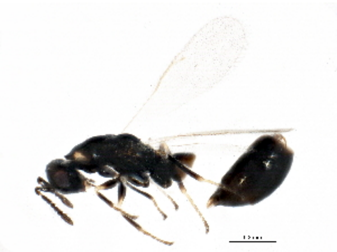

BIOUG22458-E02 [Lateral]
Eurytomidae
Family: Eurytomidae
BIN URI: BOLD:ACK5466

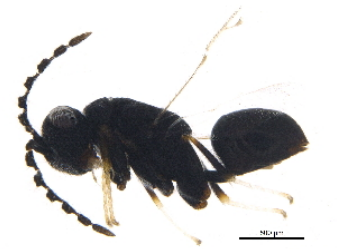

BIOUG22578-A02 [Lateral]
Eurytomidae
Family: Eurytomidae
BIN URI: BOLD:ACV3443

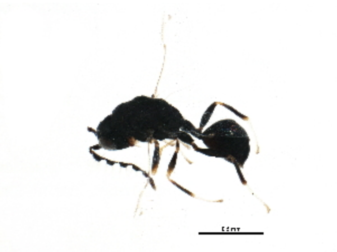

BIOUG22927-A11 [Lateral]
Eurytomidae
Family: Eurytomidae
BIN URI: BOLD:ACV5920

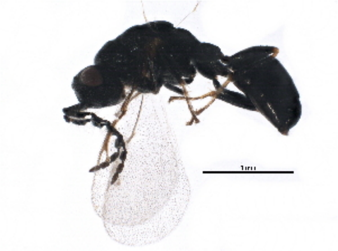

BIOUG24011-B10 [Lateral]
Eurytominae
Family: Eurytomidae

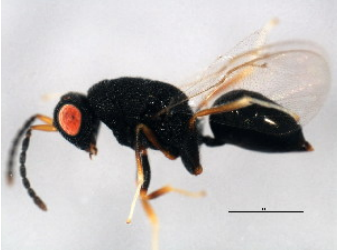

BIOUG00826-E05 [Lateral]
Eurytomidae
Family: Eurytomidae
BIN URI: BOLD:ABA9155

IMAGE NOT AVAILABLE

BIOUG22870-E12
Eurytomidae
Family: Eurytomidae

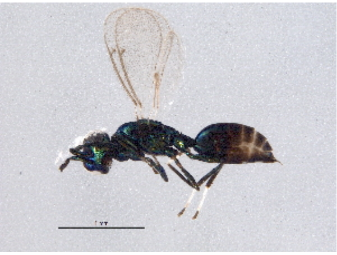

BIOUG05211-D03 [Lateral]
Eulophidae
Family: Eulophidae
BIN URI: BOLD:AAU9425

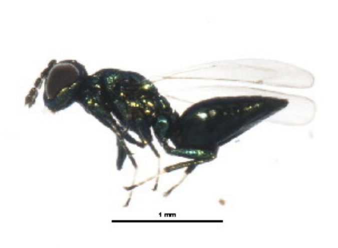

BIOUG01031-B03 [Lateral]
Entedoninae
Family: Eulophidae
BIN URI: BOLD:ABA6003

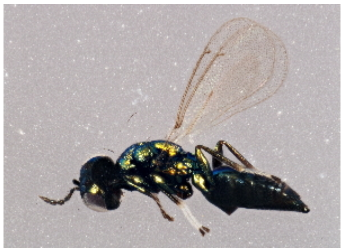

BIOUG03594-D04 [Lateral]
Entedon
Family: Eulophidae
BIN URI: BOLD:ABW3259

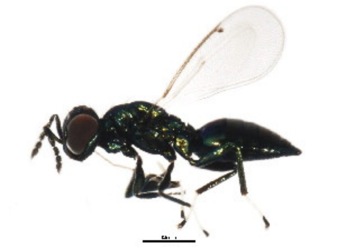

BIOUG01037-H02 [Lateral]
Entedoninae
Family: Eulophidae
BIN URI: BOLD:ABA5969

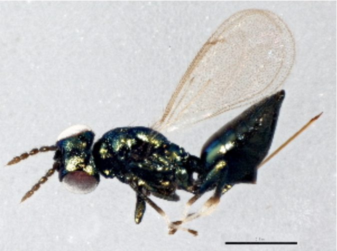

BIOUG01692-D11 [Lateral]
Eulophidae
Family: Eulophidae
BIN URI: BOLD:ACK3527

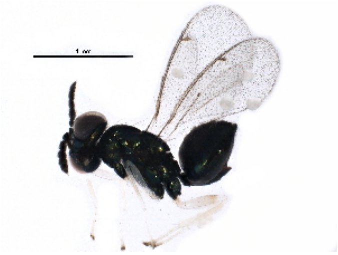

BIOUG24008-H08 [Lateral]
Eulophidae
Family: Eulophidae

IMAGE NOT AVAILABLE

BIOUG23074-A04
Eulophidae
Family: Eulophidae

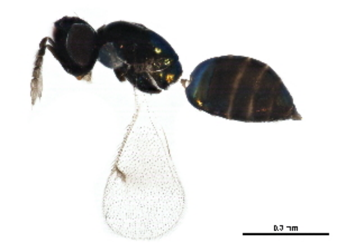

BIOUG24008-H11 [Lateral]
Eulophidae
Family: Eulophidae

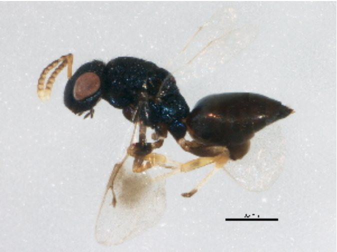

BIOUG01443-H06 [Lateral]
Pteromalidae
Family: Pteromalidae
BIN URI: BOLD:ABA6057

IMAGE NOT AVAILABLE

BIOUG21892-B07
Pteromalidae
Family: Pteromalidae

IMAGE NOT AVAILABLE

BIOUG22458-E09
Pteromalidae
Family: Pteromalidae

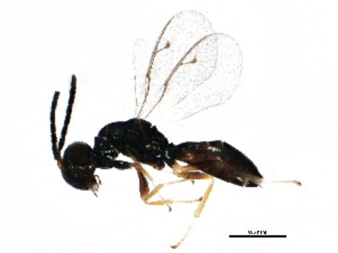

BIOUG21892-A09 [Lateral]
Pteromalidae
Family: Pteromalidae

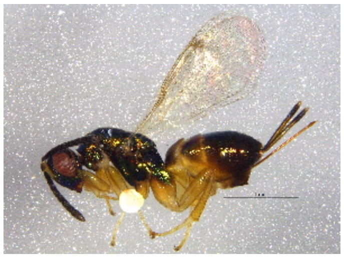

BIOUG03979-E04 [Lateral]
Torymidae
Family: Torymidae
BIN URI: BOLD:ACC1454

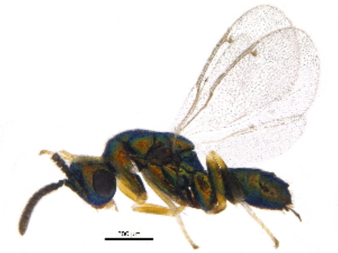

BIOUG22238-C09 [Lateral]
Torymus
Family: Torymidae
BIN URI: BOLD:ACV2473

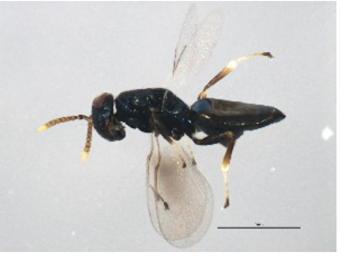

ASGLE-0552 [Lateral]
Hymenoptera

BIN URI: BOLD:AAU8478

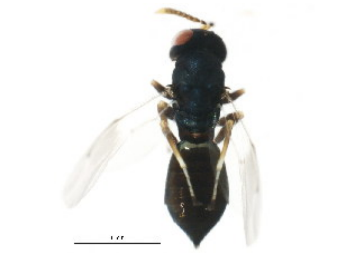

BIOUG01284-D10 [Dorsal]
Encyrtidae
Family: Encyrtidae
BIN URI: BOLD:ABA9126

IMAGE NOT AVAILABLE

BIOUG23085-G11
Encyrtidae
Family: Encyrtidae

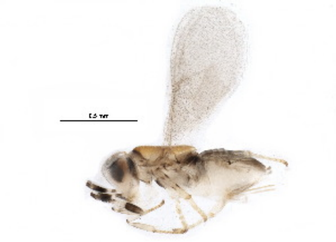

BIOUG22626-C01 [Lateral]
Encyrtidae
Family: Encyrtidae
BIN URI: BOLD:ACV5102

IMAGE NOT AVAILABLE

BIOUG23085-H08
Encyrtidae
Family: Encyrtidae

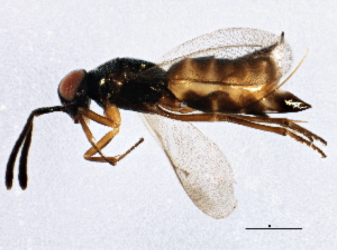

BIOUG01309-B11 [Lateral]
Mymaridae
Family: Mymaridae
BIN URI: BOLD:ABX2601

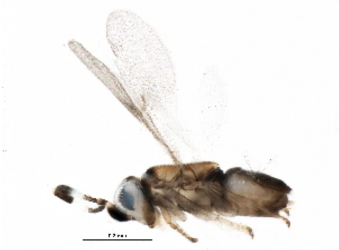

BIOUG22458-G03 [Lateral]
Encyrtidae
Family: Encyrtidae
BIN URI: BOLD:ACV3546

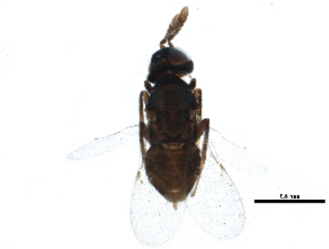

BIOUG08612-B09 [Dorsal]
Hymenoptera

BIN URI: BOLD:ACK1743

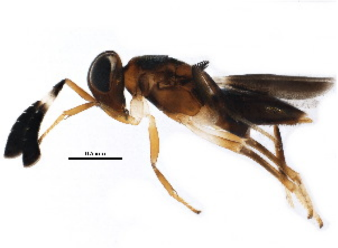

BIOUG22723-H11 [Lateral]
Trichogrammatidae
Family: Trichogrammatidae
BIN URI: BOLD:ACV4980

IMAGE NOT AVAILABLE

BIOUG22728-F03
Eulophidae
Family: Eulophidae

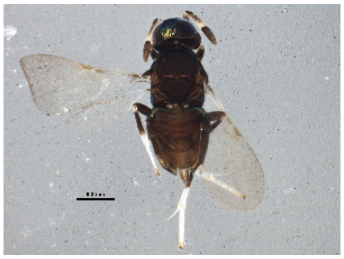

BIOUG09249-B05 [Dorsal]
Hymenoptera

BIN URI: BOLD:ACL0141

IMAGE NOT AVAILABLE

BIOUG22931-H07
Encyrtidae
Family: Encyrtidae

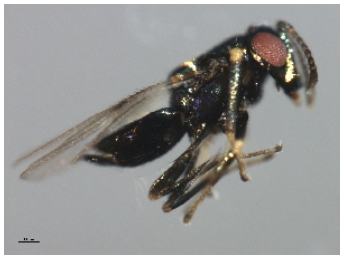

08BBHYM-1058 [Lateral]
Encyrtidae
Family: Encyrtidae
BIN URI: BOLD:AAG7879

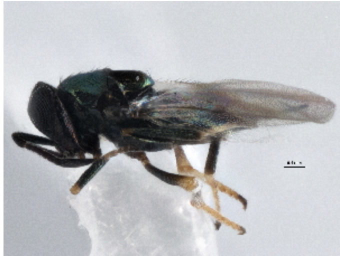

BIOUG08726-D07 [Lateral]
Hymenoptera

BIN URI: BOLD:ACI4933

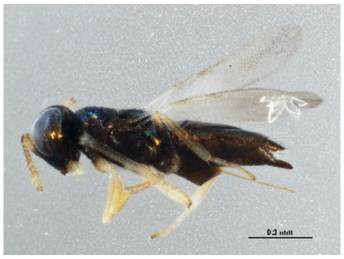

BIOUG09005-G10 [Lateral]
Encyrtidae
Family: Encyrtidae
BIN URI: BOLD:ACK5989

IMAGE NOT AVAILABLE

BIOUG23085-A10
Encyrtidae
Family: Encyrtidae

IMAGE NOT AVAILABLE

BIOUG22458-E12
Trichogrammatidae
Family: Trichogrammatidae

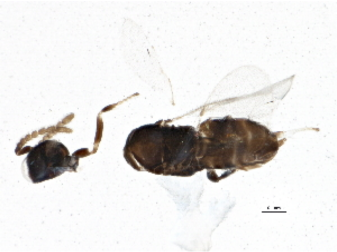

BIOUG04245-D09 [Lateral]
Hymenoptera

BIN URI: BOLD:ACD0871

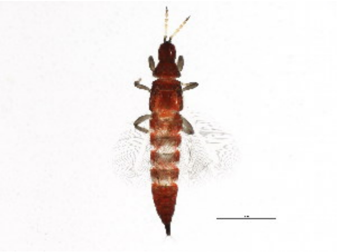

BIOUG04093-A03 [Dorsal]
Haplothrips
Family: Phlaeothripidae
BIN URI: BOLD:AAG2812

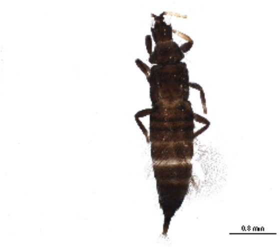

BIOUG09310-A11 [Dorsal]
Haplothrips verbasci
Family: Phlaeothripidae
BIN URI: BOLD:AAI6861

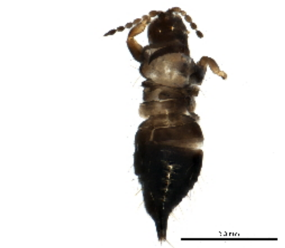

BIOUG24001-C12 [Dorsal]
Thysanoptera

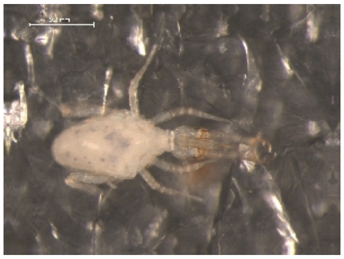

BIOUG05520-E03 [Lateral]
Cunaxidae
Family: Cunaxidae
BIN URI: BOLD:ACF7081

IMAGE NOT AVAILABLE

BIOUG22841-F07
Cunaxidae
Family: Cunaxidae

IMAGE NOT AVAILABLE

BIOUG22846-F07
Stigmaeidae
Family: Stigmaeidae
BIN URI: BOLD:ACV9372

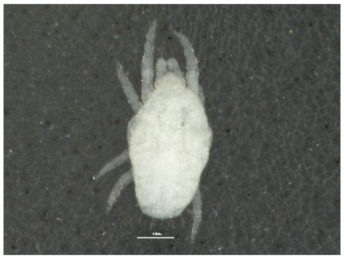

BIOUG03211-H04 [Dorsal]
Stigmaeidae
Family: Stigmaeidae
BIN URI: BOLD:ACC8365

IMAGE NOT AVAILABLE

BIOUG08405-G08
Stigmaeidae
Family: Stigmaeidae
BIN URI: BOLD:ACK3900

IMAGE NOT AVAILABLE

BIOUG24030-F11
Trombidiformes

IMAGE NOT AVAILABLE

BIOUG24030-F09
Tarsonemidae
Family: Tarsonemidae

IMAGE NOT AVAILABLE

BIOUG24030-G09
Tarsonemidae
Family: Tarsonemidae

IMAGE NOT AVAILABLE

BIOUG24005-E07
Tarsonemidae
Family: Tarsonemidae

IMAGE NOT AVAILABLE

BIOUG24030-B05
Tarsonemidae
Family: Tarsonemidae

IMAGE NOT AVAILABLE

BIOUG21886-F12
Trombidiformes

BIN URI: BOLD:ACV5754

IMAGE NOT AVAILABLE

BIOUG24030-G06
Tarsonemidae
Family: Tarsonemidae

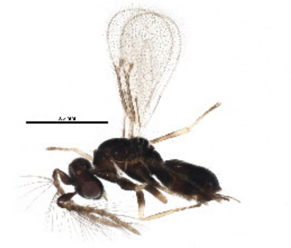

BIOUG20971-B07 [Lateral]
Hymenoptera

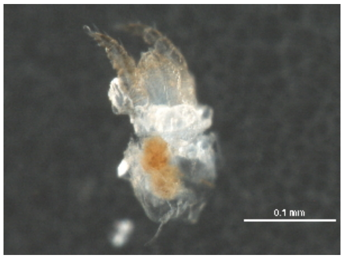

BIOUG19921-E09 [Dorsal]
Arachnida

BIN URI: BOLD:ACT7872

IMAGE NOT AVAILABLE

BIOUG24030-F07
Trombidiformes

IMAGE NOT AVAILABLE

BIOUG21884-H06
Trombidiformes

BIN URI: BOLD:ACV5529

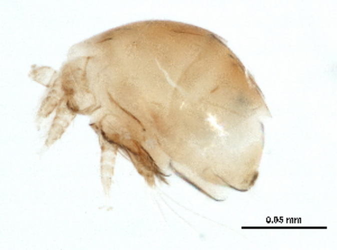

BIOUG21882-E03 [Lateral]
Scutacaridae
Family: Scutacaridae
BIN URI: BOLD:ACR0181

IMAGE NOT AVAILABLE

BIOUG24030-G01
Trombidiformes

IMAGE NOT AVAILABLE

BIOUG21899-A04
Scutacaridae
Family: Scutacaridae
BIN URI: BOLD:ACV5391

IMAGE NOT AVAILABLE

BIOUG21899-A02
Trombidiformes

BIN URI: BOLD:ACV5489

IMAGE NOT AVAILABLE

BIOUG21897-F12
Scutacaridae
Family: Scutacaridae
BIN URI: BOLD:ACV5617

IMAGE NOT AVAILABLE

BIOUG24030-B09
Trombidiformes

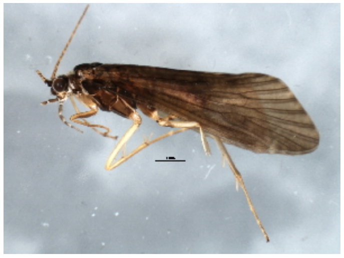

07ELEPT-112 [Lateral]
Cheumatopsyche analis
Family: Hydropsychidae
BIN URI: BOLD:AAA5695

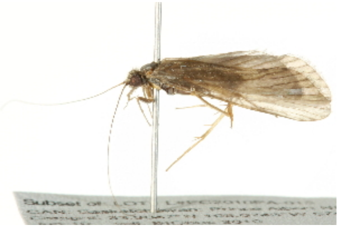

BIOUG00839-F05 [Lateral]
Cheumatopsyche campyla
Family: Hydropsychidae
BIN URI: BOLD:AAA3892

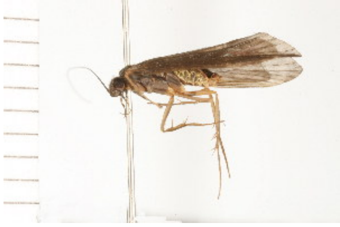

08NBEPT-0639 [Lateral]
Cheumatopsyche ela
Family: Hydropsychidae
BIN URI: BOLD:ACE5263

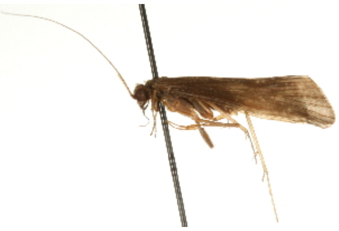

09CBCAD-329 [Lateral]
Hydropsyche sparna
Family: Hydropsychidae
BIN URI: BOLD:AAA2528

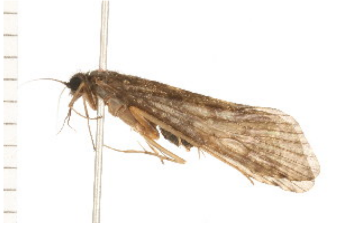

08NBEPT-0497 [Lateral]
Hydropsyche bronta
Family: Hydropsychidae
BIN URI: BOLD:ACW3105

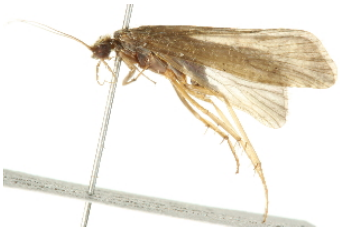

BIOUG00839-F06 [Lateral]
Ceratopsyche morosa
Family: Hydropsychidae
BIN URI: BOLD:AAA3679

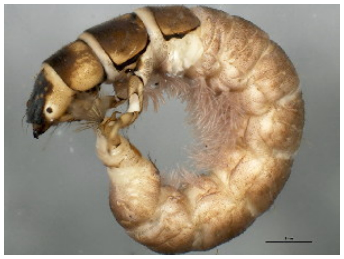

FormTOexpt146 [Lateral]
Hydropsyche morosa
Family: Hydropsychidae
BIN URI: BOLD:AAA3680

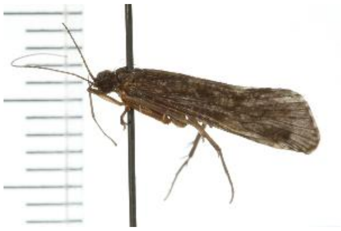

07ONCAD-0126 [Lateral]
Hydropsyche betteni
Family: Hydropsychidae
BIN URI: BOLD:AAA1669

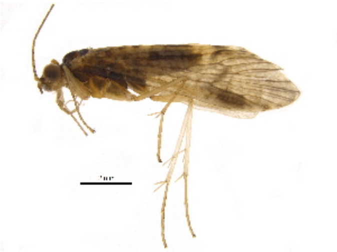

BIOUG22866-H08 [Lateral]
Hydropsyche phalerata
Family: Hydropsychidae
BIN URI: BOLD:AAC3243

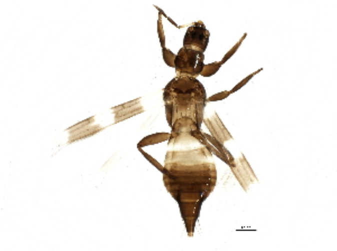

BIOUG02751-F04 [Dorsal]
Aeolothrips
Family: Aeolothripidae
BIN URI: BOLD:AAU0577

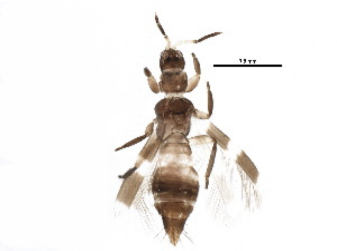

BIOUG19863-E09 [Dorsal]
Aeolothrips ericae
Family: Aeolothripidae
BIN URI: BOLD:ABA2981

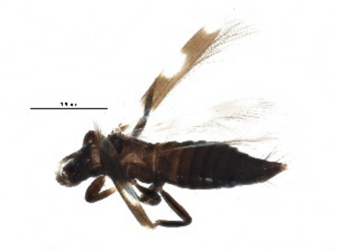

BIOUG11035-A03 [Lateral]
Aeolothripidae
Family: Aeolothripidae
BIN URI: BOLD:ACM1798

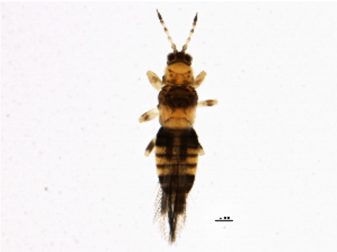

BIOUG03932-E02 [Dorsal]
Thripidae
Family: Thripidae
BIN URI: BOLD:ACN8291

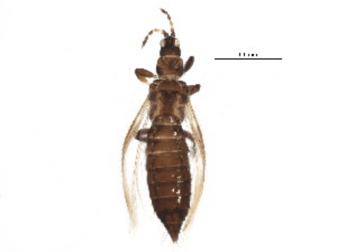

BIOUG08879-D11 [Dorsal]
Taeniothrips inconsequens
Family: Thripidae
BIN URI: BOLD:ACC0651

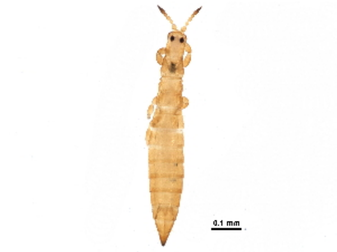

BIOUG21887-F09 [Dorsal]
Aptinothrips
Family: Thripidae
BIN URI: BOLD:AAU0618

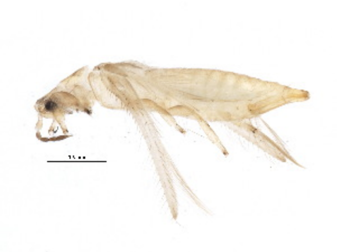

BIOUG11434-F09 [Lateral]
Thripinae
Family: Thripidae
BIN URI: BOLD:AAM7854

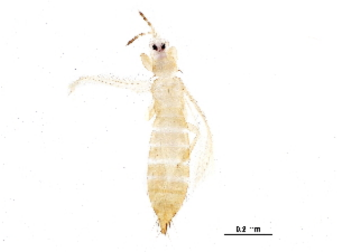

BIOUG03880-H07 [Dorsal]
Thripinae
Family: Thripidae
BIN URI: BOLD:ACD5332

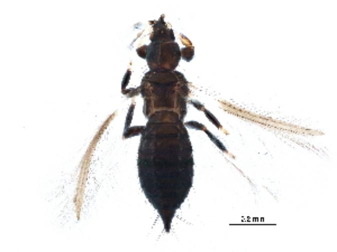

BIOUG10662-G12 [Dorsal]
Odontothrips biuncus
Family: Thripidae
BIN URI: BOLD:AAN5715

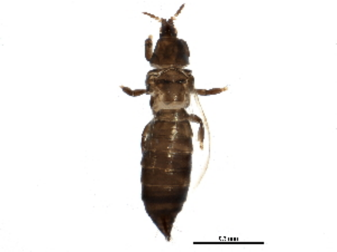

BIOUG24029-D03 [Dorsal]
Chirothrips manicatus
Family: Thripidae

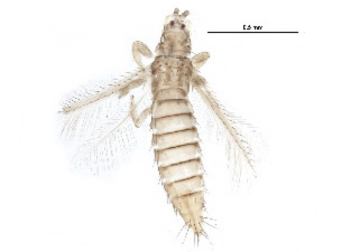

BIOUG22288-D09 [Dorsal]
Thripidae
Family: Thripidae
BIN URI: BOLD:ACV3023

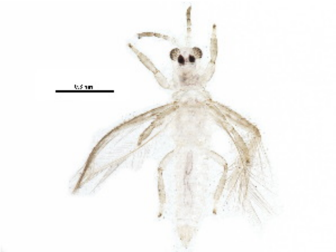

BIOUG22575-C06 [Dorsal]
Scirtothrips
Family: Thripidae
BIN URI: BOLD:ACV3625

IMAGE NOT AVAILABLE

BIOUG22354-G03
Scirtothrips
Family: Thripidae
BIN URI: BOLD:ACV4056

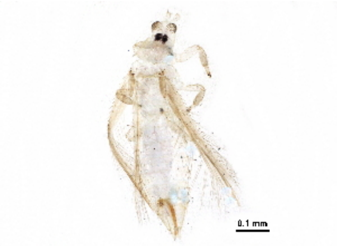

BIOUG22575-C09 [Dorsal]
Thripidae
Family: Thripidae
BIN URI: BOLD:ACV4199

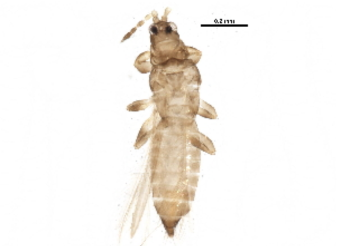

BIOUG08754-F12 [Dorsal]
Thripidae
Family: Thripidae
BIN URI: BOLD:ABW5634

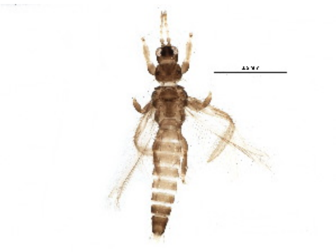

BIOUG04339-F02 [Dorsal]
Thrips physapus
Family: Thripidae
BIN URI: BOLD:AAN9105

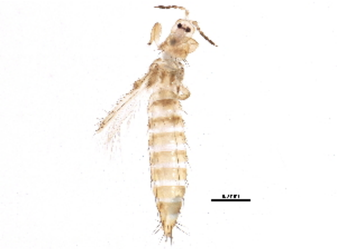

BIOUG13478-F03 [Dorsal]
Anaphothrips obscurus
Family: Thripidae
BIN URI: BOLD:AAD4600

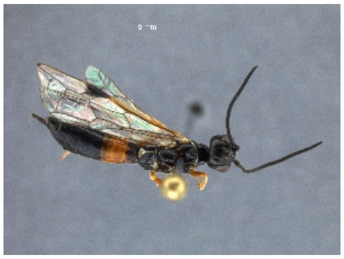

WSSF98 [Dorsal]
Janus integer
Family: Cephidae
BIN URI: BOLD:AAD8205

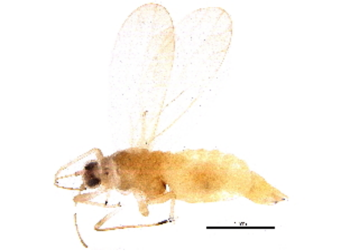

BIOUG19924-E08 [Lateral]
Psyllidae
Family: Psyllidae
BIN URI: BOLD:ACT4553

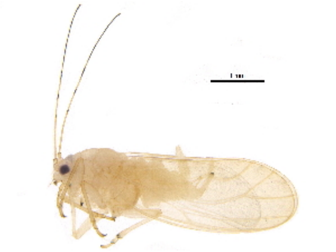

BIOUG22874-H11 [Lateral]
Psyllidae
Family: Psyllidae
BIN URI: BOLD:ACV4988

IMAGE NOT AVAILABLE

BIOUG21881-G04
Psyllidae
Family: Psyllidae

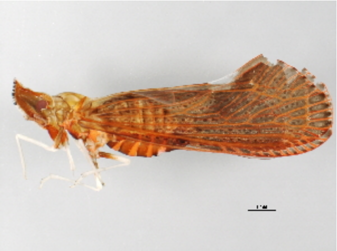

09BBEHE-057 [Lateral]
Otiocerus degeerii
Family: Derbidae
BIN URI: BOLD:AAG8977

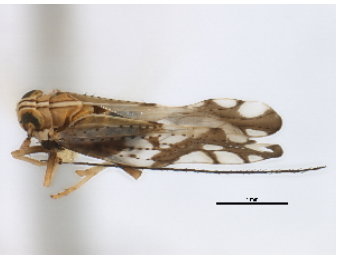

CNC#HEM405902 [Lateral]
Liburniella ornata
Family: Delphacidae
BIN URI: BOLD:AAN8272

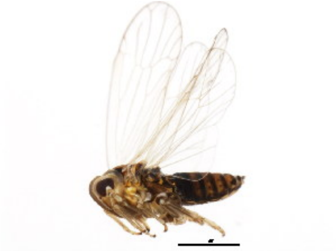

BIOUG01012-C07 [Lateral]
Muirodelphax atralabis
Family: Delphacidae
BIN URI: BOLD:AAC3517

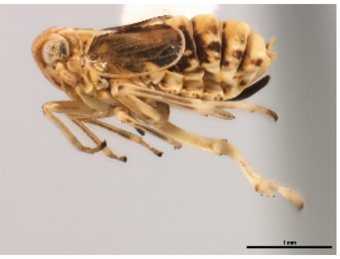

CNC#HEM405939 [Lateral]
Delphacodes kilmani
Family: Delphacidae
BIN URI: BOLD:AAV0160

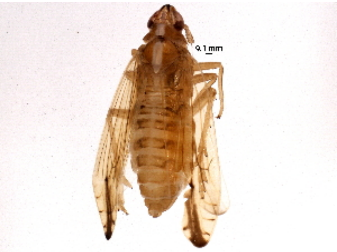

BIOUG07904-E03 [Dorsal]
Delphacidae
Family: Delphacidae
BIN URI: BOLD:ACJ0257

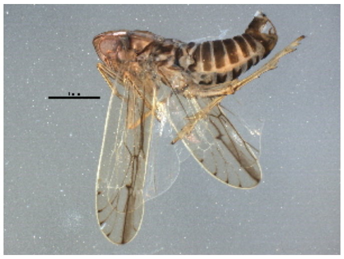

BIOUG13544-H05 [Lateral]
Delphacidae
Family: Delphacidae
BIN URI: BOLD:ACO0772

IMAGE NOT AVAILABLE

BIOUG08101-C12
Eupodidae
Family: Eupodidae
BIN URI: BOLD:ACI9912

IMAGE NOT AVAILABLE

BIOUG20568-H04
Eupodidae
Family: Eupodidae
BIN URI: BOLD:ACV5502

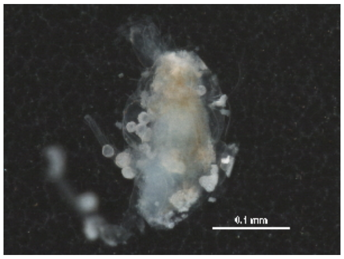

BIOUG22864-B01 [Dorsal]
Eupodes
Family: Eupodidae
BIN URI: BOLD:AAO8147

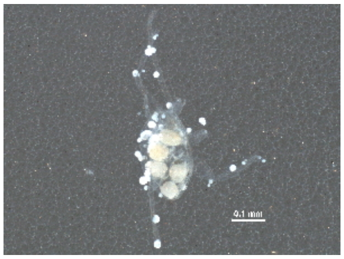

BIOUG22864-D03 [Dorsal]
Eupodidae
Family: Eupodidae
BIN URI: BOLD:ACN0389

IMAGE NOT AVAILABLE

BIOUG21897-A04
Eupodidae
Family: Eupodidae
BIN URI: BOLD:ACV5409

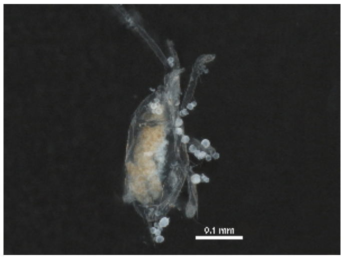

BIOUG22864-A07 [Dorsal]
Eupodidae
Family: Eupodidae
BIN URI: BOLD:ACV5912

IMAGE NOT AVAILABLE

BIOUG24030-D08
Eupodidae
Family: Eupodidae

IMAGE NOT AVAILABLE

BIOUG24030-C02
Eupodidae
Family: Eupodidae

IMAGE NOT AVAILABLE

BIOUG21884-H01
Eupodidae
Family: Eupodidae
BIN URI: BOLD:ACV5335

IMAGE NOT AVAILABLE

BIOUG24030-D01
Pachygnathidae
Family: Pachygnathidae

IMAGE NOT AVAILABLE

BIOUG21886-F01
Trombidiformes

BIN URI: BOLD:ACV5687

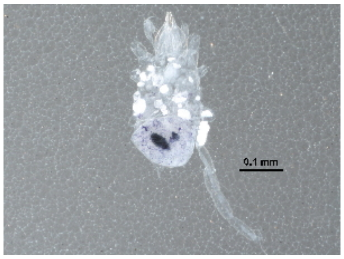

BIOUG22631-C04 [Dorsal]
Rhagidiidae
Family: Rhagidiidae
BIN URI: BOLD:ACV5446

IMAGE NOT AVAILABLE

BIOUG21899-D03
Rhagidiidae
Family: Rhagidiidae
BIN URI: BOLD:ACV4973

IMAGE NOT AVAILABLE

BIOUG21882-C12
Rhagidiidae
Family: Rhagidiidae
BIN URI: BOLD:ACV7504

IMAGE NOT AVAILABLE

BIOUG24000-H08
Rhagidiidae
Family: Rhagidiidae

IMAGE NOT AVAILABLE

BIOUG21886-C05
Tydeidae
Family: Tydeidae
BIN URI: BOLD:ACV5866

IMAGE NOT AVAILABLE

BIOUG21886-F06
Tydeidae
Family: Tydeidae
BIN URI: BOLD:ACV5008

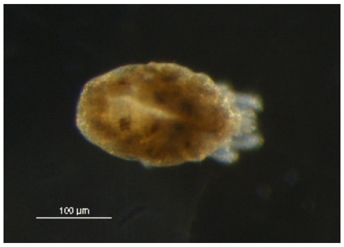

BIOUG01179-41 [Dorsal]
Tydeidae
Family: Tydeidae
BIN URI: BOLD:ABV1817

IMAGE NOT AVAILABLE

BIOUG21897-E01
Tydeidae
Family: Tydeidae
BIN URI: BOLD:ACV5486

IMAGE NOT AVAILABLE

BIOUG21882-E07
Tydeidae
Family: Tydeidae
BIN URI: BOLD:ACV6451

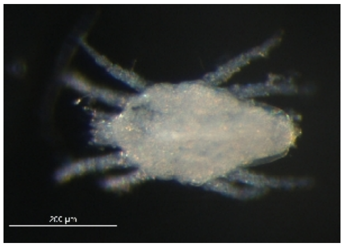

BIOUG05554-B12 [Lateral]
Tydeus
Family: Tydeidae
BIN URI: BOLD:AAH3904

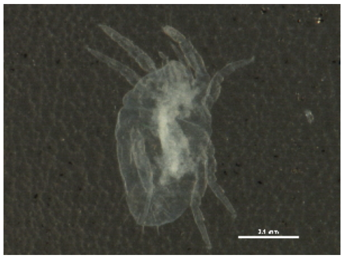

BIOUG09351-E03 [Dorsal]
Arachnida

BIN URI: BOLD:ACI9974

IMAGE NOT AVAILABLE

BIOUG21897-B12
Tydeidae
Family: Tydeidae
BIN URI: BOLD:ACV4849

IMAGE NOT AVAILABLE

BIOUG21897-D05
Tydeidae
Family: Tydeidae
BIN URI: BOLD:ACV4895

IMAGE NOT AVAILABLE

BIOUG21899-C08
Tydeidae
Family: Tydeidae
BIN URI: BOLD:ACV5527

IMAGE NOT AVAILABLE

BIOUG24000-G02
Eupodidae
Family: Eupodidae

IMAGE NOT AVAILABLE

BIOUG20921-F12
Arachnida

BIN URI: BOLD:ACV5532

IMAGE NOT AVAILABLE

BIOUG21897-C09
Eupodidae
Family: Eupodidae
BIN URI: BOLD:ACV4932

IMAGE NOT AVAILABLE

BIOUG08057-F07
Eupodidae
Family: Eupodidae
BIN URI: BOLD:ACB5424

IMAGE NOT AVAILABLE

BIOUG21882-C04
Eupodidae
Family: Eupodidae
BIN URI: BOLD:ACV8844

IMAGE NOT AVAILABLE

BIOUG24030-D02
Eupodidae
Family: Eupodidae

IMAGE NOT AVAILABLE

BIOUG24000-E07
Eupodidae
Family: Eupodidae

IMAGE NOT AVAILABLE

BIOUG24030-E07
Eupodidae
Family: Eupodidae

IMAGE NOT AVAILABLE

BIOUG08057-D02
Eupodidae
Family: Eupodidae
BIN URI: BOLD:ACI9910

IMAGE NOT AVAILABLE

BIOUG16633-G07
Eupodidae
Family: Eupodidae
BIN URI: BOLD:ACQ9955

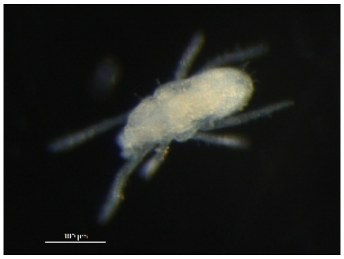

BIOUG01189-84 [Dorsal]
Trombidiformes

BIN URI: BOLD:ABV1844

IMAGE NOT AVAILABLE

BIOUG21899-D11
Trombidiformes

BIN URI: BOLD:ACV5697

IMAGE NOT AVAILABLE

BIOUG21774-F04
Trombidiformes

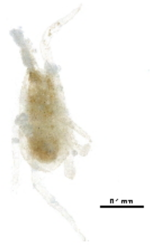

BIOUG14833-H11 [Lateral]
Arachnida

BIN URI: BOLD:ACI2899

IMAGE NOT AVAILABLE

BIOUG21773-G07
Bdellidae
Family: Bdellidae
BIN URI: BOLD:ACV2258

IMAGE NOT AVAILABLE

BIOUG22837-C05
Trombidiformes

BIN URI: BOLD:ACV9532

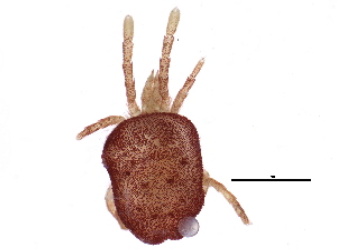

BIOUG14250-F10 [Dorsal]
Arachnida

BIN URI: BOLD:ACL1896

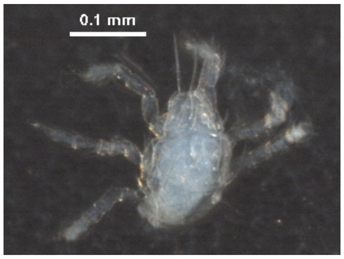

BIOUG24005-A06 [Dorsal]
Trombidiformes

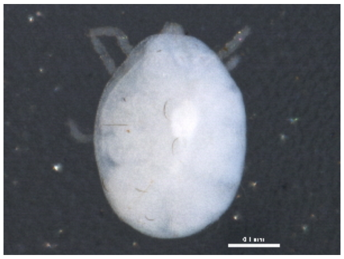

BIOUG03765-B07 [Dorsal]
Microtrombidiidae
Family: Microtrombidiidae
BIN URI: BOLD:ABW5666

IMAGE NOT AVAILABLE

BIOUG23322-G02
Microtrombidiidae
Family: Microtrombidiidae
BIN URI: BOLD:ACW0840

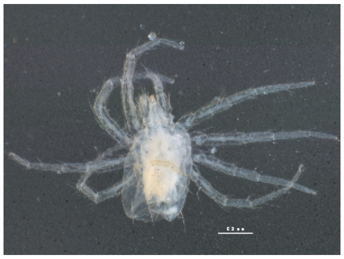

BIOUG06849-E09 [Dorsal]
Anystidae
Family: Anystidae
BIN URI: BOLD:ACJ1181

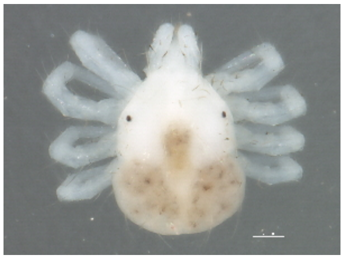

BIOUG01238-B08 [Dorsal]
Anystidae
Family: Anystidae
BIN URI: BOLD:ABY2067

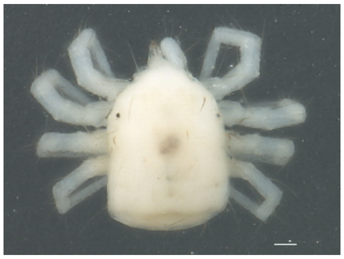

BIOUG01238-H09 [Dorsal]
Anystidae
Family: Anystidae
BIN URI: BOLD:AAM7961

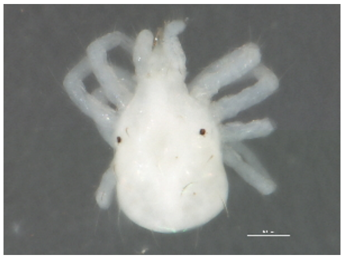

BIOUG01238-E09 [Dorsal]
Anystidae
Family: Anystidae
BIN URI: BOLD:ABW2639

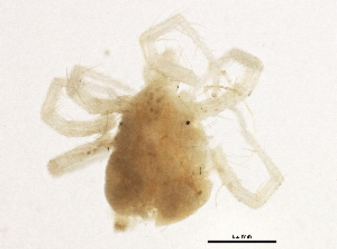

BIOUG04156-E10 [Dorsal]
Anystidae
Family: Anystidae
BIN URI: BOLD:AAF9236

IMAGE NOT AVAILABLE

BIOUG22931-D07
Anystidae
Family: Anystidae

IMAGE NOT AVAILABLE

BIOUG22632-F10
Sarcoptiformes

BIN URI: BOLD:ACV5927

IMAGE NOT AVAILABLE

BIOUG22630-H10
Sarcoptiformes

BIN URI: BOLD:ACV5101

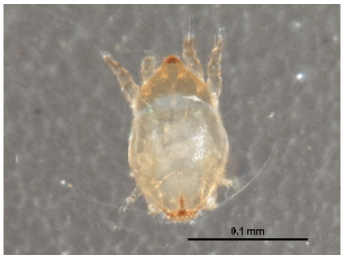

BIOUG23322-F12 [Dorsal]
Sarcoptiformes

BIN URI: BOLD:ACW1176

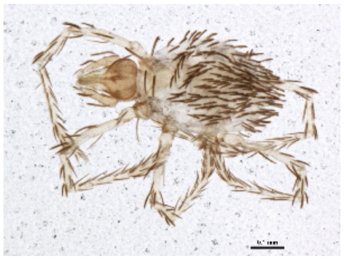

BIOUG01876-B09 [Dorsal]
Leptus
Family: Erythraeidae
BIN URI: BOLD:ABW2638

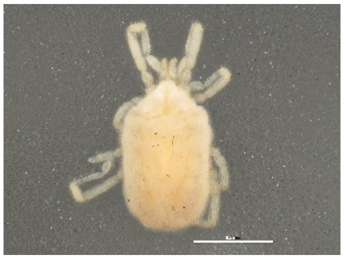

BIOUG02050-H09 [Dorsal]
Erythraeidae
Family: Erythraeidae
BIN URI: BOLD:AAN6614

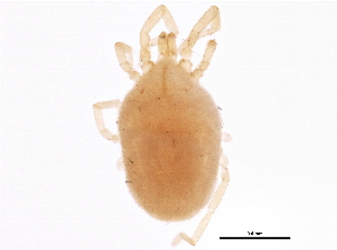

BIOUG04693-B01 [Dorsal]
Erythraeidae
Family: Erythraeidae
BIN URI: BOLD:ABW2691

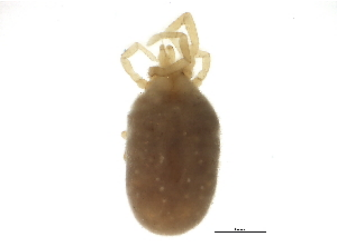

BIOUG01238-F05 [Dorsal]
Erythraeidae
Family: Erythraeidae
BIN URI: BOLD:AAH6489

IMAGE NOT AVAILABLE

BIOUG22279-D08
Hygrobatidae
Family: Hygrobatidae
BIN URI: BOLD:ACV2582

IMAGE NOT AVAILABLE

BIOUG22321-E04
Hygrobatidae
Family: Hygrobatidae

IMAGE NOT AVAILABLE

BIOUG22323-A01
Hygrobatidae
Family: Hygrobatidae

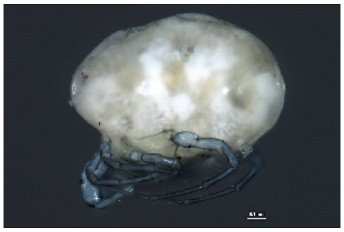

CNC-IMS090088.101 [Lateral]
Limnesia sp0988B
Family: Limnesiidae
BIN URI: BOLD:AAE6458

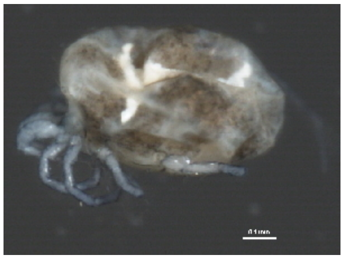

CNC-IMS090050.004 [Lateral]
Limnesia sp0950A
Family: Limnesiidae
BIN URI: BOLD:AAE6450

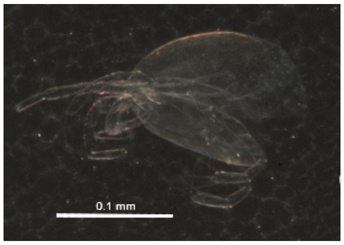

BIOUG21001-F12 [Lateral]
Arachnida

BIN URI: BOLD:ACU5009

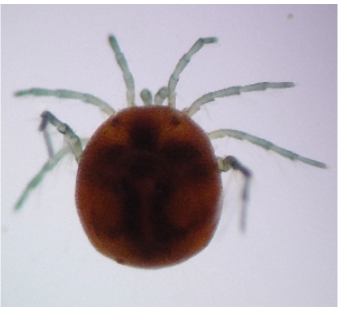

Barb120531Ar1095 [Dorsal]
Arrenurus
Family: Arrenuridae
BIN URI: BOLD:ACG4894

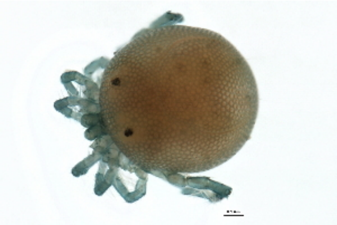

CNC-IMS090088.062 [Dorsal]
Arrenurus sp0988G
Family: Arrenuridae
BIN URI: BOLD:AAE6722

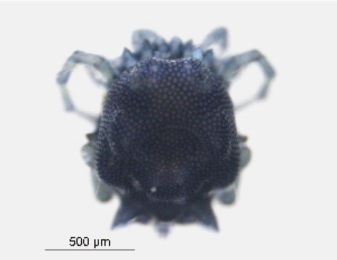

BIOUG15122-B04 [Dorsal]
Arrenurus
Family: Arrenuridae
BIN URI: BOLD:ACI3345

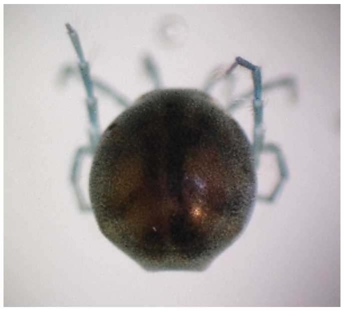

LOKB120615Mi1166 [Dorsal]
Arrenurus
Family: Arrenuridae
BIN URI: BOLD:ACL2659

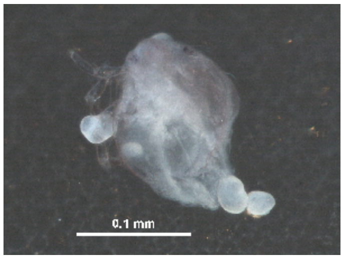

BIOUG22864-A02 [Dorsal]
Arrenuridae
Family: Arrenuridae
BIN URI: BOLD:ACV5390

IMAGE NOT AVAILABLE

BIOUG23322-F07
Arrenurus
Family: Arrenuridae
BIN URI: BOLD:ACW0801

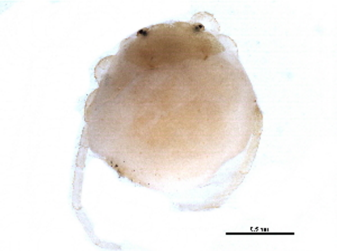

BIOUG24005-B10 [Dorsal]
Eylaidae
Family: Eylaidae

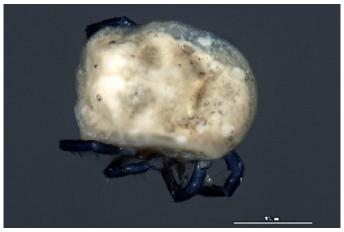

CNC-IMS090088.116 [Dorsal]
Neumania sp0988B
Family: Unionicolidae
BIN URI: BOLD:AAK8171

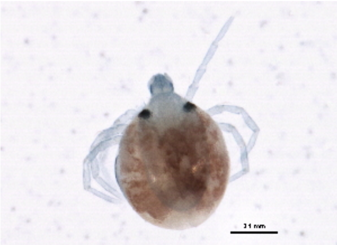

BIOUG03993-H08 [Dorsal]
Unionicolidae
Family: Unionicolidae
BIN URI: BOLD:ACC7553

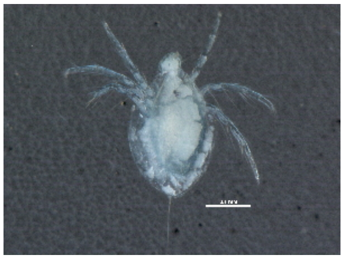

BIOUG07005-D05 [Dorsal]
Trombidiformes

BIN URI: BOLD:ACI7166

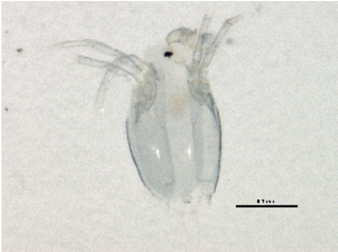

BIOUG07022-E10 [Dorsal]
Unionicola
Family: Unionicolidae
BIN URI: BOLD:ACJ8639

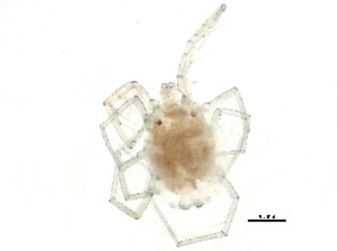

BIOUG08018-B12 [Dorsal]
Unionicolidae
Family: Unionicolidae
BIN URI: BOLD:ACH3803

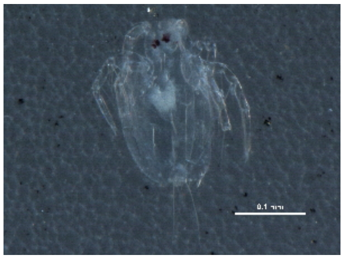

BIOUG07022-E02 [Dorsal]
Unionicolidae
Family: Unionicolidae
BIN URI: BOLD:ACI7165

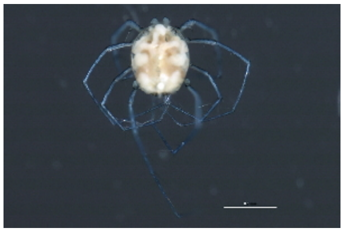

CNC-IMS090088.125 [Dorsal]
Unionicola sp0988A
Family: Unionicolidae
BIN URI: BOLD:AAE9937

IMAGE NOT AVAILABLE

BIOUG22575-G10
Unionicolidae
Family: Unionicolidae
BIN URI: BOLD:ACH4393

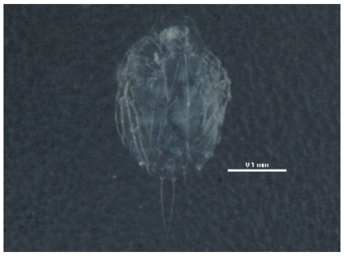

BIOUG11728-C08 [Dorsal]
Unionicola
Family: Unionicolidae
BIN URI: BOLD:ACG6284

IMAGE NOT AVAILABLE

BIOUG22575-E07
Unionicola
Family: Unionicolidae
BIN URI: BOLD:ACV4538

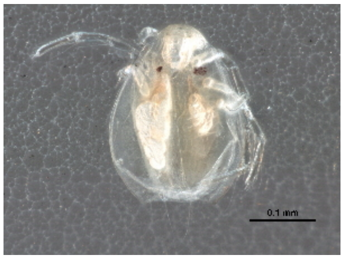

BIOUG21888-D12 [Dorsal]
Unionicolidae
Family: Unionicolidae
BIN URI: BOLD:ACV5809

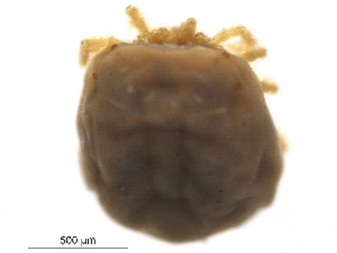

BIOUG15122-E09 [Dorsal]
Hydrodroma
Family: Hydrodromidae
BIN URI: BOLD:AAN6616

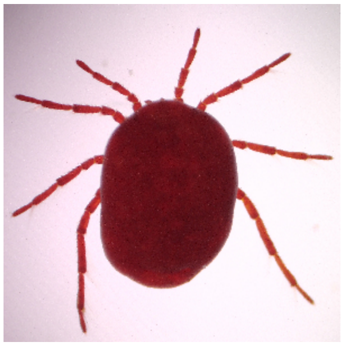

Barb120531Hy1006 [Dorsal]
Hydryphantes
Family: Hydryphantidae
BIN URI: BOLD:ACH9257

IMAGE NOT AVAILABLE

BIOUG23322-D01
Pionidae
Family: Pionidae
BIN URI: BOLD:ACW0855

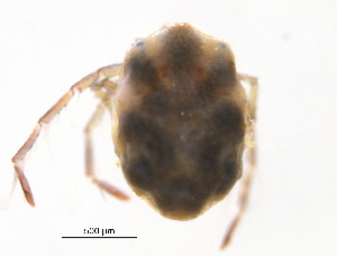

BIOUG15122-C05 [Dorsal]
Piona
Family: Pionidae
BIN URI: BOLD:ACE2606

IMAGE NOT AVAILABLE

BIOUG22328-G02
Pionidae
Family: Pionidae
BIN URI: BOLD:ACU5376

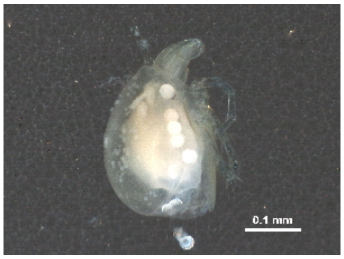

BIOUG22864-G03 [Dorsal]
Pionidae
Family: Pionidae
BIN URI: BOLD:ACV5563

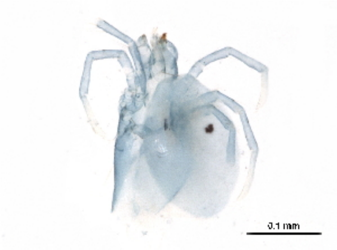

BIOUG23322-B03 [Dorsal]
Pionidae
Family: Pionidae
BIN URI: BOLD:ACW1303

IMAGE NOT AVAILABLE

BIOUG24005-D08
Pionidae
Family: Pionidae

IMAGE NOT AVAILABLE

BIOUG24005-B03
Arrenuridae
Family: Arrenuridae

IMAGE NOT AVAILABLE

BIOUG24005-B02
Trombidiformes

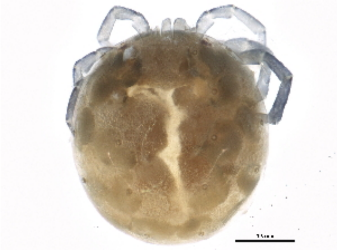

BIOUG08018-B06 [Dorsal]
Lebertia
Family: Lebertiidae
BIN URI: BOLD:ACK1955

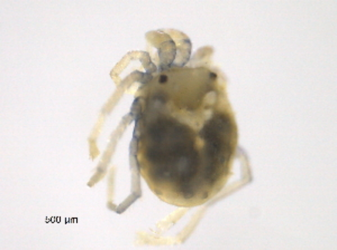

BIOUG15122-H08 [Dorsal]
Sperchon
Family: Sperchontidae
BIN URI: BOLD:ACO5111

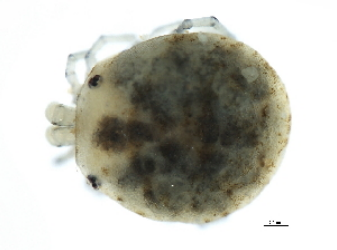

BIOUG05536-D03 [Lateral]
Trombidiformes

BIN URI: BOLD:ACF7275

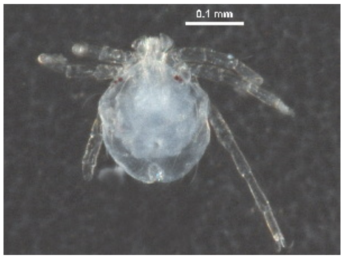

BIOUG22240-F09 [Dorsal]
Trombidiformes

BIN URI: BOLD:ACV3338

Copyright © 2014 BOLD Systems. All rights reserved.
